# Epigenetic Signatures Associated with Different Levels of Differentiation Potential in Human Stem Cells

**DOI:** 10.1371/journal.pone.0007809

**Published:** 2009-11-13

**Authors:** Pablo Aranda, Xabier Agirre, Esteban Ballestar, Enrique J. Andreu, José Román-Gómez, Inés Prieto, José Ignacio Martín-Subero, Juan Cruz Cigudosa, Reiner Siebert, Manel Esteller, Felipe Prosper

**Affiliations:** 1 Hematology Department and Area of Cell Therapy, Clínica Universidad de Navarra, Foundation for Applied Medical Research, University of Navarra, Pamplona, Spain; 2 Cancer Epigenetics and Biology Program (PEBC), The Bellvitge Institute for Biomedical Research (IDIBELL-ICO), L'Hospitalet de Llobregat, Barcelona, Spain; 3 Hematology Department, Reina Sofia Hospital, Córdoba, Spain; 4 Institute of Human Genetics, University Hospital Schleswig-Holstein Campus Kiel/Christian-Albrechts University, Kiel, Germany; 5 Molecular Cytogenetics Group, Spanish National Cancer Research Centre (CNIO), Madrid, Spain; George Washington University School of Medicine, United States of America

## Abstract

**Background:**

The therapeutic use of multipotent stem cells depends on their differentiation potential, which has been shown to be variable for different populations. These differences are likely to be the result of key changes in their epigenetic profiles.

**Methodology/Principal Findings:**

to address this issue, we have investigated the levels of epigenetic regulation in well characterized populations of pluripotent embryonic stem cells (ESC) and multipotent adult stem cells (ASC) at the trancriptome, methylome, histone modification and microRNA levels. Differences in gene expression profiles allowed classification of stem cells into three separate populations including ESC, multipotent adult progenitor cells (MAPC) and mesenchymal stromal cells (MSC). The analysis of the PcG repressive marks, histone modifications and gene promoter methylation of *differentiation* and *pluripotency* genes demonstrated that stem cell populations with a wider differentiation potential (ESC and MAPC) showed stronger representation of epigenetic repressive marks in *differentiation* genes and that this epigenetic signature was progressively lost with restriction of stem cell potential. Our analysis of microRNA established specific microRNA signatures suggesting specific microRNAs involved in regulation of pluripotent and differentiation genes.

**Conclusions/Significance:**

Our study leads us to propose a model where the level of epigenetic regulation, as a combination of DNA methylation and histone modification marks, at *differentiation* genes defines degrees of differentiation potential from progenitor and multipotent stem cells to pluripotent stem cells.

## Introduction

The progressive restriction of the differentiation potential from pluripotent embryonic stem cells (ESC) to different populations of multipotent adult stem cells (ASC) depends on the orchestrated action of key transcription factors and changes in the profile of epigenetic modifications that ultimately lead to expression of different sets of genes. ESC are unique in their capacities to self-renew and differentiate into any somatic and germ line tissue [1], [2], while, by contrast, the differentiation potential of ASC is limited.

ESC are characterized by an unusual chromatin features where marks of open chromatin, such as acetylated H3K9 and trimethylated H3K4, are combined with repressive histone modifications like H3K27 trimethylation at some non-expressed genes [3], [4], [5], [6], [7]. Specifically, various studies indicate that a number of key developmental and pluripotency genes are marked by bivalent marks of chromatin activation (H3K4me3) and repression (H3K27me3) that maintain genes in a “transcription-ready” state that allows rapid transcription activation upon differentiation of ESC [4], [5]. This bivalent domain signature is also present in differentiated cell types [7], [8], [9] suggesting that the number of promoters with bivalent modifications gradually decreases as the ESC differentiate thus corresponding to the degree of potency of a certain population of cells [9]. A key component implicated in the establishment of this epigenetic signature in the regulation of ESC is the Polycomb group family of proteins, which are responsible for maintaining the pluripotent state by epigenetic repression of developmental genes through the presence of repressive chromatin marks in the promoter regions of genes [10].

Promoter methylation is a second mechanism regulating pluripotency, commitment, and phenotypic maturation and differentiation of ESC. Previous studies indicating that methylation of key regulatory genes may play an important role in differentiation of ESC [11], [12] have been built upon by more recent ones that have used high-throughput strategies for DNA methylation profiling. The latter have demonstrated that gene regulation mediated by promoter CpG methylation in ESC complements other transcriptional mechanisms such as those mediated by OCT4 or NANOG, which are responsible for appropriate gene expression [13]. Mohn et al have proposed a model in which stem cell differentiation is associated with methylation of gene promoters (pluripotency genes) in lineage-committed progenitor cells while changes in histone marks are also acquired [14]. This suggests *de novo* DNA methylation is a dynamic switch that participates in the restriction of the developmental potential of progenitor cells.

Recent studies have provided strong evidence that microRNAs (miRNAs) also play critical roles in the differentiation potential of stem cells [15], which represents a third mechanism of stem cell regulation. miRNA expression profiles in human and mouse ESC reveal that they express a unique set of miRNAs that become downregulated when these cells differentiate, suggesting a role for miRNAs in the maintenance of pluripotency [16]. Moreover, regulation of pluripotency genes such as *NANOG*, *OCT4* and *SOX2* is mediated by specific miRNAs that have the ability to induce transcriptional silencing of these genes, resulting in differentiation of ESC [17], [18]. miRNAs are also important for ESC differentiation [19]. Knockout of Dicer, an RNase III-family nuclease required for miRNA maturation, compromises ESC proliferation and differentiation [20] while expression of certain miRNAs plays a critical role in ectodermal [21], cardiac [22] and muscle [23] lineage differentiation. The recently completed comprehensive profiling of miRNA expression in different tissues [24] will be of great use in determining whether employing the correct combination of miRNAs may facilitate the generation of homogeneous cell populations of desired lineages from ESC.

The comparison of gene expression profiles has given us a better understanding of the differences between populations of stem cells [25], [26], [27]. Although populations of stem cells with the ability to self-renew and differentiate have been isolated from most adult tissues, mesenchymal stromal cells isolated from bone marrow (MSC) and adipose tissue (ADSC) are the most thoroughly investigated populations of stem cells in the clinical setting. Uses for MSC are being explored in relation to the treatment of cardiac and vascular diseases, orthopedic diseases and, even more recently, in immune-mediated diseases such as diabetes and Crohn's disease [28]. In contrast to ESC, the potential of MSC is restricted to mesodermal cell types such as adipocytes, osteocytes, chondrocytes and, in some instances, skeletal muscle cells [29]. A population of ASC closely related to MSC, known as multipotent adult progenitor cells (MAPC), has recently been isolated from the bone marrow of humans and animals. MAPC have a greater potential to differentiate not only into the classical mesoderm-derived tissues but also into other tissues, such as endothelium and hepatocytes [30], [31], [32]. Recent studies have shown that the differences in the gene expression profile between murine ESC, MSC and MAPC reflect the stem cell differentiation potential and may form the basis of studies designed to provide insights into genes that confer the greatest developmental potency [33].

The role of epigenetic mechanisms in regulating differentiation and self-renewal of adult stem cells has been less extensively addressed. It is likely that a comparison between the epigenetic profiles of somatic stem cells and ESC will provide novel insights into the mechanisms involved in reprogramming of somatic cells and clues to understanding why certain somatic stem cells, such as neural stem cells, require fewer factors than others, such as fibroblasts [34]. In the current study, we have performed a high-throughput comparison at the gene expression, histone modification, DNA methylation and miRNA expression level of well characterized and defined populations of human stem cells, from pluripotent ESC to more restricted stromal cells derived from bone marrow and adipose tissue. Our results provide a direct connection between changes in the epigenetic signature and progression from pluripotency of ESC cells to restriction of the differentiation potential in MSC and ADSC, highlighting the existence of intermediate states of potency that exhibit specific epigenetic profiles.

## Results

### Isolation and Characterization of Stem Cell Populations

We first compared the differentiation potential of three different stem cell populations: mesenchymal stromal cells (MSC) isolated from human bone marrow, adipose tissue-derived stem cells (ADSC) and human multipotent adult progenitor cells (MAPC). MSC and ADSC populations met the criteria established by the International Society for Cellular Therapy (ISCT) for defining mesenchymal stromal cells [35]. MSC grew as adherent cells with fibroblastic morphology, were positive for HLA-ABC, CD73, CD105, CD29, CD13, CD44, CD90 and CD140b surface markers, and negative for CD34 and CD45 (not shown). MAPC were isolated from the same patients from whom MSC were obtained, and grown as previously described [30], [36]. Human MAPC expressed low levels of the CD44 antigen, were negative for HLA class I^−^, CD34^−^, CD45^−^, MHC-II^−^ and CD36^−^, and expressed CD90^+^ and CD13^+^ (not shown).

MSC and ADSC were able to differentiate to adipogenic, osteogenic and chondrogenic lineages using specific culture medium. Differentiation was not observed in human fibroblasts under the same conditions. *In vitro*, MAPC demonstrated a differentiation potential to endothelial cells, smooth muscle cells, as well as bone, cartilage and adipocytic cells, indicating a greater potential than MSC and ADSC (not shown).

### Transcriptome Analysis of Stem Cells

**Figure 1:**
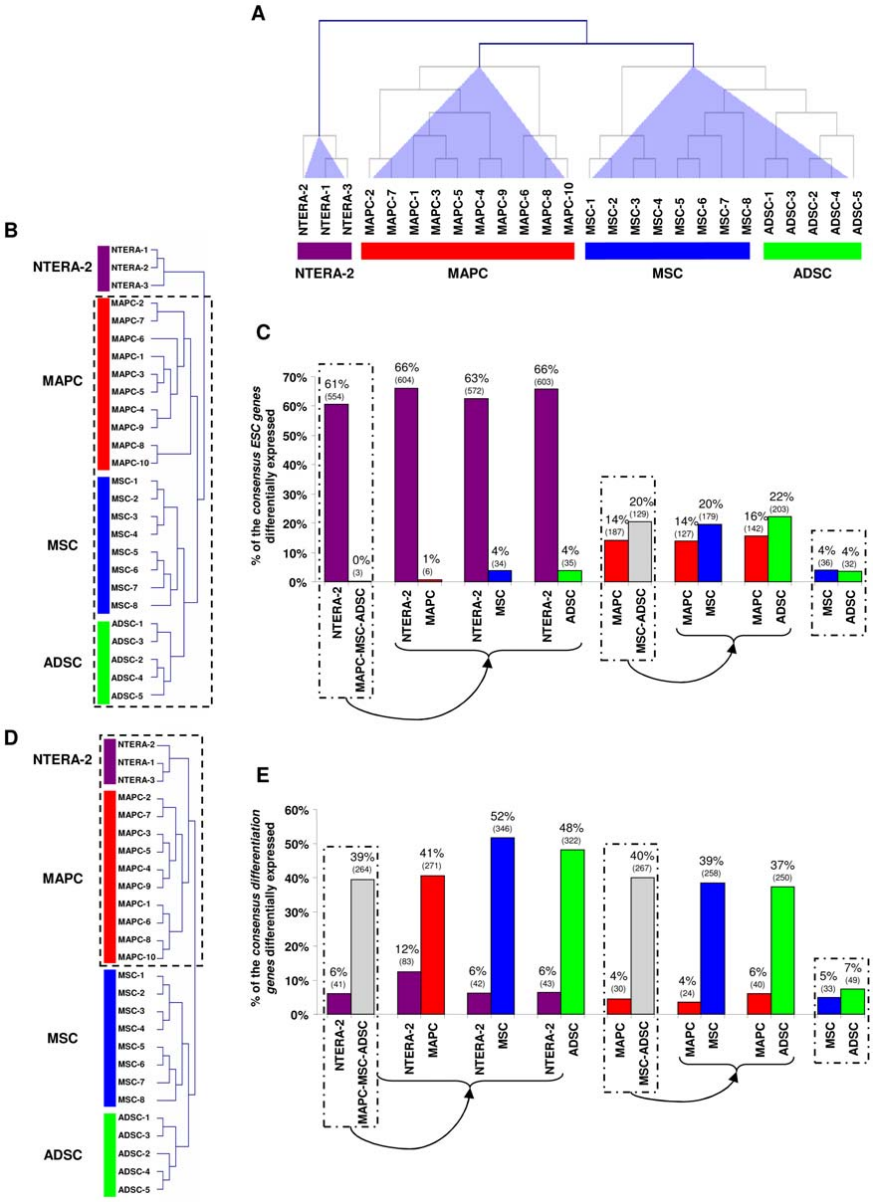
Hierarchical clustering and supervised analysis of *consensus ESC genes* and *differentiation genes*. Dendrogram of hierarchical cluster analysis based on the expression of all genes (**A**), of the *consensus ESC genes* (**B**) and *consensus differentiation genes* (**D**) included in the HG-U133 Plus 2.0 chip. Hierarchical clustering (Euclidian distance) was performed with the TIGR MeV v. 2.2 program. Analysis of the differential expression of *consensus ESC genes* (**C**) and *consensus differentiation genes* (**E**) between the populations of stem cells using the significant analysis of microarrays (SAM) algorithm. Values are shown as the percentage of genes that belong to the *consensus ESC* or *consensus differentiation gene* lists that are differentially expressed in each group after comparison with SAM. The number of deregulated genes is indicated in parentheses. Only deregulated genes with FC>2 were considered.

Gene expression was then analyzed using the Affymetrix HG-U133 Plus 2.0 GeneChip Oligonucleotide Microarray. We compared the expression profile of the above stem cell populations with NTERA-2 cells, a human embryonic carcinoma stem-cell line. Previous gene expression profile studies have demonstrated that NTERA-2 cells cluster together with human ESC supporting their use as a model of ESC [37]. Hierarchical cluster analysis generated using all the probe sets included in the array classified the different independent cell populations NTERA-2 (n = 3), MAPC (n = 10), MSC (n = 8) and ADSC (n = 5) into three discrete clusters that include NTERA-2, MAPC and a third cluster including ADSC and MSC (Figure 1A and Table S2). We also used PCA to explore further the cell-type relationships of the various stem cell populations. PCA clearly separated the MAPC, MSC, ADSC and NTERA-2 populations into four groups. Moreover, it showed that 53% of the total variance in the data was explained by the differences between the NTERA-2 cell line and the rest of the populations, 36% of the variance was due to differences between MAPC and MSC-ADSC populations, and only 6% of the variance was due to differences between MSC and ADSC populations (Figure S1).

Two additional sets of comparisons were then made. First, we compared the transcriptome of NTERA-2 with all other ASC to determine the differences between pluripotent (NTERA-2) and multipotent stem cells. Second, we also compared the different populations of ASC (MAPC versus MSC and ADSC) to determine expression profiles that might explain the differences observed between ASC, which were the second largest cause of variability in the data, as clearly indicated by the PCA. These analyses were performed with LIMMA to identify differentially expressed probe sets (see Supplementary Materials and Methods). The functional classification, using gene ontology (GO) annotation, of the differentially expressed probe sets in the first comparison (NTERA-2 versus ASC) showed that NTERA-2 cell line was enriched in genes involved in DNA modification, repair, replication, DNA packaging, chromatin modification, mitosis and transcription. The latter included transcription factors that are enriched in ESC, such as *SOX2*, *NANOG*, *OCT3/4* (*POU5F1*), *SALL3* or *ZIC3*. In contrast, some of the categories over-represented in ASC were involved in developmental processes (skeletal and blood vessel development), cell differentiation, cell adhesion, organ morphogenesis, wound healing, angiogenesis, NF-kappaB signaling and collagen fibril organization (Figure S2 and Table S3). The comparison of the populations of ASC yielded an enrichment in MAPC for genes involved in mitosis and DNA repair while categories represented in MSC and ADSC were related to development (including muscle and skeletal development), cell and cell-matrix adhesion and signaling (TGF-beta and JAK-STAT signaling) (Figure S2 and Table S4). The results of both comparisons suggest that MAPC represent a population with intermediate characteristics between ESC and other populations of adult stem cells such as MSC and ADSC.

**Figure 2:**
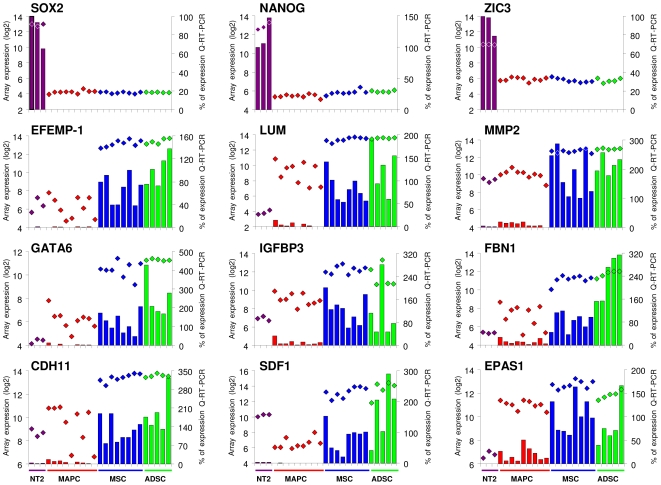
Expression of genes from the *consensus ESC* and *differentiation genes* lists in NTERA-2, MAPC, MSC and ADSC cells by Q-RT-PCR. Gene expression was measured using the relative standard curve method. *GAPDH* was used as a housekeeping control. Expression values of each sample from Affymetrix arrays (logarithmic scale: rhombuses) and from Q-RT-PCR analysis (percentages: bars) are shown for each gene. Q-RT-PCR results are expressed on the secondary vertical axis as a % of expression relative to NTERA-2 for *consensus ESC genes*, and to MSC for *consensus differentiation genes*. Each bar represents the value of a different cell line.

A recent meta-analysis of the results of 38 studies compared the transcriptome of human ESC with differentiated cells identifying a gene list that included transcripts that are over-expressed in ESC (*consensus ESC* gene list, n = 1.076), and a second list that included genes underexpressed in ESC (*consensus differentiation* gene list, n = 783) [38]. We determined the gene expression for both consensus gene lists in our cell populations using hierarchical clustering and the SAM algorithm. The hierarchical clustering of the *consensus ESC genes* in the Affymetrix array (n = 915) revealed that NTERA-2 cells grouped separately from MAPC, MSC and ADSC (Figure 1B). SAM analysis showed that NTERA-2 samples expressed more *consensus ESC genes* than each group of ASC (Figure 1C) (Table S5). Interestingly, when the same comparison was made of the *consensus differentiation genes* present in the Affymetrix array (n = 669), MAPC and NTERA-2 cells grouped together, separately from MSC and ADSC (Figure 1D). In addition, SAM analysis revealed the expression of a higher percentage of *consensus differentiation genes* in MSC and ADSC than in NTERA-2 cells and MAPC (Figure 1E) (Table S6A and S6B). These results were confirmed by real-time PCR (Q-RT-PCR) using some of the *consensus ESC genes* (*SOX2*, *NANOG*, *ZIC3*) and *consensus differentiation genes* involved in muscle development (*GATA6*, *IGFBP3*), skeletal development (*FBN1*, *CDH11*) and extracellular matrix (*EFEMP1*, *LUM*, *MMP2*). We also included two randomly selected genes (*SDF1*, *EPAS1*) from the LIMMA analysis (Figure 2). These results might explain the greater potential of MAPC compared with other populations of ASC such as MSC and ADSC [30], [31] and allow MAPC and pluripotent ESC to be distinguished.

### Stem Cell Regulation by Polycomb Proteins and Histone Modifications

**Figure 3:**
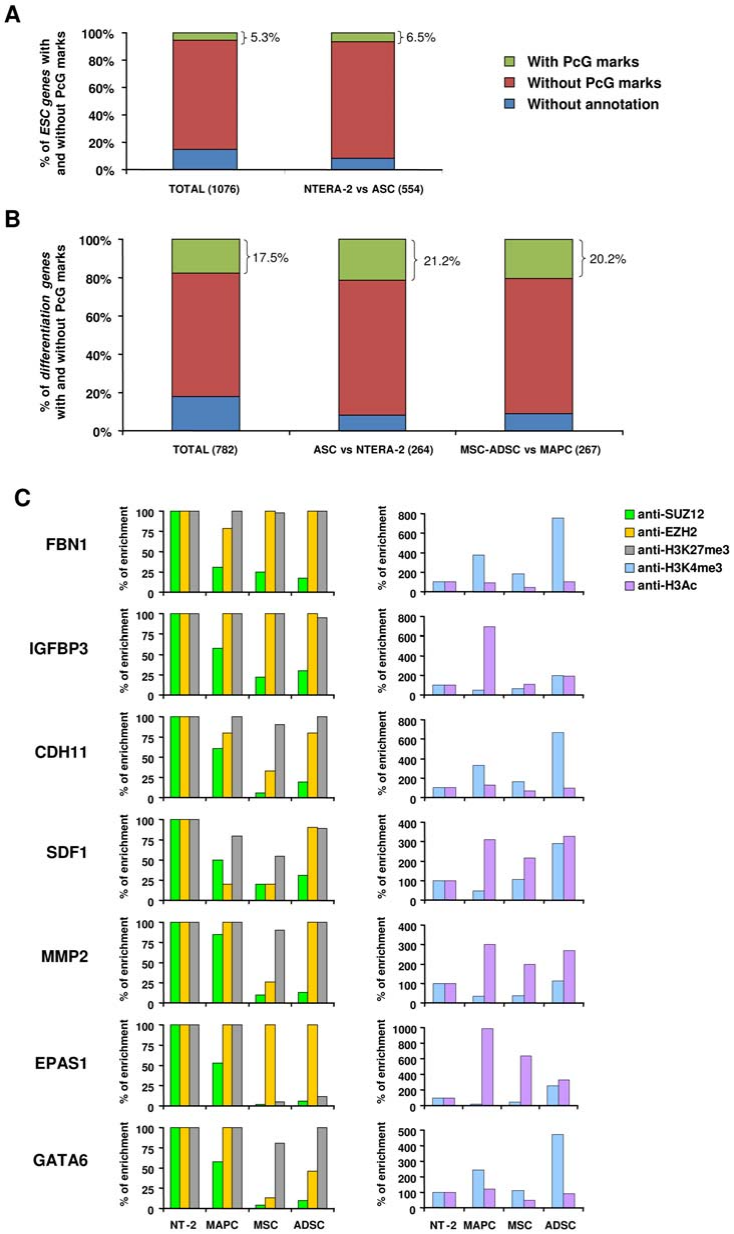
*Consensus ESC* and *differentiation genes* with predicted Polycomb group marks, and regulation of *differentiation genes* by PcG proteins in stem cells. **A**, Bar graph displaying the percentage of genes targeted by PcG marks among the *consensus ESC gene* list and among the ESC genes upregulated in NTERA-2 cells relative to ASC. **B**, Bar graph displaying the percentage of genes targeted by PcG marks among the *consensus differentiation gene* list, the differentiation genes upregulated in ASC relative to NTERA-2 cells, and differentiation genes upregulated in MSC and ADSC versus MAPC. The numbers in brackets represent the total number of genes. **C**, Occupancy of EZH2, SUZ12, H3K27m3, H3K4me3 and H3 acetylated, assessed by Q-ChIP-PCR, in the promoters of differentiation genes. Enrichment is presented as a percentage relative to NTERA-2 (100%).

Recent studies have demonstrated that Polycomb group (PcG) proteins participate in the repression of developmental genes that are activated during ESC differentiation [10], [39], [40]. For instance, mapping the sites occupied by SUZ12, one of the subunits of the Polycomb repressive complex 2 (PRC2), throughout the genome in human ESC, indicated that SUZ12 occupies the promoter regions of a large group of developmental regulators [10]. To test the role of PcG-mediated epigenetic regulation of the different expression signatures in NTERA-2 cells and ASC we used bioinformatic tools to examine whether *consensus ESC* and *differentiation genes* from our expression analysis were marked by PcG. Of all the *consensus ESC genes* described in the meta-analysis of Assou et al. [38] (n = 1,076), only 5% showed a PRC2 mark (EED, SUZ12 or H3K27me3). A similar percentage of these marks was observed in the *consensus ESC genes* in our study that were upregulated in NTERA-2 with respect to ASC (n = 554, detected by SAM analysis) (Figure 3A) (Table S5). However, we found a higher percentage (17.5%) of genes in the *consensus differentiation genes* that were marked by PcG proteins. This percentage was even higher when we considered *differentiation genes* that were upregulated in ASC relative to NTERA-2 cells and in MSC-ADSC versus MAPC in our study (Figure 3B) (Table S6A-B). These results suggest that PcG-mediated regulation of gene expression in stem cells may be more important for genes among the *consensus differentiation gene* than for genes that belong to the *consensus ESC genes*.

To demonstrate that PRC2 is indeed involved in the regulation of differentiation genes, we performed quantitative-ChIP assays using antibodies against EZH2, SUZ12 and H3K27m3 (marks associated with repressed chromatin), and two histone modifications associated with transcriptionally active chromatin, trimethylated histone H3 at lysine 4 (H3K4me3) and acetylated histone H3 (H3Ac), in the various stem cell populations. The promoters of some of the differentiation genes that were previously validated by Q-RT-PCR (Figure 2) and were associated with PcG marks according to the results of Lee et al [10] were used for the analysis. We observed that, consistent with the expression analysis, *differentiation gene* promoters in ASC showed a decrease in repressed chromatin marks (mostly SUZ12 and, to a lesser degree, EZH2 and H3K27me3) relative to NTERA-2. This decrease was also found in MSC and ADSC when they were compared with MAPC (Figure 3C). On the other hand, an increased presence of active marks (H3K4me3 and/or H3Ac) on the promoters of these genes was observed in ASC in comparison with NTERA-2 (Figure 3C). Some of the genes, such as *MMP2*, *EPAS1* and *GATA6*, exhibited a higher level of occupancy of SUZ12, EZH2 and H3K27me3 in MAPC than in MSC and ADSC, which suggests PcG-dependent regulation in MAPC. The reduction in repressed marks, together with the increase of active marks in ASC, suggests a favorable balance for the expression of differentiation genes in these cells, while the differences observed between MSC-ADSC and MAPC suggest that epigenetic mechanisms are probably involved in determining the different potential of these populations of adult stem cell.

### Promoter DNA Methylation in Stem Cells

Since promoter CpG methylation has also been demonstrated to contribute to the regulation of gene expression in ESC [13], we analyzed the DNA methylation profiles of the different populations of stem cells (MSC, ADSC, MAPC and NTERA-2 cells) using the BeadArray technology [41], [42]. The array contains 807 gene promoters, including 7.4% (n = 80) and 14.4% (n = 113) of the genes in the *consensus ESC* and *differentiation* list described by Assou et al, respectively. [38] (Table S7, S8A–S8B). Hierarchical clustering produced two discrete groups, one containing NTERA-2 samples and the other group comprised of all ASC populations (not shown).

We next compared the DNA methylation status of NTERA-2 and the various ASC populations. Of a total of 83 genes that were differentially methylated between NTERA-2 cells and ASC (Table S9), 99% (n = 82) were hypomethylated in ASC and hypermethylated in NTERA-2 cells (Figure S3). Fourteen of these genes had PcG marks (17%) and eight belonged to the *consensus differentiation genes* (10%), two of them with PcG marks. In contrast, none of the differentially methylated genes belonged to the group of the *consensus ESC genes*. According to gene ontology, hypomethylated genes in ASC were involved in signal transduction, cell-cycle arrest, induction of apoptosis and development. The hypermethylation of these genes could serve as a molecular lock to ensure proliferation in pluripotent stem cells, while prevents their differentiation. The number of genes differentially methylated between ASC populations was limited to less than 4% of the gene promoters in the array (Table S9 and Figure S3). Interestingly, 27 genes (3.3% of those analyzed) were differentially methylated between ADSC and MSC (Table S9 and Figure S3). While MSC had 24 hypermethylated genes, ADSC featured only three methylated genes. On the other hand, only seven and three genes were differentially methylated between MAPC versus MSC and MAPC versus ADSC, respectively.

**Figure 4:**
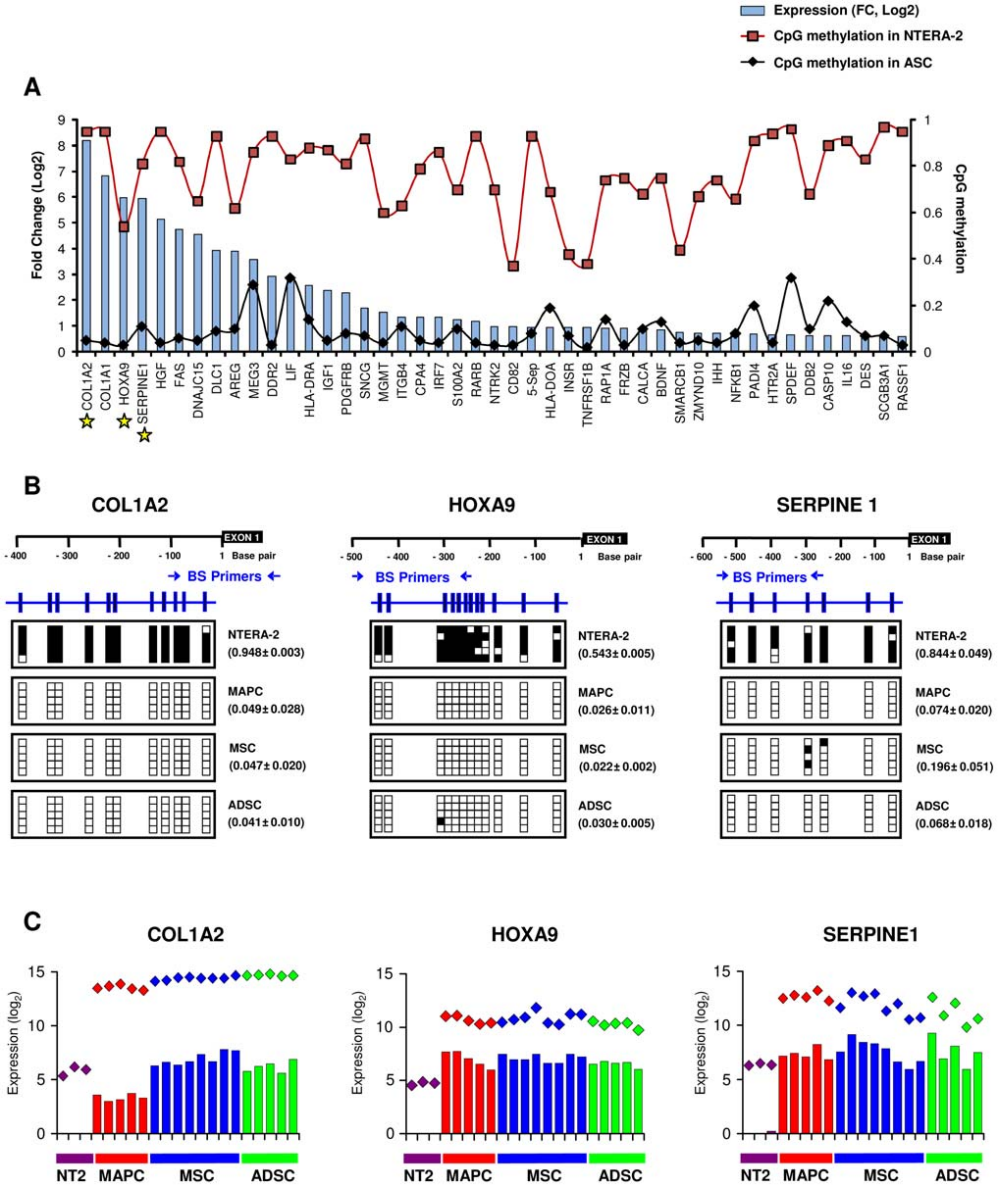
Methylation and expression of genes hypomethylated in ASC in comparison with NTERA-2 cells. **A,** Differences of expression between ASC and NTERA-2 (blue bars) and promoter methylation in ASC (black diamonds) and NTERA-2 (red squares) are presented for each gene. The differences of expression, according to Affymetrix data, are shown as fold change (FC) in log_2_. The methylation data are average BeadArray values. **B,** Bisulfite sequencing of promoter regions of *COL1A2*, *HOXA9* and *SERPINE1* in NTERA-2 and ASC cells. Promoter schematic description (black) and the location of the CpGs (blue) examined are presented for each gene. The CpG dinucleotides (clear box: unmethylated, filled box: methylated) of five clones and the BeadArray methylation values±standard deviation are shown for each sample. **C,** Expression of *COL1A2*, *HOXA9* and *SERPINE1* from Affymetrix arrays (colored rhombuses) and quantitative Q-RT-PCR (colored bars).

To establish whether promoter methylation participated in the regulation of genes differentially expressed in NTERA-2 cells and ASC we analyzed the gene expression using transcriptome data and the SAM algorithm and found that 56% of the hypomethylated genes in ASC (n = 46) were overexpressed in these cells in comparison with NTERA-2, (Table S10 and Figure 4A). Among the most differentially expressed genes were *COL1A2*, *COL1A1*, *SERPINE1*, *DLC1*, *DDR2* and *PDGFRB*, which were also listed as *differentiation genes*. These results were validated by bisulfite sequencing and Q-RT-PCR for three of the differentially methylated and expressed genes (*COL1A2*, *HOXA9* and *SERPINE1*) (Figure 4B–C). On the other hand, eight of the 27 genes differentially methylated in MSC and ADSC showed differences in expression that were greater than two (FC>2) in only two cases. Finally, the comparison between genes regulated by PcG [10] and regulated by promoter methylation indicates that these two mechanisms overlapped to a degree in the regulation of specific gene expression. Taken together, these results suggest that gene promoter methylation plays a more significant role in silencing differentiation and developmental genes in pluripotent stem cells (NTERA-2) than in regulating ESC genes in adult stem cells.

### MicroRNA Expression Profile in Stem Cells

We finally examined the expression of 250 mature human miRNAs listed in the Sanger database (version 9.0 [October 2006]) in 21 cell lines, including NTERA-2 (n = 3), MSC (n = 7), MAPC (n = 4) and ADSC (n = 7). A hierarchical cluster analysis of all the miRNAs classified the cells lines into two groups, one of which included NTERA-2 cells and the other group including all the different ASC (Figure S4 and Table S11). Differential expression of 64 miRNAs was observed in ESC relative to ASC (34 upregulated and 30 downregulated) (Table S12). However, only a few miRNAs were differentially expressed between ASC populations (*miR-143* downregulated and *miR-204* upregulated in MAPC with respect to MSC; *miR-129* and *miR-199b* downregulated and *miR-204* upregulated in MAPC respect to MSC and *miR-424* downregulated in MSC respect to ADSC) and those differences were smaller than those observed between ESC and ASC. A recent comparison of miRNAs differentially expressed between ESC and differentiated cells [43] identified a unique miRNA signature associated with ESC that, in fact, was highly coincidental with the list of miRNAs differentially expressed in our NTERA-2 cells compared with ASC (not shown).

**Figure 5:**
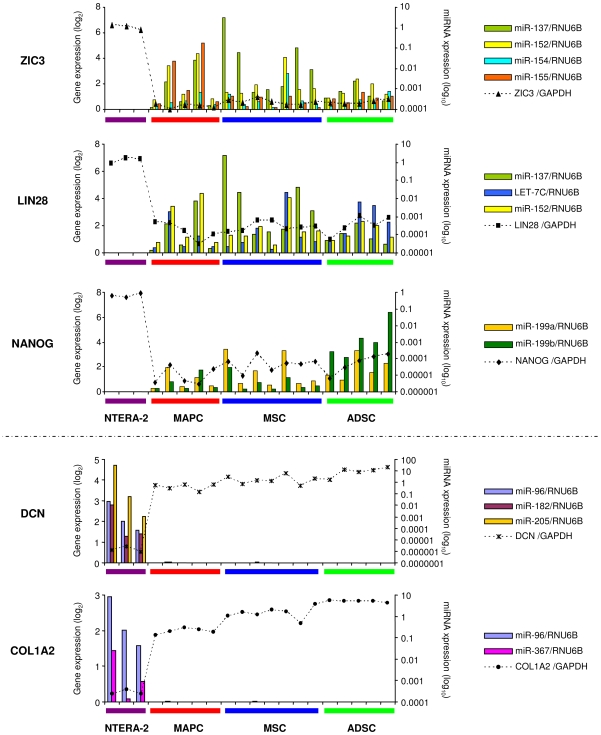
Expression of miRNAs and their putative ESC and differentiation target genes. Expression of ESC genes -*NANOG*, *LIN28* and *ZIC3*- and differentiation genes -*DCN* and *COL1A2*- (dashed lines, log_2_, left vertical axis) and expression of miRNAs (colored bars, log_10_, right vertical axis) predicted to regulate expression of these genes were analyzed by Q-RT-PCR in each cell line.

The results obtained from the transcriptome and miRNA analyses prompted us to examine whether *consensus ESC genes* and *differentiation genes* could be regulated by differentially expressed miRNAs. Using prediction programs we identified *ZIC3*, *LIN28* and *NANOG* as putative targets for miRNAs upregulated in ASC in comparison with NTERA-2 cells. Similarly, differentiation genes such as *DCN* and *COL1A2* were identified as putative targets for miRNAs downregulated in ASC in comparison with NTERA-2 cells. We directly examined the expression of these differentially regulated miRNAs and their targets by Q-RT-PCR using the different cell lines. An inverse correlation between expression of *ZIC3* and *miR-137*, *miR-152*, *miR-154* and *miR-155*; *LIN28* and *let-7c, miR-137* and *miR-152* and *NANOG* and *miR-199a* and *miR-199b* expression was found in ASC and NTERA-2 cells (Figure 5). Expression of *DCN* and *COL1A2* (upregulated in ASC compared with NTERA-2 cells) was also inversely correlated with expression of the miRNAs with target seed sequences for those genes *miR-96*, *miR-182*, *miR-205* (*DCN*) and *miR-96* and *miR-367* (*COL1A2*) (downregulated in ASC relative to NTERA-2 cells) (Figure 5). These results suggest a role for miRNAs in regulating ESC and differentiation genes as a third mechanism of stem cell gene regulation.

**Figure 6:**
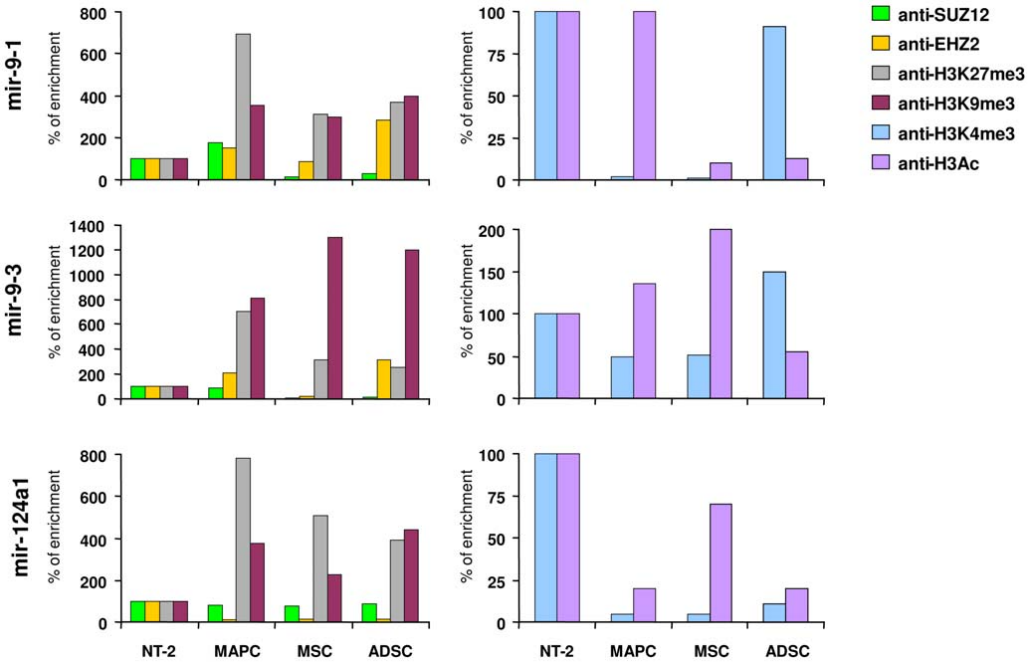
Quantitative-ChIP assay performed on *miR-9-1, miR9-3* and *-miR124a1*. Antibodies specific to SUZ12, EZH2, H3K27me3, H3K9me3, H3K4me3 and H3 acetylated were used for ChIP, and then the *miR-9-1, miR-9-3* and *miR-124a1* levels were determined using Q-RT-PCR. Enrichment is measured as percentage relative to NTERA-2 cells (100%).

A recent study has described a group of six miRNAs (*miR-155*, *miR-708*, *miR-615*, *miR-375*, *miR-124a* and *miR-9*) co-occupied simultaneously by the ESC transcription factors OCT4, SOX2, NANOG and TCF3 and by the Polycomb group proteins. These miRNAs are transcriptionally silenced in ESC but are expressed in a tissue-specific fashion in differentiated cells [18]. When we analyzed the expression of these miRNAs we observed that expression of *miR-9* and *miR124a* was upregulated in NTERA-2 cells in comparison with ASC. To understand the discrepancies with our microRNA expression data we analyzed the histone modification and DNA methylation patterns associated with these miRNAs. We performed a quantitative-ChIP assay using antibodies against EZH2, SUZ12, H3K27m3 and H3K9m3 (marks associated with repressed chromatin), and H3K4me3 and H3Ac (histone modifications associated with transcriptionally active chromatin) in three miRNAs (*miR-9-1*, *miR-9-2* and *miR-124a-1*) upregulated in NTERA-2 cells with respect to ASC. Consistent with the expression analysis, we observed that these three miRNAs showed an increase of H3K27m3 and H3K9m3 repressed chromatin marks and a decrease of open chromatin marks in ASC in comparison with NTERA-2 (Figure 6). DNA methylation analysis did not reveal any differences between NTERA-2 cells and ASC (Figure S5). The stronger expression of these three miRNAs in NTERA-2 cells could be explained by the fact that ESC transcription factors are expressed in NTERA-2 cells but not in ASC, while the pattern of proteins of the PcG bound to ASC in contrast to NTERA-2 cells supports a close chromatin conformation [18].

## Discussion

**Figure 7:**
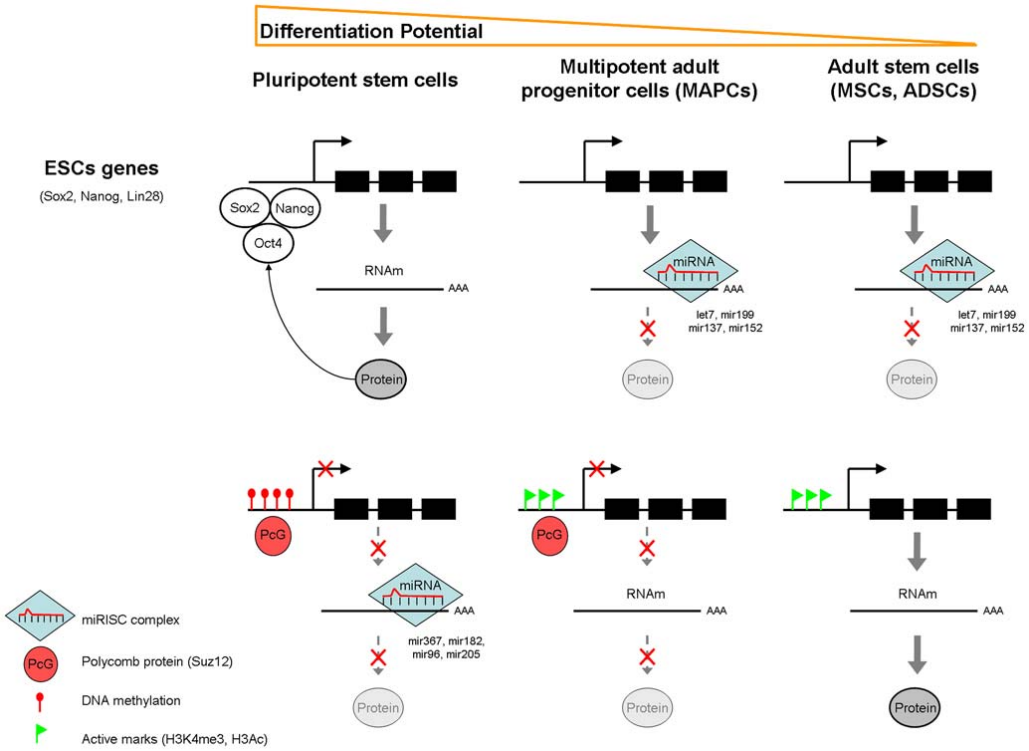
Model of epigenetic regulation of ESC and differentiation genes in different populations of human stem cells. Genes implicated in pluripotency (Oct4, Sox2, Nanog) are transcriptionally active in ESC due to the lack of repressive marks as well as by their own potential to transactivate their own transcription favoring the greater differentiation potential of ESC. *Differentiation* genes, on the contrary are silenced on ESC due to several epigenetic mechanisms that include expression of miRNA, DNA promoter methylation and repressive histone marks that all together cooperate to induce down-regulation of *differentiation* genes. A decrease in the differentiation potential of ASC (MAPC, MSC and ADSC) is mediated by the silencing of pluripotency genes downregulated through the expression of certain miRNA. The presence of PcG proteins (particularly SUZ12) on the promoters of *differentiation* genes but the lack of methylation on MAPC could explain their reduced expression. Finally, the lack of PcG marks along with the increase in open chromatin marks in the promoters of *differentiation* genes would explain the greater expression of these genes as well as the more restricted differentiation potential of ASC (MSC and ADSC).

Stem cells are characterized by their capacity to self-renew and differentiate into committed cells. While ESC can differentiate into any type of tissue, the potential of somatic stem cells is usually restricted to certain types, most prominently those derived from the same germ layer from which they are derived. Several studies have analyzed the differences in gene expression between different types of stem cells and identified signatures associated with stem cell populations as well as specific subsets of genes associated with particular types of stem cells [25], [26]. Significantly less information is available regarding the mechanism responsible for the differences in gene expression between populations of stem cells and although several studies have addressed the epigenetic make up of ESC and the role of epigenetic mechanisms in their self-renewal and differentiation [9], [10], [44], [45], few attempts have been made to analyze the differences in epigenetic regulation in somatic stem cells. In this study, our comparison of the epigenetic regulation of gene expression between well characterized populations of ASC and ESC revealed that different epigenetic signatures characterize different populations of stem cells, whereby embryonic stem cell populations, with a higher differentiation potential, exert stronger epigenetic repression of *differentiation* genes through both DNA methylation and histone modifications, versus MSC and ADSC where *differentiation* genes are active and exhibit active epigenetic profiles. Interestingly, we find that MAPC, where the differentiation potential is intermediate between ESC and these two ASC populations, exhibit an epigenetic profile where only repressive histone modification marks, but not DNA methylation associate with repression of *differentiation* genes. In this sense, our transcriptome results indicate that MAPC and MSC or ADSC are populations of somatic cells whose differences in expression can be explained by the differences in the epigenetic regulation of differentiation genes. We therefore propose a model (Figure 7) in which *differentiation* genes exhibit progressively less epigenetic constraints. In ESC cells, *differentiation* genes are repressed through both DNA methylation and repressive histone modifications. In MAPC, *differentiation* genes are still repressed and PcG marks are present but promoter DNA methylation has been lost. In MSC and ADSC repressive histone modification marks are lost.

Other studies have also demonstrated the important role for the PcG machinery in silencing genes that must be repressed to maintain self renewal and pluripotency, and that become activated during differentiation [10], [40]. These studies have been carried out in the context of muscle [46], neural [47], erythroid [48] and germ cell [49] differentiation. However, the functional loss of PRC2 components such as EED and SUZ12 results in the upregulation of genes associated with cell differentiation but do not affect the self-renewal capacity of ESC, indicating that PRC2 is not essential for pluripotency [44], [50]. This is consistent with our results in which the PRC2 repressive effect on the promoters of *differentiation genes* in ASC is lost because there is a decrease in SUZ12, leading to gene upregulation. This reduction in SUZ12 is greater in MSC and ADSC than in MAPC, which could explain the differences in gene expression between stem cell populations (Figure 3C). The possible lack of direct involvement of PRC2 in regulating pluripotency is also consistent with our observation that only 5% of the pluripotent genes, compared with 18–20% of the differentiation genes, have PcG marks. Thus PcG-mediated gene regulation in stem cells could be more relevant for genes involved in development and differentiation than for pluripotent genes.

The differences in gene promoter DNA methylation between ESC and ASC imply a role for methylation in the maintenance of pluripotency since 99% of the genes differentially hypermethylated in NTERA-2 cells relative to ASC were those known to be involved in development and differentiation. However, methylation of gene promoters in ESC was associated with downregulation in only 56% of the hypermethylated genes. The role of gene promoter methylation in stem cell biology has been highlighted by recent studies [13], [51], [52], which suggest that ESC differentiation is associated with progressive demethylation of differentiation gene promoters and lend weight to the hypothesis that the different potential of stem cells is also dependent on the greater hypermethylation observed in ESC compared with somatic stem cells. This conclusion needs to be drawn with caution since the array used in our study contained a limited number of gene promoters (n = 807), most of which are associated with cancer. The overall pattern needs to be determined in genome-wide DNA methylation studies to establish whether this analysis can be generalized to the whole genome.

Another caveat to our conclusions arises from the fact that, instead of using human embryonic cell lines, we used an embryonic carcinoma cell line, which might not be considered the best model of the pluripotent stem cell. However, recent studies support the use of NTERA-2 as well as other embryonic carcinoma cell lines as a model of ESC: 1) It has been published that the expression profiles, not only of genes but also of miRNAs, and the methylation patterns of the NTERA-2 cell line are broadly similar to that of human ESC [37], [53]; 2) Although NTERA-2 cells are derived from an embryonic carcinoma, the methylation pattern was identical to that of human ESC lines (as shown by the comparison between the methylation profile of NTERA-2 in the current study and methylation in human ESC shown in a previous study using the same platform) [51]. These findings rule out the possibility that the observed hypermethylation in NTERA-2 cells were related to the origin of the cells and different from ESC further supporting the use of NTERA-2 cells. 3) We also compared the miRNA expression of NTERA-2 cells with the results of a recent study in which miRNA expression was analyzed in 26 cell lines, including human ESC, NPC, NSC, MSC and differentiated cells. We found that miRNAs differentially expressed between NTERA-2 cells and ASC coincided with miRNAs differentially expressed between human ESC and ASC [43]. For all these reasons, we believed that our results are indeed useful to understand the biology of pluripotent stem cells such as ESC.

A final comment relates to the potential use of our results in the understanding of the reprogramming process. The demonstration that somatic cells can be reprogrammed to produce a fully pluripotent state [54], [55], [56] has been a major breakthrough in the field. Progress is being made towards a better understanding of the reprogramming process. Our results are a useful contribution to this endeavor, arguing for a stepwise model of the different epigenetic stages, from somatic cells to progenitor and multipotent stem cells and to pluripotent stem cells. This equips us with a roadmap for producing specific types of stem cells depending on clinical need.

## Materials and Methods

### Cell Populations

Human mesenchymal stromal cells (MSC) were established from bone marrow from patients of 20–60 years of age. Human adipose-derived stem cells (ADSC) were obtained from human liposuction procedures. Multipotent adult progenitor cells (MAPC) were established as described [30]. MAPC and MSC isolates were obtained from the same patients. MAPC, ADSC and MAPC were referred as adult stem cells (ASC). All samples were obtained after the donor had given their written informed consent, in accordance with the guidelines of the Committee on the Use of Human Subjects in Research of the Clinica Universidad de Navarra. The study was approved by the Ethics Committee for Research with Human subjects at the University of Navarra. The human NTERA-2 cell line was purchased from DSMZ-German Collection of Microorganisms and Cell Cultures (Braunschweig, Germany).

### Expression Microarrays

MAPC (n = 10), MSC (n = 8), ADSC (n = 5) and NTERA-2 cells (n = 3; 3 different cultures) were used for the microarray analysis. RNA isolation, labeling and hybridization to the HG-U133 Plus 2.0 GeneChip Oligonucleotide Microarray (Affymetrix Inc., Santa Clara, CA) were performed as previously described [57]. Principal components analysis (PCA) was used to reveal trends in the data and to identify predominant gene expression patterns. Statistical significance of differential expression was determined using the Significant Analysis of Microarrays (SAM) algorithm. The log-transformed and mean-centered intensity values of significant differentially expressed genes were used for PCA, which was carried out using Spotfire (Spotfire Inc., Cambridge, MA).

### Quantitative RT-PCR Analysis

RNA samples used for the Q-RT-PCR were the same as those used for hybridizing Affymetrix HG-U133 Plus 2.0 array. Primers and probes used for real-time PCR are shown in Table S1. Gene expression was calculated using the relative standard curve method. GAPDH was used as a housekeeping control.

### Chromatin Immunoprecipitation Assay

A sample of each population studied in Affymetrix arrays was subjected to chromatin immunoprecipitation. ChIP assays were performed as previously described [58], [59] and the ChIP fractions were used for quantitative-PCR assay. Immunoprecipitated fractions were obtained with the ChIP grade antibodies anti-SUZ12 (ab12073), anti-EZH2 (ab3748), anti-trimethylated Lys 27 of histone 3 (H3K27me3: ab6002), anti-trimethylated Lys 4 of histone 3 (H3K4me3: ab8580) (all from Abcam, Cambridge, MA) and anti-acetylated histone H3 (H3Ac: 06-599, Upstate Biotechnologies, Lake Placid, NY).

### DNA Methylation Profiling Using Universal BeadArrays

DNA samples from NTERA-2 (n = 2), MAPC (n = 4), MSC (n = 4) and ADSC (n = 4) cells were analyzed. We used GoldenGate® Methylation Cancer Panel I (Illumina Inc.) for the DNA methylation analysis. Methylation assay was performed as described previously [41], [53]. To validate the DNA methylation data generated by the BeadArray technology, bisulfite sequencing (BS) was performed as previously described [60], [61].

### MicroRNA Expression Analysis by Quantitative Real-Time PCR (Q-RT-PCR)

Expression of 250 miRNAs was analyzed using specific primers and TaqMan probe for each miRNA according to the TaqMan MicroRNA Assay protocol, as previously described [62]. Relative quantification of expression of microRNAs was calculated with the 2^−ΔΔCt^ method (Applied Biosystems. User Bulletin N°2 (P/N 4303859)). The data are shown as log_10_ of the relative quantity (RQ) of target miRNAs, normalized and compared with expression in NTERA-2. In order to identify microRNAs with statistically significant changes in expression between the groups, we performed a supervised analysis using the SAM algorithm.

**Supplemental Material and Methods** provides a more detailed description of the methods used in the current studies (Text S1).

## Supporting Information

Text S1 Supplemental Material and Methods
(0.11 MB DOC)

Figure S1 Exploratory data analysis with PCA method on the gene expression data of MSC, ADSC, MAPC and NTERA-2 A. Samples plotted in the first three principal components (PC); B. Variation captured in each PC
(1.13 MB TIF)

Figure S2 Functional analysis of differentially expressed probe-sets between stem cell populations Comparison between the gene expression profile of NTERA-2 versus Adult Stem Cells (A) and MAPC versus MSC-ADSC cells (B). The graphs show categories overrepresented in each group according to their p-value (line, in log10) and the number of genes in each category (bars, in log10). Only categories with a p-value less than 0.01 were selected
(0.54 MB TIF)

Figure S3 DNA methylation in stem cells Number of genes differentially methylated in each comparison from supervised analysis. The number of genes hypomethylated and hypermethylated for each comparison is shown.
(0.15 MB TIF)

Figure S4 Dendrogram of hierarchical cluster analysis based on 250 miRNA expression data. ESC, human embryonal carcinoma; ASC, human adult stem cells; MAPC, Multipotent Adult Progenitor Cells; MSC, Mesenchymal Stem Cells; ADSC, Adipose-Derived Stem Cells.
(0.20 MB TIF)

Figure S5 Promoter hypermethylation of miR-9-1, miR-9-3 and -miR-124a1 MSP analysis of the miR-124a-1, miR-9-1 and miR-9-3 CpG island regions in NTERA-2 and ASC. MAPC, Multipotent Adult Progenitor Cells; MSC, Mesenchymal Stem Cells; ADSC, Adipose-Derived Stem Cells. M: methylated allele; U: un-methylated allele.
(0.63 MB TIF)

Table S1 Primers and probes used for PCR. X: cycles of PCR; aT: annealing Temperature.
(0.06 MB DOC)

Table S2 RMA raw data (Affymetrix's data)
(6.47 MB PDF)

Table S3 Differential expression analysis between Adult Stem Cells (ASC) and NTERA-2 using LIMMA. Differentially expressed genes with B value>3 and Fold Change (FC)>2 (positive for ASC, and negative for NTERA-2, in log2) are shown. Genes marked in red were validated by Q-RT-PCR.
(0.76 MB XLS)

Table S4 Differential expression analysis between MSC-ADSC and MAPC using LIMMA. Differentially expressed genes with B value >3 and Fold Change (FC) >2 (positive for MSC and ADSC, and negative for MAPC, in log2) are shown. Genes marked in red were validated by Q-RT-PCR.
(0.59 MB XLS)

Table S5 Consensus ESC genes up-regulated in NTERA-2 in comparison with ASC. Table includes those genes with a FC>2 (in log2) and the PcG marks for ech gene, according to Lee's study. Genes marked in red were validated by Q-RT-PCR.
(0.11 MB XLS)

Table S6 A,Consensus differentiation genes up-regulated in ASC compared to NTERA-2. Table includes those genes with a FC>2 (log2) and the PcG marks for ech gene, according to Lee's study. Genes marked in red were validated by Q-RT-PCR. B, Consensus differentiation genes up-regulated in MSC-ADSC in comparison with MAPC. Table includes those genes with a FC>2 (log2) and the PcG marks for ech gene, according to Lee's study. Genes marked in red were validated by Q-RT-PCR.
(0.10 MB XLS)

Table S7 Bead-Array's raw data
(0.64 MB XLS)

Table S8 A, Consensus ESC gene promoters in BeadArray (Illumina). B, Consensus differentiation gene promoters in BeadArray (Illumina)
(0.18 MB XLS)

Table S9 Differentially methylated (DM) probe-sets between populations of stem cells. The type of promoter according to its CpG's content (LCP: low CpG content; ICP: intermediate CpG content and HCP: high CpG content), the annotation assigned by Assou et al. (ESC gene = ESC or Differentiation gene = Dif), BeadArray's data and PcG marks according to Lee et al, are indicated for each probe-set. Blue represents hypomethylated genes and yellow hypermethylated genes. A, DM Genes between NTERA-2 and ASC. B, Genes DM between NTERA-2 and MAPC. C, Genes DM between NTERA-2 and MSC. D, Genes DM between NTERA-2 and ADSC. E, Genes DM between MAPC and MSC. F, Genes DM between MAPC and ADSC. G, Genes DM between MSC and ADSC
(0.09 MB PDF)

Table S10 Differentially Methylated and Expressed Genes between NTERA-2 and ASC
(0.09 MB XLS)

Table S11 DcT (cT miRNA-cT RNU6B) of 250 miRNAs analyzed in ESC and ASC
(0.12 MB XLS)

Table S12 Differentially expressed miRNAs between NTERA-2 and ASC. Logarithmic values (log10) of those microRNAs which expression is significantly reduced/increased in ASC vs NTERA-2 cell line. These values were obtained comparing the expression value of each sample by the average value of NTERA_2 samples. A, miRNAs up-regulated in NTERA-2 and down-regulated in ASC. B, miRNAs up-regulated in ASC and down-regulated in NTERA-2.
(0.01 MB PDF)
