# Supplementary material for: Epigenetic Signatures Associated with Different Levels of Differentiation Potential in Human Stem Cells
Source: PLoS One. 2009 Nov 13;4(11):e7809. doi: 10.1371/journal.pone.0007809 (PMC2771914; doi:10.1371/journal.pone.0007809)
Supplement: Text S1 — Supplemental Material and Methods (0.11 MB DOC) [file pone.0007809.s001.doc]

**SUPPLEMENTAL MATERIALS AND METHODS**

**Cell populations**

Human mesenchymal stromal cells (MSC) were established from bone marrow from patients of 20-60 years of age. The mononuclear cell fraction was isolated by Ficoll density gradient centrifugation (Ficoll-Paque, GE Healthcare Bio-Sciences, Uppsala, Sweden), and treated with erythrocyte lysis buffer (155 mM NH_4_Cl, 10 mM KHCO_3_ and 0.1 mM EDTA). A total of 20-60 x 10^6^ mononuclear cells were seeded in T-175 flasks with growth medium, which contained αMEM without ribonucleosides (Gibco-Invitrogen, Paisely, UK), 10% fetal calf serum (FCS) (Biochrom, Berlin, Germany), 1% Pen/Strep (BioWhittaker-Lonza, Verviers, Belgium) and 1 ng/ml human fibroblast growth factor (bFGF or FGF-2) (Sigma, St. Louis, MO, USA). Human adipose-derived stem cells (ADSC) were obtained from human liposuction procedures. Lipoaspirates were digested with 2 mg/mL collagenase-A (Roche Diagnostics, Mannheim, Germany) for 45 min at 37ºC with constant shaking, followed by filtration through a 100-µm filter. After washing with PBS, cells were treated with erythrocyte lysis buffer. Resultant cells were cultivated at 1,000 cells/cm^2^ in the same medium as described for MSC. Multipotent adult progenitor cells (MAPC) were established as described (1). Briefly, the mononuclear fraction was isolated from the bone marrow by Ficoll density gradient centrifugation and plated at a density of 1 x 10^6^ cells per well in six-well plates in the presence of MAPC media containing 58% low-glucose DMEM (DMEM with 1 g/L glucose; Gibco-Invitrogen), 40% MCDB-201 (Sigma), 2% fetal calf serum (FCS) (Biochrom), ITS+1, 10^-8^ M dexamethasone, 10^-8^M ascorbic acid 2-phosphate (Sigma), 1% Pen/Strep (BioWhittaker) and 10 ng/ml of platelet-derived growth factor beta (PDGF-B) and epidermal growth factor (EGF) (R&D Systems, Minneapolis, MN, USA). MAPC and MSC isolates were obtained from the same patients. MAPC, ADSC and MAPC were referred as adult stem cells (ASC). All samples were obtained after the donor had given their informed consent, in accordance with the guidelines of the Committee on the Use of Human Subjects in Research of the Clinica Universitaria, Pamplona.

The human NTERA-2 cell line was purchased from DSMZ-German Collection of Microorganisms and Cell Cultures (Braunschweig, Germany), and maintained in high-glucose DMEM (DMEM with 4 g/L glucose; Gibco-Invitrogen) supplemented with 5% horse serum (Gibco-Invitrogen), 10% FCS (Biochrom), and 1% Pen/Strep (BioWhittaker).

**Fluorescence-activated cell sorting analysis**

For fluorescence-activated cell sorting (FACS) analysis, cells were detached with 0.05% trypsin-EDTA and washed with PBS. The following antibodies were used: HLA-DR, DP, DQ-PE, HLA-A,B,C-PE, CD44-PE, CD73-PE, CD90-APC, CD140b-PE, CD29-FITC, CD13-PE, CD31-PE, CD45-PE, CD34-PE (all from BD Pharmingen), CD105-PE (Ancell, Bayport, MN, USA), and CD117-PE (Caltag Lab, Burlingame, CA, USA), and their corresponding isotype controls (all from BD Pharmingen). Between 100,000 and 200,000 cells were incubated with primary antibody directly coupled to FITC, APC or phycoerythrin (PE) for 15 min in the dark at room temperature. Cells were fixed with 4% paraformaldehyde at 4°C.

***In vitro* differentiation**

To induce osteogenic differentiation, cells were replated at 6,000 cells/cm^2^ and cultured for three weeks in αMEM with 10 mM b-glycerophosphate (Fluka-BioChemika, Buchs, Switzerland), 0.2 mM ascorbic acid, 0.1 µM dexamethasone, 10% FCS and 1% Pen/Strep. After 21 days cells were analyzed by alkaline phosphatase and alizarin red staining. To induce adipogenic differentiation, cells were cultured, after they reached 90-100% confluence, in αMEM with 50 µM indomethacin, 0.5 mM isobutyl-methylxanthine (IBMX), 1 µM dexamethasone, 10% FCS and 1% Pen/Strep for 21 days. Oil Red-O staining was performed to identify adipocytes. To induce chondrogenic differentiation, 2 x 10^5^ cells were placed in a 15-ml conical tube to allow aggregation of the cells in micromass suspension culture, and kept for 21 days in DMEM-HG (DMEM with 4 g/L glucose; Gibco-Invitrogen) supplemented with 1% ITS+ Premix (BD Biosciences; consisting of 6.25 µg/mL insulin, 6.25 µg/mL transferrin, 6.25 ng/mL selenious acid, 1.25 mg/mL serum albumin, and 5.35 µg/mL linoleic acid), 100 µg/ml sodium pyruvate, 50 µg/mL ascorbate-2-phosphate, 0.1 µM dexamethasone, 500 ng/ml BMP6, 10 ng/ml TGFβ-3 and 1% Pen/Strep. After three weeks, the degree of differentiation was analyzed by staining the micromasses with toluidine blue in 5 µm sections. In all cases, media were changed every 3-4 days.

**Expression microarrays**

MAPC (n = 10), MSC (n = 8), ADSC (n = 5) and NTERA-2 cells (n = 3) were used for the microarray analysis. RNA isolation, labeling and hybridization to the HG-U133 Plus 2.0 GeneChip Oligonucleotide Microarray (Affymetrix Inc., Santa Clara, CA) were performed as previously described (2). Total RNA was extracted with Ultraspec (Biotecx, Houston, TX,) following the manufacturer’s instructions. RNA integrity was assessed using Agilent 2100 Bioanalyzer (Agilent, Palo Alto, CA). Samples were labeled and hybridized according to protocols from Affymetrix. Briefly, 100 ng of total RNA were amplified and labeled using the GeneChip two-cycle cDNA synthesis kit and GeneChip IVT labeling kit (Affymetrix Inc., Santa Clara, CA) and then hybridized to Human Genome U133 Plus 2.0 microarray (Affymetrix), after quality checking on GeneChips Test3 Arrays. Material was washed and scanned using a Fluidics Station 400 and a GeneChip Scanner (Affymetrix Inc.).

**Microarray data analysis**

*Principle components analysis:* Principal components analysis (PCA) was used to reveal trends in the data and to identify predominant gene expression patterns. Data used for this study were normalized using the dCHIP algorithm. The mean of mean intensity values for all chips was used to normalize the mean intensity of each chip. Probes with at least a two-fold differential expression in one group with respect to any other group were considered differentially expressed. Statistical significance of differential expression was determined using the Significant Analysis of Microarrays (SAM) algorithm. The log-transformed and mean-centered intensity values of significant differentially expressed genes were used for PCA, which was carried out using Spotfire (Spotfire Inc., Cambridge, MA).

*Data pre-processing and differential expression analysis:* Data were background-corrected and normalized using the RMA (Robust Multichip Average) algorithm (3), then filtered to eliminate low-expression probe sets. Applying the criterion of an expression value greater than 6.4 in all samples, 42,459 probe sets were selected for the statistical analysis. LIMMA (Linear Models for Microarray Data) (4) was used to identify the probe sets with significant differential expression between experimental conditions. Genes were selected as significant using a B statistic cut-off (B > 3). A second supervised analysis was performed using SAM to identify significant changes in the expression of a set of genes selected from the relevant research literature (5, 6). All possible comparisons were done using the two-class (unpaired) format. Significant genes were selected using the lowest false-discovery ratio, and only those with a more than two-fold change in each comparison were included in the analysis.

**Gene function analysis**

Significant probe sets identified in the LIMMA analysis were functionally annotated and grouped according to their gene ontology (GO) descriptions. Functionally enriched GO categories was determined by the standard hypergeometric test (7) using Bioconductor packages. Additional functional analysis was performed with WebGestalt using the list of probe sets with a greater than two-fold in each contrast as the input. The categories that proved to be significant in both analyses were studied further.

**Quantitative RT-PCR analysis**

RNA samples used for the Q-RT-PCR were the same as those used for hybridizing Affymetrix HG-U133 Plus 2.0 array. The first-strand cDNA was synthesized from l µg total RNA using random primers and MMLV retrotranscriptase. PCRs were performed using MJ Opticon (Bio Rad, Hercules, CA) and all reactions were performed in triplicate. Primers and probes were designed by Primer Express 2.0 Software (Applied Biosystems, Foster City, CA) and synthesized by Invitrogen. All primers were designed to overlay a junction between two exons to avoid hybridization to genomic DNA. Primers and probes used for real-time PCR are shown in Table S1. PCR amplifications were done in 12 µl reaction mixture for each sample, including 1 µl cDNA, 6 µl Taqman Master Mix 2x (Applied Biosystems), 0.5 µl mixture of primers (10 µM) and probe labeled with FAM (5 µM), and 4.5 µl distilled water. Cycling parameters were 95°C for 10 min followed by 40 cycles of 95°C for 15 s and 60°C for 1 min. Gene expression was calculated using the relative standard curve method. GAPDH was used as a housekeeping control.

**Chromatin immunoprecipitation assay**

A sample of each population studied in Affymetrix arrays was subjected to chromatin immunoprecipitation. ChIP assays were performed as previously described (8, 9) and the ChIP fractions were used for quantitative-PCR assay. Immunoprecipitated fractions were obtained with the antibodies anti-SUZ12 (ab12073), anti-EZH2 (ab3748), anti-trimethylated Lys 27 of histone 3 (H3K27me3: ab6002), anti-trimethylated Lys 4 of histone 3 (H3K4me3: ab8580) (all from Abcam, Cambridge, MA) and anti-acetylated histone H3 (H3Ac: 06-599, Upstate Biotechnologies, Lake Placid, NY, USA).

Quantitative-PCR assays were performed in order to assess the SUZ12, EZH2, H3K27me3, H3K4me3 and H3Ac modifications in the gene promoters of *FBN-1*, *IGFBP3*, *CDH11*, *SDF1*, *MMP2*, *EPAS1*, and *GATA6*. Immunoprecipitated DNA fractions (10 ng) from antibody-bound, no-antibody samples and input chromatin were analyzed by real-time PCR using SYBR Green detection and the LightCycler platform (Roche). PCR amplifications were performed with the primers described in Table S1. The amplification of the immunoprecipitation fraction (IP) was used as a target sequence. The following program conditions were applied for Q-PCR-Chip: denaturation program, consisting of one cycle at 95ºC for 10 min; amplification program, consisting of 45 cycles at 95ºC for 10 s, 60ºC for 10 s and 72ºC for 10 s; melting program, one cycle at 95ºC for 10 s, 40ºC for 60 s and 90ºC for 10 s; and cooling program, one cycle at 40ºC for 60 s. The temperature transition rate was 20ºC/s, except for the melting program, which was 0.2ºC/s between 40ºC and 90ºC. Amplification of the input DNA (In) was performed as a reference sequence. It was amplified in the same run and following the same procedure described above for IP. A procedure based on the relative quantification of target sequence (IP) vs. their controls/calibrators in relation to the reference sequence (In) was used to assess the levels of the different histone marks. Values were automatically calculated by the LightCycler program (RealQuant, version 1.0, Roche). Normalized ratios were calculated according to the following equation and expressed as a percentage of the control/calibrator:

Normalized ratio (N) = (E_target_)^ΔCp target (control – sample)^ ÷ (E_ref_)^ΔCp ref (control – sample)^

The efficiency (E) of each gene was calculated from the slopes of crossover points (Cp) versus DNA concentration plot, according to the formula E = 10^(-1/slope)^. ΔCp corresponds to the difference between control/calibrator Cp and sample Cp, either for the target or for the reference sequences. The selected control/calibrator was the NTERA-2 cell line, which was taken as 100%. The level of histone modifications relative to input chromatin was determined for each sample.

**DNA methylation profiling using universal BeadArrays**

DNA samples from NTERA-2 (n = 2), MAPC (n = 4), MSC (n = 4) and ADSC (n = 4) cells were analyzed. All samples, except MAPC, were the same as those used for the expression arrays. We used GoldenGate® Methylation Cancer Panel I (Illumina Inc., San Diego, CA, USA) for the DNA methylation analysis. The panel is developed to analyze 1,505 CpG sites selected from 807 genes, which include oncogenes and tumor suppressor genes, previously reported to be differentially methylated or differentially expressed genes, imprinted genes, genes involved in various signaling pathways, genes responsible for DNA repair, cell cycle control, metastasis, differentiation and apoptosis. Methylation assay was performed as described previously (10, 11). Briefly, bisulfite conversion of DNA samples was done using the CpGenomic^TM^ DNA Modification Kit (Intergen Company, Purchase, NY, USA). After sodium bisulfite treatment, the remaining assay steps were identical to the GoldenGate genotyping assay (12) using Illumina-supplied reagents and conditions. The array was hybridized under a temperature gradient program, and arrays were imaged using a BeadArray Reader (Illumina Inc.). Image processing and intensity data extraction were done as described previously (13, 14). Each methylation data point is represented by fluorescent signals from the M (methylated) and U (unmethylated) alleles. Background intensity computed from a set of negative controls was subtracted from each analytical data point. The ratio of fluorescent signals was then computed from the two alleles according to the following formula:

*Max(M,0)*

=

=

*Max(U,0)+Max(M,0)+100*

*β*

=

Hierarchical clustering was performed using the Cluster Analysis tool of the BeadStudio program (Illumina) excluding CpGs located on X-chromosomal genes (1,421 CpGs from 767 genes were included in the analysis). Differential methylation analysis (DMA) was performed using the BeadStudio program. MAPC, MSC and ADSC series were compared with NTERA-2 samples. Two criteria were used to detect significantly differentially methylated CpGs: a false-discovery rate (FDR) below 0.01 (Mann-Whitney U test), and mean beta values between NTERA-2 and ASC samples with a difference of at least 0.3. A CpG was classified as differentially methylated if either or both criteria were met.

**Sequencing after sodium bisulfite treatment**

To validate the DNA methylation data generated by the BeadArray technology, bisulfite sequencing (BS) was performed as previously described (15, 16). The methylation status of *COL1A2* (GeneBank: NM_000089), *HOXA9* (GeneBank: NM_152739) and *SERPINE1* (GeneBank: NM_000602) promoters were analyzed. The primer sequences used in BS PCR reactions were designed using Methprimer (<http://www.urogene.org/methprimer/>) and are shown in Table S1. After bisulfite modification *COL1A2* and *SERPINE1* promoters were amplified by nested PCR and *HOXA9* by single PCR. First, PCR was carried out using 3 µl modified DNA and BS1 and BS2 primers; the second PCR, to *COL1A2* and *SERPINE1*, used 1 µl of the first PCR product and BS3 and BS4 primers. Both PCR reactions were carried out in a total volume of 25 µl, with 1 U high-fidelity Platinum Taq DNA polymerase, 1.5 mM MgClR_2R_, 0.2 mM dNTPs and 50 pmol of each primer and were performed under the following conditions: 94°C for 10 min, X (number of cycles) cycles at 94°C for 1 min, annealing temperature (aT) for 1 min and 72°C for 1 min, and a final elongation cycle at 72ºC for 10 min. The PCR products were subcloned for sequencing using the pGEM®-T Easy plasmid and JM109 competent cells included in the pGEM®-T Easy Vector System II kit (Promega, Madison, WI, USA). Colonies with recombinant plasmids containing the described PCR products were screened by digestion with *Eco*R I (Takara Bio Inc., Otsu, Japan). A minimum of six candidate plasmid clones for each sample were sequenced with an ABI-PRISM™ d-Rhodamine Terminator Cycle Sequencing Kit (Applied Biosystems) in an ABI PRISM™ 377 DNA Sequencer (Applied Biosystems) using SP6 and T7 universal forward and reverse primers.

**MicroRNA expression analysis by quantitative real-time PCR (Q-RT-PCR)**

Expression of 250 microRNAs was analyzed using specific primers and TaqMan probe for each miRNA according to the TaqMan MicroRNA Assay protocol, as previously described (17). Briefly, Q-RT-PCR was performed in an Applied Biosystems 7300 Sequence Detection System, using 0.7 μl of RT product of each miRNA in a reaction volume of 10 μl with 1x TaqMan Universal PCR master mix and 1 μl mix of primers and probe according to the TaqMan MicroRNA Assay protocol (Applied Biosystems). The reactions were incubated in a 96-well optical plate at 95ºC for 10 min, followed by 40 cycles of 95ºC for 15s and 60ºC for 10 min. The Ct data were determined using default threshold settings. Expression of miRNAs was normalized using the expression of *RNU6B* gene in each sample. For expression of *RNU6B* we used TaqMan RNU6B assay (Part Number (PN): 4373381) (Applied Biosystems). Relative quantification of expression of microRNAs was calculated with the 2^-∆∆Ct^ method (Applied Biosystems. User Bulletin Nº2 (P/N 4303859)). The data are shown as log_10_ of the relative quantity (RQ) of target miRNAs, normalized and compared with expression in NTERA-2.

In order to identify microRNAs with statistically significant changes in expression between the groups, we performed a supervised analysis using the SAM algorithm. All data were permuted over 100 cycles by using the two-class (unpaired) format. miRNAs that could possibly target *ZIC3*, *NANOG*, *LIN28*, *DCN* and *COL1A2* were validated by Q-RT-PCR using the standard curve method (Applied Biosystems. User Bulletin N°2 (P/N 4303859). Individual TaqMan miRNA assays for *hsa-miR-137* (P/N: 4373174), *hsa-miR-152* (P/N: 4373126), *hsa-miR-154* (P/N: 4373270), *hsa-miR-155* (P/N: 4373124), *hsa-miR-199a* (P/N: 4373272), *hsa-miR-199b* (P/N: 4373100), *hsa-let-7c* (P/N: 4373167), *hsa-miR-*96 (P/N: 4373010), *hsa-miR-182* (P/N: 4373271), *hsa-miR-205* (P/N: 4373093) and *hsa-miR-367* (P/N: 4373034) were used (Applied Biosystems). The expression of *LIN28, ZIC3, NANOG, DCN* and *COL1A2* was also analyzed by means of Q-RT-PCR using the primers and conditions described in Table S1.

**REFERENCES**

1. Aranguren, X.L., A. Luttun, C. Clavel, C. Moreno, G. Abizanda, M.A. Barajas, B. Pelacho, M. Uriz, M. Arana, A. Echavarri, M. Soriano, E.J. Andreu, J. Merino, J.M. Garcia-Verdugo, C.M. Verfaillie, and F. Prosper. 2007. In vitro and in vivo arterial differentiation of human multipotent adult progenitor cells. *Blood* 109:2634-2642.

2. Jose-Eneriz, E.S., J. Roman-Gomez, L. Cordeu, E. Ballestar, L. Garate, E.J. Andreu, I. Isidro, E. Guruceaga, A. Jimenez-Velasco, A. Heiniger, A. Torres, M.J. Calasanz, M. Esteller, N.C. Gutierrez, A. Rubio, I. Perez-Roger, X. Agirre, and F. Prosper. 2008. BCR-ABL1-induced expression of HSPA8 promotes cell survival in chronic myeloid leukaemia. *Br J Haematol* 142:571-582.

3. Irizarry, R.A., B. Hobbs, F. Collin, Y.D. Beazer-Barclay, K.J. Antonellis, U. Scherf, and T.P. Speed. 2003. Exploration, normalization, and summaries of high density oligonucleotide array probe level data. *Biostatistics* 4:249-264.

4. Wettenhall, J.M., and G.K. Smyth. 2004. limmaGUI: a graphical user interface for linear modeling of microarray data. *Bioinformatics* 20:3705-3706.

5. Assou, S., T. Le Carrour, S. Tondeur, S. Strom, A. Gabelle, S. Marty, L. Nadal, V. Pantesco, T. Reme, J.P. Hugnot, S. Gasca, O. Hovatta, S. Hamamah, B. Klein, and J. De Vos. 2007. A meta-analysis of human embryonic stem cells transcriptome integrated into a web-based expression atlas. *Stem Cells* 25:961-973.

6. Adewumi, O., B. Aflatoonian, L. Ahrlund-Richter, M. Amit, P.W. Andrews, G. Beighton, P.A. Bello, N. Benvenisty, L.S. Berry, S. Bevan, B. Blum, J. Brooking, K.G. Chen, A.B. Choo, G.A. Churchill, M. Corbel, I. Damjanov, J.S. Draper, P. Dvorak, K. Emanuelsson, R.A. Fleck, A. Ford, K. Gertow, M. Gertsenstein, P.J. Gokhale, R.S. Hamilton, A. Hampl, L.E. Healy, O. Hovatta, J. Hyllner, M.P. Imreh, J. Itskovitz-Eldor, J. Jackson, J.L. Johnson, M. Jones, K. Kee, B.L. King, B.B. Knowles, M. Lako, F. Lebrin, B.S. Mallon, D. Manning, Y. Mayshar, R.D. McKay, A.E. Michalska, M. Mikkola, M. Mileikovsky, S.L. Minger, H.D. Moore, C.L. Mummery, A. Nagy, N. Nakatsuji, C.M. O'Brien, S.K. Oh, C. Olsson, T. Otonkoski, K.Y. Park, R. Passier, H. Patel, M. Patel, R. Pedersen, M.F. Pera, M.S. Piekarczyk, R.A. Pera, B.E. Reubinoff, A.J. Robins, J. Rossant, P. Rugg-Gunn, T.C. Schulz, H. Semb, E.S. Sherrer, H. Siemen, G.N. Stacey, M. Stojkovic, H. Suemori, J. Szatkiewicz, T. Turetsky, T. Tuuri, S. van den Brink, K. Vintersten, S. Vuoristo, D. Ward, T.A. Weaver, L.A. Young, and W. Zhang. 2007. Characterization of human embryonic stem cell lines by the International Stem Cell Initiative. *Nat Biotechnol* 25:803-816.

7. Segura, V., A. Podhorski, E. Guruceaga, J.L. Sevilla, F.J. Corrales, and A. Rubio. 2006. GARBAN II: an integrative framework for extracting biological information from proteomic and genomic data. *Proteomics* 6 Suppl 1:S12-15.

8. Lujambio, A., S. Ropero, E. Ballestar, M.F. Fraga, C. Cerrato, F. Setien, S. Casado, A. Suarez-Gauthier, M. Sanchez-Cespedes, A. Gitt, I. Spiteri, P.P. Das, C. Caldas, E. Miska, and M. Esteller. 2007. Genetic unmasking of an epigenetically silenced microRNA in human cancer cells. *Cancer Res* 67:1424-1429.

9. Roman-Gomez, J., X. Agirre, A. Jimenez-Velasco, V. Arqueros, A. Vilas-Zornoza, P. Rodriguez-Otero, I. Martin-Subero, L. Garate, L. Cordeu, E.S. Jose-Eneriz, V. Martin, J.A. Castillejo, E. Bandres, M.J. Calasanz, R. Siebert, A. Heiniger, A. Torres, and F. Prosper. 2009. Epigenetic Regulation of MicroRNAs in Acute Lymphoblastic Leukemia. *J Clin Oncol*

10. Bibikova, M., E. Chudin, B. Wu, L. Zhou, E.W. Garcia, Y. Liu, S. Shin, T.W. Plaia, J.M. Auerbach, D.E. Arking, R. Gonzalez, J. Crook, B. Davidson, T.C. Schulz, A. Robins, A. Khanna, P. Sartipy, J. Hyllner, P. Vanguri, S. Savant-Bhonsale, A.K. Smith, A. Chakravarti, A. Maitra, M. Rao, D.L. Barker, J.F. Loring, and J.B. Fan. 2006. Human embryonic stem cells have a unique epigenetic signature. *Genome Res* 16:1075-1083.

11. Martin-Subero, J.I., M. Kreuz, M. Bibikova, S. Bentink, O. Ammerpohl, E. Wickham-Garcia, M. Rosolowski, J. Richter, L. Lopez-Serra, E. Ballestar, H. Berger, X. Agirre, H.W. Bernd, V. Calvanese, S.B. Cogliatti, H.G. Drexler, J.B. Fan, M.F. Fraga, M.L. Hansmann, M. Hummel, W. Klapper, B. Korn, R. Kuppers, R.A. Macleod, P. Moller, G. Ott, C. Pott, F. Prosper, A. Rosenwald, C. Schwaenen, D. Schubeler, M. Seifert, B. Sturzenhofecker, M. Weber, S. Wessendorf, M. Loeffler, L. Trumper, H. Stein, R. Spang, M. Esteller, D. Barker, D. Hasenclever, and R. Siebert. 2008. New insights into the biology and origin of mature aggressive B-cell lymphomas by combined epigenomic, genomic and transcriptional profiling. *Blood*

12. Fan, J.B., A. Oliphant, R. Shen, B.G. Kermani, F. Garcia, K.L. Gunderson, M. Hansen, F. Steemers, S.L. Butler, P. Deloukas, L. Galver, S. Hunt, C. McBride, M. Bibikova, T. Rubano, J. Chen, E. Wickham, D. Doucet, W. Chang, D. Campbell, B. Zhang, S. Kruglyak, D. Bentley, J. Haas, P. Rigault, L. Zhou, J. Stuelpnagel, and M.S. Chee. 2003. Highly parallel SNP genotyping. *Cold Spring Harb Symp Quant Biol* 68:69-78.

13. Galinsky, V.L. 2003. Automatic registration of microarray images. II. Hexagonal grid. *Bioinformatics* 19:1832-1836.

14. Galinsky, V.L. 2003. Automatic registration of microarray images. I. Rectangular grid. *Bioinformatics* 19:1824-1831.

15. Roman-Gomez, J., A. Jimenez-Velasco, L. Cordeu, A. Vilas-Zornoza, E. San Jose-Eneriz, L. Garate, J.A. Castillejo, V. Martin, F. Prosper, A. Heiniger, A. Torres, and X. Agirre. 2007. WNT5A, a putative tumour suppressor of lymphoid malignancies, is inactivated by aberrant methylation in acute lymphoblastic leukaemia. *Eur J Cancer* 43:2736-2746.

16. Agirre, X., J. Roman-Gomez, A. Jimenez-Velasco, L. Garate, C. Montiel-Duarte, G. Navarro, I. Vazquez, M. Zalacain, M.J. Calasanz, A. Heiniger, A. Torres, J.D. Minna, and F. Prosper. 2006. ASPP1, a common activator of TP53, is inactivated by aberrant methylation of its promoter in acute lymphoblastic leukemia. *Oncogene* 25:1862-1870.

17. Agirre, X., A. Jimenez-Velasco, E. San Jose-Eneriz, L. Garate, E. Bandres, L. Cordeu, O. Aparicio, B. Saez, G. Navarro, A. Vilas-Zornoza, I. Perez-Roger, J. Garcia-Foncillas, A. Torres, A. Heiniger, M.J. Calasanz, P. Fortes, J. Roman-Gomez, and F. Prosper. 2008. Down-Regulation of hsa-miR-10a in Chronic Myeloid Leukemia CD34+ Cells Increases USF2-Mediated Cell Growth. *Mol Cancer Res* 6:1830-1840.
