# Supplementary figures and images for: Epigenetic Signatures Associated with Different Levels of Differentiation Potential in Human Stem Cells
Source: PLoS One. 2009 Nov 13;4(11):e7809. doi: 10.1371/journal.pone.0007809 (PMC2771914; doi:10.1371/journal.pone.0007809)

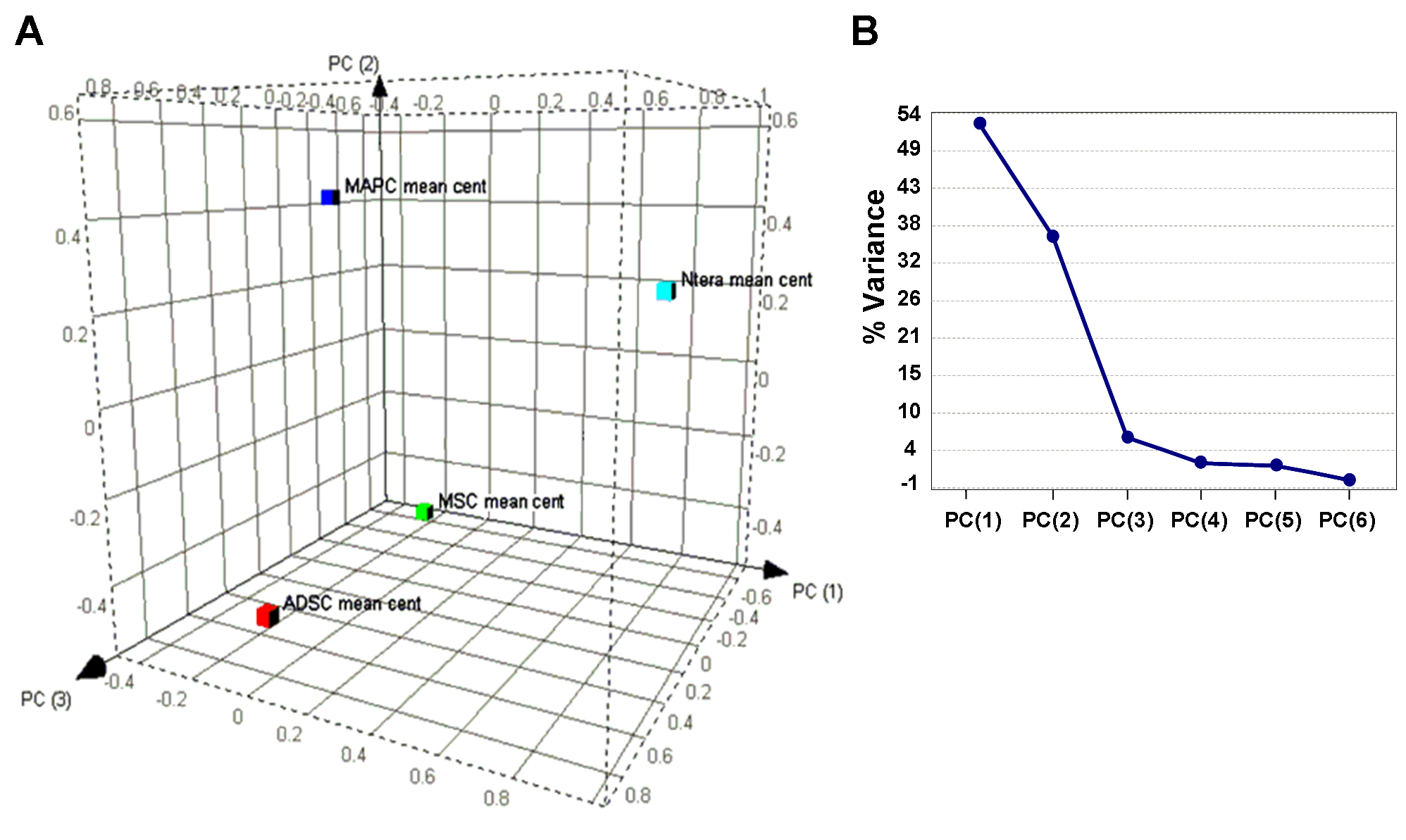

Supplement: Figure S1 — Exploratory data analysis with PCA method on the gene expression data of MSC, ADSC, MAPC and NTERA-2 A. Samples plotted in the first three principal components (PC); B. Variation captured in each PC (1.13 MB TIF) [file pone.0007809.s002.tif]

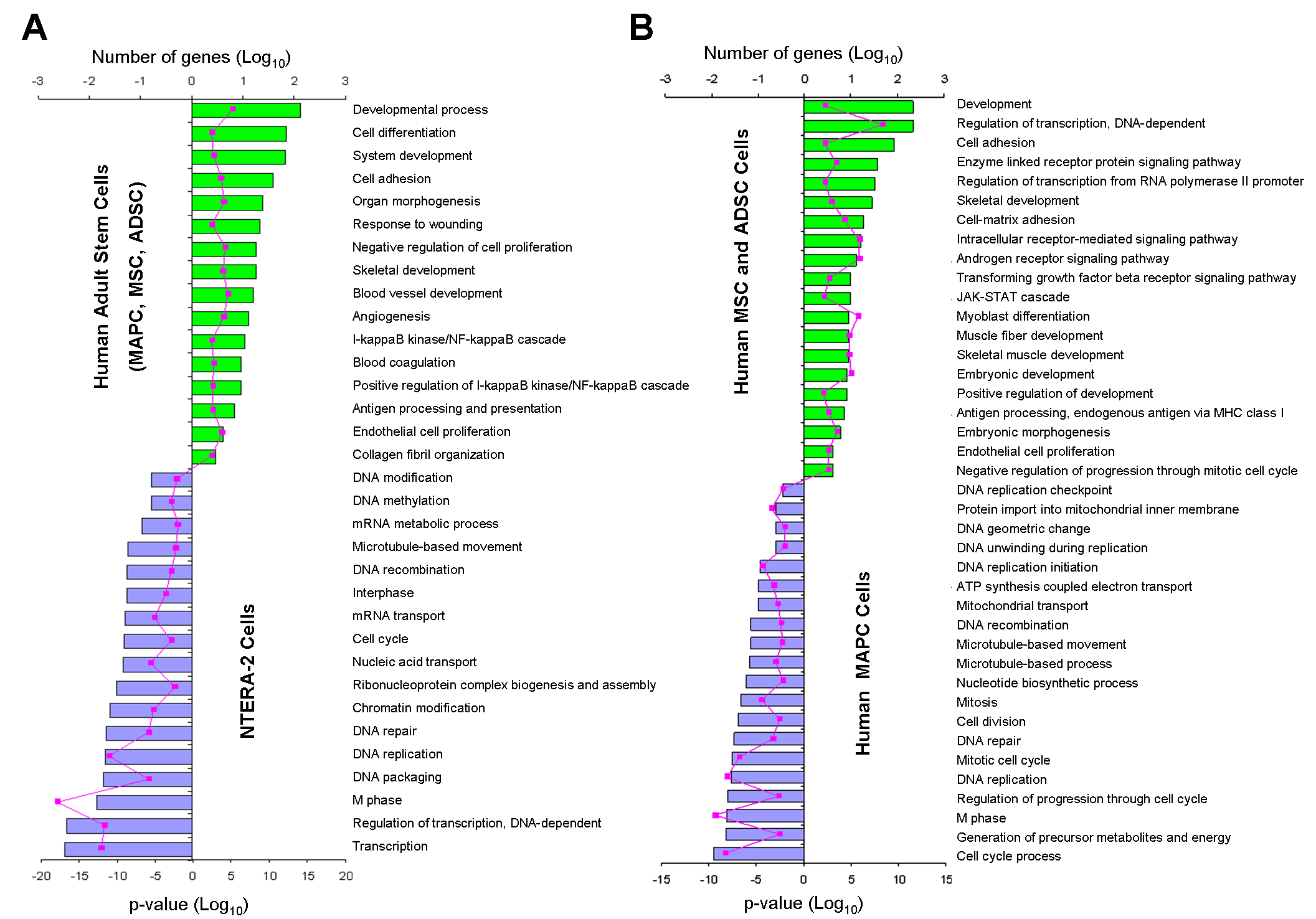

Supplement: Figure S2 — Functional analysis of differentially expressed probe-sets between stem cell populations Comparison between the gene expression profile of NTERA-2 versus Adult Stem Cells (A) and MAPC versus MSC-ADSC cells (B). The graphs show categories overrepresented in each group according to their p-value (line, in log10) and the number of genes in each category (bars, in log10). Only categories with a p-value less than 0.01 were selected (0.54 MB TIF) [file pone.0007809.s003.tif]

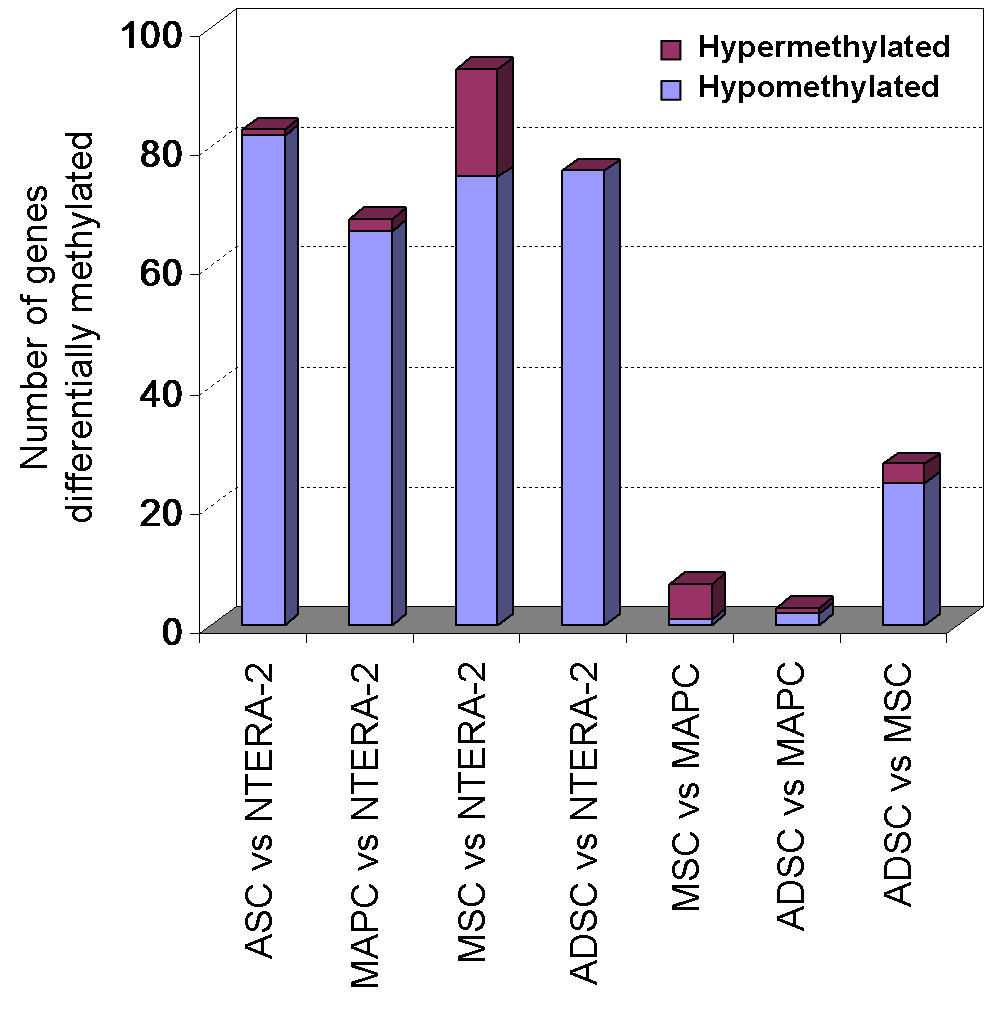

Supplement: Figure S3 — DNA methylation in stem cells Number of genes differentially methylated in each comparison from supervised analysis. The number of genes hypomethylated and hypermethylated for each comparison is shown. (0.15 MB TIF) [file pone.0007809.s004.tif]

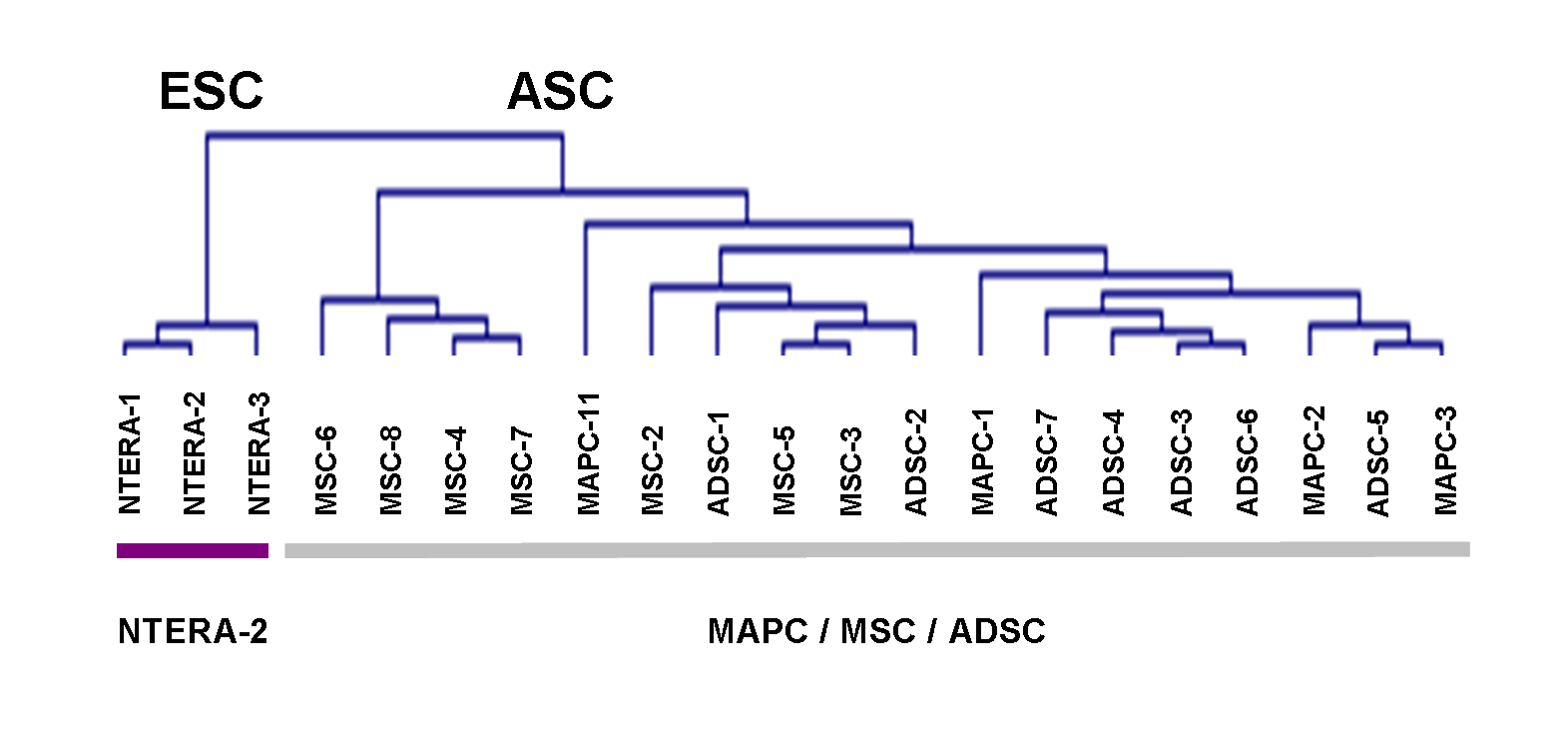

Supplement: Figure S4 — Dendrogram of hierarchical cluster analysis based on 250 miRNA expression data. ESC, human embryonal carcinoma; ASC, human adult stem cells; MAPC, Multipotent Adult Progenitor Cells; MSC, Mesenchymal Stem Cells; ADSC, Adipose-Derived Stem Cells. (0.20 MB TIF) [file pone.0007809.s005.tif]

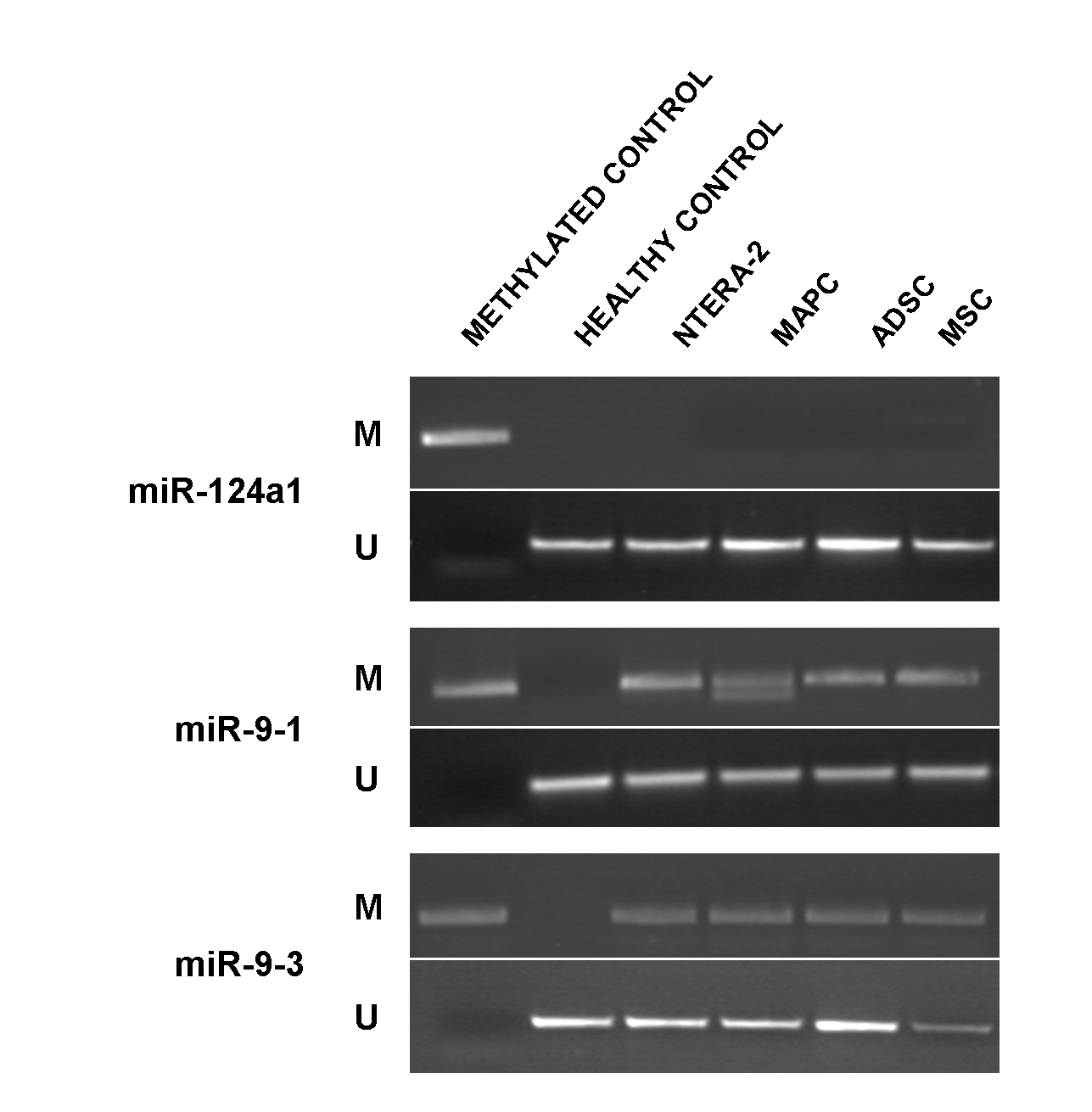

Supplement: Figure S5 — Promoter hypermethylation of miR-9-1, miR-9-3 and -miR-124a1 MSP analysis of the miR-124a-1, miR-9-1 and miR-9-3 CpG island regions in NTERA-2 and ASC. MAPC, Multipotent Adult Progenitor Cells; MSC, Mesenchymal Stem Cells; ADSC, Adipose-Derived Stem Cells. M: methylated allele; U: un-methylated allele. (0.63 MB TIF) [file pone.0007809.s006.tif]
