# Supplementary material for: Epigenetic Signatures Associated with Different Levels of Differentiation Potential in Human Stem Cells
Source: PLoS One. 2009 Nov 13;4(11):e7809. doi: 10.1371/journal.pone.0007809 (PMC2771914; doi:10.1371/journal.pone.0007809)
Supplement: Table S1 — Primers and probes used for PCR. X: cycles of PCR; aT: annealing Temperature. (0.06 MB DOC) [file pone.0007809.s007.doc]

|  | **Primers used for Real Time RT-PCR** | | |
| --- | --- | --- | --- |
|  | **Sense** | **Antisense** | **Probe** |
| ***GAPDH*** | TGGTATCGTGGAAGGACTCATGAC | ATGCCAGTGAGCTTCCCGTTCAGC | CCCAGAAGACTGTGGATGGCCCC |
| ***ZIC3*** | CAAAGTGTGCGACAAGTCCTACA | GGGAGGAATCTGACCCTTGAG | CACCCGAGCTCCCTGCGCA |
| ***SOX2*** | CACTGCCCCTCTCACACATG | CCCATTTCCCTCGTTTTTCTT | AGGGCCGGACAGCGAACTGG |
| ***LIN28*** | CCCCCAGTGGATGTCTTTGT | GCCTCACCCTCCTTCAAGCT | AGTAAGCTGCACATGGAAGGGTTCC |
| ***NANOG*** | TTTGTGGGCCTGAAGAAAAC | CAGATCCATGGAGGAAGGAA | AGATGCCTCACACGGAGACT |
| ***CDH11*** | GCCCTGATCGCCATCCT | CCTTCTCAGGGTCACAAACAATACT | CCTGCATCGTCATTCTCCTGGTCATTG |
| ***EFEMP1*** | GAAAATGGAGAGTTCTACCTACGACAA | TGTTCTCTTGGTCCTGATAATGACTTC | AAGTCCTGTAAGTGCAATGCTTGTGCTCG |
| ***FBN1*** | TCTGAGACAGAAGCCAATGTGAGT | CGTGGGAAATATTGAAAGCAAAG | TGCAAGTTGGGATGTTGAGAAGACAGCC |
| ***IGFBP3*** | AGGAGGACGTGCACTGCTACA | ATAAGGCATATTTGAGCTCCACATTA | CAGAGCAAGTAGACGCCTGCCGC |
| ***LUM*** | CCTGGAGGTCAATCAACTTGAGA | AATGCTTGATCTTGGAGTAGGATAATG | TGACATAAAGAGCTTCTGCAAGATCCTGGG |
| ***MMP2*** | TCACTCCTGAGATCTGCAAACAG | TCACAGTCCGCCAAATGAAC | ACATTGTATTTGATGGCATCGCTCAGATCC |
| ***GATA6*** | ATACTTCCCCCACAACACAACCT | TCTCGGGATTGGTGCTCTCT | CCCGGTGATGACTGGTGCG |
| ***SDF1*** | GCTGAAGAACAACAACAGACAAGTG | TGACCCTCTCACATCTTGAACCT | CATTGACCCGAAGCTAAAGTGGATTCAGG |
| ***EPAS1*** | GACTCCGAGAACATGACCAAGAG | CATCCGGTACTGGCCACTTAC | CCAGAACTTGTGCACCAAGGGTCAGGT |
| ***COL1A2*** | TGAGACTCAGCCACCCAGAGT | TGGCTTCCATAGTGCATCCTT | AGCAGCGGTTACTACTGGATTGACCCCA |
| ***DCN*** | TGGGCTGGCAGAGCATAAG | GCAGAAGTCACTTGATCCAACTACAG | CATCCAGGTTGTCTACCTTCATAACAA |
|  | **Primers used for qPCR in ChIP assays** | | |
| ***CDH11*** | CTCTGCGAGGCCCATCTC | AGTGGCAGGAATGAGAAACC | SYBER GREEN |
| ***FBN1*** | CTGATCACCTCTGCCTCCTC | AAAATGACACCCCCTCCTTC | SYBER GREEN |
| ***IGFBP3*** | GAGAGCGGAAGGGGTAAGG | CGGGTCACCTTGTCGTCTAC | SYBER GREEN |
| ***SDF1*** | CACTTTACGCCTAAGGTCCTC | GGCCTTTGACCTTCTCAGG | SYBER GREEN |
| ***MMP2*** | AAGGGCCTAGAGCGACAGAT | TCCTTTCTCCACCTCTTTTCC | SYBER GREEN |
| ***EPAS1*** | TTTCCTTGCACTGCTTCTCC | GCCAGGTGGGGATAATTTCT | SYBER GREEN |
| ***GATA6*** | GTCGCTAGCCAGGTCAGG | CCAGGCAGACAATGAGAGC | SYBER GREEN |
| ***COL1A2*** | CGCCACGCTATCGAGTCT | CAGTCCCGCCTCACCTGT | SYBER GREEN |
| ***HOXA9*** | CGTTGGCCACAATTAAAACA | AAATCACTCCGCACGCTATT | SYBER GREEN |
| ***SERPINE1*** | AGCAGCACACACACACACAC | CACCCACTCACTGGCTCTG | SYBER GREEN |
|  | **Primers used for Bisulphite Sequencing assays** | | |
| ***HOXA9*** | BS1:TTTGTGTGGTTTTTGAAATAATAATTTT | BS2:AAAATCAAATCTAACCTTATCTCTATACTC | X: 36 aT: 55ºC |
| ***COL1A2***  ***Nested*** | BS1:GTTTTAAAGTTAGAGAAAAGTTGGA | BS2:ACAACCACAACTAAAAAAAACCTAC | X: 30 aT: 55ºC |
|  | BS3:AGTGGTTTATAGGGTATAGGTGAGG | BS4:ACACTTTTAAAACTTTCAAAAAAAA | X: 26 aT: 55ºC |
| ***SERPINE1***  ***Nested*** | BS1:TAGGGGTATAGAGAGAGTTTGGATA | BS2:AACCCCAATAACCTTAACCTAAAAA | X: 30 aT: 56ºC |
|  | BS3:GTTGGTTGTATGTTTTGTGGTTGT | BS4:CCCTCTACCTATATCTATCTCTCCC | X: 26 aT: 57ºC |
