# Supplementary material for: Epigenetic Signatures Associated with Different Levels of Differentiation Potential in Human Stem Cells
Source: PLoS One. 2009 Nov 13;4(11):e7809. doi: 10.1371/journal.pone.0007809 (PMC2771914; doi:10.1371/journal.pone.0007809)
Supplement: Table S9 — Differentially methylated (DM) probe-sets between populations of stem cells. The type of promoter according to its CpG's content (LCP: low CpG content; ICP: intermediate CpG content and HCP: high CpG content), the annotation assigned by Assou et al. (ESC gene = ESC or Differentiation gene = Dif), BeadArray's data and PcG marks according to Lee et al, are indicated for each probe-set. Blue represents hypomethylated genes and yellow hypermethylated genes. A, DM Genes between NTERA-2 and ASC. B, Genes DM between NTERA-2 and MAPC. C, Genes DM between NTERA-2 and MSC. D, Genes DM between NTERA-2 and ADSC. E, Genes DM between MAPC and MSC. F, Genes DM between MAPC and ADSC. G, Genes DM between MSC and ADSC (0.09 MB PDF) [file pone.0007809.s015.pdf]

Probe Sets				DNA Methylation			PcG Occupation (Lee, 2006)
TargetID	ProbeID	Promotor-Class	ESC/Dif. (Assou, 2007)	NTERA2. AVG_Beta	ASC. AVG_Beta	Δ ASC-NTERA2	
GUCY2D_E419_R	2999	ICP		0,959	0,030	-0,929	+
RASSF1_E116_F	3865	HCP		0,950	0,032	-0,918	ND
HGF_E102_R	2755	LCP		0,952	0,036	-0,915	–
STAT5A_E42_F	5736	LCP		0,963	0,051	-0,913	–
HTR2A_P853_F	1105	LCP		0,944	0,037	-0,907	–
COL1A1_P5_F	3253	ICP	Dif	0,946	0,040	-0,907	ND
COL1A2_P48_R	315	ICP	Dif	0,946	0,046	-0,901	–
SCGB3A1_E55_R	3888	NA		0,967	0,069	-0,898	–
DDR2_P743_R	4305	LCP	Dif	0,927	0,031	-0,895	–
RASSF1_P244_F	1836	HCP		0,923	0,028	-0,895	ND
DDR2_E331_F	5516	LCP	Dif	0,961	0,067	-0,894	–
RARB_P60_F	2853	LCP		0,929	0,039	-0,891	–
P2RX7_E323_R	2854	LCP		0,946	0,059	-0,887	–
ASCL2_P609_R	105	HCP		0,968	0,087	-0,881	+
NPY_P295_F	1644	HCP		0,964	0,092	-0,871	–
COL1A2_E299_F	3083	ICP	Dif	0,916	0,064	-0,852	–
SEPT5_P464_R	5997	NA		0,935	0,083	-0,852	–
SEPT9_P374_F	6004	NA		0,886	0,038	-0,848	–
FASTK_P598_R	4874	NA		0,930	0,083	-0,847	–
SNCG_P53_F	2022	LCP		0,916	0,070	-0,846	–
JAK3_P156_R	4990	ICP		0,951	0,110	-0,842	–
DLC1_P88_R	2431	LCP	Dif	0,928	0,093	-0,835	+
SEMA3B_E96_F	3894	LCP		0,894	0,059	-0,835	+
IFNGR2_P377_R	4125	ICP		0,905	0,073	-0,832	–
SEPT5_P441_F	5988	NA		0,897	0,066	-0,832	–
RARB_E114_F	4165	LCP		0,852	0,023	-0,829	–
HCK_P858_F	5768	HCP		0,956	0,135	-0,821	–
ASCL2_P360_F	103	HCP		0,900	0,081	-0,819	+
MOS_E60_R	4133	ICP		0,875	0,058	-0,817	–
IRF7_E236_R	3520	HCP		0,859	0,043	-0,816	–
HHIP_P578_R	2230	HCP		0,853	0,039	-0,814	+
IGF1_E394_F	648	LCP		0,869	0,054	-0,814	–

SCGB3A1_P103_R	1883	NA		0,920	0,117	-0,802	–
SEMA3B_P110_R	1886	LCP		0,843	0,045	-0,798	+
COL1A1_P117_R	3366	ICP	Dif	0,859	0,064	-0,795	ND
MPO_P883_R	2373	LCP		0,872	0,078	-0,794	–
MLF1_E243_F	891	NA		0,824	0,041	-0,784	–
ASCL2_E76_R	3019	HCP		0,859	0,078	-0,782	+
IL16_P226_F	1171	LCP		0,907	0,125	-0,781	–
NAT2_P11_F	4177	LCP		0,947	0,177	-0,770	–
IL16_P93_R	1173	LCP		0,830	0,065	-0,765	–
DES_E228_R	5517	HCP		0,830	0,070	-0,760	–
FAS_P322_R	4870	NA		0,820	0,063	-0,757	–
MLF1_P97_F	4276	NA		0,786	0,032	-0,753	–
JAK3_E64_F	5615	ICP		0,790	0,038	-0,752	–
HLA-DRA_P132_R	1044	LCP		0,883	0,136	-0,747	–
FANCE_P356_R	4848	HCP		0,839	0,097	-0,742	–
SEPT9_P58_R	6002	NA		0,792	0,051	-0,741	–
CPA4_E20_F	3087	LCP		0,793	0,053	-0,740	–
P2RX7_P119_R	2170	LCP		0,835	0,105	-0,730	–
PDGFRB_P273_F	2810	LCP	Dif	0,806	0,076	-0,730	–
FRZB_E186_R	623	ICP	Dif	0,752	0,031	-0,721	–
IGF1_P933_F	4132	LCP		0,757	0,041	-0,715	–
IRF7_P277_R	1227	HCP		0,747	0,039	-0,709	–
IHH_E186_F	5583	HCP		0,742	0,038	-0,705	+
PADI4_P1158_R	1658	LCP		0,906	0,202	-0,705	–
SERPINE1_P519_F	5177	NA	Dif	0,810	0,113	-0,697	–
TNFSF10_E53_F	1109	LCP		0,829	0,134	-0,695	–
KLK10_P268_R	1272	LCP		0,865	0,172	-0,694	ND
CDKN1B_P1161_F	2375	HCP		0,724	0,042	-0,682	ND
FZD9_E458_F	5551	HCP		0,808	0,132	-0,676	–
CASP10_P334_F	3158	ICP		0,892	0,218	-0,674	–
NTRK2_P395_R	4919	HCP		0,702	0,031	-0,670	+
CSF3R_P8_F	3463	LCP		0,906	0,240	-0,666	–
PYCARD_P393_F	1799	HCP		0,759	0,098	-0,660	+
IRAK3_P185_F	4954	HCP		0,732	0,079	-0,653	+
SPDEF_P6_R	2048	LCP		0,957	0,323	-0,634	–

BDNF_E19_R	2840	LCP		0,755	0,128	-0,626	–
GSTM1_P266_F	4902	ICP		0,788	0,163	-0,625	–
ZMYND10_P329_F	2208	HCP		0,670	0,051	-0,619	–
RYK_P493_F	5179	HCP		0,676	0,060	-0,616	–
PADI4_P1011_R	1655	LCP		0,843	0,234	-0,609	–
S100A2_E36_R	3887	LCP		0,705	0,100	-0,605	–
RAP1A_P285_R	4896	ICP		0,744	0,141	-0,603	–
CLK1_P538_F	4290	HCP		0,694	0,092	-0,601	–
DNAJC15_E26_R	3155	NA		0,653	0,055	-0,598	–
EPHB3_P569_R	2160	HCP		0,672	0,081	-0,591	+
CALCA_E174_R	3043	NA		0,684	0,098	-0,586	+
NFKB1_P496_F	4185	HCP		0,657	0,079	-0,577	–
DDB2_P613_R	3632	ICP		0,677	0,101	-0,577	–
GSTM2_P453_R	944	ICP		0,697	0,130	-0,566	–
MEG3_E91_F	3640	NA		0,856	0,293	-0,563	ND
MGMT_P281_F	1407	HCP		0,596	0,042	-0,554	–
PXN_P308_F	4826	ICP		0,634	0,082	-0,552	ND
MAP2K6_P297_R	5050	NA		0,728	0,188	-0,541	–
CSK_P740_R	3479	HCP		0,634	0,098	-0,536	–
GUCY2D_P48_R	1950	ICP		0,700	0,168	-0,531	+
ITGB4_P517_F	3996	HCP		0,632	0,107	-0,525	–
AREG_P217_R	3002	HCP		0,622	0,104	-0,518	–
HOXA9_P303_F	3908	HCP		0,540	0,026	-0,514	+
PDGFRB_P343_F	2808	LCP	Dif	0,533	0,023	-0,510	–
HLA-DOA_P191_R	1009	LCP		0,691	0,188	-0,502	–
LIF_P383_R	4029	HCP		0,826	0,324	-0,502	–
DDB2_P407_F	3399	ICP		0,568	0,075	-0,493	–
MOS_P27_R	2748	ICP		0,489	0,050	-0,439	–
TJP1_P390_F	5268	NA		0,455	0,036	-0,418	ND
MTA1_P478_F	5764	HCP		0,461	0,043	-0,418	–
SMARCB1_P220_R	2354	NA		0,444	0,038	-0,406	–
GAS7_P622_R	4060	HCP	Dif	0,599	0,207	-0,392	+
ZP3_E90_F	4018	ICP		0,439	0,064	-0,375	–
TNFRSF1B_P167_F	5170	HCP		0,378	0,023	-0,355	+
INSR_P1063_R	1994	NA		0,418	0,073	-0,345	–

CD82_P557_R	1967	NA		0,374	0,032	-0,342	–
FAS_P65_F	4863	NA		0,415	0,078	-0,337	–
PTPRO_P371_F	1786	HCP		0,450	0,119	-0,331	–
CREBBP_P712_R	3290	HCP		0,413	0,844	0,431	–

Probe Sets				DNA Methylation			PcG Occupation (Lee, 2006)
TargetID	ProbeID	Promotor-Class	ESC/Dif. (Assou, 2007)	NTERA2. AVG_Beta	MAPC. AVG_Beta	Δ MAPC-NTERA2	
CARD15_P302_R	4256	LCP		0,968	0,037	-0,931	–
SCGB3A1_E55_R	3888	NA		0,967	0,038	-0,929	–
GUCY2D_E419_R	2999	ICP		0,959	0,035	-0,924	+
BCR_P346_F	2298	HCP		0,972	0,049	-0,923	–
HGF_E102_R	2755	LCP		0,952	0,033	-0,919	–
STAT5A_E42_F	5736	LCP		0,963	0,044	-0,919	–
RASSF1_E116_F	3865	HCP		0,950	0,032	-0,918	ND
HTR2A_P853_F	1105	LCP		0,944	0,032	-0,912	–
COL1A1_P5_F	3253	ICP	Dif	0,946	0,036	-0,911	ND
DDR2_E331_F	5516	LCP	Dif	0,961	0,056	-0,905	–
DDR2_P743_R	4305	LCP	Dif	0,927	0,027	-0,900	–
S100A4_E315_F	1714	LCP	Dif	0,974	0,076	-0,898	–
COL1A2_P48_R	315	ICP	Dif	0,946	0,049	-0,897	–
RASSF1_P244_F	1836	HCP		0,923	0,026	-0,896	ND
RARB_P60_F	2853	LCP		0,929	0,036	-0,894	–
P2RX7_E323_R	2854	LCP		0,946	0,055	-0,891	–
IL8_E118_R	754	LCP		0,953	0,067	-0,885	–
ASCL2_P609_R	105	HCP		0,968	0,083	-0,884	+
JAK3_P156_R	4990	ICP		0,951	0,079	-0,873	–
FASTK_P598_R	4874	NA		0,930	0,077	-0,853	–
COL1A2_E299_F	3083	ICP	Dif	0,916	0,066	-0,851	–
SEPT5_P464_R	5997	NA		0,935	0,086	-0,849	–
SEMA3B_E96_F	3894	LCP		0,894	0,046	-0,848	+
SEPT9_P374_F	6004	NA		0,886	0,039	-0,847	–
SNCG_P53_F	2022	LCP		0,916	0,071	-0,845	–

RARB_E114_F	4165	LCP		0,852	0,026	-0,827	–
SEPT5_P441_F	5988	NA		0,897	0,072	-0,825	–
TGFB3_E58_R	1269	ICP		0,962	0,139	-0,824	–
HHIP_P578_R	2230	HCP		0,853	0,032	-0,821	+
IRF7_E236_R	3520	HCP		0,859	0,039	-0,820	–
IGF1_E394_F	648	LCP		0,869	0,051	-0,818	–
MOS_E60_R	4133	ICP		0,875	0,058	-0,817	–
ASCL2_P360_F	103	HCP		0,900	0,091	-0,809	+
SEMA3B_P110_R	1886	LCP		0,843	0,036	-0,807	+
SCGB3A1_P103_R	1883	NA		0,920	0,114	-0,805	–
COL1A1_P117_R	3366	ICP	Dif	0,859	0,073	-0,785	ND
MLF1_E243_F	891	NA		0,824	0,045	-0,780	–
ASCL2_E76_R	3019	HCP		0,859	0,095	-0,764	+
DES_E228_R	5517	HCP		0,830	0,071	-0,758	–
IL16_P93_R	1173	LCP		0,830	0,073	-0,757	–
FAS_P322_R	4870	NA		0,820	0,068	-0,752	–
JAK3_E64_F	5615	ICP		0,790	0,039	-0,752	–
MLF1_P97_F	4276	NA		0,786	0,034	-0,751	–
P2RX7_P119_R	2170	LCP		0,835	0,086	-0,749	–
SEPT9_P58_R	6002	NA		0,792	0,044	-0,748	–
MPO_P883_R	2373	LCP		0,872	0,125	-0,746	–
CPA4_E20_F	3087	LCP		0,793	0,050	-0,744	–
SERPINE1_P519_F	5177	NA	Dif	0,810	0,074	-0,736	–
PDGFRB_P273_F	2810	LCP	Dif	0,806	0,075	-0,731	–
FRZB_E186_R	623	ICP	Dif	0,752	0,031	-0,721	–
IRF7_P277_R	1227	HCP		0,747	0,035	-0,713	–
IHH_E186_F	5583	HCP		0,742	0,038	-0,704	+
CDKN1B_P1161_F	2375	HCP		0,724	0,038	-0,687	ND
MMP3_P55_F	4851	LCP		0,785	0,113	-0,673	–
S100A2_E36_R	3887	LCP		0,705	0,042	-0,663	–
NTRK2_P395_R	4919	HCP		0,702	0,043	-0,658	+
IRAK3_P185_F	4954	HCP		0,732	0,077	-0,655	+
ZMYND10_P329_F	2208	HCP		0,670	0,035	-0,635	–
CALCA_E174_R	3043	NA		0,684	0,056	-0,628	+
RAP1A_P285_R	4896	ICP		0,744	0,123	-0,621	–

Probe Sets				DNA Methylation			PcG Occupation (Lee, 2006)
TargetID	ProbeID	Promotor-Class	ESC/Dif. (Assou, 2007)	NTERA2. AVG_Beta	MSC. AVG_Beta	Δ MSC-NTERA2	
CLK1_P538_F	4290	HCP		0,694	0,073	-0,620	–
DDB2_P613_R	3632	ICP		0,677	0,063	-0,614	–
DNAJC15_E26_R	3155	NA		0,653	0,044	-0,608	–
RYK_P493_F	5179	HCP		0,676	0,071	-0,605	–
EPHB3_P569_R	2160	HCP		0,672	0,072	-0,600	+
S100A2_P1186_F	1876	LCP		0,633	0,059	-0,573	–
PXN_P308_F	4826	ICP		0,634	0,066	-0,568	ND
NFKB1_P496_F	4185	HCP		0,657	0,096	-0,561	–
MGMT_P281_F	1407	HCP		0,596	0,041	-0,555	–
ITGB4_P517_F	3996	HCP		0,632	0,078	-0,553	–
CSK_P740_R	3479	HCP		0,634	0,093	-0,541	–
GSTM2_P453_R	944	ICP		0,697	0,162	-0,535	–
DDB2_P407_F	3399	ICP		0,568	0,038	-0,530	–
HOXA9_P303_F	3908	HCP		0,540	0,026	-0,513	+
PDGFRB_P343_F	2808	LCP	Dif	0,533	0,023	-0,511	–
MMP7_E59_F	931	LCP		0,608	0,120	-0,488	–
MOS_P27_R	2748	ICP		0,489	0,061	-0,428	–
TJP1_P390_F	5268	NA		0,455	0,031	-0,423	ND
MTA1_P478_F	5764	HCP		0,461	0,043	-0,418	–
SMARCB1_P220_R	2354	NA		0,444	0,037	-0,407	–
ZP3_E90_F	4018	ICP		0,439	0,076	-0,363	–
TNFRSF1B_P167_F	5170	HCP		0,378	0,027	-0,351	+
INSR_P1063_R	1994	NA		0,418	0,067	-0,351	–
CD82_P557_R	1967	NA		0,374	0,026	-0,348	–
TNK1_P41_R	5291	ICP		0,329	0,028	-0,300	–
HOXA11_P698_F	1066	ICP		0,155	0,877	0,722	ND
TDGF1_E53_R	1250	ICP	hESC	0,033	0,831	0,798	–

GUCY2D_E419_R	2999	ICP		0,959	0,028	-0,931	+
RASSF1_E116_F	3865	HCP		0,950	0,030	-0,920	ND
HTR2A_P853_F	1105	LCP		0,944	0,030	-0,914	–
COL1A1_P5_F	3253	ICP	Dif	0,946	0,037	-0,909	ND
DDR2_E331_F	5516	LCP	Dif	0,961	0,056	-0,906	–
HGF_E102_R	2755	LCP		0,952	0,047	-0,904	–
RASSF1_P244_F	1836	HCP		0,923	0,023	-0,900	ND
COL1A2_P48_R	315	ICP	Dif	0,946	0,047	-0,899	–
DDR2_P743_R	4305	LCP	Dif	0,927	0,028	-0,899	–
STAT5A_E42_F	5736	LCP		0,963	0,070	-0,894	–
RARB_P60_F	2853	LCP		0,929	0,039	-0,891	–
P2RX7_E323_R	2854	LCP		0,946	0,057	-0,889	–
NPY_P295_F	1644	HCP		0,964	0,079	-0,885	–
IL8_E118_R	754	LCP		0,953	0,072	-0,881	–
DLC1_P88_R	2431	LCP	Dif	0,928	0,067	-0,861	+
IFNGR2_P377_R	4125	ICP		0,905	0,048	-0,858	–
DLC1_E276_F	4082	LCP	Dif	0,929	0,076	-0,853	+
ASCL2_P609_R	105	HCP		0,968	0,119	-0,849	+
SEPT9_P374_F	6004	NA		0,886	0,039	-0,847	–
CASP10_P186_F	3056	ICP		0,911	0,065	-0,846	–
COL1A2_E299_F	3083	ICP	Dif	0,916	0,071	-0,846	–
PADI4_P1158_R	1658	LCP		0,906	0,063	-0,844	–
JAK3_P156_R	4990	ICP		0,951	0,111	-0,840	–
FASTK_P598_R	4874	NA		0,930	0,090	-0,839	–
SNCG_P53_F	2022	LCP		0,916	0,079	-0,837	–
SEPT5_P464_R	5997	NA		0,935	0,100	-0,835	–
RARB_E114_F	4165	LCP		0,852	0,019	-0,833	–
MOS_E60_R	4133	ICP		0,875	0,044	-0,831	–
SEPT5_P441_F	5988	NA		0,897	0,068	-0,830	–
IRF7_E236_R	3520	HCP		0,859	0,036	-0,823	–
SEMA3B_E96_F	3894	LCP		0,894	0,076	-0,818	+
IGF1_E394_F	648	LCP		0,869	0,054	-0,815	–
MPO_P883_R	2373	LCP		0,872	0,064	-0,807	–
ASCL2_P360_F	103	HCP		0,900	0,098	-0,802	+
HHIP_P578_R	2230	HCP		0,853	0,054	-0,799	+

COL1A1_P117_R	3366	ICP	Dif	0,859	0,061	-0,797	ND
SCGB3A1_P103_R	1883	NA		0,920	0,131	-0,789	–
MLF1_E243_F	891	NA		0,824	0,036	-0,788	–
HCK_P858_F	5768	HCP		0,956	0,173	-0,784	–
HLA-DRA_P132_R	1044	LCP		0,883	0,101	-0,782	–
SEMA3B_P110_R	1886	LCP		0,843	0,062	-0,781	+
IL16_P226_F	1171	LCP		0,907	0,126	-0,780	–
ASCL2_E76_R	3019	HCP		0,859	0,079	-0,780	+
IL16_P93_R	1173	LCP		0,830	0,057	-0,773	–
FANCE_P356_R	4848	HCP		0,839	0,076	-0,763	–
FAS_P322_R	4870	NA		0,820	0,062	-0,758	–
MLF1_P97_F	4276	NA		0,786	0,027	-0,758	–
JAK3_E64_F	5615	ICP		0,790	0,040	-0,751	–
DES_E228_R	5517	HCP		0,830	0,086	-0,744	–
SEPT9_P58_R	6002	NA		0,792	0,049	-0,743	–
CPA4_E20_F	3087	LCP		0,793	0,053	-0,741	–
TNFSF10_E53_F	1109	LCP		0,829	0,089	-0,740	–
PDGFRB_P273_F	2810	LCP	Dif	0,806	0,067	-0,739	–
IGF1_P933_F	4132	LCP		0,757	0,035	-0,722	–
FRZB_E186_R	623	ICP	Dif	0,752	0,033	-0,720	–
IRF7_P277_R	1227	HCP		0,747	0,033	-0,715	–
IHH_E186_F	5583	HCP		0,742	0,038	-0,704	+
P2RX7_P119_R	2170	LCP		0,835	0,139	-0,697	–
NAT2_P11_F	4177	LCP		0,947	0,260	-0,687	–
CDKN1B_P1161_F	2375	HCP		0,724	0,045	-0,679	ND
NTRK2_P395_R	4919	HCP		0,702	0,025	-0,677	+
PYCARD_P393_F	1799	HCP		0,759	0,089	-0,669	+
KLK10_P268_R	1272	LCP		0,865	0,197	-0,669	ND
FZD9_E458_F	5551	HCP		0,808	0,148	-0,660	–
BDNF_E19_R	2840	LCP		0,755	0,115	-0,640	–
RYK_P493_F	5179	HCP		0,676	0,043	-0,634	–
HLA-DPB1_E2_R	3440	LCP		0,938	0,309	-0,629	–
DDB2_P613_R	3632	ICP		0,677	0,059	-0,618	–
TSC2_E140_F	4202	HCP		0,798	0,189	-0,609	–
DNAJC15_E26_R	3155	NA		0,653	0,047	-0,605	–

S100A2_E36_R	3887	LCP		0,705	0,100	-0,605	–
RAP1A_P285_R	4896	ICP		0,744	0,143	-0,601	–
DMP1_P134_F	651	LCP		0,772	0,173	-0,600	ND
BSG_P211_R	4248	HCP		0,685	0,093	-0,593	–
EPHB3_P569_R	2160	HCP		0,672	0,082	-0,591	+
NFKB1_P496_F	4185	HCP		0,657	0,085	-0,572	–
CLK1_P538_F	4290	HCP		0,694	0,124	-0,570	–
MGMT_P281_F	1407	HCP		0,596	0,039	-0,556	–
DDB2_P407_F	3399	ICP		0,568	0,030	-0,538	–
CSK_P740_R	3479	HCP		0,634	0,104	-0,530	–
HOXA9_P303_F	3908	HCP		0,540	0,022	-0,518	+
GSTM2_P453_R	944	ICP		0,697	0,180	-0,516	–
PDGFRB_P343_F	2808	LCP	Dif	0,533	0,019	-0,515	–
PXN_P308_F	4826	ICP		0,634	0,127	-0,507	ND
AREG_P217_R	3002	HCP		0,622	0,117	-0,505	–
MAP2K6_P297_R	5050	NA		0,728	0,236	-0,493	–
MOS_P27_R	2748	ICP		0,489	0,040	-0,449	–
MTA1_P478_F	5764	HCP		0,461	0,047	-0,414	–
TJP1_P390_F	5268	NA		0,455	0,041	-0,413	ND
EIF2AK2_P313_F	5618	NA		0,448	0,040	-0,408	–
SMARCB1_P220_R	2354	NA		0,444	0,039	-0,405	–
ZP3_E90_F	4018	ICP		0,439	0,059	-0,380	–
TNFRSF1B_P167_F	5170	HCP		0,378	0,020	-0,358	+
FAS_P65_F	4863	NA		0,415	0,073	-0,342	–
CD82_P557_R	1967	NA		0,374	0,038	-0,336	–
CDK2_P330_R	2360	ICP		0,338	0,032	-0,306	–
MSH3_P13_R	2787	HCP		0,466	0,802	0,336	–
MSH3_E3_F	4137	HCP		0,499	0,862	0,363	–
SPP1_P647_F	5214	LCP		0,424	0,809	0,385	–
CREBBP_P712_R	3290	HCP		0,413	0,904	0,491	–
MATK_P190_R	5839	HCP	hESC	0,362	0,877	0,515	ND
ZIM2_E110_F	4009	NA		0,114	0,691	0,577	–
FGFR3_P1152_R	2536	HCP	hESC	0,257	0,894	0,638	–
MMP10_E136_R	920	LCP		0,083	0,728	0,645	–
VAMP8_P114_F	2187	LCP		0,138	0,807	0,669	ND
ZIM2_P22_F	2198	NA		0,022	0,694	0,671	–

PLAGL1_P236_R	1689	HCP	Dif	0,081	0,754	0,673	–
LTA_E28_R	820	LCP		0,243	0,920	0,676	–
SNURF_E256_R	3937	NA		0,026	0,707	0,681	–
SGCE_P250_R	1969	HCP		0,124	0,831	0,707	–
BMP4_P199_R	3050	ICP	Dif	0,167	0,881	0,713	–
SNRPN_E14_F	3936	ICP	hESC	0,059	0,790	0,731	–
SNURF_P78_F	2035	NA		0,040	0,778	0,737	–
SNRPN_seq_18_S99_F	6124	ICP	hESC	0,031	0,810	0,779	–
ER_seq_a1_S60_F	6020	HCP		0,028	0,865	0,836	+
PLAGL1_E68_R	3780	HCP	Dif	0,029	0,865	0,837	–
TDGF1_E53_R	1250	ICP	hESC	0,033	0,916	0,883	–
H19_P1411_R	950	NA		0,015	0,927	0,912	ND
EYA4_P794_F	759	HCP		0,024	0,951	0,927	+

**Probe Sets**				**DNA Methylation**			**PcG Occupation (Lee, 2006)**
TargetID	ProbeID	Promotor-Class	hESC/Dif. (Assou, 2007)	NTERA2. AVG_Beta	ADSC. AVG_Beta	Δ ADSC-NTERA2	
GUCY2D_E419_R	2999	ICP		0,959	0,028	-0,931	+
TGFB3_E58_R	1269	ICP		0,962	0,031	-0,931	–
SCGB3A1_E55_R	3888	NA		0,967	0,038	-0,929	–
S100A4_E315_F	1714	LCP	Dif	0,974	0,048	-0,926	–
STAT5A_E42_F	5736	LCP		0,963	0,038	-0,925	–
HGF_E102_R	2755	LCP		0,952	0,029	-0,923	–
RASSF1_E116_F	3865	HCP		0,950	0,034	-0,916	ND
BCR_P346_F	2298	HCP		0,972	0,057	-0,915	–
ASCL2_P609_R	105	HCP		0,968	0,059	-0,909	+
COL1A2_P48_R	315	ICP	Dif	0,946	0,041	-0,905	–
COL1A1_P5_F	3253	ICP	Dif	0,946	0,047	-0,899	ND
HIC-1_seq_48_S103_R	6059	HCP		0,938	0,041	-0,897	+
HTR2A_P853_F	1105	LCP		0,944	0,048	-0,896	–
RASSF1_P244_F	1836	HCP		0,923	0,035	-0,888	ND
DDR2_P743_R	4305	LCP	Dif	0,927	0,039	-0,888	–
RARB_P60_F	2853	LCP		0,929	0,042	-0,888	–

P2RX7_E323_R	2854	LCP		0,946	0,065	-0,881	–
DLC1_P695_F	2430	LCP	Dif	0,929	0,048	-0,881	+
NPY_P295_F	1644	HCP		0,964	0,085	-0,879	–
DDR2_E331_F	5516	LCP	Dif	0,961	0,088	-0,873	–
SEPT5_P464_R	5997	NA		0,935	0,062	-0,873	–
DLC1_P88_R	2431	LCP	Dif	0,928	0,062	-0,866	+
COL1A2_P407_R	331	ICP	Dif	0,910	0,047	-0,863	–
COL1A2_E299_F	3083	ICP	Dif	0,916	0,056	-0,860	–
SNCG_P53_F	2022	LCP		0,916	0,059	-0,857	–
IL1RN_P93_R	4198	LCP		0,897	0,048	-0,849	ND
FASTK_P598_R	4874	NA		0,930	0,081	-0,849	–
SEPT9_P374_F	6004	NA		0,886	0,038	-0,848	–
ASCL2_P360_F	103	HCP		0,900	0,054	-0,845	+
HCK_P858_F	5768	HCP		0,956	0,116	-0,841	–
IFNGR2_P377_R	4125	ICP		0,905	0,065	-0,840	–
SEPT5_P441_F	5988	NA		0,897	0,058	-0,839	–
SEMA3B_E96_F	3894	LCP		0,894	0,055	-0,839	+
MPO_P883_R	2373	LCP		0,872	0,044	-0,827	–
RARB_E114_F	4165	LCP		0,852	0,026	-0,826	–
HHIP_P578_R	2230	HCP		0,853	0,030	-0,823	+
CEACAM1_E57_R	113	LCP		0,909	0,095	-0,814	–
SCGB3A1_P103_R	1883	NA		0,920	0,106	-0,813	–
CASP10_P334_F	3158	ICP		0,892	0,082	-0,810	–
IGF1_E394_F	648	LCP		0,869	0,058	-0,810	–
IRF7_E236_R	3520	HCP		0,859	0,053	-0,806	–
SEMA3B_P110_R	1886	LCP		0,843	0,037	-0,805	+
MOS_E60_R	4133	ICP		0,875	0,071	-0,804	–
RARA_P1076_R	5118	LCP		0,931	0,129	-0,802	+
COL1A1_P117_R	3366	ICP	Dif	0,859	0,056	-0,802	ND
ASCL2_E76_R	3019	HCP		0,859	0,059	-0,801	+
HLA-DRA_P132_R	1044	LCP		0,883	0,090	-0,793	–
MLF1_E243_F	891	NA		0,824	0,042	-0,783	–
DES_E228_R	5517	HCP		0,830	0,053	-0,777	–
TNFSF10_E53_F	1109	LCP		0,829	0,053	-0,776	–
CASP10_P186_F	3056	ICP		0,911	0,140	-0,771	–

IL16_P93_R	1173	LCP		0,830	0,066	-0,764	–
FAS_P322_R	4870	NA		0,820	0,058	-0,762	–
JAK3_E64_F	5615	ICP		0,790	0,037	-0,754	–
MLF1_P97_F	4276	NA		0,786	0,035	-0,750	–
P2RX7_P119_R	2170	LCP		0,835	0,091	-0,745	–
GSTM1_P266_F	4902	ICP		0,788	0,044	-0,744	–
SERPINE1_P519_F	5177	NA	Dif	0,810	0,068	-0,742	–
KLK10_P268_R	1272	LCP		0,865	0,126	-0,740	ND
FANCE_P356_R	4848	HCP		0,839	0,100	-0,739	–
CPA4_E20_F	3087	LCP		0,793	0,058	-0,735	–
SEPT9_P58_R	6002	NA		0,792	0,060	-0,732	–
FRZB_E186_R	623	ICP	Dif	0,752	0,029	-0,723	–
IGF1_P933_F	4132	LCP		0,757	0,036	-0,720	–
PDGFRB_P273_F	2810	LCP	Dif	0,806	0,086	-0,720	–
PYCARD_P393_F	1799	HCP		0,759	0,045	-0,714	+
AATK_P519_R	3	NA		0,933	0,226	-0,707	ND
IHH_E186_F	5583	HCP		0,742	0,036	-0,706	+
IRF7_P277_R	1227	HCP		0,747	0,049	-0,699	–
CDKN1B_P1161_F	2375	HCP		0,724	0,045	-0,680	ND
NTRK2_P395_R	4919	HCP		0,702	0,026	-0,676	+
POMC_P400_R	1743	HCP		0,733	0,065	-0,668	+
BDNF_E19_R	2840	LCP		0,755	0,088	-0,667	–
IRAK3_P185_F	4954	HCP		0,732	0,071	-0,661	+
GSTM2_P453_R	944	ICP		0,697	0,048	-0,648	–
CALCA_E174_R	3043	NA		0,684	0,046	-0,638	+
BSG_P211_R	4248	HCP		0,685	0,049	-0,636	–
ZMYND10_P329_F	2208	HCP		0,670	0,055	-0,615	–
RYK_P493_F	5179	HCP		0,676	0,068	-0,609	–
NFKB1_P496_F	4185	HCP		0,657	0,058	-0,599	–
ITGB4_P517_F	3996	HCP		0,632	0,046	-0,586	–
EPHB3_P569_R	2160	HCP		0,672	0,090	-0,582	+
PXN_P308_F	4826	ICP		0,634	0,053	-0,580	ND
MGMT_P281_F	1407	HCP		0,596	0,045	-0,551	–
HOXA9_P303_F	3908	HCP		0,540	0,030	-0,509	+
PDGFRB_P343_F	2808	LCP	Dif	0,533	0,029	-0,504	–

MOS_P27_R	2748	ICP		0,489	0,049	-0,440	–
MTA1_P478_F	5764	HCP		0,461	0,039	-0,422	–
TJP1_P390_F	5268	NA		0,455	0,037	-0,417	ND
SMARCB1_P220_R	2354	NA		0,444	0,039	-0,405	–
EIF2AK2_P313_F	5618	NA		0,448	0,046	-0,402	–
ZP3_E90_F	4018	ICP		0,439	0,058	-0,381	–
TNF_P158_F	2915	LCP		0,410	0,034	-0,376	–
TNFRSF1B_P167_F	5170	HCP		0,378	0,023	-0,356	+
ICAM1_E242_F	4104	HCP		0,466	0,120	-0,346	ND
CD82_P557_R	1967	NA		0,374	0,032	-0,343	–
CALCA_P75_F	178	NA		0,335	0,029	-0,306	+

Probe Sets				DNA Methylation			PcG Occupation (Lee, 2006)
TargetID	ProbeID	Promotor-Class	hESC/Dif. (Assou, 2007)	MAPC. AVG_Beta	MSC. AVG_Beta	Δ MSC-MAPC	
RUNX3_P247_F	1869	HCP		0,946	0,093	-0,852	–
RUNX3_E27_R	3879	HCP		0,895	0,089	-0,806	–
RUNX3_P393_R	1870	HCP		0,960	0,181	-0,779	–
LEFTY2_P561_F	5906	NA	hESC	0,240	0,799	0,560	ND
MMP3_P55_F	4851	LCP		0,113	0,719	0,607	–
CAPG_E228_F	3045	LCP		0,160	0,893	0,733	–
S100A2_P1186_F	1876	LCP		0,059	0,816	0,756	–
ER_seq_a1_S60_F	6020	HCP		0,080	0,865	0,784	+
MMP3_P16_R	4850	LCP		0,082	0,870	0,789	–
S100A4_E315_F	1714	LCP	hESC	0,076	0,892	0,816	–

Probe Sets				DNA Methylation			PcG Occupation (Lee, 2006)
TargetID	ProbeID	Promotor-Class	hESC/Dif. (Assou, 2007)	MAPC. AVG_Beta	ADSC. AVG_Beta	Δ ADSC-MAPC	
HIC-1_seq_48_S103_R	6059	HCP		0,904	0,041	-0,863	+
HOXA5_P1324_F	1072	HCP	Dif	0,771	0,144	-0,627	ND

IL8_P83_F	4225	LCP		0,142	0,800	0,658	–

Probe Sets				DNA Methylation			PcG Occupation (Lee, 2006)
TargetID	ProbeID	Promotor-Class	hESC/Dif. (Assou, 2007)	MSC. AVG_Beta	ADSC. AVG_Beta	Δ ADSC-MSC	
HIC-1_seq_48_S103_R	6059	HCP		0,956	0,041	-0,915	+
S100A4_P194_R	6015	LCP	Dif	0,947	0,101	-0,846	–
S100A4_E315_F	1714	LCP	Dif	0,892	0,048	-0,844	–
FGFR3_P1152_R	2536	HCP	hESC	0,894	0,063	-0,831	–
MATK_P190_R	5839	HCP	hESC	0,877	0,109	-0,768	ND
DLC1_P695_F	2430	LCP	Dif	0,793	0,048	-0,744	+
LEFTY2_P561_F	5906	NA	hESC	0,799	0,067	-0,733	ND
BMP4_P199_R	3050	ICP	Dif	0,881	0,156	-0,725	–
COL1A2_P407_R	331	ICP	Dif	0,769	0,047	-0,722	–
ER_seq_a1_S60_F	6020	HCP		0,865	0,160	-0,704	+
S100A4_P887_R	5985	LCP	Dif	0,919	0,231	-0,688	–
THBS2_P605_R	2907	ICP		0,938	0,263	-0,675	+
S100A2_P1186_F	1876	LCP		0,816	0,197	-0,618	–
ASB4_P391_F	96	ICP		0,874	0,277	-0,597	–
CAPG_E228_F	3045	LCP		0,893	0,297	-0,596	–
IL1RN_E42_F	745	LCP		0,858	0,277	-0,582	ND
AATK_P519_R	3	NA		0,775	0,226	-0,550	ND
TNFRSF10C_P7_F	2143	ICP		0,604	0,076	-0,528	–
RARA_P1076_R	5118	LCP		0,630	0,129	-0,501	+
MUC1_P191_F	5784	ICP		0,630	0,130	-0,499	–
STAT5A_P704_R	5243	LCP		0,764	0,268	-0,496	–
AATK_P709_R	10	NA		0,592	0,103	-0,489	ND
IL12B_P1453_F	4190	LCP		0,710	0,226	-0,485	–
SNCG_E119_F	3934	LCP		0,579	0,096	-0,484	–
MYLK_P469_R	5076	HCP	Dif	0,557	0,085	-0,471	–
MXI1_P1269_F	4166	HCP		0,679	0,269	-0,410	–

RARA_P176_R	5097	LCP		0,427	0,055	-0,372	+
CEACAM1_E57_R	113	LCP		0,463	0,095	-0,368	–
RIPK1_P744_R	5140	LCP		0,290	0,824	0,534	–
FGF1_E5_F	619	LCP		0,203	0,773	0,570	–
RUNX3_P393_R	1870	HCP		0,181	0,931	0,750	–
RUNX3_E27_R	3879	HCP		0,089	0,888	0,800	–
RUNX3_P247_F	1869	HCP		0,093	0,900	0,807	–
