# Supplementary material for: Epigenetic Signatures Associated with Different Levels of Differentiation Potential in Human Stem Cells
Source: PLoS One. 2009 Nov 13;4(11):e7809. doi: 10.1371/journal.pone.0007809 (PMC2771914; doi:10.1371/journal.pone.0007809)
Supplement: Table S12 — Differentially expressed miRNAs between NTERA-2 and ASC. Logarithmic values (log10) of those microRNAs which expression is significantly reduced/increased in ASC vs NTERA-2 cell line. These values were obtained comparing the expression value of each sample by the average value of NTERA_2 samples. A, miRNAs up-regulated in NTERA-2 and down-regulated in ASC. B, miRNAs up-regulated in ASC and down-regulated in NTERA-2. (0.01 MB PDF) [file pone.0007809.s018.pdf]

	MSCs							ADSCs							MAPCs			
	MSC-2	MSC-3	MSC-4	MSC-5	MSC-6	MSC-7	MSC-8	ADSC-1	ADSC-2	ADSC-3	ADSC-4	ADSC-5	ADSC-6	ADSC-7	MAPC-1	MAPC-2	MAPC-3	MAPC-4
MIR 9	-3,060	-2,204	-2,257	-4,281	-2,068	-2,016	-1,550	-2,711	-2,323	-1,620	-1,323	-1,761	-1,921	-1,880	-2,460	-1,776	-2,098	-1,958
MIR 20B	-3,392	-3,184	-2,180	-3,452	-2,388	-2,214	-2,242	-2,671	-2,689	-2,140	-2,347	-2,341	-2,178	-2,387	-2,443	-2,104	-2,082	-2,415
MIR 96	-2,095	-2,796	-2,612	-2,941	-2,699	-1,880	-1,909	-3,182	-3,273	-2,093	-2,080	-2,686	-2,380	-2,415	-2,360	-2,196	-2,280	-2,694
MIR 124A	-2,729	-3,430	-3,246	-3,575	-1,592	-2,514	-2,543	-3,816	-3,907	-2,727	-2,714	-3,320	-3,014	-3,049	-2,994	-2,830	-2,914	-3,328
MIR 135A	-4,555	-5,256	-2,790	-5,401	-3,767	-4,340	-4,370	-5,642	-4,529	-3,421	-4,541	-4,640	-4,841	-4,584	-4,820	-3,452	-3,844	-5,154
MIR 135B	-3,165	-2,292	-1,474	-3,389	-1,530	-1,803	-1,939	-2,365	-2,347	-1,772	-2,319	-2,046	-1,852	-2,132	-3,289	-2,678	-2,413	-3,641
MIR 182	-2,403	-3,759	-2,836	-3,882	-2,094	-2,216	-4,207	-3,941	-3,405	-1,903	-1,499	-2,438	-3,016	-3,515	-3,391	-3,578	-2,195	-2,499
MIR 183	-2,859	-2,933	-3,441	-2,569	-2,954	-2,672	-3,459	-3,194	-3,861	-2,750	-2,661	-2,300	-3,347	-2,767	-1,839	-2,769	-3,030	-2,840
MIR 187	-3,775	-2,797	-1,967	-2,549	-1,724	-1,282	-1,768	-2,465	-4,777	-3,665	-3,576	-3,161	-4,263	-3,683	-3,559	-2,320	-2,632	-4,444
MIR 205	-3,695	-3,848	-2,512	-3,971	-3,790	-3,509	-4,295	-4,030	-3,165	-3,586	-3,497	-3,979	-4,184	-3,603	-2,068	-3,666	-3,866	-4,364
MIR 302A#	-4,211	-4,363	-4,762	-4,487	-4,306	-4,024	-4,811	-4,545	-5,213	-4,102	-4,013	-4,324	-4,034	-4,119	-3,995	-4,182	-4,382	-4,880
MIR 302#B	-3,143	-3,295	-3,725	-3,418	-3,238	-2,956	-3,743	-3,477	-4,145	-3,033	-2,944	-3,426	-3,631	-3,051	-2,927	-3,113	-3,313	-3,812
MIR 302D	-6,381	-4,861	-3,675	-4,714	-6,476	-6,195	-4,890	-6,716	-5,601	-6,272	-5,659	-4,181	-5,860	-6,290	-6,166	-4,702	-4,634	-5,380
MIR 367	-6,261	-4,839	-3,776	-4,450	-4,508	-6,074	-6,861	-6,595	-5,774	-4,427	-6,062	-4,481	-6,749	-6,169	-6,045	-4,764	-5,010	-4,780
MIR 372	-3,452	-3,604	-2,481	-3,727	-3,547	-3,265	-4,052	-3,786	-4,454	-3,342	-3,253	-3,735	-3,940	-3,360	-3,236	-3,422	-3,622	-4,121
MIR 512-5P	-3,644	-3,039	-2,880	-3,311	-2,220	-3,316	-3,346	-4,136	-2,911	-3,111	-3,142	-2,692	-3,307	-2,544	-2,753	-2,619	-3,116	-3,655
MIR 515-3P	-2,017	-2,266	-0,711	-2,519	-1,755	-1,915	-1,674	-2,209	-1,190	-1,523	-2,078	-1,476	-1,304	-2,007	-2,215	-1,149	-2,198	-1,913
MIR 515-5P	-2,742	-2,137	-2,619	-2,409	-2,781	-2,414	-2,445	-3,234	-2,599	-2,209	-2,240	-2,574	-2,406	-1,643	-1,851	-1,717	-2,215	-2,753
MIR 517A	-2,525	-1,920	-2,402	-2,192	-2,564	-2,197	-2,227	-3,017	-2,382	-1,992	-2,023	-2,357	-2,188	-1,426	-1,634	-1,500	-1,997	-2,536
MIR 517B	-2,684	-2,079	-2,561	-2,351	-2,723	-2,356	-2,387	-3,177	-2,541	-2,151	-2,182	-2,516	-2,348	-1,585	-1,793	-1,659	-2,157	-2,695
MIR 517C	-4,129	-3,524	-3,458	-3,796	-3,949	-3,801	-3,831	-4,621	-3,011	-3,596	-3,627	-3,961	-3,792	-3,029	-3,238	-3,104	-3,601	-4,140
MIR 518A	-2,617	-2,364	-1,785	-2,636	-2,095	-1,872	-2,102	-2,933	-1,892	-1,956	-2,102	-1,990	-2,523	-1,870	-2,078	-1,944	-2,441	-2,980
MIR 518B	-2,407	-2,610	-1,765	-2,010	-2,878	-1,774	-2,110	-2,614	-1,748	-2,353	-1,789	-2,350	-1,364	-2,512	-1,349	-2,586	-2,405	-2,331
MIR 518C	-3,444	-2,839	-2,576	-3,111	-3,266	-3,116	-2,366	-3,763	-3,301	-2,911	-2,942	-2,684	-2,845	-1,960	-2,553	-2,419	-2,916	-3,455
MIR 519C	-2,702	-2,142	-2,303	-2,428	-2,272	-3,212	-2,514	-2,625	-2,002	-1,939	-2,532	-2,253	-2,451	-2,531	-2,515	-1,469	-3,102	-2,850
MIR 519D	-2,900	-2,809	-2,357	-3,081	-3,211	-2,746	-2,739	-3,032	-3,158	-2,753	-2,912	-3,246	-3,077	-2,314	-2,523	-2,389	-2,886	-3,425
MIR 520B	-3,304	-2,699	-2,306	-2,971	-2,099	-2,976	-1,559	-3,797	-2,106	-2,771	-2,802	-3,136	-2,968	-2,205	-2,413	-2,279	-2,777	-3,315
MIR 520C	-2,739	-2,134	-2,617	-2,406	-2,281	-2,411	-2,442	-2,650	-1,880	-2,206	-2,237	-2,571	-2,403	-1,640	-1,848	-1,549	-2,212	-2,510
MIR 520D	-2,579	-1,974	-1,539	-2,246	-1,481	-2,251	-2,220	-3,071	-2,436	-1,837	-2,077	-2,411	-2,242	-1,479	-1,688	-1,509	-1,822	-2,590
MIR 520E	-2,882	-2,277	-2,760	-2,549	-2,921	-2,554	-2,585	-2,627	-2,739	-2,349	-2,380	-2,714	-2,546	-1,783	-1,991	-1,857	-2,355	-2,893

MIR 520F	-3,141	-2,536	-3,019	-2,808	-2,636	-2,813	-2,287	-2,567	-2,579	-2,608	-2,639	-2,973	-2,805	-2,042	-2,250	-2,116	-2,614	-3,153
MIR 520G	-3,717	-3,112	-3,595	-3,384	-3,756	-3,389	-3,420	-4,210	-3,574	-3,184	-3,215	-3,549	-3,381	-2,618	-2,826	-2,692	-3,190	-3,728
MIR 520H	-2,884	-3,213	-3,063	-3,485	-3,857	-3,334	-3,520	-3,602	-3,074	-2,765	-3,278	-3,650	-3,394	-2,719	-2,927	-2,793	-3,290	-2,129
MIR 526A	-2,276	-1,670	-2,153	-1,942	-2,314	-1,947	-1,978	-2,768	-2,132	-1,743	-1,773	-2,108	-1,939	-1,176	-1,384	-1,250	-1,748	-2,287

	MSCs							ADSCs							MAPCs			
	MSC-2	MSC-3	MSC-4	MSC-5	MSC-6	MSC-7	MSC-8	ADSC-1	ADSC-2	ADSC-3	ADSC-4	ADSC-5	ADSC-6	ADSC-7	MAPC-1	MAPC-2	MAPC-3	MAPC-4
LET 7A	2,589	3,258	4,085	2,563	3,599	3,953	4,281	2,678	3,715	3,642	3,459	3,388	3,761	3,517	3,070	2,651	3,680	3,073
LET 7B	4,760	5,273	5,488	4,655	5,377	5,935	6,217	4,747	5,035	5,905	5,545	5,286	5,889	5,605	4,913	4,349	5,489	5,034
LET 7C	4,706	5,057	5,238	4,334	5,363	5,681	5,883	4,595	5,229	5,713	5,466	5,350	5,738	5,465	4,650	4,870	5,174	4,910
LET 7D	3,901	4,272	4,894	3,833	4,612	4,956	5,083	3,748	3,923	4,658	4,776	4,183	4,900	4,134	4,281	4,034	4,594	3,941
LET 7E	2,605	3,045	3,684	2,493	3,406	3,926	3,640	2,184	2,137	2,599	2,831	2,097	2,667	2,587	2,884	2,594	3,091	2,329
LET 7F	2,185	2,794	4,034	2,112	3,609	3,625	3,993	3,100	3,277	3,385	3,259	3,615	3,577	3,600	2,892	3,280	3,238	3,329
LET7G	4,002	4,328	5,372	3,848	5,163	5,165	5,625	4,503	4,535	4,791	4,864	4,837	5,177	4,547	4,405	4,921	4,817	4,667
MIR 10A	3,542	3,650	3,883	2,623	3,442	3,787	3,531	2,983	3,851	4,470	4,338	2,961	3,920	4,463	3,167	2,801	3,317	3,867
MIR 10B	3,191	3,219	3,541	2,782	3,156	3,578	3,416	3,186	3,462	4,126	3,627	3,105	3,820	3,581	2,678	2,599	3,291	3,086
MIR 21	1,196	1,578	2,512	1,057	2,566	2,329	2,644	1,092	1,301	1,773	1,799	1,222	1,960	1,899	1,077	1,012	1,672	1,265
MIR 23A	1,848	1,959	2,157	1,453	1,997	2,603	2,464	1,246	1,437	2,037	2,293	1,566	2,182	1,824	0,971	1,581	1,876	1,630
MIR 27A	1,401	1,790	1,949	1,606	1,858	2,462	1,950	1,437	1,627	2,097	2,179	1,998	2,153	2,087	1,552	1,867	2,133	1,874
MIR 29A	1,314	1,699	2,569	1,163	2,189	2,127	2,226	0,984	1,578	1,934	1,790	1,759	2,094	1,693	1,716	1,799	1,861	1,766
MIR 98	3,508	2,239	4,413	3,012	4,176	4,622	4,647	3,335	3,544	3,544	3,981	4,060	3,885	3,794	2,972	3,072	3,809	3,579
MIR 99A	3,246	2,413	3,819	2,676	4,225	3,970	4,194	2,460	2,639	3,398	2,959	3,350	3,188	3,107	2,074	3,236	3,456	3,327
MIR 100	2,854	2,550	4,324	1,857	4,478	3,721	4,362	2,495	2,630	3,193	3,449	3,397	3,310	3,205	2,816	3,198	3,386	3,237
MIR 125B	3,043	2,799	3,190	2,147	3,234	3,449	3,298	2,200	2,495	2,865	3,083	2,827	2,901	2,762	2,212	2,507	2,968	2,615
MIR 134	1,723	1,437	2,588	1,299	2,444	2,451	2,914	1,324	1,848	2,474	2,379	2,514	2,469	2,091	1,636	2,412	2,231	1,747
MIR 137	1,611	2,625	3,148	2,006	2,759	2,239	2,689	1,982	2,034	2,376	2,166	2,230	1,697	2,171	1,630	1,720	2,137	2,985
MIR 138	1,746	1,986	2,029	1,711	2,334	2,660	3,172	1,568	2,067	2,381	2,320	2,136	1,786	1,664	2,744	2,828	2,624	2,037
MIR 143	3,452	3,716	4,826	3,241	4,042	4,390	4,599	3,423	3,358	3,978	4,096	3,005	3,809	3,990	2,319	2,689	3,235	1,400
MIR 152	1,173	2,117	1,931	1,868	2,566	2,392	2,617	2,439	1,699	1,833	2,450	2,034	1,800	2,216	1,923	1,766	1,866	1,329
MIR 154	2,212	2,699	2,780	2,309	3,710	2,906	2,999	2,801	2,633	2,648	2,505	2,907	3,129	2,569	0,951	3,143	2,805	2,442
MIR 155	2,172	2,872	3,131	2,365	4,078	3,442	2,892	3,582	2,686	2,731	3,619	3,265	2,636	3,660	3,691	3,745	3,040	2,646
MIR 196A	3,197	4,156	4,355	4,123	4,848	4,486	4,303	3,451	4,096	3,639	4,458	3,892	4,188	4,516	4,316	4,468	4,245	3,769

MIR 196B	3,254	4,546	5,005	4,316	4,792	5,031	4,710	4,612	3,090	4,150	4,769	4,375	4,036	5,007	4,622	4,172	4,206	3,650
MIR 199A	3,343	3,884	4,114	3,537	4,756	4,228	4,273	4,529	4,114	3,940	4,666	4,064	4,374	4,632	3,511	3,883	3,811	3,220
MIR 199B	0,869	1,461	2,164	1,479	2,979	2,109	2,665	3,039	2,697	2,437	3,480	3,073	2,976	2,939	1,535	2,029	1,739	1,353
MIR 214	3,376	4,015	3,893	3,374	4,126	4,264	3,883	3,949	3,610	3,957	4,443	4,113	4,063	4,288	3,726	3,388	3,664	2,829
MIR 299-5P	1,392	1,768	1,503	1,484	2,480	2,092	2,018	1,739	1,606	1,782	2,152	1,929	2,029	2,062	1,729	1,948	2,126	0,738
