# Supplementary material for: Epigenetic Signatures Associated with Different Levels of Differentiation Potential in Human Stem Cells
Source: PLoS One. 2009 Nov 13;4(11):e7809. doi: 10.1371/journal.pone.0007809 (PMC2771914; doi:10.1371/journal.pone.0007809)
Supplement: Table S2 — RMA raw data (Affymetrix's data) (6.47 MB PDF) [file pone.0007809.s008.pdf]

Table S2. RMA raw data (Affymetrix´s data)

ProbeSet	NTERA-1	NTERA-2	NTERA-3	MAPC-1	MAPC-2	MAPC-3
1007_s_at	9,820	9,566	9,738	8,918	8,838	9,151
1053_at	9,815	9,598	9,721	9,359	8,037	9,052
117_at	7,336	7,313	7,255	7,883	7,818	7,814
121_at	8,830	8,909	8,921	9,624	9,706	10,059
1255_g_at	8,662	8,917	9,252	5,485	5,474	5,471
1294_at	6,972	7,141	6,777	7,835	7,802	8,046
1316_at	6,892	7,165	7,170	7,645	7,693	8,155
1320_at	7,139	7,039	7,000	7,574	7,378	7,881
1405_i_at	4,977	4,779	4,859	4,915	4,591	4,668
1431_at	6,167	5,925	6,161	5,952	5,646	5,881
1438_at	7,219	7,014	7,147	7,487	7,282	7,506
1487_at	8,280	8,179	8,361	8,786	7,964	8,750
1494_f_at	7,384	7,287	7,432	7,521	7,548	7,475
1552256_a_at	9,267	9,212	9,333	9,492	8,982	9,513
1552257_a_at	9,837	9,611	10,266	9,571	8,861	9,298
1552258_at	7,038	7,216	7,148	7,121	8,043	7,179
1552261_at	6,570	6,748	6,648	7,552	7,350	7,618
1552263_at	6,582	7,014	6,974	8,070	7,816	8,058
1552264_a_at	7,000	7,230	6,713	7,732	7,403	7,922
1552266_at	5,893	5,688	5,932	6,184	6,196	6,168
1552269_at	6,615	7,242	5,891	5,290	4,952	5,284
1552271_at	7,620	7,314	7,391	7,947	7,650	7,958
1552272_a_at	6,981	6,513	6,831	6,310	6,332	6,454
1552274_at	6,631	7,131	6,889	7,951	7,001	7,518
1552275_s_at	7,082	7,377	6,789	7,821	6,840	7,465
1552276_a_at	7,152	7,083	6,925	7,455	7,269	7,532
1552277_a_at	9,890	9,919	9,881	10,625	10,089	10,273
1552278_a_at	5,787	5,512	6,059	6,343	6,102	6,317
1552279_a_at	7,459	7,469	7,441	7,977	7,611	7,821
1552280_at	5,602	5,579	5,524	5,826	5,456	5,611
1552281_at	8,299	8,180	8,489	9,219	9,159	9,217
1552283_s_at	8,403	7,969	8,273	6,590	6,834	6,809
1552286_at	9,763	9,773	8,996	8,626	8,080	8,518
1552287_s_at	9,219	8,630	9,188	6,451	7,299	6,551
1552288_at	5,850	6,315	6,038	7,636	7,778	7,792
1552289_a_at	7,594	7,507	7,555	7,520	7,179	7,551
1552291_at	8,680	8,866	8,400	8,998	8,991	8,644
1552293_at	6,641	7,351	7,243	7,017	6,910	7,177
1552295_a_at	8,470	8,538	8,237	9,541	9,114	9,885
1552296_at	6,294	6,449	6,404	6,272	6,189	6,417
1552299_at	6,243	6,078	5,690	6,272	5,715	6,312
1552301_a_at	6,869	6,661	6,851	7,191	7,322	7,423
1552302_at	4,771	4,368	5,192	6,962	7,481	7,974
1552303_a_at	6,014	6,140	6,169	6,964	7,143	7,754
1552304_at	8,448	8,567	8,320	6,189	6,112	6,184
1552306_at	8,275	8,187	8,097	6,822	5,861	6,555
1552307_a_at	7,385	8,044	7,973	7,917	7,468	7,623
1552309_a_at	7,722	7,384	7,479	6,573	9,150	7,152
1552310_at	9,515	9,408	9,436	8,359	7,881	7,610
1552311_a_at	8,763	8,645	8,718	9,446	9,249	9,234
1552312_a_at	7,609	7,156	7,627	6,976	6,737	6,760
1552314_a_at	7,273	7,657	6,772	6,707	6,491	6,655
1552315_at	6,097	6,063	5,996	6,329	6,131	6,417
1552316_a_at	3,978	4,141	4,434	5,031	5,025	4,911

1552318_at	5,642	5,692	5,690	5,896	5,853	5,853
1552319_a_at	6,633	6,499	6,427	7,157	7,201	7,437
1552320_a_at	4,677	4,641	4,709	4,872	4,650	4,787
1552321_a_at	4,697	4,398	4,554	5,055	4,722	5,443
1552322_at	3,887	4,229	4,217	4,037	4,072	4,081
1552323_s_at	6,276	6,560	6,327	5,950	5,990	5,884
1552325_at	3,538	3,851	3,900	5,786	6,254	6,276
1552326_a_at	6,635	6,579	6,428	6,894	6,505	7,123
1552327_at	5,645	5,687	4,967	4,872	4,821	4,902
1552329_at	8,160	8,587	8,441	6,907	5,710	6,114
1552330_at	6,380	5,779	6,400	5,570	5,025	5,563
1552332_at	8,576	8,359	8,431	9,421	9,161	9,277
1552334_at	6,041	6,413	6,529	7,315	6,653	7,077
1552335_at	7,290	7,286	7,264	8,147	8,030	8,061
1552337_s_at	6,903	6,832	6,930	7,457	6,958	7,320
1552338_at	6,060	6,027	6,034	6,210	6,059	6,186
1552340_at	6,478	6,068	6,485	6,785	6,626	6,676
1552343_s_at	6,716	6,336	6,425	5,824	5,865	5,788
1552344_s_at	8,643	8,444	8,397	7,386	7,270	7,320
1552347_at	8,611	8,515	7,913	6,480	6,645	6,409
1552348_at	7,242	7,337	7,285	7,558	7,402	7,377
1552349_a_at	6,620	6,268	6,180	7,415	7,040	7,206
1552354_at	7,539	7,227	7,435	8,305	7,796	7,950
1552355_s_at	6,766	6,560	6,509	7,205	7,010	7,074
1552359_at	4,605	4,660	4,575	4,855	5,093	5,256
1552360_a_at	5,913	5,661	5,994	5,840	5,711	5,828
1552362_a_at	7,712	7,357	7,569	6,162	5,908	5,812
1552364_s_at	7,115	7,201	6,921	6,984	6,417	6,888
1552365_at	4,683	4,619	4,509	4,335	4,365	4,445
1552367_a_at	5,439	5,764	5,357	5,911	5,709	5,723
1552368_at	6,640	7,110	6,536	6,308	6,080	6,233
1552370_at	5,219	5,676	5,146	5,509	6,109	6,227
1552372_at	3,435	3,881	3,707	3,634	3,938	3,788
1552373_s_at	3,783	3,754	3,746	3,948	3,739	3,899
1552375_at	5,915	6,172	5,974	5,523	5,768	5,664
1552377_s_at	8,103	7,971	8,205	8,655	8,288	8,082
1552378_s_at	5,753	6,030	5,680	7,167	6,856	7,262
1552379_at	3,610	3,386	3,643	3,699	3,690	3,861
1552381_at	5,324	5,338	5,082	5,202	5,028	5,326
1552383_at	8,146	8,065	8,246	9,265	9,237	9,306
1552384_a_at	6,631	6,696	6,738	7,571	7,717	7,720
1552386_at	3,770	3,858	3,954	3,825	3,752	4,106
1552388_at	7,657	7,361	7,208	7,918	7,776	7,962
1552389_at	4,702	4,880	4,965	6,041	6,082	6,281
1552390_a_at	4,909	5,539	5,268	4,675	4,408	4,545
1552391_at	5,790	5,668	5,791	7,157	7,106	7,396
1552393_at	3,776	3,795	4,038	4,055	3,940	4,019
1552394_a_at	3,674	4,304	4,061	4,406	4,372	4,315
1552395_at	8,288	8,411	8,418	8,499	8,181	8,576
1552396_at	5,002	5,330	5,085	5,780	5,605	5,922
1552398_a_at	4,222	4,056	4,281	4,812	4,453	4,703
1552399_a_at	5,767	5,647	5,655	6,159	6,086	6,271
1552400_a_at	7,418	7,331	7,872	7,178	7,435	7,688
1552401_a_at	5,127	5,280	5,346	5,629	5,890	5,960
1552402_at	5,951	6,255	6,093	6,299	6,286	6,395
1552405_at	6,826	6,278	6,967	7,638	7,469	7,613

1552408_at	5,498	5,507	5,425	5,650	5,700	5,829
1552409_a_at	7,759	7,480	7,959	8,364	7,920	8,329
1552410_at	6,696	6,608	6,981	7,460	7,316	7,572
1552411_at	7,717	7,646	7,972	8,127	8,604	8,291
1552412_a_at	5,765	6,033	6,042	6,211	6,498	6,335
1552414_at	6,461	6,008	6,038	7,133	6,822	6,791
1552415_a_at	5,950	6,079	6,337	6,871	6,652	6,657
1552417_a_at	8,223	8,657	7,802	6,811	6,011	6,625
1552418_at	6,951	6,863	7,000	7,213	7,082	7,380
1552419_s_at	7,956	7,774	8,019	8,629	8,700	8,510
1552421_a_at	5,585	5,421	5,776	6,011	5,919	6,109
1552422_at	7,054	6,502	6,972	7,728	7,516	7,386
1552423_at	7,663	7,660	7,750	7,819	7,804	7,860
1552424_at	4,502	4,587	5,278	5,725	5,929	6,129
1552425_a_at	5,790	5,737	6,177	6,557	6,385	6,418
1552426_a_at	11,613	11,479	11,405	11,182	10,577	11,070
1552427_at	7,598	7,578	7,213	5,426	4,794	4,711
1552430_at	3,928	3,903	3,872	4,130	4,203	4,181
1552432_at	6,750	6,830	6,775	6,912	6,897	6,966
1552436_a_at	6,811	6,624	6,484	6,719	6,632	6,759
1552438_a_at	6,576	6,578	6,827	6,733	6,573	6,683
1552439_s_at	4,805	4,338	4,157	4,064	3,912	4,089
1552440_at	6,542	6,321	6,504	7,428	7,863	7,601
1552445_a_at	5,925	5,783	5,939	5,969	6,124	6,001
1552448_a_at	5,319	5,048	5,237	5,269	5,189	5,343
1552449_a_at	5,346	4,683	5,161	5,893	6,061	6,056
1552450_a_at	7,110	6,982	6,801	7,119	7,061	7,202
1552452_at	6,389	6,139	6,149	6,646	6,621	6,735
1552453_a_at	5,283	5,225	5,186	5,203	5,318	5,306
1552455_at	6,752	6,812	7,021	5,771	6,826	5,829
1552456_a_at	6,079	6,240	6,140	7,209	7,554	7,191
1552457_a_at	5,482	6,641	6,047	5,647	7,261	6,526
1552458_at	4,539	4,349	4,781	4,623	4,835	4,548
1552459_a_at	5,914	6,164	5,973	6,882	6,504	6,735
1552461_at	4,535	4,113	4,463	4,723	4,490	4,387
1552463_at	5,304	5,555	5,343	6,044	5,770	5,876
1552466_x_at	4,391	4,769	4,302	5,463	5,455	5,593
1552467_at	7,580	7,347	7,469	8,664	8,431	8,498
1552468_a_at	6,128	6,153	5,917	6,689	6,818	7,021
1552470_a_at	4,719	4,500	4,863	4,613	4,597	4,679
1552472_a_at	8,280	8,400	7,685	8,130	8,551	7,795
1552473_at	9,120	9,119	9,088	9,325	9,041	9,239
1552474_a_at	8,733	8,582	8,492	9,295	8,972	9,414
1552476_s_at	8,364	8,499	8,546	9,033	8,415	8,654
1552477_a_at	5,137	5,297	4,810	4,995	4,786	4,826
1552478_a_at	4,485	4,609	4,514	4,630	4,931	5,090
1552480_s_at	3,423	3,649	3,745	3,639	3,533	3,380
1552481_s_at	6,622	6,498	6,308	6,109	6,422	6,288
1552482_at	5,493	5,387	5,431	5,115	6,037	5,488
1552484_at	6,986	6,769	6,825	7,637	7,506	7,636
1552485_at	6,885	7,588	7,071	9,741	8,945	9,540
1552486_s_at	6,662	7,266	6,233	7,230	7,056	7,256
1552487_a_at	4,963	5,163	5,200	7,258	5,805	6,544
1552489_s_at	5,151	5,089	4,857	6,002	6,350	6,464
1552490_at	5,567	5,874	5,749	5,806	5,777	5,652
1552491_at	6,197	5,580	6,061	6,386	6,644	6,571

1552493_s_at	6,132	5,783	6,079	6,773	6,712	6,999
1552494_at	5,885	5,651	5,779	6,554	6,560	6,677
1552496_a_at	6,289	6,530	6,358	5,896	5,658	5,831
1552497_a_at	6,996	6,419	7,046	7,326	7,349	7,249
1552498_at	5,557	5,598	5,905	5,796	5,726	6,070
1552499_a_at	6,461	6,566	6,613	5,904	5,982	6,179
1552501_a_at	7,830	7,436	7,556	8,056	8,009	8,068
1552502_s_at	3,833	4,265	4,280	5,036	4,925	5,063
1552503_at	6,540	6,272	6,863	7,003	6,756	7,022
1552504_a_at	6,693	6,350	6,444	6,512	6,907	6,591
1552506_at	5,907	5,512	5,573	5,800	5,930	5,904
1552507_at	5,099	5,395	5,266	8,192	7,565	8,291
1552508_at	6,218	6,407	5,849	7,216	6,411	6,799
1552509_a_at	5,442	5,238	5,347	5,556	5,462	5,395
1552510_at	9,157	9,063	8,935	9,752	9,414	9,633
1552511_a_at	4,564	4,747	4,617	4,658	4,528	4,588
1552514_at	4,128	4,387	4,225	4,252	4,192	4,191
1552515_at	5,688	5,595	5,378	5,860	5,963	6,185
1552516_a_at	6,646	6,363	6,225	6,357	6,617	6,646
1552518_s_at	5,637	5,372	5,611	5,221	5,396	5,110
1552519_at	6,842	7,822	6,399	5,323	5,245	5,285
1552520_at	6,909	7,689	7,677	6,171	5,677	5,705
1552521_a_at	7,211	7,331	7,260	4,850	4,653	4,488
1552522_at	3,797	3,752	3,932	3,890	4,258	4,128
1552523_a_at	6,236	5,981	6,123	6,701	6,748	7,007
1552524_at	7,519	7,446	7,329	7,921	7,759	8,003
1552526_at	5,847	6,474	5,989	7,051	6,997	7,107
1552528_at	7,191	7,351	7,377	7,054	6,980	7,199
1552531_a_at	6,125	6,229	6,085	6,059	5,654	5,824
1552532_a_at	4,719	4,972	4,805	5,287	4,959	5,455
1552535_at	7,041	6,993	7,069	7,406	7,128	7,353
1552536_at	6,700	6,188	6,554	6,636	7,177	6,909
1552538_a_at	4,457	4,223	4,265	5,146	5,012	5,275
1552540_s_at	7,122	7,040	7,169	8,041	8,013	8,026
1552541_at	7,952	7,746	8,021	8,708	8,393	8,796
1552542_s_at	5,654	5,702	5,654	5,840	5,873	5,670
1552543_a_at	5,685	5,947	5,648	5,567	5,181	5,398
1552544_at	6,049	6,171	6,073	6,557	6,491	6,704
1552546_a_at	5,303	5,591	5,429	6,616	5,824	6,712
1552548_at	5,912	5,432	5,724	6,343	6,215	6,533
1552549_a_at	7,239	7,347	7,441	8,263	7,928	7,956
1552552_s_at	5,389	5,472	5,818	5,633	5,253	5,392
1552553_a_at	5,603	5,627	5,295	5,558	5,558	5,584
1552554_a_at	6,075	6,278	6,267	6,813	6,777	6,507
1552555_at	8,016	7,981	8,060	8,254	8,210	8,187
1552557_a_at	7,192	7,013	7,531	4,785	5,062	4,791
1552558_a_at	8,145	8,066	8,261	8,934	8,672	8,969
1552559_a_at	6,630	6,548	6,322	7,508	6,658	7,177
1552562_at	6,257	6,343	6,457	6,236	6,210	5,976
1552563_a_at	4,845	4,515	4,958	4,211	4,082	4,131
1552564_at	5,516	4,686	5,112	5,726	5,772	5,884
1552566_at	5,328	5,256	5,469	5,518	5,672	5,566
1552568_at	6,028	6,407	6,611	7,666	7,274	7,351
1552569_a_at	5,701	5,955	6,415	6,484	6,522	6,694
1552570_at	4,447	4,660	4,485	4,750	4,701	4,687
1552572_a_at	3,793	4,316	3,800	3,739	4,017	3,781

1552573_s_at	4,513	4,595	4,460	5,237	5,124	5,157
1552575_a_at	7,862	6,943	7,772	6,395	6,454	6,394
1552576_at	6,597	6,014	6,057	6,169	6,251	6,475
1552578_a_at	4,600	5,528	4,994	4,347	4,157	4,285
1552579_a_at	5,753	5,740	5,628	6,546	6,795	6,529
1552580_at	8,326	8,512	8,343	7,719	7,702	7,838
1552582_at	5,026	4,440	4,685	5,209	5,102	4,971
1552583_s_at	5,046	4,733	5,384	5,145	5,178	5,199
1552584_at	6,079	5,887	6,181	6,629	6,146	6,551
1552585_s_at	4,171	4,185	4,224	4,426	4,439	4,466
1552586_at	6,835	7,126	6,925	8,112	7,844	8,010
1552587_at	6,218	5,744	5,788	5,640	5,286	5,501
1552588_a_at	4,450	4,487	4,368	4,811	4,521	4,422
1552590_a_at	5,051	5,246	5,045	4,876	4,721	4,870
1552592_at	3,727	3,901	4,019	3,900	4,021	3,915
1552594_at	7,349	7,496	7,516	7,852	7,586	7,874
1552596_at	7,144	7,145	7,433	8,076	8,173	8,384
1552598_at	6,756	6,426	6,684	7,102	7,351	7,258
1552599_at	4,274	4,497	4,322	4,475	4,640	4,540
1552602_at	6,597	6,342	6,601	7,137	6,771	6,893
1552604_at	5,564	6,098	5,735	6,529	6,550	6,539
1552605_s_at	5,093	4,604	4,801	5,996	5,451	5,765
1552607_at	6,847	6,896	6,847	7,321	6,985	7,269
1552608_at	6,464	6,451	6,630	7,564	7,541	7,397
1552609_s_at	6,213	6,465	5,951	6,716	6,879	7,011
1552610_a_at	7,638	7,867	7,642	8,481	8,585	8,198
1552611_a_at	7,354	7,527	7,495	8,088	7,206	8,077
1552612_at	7,112	7,009	6,948	6,523	6,629	6,590
1552613_s_at	6,429	6,265	6,187	5,984	6,338	6,220
1552615_at	9,215	9,034	9,162	9,877	9,866	9,997
1552616_a_at	6,375	6,418	6,347	6,572	6,318	6,504
1552617_a_at	9,834	9,751	9,592	7,955	7,952	8,255
1552618_at	9,214	8,879	9,258	9,500	9,003	9,365
1552619_a_at	10,062	10,374	9,020	8,661	9,118	7,235
1552620_at	7,787	7,690	7,946	8,007	7,616	7,832
1552621_at	9,370	8,287	8,558	8,182	8,420	8,128
1552622_s_at	7,994	7,146	7,060	6,105	6,465	6,358
1552623_at	6,808	6,857	6,943	7,037	6,758	6,909
1552625_a_at	7,270	7,257	7,103	6,227	6,923	6,160
1552626_a_at	6,012	6,874	6,716	5,475	5,285	5,244
1552627_a_at	5,846	6,107	5,505	6,159	6,525	6,411
1552628_a_at	8,161	8,056	7,851	6,744	6,632	6,999
1552629_a_at	5,906	5,875	5,691	5,832	5,712	5,941
1552630_a_at	6,578	6,290	6,410	6,713	6,517	6,904
1552631_a_at	6,334	6,582	6,715	7,729	7,729	8,081
1552632_a_at	5,901	6,224	6,139	6,359	6,113	6,796
1552633_at	7,063	7,465	7,341	6,745	6,644	6,352
1552634_a_at	7,350	8,020	7,640	6,764	6,225	6,144
1552637_at	4,840	4,636	4,912	5,347	5,005	5,158
1552639_at	7,276	6,904	7,062	7,672	7,527	7,696
1552641_s_at	7,741	7,279	7,542	7,943	7,367	7,909
1552643_at	8,201	8,248	8,131	8,036	7,875	8,517
1552644_a_at	6,702	6,841	6,722	5,706	6,329	6,049
1552646_at	7,030	6,936	6,886	7,843	7,615	7,927
1552648_a_at	7,385	7,289	7,637	8,054	7,383	7,386
1552649_a_at	5,388	5,898	6,204	6,898	6,293	6,461

1552651_a_at	7,618	7,275	7,531	8,568	7,848	8,252
1552652_at	8,145	8,493	8,089	9,273	8,295	8,265
1552656_s_at	6,921	6,545	6,439	6,393	6,539	6,807
1552657_a_at	6,978	6,669	6,799	7,551	7,573	7,523
1552658_a_at	5,838	5,382	4,960	7,270	8,037	6,984
1552660_a_at	6,079	6,184	5,858	6,079	6,106	6,103
1552661_at	3,546	3,456	3,528	3,397	3,605	3,671
1552662_a_at	6,372	6,474	6,377	6,665	6,169	6,358
1552663_a_at	7,396	7,470	7,573	7,667	7,672	7,554
1552664_at	7,400	8,279	8,410	8,176	8,203	8,273
1552665_at	6,735	6,256	6,400	7,877	7,146	7,554
1552666_a_at	4,401	4,311	4,501	4,898	4,757	4,943
1552667_a_at	6,532	7,162	6,230	6,969	7,078	7,177
1552669_at	6,015	6,442	6,180	6,421	6,090	6,151
1552670_a_at	6,817	6,665	6,840	6,862	6,471	6,663
1552671_a_at	6,686	6,710	6,657	7,202	6,653	7,250
1552672_a_at	6,342	6,131	6,388	6,086	6,139	6,380
1552673_at	3,950	3,904	4,154	4,898	4,488	4,401
1552674_at	4,027	3,910	4,100	4,111	3,927	3,830
1552675_at	3,731	4,141	3,715	4,604	4,473	4,612
1552676_at	7,689	7,796	7,781	8,386	8,204	8,469
1552677_a_at	7,311	7,113	7,263	7,532	7,494	7,727
1552678_a_at	8,435	8,768	8,324	7,210	6,845	6,805
1552680_a_at	7,847	8,167	7,401	7,151	6,772	6,408
1552682_a_at	6,179	5,849	6,122	5,892	5,900	4,809
1552684_a_at	6,465	6,475	6,320	6,154	5,783	6,409
1552685_a_at	6,162	5,943	6,080	7,323	6,963	7,127
1552686_at	6,755	6,816	6,771	7,346	7,104	7,020
1552687_a_at	6,562	6,267	6,127	6,628	6,667	6,704
1552689_at	8,175	8,540	8,714	8,165	7,874	8,074
1552690_a_at	5,910	6,311	6,022	6,494	6,481	6,798
1552691_at	3,458	3,695	3,564	3,536	3,445	3,567
1552693_at	4,463	4,610	4,700	4,780	4,514	4,556
1552694_at	4,669	4,953	4,334	4,561	4,433	5,005
1552695_a_at	6,036	6,456	6,000	5,992	5,801	6,193
1552696_at	5,524	5,631	5,636	5,595	5,661	6,057
1552698_at	7,226	7,343	7,320	7,343	7,204	7,275
1552701_a_at	5,564	6,397	6,363	8,336	7,385	7,582
1552703_s_at	4,953	4,672	4,865	8,182	7,114	7,261
1552705_at	4,235	4,533	4,222	3,721	4,135	3,544
1552708_a_at	5,432	6,310	4,837	5,124	5,023	5,054
1552711_a_at	8,735	8,529	9,291	8,585	9,000	8,566
1552712_a_at	7,878	7,833	7,645	7,507	7,577	7,741
1552713_a_at	5,890	5,701	5,957	6,564	6,308	6,305
1552714_at	5,034	5,273	5,164	5,560	5,225	5,535
1552715_a_at	4,543	4,209	4,434	4,538	4,376	4,264
1552716_at	5,853	5,668	6,207	6,252	5,991	6,193
1552717_s_at	6,177	6,077	5,996	6,484	6,347	6,340
1552718_at	6,005	5,902	6,692	7,063	7,001	7,120
1552719_at	7,799	7,415	7,548	7,136	7,703	7,394
1552721_a_at	4,413	4,766	5,130	4,786	5,818	5,075
1552722_at	3,673	3,656	3,826	3,866	3,805	3,815
1552724_at	7,091	7,295	7,181	7,525	7,204	7,702
1552725_s_at	6,131	5,628	6,363	7,484	6,953	7,440
1552726_at	5,673	5,832	5,863	6,866	6,606	6,562
1552727_s_at	5,504	5,589	5,637	5,570	5,393	5,346

1552729_at	7,980	8,009	7,928	7,950	8,003	8,179
1552730_at	5,450	5,895	5,431	6,065	6,012	6,296
1552731_at	5,225	4,933	4,917	6,062	5,695	6,012
1552732_at	3,867	3,771	3,886	4,093	4,161	3,728
1552733_at	5,129	4,882	5,101	4,972	5,812	4,935
1552734_at	5,264	5,681	5,107	6,006	6,185	5,947
1552735_at	5,775	5,853	6,106	5,637	5,553	5,511
1552736_a_at	7,726	7,899	7,557	5,424	5,199	5,281
1552737_s_at	5,165	4,953	4,897	4,780	4,525	4,467
1552738_a_at	5,630	5,733	5,815	5,200	5,225	5,332
1552739_s_at	7,286	7,673	7,543	6,591	6,325	6,843
1552740_at	3,746	4,464	3,824	3,764	3,675	3,681
1552742_at	5,473	5,692	5,164	5,108	4,827	5,020
1552743_at	6,764	6,818	6,768	6,930	6,807	6,806
1552745_at	5,985	6,142	5,288	5,260	5,107	5,409
1552747_a_at	3,794	3,649	4,293	4,113	4,069	4,145
1552749_a_at	8,495	8,520	8,601	8,869	8,928	8,890
1552750_at	4,230	4,282	4,634	5,283	5,204	5,286
1552751_a_at	7,308	7,046	7,735	8,106	8,458	8,358
1552752_a_at	6,010	6,076	6,180	5,896	5,934	5,812
1552754_a_at	4,567	4,721	4,902	4,214	3,938	4,055
1552755_at	4,223	4,162	4,356	4,529	4,354	4,429
1552757_s_at	6,188	5,879	5,817	5,918	5,935	5,887
1552758_at	5,342	5,168	5,395	5,805	5,773	6,066
1552760_at	5,712	5,058	5,084	5,289	4,757	4,899
1552761_at	7,914	7,758	7,745	8,333	8,288	8,663
1552763_at	3,886	3,934	3,729	3,987	3,861	4,200
1552765_x_at	5,958	6,076	5,849	5,805	5,646	5,749
1552766_at	5,559	5,313	5,001	4,345	4,353	4,504
1552767_a_at	10,229	9,686	9,920	4,077	4,340	4,203
1552768_at	7,534	7,717	7,266	8,071	8,402	8,292
1552769_at	7,547	7,895	7,522	7,568	7,548	7,468
1552770_s_at	6,037	6,041	6,083	6,385	6,254	6,227
1552771_a_at	5,238	5,230	4,973	5,445	5,490	5,547
1552772_at	7,397	7,726	7,492	6,264	6,002	6,296
1552773_at	8,653	9,343	8,653	6,332	6,115	6,318
1552774_a_at	6,347	6,676	5,997	6,362	6,796	6,404
1552775_at	6,385	6,334	6,640	6,446	6,413	6,409
1552777_a_at	5,609	5,327	5,602	5,589	5,450	5,731
1552779_a_at	4,283	4,134	4,237	4,606	4,330	4,357
1552782_at	5,570	5,435	4,890	4,867	5,010	4,959
1552783_at	3,814	3,933	3,581	3,785	3,723	3,861
1552785_at	5,230	5,202	5,126	5,306	5,703	5,328
1552787_at	6,001	6,388	6,250	5,339	5,445	4,941
1552788_a_at	7,567	7,259	7,591	7,245	7,151	7,389
1552789_at	7,071	7,122	6,612	5,310	6,088	5,257
1552790_a_at	9,292	9,275	9,157	7,615	8,255	6,924
1552791_a_at	3,945	3,689	4,091	4,488	3,936	4,002
1552792_at	5,466	5,764	5,530	4,639	4,932	4,620
1552793_at	6,127	5,677	6,165	6,207	5,814	6,115
1552794_a_at	5,815	5,272	4,974	4,600	4,233	4,383
1552796_a_at	5,485	5,391	5,655	6,327	6,032	6,378
1552797_s_at	4,807	4,950	4,731	4,926	4,753	4,735
1552798_a_at	6,270	6,210	6,402	7,122	7,104	7,262
1552799_at	6,215	6,198	6,291	7,199	6,573	6,898
1552800_at	7,675	7,463	7,497	8,225	8,013	8,222

1552801_at	5,744	5,063	5,332	5,960	5,604	6,007
1552803_a_at	6,131	6,151	6,285	6,292	6,002	6,355
1552804_a_at	7,034	6,950	6,659	7,299	7,185	7,631
1552806_a_at	5,863	5,419	6,015	6,806	7,033	7,111
1552807_a_at	6,342	6,887	7,031	7,529	7,796	8,042
1552808_at	4,227	3,969	4,073	4,552	4,612	4,699
1552809_at	4,719	4,520	4,558	4,867	5,076	5,206
1552811_at	8,114	7,748	7,493	8,295	8,475	8,453
1552812_a_at	6,483	6,143	6,488	6,044	5,439	5,545
1552813_at	6,274	5,876	5,900	6,609	6,176	6,280
1552814_a_at	5,998	6,237	5,969	6,297	6,207	6,349
1552816_at	3,855	4,074	3,700	4,133	4,053	4,061
1552818_a_at	5,005	5,400	4,719	5,243	4,785	5,084
1552819_at	6,661	6,695	6,435	6,876	6,366	6,737
1552822_at	4,660	5,642	5,269	6,291	5,978	6,068
1552825_at	5,536	5,510	5,829	5,598	5,560	5,658
1552826_at	3,789	3,680	3,625	3,711	3,679	3,749
1552827_s_at	5,703	5,515	5,776	6,110	5,804	5,890
1552828_at	6,371	6,261	6,198	6,561	6,120	6,155
1552829_at	7,150	6,624	6,869	7,013	7,003	6,898
1552830_at	6,990	6,988	7,083	8,004	7,332	7,744
1552833_at	7,066	7,053	6,992	7,283	7,051	7,525
1552834_at	7,141	7,306	7,729	7,851	8,315	7,989
1552835_at	5,070	5,526	4,992	5,022	5,436	5,212
1552836_at	7,297	7,071	7,362	7,802	7,210	7,369
1552837_at	3,482	3,744	3,685	3,427	3,516	3,490
1552839_at	5,749	5,601	5,816	6,316	6,033	6,120
1552841_s_at	4,458	4,444	4,656	5,216	4,715	4,930
1552842_at	6,445	6,435	6,291	7,271	7,051	7,317
1552843_at	6,285	5,983	6,248	6,654	6,531	6,647
1552845_at	6,819	6,560	6,666	6,531	6,887	6,689
1552846_s_at	7,877	8,242	8,120	7,429	7,516	7,701
1552848_a_at	8,210	7,545	8,074	5,864	5,760	5,845
1552849_at	5,529	5,526	5,317	4,548	4,312	4,602
1552851_at	3,784	3,802	4,093	4,090	3,916	3,989
1552852_a_at	5,274	5,364	5,458	5,445	5,186	5,281
1552853_at	6,617	6,561	6,172	7,218	6,420	7,136
1552854_a_at	6,239	6,134	5,965	6,038	5,921	6,026
1552856_at	4,824	5,048	5,264	5,591	5,295	5,917
1552857_a_at	6,407	5,634	6,528	7,121	6,695	6,725
1552858_at	3,412	3,471	3,771	3,532	3,517	3,714
1552860_at	6,762	6,698	6,441	6,996	6,942	7,097
1552862_at	5,643	5,986	5,936	6,218	5,691	5,557
1552863_a_at	8,626	8,402	8,580	9,037	8,757	9,028
1552865_a_at	7,070	7,247	7,150	7,497	7,439	7,473
1552867_at	4,991	4,661	4,880	4,314	4,433	4,320
1552868_at	7,445	7,210	7,799	7,741	7,664	7,806
1552870_s_at	4,350	4,309	4,422	4,468	4,269	4,136
1552872_at	8,298	7,564	7,693	8,774	8,116	8,366
1552873_s_at	7,599	7,719	7,576	7,688	7,730	7,908
1552875_a_at	4,631	4,713	5,002	5,354	4,990	5,406
1552876_at	4,704	5,115	4,967	5,209	5,076	5,245
1552877_s_at	6,146	6,104	6,171	6,752	6,605	6,637
1552878_at	3,495	3,550	3,554	3,735	3,565	3,829
1552879_a_at	6,466	6,977	6,502	6,281	6,233	6,262
1552880_at	4,898	5,015	4,861	5,593	5,296	5,696

1552882_a_at	6,214	6,233	6,525	6,289	6,184	6,438
1552884_at	6,853	6,719	6,651	7,804	7,373	7,730
1552885_a_at	8,156	7,905	7,733	8,612	8,644	8,715
1552887_at	7,426	7,651	7,731	8,227	7,917	7,973
1552889_a_at	8,127	8,190	8,245	8,462	8,402	8,413
1552890_a_at	5,989	5,875	5,955	7,029	6,887	7,052
1552892_at	7,620	7,690	7,640	8,176	8,202	8,614
1552893_at	6,675	6,624	6,546	6,722	6,798	6,885
1552895_a_at	3,805	3,900	3,958	4,232	4,226	4,318
1552897_a_at	9,696	9,644	9,698	4,768	4,781	5,043
1552898_a_at	5,169	5,056	5,252	5,163	5,048	5,366
1552899_at	5,986	5,843	5,885	6,954	6,492	6,054
1552900_a_at	4,524	4,709	4,737	4,444	4,318	4,482
1552902_a_at	5,658	5,540	5,635	5,849	5,698	5,938
1552903_at	6,165	6,296	6,072	7,079	6,652	6,724
1552904_at	3,707	3,741	3,750	3,652	3,786	3,874
1552906_at	3,789	3,611	3,609	3,905	3,764	3,861
1552908_at	4,567	4,408	4,517	5,290	4,743	5,306
1552910_at	6,260	6,020	6,236	6,617	6,422	6,727
1552911_at	6,143	6,188	6,573	7,711	7,669	7,914
1552912_a_at	4,553	4,689	4,762	5,168	4,882	4,848
1552913_at	3,345	3,868	3,657	3,421	3,402	3,462
1552914_a_at	6,910	6,844	6,908	7,220	7,056	7,531
1552915_at	6,052	5,800	5,868	7,580	6,436	6,540
1552917_at	6,707	6,365	6,546	7,334	7,008	7,214
1552919_at	4,666	4,435	4,401	4,781	5,003	5,269
1552921_a_at	7,887	7,852	7,221	6,624	6,413	6,238
1552922_at	4,861	4,864	5,032	5,127	5,122	5,001
1552923_a_at	6,736	6,522	6,408	7,075	6,919	7,119
1552924_a_at	6,422	6,541	6,700	6,459	6,110	6,462
1552925_at	5,419	5,569	5,581	5,858	5,809	5,966
1552927_at	6,541	7,333	6,480	5,936	6,217	6,455
1552928_s_at	6,595	7,128	6,325	4,771	5,005	4,794
1552929_at	4,522	4,397	4,886	4,961	4,943	4,985
1552930_at	7,843	8,171	8,058	8,224	8,381	8,245
1552931_a_at	6,845	7,087	6,601	7,336	7,531	7,503
1552932_at	5,453	5,004	5,462	6,287	6,397	6,311
1552933_at	4,395	4,494	4,625	4,481	4,414	4,378
1552935_at	7,026	7,007	7,075	7,448	7,585	7,838
1552937_s_at	8,441	7,889	8,259	7,661	7,036	7,425
1552938_at	6,454	6,290	6,451	6,992	6,753	7,078
1552939_at	3,591	3,508	3,672	3,763	3,661	3,721
1552940_at	4,724	4,540	4,874	5,267	5,035	5,374
1552942_at	6,652	6,451	6,744	7,047	7,248	7,412
1552943_at	3,673	3,521	3,746	3,763	3,643	3,666
1552944_a_at	8,929	8,614	8,900	9,627	9,375	9,788
1552946_at	7,048	7,413	6,905	4,743	4,665	4,547
1552947_x_at	7,666	7,623	7,721	5,717	5,396	5,506
1552948_at	6,238	6,633	6,643	8,598	8,338	8,753
1552950_at	4,218	3,783	3,765	4,665	4,445	4,791
1552952_at	4,571	4,332	4,527	4,516	4,286	4,279
1552953_a_at	7,464	7,430	7,156	7,530	7,427	7,605
1552954_at	7,783	7,976	7,744	6,245	6,528	6,334
1552955_at	6,261	6,483	6,448	6,983	6,973	7,232
1552957_at	4,449	4,759	4,659	5,660	5,442	5,598
1552960_at	6,919	6,742	6,743	7,328	7,368	7,394

1552961_at	6,537	6,560	6,363	7,535	7,407	7,583
1552964_at	7,193	6,501	6,482	7,344	7,162	7,350
1552965_a_at	6,149	5,972	6,466	6,378	6,050	6,295
1552967_at	5,722	5,354	5,515	5,800	5,523	5,736
1552969_a_at	4,583	4,384	4,222	4,759	4,793	4,946
1552970_s_at	4,232	4,030	4,389	4,665	4,502	4,837
1552971_at	4,955	5,047	4,749	5,847	5,755	5,792
1552972_at	4,026	4,178	3,909	4,137	3,736	3,750
1552973_at	6,700	6,298	6,482	6,959	7,098	7,193
1552974_at	3,355	3,373	3,679	4,155	4,029	4,363
1552975_x_at	3,570	3,110	3,753	4,442	4,144	4,658
1552976_at	6,593	6,800	6,879	7,429	7,073	7,235
1552977_a_at	7,118	6,951	6,799	6,942	6,620	7,043
1552978_a_at	7,554	7,700	7,164	7,393	7,617	7,906
1552979_at	5,017	5,363	5,311	5,000	4,863	5,034
1552980_at	4,856	5,431	5,607	5,539	5,442	5,691
1552982_a_at	6,822	7,037	6,823	6,784	6,534	6,672
1552985_at	5,885	5,734	6,087	6,392	6,195	6,182
1552986_at	6,719	6,099	6,255	7,089	6,722	7,015
1552987_a_at	3,635	3,752	3,658	3,679	3,630	3,769
1552988_at	4,647	4,865	4,103	4,414	4,223	4,234
1552990_at	5,326	5,264	5,119	5,839	5,784	5,902
1552991_at	5,869	5,709	5,590	5,958	6,107	5,891
1552993_at	5,233	5,444	5,321	5,660	5,163	5,632
1552995_at	7,769	7,677	7,816	8,158	7,965	8,211
1552997_a_at	6,581	6,491	6,814	7,139	6,968	7,059
1552998_at	5,856	5,615	5,630	6,772	6,243	6,753
1552999_a_at	6,156	6,203	5,960	6,981	6,610	6,719
1553002_at	4,768	4,446	4,915	5,462	5,360	5,256
1553003_at	5,997	6,069	6,207	6,269	6,333	6,224
1553004_at	5,162	5,222	5,223	5,332	5,280	5,423
1553006_at	5,567	5,927	5,597	6,319	6,358	6,522
1553007_a_at	5,800	5,681	6,316	6,612	6,270	6,505
1553009_s_at	6,658	6,410	6,321	6,508	6,572	6,500
1553011_at	6,409	6,270	6,209	6,294	6,715	6,439
1553013_at	4,932	4,862	4,836	5,381	5,185	5,254
1553015_a_at	8,922	8,509	8,761	8,548	8,055	8,580
1553016_at	6,938	6,639	6,528	6,791	6,713	6,833
1553019_at	6,857	6,915	6,681	7,373	7,177	7,277
1553020_at	4,950	5,150	4,807	5,482	5,754	5,597
1553021_s_at	6,652	6,580	6,774	6,909	6,797	7,085
1553022_at	3,921	4,137	4,138	4,654	4,180	4,450
1553023_a_at	6,347	5,879	5,846	6,516	6,319	6,491
1553024_at	3,725	4,055	3,856	3,880	3,826	3,850
1553025_at	4,701	4,759	4,960	5,166	5,065	5,035
1553027_a_at	8,099	8,258	7,826	7,588	7,807	7,789
1553028_at	6,545	6,145	6,171	6,924	6,847	7,006
1553030_a_at	6,763	6,615	6,500	6,687	6,631	7,054
1553031_at	6,252	6,143	5,632	6,162	6,666	6,369
1553032_at	5,417	5,438	5,540	5,900	5,847	5,884
1553033_at	5,172	5,176	4,984	5,772	5,571	5,447
1553034_at	5,507	5,393	5,569	7,010	6,706	7,508
1553036_at	4,718	4,283	4,582	4,684	4,643	4,550
1553037_a_at	5,887	5,906	6,081	6,199	6,437	6,511
1553039_a_at	8,256	8,329	8,419	8,523	8,506	8,871
1553041_at	7,098	7,342	7,152	7,961	7,937	8,012

1553042_a_at	6,174	6,281	6,374	6,925	6,541	6,702
1553043_a_at	6,603	6,524	6,681	7,272	6,729	6,784
1553044_at	6,060	6,183	6,439	6,689	6,337	6,470
1553045_at	5,715	5,944	6,103	6,536	6,675	6,859
1553046_s_at	7,244	6,964	7,168	7,778	7,598	7,730
1553047_at	7,478	7,513	7,694	8,709	8,466	8,731
1553048_a_at	7,870	7,402	7,555	7,257	7,003	7,300
1553051_s_at	5,683	5,635	5,632	6,658	6,473	6,070
1553052_at	5,781	5,850	5,657	5,947	5,980	6,254
1553053_at	6,368	6,372	6,464	7,086	6,864	7,233
1553055_a_at	4,615	4,472	4,154	5,791	4,886	5,432
1553057_at	6,262	5,764	6,249	6,634	6,489	6,662
1553058_at	6,029	5,636	5,467	6,449	6,385	6,580
1553059_at	5,715	5,199	5,079	5,721	5,349	5,658
1553060_at	6,407	5,973	6,094	6,731	6,486	6,952
1553061_at	6,058	5,642	6,092	6,751	6,566	6,594
1553062_at	6,423	6,182	6,110	6,843	6,548	6,592
1553063_at	6,905	6,838	6,830	7,710	7,077	7,485
1553064_at	6,892	6,210	6,835	7,745	8,054	8,199
1553066_at	5,753	5,672	5,744	6,074	6,090	5,936
1553067_a_at	6,392	6,216	6,845	6,689	6,746	7,000
1553069_at	5,662	5,644	5,550	5,638	5,238	5,655
1553070_a_at	5,887	5,621	5,536	5,848	5,317	5,888
1553071_a_at	7,211	6,732	6,818	7,620	7,640	7,277
1553072_at	5,872	5,995	5,989	6,528	6,645	6,417
1553074_at	5,565	5,653	6,126	6,412	6,225	6,283
1553075_a_at	3,589	3,523	3,687	3,668	3,795	3,712
1553076_at	5,158	5,242	5,105	5,500	5,515	5,591
1553077_at	5,040	4,778	5,260	5,468	5,310	5,213
1553078_at	6,504	6,297	6,649	6,945	6,697	6,769
1553079_at	5,313	5,186	5,340	6,538	6,177	6,688
1553080_at	4,902	4,866	4,980	4,891	4,871	4,878
1553081_at	7,483	7,201	7,353	8,158	8,195	8,203
1553082_at	6,558	6,532	6,683	7,197	6,834	7,240
1553083_at	6,660	6,682	6,792	7,135	6,883	7,141
1553084_at	4,256	4,255	4,540	4,465	4,382	4,472
1553086_at	4,491	4,786	4,439	4,921	4,827	4,827
1553087_at	6,072	6,048	6,081	6,183	6,178	6,000
1553088_a_at	8,507	8,363	8,318	9,770	9,774	9,842
1553089_a_at	5,413	5,833	5,475	5,700	5,599	5,893
1553093_a_at	4,737	4,855	5,225	5,591	5,486	5,457
1553094_at	6,390	6,209	6,293	8,746	8,795	8,856
1553096_s_at	8,997	9,050	8,740	8,637	8,295	8,750
1553099_at	7,285	7,555	6,972	6,294	5,970	6,108
1553101_a_at	7,785	7,544	7,549	7,522	6,806	7,473
1553102_a_at	5,627	5,469	5,118	4,758	4,769	4,605
1553103_at	9,255	9,445	9,627	8,439	8,798	7,992
1553105_s_at	6,669	6,461	6,042	5,191	4,631	4,955
1553106_at	6,897	6,765	5,943	7,273	6,832	7,219
1553107_s_at	7,981	7,950	7,996	8,681	8,467	8,683
1553108_at	6,230	5,875	5,989	7,158	7,270	7,345
1553111_a_at	7,839	7,535	7,781	5,796	6,053	5,700
1553112_s_at	8,537	8,426	7,420	7,673	8,081	7,839
1553113_s_at	9,912	10,155	9,513	8,680	9,047	8,622
1553114_a_at	6,657	6,657	6,780	6,870	6,662	6,820
1553115_at	8,062	7,829	8,087	8,739	8,509	8,917

1553117_a_at	6,438	6,372	6,202	6,076	5,987	6,120
1553118_at	9,850	9,515	9,752	7,803	7,472	7,605
1553120_at	4,738	4,633	4,458	4,709	4,933	4,999
1553122_s_at	6,770	6,764	5,961	4,455	4,578	4,855
1553123_at	7,510	7,067	7,293	8,475	8,479	8,721
1553125_x_at	6,519	6,789	6,544	8,189	8,228	8,455
1553126_a_at	4,214	4,072	4,271	4,485	4,351	4,539
1553127_a_at	7,067	6,774	6,715	7,356	7,404	7,530
1553129_at	4,178	4,133	4,241	4,475	5,020	4,421
1553130_at	7,697	7,299	7,606	8,049	7,757	7,774
1553131_a_at	6,406	6,305	6,300	7,053	6,720	7,108
1553132_a_at	3,885	5,708	4,228	3,721	3,666	3,773
1553133_at	7,279	8,537	7,661	7,301	7,062	6,627
1553134_s_at	4,382	4,680	3,959	4,300	4,163	4,104
1553136_at	8,498	8,469	8,574	8,494	8,705	8,592
1553137_s_at	5,623	5,534	5,516	6,024	5,515	5,930
1553138_a_at	7,481	7,175	7,394	6,041	6,257	6,249
1553139_s_at	6,186	6,270	6,192	6,530	6,133	6,442
1553140_at	5,722	5,638	5,543	6,012	6,105	6,045
1553141_at	5,149	5,592	5,204	5,702	5,323	5,545
1553142_at	3,758	4,058	3,775	5,271	4,720	4,740
1553145_at	7,987	7,604	7,202	6,175	5,917	5,599
1553146_at	5,110	5,540	5,778	5,626	5,298	5,419
1553147_at	3,975	4,094	4,139	3,856	4,056	4,061
1553148_a_at	6,489	6,467	5,667	5,183	5,602	4,950
1553150_at	4,203	4,317	4,344	4,494	4,443	4,476
1553151_at	4,668	4,589	4,886	5,238	5,270	5,788
1553153_at	7,290	7,310	7,329	7,824	7,661	7,673
1553155_x_at	5,868	6,209	6,266	6,848	6,625	6,580
1553156_at	6,649	6,204	7,128	7,456	7,435	7,621
1553157_at	6,239	6,223	6,479	7,530	7,500	7,682
1553158_at	6,221	6,163	5,913	6,699	6,514	6,571
1553159_at	7,431	7,536	7,210	7,894	7,719	7,760
1553162_x_at	6,867	6,488	6,724	6,654	6,563	6,882
1553163_at	6,979	6,842	7,154	7,198	6,995	7,295
1553165_at	5,434	5,861	5,604	6,888	6,751	6,836
1553166_at	7,463	7,268	7,254	8,036	7,648	7,713
1553167_a_at	6,656	7,107	6,324	6,156	6,180	5,370
1553168_at	6,844	6,993	7,144	7,304	7,132	7,516
1553169_at	7,197	7,126	7,040	7,098	7,016	7,105
1553171_x_at	5,889	5,802	5,690	6,253	5,766	5,975
1553172_at	8,428	8,067	8,510	9,076	8,327	8,637
1553174_at	6,816	6,875	6,992	7,721	7,249	7,617
1553175_s_at	5,802	5,976	5,718	6,274	5,767	6,002
1553176_at	4,724	4,801	4,796	5,105	5,079	5,231
1553177_at	5,214	5,531	5,993	5,354	5,370	5,370
1553178_a_at	7,791	7,841	7,851	8,064	8,049	8,263
1553179_at	8,136	7,923	7,668	6,323	6,436	6,312
1553180_at	4,402	4,649	3,910	3,559	3,264	3,686
1553181_at	6,568	6,530	6,404	6,916	6,907	6,911
1553183_at	4,822	4,748	5,514	4,623	4,701	4,485
1553185_at	11,346	11,266	10,657	7,829	8,925	8,159
1553186_x_at	10,401	10,321	9,779	6,798	8,179	7,068
1553188_s_at	6,630	5,970	6,340	7,401	7,051	7,169
1553190_s_at	7,273	7,341	7,389	7,833	7,763	7,820
1553191_at	4,128	3,960	3,994	4,104	4,197	4,073

1553192_at	3,821	3,871	3,873	3,781	3,676	3,707
1553193_at	4,183	5,101	4,083	4,797	5,152	4,575
1553194_at	5,390	5,171	4,492	5,519	6,720	6,216
1553196_a_at	5,833	5,677	5,575	5,999	5,854	6,044
1553197_at	4,680	4,259	4,319	5,041	5,269	4,969
1553199_at	3,873	4,012	3,631	4,296	4,351	4,880
1553202_at	5,909	5,923	6,060	6,096	6,177	6,336
1553204_at	6,459	6,666	6,810	7,596	7,591	7,811
1553205_at	7,335	7,386	7,510	7,686	7,815	7,913
1553207_at	7,048	7,371	7,341	8,719	8,763	8,919
1553208_s_at	5,445	5,608	5,520	5,426	5,317	5,398
1553209_at	6,634	6,228	6,496	7,191	6,852	6,896
1553211_at	5,465	5,487	5,264	6,168	6,113	6,267
1553212_at	6,134	6,433	6,688	7,554	7,114	7,618
1553213_a_at	4,993	4,875	5,380	5,962	5,881	5,829
1553214_a_at	3,755	3,667	3,812	3,889	3,698	4,104
1553215_s_at	5,592	5,725	5,636	6,993	6,340	6,839
1553216_at	6,472	6,204	6,035	6,076	5,929	5,958
1553217_s_at	6,029	6,108	5,502	5,730	5,902	5,826
1553218_a_at	9,078	8,991	8,791	8,069	8,064	8,032
1553219_a_at	6,021	5,796	5,875	5,824	6,032	5,944
1553220_at	7,390	7,681	8,150	6,504	6,194	5,868
1553221_at	6,678	6,852	7,106	7,121	7,077	6,833
1553222_at	9,267	9,233	9,027	9,816	9,553	9,756
1553224_at	4,870	4,472	5,277	4,616	4,533	4,794
1553225_s_at	6,028	6,175	6,166	6,145	5,810	6,011
1553226_at	6,224	6,165	6,286	7,076	7,089	6,920
1553227_s_at	7,077	7,601	7,038	6,245	6,347	5,761
1553228_at	3,708	4,711	4,488	4,567	4,410	4,486
1553229_at	6,552	6,256	6,318	5,991	6,048	6,159
1553232_at	6,924	6,808	6,682	7,595	7,526	7,369
1553234_at	7,685	7,080	7,163	8,038	7,814	8,143
1553235_at	6,787	6,191	6,014	6,978	6,697	6,786
1553237_x_at	7,157	6,582	6,660	7,325	6,818	6,897
1553238_a_at	7,857	7,827	8,054	8,503	8,305	8,645
1553239_at	7,522	7,533	7,742	8,864	8,591	8,809
1553241_at	5,460	5,202	5,280	5,657	5,485	5,402
1553243_at	4,937	4,925	4,783	5,004	4,828	4,888
1553244_at	6,407	6,650	6,388	5,139	5,147	5,090
1553246_a_at	7,165	7,009	7,060	7,455	7,522	7,547
1553247_a_at	4,913	5,662	5,173	4,188	4,175	4,026
1553248_at	6,925	6,431	6,549	7,510	7,488	7,871
1553249_at	5,536	5,650	5,504	5,941	5,869	6,056
1553252_a_at	8,638	9,255	8,953	6,591	6,782	6,426
1553253_at	8,397	8,057	8,213	8,795	8,787	8,446
1553254_at	5,092	5,317	5,637	5,988	5,772	5,537
1553255_at	4,926	4,777	4,906	5,268	5,113	5,124
1553257_at	7,339	7,249	7,331	7,211	6,966	7,076
1553258_at	6,762	6,692	6,685	7,321	6,716	7,109
1553260_s_at	4,121	3,914	3,905	4,300	4,438	4,180
1553261_x_at	4,174	4,115	4,405	4,500	4,539	4,430
1553262_a_at	7,976	7,715	7,879	8,002	7,688	7,958
1553263_at	8,357	8,418	8,335	8,302	8,276	8,237
1553264_a_at	7,675	6,991	7,353	8,345	8,020	8,035
1553265_at	5,742	5,808	6,048	6,467	6,433	6,780
1553266_at	3,647	3,602	3,505	4,029	3,836	3,916

1553267_a_at	5,026	5,215	4,763	5,095	4,528	4,818
1553268_at	7,526	7,904	7,887	7,515	7,117	7,174
1553269_at	6,768	6,097	6,210	4,532	5,185	4,602
1553271_at	4,229	4,268	4,960	3,721	3,921	3,778
1553272_at	7,218	7,054	7,380	7,867	8,154	7,959
1553274_a_at	8,538	8,869	7,981	6,956	7,671	7,538
1553275_s_at	5,072	4,865	5,374	4,466	5,334	4,996
1553276_at	7,784	7,632	7,456	4,076	4,061	4,102
1553277_at	3,857	3,731	3,721	3,944	3,657	3,736
1553279_at	8,142	8,089	8,088	8,582	8,402	8,382
1553280_at	6,138	6,419	6,400	6,920	6,776	6,782
1553281_at	6,755	6,670	7,002	7,270	7,019	7,154
1553282_at	6,644	6,257	6,344	6,963	6,858	6,973
1553284_s_at	6,028	5,461	6,019	5,776	5,489	5,903
1553285_s_at	4,470	4,535	4,528	4,421	4,390	4,448
1553286_at	8,129	8,677	7,354	6,414	6,730	6,419
1553288_a_at	6,695	6,628	7,076	6,781	6,768	6,867
1553292_s_at	8,985	8,651	8,661	5,282	5,747	5,616
1553293_at	5,240	5,121	5,002	5,388	5,303	5,571
1553295_at	3,741	3,873	4,290	4,224	4,063	4,114
1553296_at	4,802	4,833	4,626	4,908	4,769	4,846
1553297_a_at	7,261	7,271	7,391	7,523	7,400	7,514
1553298_at	3,923	3,647	4,008	4,059	3,975	4,141
1553299_at	5,902	6,179	5,970	4,235	3,937	3,934
1553300_a_at	6,023	5,542	5,801	6,099	6,498	6,501
1553301_a_at	4,776	4,977	5,140	4,696	4,788	4,789
1553303_at	7,218	7,265	7,184	7,833	7,269	7,469
1553304_at	6,143	6,399	6,230	6,834	6,485	6,769
1553306_at	5,598	5,088	5,053	5,545	5,702	5,599
1553310_at	4,935	4,468	4,261	6,167	6,281	6,509
1553311_at	4,313	4,607	4,223	5,065	6,428	4,849
1553313_s_at	6,254	6,461	6,077	7,187	7,623	6,881
1553314_a_at	5,919	5,950	5,628	6,326	6,284	6,444
1553315_at	4,694	4,267	4,402	4,327	4,215	4,256
1553316_at	5,717	5,665	5,639	5,928	5,830	5,875
1553317_s_at	3,737	3,788	3,677	3,675	3,694	3,768
1553318_at	7,149	7,213	6,845	7,715	7,708	7,789
1553319_at	5,050	5,077	5,410	5,286	5,075	5,209
1553320_s_at	3,452	3,826	3,667	3,460	3,607	3,478
1553321_a_at	4,382	4,343	4,675	4,844	4,553	4,843
1553322_s_at	4,639	4,976	4,687	5,929	5,514	5,840
1553323_a_at	4,787	4,773	4,804	4,849	4,420	4,875
1553325_at	6,996	7,249	6,857	7,010	6,654	6,633
1553326_at	4,884	4,636	4,911	5,200	5,131	5,372
1553327_a_at	6,325	6,522	6,524	7,409	7,269	7,299
1553328_a_at	5,664	5,514	5,885	5,951	5,884	6,145
1553329_at	4,163	4,318	4,185	5,040	4,716	4,715
1553330_at	5,032	5,306	5,266	5,781	5,562	5,662
1553333_at	3,846	3,717	3,936	4,414	4,124	4,161
1553335_x_at	5,709	5,936	5,069	6,508	6,307	6,237
1553336_a_at	5,074	5,322	5,137	5,579	5,637	5,277
1553338_at	7,984	7,981	8,329	7,640	7,678	7,293
1553340_s_at	7,376	7,545	7,719	8,310	8,266	8,333
1553341_at	5,397	5,662	5,761	6,050	5,915	6,087
1553344_at	3,523	4,024	3,746	3,788	3,738	3,902
1553346_a_at	5,581	5,879	5,361	5,333	5,251	5,418

1553347_s_at	6,487	6,522	6,463	6,924	6,877	7,087
1553348_a_at	6,540	6,472	6,257	6,646	6,558	6,614
1553349_at	7,278	6,795	7,259	5,077	5,766	4,935
1553350_at	6,576	6,619	6,306	7,292	6,942	6,993
1553351_at	6,327	6,516	6,249	6,099	5,980	6,005
1553352_x_at	6,203	5,885	6,323	7,128	6,996	6,915
1553354_a_at	6,302	6,176	6,365	5,913	6,169	6,076
1553355_at	6,185	6,527	6,091	6,216	5,906	6,295
1553356_at	4,418	4,547	4,385	4,363	4,512	4,638
1553357_at	4,187	4,458	4,695	4,138	4,217	4,247
1553359_at	8,454	8,417	8,523	9,010	9,047	9,285
1553361_x_at	8,145	8,296	8,196	8,466	8,751	8,676
1553362_at	3,458	3,761	3,372	3,409	3,495	3,573
1553363_at	6,019	6,279	6,304	6,649	6,317	6,226
1553364_at	5,318	5,186	5,204	5,668	5,412	5,249
1553365_at	4,984	4,799	5,357	5,015	5,024	5,202
1553366_s_at	7,154	7,409	7,517	7,774	7,947	7,905
1553367_a_at	7,204	7,057	6,825	8,002	7,644	7,954
1553369_at	6,888	6,702	6,968	7,079	7,063	7,133
1553371_at	5,209	4,873	5,335	5,599	5,908	5,841
1553372_at	6,422	5,551	5,633	7,335	6,904	7,217
1553373_at	4,981	5,453	5,036	5,484	5,712	5,974
1553374_at	5,004	4,695	4,939	5,130	5,036	4,930
1553375_at	6,359	5,811	6,048	6,441	6,116	6,464
1553376_a_at	4,175	4,433	4,154	5,090	4,586	4,967
1553378_a_at	4,925	4,625	4,753	5,468	4,963	5,139
1553380_at	4,018	4,052	4,457	4,210	4,083	4,384
1553383_at	4,249	4,182	4,279	5,095	4,668	5,041
1553385_at	7,070	6,817	6,933	7,952	7,665	8,064
1553386_at	6,294	5,904	6,472	6,593	6,534	6,576
1553387_at	5,985	5,793	5,910	5,702	5,822	5,502
1553388_at	5,232	4,891	5,270	5,417	5,315	5,464
1553389_at	5,504	5,795	5,589	5,722	5,390	5,584
1553390_at	6,134	6,069	6,036	6,975	6,790	6,878
1553391_at	5,127	5,509	5,749	5,698	5,439	5,762
1553392_at	4,769	4,141	4,707	4,552	4,500	4,379
1553394_a_at	5,109	5,083	5,264	5,589	5,295	5,231
1553395_a_at	6,848	6,537	6,841	7,331	7,197	7,212
1553396_a_at	7,538	7,132	7,479	8,476	8,272	8,885
1553397_at	8,013	7,888	8,025	8,643	8,736	8,703
1553398_at	5,316	4,819	4,865	5,421	5,133	4,908
1553399_a_at	7,590	7,340	7,584	8,768	8,901	9,111
1553400_a_at	6,567	7,126	6,426	5,621	5,757	6,021
1553401_at	3,579	3,761	3,805	4,393	4,148	4,479
1553402_a_at	4,736	4,876	4,600	5,919	5,606	5,509
1553403_at	6,741	6,615	6,476	6,939	6,852	6,932
1553405_a_at	5,511	5,207	5,217	6,673	6,586	7,073
1553407_at	6,304	5,742	6,605	5,594	6,173	5,894
1553409_at	4,457	4,376	4,401	4,729	4,812	4,822
1553410_a_at	6,066	6,083	5,891	6,225	6,083	6,215
1553411_s_at	8,321	7,827	8,341	6,301	6,086	6,193
1553412_at	6,305	6,725	6,851	7,278	7,479	7,651
1553413_at	6,201	5,891	6,073	6,471	5,846	6,255
1553415_at	3,727	3,586	3,528	3,579	3,437	3,392
1553417_at	5,671	6,174	6,143	6,109	5,933	6,120
1553418_a_at	5,956	6,293	6,175	6,685	6,356	6,659

1553420_at	4,584	4,832	4,863	4,912	4,965	5,243
1553422_s_at	7,418	7,625	7,133	6,989	6,787	6,938
1553423_a_at	6,065	5,948	4,964	4,937	4,896	5,131
1553424_at	5,268	4,880	4,799	6,017	5,484	5,686
1553425_at	6,810	6,668	6,571	6,760	6,680	6,736
1553426_at	5,078	4,533	5,266	5,134	5,023	5,193
1553427_at	6,650	6,949	6,640	7,277	7,130	7,415
1553428_at	4,728	4,421	5,039	5,070	5,295	4,950
1553429_at	5,865	6,034	6,426	6,955	6,927	7,127
1553430_a_at	6,488	6,382	6,436	6,766	6,469	6,614
1553432_s_at	6,209	6,068	6,106	6,675	6,487	6,329
1553433_at	4,012	3,829	3,810	4,031	4,407	4,916
1553434_at	4,575	4,333	4,623	5,167	4,713	5,088
1553435_at	6,584	6,335	6,410	6,693	6,322	6,426
1553436_at	5,888	5,766	5,963	5,848	5,916	5,996
1553437_at	6,349	6,161	6,156	7,496	6,666	6,820
1553438_at	8,073	8,075	8,083	8,350	8,401	8,468
1553439_at	6,345	6,460	6,465	6,922	6,729	6,802
1553440_at	4,092	4,196	3,974	4,355	4,179	4,327
1553441_at	3,556	3,519	3,748	3,380	3,507	3,516
1553442_a_at	5,808	5,515	5,520	7,006	6,692	6,748
1553443_at	5,763	5,331	5,597	6,233	6,281	6,300
1553444_a_at	7,726	7,603	7,674	7,867	7,887	8,036
1553446_at	4,433	4,708	4,767	4,569	4,592	4,493
1553447_at	6,233	6,181	6,375	6,715	6,610	6,884
1553448_at	4,812	4,732	4,801	5,130	4,892	5,083
1553449_at	5,681	5,757	5,801	7,133	7,030	7,227
1553450_s_at	5,014	4,887	4,879	5,501	5,285	5,586
1553451_at	5,844	5,923	5,949	6,397	6,105	6,046
1553452_at	4,895	4,706	4,653	4,909	4,760	4,867
1553453_at	7,222	7,058	7,271	7,854	7,134	7,430
1553454_at	4,969	4,878	4,846	5,363	5,053	5,084
1553456_at	5,781	6,010	6,033	5,992	5,886	6,087
1553457_at	7,558	7,427	7,442	7,647	7,548	7,709
1553458_at	6,562	6,615	6,536	7,406	7,063	7,514
1553459_at	6,840	7,219	6,838	6,525	6,485	6,624
1553460_at	6,018	6,526	6,584	6,765	6,245	6,779
1553461_at	5,594	5,568	5,417	6,039	5,703	5,812
1553462_at	3,627	3,687	4,088	4,250	4,681	4,696
1553464_at	8,330	8,191	8,332	8,435	8,448	8,375
1553465_a_at	5,480	5,626	5,403	5,983	5,812	5,918
1553466_at	6,855	6,708	6,756	7,046	7,087	7,148
1553467_at	5,302	5,325	5,202	5,465	5,318	5,524
1553468_at	4,425	4,087	4,984	4,536	4,339	4,412
1553470_at	5,078	5,428	5,258	5,949	5,817	6,166
1553471_at	5,363	5,479	5,586	6,060	6,068	6,445
1553472_at	5,384	5,501	5,418	5,707	5,560	5,746
1553474_at	3,629	3,613	3,673	3,869	3,812	3,980
1553475_at	4,831	4,855	4,755	5,132	5,491	5,185
1553478_at	5,342	5,490	5,359	5,867	5,831	6,234
1553479_at	6,161	6,343	6,301	6,095	6,082	6,313
1553482_at	5,327	5,592	5,518	5,744	5,452	5,480
1553483_at	7,702	7,733	7,856	7,692	7,662	7,872
1553484_at	4,558	4,508	4,453	4,644	4,829	4,778
1553485_at	7,010	6,945	6,796	7,439	6,821	6,883
1553486_a_at	4,626	5,177	4,655	5,183	5,044	5,325

1553488_at	6,289	6,352	6,158	6,579	6,849	7,135
1553489_a_at	5,905	5,332	5,663	6,000	6,226	6,372
1553491_at	6,085	5,819	6,068	6,349	6,238	6,434
1553492_a_at	6,924	6,771	6,596	6,978	6,763	7,101
1553493_a_at	6,451	6,745	6,351	6,842	6,698	7,051
1553494_at	4,734	5,712	4,555	3,980	3,862	3,973
1553497_at	4,306	4,250	4,418	4,343	4,213	4,337
1553498_at	6,975	6,487	6,534	7,018	6,764	6,734
1553499_s_at	6,894	6,444	6,776	8,008	7,729	7,705
1553500_at	5,979	5,690	5,835	5,647	5,488	5,588
1553502_a_at	5,282	4,876	4,793	6,053	6,242	6,223
1553504_at	5,867	5,527	5,599	5,941	5,852	6,054
1553505_at	6,325	6,478	6,168	6,557	6,290	6,819
1553506_at	6,763	6,963	6,567	6,932	6,930	6,818
1553507_a_at	6,991	7,064	6,795	6,973	6,899	7,099
1553508_at	6,408	5,789	6,673	6,729	6,879	7,074
1553510_s_at	9,059	9,119	9,105	9,690	8,701	9,066
1553511_at	7,169	6,978	7,252	7,698	6,970	7,166
1553512_at	7,097	6,883	6,948	7,109	6,938	6,849
1553513_at	4,666	4,685	4,729	4,866	4,578	4,912
1553514_a_at	5,388	5,299	5,304	6,266	6,388	5,971
1553515_at	5,252	4,964	5,458	5,850	6,177	6,361
1553517_at	6,855	6,797	6,840	7,681	7,404	7,661
1553518_at	7,006	6,642	7,081	7,509	7,059	7,310
1553519_at	4,002	4,006	3,968	4,651	4,931	5,185
1553521_at	5,819	5,784	5,750	6,116	6,055	6,204
1553523_at	6,408	6,053	6,057	7,189	7,261	7,324
1553524_at	4,993	4,773	5,441	6,151	5,996	6,127
1553525_at	7,464	7,715	7,636	8,229	8,368	8,235
1553526_at	6,063	6,002	6,250	6,932	6,903	6,754
1553527_at	6,772	6,589	6,756	7,261	7,080	7,316
1553528_a_at	10,348	10,470	9,862	7,183	7,005	6,451
1553529_at	5,462	5,719	6,115	6,134	6,073	6,017
1553530_a_at	9,540	9,619	8,927	9,789	10,608	10,588
1553531_at	6,347	6,259	6,033	6,035	6,240	6,225
1553532_a_at	7,168	6,605	7,165	7,425	7,224	7,342
1553533_at	5,885	5,923	5,819	5,969	5,494	5,775
1553534_at	6,042	5,890	5,789	6,260	6,105	6,362
1553535_a_at	8,252	8,167	8,148	8,882	7,949	8,600
1553536_at	5,273	5,547	5,342	5,749	5,674	5,902
1553537_at	5,989	5,686	6,042	6,333	6,111	6,426
1553538_s_at	14,595	14,493	14,682	13,794	14,241	13,983
1553539_at	6,718	6,197	6,544	7,691	7,542	7,864
1553540_a_at	7,237	7,105	7,208	7,601	7,365	7,329
1553541_at	5,606	5,532	5,518	6,399	6,046	5,942
1553542_at	6,157	5,920	5,391	7,154	6,850	6,908
1553544_at	6,047	6,375	6,699	7,323	6,964	7,445
1553545_at	5,716	5,876	6,039	6,439	6,495	6,676
1553546_at	3,527	3,985	3,667	3,945	3,662	3,938
1553547_at	4,918	4,833	4,430	4,901	5,299	4,847
1553549_at	6,443	6,750	6,653	6,624	6,526	6,266
1553550_at	5,564	5,915	5,924	6,780	6,797	6,561
1553551_s_at	13,375	12,894	14,053	12,746	13,032	12,984
1553552_at	6,327	6,381	6,520	7,056	6,638	6,883
1553553_at	5,121	5,381	5,366	5,679	5,750	5,923
1553555_at	6,003	5,683	6,232	6,849	6,557	6,759

1553556_at	4,451	4,757	4,154	4,382	4,494	4,567
1553558_at	5,554	5,776	5,780	6,304	6,016	6,110
1553559_at	5,786	5,720	5,764	7,235	6,827	7,265
1553560_at	5,645	5,381	5,658	6,302	6,492	6,346
1553561_at	4,993	4,827	5,019	5,498	5,406	5,473
1553562_at	6,096	6,356	6,424	7,163	7,119	7,595
1553564_at	5,777	5,739	6,158	5,525	5,598	5,626
1553565_s_at	6,432	6,130	6,400	6,827	6,900	7,107
1553566_at	7,638	7,361	7,655	7,860	7,919	8,038
1553567_s_at	14,256	13,806	14,313	13,399	13,881	13,740
1553568_a_at	6,430	6,547	6,388	6,269	6,599	6,699
1553569_at	14,444	14,178	14,448	13,192	13,663	13,658
1553570_x_at	14,463	14,187	14,448	13,207	13,676	13,692
1553572_a_at	7,565	7,625	7,641	8,088	8,231	8,228
1553573_s_at	6,077	6,028	6,139	6,576	6,489	6,692
1553574_at	5,157	5,115	5,209	5,346	5,665	5,354
1553575_at	10,429	9,953	9,995	8,674	10,652	9,462
1553579_a_at	4,324	4,503	4,236	4,810	5,154	4,861
1553581_s_at	9,259	9,655	9,264	10,260	10,336	10,277
1553582_a_at	6,267	6,350	5,977	6,789	6,672	6,553
1553583_a_at	7,632	7,324	7,334	8,070	8,255	8,420
1553584_at	6,950	6,750	7,038	7,502	7,382	7,553
1553585_a_at	5,072	5,257	5,072	5,467	5,837	5,801
1553586_at	6,649	6,300	6,663	7,297	7,260	7,341
1553587_a_at	8,760	8,682	8,792	9,308	8,644	9,366
1553588_at	14,476	14,248	14,459	13,731	13,536	14,117
1553589_a_at	6,817	6,171	6,771	7,016	6,676	6,853
1553590_at	7,142	7,002	7,096	8,188	7,855	8,158
1553591_at	7,997	7,927	8,049	8,533	8,122	8,506
1553592_x_at	8,060	7,662	8,001	8,261	8,161	8,300
1553593_a_at	6,524	6,343	6,389	7,242	6,799	6,860
1553594_a_at	6,861	6,913	7,138	7,187	6,872	7,315
1553599_a_at	3,730	3,909	3,913	4,044	3,891	3,963
1553600_at	6,232	6,450	6,570	7,844	7,757	7,840
1553601_a_at	5,649	5,697	5,458	6,292	5,903	6,460
1553602_at	6,188	6,420	6,739	6,480	6,416	6,574
1553603_s_at	7,744	7,989	7,515	6,345	6,420	6,297
1553604_at	3,305	3,269	3,476	3,305	3,281	3,359
1553605_a_at	3,676	3,807	3,746	3,561	3,596	3,527
1553607_at	3,824	4,218	4,335	3,851	3,833	3,821
1553608_a_at	6,279	6,483	6,076	5,928	6,025	5,862
1553611_s_at	6,721	6,514	6,466	6,772	7,069	6,998
1553612_at	6,509	6,904	6,332	6,418	6,404	6,366
1553613_s_at	5,598	6,758	6,099	9,100	8,624	8,860
1553614_a_at	5,765	6,014	4,960	5,498	5,302	5,534
1553618_at	5,110	5,183	5,576	6,276	6,317	6,400
1553619_a_at	5,944	6,017	6,314	6,681	6,738	6,444
1553620_at	4,209	4,189	4,133	4,633	4,442	4,641
1553622_a_at	4,776	4,698	4,953	5,242	5,340	5,478
1553623_at	5,655	5,739	5,614	5,764	5,611	5,884
1553625_at	3,347	3,443	3,510	3,369	3,243	3,383
1553626_a_at	7,059	7,042	6,471	7,251	6,246	6,276
1553627_s_at	4,014	4,183	3,838	3,888	3,791	3,863
1553629_a_at	6,897	6,692	6,890	8,097	7,966	7,980
1553630_at	3,814	3,703	3,701	4,260	4,029	4,331
1553633_s_at	6,302	7,058	6,291	6,334	6,266	6,205

1553634_a_at	6,155	5,788	5,837	5,955	5,438	5,765
1553635_s_at	6,909	7,402	6,305	6,293	6,691	6,075
1553636_at	3,645	3,619	3,687	4,554	4,612	4,562
1553637_s_at	6,547	6,732	6,959	6,650	6,650	6,557
1553639_a_at	7,760	7,938	8,330	8,510	7,683	7,935
1553640_at	7,088	7,337	7,447	6,175	6,081	6,406
1553641_a_at	6,161	6,065	6,206	7,140	6,834	6,841
1553642_at	7,964	7,547	8,080	8,241	8,483	8,578
1553644_at	6,274	6,008	5,823	7,028	6,762	6,823
1553645_at	4,887	6,120	6,294	4,344	4,203	4,270
1553646_at	6,681	6,628	6,534	4,826	5,040	4,566
1553647_at	7,027	7,319	7,429	8,494	8,108	8,304
1553651_at	4,045	4,010	3,981	3,990	4,128	4,302
1553652_a_at	5,696	5,745	4,958	5,487	5,326	5,182
1553654_at	4,951	5,507	5,215	4,231	4,030	4,042
1553655_at	5,478	5,661	5,813	6,188	5,853	6,307
1553656_at	7,790	8,022	8,137	8,335	7,912	8,027
1553657_at	8,678	8,200	8,522	9,918	9,303	9,365
1553658_at	7,304	7,200	7,176	7,619	7,374	7,542
1553660_at	6,831	7,030	6,877	8,324	8,221	8,539
1553661_a_at	5,786	5,978	6,113	5,596	5,187	5,378
1553663_a_at	8,037	7,852	8,059	8,241	7,760	8,080
1553666_at	5,295	5,091	5,426	5,292	5,244	5,394
1553668_at	6,980	6,850	6,755	7,681	7,350	7,698
1553670_at	5,873	5,752	5,458	5,617	5,586	5,343
1553672_at	6,207	6,655	6,396	7,766	8,296	7,909
1553673_at	6,082	6,295	5,502	6,461	6,310	6,537
1553674_at	6,282	6,492	6,393	6,221	5,462	5,518
1553675_at	5,267	5,661	5,859	5,821	5,609	5,705
1553677_a_at	9,459	9,752	9,313	9,162	8,865	8,922
1553678_a_at	10,114	10,005	9,305	10,190	11,500	10,936
1553679_s_at	7,733	7,525	7,156	6,828	6,673	6,671
1553681_a_at	7,567	7,529	7,667	8,345	8,005	8,084
1553682_at	5,935	6,895	6,199	5,690	5,115	5,120
1553683_s_at	6,457	6,959	6,770	6,114	6,283	6,110
1553684_at	6,106	7,218	6,132	6,515	6,635	6,935
1553685_s_at	5,038	4,700	4,956	4,800	4,985	5,124
1553686_at	6,504	6,066	6,157	6,833	6,655	6,736
1553688_at	6,905	6,772	7,104	7,613	7,630	7,614
1553689_s_at	9,217	9,085	9,084	8,571	8,450	8,468
1553690_at	7,303	6,728	7,328	6,801	6,758	6,310
1553691_at	5,665	5,632	5,518	5,784	5,630	5,982
1553693_s_at	8,171	8,152	7,104	5,988	6,527	6,059
1553694_a_at	6,125	6,322	5,801	5,332	5,411	5,351
1553695_a_at	7,401	7,425	7,327	7,845	7,713	8,020
1553696_s_at	8,846	9,006	8,446	6,410	7,003	6,296
1553697_at	4,437	4,537	4,147	4,322	3,922	3,953
1553698_a_at	4,054	4,209	4,163	4,006	3,842	3,894
1553701_a_at	6,849	6,850	6,506	7,235	7,197	7,407
1553702_at	6,858	6,769	6,771	7,220	7,372	7,393
1553703_at	9,816	9,931	9,684	7,024	7,747	7,083
1553704_x_at	9,567	9,685	9,365	6,913	7,532	6,788
1553705_a_at	7,103	7,568	7,328	6,447	6,258	6,257
1553706_at	5,149	5,361	5,093	4,992	5,041	5,137
1553708_at	4,787	4,944	4,926	6,100	6,172	6,223
1553709_a_at	10,531	10,335	10,402	9,009	8,200	8,611

1553710_at	6,372	5,664	5,716	5,147	4,966	5,184
1553711_a_at	6,014	5,555	5,771	6,408	5,766	5,801
1553713_a_at	8,659	9,564	9,213	8,044	7,765	7,886
1553715_s_at	9,948	9,909	9,939	9,829	8,932	9,698
1553717_at	7,400	7,339	7,217	7,318	7,375	7,497
1553718_at	9,875	9,853	9,805	11,035	10,109	10,017
1553719_s_at	8,350	8,674	8,268	6,612	6,598	6,480
1553720_a_at	7,322	7,336	7,584	7,717	7,496	7,464
1553721_at	5,169	5,181	5,294	5,167	5,233	5,366
1553722_s_at	4,875	4,815	4,925	4,693	4,821	4,745
1553723_at	5,855	5,902	6,211	6,194	6,422	6,418
1553725_s_at	7,034	7,034	6,839	4,730	5,087	4,838
1553726_s_at	4,100	5,128	4,081	4,756	5,455	4,973
1553727_at	7,111	6,809	7,091	7,324	7,139	7,424
1553728_at	9,221	9,241	9,301	9,528	9,202	9,503
1553729_s_at	5,839	5,742	5,715	6,013	5,775	6,140
1553730_x_at	8,050	7,896	8,093	8,694	8,604	8,825
1553732_s_at	8,005	7,998	7,858	7,823	7,997	8,091
1553734_at	6,825	7,442	6,413	6,281	6,008	6,127
1553736_at	5,120	5,708	5,555	4,854	5,553	5,211
1553739_at	4,290	3,973	4,235	5,382	4,614	4,540
1553740_a_at	6,762	6,472	6,375	7,695	7,757	7,882
1553741_at	6,576	6,375	6,861	6,652	6,550	6,372
1553743_at	7,393	9,037	7,501	6,443	6,152	6,566
1553746_a_at	4,916	5,532	4,677	4,763	4,952	5,577
1553747_at	4,503	4,313	4,445	5,148	5,018	4,849
1553749_at	6,652	6,923	6,680	5,964	6,003	5,779
1553750_a_at	5,292	5,925	5,706	4,994	5,223	5,303
1553752_at	6,799	6,082	6,391	7,915	7,814	8,117
1553755_at	8,339	8,069	8,147	8,204	7,928	7,890
1553756_at	6,938	7,038	6,825	7,274	7,107	7,252
1553757_at	6,275	5,939	6,173	6,396	6,258	6,319
1553759_at	5,478	4,895	4,978	4,417	4,453	4,444
1553761_at	6,349	5,987	6,215	6,508	6,485	6,597
1553764_a_at	6,804	7,063	6,780	9,119	8,652	9,597
1553765_a_at	3,666	4,092	3,934	3,689	3,657	3,838
1553768_a_at	7,614	8,216	8,465	10,275	9,741	10,993
1553769_at	5,801	5,544	5,880	6,449	6,012	6,286
1553770_a_at	7,346	7,325	7,240	8,202	7,714	8,005
1553772_at	5,521	6,140	5,480	5,497	5,751	5,624
1553775_at	6,606	6,508	7,084	6,462	6,278	6,890
1553776_at	3,726	3,877	3,800	3,844	3,983	4,025
1553778_at	7,706	7,621	7,536	8,534	7,863	8,091
1553780_at	7,847	8,008	8,111	8,405	8,047	8,301
1553781_at	3,474	3,646	3,839	3,812	3,554	3,729
1553785_at	4,374	4,310	4,505	4,732	4,601	4,515
1553787_at	6,512	6,068	6,002	5,899	6,025	5,925
1553789_a_at	7,348	6,670	7,005	6,164	5,718	5,961
1553792_at	3,665	3,677	3,743	3,676	3,800	3,611
1553793_a_at	4,590	4,569	4,554	5,392	5,598	5,620
1553794_at	5,328	5,506	5,273	5,517	5,480	5,511
1553796_at	5,398	5,592	5,324	6,156	5,819	5,949
1553797_a_at	4,389	4,262	4,416	4,472	4,312	4,341
1553798_a_at	5,598	5,631	5,729	5,064	5,409	5,063
1553799_at	4,388	4,645	4,420	4,184	4,305	4,678
1553801_a_at	7,149	7,034	7,142	7,970	7,346	7,530

1553802_a_at	6,729	6,065	6,228	6,612	6,378	6,741
1553803_at	9,122	9,168	9,223	9,660	9,492	9,892
1553804_a_at	6,339	5,649	6,097	6,910	6,847	7,143
1553805_at	6,860	6,786	7,001	7,666	7,274	7,114
1553808_a_at	5,056	5,020	4,826	5,263	5,296	5,143
1553809_a_at	6,255	6,343	6,188	7,138	7,040	6,942
1553810_a_at	8,121	7,819	7,385	5,919	6,100	5,156
1553811_at	5,540	4,995	5,288	6,142	5,887	6,138
1553812_at	5,971	5,963	6,156	7,366	7,347	7,923
1553813_s_at	5,667	5,458	5,610	5,910	5,356	5,854
1553815_a_at	7,162	7,187	7,433	6,399	5,997	5,740
1553817_at	4,749	5,132	4,782	5,133	5,029	5,413
1553818_x_at	6,049	5,842	5,920	6,128	6,158	6,340
1553821_at	5,432	5,787	5,283	5,706	5,441	5,595
1553822_at	4,127	4,603	4,592	4,092	3,828	4,004
1553823_a_at	5,699	5,787	5,823	6,406	5,925	6,387
1553826_a_at	8,469	8,330	8,652	8,814	8,530	8,829
1553828_at	5,840	6,045	5,636	6,297	6,072	6,315
1553829_at	5,557	5,524	5,445	5,696	5,602	5,949
1553830_s_at	4,858	5,426	4,555	5,133	5,101	5,134
1553831_at	7,324	7,257	7,599	8,222	7,899	8,011
1553832_at	7,065	6,973	7,211	7,072	7,040	7,281
1553834_at	4,421	4,201	4,226	4,691	4,473	4,443
1553835_a_at	4,876	5,023	5,244	5,148	4,938	5,108
1553837_at	6,493	6,796	6,525	7,338	6,949	7,137
1553838_at	5,965	5,819	5,859	6,301	6,139	6,143
1553839_at	7,439	7,048	7,611	7,975	7,873	7,832
1553840_a_at	7,870	7,551	7,486	8,407	8,037	8,181
1553842_at	5,375	5,569	5,556	6,256	6,041	5,867
1553843_at	8,428	8,441	8,593	8,692	8,419	8,683
1553844_a_at	6,014	6,433	6,366	6,531	6,508	6,735
1553845_x_at	8,411	8,159	8,554	8,683	8,538	8,687
1553846_at	7,006	6,834	6,774	7,601	7,436	7,448
1553847_a_at	7,002	6,750	6,523	7,517	7,276	7,671
1553849_at	3,814	3,976	3,936	4,077	3,988	3,930
1553850_at	5,718	5,435	5,858	5,975	5,658	5,402
1553851_at	3,954	3,864	3,899	3,929	3,843	4,002
1553852_at	5,099	4,714	4,978	5,077	5,111	5,210
1553856_s_at	5,901	5,827	5,597	6,067	6,013	6,279
1553857_at	7,233	7,343	7,334	8,186	7,954	8,172
1553858_at	8,409	8,576	8,765	8,198	7,860	8,271
1553859_at	4,968	4,735	4,941	5,592	5,201	5,798
1553860_at	6,699	6,472	6,651	6,535	6,606	6,728
1553861_at	5,041	5,651	5,259	5,659	5,745	5,627
1553863_at	5,556	5,772	5,980	6,228	6,430	6,324
1553864_at	5,466	5,381	5,635	6,959	7,208	7,279
1553865_a_at	6,951	6,470	6,932	6,872	6,921	6,950
1553868_a_at	3,558	3,819	3,837	3,724	3,728	3,609
1553869_at	4,196	4,226	4,288	4,045	4,086	4,361
1553871_at	7,444	6,993	7,244	7,388	7,196	7,344
1553872_at	7,352	6,993	7,086	7,920	8,073	8,117
1553873_at	4,696	3,939	4,131	4,398	4,505	4,652
1553874_a_at	9,204	8,865	9,609	6,056	5,877	6,380
1553875_s_at	8,788	8,244	8,715	6,298	5,962	6,203
1553876_at	5,089	5,276	5,145	5,473	5,463	5,664
1553878_at	5,741	5,959	6,590	7,097	7,303	7,193

1553879_a_at	6,028	6,280	6,397	6,868	7,042	7,077
1553880_at	7,370	6,587	6,624	8,033	7,802	8,278
1553881_at	5,049	4,855	5,049	5,095	5,102	4,981
1553882_at	5,195	5,194	5,291	5,923	5,772	6,113
1553883_at	6,969	7,365	6,140	3,463	3,500	3,478
1553885_x_at	6,708	7,167	6,521	3,461	3,632	3,530
1553886_at	3,576	4,012	3,965	4,188	4,134	4,241
1553887_at	6,021	5,944	5,805	5,948	5,949	5,969
1553888_at	7,042	7,231	7,049	7,371	7,105	7,497
1553889_at	6,711	6,907	7,073	7,379	7,108	7,379
1553890_s_at	7,096	7,054	7,321	7,565	7,713	7,843
1553891_at	7,021	6,943	7,023	7,299	7,320	7,465
1553893_at	7,285	6,936	7,388	7,514	7,543	7,754
1553894_at	4,273	4,329	4,230	3,889	4,021	3,757
1553895_at	6,052	6,234	5,931	7,176	7,026	7,186
1553898_a_at	4,696	4,828	4,609	5,483	5,558	5,936
1553900_s_at	6,929	6,880	7,049	7,628	7,370	7,721
1553901_x_at	5,200	5,068	5,323	5,792	5,910	5,919
1553905_at	5,568	5,716	5,591	5,678	5,708	5,699
1553906_s_at	9,442	9,333	9,440	10,042	9,705	10,243
1553907_a_at	4,749	4,844	4,514	4,662	4,469	4,445
1553909_x_at	7,249	6,901	7,235	6,662	7,059	6,976
1553910_at	5,408	5,124	5,214	5,686	5,557	6,045
1553911_at	5,933	5,443	5,645	6,540	6,213	6,460
1553912_at	3,941	4,622	4,251	4,487	4,426	4,492
1553914_at	5,870	6,178	5,867	6,767	6,210	6,548
1553915_at	3,734	3,687	4,038	4,133	3,812	4,260
1553917_at	6,714	6,715	6,865	6,910	7,046	6,935
1553918_at	7,106	6,731	7,188	7,324	6,988	7,208
1553919_at	7,169	6,662	6,909	7,715	7,407	7,484
1553920_at	4,108	4,160	4,121	4,226	4,043	4,008
1553923_at	5,833	6,118	6,327	6,970	7,011	6,985
1553924_at	4,701	4,435	5,135	5,333	5,042	5,281
1553927_at	6,296	6,349	6,432	7,283	7,466	7,622
1553928_at	6,428	7,211	7,111	7,878	7,792	8,172
1553929_at	6,688	6,593	6,817	8,047	7,299	7,818
1553930_at	3,579	3,690	3,662	3,766	3,699	3,733
1553931_at	4,261	4,252	4,168	4,748	4,539	4,952
1553934_at	4,777	4,202	4,845	5,424	5,144	5,189
1553935_at	4,085	4,320	3,997	4,517	4,605	4,559
1553936_a_at	6,726	6,636	6,743	7,055	6,770	6,811
1553938_a_at	5,668	5,816	6,222	6,402	6,239	6,563
1553940_a_at	5,900	6,367	6,181	6,366	5,920	6,509
1553943_at	6,951	6,746	6,934	7,597	7,581	7,681
1553944_at	4,978	4,762	4,808	5,362	5,238	5,726
1553945_at	7,055	6,371	7,014	7,361	7,022	7,522
1553946_at	6,837	6,909	6,979	7,302	6,735	7,038
1553947_at	7,347	6,701	6,954	7,352	6,908	7,527
1553949_at	6,418	6,089	6,277	6,858	6,673	6,544
1553952_at	8,511	8,277	8,584	8,700	8,297	8,801
1553954_at	7,945	7,849	8,145	9,458	8,327	9,348
1553955_at	9,774	10,148	10,168	8,066	9,187	8,406
1553956_at	10,284	10,325	10,138	9,224	9,417	9,771
1553957_at	8,421	8,345	7,928	7,000	6,862	6,724
1553959_a_at	10,697	10,787	11,351	11,305	10,784	11,459
1553960_at	7,855	8,091	8,121	7,844	8,268	8,320

1553961_s_at	8,181	8,257	8,665	8,857	8,626	8,710
1553962_s_at	5,768	5,705	5,735	6,634	6,216	6,536
1553963_at	3,453	3,501	3,314	3,589	3,425	3,703
1553965_x_at	4,336	4,333	4,476	4,573	4,293	4,412
1553967_at	6,164	5,436	6,127	6,552	6,387	6,728
1553968_a_at	9,055	8,896	9,161	9,079	8,464	8,884
1553970_s_at	7,211	6,680	6,855	5,082	4,718	4,970
1553971_a_at	7,383	7,030	7,361	6,968	7,009	6,674
1553972_a_at	11,917	11,767	11,598	9,078	8,928	9,467
1553973_a_at	6,097	5,876	6,097	6,296	6,404	6,631
1553974_at	5,880	6,340	5,916	5,115	4,986	5,345
1553976_a_at	8,678	8,485	9,088	9,719	9,555	9,927
1553977_a_at	6,452	6,522	6,603	7,164	7,130	7,044
1553978_at	7,922	7,554	8,533	9,881	9,120	9,773
1553979_at	12,182	11,890	11,921	8,736	9,286	8,106
1553982_a_at	7,362	7,218	7,268	8,877	8,245	8,362
1553983_at	8,988	8,637	8,837	9,184	9,093	8,982
1553984_s_at	11,051	11,031	11,496	11,538	10,891	11,277
1553986_at	7,087	7,108	6,771	6,444	6,660	6,278
1553987_at	8,915	9,211	8,679	8,328	7,616	8,141
1553989_a_at	7,390	8,039	7,817	6,966	6,705	7,330
1553990_at	7,961	7,416	7,305	7,141	7,174	7,363
1553991_s_at	7,338	7,092	8,084	6,913	6,391	6,564
1553992_s_at	8,208	8,477	8,694	7,433	7,730	7,277
1553993_s_at	8,709	8,778	9,251	8,182	7,964	8,213
1553994_at	4,816	4,717	5,026	12,079	12,010	12,120
1553995_a_at	5,292	5,086	5,209	10,215	9,844	10,022
1553997_a_at	8,853	8,713	8,750	8,555	8,260	8,694
1553998_at	5,945	6,369	5,719	5,760	5,232	5,359
1554001_at	7,351	7,929	7,428	7,412	7,836	7,087
1554003_at	7,389	7,249	7,261	8,920	8,997	8,524
1554004_a_at	5,540	5,905	5,638	6,494	7,860	6,467
1554006_a_at	7,100	7,370	7,201	7,528	7,409	7,628
1554007_at	13,119	13,172	12,964	5,306	5,147	4,990
1554008_at	6,506	6,192	5,907	8,314	8,396	7,909
1554010_at	7,507	7,592	8,065	9,354	8,839	8,811
1554012_at	4,345	4,469	3,927	4,333	4,273	4,351
1554014_at	7,956	8,427	7,818	7,334	8,897	7,363
1554015_a_at	7,322	7,585	7,159	7,110	7,763	7,166
1554016_a_at	9,104	9,023	9,630	9,145	8,157	8,764
1554017_at	7,396	7,137	7,348	7,991	7,708	7,806
1554018_at	3,854	3,695	3,479	7,946	7,363	6,588
1554019_s_at	6,376	6,518	5,633	5,440	5,687	5,288
1554020_at	10,713	11,648	11,418	9,506	9,072	9,044
1554021_a_at	9,696	9,983	9,431	6,475	7,831	6,747
1554022_at	4,913	4,880	5,063	5,086	4,919	4,826
1554023_s_at	7,229	7,231	7,379	7,684	7,727	7,967
1554026_a_at	7,347	7,393	7,701	7,779	9,256	9,167
1554027_a_at	4,357	4,978	4,201	4,937	4,673	5,006
1554029_a_at	6,540	6,867	5,949	5,153	5,750	5,090
1554030_at	6,484	6,955	6,973	7,603	7,100	7,644
1554032_at	4,359	4,405	4,305	5,260	5,049	4,936
1554033_at	5,781	5,889	5,827	7,402	7,308	7,310
1554034_a_at	4,900	5,166	5,356	5,001	5,143	4,976
1554036_at	7,613	8,850	8,429	8,131	8,211	8,265
1554037_a_at	6,328	7,191	6,754	6,696	6,488	6,722

1554038_at	6,704	6,972	7,201	7,121	7,813	7,436
1554039_at	8,902	8,711	8,767	9,699	9,454	9,407
1554041_at	6,326	6,426	6,704	7,614	7,563	7,678
1554042_s_at	6,724	6,724	6,882	7,419	7,333	7,622
1554043_a_at	9,035	9,778	8,975	8,338	8,036	8,229
1554044_a_at	7,107	6,695	7,068	7,555	7,261	7,531
1554045_at	5,720	6,371	6,578	7,144	6,548	6,992
1554047_at	6,537	6,802	6,733	6,598	6,578	7,089
1554049_s_at	6,092	5,609	6,220	6,769	6,299	6,404
1554050_at	7,994	7,858	8,164	8,263	8,146	8,400
1554052_at	6,322	6,927	6,119	6,596	6,478	6,191
1554053_at	6,033	6,278	6,193	7,703	7,067	8,028
1554057_at	9,943	10,538	10,356	9,851	9,155	9,171
1554059_at	7,186	6,987	7,332	7,177	6,825	6,924
1554060_s_at	6,304	5,849	6,523	6,853	6,429	6,433
1554062_at	5,465	5,758	5,160	6,450	6,828	6,214
1554063_at	7,574	7,933	7,543	8,050	7,702	7,875
1554065_at	6,845	6,968	7,135	7,848	7,597	7,754
1554067_at	8,202	8,540	7,903	5,281	5,428	4,728
1554068_s_at	6,358	6,335	5,552	5,914	5,869	5,739
1554069_at	7,499	7,682	7,821	7,142	7,235	7,237
1554071_at	4,079	4,004	3,907	4,339	4,096	4,267
1554072_s_at	3,540	3,551	3,738	3,513	3,624	3,567
1554074_s_at	7,627	7,145	7,160	7,847	7,718	7,939
1554076_s_at	8,259	8,312	8,208	8,850	8,427	8,736
1554077_a_at	8,088	7,969	8,249	8,067	7,243	8,062
1554078_s_at	11,557	11,673	12,020	11,570	11,305	11,437
1554079_at	6,918	6,956	6,857	7,657	7,107	7,828
1554080_at	9,024	9,127	9,160	9,842	9,239	9,455
1554082_a_at	8,124	7,989	7,895	8,750	8,256	8,369
1554084_a_at	8,810	8,580	8,897	8,826	8,590	8,764
1554085_at	8,176	7,780	8,555	8,395	7,778	7,996
1554086_at	5,902	6,205	6,288	6,419	5,833	6,140
1554089_s_at	8,196	8,525	8,261	9,577	8,743	9,714
1554091_a_at	7,838	7,857	7,944	8,644	7,819	8,296
1554093_a_at	9,279	9,598	9,034	8,994	8,643	8,819
1554094_at	6,539	6,438	6,415	6,963	6,873	6,814
1554095_at	7,446	7,906	7,482	9,175	8,723	8,998
1554096_a_at	5,112	5,614	5,256	5,546	4,959	5,720
1554097_a_at	7,041	7,063	6,788	8,336	8,004	8,532
1554098_at	4,313	4,517	4,469	3,991	4,007	4,162
1554099_a_at	9,222	9,005	9,144	6,852	6,911	7,080
1554101_a_at	6,823	7,226	6,378	5,862	5,129	5,394
1554102_a_at	7,877	7,904	7,478	7,777	7,565	7,641
1554103_at	6,608	6,542	6,312	6,822	6,409	6,622
1554105_at	7,562	7,980	7,475	8,534	8,110	8,170
1554106_at	6,374	5,975	5,418	6,589	6,854	6,868
1554108_at	4,849	4,848	5,039	4,361	4,402	4,091
1554110_at	5,571	5,787	5,840	6,999	6,493	6,828
1554112_a_at	6,648	6,882	6,562	6,495	6,354	6,373
1554113_a_at	7,964	8,365	7,917	7,669	7,419	7,574
1554114_s_at	4,651	4,831	4,891	5,075	4,871	5,274
1554115_at	6,433	6,560	6,442	6,958	6,627	6,610
1554116_s_at	6,988	6,516	6,826	6,318	6,171	6,222
1554117_at	7,205	7,248	6,925	7,931	7,742	8,188
1554119_at	6,485	6,303	6,543	7,994	7,626	8,085

1554121_at	5,149	5,032	4,960	4,814	4,793	5,000
1554122_a_at	6,614	7,670	6,783	6,240	6,411	7,950
1554125_a_at	5,830	5,381	5,621	5,554	5,821	6,447
1554126_at	7,997	7,895	8,030	9,217	8,766	9,160
1554127_s_at	6,219	6,145	5,273	8,878	8,068	8,897
1554128_at	7,598	7,542	7,613	8,966	9,037	8,373
1554129_a_at	7,627	7,343	7,408	8,226	7,935	8,106
1554131_at	4,149	5,015	3,740	4,044	4,011	4,059
1554132_a_at	6,684	7,594	6,543	6,782	6,811	6,920
1554133_at	3,429	3,482	3,611	3,838	3,826	3,901
1554140_at	6,072	6,182	6,129	5,534	6,657	5,511
1554141_s_at	6,541	6,712	6,516	6,500	6,897	6,390
1554142_at	3,545	3,695	3,477	3,570	3,551	3,901
1554143_a_at	4,150	4,353	4,031	4,577	4,502	4,574
1554145_a_at	6,942	6,817	7,005	6,610	5,968	6,244
1554147_s_at	5,188	5,461	4,968	5,168	5,278	5,505
1554148_a_at	8,010	8,063	7,824	8,328	7,997	8,330
1554149_at	11,026	11,492	10,841	9,610	9,867	9,858
1554151_at	7,497	7,386	7,388	8,104	7,738	8,027
1554152_a_at	5,202	5,033	5,518	5,563	5,241	5,547
1554153_a_at	8,646	8,753	8,678	7,161	7,153	6,992
1554154_at	8,355	8,360	7,893	6,984	7,184	7,214
1554155_at	9,548	9,452	9,531	7,110	6,655	6,720
1554156_a_at	4,665	4,831	4,696	5,495	5,362	5,292
1554157_a_at	6,849	6,739	6,668	7,279	6,911	7,167
1554158_at	6,188	6,222	5,917	6,876	6,573	6,498
1554159_a_at	7,462	7,282	7,119	7,941	7,887	8,038
1554160_a_at	8,000	8,003	8,128	8,509	8,361	8,685
1554161_at	5,238	5,735	5,186	5,777	5,857	5,521
1554163_at	5,820	5,529	5,668	7,798	7,386	7,720
1554165_at	4,896	4,622	4,887	6,316	6,331	6,503
1554167_a_at	8,963	9,035	8,617	8,862	8,322	8,625
1554168_a_at	9,441	9,919	9,709	10,156	10,301	10,561
1554170_a_at	7,809	8,119	8,056	8,546	8,276	8,450
1554171_at	8,761	8,929	8,890	9,094	8,969	9,139
1554172_a_at	7,824	7,665	7,779	7,386	6,885	7,385
1554173_at	8,393	7,847	8,094	8,658	8,617	8,724
1554175_at	7,389	7,477	7,534	8,712	8,537	8,670
1554176_a_at	5,212	5,682	5,186	4,508	4,434	4,900
1554177_a_at	8,552	8,112	8,111	8,359	7,998	8,139
1554178_a_at	7,362	7,050	7,068	6,077	7,378	6,012
1554179_s_at	8,090	7,698	7,945	8,575	8,049	8,208
1554181_at	6,554	6,375	6,285	6,826	6,798	7,110
1554182_at	5,302	5,473	5,178	5,457	5,461	5,334
1554183_s_at	8,175	8,122	8,191	8,958	8,822	9,287
1554185_at	6,174	5,925	5,996	6,696	6,281	6,507
1554187_at	6,561	6,322	6,327	7,112	6,964	7,084
1554188_at	7,418	7,562	7,725	7,950	7,861	8,150
1554190_s_at	3,726	4,039	3,810	4,302	3,865	4,059
1554192_s_at	7,382	7,469	7,641	8,233	7,810	7,901
1554193_s_at	5,219	5,556	4,975	5,557	5,393	5,435
1554194_at	5,698	6,128	5,975	6,254	5,987	6,133
1554195_a_at	6,939	7,166	6,823	8,287	8,333	8,090
1554196_at	4,994	4,652	4,781	5,426	5,452	5,579
1554198_at	7,369	7,163	7,223	7,206	7,269	7,424
1554199_at	8,326	8,149	8,178	5,104	5,273	5,221

1554201_at	8,208	8,161	8,328	9,118	8,865	9,205
1554202_x_at	7,742	7,410	7,665	8,541	8,300	8,582
1554203_at	5,995	5,971	6,094	7,254	7,056	7,418
1554205_s_at	7,710	8,023	7,172	7,310	7,434	7,903
1554206_at	6,967	7,232	6,730	7,688	7,412	7,784
1554208_at	7,590	7,566	7,564	7,781	7,826	7,838
1554210_at	5,727	6,148	6,100	6,564	6,659	6,703
1554212_s_at	7,662	7,284	7,466	8,649	8,736	8,867
1554213_at	8,201	7,290	7,450	7,024	6,951	7,235
1554214_at	5,450	5,477	5,400	6,012	5,676	6,085
1554216_at	4,615	4,298	4,154	3,974	3,904	3,935
1554217_a_at	4,737	4,958	4,680	4,813	4,942	4,958
1554219_at	7,073	7,041	7,116	8,164	8,010	8,010
1554220_a_at	5,856	5,619	5,716	6,021	5,809	5,960
1554222_at	7,796	7,612	7,606	8,365	8,253	8,281
1554224_at	6,526	5,844	6,141	6,910	6,511	7,111
1554225_a_at	3,980	4,166	4,129	4,087	4,093	4,187
1554228_a_at	7,061	6,649	6,929	7,641	7,503	7,991
1554229_at	5,857	6,566	6,098	6,049	7,390	7,177
1554231_a_at	6,780	6,328	6,966	7,151	7,144	7,204
1554232_a_at	5,106	5,370	5,504	5,687	5,346	5,617
1554233_at	3,739	3,815	3,676	4,167	3,959	4,174
1554234_at	6,696	6,682	6,378	6,750	6,314	6,972
1554235_at	5,201	4,882	4,990	5,783	4,789	5,471
1554237_at	4,991	5,542	5,071	5,016	5,223	4,964
1554239_s_at	7,359	7,920	7,922	9,618	8,979	9,385
1554240_a_at	5,200	5,087	5,285	5,398	5,519	5,634
1554241_at	8,684	8,962	8,243	7,752	9,366	7,555
1554242_a_at	8,027	8,065	7,818	5,206	5,568	5,318
1554245_x_at	7,758	7,726	8,000	8,263	7,648	7,939
1554246_at	5,397	5,478	5,193	5,457	5,467	5,621
1554248_at	8,861	9,406	9,272	6,564	7,899	6,359
1554249_a_at	7,825	8,099	7,097	5,946	6,151	5,967
1554250_s_at	6,813	7,275	7,477	5,823	6,791	6,147
1554251_at	11,007	10,857	11,058	8,982	9,561	9,517
1554252_a_at	4,992	5,116	4,857	4,679	4,785	4,829
1554253_a_at	6,168	5,923	6,218	6,547	6,813	6,776
1554256_a_at	8,907	9,419	9,221	6,943	6,934	6,863
1554258_a_at	6,945	7,135	6,866	7,271	7,482	7,369
1554259_at	7,450	7,416	7,260	7,958	7,689	8,036
1554260_a_at	6,917	7,382	6,647	6,516	7,717	6,782
1554261_at	5,559	5,450	5,758	6,003	5,763	5,794
1554262_s_at	7,026	7,386	7,309	6,992	7,199	7,169
1554264_at	5,425	6,527	5,805	5,042	5,750	5,133
1554266_at	5,826	5,968	6,027	5,871	5,936	6,012
1554268_at	5,852	5,898	6,030	6,280	6,339	6,498
1554271_a_at	7,923	8,082	7,980	7,008	6,577	6,805
1554272_at	3,748	3,712	3,630	4,050	3,790	3,883
1554273_a_at	4,419	4,630	3,959	4,600	4,588	4,554
1554274_a_at	6,263	6,558	6,400	7,237	7,238	6,989
1554276_at	5,399	5,959	5,390	5,932	5,261	5,622
1554277_s_at	5,589	5,921	5,235	5,374	5,334	5,067
1554279_a_at	7,308	7,266	7,331	7,631	7,339	7,687
1554280_a_at	5,963	5,107	5,663	5,905	5,554	5,599
1554281_at	4,474	4,281	4,575	4,374	4,270	4,405
1554283_at	7,019	7,010	7,694	8,301	7,079	7,711

1554285_at	5,853	5,876	5,846	6,828	6,531	6,904
1554286_at	7,297	7,252	7,403	7,412	7,506	7,486
1554287_at	7,688	7,060	6,964	7,155	6,830	6,822
1554288_at	5,841	5,555	5,448	6,266	6,113	6,271
1554290_at	4,445	4,907	5,034	5,617	5,996	5,790
1554291_at	5,275	5,737	5,618	5,481	5,537	5,260
1554292_a_at	6,213	5,915	5,866	6,160	6,272	5,958
1554293_at	4,605	4,449	4,026	7,132	7,338	7,387
1554294_s_at	5,058	5,438	4,880	5,498	5,313	5,408
1554295_x_at	4,790	5,084	4,862	6,528	6,632	6,189
1554296_at	4,009	4,193	4,191	4,668	4,497	4,738
1554298_a_at	3,755	4,364	3,910	3,744	3,538	3,567
1554299_at	5,219	6,263	6,293	5,711	5,248	5,282
1554300_a_at	5,999	5,947	6,210	4,914	5,081	5,010
1554301_at	4,196	4,093	4,146	4,519	4,605	4,822
1554302_s_at	5,495	5,037	5,344	5,621	5,645	5,682
1554303_at	6,235	6,107	6,062	6,522	6,532	6,647
1554305_at	7,114	7,323	7,314	8,092	7,695	7,669
1554306_at	4,435	4,592	4,574	4,116	4,076	4,133
1554307_at	4,733	4,453	4,656	5,192	4,870	5,262
1554308_s_at	4,043	4,039	4,170	4,099	4,003	4,064
1554309_at	5,496	7,369	6,743	5,508	6,222	5,343
1554310_a_at	6,091	6,430	6,649	6,440	6,536	6,683
1554311_a_at	4,598	4,147	4,349	4,916	4,759	4,884
1554312_at	7,283	7,546	7,527	7,667	7,718	7,892
1554314_at	6,347	4,358	6,097	3,532	3,566	3,464
1554316_at	7,393	7,184	7,179	6,881	6,694	6,775
1554317_s_at	6,727	6,795	6,945	7,676	7,240	7,627
1554318_at	6,313	6,207	5,953	6,432	6,776	6,670
1554319_at	3,882	3,780	3,726	3,792	3,631	3,829
1554321_a_at	6,134	5,843	6,366	6,640	6,073	6,774
1554322_a_at	6,754	6,730	6,782	7,790	7,219	7,654
1554324_s_at	4,423	4,920	4,701	4,424	4,410	4,295
1554325_at	5,185	4,815	4,968	5,868	5,848	6,041
1554327_a_at	6,808	6,769	6,744	7,834	7,525	7,817
1554328_at	5,021	4,255	4,592	4,154	4,171	4,492
1554329_x_at	4,636	4,797	4,439	4,193	4,216	4,437
1554331_a_at	7,525	7,069	7,277	7,162	7,061	7,099
1554332_a_at	6,834	6,626	6,486	7,646	7,326	7,584
1554333_at	3,269	3,346	3,314	3,684	3,649	3,657
1554334_a_at	6,423	6,603	6,553	7,339	7,622	8,004
1554335_at	5,507	5,621	5,801	5,363	5,370	5,424
1554337_at	6,044	5,984	5,984	6,627	6,729	6,874
1554339_a_at	5,328	5,879	5,030	4,682	4,653	4,753
1554340_a_at	7,487	7,272	7,718	4,948	4,820	4,985
1554341_a_at	6,764	7,102	6,788	6,795	6,951	6,749
1554342_s_at	6,802	7,407	6,836	6,897	6,961	7,009
1554343_a_at	4,988	4,611	5,179	5,321	5,288	5,468
1554344_s_at	9,321	9,225	9,213	9,987	9,760	10,219
1554345_a_at	8,619	8,970	8,413	7,438	7,778	7,407
1554346_at	7,048	7,188	7,144	7,384	7,213	7,286
1554348_s_at	7,212	7,140	7,191	6,753	6,567	6,613
1554349_at	7,083	6,886	6,803	7,368	7,220	7,496
1554351_a_at	8,334	8,984	8,369	9,093	7,830	8,757
1554352_s_at	7,055	7,706	6,773	7,189	8,556	7,186
1554354_at	3,285	3,384	3,518	3,461	3,395	3,522

1554355_a_at	4,109	4,285	4,980	4,210	4,197	4,443
1554356_at	5,857	5,709	6,099	5,639	5,461	5,593
1554360_at	6,635	6,303	6,697	6,964	6,965	6,924
1554362_at	3,789	3,816	3,997	3,970	3,752	3,995
1554364_at	4,122	4,524	4,264	5,057	4,886	5,082
1554365_a_at	9,077	9,371	8,328	8,655	8,254	8,402
1554367_at	6,307	6,343	6,638	7,076	6,952	7,193
1554368_at	3,990	4,199	4,036	4,164	3,977	4,162
1554371_at	6,181	6,101	6,262	6,757	6,491	6,836
1554372_at	5,678	5,496	5,533	6,745	6,773	6,912
1554374_at	5,522	5,061	5,241	5,533	5,664	5,756
1554375_a_at	4,587	4,657	4,770	5,167	5,093	5,030
1554376_s_at	6,919	6,744	7,068	7,767	7,781	8,185
1554377_a_at	5,426	5,289	5,636	6,298	5,894	6,501
1554378_a_at	4,734	4,697	4,611	5,302	5,049	5,363
1554379_a_at	7,810	7,438	7,825	7,729	8,119	8,347
1554380_at	5,420	5,614	5,501	6,441	6,285	6,494
1554382_at	7,243	7,414	7,291	8,018	7,964	7,826
1554383_a_at	7,281	7,288	7,279	7,613	7,822	7,786
1554384_at	4,332	4,635	4,276	4,320	4,003	4,029
1554385_a_at	6,133	6,203	5,847	6,715	6,127	6,346
1554386_at	7,503	6,831	6,906	7,668	7,622	7,851
1554388_at	7,062	6,785	7,094	7,336	7,429	7,376
1554390_s_at	9,298	9,528	9,159	9,913	9,474	9,734
1554391_at	6,503	6,263	6,390	6,838	6,414	6,937
1554393_a_at	7,220	7,151	7,119	7,539	7,221	7,415
1554394_at	6,002	5,591	5,821	6,726	6,369	6,741
1554396_at	7,425	7,512	7,630	8,550	7,767	7,926
1554397_s_at	6,289	6,143	6,207	7,412	6,846	6,490
1554398_at	5,742	6,131	5,920	5,808	5,886	6,061
1554400_at	8,901	8,374	8,684	10,623	10,097	10,157
1554401_a_at	5,270	5,092	5,355	6,087	6,121	6,294
1554403_a_at	4,314	4,604	4,155	4,650	4,505	4,588
1554404_a_at	6,344	6,911	6,621	7,889	7,574	7,415
1554405_a_at	5,886	6,030	5,601	6,524	5,906	6,072
1554406_a_at	7,142	7,159	7,304	7,887	7,566	7,646
1554408_a_at	8,728	8,436	8,669	9,722	9,127	9,592
1554409_at	4,857	4,646	5,063	5,370	5,387	5,680
1554410_a_at	8,366	8,300	8,576	8,376	8,056	8,365
1554411_at	5,087	4,874	4,999	5,025	5,240	4,897
1554413_s_at	5,373	5,480	5,166	6,696	6,356	6,909
1554414_a_at	7,340	7,125	7,032	8,286	7,604	7,854
1554415_at	7,933	8,764	8,217	7,329	7,609	7,159
1554417_s_at	7,787	7,755	7,639	7,782	7,532	7,760
1554418_s_at	4,299	4,457	4,219	4,489	4,269	4,429
1554419_x_at	6,812	6,804	6,911	7,198	7,027	7,176
1554420_at	6,366	6,191	6,273	7,672	7,448	7,486
1554423_a_at	9,189	9,067	8,936	9,005	8,641	9,034
1554424_at	6,368	6,103	5,940	5,922	5,600	5,920
1554428_s_at	8,272	8,107	8,251	8,149	8,037	8,125
1554429_a_at	8,414	8,452	8,677	9,101	8,938	9,554
1554430_at	7,528	7,781	7,461	8,436	7,628	8,218
1554432_x_at	7,851	7,743	7,826	8,842	7,677	8,138
1554433_a_at	8,336	8,179	7,462	6,081	6,057	5,908
1554436_a_at	5,244	5,127	5,217	5,512	5,291	5,300
1554438_at	5,379	5,649	5,483	6,120	6,020	5,702

1554440_at	5,405	5,750	5,280	5,854	5,363	5,280
1554441_a_at	8,990	8,750	8,548	8,078	8,967	7,651
1554442_at	8,855	8,690	8,628	8,822	8,758	9,274
1554443_s_at	5,718	5,489	5,752	6,399	6,206	6,431
1554444_s_at	8,265	8,114	8,401	8,529	8,150	8,513
1554445_at	8,047	8,090	8,220	8,642	7,130	7,948
1554447_at	7,576	7,668	7,456	5,431	5,633	5,331
1554448_at	6,597	6,027	6,468	6,146	5,841	5,988
1554449_at	4,626	4,973	4,555	4,291	4,190	4,256
1554450_s_at	8,787	8,668	8,092	6,594	6,583	6,492
1554451_s_at	7,332	7,077	7,297	6,535	6,342	6,460
1554452_a_at	8,738	8,320	8,402	7,615	7,395	7,802
1554453_at	5,576	5,701	6,061	6,553	6,559	6,873
1554455_at	7,926	7,410	6,923	5,839	6,686	5,749
1554456_a_at	7,058	7,024	5,636	5,253	4,983	5,063
1554459_s_at	6,986	7,069	6,341	5,825	5,861	5,843
1554460_at	3,770	3,860	3,849	3,658	3,861	4,013
1554462_a_at	7,764	7,630	7,678	7,972	7,891	8,092
1554464_a_at	7,785	7,913	7,927	9,173	7,275	8,882
1554465_s_at	5,825	6,130	5,623	5,672	5,143	5,513
1554466_a_at	7,511	7,382	7,341	7,314	6,761	7,452
1554468_s_at	7,160	6,864	6,993	7,621	7,297	7,487
1554469_at	4,856	5,308	5,258	4,861	5,012	4,855
1554470_s_at	5,718	5,932	6,085	5,558	5,005	5,379
1554471_a_at	7,962	8,102	7,680	6,728	7,523	6,992
1554472_a_at	6,695	7,249	6,678	5,375	5,439	5,262
1554473_at	4,138	4,203	4,360	4,901	6,517	5,153
1554474_a_at	6,726	6,882	6,223	7,602	8,925	7,759
1554475_a_at	7,177	6,786	6,838	7,510	7,607	7,637
1554476_x_at	6,239	5,880	5,458	7,708	7,887	7,937
1554478_a_at	6,627	6,624	6,859	6,575	6,318	6,578
1554479_a_at	7,617	7,718	7,305	7,340	7,483	7,102
1554480_a_at	9,947	9,990	10,091	8,450	8,093	8,036
1554481_a_at	7,307	6,705	7,125	7,196	7,198	7,547
1554482_a_at	6,461	6,530	6,158	7,770	7,484	8,155
1554483_at	8,363	8,473	8,437	8,694	8,781	8,890
1554485_s_at	6,586	6,799	6,660	5,815	6,005	6,568
1554486_a_at	4,543	4,804	4,390	5,362	5,290	5,118
1554487_a_at	5,072	4,609	4,889	5,187	5,415	5,433
1554488_at	4,756	5,479	4,973	4,907	4,756	5,019
1554489_a_at	7,219	7,671	6,401	5,940	5,777	5,159
1554491_a_at	5,179	5,114	5,390	5,508	5,300	5,421
1554492_at	3,960	4,519	4,054	3,682	3,730	3,628
1554493_s_at	6,931	7,692	6,636	6,152	6,110	6,137
1554494_at	5,519	5,318	5,491	6,812	6,349	6,837
1554495_s_at	6,235	6,185	6,472	7,178	6,837	7,062
1554496_at	6,211	6,222	6,641	7,629	7,434	7,458
1554499_s_at	6,323	6,406	6,324	7,527	6,498	6,833
1554500_a_at	5,189	5,234	4,739	5,161	5,076	5,125
1554501_at	6,099	5,267	5,626	6,388	6,355	6,158
1554503_a_at	6,849	6,492	6,581	6,998	6,590	6,590
1554504_at	3,678	3,586	3,449	3,436	3,434	3,658
1554506_x_at	3,630	3,860	3,578	3,734	3,646	3,867
1554507_at	4,406	4,873	3,931	3,449	3,531	3,490
1554508_at	4,658	4,996	4,487	5,095	4,857	4,986
1554509_a_at	7,238	7,397	7,022	7,747	7,652	7,812

1554510_s_at	9,853	9,744	9,725	10,430	9,948	10,538
1554511_at	6,798	6,641	6,324	7,442	6,896	7,216
1554512_a_at	5,740	5,703	5,951	6,470	6,335	6,482
1554513_s_at	4,976	4,314	4,649	4,646	4,591	4,710
1554514_at	5,714	5,316	6,136	6,572	6,601	6,700
1554516_at	8,320	7,986	8,100	8,466	8,617	8,508
1554517_x_at	7,775	7,677	7,638	7,831	8,147	8,136
1554518_at	7,999	7,867	7,757	7,769	7,466	7,739
1554519_at	5,682	5,610	5,396	6,323	6,117	5,971
1554520_at	7,052	6,464	6,460	6,483	6,237	6,521
1554522_at	3,689	4,106	4,132	4,383	4,246	4,586
1554523_a_at	7,428	7,348	7,652	8,249	7,701	8,141
1554524_a_at	4,185	3,984	4,162	4,357	4,094	4,283
1554526_at	3,670	4,077	3,651	3,856	3,807	3,690
1554528_at	5,087	5,040	4,857	5,196	5,122	5,376
1554530_at	5,221	5,464	5,180	5,042	5,054	4,940
1554531_at	6,979	6,982	7,063	7,305	7,155	7,248
1554533_at	4,875	4,544	5,227	5,810	6,030	6,108
1554534_at	6,257	5,992	5,880	6,139	6,036	6,297
1554536_at	4,097	3,838	4,647	4,808	4,544	4,846
1554537_at	5,568	5,493	5,331	6,204	5,659	5,502
1554538_at	7,270	7,376	7,174	7,368	7,468	7,153
1554539_a_at	7,345	7,085	7,309	7,565	7,190	7,573
1554540_at	4,135	4,074	4,476	4,011	3,821	4,068
1554541_a_at	7,710	7,657	7,960	7,051	7,165	7,338
1554542_at	7,577	7,496	7,597	7,993	8,113	8,117
1554543_at	6,295	5,853	5,925	5,998	6,627	5,902
1554544_a_at	7,988	7,989	7,879	8,759	8,528	8,513
1554545_at	5,497	5,322	5,511	5,880	5,458	5,478
1554547_at	8,282	8,307	7,924	5,161	5,154	4,954
1554549_a_at	6,925	6,640	6,708	4,988	5,813	5,287
1554550_at	3,657	3,898	3,672	3,922	3,983	4,021
1554553_s_at	7,670	7,641	7,437	8,510	7,913	8,803
1554554_at	6,561	7,067	6,864	8,142	8,114	8,227
1554555_a_at	9,891	9,536	9,819	8,457	7,943	8,158
1554556_a_at	7,808	7,539	8,034	8,894	9,103	8,902
1554557_at	5,967	5,769	5,850	6,158	6,780	6,434
1554558_at	4,971	5,432	4,831	4,523	4,677	4,554
1554559_at	7,201	6,923	7,489	8,202	7,760	7,869
1554560_at	4,426	4,533	4,860	4,892	4,951	4,916
1554564_a_at	6,275	6,163	6,577	6,975	7,326	7,252
1554565_x_at	7,604	7,182	7,556	7,287	7,687	7,552
1554566_at	7,441	7,148	7,224	7,980	7,770	7,578
1554568_at	5,369	5,592	5,317	5,542	5,414	5,434
1554569_a_at	7,101	6,850	6,951	7,660	7,092	7,283
1554571_at	5,766	5,804	5,455	6,321	6,293	6,420
1554572_a_at	10,612	10,431	10,481	9,467	9,080	8,621
1554574_a_at	7,844	7,666	7,854	9,200	9,237	9,838
1554575_a_at	8,160	8,333	8,145	8,339	8,075	8,380
1554576_a_at	9,125	8,823	8,802	7,694	7,378	7,770
1554577_a_at	9,993	10,141	9,623	8,877	8,519	9,057
1554578_at	7,054	7,394	7,445	7,634	7,487	7,722
1554579_a_at	6,370	6,670	6,771	7,143	6,776	7,148
1554580_a_at	6,922	7,145	7,290	7,501	7,538	7,532
1554582_a_at	6,250	6,636	6,724	5,734	5,425	5,591
1554583_a_at	6,458	6,787	6,867	7,021	7,048	6,892

1554584_at	6,092	5,964	6,325	6,581	6,188	6,340
1554586_a_at	7,130	6,869	7,306	7,683	7,245	7,497
1554588_a_at	6,538	6,379	6,706	6,593	5,206	5,884
1554589_at	6,104	6,161	6,079	6,806	6,672	6,864
1554591_at	6,659	6,965	6,842	6,843	6,965	6,895
1554592_a_at	8,363	8,277	8,130	4,220	4,329	3,999
1554593_s_at	9,243	9,453	9,342	4,020	3,885	3,908
1554594_at	4,913	4,518	4,638	5,743	5,291	5,534
1554595_at	9,560	8,790	9,403	8,557	8,728	8,445
1554597_at	8,965	9,047	9,263	7,746	7,839	7,887
1554599_x_at	8,779	8,894	9,262	7,870	7,643	7,785
1554600_s_at	7,696	7,258	7,694	10,390	10,131	10,880
1554601_at	5,582	5,606	5,191	5,501	5,561	5,403
1554602_at	7,958	8,011	7,756	7,779	7,940	7,819
1554604_at	5,961	5,949	6,302	5,938	5,922	6,012
1554606_at	4,962	4,943	4,778	5,144	4,551	4,800
1554607_at	5,574	5,488	5,645	5,766	5,720	5,409
1554608_at	4,661	4,565	4,420	4,414	4,639	4,663
1554609_at	6,611	7,430	6,288	5,201	5,056	5,254
1554610_at	5,882	5,544	5,610	6,636	6,834	7,023
1554612_at	4,863	4,621	5,046	6,228	5,929	6,630
1554613_a_at	7,607	7,398	7,480	8,348	8,371	8,349
1554614_a_at	5,635	5,252	4,434	4,158	4,145	4,203
1554615_at	5,299	5,272	5,581	5,915	5,783	5,602
1554616_at	7,433	7,481	7,592	8,013	7,763	7,883
1554618_at	6,101	6,223	6,124	6,768	6,555	6,845
1554619_at	4,813	4,763	4,886	4,857	4,878	5,373
1554621_at	5,623	6,239	5,602	7,652	7,697	8,024
1554623_x_at	6,538	6,495	6,592	6,671	6,756	6,647
1554624_a_at	4,529	4,670	4,676	4,526	4,532	4,816
1554625_at	7,397	7,478	7,529	8,168	7,871	8,284
1554627_a_at	9,039	9,273	8,633	8,596	8,455	8,342
1554628_at	8,301	9,117	8,411	6,560	6,820	5,770
1554629_at	4,180	3,664	4,186	4,587	4,560	4,386
1554631_at	5,836	5,852	5,794	5,710	5,607	5,745
1554633_a_at	5,034	5,317	5,363	5,447	5,483	5,526
1554634_at	4,414	4,209	4,403	4,388	4,355	4,529
1554635_a_at	6,945	7,099	6,861	7,202	7,120	7,487
1554636_at	4,304	3,999	3,909	3,786	4,098	3,887
1554637_a_at	6,261	6,862	6,445	6,814	6,829	6,765
1554638_at	6,162	6,670	6,232	5,600	6,287	5,489
1554640_at	7,394	7,020	6,898	6,131	6,111	5,869
1554641_a_at	7,625	7,389	7,816	7,798	7,745	7,851
1554642_at	6,959	7,019	7,275	7,365	7,401	7,494
1554643_at	5,334	5,534	5,535	5,809	5,863	6,112
1554646_at	8,248	8,072	7,855	7,577	7,345	7,386
1554648_a_at	5,822	6,129	6,179	6,771	6,214	6,522
1554649_at	6,437	6,695	6,536	6,577	6,489	6,488
1554650_a_at	7,277	7,034	7,359	7,858	7,581	7,635
1554652_s_at	6,651	6,511	7,051	7,205	6,857	7,104
1554655_a_at	7,456	7,792	7,650	6,281	6,473	6,454
1554657_a_at	6,110	6,278	6,412	6,956	6,924	6,991
1554659_at	7,534	7,139	7,987	7,375	7,289	7,301
1554660_a_at	6,606	7,333	7,069	6,837	6,999	6,971
1554661_s_at	8,016	8,282	7,963	7,403	7,820	7,425
1554662_at	6,084	6,084	6,181	7,221	7,163	7,180

1554663_a_at	4,414	4,386	4,596	5,017	4,595	4,906
1554664_at	5,583	5,557	5,463	5,751	5,764	5,742
1554665_at	4,696	4,579	4,593	4,240	4,358	4,602
1554666_at	6,684	6,847	7,051	7,398	7,412	7,597
1554667_s_at	9,027	8,994	9,008	9,145	9,237	9,069
1554668_a_at	6,597	6,664	6,708	6,990	6,959	7,106
1554670_at	4,535	4,593	4,258	3,931	4,138	4,435
1554671_a_at	8,622	8,497	8,544	8,032	7,929	8,286
1554672_at	6,101	6,477	5,959	6,648	6,279	6,537
1554675_a_at	5,852	6,099	5,924	7,303	7,284	7,772
1554676_at	6,004	6,428	6,288	6,407	6,520	6,503
1554677_s_at	6,772	6,908	6,975	7,271	7,051	7,386
1554678_s_at	11,021	10,657	10,227	9,830	9,620	9,680
1554679_a_at	10,111	9,670	9,861	7,472	6,240	6,729
1554680_s_at	6,814	6,586	6,628	7,190	6,874	7,080
1554681_a_at	6,676	6,265	6,432	7,897	7,924	8,029
1554683_a_at	6,510	6,649	6,475	6,852	6,628	6,713
1554685_a_at	7,164	6,711	6,588	7,415	7,182	7,428
1554686_at	5,022	5,968	4,780	5,445	4,963	5,327
1554689_a_at	7,362	7,125	7,671	6,973	7,137	7,184
1554690_a_at	6,961	7,370	6,600	7,733	7,543	7,712
1554691_a_at	8,126	7,813	8,003	8,484	8,315	8,552
1554692_at	6,116	6,145	6,278	6,494	6,528	6,767
1554694_at	5,585	5,353	5,704	6,347	6,207	6,646
1554696_s_at	10,014	9,779	9,521	9,600	8,821	9,206
1554697_at	5,766	5,709	5,849	5,948	5,810	6,002
1554699_at	5,295	5,613	5,474	5,542	5,351	5,482
1554700_at	3,834	4,078	3,830	3,937	3,908	3,786
1554701_a_at	8,361	7,940	8,420	8,711	8,197	8,712
1554702_at	5,239	5,723	5,340	5,379	5,328	5,252
1554703_at	8,557	8,195	8,356	7,040	7,231	7,025
1554704_at	6,924	7,646	6,559	7,115	7,023	6,963
1554705_at	6,760	7,082	6,662	7,102	6,958	7,233
1554706_at	3,745	3,832	3,834	3,730	3,957	4,166
1554707_at	5,807	5,813	6,282	7,327	7,679	7,676
1554708_s_at	6,438	6,454	6,630	6,789	7,157	6,945
1554710_at	4,982	5,058	5,226	5,672	5,750	5,726
1554711_at	6,615	6,793	6,718	7,191	7,137	7,150
1554712_a_at	6,445	6,343	6,219	6,482	6,405	6,397
1554714_at	5,678	5,728	5,957	7,136	6,831	7,054
1554715_at	6,558	6,745	6,829	6,867	6,790	7,030
1554716_s_at	5,499	5,551	5,595	5,532	5,758	5,690
1554717_a_at	5,251	5,260	5,556	5,567	5,085	5,338
1554719_at	6,095	6,680	6,885	8,224	7,422	8,135
1554721_a_at	8,313	8,071	8,011	8,171	8,423	8,238
1554722_at	5,219	5,239	5,039	5,415	5,709	5,668
1554723_x_at	3,507	3,550	3,689	3,835	3,667	3,835
1554724_at	6,071	5,714	5,921	4,081	3,967	3,934
1554725_at	4,126	4,540	4,236	4,173	4,285	4,086
1554726_at	5,267	5,438	4,996	4,280	4,549	4,227
1554728_at	5,574	5,574	5,541	6,305	6,050	6,252
1554730_at	6,692	6,742	6,617	7,343	7,480	7,230
1554732_at	6,302	6,156	6,244	6,731	6,501	6,410
1554733_at	4,289	4,153	4,081	4,057	3,995	3,992
1554735_a_at	6,955	6,855	7,066	7,637	7,718	7,666
1554736_at	5,314	4,748	4,726	5,875	5,877	5,990

1554737_at	4,967	5,434	5,346	5,376	5,142	5,355
1554739_at	5,010	5,615	5,214	5,415	5,078	4,775
1554740_a_at	8,285	8,632	7,834	7,353	6,971	7,019
1554741_s_at	5,229	6,012	5,301	7,960	5,369	5,195
1554742_at	5,009	5,258	5,377	4,617	5,038	4,639
1554743_x_at	6,243	6,518	6,799	5,682	5,983	5,678
1554744_at	4,241	4,225	4,275	4,168	4,176	4,138
1554745_at	5,705	5,464	5,817	6,461	6,088	6,731
1554747_a_at	9,875	9,860	9,493	8,625	9,210	9,000
1554748_at	7,149	7,681	7,073	7,790	7,992	7,989
1554749_s_at	7,581	7,642	7,505	8,289	7,574	7,857
1554751_at	5,259	5,932	4,478	6,211	6,285	6,404
1554752_a_at	6,941	6,779	6,957	7,688	7,259	7,403
1554755_a_at	5,778	5,981	5,980	6,460	6,167	6,444
1554757_a_at	5,618	5,561	5,807	6,582	6,276	6,962
1554758_a_at	6,903	6,849	6,773	7,367	6,989	7,283
1554759_at	7,064	6,940	7,178	7,497	7,452	7,531
1554761_a_at	7,838	7,292	7,509	6,627	5,689	6,372
1554762_a_at	7,862	7,549	7,615	7,363	7,196	7,452
1554763_at	5,772	5,592	5,719	6,096	5,732	6,068
1554764_a_at	3,649	4,201	3,740	3,806	3,727	3,637
1554765_a_at	5,587	5,632	5,717	6,509	6,167	6,435
1554766_s_at	5,168	4,751	5,352	6,027	5,757	5,960
1554767_s_at	6,509	6,445	5,633	5,346	5,379	5,407
1554768_a_at	9,512	9,457	8,488	7,091	6,733	7,199
1554769_at	4,875	4,409	4,702	4,405	4,051	4,656
1554770_x_at	5,083	4,419	4,724	4,175	4,205	4,541
1554771_at	7,224	6,321	6,306	5,786	5,731	5,614
1554772_at	5,195	5,398	5,150	5,656	5,377	5,528
1554773_at	6,218	6,131	6,174	6,636	6,772	6,874
1554774_at	6,564	6,734	6,323	6,132	5,965	6,137
1554776_at	4,564	4,504	4,555	4,648	4,493	4,610
1554777_at	4,805	5,075	4,821	5,798	5,864	5,487
1554778_at	3,602	3,562	3,405	3,993	3,805	3,688
1554779_s_at	5,990	5,970	6,291	5,837	6,016	6,031
1554780_a_at	7,184	7,284	6,791	6,816	6,741	6,785
1554781_at	4,205	4,054	4,244	4,625	4,410	4,359
1554782_at	6,476	6,364	6,434	6,990	6,398	6,699
1554783_s_at	6,180	6,596	6,545	7,301	7,111	7,727
1554784_at	4,323	4,257	4,245	4,514	4,526	4,426
1554785_at	4,336	4,624	4,405	4,081	4,430	4,156
1554786_at	4,449	4,675	4,382	4,464	4,497	4,527
1554787_at	7,163	7,103	6,859	7,288	7,049	7,143
1554789_a_at	4,640	4,861	4,744	5,013	5,048	4,988
1554790_at	6,700	6,719	6,545	6,642	6,459	6,383
1554791_a_at	6,947	6,601	5,982	5,915	5,326	5,723
1554793_at	3,828	3,957	3,929	5,749	5,719	5,466
1554794_a_at	6,167	6,553	6,459	8,128	7,656	7,783
1554795_a_at	6,978	7,113	7,032	6,974	7,079	7,282
1554796_at	7,490	6,883	7,755	6,637	6,832	6,581
1554797_at	3,739	4,115	3,818	3,970	3,961	3,886
1554798_at	6,327	6,533	6,553	6,777	6,518	6,641
1554800_at	6,862	6,871	6,810	6,395	6,006	6,240
1554801_at	5,272	5,060	4,906	5,459	5,640	5,538
1554803_s_at	6,515	6,459	6,500	6,997	7,013	6,952
1554804_a_at	8,481	8,734	8,705	8,326	8,665	8,909

1554805_at	6,693	6,696	6,837	7,099	7,256	7,291
1554806_a_at	8,453	9,258	8,523	9,305	9,152	9,339
1554807_a_at	7,402	7,354	7,571	7,726	7,767	7,965
1554808_at	5,206	5,027	5,138	5,458	5,610	5,788
1554809_at	7,558	7,096	7,199	7,926	7,713	7,886
1554810_at	5,241	5,495	4,866	6,936	6,681	6,995
1554812_at	4,833	4,564	4,652	5,056	5,216	5,179
1554813_at	6,133	5,857	6,372	6,221	6,419	6,131
1554814_at	7,830	7,512	7,548	8,026	8,007	8,277
1554816_at	4,023	4,178	4,286	4,252	4,172	4,104
1554818_s_at	5,470	5,291	5,366	6,367	6,037	6,313
1554819_a_at	7,639	7,538	7,663	7,827	7,987	8,276
1554820_at	3,885	3,744	4,008	4,271	4,220	4,386
1554821_a_at	8,291	7,821	8,359	7,090	6,342	7,143
1554822_at	6,461	6,841	6,360	6,459	6,653	6,538
1554824_at	4,760	4,842	4,646	5,260	4,779	5,318
1554825_at	4,979	5,010	4,991	6,024	6,308	6,311
1554826_at	6,464	6,804	6,762	7,195	7,384	7,717
1554827_a_at	7,683	7,225	7,783	8,519	8,272	8,681
1554828_at	4,979	4,363	5,094	5,209	5,015	5,000
1554830_a_at	6,976	7,397	7,350	9,043	8,411	8,312
1554831_x_at	4,461	4,579	4,856	5,289	5,917	5,541
1554833_at	5,380	5,563	5,390	5,367	5,405	5,416
1554834_a_at	6,414	6,213	6,249	6,998	6,532	6,836
1554835_a_at	6,843	6,834	6,696	7,092	7,023	7,035
1554836_at	5,865	5,840	6,150	5,690	5,858	5,698
1554837_a_at	6,677	6,950	6,732	7,630	7,665	7,886
1554839_at	6,659	6,683	6,960	6,689	6,205	6,543
1554840_at	5,931	6,212	6,427	6,391	6,241	6,218
1554841_at	4,414	4,564	4,783	5,083	4,605	4,911
1554842_at	6,647	6,533	6,788	8,543	8,655	9,094
1554844_at	4,588	4,409	4,497	4,836	4,615	5,040
1554846_at	4,768	4,690	4,742	4,769	5,123	5,097
1554847_at	6,897	6,508	6,726	7,174	7,281	7,453
1554849_at	5,513	5,471	5,487	5,927	5,621	5,835
1554850_at	6,429	6,084	6,542	7,034	7,184	7,298
1554852_a_at	6,601	6,909	6,369	6,164	6,113	6,480
1554853_at	4,504	4,731	4,361	5,286	5,245	5,257
1554855_at	4,913	5,078	4,734	4,732	4,687	4,858
1554857_at	7,258	7,262	7,412	7,888	7,574	8,219
1554860_at	6,467	6,556	6,595	6,671	6,602	6,791
1554862_at	4,243	4,267	4,270	4,400	4,430	4,587
1554863_s_at	8,060	8,223	7,926	8,242	8,015	7,765
1554864_a_at	7,342	6,324	7,476	8,196	8,209	8,466
1554865_at	3,802	3,666	4,053	4,304	3,975	4,013
1554866_at	3,956	3,976	4,081	3,999	4,067	4,158
1554867_a_at	7,072	6,287	6,872	7,078	7,628	8,073
1554868_s_at	10,574	10,314	9,775	8,373	8,843	8,219
1554869_at	7,059	7,249	7,140	7,875	7,587	7,880
1554871_at	3,800	4,356	4,036	3,864	3,806	3,680
1554872_a_at	7,089	6,890	6,827	7,224	7,086	7,180
1554873_at	5,697	5,250	5,621	5,738	5,887	5,986
1554874_at	4,398	4,178	4,269	4,441	4,261	4,715
1554875_at	5,525	4,645	5,801	4,959	4,530	4,926
1554876_a_at	5,113	5,076	4,776	5,636	5,416	5,647
1554878_a_at	6,265	6,530	6,805	7,946	6,076	6,472

1554880_at	7,587	7,578	7,564	7,958	7,837	7,940
1554882_at	5,076	5,511	4,802	4,931	4,784	5,127
1554883_a_at	7,041	7,219	6,412	6,081	5,886	6,702
1554884_at	4,633	4,172	4,622	3,985	4,156	4,008
1554885_a_at	6,686	6,834	6,027	6,464	5,973	6,450
1554886_a_at	6,153	5,815	5,739	7,397	6,886	7,426
1554887_at	5,094	4,746	4,888	5,366	5,048	5,577
1554889_at	5,055	4,766	5,112	4,839	4,478	4,650
1554890_a_at	8,286	8,276	8,217	7,409	7,447	7,613
1554891_at	6,526	6,630	6,732	6,594	6,567	6,578
1554892_a_at	5,009	4,426	4,862	5,229	5,121	5,341
1554894_a_at	7,216	6,773	6,969	8,069	7,438	7,899
1554895_a_at	7,471	6,615	6,660	8,135	8,368	8,520
1554897_s_at	6,843	6,700	6,746	7,801	7,680	7,880
1554899_s_at	5,166	5,142	5,094	5,509	5,445	5,331
1554901_at	4,878	4,838	4,596	4,997	4,932	4,898
1554903_at	7,339	7,689	7,763	8,151	8,400	8,392
1554905_x_at	7,541	7,988	7,908	8,353	8,377	8,594
1554906_a_at	5,743	6,190	5,948	5,835	6,042	6,444
1554907_a_at	6,592	6,389	6,626	7,282	7,181	7,308
1554908_at	4,808	4,469	4,983	5,565	5,254	5,310
1554909_at	6,955	6,644	6,730	6,567	6,628	6,610
1554910_at	4,982	4,511	5,275	4,042	4,154	3,964
1554911_at	7,172	6,849	7,070	7,359	7,137	7,465
1554912_at	5,950	5,269	5,456	4,466	4,099	4,293
1554914_at	6,704	6,900	6,815	6,898	6,906	7,044
1554915_a_at	7,415	7,012	7,592	8,299	7,285	7,585
1554916_a_at	6,262	5,762	6,479	7,092	6,745	6,993
1554918_a_at	6,574	6,184	5,996	6,213	6,500	7,139
1554919_s_at	6,307	6,415	6,333	5,781	5,862	5,793
1554920_at	3,269	3,322	3,387	3,255	3,319	3,320
1554921_a_at	4,090	4,522	4,194	4,095	3,940	4,245
1554922_at	5,080	5,677	5,416	4,381	4,313	4,235
1554923_at	6,615	6,065	5,971	6,799	6,602	6,915
1554927_at	5,149	4,678	4,896	5,633	5,406	5,460
1554929_at	4,917	4,777	4,792	5,350	5,162	5,237
1554930_a_at	7,643	7,264	7,170	6,794	6,722	6,972
1554931_at	5,691	5,444	5,419	5,966	5,861	5,952
1554932_at	4,666	4,890	4,772	5,860	5,739	5,746
1554933_at	6,054	6,291	6,546	4,292	4,294	4,531
1554934_at	5,497	5,242	5,428	5,698	5,605	5,601
1554937_x_at	7,199	7,257	7,312	7,887	7,538	7,826
1554938_a_at	7,572	7,694	7,350	8,689	8,860	8,853
1554940_a_at	6,897	6,791	6,541	7,455	7,428	7,499
1554941_at	5,179	4,997	5,402	5,668	5,665	5,703
1554942_a_at	7,566	7,621	7,418	7,521	7,126	7,574
1554943_at	5,982	6,127	6,112	6,264	6,205	6,567
1554945_x_at	4,926	5,060	5,136	5,425	5,430	5,555
1554946_at	4,820	5,057	5,230	5,506	5,621	5,629
1554947_at	4,627	4,609	4,818	4,806	4,779	4,794
1554948_at	5,080	4,524	4,551	4,335	4,181	4,391
1554949_at	7,352	7,123	7,196	7,791	7,730	7,870
1554950_at	6,601	6,732	6,612	7,044	6,697	6,541
1554951_at	5,550	5,451	5,444	6,013	5,940	6,267
1554952_s_at	6,851	6,729	6,933	7,039	7,056	7,270
1554953_a_at	8,222	7,514	7,994	8,997	9,049	9,179

1554954_at	6,628	6,429	6,363	6,694	6,850	6,878
1554957_at	5,722	5,886	5,753	5,739	5,716	5,837
1554958_at	5,659	5,663	5,021	5,918	5,655	6,024
1554960_at	4,444	4,762	4,555	4,806	4,895	4,801
1554961_at	5,840	6,016	6,067	7,047	6,875	7,102
1554962_a_at	7,935	7,607	7,608	7,765	7,492	7,549
1554963_at	5,588	6,260	5,693	5,488	5,633	5,455
1554964_x_at	5,413	5,464	5,635	5,280	5,393	5,351
1554965_at	5,500	5,293	5,343	5,489	5,518	5,733
1554966_a_at	4,818	6,051	5,340	6,407	6,526	6,153
1554967_at	6,487	6,749	6,492	6,420	6,299	6,356
1554969_x_at	6,354	6,819	6,458	6,591	6,466	6,409
1554970_at	6,260	5,581	5,643	6,615	6,318	6,706
1554971_at	6,681	6,714	6,536	6,275	6,263	6,248
1554972_at	6,806	6,560	6,532	7,381	7,503	7,685
1554973_a_at	6,966	6,933	6,817	7,059	6,859	6,917
1554974_at	7,611	7,164	7,091	7,827	7,729	8,097
1554976_a_at	4,485	4,680	4,689	5,104	5,213	5,157
1554977_at	8,061	7,763	7,913	8,094	7,966	8,111
1554978_at	7,454	7,480	7,350	7,427	7,357	7,404
1554980_a_at	7,607	7,942	7,735	7,957	7,534	8,196
1554981_at	3,603	3,739	3,714	3,674	3,519	3,702
1554982_a_at	4,075	4,090	4,289	5,104	4,738	5,154
1554983_at	5,214	5,413	5,749	6,407	5,841	5,879
1554984_a_at	6,919	6,936	6,920	7,630	7,703	7,952
1554985_at	6,229	6,503	6,043	6,396	6,496	6,550
1554986_a_at	5,403	4,482	5,531	6,089	5,677	6,055
1554987_at	6,191	6,492	6,331	7,537	7,440	7,339
1554988_at	4,313	4,152	4,193	4,630	4,515	4,436
1554989_at	6,971	7,071	7,193	7,836	7,741	7,814
1554992_at	6,903	7,240	6,832	7,837	7,649	7,852
1554994_at	4,063	4,250	4,408	4,748	4,590	4,657
1554996_at	3,920	4,469	4,311	4,424	4,200	4,645
1554997_a_at	4,244	4,211	4,352	7,058	7,121	5,497
1554999_at	4,714	5,099	5,087	5,443	5,635	5,554
1555000_at	5,340	5,352	5,503	5,886	5,558	5,578
1555001_at	4,723	5,080	4,922	5,237	5,198	5,091
1555002_at	3,634	3,535	3,918	3,615	3,584	3,519
1555003_at	5,333	5,117	5,042	5,684	5,740	5,277
1555004_a_at	6,132	5,534	5,488	6,575	6,446	6,180
1555006_at	6,229	6,388	6,536	6,959	6,617	6,737
1555007_s_at	5,587	5,831	5,841	6,154	5,646	5,995
1555009_a_at	6,163	6,393	6,327	6,839	6,534	6,634
1555011_at	4,623	4,849	4,510	4,571	4,701	4,674
1555014_x_at	7,364	6,308	7,331	7,139	7,627	7,580
1555015_a_at	8,081	7,138	8,591	7,278	6,655	6,655
1555016_at	8,035	7,541	7,811	8,717	8,242	8,397
1555018_at	3,749	3,452	3,749	3,815	4,079	3,924
1555019_at	5,969	6,274	6,249	5,689	5,790	5,824
1555020_a_at	7,071	6,945	7,240	7,639	7,738	7,980
1555021_a_at	6,217	6,434	6,426	6,943	6,706	7,253
1555022_at	4,875	4,532	4,429	4,987	4,939	5,054
1555024_at	3,455	3,511	3,792	3,925	3,833	3,711
1555025_at	4,059	3,906	3,824	3,957	3,926	3,978
1555027_at	4,528	5,202	4,723	5,742	5,341	5,898
1555028_at	6,542	6,509	6,435	7,348	7,258	7,513

1555031_at	5,453	5,523	5,511	5,584	5,783	5,599
1555032_at	6,024	5,962	6,320	7,016	7,023	7,163
1555033_a_at	5,873	5,577	5,518	6,291	5,864	5,772
1555034_at	4,817	4,763	4,730	4,424	4,600	4,140
1555035_a_at	5,753	5,675	5,356	6,946	6,611	7,078
1555037_a_at	12,917	12,960	12,481	10,545	10,845	10,756
1555038_at	5,003	4,854	5,469	4,979	4,646	4,779
1555039_a_at	6,527	6,051	6,033	6,695	6,247	6,530
1555040_at	5,633	5,550	5,351	5,834	5,523	5,837
1555041_a_at	8,022	8,252	8,255	8,546	8,484	9,134
1555042_at	6,377	6,314	6,085	7,284	6,865	6,928
1555043_at	5,335	5,264	5,640	5,736	5,739	6,056
1555044_a_at	6,729	6,692	6,555	7,448	7,238	7,268
1555046_at	4,042	4,148	4,295	3,857	3,870	3,931
1555048_a_at	5,791	6,083	5,806	5,980	5,969	6,089
1555049_at	6,017	6,059	6,443	6,856	6,916	7,004
1555051_at	5,648	5,550	5,733	6,183	6,193	6,289
1555052_a_at	6,470	6,166	5,465	6,557	6,131	6,460
1555053_at	5,886	5,584	5,706	6,304	6,067	6,174
1555055_at	3,586	3,796	3,596	3,639	3,529	3,575
1555056_at	7,307	7,173	7,033	7,816	7,737	7,854
1555057_at	6,039	5,655	5,417	5,239	5,202	5,071
1555058_a_at	8,398	8,692	8,436	8,360	7,993	8,580
1555059_at	5,730	5,793	5,551	6,104	5,897	5,989
1555060_a_at	6,810	6,885	7,126	7,483	7,081	7,156
1555062_s_at	7,110	7,400	7,532	6,848	6,335	7,023
1555063_at	6,764	6,914	6,717	8,002	8,131	8,335
1555065_x_at	6,508	6,031	6,487	7,033	7,100	7,408
1555068_at	4,676	5,490	5,091	4,861	4,754	5,030
1555071_at	4,122	4,079	3,560	3,812	3,843	3,847
1555073_at	3,690	3,831	4,047	3,796	3,790	3,743
1555074_a_at	6,436	5,588	5,850	5,648	5,440	5,788
1555076_at	6,380	6,233	6,518	6,540	6,605	6,359
1555078_at	7,788	7,630	7,650	8,430	8,312	8,560
1555079_at	4,886	4,676	5,046	5,318	5,266	5,156
1555082_a_at	6,693	6,695	6,593	7,415	7,360	7,598
1555083_at	8,394	7,911	8,066	8,696	8,409	8,265
1555084_at	7,160	7,211	7,412	8,022	8,299	8,285
1555085_at	6,145	6,039	6,161	6,176	6,155	6,217
1555086_at	7,721	7,584	7,593	8,130	8,680	8,499
1555088_x_at	8,057	7,977	7,916	8,316	8,816	8,654
1555090_x_at	6,562	6,694	6,620	6,317	6,339	6,354
1555091_at	5,125	5,228	5,147	6,774	6,177	6,584
1555092_at	6,378	6,375	6,457	6,527	6,249	6,555
1555095_at	4,920	5,100	4,742	4,727	4,707	4,606
1555096_at	6,326	6,027	6,337	6,574	6,474	6,418
1555097_a_at	4,586	4,985	4,515	6,324	6,367	5,787
1555098_a_at	6,340	5,935	5,848	6,469	6,241	6,379
1555099_at	5,233	5,306	5,257	5,909	5,803	5,992
1555100_at	5,774	5,326	5,988	7,012	7,110	7,039
1555102_at	3,151	3,430	3,751	3,322	3,335	3,411
1555103_s_at	4,083	3,873	4,481	4,513	4,442	4,532
1555105_a_at	7,617	7,906	7,487	6,791	7,211	6,542
1555106_a_at	5,194	5,488	5,237	4,692	4,601	4,518
1555107_a_at	7,055	7,128	6,917	7,371	7,284	7,651
1555108_at	5,345	5,571	4,946	5,387	5,520	5,585

1555110_a_at	6,170	6,191	6,171	7,128	7,030	7,288
1555111_at	4,226	3,865	3,503	3,476	3,603	3,542
1555112_a_at	8,915	9,336	8,878	6,224	6,353	6,170
1555113_at	4,795	5,339	5,326	5,044	5,212	4,997
1555114_at	6,491	6,352	6,526	6,812	6,392	6,812
1555116_s_at	4,833	4,039	4,038	4,363	4,030	4,221
1555117_at	6,444	6,311	6,249	5,317	5,351	5,682
1555118_at	4,786	5,076	4,969	5,030	5,064	5,020
1555120_at	5,313	5,969	5,229	5,183	5,094	5,020
1555122_at	4,303	4,704	4,283	4,234	4,205	4,133
1555123_at	4,627	4,812	5,157	4,867	4,727	4,896
1555124_at	6,432	6,398	6,799	6,863	7,190	7,288
1555125_at	7,892	8,489	8,147	6,814	6,623	6,132
1555127_at	4,543	4,128	4,757	5,559	5,172	5,662
1555129_at	7,010	6,902	6,918	7,719	7,472	7,698
1555130_at	4,134	4,198	3,828	4,269	4,224	4,125
1555131_a_at	6,204	6,378	6,509	6,337	6,373	6,261
1555132_at	6,995	6,800	6,861	7,321	7,017	7,168
1555133_at	4,640	4,386	4,545	4,963	4,878	4,915
1555135_at	6,302	5,841	6,517	6,748	6,339	6,579
1555136_at	5,580	5,996	5,680	4,735	5,458	4,932
1555137_a_at	5,574	6,517	5,506	4,333	4,220	4,225
1555139_a_at	6,900	6,820	6,612	7,190	6,962	7,170
1555140_a_at	5,769	5,910	5,716	6,347	6,323	6,324
1555141_a_at	3,845	4,682	3,988	3,663	3,603	3,637
1555142_at	4,813	5,460	4,677	4,914	4,750	4,955
1555144_at	5,156	5,202	5,339	4,928	4,959	5,044
1555145_at	5,781	5,613	5,701	6,087	6,176	6,028
1555146_at	4,414	4,509	4,402	4,282	4,366	4,026
1555148_a_at	4,934	5,123	4,684	4,950	4,686	4,901
1555151_s_at	6,151	6,290	6,121	5,457	5,495	5,791
1555153_s_at	5,123	5,106	4,960	5,911	6,003	5,960
1555154_a_at	7,609	7,093	6,809	6,997	6,460	6,840
1555155_at	7,123	6,852	6,758	7,659	7,665	7,664
1555157_at	5,854	5,467	5,667	6,274	5,932	6,258
1555158_at	5,812	5,467	5,651	6,552	6,241	6,663
1555159_at	5,540	5,506	5,436	5,743	5,844	5,794
1555162_at	6,233	6,216	6,121	6,252	5,886	5,784
1555163_at	5,338	5,692	5,402	4,856	4,742	4,847
1555164_at	6,513	6,498	6,684	6,940	6,920	6,972
1555165_a_at	6,289	6,358	6,425	6,580	6,599	6,770
1555166_a_at	3,871	3,914	3,826	4,048	3,976	3,976
1555167_s_at	6,622	7,425	6,553	7,720	7,430	7,525
1555168_a_at	7,115	7,299	7,412	7,607	7,069	7,405
1555169_at	5,274	4,968	5,064	5,639	5,536	5,746
1555171_at	4,300	3,824	4,106	4,871	4,916	4,530
1555173_at	5,078	5,087	4,983	4,984	5,149	5,262
1555174_at	5,786	5,729	5,648	6,459	6,150	6,412
1555175_a_at	4,356	4,880	4,756	5,364	4,977	5,308
1555176_at	4,970	5,031	4,983	5,505	5,604	5,463
1555177_at	4,114	4,363	4,363	4,121	4,109	4,144
1555179_at	4,926	4,856	4,978	4,764	4,818	4,970
1555180_at	5,271	4,815	4,992	5,893	5,229	5,912
1555181_a_at	9,368	9,426	9,494	9,881	9,841	10,081
1555183_at	4,761	4,540	4,805	5,605	5,391	5,546
1555185_x_at	7,202	7,262	7,349	8,129	7,629	7,845

1555186_at	5,238	5,148	5,399	5,480	5,373	5,642
1555187_at	5,232	5,497	5,255	5,437	5,241	5,269
1555188_at	6,746	6,630	6,926	7,721	7,501	7,767
1555189_a_at	6,102	5,924	6,149	6,213	6,206	6,170
1555191_a_at	5,484	5,234	5,257	5,823	5,954	5,831
1555192_at	4,524	4,031	4,052	3,546	3,727	3,501
1555193_a_at	7,106	7,646	6,088	5,284	5,721	5,746
1555194_at	4,875	4,551	4,996	5,474	5,372	5,484
1555195_at	4,286	4,593	4,285	5,424	5,228	5,374
1555196_at	6,540	7,126	6,744	7,140	7,181	7,291
1555197_a_at	6,262	5,993	5,805	5,679	5,437	5,549
1555198_x_at	7,933	7,997	7,614	7,891	7,574	7,767
1555199_at	4,214	4,290	3,843	3,870	4,183	4,089
1555201_a_at	7,841	8,399	8,209	8,083	7,449	7,831
1555202_a_at	7,310	6,972	7,566	7,454	7,351	7,313
1555203_s_at	5,593	5,569	5,684	5,983	5,950	5,952
1555204_at	5,204	5,241	5,125	5,451	5,070	5,191
1555205_at	7,098	7,007	6,817	7,339	7,008	7,106
1555206_at	6,080	6,198	6,254	6,672	6,479	6,545
1555209_at	6,628	6,345	5,987	6,720	7,002	6,740
1555210_at	6,383	6,126	5,920	6,468	6,314	6,380
1555212_at	6,581	6,487	6,420	6,730	6,770	6,714
1555213_a_at	7,277	7,380	7,402	7,960	7,691	8,182
1555214_a_at	3,569	3,546	3,607	5,141	4,768	5,136
1555215_a_at	7,294	7,294	7,297	8,013	7,688	7,790
1555216_a_at	6,890	6,434	6,833	8,477	7,941	8,300
1555217_at	5,727	5,645	5,691	6,181	5,864	6,100
1555219_at	6,666	6,332	6,269	6,861	6,786	6,719
1555220_a_at	6,723	6,769	6,734	6,812	6,012	6,296
1555221_at	5,524	5,357	5,458	5,953	5,856	6,131
1555222_a_at	8,005	8,182	8,103	9,028	8,998	9,306
1555224_at	4,398	4,784	4,724	5,536	5,554	5,573
1555225_at	9,502	8,914	9,914	9,733	8,185	8,806
1555226_s_at	11,258	11,114	10,702	10,161	9,279	10,104
1555227_a_at	4,577	5,191	4,731	4,667	4,695	4,472
1555229_a_at	5,099	4,969	4,729	5,516	5,547	5,366
1555230_a_at	5,449	5,542	5,496	6,310	6,056	6,272
1555231_a_at	6,983	7,067	7,293	7,562	7,636	7,813
1555233_at	5,005	4,694	4,741	5,294	4,924	5,108
1555234_a_at	7,188	7,269	6,950	7,920	7,595	7,834
1555235_s_at	6,431	6,089	5,849	6,972	6,713	6,881
1555236_a_at	6,813	6,590	6,688	7,503	7,212	7,404
1555237_at	6,835	6,983	6,393	7,688	7,594	7,807
1555238_at	9,027	8,846	9,162	9,357	9,498	9,546
1555240_s_at	5,983	5,867	5,932	7,404	7,359	7,900
1555241_at	5,393	5,908	5,285	6,241	6,685	6,861
1555243_x_at	5,185	5,335	5,268	6,068	6,632	6,616
1555244_at	4,454	5,015	4,510	5,613	5,620	5,698
1555245_s_at	6,300	6,484	6,854	6,422	6,580	6,451
1555246_a_at	5,200	4,970	5,422	5,886	5,564	5,741
1555247_a_at	7,428	7,833	7,281	5,514	7,093	5,514
1555248_a_at	6,916	6,408	6,765	7,226	7,005	7,136
1555250_a_at	3,769	3,832	4,160	3,831	3,853	3,878
1555251_a_at	6,332	6,204	6,330	6,643	6,475	6,426
1555252_a_at	7,112	6,814	6,969	7,408	7,247	7,253
1555253_at	3,947	4,360	4,123	4,367	4,410	4,772

1555255_a_at	6,894	6,894	6,770	6,732	6,901	6,824
1555256_at	6,854	6,754	6,760	7,236	6,760	7,253
1555257_a_at	5,299	5,349	4,973	5,567	5,603	5,712
1555259_at	6,645	6,800	6,507	6,225	6,781	6,263
1555261_at	5,564	4,865	5,197	5,267	5,059	5,555
1555262_a_at	7,105	6,870	6,952	6,833	6,798	6,897
1555263_at	4,151	4,097	4,023	3,917	4,126	3,895
1555264_a_at	5,432	5,327	5,822	5,958	6,155	5,927
1555265_at	5,027	4,882	4,885	5,250	5,216	5,932
1555266_a_at	6,715	6,608	6,586	6,684	6,577	6,574
1555267_at	6,546	6,250	6,401	6,807	7,033	6,915
1555268_a_at	7,383	7,300	7,352	7,445	7,246	7,418
1555269_a_at	7,192	7,291	7,066	8,157	8,288	8,632
1555270_a_at	7,773	8,109	7,947	7,877	7,606	8,167
1555271_a_at	6,778	6,831	6,981	7,115	6,782	7,005
1555272_at	7,506	7,720	7,515	6,996	7,218	7,273
1555273_at	5,952	6,215	6,121	6,111	6,204	6,033
1555274_a_at	9,681	9,789	9,697	9,481	9,642	9,568
1555275_a_at	4,749	4,869	4,782	4,799	4,776	4,687
1555277_a_at	6,896	6,560	6,678	7,412	7,059	7,431
1555278_a_at	10,529	10,916	10,007	8,475	8,711	8,335
1555279_at	8,915	9,292	8,296	7,551	7,964	7,807
1555281_x_at	8,949	9,172	8,399	7,578	7,680	7,669
1555282_a_at	7,452	7,410	7,214	7,159	7,010	7,254
1555284_at	4,501	4,642	4,469	4,381	4,327	4,442
1555286_at	6,362	6,513	6,317	6,881	6,837	7,021
1555288_s_at	7,581	7,582	7,904	8,298	8,095	8,448
1555289_at	7,525	7,517	7,496	7,773	7,579	7,608
1555291_at	5,119	4,787	5,182	5,831	5,481	5,593
1555292_at	7,758	7,918	7,936	7,216	6,846	6,538
1555294_a_at	7,830	7,642	7,355	7,433	7,207	7,184
1555298_a_at	5,778	5,246	5,518	5,856	6,056	5,746
1555299_s_at	6,037	5,476	6,058	6,576	6,237	6,253
1555300_a_at	6,874	7,042	6,826	7,365	7,006	7,346
1555301_a_at	6,402	7,093	6,787	6,832	6,279	6,712
1555303_at	5,923	5,406	5,962	5,477	5,685	5,742
1555304_a_at	6,577	6,710	6,261	7,427	7,259	7,514
1555305_at	5,398	5,405	5,681	6,059	5,895	6,081
1555306_a_at	7,573	7,610	7,730	7,960	7,775	8,160
1555307_at	4,266	4,518	3,992	4,243	4,081	4,683
1555308_at	6,093	6,027	6,387	6,400	6,086	6,352
1555309_a_at	6,629	5,892	6,048	6,603	6,573	6,555
1555310_a_at	7,787	7,704	7,952	8,187	8,123	8,214
1555311_at	7,742	7,633	7,656	8,386	7,977	8,447
1555313_a_at	4,353	4,495	4,208	4,404	4,565	4,791
1555314_at	4,492	4,617	4,822	5,234	5,055	5,358
1555315_a_at	4,431	4,836	4,842	4,648	4,577	4,665
1555316_a_at	4,620	4,627	4,688	5,278	5,513	5,549
1555317_at	3,635	3,644	3,539	3,790	3,641	3,695
1555318_at	3,755	3,921	3,459	4,336	4,082	4,216
1555319_at	6,212	6,816	6,819	7,066	7,053	7,277
1555320_a_at	5,447	5,452	5,383	6,208	6,043	6,339
1555321_at	6,770	6,792	6,961	6,739	6,681	6,873
1555322_at	6,772	6,441	6,830	6,560	6,598	6,580
1555323_at	7,451	7,045	7,121	7,921	7,485	7,475
1555324_at	4,641	4,478	4,503	5,002	4,485	4,559

1555325_s_at	7,705	7,584	7,786	5,995	6,363	5,977
1555326_a_at	6,655	6,863	5,780	6,969	8,731	7,735
1555330_at	6,993	7,012	7,076	7,085	7,585	7,225
1555332_at	4,826	4,953	5,308	5,225	4,845	5,160
1555334_s_at	7,867	8,155	7,362	8,081	8,111	8,179
1555335_at	4,479	4,455	4,564	5,306	5,691	5,696
1555336_a_at	5,560	5,382	5,639	6,121	5,952	6,153
1555337_a_at	6,737	6,743	6,758	6,073	5,853	6,149
1555338_s_at	6,906	7,022	7,047	7,228	7,004	7,130
1555339_at	6,118	6,126	6,108	6,666	6,591	6,885
1555340_x_at	5,933	5,971	6,132	6,902	6,247	6,873
1555341_at	3,877	3,930	4,134	4,154	4,400	4,256
1555342_a_at	6,611	6,678	6,787	6,847	6,709	6,747
1555343_at	7,444	7,513	7,434	7,819	7,666	7,744
1555344_at	4,211	4,085	4,353	4,554	4,258	4,411
1555345_at	4,369	4,645	4,765	4,718	4,618	4,543
1555346_at	3,789	4,073	3,961	3,981	4,228	4,218
1555347_at	6,446	6,647	6,686	6,713	6,651	6,513
1555348_at	7,896	7,938	8,127	8,176	8,169	8,425
1555349_a_at	6,093	6,476	6,369	6,956	6,634	7,158
1555350_at	6,939	6,600	7,151	6,800	6,678	6,237
1555351_s_at	8,002	7,734	7,949	7,446	7,517	7,372
1555352_at	5,287	5,365	5,574	6,080	5,967	6,132
1555353_at	6,468	6,165	6,167	6,434	6,667	6,754
1555355_a_at	6,110	6,622	7,779	9,500	8,818	8,814
1555356_a_at	4,584	4,438	4,372	4,999	4,769	4,711
1555357_at	4,007	3,830	4,127	4,422	4,159	4,190
1555358_a_at	5,478	5,182	5,723	5,688	5,158	5,729
1555359_at	8,087	7,994	8,132	8,547	8,460	8,693
1555360_a_at	9,187	8,629	9,158	9,038	8,548	8,955
1555363_s_at	5,344	5,879	5,215	5,461	5,216	5,682
1555364_at	3,855	3,972	3,833	4,124	4,369	4,389
1555365_x_at	3,854	3,966	3,645	4,084	4,474	4,212
1555366_at	5,432	5,646	5,605	5,541	5,391	5,279
1555367_at	3,893	4,078	4,017	4,141	4,118	4,179
1555368_x_at	5,032	4,923	5,120	5,246	5,175	5,623
1555369_at	3,991	4,129	4,099	3,987	4,169	4,107
1555370_a_at	7,714	7,957	7,027	4,452	4,164	4,548
1555371_at	6,925	6,832	6,856	7,256	7,392	7,237
1555372_at	8,722	7,880	8,647	6,089	6,143	6,004
1555373_at	5,038	4,932	4,804	4,702	4,913	4,607
1555374_at	7,999	7,986	8,263	7,998	8,369	8,267
1555375_at	3,887	3,942	3,897	3,778	3,849	3,654
1555377_at	6,216	6,241	6,566	6,323	6,117	6,303
1555378_at	7,334	7,093	7,156	8,003	8,065	8,312
1555379_at	6,218	6,249	6,269	6,919	7,426	7,228
1555380_at	7,113	7,086	6,548	7,402	7,431	7,569
1555382_at	4,106	3,670	3,597	3,557	3,508	3,436
1555383_a_at	4,077	4,018	4,306	4,758	4,477	4,625
1555384_a_at	6,795	6,941	7,458	6,979	6,485	6,719
1555385_at	6,043	5,260	5,500	6,001	5,535	6,008
1555387_at	7,040	7,118	7,072	7,583	6,713	6,963
1555388_s_at	7,499	7,756	7,081	7,001	7,134	7,276
1555390_at	5,991	6,091	5,816	7,197	6,598	6,983
1555391_a_at	6,158	6,237	5,964	6,307	6,307	6,778
1555392_at	5,681	6,139	6,160	5,659	6,196	5,691

1555393_s_at	7,510	7,216	7,552	7,525	7,679	7,758
1555394_at	4,353	4,747	4,295	4,333	4,692	4,550
1555395_at	5,684	5,842	5,917	6,594	6,442	6,737
1555396_s_at	4,836	4,851	4,802	5,655	5,389	5,513
1555397_at	4,665	4,618	4,452	5,252	5,119	5,405
1555399_a_at	8,139	8,075	8,168	8,653	8,732	9,015
1555400_at	4,518	4,318	4,391	4,623	4,624	4,635
1555401_at	5,474	5,486	5,504	5,724	5,411	5,675
1555403_a_at	4,594	4,997	4,928	4,997	4,720	4,817
1555404_a_at	6,806	6,695	6,786	7,816	7,783	7,883
1555405_at	4,468	4,240	4,322	4,815	4,481	4,680
1555407_s_at	6,254	6,351	6,174	6,512	6,370	6,785
1555408_at	6,978	6,817	6,866	6,518	5,771	5,695
1555409_a_at	5,019	4,565	5,211	5,163	5,033	5,361
1555410_at	6,562	6,707	6,834	7,118	7,105	7,361
1555411_a_at	8,516	9,596	8,539	6,542	9,347	7,521
1555412_at	4,744	5,174	4,654	3,782	3,866	4,114
1555413_s_at	5,233	5,552	5,356	5,569	5,542	5,622
1555415_at	7,256	6,715	6,877	7,821	7,684	7,899
1555416_a_at	5,243	5,142	5,263	5,468	5,651	5,580
1555417_a_at	5,341	6,042	6,032	6,584	6,565	6,395
1555419_a_at	8,902	9,421	8,447	7,955	8,742	7,932
1555420_a_at	7,732	7,872	7,852	7,900	7,591	7,727
1555421_at	3,692	4,041	3,827	4,064	4,184	4,188
1555423_at	5,409	5,297	5,407	5,524	5,251	5,572
1555425_x_at	5,539	5,606	5,617	6,317	5,661	5,995
1555426_a_at	8,003	7,864	8,038	7,779	7,625	8,101
1555427_s_at	10,815	10,185	10,412	8,927	10,009	8,876
1555429_at	6,698	6,381	6,685	7,255	7,464	7,646
1555430_at	5,645	5,745	5,789	5,686	5,696	5,466
1555431_a_at	3,978	3,932	3,953	4,413	4,077	4,258
1555433_at	4,875	4,930	5,111	5,832	5,192	5,599
1555434_a_at	6,987	6,962	7,075	7,717	7,390	7,643
1555435_at	3,838	3,988	4,050	4,050	4,121	4,139
1555436_a_at	6,491	6,479	6,793	6,618	6,764	6,530
1555437_at	6,631	6,721	6,672	7,293	7,272	7,483
1555439_at	6,508	6,532	6,073	3,914	4,588	4,074
1555441_at	5,193	4,982	4,977	5,758	5,662	5,636
1555443_at	10,332	10,839	10,400	6,918	6,887	7,188
1555444_a_at	3,557	3,661	3,612	3,825	3,725	3,711
1555445_at	4,993	5,359	5,365	5,757	5,501	5,666
1555446_s_at	8,035	7,890	8,415	8,428	7,855	8,144
1555447_at	7,103	6,972	7,177	7,414	7,303	7,415
1555448_at	5,295	5,436	4,491	4,837	4,920	5,104
1555450_a_at	7,669	6,995	6,693	6,387	6,287	6,289
1555452_at	7,826	7,661	7,879	8,538	8,338	8,551
1555454_at	4,572	5,032	4,782	5,019	4,896	5,161
1555456_at	5,506	5,376	5,604	5,550	5,535	5,794
1555457_at	5,102	5,334	4,893	5,469	5,152	5,341
1555460_a_at	6,994	6,986	6,406	6,514	6,584	6,515
1555461_at	8,210	7,784	7,997	5,982	6,339	5,886
1555462_at	6,004	6,573	6,536	6,293	5,813	5,839
1555463_a_at	6,259	6,017	5,871	5,971	5,987	6,213
1555464_at	3,942	3,949	3,659	4,381	4,469	4,345
1555465_at	6,711	6,782	6,536	7,086	7,040	7,281
1555467_a_at	8,495	8,335	8,392	7,184	7,135	7,346

1555468_at	6,917	6,752	6,881	7,058	6,849	7,202
1555469_a_at	7,675	7,182	7,294	7,123	6,880	6,795
1555470_a_at	8,121	8,072	8,055	8,566	8,663	8,832
1555471_a_at	5,084	5,408	5,029	5,267	5,870	5,677
1555472_at	6,043	5,932	6,196	6,266	6,149	6,242
1555474_at	5,894	5,966	5,889	6,745	6,534	6,538
1555475_x_at	7,519	7,640	7,309	7,928	7,986	7,822
1555476_at	5,255	6,251	6,198	5,267	6,058	5,941
1555478_at	8,312	7,844	7,959	8,794	8,323	8,436
1555480_a_at	7,638	7,532	7,364	7,669	7,460	7,838
1555483_x_at	7,482	7,265	7,383	7,718	7,358	7,636
1555485_s_at	5,088	5,554	5,068	5,315	5,487	5,615
1555486_a_at	7,925	7,989	8,038	8,558	7,676	8,275
1555487_a_at	9,541	9,289	9,017	7,453	7,257	7,216
1555488_at	6,531	6,560	6,570	6,697	6,863	6,830
1555489_at	4,579	4,738	5,185	4,813	4,809	4,722
1555490_s_at	7,608	7,429	7,452	7,736	7,829	7,978
1555491_a_at	7,383	7,524	7,281	7,577	7,523	7,765
1555492_a_at	3,984	3,809	3,923	4,286	4,346	4,457
1555495_a_at	10,454	10,938	10,080	9,222	9,680	8,994
1555497_a_at	6,892	7,065	7,260	7,311	7,198	7,346
1555498_at	5,577	5,072	5,030	4,872	5,033	5,079
1555499_a_at	7,632	7,524	7,677	8,233	8,026	8,161
1555500_s_at	7,519	6,765	7,082	8,220	7,611	8,160
1555501_s_at	8,999	9,322	8,389	8,946	9,311	7,652
1555502_at	5,107	5,056	4,962	5,265	5,136	5,487
1555503_a_at	6,259	6,352	6,489	7,810	7,503	7,762
1555504_at	3,525	3,563	3,764	3,536	3,634	3,605
1555505_a_at	5,917	5,370	5,891	5,895	5,756	6,133
1555509_a_at	6,785	6,642	6,727	7,338	7,157	7,265
1555510_at	3,828	3,996	4,034	4,984	4,117	4,501
1555513_at	3,546	3,803	3,888	3,763	3,621	3,747
1555514_a_at	7,982	8,192	7,961	7,431	7,443	7,521
1555515_a_at	5,636	5,645	5,369	5,989	5,749	5,920
1555516_at	4,268	4,031	4,468	5,078	4,604	4,821
1555517_at	4,256	4,100	3,986	4,370	4,347	4,402
1555519_at	3,788	3,896	3,788	4,292	4,110	4,164
1555520_at	7,012	7,279	6,812	6,077	5,751	6,053
1555522_s_at	9,113	9,063	9,070	9,049	8,696	9,193
1555523_a_at	8,054	8,148	8,427	9,014	8,790	8,788
1555526_a_at	7,463	7,347	6,771	6,317	6,373	6,357
1555527_at	4,114	4,296	4,452	4,165	4,114	4,187
1555529_at	6,675	6,939	6,723	7,448	7,283	7,460
1555533_at	4,627	4,429	4,702	5,802	5,064	5,409
1555535_at	5,243	4,758	4,747	5,626	5,295	5,629
1555536_at	5,685	5,577	5,797	5,767	5,818	5,873
1555538_s_at	4,731	4,609	4,624	5,329	5,118	5,053
1555539_at	6,422	6,399	6,568	7,110	6,783	7,082
1555540_at	5,259	5,443	5,390	6,100	6,236	6,251
1555542_at	6,838	6,688	6,646	7,831	7,322	7,763
1555543_a_at	8,419	8,379	7,914	8,047	8,165	8,204
1555544_a_at	5,725	5,694	6,093	6,099	6,045	6,237
1555545_at	5,839	5,837	5,857	6,197	6,028	6,266
1555546_a_at	7,695	7,339	7,385	8,295	8,099	8,204
1555547_at	4,228	4,144	4,085	4,224	4,595	4,535
1555548_at	6,887	6,861	6,829	7,412	7,343	7,562

1555550_at	7,145	6,934	7,196	7,953	7,920	8,349
1555551_at	3,782	3,825	3,630	3,913	3,777	4,076
1555553_a_at	6,853	6,828	6,874	7,353	7,280	7,583
1555554_at	6,333	6,368	6,051	6,609	6,582	6,829
1555555_at	6,771	6,680	6,661	6,912	6,104	6,327
1555557_a_at	5,670	5,425	5,696	5,777	5,454	5,894
1555558_at	3,852	3,623	3,268	4,562	4,676	4,788
1555559_s_at	6,022	6,080	5,541	5,513	5,326	5,418
1555560_at	5,073	5,357	4,555	3,843	3,874	3,994
1555561_a_at	6,633	7,570	6,386	6,058	5,787	6,448
1555562_a_at	7,472	7,479	6,004	6,082	5,916	6,272
1555564_a_at	4,256	4,696	4,763	5,125	5,172	5,490
1555565_s_at	6,523	6,909	7,040	7,930	7,575	7,882
1555567_s_at	5,797	5,598	5,649	6,009	5,977	6,212
1555568_at	4,788	4,516	4,555	5,629	5,191	5,712
1555569_a_at	6,608	6,705	6,772	7,501	7,418	7,728
1555571_at	4,708	4,626	4,475	4,404	4,629	4,360
1555572_at	4,286	4,011	4,231	4,379	4,234	4,586
1555573_at	4,370	4,855	4,826	4,432	4,544	4,276
1555575_a_at	11,288	11,202	11,286	11,891	11,678	12,136
1555578_at	5,513	5,406	5,197	5,620	5,891	5,636
1555579_s_at	7,891	8,663	8,028	8,859	10,971	9,844
1555580_at	6,711	6,625	6,831	7,856	8,081	8,109
1555581_a_at	4,389	4,289	4,734	4,606	4,551	4,701
1555585_a_at	5,258	4,990	5,571	5,974	5,645	5,979
1555586_at	5,824	5,770	5,627	5,710	5,729	5,714
1555587_at	5,627	5,520	5,765	6,436	6,019	6,276
1555590_a_at	7,807	7,502	7,754	8,195	7,851	7,974
1555591_at	5,485	5,617	5,530	6,089	6,230	6,156
1555594_a_at	5,482	5,312	5,523	6,269	6,020	6,267
1555595_at	4,865	5,013	4,803	5,078	5,023	5,029
1555598_a_at	6,614	6,596	6,035	6,786	6,486	6,825
1555600_s_at	5,914	6,224	6,129	6,616	6,477	6,520
1555601_at	4,211	3,689	4,138	4,109	4,176	4,140
1555602_a_at	5,709	5,238	5,511	5,584	5,443	5,861
1555603_at	6,342	6,348	6,623	6,372	6,196	6,332
1555605_x_at	5,929	6,125	5,925	5,871	5,975	6,016
1555606_a_at	6,026	6,585	6,187	5,438	5,361	5,457
1555608_at	6,729	6,744	7,201	7,397	7,192	6,986
1555609_a_at	6,359	6,530	6,291	6,635	6,537	6,949
1555610_at	4,256	5,401	5,067	3,961	3,758	3,824
1555611_s_at	6,550	6,615	6,687	7,280	7,169	7,576
1555612_s_at	6,625	6,926	6,740	7,148	6,891	7,160
1555613_a_at	5,623	5,663	5,789	6,164	5,556	6,302
1555614_at	6,907	6,961	6,960	7,618	6,886	7,460
1555616_at	6,867	6,869	6,894	6,338	6,488	6,678
1555617_x_at	6,517	6,556	6,690	6,214	6,308	6,536
1555618_s_at	10,202	9,825	9,750	8,886	8,321	8,671
1555620_a_at	8,511	8,365	8,401	8,662	8,238	8,436
1555623_at	3,986	3,818	4,081	7,305	3,931	4,077
1555624_a_at	4,904	4,462	4,800	5,734	5,939	6,108
1555626_a_at	6,422	6,366	6,275	6,730	6,363	6,778
1555627_s_at	7,583	7,169	7,579	8,514	8,376	8,673
1555628_a_at	7,362	7,490	7,434	8,497	8,051	8,673
1555629_at	5,788	5,864	5,806	5,881	5,803	5,978
1555630_a_at	10,060	9,598	10,438	10,952	9,912	10,877

1555631_at	7,254	7,319	7,544	7,946	7,582	7,998
1555632_at	5,676	5,851	5,972	6,175	6,352	6,468
1555634_a_at	6,407	6,499	6,744	6,719	6,794	6,777
1555636_at	4,464	4,816	4,677	5,173	5,024	5,263
1555638_a_at	6,648	6,310	6,462	6,903	6,441	6,452
1555639_a_at	7,307	6,813	6,462	6,063	5,417	5,974
1555640_at	6,559	5,774	6,596	7,328	7,243	7,551
1555643_s_at	5,421	5,376	5,399	6,064	5,856	5,995
1555645_at	7,587	7,331	7,468	8,252	7,849	8,040
1555646_at	4,201	4,083	4,355	4,582	4,493	4,501
1555647_a_at	5,079	5,131	4,998	5,527	5,535	5,656
1555648_at	3,843	3,807	3,778	4,232	4,106	4,198
1555650_at	6,885	6,612	6,584	7,265	7,286	7,354
1555651_at	6,237	6,143	6,084	6,995	5,986	6,307
1555653_at	12,751	12,721	13,192	12,100	12,965	12,224
1555655_at	6,068	6,168	6,410	7,497	6,921	7,365
1555656_at	9,166	9,054	9,118	9,407	9,287	9,606
1555659_a_at	7,011	6,893	7,185	7,753	7,296	7,383
1555661_at	6,034	6,373	6,281	6,478	6,541	6,454
1555662_s_at	5,865	6,208	5,996	6,737	6,681	6,712
1555664_at	6,762	6,685	6,804	6,801	7,130	6,971
1555665_at	6,646	6,385	6,461	7,390	7,582	7,854
1555666_at	5,682	5,787	5,823	6,142	5,992	6,047
1555668_a_at	4,552	4,486	4,511	5,146	5,004	4,962
1555671_at	5,274	5,638	5,278	5,830	5,724	5,784
1555673_at	6,151	5,500	5,567	11,388	10,639	12,158
1555675_at	4,875	5,205	5,216	5,307	5,411	5,270
1555676_s_at	6,501	6,183	6,241	6,634	6,580	7,026
1555677_s_at	6,404	6,897	6,327	6,066	6,042	6,275
1555678_at	6,827	6,838	6,380	7,402	7,029	7,333
1555679_a_at	9,277	9,512	9,389	7,732	7,262	7,225
1555680_a_at	7,668	7,285	7,171	8,352	7,819	8,038
1555681_at	7,181	7,147	7,604	7,759	7,716	7,567
1555682_at	7,633	7,316	7,315	8,349	7,879	7,964
1555685_at	5,348	5,432	5,452	5,530	5,795	5,688
1555687_a_at	3,847	3,925	4,161	3,973	4,121	4,089
1555688_s_at	6,877	6,744	7,168	7,849	7,526	7,616
1555689_at	3,931	3,786	3,773	4,065	4,065	4,010
1555691_a_at	6,594	6,360	6,565	6,828	6,854	7,008
1555692_at	5,415	5,015	5,379	5,443	5,329	5,358
1555694_a_at	6,978	6,981	7,208	7,582	7,808	7,836
1555695_a_at	7,335	7,229	7,489	7,512	7,467	7,816
1555697_at	8,843	8,602	8,817	9,472	8,663	8,942
1555702_a_at	7,231	7,299	7,317	7,417	7,289	7,599
1555703_at	5,494	5,244	5,353	5,609	5,780	5,699
1555704_at	6,717	6,815	6,938	7,700	7,712	7,858
1555705_a_at	8,878	8,776	8,763	8,075	7,375	7,720
1555707_at	6,476	6,551	6,393	6,548	6,968	6,791
1555708_a_at	5,137	5,137	5,136	5,122	5,159	5,349
1555710_at	4,197	3,944	3,949	4,367	4,301	4,518
1555711_x_at	4,437	4,656	4,401	5,158	5,005	5,105
1555713_at	7,580	7,438	7,408	8,071	7,759	8,121
1555715_a_at	7,510	7,517	7,607	7,455	7,369	7,468
1555716_a_at	6,056	5,830	5,714	5,034	4,976	5,204
1555717_at	3,489	3,553	3,653	3,533	3,686	3,608
1555718_x_at	3,958	3,741	3,626	3,752	3,846	3,814

1555719_a_at	4,540	4,808	4,192	4,834	4,873	5,383
1555722_at	5,242	5,439	5,570	5,790	5,569	5,624
1555723_at	5,689	5,397	5,641	5,780	5,935	5,897
1555724_s_at	7,065	6,756	6,957	6,873	7,557	8,861
1555725_a_at	6,249	6,011	5,837	6,721	6,561	6,518
1555726_at	4,033	4,949	4,730	5,123	5,157	5,229
1555727_at	4,950	4,964	5,257	4,948	5,256	4,875
1555728_a_at	4,604	4,840	4,970	6,175	5,464	5,810
1555729_a_at	6,368	6,086	6,412	6,736	6,776	6,736
1555730_a_at	10,775	10,272	10,869	10,724	9,935	11,107
1555731_a_at	4,059	4,686	3,780	3,918	4,171	3,814
1555733_s_at	3,822	3,528	3,777	4,197	4,007	4,147
1555734_x_at	4,887	4,964	4,858	5,661	5,668	5,805
1555735_a_at	7,554	7,070	7,040	6,501	6,138	6,680
1555736_a_at	7,645	7,378	7,437	8,177	7,450	7,886
1555737_a_at	6,099	5,996	6,330	7,142	6,735	6,933
1555738_at	6,750	6,682	6,782	7,230	6,861	7,148
1555740_a_at	5,353	5,272	5,797	6,064	5,740	6,099
1555741_at	6,038	5,946	5,935	6,340	5,803	6,035
1555742_at	7,245	6,909	7,465	7,023	6,593	6,641
1555743_s_at	9,639	9,755	10,072	9,780	9,571	9,951
1555745_a_at	6,125	6,172	5,882	7,051	6,994	6,969
1555746_at	6,606	6,160	6,453	7,926	8,174	8,233
1555748_x_at	6,456	6,316	6,467	8,057	8,208	8,510
1555749_at	5,354	4,975	5,759	5,801	5,558	5,614
1555751_a_at	8,420	8,320	8,526	8,860	8,191	8,930
1555752_at	6,207	5,940	6,062	7,259	6,436	6,768
1555753_x_at	6,423	6,361	6,400	6,779	6,087	6,109
1555754_s_at	8,574	8,358	8,548	9,877	9,642	9,845
1555755_at	4,309	4,518	4,590	4,787	4,461	4,679
1555756_a_at	3,499	3,498	3,740	3,687	3,585	3,647
1555757_at	6,980	6,732	7,009	7,620	7,302	7,518
1555758_a_at	9,201	9,561	8,842	9,189	8,569	8,587
1555759_a_at	7,450	7,435	7,548	7,931	7,524	7,785
1555760_a_at	10,435	10,324	10,595	8,869	8,658	8,821
1555761_x_at	8,382	8,616	8,014	7,887	7,784	8,141
1555762_s_at	10,938	10,922	11,035	8,237	8,374	7,715
1555763_x_at	8,545	7,771	8,090	8,487	8,411	7,911
1555764_s_at	11,098	10,795	11,224	11,688	11,172	11,973
1555765_a_at	7,337	7,488	6,999	6,221	6,092	6,133
1555766_a_at	5,502	5,420	5,208	5,804	5,487	5,697
1555772_a_at	9,890	9,255	9,747	7,759	6,352	7,048
1555773_at	6,542	6,343	6,438	6,638	6,317	6,549
1555774_at	6,098	5,529	6,042	6,437	6,339	6,226
1555775_a_at	5,894	5,983	6,022	5,979	5,697	6,018
1555777_at	4,287	4,426	4,620	4,984	4,931	5,066
1555778_a_at	5,182	5,156	4,772	5,368	5,614	6,876
1555779_a_at	9,104	9,170	9,199	9,786	9,433	9,818
1555780_a_at	10,561	10,591	10,170	10,032	9,066	9,946
1555781_at	6,964	7,027	7,102	7,972	6,685	7,290
1555783_x_at	7,490	7,490	7,648	8,397	7,248	7,911
1555784_s_at	6,722	6,693	6,817	7,839	7,643	7,985
1555785_a_at	5,072	5,376	4,895	5,098	5,189	4,550
1555786_s_at	9,905	9,620	9,700	11,911	11,267	11,012
1555787_at	5,003	4,910	4,898	5,391	5,314	5,458
1555788_a_at	6,509	6,860	6,690	6,791	6,540	6,948

1555789_s_at	8,528	8,226	8,636	8,014	7,143	7,876
1555790_a_at	9,251	9,540	9,044	9,784	9,667	9,079
1555792_a_at	7,816	7,118	7,446	8,796	8,524	8,784
1555793_a_at	9,481	9,424	8,785	6,940	6,841	6,795
1555794_at	3,983	3,802	3,691	3,869	3,648	3,866
1555797_a_at	9,182	9,240	8,911	9,914	9,190	10,029
1555798_at	4,099	3,783	3,814	4,253	4,418	4,614
1555799_at	5,359	5,637	5,415	5,758	5,536	5,765
1555800_at	4,322	4,906	4,346	4,137	4,151	4,152
1555801_s_at	4,100	4,589	4,156	4,094	3,976	3,899
1555803_a_at	8,662	8,377	8,450	7,768	7,629	7,418
1555804_a_at	5,675	5,638	5,795	6,167	6,034	6,095
1555807_a_at	6,589	6,616	6,579	6,606	6,544	6,650
1555808_a_at	6,758	6,993	6,798	7,203	7,026	7,371
1555809_at	6,799	6,961	6,777	7,691	7,280	7,540
1555811_at	7,956	8,045	7,758	8,611	8,558	8,859
1555812_a_at	6,382	6,295	6,659	7,864	8,106	7,964
1555814_a_at	9,536	9,376	9,327	9,089	8,669	9,774
1555815_a_at	7,927	7,380	7,655	7,729	7,471	7,821
1555816_at	3,964	4,195	4,239	4,192	4,355	4,603
1555817_s_at	4,178	4,405	4,417	3,962	3,938	4,056
1555819_s_at	6,872	7,237	7,297	7,368	7,318	7,736
1555820_a_at	7,134	6,953	7,107	7,474	7,268	7,331
1555821_a_at	6,591	6,158	6,514	6,977	6,604	6,987
1555822_at	6,430	6,160	6,610	7,318	7,228	7,604
1555823_at	10,996	10,925	10,938	10,810	10,751	11,033
1555824_a_at	8,894	8,557	9,079	8,325	8,651	8,725
1555826_at	8,693	9,518	10,004	8,834	8,221	8,317
1555827_at	6,117	7,743	6,412	6,191	7,946	5,805
1555829_at	5,165	4,803	4,155	4,257	4,117	4,244
1555830_s_at	5,179	5,279	4,714	5,429	4,898	5,471
1555831_s_at	8,786	8,439	8,606	8,495	7,700	8,752
1555832_s_at	8,771	9,834	9,567	10,648	11,608	11,274
1555833_a_at	9,302	9,084	9,397	8,498	8,443	9,020
1555834_at	6,123	6,960	6,714	6,469	6,532	6,444
1555836_at	5,304	4,862	5,155	4,874	5,167	4,784
1555837_s_at	13,168	12,958	12,796	11,908	12,523	11,421
1555839_a_at	4,387	4,859	4,439	4,715	4,312	4,465
1555841_at	10,043	9,831	9,905	9,934	9,878	9,655
1555842_at	8,301	7,966	8,213	7,643	7,747	7,839
1555843_at	7,830	8,048	8,450	7,867	7,855	7,684
1555844_s_at	13,218	13,084	13,251	12,550	12,512	12,154
1555845_at	10,195	10,036	10,261	7,217	7,513	7,152
1555846_a_at	8,931	8,689	8,916	6,560	6,719	6,335
1555847_a_at	7,993	7,746	8,062	9,443	9,867	10,099
1555848_at	7,734	8,104	7,877	7,459	7,602	7,574
1555851_s_at	12,538	12,314	12,500	12,096	11,773	12,330
1555852_at	5,189	4,943	4,726	6,525	5,373	5,356
1555853_at	6,762	6,911	6,959	7,805	8,159	8,000
1555854_at	4,056	4,672	4,724	7,678	8,345	7,632
1555855_at	6,551	6,267	6,434	6,792	6,638	7,123
1555856_s_at	7,019	7,121	7,594	8,450	8,750	8,953
1555858_at	8,134	7,845	7,184	6,074	6,178	5,724
1555860_x_at	8,520	8,379	7,559	6,629	6,755	6,254
1555862_s_at	5,292	5,656	5,892	6,069	7,092	7,589
1555864_s_at	11,862	12,106	11,872	11,441	11,213	11,383

1555865_at	6,208	6,257	6,379	6,821	5,962	6,624
1555866_a_at	8,463	8,376	8,389	8,208	8,336	8,452
1555867_at	10,070	9,571	9,862	4,850	4,811	5,073
1555868_at	5,312	5,255	5,630	6,318	6,411	6,405
1555869_a_at	4,214	4,130	4,154	4,556	4,523	4,415
1555870_at	7,205	7,020	7,351	7,155	7,499	7,395
1555871_at	6,915	6,558	6,870	6,891	6,953	7,189
1555872_a_at	8,524	7,924	8,974	8,423	7,171	7,239
1555874_x_at	8,443	8,033	8,855	8,515	7,708	7,974
1555875_at	7,096	6,993	7,115	7,137	7,961	6,963
1555876_at	6,900	6,462	6,648	7,680	7,824	7,784
1555878_at	9,423	8,249	7,728	5,564	7,513	7,019
1555880_at	5,608	5,530	5,370	6,299	6,766	6,689
1555881_s_at	8,979	8,588	8,922	8,881	8,452	8,739
1555882_at	8,168	7,769	7,026	4,836	4,963	5,021
1555883_s_at	8,424	8,462	8,244	6,883	7,250	6,812
1555884_at	4,117	4,497	4,059	3,554	3,745	3,656
1555886_at	8,147	8,860	8,269	9,206	8,521	8,947
1555888_at	6,682	7,448	6,592	5,863	5,897	5,904
1555889_a_at	10,419	10,865	11,336	12,392	11,938	12,396
1555890_at	4,338	5,424	4,504	4,760	4,581	4,872
1555892_s_at	5,957	6,008	5,915	5,353	5,311	5,282
1555893_at	4,920	4,506	5,216	4,681	4,402	4,763
1555894_s_at	5,756	6,060	6,702	6,827	6,994	7,140
1555895_at	7,969	7,525	8,478	7,464	7,529	7,520
1555896_a_at	8,684	8,625	8,947	9,122	9,100	9,430
1555897_at	6,049	6,089	6,176	6,112	6,147	5,983
1555898_at	4,671	4,885	4,997	5,193	5,458	5,276
1555900_at	8,117	7,586	8,036	7,502	7,118	7,148
1555902_at	7,329	7,061	7,213	7,599	6,594	7,303
1555904_at	6,191	6,055	6,017	6,230	6,390	6,423
1555905_a_at	6,282	6,926	5,983	6,292	5,507	6,168
1555906_s_at	8,010	8,240	8,210	8,579	8,162	8,646
1555907_at	7,056	6,972	7,003	8,419	7,610	8,285
1555908_at	5,703	6,332	6,360	7,747	7,155	7,846
1555910_at	8,495	8,981	8,342	8,819	8,890	8,374
1555912_at	7,118	7,742	6,656	6,236	6,127	6,264
1555913_at	9,242	8,721	9,209	7,022	7,124	7,466
1555914_a_at	5,877	5,925	5,636	6,908	6,825	6,921
1555916_at	9,311	9,218	9,712	9,344	8,482	8,631
1555920_at	10,117	10,165	9,556	6,235	7,170	6,931
1555922_at	8,505	8,351	8,409	8,611	8,581	8,668
1555923_a_at	7,439	7,209	7,357	7,453	6,829	7,348
1555925_at	4,339	3,955	3,745	4,295	4,388	4,447
1555926_a_at	4,348	4,096	4,507	4,641	4,669	4,391
1555928_at	4,035	3,924	3,993	3,870	4,349	3,832
1555929_s_at	6,521	4,874	5,637	3,937	5,983	4,516
1555931_at	6,612	7,036	6,765	7,141	6,894	6,928
1555932_at	3,847	3,761	3,703	3,764	3,835	3,954
1555933_at	8,386	8,099	8,158	8,722	8,422	8,554
1555935_s_at	9,308	8,698	9,167	6,939	6,854	7,023
1555938_x_at	6,218	6,458	5,660	6,017	8,961	5,581
1555939_at	3,273	3,430	3,477	3,484	3,556	3,554
1555940_a_at	4,932	4,985	4,886	5,183	5,152	5,351
1555942_a_at	6,399	6,215	6,077	6,750	6,841	7,015
1555943_at	7,018	6,801	7,307	7,539	6,941	7,529

1555944_at	7,472	7,638	7,732	7,387	7,095	6,856
1555945_s_at	8,974	8,944	9,574	10,251	10,453	10,742
1555947_at	6,101	5,270	5,573	7,011	7,324	7,549
1555948_s_at	8,235	8,180	8,609	9,176	9,562	9,743
1555950_a_at	9,391	9,782	9,239	6,956	8,068	7,190
1555952_at	8,165	7,909	8,751	8,460	7,924	8,349
1555953_at	8,171	7,830	8,284	7,746	7,830	7,907
1555958_at	6,026	5,720	5,937	6,150	6,074	5,812
1555960_at	7,217	6,899	7,227	6,827	6,865	6,957
1555961_a_at	13,493	13,628	13,640	13,388	13,465	13,627
1555962_at	7,260	7,847	8,012	7,520	7,855	8,017
1555963_x_at	6,871	7,388	7,436	7,234	7,540	7,803
1555964_at	6,679	6,764	7,352	7,336	7,482	7,702
1555967_at	6,953	7,064	7,087	8,997	9,569	9,413
1555968_a_at	5,567	5,937	5,759	6,460	6,650	6,471
1555970_at	7,026	7,165	7,090	7,574	7,280	7,785
1555971_s_at	11,333	11,371	11,465	9,870	9,978	9,572
1555972_s_at	5,075	4,640	4,782	4,210	4,197	4,095
1555973_at	6,410	6,479	6,639	6,119	6,018	6,262
1555974_a_at	7,344	7,213	7,659	6,704	6,775	6,776
1555976_s_at	7,421	7,922	7,448	7,832	7,216	7,519
1555977_at	7,571	6,879	6,873	6,065	6,027	6,128
1555978_s_at	6,809	5,937	5,628	5,549	5,058	5,636
1555979_at	5,722	6,133	5,632	6,943	7,201	7,299
1555980_a_at	7,297	7,126	7,257	8,230	8,149	8,591
1555981_at	6,913	6,270	6,630	6,627	6,676	6,770
1555982_at	6,373	6,481	5,875	6,307	6,598	6,191
1555985_at	6,047	5,837	5,786	6,694	6,404	6,513
1555988_a_at	6,857	6,757	6,788	7,071	7,230	7,502
1555989_at	8,188	8,660	7,868	6,498	6,054	6,322
1555990_at	5,845	6,140	5,893	5,670	5,568	5,768
1555991_s_at	4,357	4,689	4,700	4,811	4,754	4,794
1555993_at	6,302	6,662	6,466	6,065	6,113	6,074
1555994_at	7,352	6,677	7,077	7,878	7,789	8,135
1555995_a_at	6,242	5,676	6,117	6,280	5,890	6,139
1555996_s_at	6,872	6,590	6,106	4,784	5,271	4,715
1555997_s_at	4,955	5,124	5,319	5,060	5,154	5,095
1555998_at	8,221	7,918	7,653	8,168	8,099	8,098
1556000_s_at	9,224	9,032	9,113	8,664	9,022	8,531
1556001_at	8,872	8,693	8,824	9,038	8,893	9,089
1556003_a_at	6,512	6,428	6,617	6,466	6,592	6,629
1556004_at	5,995	5,508	5,319	5,706	5,676	5,685
1556006_s_at	6,375	6,828	6,365	5,279	8,164	5,206
1556007_s_at	8,119	7,551	7,521	5,049	8,073	5,808
1556008_a_at	5,647	5,134	5,853	4,873	4,633	4,971
1556009_at	8,148	8,086	7,997	7,259	6,671	7,387
1556012_at	9,809	9,402	9,686	7,893	7,925	8,005
1556014_at	7,888	7,710	7,596	8,675	8,715	8,886
1556015_a_at	7,823	8,129	7,900	7,637	7,358	7,493
1556017_at	6,719	6,628	6,975	6,737	6,986	7,287
1556019_at	5,344	5,607	4,989	5,665	5,535	5,431
1556021_at	7,388	6,972	6,818	5,348	5,076	4,742
1556024_at	7,902	7,864	8,097	8,252	8,261	8,487
1556026_at	3,749	3,666	3,779	3,812	4,011	3,904
1556029_s_at	6,095	7,010	6,520	6,259	5,990	6,269
1556030_at	7,649	7,617	7,599	8,069	7,954	8,081

1556033_at	8,016	7,758	7,734	7,207	7,062	6,992
1556034_s_at	7,645	7,830	7,569	8,567	8,570	8,675
1556035_s_at	8,542	8,705	8,564	5,992	8,900	6,707
1556037_s_at	8,702	7,804	8,149	10,410	11,011	11,348
1556038_at	6,390	5,991	6,194	6,396	6,405	6,491
1556039_s_at	7,680	7,285	7,470	6,698	6,813	6,732
1556042_s_at	8,955	8,476	8,954	8,574	8,450	8,767
1556043_a_at	6,112	6,303	6,130	5,858	6,265	6,069
1556046_a_at	9,877	10,134	9,598	9,040	9,282	9,070
1556047_s_at	9,345	9,067	9,459	7,128	7,802	7,703
1556048_at	6,837	6,946	6,882	7,053	6,680	6,729
1556049_at	8,323	7,646	7,807	6,805	8,140	7,038
1556051_a_at	8,845	9,075	8,745	7,669	7,781	7,581
1556053_at	8,691	8,492	8,910	7,452	8,146	7,465
1556054_at	7,145	4,855	5,532	4,138	4,325	4,173
1556055_at	6,609	6,288	6,195	6,105	6,312	6,013
1556056_at	8,109	8,077	8,334	8,256	8,328	8,176
1556057_s_at	5,790	5,735	5,544	5,864	5,672	5,878
1556058_s_at	5,434	5,534	5,734	6,513	6,396	6,463
1556059_s_at	8,352	8,112	8,254	7,198	8,007	7,462
1556060_a_at	10,078	9,351	9,806	8,786	10,161	8,944
1556061_at	7,608	7,658	7,756	8,402	7,489	7,936
1556062_at	4,160	3,906	3,879	3,545	3,789	3,581
1556063_s_at	6,003	5,788	6,001	6,179	6,269	5,960
1556064_at	7,451	7,664	7,711	6,673	6,122	6,696
1556065_at	4,427	4,406	4,582	4,308	4,348	4,401
1556066_at	7,994	7,609	8,010	7,548	6,977	7,581
1556067_a_at	7,589	7,552	7,493	7,326	6,990	7,512
1556069_s_at	6,037	6,086	6,058	5,919	5,946	5,871
1556072_at	4,970	4,822	4,695	5,187	5,108	5,354
1556075_at	8,331	8,164	8,726	9,374	9,013	9,218
1556078_at	5,941	5,871	5,726	6,299	6,132	6,203
1556081_at	4,800	4,541	5,079	4,165	4,484	4,303
1556082_a_at	6,684	6,267	6,363	6,475	6,597	6,065
1556084_at	5,211	5,479	5,438	5,376	5,105	5,314
1556086_at	4,655	4,805	5,111	4,945	4,930	4,964
1556088_at	7,558	6,639	6,505	4,486	5,208	4,774
1556090_at	6,272	6,003	6,195	6,255	6,157	6,377
1556092_s_at	6,248	6,134	5,993	6,529	6,340	6,075
1556095_at	3,626	4,118	3,873	3,572	3,594	3,613
1556096_s_at	4,265	4,282	4,311	4,430	4,227	4,480
1556097_at	6,823	6,525	6,243	3,798	3,665	3,653
1556099_at	3,702	3,978	3,746	5,144	4,861	5,238
1556102_x_at	8,892	8,856	8,919	8,851	8,640	8,759
1556103_at	8,426	8,794	8,645	7,900	7,936	8,239
1556105_at	5,518	5,937	5,788	5,593	5,991	5,284
1556107_at	6,434	6,433	6,584	6,920	6,602	6,750
1556110_at	5,441	5,137	5,080	5,037	5,314	5,254
1556111_s_at	5,868	5,970	6,251	6,265	6,436	6,278
1556113_at	6,993	7,415	7,531	8,095	8,647	8,217
1556114_a_at	6,249	6,231	6,186	7,255	7,960	7,538
1556116_s_at	6,675	6,338	6,551	7,460	7,318	7,686
1556117_at	5,502	6,165	5,632	6,344	5,796	5,961
1556121_at	10,296	10,335	10,024	6,789	7,555	6,270
1556122_at	5,445	5,736	5,684	6,205	6,130	6,474
1556123_a_at	7,809	7,759	7,777	6,859	6,508	7,103

1556125_at	4,574	3,871	4,082	3,934	4,045	3,904
1556126_s_at	7,956	7,050	7,711	6,253	7,319	6,503
1556127_at	6,958	7,168	6,968	7,583	7,579	7,687
1556128_a_at	7,901	8,002	8,352	8,289	8,024	8,135
1556129_at	9,311	8,890	9,227	8,657	8,370	8,549
1556131_s_at	7,816	7,640	7,905	8,408	8,119	8,263
1556133_s_at	4,109	4,288	4,217	4,154	4,361	4,412
1556134_a_at	4,900	4,888	4,781	6,490	6,511	6,265
1556136_at	5,328	5,758	5,735	5,958	5,868	5,932
1556138_a_at	6,896	6,598	6,929	7,893	7,814	8,230
1556144_at	5,604	5,976	5,544	5,276	5,652	5,203
1556145_a_at	6,187	5,464	5,821	6,893	7,336	7,231
1556147_at	3,716	3,429	3,549	3,762	3,756	3,762
1556148_s_at	5,357	5,173	5,523	6,319	6,451	6,013
1556149_at	7,227	7,083	7,262	7,994	7,920	8,180
1556151_at	10,750	11,187	10,655	11,254	11,880	11,378
1556153_s_at	5,554	5,438	5,508	5,722	5,600	5,509
1556154_a_at	6,489	6,188	6,457	6,916	6,700	7,202
1556155_at	5,599	5,475	5,604	5,975	5,964	6,290
1556156_at	5,367	5,165	5,212	5,279	5,271	5,393
1556158_at	4,322	4,172	4,311	4,247	4,358	5,430
1556159_at	4,392	4,034	4,398	4,544	4,360	4,362
1556160_a_at	5,107	5,301	5,449	5,334	5,491	5,378
1556161_a_at	6,583	6,280	6,341	6,881	6,685	6,851
1556162_at	7,374	7,227	7,503	8,194	7,879	8,167
1556163_a_at	7,993	8,007	8,001	8,510	8,117	8,422
1556165_at	7,865	7,994	8,109	8,426	8,634	8,452
1556166_x_at	5,184	5,620	5,102	5,826	6,053	6,055
1556167_at	5,436	5,565	5,426	5,263	4,798	5,065
1556168_s_at	6,104	6,131	6,021	6,489	5,825	6,219
1556170_at	3,474	3,526	3,679	3,568	3,422	3,520
1556171_a_at	5,569	5,569	5,774	6,399	6,388	6,530
1556172_at	7,153	6,678	6,934	6,258	6,136	6,400
1556173_a_at	7,739	7,342	7,882	6,822	7,252	6,911
1556175_at	4,745	4,718	4,607	4,440	4,396	4,229
1556176_at	5,495	5,612	5,914	6,163	6,300	6,370
1556178_x_at	6,436	6,609	6,616	7,028	6,894	7,011
1556180_at	6,385	5,988	6,241	6,513	5,984	6,217
1556181_at	6,632	6,450	6,771	6,930	6,743	6,972
1556182_x_at	6,777	6,793	7,164	7,072	7,010	6,997
1556183_at	7,163	7,641	6,884	7,022	7,292	6,727
1556185_a_at	3,717	3,649	3,808	3,839	3,860	3,764
1556186_s_at	7,298	6,837	7,410	7,173	6,700	7,812
1556187_at	3,980	4,835	3,700	5,072	5,451	5,603
1556188_a_at	7,071	7,083	6,656	7,275	6,949	7,157
1556190_s_at	4,434	4,306	4,933	7,154	6,724	6,876
1556192_x_at	4,737	4,953	4,640	5,614	5,866	5,805
1556194_a_at	4,051	4,738	4,476	4,736	5,122	4,741
1556195_a_at	6,842	6,965	6,856	7,101	6,997	7,214
1556199_a_at	6,099	6,194	6,157	6,589	6,109	6,174
1556200_a_at	5,819	6,051	5,797	9,095	8,548	7,692
1556201_at	5,707	5,594	5,800	6,063	5,584	5,768
1556202_at	7,149	7,087	7,593	6,129	6,266	5,884
1556203_a_at	7,794	7,933	8,216	5,895	7,406	6,404
1556204_a_at	4,495	4,018	4,251	4,341	4,187	4,185
1556205_at	8,006	8,088	8,127	8,095	7,949	7,687

1556206_at	6,618	6,340	6,513	7,250	7,057	7,232
1556207_a_at	3,432	3,616	3,427	3,653	3,530	3,547
1556209_at	4,414	4,656	4,609	4,700	4,893	4,950
1556210_at	4,923	4,068	4,566	4,265	4,355	4,238
1556211_a_at	7,244	6,142	7,084	4,903	5,679	5,064
1556212_x_at	4,563	4,285	4,705	3,942	4,269	4,130
1556213_a_at	6,229	6,550	6,413	6,156	6,080	6,008
1556216_s_at	4,601	4,688	4,509	5,440	4,841	4,766
1556220_at	4,577	4,273	4,036	4,907	4,826	4,720
1556221_a_at	6,973	6,695	6,765	5,097	5,412	5,485
1556222_at	4,588	4,408	4,691	4,338	4,449	4,513
1556224_a_at	5,890	6,162	5,947	6,926	6,781	7,254
1556226_at	4,722	4,406	4,576	4,278	4,602	4,446
1556227_at	6,561	6,348	6,381	6,689	6,983	6,593
1556228_a_at	8,154	7,847	7,902	6,949	8,171	6,702
1556229_at	6,544	6,227	6,336	6,238	6,175	6,443
1556231_a_at	5,225	5,063	5,333	6,485	6,625	6,743
1556232_at	6,726	6,929	6,870	8,257	8,175	8,144
1556233_s_at	5,673	5,905	5,491	6,064	5,803	6,195
1556235_at	5,137	5,162	5,341	5,699	5,476	5,620
1556236_at	7,018	6,657	6,863	6,045	5,981	5,871
1556238_at	3,806	3,693	3,825	3,556	3,591	3,599
1556239_a_at	5,557	5,851	5,539	4,955	5,557	5,573
1556240_at	5,936	5,801	6,016	6,678	6,523	6,499
1556242_a_at	7,339	6,816	7,614	8,661	8,138	8,516
1556244_s_at	5,021	4,689	4,891	5,954	5,521	6,397
1556247_a_at	7,024	7,334	7,097	7,547	7,590	7,584
1556248_at	8,028	8,015	8,162	8,361	8,694	8,723
1556249_a_at	7,323	7,545	7,555	8,437	8,144	8,214
1556250_at	5,476	5,724	5,639	6,382	5,886	5,609
1556253_s_at	4,646	4,459	4,864	4,744	4,816	4,926
1556255_a_at	8,313	8,237	8,329	8,211	8,330	8,400
1556256_a_at	5,939	6,547	6,298	6,713	6,969	7,033
1556257_at	3,855	4,044	3,764	4,076	4,056	3,863
1556259_at	5,189	4,958	4,926	5,627	5,497	5,554
1556261_a_at	6,584	6,641	6,258	5,763	5,418	5,481
1556262_at	5,764	5,794	5,714	5,689	5,708	5,741
1556263_s_at	4,817	4,243	4,355	5,627	5,443	5,602
1556265_at	5,946	5,717	5,847	6,261	5,920	6,200
1556266_a_at	6,868	6,996	6,955	7,493	7,634	7,699
1556267_at	7,634	7,229	7,297	7,382	7,136	7,159
1556269_at	7,575	7,763	8,115	7,701	7,311	7,484
1556271_at	6,159	6,118	6,275	5,840	5,868	5,853
1556272_a_at	4,803	4,973	5,087	5,601	5,480	5,486
1556274_at	7,587	7,357	7,599	8,524	8,092	8,168
1556277_a_at	7,811	7,331	7,543	6,158	7,308	6,615
1556279_at	6,052	5,968	6,038	5,921	6,127	6,377
1556281_at	6,767	6,823	6,871	7,319	7,398	7,386
1556282_at	3,731	3,767	3,702	3,676	3,695	3,709
1556283_s_at	8,517	8,375	8,840	9,703	9,236	9,230
1556284_at	4,964	5,320	5,210	6,843	6,117	6,982
1556285_s_at	9,192	9,520	9,215	9,767	9,252	9,906
1556287_a_at	4,890	4,421	5,103	5,522	5,552	5,722
1556288_at	4,543	4,424	4,573	4,894	4,598	4,812
1556289_at	4,836	5,061	5,073	5,370	5,471	5,471
1556291_at	9,050	8,719	8,975	9,286	9,266	9,412

1556292_s_at	7,176	7,232	7,403	7,781	7,549	7,638
1556294_at	5,471	5,147	5,514	5,932	5,580	5,467
1556296_at	8,515	8,337	8,172	8,596	8,435	8,675
1556297_a_at	4,666	4,883	4,984	5,181	4,931	5,098
1556299_s_at	6,289	6,404	6,421	6,801	6,681	7,038
1556300_s_at	3,927	3,849	4,099	3,647	3,399	3,594
1556301_at	6,189	5,879	6,669	5,513	4,959	5,378
1556304_s_at	6,588	6,560	7,047	7,508	7,620	7,598
1556306_at	7,310	7,563	7,260	5,171	7,516	5,669
1556308_at	8,934	9,004	9,414	8,385	8,118	8,492
1556309_s_at	7,048	7,037	7,135	7,671	7,235	7,518
1556312_at	6,084	6,215	5,984	6,870	6,883	7,124
1556314_a_at	5,225	5,124	5,344	5,731	5,606	5,583
1556316_s_at	7,386	7,972	8,380	7,425	7,771	7,187
1556318_s_at	5,123	4,365	5,095	3,502	3,861	3,699
1556319_at	5,147	5,532	5,230	4,981	5,149	4,801
1556320_at	8,227	7,857	8,066	8,603	8,550	8,481
1556321_a_at	6,733	6,642	6,875	6,048	6,000	5,937
1556322_a_at	6,812	6,290	6,635	6,442	6,259	6,440
1556323_at	5,200	5,477	5,448	6,186	5,976	6,154
1556325_at	3,923	4,491	3,635	3,760	3,538	3,886
1556327_a_at	5,752	5,556	5,461	6,060	6,225	6,169
1556328_at	3,309	3,523	3,500	3,349	3,489	3,340
1556329_a_at	3,727	3,629	4,484	3,814	3,704	3,838
1556331_a_at	4,379	4,384	4,412	4,188	4,429	4,282
1556332_at	5,837	6,141	5,806	5,686	5,751	5,665
1556333_at	6,397	6,775	6,589	6,769	6,557	6,679
1556334_s_at	4,378	4,617	4,801	4,280	4,143	4,321
1556336_at	4,920	4,902	5,157	5,889	5,606	5,512
1556338_at	6,263	5,780	5,847	4,727	4,608	4,820
1556339_a_at	5,597	6,079	5,524	4,984	5,217	5,145
1556340_at	8,603	8,272	8,567	8,457	8,281	8,452
1556341_s_at	8,547	8,312	8,624	8,358	8,723	9,322
1556344_at	6,993	7,318	7,137	6,432	6,499	6,553
1556345_s_at	7,524	7,347	7,496	7,231	7,271	7,213
1556346_at	6,823	7,361	6,957	8,715	8,566	9,779
1556347_at	5,669	4,977	5,738	4,041	4,346	3,849
1556348_at	7,341	7,094	6,905	6,171	5,776	5,829
1556350_a_at	3,981	4,382	3,845	4,283	4,172	4,079
1556351_at	4,337	4,084	4,378	3,830	3,771	3,695
1556352_at	7,494	7,551	7,517	6,941	6,845	6,151
1556354_s_at	4,612	4,950	4,992	4,660	4,779	4,819
1556355_x_at	6,778	6,773	6,775	6,997	7,045	7,127
1556356_at	7,623	7,297	7,598	8,087	7,900	7,887
1556357_s_at	4,358	4,296	4,420	4,482	4,340	4,495
1556359_at	5,376	4,850	5,096	5,418	5,550	5,580
1556361_s_at	7,245	6,743	7,603	5,960	6,542	6,803
1556362_at	5,797	5,857	5,811	5,654	5,737	5,801
1556364_at	7,175	6,928	7,169	7,337	6,630	6,932
1556365_at	6,368	6,583	6,645	6,606	6,379	6,549
1556366_s_at	3,489	3,784	3,341	3,668	3,717	3,715
1556368_at	4,845	5,009	5,094	5,246	5,364	5,245
1556369_a_at	5,882	5,488	6,109	6,099	5,776	5,813
1556371_at	4,679	4,695	4,737	4,881	4,716	4,980
1556373_a_at	6,699	6,641	6,993	6,322	6,827	6,500
1556374_s_at	6,107	6,386	6,385	6,864	6,476	6,482

1556375_at	6,929	6,440	6,890	6,495	7,027	6,544
1556377_s_at	6,673	6,317	6,358	7,197	7,117	7,337
1556378_a_at	4,666	4,979	4,797	4,875	4,865	5,185
1556381_at	4,979	5,368	5,231	5,571	5,473	5,277
1556382_a_at	6,302	6,326	6,348	6,913	6,788	6,782
1556383_at	7,604	7,361	7,479	7,668	7,802	7,828
1556385_at	7,742	7,459	7,701	7,231	8,303	7,805
1556387_at	6,908	7,052	7,170	7,362	7,242	6,880
1556388_a_at	4,460	4,265	4,683	4,562	4,590	4,535
1556389_at	6,993	7,012	7,173	6,719	6,761	7,044
1556392_a_at	3,937	4,607	3,802	3,725	3,637	3,805
1556393_at	5,111	5,038	4,822	4,638	4,854	4,783
1556394_a_at	5,009	5,584	5,292	5,641	5,708	5,778
1556395_at	4,062	4,066	4,298	4,279	4,046	4,233
1556397_at	7,444	7,277	7,626	7,937	8,029	8,073
1556398_a_at	6,185	5,877	6,210	7,091	6,742	6,487
1556400_at	6,497	6,236	6,488	7,312	7,188	7,346
1556401_a_at	5,138	5,067	4,880	5,463	5,540	5,887
1556402_at	6,415	6,640	6,248	7,161	6,820	7,060
1556404_a_at	4,804	4,912	4,601	4,756	4,777	4,787
1556405_s_at	5,819	6,440	6,516	7,954	7,289	7,501
1556406_at	5,255	4,883	5,111	5,853	5,719	5,915
1556409_a_at	6,156	6,145	6,160	6,305	5,837	6,251
1556410_a_at	4,211	4,260	4,067	4,091	4,096	4,158
1556411_s_at	5,521	5,531	5,533	5,992	6,001	6,242
1556412_at	7,685	7,815	7,991	7,807	8,151	7,630
1556413_a_at	5,554	5,878	5,816	6,845	6,504	6,933
1556414_at	6,519	7,249	6,193	5,778	5,764	5,973
1556416_s_at	6,708	6,702	6,349	6,894	8,095	7,623
1556417_a_at	4,435	3,794	4,473	5,012	5,081	4,846
1556420_s_at	5,894	5,925	6,204	6,162	6,352	6,249
1556421_at	5,392	5,687	5,557	4,510	4,119	4,283
1556422_at	6,543	6,269	6,524	7,354	6,926	6,899
1556423_at	4,579	4,953	4,641	5,252	5,440	5,604
1556425_a_at	7,455	7,101	7,350	6,599	6,922	6,970
1556426_at	6,357	6,472	6,641	6,792	6,649	6,638
1556427_s_at	6,562	6,835	7,182	6,878	6,721	6,852
1556429_a_at	7,382	7,864	6,919	5,481	5,440	5,103
1556432_at	7,408	6,632	7,202	5,917	6,383	6,083
1556434_at	7,223	6,878	6,888	7,320	7,208	7,558
1556435_at	5,719	5,405	5,551	6,008	5,718	5,783
1556436_at	4,949	4,932	5,352	5,318	5,320	5,571
1556437_at	4,533	4,828	5,019	6,008	6,121	6,333
1556439_at	4,019	4,041	3,873	4,333	4,211	4,454
1556442_x_at	8,593	8,674	8,624	7,975	8,560	8,431
1556444_a_at	5,949	6,246	5,961	6,101	5,864	5,762
1556445_at	7,171	7,466	7,261	7,753	7,762	8,046
1556447_at	4,083	4,157	4,110	4,280	4,209	4,196
1556448_a_at	5,444	5,618	5,371	5,938	5,966	5,845
1556449_at	7,781	7,593	7,496	8,732	8,540	8,824
1556451_at	6,715	6,878	6,399	6,450	6,558	6,521
1556452_a_at	6,219	5,988	6,281	6,533	6,423	6,548
1556453_at	6,331	6,572	6,817	7,538	7,573	7,827
1556454_a_at	4,028	3,906	4,082	4,701	4,424	4,338
1556456_at	5,858	6,384	6,017	6,196	5,954	5,616
1556457_s_at	4,292	4,371	4,464	4,493	4,495	4,636

1556459_at	7,120	7,059	7,261	7,555	7,398	7,548
1556460_a_at	6,184	6,187	6,290	6,641	6,541	6,787
1556461_at	3,752	3,864	3,777	3,862	3,947	3,976
1556462_a_at	4,398	4,265	4,568	4,303	4,489	4,329
1556464_a_at	8,341	8,244	8,235	8,121	8,192	8,295
1556465_at	5,626	5,617	5,949	6,539	6,788	6,635
1556467_at	5,419	5,374	5,367	5,383	5,362	5,410
1556468_at	7,151	6,972	7,195	7,398	7,403	7,714
1556469_s_at	8,676	8,877	9,092	5,134	5,033	5,116
1556471_at	6,380	6,311	6,210	6,418	6,352	6,626
1556472_s_at	6,166	6,530	6,046	6,250	5,969	6,186
1556473_at	4,145	3,940	4,018	3,764	3,580	3,589
1556474_a_at	5,703	4,758	4,990	4,220	4,288	4,142
1556476_at	5,388	5,304	5,240	6,009	5,663	5,535
1556477_a_at	5,820	5,854	5,623	6,782	6,568	6,748
1556479_at	7,694	7,711	7,681	7,935	8,019	8,211
1556480_a_at	6,668	6,284	6,565	6,927	6,622	6,847
1556484_at	7,452	7,317	7,501	7,376	7,356	7,402
1556486_at	5,031	5,286	5,101	5,403	5,301	5,214
1556487_a_at	5,257	5,132	4,952	5,541	5,161	5,535
1556488_s_at	4,971	5,247	4,812	4,571	4,219	4,112
1556489_at	4,885	5,349	5,328	5,111	5,103	5,225
1556490_a_at	6,031	5,911	6,327	6,680	6,654	6,727
1556491_at	4,092	4,203	4,170	4,139	4,017	4,295
1556492_a_at	4,000	4,406	3,805	4,472	4,380	4,831
1556493_a_at	6,112	5,767	5,818	5,722	6,576	6,031
1556494_at	6,713	6,527	6,738	7,494	6,884	7,297
1556496_a_at	6,741	6,203	6,939	7,628	7,579	7,538
1556497_a_at	3,763	3,994	3,867	4,006	4,411	4,509
1556498_at	6,064	6,049	6,466	5,964	6,094	5,884
1556499_s_at	7,166	7,808	7,642	12,775	13,857	13,964
1556500_a_at	4,488	4,364	4,311	4,368	4,293	4,361
1556502_at	5,965	5,687	5,713	6,580	6,546	6,939
1556504_at	4,349	4,421	4,400	4,689	4,605	4,790
1556505_at	6,123	6,395	6,115	6,255	5,734	6,014
1556507_at	8,977	8,698	8,951	9,505	9,428	9,803
1556508_s_at	5,659	5,199	5,284	5,867	5,722	6,068
1556510_at	6,654	6,393	6,337	7,399	7,053	7,188
1556511_a_at	4,873	4,914	4,815	5,004	5,128	5,100
1556513_at	5,978	6,429	6,249	6,049	5,928	6,153
1556514_at	5,136	5,015	4,913	6,061	5,480	5,791
1556516_at	5,417	5,376	5,481	5,729	5,784	5,511
1556518_at	4,556	4,460	4,790	4,781	4,663	4,807
1556520_at	5,382	5,031	5,335	5,323	5,387	5,535
1556521_a_at	4,149	4,078	4,008	5,247	5,319	5,525
1556527_a_at	5,587	5,410	5,440	5,361	5,667	5,343
1556528_at	4,122	4,145	4,199	4,523	4,433	4,655
1556529_a_at	3,914	3,990	4,026	4,353	4,209	4,277
1556531_at	5,072	4,953	4,944	5,660	5,275	5,744
1556533_at	8,109	7,385	8,110	8,701	8,670	8,909
1556536_at	6,517	6,604	6,779	6,724	6,800	7,080
1556538_at	7,085	7,545	7,607	8,225	8,012	8,206
1556540_a_at	8,608	8,455	8,476	9,238	9,050	9,376
1556541_s_at	4,252	4,284	4,462	4,204	4,270	4,205
1556542_a_at	6,372	6,737	6,351	6,030	5,489	5,639
1556543_at	8,590	8,099	8,629	5,128	7,120	5,115

1556545_at	6,195	6,680	6,499	6,953	7,928	7,927
1556551_s_at	8,135	8,024	8,061	8,093	7,620	8,128
1556552_a_at	7,730	7,254	7,409	7,831	7,463	7,491
1556553_at	5,831	5,580	5,897	6,472	6,324	6,253
1556554_at	7,172	7,331	7,513	7,362	7,523	7,584
1556555_at	6,697	7,039	7,104	6,936	6,439	6,984
1556558_s_at	5,849	5,965	6,276	6,003	5,545	5,861
1556560_a_at	8,362	8,321	8,249	7,920	7,546	7,827
1556564_at	6,774	6,641	7,219	7,384	7,388	7,606
1556567_at	8,287	8,057	8,425	7,580	7,836	7,631
1556568_a_at	7,276	6,863	7,050	4,059	5,299	4,324
1556571_at	4,512	4,540	4,661	4,957	4,908	4,704
1556573_s_at	6,404	6,258	6,580	6,690	6,872	7,089
1556578_a_at	5,067	4,953	5,089	5,670	5,664	5,581
1556579_s_at	5,445	6,036	5,826	5,758	5,785	5,622
1556580_a_at	3,941	4,026	3,914	3,776	3,943	3,957
1556581_at	6,524	6,865	6,953	6,216	6,392	6,098
1556582_at	8,811	8,688	8,999	8,803	8,872	9,015
1556583_a_at	3,969	3,900	4,013	4,194	4,979	4,077
1556584_at	6,821	6,228	6,359	6,816	6,598	6,533
1556586_x_at	7,020	6,559	6,851	7,033	6,686	6,732
1556588_at	7,120	6,922	7,499	8,694	7,749	8,078
1556589_at	6,824	6,644	6,692	6,847	7,281	7,105
1556590_s_at	4,542	4,265	4,542	5,089	5,263	5,077
1556592_at	3,545	3,654	3,578	4,014	3,794	4,098
1556593_s_at	5,403	5,397	5,576	6,103	6,066	6,101
1556595_at	6,066	5,487	5,789	5,195	4,925	5,142
1556597_a_at	6,190	5,531	5,186	4,888	5,130	4,645
1556598_at	3,574	3,590	3,539	3,490	3,445	3,471
1556599_s_at	3,908	4,183	3,867	3,919	3,938	3,929
1556601_a_at	6,651	6,008	6,745	5,909	6,153	5,911
1556602_at	4,993	4,902	5,313	3,956	4,721	4,046
1556606_at	6,056	5,801	5,745	6,708	8,696	6,984
1556607_at	5,634	5,474	5,565	6,983	7,153	7,407
1556608_a_at	6,507	5,923	6,466	6,408	6,389	6,496
1556609_at	4,631	4,307	4,814	4,144	4,171	4,202
1556613_s_at	8,555	9,076	7,942	8,106	8,379	8,349
1556616_a_at	6,104	5,385	6,240	5,209	5,195	5,489
1556617_a_at	4,595	4,519	4,822	4,900	4,755	4,897
1556618_at	6,320	6,323	6,623	6,576	6,262	6,581
1556619_at	6,326	6,011	6,328	6,743	6,867	7,196
1556620_at	6,666	6,482	6,473	6,998	6,754	7,087
1556621_a_at	6,491	6,146	6,282	6,937	6,802	7,026
1556622_s_at	3,740	3,611	3,867	4,022	3,955	3,910
1556623_at	4,658	5,470	4,899	5,009	4,786	5,370
1556624_at	3,486	3,649	3,655	3,614	3,662	4,034
1556625_a_at	3,357	3,577	3,406	3,687	3,979	3,770
1556627_at	5,363	5,264	5,450	5,675	5,702	5,715
1556629_a_at	4,831	4,778	4,955	4,528	5,807	4,704
1556630_at	5,066	4,638	5,098	5,887	5,954	5,864
1556633_at	6,438	6,121	6,256	6,840	6,706	6,570
1556636_at	6,090	6,008	5,710	6,650	6,511	6,645
1556638_at	5,468	5,726	5,013	5,702	5,534	5,610
1556639_at	6,106	6,133	5,793	5,830	6,279	6,039
1556641_at	6,078	6,232	6,327	6,431	6,525	6,573
1556643_at	7,879	8,406	7,994	7,135	6,914	6,480

1556645_s_at	6,116	5,826	5,998	6,232	6,409	6,628
1556646_at	10,604	10,165	10,698	8,331	9,235	8,435
1556648_a_at	4,107	4,077	4,646	4,741	4,812	4,841
1556649_at	6,440	6,520	6,590	7,183	6,732	7,004
1556650_at	7,343	6,205	6,702	6,388	6,183	5,993
1556651_at	4,327	4,278	4,244	4,709	4,553	4,731
1556653_at	4,311	4,497	4,293	4,915	4,910	4,918
1556654_at	5,919	6,153	6,086	7,492	7,314	7,897
1556655_s_at	5,126	4,866	4,984	5,838	5,985	6,129
1556656_at	7,910	7,882	8,184	6,669	7,546	6,916
1556657_at	5,199	5,579	5,356	5,252	6,016	5,354
1556658_a_at	5,406	4,706	5,240	4,299	6,246	5,095
1556659_at	6,768	7,053	6,470	6,198	6,370	6,291
1556661_at	6,065	6,049	6,277	6,638	6,386	6,723
1556662_at	6,888	6,953	6,966	7,823	7,834	7,834
1556663_s_at	5,824	6,074	5,836	6,165	6,089	6,186
1556665_at	5,790	5,915	5,847	6,218	6,334	6,206
1556666_a_at	4,063	4,139	4,306	4,277	4,230	4,191
1556667_at	5,126	5,544	4,962	5,623	5,625	5,685
1556669_a_at	7,142	7,053	7,069	7,448	7,141	7,273
1556670_at	6,764	6,644	6,426	7,686	7,566	7,448
1556671_s_at	6,749	6,704	6,643	7,146	7,072	7,031
1556672_a_at	6,735	6,378	6,388	6,115	6,173	5,915
1556675_s_at	6,925	7,004	6,812	7,321	6,955	7,041
1556676_a_at	5,619	5,986	5,636	5,590	5,565	6,106
1556677_at	4,594	4,469	4,362	4,725	4,624	4,565
1556678_a_at	4,475	3,801	4,135	4,175	4,500	4,414
1556679_at	6,946	6,834	6,895	7,189	6,983	7,170
1556682_s_at	5,542	5,010	5,017	4,673	5,181	5,065
1556683_x_at	5,069	5,139	5,116	5,015	4,867	4,984
1556684_at	6,722	6,786	6,695	7,521	7,301	7,523
1556685_at	5,432	4,890	5,171	6,204	5,612	6,019
1556687_a_at	5,765	5,750	5,820	5,936	6,041	6,321
1556689_a_at	7,193	6,909	7,255	8,634	7,990	8,079
1556690_s_at	7,560	7,456	7,616	7,986	8,351	8,311
1556694_a_at	7,710	6,979	7,831	4,759	5,101	4,938
1556695_a_at	4,466	4,573	4,655	5,701	6,344	6,158
1556696_s_at	4,686	5,290	5,310	5,793	6,992	7,044
1556697_at	3,445	3,387	3,578	3,329	3,404	3,302
1556698_a_at	4,326	3,978	4,177	4,718	4,315	4,583
1556699_at	6,893	6,819	6,692	7,032	6,802	7,109
1556700_a_at	5,101	5,258	5,273	5,749	5,868	5,920
1556701_at	7,416	7,332	7,486	7,738	7,744	7,892
1556704_s_at	4,419	4,561	4,479	4,978	6,349	5,938
1556706_at	4,630	5,001	4,495	4,915	4,813	4,688
1556709_a_at	7,244	7,406	7,250	7,956	7,844	7,702
1556711_at	4,984	5,155	5,132	5,068	5,079	5,128
1556713_at	3,897	3,872	4,081	4,035	4,024	4,186
1556715_at	6,502	6,628	6,716	7,311	7,394	7,239
1556717_at	6,569	6,458	6,691	6,868	6,683	6,880
1556718_s_at	8,252	8,159	8,127	8,403	8,364	8,709
1556721_at	7,036	6,388	6,416	7,944	6,768	7,183
1556722_a_at	8,385	7,973	8,130	9,009	8,854	8,887
1556724_at	5,961	6,079	6,145	5,716	5,715	5,958
1556725_a_at	5,799	5,636	6,157	5,548	5,583	6,054
1556727_at	7,178	7,133	7,073	7,763	7,561	7,652

1556728_at	5,280	5,139	5,109	5,345	5,382	5,540
1556730_at	4,798	4,419	4,614	4,979	5,160	4,979
1556732_at	5,594	5,139	5,183	4,269	4,686	4,155
1556734_at	7,403	7,192	7,723	8,044	8,117	7,873
1556735_at	6,597	6,243	6,357	5,997	7,266	7,413
1556736_at	6,960	7,123	7,129	6,933	6,643	6,917
1556737_at	7,128	7,032	7,338	7,882	7,748	7,859
1556739_at	9,158	9,032	9,155	9,912	9,677	10,246
1556740_at	6,489	6,122	6,414	7,610	7,454	7,781
1556742_at	4,666	4,939	4,601	4,943	4,714	4,727
1556743_at	6,942	5,906	5,598	4,163	4,780	3,958
1556744_a_at	7,803	7,160	7,127	5,445	5,675	5,398
1556747_a_at	6,835	6,727	6,835	7,492	7,266	7,568
1556748_x_at	7,681	7,554	7,789	8,044	8,260	8,181
1556749_at	5,101	4,781	5,243	5,276	4,826	5,142
1556750_at	4,971	4,953	4,836	5,571	5,734	5,965
1556753_s_at	5,714	5,930	5,792	6,638	6,326	7,075
1556754_at	5,545	5,137	5,866	6,064	6,148	6,281
1556755_s_at	7,235	6,933	7,117	7,902	7,572	7,879
1556757_a_at	4,301	4,570	4,360	5,409	5,315	5,770
1556758_at	5,602	5,834	5,669	5,597	5,619	5,682
1556759_at	3,821	4,141	3,729	3,746	3,790	3,744
1556760_a_at	4,471	4,358	4,311	4,444	4,524	4,572
1556761_at	6,257	5,914	6,487	6,055	5,837	5,832
1556762_a_at	6,761	6,274	6,805	7,037	7,137	7,313
1556763_at	5,717	5,337	4,948	5,658	5,632	6,081
1556764_s_at	6,194	5,983	5,653	5,697	5,326	5,275
1556766_at	7,121	6,695	7,176	7,548	7,128	7,252
1556768_at	7,460	7,467	7,226	8,223	7,618	8,162
1556769_a_at	4,672	4,469	4,689	5,017	4,806	4,938
1556770_a_at	3,979	3,980	4,177	4,041	4,303	4,453
1556771_a_at	5,612	5,506	5,492	6,127	5,874	6,250
1556773_at	4,731	4,864	4,648	5,223	5,255	5,359
1556775_at	4,512	4,499	4,954	4,772	4,964	4,971
1556776_a_at	4,909	4,748	5,258	4,459	4,530	4,431
1556777_a_at	6,240	6,128	6,197	7,056	7,249	7,645
1556778_at	4,878	4,152	4,468	5,937	5,915	6,402
1556779_s_at	5,733	6,141	5,970	7,171	7,061	7,286
1556781_at	3,876	3,919	3,842	3,821	3,888	3,831
1556783_a_at	4,176	4,144	4,180	3,908	4,145	3,998
1556784_at	6,012	6,127	5,979	6,231	6,004	6,239
1556786_at	3,930	4,405	3,743	4,312	4,104	4,464
1556787_s_at	4,821	4,701	4,583	4,959	4,607	4,666
1556789_a_at	5,404	5,537	4,755	5,621	5,787	5,945
1556793_a_at	5,780	5,741	5,833	7,001	7,107	7,328
1556794_at	4,502	5,219	4,787	4,069	4,037	4,046
1556796_at	6,053	6,011	6,328	6,494	6,185	6,276
1556797_at	7,650	7,671	7,986	7,817	7,519	7,498
1556798_a_at	5,552	5,542	5,331	4,657	4,691	4,898
1556800_a_at	5,722	5,261	5,500	5,249	5,204	5,323
1556801_at	6,912	7,627	7,093	7,619	7,488	7,869
1556803_at	6,205	5,992	6,000	6,595	6,579	6,624
1556804_s_at	3,950	3,777	3,886	4,332	4,136	4,260
1556805_at	5,099	4,575	4,985	5,497	5,618	5,571
1556806_at	6,784	7,282	7,434	7,256	7,406	7,400
1556808_at	6,088	6,128	6,090	6,016	6,120	5,845

1556809_a_at	6,019	6,313	6,039	5,864	6,111	5,874
1556810_a_at	4,031	4,110	3,894	4,019	3,993	4,084
1556812_a_at	5,729	5,819	6,013	6,063	6,080	6,132
1556813_at	4,794	4,763	4,847	5,028	4,830	4,767
1556814_a_at	4,815	5,015	4,827	5,006	4,813	5,040
1556815_at	4,219	4,114	4,408	3,859	3,781	3,986
1556817_a_at	4,202	4,319	4,264	4,296	4,304	4,451
1556818_at	7,279	7,046	7,759	6,029	7,582	6,084
1556820_a_at	5,626	4,621	4,863	4,067	4,518	4,031
1556821_x_at	8,076	7,053	7,119	4,091	4,834	4,328
1556822_s_at	5,994	5,613	5,854	6,025	5,758	6,305
1556823_s_at	6,155	6,594	5,673	6,999	6,746	6,909
1556824_at	7,449	7,413	7,525	8,648	8,545	8,500
1556826_s_at	6,367	6,319	6,308	6,564	6,630	6,840
1556827_at	4,995	4,431	5,022	5,658	5,562	5,852
1556828_at	4,983	5,389	4,820	5,506	5,680	5,660
1556829_at	6,790	6,843	7,024	7,075	7,384	7,299
1556831_at	7,183	6,903	7,036	7,310	7,380	7,137
1556832_at	4,000	3,381	3,935	4,562	4,499	4,620
1556834_at	3,859	3,794	3,864	3,663	3,703	3,625
1556835_s_at	7,274	7,287	7,060	8,091	7,705	7,684
1556836_at	5,300	5,606	5,625	5,817	5,684	5,543
1556837_a_at	6,528	6,617	6,364	7,353	7,112	7,429
1556839_s_at	5,832	5,645	5,834	6,646	6,432	6,617
1556841_a_at	5,763	6,211	5,601	5,874	6,049	5,632
1556842_at	4,496	4,845	4,448	5,111	5,193	5,206
1556844_at	5,021	4,986	5,214	5,532	5,480	5,586
1556846_at	6,572	6,609	6,904	7,516	7,003	7,405
1556847_s_at	5,406	5,434	5,410	6,136	5,866	6,022
1556849_at	4,634	3,979	4,073	3,554	3,463	3,475
1556850_at	4,983	5,821	5,137	5,609	5,599	5,574
1556851_at	5,136	4,880	5,014	5,615	5,534	5,699
1556852_a_at	7,889	7,794	7,815	8,140	8,187	8,485
1556854_at	6,683	6,407	7,122	7,180	7,104	7,312
1556855_a_at	6,973	7,082	7,093	7,827	7,431	7,562
1556856_at	5,736	5,774	5,267	6,024	5,939	5,950
1556859_a_at	5,190	4,841	5,171	5,530	5,703	5,755
1556864_at	8,915	8,776	8,796	9,148	8,725	9,166
1556865_at	6,609	6,131	6,659	5,849	6,301	6,187
1556867_at	3,822	3,794	3,842	3,909	3,878	3,892
1556872_s_at	5,879	6,017	5,896	6,079	5,907	5,969
1556873_at	3,794	4,294	3,610	3,623	3,518	3,702
1556874_a_at	6,342	7,361	6,522	6,033	5,996	6,147
1556876_s_at	4,615	4,388	4,435	4,302	4,192	4,398
1556877_at	3,429	3,514	3,619	3,934	3,842	4,312
1556879_at	5,574	5,747	5,767	5,939	5,902	5,995
1556881_at	4,955	4,947	4,811	4,859	5,001	4,787
1556883_a_at	5,745	5,770	5,593	7,075	6,734	7,120
1556884_a_at	3,675	3,585	3,976	3,595	3,673	3,575
1556885_at	5,618	5,269	5,360	5,727	5,657	5,671
1556886_a_at	4,671	4,540	5,119	5,575	5,538	5,875
1556887_at	5,955	5,896	6,105	6,187	6,129	6,038
1556888_at	3,896	3,874	4,007	4,069	4,121	4,227
1556889_s_at	3,333	3,516	3,569	3,642	3,478	3,364
1556891_at	5,217	4,962	4,756	5,117	5,141	4,887
1556894_at	5,646	5,502	6,234	6,713	6,996	7,028

1556895_a_at	6,861	6,659	6,744	7,078	6,830	6,837
1556896_at	7,158	7,087	7,054	7,128	7,086	7,026
1556898_at	4,412	4,299	4,456	4,644	4,692	4,659
1556899_at	5,128	5,178	5,233	5,401	5,308	5,111
1556900_at	8,430	8,312	8,403	9,550	9,545	9,541
1556901_s_at	4,326	4,276	4,257	4,700	4,415	4,572
1556903_at	5,022	4,461	5,060	5,130	4,962	4,929
1556904_at	3,872	4,014	3,995	4,264	4,102	4,310
1556905_at	6,110	5,917	6,087	6,702	6,604	6,776
1556907_at	6,660	6,588	6,130	6,757	6,569	6,793
1556909_at	7,118	7,166	7,049	7,341	7,102	7,246
1556911_at	8,721	9,069	8,243	5,556	6,002	5,738
1556912_at	4,121	3,926	3,865	5,514	5,021	5,537
1556913_a_at	4,478	4,366	4,341	4,477	4,264	4,428
1556914_at	7,536	7,265	7,273	8,743	7,947	8,244
1556916_a_at	5,223	4,789	5,390	6,329	6,647	6,504
1556917_a_at	4,507	4,448	4,568	5,472	5,734	5,964
1556919_at	6,652	6,551	6,598	6,955	6,806	6,974
1556920_s_at	5,334	5,672	5,489	6,096	5,777	5,973
1556921_at	4,793	4,784	5,039	5,363	5,364	5,453
1556923_at	6,066	6,006	6,001	5,986	6,152	6,049
1556924_at	4,952	5,018	4,757	5,804	5,523	5,639
1556925_at	4,819	5,100	4,956	4,641	4,798	4,839
1556927_at	4,988	4,409	4,453	5,007	4,774	4,749
1556928_at	8,191	8,030	8,238	8,925	8,624	8,947
1556929_at	6,961	6,591	7,084	7,058	7,016	6,713
1556931_at	6,233	6,002	6,102	6,155	6,623	6,151
1556932_at	6,507	5,843	5,494	5,609	5,567	5,750
1556933_at	5,109	5,142	5,121	3,609	3,640	3,696
1556935_at	5,768	5,653	5,949	5,984	7,378	5,993
1556936_at	3,840	3,571	3,715	3,486	3,578	3,523
1556937_at	7,037	6,861	7,122	7,107	6,884	7,007
1556938_a_at	4,746	4,609	4,555	4,891	4,806	5,140
1556940_at	4,875	4,790	4,856	5,218	5,356	5,201
1556941_a_at	6,199	6,099	6,210	6,973	6,680	6,990
1556942_at	5,393	4,913	5,441	6,443	6,360	6,474
1556944_at	3,777	3,953	3,899	5,197	5,326	5,636
1556945_a_at	5,024	5,116	5,021	6,385	6,344	6,318
1556947_at	7,096	6,646	7,068	6,847	6,847	6,626
1556950_s_at	7,202	6,697	7,076	7,246	8,123	7,367
1556951_at	5,672	5,627	5,519	6,076	6,248	6,219
1556952_at	6,085	6,204	6,304	7,161	7,098	6,899
1556954_at	4,717	4,921	4,960	5,746	5,507	5,951
1556956_at	6,995	7,296	7,565	8,209	7,781	7,869
1556958_at	6,579	6,545	6,388	6,731	6,602	6,919
1556959_at	8,116	8,190	8,282	9,145	9,032	9,349
1556960_a_at	6,698	6,864	6,324	6,658	6,371	6,520
1556962_at	7,714	7,445	7,715	8,382	8,504	8,699
1556963_at	5,522	5,724	6,131	6,637	6,183	6,413
1556964_s_at	5,757	5,524	5,220	4,398	4,306	4,256
1556967_at	4,292	4,482	4,449	4,171	4,451	4,515
1556969_at	6,541	6,562	6,660	6,763	6,660	6,975
1556970_at	6,133	5,901	5,996	7,019	6,930	6,869
1556971_a_at	6,851	7,132	7,292	7,136	6,978	7,453
1556972_at	6,429	6,293	6,179	7,067	6,829	6,863
1556976_s_at	6,839	6,762	6,896	7,826	7,269	7,640

1556978_a_at	7,309	7,202	7,166	7,366	7,113	7,022
1556980_at	5,109	5,079	5,169	5,168	5,152	5,362
1556982_at	8,085	8,285	8,253	8,472	8,599	8,816
1556983_a_at	5,781	5,768	5,557	5,682	5,656	5,665
1556984_at	7,977	7,903	7,920	7,717	7,952	7,514
1556985_at	7,091	6,757	6,957	6,923	6,850	6,927
1556986_at	6,465	6,235	6,255	6,650	6,750	6,575
1556987_s_at	6,361	6,475	6,582	7,110	6,899	7,114
1556988_s_at	10,599	10,790	10,526	9,579	9,618	9,273
1556989_at	7,142	5,432	6,291	5,177	6,106	5,453
1556990_at	5,446	5,376	5,548	6,271	5,853	5,861
1556992_at	6,982	6,670	6,770	7,148	6,551	7,403
1556994_at	7,160	6,925	7,148	7,541	6,808	7,356
1556996_at	4,918	5,277	4,880	4,863	4,942	5,019
1556997_a_at	3,919	3,756	4,159	4,518	6,614	4,530
1556999_at	7,449	7,560	7,435	7,744	7,649	8,022
1557000_at	8,098	8,070	8,064	9,074	9,109	9,252
1557002_x_at	7,379	7,466	7,370	8,098	8,120	8,257
1557003_at	7,025	7,182	7,338	7,576	7,549	7,574
1557004_at	6,020	5,990	6,129	6,377	6,169	6,099
1557007_a_at	6,189	5,931	6,171	7,179	7,352	7,393
1557008_at	8,117	8,117	8,429	9,445	9,669	10,037
1557009_a_at	3,887	4,002	4,003	4,526	4,130	4,283
1557012_a_at	3,928	3,809	3,738	3,703	3,729	3,725
1557014_a_at	6,514	7,415	6,628	3,718	3,849	3,799
1557016_a_at	6,321	6,389	6,318	6,724	6,347	6,441
1557017_at	3,419	3,857	3,536	3,495	3,489	3,656
1557018_a_at	4,025	4,381	3,761	4,236	4,210	4,108
1557021_s_at	6,637	6,777	6,957	7,280	7,332	7,314
1557022_at	5,908	5,892	5,937	6,361	6,512	6,917
1557024_at	4,021	3,642	3,936	3,939	4,066	4,128
1557025_a_at	6,924	6,868	6,901	7,435	7,445	7,285
1557026_at	6,312	6,217	5,957	7,052	6,627	7,135
1557027_at	4,485	4,339	4,479	4,932	4,915	5,177
1557029_at	7,026	7,694	6,561	6,122	6,119	5,910
1557030_at	4,057	4,099	4,008	4,285	4,126	4,067
1557031_at	7,160	7,178	7,065	7,783	8,087	8,063
1557034_s_at	7,982	7,365	8,125	7,882	7,288	7,511
1557036_at	3,832	4,000	4,448	4,648	4,915	4,503
1557037_a_at	4,242	3,950	4,318	4,147	4,029	4,240
1557038_s_at	8,398	8,045	8,846	8,119	7,497	7,393
1557042_at	5,358	5,684	5,590	5,999	6,067	5,801
1557044_at	4,158	3,903	4,476	4,161	4,133	4,545
1557046_x_at	4,585	4,759	4,769	4,861	4,712	4,889
1557047_at	9,295	9,114	9,366	9,239	9,654	9,548
1557049_at	5,398	5,906	5,836	6,524	6,522	6,930
1557050_at	3,801	3,828	3,865	3,859	3,825	3,875
1557051_s_at	5,825	5,920	5,905	6,992	7,120	8,286
1557052_at	5,834	6,050	6,365	6,855	6,910	7,395
1557053_s_at	10,176	10,250	10,286	9,301	9,774	9,420
1557055_s_at	8,455	8,538	8,554	9,598	8,954	9,041
1557056_at	5,980	6,375	6,231	6,319	6,206	6,394
1557057_a_at	3,813	4,496	3,716	3,903	3,837	3,832
1557059_at	6,343	7,330	6,415	5,656	5,551	6,124
1557060_at	5,733	5,745	5,927	6,069	6,091	5,888
1557062_at	3,794	3,901	3,528	3,869	3,713	4,062

1557063_at	5,452	5,382	5,587	6,479	6,117	6,363
1557064_s_at	6,341	6,192	5,987	6,240	6,150	6,342
1557065_at	8,882	8,358	8,567	7,859	7,414	7,121
1557066_at	4,474	4,795	4,333	4,224	4,368	3,957
1557067_s_at	8,823	8,334	8,995	7,143	6,183	7,142
1557068_at	4,654	4,357	4,002	4,569	4,604	4,528
1557070_at	7,563	7,412	7,575	8,466	8,160	8,251
1557071_s_at	7,128	6,545	6,171	6,591	6,386	6,277
1557073_s_at	6,383	6,201	6,033	5,965	5,526	6,100
1557077_a_at	7,726	7,820	7,961	6,472	6,804	6,585
1557078_at	5,304	5,359	5,302	7,057	6,890	6,883
1557079_at	3,993	4,405	4,096	4,585	6,567	5,423
1557080_s_at	5,034	5,104	4,774	8,804	10,561	9,960
1557081_at	9,541	9,608	8,960	7,089	8,331	7,182
1557085_at	4,244	4,405	4,053	4,660	4,634	4,621
1557089_at	8,021	7,436	7,466	7,594	7,537	7,839
1557091_at	6,849	6,644	6,831	7,346	6,839	7,386
1557094_at	5,309	5,004	5,458	6,935	6,786	7,295
1557098_s_at	6,659	6,631	6,345	6,999	6,899	6,927
1557099_at	6,704	6,621	6,559	6,865	6,844	6,861
1557100_s_at	7,544	6,920	7,014	7,725	8,087	7,520
1557103_a_at	6,718	6,037	6,466	7,343	7,170	7,266
1557104_at	5,292	5,009	4,963	5,069	5,234	4,826
1557105_a_at	8,406	8,445	8,028	7,793	7,729	8,017
1557107_at	3,914	4,101	4,221	3,939	4,203	3,934
1557112_a_at	7,474	7,492	7,582	8,548	7,846	7,992
1557113_at	4,535	4,535	4,727	4,468	4,350	4,104
1557114_a_at	7,211	7,047	7,335	7,226	7,142	7,207
1557116_at	7,004	6,515	6,820	7,981	7,722	7,541
1557117_at	6,780	6,695	6,893	7,338	7,339	7,641
1557118_a_at	6,120	6,641	5,801	5,055	5,038	5,408
1557119_a_at	7,082	7,528	6,788	8,113	8,471	8,602
1557120_at	8,188	8,161	8,251	9,279	9,046	9,120
1557121_s_at	8,791	8,956	8,828	8,208	8,245	8,221
1557122_s_at	4,268	4,480	4,296	4,195	4,185	4,223
1557123_a_at	6,605	6,492	6,746	7,692	7,766	7,830
1557124_at	7,530	7,360	7,322	8,052	7,925	8,228
1557126_a_at	4,573	4,503	4,638	5,478	5,385	5,127
1557128_at	6,223	6,343	6,213	6,855	6,735	6,664
1557129_a_at	5,019	4,824	4,873	6,024	6,120	5,311
1557131_at	6,732	6,436	6,193	6,756	6,557	7,025
1557132_at	5,625	6,303	4,548	3,729	4,246	3,851
1557133_at	4,292	4,152	4,081	4,778	4,788	5,022
1557135_at	6,923	6,322	6,835	6,058	6,041	6,170
1557136_at	5,578	5,636	5,394	6,455	6,130	6,447
1557137_at	8,468	8,828	8,439	8,236	7,344	8,130
1557139_at	5,012	5,526	5,598	4,302	4,463	4,555
1557141_at	7,303	7,418	7,157	7,791	7,714	7,904
1557143_at	6,261	5,968	6,035	5,290	5,421	5,466
1557145_at	5,509	5,315	5,437	5,323	5,321	5,729
1557146_a_at	6,667	6,852	6,687	7,383	6,862	6,950
1557147_a_at	6,466	6,241	6,549	6,768	6,412	6,773
1557148_at	6,650	6,759	6,551	7,438	7,517	7,522
1557149_at	6,239	6,759	6,401	6,137	6,078	5,849
1557151_at	4,457	4,351	4,081	5,305	5,689	5,326
1557154_at	7,553	7,476	7,728	7,948	7,756	7,729

1557155_a_at	6,368	6,549	6,827	6,783	6,812	7,085
1557158_s_at	6,554	6,149	6,085	6,819	6,711	6,785
1557161_at	4,836	4,669	4,489	5,466	5,002	5,055
1557162_at	7,223	7,134	7,114	7,507	7,475	7,456
1557164_a_at	8,040	7,794	8,153	6,198	7,062	7,348
1557165_s_at	7,706	7,721	7,824	8,225	7,808	8,344
1557166_at	4,655	4,733	4,329	4,664	4,650	4,481
1557167_at	5,861	5,703	4,861	4,037	3,912	3,942
1557169_x_at	6,031	5,520	5,458	4,176	4,478	4,231
1557170_at	7,593	7,099	7,272	7,098	7,128	7,096
1557172_x_at	8,485	8,177	8,658	8,035	8,174	8,078
1557174_a_at	7,223	7,329	6,568	5,573	5,697	5,028
1557176_a_at	7,846	7,559	7,251	7,140	7,020	7,788
1557177_at	4,156	4,332	4,268	4,527	4,670	4,612
1557179_s_at	3,907	3,976	4,323	4,197	3,964	4,105
1557180_at	3,850	3,536	3,450	3,615	3,510	3,499
1557181_s_at	5,425	5,432	5,020	4,643	4,912	4,783
1557185_at	7,704	7,466	7,845	8,903	9,138	9,222
1557186_s_at	8,278	8,377	8,338	9,553	9,435	9,669
1557189_at	6,971	6,844	6,844	7,025	6,863	6,917
1557190_at	4,937	4,881	4,816	5,755	5,895	5,717
1557192_at	6,387	6,424	6,489	6,326	6,170	5,985
1557193_at	7,515	6,880	7,166	6,171	6,816	6,210
1557194_a_at	7,699	6,763	7,241	7,829	7,232	7,810
1557195_at	4,515	4,601	4,789	5,038	4,983	5,319
1557196_a_at	5,775	5,999	5,813	6,240	6,435	6,557
1557197_a_at	5,930	5,870	5,978	6,534	6,795	6,427
1557203_at	8,565	9,016	8,811	7,908	7,526	7,718
1557206_at	4,607	5,147	4,732	3,791	3,798	3,899
1557207_s_at	8,435	8,145	8,452	8,928	8,537	8,722
1557208_at	5,201	5,231	5,660	6,069	5,849	5,967
1557211_a_at	5,424	5,303	5,186	5,410	5,492	5,455
1557212_at	7,391	7,317	7,397	7,689	7,551	7,327
1557214_at	5,464	5,431	5,240	6,648	5,955	6,216
1557215_at	8,450	8,231	7,641	4,712	4,666	4,695
1557217_a_at	6,099	6,863	5,861	4,173	3,965	4,230
1557218_s_at	7,334	7,364	7,314	5,774	5,603	5,823
1557219_at	5,015	4,868	4,880	4,930	4,867	4,871
1557222_at	3,859	3,792	3,749	4,361	3,870	4,517
1557223_at	5,838	6,071	6,426	5,959	6,033	6,083
1557224_at	6,049	6,177	5,742	4,820	5,370	5,135
1557225_at	6,191	5,938	5,950	6,557	6,274	6,614
1557226_a_at	7,808	7,570	7,433	8,056	7,933	8,230
1557227_s_at	8,519	8,426	7,484	5,922	5,748	5,340
1557228_at	7,204	7,253	7,267	7,872	7,774	7,702
1557232_at	7,167	7,616	7,310	6,886	7,221	7,406
1557233_at	5,287	4,953	5,500	6,273	6,043	6,220
1557235_at	6,182	6,234	6,364	6,705	6,570	6,538
1557236_at	4,130	4,328	4,394	4,150	4,732	4,458
1557238_s_at	6,935	6,345	6,160	4,008	4,654	4,394
1557239_at	6,942	6,185	5,964	5,074	5,835	4,922
1557240_a_at	7,104	6,549	6,224	3,768	5,521	4,012
1557241_a_at	5,831	5,463	5,553	6,306	6,693	6,659
1557242_at	6,794	6,477	6,815	6,858	6,865	7,226
1557244_a_at	6,915	6,407	6,526	6,673	6,710	6,732
1557246_at	7,872	7,254	7,424	6,813	7,558	6,774

1557248_at	7,657	6,732	7,535	5,874	5,468	5,573
1557252_at	7,558	7,268	7,618	6,147	6,525	6,157
1557256_a_at	3,798	3,982	3,726	4,154	4,436	4,100
1557257_at	5,930	7,545	7,041	6,368	6,517	6,818
1557258_a_at	6,946	7,642	7,197	7,589	7,271	7,440
1557260_a_at	7,640	8,111	7,223	6,568	6,510	6,335
1557261_at	5,618	6,569	5,459	5,697	6,930	6,705
1557262_at	4,687	4,587	4,663	4,647	4,471	4,575
1557263_s_at	7,696	7,021	7,311	8,246	8,716	8,503
1557265_at	7,705	7,611	7,662	7,881	7,920	8,016
1557267_s_at	5,744	6,163	4,744	3,880	3,989	4,001
1557270_at	8,137	7,190	8,074	5,732	6,394	5,755
1557274_at	6,597	6,641	6,224	7,151	6,672	7,013
1557275_a_at	5,257	4,472	5,587	6,468	7,148	6,699
1557276_at	8,194	8,402	8,344	8,640	8,573	8,820
1557277_a_at	4,014	4,024	4,338	5,030	5,048	5,340
1557278_s_at	6,758	6,181	6,503	5,481	6,386	6,070
1557279_at	5,695	5,889	6,025	5,802	6,013	5,727
1557280_s_at	4,702	4,868	4,899	4,686	4,604	4,582
1557283_a_at	6,235	6,000	5,689	5,309	5,294	5,289
1557285_at	3,429	3,518	3,581	3,795	3,982	3,964
1557286_at	5,252	5,128	4,992	5,657	6,267	5,900
1557287_at	6,653	6,317	6,253	6,743	6,662	6,751
1557289_s_at	5,696	5,609	5,749	5,913	6,063	5,977
1557290_at	3,999	4,183	4,279	4,410	4,278	4,417
1557292_a_at	6,774	6,049	6,371	6,655	6,512	6,683
1557293_at	5,722	5,167	5,186	3,737	4,153	3,552
1557295_a_at	5,466	5,715	5,612	5,946	5,957	6,309
1557296_at	5,865	5,735	5,625	6,345	6,212	6,432
1557297_at	4,590	4,284	4,278	5,079	4,696	4,999
1557300_s_at	8,566	7,667	7,807	5,390	6,065	5,821
1557301_a_at	4,564	4,471	4,698	4,665	4,768	5,106
1557302_at	8,542	8,214	8,283	7,612	7,332	7,038
1557303_at	7,528	7,403	7,282	7,593	7,471	7,833
1557305_at	6,205	6,168	6,373	6,499	6,450	6,394
1557309_at	4,598	4,884	4,824	4,504	4,527	4,642
1557311_at	6,677	6,637	7,048	7,007	6,653	6,840
1557312_at	5,359	5,526	5,893	5,927	5,656	5,871
1557314_at	5,522	5,917	5,849	5,274	4,980	5,322
1557315_a_at	6,768	6,726	6,761	7,142	7,035	7,014
1557316_at	5,956	5,842	6,231	6,289	6,152	6,407
1557319_at	7,785	7,546	7,677	8,075	8,215	8,062
1557321_a_at	3,769	3,738	3,754	3,831	3,665	3,717
1557322_at	6,131	5,968	5,949	4,269	4,368	4,603
1557325_at	7,193	7,033	6,664	7,773	7,610	7,843
1557326_at	3,952	4,595	4,074	3,950	3,956	4,036
1557328_at	4,445	4,477	4,366	4,728	4,870	4,916
1557329_at	6,203	6,017	6,042	6,148	6,134	6,432
1557330_at	7,581	7,512	7,704	8,180	7,559	7,654
1557331_at	8,301	8,141	8,211	7,232	7,112	6,850
1557334_a_at	5,534	5,869	5,989	6,334	6,404	6,821
1557335_at	6,725	6,414	6,522	6,997	6,716	6,913
1557336_at	4,265	4,473	4,847	4,988	4,956	4,877
1557338_x_at	7,072	6,937	6,948	7,533	7,070	7,479
1557339_at	4,441	4,292	4,345	4,462	4,394	4,413
1557341_x_at	4,318	4,712	4,211	4,371	4,350	4,412

1557342_a_at	7,021	6,923	7,135	7,815	7,568	7,463
1557343_at	5,014	5,227	5,196	5,183	5,003	5,073
1557345_at	4,789	4,542	4,563	4,739	4,997	5,160
1557346_a_at	5,525	4,968	5,424	5,697	5,563	5,911
1557347_at	6,854	6,527	7,022	7,185	7,758	7,348
1557348_at	4,946	4,998	5,059	4,282	4,228	4,170
1557349_at	5,983	6,266	6,129	6,431	6,270	6,331
1557350_at	6,136	6,287	6,439	5,226	5,499	5,145
1557352_at	8,599	8,754	7,641	6,547	6,827	6,664
1557353_at	6,262	6,591	6,584	6,656	6,187	6,357
1557354_at	4,706	4,942	4,880	4,949	4,882	4,867
1557357_at	6,378	6,860	6,563	6,908	6,905	7,118
1557359_at	4,969	5,079	5,148	5,083	5,131	5,271
1557360_at	8,326	7,876	8,399	6,171	8,171	6,100
1557362_at	7,425	7,377	7,449	7,397	7,289	7,371
1557363_a_at	4,668	4,996	4,873	3,685	3,580	3,544
1557366_at	3,400	3,328	3,567	5,167	4,827	5,225
1557369_a_at	9,847	10,253	9,831	6,158	6,100	6,194
1557370_s_at	7,581	7,570	7,679	8,040	8,095	7,893
1557371_a_at	8,150	8,035	8,213	8,955	8,716	8,772
1557372_at	5,843	6,041	5,979	5,657	5,750	5,725
1557373_at	5,811	6,245	6,294	6,222	6,058	6,462
1557374_at	5,424	5,496	5,429	5,703	6,133	5,703
1557375_at	6,407	6,278	6,193	6,346	6,526	6,529
1557380_at	6,107	5,855	5,782	6,394	6,417	6,460
1557382_x_at	6,759	6,727	6,792	7,422	7,105	7,422
1557383_a_at	4,752	4,752	4,774	4,034	3,781	3,812
1557384_at	8,653	8,520	8,940	6,822	8,005	7,285
1557385_at	8,678	9,032	8,344	6,686	6,464	6,613
1557386_at	5,290	5,186	5,468	6,086	6,185	6,474
1557388_at	7,680	7,859	7,591	7,053	6,664	6,893
1557389_at	6,224	5,837	6,031	6,898	6,672	6,952
1557394_at	6,907	6,906	6,650	6,913	6,825	6,669
1557395_at	4,992	5,441	5,222	5,374	5,153	5,453
1557398_at	5,276	5,451	5,241	5,306	5,488	5,748
1557399_at	6,229	5,678	6,652	6,108	6,109	6,363
1557400_at	7,071	7,134	6,605	6,568	6,240	6,570
1557402_at	4,934	5,044	5,105	5,154	4,994	5,174
1557403_s_at	4,279	3,994	3,861	3,789	3,985	3,820
1557404_at	6,465	6,363	6,688	7,761	7,604	7,647
1557405_at	5,073	5,463	5,190	5,667	5,875	5,682
1557406_s_at	6,188	5,994	6,007	7,087	6,850	7,005
1557409_at	8,640	7,518	8,216	7,739	8,260	7,973
1557410_at	7,021	6,816	6,958	7,245	7,072	7,112
1557411_s_at	8,702	8,984	8,706	9,346	9,273	9,538
1557413_a_at	6,600	6,773	6,623	7,120	6,606	6,989
1557415_s_at	6,537	6,601	7,099	6,327	6,047	6,089
1557417_s_at	5,096	5,335	5,182	6,151	5,859	6,073
1557418_at	6,051	6,163	5,838	6,911	6,994	7,103
1557419_a_at	6,563	6,008	5,882	5,967	6,012	6,145
1557421_at	7,441	7,333	7,681	7,716	7,708	7,806
1557422_at	6,247	6,282	6,268	6,485	6,003	6,394
1557424_at	3,789	4,101	3,725	4,009	3,960	4,195
1557427_at	4,891	4,509	4,971	5,114	4,836	5,054
1557429_s_at	5,021	4,674	4,742	4,422	4,287	4,517
1557430_at	4,555	4,881	4,328	4,361	4,433	4,425

1557431_at	3,919	3,842	4,145	3,820	3,549	3,716
1557432_at	7,757	6,911	7,063	5,407	6,431	5,781
1557433_at	8,419	7,968	8,172	6,018	6,404	5,814
1557434_at	5,326	5,388	5,631	5,543	5,493	5,807
1557436_at	7,515	7,501	7,617	7,365	7,289	7,405
1557437_a_at	3,444	3,689	3,569	3,602	3,759	3,779
1557438_at	5,279	6,056	5,266	4,413	4,492	4,671
1557439_at	5,114	4,841	5,088	5,524	5,095	5,326
1557443_s_at	3,862	4,125	4,314	4,489	4,122	4,363
1557444_at	5,827	5,604	6,279	6,429	6,135	6,353
1557446_x_at	5,724	5,879	5,770	6,308	6,076	6,383
1557448_a_at	4,836	4,909	4,717	5,553	5,171	5,277
1557450_s_at	8,995	8,591	8,865	9,442	9,121	9,054
1557451_at	5,566	4,858	5,224	6,245	6,337	6,432
1557452_at	7,192	6,597	6,783	6,524	6,682	6,663
1557453_at	7,323	7,094	7,157	7,604	7,385	7,642
1557455_s_at	7,680	8,028	7,845	8,016	7,784	7,867
1557456_a_at	4,877	4,990	4,964	5,203	5,604	5,593
1557457_at	7,909	7,596	7,894	7,937	8,168	8,368
1557458_s_at	8,513	8,528	8,643	9,069	9,044	9,098
1557459_at	5,212	5,586	5,676	4,448	7,057	5,219
1557463_at	4,002	4,236	4,302	4,393	4,329	4,376
1557465_at	5,766	5,818	5,631	6,346	6,467	6,576
1557466_at	6,856	6,481	6,937	6,653	7,001	6,937
1557468_at	3,953	3,769	4,049	3,974	4,078	4,069
1557470_at	6,452	6,397	6,068	6,499	6,356	6,791
1557472_a_at	4,860	4,437	5,031	5,302	5,114	4,954
1557474_at	5,336	5,142	5,097	5,191	5,115	5,221
1557475_at	3,662	3,838	4,045	3,693	3,740	3,666
1557476_at	6,940	6,988	6,937	7,268	6,897	7,217
1557477_at	6,179	6,000	6,057	5,577	5,510	5,582
1557478_at	6,986	6,761	7,231	7,709	7,473	7,274
1557479_at	7,234	7,164	7,209	7,165	7,242	7,332
1557480_a_at	8,334	8,286	8,513	8,625	8,392	8,466
1557481_a_at	4,388	4,626	4,349	5,351	4,407	4,731
1557483_at	6,399	6,144	6,614	7,409	7,003	7,423
1557484_at	6,470	6,581	6,252	7,021	6,552	6,273
1557486_at	3,905	4,099	3,993	4,689	4,542	4,797
1557487_at	7,888	6,799	7,903	6,034	5,975	5,190
1557488_at	4,099	4,469	4,488	3,766	3,723	3,776
1557491_at	5,741	5,831	6,220	6,483	6,472	6,609
1557493_x_at	6,281	6,235	5,891	6,465	6,386	6,434
1557495_at	5,627	5,419	5,561	6,050	5,606	5,838
1557498_a_at	5,433	5,305	5,310	6,337	5,777	5,849
1557501_a_at	6,144	5,968	6,210	6,815	6,607	6,906
1557502_at	5,665	4,901	5,164	5,803	5,827	5,802
1557504_at	5,293	5,048	4,837	4,153	4,561	4,799
1557505_a_at	5,854	5,970	6,085	5,312	5,790	5,384
1557506_a_at	5,642	5,122	5,039	4,165	4,625	4,165
1557507_at	6,860	7,174	7,141	6,579	6,499	6,642
1557511_at	5,943	6,186	6,076	6,709	6,692	6,747
1557512_at	5,229	5,297	5,170	5,286	5,350	5,123
1557513_a_at	5,408	5,532	5,662	6,491	6,243	6,632
1557514_a_at	5,385	5,248	5,419	5,584	5,436	5,690
1557515_at	5,628	5,926	5,646	6,455	6,359	6,716
1557517_a_at	4,369	4,499	4,430	4,598	4,770	4,910

1557518_a_at	6,481	6,373	6,414	6,571	6,612	6,700
1557519_at	4,333	4,254	4,289	4,309	4,408	4,206
1557520_a_at	4,250	3,951	4,359	4,453	4,037	4,249
1557521_a_at	8,948	8,549	9,193	7,039	7,478	7,253
1557522_x_at	6,684	6,173	7,000	4,802	5,178	4,885
1557523_at	5,323	5,292	5,376	5,812	5,363	5,605
1557527_at	5,123	5,377	5,382	5,660	6,222	5,505
1557528_at	6,853	6,412	6,825	6,777	6,960	6,696
1557529_at	5,945	6,023	5,992	6,191	6,137	6,162
1557531_a_at	4,428	4,719	4,311	4,710	4,555	4,521
1557532_at	7,746	7,869	8,361	7,998	7,849	7,848
1557533_at	3,939	3,971	3,867	4,228	4,543	4,333
1557534_at	4,758	4,815	5,039	4,173	4,346	4,158
1557535_at	5,293	5,020	4,688	5,761	5,766	5,740
1557536_at	7,551	7,312	7,202	7,538	7,674	7,567
1557538_at	6,110	5,277	5,912	5,619	5,856	5,690
1557539_at	7,263	6,613	6,649	6,955	6,962	7,279
1557540_at	6,828	6,928	7,017	7,634	7,830	8,118
1557541_at	5,973	6,526	6,309	5,704	5,607	5,869
1557542_at	6,598	5,912	6,618	6,297	5,983	6,313
1557543_at	7,166	6,493	6,527	5,428	6,991	5,608
1557544_at	5,160	5,316	5,044	5,330	5,067	4,924
1557545_s_at	6,964	6,306	6,684	4,518	4,197	4,339
1557548_at	4,688	4,698	4,971	5,034	5,052	5,016
1557550_at	6,698	6,541	6,744	7,330	7,472	7,519
1557551_at	5,332	5,013	4,891	4,883	5,109	4,819
1557553_at	4,918	5,083	5,619	4,744	5,167	5,006
1557555_at	7,286	6,591	6,727	6,273	6,333	6,430
1557557_at	4,082	4,766	4,209	5,314	5,225	5,456
1557558_s_at	5,575	5,904	5,722	5,686	5,204	5,722
1557560_at	4,486	4,609	4,555	4,446	4,370	4,505
1557562_at	5,830	4,843	5,013	5,036	4,937	5,059
1557564_at	5,336	5,314	5,249	6,511	6,701	6,759
1557565_a_at	5,644	5,841	5,814	5,780	5,958	5,938
1557566_at	3,866	4,401	4,307	4,011	4,032	4,073
1557567_a_at	6,293	6,101	6,912	6,273	5,780	6,256
1557569_at	6,640	6,311	7,331	7,328	7,416	7,539
1557570_a_at	5,386	5,106	5,117	5,650	5,858	6,008
1557571_at	4,137	4,470	4,236	4,678	4,498	4,474
1557575_at	5,511	5,415	5,681	6,541	6,242	6,203
1557576_at	5,699	5,561	5,625	5,836	5,692	5,619
1557578_at	5,921	6,246	5,976	5,542	6,007	5,587
1557580_at	4,239	4,067	4,152	3,922	4,585	4,414
1557581_x_at	4,836	4,955	4,942	4,489	4,909	4,981
1557582_at	7,308	7,164	7,320	7,469	7,432	7,558
1557583_at	3,803	4,119	4,350	4,406	4,100	4,586
1557584_at	7,334	7,249	7,280	7,643	7,864	7,492
1557585_at	4,460	4,511	4,525	4,576	4,472	4,226
1557586_s_at	7,348	7,028	7,568	6,892	7,387	6,892
1557588_at	7,519	7,332	7,575	8,392	8,017	8,216
1557589_a_at	6,989	6,952	6,630	7,137	7,286	7,158
1557590_at	7,456	7,408	7,658	8,090	7,714	8,047
1557591_at	4,625	4,541	4,744	4,506	4,693	4,513
1557593_at	6,097	6,285	5,996	4,420	4,267	4,275
1557595_at	9,180	9,127	9,288	9,164	9,243	9,177
1557597_at	3,987	3,934	3,860	4,278	4,107	4,220

1557598_at	4,552	4,774	4,701	4,392	4,239	4,201
1557599_a_at	3,741	3,801	3,756	3,558	3,553	3,677
1557601_s_at	7,350	7,256	7,524	8,678	8,621	8,726
1557602_at	6,171	6,123	6,359	6,326	6,128	6,113
1557604_at	4,304	4,212	4,467	4,585	4,374	4,520
1557605_a_at	8,114	8,015	7,750	8,327	8,176	8,048
1557606_at	5,072	5,148	5,126	5,266	5,253	5,474
1557607_at	6,673	6,190	6,116	7,031	6,834	6,914
1557608_a_at	6,499	6,421	6,431	6,898	6,836	6,639
1557609_s_at	4,319	4,661	4,733	5,224	4,845	5,275
1557610_at	5,804	5,671	5,932	6,108	6,038	5,965
1557611_at	8,364	8,033	8,582	8,943	9,098	9,004
1557613_at	7,460	7,174	7,176	8,667	8,278	8,546
1557615_a_at	7,568	7,231	7,391	7,331	7,266	7,307
1557616_at	7,649	7,971	7,897	6,993	6,567	6,767
1557617_at	3,836	3,713	3,677	4,000	4,024	4,178
1557618_at	6,325	6,504	6,301	6,593	6,567	6,587
1557620_a_at	5,683	6,184	6,161	7,020	6,497	6,510
1557621_at	6,957	6,778	6,663	6,471	6,317	6,582
1557623_at	6,624	6,842	6,747	6,884	7,043	6,952
1557624_at	5,731	5,532	6,536	6,017	5,333	5,927
1557626_at	6,429	7,205	7,085	6,776	6,790	6,696
1557627_at	4,205	4,436	4,641	4,436	4,431	4,577
1557628_s_at	6,397	6,340	6,421	7,045	6,935	7,033
1557630_s_at	7,403	7,449	7,225	7,197	7,153	7,053
1557631_at	4,049	4,125	4,276	4,379	4,654	4,408
1557632_at	6,892	6,706	6,481	7,927	7,842	8,069
1557633_at	5,716	5,848	6,185	5,947	5,922	6,276
1557636_a_at	3,529	4,152	3,755	3,457	3,509	3,552
1557637_at	4,695	5,251	4,698	4,641	4,595	4,672
1557638_at	5,318	5,610	6,184	5,388	5,261	5,398
1557639_at	4,340	4,652	4,636	4,566	4,219	4,545
1557641_at	4,666	4,535	4,663	5,513	5,223	5,281
1557642_at	4,295	4,264	3,923	4,266	4,304	4,212
1557644_at	4,389	4,433	4,369	4,158	4,190	4,153
1557645_at	4,434	4,495	4,647	4,795	4,645	4,709
1557646_at	4,216	4,357	4,377	4,224	4,352	4,225
1557647_a_at	3,870	3,960	3,750	3,878	3,927	4,005
1557649_at	8,683	8,693	8,899	9,110	9,212	9,456
1557651_x_at	7,383	7,331	7,183	7,924	7,958	8,147
1557652_a_at	6,235	6,726	6,416	6,734	6,502	6,731
1557656_at	8,268	7,924	8,317	8,743	8,859	8,795
1557657_a_at	5,837	5,882	6,000	6,842	6,612	6,569
1557658_at	6,329	6,195	6,239	6,437	6,118	6,064
1557659_a_at	5,364	5,025	4,818	5,321	5,194	5,046
1557660_s_at	7,454	7,246	7,372	8,374	8,119	8,033
1557661_at	5,257	5,542	5,547	5,577	5,859	5,679
1557664_at	6,528	6,564	6,637	6,537	6,666	6,653
1557665_at	6,114	5,916	6,176	7,166	6,474	6,707
1557666_s_at	8,069	7,729	7,865	8,333	7,906	7,887
1557667_at	7,360	7,050	7,268	6,043	6,093	6,173
1557669_at	7,531	7,301	7,242	7,875	7,460	7,713
1557672_s_at	4,893	4,774	4,987	5,178	5,285	5,476
1557673_at	7,029	6,813	7,126	7,193	6,931	6,892
1557674_s_at	4,595	4,503	4,555	5,600	5,635	5,463
1557675_at	9,248	8,931	9,114	7,275	8,333	7,549

1557676_at	7,156	6,924	6,925	7,883	7,563	7,263
1557677_a_at	5,874	6,080	5,914	7,456	7,142	7,564
1557679_at	7,110	7,748	7,342	7,142	6,837	7,021
1557680_at	7,517	7,338	7,604	8,738	8,055	7,778
1557681_s_at	5,765	5,759	5,460	6,971	6,967	7,156
1557682_a_at	6,399	6,334	6,744	7,692	7,697	7,889
1557683_at	3,452	3,688	3,538	3,473	3,578	3,391
1557684_at	9,103	8,619	8,526	6,772	7,114	6,729
1557685_at	6,325	6,525	6,308	6,273	7,574	6,361
1557688_at	5,357	5,393	5,759	5,314	5,413	5,373
1557689_at	7,778	7,721	7,537	8,136	8,471	8,016
1557690_x_at	8,127	8,000	8,023	8,369	8,399	8,274
1557692_a_at	3,533	3,617	3,513	3,733	3,640	3,854
1557693_at	5,658	5,391	5,454	6,302	6,275	6,443
1557697_at	5,989	6,014	6,109	6,041	6,021	5,933
1557698_at	5,032	5,352	4,824	4,037	4,414	4,143
1557699_x_at	5,168	5,055	4,913	4,267	4,286	4,245
1557700_at	6,436	6,783	6,940	7,188	7,039	6,923
1557701_s_at	5,901	6,246	5,518	6,588	6,136	6,665
1557702_at	6,528	6,253	6,477	6,456	6,316	6,557
1557703_at	4,516	4,748	4,507	4,641	4,552	4,498
1557704_a_at	5,469	5,321	5,072	4,981	5,274	5,056
1557705_a_at	4,412	4,357	4,276	4,657	4,715	4,714
1557706_at	5,247	5,080	5,334	5,108	5,123	5,365
1557707_at	6,213	6,028	6,732	5,437	6,552	6,018
1557709_at	5,403	5,362	5,847	5,689	5,599	5,807
1557710_at	7,526	7,674	7,458	6,783	6,426	6,656
1557712_x_at	8,949	8,858	8,868	8,394	7,978	8,225
1557713_at	6,086	6,078	5,944	6,088	6,223	6,121
1557714_at	7,794	7,677	7,920	7,610	7,683	7,590
1557717_at	3,749	3,794	3,647	3,722	3,806	3,711
1557718_at	7,251	8,436	8,301	5,694	7,841	6,304
1557719_at	6,854	7,041	7,027	7,464	7,538	7,303
1557720_s_at	6,485	6,585	6,430	6,959	7,072	7,233
1557721_at	5,765	5,773	5,496	6,042	5,805	6,013
1557723_at	7,530	7,291	7,585	7,718	7,644	7,775
1557724_a_at	6,075	6,305	5,962	6,274	6,297	6,234
1557726_at	5,915	6,046	6,075	6,141	5,953	6,106
1557727_at	6,084	5,978	5,976	6,752	7,162	6,980
1557729_at	7,422	7,099	7,222	7,384	7,368	7,643
1557731_at	6,590	6,682	6,986	7,589	7,515	7,612
1557733_a_at	5,037	5,065	5,656	5,183	4,856	5,289
1557734_s_at	6,078	6,357	6,141	6,729	6,625	6,780
1557735_at	3,638	3,400	3,456	4,030	3,979	3,877
1557736_at	5,743	5,135	5,400	5,817	5,621	5,941
1557737_s_at	6,972	6,539	6,624	4,327	5,074	4,655
1557738_at	6,517	5,894	6,702	4,286	5,299	4,448
1557739_at	5,037	5,034	5,058	5,082	5,251	5,439
1557740_a_at	4,910	5,031	4,732	5,502	5,125	5,262
1557741_at	4,379	4,597	4,096	4,841	4,677	5,085
1557742_a_at	4,377	4,840	4,659	5,050	5,097	5,228
1557744_at	5,630	5,753	5,922	6,620	6,291	6,571
1557745_at	5,045	4,453	4,653	4,714	4,727	4,641
1557746_at	3,662	3,616	3,693	3,457	3,415	3,398
1557749_at	3,990	3,955	4,049	4,595	4,571	4,657
1557751_at	5,957	6,155	6,201	4,520	5,362	4,587

1557753_at	4,814	5,189	5,545	5,008	4,788	4,800
1557754_at	6,436	6,624	7,053	6,116	6,016	6,018
1557755_at	6,462	6,589	6,512	7,131	6,992	7,618
1557756_a_at	5,753	5,915	5,433	4,675	4,857	4,480
1557757_at	7,549	7,349	7,581	7,790	7,691	8,205
1557758_at	4,948	5,130	5,199	5,244	5,481	5,332
1557759_at	5,530	5,837	5,893	5,497	5,558	5,887
1557761_s_at	6,781	6,743	6,682	7,429	7,100	7,448
1557762_at	3,509	3,609	3,647	4,304	4,080	4,200
1557763_at	4,362	4,262	4,465	4,371	4,011	4,246
1557765_at	4,696	5,307	4,118	3,667	3,655	3,632
1557767_at	6,189	5,892	5,996	6,818	6,515	6,662
1557768_at	7,154	7,136	7,250	7,700	7,554	7,809
1557769_at	6,234	6,099	6,353	7,155	6,210	6,622
1557770_at	5,654	5,952	6,043	5,595	5,568	5,704
1557771_at	6,377	6,358	6,387	7,231	7,161	7,255
1557772_at	3,829	3,787	3,875	3,423	3,557	3,517
1557774_at	7,405	7,199	7,470	7,752	8,121	8,004
1557775_a_at	4,715	4,620	4,476	4,065	4,376	4,157
1557776_at	4,898	4,637	4,936	5,482	5,960	5,573
1557777_at	6,986	6,759	7,011	7,311	6,956	7,088
1557778_at	4,698	4,857	4,787	5,613	4,982	5,608
1557779_at	4,636	4,422	4,438	4,412	4,555	4,448
1557780_at	7,307	6,203	6,654	6,448	6,722	6,666
1557782_s_at	6,507	6,732	6,543	6,016	5,681	5,955
1557783_at	6,206	6,254	6,058	6,736	6,306	6,678
1557786_s_at	6,698	7,163	7,060	6,942	6,758	6,891
1557787_at	4,377	4,197	4,722	4,892	5,061	5,206
1557788_a_at	6,075	5,895	5,961	6,206	5,765	6,381
1557789_at	5,614	5,907	5,773	5,825	5,898	5,902
1557790_at	7,097	6,982	7,302	7,596	7,568	7,727
1557791_at	5,076	5,177	4,755	5,456	4,688	4,795
1557793_at	4,932	5,286	5,035	5,343	5,245	5,244
1557795_s_at	7,439	7,461	7,427	8,030	8,207	8,200
1557796_at	5,535	5,360	5,390	5,728	5,599	5,894
1557797_a_at	5,946	5,298	5,287	6,524	7,426	6,208
1557798_at	4,498	4,357	4,616	4,547	4,487	4,987
1557799_at	7,575	7,555	7,977	7,561	6,774	7,251
1557801_x_at	7,742	7,671	7,911	7,717	7,080	7,471
1557802_at	4,955	4,763	4,894	4,946	4,560	4,682
1557803_at	5,411	4,817	4,341	4,491	4,386	4,201
1557804_at	7,690	6,863	6,922	4,950	5,070	4,914
1557805_at	4,263	4,892	4,096	4,472	4,295	4,362
1557807_a_at	5,977	5,616	5,595	7,068	7,180	7,349
1557809_a_at	7,992	8,052	8,509	6,379	6,355	6,318
1557810_at	7,241	7,178	6,355	5,139	5,409	5,204
1557811_a_at	6,201	6,129	6,192	6,108	6,211	6,239
1557812_a_at	5,671	5,011	5,304	5,764	5,345	5,352
1557813_at	9,184	8,981	8,851	8,061	9,222	7,969
1557814_a_at	8,452	8,663	8,674	8,377	9,112	8,423
1557816_a_at	8,030	8,007	8,486	7,984	7,862	7,929
1557817_a_at	8,952	8,444	8,989	8,960	9,383	9,341
1557818_x_at	7,316	7,274	7,327	8,400	7,838	7,996
1557820_at	12,727	12,596	12,851	12,462	12,580	12,225
1557821_at	4,646	4,708	4,717	4,920	4,705	4,734
1557822_at	3,521	3,587	3,353	3,725	4,022	3,756

1557823_s_at	4,513	4,402	4,295	4,477	4,385	4,596
1557825_at	6,817	7,025	7,208	7,368	7,224	7,294
1557826_at	5,958	6,536	6,359	6,624	6,410	6,415
1557827_at	6,548	6,693	6,694	6,683	6,710	6,755
1557828_a_at	6,762	6,156	6,654	3,903	6,006	4,846
1557829_at	4,450	4,660	4,778	4,758	4,753	4,597
1557830_at	7,770	7,530	7,850	5,626	6,010	5,675
1557832_at	3,512	3,653	3,706	3,646	3,671	3,551
1557833_at	6,323	6,187	6,583	6,835	6,748	6,349
1557835_at	6,356	5,642	5,781	5,519	5,524	5,457
1557836_at	5,762	5,658	5,843	5,715	5,370	5,700
1557837_a_at	4,695	4,662	4,691	4,937	5,011	4,950
1557838_at	4,081	4,249	4,361	4,444	4,660	4,626
1557840_at	7,152	6,866	7,110	7,686	7,742	7,965
1557841_at	6,588	6,591	6,633	7,037	7,017	6,963
1557842_at	3,833	3,947	4,472	4,000	3,998	3,908
1557843_at	4,137	4,377	4,327	4,419	4,371	4,470
1557845_at	4,116	4,203	4,404	4,188	4,192	4,262
1557848_at	7,276	7,394	7,338	7,999	7,869	7,882
1557849_at	3,653	3,387	3,422	3,854	3,733	3,766
1557850_at	6,143	5,848	6,109	6,794	6,683	6,623
1557852_at	6,893	6,736	7,309	6,078	7,320	6,346
1557856_at	4,254	4,665	4,247	4,865	4,700	5,030
1557857_a_at	4,875	4,688	4,418	5,102	4,597	4,876
1557861_at	4,442	4,218	4,705	5,013	4,766	5,168
1557862_at	4,448	4,156	4,053	5,504	5,233	5,497
1557863_at	3,909	3,641	3,806	4,042	3,842	3,721
1557864_x_at	3,803	3,942	3,845	4,125	4,270	4,168
1557865_at	4,196	4,430	4,758	4,677	4,351	4,722
1557866_at	6,650	6,630	6,532	7,453	7,218	7,396
1557867_s_at	7,966	7,897	7,910	8,375	8,222	8,329
1557869_at	4,863	4,786	5,031	5,299	5,433	5,513
1557871_at	3,696	3,796	3,774	4,463	4,371	4,521
1557873_at	5,765	5,430	5,816	6,440	6,566	6,534
1557874_at	4,688	4,873	4,818	4,884	4,606	4,786
1557875_at	5,155	5,331	5,197	5,219	5,664	5,524
1557876_at	3,821	4,005	3,572	5,387	4,795	5,366
1557877_s_at	4,866	4,314	4,631	4,914	4,790	4,750
1557878_at	7,367	7,654	7,470	7,520	7,442	7,540
1557879_at	6,515	6,536	6,410	7,490	7,510	7,457
1557880_at	5,561	5,376	5,390	5,761	5,495	5,614
1557881_at	4,303	4,485	3,948	4,729	4,634	4,897
1557882_at	4,427	4,411	4,257	4,653	4,496	4,662
1557883_a_at	4,166	4,089	4,394	4,221	3,927	3,991
1557885_at	5,914	5,847	5,914	5,869	5,616	5,837
1557886_at	7,176	7,141	6,995	7,636	7,566	7,693
1557888_at	6,858	6,743	6,441	7,502	7,094	7,342
1557889_at	7,325	7,058	7,505	6,776	7,084	6,935
1557890_at	4,333	4,665	4,658	5,055	4,949	5,161
1557891_s_at	4,319	4,138	4,230	4,898	5,371	4,970
1557892_at	7,689	7,486	7,706	7,883	7,937	8,162
1557893_a_at	5,840	5,586	6,094	6,895	6,560	6,591
1557895_at	4,749	4,675	4,398	3,820	4,534	4,143
1557896_at	5,051	5,153	4,963	5,252	5,369	5,663
1557897_at	7,668	7,559	7,644	8,136	8,177	8,257
1557898_at	5,110	5,697	5,554	5,392	5,521	5,325

1557900_at	5,343	5,344	5,335	6,121	5,767	5,855
1557905_s_at	6,105	6,819	6,685	9,630	8,196	9,215
1557906_at	5,973	5,610	5,682	6,149	6,065	5,882
1557907_x_at	5,372	5,105	5,274	6,240	6,001	6,164
1557908_at	5,445	5,324	5,636	5,878	5,755	5,951
1557910_at	11,138	10,743	10,790	9,684	8,127	9,904
1557914_s_at	4,783	5,099	4,968	5,440	5,106	5,367
1557915_s_at	9,616	10,035	10,120	12,195	11,567	11,976
1557918_s_at	9,377	9,067	8,570	7,189	7,762	7,530
1557919_a_at	5,934	5,923	5,825	6,204	6,225	6,082
1557921_s_at	4,420	4,265	4,298	4,481	4,466	4,195
1557924_s_at	5,710	5,465	5,510	6,194	6,093	6,240
1557938_s_at	7,200	7,071	7,206	7,875	7,708	8,340
1557943_at	6,732	6,775	7,009	7,424	6,754	7,127
1557944_s_at	7,980	7,990	7,884	8,248	7,905	8,098
1557945_at	5,669	6,245	5,578	5,294	4,989	4,913
1557948_at	7,088	7,655	7,187	7,115	7,029	7,218
1557950_at	8,237	8,488	7,621	6,815	7,476	6,578
1557953_at	8,486	8,475	8,314	6,760	7,816	7,020
1557954_at	10,323	10,346	9,695	7,315	7,757	6,971
1557961_s_at	6,626	7,247	6,078	6,364	6,406	6,627
1557963_at	7,958	7,342	7,667	7,287	7,738	7,391
1557964_at	8,244	8,189	8,303	8,786	8,737	8,773
1557965_at	9,519	9,370	9,436	8,817	8,894	8,788
1557966_x_at	9,495	9,382	9,349	8,820	8,890	8,785
1557970_s_at	6,852	7,145	7,012	7,681	7,612	7,630
1557981_at	6,449	6,318	6,405	6,990	6,979	7,221
1557984_s_at	7,805	7,898	6,708	6,428	6,140	5,863
1557985_s_at	9,357	9,259	8,816	7,293	7,269	6,679
1557986_s_at	6,183	6,204	6,480	6,623	6,536	6,853
1557987_at	9,987	9,047	9,512	7,147	9,396	7,237
1557991_at	5,206	5,955	5,470	6,125	5,740	5,934
1557993_at	4,109	4,132	4,496	5,445	5,633	5,596
1557994_at	5,942	6,378	6,199	7,082	6,912	7,152
1557996_at	8,576	7,553	8,007	7,160	7,583	7,298
1557998_at	4,763	4,204	4,792	4,998	5,642	5,420
1558000_at	6,208	6,259	5,849	7,107	6,667	6,859
1558001_s_at	5,482	5,779	5,795	6,535	6,457	6,687
1558002_at	9,593	10,429	10,427	6,529	8,223	7,463
1558007_s_at	6,542	6,296	6,140	6,875	6,445	6,804
1558009_at	3,926	3,747	4,224	4,040	4,026	3,947
1558010_s_at	5,051	4,932	5,216	5,268	5,145	5,345
1558011_at	4,222	4,614	4,557	4,316	4,739	4,322
1558014_s_at	5,963	6,302	5,190	5,462	6,229	5,041
1558015_s_at	7,985	8,065	7,434	7,712	7,179	7,497
1558017_s_at	7,895	7,567	7,235	5,550	6,925	6,020
1558019_at	6,682	6,626	6,436	5,935	6,785	5,663
1558020_at	6,770	6,428	6,304	7,586	7,228	7,297
1558021_at	4,170	4,379	4,105	4,821	4,722	4,526
1558027_s_at	8,756	8,975	8,734	6,114	6,915	6,513
1558028_x_at	8,234	7,956	8,229	6,807	7,040	7,523
1558034_s_at	5,761	5,285	5,369	5,746	5,079	5,360
1558041_a_at	7,360	7,868	7,408	7,239	7,434	7,663
1558044_s_at	8,090	7,959	8,465	7,649	7,387	7,639
1558045_a_at	7,277	7,389	7,240	5,684	4,967	5,573
1558046_x_at	6,816	6,813	6,914	7,016	6,495	6,787

1558048_x_at	3,964	4,312	3,867	5,870	3,425	3,725
1558050_at	6,475	6,684	6,731	7,433	7,306	7,272
1558052_at	6,187	6,049	5,796	6,234	6,195	6,265
1558053_s_at	5,700	5,808	5,916	5,586	5,574	5,369
1558062_at	4,935	5,084	5,037	5,607	4,990	5,371
1558066_s_at	7,118	7,206	7,341	7,190	7,214	7,365
1558075_at	3,995	3,966	4,089	3,934	3,977	4,135
1558076_at	4,577	5,501	4,755	4,680	4,274	4,312
1558077_s_at	7,198	7,232	7,352	5,793	5,969	6,139
1558078_at	6,850	6,021	6,178	5,514	5,861	5,367
1558080_s_at	7,767	7,579	7,436	8,708	9,670	8,489
1558081_at	6,091	6,389	6,417	7,269	7,353	7,139
1558082_at	4,277	4,833	4,019	3,859	4,163	3,823
1558088_a_at	6,687	5,909	5,815	5,422	5,519	5,557
1558093_s_at	8,730	8,816	7,842	7,082	6,865	7,289
1558094_s_at	7,075	7,177	7,504	7,529	7,373	7,865
1558097_at	11,960	11,662	11,911	9,204	9,989	8,951
1558101_at	5,517	5,826	5,401	5,679	5,411	5,493
1558102_at	9,818	9,530	9,450	8,208	7,969	7,705
1558103_a_at	9,315	9,389	8,990	7,592	7,730	7,206
1558105_a_at	6,723	6,719	6,173	6,573	7,174	6,752
1558107_at	3,526	3,731	3,359	3,674	3,740	3,896
1558109_x_at	5,295	5,362	5,418	6,047	5,567	5,764
1558111_at	5,894	6,131	5,722	5,857	7,707	7,528
1558113_at	6,696	6,314	6,579	7,365	7,120	7,181
1558115_at	6,965	7,894	7,017	7,374	7,321	7,675
1558116_x_at	6,728	7,592	6,892	7,290	7,240	7,687
1558117_s_at	7,713	7,400	7,536	6,382	6,785	6,494
1558118_at	7,237	7,287	6,944	7,252	7,099	7,493
1558119_at	6,885	7,017	6,870	7,492	7,139	7,328
1558120_at	5,877	5,503	6,194	6,310	5,970	6,195
1558121_at	4,208	4,162	4,020	4,230	4,259	4,370
1558122_s_at	5,161	5,245	4,819	5,614	5,378	5,635
1558123_at	6,095	5,978	6,015	6,960	7,107	7,161
1558124_at	5,836	5,705	6,419	5,669	5,731	5,656
1558128_at	6,524	6,429	6,336	6,803	7,007	6,635
1558135_at	5,885	5,997	6,126	5,456	5,538	5,606
1558136_s_at	8,317	7,992	8,072	7,109	6,607	7,289
1558139_at	7,755	7,501	7,818	7,731	7,538	8,293
1558140_at	6,542	6,773	6,593	7,245	7,319	7,357
1558142_at	9,598	9,481	9,617	6,674	8,602	6,535
1558143_a_at	8,794	9,076	9,496	7,119	6,275	6,456
1558144_at	6,060	6,051	5,813	6,640	6,877	6,930
1558147_a_at	8,646	8,386	8,588	8,860	8,873	8,738
1558148_x_at	6,831	6,833	7,001	7,530	7,279	7,428
1558152_at	6,359	7,110	7,182	9,972	9,865	10,307
1558154_at	7,321	7,169	7,306	7,903	7,761	7,928
1558160_at	5,362	5,265	5,625	6,032	5,925	6,071
1558163_at	6,194	6,491	4,801	4,564	3,908	4,166
1558164_s_at	8,749	8,919	7,926	6,842	6,308	6,831
1558166_at	4,728	5,535	5,789	5,968	5,630	5,624
1558167_a_at	6,389	6,868	6,851	7,046	6,712	7,092
1558168_at	6,486	6,209	6,517	6,943	6,768	6,998
1558170_at	4,433	4,046	4,478	4,673	4,657	4,738
1558173_a_at	10,016	9,789	10,261	9,477	9,623	9,619
1558174_at	7,586	7,405	7,350	7,250	7,277	7,501

1558176_at	6,997	6,841	6,919	7,549	7,391	7,474
1558177_at	4,541	4,832	4,936	4,981	4,671	4,792
1558179_at	6,825	6,496	6,357	7,091	7,024	7,115
1558181_at	6,520	6,695	6,630	6,792	6,887	7,084
1558183_at	5,427	5,377	5,581	5,490	5,326	5,559
1558184_s_at	5,438	5,589	5,929	4,730	5,011	4,815
1558185_at	4,394	4,152	4,174	4,707	4,979	4,848
1558186_s_at	4,292	4,527	4,391	4,317	4,327	4,250
1558189_a_at	5,760	5,830	5,946	6,524	6,185	6,749
1558193_at	6,302	6,721	6,217	6,205	6,213	6,384
1558195_at	6,050	6,183	5,745	6,350	6,205	6,341
1558199_at	5,567	5,334	5,684	7,543	10,765	9,240
1558201_s_at	9,175	9,418	8,914	8,451	9,037	8,325
1558202_at	4,408	4,361	4,441	4,232	4,287	4,335
1558208_at	6,737	7,363	6,863	7,042	7,042	7,312
1558210_at	5,426	5,101	5,499	6,526	6,834	6,853
1558211_s_at	6,310	6,329	6,113	6,722	6,763	6,992
1558212_at	7,976	9,193	8,747	7,446	7,645	7,551
1558214_s_at	7,159	7,143	7,056	5,522	5,481	6,170
1558215_s_at	7,492	7,106	7,197	6,608	6,420	6,661
1558216_at	5,417	5,542	5,126	5,660	5,400	5,625
1558217_at	5,104	6,008	5,186	4,709	4,863	4,992
1558220_at	5,107	4,705	4,724	4,547	5,044	4,635
1558225_at	3,574	3,741	3,640	3,797	3,637	3,507
1558226_a_at	3,291	3,693	3,750	3,622	3,491	3,567
1558230_at	7,491	7,709	7,435	8,074	7,430	7,849
1558233_s_at	9,294	9,501	9,237	8,728	8,431	8,697
1558234_at	4,383	4,195	4,490	4,394	4,467	4,467
1558236_at	8,129	7,604	8,166	7,629	8,061	7,656
1558237_x_at	8,227	7,571	7,941	8,007	8,238	7,795
1558238_at	5,699	5,851	5,994	7,242	7,111	7,138
1558247_s_at	5,292	5,223	5,338	5,729	5,483	5,720
1558248_at	6,990	7,144	6,817	7,073	7,131	7,025
1558249_s_at	7,018	6,459	6,352	7,326	7,612	7,421
1558250_s_at	9,141	8,576	8,663	6,785	8,447	6,410
1558251_a_at	5,864	6,163	5,900	5,802	5,815	5,777
1558253_x_at	6,586	6,736	6,264	5,872	6,179	6,217
1558254_s_at	8,133	8,503	7,964	7,094	7,065	6,879
1558256_at	6,996	7,249	7,039	6,658	6,352	6,541
1558262_at	4,736	5,099	5,107	5,379	4,968	5,078
1558273_a_at	6,007	6,286	5,767	6,256	6,333	6,408
1558275_at	8,429	7,980	8,117	7,672	8,326	7,857
1558277_at	5,999	5,978	6,031	6,483	6,267	6,657
1558279_a_at	6,619	6,658	6,399	6,605	7,111	6,344
1558280_s_at	5,317	5,298	5,838	5,588	6,670	5,862
1558281_a_at	6,481	6,072	6,262	7,698	7,512	7,684
1558289_at	6,139	5,999	6,126	6,555	6,529	6,603
1558290_a_at	7,252	6,928	7,802	7,541	7,969	7,931
1558292_s_at	9,016	8,527	8,856	9,425	9,031	8,779
1558293_at	5,342	6,253	4,716	4,717	4,875	4,581
1558295_a_at	5,529	5,286	5,422	5,388	5,325	5,468
1558299_at	7,865	7,900	7,793	7,373	7,344	7,344
1558300_at	4,453	4,298	4,609	4,598	4,353	4,362
1558301_a_at	3,833	4,078	3,822	4,231	4,163	4,454
1558304_s_at	9,240	9,292	9,332	8,995	8,506	8,920
1558305_at	6,124	6,073	5,811	6,474	5,808	6,235

1558306_at	5,705	5,617	5,891	6,033	6,309	6,232
1558308_at	5,378	5,320	5,307	7,717	7,231	6,795
1558310_s_at	5,342	5,127	5,508	5,822	5,949	5,964
1558315_s_at	4,907	4,896	4,633	5,130	4,736	5,104
1558320_at	4,808	4,410	4,893	5,257	4,724	5,254
1558322_a_at	8,387	7,378	8,219	5,034	5,035	4,929
1558323_at	4,459	4,923	4,939	4,952	4,941	4,883
1558324_a_at	6,716	6,635	6,965	7,762	7,443	7,643
1558327_at	7,178	6,742	6,940	7,636	7,677	7,348
1558329_at	7,967	7,663	7,887	8,453	7,702	8,087
1558330_x_at	7,980	7,998	8,020	8,376	7,876	8,000
1558331_at	6,127	5,604	6,055	6,555	6,673	6,678
1558333_at	6,919	6,924	6,756	7,271	7,248	7,338
1558334_a_at	7,273	7,347	7,353	7,727	7,487	7,811
1558336_at	4,800	5,072	4,651	5,513	5,319	5,314
1558337_at	5,967	5,708	5,734	6,050	5,841	6,150
1558338_at	4,946	5,134	4,943	5,148	5,085	4,990
1558340_at	7,482	7,749	7,805	7,885	7,711	8,214
1558342_x_at	7,426	7,423	7,334	7,819	7,737	8,070
1558343_at	4,912	4,752	4,940	5,016	4,922	4,953
1558345_a_at	6,842	6,885	6,986	8,079	7,172	7,979
1558346_at	5,211	5,117	5,045	4,761	4,889	4,725
1558348_at	6,655	6,641	6,440	5,532	5,837	5,612
1558354_s_at	4,172	4,246	4,087	4,198	4,583	4,337
1558356_at	5,442	5,388	5,470	5,836	5,645	5,764
1558365_at	5,779	5,759	5,693	4,484	4,666	4,641
1558368_s_at	8,821	8,245	8,749	7,454	7,178	7,306
1558369_at	9,650	9,689	9,423	6,638	7,195	6,162
1558371_a_at	7,172	7,440	7,454	6,358	5,838	6,537
1558372_at	3,556	3,710	3,533	3,758	3,638	3,740
1558373_s_at	4,097	4,058	4,058	4,169	4,348	4,217
1558375_at	4,060	4,203	4,052	5,004	4,975	5,194
1558378_a_at	6,567	6,567	6,525	7,949	7,933	8,281
1558381_a_at	5,763	5,923	5,761	6,631	6,305	6,064
1558383_at	4,569	4,383	4,274	4,518	4,284	4,200
1558385_at	4,377	4,203	4,389	4,119	4,365	4,322
1558387_at	3,496	3,794	3,428	3,342	3,414	3,336
1558388_a_at	5,617	4,990	4,805	3,850	3,814	3,935
1558390_at	5,627	5,270	5,174	6,937	7,349	7,145
1558391_s_at	5,972	6,448	6,057	5,976	5,751	5,873
1558392_at	5,126	4,678	4,911	5,427	5,136	5,445
1558393_at	9,084	8,739	8,851	8,979	9,033	9,037
1558394_s_at	4,521	4,972	4,618	5,253	5,133	5,103
1558397_at	5,423	5,147	5,152	6,151	5,961	6,231
1558398_at	6,899	6,853	7,097	7,042	6,982	7,227
1558400_x_at	6,950	6,706	7,189	7,306	7,411	7,363
1558401_at	6,348	6,148	6,218	5,046	5,405	4,698
1558402_at	5,224	4,728	5,113	5,282	5,175	5,787
1558404_at	8,229	7,983	8,146	5,897	4,991	4,911
1558406_a_at	4,414	4,581	4,678	4,873	4,887	5,001
1558407_at	8,649	8,444	8,532	8,947	9,060	9,271
1558409_at	5,494	4,687	5,129	4,880	5,055	5,126
1558410_s_at	7,710	7,112	7,261	6,819	7,406	7,091
1558411_at	4,451	4,171	4,077	3,943	3,964	3,859
1558412_at	7,231	6,740	6,845	7,137	7,053	7,255
1558414_at	6,300	6,423	6,406	6,670	6,373	6,357

1558418_at	5,625	5,817	5,670	5,660	5,538	5,965
1558420_at	7,424	7,604	7,132	8,693	8,503	8,753
1558421_a_at	7,117	7,024	7,267	7,967	8,142	8,182
1558423_at	5,751	5,563	5,599	6,466	6,102	6,278
1558425_x_at	5,604	5,641	5,501	6,489	6,103	6,541
1558426_x_at	9,627	9,148	9,446	9,478	9,203	9,737
1558428_at	4,298	4,405	4,381	4,477	4,935	4,870
1558430_at	5,639	5,341	5,339	4,969	5,032	5,123
1558431_at	8,608	8,575	8,561	9,366	9,178	9,559
1558436_a_at	6,424	6,737	6,869	5,783	5,744	5,869
1558437_at	6,207	6,423	6,450	6,953	6,896	7,074
1558438_a_at	7,088	6,743	6,987	7,421	7,527	7,210
1558440_at	5,787	5,985	5,673	5,830	5,762	5,828
1558444_at	7,730	8,063	7,978	6,647	6,778	6,818
1558445_at	7,668	7,609	7,853	5,348	6,358	5,461
1558447_at	5,144	4,943	4,586	4,857	4,809	4,849
1558448_a_at	5,374	5,051	5,165	5,413	5,512	5,549
1558449_at	5,107	4,955	5,156	6,223	5,676	6,280
1558450_at	6,286	6,772	6,445	7,047	7,006	7,050
1558451_at	4,377	4,720	4,329	4,310	4,136	4,265
1558452_at	4,908	5,079	4,990	4,962	4,994	5,069
1558458_at	7,188	7,284	7,278	7,002	7,236	7,356
1558459_s_at	8,546	8,838	8,615	8,241	8,763	8,817
1558460_at	6,412	6,312	6,559	7,916	6,781	7,213
1558463_s_at	6,716	6,617	6,600	7,329	6,994	7,340
1558466_at	4,836	4,938	4,709	3,949	4,737	4,076
1558467_a_at	7,626	7,295	7,545	6,234	7,179	6,370
1558469_at	6,021	6,131	5,952	6,080	6,089	6,125
1558470_at	7,612	7,417	7,394	8,414	8,067	8,299
1558473_at	4,419	4,892	5,114	4,434	4,414	4,420
1558474_at	6,525	6,171	6,153	6,492	6,791	6,738
1558475_at	7,083	6,925	6,823	7,580	7,283	7,653
1558476_at	6,731	5,965	5,965	5,974	5,896	6,109
1558477_at	5,395	5,398	5,186	5,691	5,800	6,019
1558480_at	3,905	3,842	3,983	3,805	3,654	3,733
1558481_s_at	5,057	4,969	4,931	5,602	4,990	4,697
1558483_at	5,960	5,691	5,638	5,900	5,577	5,919
1558484_s_at	7,400	7,371	7,081	7,611	7,683	7,750
1558486_at	10,112	9,688	9,891	7,199	8,035	7,341
1558487_a_at	9,108	8,804	10,132	10,175	9,863	10,160
1558489_at	4,028	4,097	4,351	4,226	4,679	4,245
1558493_at	4,583	4,787	4,891	4,977	4,896	5,104
1558494_at	4,530	4,756	4,795	5,132	4,960	5,023
1558496_at	3,904	3,867	3,919	4,112	4,154	4,117
1558497_a_at	4,628	4,744	4,806	5,136	5,035	5,106
1558501_at	4,032	4,308	4,129	3,854	3,671	3,884
1558502_s_at	6,492	6,498	6,660	6,890	7,077	7,075
1558504_at	7,071	6,651	6,858	6,688	6,788	6,623
1558507_at	5,257	5,655	5,675	6,408	6,363	6,659
1558508_a_at	7,646	7,833	8,602	9,333	8,245	9,183
1558511_s_at	9,252	9,353	9,041	9,447	10,122	10,551
1558512_at	6,329	5,842	5,262	4,847	4,884	4,898
1558515_at	8,965	7,527	8,132	6,062	6,360	6,103
1558517_s_at	6,824	6,677	6,855	7,160	7,540	7,603
1558518_at	5,956	6,109	6,085	6,361	6,220	6,210
1558519_at	3,749	3,740	3,802	3,871	3,837	3,840

1558522_at	9,232	8,946	9,154	6,719	6,820	6,161
1558523_at	5,474	5,871	5,130	4,608	4,729	4,722
1558525_at	6,880	6,704	6,644	7,559	6,916	7,288
1558527_at	5,277	5,216	5,123	5,894	5,884	6,273
1558529_s_at	6,242	6,168	6,839	6,394	6,530	6,607
1558530_at	6,993	6,696	7,374	8,874	8,909	9,274
1558532_at	6,186	6,014	5,748	5,915	6,091	6,512
1558533_at	7,286	6,872	7,071	7,350	7,325	7,474
1558534_at	5,961	6,170	6,061	5,738	5,625	5,791
1558537_x_at	5,046	5,167	5,112	5,232	5,302	5,305
1558540_s_at	6,697	6,833	6,881	6,721	5,887	6,408
1558541_at	5,185	5,089	5,150	5,206	5,227	5,282
1558544_at	7,617	7,445	7,657	8,074	8,114	8,229
1558546_at	7,815	7,643	7,921	7,749	7,290	7,683
1558549_s_at	5,342	5,462	5,676	6,204	6,674	6,396
1558552_s_at	4,223	4,430	3,866	5,197	5,246	5,103
1558553_at	6,441	6,646	6,623	6,780	7,005	6,772
1558556_at	6,942	6,769	6,652	6,634	6,759	6,798
1558557_at	4,966	4,637	4,933	4,987	5,317	5,205
1558560_s_at	5,428	6,229	5,257	4,895	5,160	4,798
1558561_at	6,003	6,019	6,393	6,179	6,376	6,344
1558565_at	5,831	5,585	5,055	6,330	6,292	6,328
1558568_a_at	7,372	6,769	7,348	7,434	7,301	7,533
1558569_at	6,284	6,383	6,181	6,225	6,726	5,966
1558570_at	5,150	5,572	6,026	5,983	6,201	6,206
1558571_at	4,444	4,513	4,534	5,007	4,921	4,855
1558573_at	6,345	5,986	6,106	6,518	6,366	6,292
1558574_at	6,517	6,509	6,698	6,864	6,774	6,965
1558577_at	5,173	6,089	5,133	5,257	5,106	5,615
1558578_a_at	4,888	4,597	4,989	5,057	4,890	4,992
1558579_at	4,388	5,055	3,994	3,580	3,658	3,636
1558581_at	7,392	6,733	6,924	7,417	7,348	7,474
1558584_at	7,727	7,817	7,739	8,102	8,119	8,137
1558586_at	5,496	4,916	5,398	4,712	4,587	4,567
1558587_at	4,580	4,726	4,633	4,548	4,469	4,479
1558588_at	7,241	7,065	7,149	6,939	6,942	6,983
1558589_at	5,909	6,096	5,572	6,737	6,716	6,889
1558590_at	7,010	7,051	7,296	7,486	7,674	7,732
1558592_at	4,700	4,408	4,801	4,418	4,548	4,543
1558594_at	6,465	6,539	6,637	7,761	7,932	7,517
1558595_at	3,889	3,617	3,797	3,795	3,670	3,559
1558599_at	4,758	5,577	5,023	4,731	5,073	5,178
1558600_a_at	6,009	6,834	6,107	5,117	5,222	5,122
1558601_at	5,002	5,435	5,009	5,036	4,968	5,535
1558602_a_at	5,653	5,674	6,103	6,265	6,143	6,152
1558603_at	5,326	5,488	5,430	5,922	5,762	5,734
1558604_a_at	7,485	7,300	7,221	7,831	7,570	7,805
1558605_at	9,091	9,482	9,644	6,754	7,257	6,959
1558606_s_at	5,151	5,395	5,415	4,042	4,113	4,336
1558607_at	5,428	5,559	5,678	7,232	7,122	7,076
1558608_a_at	5,485	5,454	5,476	6,702	6,996	7,008
1558611_at	5,981	5,407	5,891	6,665	5,909	6,219
1558612_a_at	5,533	5,717	5,716	6,025	5,841	6,221
1558613_at	6,870	6,574	6,598	6,959	6,832	6,913
1558619_at	7,751	7,783	7,709	8,309	8,471	8,556
1558620_at	8,796	8,633	8,644	7,986	8,434	7,829

1558621_at	6,087	5,946	6,313	6,130	6,250	6,277
1558622_a_at	5,209	5,924	5,341	5,222	5,736	5,445
1558623_at	4,889	4,364	4,834	5,236	4,992	5,561
1558624_at	5,764	4,755	4,664	5,492	5,253	5,228
1558626_at	6,128	6,344	5,805	6,212	6,401	6,771
1558628_at	5,618	5,344	5,102	5,789	5,811	5,741
1558630_at	7,411	7,301	7,603	7,259	7,412	7,434
1558631_at	6,258	6,259	6,371	6,632	6,404	6,794
1558636_s_at	3,695	4,300	3,805	4,458	4,350	4,195
1558640_a_at	4,740	4,593	4,985	4,727	4,739	4,686
1558641_at	11,484	11,202	11,361	11,784	11,384	11,487
1558643_s_at	5,466	5,504	5,679	6,021	6,022	6,366
1558645_at	4,214	3,883	4,170	4,408	4,502	4,453
1558647_at	7,273	7,323	7,182	7,762	7,446	7,836
1558648_at	6,712	6,659	6,849	7,632	7,552	7,808
1558649_at	5,875	6,243	5,949	5,949	5,896	6,137
1558651_at	4,630	4,374	4,560	4,121	4,203	4,487
1558652_at	5,856	5,316	5,938	5,738	5,427	5,652
1558653_at	4,710	4,820	5,016	5,038	4,798	4,972
1558654_at	5,689	5,955	5,961	7,519	7,732	7,900
1558658_at	3,759	3,902	4,137	4,578	4,365	4,113
1558660_at	5,104	4,905	5,103	5,191	5,041	5,229
1558661_at	5,532	5,489	5,211	5,704	5,447	5,613
1558662_s_at	5,099	4,382	4,724	5,221	4,947	5,216
1558666_at	6,236	5,724	6,117	7,028	6,861	6,901
1558667_at	3,779	3,948	3,971	3,931	3,870	4,080
1558668_s_at	3,866	3,944	4,142	4,326	3,917	4,128
1558670_at	7,699	7,297	7,523	7,982	8,144	8,337
1558672_at	4,727	4,739	5,249	4,789	4,803	4,610
1558673_s_at	6,728	6,817	7,051	6,848	6,658	6,699
1558675_s_at	7,902	8,234	7,298	6,656	8,275	6,109
1558678_s_at	12,374	12,199	11,790	9,732	12,788	11,833
1558679_at	6,058	5,702	5,964	6,745	6,566	6,884
1558680_s_at	4,461	4,440	4,595	4,954	5,446	5,030
1558682_at	4,502	4,434	4,822	6,827	6,396	6,893
1558683_a_at	5,699	5,839	6,435	8,850	8,026	8,114
1558685_a_at	8,140	8,267	8,443	8,916	8,398	8,431
1558686_at	6,550	6,159	6,627	4,732	4,956	4,699
1558687_a_at	5,636	5,779	5,167	5,890	5,661	5,891
1558688_at	10,419	10,235	10,484	12,031	11,912	12,165
1558689_a_at	4,036	4,295	4,195	4,458	4,338	4,173
1558691_a_at	3,618	4,018	3,881	3,656	3,790	3,805
1558692_at	6,993	7,630	7,111	8,367	7,948	8,785
1558693_s_at	8,908	8,727	9,008	9,706	8,533	9,792
1558695_at	10,606	10,178	10,598	7,605	8,557	7,568
1558697_a_at	4,805	4,871	4,962	5,081	4,940	4,794
1558698_at	5,776	5,778	5,929	5,975	6,019	5,985
1558699_a_at	6,391	5,936	6,094	4,847	4,575	4,937
1558700_s_at	9,344	9,043	8,572	6,181	6,901	5,549
1558702_at	5,794	5,924	6,054	5,279	5,705	5,854
1558703_at	8,191	8,240	8,182	7,935	8,143	8,511
1558705_at	6,327	6,303	6,233	6,910	7,292	7,299
1558706_a_at	5,820	5,925	5,981	7,558	9,235	8,881
1558708_at	3,624	3,532	3,630	3,784	3,657	3,739
1558710_at	6,812	6,579	6,998	5,846	6,167	5,728
1558711_at	7,113	6,924	6,821	6,968	7,166	7,155

1558712_at	5,947	6,043	6,121	6,595	6,431	6,500
1558714_at	8,900	7,978	8,184	6,037	6,758	5,688
1558719_s_at	7,206	7,007	6,930	6,851	6,935	6,859
1558720_at	7,970	7,789	7,858	8,407	8,553	8,116
1558722_at	4,992	4,781	5,074	4,789	5,064	4,663
1558723_at	6,160	6,204	5,916	5,966	5,917	6,153
1558724_at	3,931	4,154	4,154	4,607	4,598	4,748
1558725_at	4,454	4,970	4,500	5,498	5,253	5,325
1558727_at	6,996	6,860	6,761	7,695	8,015	7,954
1558728_at	7,945	7,774	8,037	7,946	7,933	7,966
1558732_at	8,564	7,914	8,318	7,455	8,335	7,421
1558733_at	6,999	7,088	6,598	8,055	8,025	8,191
1558738_at	6,984	7,080	6,712	7,192	7,636	7,276
1558739_at	5,501	5,341	5,648	3,677	3,824	3,483
1558740_s_at	7,576	7,200	7,456	6,707	6,809	6,719
1558742_at	6,097	5,749	6,567	5,475	5,746	5,831
1558743_at	4,314	3,805	3,943	3,913	3,648	3,747
1558745_at	4,902	4,925	5,077	5,298	4,847	5,056
1558747_at	8,828	8,925	8,539	7,624	7,787	7,641
1558748_at	5,664	5,526	4,801	4,249	4,347	4,119
1558750_a_at	7,432	7,505	7,497	4,826	5,957	4,779
1558754_at	3,849	4,067	4,021	3,787	3,737	3,736
1558755_x_at	4,726	5,575	4,726	4,614	4,643	4,804
1558756_at	4,285	4,442	4,538	4,937	4,709	4,904
1558757_at	5,510	4,620	5,754	4,521	4,276	4,632
1558758_s_at	7,006	7,059	6,887	6,894	6,927	7,135
1558759_s_at	7,141	6,662	6,677	4,855	5,616	5,051
1558760_at	4,995	4,951	5,174	5,026	4,962	4,955
1558761_a_at	6,507	6,269	7,060	5,867	5,887	5,879
1558762_a_at	7,310	7,435	7,554	7,293	7,373	7,364
1558764_at	4,628	4,487	4,432	5,087	5,118	4,944
1558765_a_at	6,218	6,311	5,960	6,521	6,290	6,283
1558766_at	8,153	7,435	7,809	6,754	6,247	6,452
1558768_at	6,099	5,804	6,126	7,010	6,636	6,907
1558769_s_at	6,181	6,246	6,104	6,764	6,744	6,896
1558770_a_at	6,081	6,109	6,395	6,712	6,916	7,087
1558773_s_at	7,361	7,249	6,866	7,126	7,067	7,168
1558775_s_at	5,528	5,549	5,805	5,950	5,964	5,823
1558777_at	7,812	7,669	7,497	7,858	7,364	7,265
1558778_s_at	5,791	5,675	5,520	6,941	6,920	7,195
1558779_at	3,413	3,603	3,542	3,514	3,404	3,478
1558780_a_at	5,903	6,238	5,862	6,166	6,215	6,349
1558782_a_at	8,282	8,568	8,197	7,779	7,523	7,591
1558783_at	7,580	7,669	8,004	4,607	7,524	5,190
1558784_at	9,326	9,039	9,116	9,225	9,481	9,159
1558785_a_at	6,933	6,539	6,518	6,997	6,917	6,833
1558786_at	3,794	4,329	4,342	5,007	4,899	5,343
1558787_a_at	4,720	4,681	5,326	4,747	4,854	4,739
1558790_s_at	4,701	4,392	4,664	5,155	5,047	5,336
1558791_at	6,863	6,675	7,048	6,345	5,980	6,301
1558792_x_at	7,858	7,321	7,706	7,317	7,729	7,175
1558793_at	3,910	3,842	3,943	4,603	4,033	4,060
1558794_at	6,016	5,889	6,642	5,559	5,427	5,643
1558795_at	6,032	6,168	5,058	6,844	6,927	6,896
1558796_a_at	4,425	5,432	4,932	4,965	5,053	5,135
1558797_at	3,936	4,086	4,234	4,527	4,448	4,283

1558801_at	6,953	6,639	7,074	6,364	6,289	5,450
1558802_at	7,063	7,110	7,160	5,114	6,329	5,166
1558803_at	4,895	4,959	5,038	5,485	5,399	5,441
1558804_at	4,794	4,458	4,810	4,663	4,688	4,629
1558806_at	5,286	5,477	5,440	6,116	6,057	6,066
1558807_at	5,128	4,948	4,647	5,019	4,701	4,774
1558809_s_at	7,066	7,137	7,165	5,899	5,925	6,194
1558813_at	4,379	4,001	4,173	4,062	3,992	4,190
1558814_s_at	8,063	8,033	7,821	8,361	8,121	8,000
1558815_at	5,976	5,719	5,924	5,627	6,535	5,819
1558816_at	8,225	8,240	8,485	7,702	7,339	7,485
1558819_at	6,776	6,631	6,687	4,735	4,951	4,986
1558820_a_at	6,631	6,659	7,214	7,192	7,160	7,359
1558821_s_at	7,454	7,758	7,842	7,535	7,650	7,613
1558822_at	8,095	7,921	8,341	7,615	8,086	7,786
1558825_s_at	6,665	6,357	6,767	7,630	7,380	7,483
1558826_at	3,791	3,700	3,805	4,458	4,626	4,694
1558827_a_at	4,557	4,804	4,480	4,758	4,578	4,720
1558828_s_at	5,610	5,200	5,296	5,114	5,223	5,470
1558830_at	4,212	4,085	4,545	4,516	5,527	4,964
1558831_x_at	7,141	6,571	6,719	6,546	7,243	6,817
1558832_at	7,431	7,236	7,133	7,597	8,124	7,633
1558834_s_at	3,870	4,078	3,533	4,379	4,312	4,595
1558836_at	7,235	7,435	7,654	6,743	7,660	7,004
1558837_a_at	8,139	8,152	8,467	7,785	8,578	8,191
1558839_at	5,971	5,946	6,170	6,729	6,830	6,981
1558841_at	5,996	6,212	6,129	5,996	5,871	6,017
1558842_at	6,570	6,472	6,463	3,761	3,828	4,017
1558844_at	6,852	6,835	6,806	7,563	7,210	7,373
1558845_at	5,339	4,788	5,084	5,579	5,467	5,759
1558846_at	4,234	3,890	4,233	5,144	5,133	5,254
1558847_at	6,615	6,489	6,605	7,801	7,051	7,172
1558848_at	6,150	6,127	5,765	6,946	7,019	7,184
1558849_at	6,514	6,892	6,965	6,743	6,628	6,752
1558850_s_at	6,911	6,380	6,865	7,339	7,299	7,105
1558852_at	3,905	3,834	4,049	3,792	3,775	3,824
1558854_a_at	4,797	4,927	5,189	3,702	3,986	3,814
1558855_at	5,598	5,927	5,925	6,289	6,250	6,336
1558856_at	6,936	7,012	6,985	7,846	7,565	7,751
1558857_at	4,954	4,672	4,801	5,206	4,828	5,203
1558858_at	4,894	4,974	5,105	4,970	5,330	5,303
1558859_at	5,255	5,278	5,103	5,255	5,167	5,181
1558862_at	5,343	5,129	5,026	5,306	5,203	5,339
1558867_at	4,236	3,678	4,212	4,323	4,771	4,722
1558868_a_at	5,648	5,582	5,384	6,404	6,659	6,634
1558869_at	5,120	5,359	5,383	5,560	5,875	5,710
1558871_at	4,928	4,897	4,616	5,840	5,762	5,735
1558874_a_at	6,585	6,683	6,352	6,745	6,689	7,125
1558875_at	6,521	6,311	6,506	7,174	6,991	7,212
1558876_at	6,884	7,176	7,213	8,502	8,422	8,271
1558877_at	5,620	5,122	5,949	4,758	4,805	4,875
1558881_at	6,620	6,201	6,617	6,956	6,634	6,223
1558882_at	4,383	4,233	4,172	4,976	4,953	5,289
1558885_at	5,238	5,277	5,349	5,720	5,394	5,559
1558887_at	4,401	4,016	3,927	3,990	3,981	3,924
1558888_x_at	4,296	4,165	4,378	4,040	3,996	3,951

1558889_at	6,714	6,756	6,898	6,806	6,687	6,878
1558890_at	7,318	6,992	6,831	8,335	8,252	8,429
1558893_a_at	3,496	3,401	3,691	4,608	4,092	4,736
1558894_a_at	5,840	5,804	5,599	6,183	6,015	6,199
1558896_at	6,440	6,453	6,518	6,516	6,138	6,314
1558897_at	6,599	6,464	6,744	7,040	6,904	7,060
1558899_s_at	6,026	5,968	6,269	7,146	6,916	7,182
1558900_at	3,355	3,353	3,481	3,534	3,452	3,485
1558903_at	5,579	5,676	5,861	6,042	5,888	5,842
1558905_at	3,954	3,971	3,825	4,044	3,909	4,165
1558906_a_at	6,185	6,313	6,293	6,371	6,302	6,399
1558914_at	8,011	8,101	7,999	8,825	8,077	8,220
1558915_a_at	6,096	5,812	5,801	6,318	6,009	6,151
1558919_a_at	6,562	6,728	6,621	6,280	6,365	6,188
1558920_at	4,221	4,469	4,509	4,866	4,715	4,894
1558922_at	6,703	5,708	5,966	3,597	3,663	3,654
1558924_s_at	7,768	7,832	7,370	7,633	7,903	7,523
1558930_at	7,254	7,156	7,031	8,273	7,734	8,063
1558931_at	4,749	4,629	4,545	5,009	5,396	5,530
1558934_a_at	5,692	5,322	5,414	5,892	5,396	5,630
1558937_s_at	5,603	5,332	5,762	4,746	5,167	5,011
1558938_at	6,189	6,716	6,501	6,399	6,423	6,473
1558941_at	5,319	5,055	5,310	5,834	5,620	5,819
1558942_at	9,220	9,071	8,453	7,622	7,641	7,990
1558943_x_at	9,424	9,154	9,376	7,167	7,706	6,528
1558944_at	5,032	5,190	4,872	6,560	6,727	6,861
1558945_s_at	4,650	5,015	4,880	5,887	4,837	4,796
1558947_at	5,190	5,168	4,641	5,894	5,312	5,517
1558948_a_at	4,577	4,309	4,515	4,496	4,724	4,845
1558949_at	6,174	6,247	6,217	6,650	6,665	6,749
1558950_at	4,922	5,169	4,974	6,466	6,388	6,520
1558953_s_at	9,595	9,342	9,796	9,174	9,738	9,339
1558954_at	8,019	8,182	8,274	7,918	7,981	8,185
1558956_s_at	10,622	10,906	9,729	8,027	9,641	7,512
1558957_s_at	3,481	3,658	3,374	3,658	3,580	3,694
1558959_at	4,974	5,122	4,793	5,402	5,194	5,358
1558960_a_at	6,399	6,375	6,674	6,753	6,997	6,962
1558963_at	5,615	5,982	5,812	7,008	6,906	7,271
1558964_at	6,348	5,362	7,141	4,733	4,815	4,725
1558965_at	6,083	6,139	5,737	6,017	5,814	5,986
1558967_s_at	7,273	7,315	7,498	7,530	6,974	7,022
1558968_at	4,999	4,996	4,719	4,786	4,764	4,606
1558969_a_at	8,016	7,953	8,194	8,011	7,503	7,960
1558971_at	4,069	4,214	3,927	3,735	4,088	3,827
1558972_s_at	3,670	3,772	3,663	3,788	3,712	3,812
1558975_at	6,654	6,922	6,619	6,601	6,867	6,996
1558976_x_at	7,098	7,166	6,931	6,969	7,256	7,534
1558977_at	4,150	4,462	4,628	4,407	4,250	4,374
1558982_at	5,663	5,689	5,647	7,207	7,102	7,048
1558983_at	5,308	4,678	5,445	4,975	4,723	4,715
1558984_at	6,716	6,705	6,788	7,028	6,627	6,865
1558987_at	4,832	4,941	5,066	5,109	4,899	5,167
1558990_at	4,971	4,465	4,395	4,268	4,277	4,264
1558995_at	4,498	4,619	4,707	4,583	4,574	4,512
1558996_at	7,315	6,874	7,207	7,418	7,774	7,158
1558999_x_at	4,007	3,556	3,863	3,461	3,519	3,478

1559000_at	6,882	7,157	6,861	7,357	7,226	7,089
1559002_at	7,774	7,461	7,946	7,699	7,081	7,216
1559003_a_at	7,687	7,849	8,054	7,512	7,733	7,750
1559005_s_at	6,464	6,899	6,048	5,522	5,512	5,765
1559006_at	8,425	7,836	8,600	6,273	6,289	6,008
1559007_s_at	7,215	6,009	7,383	5,638	5,779	5,693
1559009_at	4,811	4,953	4,912	5,284	4,702	4,875
1559011_at	6,588	5,926	6,566	6,284	6,427	6,446
1559017_at	6,608	6,152	6,017	6,262	5,993	6,221
1559018_at	5,497	5,544	5,633	5,639	5,693	5,660
1559020_a_at	6,965	6,468	6,689	7,163	7,245	7,461
1559021_at	6,216	6,356	6,168	6,095	6,299	6,077
1559022_at	6,778	5,592	6,042	5,287	5,169	5,274
1559023_a_at	8,518	8,489	8,475	7,106	7,248	7,286
1559025_at	6,034	5,543	5,609	5,773	6,126	6,009
1559026_at	6,990	6,529	6,058	6,285	6,379	6,306
1559028_at	7,380	7,351	7,371	7,417	7,408	7,552
1559029_at	6,108	6,310	6,319	7,070	6,820	7,003
1559030_a_at	5,129	5,193	5,638	5,470	5,310	5,171
1559031_at	6,528	6,598	6,269	6,947	7,082	7,071
1559033_at	6,122	5,886	6,397	6,516	6,150	6,551
1559034_at	5,680	5,316	5,577	6,149	6,250	6,207
1559035_a_at	4,403	4,334	4,171	4,404	4,201	4,290
1559036_at	5,413	5,430	5,628	6,197	6,131	6,000
1559037_a_at	6,933	6,210	6,434	6,548	7,000	6,493
1559038_at	9,943	9,680	9,310	6,975	8,657	7,652
1559039_at	7,318	7,355	7,133	6,486	7,040	6,569
1559042_at	5,821	6,440	6,284	4,224	4,379	4,004
1559044_at	5,257	5,162	4,960	4,313	4,845	4,590
1559045_at	4,569	4,151	4,128	4,081	4,235	4,363
1559048_at	9,003	8,765	8,800	9,371	9,068	9,131
1559049_a_at	5,625	6,254	6,274	6,588	6,630	6,874
1559050_at	5,564	5,837	5,838	6,829	6,647	6,373
1559051_s_at	6,989	6,814	6,993	7,838	6,480	7,193
1559052_s_at	6,823	6,909	7,155	6,766	6,722	6,551
1559053_at	6,194	6,188	5,996	6,274	6,545	6,323
1559054_a_at	7,447	6,596	6,890	7,102	7,698	7,104
1559057_at	4,659	5,195	4,742	4,824	4,774	5,015
1559059_s_at	5,222	5,497	5,683	5,161	5,221	5,191
1559060_a_at	5,344	5,839	5,893	6,133	6,872	6,565
1559061_at	7,023	7,253	7,203	7,827	7,873	7,605
1559062_at	5,431	5,279	5,968	4,185	4,590	4,449
1559063_at	5,973	5,862	6,118	6,311	6,615	6,512
1559064_at	6,542	6,531	6,394	7,818	6,996	7,567
1559065_a_at	6,054	5,802	5,768	6,912	7,322	6,984
1559066_at	4,187	4,602	5,084	5,960	5,954	6,200
1559067_a_at	6,357	6,429	6,762	4,866	5,567	5,277
1559069_at	4,424	4,479	5,406	4,409	4,216	4,273
1559072_a_at	6,187	7,079	6,252	7,362	6,431	6,915
1559073_at	6,828	6,457	6,579	6,749	6,657	6,801
1559075_s_at	5,900	5,824	6,200	6,294	6,443	6,344
1559077_at	4,371	4,448	4,775	5,874	5,381	4,870
1559078_at	8,326	7,734	8,347	6,821	6,647	6,640
1559079_at	5,177	5,328	5,331	5,909	6,221	6,048
1559083_x_at	7,593	7,142	7,249	8,314	8,207	8,345
1559086_at	5,046	4,602	4,960	5,135	5,143	5,098

1559091_s_at	7,319	7,253	7,509	8,242	8,127	8,290
1559092_at	6,637	6,946	6,775	8,050	7,928	8,075
1559093_at	5,553	5,705	6,086	6,047	6,104	6,118
1559094_at	5,306	5,156	4,806	4,703	5,238	4,676
1559096_x_at	6,083	5,449	5,439	5,407	5,986	5,264
1559097_at	6,035	6,079	6,118	6,305	6,693	6,345
1559101_at	7,421	7,229	7,370	6,838	7,379	6,926
1559102_at	5,964	6,406	6,224	5,818	5,783	5,704
1559103_s_at	4,401	4,131	4,613	3,676	3,849	3,974
1559105_at	5,583	6,276	5,898	5,712	5,346	5,493
1559108_at	6,684	6,818	7,119	6,054	6,377	6,166
1559109_a_at	5,834	5,789	6,037	5,758	5,914	6,019
1559110_at	7,047	6,816	7,165	6,602	6,743	6,812
1559111_a_at	7,427	7,101	7,126	6,444	6,609	6,521
1559114_a_at	5,654	5,659	5,693	6,195	6,310	6,451
1559116_s_at	6,990	6,913	6,856	7,838	7,203	7,147
1559117_at	6,501	6,644	6,192	6,972	6,848	6,672
1559119_at	6,041	6,219	6,035	6,202	5,931	6,197
1559121_s_at	6,227	5,968	7,047	5,584	5,421	5,292
1559124_at	3,960	4,187	4,147	4,575	4,379	4,575
1559125_at	4,256	4,152	4,344	4,879	4,765	5,003
1559126_at	6,420	6,540	6,683	7,132	7,276	7,175
1559127_x_at	6,972	7,013	7,078	7,232	7,527	7,385
1559128_at	5,032	4,963	4,834	5,005	5,405	4,686
1559129_a_at	5,480	6,168	5,906	6,213	5,812	6,114
1559131_a_at	4,805	4,944	4,871	5,217	5,395	5,825
1559132_at	7,483	7,273	7,279	8,303	7,512	7,669
1559133_at	3,571	3,519	3,653	3,834	3,818	3,834
1559134_a_at	4,050	4,006	4,109	4,432	4,377	4,301
1559136_s_at	7,704	7,596	7,681	7,961	7,784	8,161
1559138_a_at	6,394	6,452	6,303	6,823	6,653	6,760
1559139_at	6,293	6,057	6,127	6,125	6,216	6,292
1559140_at	6,233	6,341	6,754	7,017	7,310	6,967
1559141_s_at	5,154	5,191	5,127	5,410	5,616	5,538
1559142_at	5,332	5,202	5,072	5,200	5,517	5,540
1559144_x_at	4,998	5,187	5,209	6,045	5,757	5,662
1559145_at	5,728	5,791	5,552	6,637	6,295	6,704
1559147_at	3,959	4,075	4,503	4,224	4,200	4,261
1559148_at	7,658	7,303	7,173	7,193	7,196	7,142
1559149_at	6,409	6,152	6,536	5,955	5,701	5,652
1559151_at	4,772	4,659	4,843	4,774	4,765	4,550
1559152_at	4,686	4,680	4,726	5,326	5,110	5,395
1559154_at	5,999	5,879	5,562	5,968	5,985	5,817
1559156_at	8,867	7,647	7,575	5,171	6,967	5,306
1559159_at	6,899	7,118	7,112	7,375	7,445	7,380
1559161_at	5,843	6,351	6,433	5,855	5,593	5,748
1559163_at	3,665	3,888	3,805	3,514	3,465	3,559
1559166_at	6,441	6,404	6,420	6,683	6,455	6,858
1559167_x_at	9,798	9,663	9,840	9,932	9,869	9,991
1559169_at	5,852	5,973	5,960	6,451	6,372	6,632
1559170_at	5,764	6,254	6,157	7,073	6,624	6,875
1559171_at	6,239	6,500	6,344	6,406	6,484	6,473
1559172_at	4,253	4,045	3,975	4,431	4,364	4,673
1559174_at	6,597	5,786	5,760	6,338	6,348	6,664
1559180_at	4,525	4,609	4,600	4,792	4,816	4,757
1559186_at	5,719	5,891	5,889	6,264	6,219	6,342

1559188_x_at	6,440	6,519	6,703	7,101	7,276	6,858
1559190_s_at	7,943	8,206	8,109	6,838	7,076	7,361
1559194_a_at	5,683	5,344	5,480	6,173	5,765	6,089
1559195_at	6,932	6,789	7,103	7,316	7,157	7,509
1559201_a_at	7,919	7,605	7,963	7,778	8,057	7,924
1559203_s_at	5,631	6,138	5,973	4,330	4,209	4,205
1559204_x_at	7,756	8,738	8,321	7,778	7,693	7,853
1559205_s_at	7,702	7,510	7,712	7,883	7,819	7,954
1559206_at	4,466	4,191	4,280	4,742	4,661	5,013
1559208_at	3,865	4,501	4,216	3,963	3,948	3,971
1559210_at	6,659	6,285	6,659	6,808	7,107	7,011
1559213_at	3,643	3,716	3,958	3,736	3,621	3,707
1559214_at	6,122	6,010	5,134	3,459	3,762	3,544
1559218_s_at	7,083	6,891	7,244	7,369	6,786	7,459
1559220_at	6,939	7,042	6,968	7,797	7,759	7,674
1559221_at	4,512	4,307	4,661	4,849	5,497	5,534
1559222_at	4,738	4,692	4,651	4,542	4,378	4,386
1559224_at	7,388	7,532	7,798	7,912	7,880	7,875
1559226_x_at	7,630	7,520	7,579	8,207	8,121	8,158
1559227_s_at	6,467	6,515	6,975	6,716	6,678	6,536
1559228_at	6,390	6,133	6,567	7,986	7,729	7,790
1559229_at	6,263	6,321	6,175	6,762	6,365	6,347
1559232_a_at	6,727	6,414	6,662	6,285	6,368	6,245
1559235_a_at	4,993	4,798	4,937	5,026	4,924	5,155
1559237_a_at	6,808	6,741	6,838	7,916	7,569	7,776
1559239_s_at	5,427	5,788	5,515	5,678	5,830	5,802
1559240_at	6,723	7,052	6,851	7,140	6,597	6,832
1559244_at	6,954	6,268	6,659	6,858	6,827	6,711
1559245_at	7,649	7,529	7,594	8,041	7,715	7,671
1559249_at	5,252	5,458	5,535	5,117	5,860	5,483
1559252_a_at	5,858	5,518	5,623	6,730	7,059	7,082
1559254_at	5,326	5,447	5,614	5,982	5,790	5,993
1559255_a_at	4,785	5,030	4,879	5,338	5,408	5,220
1559256_at	4,566	4,565	4,968	4,629	4,518	4,522
1559257_a_at	7,121	7,163	7,191	6,732	6,407	6,541
1559258_a_at	5,028	4,955	4,796	4,979	5,048	5,107
1559259_at	5,274	4,963	4,801	4,582	4,828	4,726
1559261_a_at	6,848	6,564	6,721	7,207	6,980	7,133
1559263_s_at	3,987	4,195	3,935	4,217	4,014	4,109
1559264_at	3,750	3,824	4,011	3,964	4,074	4,107
1559265_at	3,971	4,865	5,007	3,851	3,944	3,996
1559266_s_at	5,480	5,047	5,939	5,196	5,095	5,018
1559268_at	4,924	4,314	4,711	5,979	5,061	5,191
1559270_at	5,305	5,083	5,101	5,136	5,202	5,475
1559272_at	7,405	7,036	7,459	7,868	7,595	7,898
1559274_at	3,696	3,748	3,805	4,721	4,315	4,687
1559275_x_at	3,695	3,584	3,597	4,249	4,134	4,151
1559276_at	5,085	5,155	5,551	5,198	5,222	5,162
1559277_at	4,377	4,625	4,694	5,047	5,214	5,192
1559278_at	4,666	4,566	4,125	4,692	4,606	4,681
1559280_a_at	8,766	9,220	8,296	3,868	4,050	3,904
1559282_at	4,021	4,016	4,008	4,099	4,005	4,062
1559283_a_at	4,540	4,650	4,443	4,851	4,506	4,751
1559284_at	3,934	3,823	4,092	4,213	4,241	4,203
1559285_at	3,634	3,814	3,851	3,878	3,719	3,912
1559287_at	4,260	4,225	4,449	4,635	4,488	4,329

1559288_at	4,627	4,991	4,293	4,618	4,625	4,602
1559289_at	4,997	4,928	5,279	5,814	5,604	5,379
1559291_at	6,857	6,933	6,862	7,058	7,005	7,221
1559292_s_at	4,708	5,260	5,370	5,333	5,352	5,442
1559293_x_at	5,966	6,101	5,901	6,374	6,336	6,488
1559295_at	6,556	5,923	6,374	6,835	6,632	6,905
1559296_at	5,266	5,010	5,296	5,441	5,495	5,322
1559297_at	5,620	5,746	5,420	6,109	6,035	6,262
1559298_a_at	6,266	6,164	6,173	6,225	6,113	6,386
1559302_at	6,074	5,733	6,277	6,920	6,809	6,776
1559303_at	6,214	6,318	6,501	7,068	6,875	7,099
1559304_at	4,665	4,605	5,054	5,364	5,281	5,228
1559307_s_at	5,360	5,149	5,016	6,310	5,556	5,998
1559309_at	5,080	5,217	5,445	6,260	5,862	5,967
1559310_at	4,292	4,509	4,371	4,625	4,453	4,622
1559311_at	7,194	7,149	7,200	7,374	7,378	7,517
1559313_at	5,396	5,828	6,020	6,414	6,490	6,703
1559315_s_at	5,800	5,700	5,710	6,174	5,805	5,846
1559316_at	4,952	5,157	5,039	5,819	5,780	5,987
1559321_at	7,412	7,164	7,433	8,007	7,490	7,455
1559322_at	5,066	5,543	5,531	5,324	5,128	5,594
1559324_at	5,973	6,090	6,563	7,454	7,469	7,606
1559326_at	7,124	6,401	7,090	7,643	7,630	7,753
1559331_x_at	4,690	4,823	4,998	5,681	5,708	5,973
1559332_at	7,230	6,918	7,199	6,442	7,073	5,899
1559333_at	6,501	6,430	6,139	6,380	6,317	6,112
1559336_at	3,572	3,917	3,873	3,904	3,842	3,632
1559340_at	5,711	5,679	5,749	6,581	6,531	6,606
1559341_at	3,617	3,714	3,583	4,020	3,913	4,036
1559342_a_at	4,200	4,208	4,265	4,523	4,239	4,394
1559343_at	5,502	6,076	6,102	5,260	5,570	5,068
1559344_at	6,816	6,693	6,932	7,356	7,053	7,154
1559347_at	5,767	5,885	6,121	6,039	5,830	5,784
1559348_a_at	5,950	6,279	5,798	6,553	6,326	6,107
1559350_at	6,636	6,329	6,168	6,801	6,926	6,993
1559351_at	8,393	7,763	8,154	8,303	8,395	8,516
1559352_a_at	7,489	7,682	7,796	8,287	7,802	8,290
1559353_at	3,409	3,271	3,305	3,506	3,411	3,499
1559354_a_at	6,727	6,421	6,799	7,303	7,060	7,394
1559355_at	5,905	6,146	6,319	6,455	6,653	6,527
1559360_at	8,655	7,606	7,736	5,191	7,018	5,645
1559361_at	6,062	6,146	6,234	6,182	6,329	6,322
1559362_at	4,426	4,487	4,633	4,609	4,713	4,604
1559363_at	3,714	3,690	3,952	4,005	3,801	3,810
1559369_at	5,026	5,124	4,944	5,029	5,217	4,946
1559370_at	3,695	3,799	3,890	4,076	3,936	3,982
1559372_at	5,497	5,096	5,186	5,795	5,760	5,679
1559374_at	5,417	4,923	4,758	5,207	5,007	5,217
1559375_s_at	5,449	5,222	5,966	6,086	6,244	6,246
1559376_at	7,408	7,639	7,190	8,259	8,420	8,607
1559377_at	5,091	4,307	4,152	5,345	5,295	5,733
1559382_at	7,163	6,979	7,576	8,635	7,821	8,110
1559384_at	4,477	4,493	4,406	5,141	4,992	5,181
1559388_a_at	6,970	7,103	7,240	7,619	7,109	7,556
1559390_a_at	5,530	5,740	5,813	5,065	4,897	5,101
1559391_s_at	8,735	8,097	8,723	4,827	6,249	5,149

1559392_s_at	6,293	6,110	6,250	6,978	6,324	6,940
1559393_at	4,772	5,650	5,232	6,939	7,122	6,937
1559394_a_at	8,414	7,175	8,538	5,569	8,204	5,782
1559397_s_at	7,081	5,709	5,969	6,664	6,575	6,820
1559399_s_at	6,419	5,968	6,177	7,935	7,252	7,568
1559400_s_at	8,033	8,130	7,787	8,999	8,670	8,689
1559401_a_at	8,548	7,425	7,961	5,884	6,403	6,061
1559402_a_at	7,505	7,356	7,383	7,675	7,733	7,684
1559403_at	5,224	5,128	5,297	5,048	5,201	5,121
1559404_a_at	4,283	4,678	4,379	5,754	6,159	6,170
1559405_a_at	6,472	6,066	6,787	7,517	7,490	7,652
1559406_at	4,846	5,260	5,063	4,816	4,770	4,887
1559409_a_at	5,499	5,224	5,112	6,064	5,997	6,212
1559410_at	7,282	6,920	7,263	3,955	4,085	4,214
1559412_at	5,066	5,370	5,192	5,768	5,955	5,758
1559413_at	5,437	5,419	5,198	5,911	6,103	5,864
1559419_at	4,668	4,569	4,613	4,963	5,135	4,961
1559420_x_at	4,556	4,375	4,323	4,651	4,847	4,683
1559421_at	6,831	7,012	7,136	7,055	7,197	6,899
1559425_at	6,737	6,528	6,605	6,448	6,365	6,035
1559426_at	6,954	7,100	7,052	7,352	7,459	7,487
1559427_at	6,176	6,067	6,108	6,602	6,435	6,525
1559429_a_at	6,613	6,117	6,602	7,613	7,084	7,388
1559432_at	6,051	5,980	5,858	6,229	6,192	6,280
1559433_at	4,821	4,496	4,778	5,661	4,944	5,664
1559434_at	3,941	4,119	3,866	4,420	4,348	4,236
1559436_x_at	10,110	9,725	9,864	9,546	10,269	9,859
1559437_at	6,318	6,128	6,280	6,999	6,786	6,780
1559438_at	7,016	6,785	7,125	7,308	7,424	7,608
1559439_s_at	4,669	4,811	4,653	4,703	4,774	4,819
1559441_s_at	4,410	4,189	4,333	4,349	4,374	4,315
1559443_s_at	7,650	7,299	7,873	7,847	7,697	7,860
1559444_at	7,441	7,049	7,418	7,595	7,428	7,243
1559449_a_at	7,417	6,777	7,091	5,510	5,577	5,715
1559450_at	5,552	4,828	4,359	5,900	5,687	5,738
1559452_a_at	6,619	6,225	6,546	6,980	7,228	6,931
1559455_at	3,782	4,105	3,905	3,825	3,977	4,151
1559458_at	5,221	5,586	5,477	5,700	5,757	6,027
1559459_at	4,974	4,841	5,112	5,228	5,077	5,121
1559461_at	7,913	7,397	7,727	8,340	8,030	8,157
1559462_at	6,443	6,230	6,400	6,088	6,073	6,240
1559467_at	8,575	8,669	8,852	8,171	8,410	8,190
1559469_s_at	7,947	8,067	8,106	7,767	7,573	7,701
1559470_at	7,517	8,032	7,368	4,973	4,853	4,915
1559471_s_at	8,524	9,005	8,641	6,273	6,355	6,258
1559474_at	7,113	7,116	7,255	7,541	7,469	7,497
1559477_s_at	5,395	5,260	5,593	6,740	6,991	6,797
1559478_at	7,030	6,942	7,240	7,633	7,795	7,805
1559479_at	4,439	4,366	4,124	4,642	4,924	4,604
1559481_at	5,892	6,417	5,853	5,806	5,938	5,733
1559483_at	4,299	4,190	4,146	5,542	5,590	5,605
1559485_at	5,889	5,407	5,564	5,784	6,038	5,700
1559488_at	6,013	6,145	5,810	6,117	5,854	5,712
1559489_a_at	7,906	7,852	7,803	9,351	9,151	9,255
1559490_at	7,693	7,185	7,714	4,781	6,244	5,126
1559491_at	8,205	7,177	7,567	7,070	7,471	6,879

1559492_at	3,451	3,850	3,861	3,878	3,866	3,838
1559494_at	6,873	6,669	6,825	7,036	7,052	7,132
1559495_at	5,748	6,015	6,331	6,009	5,960	5,902
1559496_at	7,816	7,757	7,271	6,493	8,270	6,799
1559497_at	3,755	3,732	3,867	3,908	3,848	3,910
1559500_at	5,079	4,591	4,561	4,386	4,780	4,587
1559501_at	5,042	5,758	5,311	5,783	5,668	5,636
1559502_s_at	7,745	7,406	7,540	8,659	8,152	8,393
1559503_a_at	3,591	4,069	3,714	3,392	3,340	3,272
1559504_at	3,665	3,539	3,588	3,496	3,381	3,549
1559506_x_at	3,731	3,860	4,098	4,009	3,954	4,094
1559507_at	5,289	5,447	5,248	4,856	5,424	5,067
1559508_at	6,789	6,434	6,267	6,115	6,148	6,318
1559509_at	5,466	5,266	5,472	5,113	4,785	5,133
1559510_at	7,085	7,238	6,671	4,651	4,355	4,152
1559513_a_at	5,436	5,341	5,590	5,440	5,301	5,351
1559514_at	4,806	4,416	4,520	5,228	5,577	5,433
1559515_at	5,365	5,628	4,969	4,147	4,298	4,292
1559517_a_at	6,367	6,235	5,650	5,787	5,873	5,992
1559518_at	4,562	4,759	4,329	4,505	4,748	4,969
1559520_at	3,637	3,695	3,768	4,678	4,035	4,224
1559521_at	6,818	6,613	6,671	7,567	7,428	7,604
1559522_at	7,293	7,413	7,310	8,201	8,159	8,237
1559523_at	5,849	5,807	5,653	5,315	5,417	5,419
1559524_at	5,892	5,762	5,988	4,355	4,276	4,454
1559526_at	6,300	5,867	6,333	6,979	7,121	7,141
1559528_at	5,513	4,496	4,055	5,021	4,733	5,032
1559529_at	7,183	6,898	7,779	5,280	6,932	5,469
1559530_at	7,269	7,504	7,456	8,719	8,935	8,706
1559532_at	5,792	6,021	5,616	5,765	5,495	5,864
1559533_at	7,325	7,354	7,356	8,027	8,123	8,401
1559534_at	4,093	3,947	4,018	3,677	3,470	3,633
1559535_s_at	4,395	5,061	4,743	4,205	4,294	4,283
1559537_at	6,401	6,840	6,884	8,498	7,812	8,172
1559538_at	8,675	8,345	8,684	7,248	6,584	6,869
1559540_at	6,115	5,933	6,169	6,267	5,917	6,039
1559542_a_at	3,847	4,036	4,311	3,830	3,751	3,929
1559543_at	4,214	4,246	4,319	6,018	6,169	6,347
1559544_s_at	5,972	6,117	6,159	6,345	6,073	5,957
1559545_at	4,089	4,262	3,989	4,442	4,583	4,496
1559546_s_at	4,135	3,850	4,211	4,077	3,839	4,022
1559548_at	6,837	6,769	6,868	6,124	5,993	6,183
1559550_s_at	6,123	6,034	6,314	6,662	6,493	6,878
1559551_at	5,671	5,703	6,124	6,162	6,378	6,075
1559559_at	5,597	5,270	5,717	7,097	6,922	7,351
1559561_at	7,061	6,681	7,088	8,144	7,985	7,731
1559563_at	7,348	7,098	6,985	7,069	7,121	6,990
1559565_x_at	6,954	6,808	7,431	7,298	7,228	7,159
1559566_at	4,921	5,062	4,908	4,694	4,585	4,732
1559567_at	6,061	6,336	6,031	6,559	6,230	6,332
1559568_at	6,416	5,841	6,057	6,718	6,351	6,112
1559571_a_at	5,519	5,241	5,518	5,689	5,414	5,520
1559573_at	4,269	4,572	4,479	4,580	4,545	4,687
1559575_a_at	5,986	5,954	5,533	6,769	6,459	6,672
1559576_at	5,998	5,234	5,475	5,096	4,992	5,010
1559577_at	5,618	5,590	5,844	6,095	5,799	6,388

1559578_at	7,131	7,247	7,347	7,809	7,552	7,808
1559579_at	6,047	6,032	6,112	6,966	7,102	7,030
1559580_at	4,667	4,386	4,230	4,207	4,183	4,031
1559582_at	7,094	7,308	7,266	6,865	7,266	6,947
1559583_at	6,137	5,908	6,081	5,763	5,968	5,909
1559584_a_at	4,090	3,622	4,081	3,452	3,466	3,463
1559585_at	5,419	6,243	5,901	6,129	5,978	5,810
1559586_at	6,351	6,311	6,362	6,559	6,366	6,436
1559587_at	5,319	5,160	5,350	5,319	5,371	5,513
1559589_a_at	6,529	6,428	5,520	5,194	5,563	5,301
1559590_at	4,501	4,431	4,695	4,681	4,678	4,664
1559591_s_at	8,960	8,347	8,796	8,556	8,826	8,716
1559593_a_at	7,431	7,247	7,107	6,854	7,523	7,250
1559595_at	5,671	5,222	5,458	5,846	5,603	5,912
1559597_at	5,781	5,900	5,861	6,108	6,199	6,042
1559598_at	4,666	4,458	4,383	4,239	4,385	4,219
1559600_at	8,182	7,689	8,111	7,391	7,626	7,857
1559601_at	5,349	5,485	5,325	6,093	5,895	5,951
1559603_at	5,979	5,880	5,957	6,133	5,818	6,153
1559605_a_at	5,853	6,148	6,087	6,162	6,190	6,415
1559606_at	3,883	3,666	3,805	4,071	4,405	4,633
1559607_s_at	4,623	4,938	5,098	5,350	5,618	5,394
1559611_at	4,927	4,986	5,005	5,516	5,332	5,347
1559612_at	3,400	3,476	3,350	3,440	3,508	3,605
1559614_at	5,087	4,832	4,604	5,446	5,407	5,839
1559616_x_at	5,757	5,730	6,085	6,270	6,482	6,040
1559617_at	4,992	4,851	4,705	6,159	6,283	6,134
1559618_at	7,124	7,047	6,972	6,943	6,822	7,300
1559620_at	6,773	6,300	6,757	7,258	7,265	7,212
1559621_at	3,983	4,115	3,936	4,374	4,197	4,221
1559622_at	6,771	6,655	6,844	7,008	6,810	6,912
1559623_at	6,571	6,815	6,595	7,373	7,163	7,060
1559624_at	4,757	4,880	5,058	5,520	5,455	5,369
1559627_at	5,397	5,638	5,604	5,825	5,912	5,995
1559629_at	7,502	7,722	7,853	7,878	7,895	7,830
1559630_at	5,581	5,779	5,351	6,411	6,354	6,391
1559631_at	5,528	5,501	5,509	5,368	5,369	5,450
1559633_a_at	6,535	6,729	6,581	4,724	4,529	4,630
1559634_at	5,078	5,951	5,077	5,741	5,583	5,875
1559635_at	6,778	6,930	6,897	7,004	6,768	6,754
1559638_at	8,866	8,698	8,667	9,520	9,159	9,453
1559640_at	4,292	4,333	4,650	4,336	4,234	4,236
1559641_at	5,046	5,528	5,155	5,405	5,377	5,271
1559642_a_at	5,917	5,886	5,960	6,892	6,982	6,856
1559645_at	6,618	6,367	6,376	8,449	8,684	8,513
1559646_a_at	6,449	5,445	6,159	7,250	7,121	7,262
1559648_at	4,881	4,984	4,711	5,932	5,251	5,793
1559650_at	4,324	4,148	4,354	5,305	5,048	4,759
1559651_at	3,953	4,014	3,582	4,965	4,531	4,826
1559652_at	3,461	3,700	3,839	3,650	3,650	3,677
1559653_at	3,521	3,452	3,336	3,455	3,478	3,434
1559654_s_at	6,912	6,781	6,878	6,804	6,557	7,033
1559655_at	5,949	6,060	6,082	6,257	6,534	6,429
1559656_a_at	6,008	6,116	5,923	7,077	6,923	7,090
1559658_at	6,907	7,447	6,631	5,951	6,158	5,978
1559661_at	4,726	4,978	4,176	5,875	5,606	5,744

1559663_at	5,591	4,899	5,481	4,566	4,861	4,377
1559664_at	4,537	4,175	4,394	4,680	4,480	4,793
1559665_at	4,103	4,204	4,100	4,465	4,254	4,574
1559667_at	7,646	7,406	8,025	8,839	8,846	9,117
1559668_s_at	6,050	5,971	6,160	6,357	6,615	6,615
1559670_at	4,527	4,070	4,117	4,628	4,934	5,277
1559672_a_at	5,829	5,715	5,410	6,245	5,818	5,801
1559674_at	6,631	6,841	6,567	6,474	6,767	6,874
1559675_at	5,951	6,027	5,906	6,202	5,971	5,982
1559676_a_at	6,244	6,433	6,459	7,100	7,114	6,946
1559678_s_at	4,987	4,968	4,743	5,026	4,490	4,844
1559679_a_at	6,071	6,104	5,686	6,087	6,077	6,249
1559680_at	5,412	4,815	5,489	5,462	5,336	5,456
1559681_a_at	5,722	5,966	5,856	6,157	5,288	5,963
1559682_at	7,484	7,248	7,783	7,948	7,885	7,904
1559683_at	5,286	5,467	5,269	5,918	6,231	6,223
1559685_at	3,781	4,009	4,003	4,059	4,016	4,076
1559686_a_at	5,611	5,623	5,393	5,491	5,551	5,699
1559687_at	6,733	6,391	7,144	7,000	6,669	6,887
1559688_at	4,572	4,513	4,809	5,722	5,430	5,777
1559689_a_at	5,149	5,265	4,847	5,972	5,788	6,085
1559691_at	5,194	5,334	5,433	5,808	5,508	5,728
1559692_at	7,053	7,050	6,803	6,747	6,732	6,735
1559695_a_at	5,023	4,566	4,785	5,807	5,407	5,690
1559696_at	7,191	6,974	7,150	7,227	7,940	7,391
1559697_a_at	5,046	5,176	5,187	5,134	5,102	5,128
1559699_at	4,348	3,936	4,345	4,779	4,858	4,686
1559701_s_at	6,439	5,987	6,366	7,658	6,930	7,539
1559702_at	6,272	6,277	6,363	7,236	6,999	7,189
1559705_s_at	6,210	6,015	5,946	6,157	6,373	6,398
1559706_at	4,195	4,499	4,020	4,204	4,321	4,131
1559707_at	4,119	4,587	3,878	4,145	4,319	4,341
1559709_at	5,548	4,773	5,633	4,824	4,632	4,770
1559711_at	5,099	5,840	5,379	5,735	5,732	5,832
1559712_at	6,661	6,727	6,737	7,315	6,741	7,264
1559713_at	7,143	7,166	7,373	7,836	7,840	8,184
1559714_at	6,473	6,910	6,716	7,302	7,041	7,257
1559715_at	7,009	7,096	7,485	7,566	7,714	7,917
1559716_at	7,673	7,492	7,800	7,342	7,566	7,382
1559722_at	4,019	3,739	3,638	4,098	4,086	3,990
1559723_s_at	6,451	6,341	6,642	4,868	5,152	4,759
1559724_at	4,726	4,658	4,663	4,912	4,724	4,668
1559725_at	3,729	3,739	3,973	3,787	3,536	3,445
1559728_at	6,930	6,886	6,868	7,484	7,339	7,455
1559731_x_at	5,536	4,535	4,876	4,586	5,145	4,437
1559732_at	5,811	6,036	5,636	6,069	5,887	6,195
1559735_at	5,286	5,385	4,998	5,707	5,967	5,938
1559737_at	5,179	5,405	5,451	6,079	5,811	6,288
1559739_at	5,069	5,031	4,828	4,500	4,299	4,591
1559745_at	5,474	5,414	5,659	5,949	5,659	5,929
1559746_a_at	6,708	6,670	6,662	6,543	6,401	6,445
1559747_at	5,661	4,880	5,581	5,681	5,724	5,838
1559748_at	6,961	6,894	6,691	7,633	7,550	7,728
1559753_at	7,547	7,668	7,665	8,773	8,881	9,031
1559754_at	7,365	7,009	7,324	8,257	8,116	8,458
1559755_at	7,058	6,821	7,035	7,460	7,316	7,508

1559756_at	4,115	4,102	4,065	4,235	4,144	4,384
1559759_at	6,149	6,619	6,129	6,751	6,661	6,665
1559760_at	3,737	3,876	3,784	4,586	3,999	4,098
1559762_at	4,429	4,450	4,719	4,410	4,490	4,534
1559763_at	4,039	4,045	4,024	4,348	4,402	4,302
1559765_a_at	7,894	7,782	7,695	8,371	8,106	8,329
1559766_at	7,047	7,347	7,047	7,637	7,627	7,690
1559770_at	4,280	4,246	4,319	4,525	4,536	4,475
1559771_at	7,077	6,869	7,069	7,213	7,172	7,082
1559776_at	6,945	7,103	6,137	6,447	6,405	6,659
1559777_at	5,607	5,747	5,493	5,514	5,295	5,436
1559780_at	5,541	5,420	5,569	5,352	5,782	5,474
1559785_at	5,069	5,018	5,223	5,724	5,434	5,443
1559787_at	5,091	5,065	5,044	5,243	5,043	5,185
1559788_at	4,988	5,404	5,634	5,992	5,907	6,299
1559789_a_at	5,752	6,131	5,395	5,607	5,698	5,652
1559790_at	4,755	4,569	4,744	5,071	5,296	5,277
1559792_at	3,885	3,923	3,886	4,224	4,159	4,206
1559795_at	8,114	8,205	8,240	8,062	7,996	8,382
1559796_at	7,028	7,231	7,394	8,175	8,061	7,853
1559800_a_at	5,590	5,060	5,337	6,095	6,181	6,376
1559804_at	5,214	4,604	5,003	5,073	4,987	5,352
1559806_at	6,774	6,629	6,688	7,663	7,586	7,568
1559807_at	3,954	4,141	4,476	4,016	4,210	4,349
1559808_at	7,569	7,522	7,675	7,396	7,324	7,465
1559810_at	5,827	5,795	5,620	5,794	5,506	5,448
1559812_at	6,399	6,160	6,481	7,160	6,879	6,975
1559813_at	5,471	5,692	5,734	5,769	6,264	5,777
1559814_at	4,824	4,953	4,880	5,928	5,952	5,928
1559817_at	6,745	7,232	6,973	6,980	6,964	6,931
1559820_at	6,298	6,139	5,817	6,514	6,038	5,938
1559821_at	7,749	7,957	8,164	8,144	8,412	8,649
1559822_s_at	7,472	7,345	7,592	8,006	8,124	7,669
1559826_a_at	6,372	6,551	6,513	6,557	6,613	7,049
1559827_at	7,287	7,665	7,040	6,433	7,432	8,051
1559828_at	5,895	5,787	5,587	7,016	6,731	6,745
1559833_at	3,707	3,519	4,119	4,236	3,981	3,765
1559835_at	6,497	5,897	6,331	6,416	6,146	6,478
1559837_at	5,050	4,903	5,051	5,193	4,863	5,015
1559839_at	3,660	3,687	3,594	4,334	4,339	4,293
1559840_s_at	4,738	4,582	4,921	5,982	5,299	5,167
1559842_at	3,755	3,930	3,805	3,943	4,046	4,126
1559843_s_at	5,502	5,450	5,458	5,907	5,749	5,588
1559848_at	4,626	4,675	4,658	3,628	3,765	3,658
1559849_at	3,474	3,786	3,845	4,078	4,077	4,263
1559856_s_at	4,216	4,163	4,125	4,166	4,110	4,227
1559861_at	6,242	6,184	5,949	6,753	6,278	6,567
1559862_at	3,946	3,955	3,980	3,669	4,464	3,888
1559863_a_at	7,584	7,299	7,478	7,419	7,320	7,500
1559864_at	6,326	5,886	6,000	6,676	6,645	6,689
1559865_at	3,720	3,978	3,717	3,895	3,962	3,897
1559867_at	5,598	5,956	5,736	7,025	7,047	6,938
1559870_at	8,134	7,951	8,085	8,767	8,264	8,726
1559871_s_at	6,024	6,155	6,180	6,585	6,359	6,564
1559876_at	6,780	6,552	7,035	6,557	6,566	6,538
1559877_at	5,829	5,645	5,589	7,334	6,967	7,338

1559879_a_at	3,964	4,141	3,929	4,154	4,155	4,236
1559880_at	5,942	5,982	6,042	6,927	6,693	6,742
1559881_s_at	8,501	8,358	7,712	6,053	6,233	5,842
1559882_at	5,426	5,718	5,462	5,071	4,791	5,088
1559883_s_at	4,414	4,411	4,404	4,150	4,034	4,099
1559884_at	4,299	4,096	4,252	4,530	4,496	4,568
1559889_at	5,118	4,790	5,218	5,643	5,398	5,558
1559890_a_at	4,647	4,535	4,392	4,560	4,905	4,613
1559891_at	7,051	6,669	6,913	7,769	7,998	7,796
1559893_at	4,782	5,558	4,887	5,950	6,281	5,375
1559895_x_at	3,964	3,954	3,787	3,731	3,634	3,747
1559901_s_at	5,132	5,450	4,896	6,410	6,439	7,009
1559902_at	7,225	7,110	7,146	7,732	7,702	7,758
1559909_a_at	6,339	6,440	6,362	6,880	6,436	6,529
1559910_at	6,587	7,188	7,028	7,131	8,086	7,217
1559911_at	5,582	5,556	5,403	6,600	6,275	6,113
1559914_at	4,504	4,838	4,749	5,212	4,856	4,877
1559915_at	7,118	7,097	7,147	7,689	7,397	7,584
1559916_a_at	6,255	5,403	5,633	5,765	5,867	5,994
1559917_a_at	7,338	7,235	7,360	7,587	7,525	7,557
1559920_a_at	5,169	5,182	4,906	5,348	5,162	5,252
1559921_at	7,671	7,477	7,832	8,184	8,075	8,413
1559922_at	6,407	6,719	6,467	6,237	5,540	5,717
1559923_at	4,018	3,853	4,141	4,193	4,220	4,323
1559924_at	8,227	8,143	8,358	8,837	8,758	8,753
1559925_s_at	5,365	5,180	5,574	6,066	5,713	5,736
1559926_at	5,542	5,311	5,452	6,306	6,049	6,031
1559927_a_at	7,366	7,147	7,277	8,092	7,764	8,194
1559928_at	5,999	6,069	5,956	6,275	6,370	6,156
1559929_at	6,818	7,011	6,890	7,064	7,079	7,048
1559930_at	4,091	3,967	3,936	4,320	4,198	4,418
1559932_at	6,096	5,688	5,873	5,634	5,811	5,557
1559936_at	5,466	4,707	5,328	5,455	5,583	5,724
1559939_at	6,719	6,641	6,339	7,170	8,118	7,349
1559940_s_at	6,565	6,937	6,848	7,774	7,395	7,834
1559942_at	6,764	6,758	7,170	9,132	8,243	8,729
1559945_at	5,583	5,706	6,249	6,011	5,844	6,005
1559946_s_at	11,208	11,217	11,465	11,686	11,353	11,730
1559948_at	6,525	6,120	6,641	7,980	7,685	7,834
1559949_at	4,542	4,755	4,682	4,552	5,083	4,748
1559950_at	6,552	6,496	6,389	6,306	6,330	6,335
1559952_x_at	6,665	6,925	6,976	6,668	6,302	6,616
1559953_at	3,729	3,870	3,704	3,694	3,816	3,742
1559954_s_at	7,964	7,550	7,926	6,212	6,226	6,539
1559956_at	6,913	6,888	7,030	7,866	7,822	7,818
1559957_a_at	9,180	8,763	9,031	7,973	8,683	8,893
1559960_x_at	8,481	8,226	7,767	7,196	6,530	6,670
1559964_at	5,791	4,937	5,090	3,745	3,912	3,784
1559965_at	4,002	4,333	4,536	4,753	7,906	8,761
1559966_a_at	6,782	6,750	6,323	8,475	8,987	9,375
1559970_at	5,168	5,389	4,919	5,259	5,133	5,089
1559971_at	6,313	6,223	5,644	6,031	6,073	6,095
1559975_at	4,898	6,199	6,190	5,414	6,153	5,799
1559976_at	5,698	5,403	5,162	6,053	5,882	6,202
1559977_a_at	8,105	8,095	8,205	7,950	7,673	7,971
1559979_at	6,262	6,407	5,923	6,417	6,114	6,427

1559980_at	4,706	4,934	4,846	5,147	5,018	4,934
1559982_s_at	6,595	6,293	6,267	8,109	8,450	8,551
1559986_at	3,920	4,015	4,043	4,207	3,987	4,054
1559987_at	5,976	5,576	5,693	5,615	5,637	5,797
1559988_at	7,127	7,462	7,124	6,064	5,861	5,862
1559989_at	7,758	7,833	7,644	7,452	7,440	7,762
1559990_at	5,267	5,085	5,263	5,562	5,676	5,723
1559991_s_at	7,200	7,059	7,263	7,618	7,701	7,987
1559992_a_at	3,726	3,578	3,937	3,769	3,575	3,897
1559993_at	7,688	8,065	8,132	5,194	6,017	5,997
1559995_at	6,847	6,914	6,902	7,897	7,625	7,686
1559996_s_at	6,386	6,581	6,567	7,126	7,065	6,943
1559997_x_at	6,999	6,784	7,073	8,432	8,443	8,249
1560001_at	7,519	7,483	7,533	7,613	7,795	7,765
1560002_at	4,714	5,161	5,133	5,142	4,980	5,191
1560006_a_at	6,728	6,622	6,888	7,485	7,497	7,736
1560007_at	5,428	5,504	5,518	5,445	5,594	5,764
1560010_a_at	6,293	6,066	5,969	6,722	6,580	6,913
1560011_at	5,860	5,904	6,054	6,973	7,026	6,807
1560012_at	4,757	4,408	4,765	5,030	4,858	4,982
1560013_at	5,575	5,617	5,031	5,046	5,323	5,298
1560014_s_at	7,084	6,712	6,847	6,558	6,631	6,514
1560017_at	7,209	7,570	6,992	7,379	7,109	6,874
1560018_at	4,826	5,715	5,156	4,759	4,895	4,638
1560019_at	7,702	8,093	8,330	5,796	5,814	5,831
1560020_at	6,535	6,973	6,751	6,826	6,562	6,649
1560021_at	5,208	5,100	5,391	5,426	5,292	5,540
1560023_x_at	5,730	5,632	5,581	5,201	5,434	5,157
1560024_at	7,155	7,130	7,111	7,826	7,351	7,675
1560025_at	4,974	5,279	5,177	5,842	5,444	5,519
1560026_at	6,966	6,606	6,569	6,128	6,500	6,540
1560028_at	4,687	4,929	4,829	4,595	4,652	4,597
1560029_a_at	5,071	5,125	5,040	4,645	5,109	4,799
1560030_at	5,458	5,424	5,652	6,110	6,036	6,188
1560031_at	6,561	6,001	6,346	5,580	6,373	5,285
1560033_at	4,554	4,897	4,529	4,515	4,419	4,411
1560034_a_at	4,549	4,706	5,140	5,033	5,207	5,182
1560035_at	7,332	7,355	7,332	8,152	8,165	8,227
1560038_at	4,405	4,509	4,447	4,758	5,089	5,343
1560042_at	6,061	5,579	5,390	5,488	5,531	5,354
1560043_at	6,128	6,043	6,136	6,498	6,500	6,474
1560047_s_at	7,068	6,764	6,862	7,499	7,882	7,907
1560048_at	4,004	3,972	3,917	4,409	4,536	4,688
1560049_at	8,092	7,980	8,208	7,458	7,157	7,049
1560051_at	4,298	4,506	4,335	4,498	4,164	4,079
1560058_at	5,853	5,731	5,658	6,519	6,021	6,519
1560059_at	7,757	7,607	7,701	8,644	8,444	8,826
1560060_s_at	11,545	11,741	12,193	10,689	10,732	10,861
1560062_at	6,805	6,560	6,718	7,012	7,026	7,266
1560063_a_at	4,704	4,815	4,754	5,598	5,274	5,562
1560065_at	6,131	6,189	5,678	6,528	6,014	6,349
1560066_at	3,774	4,248	4,278	4,194	4,049	4,153
1560068_a_at	6,936	6,612	6,925	7,189	6,876	7,095
1560069_at	5,448	5,697	5,757	5,281	5,930	5,382
1560070_at	5,737	6,223	5,773	6,126	5,902	6,007
1560071_a_at	6,334	6,408	6,555	5,455	6,086	5,624

1560074_at	7,460	7,199	7,529	8,591	8,701	8,979
1560075_at	4,519	4,498	4,232	5,479	5,060	4,992
1560078_at	5,933	5,955	5,652	7,276	7,380	7,551
1560080_at	3,645	3,894	4,050	3,956	4,010	3,887
1560081_at	7,570	7,757	7,434	6,690	6,745	6,513
1560082_at	8,108	7,573	8,320	6,024	6,895	6,281
1560086_at	4,920	4,945	4,783	5,156	5,277	5,386
1560087_a_at	5,564	5,679	5,148	4,818	4,801	5,125
1560089_at	8,640	8,372	8,950	8,003	8,489	8,304
1560091_a_at	6,648	6,950	6,363	5,437	5,430	5,555
1560094_at	6,934	6,567	6,850	7,417	7,376	7,521
1560095_s_at	6,383	6,127	6,515	7,297	6,485	6,975
1560097_at	6,667	7,098	7,135	7,757	7,516	7,650
1560098_at	3,792	3,990	4,123	3,922	3,889	3,836
1560099_at	3,355	3,395	3,759	3,508	3,407	3,527
1560100_at	7,477	7,366	7,316	7,560	7,503	7,666
1560101_at	4,023	4,530	4,586	3,954	4,007	3,909
1560102_at	4,318	4,014	4,162	4,446	4,577	4,764
1560104_at	3,921	3,893	4,166	4,177	3,918	4,205
1560105_at	4,412	4,089	4,572	4,728	4,758	4,982
1560107_at	3,412	3,900	3,550	3,864	4,048	4,065
1560108_at	5,149	5,015	5,085	5,359	5,458	5,202
1560109_s_at	7,040	6,942	6,951	6,959	7,242	7,307
1560111_at	3,611	3,515	4,011	4,758	4,424	4,499
1560112_at	5,256	5,404	5,442	4,817	5,032	4,711
1560115_a_at	7,308	7,454	7,589	7,384	7,155	7,543
1560116_a_at	10,612	10,945	10,160	9,148	9,682	8,507
1560117_at	6,713	6,565	6,666	7,070	6,397	6,744
1560118_at	5,759	5,445	5,648	5,694	5,635	5,776
1560119_at	6,899	6,577	6,952	6,435	6,676	6,414
1560128_x_at	6,627	6,500	6,939	6,716	7,232	6,891
1560129_at	4,478	4,544	4,724	5,120	4,947	5,072
1560130_at	4,713	4,839	4,353	5,198	4,693	5,058
1560131_at	6,040	5,592	5,967	6,467	6,502	6,274
1560132_a_at	5,007	4,790	4,713	4,832	4,706	4,933
1560133_at	6,142	5,301	5,174	3,712	5,283	3,725
1560135_at	5,218	5,018	5,303	5,449	6,675	5,670
1560136_at	7,318	7,415	7,289	7,722	7,848	8,005
1560137_at	6,710	6,414	6,590	6,395	6,902	6,728
1560138_at	5,203	5,413	5,263	5,889	5,993	5,806
1560141_at	7,180	6,932	7,240	7,319	7,377	7,417
1560142_at	3,877	3,741	3,844	3,925	3,894	3,970
1560144_at	3,898	3,960	4,020	3,775	3,861	3,813
1560145_at	7,210	7,347	7,106	5,793	6,714	5,966
1560147_at	5,512	5,324	5,280	5,527	5,754	5,626
1560148_at	6,161	5,819	5,764	5,731	5,751	5,498
1560149_at	6,709	6,698	6,788	7,499	7,640	8,005
1560151_x_at	6,822	7,140	7,157	7,652	7,764	8,187
1560153_at	6,059	6,443	6,249	5,720	5,594	6,023
1560154_a_at	4,749	4,876	5,207	5,029	5,150	5,062
1560156_at	8,407	8,649	8,470	6,278	5,367	5,568
1560161_at	4,347	4,343	4,346	4,240	4,175	4,051
1560162_at	7,634	7,749	7,483	7,832	7,465	7,286
1560163_at	5,926	6,204	5,954	5,995	5,947	6,134
1560164_at	5,239	5,054	5,346	5,542	4,962	5,489
1560168_at	3,569	3,461	3,687	3,639	3,897	3,725

1560169_at	4,389	4,616	4,770	4,791	4,510	5,013
1560171_at	4,686	4,846	4,533	4,271	4,424	4,043
1560172_at	4,274	3,972	4,088	4,516	4,746	4,533
1560174_at	4,151	4,213	4,555	4,131	4,132	4,258
1560175_at	6,896	6,714	6,607	7,222	7,141	7,095
1560176_s_at	5,674	5,981	5,870	6,386	5,812	6,127
1560177_at	6,253	6,620	6,528	6,341	6,372	5,814
1560178_at	5,370	5,173	5,581	6,617	6,437	6,561
1560180_at	6,463	6,565	6,734	7,378	7,157	7,237
1560181_at	5,425	5,697	5,726	6,133	6,361	6,442
1560182_at	3,579	3,791	3,808	3,942	3,861	3,957
1560184_at	3,665	3,819	3,578	4,200	4,009	4,129
1560185_at	4,476	4,233	4,394	4,727	4,565	4,689
1560187_at	5,107	4,690	5,110	5,271	5,070	5,028
1560189_at	6,532	6,546	6,191	7,976	7,900	8,057
1560197_at	3,796	3,771	3,997	3,882	3,841	3,881
1560198_at	8,147	8,162	8,292	8,666	8,740	8,807
1560199_x_at	8,338	7,926	8,243	7,167	8,805	7,650
1560201_at	4,160	4,305	4,485	3,902	3,905	3,828
1560202_at	3,546	3,384	3,537	3,775	3,639	3,681
1560204_at	4,784	4,961	4,832	5,020	5,243	4,950
1560207_at	4,768	4,393	4,827	5,485	5,192	4,955
1560208_at	5,543	5,973	5,623	7,375	7,464	7,095
1560209_at	4,318	4,203	4,600	4,587	4,139	4,264
1560212_a_at	6,832	6,971	7,091	7,428	7,289	7,440
1560213_at	6,703	6,735	6,673	7,489	7,378	7,448
1560214_at	7,908	8,072	8,253	8,456	8,448	8,324
1560219_at	6,557	6,729	6,877	6,752	6,391	6,942
1560220_a_at	6,279	6,239	6,509	5,873	5,883	6,123
1560222_at	4,708	4,681	4,294	4,001	3,842	3,993
1560224_at	5,329	5,084	4,870	4,715	4,857	4,672
1560225_at	6,124	5,190	5,915	5,285	5,204	5,429
1560226_at	5,184	5,176	4,898	5,421	5,378	5,422
1560227_at	5,322	5,260	5,039	4,502	5,032	4,667
1560228_at	4,845	4,794	4,954	4,768	4,759	5,000
1560230_at	4,993	5,217	5,130	5,880	6,671	6,405
1560237_at	4,376	4,757	4,795	4,810	4,745	4,757
1560238_at	5,962	6,089	6,329	6,433	6,249	6,255
1560240_at	5,420	5,092	5,039	5,536	5,326	5,677
1560241_at	5,381	5,844	5,416	5,895	5,671	5,954
1560246_at	4,832	4,831	4,798	5,089	5,106	5,370
1560250_s_at	5,381	5,842	5,764	5,313	5,264	5,629
1560251_at	3,789	4,028	3,831	3,535	3,671	3,624
1560253_at	5,616	5,918	5,636	6,531	6,556	6,674
1560255_at	6,076	6,198	6,076	6,506	6,161	6,647
1560257_at	4,497	4,744	5,367	5,080	4,845	4,713
1560258_a_at	7,767	8,244	8,024	6,951	6,730	6,816
1560259_at	6,981	7,285	6,925	6,573	7,347	6,998
1560260_at	5,015	4,874	5,188	5,542	4,895	4,931
1560262_at	6,332	6,198	6,306	7,322	7,497	7,780
1560263_at	5,386	5,149	5,738	5,322	5,241	5,366
1560264_at	4,518	4,013	4,447	4,624	4,327	4,264
1560265_at	4,617	4,547	4,493	4,332	4,253	4,347
1560271_at	8,931	8,330	8,228	4,731	7,171	4,821
1560274_at	6,195	6,500	6,382	6,203	6,395	6,138
1560275_at	7,651	7,489	7,457	8,082	8,434	8,299

1560276_at	5,339	5,200	5,041	5,648	5,714	5,710
1560277_a_at	7,697	7,576	7,637	8,310	8,028	8,175
1560278_at	7,829	7,721	8,015	8,801	8,746	8,994
1560279_a_at	6,524	6,588	6,399	6,477	6,484	6,657
1560281_a_at	7,399	7,066	7,649	7,691	7,552	7,771
1560282_at	4,562	4,811	4,498	5,296	5,003	5,154
1560284_at	5,973	5,657	6,074	6,557	6,181	6,647
1560285_at	5,131	6,241	5,183	5,710	5,526	5,455
1560286_s_at	6,154	6,641	6,812	6,788	6,696	6,684
1560288_at	3,618	3,482	3,507	3,917	3,854	4,049
1560290_at	5,580	5,320	5,574	5,024	5,490	5,029
1560291_at	4,558	4,599	4,700	5,210	5,163	5,467
1560292_a_at	7,185	6,825	7,389	7,497	7,319	7,657
1560295_at	6,968	7,127	7,195	7,414	7,445	7,407
1560296_at	8,290	7,657	7,777	8,489	8,626	8,526
1560297_at	7,414	6,809	6,977	6,772	7,416	6,909
1560298_at	5,323	5,381	5,518	5,419	5,446	5,520
1560300_a_at	8,219	8,159	8,289	9,155	9,168	9,397
1560303_at	7,711	7,110	8,189	6,176	5,943	5,865
1560305_x_at	7,181	7,055	7,296	5,962	5,981	5,884
1560306_at	4,892	4,540	4,787	5,160	5,018	5,474
1560309_s_at	5,916	5,732	5,791	6,509	6,007	6,174
1560315_at	5,955	5,790	5,879	6,828	6,292	6,546
1560316_s_at	5,876	5,925	5,939	6,412	6,025	6,102
1560317_s_at	5,334	5,158	5,218	6,316	5,960	6,668
1560318_at	4,409	3,927	4,572	4,685	5,754	4,884
1560320_a_at	7,259	7,018	7,172	8,247	8,211	8,374
1560322_at	4,220	4,206	4,203	4,036	3,962	4,109
1560325_at	3,943	4,203	4,196	4,345	4,316	4,445
1560327_at	8,271	8,145	8,302	9,197	8,738	9,256
1560328_s_at	3,483	3,994	3,570	3,629	3,497	3,720
1560330_at	3,821	3,666	3,945	4,037	3,804	3,870
1560332_at	6,568	6,519	6,418	7,328	7,174	7,426
1560334_at	5,595	5,595	5,419	5,396	5,203	5,499
1560337_at	5,369	5,553	5,575	5,930	5,402	5,930
1560339_s_at	9,047	8,904	8,937	8,159	7,868	7,916
1560340_s_at	4,776	4,756	4,970	4,296	4,594	4,152
1560342_at	6,150	5,366	6,097	5,150	5,361	4,845
1560346_at	4,377	4,943	4,498	3,921	3,984	4,061
1560347_at	5,633	5,268	5,783	5,998	6,173	6,137
1560348_at	6,779	7,102	7,115	7,101	6,968	7,053
1560349_at	4,479	4,520	4,061	3,754	3,946	3,718
1560352_at	5,681	5,480	5,625	6,393	6,051	6,068
1560353_at	7,244	7,201	7,433	7,533	7,296	7,473
1560354_at	5,659	6,398	5,801	6,252	5,885	6,172
1560358_at	5,706	5,705	5,886	6,520	6,491	6,504
1560359_at	4,057	4,482	3,963	4,292	3,974	3,987
1560369_at	6,072	5,763	6,325	7,165	6,796	6,832
1560370_x_at	5,796	6,063	6,026	6,716	6,698	6,686
1560371_at	5,618	5,877	5,987	5,397	5,151	5,141
1560372_at	3,482	3,688	3,465	3,702	3,678	3,815
1560373_a_at	4,610	5,646	4,837	4,815	4,615	5,005
1560378_at	6,177	5,948	6,271	6,829	6,649	7,082
1560380_at	4,689	4,759	4,721	5,504	5,377	5,527
1560382_at	7,403	7,566	6,900	7,668	7,769	7,987
1560383_at	4,197	4,652	3,936	4,805	4,751	5,325

1560384_a_at	6,329	6,440	6,085	7,288	7,377	7,629
1560385_x_at	3,423	3,776	3,528	3,685	3,811	3,682
1560386_at	6,831	6,059	6,546	4,896	5,008	5,082
1560390_s_at	5,646	5,484	5,402	5,556	5,566	5,580
1560391_at	5,733	4,923	5,316	5,914	5,649	5,816
1560395_at	4,906	4,578	4,876	4,100	4,901	4,270
1560396_at	4,765	5,151	5,091	5,133	4,980	5,084
1560397_s_at	6,115	6,132	6,097	6,396	6,492	6,652
1560399_a_at	6,136	5,980	6,136	6,839	6,899	6,837
1560402_at	5,658	6,098	5,978	5,300	5,611	5,251
1560404_a_at	4,111	4,333	3,991	4,072	4,083	3,965
1560405_at	5,994	6,061	6,085	6,889	6,596	6,807
1560407_at	7,163	7,766	6,652	6,718	6,448	6,902
1560409_at	6,712	6,546	6,799	7,062	7,138	7,033
1560410_at	7,661	7,414	7,550	7,711	7,455	7,530
1560411_at	5,497	5,400	5,693	5,634	5,541	5,628
1560412_at	5,868	5,864	6,005	6,745	6,640	6,687
1560413_at	4,429	4,589	4,320	4,337	4,475	4,447
1560416_at	4,650	4,559	4,454	4,414	4,505	4,439
1560418_at	4,638	5,015	4,361	5,979	6,092	6,122
1560419_at	4,292	3,987	4,187	4,232	4,028	4,293
1560422_at	5,072	5,025	4,900	4,548	4,580	4,637
1560424_at	3,745	3,718	3,343	3,901	3,832	3,981
1560425_s_at	4,010	3,937	4,101	3,937	3,791	3,877
1560426_at	4,028	4,047	4,166	4,219	4,103	4,201
1560429_at	4,123	4,118	4,007	4,258	4,252	4,173
1560430_at	7,434	7,356	7,420	7,380	7,396	7,535
1560431_at	4,621	4,550	4,485	4,490	5,236	4,410
1560432_at	5,965	6,197	6,065	6,512	6,361	6,412
1560433_at	7,660	7,722	8,580	6,026	6,166	5,902
1560434_x_at	7,339	7,258	8,111	5,961	5,918	5,618
1560435_at	6,562	6,234	6,784	6,781	6,970	6,946
1560439_at	8,121	8,087	8,015	8,431	8,347	8,689
1560443_at	6,171	6,015	6,384	6,269	6,224	6,138
1560445_x_at	8,882	8,551	8,591	8,671	9,246	8,723
1560446_at	6,214	5,921	6,877	6,213	5,882	6,205
1560448_at	5,919	5,968	5,550	6,458	6,108	6,219
1560449_at	6,076	6,234	6,233	6,599	6,644	6,759
1560451_at	8,417	8,027	7,902	8,731	8,263	8,643
1560453_at	5,268	5,305	5,274	5,436	5,350	5,282
1560455_at	5,670	5,512	5,960	6,197	6,159	6,124
1560456_at	7,561	7,267	7,264	8,394	8,085	8,268
1560457_x_at	6,986	6,888	6,803	7,672	7,764	7,570
1560458_s_at	3,474	3,541	3,820	3,614	3,742	3,789
1560460_at	4,421	4,403	4,653	4,676	4,687	4,675
1560463_at	6,239	6,582	6,396	6,398	6,552	6,455
1560464_at	4,780	4,656	5,039	5,159	5,173	5,305
1560467_at	4,543	4,754	4,837	4,553	4,505	4,497
1560469_at	4,175	3,733	3,733	3,604	3,675	3,658
1560472_at	6,570	6,066	6,346	6,692	6,717	6,617
1560474_at	7,095	7,468	7,109	7,036	7,306	7,225
1560475_at	4,623	4,778	4,703	5,539	5,169	5,014
1560476_at	7,058	6,569	6,828	7,511	7,554	7,534
1560477_a_at	8,224	7,995	8,477	7,818	7,887	8,142
1560480_at	7,315	7,200	7,155	7,396	7,135	7,454
1560482_at	5,424	6,068	5,484	5,978	5,911	6,000

1560483_at	4,270	4,079	4,196	4,108	4,007	4,303
1560485_at	6,020	6,361	6,464	6,218	5,812	5,858
1560486_at	6,082	6,005	6,209	4,022	5,483	4,993
1560488_at	7,937	7,743	7,893	8,109	8,010	8,008
1560489_at	6,165	6,560	6,500	7,278	6,891	7,050
1560490_at	4,364	4,683	5,053	3,888	4,076	3,999
1560491_at	5,029	5,211	4,850	5,469	5,347	5,624
1560492_at	7,993	7,614	7,822	8,137	7,797	7,808
1560493_a_at	3,691	3,728	3,814	4,044	3,937	4,153
1560494_a_at	3,631	3,596	3,949	3,674	3,628	3,598
1560495_at	4,092	4,013	4,121	4,212	4,203	4,346
1560496_at	8,443	8,390	8,377	8,681	8,585	8,901
1560498_at	6,255	6,502	6,406	6,324	6,506	6,329
1560499_at	7,182	6,793	7,043	7,222	7,160	7,122
1560500_at	6,527	6,353	6,379	6,747	7,124	6,242
1560503_a_at	6,127	5,874	6,021	6,060	5,404	5,626
1560506_at	4,844	4,494	4,953	4,962	4,817	4,739
1560507_at	6,611	6,819	6,960	7,459	7,332	7,508
1560509_at	7,604	7,088	7,447	6,568	6,546	6,644
1560510_at	4,838	5,037	4,786	4,971	4,920	4,913
1560511_at	6,325	6,304	6,082	7,338	7,060	7,389
1560512_at	6,131	6,300	6,320	5,916	6,148	6,066
1560513_at	5,718	6,019	6,103	6,661	6,592	6,800
1560514_at	6,410	6,563	6,502	6,428	6,544	6,372
1560516_at	3,888	3,823	3,621	4,229	4,244	4,322
1560517_s_at	3,994	4,059	4,064	4,626	4,557	4,623
1560520_at	6,905	6,604	6,710	7,154	7,203	7,146
1560522_at	4,583	4,554	4,880	5,196	5,203	5,389
1560523_at	5,511	5,269	5,331	5,368	5,103	5,329
1560524_at	6,285	6,490	6,673	7,255	6,957	7,300
1560525_at	5,854	5,489	5,757	5,696	5,640	5,560
1560526_at	4,169	4,360	4,103	4,238	4,567	4,392
1560527_at	5,042	5,197	5,157	5,871	5,938	5,992
1560528_at	3,770	3,781	3,845	3,808	3,882	3,766
1560531_at	5,328	5,662	5,641	6,274	6,215	5,975
1560533_at	4,581	4,289	4,724	4,641	4,490	4,305
1560536_at	7,490	7,557	7,751	7,526	7,599	7,494
1560537_at	6,332	6,091	6,437	6,369	6,143	6,350
1560538_at	3,570	3,733	3,641	3,663	3,558	3,630
1560540_x_at	3,703	3,658	3,379	4,041	4,737	4,283
1560542_at	4,369	4,026	3,964	4,230	4,269	4,241
1560543_at	3,563	3,662	3,505	3,650	3,760	3,847
1560547_at	4,769	4,388	4,691	4,654	4,724	4,701
1560548_at	6,136	6,296	6,292	6,628	6,436	6,434
1560550_at	6,463	6,304	6,645	7,139	7,073	6,978
1560552_a_at	4,156	4,098	4,262	4,262	4,280	4,363
1560553_at	6,542	6,518	6,607	6,974	7,066	7,318
1560554_a_at	4,787	4,991	5,334	4,664	4,870	5,073
1560556_a_at	7,007	7,265	7,820	4,051	4,636	3,981
1560557_at	4,952	5,140	5,282	5,588	5,522	5,654
1560558_at	3,919	4,068	3,869	4,224	4,064	4,200
1560559_at	5,341	5,366	5,502	6,678	6,146	6,220
1560560_at	7,787	7,907	8,098	8,700	8,759	8,973
1560562_a_at	8,138	7,957	7,775	5,994	5,926	6,304
1560563_at	6,506	6,166	6,169	7,201	6,438	6,818
1560565_at	5,183	4,970	5,134	5,706	5,535	5,441

1560566_at	4,480	4,571	4,649	5,070	4,871	4,851
1560570_a_at	4,312	4,391	4,397	3,832	4,101	4,088
1560573_at	6,123	6,184	6,049	6,683	6,262	6,663
1560576_at	7,145	6,833	6,830	8,300	8,490	8,386
1560577_at	6,316	5,830	6,129	6,437	6,079	6,142
1560579_s_at	7,102	6,752	6,770	7,705	7,176	7,657
1560581_at	4,814	4,882	5,267	5,461	5,124	5,473
1560582_a_at	5,488	5,570	5,847	6,740	7,021	7,133
1560587_s_at	12,161	12,445	12,251	11,566	10,890	12,144
1560588_at	6,171	6,099	6,209	6,389	6,463	6,465
1560589_a_at	5,760	5,975	5,970	6,223	5,763	6,432
1560590_s_at	5,538	5,477	5,617	5,635	5,610	5,830
1560595_at	5,337	5,116	5,133	5,435	4,981	5,400
1560596_at	5,885	5,932	6,421	5,857	5,790	5,887
1560599_a_at	6,698	6,785	6,882	7,599	6,836	7,589
1560604_at	5,935	6,220	5,870	6,518	6,362	6,153
1560609_at	4,934	4,922	4,864	5,684	5,248	5,747
1560610_at	6,513	6,300	6,178	6,386	6,034	6,108
1560614_at	3,919	3,927	4,241	4,345	4,568	4,409
1560615_a_at	5,818	5,846	5,625	6,223	5,941	5,935
1560617_at	6,138	6,038	6,085	6,993	6,928	7,338
1560618_at	6,993	6,930	6,791	7,147	6,978	7,532
1560619_at	5,416	5,676	5,442	5,744	5,701	5,884
1560620_at	6,212	6,451	6,363	6,911	6,776	6,876
1560621_at	6,597	6,650	6,975	7,578	7,616	7,684
1560622_at	8,755	8,879	8,644	7,242	9,370	8,251
1560625_s_at	7,481	7,823	7,365	7,627	7,309	7,400
1560628_at	4,835	4,694	4,766	6,340	6,218	6,341
1560630_at	7,380	7,156	7,454	7,721	7,637	7,656
1560631_at	5,585	5,451	5,463	5,999	5,847	6,009
1560633_a_at	6,346	6,157	6,062	6,695	6,548	7,060
1560634_a_at	5,415	5,403	5,355	5,516	5,684	5,737
1560636_a_at	5,753	5,816	6,072	6,325	6,112	6,066
1560637_at	5,049	4,754	4,952	4,794	5,117	4,861
1560638_a_at	5,264	5,275	4,880	5,765	5,831	5,696
1560639_at	7,867	7,605	8,207	8,716	8,753	8,665
1560642_at	5,147	4,655	4,590	5,635	5,177	5,441
1560643_x_at	5,594	4,824	5,043	6,170	5,977	6,117
1560646_at	4,810	5,195	5,268	5,693	5,754	5,902
1560647_at	3,894	3,886	4,170	3,983	4,016	3,814
1560648_s_at	5,519	5,469	5,347	6,031	5,950	5,988
1560652_at	7,063	6,723	6,805	4,794	4,807	4,802
1560654_at	4,954	5,086	5,007	4,715	4,735	4,815
1560656_at	5,641	5,711	5,961	6,171	6,162	6,260
1560657_at	4,647	4,661	4,550	5,082	4,878	5,108
1560659_at	4,627	4,395	4,344	4,443	5,449	4,532
1560661_x_at	5,276	4,880	5,173	5,157	5,393	4,955
1560662_s_at	4,079	4,511	4,394	4,460	4,852	4,573
1560665_at	6,280	6,105	6,207	6,509	6,784	6,616
1560671_at	6,541	6,769	6,350	6,924	7,055	7,044
1560673_at	4,536	4,318	4,837	5,583	5,344	5,878
1560676_at	4,495	4,583	4,651	5,160	4,845	5,266
1560678_at	5,940	6,392	6,284	6,911	6,879	6,874
1560679_at	5,257	4,634	5,099	5,642	5,779	5,499
1560680_at	5,887	5,923	5,838	5,730	6,012	5,488
1560683_at	3,840	4,157	4,006	4,020	4,061	4,159

1560684_x_at	4,556	4,654	4,596	4,557	4,715	4,867
1560686_at	7,286	6,958	7,259	7,380	7,234	7,595
1560689_s_at	6,546	6,527	6,925	6,369	6,367	6,297
1560690_at	4,370	4,359	4,394	5,031	4,628	5,158
1560692_at	4,051	4,180	4,205	4,097	3,916	4,202
1560694_at	7,433	7,750	7,627	7,682	7,924	7,865
1560695_at	4,489	4,920	4,082	5,392	5,207	5,634
1560696_x_at	5,401	5,561	5,636	6,156	6,036	5,788
1560697_at	5,294	5,454	5,428	5,395	5,579	5,509
1560698_a_at	5,590	5,661	5,816	6,125	5,864	5,938
1560703_at	5,553	4,984	5,599	5,355	5,124	5,083
1560705_at	5,178	5,036	5,380	5,725	5,961	6,001
1560706_at	5,008	5,362	5,232	6,308	5,876	6,186
1560707_at	5,154	5,142	5,257	5,480	5,336	5,425
1560712_at	4,225	3,813	4,232	4,839	4,541	4,464
1560713_a_at	6,823	7,121	6,889	7,608	7,634	7,823
1560714_at	4,983	4,984	5,713	5,183	5,253	4,823
1560715_at	4,949	5,167	5,069	5,455	5,338	5,085
1560716_at	4,379	4,528	4,322	4,290	4,394	4,259
1560717_at	3,886	3,682	3,957	3,873	3,843	4,223
1560720_at	5,769	5,637	5,458	5,621	5,960	5,984
1560721_at	4,178	4,062	4,373	4,281	4,504	4,388
1560723_at	4,553	4,535	4,502	4,589	4,333	4,503
1560724_at	6,002	5,887	6,042	6,075	5,954	5,622
1560725_at	5,059	5,114	5,967	6,655	6,449	6,833
1560727_at	6,604	6,435	6,460	7,329	6,973	7,095
1560728_at	5,716	6,199	5,994	5,971	5,679	5,712
1560733_at	4,994	4,567	4,688	4,652	4,865	4,789
1560734_at	5,024	5,067	5,184	5,132	4,890	4,945
1560735_s_at	4,383	4,728	5,326	4,641	4,665	4,655
1560738_at	7,340	8,886	7,415	5,937	5,710	5,599
1560739_a_at	8,639	9,886	8,626	5,426	5,268	5,394
1560741_at	9,284	9,035	9,316	6,037	6,233	6,170
1560742_at	5,072	4,887	5,605	4,995	4,862	4,827
1560743_a_at	4,713	4,608	4,875	4,707	4,478	4,767
1560744_at	6,671	6,598	6,345	6,640	6,465	6,825
1560745_at	4,208	4,289	4,138	4,200	4,184	4,214
1560746_at	3,634	4,088	4,230	3,921	4,163	4,310
1560747_at	5,841	5,945	5,987	5,769	5,658	5,704
1560748_at	3,990	3,777	3,681	4,045	3,868	4,125
1560750_at	5,733	5,800	5,524	6,661	6,469	6,675
1560751_at	6,100	5,802	5,834	6,320	6,056	6,349
1560752_at	5,450	4,983	5,158	5,880	5,702	5,757
1560753_at	6,247	6,086	6,463	6,202	6,290	6,185
1560754_at	6,264	6,023	5,935	6,171	6,534	6,537
1560755_at	4,753	4,536	5,006	4,935	4,822	4,714
1560756_at	4,498	4,678	3,805	5,555	4,498	4,655
1560757_at	5,669	5,927	5,819	6,280	6,317	6,314
1560758_at	5,406	5,559	5,453	5,504	5,655	5,609
1560760_s_at	4,220	4,613	3,988	3,654	3,645	3,537
1560762_at	4,130	3,908	3,981	4,451	4,438	4,512
1560763_at	6,309	5,787	6,136	5,307	5,662	4,800
1560764_at	6,111	5,988	6,142	6,293	5,966	6,431
1560765_a_at	6,433	6,560	6,753	7,183	6,942	7,147
1560767_at	7,499	7,121	7,371	8,432	8,415	8,649
1560769_at	4,632	4,675	4,635	5,015	4,886	4,994

1560770_at	5,349	5,348	5,745	6,015	6,335	5,950
1560771_at	5,527	5,400	5,639	6,155	6,206	6,584
1560772_a_at	5,109	4,922	5,447	6,044	6,101	5,902
1560773_at	3,821	4,265	4,002	3,763	3,834	3,840
1560774_at	5,792	5,614	5,714	6,252	6,086	5,844
1560775_at	4,407	4,518	5,035	4,692	4,440	4,821
1560776_at	4,013	3,980	3,819	3,993	4,168	4,127
1560779_a_at	6,103	5,920	6,074	6,491	6,195	6,100
1560781_at	3,840	3,834	4,193	3,804	3,671	3,860
1560782_at	8,148	8,129	8,121	9,351	9,422	9,551
1560784_x_at	7,689	7,169	7,652	8,595	8,728	8,995
1560785_at	5,917	5,831	5,496	5,956	6,181	6,262
1560787_at	6,466	7,042	6,488	7,325	7,301	7,318
1560788_at	5,612	6,068	5,814	6,121	5,756	5,754
1560790_at	5,430	5,087	5,408	5,751	5,612	5,900
1560791_at	3,622	3,644	3,957	3,846	3,875	3,890
1560792_at	7,623	7,606	7,366	7,191	7,063	7,132
1560794_at	5,692	6,018	5,798	6,076	6,194	6,071
1560797_s_at	4,470	4,275	4,255	4,458	4,263	4,631
1560798_at	3,890	4,053	4,086	4,069	4,098	4,089
1560800_at	3,629	3,683	3,657	3,680	3,723	3,870
1560803_at	5,267	4,777	4,859	4,691	4,649	4,852
1560806_at	6,571	6,229	6,558	7,609	7,360	7,675
1560810_at	5,585	5,214	5,107	6,432	6,132	6,049
1560812_at	6,349	5,848	5,570	6,479	6,740	6,817
1560813_at	5,063	5,009	5,017	4,552	4,793	4,711
1560814_a_at	5,528	5,271	5,817	5,707	5,261	5,398
1560817_at	6,095	6,425	6,366	5,623	5,942	6,019
1560818_at	7,620	7,180	7,202	8,091	7,938	7,759
1560819_a_at	5,387	4,956	5,596	5,651	5,724	5,734
1560821_at	5,351	5,700	5,372	6,671	6,615	6,303
1560823_at	3,616	3,668	3,630	3,789	3,719	3,787
1560826_at	6,331	6,292	6,440	7,092	7,087	7,778
1560827_at	6,152	5,854	6,883	6,546	6,282	6,505
1560830_a_at	8,079	7,956	8,061	8,622	8,130	8,430
1560832_at	6,067	6,057	5,924	6,777	6,314	6,677
1560833_at	3,930	4,190	4,117	4,328	4,264	4,252
1560834_a_at	7,117	7,924	7,319	6,662	6,662	6,800
1560836_at	5,785	5,802	5,828	6,222	6,121	6,356
1560838_at	4,625	4,922	4,708	5,025	4,697	4,635
1560841_at	6,463	6,417	6,290	6,451	6,123	6,217
1560842_a_at	4,026	3,960	3,995	4,027	3,811	4,012
1560844_at	6,026	5,422	5,565	7,015	6,915	6,937
1560846_at	4,273	4,699	4,354	4,141	4,408	4,231
1560848_at	4,900	4,875	4,881	6,102	6,225	6,112
1560850_at	4,879	4,880	4,660	5,398	5,559	5,788
1560851_at	3,809	3,938	3,948	5,299	4,852	4,813
1560853_x_at	8,397	9,104	8,297	7,907	7,563	7,935
1560854_s_at	6,325	6,541	6,326	6,705	6,393	6,641
1560855_at	6,010	6,118	6,226	6,605	6,977	6,853
1560856_at	6,429	5,626	6,332	7,189	7,093	7,509
1560859_at	5,192	4,814	5,326	4,031	4,125	4,324
1560861_at	4,955	4,599	4,673	5,584	5,452	5,669
1560862_at	6,935	6,853	6,861	6,916	6,905	6,778
1560863_a_at	5,448	5,530	5,064	6,090	5,920	5,852
1560864_at	4,252	4,466	4,336	4,754	4,674	4,746

1560865_a_at	4,457	4,203	4,555	4,606	4,684	4,473
1560866_at	7,447	7,474	7,830	8,106	8,046	8,132
1560867_a_at	5,082	5,326	5,010	5,499	5,468	5,600
1560868_s_at	7,159	7,505	7,287	7,279	7,462	7,499
1560869_a_at	4,583	4,241	4,378	3,758	5,310	3,769
1560870_a_at	5,846	5,914	6,057	6,298	5,854	6,049
1560871_a_at	5,959	5,872	5,792	6,698	6,442	6,219
1560872_at	6,109	5,966	6,288	6,605	6,461	6,727
1560874_at	6,995	7,153	6,782	8,377	8,570	8,157
1560877_a_at	5,484	5,665	5,400	6,036	5,505	5,630
1560878_at	9,617	9,405	9,440	10,294	9,793	9,854
1560879_a_at	6,908	6,910	7,107	7,632	7,124	7,431
1560881_a_at	6,473	6,415	6,288	6,258	6,372	6,134
1560883_s_at	7,919	7,749	8,034	8,646	8,466	8,620
1560884_at	3,625	3,547	3,554	3,705	3,680	3,790
1560885_x_at	4,178	4,100	4,173	4,069	4,145	4,098
1560887_a_at	5,105	4,899	4,987	5,424	5,384	5,097
1560888_x_at	5,117	5,120	5,631	5,910	6,306	6,312
1560889_a_at	4,113	4,471	4,611	4,998	4,660	4,857
1560890_at	3,815	3,695	4,051	4,482	4,760	4,728
1560891_a_at	4,955	5,417	5,093	5,494	5,274	5,604
1560893_at	4,210	4,333	4,131	4,883	4,600	4,622
1560895_at	4,333	4,436	4,375	5,426	5,033	5,149
1560896_a_at	6,141	6,279	6,300	6,323	6,113	6,158
1560897_a_at	7,503	7,266	7,599	9,109	8,284	8,552
1560898_at	4,745	4,591	4,973	4,864	5,133	5,273
1560899_at	3,977	3,806	3,790	4,057	4,242	4,227
1560900_a_at	4,465	4,485	4,666	4,847	4,769	4,802
1560901_at	7,660	7,742	7,731	7,457	7,248	7,542
1560903_at	4,566	4,699	4,545	5,126	5,019	5,012
1560905_at	5,669	5,935	5,774	5,770	5,760	5,840
1560906_at	4,756	4,731	4,843	4,605	4,329	4,602
1560908_at	6,884	6,824	6,890	6,694	6,792	6,788
1560909_x_at	4,432	4,061	4,378	4,155	4,555	4,349
1560910_at	7,036	7,056	7,179	7,909	7,488	7,401
1560911_at	7,085	7,231	7,317	8,144	8,083	8,370
1560912_at	6,072	6,001	6,210	6,424	6,358	6,410
1560913_at	6,322	6,419	6,342	6,571	6,331	6,541
1560915_at	4,086	4,333	4,227	4,243	4,111	4,289
1560916_a_at	8,330	8,352	8,062	8,124	8,030	8,268
1560917_at	6,440	6,121	6,336	6,829	6,724	7,014
1560918_at	4,185	4,063	4,456	4,982	4,803	4,937
1560920_s_at	8,224	8,154	8,164	8,393	8,213	8,234
1560921_at	7,438	7,277	7,489	7,620	7,656	8,086
1560922_s_at	6,614	6,437	6,092	6,304	6,391	6,465
1560924_at	5,629	5,736	5,938	6,715	6,311	6,728
1560926_at	8,070	7,430	7,742	5,460	6,335	5,577
1560928_at	6,725	6,721	7,195	7,052	7,519	7,545
1560929_at	4,161	4,117	3,903	4,620	4,273	4,296
1560932_at	5,899	6,061	5,894	6,549	6,531	6,734
1560934_at	5,704	5,837	5,852	6,540	6,081	6,390
1560935_s_at	5,836	5,966	6,201	7,238	7,186	7,094
1560937_at	5,799	5,718	5,866	6,050	6,105	6,064
1560939_at	7,111	7,275	7,346	7,767	7,439	7,801
1560940_at	3,783	4,005	4,081	4,179	4,374	4,277
1560941_a_at	4,774	4,742	4,967	5,093	5,026	5,138

1560942_at	6,648	6,832	6,979	7,245	7,145	7,242
1560943_s_at	6,369	6,396	6,091	7,061	6,728	7,124
1560944_at	6,005	5,859	6,012	6,142	5,737	6,005
1560946_at	5,295	5,320	5,859	6,263	6,238	6,507
1560950_at	3,497	3,564	3,994	4,057	3,705	3,989
1560952_at	7,097	6,766	6,828	7,502	7,478	7,412
1560954_at	4,192	4,065	4,230	4,558	4,707	4,789
1560955_at	5,667	5,661	5,999	5,990	5,943	6,124
1560957_at	4,709	4,525	4,877	5,165	5,137	5,466
1560958_s_at	5,605	5,718	5,949	6,085	5,866	5,927
1560959_a_at	4,443	4,621	4,605	4,913	4,812	4,763
1560960_at	5,079	4,997	4,809	5,673	5,814	5,955
1560962_at	3,903	4,087	3,942	4,249	4,044	4,220
1560963_a_at	5,915	5,622	5,618	6,564	6,257	6,131
1560964_at	5,568	5,546	5,539	5,937	5,652	5,972
1560966_at	5,835	6,157	6,352	6,730	6,311	6,375
1560968_at	6,682	7,404	7,061	7,375	7,362	7,177
1560971_a_at	5,968	5,916	6,522	6,245	6,225	6,103
1560973_a_at	4,404	4,428	4,149	4,217	4,125	4,090
1560974_s_at	6,694	6,660	6,651	6,762	6,522	6,597
1560977_a_at	5,243	5,911	5,920	5,416	5,225	5,242
1560978_at	7,071	7,199	7,198	7,582	7,775	7,993
1560979_a_at	5,096	4,722	4,916	5,641	5,444	5,335
1560980_a_at	3,623	3,453	3,635	3,676	3,581	3,522
1560981_a_at	4,214	4,344	4,450	4,988	5,007	5,056
1560982_at	6,814	6,702	6,933	7,183	6,927	7,190
1560986_a_at	7,423	7,084	7,186	6,959	6,975	6,517
1560987_a_at	4,687	4,433	4,739	5,288	5,117	4,998
1560988_a_at	4,391	4,968	4,721	4,690	4,581	4,689
1560989_at	4,516	4,090	3,919	4,560	4,169	4,481
1560990_a_at	3,902	4,136	3,688	4,139	3,820	3,838
1560992_at	3,458	3,561	3,408	3,742	3,747	3,671
1560994_x_at	3,621	3,744	3,762	3,734	3,836	4,022
1560995_s_at	4,945	5,429	5,099	5,045	5,048	5,142
1560996_at	4,012	4,089	4,047	4,085	4,124	4,147
1560997_at	3,363	3,287	3,313	3,335	3,341	3,265
1560998_x_at	3,322	3,285	3,482	3,433	3,578	3,455
1560999_a_at	4,575	5,001	5,059	4,737	4,632	4,856
1561000_at	6,042	5,686	6,354	6,088	6,040	6,091
1561002_at	4,913	4,720	4,517	5,366	5,299	5,703
1561003_at	3,990	3,786	4,146	4,346	4,231	4,283
1561004_at	6,181	6,534	6,436	6,158	6,073	6,030
1561006_at	5,529	5,862	5,748	7,188	6,580	7,541
1561009_at	4,073	4,186	4,084	4,311	4,239	4,073
1561010_a_at	4,922	5,268	5,364	5,827	5,516	5,674
1561012_at	4,586	4,724	4,703	5,204	5,219	5,527
1561014_at	5,886	5,413	5,235	5,809	5,604	5,637
1561015_at	7,224	7,091	7,020	6,973	6,891	6,874
1561016_at	3,911	3,753	3,873	3,848	3,836	3,861
1561017_at	6,317	6,244	5,938	4,811	5,148	4,821
1561018_at	6,418	5,943	6,176	5,500	5,689	5,416
1561019_at	7,144	7,161	7,227	7,477	7,441	7,432
1561021_at	3,601	3,930	3,681	3,546	3,466	3,641
1561025_at	3,576	3,887	3,872	3,703	3,911	3,861
1561026_a_at	5,728	5,564	6,066	5,856	5,992	6,062
1561027_at	4,784	4,708	4,905	5,492	5,070	5,190

1561029_at	6,159	6,586	6,253	7,028	6,883	7,106
1561030_at	3,634	3,557	3,985	4,384	4,042	4,633
1561033_at	4,113	4,421	4,494	4,570	4,364	4,617
1561034_at	5,598	5,279	5,261	6,225	5,610	5,980
1561035_at	6,317	6,204	6,339	6,905	6,205	6,533
1561036_at	7,051	7,133	7,213	7,380	7,130	7,311
1561037_a_at	6,769	6,622	6,741	6,866	6,534	7,078
1561038_at	6,325	6,531	6,604	7,627	7,574	7,783
1561039_a_at	6,514	6,670	6,543	6,379	6,452	6,785
1561041_at	6,870	7,076	6,541	7,452	7,339	7,484
1561042_at	5,596	6,131	6,654	5,063	5,542	5,463
1561044_at	7,903	7,545	7,458	8,571	7,887	7,851
1561045_a_at	6,284	6,199	6,319	6,956	6,630	6,844
1561048_at	6,622	6,639	6,617	7,296	6,947	7,152
1561050_a_at	6,609	6,072	6,725	7,633	7,720	7,811
1561052_s_at	6,072	6,204	6,210	6,965	6,889	7,247
1561053_at	4,269	4,105	4,595	4,541	4,469	4,610
1561054_a_at	4,748	4,650	4,417	4,855	4,958	4,577
1561055_at	4,445	4,302	5,268	4,898	4,670	4,826
1561056_a_at	6,606	6,607	6,922	7,222	7,050	7,188
1561058_at	5,086	5,274	5,223	5,827	5,611	5,721
1561059_a_at	5,109	5,031	4,724	5,529	5,650	5,644
1561060_at	6,363	6,659	6,782	6,826	6,994	6,948
1561061_at	6,665	6,553	6,764	7,570	7,440	7,505
1561062_a_at	5,576	5,348	5,979	5,776	5,738	5,619
1561064_a_at	5,286	5,515	5,421	6,358	6,304	6,475
1561065_at	5,551	5,414	5,735	5,832	5,907	5,902
1561067_at	4,383	4,263	4,701	4,681	4,629	4,551
1561069_at	3,816	3,645	3,708	4,329	4,190	4,463
1561073_at	4,200	4,043	4,284	4,326	4,160	4,311
1561075_at	6,086	6,370	6,201	6,473	6,300	6,461
1561076_at	4,178	4,294	4,342	4,456	4,345	4,315
1561078_at	6,685	6,311	6,494	6,649	6,288	6,349
1561079_at	6,585	5,921	5,532	5,291	7,406	5,257
1561080_at	3,829	3,910	3,764	3,807	3,648	3,960
1561082_at	7,107	6,857	7,074	7,319	7,329	7,407
1561083_at	4,975	4,613	4,844	5,400	5,209	5,476
1561084_at	4,989	4,415	4,576	5,107	5,047	4,925
1561085_at	7,486	7,408	7,396	7,924	7,946	8,164
1561086_at	4,548	4,648	4,686	4,636	4,570	4,454
1561087_at	4,527	4,754	4,382	4,391	4,234	4,376
1561088_at	4,507	4,437	4,390	4,661	4,555	4,516
1561089_at	5,973	6,178	5,848	6,487	6,548	6,758
1561090_at	3,942	4,213	4,201	4,125	3,932	4,062
1561091_at	5,423	5,802	5,770	6,655	6,978	7,079
1561092_at	5,323	5,359	5,112	5,477	5,358	5,568
1561093_at	8,219	8,268	8,426	8,363	8,442	8,416
1561094_a_at	3,679	3,863	4,042	4,033	3,771	3,950
1561096_at	4,454	4,685	5,076	4,675	4,613	4,685
1561097_at	6,058	6,530	6,289	6,689	6,514	6,581
1561098_at	4,421	4,365	4,755	5,551	5,265	5,570
1561099_at	5,695	5,572	6,093	6,156	6,264	6,217
1561100_at	5,524	5,376	5,572	6,236	6,182	6,495
1561101_at	8,951	8,899	9,749	5,950	5,955	6,091
1561102_at	7,719	7,854	7,701	8,240	8,188	8,177
1561103_at	5,779	5,564	5,884	6,133	5,629	5,514

1561104_at	4,895	5,103	4,510	5,325	5,127	5,351
1561105_at	4,351	4,473	4,154	4,814	4,749	5,067
1561106_at	5,412	5,550	5,333	5,445	5,430	5,465
1561107_at	7,768	7,358	7,614	7,797	7,568	7,800
1561110_at	3,327	3,519	3,361	4,040	4,014	4,111
1561112_at	4,718	4,867	4,761	4,993	5,049	5,080
1561113_at	4,884	4,481	4,941	5,375	4,930	4,914
1561114_a_at	4,244	4,451	4,186	4,174	3,797	4,101
1561116_at	6,658	6,652	6,790	7,272	6,928	7,345
1561121_at	4,736	4,325	4,667	4,188	4,287	4,104
1561122_a_at	6,783	6,744	6,799	6,911	6,557	6,941
1561123_at	4,836	4,670	5,212	5,339	5,481	4,986
1561125_at	5,579	5,693	5,508	6,399	6,196	6,255
1561126_at	5,936	5,964	5,921	6,607	6,590	6,991
1561127_at	7,850	7,980	8,006	8,537	8,343	8,447
1561128_at	5,454	5,831	5,788	6,713	6,877	7,159
1561129_at	4,457	4,540	4,259	4,501	4,578	4,683
1561130_at	5,308	4,915	5,951	5,521	5,603	5,867
1561132_at	5,313	5,289	5,775	6,138	6,078	6,300
1561133_at	7,205	6,900	7,109	7,653	7,158	7,303
1561134_at	5,116	5,199	5,348	6,078	5,464	5,521
1561135_at	6,606	6,941	6,952	6,527	6,562	6,515
1561136_at	3,457	3,595	3,592	3,636	3,558	3,679
1561137_s_at	3,652	3,685	3,780	3,721	3,627	3,575
1561139_at	5,444	5,354	5,621	4,664	4,946	4,518
1561140_at	4,433	4,717	4,515	4,761	4,548	4,929
1561141_at	6,721	6,582	6,219	7,000	6,919	6,960
1561143_at	5,564	5,517	5,322	5,851	5,431	5,761
1561144_at	4,865	4,812	4,847	4,872	4,565	4,660
1561146_at	6,255	6,034	5,913	5,430	5,217	5,351
1561148_at	5,040	5,125	4,851	4,848	5,209	5,105
1561149_at	5,019	4,713	4,939	4,541	4,441	4,451
1561151_a_at	3,586	3,990	3,640	3,465	3,518	3,626
1561152_a_at	5,598	5,639	5,143	5,322	5,181	5,285
1561153_at	7,009	7,136	7,181	7,456	7,456	7,545
1561154_at	3,737	3,834	3,759	3,876	3,752	3,949
1561155_at	5,630	5,994	5,736	5,352	5,712	5,567
1561156_at	3,407	3,352	3,530	3,515	3,468	3,593
1561158_at	5,493	4,746	5,477	4,921	4,584	4,707
1561161_at	5,511	5,747	5,326	5,143	5,134	5,215
1561164_at	4,347	3,886	4,393	4,310	4,372	4,324
1561165_a_at	5,289	5,575	5,202	6,236	5,898	5,899
1561166_a_at	6,438	5,245	5,783	3,230	3,250	3,265
1561167_at	5,993	5,631	6,851	4,476	5,851	4,726
1561169_at	5,624	5,361	5,460	5,991	5,372	5,406
1561170_at	3,956	4,353	4,427	5,144	5,074	5,493
1561171_a_at	7,541	7,111	7,220	8,185	7,912	8,124
1561175_at	5,728	5,660	5,990	6,492	6,616	6,464
1561177_at	3,334	3,456	3,393	3,307	3,381	3,411
1561179_s_at	7,824	8,022	8,209	8,751	8,796	9,001
1561180_at	4,377	4,469	4,552	5,496	5,346	5,687
1561181_at	5,574	5,703	5,660	5,928	6,751	6,299
1561185_at	6,312	6,089	6,541	6,782	6,420	6,795
1561187_at	5,747	5,658	5,759	5,962	5,885	5,922
1561190_at	7,382	6,416	7,076	7,056	7,644	7,129
1561191_at	5,520	5,436	5,569	5,718	5,696	5,756

1561192_at	4,579	4,551	4,513	4,558	4,706	4,826
1561194_at	5,486	6,061	5,637	6,218	6,113	6,396
1561195_at	8,650	8,247	8,405	5,608	6,033	5,645
1561196_at	4,360	4,624	4,532	5,073	4,776	5,098
1561197_at	5,026	4,961	5,140	5,030	5,348	5,144
1561198_at	3,889	3,720	3,719	3,636	3,444	3,671
1561199_at	4,930	4,672	5,144	5,359	5,164	5,396
1561200_at	3,727	3,773	4,283	4,318	4,367	4,446
1561202_at	6,453	6,575	6,606	6,845	6,797	6,569
1561204_at	4,027	4,087	4,209	4,466	4,229	4,344
1561205_at	5,039	5,319	5,529	5,490	5,483	5,806
1561206_at	5,346	5,277	5,471	4,045	4,419	4,037
1561207_at	6,338	6,262	6,821	6,737	6,809	6,761
1561208_at	5,398	5,501	5,704	5,720	5,505	5,622
1561210_at	4,039	3,917	3,670	4,351	4,052	4,140
1561211_at	6,753	6,719	6,621	6,542	6,352	6,517
1561212_at	4,981	4,768	4,892	4,920	5,034	4,723
1561213_at	3,272	3,525	3,616	3,508	3,351	3,332
1561214_at	6,318	6,149	6,531	6,885	6,894	6,861
1561215_at	3,922	3,932	3,972	3,835	3,597	3,769
1561216_at	3,605	3,914	3,751	4,146	4,100	4,289
1561217_at	5,497	5,928	5,404	5,643	5,608	5,535
1561218_s_at	5,259	5,134	5,322	5,390	5,485	5,642
1561219_x_at	5,994	5,964	6,068	6,200	6,044	6,312
1561221_x_at	6,062	6,107	6,008	6,100	6,033	6,205
1561222_at	3,551	3,487	3,416	3,407	3,374	3,383
1561223_at	5,893	5,667	5,617	6,750	6,584	6,712
1561224_at	5,366	5,402	5,769	6,002	5,501	5,894
1561225_at	6,114	6,296	6,535	6,460	6,221	6,336
1561226_at	5,882	5,079	5,567	6,965	6,410	6,417
1561228_at	6,422	5,920	6,116	6,533	6,168	6,393
1561229_at	4,352	4,253	4,363	4,753	4,613	4,992
1561230_at	3,640	3,631	3,562	3,856	3,819	4,208
1561232_at	5,773	5,233	5,566	5,954	5,753	5,834
1561233_at	6,746	6,788	6,513	7,223	7,349	7,052
1561234_at	3,860	3,844	3,805	3,645	3,509	3,583
1561236_at	4,875	5,260	4,921	5,072	5,160	5,185
1561237_at	6,532	6,233	6,656	7,562	7,011	7,028
1561238_at	4,885	4,761	4,837	4,501	4,468	4,605
1561239_at	4,367	4,742	4,520	4,499	4,512	4,328
1561240_at	3,527	3,589	3,528	3,535	3,435	3,932
1561242_at	4,688	5,201	4,731	4,913	5,299	4,873
1561243_at	8,220	8,215	8,439	9,063	9,269	9,203
1561244_at	4,033	4,034	4,084	4,224	4,176	4,429
1561245_at	5,899	5,831	5,704	6,720	6,672	7,003
1561247_at	3,741	3,593	3,746	4,040	3,811	3,946
1561249_a_at	7,298	6,863	7,060	8,008	7,758	8,105
1561250_at	5,032	5,125	5,172	5,374	5,753	5,759
1561251_at	6,642	6,817	6,908	7,041	6,952	6,874
1561252_at	5,102	4,972	5,072	5,616	5,355	5,582
1561253_at	4,360	4,787	4,410	4,695	4,735	4,830
1561254_at	4,671	5,074	4,939	4,839	4,774	4,861
1561255_at	4,522	4,421	4,627	4,614	4,591	4,595
1561256_at	5,371	5,135	5,112	4,976	4,836	4,786
1561257_at	6,271	5,782	5,980	6,645	6,609	6,599
1561258_at	5,777	6,184	5,752	6,738	6,378	6,532

1561260_at	4,672	4,953	5,175	5,157	5,375	5,315
1561261_at	5,627	5,602	5,947	6,312	5,981	6,161
1561262_at	4,811	4,974	4,314	5,031	5,159	5,060
1561263_at	4,935	5,251	5,178	4,932	5,201	5,191
1561264_at	4,017	3,684	3,908	4,095	3,862	3,958
1561266_at	5,826	5,879	6,249	6,447	6,498	6,898
1561268_at	5,795	6,009	5,888	6,031	6,108	6,266
1561269_at	5,702	6,116	5,775	5,897	6,087	6,002
1561270_at	6,388	6,351	6,605	6,741	6,474	6,693
1561271_at	4,386	4,158	4,150	4,479	4,518	4,758
1561272_at	5,192	5,087	4,633	4,480	4,606	4,653
1561273_at	6,125	6,472	5,931	6,269	6,155	6,318
1561274_at	6,048	5,573	5,545	5,476	5,491	5,687
1561276_at	4,919	4,592	4,850	4,725	4,780	4,566
1561277_at	4,954	5,198	4,855	5,640	5,594	6,026
1561278_at	5,272	5,120	5,000	5,239	5,420	5,311
1561279_at	5,007	5,015	4,774	5,034	5,068	5,324
1561280_at	3,614	3,955	3,481	3,619	3,639	3,633
1561281_a_at	4,911	5,546	5,332	5,829	5,535	5,765
1561286_a_at	6,289	6,701	6,547	6,759	6,168	6,322
1561287_at	4,283	4,407	4,400	4,495	4,524	4,403
1561288_at	4,849	4,975	5,013	6,117	6,262	6,533
1561289_at	4,229	4,192	4,230	4,587	4,257	4,203
1561290_at	3,986	4,114	3,884	4,044	4,044	4,059
1561291_at	5,391	5,372	4,992	5,385	5,808	5,614
1561292_at	5,936	5,827	5,620	5,367	5,293	5,322
1561293_at	6,721	7,053	6,701	7,059	6,861	6,999
1561294_a_at	5,920	5,716	5,518	6,105	5,916	6,025
1561297_at	4,435	4,521	4,421	4,607	4,693	4,694
1561298_at	6,367	6,472	6,531	6,759	7,435	6,874
1561300_at	3,952	4,275	4,139	4,469	4,343	4,703
1561301_at	6,348	6,226	6,384	7,029	7,053	6,956
1561303_at	4,981	5,050	5,320	5,150	5,082	5,181
1561304_a_at	7,806	7,835	7,691	8,710	8,585	8,600
1561305_at	6,628	6,846	6,785	7,597	7,656	7,269
1561306_s_at	6,716	6,778	6,881	6,968	6,703	6,859
1561307_at	5,269	4,927	5,190	5,504	5,443	5,660
1561309_x_at	6,272	5,847	6,172	6,773	6,510	6,605
1561310_at	5,513	5,455	5,725	5,902	5,681	5,877
1561311_at	5,452	5,531	5,649	5,977	5,950	6,326
1561313_at	6,595	6,615	6,403	6,714	6,635	6,575
1561314_at	6,579	6,965	6,831	7,473	7,175	7,476
1561316_at	4,207	5,474	5,363	4,121	4,040	4,288
1561318_at	7,049	6,966	7,126	7,859	7,800	7,790
1561319_at	5,470	4,910	5,226	5,232	4,972	5,149
1561320_at	5,958	6,262	6,322	7,044	6,649	7,303
1561322_at	3,726	4,046	3,816	4,121	4,277	4,190
1561323_at	6,304	6,296	6,187	6,725	6,375	6,519
1561324_at	5,574	5,659	5,485	5,626	5,364	5,718
1561327_at	6,420	6,233	6,413	7,260	6,778	7,040
1561328_at	4,010	4,086	4,044	4,205	4,202	4,097
1561329_s_at	7,244	6,933	7,058	7,206	7,244	7,082
1561330_at	3,990	3,711	3,904	4,183	4,233	4,316
1561331_at	3,630	3,771	3,730	3,859	3,638	3,824
1561332_at	6,656	6,719	6,882	7,442	6,619	7,117
1561333_at	5,024	4,825	4,845	5,263	5,362	5,206

1561334_at	5,172	5,328	5,246	5,260	5,333	5,603
1561335_at	7,665	7,350	7,694	8,307	7,683	8,282
1561336_at	6,212	6,136	6,334	6,865	6,711	6,779
1561339_at	4,636	4,953	4,876	4,969	4,767	4,826
1561340_at	3,696	4,014	3,993	3,843	3,916	3,717
1561341_at	8,611	7,596	8,275	5,732	5,780	5,726
1561342_at	5,224	5,024	5,318	7,341	7,184	7,509
1561343_a_at	3,695	3,912	4,009	4,071	4,030	4,049
1561345_at	5,356	5,520	5,849	6,304	6,214	6,377
1561346_at	9,194	8,998	9,001	8,933	9,792	9,159
1561347_a_at	4,214	4,232	4,629	4,419	4,334	4,538
1561348_at	5,958	6,454	5,862	7,263	6,710	6,910
1561351_at	4,367	4,467	4,681	4,340	4,344	4,275
1561352_at	5,973	5,951	6,168	6,352	6,036	6,162
1561353_at	4,498	4,311	4,464	4,690	4,413	4,420
1561354_at	6,164	6,250	6,729	7,253	7,264	7,497
1561355_at	8,496	8,200	8,543	8,501	8,239	8,318
1561358_at	7,200	6,567	6,564	7,453	7,503	7,290
1561360_at	6,459	6,599	6,716	6,781	6,589	6,880
1561361_at	7,749	7,730	7,124	6,073	6,142	6,191
1561362_at	4,162	4,149	4,215	3,920	4,303	4,042
1561363_a_at	4,366	4,437	4,476	4,444	4,357	4,601
1561364_at	4,038	3,952	4,122	5,541	5,030	5,648
1561365_at	3,771	4,076	3,739	4,627	4,707	4,714
1561367_a_at	5,627	6,149	5,869	6,164	5,738	6,007
1561368_at	3,665	3,856	3,647	4,111	3,997	3,967
1561369_at	7,605	7,286	7,793	7,687	7,620	7,922
1561370_at	5,715	5,964	5,832	6,061	6,182	6,175
1561371_at	6,064	5,939	5,775	6,301	6,012	6,079
1561373_at	6,648	6,646	6,322	6,392	6,451	6,533
1561375_at	5,643	5,634	5,837	6,190	5,959	5,953
1561378_at	5,521	5,640	5,838	6,538	6,231	6,671
1561383_at	5,360	5,409	5,048	6,039	5,800	5,651
1561384_a_at	5,555	5,355	5,414	5,876	5,949	5,622
1561386_at	6,751	6,624	6,876	7,267	7,367	7,363
1561387_a_at	6,486	6,113	6,269	6,923	7,068	7,106
1561389_at	5,788	6,041	5,967	6,509	6,044	6,326
1561390_at	7,776	7,637	7,456	7,911	8,024	8,159
1561391_at	7,200	6,844	7,332	7,307	6,856	7,121
1561392_at	5,304	5,289	5,280	5,491	5,262	5,598
1561393_at	8,064	7,632	7,859	8,426	8,223	8,121
1561394_s_at	6,690	6,765	6,170	7,536	7,460	7,695
1561396_at	3,857	4,002	3,860	4,187	3,986	4,044
1561398_at	5,007	5,026	5,749	6,242	5,962	5,811
1561400_at	5,983	5,675	5,993	6,296	6,295	6,242
1561401_at	5,181	4,797	5,286	5,396	5,168	5,264
1561402_at	7,387	7,053	7,173	7,957	7,599	7,588
1561403_at	7,024	7,170	7,127	7,799	7,813	8,004
1561405_s_at	5,661	5,938	5,899	6,186	6,113	6,091
1561407_at	5,432	5,566	5,712	6,256	6,177	6,323
1561408_at	5,133	5,040	5,172	5,434	5,156	5,328
1561409_at	6,632	6,535	6,809	6,995	6,878	6,919
1561411_at	6,457	6,355	6,590	6,979	6,749	6,874
1561413_at	5,873	6,083	5,875	6,752	6,783	6,834
1561414_at	3,894	3,753	3,968	4,798	4,346	4,491
1561415_at	3,563	4,049	3,749	4,208	3,857	4,053

1561417_x_at	4,011	4,118	4,272	4,866	4,719	4,664
1561418_at	5,489	5,398	5,668	6,140	5,882	6,124
1561419_at	7,823	7,545	7,485	7,915	8,086	8,032
1561420_a_at	5,402	5,413	5,488	5,734	5,818	5,786
1561421_a_at	7,151	7,212	7,384	7,296	7,480	7,556
1561422_at	6,215	6,141	6,146	6,216	6,205	6,227
1561423_at	6,698	6,511	6,686	7,530	7,618	7,400
1561424_at	4,861	5,081	4,920	5,046	4,940	5,009
1561425_a_at	5,485	5,000	5,725	5,195	4,959	5,037
1561427_at	5,927	5,986	6,081	5,698	5,999	5,892
1561429_a_at	5,925	5,921	5,975	6,945	6,730	7,014
1561430_s_at	5,867	5,532	5,853	6,444	6,421	6,591
1561431_at	4,032	4,006	4,230	4,718	4,225	4,546
1561432_at	4,592	4,874	4,641	5,192	4,941	5,312
1561433_at	6,867	6,886	6,719	7,531	7,189	7,687
1561434_at	6,286	6,391	6,136	6,293	6,604	6,735
1561436_at	6,040	6,220	6,294	6,898	6,788	6,867
1561437_at	3,453	3,773	3,598	3,639	3,646	3,702
1561439_at	7,343	7,073	7,266	7,945	7,830	7,965
1561440_at	6,311	6,219	6,628	6,344	6,245	6,271
1561442_at	4,375	4,020	4,419	4,383	4,011	4,218
1561443_at	5,186	4,949	5,474	6,028	5,721	5,845
1561445_at	5,260	5,399	5,172	6,001	5,705	6,049
1561446_at	6,175	5,865	6,227	6,311	6,482	6,181
1561448_at	5,058	5,260	5,197	5,347	5,296	5,321
1561449_at	7,487	7,321	7,372	7,846	7,624	7,997
1561450_at	4,160	4,194	4,293	4,251	4,485	4,681
1561451_a_at	6,260	5,838	6,129	6,039	5,998	5,851
1561452_at	4,178	4,026	4,237	4,737	4,331	4,651
1561453_at	6,557	6,013	6,378	6,969	7,168	6,672
1561454_at	6,912	7,167	7,069	7,567	7,440	7,495
1561455_at	5,575	5,416	5,584	5,894	5,917	6,046
1561459_at	6,451	7,096	7,139	7,286	7,134	7,546
1561460_at	6,073	6,010	6,139	6,421	6,494	6,538
1561461_at	4,666	4,656	4,494	4,749	4,820	4,867
1561462_at	5,599	5,400	4,940	6,057	6,067	6,432
1561463_at	4,527	4,533	4,765	4,608	4,374	4,545
1561467_at	4,194	4,356	4,104	4,066	3,982	4,142
1561468_at	4,438	4,770	4,920	5,707	5,293	5,371
1561469_at	5,064	5,102	5,290	5,582	5,855	5,841
1561471_at	5,665	5,488	5,343	5,475	5,377	5,519
1561472_at	6,083	6,059	5,649	6,634	6,751	6,892
1561473_at	3,941	4,169	4,205	4,189	4,224	4,416
1561474_at	5,564	5,566	5,405	6,649	6,636	6,807
1561476_at	5,661	5,598	5,605	5,465	5,561	5,631
1561477_at	3,914	4,224	3,981	3,976	4,140	3,961
1561478_at	4,679	4,578	4,784	5,463	5,085	5,221
1561479_at	5,640	5,636	5,719	6,469	6,344	6,417
1561480_a_at	6,337	5,879	6,137	6,578	6,440	6,490
1561481_at	7,281	7,214	7,463	7,862	8,030	8,140
1561482_at	4,121	4,594	4,309	4,484	4,184	4,113
1561484_at	6,621	6,723	6,889	7,529	7,546	7,550
1561485_at	6,570	6,691	6,375	7,056	6,789	6,779
1561486_at	4,026	4,044	4,173	3,840	3,770	3,663
1561487_at	5,786	5,549	5,862	5,942	5,870	6,057
1561488_at	4,104	4,174	4,285	4,310	4,092	4,137

1561489_at	5,249	4,953	4,985	4,873	4,974	5,033
1561490_at	5,763	5,846	5,721	5,891	5,823	5,844
1561491_at	4,948	4,982	4,808	5,918	5,642	5,809
1561492_at	4,890	5,017	5,102	5,307	5,260	5,217
1561493_at	4,589	4,697	4,621	5,203	5,028	5,370
1561497_at	6,677	6,374	6,535	7,454	7,515	7,858
1561499_at	5,959	6,144	5,922	6,350	6,191	6,334
1561500_at	7,117	7,043	7,314	8,291	8,431	8,529
1561501_s_at	4,267	4,155	4,326	5,871	6,098	6,313
1561502_x_at	6,971	7,179	7,418	7,987	8,296	8,473
1561503_at	6,128	5,934	6,203	6,240	5,929	6,354
1561504_s_at	4,191	4,435	4,264	4,311	4,179	4,120
1561506_at	6,998	6,967	6,974	7,335	7,058	7,128
1561507_at	4,446	4,686	4,705	4,573	4,553	4,669
1561508_at	5,614	5,595	5,711	6,225	6,218	6,226
1561509_at	5,638	5,275	5,937	6,129	6,480	6,379
1561511_at	3,648	4,141	4,008	3,740	3,704	3,816
1561512_at	4,496	4,525	4,278	4,837	4,726	4,744
1561513_at	4,072	4,053	4,027	4,190	4,073	4,178
1561514_at	4,292	4,061	4,067	4,736	4,623	5,005
1561516_at	4,646	4,609	4,736	5,260	5,170	4,962
1561517_at	4,213	4,412	4,335	4,643	4,369	4,627
1561518_at	6,942	7,012	7,046	7,387	7,404	7,661
1561519_at	5,620	5,802	6,125	5,733	5,611	5,397
1561521_at	3,498	3,468	3,715	3,559	3,514	3,567
1561523_at	5,155	5,060	5,426	5,882	5,315	5,836
1561525_at	4,616	4,478	4,974	4,651	4,673	4,536
1561526_at	6,303	6,283	6,305	6,608	6,706	6,606
1561527_at	4,543	4,977	4,685	6,060	5,654	6,056
1561528_at	5,402	4,947	5,209	5,820	5,863	6,299
1561529_at	4,247	4,001	4,305	5,012	4,628	5,297
1561530_at	6,641	6,636	6,676	7,015	6,704	6,819
1561531_at	6,974	6,600	6,525	7,196	7,170	7,355
1561532_at	5,140	5,098	5,069	5,755	5,645	5,713
1561534_at	6,860	6,506	6,964	6,579	6,681	6,708
1561535_at	5,482	5,528	5,845	5,737	5,560	5,734
1561536_at	5,856	5,835	5,831	5,861	5,775	5,883
1561537_at	7,953	7,743	7,825	8,360	8,443	8,497
1561538_at	7,139	7,161	7,104	7,497	7,177	7,429
1561539_at	6,812	7,108	7,127	8,597	8,143	8,243
1561540_at	5,410	5,366	4,887	5,502	5,403	5,479
1561542_at	7,104	7,196	7,128	7,388	7,236	7,260
1561543_at	6,386	6,195	6,536	6,739	6,541	6,798
1561544_at	4,836	4,627	5,071	5,307	5,290	5,478
1561545_at	6,173	5,390	5,986	5,778	6,080	5,913
1561546_at	7,473	7,366	7,467	8,303	8,234	8,091
1561550_at	4,396	4,133	4,440	4,118	4,222	4,409
1561553_at	3,608	3,672	3,625	3,858	3,783	4,013
1561554_at	4,485	4,790	4,458	4,872	4,520	4,656
1561555_at	6,691	6,667	6,934	6,737	6,615	6,760
1561556_at	5,598	5,468	5,468	5,455	5,284	5,429
1561557_at	6,414	6,240	6,100	6,409	6,217	6,460
1561558_at	4,746	4,235	4,772	4,812	4,850	5,124
1561559_at	5,284	4,944	5,390	5,813	5,537	5,746
1561560_at	4,353	4,808	4,592	4,551	4,490	4,634
1561561_x_at	5,564	5,743	5,799	5,901	5,782	6,121

1561562_at	3,779	3,757	3,794	3,793	3,891	4,051
1561564_at	3,347	3,661	3,436	3,678	3,602	3,691
1561565_at	5,631	5,308	5,310	5,766	5,739	5,737
1561566_at	4,659	4,510	4,576	4,610	4,458	4,372
1561567_at	4,130	3,953	4,308	4,290	4,341	4,272
1561571_at	9,520	9,051	9,450	9,987	9,782	10,204
1561572_at	5,826	5,486	5,801	5,864	6,049	5,931
1561573_at	5,300	5,294	5,454	5,474	5,888	5,684
1561574_at	6,370	6,190	6,298	7,010	7,313	7,199
1561575_at	4,428	4,587	4,788	4,988	4,952	4,772
1561576_a_at	4,910	4,889	4,730	4,997	4,720	4,712
1561577_at	4,453	4,355	4,476	4,858	4,631	4,739
1561578_s_at	6,307	5,856	6,044	5,906	5,653	5,799
1561579_at	6,560	6,766	6,730	7,587	7,551	7,571
1561581_at	3,588	3,695	3,518	4,902	4,659	4,889
1561584_at	3,881	3,977	3,769	4,053	4,003	4,196
1561585_at	5,853	5,815	5,782	6,299	6,049	6,265
1561587_at	6,543	6,544	6,632	7,293	6,880	7,133
1561589_a_at	4,604	4,229	4,681	4,728	5,022	4,850
1561590_a_at	5,493	5,950	6,104	6,788	6,552	6,978
1561591_at	5,506	5,551	5,328	6,009	6,024	6,102
1561592_at	5,329	5,390	4,919	6,536	6,599	6,703
1561593_at	3,805	3,842	4,034	3,846	3,855	3,760
1561595_x_at	5,948	6,070	6,097	6,018	5,832	6,049
1561596_at	4,405	3,487	4,154	5,457	5,216	5,350
1561597_x_at	6,736	6,514	6,748	7,714	7,898	7,527
1561598_at	7,745	7,706	7,632	7,828	7,246	7,319
1561599_at	6,253	6,162	6,178	6,249	6,299	6,373
1561600_at	4,065	4,176	3,998	4,354	4,776	4,688
1561602_at	6,948	7,106	6,961	7,978	8,031	8,116
1561604_at	4,647	4,562	4,518	4,726	4,672	4,597
1561605_at	4,856	5,028	4,851	5,248	5,108	5,092
1561606_at	3,729	3,717	3,936	3,756	3,777	3,790
1561607_at	5,757	5,754	5,978	6,627	6,106	6,610
1561608_at	5,481	5,787	5,247	6,154	6,181	6,038
1561609_at	4,603	4,992	5,158	5,199	5,245	5,182
1561610_at	5,859	5,661	5,478	6,621	6,225	6,406
1561611_at	7,421	7,697	7,558	8,051	7,827	8,020
1561612_at	5,701	5,846	6,196	6,243	6,290	6,249
1561613_at	4,637	4,325	4,697	5,396	5,043	5,305
1561614_at	4,744	4,613	4,814	4,776	4,824	4,808
1561615_s_at	4,955	5,204	5,204	5,246	5,367	5,682
1561616_a_at	7,245	7,053	7,171	7,546	7,554	7,574
1561617_at	4,627	4,601	4,399	4,979	4,737	4,836
1561618_at	4,751	4,839	5,139	5,255	5,073	5,260
1561619_at	7,743	7,443	7,618	8,188	7,854	7,844
1561622_at	4,998	4,915	4,867	5,848	5,428	5,517
1561624_at	6,529	6,632	7,124	6,792	6,745	6,791
1561625_at	5,364	5,202	5,271	5,604	5,982	5,795
1561626_at	7,545	7,347	7,547	8,048	8,171	8,214
1561627_at	5,487	5,139	5,193	5,757	5,584	5,553
1561631_at	4,403	4,419	5,039	4,537	5,173	4,481
1561633_at	4,251	4,361	4,608	7,544	7,162	8,301
1561635_at	6,728	6,509	6,853	7,664	7,358	7,879
1561638_at	5,085	4,962	5,006	5,145	5,184	5,041
1561639_at	5,711	5,820	5,587	5,900	5,517	5,909

1561640_at	6,634	6,429	6,078	6,087	6,111	6,060
1561642_at	5,497	4,804	4,745	4,393	4,149	4,159
1561644_x_at	3,359	3,604	3,486	3,343	3,601	3,560
1561650_s_at	4,621	4,911	4,789	5,381	5,357	5,707
1561651_s_at	7,996	7,765	8,204	9,024	8,489	8,641
1561652_at	5,637	5,640	5,557	6,277	5,801	5,951
1561653_at	3,914	3,970	3,810	3,838	3,829	3,908
1561654_at	5,984	5,356	6,095	5,705	5,689	5,758
1561657_at	3,918	3,991	4,201	3,926	3,839	3,834
1561658_at	4,563	4,469	4,354	4,861	5,067	5,082
1561660_at	5,556	5,925	5,402	4,172	4,351	4,282
1561662_at	4,153	4,581	4,325	4,659	4,509	4,610
1561663_at	3,727	3,452	3,941	3,822	3,698	3,738
1561665_at	5,537	4,985	5,256	6,208	5,813	6,056
1561666_a_at	8,238	7,866	8,386	6,879	7,463	7,315
1561667_at	6,252	6,418	6,688	6,748	6,614	6,695
1561668_at	6,540	6,719	6,688	6,868	6,760	6,814
1561669_at	4,843	4,825	4,675	5,171	4,864	4,669
1561670_at	4,875	4,997	5,096	5,983	5,800	5,578
1561671_at	6,072	6,081	6,241	6,713	6,551	6,775
1561672_at	4,214	4,690	4,046	4,130	4,199	4,133
1561673_at	5,982	6,107	5,922	6,120	6,317	6,071
1561676_at	5,159	5,115	5,532	5,194	5,161	4,974
1561677_at	4,577	4,272	4,527	4,845	4,592	4,769
1561678_at	4,372	4,772	4,517	4,573	4,457	4,891
1561679_at	5,066	5,064	5,049	5,215	5,052	4,821
1561680_at	3,905	3,985	4,108	4,134	4,015	4,048
1561682_at	6,359	6,524	6,628	6,635	6,522	6,593
1561683_at	6,518	6,817	6,623	6,942	6,950	6,930
1561685_a_at	6,600	6,435	6,621	6,977	7,009	7,096
1561686_at	6,656	6,289	6,454	6,043	6,100	6,165
1561687_a_at	6,018	5,989	6,058	5,156	4,810	4,573
1561688_at	6,180	5,930	5,796	6,924	6,843	6,904
1561689_at	6,531	6,841	6,476	7,277	7,137	7,453
1561690_at	5,945	6,275	6,047	5,444	5,799	5,268
1561691_at	5,026	5,429	5,720	6,412	5,804	5,910
1561692_at	4,364	4,644	4,346	4,645	4,457	4,442
1561693_at	5,813	6,026	5,813	5,609	5,389	5,464
1561699_a_at	3,857	4,040	3,942	4,159	3,983	4,058
1561700_at	5,977	6,219	6,399	6,502	6,435	6,532
1561702_at	6,093	6,278	6,399	6,860	6,590	6,817
1561703_at	3,794	3,881	4,231	4,225	4,450	4,688
1561704_at	4,224	4,313	4,356	5,086	4,676	5,005
1561705_at	7,145	6,884	7,392	8,030	7,726	7,916
1561706_at	6,419	6,126	6,281	6,192	6,203	5,982
1561707_at	4,612	4,470	4,419	5,858	6,298	6,282
1561708_at	4,716	4,652	4,991	5,026	4,810	4,930
1561710_at	5,147	5,138	5,311	5,904	5,787	5,821
1561712_at	6,466	6,313	6,665	6,935	6,861	6,688
1561713_at	3,987	4,555	3,922	4,068	3,940	4,219
1561714_a_at	6,125	6,582	6,369	6,308	6,603	6,392
1561718_at	5,532	5,358	5,835	6,057	6,306	5,948
1561719_at	3,990	4,110	3,897	4,722	4,705	4,766
1561720_at	4,121	3,981	4,014	4,440	4,372	4,467
1561721_a_at	3,732	3,806	3,805	3,825	3,809	3,818
1561723_at	5,238	5,206	5,460	5,298	5,594	5,440

1561726_s_at	7,265	7,339	6,953	7,428	7,196	7,508
1561728_a_at	5,308	5,892	5,880	7,112	6,816	7,391
1561731_at	4,312	4,678	4,077	5,065	5,021	5,129
1561732_at	6,282	6,187	6,579	7,234	7,072	7,580
1561733_at	6,291	5,806	6,043	5,825	5,843	5,977
1561737_at	6,154	6,848	6,330	6,285	6,007	6,196
1561738_at	5,350	5,104	5,564	5,918	5,667	5,801
1561739_at	5,949	5,719	5,805	5,582	5,566	5,526
1561740_at	4,493	4,576	4,653	4,524	4,533	4,440
1561741_at	4,545	4,744	4,684	4,819	4,507	4,651
1561749_at	7,990	8,491	7,781	7,708	7,608	7,827
1561754_at	5,236	4,983	4,620	4,587	4,483	4,716
1561756_at	6,477	6,085	6,462	6,720	6,550	6,476
1561757_a_at	5,129	4,720	5,127	5,351	5,126	5,197
1561759_at	5,786	5,902	5,396	5,322	5,462	5,438
1561760_s_at	5,566	6,216	5,518	5,175	4,991	4,942
1561761_x_at	6,003	6,891	6,093	6,010	5,970	5,831
1561762_s_at	5,539	5,340	5,629	5,221	5,654	5,418
1561763_at	5,361	5,293	5,186	4,907	4,740	4,739
1561765_at	3,272	3,375	3,298	3,241	3,282	3,238
1561767_at	5,451	5,731	5,267	6,566	6,115	6,441
1561770_at	6,629	6,466	6,531	7,077	7,229	7,383
1561775_at	3,562	3,502	3,442	3,499	3,400	3,497
1561776_at	5,364	5,645	4,801	5,801	5,879	6,075
1561777_at	4,616	4,658	4,927	4,481	4,564	4,222
1561778_at	5,812	5,857	5,876	6,771	6,752	6,770
1561780_at	3,961	3,966	4,410	4,237	4,209	4,123
1561785_at	6,677	6,649	6,540	7,040	7,202	7,138
1561786_at	5,286	5,283	5,326	5,000	4,988	4,979
1561789_at	3,640	3,557	3,758	3,661	3,682	3,634
1561795_at	4,779	4,562	4,894	4,946	4,762	4,927
1561806_at	5,192	5,580	5,679	6,485	6,277	6,352
1561813_at	4,876	4,880	4,959	5,361	5,007	5,451
1561817_at	5,010	4,933	4,447	5,256	5,297	5,536
1561820_at	5,780	5,934	5,546	6,240	6,174	6,481
1561828_at	3,665	3,681	3,940	3,970	3,896	3,941
1561829_at	6,488	6,292	6,249	7,175	6,783	7,266
1561834_a_at	6,895	6,443	6,648	7,076	7,055	7,375
1561844_at	6,049	5,896	5,899	6,175	6,356	6,225
1561846_s_at	4,960	5,094	4,724	5,463	5,014	5,303
1561847_at	6,402	6,175	6,363	7,208	7,017	7,490
1561849_at	6,095	6,226	6,093	6,341	6,150	6,495
1561850_at	7,201	6,696	7,176	8,130	8,032	8,076
1561853_a_at	5,932	5,788	6,259	7,041	6,290	6,737
1561854_at	3,617	3,362	3,594	4,025	3,720	4,036
1561855_x_at	4,260	4,333	4,129	4,854	4,574	4,587
1561856_at	4,100	4,018	4,197	4,519	4,406	4,151
1561857_at	4,115	3,890	4,150	4,841	4,510	4,691
1561860_at	3,925	3,842	4,080	4,195	4,193	4,332
1561861_a_at	4,811	4,794	4,698	5,415	5,291	5,666
1561863_a_at	4,459	4,724	4,721	4,687	4,834	4,704
1561864_at	4,716	4,367	4,640	4,781	4,930	5,108
1561867_at	3,736	4,487	4,230	3,567	3,634	3,687
1561868_at	7,100	7,108	7,296	7,677	7,472	7,506
1561870_at	3,715	3,738	3,828	3,650	3,593	3,514
1561871_at	8,064	8,150	8,170	8,548	8,604	8,731

1561872_at	5,505	5,537	5,362	5,945	5,677	5,637
1561877_at	4,618	4,519	4,017	4,335	4,287	4,261
1561879_at	4,796	4,764	4,960	4,656	5,002	4,793
1561880_a_at	6,394	6,312	6,428	6,857	6,657	6,908
1561881_at	4,516	4,454	4,599	4,329	4,156	4,293
1561882_at	4,373	4,219	4,155	4,293	4,118	4,265
1561883_at	5,973	6,068	6,101	6,081	6,328	6,279
1561884_at	7,425	6,909	6,680	6,647	7,240	6,781
1561886_a_at	4,338	4,031	4,380	4,173	4,127	4,161
1561887_at	6,096	5,824	6,065	6,249	6,300	6,442
1561889_at	4,958	4,908	4,992	5,242	5,020	5,036
1561890_at	6,090	6,371	6,217	6,380	6,364	6,214
1561891_at	6,028	6,258	6,161	6,327	6,299	6,418
1561892_at	5,253	4,793	4,766	4,839	5,244	5,023
1561893_at	6,085	6,057	5,886	5,620	5,903	5,903
1561894_at	6,364	6,451	6,042	4,748	4,721	4,581
1561895_at	4,233	4,299	4,393	3,877	4,022	3,861
1561896_at	6,236	5,645	5,763	6,008	6,013	6,033
1561897_at	6,791	6,589	6,707	6,687	6,715	6,930
1561899_at	5,291	5,639	5,860	7,772	8,274	8,466
1561902_at	4,141	4,036	3,906	4,173	4,069	4,078
1561905_at	5,202	5,493	4,935	5,915	5,774	5,850
1561906_at	4,539	4,543	4,662	5,002	4,969	5,130
1561908_a_at	7,758	7,845	7,680	7,951	7,868	8,048
1561909_at	5,652	5,854	5,971	5,881	5,975	6,057
1561910_at	5,918	5,676	6,276	6,589	6,614	6,632
1561911_at	4,687	4,668	4,982	5,135	5,086	5,161
1561912_at	4,001	3,867	3,762	4,686	4,375	4,354
1561914_at	4,459	4,078	4,379	4,689	4,497	4,436
1561915_at	6,134	6,169	6,173	6,229	6,024	6,213
1561916_at	6,415	7,030	6,068	7,002	6,827	6,750
1561917_at	7,282	7,256	7,202	7,814	7,434	7,554
1561918_at	6,541	6,234	6,469	7,294	6,859	6,972
1561919_at	6,500	6,135	6,186	7,644	7,045	6,909
1561923_a_at	5,687	5,806	5,812	5,374	5,457	5,571
1561924_at	5,084	5,187	5,142	4,584	4,643	4,648
1561926_at	3,936	3,688	3,802	3,794	3,611	3,526
1561927_at	3,474	3,392	3,532	3,922	3,873	4,063
1561928_s_at	7,115	6,610	7,024	6,874	6,966	7,245
1561937_x_at	8,282	8,315	8,325	8,915	8,944	9,125
1561938_at	3,555	3,830	3,637	3,696	3,876	3,669
1561939_at	4,130	3,962	4,033	3,951	3,971	4,206
1561940_at	7,026	6,554	6,838	6,900	7,167	7,109
1561942_x_at	6,911	6,682	6,775	6,329	7,347	6,789
1561944_at	5,934	5,767	5,697	6,396	6,370	6,598
1561948_at	4,312	4,285	4,237	4,983	5,210	5,206
1561951_at	5,257	4,889	5,204	5,844	5,505	6,318
1561953_at	4,781	5,047	4,702	4,322	4,207	4,385
1561956_at	5,562	5,428	5,919	5,795	5,776	5,259
1561959_x_at	3,340	3,379	3,448	3,443	3,320	3,405
1561960_at	6,618	6,856	6,795	7,082	6,781	6,724
1561961_at	4,813	4,748	4,825	5,079	5,056	5,185
1561962_at	4,486	4,601	4,749	4,584	4,767	5,056
1561963_at	5,460	5,122	5,590	6,145	5,916	6,065
1561964_at	4,993	5,200	5,017	5,080	5,149	5,084
1561965_at	4,952	4,997	4,618	3,979	4,311	4,077

1561966_at	4,743	5,127	4,422	3,958	4,421	4,042
1561967_at	7,145	7,031	6,897	7,187	7,206	7,504
1561969_at	4,274	4,307	4,394	4,833	4,618	4,522
1561973_at	6,434	6,197	6,121	4,947	4,909	5,036
1561976_at	6,912	6,379	6,906	7,757	7,685	7,873
1561978_at	7,325	7,330	7,124	7,934	8,001	8,220
1561979_at	4,500	4,232	4,532	5,484	5,106	5,385
1561983_at	4,712	4,582	4,619	4,500	4,448	4,500
1561984_at	6,279	6,306	6,129	6,288	6,617	6,348
1561985_at	5,529	6,525	5,337	4,092	4,309	3,948
1561988_at	6,263	6,121	6,243	6,529	6,436	6,647
1561989_at	7,888	7,467	8,080	7,818	7,763	7,769
1561990_at	6,303	5,940	6,123	6,111	6,357	6,243
1561995_at	6,599	6,764	6,772	7,024	7,134	6,994
1561997_at	3,835	3,836	3,648	4,198	3,986	3,981
1561998_at	4,749	4,667	5,039	5,585	5,489	5,658
1562000_at	4,468	4,833	4,928	4,781	4,683	4,731
1562002_at	8,539	8,246	8,258	9,791	9,694	9,831
1562004_x_at	7,998	7,992	8,027	9,289	9,171	9,300
1562005_at	6,226	6,290	6,056	6,597	6,591	6,583
1562006_at	5,340	5,442	5,575	6,035	5,891	6,117
1562007_at	6,733	6,897	6,795	7,415	7,021	7,220
1562009_a_at	4,207	4,242	4,285	4,935	5,291	5,185
1562010_x_at	5,691	5,542	5,515	5,908	6,141	6,021
1562011_at	4,619	4,358	4,779	4,783	4,503	4,548
1562012_at	5,235	5,202	5,051	4,984	4,962	5,038
1562013_a_at	8,051	7,959	7,919	6,346	7,141	6,788
1562016_at	7,553	7,389	7,481	8,094	7,511	8,144
1562017_at	5,778	5,995	5,688	6,273	6,074	6,116
1562019_at	4,112	4,121	4,154	4,373	4,251	4,669
1562020_s_at	5,761	4,953	5,631	6,049	5,981	6,109
1562022_s_at	8,922	8,809	8,992	8,700	8,675	9,007
1562023_at	4,486	4,503	4,473	4,286	4,219	4,403
1562026_at	7,636	7,640	7,697	8,047	8,041	8,265
1562028_at	6,264	6,435	6,471	7,479	7,166	7,338
1562029_at	5,558	5,474	5,176	5,653	5,706	5,506
1562030_at	6,431	6,768	6,359	7,571	7,838	7,554
1562031_at	4,824	4,777	4,685	5,083	6,035	5,388
1562032_at	5,716	5,837	5,783	6,432	6,450	6,609
1562033_at	6,652	6,485	6,659	6,572	6,657	6,597
1562034_at	6,595	6,540	6,766	6,856	6,611	6,831
1562035_at	4,019	3,851	3,623	4,763	4,565	4,932
1562036_at	5,440	4,907	5,518	5,224	5,144	5,368
1562038_at	4,603	4,660	4,636	4,636	4,780	4,691
1562044_at	7,197	7,100	7,130	7,997	7,667	7,771
1562045_at	6,312	6,412	6,047	6,775	6,728	6,744
1562046_at	5,038	5,347	5,029	5,067	4,887	5,217
1562048_at	5,516	5,592	5,449	5,937	5,918	5,723
1562049_at	3,870	3,904	3,573	4,465	4,396	4,489
1562051_at	4,605	4,424	4,506	4,865	5,289	5,182
1562052_at	5,930	5,781	6,092	6,205	6,013	6,003
1562053_at	4,718	4,805	4,446	3,946	3,936	3,976
1562054_at	3,684	3,887	3,824	3,946	3,883	3,990
1562055_at	6,898	6,811	6,993	7,048	6,578	6,904
1562056_at	7,939	8,542	8,677	7,645	7,319	7,871
1562057_at	5,374	5,265	5,257	5,595	5,446	5,334

1562058_at	4,615	4,429	4,527	5,170	5,313	5,535
1562059_at	5,466	5,172	5,373	5,418	5,934	5,720
1562062_at	7,757	7,065	7,021	6,128	6,932	6,185
1562063_x_at	9,935	9,564	9,622	8,660	9,889	8,821
1562065_at	5,578	5,336	5,164	6,786	6,239	6,542
1562066_at	5,398	5,245	5,309	5,640	5,360	5,372
1562067_at	5,318	5,495	5,191	5,853	5,993	5,913
1562068_at	6,605	6,607	6,732	7,096	7,096	7,393
1562069_at	5,442	5,635	5,695	5,421	5,311	5,369
1562071_at	4,520	4,528	4,555	5,048	5,260	5,277
1562073_at	5,710	5,675	6,137	6,133	6,023	6,233
1562074_a_at	6,223	5,874	6,252	6,426	6,393	6,436
1562076_at	3,609	3,641	3,804	4,415	3,953	4,258
1562078_at	6,171	6,050	5,816	6,881	6,739	6,915
1562079_at	3,511	3,811	3,805	3,829	3,808	3,673
1562080_at	4,261	4,350	3,630	4,858	4,959	4,940
1562081_a_at	7,798	7,754	7,869	8,121	7,914	8,101
1562082_at	3,770	3,667	3,703	3,828	3,754	3,783
1562083_at	4,003	3,900	4,452	4,258	3,975	4,067
1562084_at	4,910	4,399	4,751	4,874	5,118	4,714
1562086_at	4,701	4,397	4,910	4,548	4,545	4,854
1562089_at	3,984	3,934	4,191	4,150	4,382	4,488
1562091_at	5,667	5,602	5,569	6,379	6,312	6,410
1562093_at	4,590	4,029	4,533	5,614	5,428	5,667
1562094_at	4,416	4,585	4,541	5,410	5,310	4,991
1562095_at	4,089	4,477	4,326	4,903	4,893	4,669
1562098_at	7,499	6,731	7,449	7,543	7,781	7,058
1562099_at	7,552	7,465	7,298	6,543	6,367	6,520
1562100_at	5,761	5,472	5,577	5,638	5,654	5,692
1562102_at	4,426	4,609	5,458	8,905	7,869	7,913
1562103_at	6,131	6,088	6,327	6,110	6,411	6,287
1562106_at	5,845	6,173	5,942	6,832	7,215	7,170
1562107_at	4,531	4,247	4,884	5,323	4,914	4,990
1562110_at	3,907	3,850	3,850	3,878	3,889	3,826
1562111_at	4,704	4,729	4,743	4,717	4,860	4,674
1562112_at	5,385	4,256	5,682	6,653	6,281	6,557
1562116_at	6,718	6,744	6,461	7,120	6,942	7,141
1562121_at	6,041	6,031	5,831	6,285	6,138	6,465
1562122_at	4,306	4,190	4,151	4,149	4,125	4,155
1562130_at	5,065	4,844	5,120	5,633	5,610	5,903
1562133_x_at	5,404	5,542	5,545	5,751	5,419	5,537
1562137_at	6,111	6,011	5,821	6,099	5,929	6,293
1562139_a_at	4,846	4,663	4,869	5,861	5,719	6,004
1562142_at	4,818	5,010	4,942	4,873	5,153	5,030
1562144_at	7,447	7,429	7,418	7,586	7,845	7,727
1562145_at	5,920	6,152	6,250	6,114	6,109	6,169
1562152_at	4,067	4,137	3,894	4,851	4,610	4,525
1562153_a_at	6,623	6,671	6,376	7,372	7,120	7,611
1562157_at	6,026	6,333	5,901	7,181	7,069	6,853
1562158_at	6,214	6,168	5,891	6,308	6,406	6,517
1562161_at	4,906	4,925	4,724	5,144	4,966	5,127
1562162_at	5,026	5,064	5,122	5,816	5,595	5,691
1562163_at	5,446	5,491	5,274	5,452	5,079	5,123
1562165_at	4,401	4,591	4,609	5,429	5,303	5,270
1562166_at	6,609	6,702	6,363	6,836	6,550	6,858
1562167_a_at	4,557	4,836	5,001	5,154	5,140	5,141

1562168_at	4,710	4,440	4,601	4,859	4,612	4,895
1562169_at	7,200	6,378	6,789	4,410	4,384	4,474
1562173_a_at	6,123	5,858	5,755	6,533	6,347	6,532
1562187_at	6,462	6,636	6,634	7,181	6,868	6,875
1562189_at	6,355	6,378	6,346	6,821	6,479	6,754
1562190_at	4,159	4,200	4,068	4,460	4,337	4,249
1562193_at	6,639	6,680	7,136	7,244	6,948	7,191
1562194_at	7,282	6,834	6,979	7,206	9,593	7,049
1562201_x_at	3,976	3,878	3,919	3,718	3,771	3,664
1562208_a_at	5,986	5,139	5,749	5,918	5,629	5,842
1562209_at	4,620	4,540	4,635	4,734	4,894	4,778
1562211_a_at	6,936	7,098	7,270	7,694	7,089	7,515
1562214_at	6,597	6,278	6,567	6,472	6,774	6,771
1562215_at	4,635	4,653	4,686	4,876	4,684	4,798
1562216_at	4,249	4,429	4,313	5,167	4,802	4,806
1562217_at	5,980	5,612	5,539	6,405	5,971	6,187
1562219_at	7,457	7,520	7,315	7,646	7,779	7,822
1562220_at	7,649	7,494	7,573	7,428	7,170	7,161
1562222_at	5,509	5,216	5,658	6,584	6,603	6,814
1562223_at	3,320	3,514	3,528	3,492	3,415	3,544
1562226_at	7,263	6,631	6,902	4,542	4,252	4,331
1562227_at	6,627	6,407	6,154	6,966	6,313	6,449
1562228_s_at	8,403	8,388	8,556	8,845	8,661	8,615
1562229_at	5,338	5,464	5,283	4,451	4,356	4,365
1562230_at	6,990	6,804	7,176	4,878	5,236	4,947
1562231_at	7,047	7,190	7,150	7,221	7,320	7,249
1562232_at	6,976	6,924	7,312	7,321	7,037	7,437
1562234_a_at	6,192	5,824	5,815	7,077	6,730	6,692
1562235_s_at	9,454	8,521	9,392	4,910	4,773	4,892
1562236_at	3,678	3,607	3,774	3,587	3,977	3,620
1562238_at	6,247	5,480	5,425	5,684	5,391	5,143
1562239_at	6,815	6,646	7,023	7,233	7,117	7,133
1562240_at	6,089	6,425	6,400	7,562	7,562	7,817
1562244_at	7,857	8,406	8,574	6,122	5,679	5,864
1562245_a_at	7,735	8,122	8,293	5,639	5,647	6,121
1562247_at	6,049	6,276	6,555	6,628	6,522	6,373
1562249_at	4,551	4,490	4,237	3,914	4,172	4,142
1562250_at	3,818	3,860	3,788	3,600	3,580	3,589
1562251_a_at	5,693	5,228	5,334	5,726	5,371	5,445
1562253_at	4,698	4,883	4,958	5,206	5,560	5,362
1562254_at	7,698	7,382	7,648	8,024	7,563	7,736
1562255_at	4,508	4,066	4,461	4,870	5,561	4,964
1562256_at	6,277	6,277	6,552	6,519	6,417	6,403
1562257_x_at	6,442	6,582	6,476	6,450	6,515	6,494
1562259_at	4,419	4,988	4,750	4,610	4,545	4,479
1562260_at	3,830	3,649	3,942	4,031	4,084	3,929
1562261_at	7,138	6,980	7,311	7,670	7,259	7,279
1562263_at	6,779	6,729	6,993	7,329	6,717	7,104
1562264_at	6,403	6,241	6,390	7,091	7,151	7,232
1562265_at	6,170	6,041	5,977	6,404	6,517	6,430
1562267_s_at	6,290	6,388	6,330	6,411	6,499	6,540
1562269_at	6,327	6,010	6,163	6,371	6,564	6,652
1562270_at	3,890	3,922	3,766	4,027	4,456	3,917
1562271_x_at	7,828	7,804	7,666	7,942	8,378	8,130
1562272_at	6,650	6,512	6,658	7,474	7,113	7,509
1562273_at	6,321	5,981	6,378	6,803	6,590	6,844

1562274_at	6,004	6,245	5,857	6,302	6,080	6,436
1562275_at	8,726	8,522	8,810	6,295	6,346	6,359
1562276_at	3,674	3,822	4,337	4,128	4,208	4,416
1562278_at	4,638	4,531	4,556	4,881	4,564	4,561
1562280_at	8,499	7,839	8,257	5,526	5,714	5,356
1562281_at	4,674	4,907	4,659	4,987	4,730	4,709
1562282_at	6,776	6,515	6,403	6,279	6,303	6,216
1562283_at	4,749	4,601	4,968	4,865	4,799	4,774
1562286_at	4,286	4,253	4,258	4,157	4,046	4,305
1562287_at	3,743	3,525	3,771	3,684	3,690	3,806
1562288_at	3,969	4,420	4,030	4,272	4,253	4,310
1562289_at	8,099	7,891	8,120	6,755	7,114	7,093
1562290_at	3,663	4,030	4,554	4,402	4,127	4,265
1562292_at	4,052	4,141	4,418	6,547	6,651	6,713
1562294_x_at	3,849	3,790	4,154	5,616	5,601	5,915
1562295_at	6,296	5,940	6,221	6,602	6,657	6,675
1562296_at	5,534	5,317	5,280	6,005	6,052	6,147
1562297_at	4,061	4,540	4,807	4,915	4,850	4,794
1562299_at	5,366	5,180	5,167	6,024	5,444	5,371
1562300_at	5,254	5,274	5,236	5,502	5,769	5,480
1562301_at	3,606	3,907	3,578	3,761	3,678	3,660
1562303_at	4,633	4,396	4,850	4,164	4,236	4,252
1562305_x_at	5,113	5,134	5,305	5,078	5,255	4,970
1562306_at	5,277	5,183	5,405	6,382	5,751	5,762
1562307_at	5,573	5,124	5,363	5,132	5,282	5,155
1562308_at	4,969	4,935	5,075	5,756	5,405	5,635
1562309_s_at	7,452	7,606	7,491	4,929	5,026	4,889
1562310_at	3,611	3,519	3,718	3,997	3,849	4,036
1562311_at	4,458	4,298	4,523	4,889	5,037	5,005
1562313_at	4,721	4,891	4,780	5,150	4,869	5,159
1562314_at	7,344	7,312	7,318	7,369	7,039	7,335
1562316_at	6,311	5,725	6,175	6,983	7,060	7,201
1562317_at	5,116	5,028	5,721	5,899	5,826	6,386
1562319_at	6,097	5,779	5,760	7,254	7,098	7,228
1562320_at	5,010	5,180	5,342	6,042	5,841	6,049
1562321_at	4,366	4,420	4,000	4,597	7,015	5,095
1562323_at	7,387	7,119	7,107	7,776	7,771	7,639
1562324_a_at	6,699	6,597	6,654	7,116	6,983	7,455
1562326_at	5,326	5,095	5,357	5,530	5,309	5,360
1562327_at	5,457	5,823	5,508	6,169	5,918	5,904
1562329_at	3,597	3,704	3,714	3,806	3,812	3,819
1562330_s_at	5,339	5,374	5,119	5,895	5,749	6,012
1562332_at	5,963	6,146	6,206	6,573	6,205	6,102
1562336_at	5,076	5,535	5,419	5,401	5,441	5,479
1562337_at	4,636	4,719	4,812	4,878	4,972	4,839
1562338_at	4,895	5,202	5,286	5,422	5,531	5,555
1562341_at	3,858	3,952	3,968	3,859	3,855	3,768
1562342_at	3,440	3,503	3,553	3,495	3,558	3,500
1562346_at	7,118	6,994	6,974	7,206	6,873	7,121
1562348_at	7,677	7,297	7,172	5,299	5,572	5,514
1562349_at	5,435	5,375	5,058	5,415	5,278	5,290
1562351_at	5,586	5,977	5,916	6,333	6,445	6,291
1562352_at	3,908	4,008	4,029	4,336	4,482	4,471
1562353_x_at	4,584	4,711	4,340	4,552	4,751	4,716
1562358_at	4,575	4,407	4,886	5,071	4,599	4,912
1562364_at	4,583	4,348	4,582	5,415	5,007	5,926

1562365_at	4,673	4,677	4,803	4,680	4,524	4,700
1562367_at	5,089	4,920	5,091	5,472	5,328	5,555
1562368_at	6,932	7,107	7,287	7,700	7,800	7,921
1562371_s_at	5,691	5,542	5,894	5,868	5,485	5,761
1562372_at	5,054	5,298	5,551	5,946	5,472	5,700
1562373_at	4,741	4,931	4,622	4,885	4,791	5,232
1562378_s_at	8,251	8,090	8,324	9,604	8,985	9,335
1562380_at	4,951	4,425	5,138	5,383	5,275	5,427
1562381_at	3,880	3,455	4,080	4,398	4,381	4,561
1562383_at	6,253	6,036	6,116	6,506	6,373	6,437
1562384_at	5,532	5,076	5,517	5,833	5,954	5,784
1562386_s_at	5,999	5,440	5,526	5,117	4,933	5,210
1562387_at	3,689	3,870	3,674	4,222	4,242	4,031
1562388_at	5,715	5,896	5,734	6,038	5,860	5,936
1562389_at	3,902	3,985	4,310	3,813	3,879	3,765
1562390_at	5,929	5,786	6,170	6,372	5,961	6,058
1562391_at	6,566	6,046	6,265	4,664	5,399	4,967
1562392_at	6,125	6,520	6,664	7,036	6,606	6,900
1562396_at	3,423	3,396	3,494	3,900	3,694	4,008
1562398_at	4,574	4,506	4,219	5,035	4,802	5,030
1562399_at	5,835	5,765	5,888	5,974	5,994	5,830
1562400_at	3,700	3,746	3,764	3,898	3,810	3,851
1562403_a_at	6,686	6,593	7,078	7,446	7,161	7,163
1562406_at	3,942	3,936	3,834	3,963	3,977	4,060
1562408_at	8,017	7,992	8,038	8,098	7,910	8,123
1562409_s_at	7,702	7,772	7,762	8,905	8,001	8,090
1562411_at	6,651	6,435	6,522	7,277	7,162	7,548
1562412_at	5,804	5,754	5,839	4,597	4,923	4,648
1562413_at	6,160	6,109	6,108	6,386	6,386	6,503
1562414_at	5,191	5,126	5,165	5,470	5,341	5,334
1562415_a_at	6,728	6,882	6,604	7,978	9,115	8,794
1562416_at	6,662	6,530	6,724	6,630	6,900	6,825
1562418_at	3,849	4,300	3,834	4,099	4,058	3,895
1562420_at	4,293	4,324	4,326	4,971	5,004	4,875
1562424_at	4,336	4,204	4,280	4,728	4,487	4,689
1562425_at	6,717	6,674	6,469	6,921	6,431	6,683
1562426_a_at	7,208	7,266	7,247	7,383	7,406	7,431
1562428_at	7,844	7,845	7,886	7,650	8,215	7,727
1562431_x_at	3,498	3,650	3,269	3,881	4,014	4,401
1562432_at	6,507	5,966	6,821	7,403	7,287	7,319
1562433_at	6,476	6,816	6,248	6,713	6,527	6,602
1562434_at	6,998	6,734	6,984	7,057	7,381	7,135
1562436_at	5,109	5,410	5,468	6,259	5,738	6,098
1562439_at	4,665	4,405	4,493	4,434	4,532	4,417
1562440_at	5,109	5,013	5,287	5,334	5,351	5,217
1562442_a_at	9,262	8,393	9,063	6,711	7,507	7,051
1562443_at	4,601	4,394	4,340	4,717	4,647	4,829
1562446_at	7,130	6,436	6,491	7,026	6,539	7,162
1562447_a_at	7,013	6,870	7,195	7,513	7,223	7,513
1562449_s_at	7,244	7,122	7,211	7,317	7,193	7,312
1562453_at	5,124	5,122	5,425	5,661	5,406	5,384
1562454_at	5,803	5,462	5,410	5,352	5,233	5,291
1562455_at	5,698	5,564	5,413	6,124	6,117	6,245
1562456_at	6,900	7,264	7,369	7,542	7,383	7,724
1562457_at	6,513	6,383	6,820	6,392	6,361	6,336
1562458_at	5,230	5,187	5,672	5,332	5,264	5,194

1562459_at	4,424	4,614	4,555	5,549	5,294	5,464
1562460_at	7,560	7,629	7,806	8,451	8,548	8,900
1562462_at	6,732	6,047	6,611	7,198	7,376	7,604
1562463_at	6,966	6,846	6,956	7,011	6,980	7,235
1562464_at	4,497	4,251	4,660	5,925	5,738	5,927
1562467_at	5,014	5,039	4,657	5,105	5,102	5,337
1562468_at	5,673	5,846	5,665	6,181	6,252	6,321
1562469_at	4,631	4,490	4,689	4,861	4,643	4,753
1562470_at	4,029	4,187	4,149	3,891	3,959	3,740
1562472_at	4,585	4,648	4,456	4,844	4,506	4,781
1562473_at	5,898	6,160	6,118	4,474	4,311	4,450
1562474_at	6,348	6,055	6,604	6,563	6,813	6,594
1562475_at	4,401	4,615	4,350	4,570	4,510	4,567
1562476_at	6,610	6,793	6,586	6,754	6,722	6,869
1562477_at	5,507	5,696	5,730	6,173	6,241	6,257
1562478_at	6,631	6,131	6,665	7,489	7,083	7,493
1562480_at	3,656	3,842	3,444	3,735	3,689	3,961
1562481_at	5,046	5,224	4,791	3,707	3,696	3,713
1562482_at	6,520	6,486	6,342	7,729	7,346	7,695
1562484_at	9,389	10,218	9,030	4,052	3,907	4,053
1562487_at	6,823	6,402	6,411	7,444	7,331	7,630
1562488_at	7,077	7,431	7,078	7,437	7,466	7,436
1562490_at	4,977	4,802	4,493	4,779	4,924	4,720
1562491_at	3,682	3,677	3,934	3,805	3,635	3,703
1562492_at	4,895	4,690	4,909	5,257	5,175	5,022
1562495_at	5,948	6,008	6,103	6,662	6,634	6,682
1562496_at	6,586	6,299	6,646	7,101	6,987	6,870
1562497_at	7,723	7,714	7,113	5,211	5,729	5,376
1562499_at	5,529	5,431	5,396	7,282	7,238	7,841
1562501_at	5,230	4,953	4,966	5,534	5,383	5,155
1562502_at	4,212	4,039	4,198	4,137	4,362	4,320
1562505_at	6,397	6,776	6,912	6,141	5,952	6,090
1562507_at	5,174	4,889	5,066	5,323	5,315	5,518
1562509_at	6,587	6,600	6,658	7,270	6,852	6,774
1562510_at	6,549	6,349	6,467	7,346	6,931	7,218
1562511_at	5,383	5,701	6,033	6,102	5,729	5,779
1562512_at	4,643	4,592	4,981	5,026	4,790	4,719
1562514_at	3,732	3,649	3,874	3,873	3,633	3,817
1562516_at	5,486	4,346	4,553	4,225	4,371	4,177
1562520_at	4,985	5,118	5,060	5,227	5,554	5,295
1562523_at	5,471	5,419	5,545	6,264	6,259	6,504
1562524_at	6,584	6,716	6,806	6,905	6,844	6,939
1562527_at	5,801	6,926	5,759	4,914	4,572	4,434
1562528_at	4,460	4,793	4,420	4,292	4,610	4,164
1562529_s_at	7,083	7,426	7,300	6,060	7,734	6,560
1562532_at	4,332	3,881	4,194	5,312	4,701	4,846
1562537_at	6,214	6,178	6,403	6,807	6,653	6,634
1562540_at	7,200	6,656	6,357	7,184	6,973	7,149
1562541_at	6,774	6,476	6,728	7,031	7,153	7,102
1562543_at	4,039	4,286	4,155	4,492	4,228	4,604
1562544_at	5,426	5,524	5,598	5,307	5,606	5,514
1562546_at	4,836	4,815	4,904	4,530	4,443	4,569
1562549_at	7,164	6,843	7,281	7,935	7,846	7,713
1562550_at	6,439	6,336	6,692	6,882	6,791	6,876
1562553_at	3,664	3,778	3,963	3,735	3,579	3,766
1562557_at	7,103	6,817	7,228	7,246	7,044	7,199

1562558_at	3,634	3,619	3,805	3,881	3,754	3,754
1562559_at	7,288	7,332	7,341	7,622	7,621	7,576
1562562_at	5,516	5,731	5,350	5,367	5,290	5,337
1562563_at	7,544	7,696	7,784	7,723	7,536	7,989
1562566_at	3,661	3,670	3,644	4,023	3,985	3,931
1562568_at	4,321	4,182	4,014	4,646	4,608	4,755
1562572_at	5,626	5,363	5,962	5,485	5,220	5,546
1562573_at	3,958	4,149	4,309	4,193	4,241	4,531
1562576_at	5,066	5,361	5,440	6,124	5,762	6,327
1562577_at	8,274	8,091	8,208	8,956	8,475	8,755
1562579_at	3,450	3,580	3,688	3,566	3,670	3,577
1562581_at	6,538	6,238	6,590	6,765	6,345	6,707
1562582_at	5,764	4,974	5,338	6,214	5,979	6,400
1562583_s_at	5,594	5,692	5,866	5,961	5,793	5,925
1562585_at	4,804	4,905	5,333	5,241	4,750	5,081
1562586_at	7,678	7,968	7,943	8,557	8,260	8,355
1562587_at	4,586	4,779	4,663	4,755	4,571	4,548
1562588_at	4,715	4,873	4,964	4,912	4,639	4,738
1562589_at	6,243	6,493	6,693	7,282	7,464	7,491
1562590_at	7,260	7,086	6,904	7,265	7,149	7,275
1562591_a_at	5,365	5,349	5,740	6,397	5,946	6,399
1562595_at	3,888	3,513	4,038	4,093	3,932	4,051
1562596_at	3,352	3,625	3,630	3,563	3,616	3,691
1562597_at	5,508	5,822	5,533	6,001	6,020	6,005
1562598_at	4,511	4,115	4,479	4,568	4,549	4,438
1562600_at	7,188	6,867	6,687	6,693	6,738	6,854
1562601_at	7,683	7,328	7,913	8,568	8,882	8,803
1562603_at	6,045	5,883	5,841	6,051	5,829	6,062
1562604_at	5,339	5,433	5,309	6,427	6,220	6,503
1562606_a_at	7,394	6,630	7,312	7,249	7,348	7,473
1562607_at	4,749	4,540	4,373	5,510	5,277	5,650
1562608_at	4,441	4,255	4,264	4,524	4,871	4,339
1562610_at	6,501	6,398	6,477	6,098	6,437	6,402
1562611_at	3,825	3,967	3,753	4,002	3,891	4,189
1562612_at	5,609	4,889	5,152	4,669	4,523	4,595
1562613_at	3,955	4,276	4,290	4,457	4,463	4,298
1562616_at	3,594	3,994	3,867	3,611	3,787	3,798
1562617_at	6,252	5,806	6,129	6,564	6,513	6,584
1562619_at	4,948	4,983	5,096	5,262	5,052	4,925
1562621_at	4,561	4,401	4,287	5,444	5,376	5,921
1562623_at	4,776	4,634	4,448	4,890	4,849	5,126
1562624_at	7,191	7,096	7,175	7,868	7,949	8,018
1562625_at	4,829	4,748	4,698	5,458	5,518	5,565
1562627_at	7,404	7,421	7,582	7,490	7,390	7,494
1562628_at	3,594	3,625	3,597	3,705	3,783	3,653
1562629_a_at	4,837	4,923	5,330	5,027	5,008	5,157
1562630_at	5,394	5,484	5,624	6,028	6,029	6,186
1562631_at	6,631	6,376	6,668	7,230	6,978	7,375
1562632_at	7,365	6,528	6,914	7,907	7,600	8,116
1562633_at	7,263	8,590	7,399	6,448	6,298	6,620
1562634_at	4,007	3,870	3,726	4,301	4,182	4,213
1562637_at	3,888	3,931	4,103	3,680	3,671	3,800
1562638_at	4,284	4,641	4,476	4,513	4,203	4,238
1562639_at	5,501	5,192	5,451	5,707	5,130	5,480
1562640_at	4,298	4,158	4,315	4,593	4,636	4,760
1562641_at	5,496	5,659	5,698	5,519	5,587	5,650

1562642_at	4,332	4,569	4,720	4,757	4,694	4,891
1562643_at	6,544	6,633	6,303	7,164	7,057	7,264
1562644_at	4,060	3,961	4,043	4,543	4,036	4,400
1562645_at	4,824	4,930	4,724	5,790	5,447	5,796
1562648_at	6,209	5,285	5,546	3,933	4,327	4,221
1562650_at	4,720	4,732	5,063	4,850	4,807	4,959
1562651_at	6,501	6,168	6,288	6,651	6,322	6,436
1562653_at	5,722	5,872	5,657	5,965	5,878	6,105
1562655_at	5,293	5,686	5,664	5,863	6,157	6,224
1562656_at	3,634	3,697	3,767	3,866	3,627	3,722
1562657_a_at	6,277	6,337	6,248	6,929	6,722	6,806
1562659_at	5,905	6,013	5,860	6,400	6,539	6,719
1562661_at	6,358	6,027	6,054	6,545	6,384	6,558
1562664_at	5,995	6,090	6,101	6,399	6,436	6,601
1562665_at	3,839	4,037	4,491	4,483	4,499	4,544
1562669_at	4,597	4,650	4,388	5,312	5,060	5,254
1562670_at	4,371	4,355	4,465	5,618	5,416	5,351
1562671_s_at	4,922	5,200	5,245	5,778	5,211	5,268
1562673_at	5,074	5,517	5,154	4,452	4,467	4,482
1562674_at	7,169	7,351	7,177	7,394	7,352	7,440
1562675_at	7,531	7,343	7,772	8,121	7,857	8,226
1562677_at	3,900	3,874	3,900	3,913	3,898	3,958
1562678_at	5,064	4,854	5,308	5,475	5,119	5,247
1562681_at	5,894	5,605	5,841	6,357	6,126	6,174
1562682_at	5,343	5,372	5,167	5,140	5,440	5,166
1562683_a_at	8,293	8,339	8,290	4,980	5,121	5,079
1562684_at	5,821	6,086	6,211	6,323	6,404	6,380
1562686_at	4,708	4,161	4,139	3,939	3,951	3,971
1562687_x_at	6,208	5,252	5,969	5,184	4,947	5,191
1562688_at	4,916	5,105	4,922	5,542	5,545	5,878
1562689_at	6,832	6,094	6,524	6,868	6,839	7,008
1562690_at	5,732	5,657	5,882	6,216	5,929	5,800
1562691_at	5,893	5,863	6,123	6,435	6,685	6,569
1562693_at	4,679	4,540	4,717	5,336	5,265	5,424
1562694_at	4,891	4,780	5,518	5,742	5,914	5,851
1562695_at	7,277	7,626	7,638	8,329	8,142	8,353
1562696_at	4,608	4,450	4,311	4,778	4,419	4,536
1562697_at	7,565	7,538	7,238	8,014	8,224	8,241
1562698_x_at	7,441	7,314	7,547	7,791	7,984	7,974
1562699_at	5,285	5,463	5,424	4,824	4,820	4,766
1562701_at	6,804	7,033	6,853	7,228	7,119	7,498
1562703_at	5,557	5,586	5,701	6,081	6,114	6,287
1562710_at	4,134	4,232	4,140	4,677	4,709	4,294
1562712_at	5,274	5,292	5,389	6,050	5,868	6,243
1562713_a_at	6,587	6,492	6,798	6,703	6,349	6,605
1562715_at	3,809	3,960	3,793	4,207	4,290	3,981
1562716_at	5,669	5,603	5,362	6,105	5,865	6,043
1562717_at	6,740	6,461	6,621	6,789	6,790	7,327
1562718_at	5,306	5,105	5,372	5,365	5,316	5,152
1562719_at	5,450	5,601	5,652	6,031	5,835	5,738
1562720_at	5,152	5,592	5,264	5,546	5,262	5,307
1562722_at	6,134	6,167	5,849	7,319	7,216	7,508
1562723_at	6,626	6,584	6,729	7,210	7,163	7,369
1562724_at	6,327	6,484	6,686	7,881	7,597	8,192
1562727_at	4,823	4,706	4,861	4,882	4,809	4,989
1562728_at	7,056	6,876	7,231	7,871	7,701	7,786

1562729_at	6,745	6,789	6,897	6,796	6,784	6,925
1562730_a_at	6,049	5,843	6,067	6,464	6,329	6,299
1562731_s_at	6,698	6,471	6,598	7,012	6,672	6,868
1562732_at	4,178	4,534	4,043	4,813	4,543	4,758
1562733_at	6,738	6,400	6,408	7,500	7,003	7,710
1562736_at	4,100	4,069	4,475	3,737	3,818	3,720
1562737_at	4,003	3,856	3,930	4,176	4,007	4,091
1562738_a_at	5,274	5,044	5,172	4,363	4,622	4,575
1562739_at	6,789	6,571	6,618	7,255	7,158	7,299
1562740_at	5,055	4,856	5,112	5,688	5,530	5,958
1562741_at	5,169	5,185	4,850	5,410	5,308	5,151
1562742_at	5,294	5,228	5,410	6,101	5,996	6,172
1562743_at	6,160	6,217	5,614	6,607	6,044	6,373
1562745_at	7,143	6,957	6,765	7,232	7,266	7,617
1562747_at	5,653	5,376	5,518	6,081	6,033	6,066
1562748_at	5,722	5,750	5,777	6,346	6,132	6,102
1562749_at	6,664	6,747	6,469	7,259	7,162	7,079
1562750_at	4,747	4,438	4,409	5,069	5,125	4,933
1562751_at	7,016	6,479	6,761	7,359	6,858	6,896
1562752_at	4,636	4,410	4,844	5,208	5,430	5,223
1562753_at	6,665	6,749	6,823	7,281	7,630	7,917
1562754_at	5,322	5,015	4,922	5,478	5,208	5,372
1562755_at	5,050	5,036	5,208	5,059	4,930	4,909
1562759_at	5,532	5,353	5,444	5,521	5,237	5,552
1562760_at	6,368	6,248	6,578	7,075	6,662	7,107
1562761_at	3,809	4,084	3,769	4,286	4,146	4,157
1562762_at	4,797	4,385	4,478	5,085	4,641	4,540
1562764_at	7,329	7,000	7,368	7,322	7,252	7,300
1562765_at	6,318	6,354	6,486	6,928	6,863	7,089
1562766_at	4,534	4,660	4,441	4,773	4,378	4,664
1562770_at	7,345	6,855	7,403	7,690	7,769	7,851
1562771_at	5,966	5,708	5,874	6,484	6,117	6,280
1562772_a_at	6,717	6,785	7,154	7,212	6,879	7,008
1562775_at	4,641	4,014	3,894	4,154	4,092	4,518
1562776_at	7,930	7,971	8,103	8,241	8,222	8,387
1562777_at	4,966	4,880	5,071	4,672	4,412	4,343
1562778_at	7,238	7,027	6,917	7,810	7,194	7,282
1562780_at	3,727	4,030	4,074	3,848	4,127	4,058
1562783_at	5,945	5,807	6,441	6,840	6,701	6,915
1562785_at	6,670	6,709	6,779	6,861	6,640	6,594
1562786_at	5,086	5,232	5,280	6,176	6,013	6,183
1562787_at	5,136	5,203	5,111	5,763	5,115	5,108
1562788_at	6,632	6,902	6,927	7,697	7,710	7,944
1562789_at	5,420	5,233	5,194	5,844	5,489	6,052
1562790_at	4,543	4,595	4,649	5,024	4,645	4,848
1562791_at	6,162	5,863	6,178	6,216	6,347	6,147
1562792_at	7,000	7,053	7,073	7,229	6,700	7,179
1562793_at	6,026	5,904	5,969	6,464	6,170	6,235
1562794_at	6,252	5,936	5,949	6,149	6,083	6,347
1562797_at	6,169	5,964	6,152	6,470	6,517	6,661
1562799_at	7,025	6,775	7,060	7,767	7,574	7,662
1562800_at	3,626	4,125	3,940	4,482	4,424	4,586
1562801_at	6,257	6,257	6,598	7,147	7,217	7,394
1562802_at	4,583	4,521	4,644	5,275	5,135	5,516
1562803_at	4,220	4,365	4,101	4,714	4,643	4,801
1562805_at	4,970	5,525	5,215	5,766	5,694	5,751

1562810_at	6,608	6,840	7,063	7,431	7,073	7,292
1562811_at	4,208	4,323	4,017	4,418	4,727	4,850
1562812_at	5,529	5,381	5,526	5,940	5,694	5,880
1562815_at	5,865	5,837	5,665	5,872	5,379	5,321
1562817_at	4,875	4,705	4,473	5,217	4,755	4,990
1562818_at	6,951	6,959	6,899	7,774	7,360	7,542
1562820_at	3,720	3,823	3,783	3,751	3,649	3,772
1562821_a_at	5,111	5,055	5,223	5,646	5,397	5,503
1562822_at	5,688	5,649	5,641	6,169	6,054	6,453
1562823_at	4,447	4,557	4,340	4,431	4,500	4,459
1562825_at	5,252	5,367	5,423	5,661	5,403	5,257
1562826_at	5,430	5,954	5,947	5,984	5,906	6,012
1562827_at	4,990	5,323	5,360	5,826	5,821	5,737
1562828_at	8,643	7,863	8,515	4,845	5,055	4,838
1562829_at	5,110	5,015	4,974	5,639	5,412	5,522
1562830_at	5,952	5,709	5,738	5,822	5,925	5,912
1562831_a_at	5,466	5,835	5,936	6,187	6,226	6,227
1562832_at	5,449	5,376	5,580	6,720	6,595	6,735
1562834_at	6,097	6,299	6,402	6,211	6,230	6,228
1562835_at	5,836	5,923	6,002	6,225	6,036	6,346
1562836_at	7,346	7,373	7,598	6,728	7,060	6,612
1562839_at	4,227	4,188	4,057	4,366	4,249	4,100
1562841_at	7,214	7,265	7,455	7,803	7,700	7,753
1562844_at	6,481	6,125	6,211	6,500	6,236	6,619
1562845_at	5,867	6,176	5,958	6,438	6,466	6,401
1562847_at	5,627	5,432	5,423	5,811	5,774	6,064
1562848_at	6,512	6,853	6,768	6,260	6,576	6,665
1562849_at	7,576	7,449	7,720	8,580	8,538	8,625
1562850_at	4,592	4,769	5,000	4,956	4,908	4,949
1562852_at	6,642	6,972	6,898	7,116	7,115	7,348
1562853_x_at	6,037	6,265	6,304	6,536	6,652	6,662
1562854_at	3,681	3,800	3,479	3,821	3,796	3,801
1562856_at	4,288	4,175	4,496	4,669	4,926	5,047
1562857_at	4,487	4,748	4,482	5,017	4,988	4,841
1562860_at	4,146	4,161	3,993	4,544	4,239	4,295
1562861_at	7,211	7,249	7,634	7,533	7,530	7,637
1562862_at	5,898	5,502	5,670	5,224	5,305	5,616
1562863_at	4,955	4,468	4,926	4,722	5,486	4,657
1562864_at	5,098	5,045	4,891	5,699	5,430	5,717
1562865_at	5,544	5,541	5,954	6,319	6,236	6,362
1562866_at	4,344	4,540	4,241	4,443	4,362	4,397
1562869_at	7,342	6,931	7,026	7,328	7,238	7,358
1562870_at	4,461	4,538	4,553	5,095	4,871	4,880
1562871_at	6,335	6,175	6,257	6,918	6,616	6,945
1562873_at	5,632	5,914	5,784	5,889	5,975	5,858
1562876_s_at	3,601	3,711	3,507	3,516	3,707	3,649
1562878_at	5,071	5,314	5,006	5,361	5,429	5,351
1562879_at	4,703	5,117	5,257	5,410	5,547	5,786
1562880_at	4,173	4,145	4,230	4,213	4,061	4,151
1562885_at	6,670	6,737	6,960	7,189	7,202	7,193
1562886_at	3,642	3,641	3,809	3,968	3,987	4,144
1562888_at	6,899	6,576	6,599	6,971	6,904	6,974
1562890_at	7,765	7,500	7,610	7,552	7,583	7,576
1562891_at	4,950	4,907	5,333	5,113	5,270	5,309
1562892_at	6,092	6,201	6,235	6,138	6,386	6,307
1562893_at	4,789	4,926	4,960	4,809	4,661	4,737

1562894_at	5,047	5,367	5,078	5,022	5,197	5,133
1562895_at	5,523	5,301	5,216	5,840	5,639	6,058
1562896_at	7,856	7,570	7,865	8,072	8,306	8,272
1562898_at	4,110	3,994	3,983	4,522	4,438	4,399
1562899_at	6,665	6,514	6,490	6,579	6,588	6,822
1562901_at	5,374	5,555	5,129	5,969	5,886	5,856
1562902_at	4,199	4,209	4,052	4,207	4,138	4,169
1562903_at	5,970	5,524	5,259	5,652	5,270	5,522
1562904_s_at	6,055	5,707	6,246	5,345	5,351	5,270
1562905_at	6,492	6,359	6,465	6,758	6,737	6,949
1562906_at	6,692	6,466	6,458	7,703	7,768	7,916
1562907_at	5,912	6,124	6,299	6,072	6,080	5,902
1562908_at	4,617	4,424	4,816	4,897	4,708	4,828
1562909_at	4,731	4,882	4,592	4,898	4,965	5,206
1562910_at	7,053	6,613	6,628	7,673	7,771	7,754
1562914_a_at	6,350	6,739	6,594	7,320	7,297	7,393
1562915_at	6,889	6,805	6,685	7,184	7,109	7,192
1562916_at	3,454	3,369	3,689	4,026	3,617	3,887
1562918_at	5,064	5,230	5,237	5,253	5,362	5,351
1562919_at	4,670	4,807	4,979	5,053	5,196	5,248
1562920_at	6,103	6,560	6,129	6,097	6,019	6,001
1562921_at	6,766	7,399	7,418	4,563	4,082	4,491
1562922_at	5,364	5,157	5,006	5,867	5,864	5,952
1562923_at	4,732	4,628	4,825	5,143	5,089	5,119
1562924_at	7,082	6,961	6,640	7,341	7,323	7,369
1562925_at	6,345	6,477	6,472	6,674	6,599	6,749
1562926_at	6,597	6,639	6,258	7,058	7,084	7,109
1562927_at	4,164	4,280	4,032	4,479	4,367	4,678
1562928_at	5,837	5,544	5,804	6,133	5,776	5,784
1562930_at	6,927	6,472	6,792	7,783	6,954	7,161
1562932_at	4,543	4,573	4,248	4,372	4,529	4,760
1562933_at	5,818	5,688	6,138	6,441	6,494	6,368
1562934_at	6,054	6,057	6,164	7,037	6,967	6,746
1562935_at	6,401	6,195	6,627	6,457	6,325	6,527
1562937_at	7,394	7,569	7,282	7,571	7,158	7,498
1562938_at	5,198	5,372	5,235	4,431	4,363	4,506
1562939_at	6,777	6,856	6,991	7,672	7,537	7,499
1562940_at	6,063	6,544	6,499	5,723	5,718	5,496
1562941_at	4,642	4,761	5,226	5,142	5,189	5,206
1562942_at	5,839	5,885	5,865	6,697	6,306	6,504
1562943_at	5,333	5,558	5,372	5,550	5,652	5,607
1562944_at	6,500	6,426	6,412	7,117	7,195	7,398
1562945_at	4,979	5,167	4,951	5,548	5,372	5,451
1562946_at	7,662	7,410	7,503	7,276	7,675	7,291
1562947_x_at	6,483	6,558	6,466	6,444	6,836	6,643
1562948_at	6,038	5,905	5,809	4,887	6,171	5,002
1562949_at	3,997	4,326	4,297	4,095	4,096	4,190
1562950_at	5,995	5,498	5,801	6,369	5,995	6,167
1562953_s_at	6,377	6,214	6,288	6,634	6,578	6,480
1562955_at	6,759	6,321	6,442	6,371	6,091	6,139
1562957_at	6,374	6,034	6,178	6,624	6,488	6,731
1562960_at	7,345	7,036	7,044	6,882	6,516	6,889
1562961_at	5,593	5,536	5,485	5,602	5,448	5,616
1562962_at	6,682	6,599	6,506	7,332	7,548	7,253
1562965_at	4,993	4,886	5,026	5,347	5,099	4,987
1562966_at	6,288	6,423	6,249	6,896	6,638	6,928

1562969_at	4,433	4,957	4,853	4,854	4,748	5,005
1562970_at	5,733	5,514	6,057	5,898	6,078	5,973
1562972_at	6,496	6,560	6,434	7,193	7,046	7,546
1562973_at	4,831	4,186	5,260	5,805	5,481	5,584
1562974_at	5,886	5,945	5,921	6,720	6,984	6,939
1562976_at	6,107	6,039	6,082	6,694	6,576	6,598
1562979_at	5,486	6,023	6,360	8,206	8,700	8,823
1562981_at	6,944	7,565	7,266	7,421	7,281	7,455
1562982_at	5,331	5,260	5,296	5,278	5,466	5,475
1562983_at	6,265	5,839	6,113	6,546	6,263	6,299
1562984_at	6,394	6,343	6,378	6,766	6,526	6,683
1562988_at	5,550	5,655	5,688	5,380	5,170	5,502
1562989_at	5,496	5,646	5,617	5,714	5,573	5,628
1562990_at	3,578	3,607	3,731	3,604	3,709	3,590
1562991_at	3,435	3,639	3,529	3,588	3,359	3,452
1562992_at	3,593	3,657	3,719	3,608	3,509	3,751
1562996_at	5,193	4,766	5,020	5,721	5,509	5,860
1562997_a_at	3,813	3,566	3,909	3,418	3,411	3,368
1562998_at	3,448	3,539	3,775	3,445	3,586	3,476
1562999_x_at	3,432	3,835	3,500	3,721	3,589	3,557
1563000_at	3,606	3,583	3,314	3,685	3,913	3,679
1563001_at	3,794	4,140	4,260	4,123	4,010	4,183
1563002_at	4,219	4,194	4,056	4,153	4,205	4,197
1563003_at	6,636	6,794	6,629	7,124	7,022	7,199
1563005_at	7,131	6,585	6,985	7,372	7,586	7,614
1563007_at	5,407	5,441	5,636	6,305	5,935	5,938
1563008_at	5,658	5,475	5,534	5,797	5,563	5,651
1563009_at	6,810	6,505	6,650	7,117	6,799	7,036
1563010_at	4,767	4,941	4,631	4,990	4,729	4,857
1563012_x_at	3,644	3,670	3,849	3,468	3,835	3,501
1563013_at	5,960	5,537	5,963	6,060	5,823	6,271
1563014_at	6,630	6,360	6,550	6,014	6,349	6,260
1563015_at	5,951	5,544	5,513	6,192	6,104	5,956
1563016_at	4,198	3,919	4,059	4,414	4,216	4,739
1563018_at	5,652	5,471	5,610	5,584	5,339	5,606
1563019_at	5,828	5,911	5,976	6,083	5,993	6,097
1563022_at	6,146	6,152	5,699	4,849	4,583	4,771
1563023_at	5,295	5,213	5,620	5,565	5,579	5,363
1563026_at	4,280	4,512	4,732	4,756	4,892	4,960
1563027_at	5,983	5,956	5,712	6,659	6,617	6,812
1563030_at	7,702	7,433	7,661	8,008	7,768	8,093
1563031_at	5,598	6,035	6,058	6,803	6,950	7,232
1563032_at	3,816	4,105	3,885	4,869	4,811	4,670
1563033_x_at	3,828	3,722	3,826	4,611	4,241	4,518
1563034_at	5,676	5,968	6,080	7,189	7,303	7,258
1563035_x_at	5,710	5,520	5,389	6,727	6,300	6,595
1563036_at	5,921	5,992	5,635	6,446	7,015	6,968
1563038_at	5,405	4,826	5,379	6,240	5,881	6,142
1563039_at	4,154	4,658	4,230	4,911	4,652	4,706
1563040_s_at	3,965	3,762	4,197	4,206	4,274	4,069
1563042_at	6,775	7,032	6,831	6,961	6,865	7,030
1563043_at	6,784	7,043	6,979	7,045	6,855	7,048
1563044_at	5,198	4,991	5,112	5,709	5,414	5,623
1563045_at	6,451	6,288	6,441	7,671	7,490	7,711
1563048_at	8,246	7,962	8,296	8,863	8,596	8,838
1563051_at	5,897	6,102	6,304	4,802	5,596	5,089

1563052_at	3,815	3,924	3,981	3,977	4,080	4,071
1563053_at	4,834	5,089	4,476	5,177	5,134	5,466
1563054_at	4,678	4,887	4,600	4,648	4,609	4,742
1563055_at	3,511	3,686	3,913	3,535	3,588	3,589
1563057_at	5,783	5,908	5,676	5,959	6,015	6,133
1563059_at	4,524	4,663	4,588	4,366	4,535	4,545
1563061_at	3,796	3,886	4,037	3,876	3,942	3,921
1563062_at	5,878	6,005	6,350	6,640	6,563	6,735
1563063_at	6,884	6,446	6,799	7,242	6,870	6,873
1563064_at	5,532	5,188	5,712	5,827	5,696	5,738
1563065_at	6,877	6,430	6,748	6,764	6,698	6,734
1563068_at	6,380	6,172	6,279	7,256	7,190	7,087
1563069_at	6,562	6,604	6,834	7,119	6,896	7,045
1563070_at	4,329	4,388	4,622	5,183	5,073	5,175
1563071_at	6,460	6,264	6,534	7,583	7,249	7,397
1563072_at	3,609	3,405	3,441	3,702	3,604	3,671
1563073_at	5,984	5,535	6,150	6,222	5,917	6,081
1563074_at	7,525	7,549	7,361	7,914	7,927	8,079
1563075_s_at	4,464	4,432	4,995	4,381	4,427	4,361
1563076_x_at	6,109	5,692	5,835	5,647	5,545	5,541
1563077_at	5,551	5,553	5,855	6,143	6,458	6,393
1563078_at	6,765	6,948	6,916	7,244	7,342	7,449
1563079_at	7,032	6,801	6,955	7,027	7,186	7,270
1563080_at	4,494	4,233	4,154	4,219	3,986	3,821
1563081_at	5,923	6,033	5,929	6,291	5,949	6,450
1563082_at	5,789	5,540	5,338	6,643	6,305	6,729
1563083_s_at	3,787	3,967	3,667	3,808	3,719	3,794
1563085_at	5,180	4,846	5,286	5,703	5,916	5,886
1563086_at	6,580	6,663	6,573	6,794	6,515	6,768
1563087_at	4,225	4,396	4,789	4,145	4,193	4,022
1563088_a_at	6,048	5,824	5,938	6,415	6,461	6,203
1563089_at	7,128	6,703	6,857	7,178	7,125	7,274
1563090_at	7,260	7,106	7,209	8,188	8,005	8,104
1563091_at	4,627	4,446	4,589	4,800	4,699	4,616
1563092_at	5,224	5,286	5,335	5,433	5,291	5,535
1563097_at	3,773	3,610	3,473	3,750	3,829	3,908
1563098_at	5,441	5,589	5,782	6,940	7,200	6,961
1563099_at	4,642	4,265	4,861	5,153	5,005	5,291
1563101_at	3,864	3,941	3,976	4,099	4,280	4,076
1563102_at	4,909	4,954	5,198	5,301	5,343	5,468
1563103_at	5,331	5,519	5,399	5,496	5,308	5,497
1563104_at	5,690	5,284	5,178	6,207	5,790	5,528
1563105_at	7,690	7,499	7,722	7,994	7,763	7,899
1563106_at	6,448	6,427	6,559	7,327	7,420	7,584
1563107_at	5,102	5,204	5,458	5,969	5,506	5,646
1563108_at	6,318	6,344	6,627	7,650	7,530	7,539
1563109_at	6,194	5,987	5,940	7,046	6,615	6,715
1563110_at	3,990	4,016	3,760	3,987	3,986	3,939
1563111_a_at	8,009	8,560	7,852	8,677	7,382	7,972
1563112_at	5,348	5,113	5,096	5,124	4,985	5,138
1563113_at	5,781	6,149	6,191	6,130	6,154	6,235
1563114_at	6,674	6,315	6,476	7,520	7,872	7,534
1563115_at	5,289	5,695	5,426	5,634	5,535	5,722
1563116_at	4,537	4,197	4,200	4,501	4,403	4,480
1563117_at	6,004	6,108	6,098	6,711	6,825	6,741
1563118_at	5,842	5,775	5,783	5,967	6,033	6,044

1563119_at	5,685	5,177	5,337	6,102	6,446	6,743
1563120_at	6,494	6,474	6,352	6,651	6,703	6,513
1563121_at	5,322	5,760	5,693	5,964	5,779	5,952
1563127_at	5,552	5,474	5,584	6,084	5,526	5,849
1563128_at	3,976	4,002	3,761	4,116	4,117	4,045
1563129_at	4,524	4,765	4,604	4,177	4,391	4,510
1563130_a_at	7,432	6,731	6,909	5,236	6,262	5,494
1563131_at	6,375	6,671	6,449	6,623	6,856	6,710
1563132_at	4,725	4,815	4,834	5,557	5,586	5,730
1563133_at	3,765	3,523	3,682	4,359	4,366	4,536
1563135_at	4,196	4,395	4,154	4,381	4,067	4,361
1563136_at	4,490	3,568	4,169	4,780	4,625	4,674
1563137_at	6,641	6,375	6,792	7,233	6,983	7,022
1563138_at	7,343	6,910	6,991	8,026	7,672	7,878
1563139_at	4,832	5,020	4,888	4,998	5,081	5,088
1563142_at	7,375	6,930	7,018	7,348	7,255	7,261
1563143_at	5,141	5,351	5,395	5,537	5,019	5,326
1563145_at	4,236	4,243	4,401	4,280	4,303	4,289
1563147_at	4,887	4,731	4,396	4,880	5,216	5,157
1563149_at	4,408	4,563	4,535	4,846	4,949	4,721
1563154_at	5,762	5,756	5,949	6,436	6,239	6,485
1563157_at	5,868	6,119	5,863	6,229	6,110	6,197
1563160_at	4,276	4,363	4,510	4,376	4,277	4,240
1563162_at	4,292	4,109	4,205	4,607	4,386	4,553
1563165_at	5,617	5,304	5,447	6,416	6,444	6,880
1563166_at	4,907	5,111	5,083	4,975	4,967	4,866
1563168_at	5,560	5,378	5,953	6,240	6,074	6,014
1563169_at	6,308	6,234	5,988	6,063	5,985	6,048
1563170_at	4,708	4,668	4,789	5,501	5,102	5,185
1563171_at	6,283	6,069	6,337	7,732	7,879	7,938
1563173_at	3,755	3,566	3,600	3,699	3,638	3,747
1563174_at	4,367	4,329	4,693	4,409	4,479	4,547
1563178_at	6,821	6,319	6,514	6,942	6,893	6,829
1563179_at	4,600	4,619	4,517	4,934	4,813	4,999
1563181_a_at	7,463	7,036	7,374	8,199	7,978	8,127
1563182_at	6,049	6,657	5,292	3,992	4,121	3,910
1563185_at	4,836	4,965	5,064	5,249	5,160	5,041
1563186_at	4,048	4,078	4,182	3,930	3,957	3,957
1563187_at	3,841	3,853	3,829	4,079	4,002	4,059
1563188_at	4,555	4,865	5,240	5,239	5,642	5,492
1563189_at	6,238	6,018	6,413	5,685	5,720	5,806
1563190_at	4,517	4,407	4,659	5,292	5,207	5,206
1563203_at	4,440	4,550	4,305	4,465	4,494	4,556
1563204_at	6,399	6,267	6,327	6,856	6,635	6,929
1563207_at	3,956	4,056	3,936	4,315	4,343	4,439
1563208_s_at	5,883	5,453	5,741	6,071	6,068	6,043
1563209_a_at	5,723	6,114	5,518	7,190	6,802	6,718
1563210_at	6,078	5,588	5,900	5,769	6,087	5,700
1563217_at	5,728	5,761	5,511	4,878	5,397	4,610
1563219_at	4,842	4,782	5,061	5,255	5,168	5,248
1563221_at	4,729	4,211	4,352	4,716	4,397	4,710
1563222_at	6,629	6,510	6,574	6,639	6,618	6,811
1563223_a_at	4,631	4,203	4,882	4,584	4,254	4,555
1563224_at	7,477	7,379	7,491	7,664	7,544	7,666
1563225_a_at	7,111	7,318	7,182	7,607	7,628	7,750
1563226_at	5,398	5,320	5,792	7,453	7,571	7,639

1563228_x_at	6,751	6,554	6,448	7,803	7,720	7,843
1563229_at	4,895	5,245	5,201	4,987	5,003	5,149
1563235_at	6,209	6,226	6,381	7,232	7,143	7,184
1563241_at	4,445	4,249	4,682	4,680	4,431	4,658
1563243_at	7,942	7,742	8,221	8,783	8,651	8,891
1563244_at	6,817	6,797	6,567	7,040	6,815	7,201
1563245_at	6,816	6,385	6,238	6,688	6,578	6,672
1563246_at	5,188	5,191	5,474	5,444	5,618	5,453
1563247_at	6,488	6,138	6,379	7,393	7,510	7,989
1563250_at	7,628	7,831	7,933	8,707	8,725	8,993
1563252_at	7,266	7,282	7,406	8,314	8,251	8,051
1563253_s_at	6,801	6,769	6,768	7,242	7,214	7,378
1563254_a_at	3,618	4,075	4,464	4,539	4,411	5,290
1563255_at	7,115	7,214	6,988	8,127	7,603	7,740
1563259_at	5,750	5,607	5,628	5,447	6,243	5,654
1563260_at	5,089	5,349	4,990	6,128	6,298	6,890
1563263_at	5,290	5,504	6,026	6,142	6,342	6,170
1563265_at	4,092	3,829	4,189	4,907	5,048	4,923
1563271_at	5,427	5,356	5,759	5,656	5,282	5,559
1563272_at	6,142	6,422	5,299	6,860	6,607	6,536
1563273_at	8,008	7,905	7,930	7,799	8,189	7,784
1563274_at	6,033	6,338	6,469	6,285	6,207	6,329
1563277_at	4,611	4,659	4,798	4,506	4,637	4,537
1563279_at	4,335	4,850	4,476	4,251	4,374	4,251
1563283_at	5,958	6,259	6,239	6,355	6,456	6,593
1563287_at	7,027	7,047	7,182	7,407	7,288	7,540
1563290_at	6,858	6,336	6,662	8,283	7,644	8,082
1563295_at	6,839	6,784	6,911	7,584	7,574	7,339
1563296_at	5,948	5,887	6,025	6,585	6,530	6,832
1563297_s_at	5,785	5,480	5,746	6,229	6,070	6,220
1563298_at	5,455	5,804	5,537	5,801	5,615	5,807
1563299_at	4,230	4,103	4,311	4,263	4,167	4,184
1563300_at	6,879	6,972	7,062	7,589	7,673	7,431
1563302_at	7,845	7,851	7,748	8,358	8,257	8,457
1563303_at	4,166	4,169	4,246	4,004	3,970	4,040
1563310_a_at	3,783	4,014	3,785	3,905	3,804	3,894
1563312_at	4,203	4,425	4,253	4,949	4,674	4,898
1563315_s_at	8,369	8,076	7,831	8,513	8,145	8,561
1563316_at	6,770	6,660	6,610	6,860	7,062	6,909
1563318_s_at	7,541	7,294	7,279	8,274	8,027	8,365
1563319_at	6,117	5,776	6,235	6,739	6,695	6,718
1563320_at	6,206	5,782	6,288	6,045	5,964	6,091
1563321_s_at	7,156	7,135	6,636	6,158	6,380	6,174
1563324_at	6,268	6,099	6,412	6,849	6,397	6,864
1563325_at	5,402	5,125	5,390	5,697	5,561	5,702
1563327_a_at	6,112	6,292	6,396	6,929	6,888	7,126
1563329_s_at	3,782	3,979	3,675	3,702	3,760	3,654
1563331_at	4,702	4,827	4,886	5,406	5,589	5,638
1563332_at	4,407	4,452	4,174	4,718	4,865	4,900
1563333_at	4,061	4,132	4,194	4,187	4,092	4,118
1563335_at	4,681	4,695	4,543	4,963	4,933	5,022
1563339_at	5,833	5,570	5,854	5,937	5,968	6,115
1563340_at	6,390	6,129	6,217	6,907	6,421	6,965
1563341_at	6,000	5,511	5,822	7,267	7,205	7,303
1563344_at	6,899	6,805	6,989	7,208	7,365	7,567
1563345_at	6,882	6,931	7,126	6,279	6,214	6,556

1563346_at	5,195	4,621	4,880	4,878	4,724	4,815
1563348_at	6,218	6,138	6,362	6,120	5,927	5,866
1563351_at	3,825	3,794	4,250	4,646	4,644	4,979
1563357_at	4,323	4,365	5,450	4,318	4,279	4,381
1563360_at	5,932	5,336	5,775	6,526	6,100	6,061
1563361_at	5,431	5,469	5,616	6,268	6,127	6,397
1563362_at	6,643	6,245	6,177	6,733	7,180	7,351
1563364_at	5,339	5,139	5,794	4,474	4,744	4,541
1563367_at	4,107	3,816	4,538	4,282	4,346	4,481
1563369_at	6,315	6,125	5,935	5,809	5,942	5,970
1563370_at	5,341	5,309	5,041	5,910	6,012	6,175
1563371_at	4,426	4,078	4,358	4,286	4,242	4,426
1563373_at	5,031	4,796	4,919	6,049	6,136	6,308
1563376_at	4,601	4,555	4,582	4,949	4,681	4,875
1563379_at	5,008	4,754	4,778	5,468	5,389	5,505
1563385_at	3,899	3,887	3,805	4,354	4,393	4,270
1563386_at	5,329	4,169	4,613	4,290	5,206	4,073
1563389_at	4,303	4,493	4,487	4,382	4,424	4,407
1563391_at	6,756	6,368	6,490	7,039	6,994	7,011
1563392_at	3,997	4,080	4,078	4,238	4,152	4,190
1563396_x_at	3,526	3,492	3,375	3,326	3,516	3,209
1563397_at	4,963	4,581	4,562	5,039	4,890	5,138
1563398_at	5,464	5,602	5,155	5,601	5,604	5,498
1563404_at	6,951	6,996	6,955	7,770	7,943	8,054
1563405_at	6,993	6,989	6,993	7,275	7,134	7,326
1563407_x_at	6,718	6,769	6,840	7,013	6,973	7,197
1563414_at	5,826	5,298	5,765	4,510	5,532	5,002
1563420_at	5,103	4,835	5,254	5,934	5,881	5,845
1563425_at	7,225	7,155	7,334	7,754	7,382	7,876
1563426_a_at	6,318	6,059	6,177	6,233	6,031	6,208
1563427_at	5,071	5,147	4,731	5,798	5,814	6,009
1563431_x_at	8,537	8,503	8,771	8,528	9,176	9,104
1563445_x_at	7,627	6,619	6,971	8,801	8,023	8,246
1563450_at	4,297	4,469	3,687	5,361	5,254	5,669
1563452_at	4,957	5,357	5,125	5,645	5,703	5,643
1563453_at	6,239	6,355	6,598	7,718	7,153	7,259
1563454_at	3,995	4,078	4,058	4,225	4,144	4,199
1563455_at	7,444	6,981	7,410	6,781	7,896	7,316
1563456_at	3,888	4,076	4,089	3,965	3,909	3,807
1563458_at	6,289	6,237	6,551	6,860	7,370	6,802
1563460_at	4,592	4,456	4,452	4,426	4,401	4,479
1563461_at	3,849	3,769	4,299	3,787	3,858	3,875
1563462_at	3,956	4,046	4,170	4,262	4,360	4,386
1563465_at	3,400	3,466	3,035	3,939	4,026	4,050
1563466_at	5,466	4,759	5,141	5,286	5,005	5,164
1563467_at	5,399	5,773	5,580	5,366	9,121	6,917
1563468_at	5,743	5,907	5,552	5,465	5,487	5,549
1563469_at	6,266	6,201	6,264	6,250	7,318	6,505
1563471_at	6,079	5,750	5,824	6,287	6,386	6,278
1563472_at	7,085	7,010	6,565	7,359	7,333	7,277
1563473_at	6,614	6,769	6,870	6,160	6,012	5,919
1563474_at	4,702	4,701	4,738	4,104	4,027	4,224
1563475_s_at	5,503	5,106	5,356	4,124	4,218	4,302
1563477_at	4,951	4,624	4,913	5,291	4,893	5,311
1563478_at	5,218	5,147	5,221	5,395	5,339	5,673
1563480_at	5,973	5,561	5,960	6,445	6,442	6,649

1563482_at	7,092	6,688	6,996	7,072	7,185	7,271
1563483_at	5,203	5,078	4,598	5,017	5,102	4,859
1563484_at	7,055	6,967	6,558	7,304	7,071	7,346
1563485_at	8,146	7,971	8,099	8,576	8,320	8,777
1563486_at	3,990	4,051	4,000	4,125	4,115	4,257
1563487_at	4,498	4,364	4,710	4,775	4,964	4,890
1563488_at	6,301	5,964	6,127	5,980	6,051	6,244
1563489_at	6,575	6,965	6,680	7,767	7,546	7,724
1563494_at	4,978	4,577	4,768	5,132	5,128	5,128
1563495_at	4,894	4,891	5,370	5,384	4,926	5,462
1563496_at	4,494	4,518	4,803	4,788	4,462	4,640
1563497_at	8,459	8,829	9,045	6,410	6,768	6,563
1563498_s_at	3,765	3,960	3,928	3,896	4,000	4,327
1563502_at	6,397	5,899	5,788	5,961	5,987	6,067
1563503_at	3,710	3,667	3,743	3,656	3,654	3,713
1563505_at	6,545	6,283	6,389	6,089	6,019	6,049
1563507_at	5,910	5,694	5,936	6,474	6,117	6,583
1563509_at	7,247	7,213	7,204	7,880	7,845	7,955
1563511_at	6,449	6,381	6,438	7,413	6,904	6,953
1563512_at	7,414	7,178	7,368	7,568	7,224	7,303
1563513_at	4,708	4,747	4,608	4,895	4,561	4,548
1563519_at	4,297	4,280	4,693	4,997	4,923	5,014
1563521_at	7,427	7,123	7,267	7,143	6,991	7,125
1563522_at	5,144	5,307	5,335	5,919	5,439	5,630
1563523_at	4,779	4,756	4,785	4,620	4,594	4,769
1563524_a_at	4,441	4,421	4,599	4,361	4,159	4,323
1563528_at	4,945	5,202	4,807	5,188	5,521	5,199
1563529_at	6,871	6,830	6,787	7,223	7,173	7,157
1563531_at	5,856	5,678	5,911	6,672	6,340	6,342
1563532_at	5,767	5,842	6,264	6,672	6,563	6,789
1563533_at	5,109	4,890	5,031	6,088	6,096	6,258
1563536_at	3,947	4,140	4,228	4,159	4,184	4,302
1563539_at	5,986	6,182	6,203	5,915	5,890	5,854
1563540_at	5,978	6,221	5,903	6,104	5,803	5,693
1563541_at	4,816	4,493	4,704	4,815	4,687	4,790
1563542_a_at	6,772	7,091	7,140	7,706	7,535	7,762
1563546_at	3,559	3,744	3,768	3,648	3,670	3,791
1563549_a_at	8,132	8,086	8,271	7,955	7,607	7,851
1563553_at	6,082	5,685	6,065	5,810	5,764	6,006
1563557_at	5,443	5,299	5,552	5,528	5,465	5,489
1563559_at	4,772	4,847	4,791	5,216	5,004	5,327
1563560_at	6,617	6,413	6,232	6,754	6,786	6,805
1563561_at	3,712	3,566	3,755	4,660	4,665	4,683
1563563_at	5,628	5,500	5,641	6,529	6,456	6,480
1563565_at	5,919	5,800	5,758	6,478	6,058	5,881
1563568_at	4,889	5,194	5,041	5,735	5,570	5,785
1563569_at	4,739	4,678	4,739	5,310	5,202	5,541
1563571_at	6,442	6,179	6,099	7,083	7,133	7,259
1563572_at	4,060	4,137	4,186	4,314	4,145	4,150
1563573_at	4,808	4,702	4,804	5,171	4,791	4,947
1563577_at	7,874	7,968	8,085	8,091	7,729	7,891
1563581_at	4,797	4,627	4,894	5,050	5,019	5,100
1563584_at	5,121	5,849	5,534	5,372	5,379	5,271
1563586_at	6,087	5,848	5,967	6,664	6,501	6,615
1563587_at	5,872	6,236	6,199	7,028	6,507	6,966
1563589_at	4,292	4,345	4,110	4,452	4,164	4,255

1563590_at	5,752	5,872	6,069	6,614	6,298	6,706
1563591_at	4,952	4,791	5,033	5,497	5,070	5,195
1563595_at	4,965	4,861	4,724	5,193	5,244	5,134
1563596_at	5,964	6,028	6,238	6,449	6,095	6,370
1563597_at	6,277	5,803	6,271	6,210	6,420	6,552
1563601_at	5,506	5,209	5,636	5,237	5,143	5,118
1563606_a_at	5,856	6,323	6,214	6,846	6,483	6,602
1563607_x_at	6,508	6,869	7,037	7,594	7,560	7,666
1563608_a_at	5,956	5,226	5,473	6,432	5,990	6,277
1563610_at	5,273	5,061	5,123	5,694	5,708	5,744
1563611_at	4,531	4,655	4,632	4,958	5,005	4,885
1563612_at	5,120	5,009	5,119	5,312	5,340	5,509
1563614_at	4,958	4,818	4,220	4,105	4,093	4,070
1563620_at	3,804	3,852	3,788	3,733	3,663	3,746
1563621_at	6,449	5,787	5,788	5,571	5,597	5,672
1563629_a_at	6,880	6,794	6,759	6,482	7,133	6,460
1563632_at	6,892	6,829	7,034	7,577	7,430	7,708
1563635_at	6,174	5,966	6,129	6,520	6,598	6,582
1563637_at	3,520	3,814	3,687	3,584	3,536	3,602
1563638_at	6,764	7,514	7,500	5,835	6,017	6,308
1563639_a_at	7,919	7,824	7,972	8,320	8,310	8,673
1563641_a_at	6,312	6,087	6,315	6,638	6,496	6,595
1563643_at	4,449	4,342	4,476	4,595	4,412	4,426
1563646_a_at	8,347	8,543	8,138	8,354	8,295	8,186
1563649_at	3,998	3,816	4,167	4,498	4,473	4,602
1563654_at	7,171	7,385	7,172	7,584	7,145	7,382
1563655_at	7,201	7,134	7,228	7,476	7,114	7,316
1563656_at	4,899	4,984	5,091	5,185	5,329	5,257
1563657_at	8,239	8,137	8,283	8,583	8,589	8,810
1563658_a_at	7,098	7,272	7,076	7,450	7,067	7,372
1563659_at	5,168	5,319	5,211	5,511	5,294	5,419
1563660_at	3,672	3,383	3,675	3,693	3,741	3,931
1563662_at	5,631	5,841	5,827	5,803	5,641	5,742
1563668_at	6,730	6,523	6,711	7,316	7,101	7,338
1563670_at	3,646	3,815	3,933	3,976	4,133	4,089
1563671_at	4,602	4,609	5,314	4,775	4,613	4,783
1563673_a_at	4,636	4,695	4,757	4,971	4,930	4,937
1563674_at	6,436	6,326	6,455	6,361	6,383	6,398
1563677_at	4,227	4,190	4,260	4,754	4,448	5,023
1563679_at	5,753	6,105	5,525	6,068	5,975	5,864
1563680_at	3,998	3,902	4,087	3,903	3,839	3,733
1563681_at	6,138	5,894	5,649	6,240	6,299	6,350
1563685_at	5,672	5,444	5,765	6,564	6,694	6,448
1563687_a_at	6,101	5,905	5,931	6,954	7,035	7,273
1563690_at	4,445	4,070	3,956	4,703	4,588	4,929
1563691_at	5,999	5,968	6,524	6,350	6,353	6,353
1563692_at	6,237	6,472	6,061	6,869	6,733	6,692
1563693_at	5,570	5,395	5,487	6,291	5,630	5,985
1563696_at	3,784	4,028	4,273	3,953	3,831	3,973
1563704_at	5,320	5,177	5,215	5,848	5,742	6,022
1563706_at	7,030	6,837	6,847	6,952	6,990	6,820
1563707_at	7,554	7,415	7,640	8,201	8,162	7,993
1563708_at	6,256	6,543	6,567	6,616	7,015	6,885
1563709_at	7,084	6,839	6,868	7,551	7,297	7,556
1563715_at	5,982	6,009	6,446	6,088	6,092	6,092
1563719_a_at	5,682	5,829	5,590	5,400	5,606	5,365

1563720_at	5,840	5,614	5,632	5,280	5,344	5,318
1563721_at	5,159	4,407	4,138	4,068	4,163	4,125
1563724_at	4,464	4,215	3,876	3,546	3,717	3,633
1563725_at	4,875	5,089	5,134	5,112	5,033	4,868
1563728_at	5,233	5,314	5,343	5,906	5,606	5,668
1563742_at	5,571	5,930	5,920	5,996	6,003	6,101
1563743_at	5,568	5,321	5,411	5,539	5,439	5,584
1563744_a_at	6,745	6,573	6,792	7,929	7,382	7,646
1563745_a_at	6,430	6,801	6,608	8,053	7,172	7,344
1563750_at	5,304	5,429	5,426	5,440	5,429	5,474
1563753_at	7,181	7,240	6,900	7,313	7,196	7,362
1563754_at	4,534	4,185	4,614	4,548	4,548	4,578
1563770_at	6,249	6,071	6,034	5,862	6,140	6,016
1563771_a_at	5,169	5,284	5,326	5,416	5,303	5,466
1563772_a_at	7,146	7,175	7,151	8,150	8,147	8,491
1563776_at	5,867	6,225	6,210	7,404	6,904	7,175
1563781_at	7,587	7,526	7,666	7,965	7,951	7,964
1563783_a_at	7,423	7,331	7,460	8,236	7,542	7,956
1563787_a_at	3,958	4,065	4,008	4,550	4,214	4,499
1563792_at	6,656	6,923	6,888	7,303	7,771	7,925
1563793_at	5,726	5,968	6,193	6,151	6,110	6,049
1563794_s_at	4,368	4,177	4,435	4,560	4,881	5,016
1563796_s_at	9,417	9,051	9,695	9,399	8,676	8,854
1563797_at	4,100	4,197	3,715	4,238	4,515	4,363
1563800_at	8,363	8,470	8,670	9,095	9,180	8,899
1563801_at	4,997	5,331	5,344	5,951	5,637	5,788
1563802_at	6,682	6,431	6,920	7,431	7,303	7,168
1563805_a_at	6,373	6,415	6,697	8,166	8,282	8,338
1563808_at	8,876	8,528	8,696	9,695	9,217	9,518
1563809_a_at	6,167	6,159	6,501	6,764	6,705	7,014
1563814_at	6,125	6,131	6,375	7,517	7,172	7,317
1563816_at	7,209	6,949	6,874	7,632	7,384	7,659
1563821_at	5,792	5,875	5,847	6,623	6,429	6,640
1563822_at	6,004	6,105	5,843	6,312	6,136	6,368
1563825_at	3,627	3,716	3,715	4,044	3,858	4,070
1563827_at	3,801	3,828	3,731	3,922	4,058	3,952
1563828_at	6,400	6,489	6,521	6,546	6,492	6,437
1563830_a_at	6,178	6,053	5,403	6,388	6,033	5,974
1563833_at	3,913	3,670	4,241	4,647	4,810	4,942
1563834_a_at	4,382	4,062	4,261	5,865	6,344	6,131
1563839_at	5,263	5,086	5,319	5,428	5,140	5,301
1563840_at	7,307	7,061	7,433	8,614	8,102	8,487
1563841_at	5,816	5,887	6,121	6,058	6,449	6,236
1563842_at	6,661	6,262	6,289	7,539	7,431	7,609
1563845_at	4,838	4,648	5,452	4,743	4,581	4,541
1563849_at	5,342	5,707	5,551	5,674	5,530	5,770
1563853_at	7,685	7,488	7,542	8,376	8,107	8,251
1563854_s_at	5,894	6,158	6,167	6,367	6,391	6,487
1563856_at	4,601	4,435	4,703	4,983	4,935	5,427
1563858_at	3,586	3,627	3,659	3,852	3,568	3,846
1563860_at	5,839	5,810	5,833	6,643	6,069	6,492
1563862_at	5,860	5,709	6,111	5,452	5,996	5,850
1563863_x_at	5,546	5,372	5,585	5,609	5,551	5,569
1563867_at	6,920	6,812	6,898	7,550	7,544	7,438
1563868_a_at	6,398	6,216	6,331	7,446	7,105	7,284
1563872_at	8,305	7,906	8,222	8,497	8,490	8,886

1563874_at	5,909	6,641	5,580	6,298	5,948	6,068
1563876_at	5,021	5,595	5,221	5,239	5,233	5,017
1563878_a_at	7,539	7,331	7,244	7,990	8,034	7,849
1563881_at	5,903	5,301	5,689	3,504	4,015	3,747
1563882_a_at	4,780	5,145	4,635	4,843	4,849	5,044
1563884_at	4,617	4,807	4,668	5,011	4,965	4,860
1563886_at	6,125	5,486	5,804	6,112	5,906	6,358
1563887_at	4,401	4,308	4,480	4,869	4,812	4,777
1563894_at	6,483	6,268	6,149	6,932	6,777	6,805
1563897_at	5,353	4,892	5,112	5,416	5,386	5,163
1563898_at	5,637	5,979	5,769	6,320	5,972	6,214
1563899_at	5,411	5,520	4,716	3,793	3,769	4,004
1563900_at	4,708	4,629	4,664	4,582	4,587	4,613
1563902_at	7,255	6,917	6,771	8,023	7,516	7,431
1563903_x_at	6,584	6,575	6,902	7,170	7,299	7,293
1563904_at	3,696	3,894	3,606	3,830	4,003	3,931
1563906_at	7,399	7,178	6,724	6,646	6,558	6,389
1563907_at	5,257	5,307	5,395	5,767	5,878	5,863
1563913_at	5,642	5,905	5,713	6,430	6,190	6,264
1563916_at	6,727	6,504	6,818	6,734	6,263	6,927
1563919_a_at	7,239	7,162	7,430	8,109	8,225	8,449
1563920_at	4,955	4,727	4,832	5,013	5,248	5,333
1563927_a_at	3,682	3,842	4,005	3,686	3,771	3,642
1563933_a_at	5,883	5,817	5,005	3,905	3,782	3,769
1563934_at	6,434	6,027	5,946	6,133	6,116	6,150
1563941_at	4,866	5,029	4,981	4,709	4,598	4,627
1563943_at	7,040	6,886	7,322	7,514	7,161	7,332
1563945_at	7,758	7,607	7,809	7,916	8,012	7,857
1563946_at	7,890	7,795	7,993	8,265	8,173	8,489
1563947_a_at	9,203	9,133	9,248	8,362	9,411	8,561
1563949_at	7,621	7,487	6,993	6,972	6,933	6,828
1563950_at	5,243	4,992	4,982	5,673	5,485	5,598
1563958_at	5,690	5,832	5,723	6,023	6,113	6,331
1563961_at	5,507	5,469	5,567	5,819	5,901	6,205
1563963_at	5,508	5,578	5,079	5,117	5,242	5,096
1563969_at	5,175	5,489	5,050	5,322	5,411	5,335
1563970_at	4,913	5,181	5,101	5,343	5,326	5,277
1563975_at	7,983	8,124	7,798	8,165	7,662	7,915
1563978_at	4,677	4,749	5,012	5,014	5,160	5,483
1563983_at	6,004	5,709	5,848	5,995	5,858	6,088
1563985_at	5,126	4,872	5,226	5,521	5,394	5,634
1563993_at	4,864	4,934	4,948	5,590	5,481	5,375
1563997_at	5,941	5,860	5,760	5,920	6,041	6,002
1564000_at	6,202	5,971	5,922	7,019	6,059	6,093
1564002_a_at	4,981	5,097	5,293	5,188	5,306	5,398
1564003_at	4,626	4,397	4,415	4,871	4,544	4,567
1564004_at	6,005	5,913	6,136	6,498	6,180	6,382
1564007_at	3,619	3,671	3,938	4,083	3,901	3,996
1564008_at	6,391	6,173	6,355	6,531	6,617	6,805
1564010_at	5,568	5,703	5,900	5,758	5,873	6,021
1564012_at	6,792	6,743	6,756	7,518	7,135	7,388
1564014_at	5,839	6,016	5,955	6,406	6,403	6,145
1564015_at	4,015	4,140	3,861	3,910	3,629	3,727
1564017_at	6,501	5,819	6,048	6,698	6,284	6,564
1564021_at	7,987	7,610	8,065	8,435	8,024	8,437
1564022_at	4,352	4,418	4,724	4,634	4,577	4,506

1564027_a_at	4,746	4,646	4,966	4,947	4,827	4,998
1564028_s_at	5,763	5,739	5,781	6,273	6,402	6,182
1564029_at	5,377	5,134	5,353	5,799	5,419	5,656
1564031_a_at	8,231	8,072	8,024	8,128	7,879	8,160
1564039_at	7,022	6,900	6,373	6,573	6,324	6,099
1564040_a_at	5,840	6,323	6,214	6,091	6,205	6,088
1564044_at	7,736	7,689	7,868	8,613	7,830	8,434
1564049_at	5,660	5,625	5,375	5,788	5,512	5,695
1564050_at	6,259	6,299	6,242	6,297	6,147	6,203
1564051_at	4,842	5,159	4,992	5,306	5,146	5,510
1564052_at	7,003	6,812	7,066	7,657	7,719	7,652
1564053_a_at	7,896	7,564	7,519	5,801	6,881	6,326
1564056_at	5,662	5,640	5,700	5,837	5,703	5,756
1564060_at	6,596	6,093	6,496	7,056	7,060	7,296
1564063_a_at	8,050	7,694	7,635	9,340	9,508	9,815
1564064_a_at	7,587	7,686	7,390	7,908	8,262	7,710
1564066_at	6,567	6,468	7,284	8,960	9,475	9,449
1564067_x_at	6,958	6,651	7,220	9,384	9,526	9,642
1564069_at	8,824	8,541	8,518	9,258	8,876	8,775
1564070_s_at	6,034	6,063	6,007	6,035	5,995	6,169
1564072_at	5,597	4,928	5,173	5,779	5,484	6,007
1564074_at	6,085	5,934	6,434	7,878	7,846	7,818
1564075_a_at	5,742	5,567	5,514	6,013	5,675	5,999
1564077_at	4,246	4,710	5,121	4,269	4,246	4,263
1564078_at	4,045	4,271	4,131	4,550	4,503	4,815
1564083_at	6,052	6,323	7,571	4,869	4,878	4,922
1564084_at	6,920	6,670	6,727	6,783	6,827	6,714
1564093_at	4,756	4,661	4,672	4,554	5,481	4,747
1564097_at	4,154	4,203	4,394	4,558	4,329	4,812
1564107_at	3,811	3,809	3,949	3,949	3,673	3,881
1564109_at	6,974	7,203	7,316	7,453	7,295	7,356
1564112_at	7,574	7,767	7,737	8,301	8,149	8,132
1564117_at	7,607	7,860	7,642	8,167	7,687	7,912
1564121_at	4,878	4,908	4,897	5,317	5,338	5,688
1564122_at	6,612	6,241	6,176	7,582	7,384	7,700
1564125_at	6,285	5,784	5,896	6,666	6,406	6,619
1564128_at	3,493	3,649	3,623	3,520	3,352	3,521
1564129_a_at	8,351	8,099	8,491	9,338	9,370	9,397
1564130_x_at	4,451	4,142	3,982	4,024	4,051	4,206
1564131_a_at	6,682	6,727	6,733	6,634	6,373	6,344
1564134_at	3,762	3,744	4,025	3,776	3,805	3,967
1564138_at	6,156	6,408	5,936	6,596	6,427	6,659
1564139_at	7,921	7,572	7,880	5,743	5,370	5,652
1564143_at	6,461	6,547	6,781	6,838	6,664	6,641
1564144_at	5,280	5,263	5,155	5,511	5,272	5,492
1564148_at	5,111	5,032	5,362	5,043	4,846	4,995
1564149_at	5,590	5,545	5,695	6,281	6,034	6,104
1564150_a_at	4,871	4,707	4,394	4,998	4,965	4,929
1564151_at	4,890	4,699	4,154	4,477	4,695	4,638
1564152_at	5,840	5,671	5,548	5,944	5,899	5,998
1564154_at	8,152	8,276	8,866	7,649	7,373	7,259
1564155_x_at	8,711	8,872	9,220	8,290	7,932	8,061
1564157_at	7,365	7,067	7,399	8,020	7,769	7,730
1564158_a_at	5,240	5,431	5,742	6,225	6,196	6,073
1564160_at	4,330	4,632	4,154	4,630	4,754	4,881
1564164_at	6,618	6,494	6,822	4,806	7,061	4,812

1564165_at	7,127	6,828	7,282	7,846	7,839	8,306
1564166_s_at	6,070	5,761	6,403	6,203	5,773	6,404
1564168_a_at	5,255	4,904	5,555	6,068	5,566	5,912
1564169_at	3,868	3,847	3,910	4,183	4,098	4,034
1564175_at	5,160	5,368	4,851	4,755	5,060	5,085
1564178_at	6,182	5,892	6,175	6,911	6,538	6,805
1564190_x_at	7,042	7,472	6,608	6,522	6,559	6,657
1564192_at	3,845	3,892	4,023	4,398	4,360	4,333
1564193_at	6,228	5,987	6,162	6,391	6,254	6,418
1564194_a_at	4,568	5,077	5,270	5,319	4,929	5,333
1564198_a_at	4,708	4,815	4,857	6,127	5,314	6,000
1564200_at	5,721	5,867	5,597	6,124	6,164	6,265
1564202_at	5,480	5,168	5,570	5,856	5,783	6,146
1564203_at	5,297	5,360	5,418	5,752	5,775	5,814
1564204_at	4,309	4,014	4,285	4,624	4,647	4,603
1564207_at	9,009	9,015	9,078	9,346	9,338	9,510
1564208_x_at	7,045	6,955	6,994	7,148	7,315	7,341
1564209_at	4,214	4,000	4,225	4,770	4,444	4,673
1564211_at	5,509	5,539	5,802	6,573	6,714	6,963
1564212_at	4,870	4,844	4,936	5,131	4,900	4,979
1564215_at	5,758	5,559	5,640	5,562	5,703	5,591
1564220_a_at	6,883	7,054	6,293	4,482	4,392	4,646
1564224_x_at	7,317	6,922	7,222	8,017	7,874	8,243
1564227_at	4,912	4,748	4,735	4,821	4,715	4,589
1564229_at	6,944	6,786	6,709	7,190	6,753	6,779
1564231_at	6,669	6,034	6,261	6,505	6,370	6,453
1564232_at	6,994	6,778	7,106	7,319	6,786	7,144
1564233_at	6,619	6,796	6,749	7,422	7,588	7,722
1564235_at	4,678	4,664	4,356	4,614	4,749	4,805
1564236_at	4,015	4,401	4,289	4,638	4,507	4,481
1564237_at	6,708	6,801	6,781	7,184	6,995	7,109
1564238_a_at	5,636	5,976	5,954	5,461	5,359	5,452
1564240_at	5,290	5,669	5,830	4,965	4,881	4,978
1564241_at	5,401	5,570	5,456	5,605	5,390	5,608
1564242_at	7,299	7,096	7,179	8,447	8,614	8,727
1564244_a_at	4,100	3,785	4,207	4,371	4,690	4,336
1564248_at	6,976	6,953	6,867	6,447	6,690	6,506
1564250_at	3,404	3,747	4,035	4,011	3,643	3,884
1564251_at	6,668	6,755	7,139	7,888	7,817	7,801
1564253_at	5,080	5,255	5,341	5,649	5,428	5,571
1564254_at	6,928	6,902	7,070	7,892	8,013	8,022
1564257_at	6,536	6,485	6,287	6,549	6,688	6,724
1564259_at	6,745	6,530	6,859	6,749	6,610	6,463
1564262_at	4,214	4,484	4,555	4,600	4,564	4,659
1564263_at	6,805	6,633	6,812	7,299	7,114	6,880
1564264_at	6,997	6,863	6,921	7,196	7,021	7,071
1564272_a_at	3,377	3,570	3,674	3,476	3,579	3,457
1564273_at	4,941	5,096	5,226	5,431	5,199	5,566
1564274_at	6,201	6,534	6,313	6,944	7,488	7,082
1564276_at	4,039	4,126	3,975	4,572	4,908	4,840
1564277_a_at	7,697	7,850	7,957	9,524	9,418	9,546
1564280_x_at	4,065	4,045	4,140	4,421	4,693	4,632
1564281_at	7,969	7,900	8,081	8,839	8,692	8,715
1564282_a_at	5,464	6,008	5,924	5,514	4,974	4,916
1564285_at	4,456	4,644	4,563	4,692	4,724	4,652
1564287_at	5,844	5,890	6,292	7,175	7,321	7,448

1564291_at	4,855	4,673	4,967	5,451	5,181	5,221
1564292_at	5,103	5,044	5,046	4,030	3,905	4,075
1564294_at	3,996	3,911	3,741	4,140	3,845	4,054
1564295_at	6,587	6,547	6,577	7,335	6,933	7,149
1564299_at	4,734	4,658	4,957	5,440	5,208	5,662
1564300_at	5,210	5,664	5,865	6,056	5,681	6,240
1564301_a_at	4,236	3,820	3,826	4,429	4,349	4,197
1564306_at	5,311	5,376	5,518	4,872	5,089	4,923
1564307_a_at	5,725	5,668	5,316	5,401	5,053	5,100
1564308_a_at	6,241	6,270	6,185	6,504	6,232	6,374
1564309_at	4,773	4,353	4,500	4,929	5,049	5,114
1564310_a_at	4,758	4,949	4,739	5,351	5,118	5,426
1564314_at	7,068	6,959	7,044	7,299	7,181	7,301
1564315_at	4,181	4,009	4,165	5,199	4,675	5,011
1564317_at	6,241	6,192	6,039	6,651	6,891	6,771
1564318_at	5,606	4,813	5,655	5,027	5,372	5,083
1564319_at	7,539	7,458	7,612	7,782	7,799	7,741
1564323_at	4,004	4,303	4,230	4,439	4,175	4,358
1564327_at	5,185	5,407	5,440	6,102	5,535	6,014
1564331_at	4,621	5,066	4,720	5,202	5,039	5,131
1564333_a_at	7,606	7,266	7,231	8,298	8,018	8,029
1564334_at	6,916	6,793	6,939	6,750	6,673	6,780
1564336_at	6,542	6,692	6,793	7,160	6,901	6,985
1564337_at	4,973	5,133	5,115	5,043	5,121	5,437
1564338_at	6,337	6,327	6,364	6,987	6,988	7,145
1564339_a_at	7,339	7,558	7,198	7,793	7,488	7,861
1564344_at	6,675	6,425	6,439	8,305	7,697	7,934
1564351_at	4,524	4,300	4,273	4,321	4,329	4,287
1564352_at	6,004	5,944	6,053	6,201	6,037	6,175
1564356_at	4,543	4,641	4,461	4,264	4,356	4,259
1564357_at	3,953	3,985	4,081	4,277	4,104	4,382
1564358_at	5,120	5,228	5,261	5,856	5,910	5,646
1564359_a_at	4,094	3,969	4,285	4,108	4,215	4,295
1564360_a_at	4,467	4,740	4,620	4,540	4,431	4,326
1564362_x_at	8,918	8,556	9,061	9,406	9,425	9,291
1564367_at	6,515	6,628	6,648	6,780	6,601	6,719
1564368_a_at	4,396	4,348	4,593	4,547	4,402	4,603
1564369_at	6,248	6,454	6,387	6,790	6,682	6,939
1564371_a_at	4,273	4,550	4,586	4,318	4,219	4,023
1564372_s_at	4,514	4,606	5,019	4,624	4,490	4,424
1564373_a_at	6,016	5,698	5,923	6,391	6,502	6,577
1564377_at	6,277	6,221	6,659	6,919	6,780	7,007
1564378_a_at	7,088	6,166	6,594	5,986	8,171	6,125
1564379_at	6,036	5,602	6,162	6,081	6,243	6,360
1564381_s_at	6,293	6,670	6,164	5,357	5,781	5,513
1564383_s_at	7,683	7,524	7,746	7,104	7,234	7,312
1564385_at	7,739	7,486	7,614	7,787	7,756	7,725
1564386_at	6,085	6,323	6,484	6,966	6,576	6,834
1564387_at	5,365	5,525	5,255	5,616	5,382	5,590
1564389_at	4,692	4,639	4,884	4,902	4,691	5,228
1564391_at	5,842	5,882	5,949	5,814	5,941	5,749
1564392_at	4,654	4,689	4,734	5,177	4,712	5,061
1564399_a_at	7,270	7,043	7,357	7,294	7,625	7,864
1564400_at	5,504	5,725	5,555	6,094	5,984	6,027
1564401_at	6,189	6,637	6,295	6,651	6,276	6,220
1564402_at	5,529	5,466	6,074	6,415	6,302	6,433

1564403_at	8,144	7,633	7,830	8,299	8,084	8,194
1564405_at	3,987	3,988	4,275	4,260	4,051	4,024
1564407_a_at	7,006	6,463	6,898	7,251	7,050	6,951
1564413_at	8,332	8,966	8,988	8,235	8,083	8,352
1564414_a_at	4,883	4,725	4,724	5,106	4,826	5,161
1564421_at	6,698	6,673	6,484	7,245	6,950	7,105
1564423_a_at	7,056	7,084	6,905	7,520	7,218	7,180
1564424_at	6,368	6,111	6,313	6,138	6,811	6,507
1564426_x_at	6,645	6,863	6,756	7,941	8,141	8,119
1564429_at	8,005	7,759	7,963	8,877	7,927	8,115
1564430_at	6,099	7,012	7,011	6,073	5,968	5,970
1564431_a_at	7,029	7,013	7,124	6,998	6,720	7,004
1564435_a_at	7,372	7,507	7,333	8,058	7,970	8,120
1564438_at	4,744	5,120	4,695	5,436	5,102	5,058
1564439_a_at	7,684	7,213	7,401	8,050	7,884	8,252
1564443_at	6,077	5,616	6,172	6,477	6,417	6,077
1564444_at	6,575	6,502	6,344	6,892	6,907	7,024
1564446_at	6,372	6,112	6,085	6,974	6,769	7,253
1564449_at	6,484	6,628	6,837	7,144	7,074	7,276
1564451_at	5,963	5,841	5,876	6,297	6,268	6,411
1564459_at	6,062	6,017	6,348	7,200	6,657	7,034
1564460_at	6,423	6,817	6,705	7,264	6,880	7,172
1564463_at	5,919	6,418	6,117	6,753	6,799	6,916
1564466_at	4,236	4,627	4,532	4,996	4,636	5,029
1564467_at	7,114	7,348	6,090	5,286	5,208	5,279
1564468_at	3,744	3,722	3,811	3,614	3,731	3,933
1564469_at	4,179	4,159	4,154	4,344	4,455	4,562
1564471_at	5,459	5,248	5,418	6,127	6,135	6,308
1564472_at	4,969	5,195	5,156	6,012	5,455	6,088
1564473_at	4,357	4,295	4,311	4,258	4,427	4,505
1564474_at	5,861	5,300	4,723	4,508	4,919	4,681
1564475_s_at	5,954	6,173	6,272	5,982	6,421	5,970
1564479_a_at	6,488	6,761	6,576	7,458	7,270	7,338
1564482_at	7,509	7,027	6,971	6,790	7,033	6,841
1564485_at	4,450	4,634	4,724	4,719	4,557	4,677
1564490_at	6,972	7,329	7,127	7,719	7,462	7,665
1564491_at	4,504	4,475	4,428	5,054	4,939	4,754
1564494_s_at	6,866	6,566	6,925	7,644	7,304	8,981
1564498_at	7,314	7,280	7,759	8,074	7,999	8,125
1564499_at	4,234	4,600	4,386	4,122	4,292	4,221
1564504_at	5,766	5,832	5,921	5,763	5,703	5,689
1564510_at	5,534	5,382	5,693	6,564	6,142	6,420
1564511_a_at	6,398	6,496	6,579	6,762	6,839	6,974
1564514_at	5,551	5,675	5,777	6,322	6,307	6,345
1564520_s_at	10,380	10,438	11,062	11,131	10,740	11,170
1564521_x_at	9,846	9,912	10,232	10,290	9,805	10,345
1564525_at	6,329	5,843	6,457	6,355	6,578	6,517
1564533_at	4,172	4,110	4,249	4,323	4,401	4,386
1564536_at	5,633	5,486	5,753	6,594	6,262	6,761
1564537_a_at	5,957	5,844	5,996	6,500	6,323	6,682
1564539_at	5,832	5,678	5,875	5,362	5,421	5,558
1564544_x_at	7,146	6,744	7,085	7,285	7,193	7,452
1564545_a_at	6,248	5,854	5,991	6,931	6,892	6,991
1564547_x_at	4,238	4,209	4,626	4,115	4,375	4,069
1564548_at	6,808	6,489	6,556	7,243	7,339	7,524
1564552_at	5,843	5,811	5,679	6,412	6,535	6,436

1564559_at	6,917	6,683	6,836	7,315	7,393	7,668
1564561_at	5,277	5,080	5,398	5,731	5,714	5,797
1564567_at	4,229	4,528	4,443	4,426	4,578	4,679
1564568_at	5,769	6,070	6,351	6,396	6,486	6,386
1564573_at	7,542	7,473	7,549	7,794	7,732	7,981
1564580_at	5,227	5,727	5,043	4,675	4,751	4,835
1564584_at	5,256	5,168	5,524	5,885	5,943	6,165
1564590_a_at	6,017	6,168	5,931	6,705	6,637	6,832
1564591_a_at	6,534	6,502	6,385	7,015	6,863	6,949
1564595_at	4,758	4,952	4,434	5,141	4,984	5,269
1564598_a_at	6,247	6,271	6,446	7,080	6,515	6,825
1564600_a_at	7,909	7,784	7,931	8,418	8,224	8,388
1564601_at	5,373	5,142	5,208	5,729	5,480	5,530
1564603_at	8,013	7,952	7,994	8,687	8,351	8,604
1564610_at	3,899	4,279	3,885	4,022	3,860	3,978
1564620_at	5,705	5,370	5,430	6,475	6,473	6,704
1564621_a_at	6,261	6,198	6,482	6,699	6,651	6,553
1564626_at	5,800	5,771	5,846	6,425	6,436	6,528
1564627_at	4,746	5,021	4,111	5,048	5,135	5,315
1564628_at	4,310	4,323	4,646	4,027	3,893	3,874
1564630_at	6,761	6,565	6,484	7,200	6,895	7,117
1564631_at	3,288	3,446	3,434	3,456	3,311	3,474
1564632_at	4,972	4,975	5,244	5,709	5,446	5,581
1564635_a_at	5,501	5,379	5,445	5,545	5,488	5,376
1564637_a_at	9,508	9,382	9,249	8,897	8,746	8,729
1564639_at	5,000	5,051	5,131	4,895	5,231	5,217
1564640_at	3,627	3,833	3,912	3,604	3,726	3,733
1564642_at	5,149	5,291	5,047	5,730	5,725	5,657
1564651_at	7,023	6,430	7,614	7,605	6,815	7,296
1564653_s_at	5,898	5,120	6,007	6,201	6,001	5,967
1564654_at	4,556	4,735	4,408	4,731	4,749	4,605
1564656_at	4,280	4,174	4,105	3,930	4,324	3,950
1564658_at	6,950	7,054	6,722	8,284	8,218	8,326
1564660_at	7,038	6,968	7,518	7,605	7,512	7,607
1564662_at	3,972	4,604	4,128	3,681	3,374	3,571
1564665_at	6,362	6,529	6,434	6,576	6,366	6,361
1564670_at	5,328	5,521	5,676	5,640	5,133	5,369
1564672_at	3,718	3,996	3,867	3,933	3,944	4,130
1564674_a_at	7,297	7,079	6,976	7,380	7,419	7,579
1564676_a_at	6,616	5,600	6,113	5,460	4,994	5,432
1564679_at	6,951	7,012	6,997	7,317	7,026	7,210
1564683_at	7,152	6,769	6,922	7,634	7,648	7,430
1564684_at	5,439	5,165	5,140	5,040	5,114	5,183
1564685_a_at	4,281	4,325	4,288	4,380	4,330	4,578
1564688_a_at	3,902	4,055	4,311	4,120	4,032	3,995
1564690_at	3,872	3,660	4,088	3,817	3,680	3,654
1564691_at	5,180	5,455	5,175	5,031	4,969	4,920
1564697_a_at	7,396	7,379	7,509	7,478	7,608	7,742
1564699_at	3,975	3,932	4,353	3,823	3,782	3,639
1564701_at	6,559	6,349	6,728	7,147	7,476	7,392
1564705_at	3,607	3,506	3,630	3,465	3,546	3,531
1564706_s_at	6,207	6,454	6,059	5,783	5,847	6,048
1564707_x_at	4,947	5,113	5,075	5,017	5,085	5,079
1564709_at	5,464	5,552	5,365	6,388	6,534	6,790
1564713_a_at	7,552	7,455	7,695	8,597	8,414	8,572
1564718_at	4,337	4,301	4,094	4,709	4,359	4,587

1564721_at	5,078	4,934	5,196	5,593	5,439	5,669
1564729_at	6,100	6,052	5,965	6,285	6,411	6,488
1564733_at	6,391	6,732	6,888	6,086	7,494	6,306
1564736_a_at	5,840	5,295	5,467	6,242	5,870	5,962
1564744_at	5,338	5,265	5,138	5,443	5,274	5,464
1564746_at	7,045	7,447	6,925	7,819	7,358	7,371
1564749_at	6,778	6,159	6,501	6,873	6,828	6,813
1564753_at	6,563	6,205	6,131	6,084	6,274	6,190
1564757_a_at	7,168	7,657	6,799	6,153	5,811	6,399
1564758_at	4,516	4,609	4,270	4,654	4,593	4,871
1564760_at	5,212	5,273	5,270	5,601	5,535	5,593
1564763_at	6,874	6,728	6,462	7,216	6,947	7,097
1564765_at	6,133	5,918	5,724	6,277	5,921	6,115
1564767_at	3,849	3,632	3,868	3,931	4,024	3,843
1564772_at	3,845	4,414	4,041	4,174	4,083	4,046
1564773_x_at	6,587	5,867	6,276	6,355	6,760	6,612
1564776_at	5,685	4,988	5,291	5,045	5,162	5,352
1564777_at	3,892	3,602	3,910	4,074	3,923	4,120
1564778_at	6,310	6,509	6,318	6,751	6,566	6,608
1564783_x_at	5,680	5,452	5,940	6,114	5,961	6,216
1564784_at	6,668	6,417	6,407	6,833	6,512	6,787
1564785_at	6,574	6,447	6,538	7,085	7,062	7,214
1564786_at	6,245	6,216	6,284	6,920	6,522	6,990
1564787_at	7,060	6,951	6,838	7,035	7,003	7,147
1564789_at	5,390	5,072	5,201	6,411	6,147	6,449
1564790_at	4,787	4,404	4,938	5,938	5,843	6,136
1564794_at	5,785	5,493	5,681	5,927	5,943	5,728
1564795_at	4,427	4,704	4,752	4,789	4,885	4,787
1564796_at	5,627	5,292	4,728	6,504	7,933	6,645
1564798_at	6,861	7,019	7,497	6,779	6,879	6,692
1564802_at	5,530	5,320	5,343	5,852	5,944	6,244
1564803_at	6,584	6,714	6,964	7,184	7,012	7,240
1564805_a_at	5,413	5,663	6,059	5,617	5,757	5,625
1564807_at	5,295	5,161	4,922	3,869	3,962	4,215
1564813_at	3,932	4,036	4,013	4,244	4,099	4,322
1564816_at	7,593	7,523	7,509	7,611	7,497	7,555
1564819_at	4,548	4,666	4,394	4,929	4,717	4,694
1564820_at	5,319	5,167	5,455	5,222	5,373	5,202
1564821_at	7,111	6,997	6,665	7,886	7,970	7,957
1564822_at	6,109	6,339	6,342	6,729	6,571	6,295
1564823_at	7,024	7,066	7,183	8,190	7,680	7,807
1564828_at	4,794	4,896	5,276	5,395	5,291	5,429
1564836_at	5,128	5,028	5,581	5,873	5,537	5,620
1564837_at	5,497	5,446	5,741	6,743	6,981	7,234
1564838_a_at	5,471	5,776	5,516	5,729	5,704	6,086
1564840_at	4,444	4,388	4,514	5,136	5,265	5,366
1564841_at	4,133	4,078	4,034	4,267	4,363	4,479
1564851_at	6,058	6,419	6,344	6,519	6,357	6,416
1564854_at	6,793	6,928	6,904	7,524	7,396	7,673
1564855_at	4,568	4,666	4,580	4,674	4,726	4,812
1564856_s_at	4,971	4,705	4,961	5,353	5,130	4,981
1564868_a_at	6,991	7,005	7,035	7,732	7,723	7,744
1564872_at	4,936	5,256	5,153	5,794	6,005	5,939
1564876_s_at	7,664	7,660	7,706	8,830	8,494	8,689
1564878_at	4,698	4,849	4,881	4,941	4,921	4,715
1564881_at	5,928	5,454	5,807	5,963	5,854	6,027

1564882_at	4,377	4,659	4,856	4,899	4,845	4,792
1564883_a_at	6,099	6,230	6,013	7,206	7,240	7,344
1564886_at	7,847	7,651	7,679	7,550	7,657	7,589
1564887_at	8,048	7,704	8,071	8,542	8,666	8,567
1564897_at	6,669	7,169	6,793	7,779	7,822	7,648
1564905_at	5,231	5,346	4,805	6,623	6,643	6,506
1564906_at	6,070	5,813	6,160	5,554	5,290	5,226
1564907_s_at	7,647	7,749	7,496	7,842	7,617	7,835
1564909_at	4,417	4,332	4,157	4,802	4,398	4,583
1564911_at	5,064	4,502	5,116	4,809	4,587	4,953
1564921_at	7,502	7,317	7,430	8,275	8,170	8,290
1564931_at	6,261	5,939	5,990	6,493	6,300	6,477
1564932_at	4,000	4,251	4,014	4,067	3,923	4,037
1564933_at	6,302	6,824	6,426	6,099	5,866	5,861
1564936_at	4,628	4,967	4,562	5,210	5,099	4,715
1564937_at	4,747	5,031	5,091	5,342	5,137	5,099
1564940_at	4,714	4,639	4,790	5,176	5,067	5,365
1564945_at	4,644	4,953	4,494	5,323	5,081	5,303
1564949_at	5,113	5,038	5,138	5,522	5,534	5,650
1564950_at	3,841	3,594	4,110	3,756	3,559	3,703
1564960_at	6,497	6,343	6,327	7,593	7,512	7,255
1564962_at	5,190	5,320	5,154	5,385	5,372	5,646
1564963_x_at	5,472	5,274	5,180	5,812	5,865	6,197
1564964_at	6,756	6,003	6,301	4,606	4,167	4,628
1564965_at	4,081	3,936	3,776	4,227	4,125	4,537
1564970_at	5,174	5,210	5,002	5,381	5,547	5,146
1564972_x_at	5,286	5,275	5,458	5,497	5,779	5,466
1564974_at	6,721	6,536	6,898	7,485	6,980	7,353
1564984_at	6,278	5,932	6,114	6,781	6,893	7,160
1564985_a_at	5,746	5,679	5,304	6,224	5,953	6,077
1564996_at	5,499	5,159	5,524	5,962	5,701	5,880
1565000_a_at	5,649	6,008	5,953	6,017	6,153	6,141
1565002_at	5,421	5,134	5,350	5,767	5,562	5,671
1565016_at	7,087	6,638	6,777	7,450	7,695	7,621
1565026_a_at	3,757	3,638	3,963	3,735	3,496	3,629
1565027_at	7,856	7,774	7,903	7,952	8,074	8,044
1565034_s_at	4,836	4,865	4,852	5,454	5,231	5,270
1565065_at	6,579	6,302	6,524	7,100	7,015	7,189
1565073_at	5,840	5,875	6,049	6,839	6,530	6,771
1565080_at	5,893	5,648	5,870	5,916	5,947	6,207
1565082_x_at	7,144	7,087	6,937	7,301	7,155	7,486
1565105_at	5,894	5,017	4,963	5,584	5,125	5,369
1565107_x_at	5,683	5,271	5,390	6,089	5,485	5,635
1565111_x_at	4,113	4,179	4,178	4,261	4,166	4,177
1565130_at	5,104	4,922	5,383	5,574	5,162	4,944
1565131_x_at	3,998	4,034	4,107	4,499	4,301	4,214
1565132_at	6,272	6,204	6,507	6,359	6,404	6,445
1565149_at	6,353	6,492	6,600	5,739	7,121	5,841
1565150_at	5,144	4,859	4,968	5,264	5,313	5,105
1565152_at	5,201	4,935	5,322	5,677	5,845	5,782
1565162_s_at	10,886	10,725	10,079	10,560	10,440	10,977
1565192_at	3,919	4,047	3,939	4,257	4,132	4,300
1565228_s_at	3,602	4,078	3,739	5,545	5,624	6,076
1565242_at	5,627	5,491	4,977	6,362	6,462	6,595
1565254_s_at	4,636	4,027	4,756	4,898	4,892	5,302
1565265_at	4,724	4,815	4,841	5,749	5,955	5,676

1565269_s_at	7,783	8,097	7,442	7,380	7,368	7,407
1565282_at	6,007	6,049	6,012	6,301	6,068	6,183
1565306_a_at	5,521	5,342	5,500	6,933	6,958	7,196
1565320_at	8,111	7,441	7,108	7,963	7,858	7,964
1565325_at	4,179	4,292	4,040	4,657	4,692	4,628
1565329_at	4,338	4,096	4,881	5,062	4,307	4,235
1565337_at	5,035	4,314	5,067	4,966	5,064	4,998
1565338_x_at	5,618	5,591	5,580	5,907	5,993	5,894
1565339_at	5,322	5,198	5,407	5,651	5,374	5,362
1565346_a_at	4,643	4,627	4,444	4,881	4,862	5,342
1565347_s_at	5,127	5,044	5,165	5,505	5,724	5,706
1565358_at	5,706	5,592	5,779	5,797	5,020	5,461
1565389_s_at	6,195	6,121	6,248	6,942	6,386	6,819
1565406_a_at	6,589	6,842	6,384	6,466	6,481	6,594
1565407_at	6,059	6,206	6,125	6,997	6,594	7,003
1565424_at	4,365	4,641	5,213	5,171	4,823	5,270
1565436_s_at	6,628	6,056	6,216	6,501	6,015	6,082
1565446_at	3,495	3,521	3,601	3,275	3,339	3,380
1565454_at	7,853	8,098	8,217	8,638	8,411	8,590
1565483_at	9,221	9,031	9,224	12,061	11,040	11,141
1565484_x_at	9,021	8,810	9,067	11,177	9,941	10,046
1565495_at	5,625	5,673	5,255	5,569	5,458	5,407
1565525_a_at	4,757	4,414	4,692	4,497	4,518	4,459
1565527_x_at	6,314	6,120	6,032	6,327	6,013	6,088
1565535_x_at	6,428	6,458	6,612	7,827	8,161	8,038
1565537_at	8,060	7,933	7,563	8,177	7,778	7,765
1565544_at	3,823	3,939	4,071	4,031	4,084	4,004
1565546_at	4,460	4,474	4,702	4,729	4,898	4,894
1565549_at	6,784	6,619	6,630	6,334	6,299	6,404
1565554_at	7,074	6,950	7,034	8,165	8,030	8,409
1565556_at	6,718	6,662	6,249	7,671	7,267	7,449
1565557_at	4,412	4,137	4,345	4,408	4,455	4,509
1565558_at	8,143	8,014	8,256	7,974	7,928	7,780
1565559_at	7,095	6,680	7,059	7,597	7,455	7,847
1565562_at	8,388	8,441	8,318	8,892	9,013	9,183
1565563_at	3,330	3,384	3,564	3,420	3,347	3,266
1565565_at	3,507	3,661	3,622	3,764	3,850	3,807
1565566_a_at	4,900	4,822	4,653	4,758	4,889	4,793
1565567_at	6,123	5,691	5,839	6,002	6,131	5,555
1565576_at	7,695	7,596	7,765	8,436	8,036	8,271
1565577_s_at	6,826	6,649	7,167	4,670	4,799	4,639
1565578_at	4,494	4,639	4,408	5,146	4,805	5,438
1565579_at	6,032	5,999	6,395	7,280	6,386	6,950
1565580_s_at	6,335	6,241	6,429	6,633	6,568	6,591
1565581_at	7,072	6,580	6,605	7,285	6,971	7,095
1565582_at	5,696	5,157	5,664	5,617	5,562	5,711
1565583_at	6,840	6,579	6,339	7,458	7,033	7,456
1565585_at	4,840	4,895	4,933	4,946	4,864	4,980
1565587_at	6,780	6,749	6,889	7,049	6,766	6,988
1565588_at	5,432	4,813	5,230	5,701	5,351	5,480
1565595_at	7,363	7,389	8,108	6,918	6,932	6,932
1565596_at	4,414	4,405	4,094	4,403	4,308	4,295
1565597_at	6,042	6,248	6,279	5,952	5,766	6,044
1565598_at	6,534	5,880	6,466	4,980	4,795	5,096
1565599_at	4,716	5,205	4,942	5,294	5,022	5,491
1565601_at	5,280	5,297	5,458	5,276	5,286	5,175

1565602_at	3,886	3,409	3,687	4,178	4,563	4,168
1565603_at	7,849	7,638	7,937	8,390	8,421	8,399
1565604_at	7,133	7,035	7,299	7,476	7,386	7,649
1565608_at	5,349	5,142	5,291	5,788	5,628	5,531
1565610_at	5,902	6,024	6,081	6,325	6,294	6,387
1565611_at	7,992	7,874	8,078	9,316	8,738	8,994
1565612_at	5,979	6,080	6,211	7,086	7,341	6,988
1565613_at	4,875	4,830	4,845	5,273	4,510	4,957
1565614_at	3,799	3,717	3,740	3,757	3,670	3,772
1565616_at	5,302	5,317	5,599	6,054	5,631	5,840
1565617_at	7,913	7,752	7,674	8,224	8,069	8,473
1565618_at	7,030	6,623	6,911	7,330	7,607	7,487
1565620_at	7,951	6,508	7,044	5,548	5,805	5,555
1565621_at	5,416	5,541	5,378	6,259	6,235	6,190
1565627_a_at	8,450	8,333	8,888	8,852	8,712	9,103
1565628_at	4,910	5,124	4,858	5,065	4,961	5,029
1565633_at	3,914	4,038	4,371	4,045	4,325	4,252
1565635_at	5,103	5,099	5,355	5,569	5,493	5,630
1565637_at	4,399	4,652	4,460	5,066	4,827	5,030
1565638_at	3,587	3,539	3,692	4,116	4,596	4,079
1565639_a_at	7,190	7,114	7,128	7,733	8,988	7,714
1565641_at	7,694	7,659	7,739	8,273	8,110	8,522
1565644_at	4,834	5,112	4,801	5,691	5,565	5,674
1565650_at	4,803	5,203	5,026	5,259	5,338	5,218
1565651_at	7,316	7,620	7,819	6,442	6,889	6,955
1565653_at	4,613	4,648	4,418	5,833	5,864	6,208
1565656_x_at	4,531	4,460	4,492	4,596	4,434	4,711
1565657_at	3,354	3,662	3,557	3,450	3,439	3,555
1565659_at	6,971	6,735	6,851	7,609	7,635	7,772
1565660_at	7,519	7,772	7,452	7,213	7,125	6,972
1565661_x_at	7,222	7,110	7,792	6,986	6,806	6,819
1565662_at	7,686	7,491	7,742	9,105	9,014	9,189
1565666_s_at	4,727	4,846	4,456	5,410	5,663	5,806
1565668_at	4,718	4,307	4,839	4,484	4,326	4,278
1565669_at	7,038	6,776	7,097	6,755	6,726	6,865
1565671_a_at	6,550	6,208	6,842	8,448	8,256	8,449
1565672_at	6,754	6,615	6,861	7,250	6,999	7,008
1565673_at	4,560	4,883	4,923	5,055	4,861	4,927
1565674_at	6,541	6,433	6,619	6,637	6,475	6,606
1565677_at	6,447	6,323	6,448	6,510	6,509	7,139
1565680_at	8,152	8,086	8,485	8,363	8,213	8,496
1565681_s_at	8,210	8,034	7,981	8,051	8,355	8,520
1565684_at	5,207	5,172	5,420	5,516	5,650	5,708
1565685_at	3,565	3,593	3,654	4,884	4,341	4,445
1565689_at	8,215	7,936	8,426	5,245	6,548	5,398
1565690_at	6,220	5,963	6,583	4,628	4,789	4,789
1565692_at	6,744	6,358	6,211	6,154	6,026	6,124
1565693_at	4,084	4,053	4,262	4,642	4,454	4,606
1565694_at	7,118	6,981	7,017	6,974	6,966	6,940
1565696_at	6,944	7,252	7,079	7,406	7,546	7,590
1565697_at	6,593	6,723	6,771	8,013	7,607	8,036
1565698_at	3,890	3,963	3,834	4,757	4,284	4,394
1565699_at	4,972	4,372	4,998	5,527	5,495	5,353
1565700_at	5,100	4,902	5,006	5,495	5,478	5,349
1565701_at	6,419	5,954	6,224	5,929	8,039	6,536
1565702_at	3,797	3,693	3,958	3,675	3,790	3,931

1565703_at	6,587	6,334	6,260	5,249	5,617	5,256
1565705_x_at	6,479	6,359	5,847	6,431	6,950	6,611
1565706_at	6,524	6,480	6,568	6,110	6,497	6,051
1565713_at	5,778	5,488	6,110	6,935	6,732	6,499
1565714_at	6,767	6,611	6,921	6,868	6,796	6,913
1565715_at	6,951	7,096	7,253	7,311	7,116	7,381
1565716_at	7,929	7,592	6,894	5,921	6,608	6,495
1565717_s_at	8,160	7,736	7,571	7,711	7,791	8,180
1565722_at	5,470	5,713	5,158	5,808	5,819	6,069
1565723_at	4,553	4,177	4,398	4,706	4,556	4,687
1565728_at	6,185	5,935	5,921	6,764	6,609	6,826
1565729_at	6,996	7,080	7,035	7,965	8,020	8,034
1565730_at	6,978	6,731	6,838	6,795	6,673	6,881
1565731_at	8,017	7,778	7,633	8,600	8,507	8,897
1565732_at	4,671	4,647	4,448	4,935	4,739	4,976
1565733_at	5,389	5,811	5,786	6,513	6,214	6,566
1565734_at	7,412	7,022	7,300	7,778	7,579	7,796
1565735_at	7,084	6,906	7,028	7,867	7,471	7,772
1565737_at	6,694	6,404	6,269	7,004	7,245	7,347
1565738_at	6,329	6,423	6,249	6,440	6,590	6,715
1565740_at	5,725	5,383	5,204	6,123	6,042	6,499
1565741_at	5,119	4,869	4,641	5,927	5,525	5,728
1565742_at	3,607	3,612	3,869	3,607	3,487	3,578
1565743_at	4,659	4,287	4,570	3,492	3,820	3,888
1565746_at	4,289	4,273	4,300	4,983	4,737	4,938
1565748_at	5,347	5,435	5,645	5,454	5,461	5,082
1565751_at	6,861	6,721	6,466	7,642	7,412	7,500
1565752_at	7,725	7,904	7,845	7,996	7,950	7,866
1565754_x_at	7,346	7,205	7,311	7,410	7,425	7,311
1565755_at	3,587	3,567	3,473	3,549	3,559	3,666
1565756_a_at	6,255	5,873	6,288	5,398	5,574	5,418
1565758_at	5,751	5,986	5,603	5,997	6,058	6,008
1565759_at	7,416	6,778	6,815	6,672	6,584	6,906
1565760_at	3,617	3,893	3,575	3,564	3,679	3,587
1565762_at	6,532	6,458	6,719	6,558	7,275	6,640
1565763_at	7,018	6,733	6,743	7,414	7,216	7,261
1565765_x_at	5,958	6,405	6,069	5,695	6,295	6,119
1565768_at	6,491	6,362	6,807	7,202	7,195	7,053
1565769_at	4,333	4,388	4,024	5,021	5,108	5,093
1565771_at	6,898	6,278	6,622	7,378	7,302	7,390
1565772_at	6,217	6,048	6,206	6,110	6,069	6,469
1565774_at	6,219	5,916	6,023	6,878	6,827	6,585
1565775_at	7,013	6,710	7,120	7,693	7,480	7,593
1565776_at	5,097	4,902	5,168	6,088	6,130	6,166
1565777_at	5,850	5,974	5,984	7,091	6,836	7,228
1565778_at	5,921	5,885	5,949	6,134	6,327	6,354
1565780_at	5,398	5,558	5,573	5,776	5,304	5,476
1565783_at	6,894	6,940	6,908	7,210	7,426	7,508
1565784_at	5,634	5,643	5,470	6,142	6,056	6,186
1565786_x_at	7,965	7,413	7,375	7,207	7,523	7,481
1565787_at	4,685	4,633	4,857	5,216	4,890	5,103
1565788_at	3,998	3,996	3,741	4,378	4,560	4,588
1565795_at	5,500	5,493	5,458	5,735	5,817	5,862
1565797_at	5,770	5,834	6,030	5,914	5,557	5,680
1565799_at	3,520	3,441	3,500	3,373	3,392	3,422
1565800_x_at	3,299	3,392	3,451	3,436	3,366	3,665

1565801_at	5,325	5,160	5,341	5,370	5,082	5,297
1565802_at	6,871	6,752	6,597	7,627	7,284	7,447
1565803_at	5,963	5,703	5,721	6,494	6,329	6,346
1565804_at	4,162	4,266	4,001	4,324	4,332	4,537
1565805_at	5,815	6,550	6,400	6,546	5,756	6,157
1565806_at	3,509	3,512	3,394	3,481	3,333	3,454
1565807_at	5,532	5,415	5,300	6,338	6,333	6,468
1565809_x_at	4,812	4,664	4,524	4,969	4,858	5,141
1565810_at	4,781	4,893	4,188	4,214	4,235	4,321
1565811_at	8,778	9,140	9,133	6,682	7,731	6,264
1565812_at	7,585	7,183	7,207	7,802	7,261	7,688
1565814_at	7,143	6,783	6,899	7,189	6,850	6,906
1565816_at	5,060	5,581	5,070	5,572	5,212	5,257
1565817_at	6,682	7,049	7,284	7,716	7,909	9,022
1565818_s_at	6,471	6,698	6,206	6,917	6,927	6,866
1565819_at	3,662	3,794	3,809	3,922	3,957	3,960
1565820_x_at	3,455	3,626	3,727	3,935	3,858	4,006
1565821_at	5,641	5,550	5,476	5,387	5,663	5,565
1565823_at	5,919	6,121	6,310	4,494	5,385	4,717
1565825_at	5,968	6,003	5,844	6,201	6,120	6,207
1565826_at	4,412	4,454	4,311	4,843	4,959	5,112
1565829_at	6,399	6,044	6,217	5,883	5,899	5,700
1565830_at	5,668	4,888	4,525	4,227	4,095	4,206
1565832_at	7,186	6,919	7,268	7,978	7,792	7,609
1565833_at	4,878	4,740	4,886	4,625	4,620	4,522
1565834_a_at	4,187	4,587	4,032	3,589	3,536	3,704
1565836_at	5,795	5,646	5,683	6,011	5,814	5,756
1565837_at	5,690	5,609	5,547	6,020	5,745	5,943
1565838_at	4,521	4,509	4,902	5,380	5,419	5,714
1565839_a_at	5,025	5,148	5,393	5,464	5,692	5,466
1565840_at	3,851	4,004	4,322	4,214	3,909	4,103
1565843_s_at	7,036	7,081	7,124	7,129	7,087	7,261
1565844_at	6,107	6,071	5,444	6,322	6,429	6,281
1565845_at	7,376	7,286	7,605	8,199	7,970	8,129
1565846_at	5,955	5,842	6,082	6,513	6,401	6,593
1565848_at	6,543	6,136	6,630	7,385	7,422	7,546
1565849_a_at	6,355	6,263	6,085	6,709	6,505	6,918
1565851_at	3,830	4,062	3,894	4,093	4,221	4,091
1565852_at	6,857	6,905	6,726	7,327	7,171	7,198
1565856_at	6,466	6,295	6,225	6,470	6,544	6,462
1565857_at	6,480	6,618	6,419	6,750	6,625	6,615
1565858_at	6,645	6,659	6,774	7,392	6,945	7,127
1565859_at	6,460	6,504	6,863	6,876	6,738	6,708
1565860_at	4,001	3,834	4,028	4,138	4,103	3,983
1565861_at	3,789	3,779	4,219	3,960	3,936	3,891
1565862_a_at	4,953	4,979	5,312	6,078	5,775	5,973
1565863_at	4,798	4,770	5,039	4,360	5,540	4,443
1565866_a_at	6,070	6,302	5,906	6,411	6,552	6,735
1565867_a_at	6,552	6,681	6,375	6,150	6,447	6,534
1565868_at	5,452	5,149	5,418	5,796	6,223	5,891
1565873_at	5,382	5,437	5,577	5,173	5,274	5,202
1565874_at	3,848	3,886	4,526	4,999	4,861	4,872
1565875_at	4,376	3,873	4,098	4,067	4,186	3,871
1565876_x_at	4,389	4,203	4,476	3,781	3,992	3,763
1565877_at	3,645	3,950	3,995	3,598	3,833	3,753
1565879_at	6,594	6,557	6,977	6,920	6,932	7,134

1565880_at	6,655	6,276	6,574	7,920	7,901	8,023
1565881_at	4,913	4,373	4,472	4,870	5,039	4,964
1565882_at	6,845	6,735	6,812	7,076	7,235	7,212
1565886_at	7,676	7,152	7,863	5,598	7,084	5,730
1565887_at	4,498	4,881	4,862	4,419	4,719	4,472
1565888_at	4,064	4,078	4,008	4,868	4,503	4,886
1565889_at	5,627	5,340	5,257	4,712	4,468	4,456
1565890_at	5,345	4,662	4,696	4,957	4,971	4,898
1565892_at	6,200	5,985	6,091	6,694	6,500	6,544
1565894_at	7,115	7,081	7,300	8,179	8,024	7,978
1565895_at	4,681	4,757	4,651	4,936	4,959	4,806
1565896_at	5,182	5,388	5,213	5,867	5,365	5,443
1565897_at	6,174	6,059	5,930	6,875	6,844	7,026
1565898_at	4,877	5,045	5,065	4,993	5,010	4,880
1565900_at	5,086	4,922	5,166	5,348	5,244	5,148
1565903_at	6,815	7,010	6,714	8,181	8,538	8,383
1565905_at	6,719	6,627	6,730	7,240	7,212	7,526
1565906_at	6,057	6,179	6,296	6,960	6,746	6,942
1565908_at	7,130	7,126	6,910	7,587	7,626	7,418
1565909_at	6,049	6,079	6,377	7,182	6,896	7,346
1565910_at	4,280	4,563	4,615	4,423	4,313	4,390
1565911_at	6,021	6,028	6,140	6,261	6,340	6,481
1565912_at	5,620	5,885	5,459	5,810	5,873	5,925
1565913_at	5,286	5,288	5,820	5,286	5,260	5,466
1565915_at	4,570	4,411	4,726	4,652	5,161	4,775
1565917_at	4,178	4,671	4,881	5,798	5,157	6,029
1565918_a_at	6,090	6,071	6,017	6,750	6,682	6,915
1565920_at	3,924	3,923	4,064	4,613	4,324	4,262
1565921_a_at	5,572	5,692	5,312	6,267	5,996	6,317
1565923_at	3,854	4,202	3,915	4,266	4,140	4,111
1565924_a_at	4,707	4,982	4,789	4,897	5,045	4,926
1565925_at	4,178	4,626	4,366	4,507	4,241	4,744
1565927_s_at	5,318	5,167	5,080	5,350	5,363	5,581
1565928_at	5,909	6,048	6,143	6,002	5,796	6,082
1565929_s_at	6,480	6,534	6,644	7,147	6,704	6,640
1565934_at	4,464	4,217	4,506	4,530	4,455	4,609
1565935_at	7,662	7,292	7,322	7,816	8,056	7,944
1565936_a_at	5,213	5,007	4,887	4,363	4,386	4,602
1565937_a_at	4,351	4,078	4,326	4,239	4,258	4,265
1565939_at	5,173	5,220	5,688	5,348	5,211	5,183
1565947_a_at	5,383	5,480	5,324	5,496	5,304	5,359
1565949_x_at	4,821	5,044	5,212	4,836	5,078	4,936
1565951_s_at	8,243	8,226	7,836	5,532	7,153	4,974
1565974_at	7,079	6,868	7,033	7,555	7,281	7,682
1565975_at	6,637	6,533	6,246	5,778	5,769	5,890
1565976_at	4,906	4,621	4,868	4,844	5,274	4,858
1565978_a_at	4,590	4,436	4,420	4,764	4,510	4,814
1565998_at	5,262	4,999	5,424	5,229	5,158	5,408
1565999_at	4,325	3,943	3,940	4,587	4,636	4,508
1566001_at	8,025	7,917	7,775	7,491	7,577	7,334
1566002_at	6,900	6,697	7,007	8,162	8,202	8,243
1566003_x_at	7,064	6,863	6,802	8,462	8,751	8,934
1566029_at	6,707	6,840	6,813	7,628	7,680	7,734
1566030_at	4,282	4,450	4,685	4,556	4,658	4,588
1566032_at	5,103	5,368	5,390	5,497	5,318	5,422
1566033_at	5,833	5,960	5,967	5,778	5,953	5,937

1566037_at	6,006	6,027	5,702	6,304	6,459	6,446
1566038_at	6,594	6,793	6,466	7,136	7,453	7,577
1566039_a_at	5,152	5,080	5,058	5,466	5,213	5,358
1566040_at	4,696	4,580	4,547	4,839	4,767	4,781
1566041_at	6,828	6,778	6,630	7,129	7,006	7,008
1566042_at	4,684	4,625	4,524	4,794	4,558	4,681
1566043_at	5,414	4,748	5,620	6,022	5,910	5,864
1566044_at	6,377	6,168	6,036	6,646	6,691	6,521
1566046_at	5,340	5,288	5,239	5,375	5,190	5,554
1566047_at	3,992	4,153	4,051	4,673	4,751	4,794
1566079_at	8,491	8,211	8,436	6,828	7,624	7,043
1566080_at	4,666	5,080	4,591	4,949	4,767	4,980
1566081_at	5,182	4,869	4,960	5,398	5,292	5,598
1566082_at	5,259	5,212	5,268	5,276	5,129	5,304
1566084_at	8,652	8,744	8,546	6,791	5,970	6,106
1566086_at	5,455	5,895	5,524	7,149	6,457	6,942
1566087_at	4,016	3,822	3,955	4,082	3,956	4,201
1566093_at	6,307	6,185	6,303	6,349	6,389	6,404
1566094_at	4,841	4,644	4,546	5,158	5,095	5,355
1566096_x_at	4,554	4,415	4,336	4,720	4,855	4,576
1566097_at	6,810	6,233	6,385	7,128	6,831	7,037
1566098_at	6,582	6,747	6,775	7,258	7,343	7,420
1566099_at	6,494	6,991	6,949	7,538	7,472	7,383
1566101_at	5,035	4,851	4,714	6,209	6,137	6,451
1566102_at	5,681	5,588	5,718	5,500	5,649	5,762
1566105_at	6,830	6,841	6,734	7,015	7,005	7,217
1566106_at	5,524	5,592	5,498	6,444	6,662	6,993
1566108_at	5,444	5,459	5,294	5,079	5,189	5,283
1566109_at	3,615	4,016	3,722	3,686	3,673	3,576
1566110_at	7,982	7,623	7,881	8,447	8,093	8,335
1566111_at	7,273	6,888	6,948	8,017	7,690	7,843
1566113_at	5,825	5,822	5,875	5,703	5,975	5,867
1566114_at	5,789	5,679	5,572	6,120	6,125	6,144
1566115_at	4,727	4,813	4,586	4,363	4,374	4,344
1566123_at	5,474	5,716	5,592	5,890	5,951	5,765
1566124_a_at	4,591	4,815	4,741	4,611	4,544	4,612
1566126_at	5,130	4,953	4,937	5,364	5,310	5,284
1566127_at	5,097	5,125	5,058	6,230	6,239	6,407
1566129_at	5,135	4,827	5,038	5,550	6,477	5,865
1566134_at	7,498	7,247	7,554	7,709	7,491	7,764
1566135_at	6,878	6,619	6,851	6,986	7,097	7,215
1566136_at	6,717	6,651	6,688	7,584	7,408	7,638
1566137_s_at	6,382	6,170	6,444	7,097	6,525	6,813
1566138_at	6,665	6,367	6,394	7,145	7,217	7,498
1566139_at	5,894	5,851	6,426	6,714	6,353	6,366
1566140_at	5,368	5,733	5,630	6,006	5,594	5,738
1566141_at	5,999	5,595	6,103	6,071	5,692	5,619
1566142_at	5,543	5,096	5,505	5,471	5,722	5,713
1566144_at	7,359	7,295	7,350	7,894	8,138	8,205
1566145_s_at	6,508	6,168	6,643	6,517	6,757	6,644
1566146_x_at	6,621	6,671	6,269	6,395	6,617	6,288
1566147_a_at	7,375	6,974	7,623	8,041	7,949	8,091
1566149_at	7,333	6,672	6,954	7,848	7,473	7,646
1566150_at	4,913	5,796	4,932	4,679	4,701	4,607
1566151_at	6,562	6,672	6,553	6,985	7,040	6,795
1566152_a_at	8,334	7,619	7,477	5,911	5,982	5,692

1566156_at	7,923	7,742	8,014	8,334	8,154	8,290
1566157_x_at	8,012	7,859	8,018	8,654	8,377	8,603
1566158_at	5,305	5,376	5,366	5,518	5,404	5,431
1566161_at	3,835	3,699	3,775	4,006	3,946	4,108
1566162_x_at	3,645	3,504	3,902	3,618	3,650	3,711
1566163_at	4,292	4,078	4,478	4,592	4,872	4,704
1566165_at	6,536	6,532	6,652	7,303	6,826	7,220
1566166_at	7,098	7,278	7,491	5,859	8,293	6,512
1566168_at	7,297	7,064	7,290	7,946	7,589	7,971
1566169_at	4,075	3,900	4,055	4,468	4,338	4,159
1566171_at	7,846	7,594	7,969	8,624	8,324	8,533
1566176_at	4,958	4,835	5,384	5,255	5,237	5,350
1566177_at	8,033	7,896	8,087	8,664	8,944	9,025
1566178_x_at	8,033	7,840	8,134	8,898	8,918	9,074
1566181_at	6,194	5,852	6,076	6,198	6,135	6,142
1566182_at	6,268	6,241	6,425	7,190	6,967	6,880
1566183_at	6,316	6,202	6,156	6,236	6,124	6,234
1566185_at	7,023	7,443	7,220	7,354	7,379	7,513
1566186_at	6,243	6,247	6,383	6,723	6,565	6,620
1566190_at	5,077	5,238	5,160	5,205	5,193	5,348
1566191_at	5,811	4,986	5,738	4,276	4,620	4,356
1566194_at	5,136	4,829	5,148	5,190	5,003	5,295
1566195_at	7,114	7,079	7,382	7,772	7,724	8,062
1566201_at	4,315	4,543	4,424	4,556	4,347	4,378
1566202_at	3,539	3,788	3,701	3,957	3,686	3,712
1566203_at	4,717	4,465	4,526	5,228	4,759	4,795
1566204_at	5,228	5,137	5,287	5,510	5,438	5,394
1566207_at	8,279	8,128	8,309	7,805	7,997	7,940
1566208_at	6,598	6,993	6,398	6,673	6,558	6,617
1566209_at	4,460	4,956	4,691	5,381	5,382	5,477
1566210_at	6,109	6,119	5,861	6,075	6,052	6,279
1566215_at	5,547	5,743	5,648	6,885	6,539	6,888
1566217_at	5,930	5,743	5,407	6,500	6,077	6,303
1566218_at	7,022	7,103	7,038	7,017	7,076	6,986
1566219_at	4,959	4,787	4,919	4,998	4,831	5,031
1566221_at	6,294	6,372	6,630	6,690	6,728	6,668
1566222_at	5,599	5,376	5,528	6,320	6,375	6,670
1566228_at	6,692	6,711	6,630	6,946	6,585	6,578
1566229_a_at	6,152	5,920	6,007	6,589	6,524	6,721
1566231_at	7,101	7,361	7,188	7,774	7,667	7,896
1566232_at	5,827	6,035	5,694	6,196	6,309	6,167
1566235_at	6,558	6,682	6,991	7,323	7,420	7,364
1566236_at	6,357	6,237	6,115	6,809	6,634	6,842
1566242_at	6,651	6,339	6,815	7,183	6,681	6,835
1566243_at	5,837	4,919	5,363	4,965	5,138	4,872
1566248_at	6,218	6,089	6,088	6,772	6,496	6,740
1566249_at	7,475	7,518	7,518	7,488	7,507	7,495
1566251_at	3,520	3,575	3,515	3,687	3,549	3,647
1566253_at	4,045	4,139	4,067	4,092	4,094	4,139
1566256_s_at	4,249	4,265	4,308	4,496	4,437	4,459
1566257_at	7,845	7,585	7,798	6,602	6,534	5,978
1566265_at	3,899	4,037	4,039	3,807	3,903	3,805
1566266_at	3,841	3,998	3,727	4,096	4,026	4,172
1566267_at	3,665	4,336	3,805	5,060	5,062	5,314
1566268_at	6,425	6,090	6,400	6,814	6,474	6,541
1566269_at	4,788	5,123	5,091	4,986	5,058	5,087

1566271_x_at	4,242	4,405	4,394	4,106	4,200	4,380
1566272_at	4,216	3,795	4,223	4,368	4,480	4,825
1566276_at	6,439	6,462	6,331	6,708	6,883	6,513
1566277_at	5,467	5,973	5,685	5,910	5,734	5,781
1566278_at	6,500	6,222	6,179	6,442	6,674	6,240
1566279_at	5,068	5,128	5,229	5,760	5,679	5,642
1566284_at	4,679	4,787	4,666	5,349	5,100	5,239
1566285_at	4,733	4,188	4,914	4,839	4,573	4,897
1566288_at	4,823	4,670	5,303	6,641	6,426	6,493
1566289_at	4,892	5,015	4,786	5,801	5,613	5,772
1566294_at	5,092	4,627	5,094	4,787	4,643	4,602
1566295_at	5,406	4,753	4,760	6,430	6,492	6,488
1566301_at	5,612	5,542	5,506	7,016	6,991	7,250
1566302_at	5,803	5,977	6,076	7,110	6,712	6,720
1566303_s_at	8,337	8,421	8,822	9,166	8,810	9,318
1566323_at	3,663	3,699	3,836	3,722	3,651	3,838
1566324_a_at	5,247	4,471	4,305	3,955	4,051	3,777
1566337_x_at	4,302	4,859	4,552	7,125	6,510	7,218
1566339_at	4,669	4,713	4,801	5,737	5,096	5,316
1566340_at	7,091	6,513	6,924	7,697	7,324	7,854
1566342_at	5,714	6,243	6,212	7,236	6,660	6,159
1566362_at	4,170	4,241	4,188	4,604	4,442	4,822
1566363_at	6,897	7,011	6,902	7,882	7,686	7,980
1566402_at	6,808	6,896	6,876	7,087	7,077	7,110
1566403_at	5,925	5,651	6,409	6,583	5,584	5,831
1566419_at	4,667	4,631	4,854	4,641	4,882	4,854
1566424_at	6,299	6,233	5,789	5,893	5,985	5,773
1566425_at	7,532	7,428	7,641	8,044	7,857	7,962
1566426_at	6,092	6,400	6,428	6,774	6,569	6,484
1566427_at	5,894	5,743	5,581	6,660	6,276	6,619
1566428_at	3,833	4,064	4,055	3,849	3,963	3,931
1566432_at	6,356	6,409	6,300	7,850	7,400	7,819
1566433_at	6,288	6,089	6,584	6,687	6,653	6,595
1566437_at	4,285	4,416	4,371	4,732	4,237	4,407
1566438_at	4,891	4,789	4,804	5,363	5,056	5,185
1566439_at	3,345	3,271	3,556	3,472	3,473	3,530
1566440_at	6,411	6,501	6,243	6,664	6,221	6,701
1566441_at	5,795	5,724	5,735	6,675	6,721	6,576
1566442_at	4,737	4,486	4,643	4,683	4,653	4,582
1566445_at	3,833	4,192	4,118	4,832	4,474	4,814
1566446_at	5,999	6,039	5,975	5,832	7,497	5,842
1566447_at	6,056	6,024	5,754	6,175	6,180	6,046
1566448_at	6,382	6,763	6,153	7,237	6,883	7,065
1566449_at	4,054	3,921	4,097	4,195	4,231	4,474
1566452_at	7,589	7,289	7,589	7,689	7,465	7,880
1566454_at	6,759	6,647	6,774	7,591	7,065	7,659
1566455_at	5,389	5,351	5,112	6,253	5,861	5,975
1566456_at	7,973	7,905	7,863	9,287	8,626	8,866
1566457_at	6,246	6,084	6,307	6,787	6,726	6,815
1566459_at	4,302	4,262	4,159	4,169	4,191	4,113
1566461_at	4,486	4,374	4,744	4,661	4,636	4,688
1566462_at	6,061	6,122	5,908	6,365	6,312	6,343
1566463_at	5,301	4,707	4,685	6,134	6,017	6,250
1566464_at	4,162	4,181	4,094	4,430	4,235	4,276
1566465_at	5,117	5,098	5,003	5,329	5,160	5,528
1566468_at	4,100	4,092	4,116	4,040	4,134	3,943

1566469_at	5,186	5,305	5,555	5,955	5,746	5,844
1566471_at	9,726	9,435	9,489	10,134	9,912	10,132
1566472_s_at	4,955	5,712	5,031	6,611	5,823	6,577
1566473_a_at	6,397	6,597	6,969	6,587	6,750	6,698
1566474_at	7,340	7,398	6,900	7,811	7,688	8,059
1566475_at	7,046	7,124	6,816	7,354	7,374	7,373
1566476_at	6,010	6,241	6,202	6,531	6,418	6,891
1566477_at	6,524	6,594	6,899	7,770	8,056	7,948
1566478_at	4,442	4,532	4,724	4,559	4,594	4,719
1566480_x_at	7,447	7,130	7,312	7,296	7,885	7,512
1566481_at	5,517	5,615	5,560	6,131	6,271	6,155
1566482_at	6,867	6,894	6,331	6,157	7,084	6,135
1566484_at	7,582	7,691	7,514	8,288	8,015	7,954
1566485_at	3,857	4,133	4,200	4,112	4,251	4,201
1566486_at	6,281	6,388	6,605	6,265	6,190	6,304
1566487_at	3,863	3,897	3,843	4,267	4,082	4,157
1566490_at	5,719	5,380	5,484	6,429	6,373	6,322
1566491_at	5,781	5,064	5,346	4,068	4,212	4,112
1566498_at	6,808	6,782	6,813	7,456	7,210	7,180
1566499_at	5,940	5,272	5,515	6,198	6,147	6,153
1566500_at	6,115	6,093	6,099	7,069	6,839	6,908
1566501_at	6,188	6,510	6,923	7,574	7,358	7,589
1566502_at	8,302	8,208	8,360	8,982	8,463	8,760
1566503_at	6,648	6,799	6,971	6,771	6,754	6,820
1566504_at	6,820	6,930	6,997	7,088	6,769	6,674
1566505_at	5,627	5,348	5,845	6,466	6,494	6,593
1566507_a_at	5,425	5,428	5,090	5,285	5,326	5,409
1566509_s_at	9,918	9,807	10,123	10,237	9,912	10,417
1566511_at	5,098	4,995	5,176	5,304	5,008	5,372
1566512_at	7,421	7,420	7,732	7,893	7,947	7,885
1566513_a_at	6,390	6,055	6,358	6,246	6,063	6,125
1566514_at	6,125	5,861	6,297	6,264	6,212	6,294
1566515_at	3,991	4,456	4,689	3,603	3,789	3,832
1566517_at	5,344	4,991	5,229	5,335	5,384	5,501
1566518_at	4,840	4,336	4,698	4,283	4,498	4,322
1566524_a_at	6,101	6,268	6,443	6,585	6,268	6,639
1566526_at	7,136	7,160	7,351	7,288	7,368	7,515
1566527_at	6,197	6,131	6,635	7,291	7,233	7,378
1566528_at	7,534	6,865	7,700	7,153	7,082	7,088
1566535_at	6,814	7,038	6,900	7,078	7,296	7,114
1566537_at	5,399	5,442	5,511	5,447	5,299	5,572
1566538_at	5,548	5,595	5,734	5,804	5,540	5,713
1566539_at	5,212	4,772	5,543	4,345	4,372	4,403
1566540_at	4,875	4,834	5,056	5,288	5,264	5,288
1566541_at	5,109	5,015	4,948	5,266	5,118	5,034
1566543_at	5,868	5,976	6,171	6,533	6,189	6,410
1566544_at	3,579	3,658	3,668	4,390	4,720	4,386
1566548_at	7,093	6,940	7,158	7,430	7,328	7,427
1566549_at	8,081	7,557	7,935	7,928	7,792	8,013
1566550_at	5,822	5,838	6,085	6,226	5,883	5,649
1566551_at	4,221	4,331	4,438	4,542	4,497	4,588
1566555_at	5,879	6,314	6,104	6,525	6,643	6,845
1566557_at	6,910	7,254	7,150	7,365	7,637	7,858
1566558_x_at	7,395	7,689	7,876	7,815	8,049	8,001
1566569_at	7,426	7,084	7,183	7,195	7,150	7,132
1566571_at	5,570	5,513	5,768	6,178	6,057	6,403

1566576_at	5,490	5,669	5,708	6,573	6,489	6,686
1566577_at	6,602	6,898	7,098	9,083	9,059	9,350
1566580_at	6,874	6,784	6,788	7,315	7,263	7,284
1566581_at	7,242	7,125	7,575	8,453	8,587	8,591
1566582_x_at	4,482	4,292	4,057	4,576	4,861	5,130
1566585_at	4,533	4,746	4,709	4,782	4,475	4,779
1566586_at	6,754	6,742	6,580	7,394	7,281	7,520
1566596_at	6,634	6,505	6,728	6,929	6,812	6,821
1566597_at	3,943	4,106	4,028	3,981	4,043	4,153
1566598_at	5,910	5,592	6,031	6,496	6,354	6,406
1566599_at	5,775	6,005	6,120	6,071	6,135	5,999
1566600_at	4,011	4,229	3,995	4,420	4,191	4,216
1566602_at	6,729	6,392	6,867	6,798	6,918	6,924
1566603_s_at	7,490	6,695	6,539	7,196	6,355	6,736
1566605_at	3,623	3,795	3,705	3,581	3,598	3,709
1566606_a_at	4,542	4,583	4,070	4,043	4,003	4,065
1566607_at	4,255	4,555	4,574	4,508	4,379	4,195
1566608_at	4,481	4,296	4,394	4,755	4,848	5,140
1566609_at	5,583	5,899	5,609	5,987	5,887	6,170
1566622_at	6,044	5,942	6,126	6,284	6,179	6,175
1566623_at	5,523	5,356	5,318	6,065	5,692	5,632
1566624_at	3,642	3,503	3,592	3,595	3,395	3,561
1566630_at	7,017	7,034	7,148	7,828	7,452	7,612
1566631_at	5,545	6,082	5,700	5,923	6,032	5,911
1566632_at	3,900	3,877	3,697	3,919	3,890	4,138
1566633_at	6,838	6,983	6,342	6,848	7,074	7,003
1566637_at	4,324	4,278	4,474	4,206	4,140	4,029
1566638_at	6,322	5,894	6,006	5,112	5,102	5,159
1566642_at	4,848	4,811	5,178	5,848	5,690	5,938
1566643_a_at	7,268	7,057	7,169	8,047	7,612	8,153
1566644_at	4,493	4,454	4,312	4,676	4,269	4,263
1566645_at	4,870	4,820	4,840	4,945	4,594	4,901
1566646_at	7,405	7,225	7,297	8,101	8,141	8,331
1566647_s_at	6,843	6,648	6,829	8,067	7,681	7,848
1566653_at	6,932	6,663	6,501	6,994	6,657	7,133
1566656_a_at	5,894	5,911	5,641	6,855	6,635	6,313
1566658_at	6,513	6,692	7,383	5,778	5,771	5,751
1566664_at	6,206	6,243	6,196	6,801	6,777	6,890
1566665_at	4,387	4,793	4,627	5,481	5,147	5,219
1566666_at	4,279	4,799	4,495	4,648	4,664	4,741
1566670_at	7,815	7,774	7,851	8,481	8,740	8,677
1566671_a_at	6,727	6,783	7,222	7,057	6,698	6,904
1566672_at	6,108	6,130	6,046	6,298	6,205	6,250
1566673_at	4,002	3,726	4,075	3,819	3,679	3,815
1566677_at	5,226	5,312	5,370	5,368	5,138	5,206
1566678_at	6,334	6,273	6,380	7,103	7,085	7,228
1566680_at	5,653	5,731	6,026	6,447	5,709	6,124
1566688_at	6,351	6,028	6,412	6,713	6,514	6,473
1566689_at	5,875	5,503	6,062	6,175	5,962	6,324
1566690_at	7,134	7,066	7,138	7,589	7,649	7,742
1566691_at	5,118	5,142	4,952	5,292	5,153	5,172
1566693_at	5,651	6,087	5,761	6,214	5,898	6,035
1566694_at	4,192	3,983	3,906	4,434	4,391	4,811
1566695_at	4,401	4,676	4,449	4,626	4,689	4,788
1566696_at	6,378	6,557	6,488	7,354	7,228	7,234
1566697_at	6,688	6,285	6,231	6,567	6,331	6,157

1566698_at	3,546	3,989	3,759	3,979	3,852	4,127
1566700_at	7,402	7,225	7,541	7,642	7,420	7,662
1566701_at	6,079	6,206	6,360	6,857	6,819	6,679
1566702_at	5,267	5,453	5,301	5,394	5,274	5,398
1566703_at	4,152	4,374	4,338	4,425	4,190	4,240
1566709_at	6,023	5,745	5,913	6,688	6,404	6,557
1566715_at	4,124	4,426	4,333	4,399	4,511	4,344
1566716_at	3,763	3,856	3,540	3,754	3,755	3,790
1566720_at	6,246	6,264	6,434	6,776	6,651	6,732
1566721_at	6,929	6,892	7,039	7,689	7,406	7,805
1566722_a_at	5,067	4,888	4,801	5,469	5,278	5,689
1566725_at	4,192	4,560	4,617	5,062	4,779	4,811
1566726_at	5,616	5,536	5,324	5,768	5,570	5,907
1566727_at	6,239	6,294	6,246	6,430	6,300	6,436
1566728_at	5,975	6,224	6,133	6,097	6,039	6,050
1566734_at	6,442	6,753	6,817	6,746	6,476	6,940
1566735_at	5,849	5,432	6,066	6,143	6,185	6,310
1566737_at	5,301	5,131	5,169	5,510	5,327	5,484
1566739_at	5,781	5,985	5,974	6,246	6,034	6,134
1566740_at	5,901	5,826	5,747	5,875	6,093	6,252
1566748_at	5,159	4,789	4,756	4,745	4,676	4,534
1566749_at	3,531	3,509	3,486	3,705	3,702	3,978
1566760_at	7,149	6,733	6,955	7,613	7,200	7,878
1566761_a_at	7,106	7,134	7,072	7,772	7,483	7,607
1566762_at	3,517	3,788	3,724	4,038	3,690	3,691
1566763_at	5,709	5,790	5,532	6,262	6,209	6,215
1566764_at	4,117	4,938	4,023	3,964	3,784	3,838
1566766_a_at	4,799	5,201	5,257	5,809	5,501	5,749
1566767_at	5,834	5,753	5,732	6,212	5,905	5,954
1566768_at	4,552	4,489	4,229	4,709	4,278	4,624
1566771_at	5,503	5,706	5,727	6,225	5,978	5,961
1566772_at	3,991	4,080	4,194	4,441	4,245	4,400
1566775_at	7,217	7,230	7,210	8,118	8,048	7,852
1566776_at	6,367	6,054	6,288	7,043	6,816	7,014
1566777_at	4,250	4,262	4,271	4,293	4,195	4,242
1566778_at	5,585	5,603	5,761	6,301	6,217	6,336
1566779_at	6,633	6,466	6,491	7,480	7,194	7,498
1566780_at	5,249	5,358	5,326	5,635	5,458	5,498
1566783_at	5,255	5,922	5,764	4,960	4,839	5,102
1566784_at	5,141	5,068	5,249	5,568	5,208	5,215
1566785_x_at	8,373	7,886	8,037	8,149	8,679	8,157
1566786_at	6,212	6,101	6,356	7,372	7,241	7,286
1566787_at	4,970	4,889	5,222	4,286	4,664	4,477
1566798_at	5,015	5,010	4,937	4,987	5,115	5,076
1566803_at	7,337	7,302	7,486	7,689	7,216	7,556
1566804_at	4,967	5,320	5,126	5,491	5,583	5,344
1566805_at	3,862	4,014	3,917	4,384	3,943	4,043
1566807_a_at	5,865	5,592	5,845	6,486	6,352	6,323
1566809_a_at	6,257	6,315	6,022	5,766	5,987	5,902
1566814_at	6,924	6,695	6,825	7,909	7,317	7,287
1566816_at	7,512	7,337	7,496	8,438	8,101	8,251
1566821_at	7,247	7,117	7,255	7,530	7,445	7,535
1566823_a_at	6,881	6,555	6,657	7,979	7,864	8,119
1566824_at	6,856	6,703	6,888	7,120	6,570	6,899
1566825_at	4,352	4,391	3,779	4,345	4,344	4,261
1566830_at	7,403	7,130	7,260	7,759	7,885	7,866

1566831_at	6,242	6,145	6,164	6,388	6,015	6,375
1566832_at	5,181	4,902	5,124	5,044	5,111	5,140
1566833_x_at	4,006	3,966	3,881	4,001	3,949	4,182
1566834_at	5,817	5,652	5,849	5,922	5,920	6,189
1566835_at	6,302	6,012	6,118	7,066	6,926	7,287
1566836_at	5,238	5,534	5,518	5,637	5,679	5,960
1566837_at	6,405	5,673	6,398	6,614	6,523	6,851
1566839_at	6,543	6,488	6,310	7,391	7,231	7,594
1566840_at	5,402	4,857	5,094	5,542	5,253	5,472
1566841_at	6,765	6,711	6,677	7,400	7,123	7,515
1566842_at	6,018	5,704	5,556	6,530	6,050	6,344
1566843_at	4,561	4,544	4,542	4,679	4,474	4,545
1566844_at	5,886	5,771	5,915	6,205	6,217	6,283
1566846_at	4,238	3,985	4,183	4,460	4,454	4,440
1566848_x_at	7,769	7,160	7,469	7,466	7,477	7,336
1566851_at	6,004	6,516	6,382	6,829	6,441	6,730
1566852_at	5,574	5,733	6,124	5,908	5,878	5,840
1566853_at	6,493	6,194	6,470	6,393	6,569	6,637
1566854_at	6,751	7,142	6,917	7,177	7,226	7,367
1566860_at	3,919	4,053	3,945	4,175	3,916	3,955
1566861_at	4,696	4,935	5,320	5,065	5,204	5,615
1566862_at	4,054	4,071	4,188	4,126	3,989	3,945
1566863_at	4,917	4,773	4,824	5,007	4,992	4,869
1566865_at	4,131	4,322	3,932	4,728	4,871	4,720
1566866_at	5,982	5,614	5,900	6,320	5,868	6,158
1566867_at	4,962	5,023	5,120	5,601	5,687	5,954
1566868_at	5,740	5,788	5,592	5,914	5,849	5,875
1566869_at	4,011	4,121	4,141	3,973	3,992	3,983
1566870_at	6,008	5,769	5,968	6,212	6,376	6,258
1566873_at	5,448	5,310	5,505	6,126	5,927	6,162
1566874_at	5,511	5,794	5,424	6,492	6,066	6,245
1566879_at	5,579	5,671	5,800	6,102	5,861	6,091
1566880_at	6,065	5,861	6,311	6,194	6,230	6,088
1566881_at	4,832	4,549	4,590	4,984	5,032	5,030
1566882_at	5,002	5,245	5,013	5,398	4,859	5,134
1566884_at	6,864	6,833	6,739	7,570	7,366	7,450
1566885_at	6,140	6,080	6,075	6,531	6,598	6,466
1566887_x_at	9,302	8,923	9,057	8,697	9,824	9,151
1566888_at	5,657	5,509	5,889	5,871	5,826	5,739
1566889_at	5,054	4,843	4,963	4,492	4,490	4,603
1566892_at	4,291	4,387	4,442	4,590	4,633	4,500
1566893_at	4,473	4,500	4,575	4,873	4,505	4,502
1566896_at	4,182	4,263	3,982	4,360	4,352	4,428
1566897_at	6,182	6,327	7,038	5,527	6,005	5,810
1566898_at	5,158	4,790	5,155	5,351	5,077	5,247
1566899_at	4,674	4,405	4,336	4,747	4,766	5,182
1566900_at	5,478	5,498	5,862	6,181	6,125	5,903
1566901_at	7,118	6,478	6,288	4,334	5,894	5,223
1566902_at	5,256	5,220	5,200	5,879	5,805	5,916
1566903_at	7,833	7,717	8,277	7,522	7,633	7,748
1566906_at	7,584	7,438	7,497	7,673	7,531	7,707
1566908_at	6,203	5,962	6,264	6,658	6,505	6,711
1566916_at	5,326	5,264	5,496	5,949	6,136	6,053
1566917_at	6,611	6,710	7,010	6,760	6,930	6,813
1566923_at	6,965	6,681	6,925	7,113	7,006	7,151
1566924_at	6,338	6,393	6,490	7,525	7,480	7,626

1566926_at	6,567	6,174	6,491	6,842	6,653	7,079
1566927_at	6,522	6,301	5,964	6,755	6,809	6,947
1566930_at	3,291	3,381	3,299	3,368	3,341	3,441
1566931_at	7,223	6,825	7,086	7,823	7,580	8,036
1566932_x_at	7,237	6,962	7,070	7,710	7,734	7,634
1566934_at	6,867	6,752	6,792	7,481	7,497	7,664
1566935_at	5,975	5,436	5,978	6,622	5,938	6,403
1566937_at	6,206	6,100	6,055	6,207	6,301	5,965
1566938_at	5,833	5,971	6,295	6,706	6,624	6,657
1566947_at	4,919	4,890	5,042	5,785	5,456	5,273
1566948_at	5,581	5,538	5,633	5,961	5,531	5,746
1566949_at	6,848	6,784	6,845	7,296	7,204	7,422
1566950_at	3,827	3,862	3,888	4,294	4,485	4,365
1566951_at	5,391	5,936	5,557	6,469	6,162	6,194
1566952_at	4,409	4,540	4,710	4,031	4,219	3,919
1566953_x_at	4,037	4,374	4,421	3,994	4,112	3,969
1566954_at	7,420	7,671	7,829	8,194	7,936	8,342
1566955_at	6,104	5,976	6,284	6,820	6,899	7,292
1566956_at	10,213	10,076	10,136	11,185	10,608	11,125
1566957_at	7,827	7,872	7,811	8,660	8,810	9,063
1566958_at	4,919	5,343	5,252	5,373	5,261	5,158
1566959_at	5,250	4,540	4,880	3,890	4,764	4,197
1566963_at	5,193	4,819	4,975	5,611	5,262	5,441
1566964_at	5,433	5,135	5,288	5,273	5,456	5,206
1566965_at	5,558	5,645	5,460	5,928	6,197	5,632
1566966_at	4,460	4,265	4,082	5,208	5,465	5,323
1566967_at	6,082	5,975	6,221	6,240	6,240	6,203
1566968_at	7,987	7,766	7,568	7,395	7,473	7,445
1566969_at	3,580	3,618	3,838	3,859	3,812	3,751
1566970_at	3,719	3,403	3,570	3,691	3,643	3,765
1566973_at	7,483	7,170	7,285	7,748	7,634	8,040
1566974_at	7,375	7,722	7,897	7,923	7,764	8,019
1566978_at	5,004	4,844	4,753	5,283	5,159	5,133
1566979_at	5,109	5,207	5,287	5,514	5,766	5,758
1566980_at	6,503	6,059	6,292	6,836	6,801	7,023
1566981_at	5,590	5,591	5,543	5,919	5,929	6,183
1566984_at	6,529	6,621	6,980	7,179	7,036	7,206
1566986_at	4,303	4,446	4,175	4,837	4,720	5,036
1566987_s_at	5,927	6,257	6,056	6,417	6,149	6,209
1566989_at	10,476	10,771	10,750	10,729	10,692	10,914
1566990_x_at	10,570	10,486	10,781	10,775	10,761	10,961
1566991_at	5,282	5,420	5,859	6,285	5,841	6,056
1566994_at	6,823	6,760	6,849	7,380	7,359	7,424
1566995_at	4,100	4,000	3,912	4,630	4,771	4,923
1566999_at	6,172	6,387	6,023	6,836	6,653	6,753
1567000_at	3,757	3,682	3,950	3,825	3,975	3,791
1567008_at	5,590	5,542	5,729	6,013	5,950	6,176
1567009_at	4,838	4,977	4,930	5,142	5,296	5,325
1567010_at	6,977	6,956	6,937	7,456	7,450	7,337
1567011_at	5,353	5,559	5,353	5,733	5,399	5,638
1567013_at	6,237	5,892	6,290	7,713	7,722	7,737
1567014_s_at	9,368	9,475	9,367	7,438	7,327	7,503
1567015_at	6,107	5,946	6,189	6,422	5,739	6,252
1567022_at	5,363	5,363	5,636	6,555	5,949	6,495
1567023_at	6,508	6,625	6,825	7,148	6,826	7,044
1567027_at	6,513	6,729	7,083	6,689	6,374	6,759

1567028_s_at	5,781	5,696	5,841	5,419	5,684	5,439
1567030_at	7,130	6,719	6,941	7,130	7,032	7,138
1567031_at	5,218	5,259	4,958	5,424	5,443	5,315
1567032_s_at	4,190	3,907	4,271	4,895	4,633	4,791
1567035_at	6,881	6,827	6,808	7,012	7,060	7,185
1567036_at	6,564	6,606	6,760	7,615	7,921	7,992
1567044_s_at	5,031	4,547	4,966	4,498	5,092	4,956
1567045_at	6,411	6,364	6,254	6,093	6,094	5,788
1567054_at	6,118	5,623	5,427	7,543	7,349	7,808
1567055_at	5,276	5,525	5,648	5,912	6,090	5,974
1567056_at	5,757	5,253	5,838	6,427	6,300	6,520
1567058_at	4,406	4,705	4,979	4,692	4,853	4,879
1567059_at	5,762	5,516	5,543	6,266	5,723	5,846
1567060_at	5,855	5,606	5,929	6,202	6,096	6,170
1567064_at	4,896	4,435	4,817	6,402	6,188	6,426
1567065_at	6,391	6,204	6,408	6,886	7,026	6,833
1567068_at	7,044	6,624	6,724	7,658	7,966	7,933
1567069_at	6,504	6,377	6,488	7,241	7,168	7,423
1567076_at	4,608	4,916	5,021	7,604	7,263	7,797
1567078_x_at	5,251	5,301	5,087	6,441	5,973	6,112
1567079_at	6,521	6,517	6,605	7,480	7,328	7,391
1567080_s_at	9,022	8,478	9,287	8,856	8,161	8,464
1567081_x_at	8,037	8,016	8,619	8,530	7,933	8,337
1567100_at	5,338	5,437	5,520	5,982	5,885	5,862
1567101_at	5,096	5,063	5,056	5,372	5,133	5,174
1567105_at	7,427	7,186	7,121	7,254	7,194	7,574
1567107_s_at	9,338	9,120	9,071	11,169	10,438	11,529
1567139_at	5,084	5,159	5,169	6,600	6,080	6,202
1567140_at	6,250	6,593	6,513	7,034	7,085	7,287
1567166_at	7,137	7,299	7,355	7,437	7,273	7,445
1567167_at	6,664	6,541	6,211	7,377	7,576	7,481
1567173_at	3,413	3,504	3,382	3,689	3,461	3,669
1567174_at	3,667	4,016	3,775	4,090	3,898	3,974
1567179_at	3,891	4,233	3,914	3,822	3,932	3,844
1567181_x_at	4,162	4,538	4,086	4,112	3,981	4,065
1567182_at	3,423	3,391	3,712	3,408	3,365	3,408
1567183_s_at	5,247	5,328	5,146	5,065	5,133	5,073
1567213_at	7,699	7,810	7,350	6,083	6,733	5,687
1567214_a_at	11,231	11,211	10,242	8,912	10,539	7,941
1567219_at	6,890	6,792	6,710	6,983	6,893	6,808
1567222_x_at	7,171	7,153	7,197	6,973	7,097	6,706
1567223_at	4,899	4,939	4,893	5,759	5,642	5,791
1567224_at	7,044	6,757	7,149	8,449	10,170	9,752
1567237_at	5,536	5,722	5,646	7,370	7,214	7,386
1567238_at	5,437	5,037	5,487	6,838	7,233	7,552
1567240_x_at	5,509	5,360	5,510	6,683	7,141	7,252
1567241_at	5,491	5,592	5,517	5,997	5,970	6,230
1567242_at	6,157	6,366	6,478	7,128	6,925	7,172
1567244_at	4,730	4,930	4,547	5,463	5,255	5,638
1567245_at	4,819	5,030	5,324	5,203	5,291	5,323
1567246_at	3,811	3,609	3,634	4,016	4,152	4,694
1567247_at	4,022	3,910	3,695	3,804	3,671	3,747
1567248_at	4,790	4,250	4,555	5,164	4,737	5,303
1567249_at	4,083	4,085	4,146	4,253	4,288	4,345
1567250_at	6,062	5,595	6,023	6,764	6,427	6,678
1567251_at	5,326	5,165	5,459	6,000	6,060	6,256

1567252_at	6,293	6,325	6,417	7,303	7,297	7,344
1567253_at	7,019	7,091	7,083	7,365	7,333	7,279
1567254_at	6,247	5,918	6,101	7,108	7,097	7,212
1567255_at	7,135	6,787	6,900	7,528	7,181	7,608
1567256_at	6,147	6,416	6,600	7,912	8,243	8,490
1567257_at	6,870	7,118	6,913	7,890	7,718	7,685
1567270_at	6,466	6,578	6,402	7,320	7,478	7,797
1567271_at	5,441	5,664	5,641	6,084	5,866	6,264
1567272_at	6,559	7,051	6,473	7,203	6,892	7,260
1567273_at	5,953	5,634	5,465	5,874	5,560	5,992
1567274_at	8,081	8,044	7,814	8,358	8,409	8,060
1567277_at	5,447	4,860	5,274	5,697	5,445	5,825
1567280_at	4,928	4,797	4,652	5,427	5,357	5,750
1567281_at	3,557	3,577	3,712	3,567	3,648	3,767
1567282_at	6,930	7,543	6,880	8,962	9,034	9,185
1567284_at	4,590	5,126	4,801	5,233	4,960	5,215
1567285_at	7,416	7,462	7,542	8,791	8,911	8,773
1567286_at	6,386	6,421	6,630	7,157	7,057	7,200
1567287_at	4,325	3,931	4,155	4,685	4,767	4,952
1567288_at	3,525	3,619	3,830	3,817	3,781	3,965
1567297_at	7,090	7,035	7,259	7,748	7,438	7,386
1567298_at	7,256	7,224	7,427	7,915	7,844	8,212
1567303_at	3,763	3,963	4,038	4,207	4,012	4,073
1567304_at	5,864	6,036	5,740	6,545	6,042	5,831
1567319_at	6,733	6,297	6,227	7,323	7,178	7,292
1567320_at	6,143	6,643	6,407	7,278	6,985	7,275
1567333_at	4,198	4,077	4,391	4,878	4,421	4,412
1567334_at	5,202	5,324	5,486	5,639	5,602	5,829
1567341_at	6,186	6,026	6,294	7,071	6,727	7,232
1567357_at	5,506	5,124	5,716	6,107	5,867	5,938
1567358_at	5,155	5,212	5,303	6,061	5,812	5,808
1567359_at	3,793	4,139	3,978	3,890	3,874	3,802
1567361_at	5,092	4,972	4,824	4,182	4,527	4,313
1567374_at	6,135	6,157	5,966	6,617	6,438	6,530
1567375_at	8,034	8,093	8,131	8,615	8,766	8,844
1567376_at	4,844	4,609	4,460	5,459	5,248	5,333
1567377_at	5,199	5,015	4,960	6,099	6,110	6,282
1567378_x_at	4,120	4,400	4,172	4,879	4,798	4,948
1567379_at	3,685	3,806	3,746	3,663	3,687	3,783
1567380_at	3,705	3,523	3,653	3,602	3,752	3,886
1567386_at	4,546	4,814	4,476	5,126	5,604	5,740
1567387_at	3,523	3,487	3,746	4,001	4,016	4,121
1567389_at	5,122	5,721	5,730	6,543	6,226	6,506
1567390_at	4,572	4,441	4,611	5,336	5,238	5,150
1567410_at	7,433	7,510	7,716	8,137	8,005	8,152
1567440_at	6,631	6,886	6,971	7,363	7,260	7,180
1567443_x_at	6,574	6,451	6,508	7,477	7,115	7,641
1567457_at	5,440	5,292	5,423	4,992	4,760	5,005
1567458_s_at	9,965	9,844	9,469	8,869	8,548	8,773
1567519_at	5,685	6,127	5,421	6,543	6,517	6,580
1567527_at	3,977	3,960	4,024	4,076	4,004	4,146
1567539_at	7,503	7,456	7,464	7,997	7,976	7,807
1567540_at	7,385	7,432	7,667	8,606	8,692	8,325
1567558_at	5,764	6,035	6,129	6,286	6,166	6,292
1567559_s_at	5,161	5,219	5,186	5,287	5,281	5,343
1567575_at	5,890	6,008	5,541	6,138	6,107	6,149

1567576_at	3,778	3,872	4,008	4,423	4,173	4,318
1567590_at	5,136	5,320	5,106	6,818	6,601	6,770
1567591_at	6,678	6,744	6,924	7,112	7,017	7,307
1567611_at	6,412	6,407	6,164	6,618	6,169	6,541
1567612_at	5,191	4,785	5,049	5,586	5,701	5,612
1567622_at	6,656	6,729	6,631	7,250	7,318	7,386
1567623_at	5,694	5,859	6,042	8,118	7,915	7,446
1567624_at	6,259	6,030	6,001	7,255	6,857	6,862
1567627_at	7,480	7,134	7,272	8,048	8,044	8,226
1567628_at	5,527	5,894	5,980	6,585	6,610	6,752
1567656_at	3,781	4,074	3,688	3,909	4,072	3,946
1567657_at	4,104	4,349	4,072	4,207	3,893	4,139
1567663_at	3,654	3,607	3,678	3,991	3,625	3,581
1567664_at	3,884	3,753	3,551	4,208	4,040	4,282
1567679_at	4,695	4,921	4,968	5,056	4,921	5,177
1567681_at	5,658	5,180	5,386	5,890	6,176	6,087
1567682_x_at	5,062	4,735	4,865	5,679	5,703	5,961
1567686_at	5,722	5,857	5,705	6,356	6,230	6,233
1567687_at	4,794	4,953	5,580	7,624	7,657	8,006
1567696_at	3,881	4,147	4,063	4,199	4,104	4,156
1567697_at	3,854	4,203	4,052	4,940	5,096	5,077
1567698_x_at	3,551	3,884	4,100	4,476	4,545	4,525
1567702_at	3,837	4,056	4,074	4,216	4,070	4,031
1567703_at	5,230	5,410	5,102	5,735	5,200	5,435
1567704_at	5,897	6,195	6,147	6,647	6,610	6,671
1567705_at	3,561	3,806	3,805	3,768	3,671	3,721
1567706_at	3,735	3,827	3,746	3,724	3,823	3,679
1567853_at	4,267	4,434	4,359	3,929	3,791	3,884
1567855_at	4,277	4,511	4,538	4,031	4,029	4,091
1567856_x_at	5,134	4,989	4,662	4,595	4,522	4,743
1567859_at	4,883	4,544	4,666	5,253	5,462	5,382
1567860_at	4,485	5,060	4,541	5,268	5,326	5,466
1567861_at	3,652	3,586	3,618	3,560	3,610	3,589
1567862_at	4,363	4,197	4,229	4,188	4,057	4,106
1567878_at	3,606	3,475	3,578	3,719	3,570	3,720
1567906_at	6,354	6,106	6,360	6,786	6,357	6,580
1567912_s_at	3,579	3,791	3,506	4,031	3,931	3,888
1567913_at	8,482	8,505	8,513	10,426	9,246	9,216
1567985_at	8,407	8,448	8,600	9,110	8,736	9,085
1567986_at	5,257	5,373	5,772	5,817	5,618	5,757
1567997_x_at	6,302	6,560	6,363	7,064	7,009	6,778
1568012_at	3,821	3,803	3,727	3,637	3,708	3,677
1568126_at	4,586	4,018	4,346	6,878	7,066	6,096
1568190_at	3,426	3,786	3,936	3,473	3,541	3,471
1568191_at	7,818	7,929	7,974	8,576	7,823	8,171
1568248_x_at	6,365	6,106	6,497	6,849	6,386	6,397
1568249_at	9,038	8,768	8,992	11,443	10,841	10,905
1568286_at	3,656	3,649	3,678	4,143	3,924	3,888
1568287_at	3,606	3,821	4,085	5,730	5,710	4,866
1568365_at	4,137	4,520	4,519	4,398	4,496	4,398
1568366_at	3,924	4,152	3,792	4,579	4,660	4,579
1568375_at	5,185	4,880	4,979	5,275	5,229	5,312
1568377_x_at	8,046	8,023	8,204	8,741	8,396	8,928
1568406_at	4,374	4,029	4,063	3,968	3,671	3,814
1568408_x_at	8,945	8,855	9,164	8,785	8,573	8,781
1568448_at	5,874	6,240	5,612	6,445	6,386	6,457

1568449_at	3,741	4,014	4,563	5,630	5,679	5,796
1568487_x_at	4,137	4,132	4,154	3,874	4,030	4,001
1568488_at	4,175	4,356	4,317	4,403	4,354	4,386
1568513_x_at	11,464	10,855	11,045	12,777	12,355	12,423
1568554_x_at	4,231	3,768	3,932	4,568	4,199	4,356
1568558_x_at	6,190	6,159	6,212	6,600	6,429	6,265
1568574_x_at	3,869	4,032	4,230	4,666	4,124	4,183
1568589_at	5,672	5,804	5,725	6,223	6,183	6,158
1568590_at	8,856	8,751	8,628	9,081	9,001	9,231
1568592_at	7,444	7,949	7,776	7,872	7,570	7,579
1568593_a_at	7,285	7,871	7,752	6,790	6,515	6,842
1568594_s_at	5,831	5,619	6,190	5,610	5,654	5,510
1568596_a_at	8,444	8,414	8,258	8,499	8,137	8,347
1568597_at	7,902	7,586	7,872	7,358	7,355	7,400
1568598_at	7,395	7,131	7,227	8,194	7,698	8,324
1568600_at	7,957	7,773	8,034	6,739	6,699	6,667
1568601_at	5,594	5,599	5,373	5,565	5,369	5,716
1568603_at	4,758	5,642	5,629	3,999	3,854	3,976
1568604_a_at	7,832	7,806	8,533	6,244	6,088	6,186
1568605_at	8,227	7,820	8,183	7,934	7,966	8,265
1568606_at	6,614	6,613	6,439	6,842	6,976	7,217
1568609_s_at	7,931	7,793	7,937	8,012	7,463	8,335
1568611_at	7,197	7,835	7,256	7,731	8,210	7,923
1568612_at	6,636	7,597	5,998	4,146	3,925	4,164
1568613_at	9,123	9,010	8,975	9,939	9,771	10,040
1568615_a_at	5,258	5,418	5,708	6,162	5,806	5,862
1568616_a_at	6,410	6,446	6,871	7,648	7,562	7,766
1568617_a_at	6,522	5,941	6,466	6,707	6,534	6,766
1568618_a_at	10,355	10,550	10,095	9,491	11,857	10,353
1568619_s_at	7,079	6,670	6,716	8,100	8,930	8,477
1568620_at	5,865	5,783	5,749	6,212	6,028	6,331
1568623_a_at	5,856	5,923	5,989	8,125	7,497	7,654
1568625_at	6,351	6,089	6,142	6,901	6,859	7,292
1568627_at	8,001	8,532	8,213	7,438	7,224	7,252
1568629_s_at	10,027	9,862	10,297	9,921	9,631	9,864
1568633_a_at	5,722	5,600	5,394	5,974	5,724	5,975
1568634_a_at	5,346	5,449	5,454	6,478	7,168	7,138
1568635_at	5,319	5,112	5,277	5,787	5,546	5,778
1568636_a_at	5,396	5,770	5,457	5,772	5,517	5,679
1568638_a_at	5,381	5,206	5,287	5,680	5,750	5,753
1568639_a_at	7,341	7,117	7,348	8,023	7,823	8,201
1568640_at	7,441	7,820	7,351	7,114	7,324	7,228
1568643_a_at	6,258	6,279	6,552	6,137	6,045	6,134
1568644_at	6,183	6,462	5,662	3,996	3,765	4,016
1568646_x_at	7,349	7,725	6,867	4,746	4,459	4,936
1568647_at	5,083	4,944	4,981	5,201	5,157	5,380
1568648_a_at	4,946	4,782	4,953	5,062	5,177	5,021
1568649_at	9,409	9,242	9,196	9,990	9,821	10,153
1568650_a_at	4,952	4,748	4,874	5,089	5,072	5,224
1568655_a_at	5,884	5,430	5,932	6,439	6,150	6,142
1568656_at	4,571	4,694	4,586	5,366	5,186	5,228
1568658_at	9,227	9,759	8,980	8,773	7,394	8,464
1568660_a_at	4,137	4,260	4,122	4,500	4,191	4,269
1568661_at	4,386	4,469	4,577	4,224	4,219	4,227
1568662_at	3,725	3,700	3,766	3,712	3,840	3,814
1568663_a_at	5,647	5,832	5,434	6,359	5,498	5,851

1568665_at	6,218	7,716	7,581	6,784	7,316	6,921
1568666_at	6,832	7,015	7,106	8,060	7,796	8,047
1568667_s_at	7,844	7,480	7,581	8,799	8,799	9,089
1568672_at	3,558	3,808	3,504	4,608	4,454	4,674
1568673_s_at	3,779	3,860	3,596	3,877	3,738	3,835
1568675_at	6,424	6,505	6,522	7,136	7,003	7,254
1568678_s_at	9,491	9,370	9,297	10,094	9,760	9,728
1568680_s_at	7,137	6,982	7,178	7,078	6,522	6,804
1568682_a_at	6,523	6,408	6,672	7,153	6,771	6,727
1568683_at	6,428	6,364	6,123	7,043	7,226	7,340
1568685_at	4,606	4,709	5,044	5,470	5,403	5,686
1568686_at	3,690	3,672	3,776	4,025	3,889	3,690
1568687_s_at	4,398	4,620	4,708	4,786	4,499	4,880
1568689_at	4,850	4,781	5,055	5,706	5,911	6,229
1568690_a_at	5,435	5,307	5,415	5,486	5,465	5,505
1568691_at	8,277	8,300	8,539	9,450	9,269	9,503
1568695_s_at	5,498	4,484	5,228	5,306	5,323	5,353
1568696_at	3,893	4,217	3,991	4,073	4,043	4,141
1568698_at	5,211	5,272	5,474	5,703	5,642	5,620
1568699_at	7,389	7,516	7,700	7,903	7,424	7,694
1568702_a_at	7,471	7,434	7,382	7,100	7,368	7,113
1568704_a_at	9,065	8,845	9,209	9,452	9,156	9,528
1568706_s_at	6,914	7,037	7,112	7,302	7,371	7,385
1568711_a_at	3,480	3,772	3,680	3,971	3,963	3,919
1568713_a_at	5,025	5,098	5,054	5,950	5,601	5,517
1568717_a_at	5,110	5,159	4,880	5,233	5,221	5,223
1568718_at	6,991	7,143	7,040	7,438	7,245	7,603
1568719_s_at	8,040	7,167	7,545	8,584	8,414	8,750
1568720_at	6,224	7,124	6,303	4,467	4,407	5,768
1568722_at	4,895	5,089	4,912	5,279	5,273	5,171
1568723_at	6,291	6,178	6,293	6,885	7,298	6,972
1568728_s_at	7,327	7,262	7,466	8,521	8,540	8,731
1568730_at	5,766	5,352	5,247	5,973	5,709	6,069
1568732_at	5,134	4,988	5,092	4,641	4,376	4,688
1568733_at	6,159	6,257	6,311	6,450	6,256	6,309
1568734_a_at	6,893	7,020	6,891	7,364	7,225	7,291
1568735_at	5,488	5,604	5,741	5,698	5,417	5,453
1568736_s_at	4,812	4,900	4,551	4,242	4,047	4,296
1568739_at	4,936	4,523	4,461	5,970	5,516	5,775
1568742_at	5,859	6,308	5,208	5,854	6,277	6,186
1568743_at	8,005	7,681	7,727	8,330	8,105	8,249
1568745_at	6,054	6,176	6,267	6,599	6,356	6,707
1568746_a_at	5,327	5,085	5,196	6,080	5,679	5,696
1568748_at	6,797	6,610	6,758	7,135	7,160	7,183
1568749_at	4,457	4,815	4,752	4,609	4,647	4,881
1568751_at	3,316	3,384	3,432	3,387	3,443	3,495
1568752_s_at	3,579	3,577	3,631	3,886	3,687	3,826
1568754_at	7,965	7,826	7,970	9,483	9,297	9,406
1568755_a_at	4,070	3,673	4,125	4,255	4,145	4,338
1568760_at	6,944	6,781	7,056	7,359	7,463	7,382
1568762_at	5,370	5,534	5,692	5,661	5,846	5,793
1568763_s_at	9,212	9,579	9,566	9,392	9,458	9,045
1568764_x_at	6,461	6,380	6,590	6,358	6,400	6,257
1568765_at	4,781	4,678	4,697	6,102	5,941	6,320
1568768_s_at	6,933	6,805	6,659	7,337	7,143	7,235
1568770_at	6,696	6,593	6,537	7,239	6,855	6,627

1568777_at	4,716	4,914	4,842	3,967	4,016	4,076
1568779_a_at	7,400	7,039	7,020	7,827	7,518	7,876
1568780_at	9,607	8,929	9,280	5,828	6,071	6,072
1568781_at	7,705	8,130	8,675	8,846	7,547	8,104
1568782_at	4,708	4,618	4,734	4,852	4,843	4,965
1568783_at	5,289	5,146	5,326	5,486	5,348	5,466
1568784_at	3,741	3,629	3,642	3,820	3,851	3,851
1568785_a_at	3,967	4,226	3,683	4,400	4,430	4,540
1568787_at	4,482	4,944	5,076	4,447	4,230	4,386
1568789_at	5,703	5,780	5,281	6,097	6,129	5,921
1568791_s_at	5,616	5,760	5,774	6,258	6,129	6,160
1568794_at	4,971	4,941	4,827	5,153	5,033	5,131
1568795_at	4,959	5,320	5,173	5,401	5,479	5,668
1568796_at	7,492	7,513	7,909	7,940	7,531	7,904
1568799_at	6,690	6,149	6,476	6,119	6,208	5,974
1568801_at	6,533	6,165	6,495	5,899	5,995	5,910
1568802_at	3,965	4,341	4,137	4,493	4,613	4,545
1568803_at	6,125	5,266	5,688	5,606	5,592	5,669
1568804_at	6,264	6,461	6,559	6,925	6,911	7,187
1568805_at	3,925	3,842	4,006	3,873	4,032	4,003
1568807_a_at	5,472	5,442	5,405	5,097	5,148	5,167
1568812_at	5,138	5,498	5,518	5,566	5,493	5,449
1568813_at	6,477	5,453	6,800	5,201	4,899	5,206
1568814_at	7,318	6,929	7,497	8,237	7,574	7,758
1568815_a_at	8,222	7,865	8,086	6,390	7,378	6,560
1568817_at	5,281	5,229	5,126	4,492	4,405	4,498
1568820_a_at	4,667	4,779	4,797	4,918	5,234	5,050
1568821_at	7,412	7,147	7,368	7,648	7,591	7,572
1568822_at	5,547	5,333	5,306	5,585	5,703	5,919
1568826_at	5,072	5,488	5,322	5,205	5,086	5,134
1568827_at	5,485	5,745	5,710	6,299	6,188	6,329
1568828_at	6,756	6,765	6,556	6,914	6,954	7,054
1568830_at	5,578	5,616	5,411	5,811	5,694	5,677
1568832_a_at	4,814	4,724	4,842	5,014	4,724	4,977
1568834_s_at	7,568	7,141	7,286	6,395	6,959	6,592
1568836_at	4,711	4,549	4,516	4,388	4,638	4,538
1568838_at	5,598	6,530	6,514	5,482	4,845	5,398
1568840_at	4,601	4,765	4,681	4,701	4,750	4,642
1568843_at	6,050	6,272	6,159	6,651	6,696	6,796
1568844_at	5,540	5,308	6,050	6,422	6,385	6,401
1568845_at	7,984	8,094	8,036	8,498	8,343	8,342
1568846_at	5,674	6,011	5,623	6,434	6,462	6,430
1568847_at	7,094	7,033	7,119	8,023	7,882	7,978
1568848_at	3,786	3,629	3,989	4,487	4,482	4,487
1568849_at	6,194	6,073	6,036	6,614	6,365	6,776
1568850_at	6,387	6,264	6,405	7,231	6,830	7,113
1568851_at	4,798	4,609	5,000	4,899	4,881	4,959
1568852_x_at	3,870	3,825	3,875	3,941	3,756	3,934
1568853_at	7,499	7,504	7,467	8,014	7,951	7,992
1568854_at	4,632	4,744	4,876	4,556	4,641	4,534
1568856_at	4,031	4,333	4,768	4,587	4,608	4,622
1568857_a_at	5,911	5,744	6,773	5,459	5,913	5,562
1568858_at	5,264	4,976	5,352	4,228	4,387	4,257
1568859_a_at	7,044	6,852	7,092	7,483	7,173	7,434
1568864_at	5,491	5,376	5,314	5,492	5,181	5,395
1568865_at	5,726	5,646	5,530	4,889	5,129	5,184

1568866_at	4,029	3,869	3,847	3,560	3,849	3,847
1568867_x_at	3,687	3,673	3,589	3,431	3,735	3,481
1568868_at	7,184	6,705	6,542	5,539	5,778	5,757
1568869_at	6,049	6,069	5,815	6,229	6,059	6,033
1568870_at	6,302	6,470	6,238	6,328	6,034	6,774
1568871_at	3,640	3,663	4,008	4,068	4,013	3,914
1568872_at	3,590	3,995	3,630	3,780	3,795	3,900
1568873_at	8,730	8,061	8,407	5,134	5,288	4,822
1568874_at	4,695	4,845	4,731	5,301	4,723	4,955
1568876_a_at	6,000	5,965	5,872	6,305	6,064	5,646
1568877_a_at	6,874	7,277	6,839	7,359	7,306	7,238
1568878_at	5,047	5,049	5,305	5,282	5,271	5,278
1568879_a_at	7,179	7,038	7,040	8,127	7,546	7,798
1568882_at	5,210	5,762	5,535	5,276	5,281	5,661
1568883_at	7,265	7,824	6,768	5,124	5,025	5,277
1568884_at	4,172	4,408	4,103	4,143	4,256	4,197
1568887_at	5,716	5,568	5,479	6,475	6,086	6,049
1568888_at	4,560	4,468	4,479	4,962	4,492	4,659
1568889_at	4,850	4,849	4,926	4,193	4,176	4,067
1568891_x_at	4,600	4,684	4,443	4,327	4,472	4,205
1568892_at	4,259	4,452	4,167	4,177	4,178	4,219
1568894_at	6,099	6,049	6,423	6,699	6,755	6,759
1568896_at	4,103	4,066	3,894	3,991	3,977	3,948
1568897_at	4,706	4,882	4,964	5,133	5,263	5,283
1568898_at	3,588	3,607	3,713	3,884	3,799	3,796
1568899_at	4,861	4,833	4,892	5,367	4,996	5,345
1568900_a_at	5,046	5,875	4,873	5,065	5,223	4,688
1568902_at	4,505	4,307	4,057	4,511	4,231	4,276
1568903_at	4,261	4,243	3,932	4,180	4,473	4,245
1568904_at	5,668	5,423	5,726	6,343	6,362	6,012
1568905_at	5,828	5,886	5,788	6,304	6,205	6,418
1568906_at	4,967	4,962	4,999	5,311	4,930	5,030
1568907_at	6,777	6,845	6,574	6,484	6,407	6,551
1568908_at	4,524	5,035	4,726	5,398	4,954	5,383
1568913_at	5,406	5,558	5,423	6,059	5,674	5,884
1568914_at	5,166	5,274	5,486	5,878	5,372	5,447
1568915_at	7,683	7,765	7,658	8,003	8,124	8,206
1568916_at	5,994	5,771	5,582	5,810	5,802	5,755
1568919_at	3,861	3,976	3,680	4,041	4,112	4,084
1568920_at	5,497	5,244	6,009	4,309	4,319	4,344
1568921_at	5,432	5,452	5,623	5,914	5,654	5,680
1568922_at	5,860	5,879	5,814	6,951	7,190	7,238
1568923_at	5,529	5,768	5,243	6,091	5,680	5,755
1568924_a_at	4,182	4,743	4,475	4,384	4,512	4,438
1568925_at	3,509	3,392	3,563	3,972	4,092	3,934
1568926_x_at	3,948	3,834	3,952	4,415	4,339	4,567
1568929_at	9,091	8,852	8,984	9,247	9,206	9,420
1568930_at	5,309	5,371	5,246	5,269	5,337	5,327
1568931_at	5,886	5,919	5,954	6,141	6,136	6,094
1568932_at	4,992	4,469	4,819	5,053	5,384	5,049
1568933_at	5,483	5,865	5,390	5,721	5,739	5,825
1568934_at	5,622	5,840	5,935	6,386	5,959	6,193
1568935_at	3,543	3,397	3,619	4,105	3,924	3,997
1568936_a_at	4,397	4,180	4,553	4,398	4,334	4,452
1568938_at	5,233	5,398	5,182	5,273	5,316	4,997
1568939_at	3,923	3,900	4,200	4,152	4,067	4,058

1568941_a_at	7,020	6,986	6,802	6,259	6,770	6,292
1568943_at	5,116	5,151	5,283	5,913	5,602	5,889
1568949_at	7,578	7,677	7,570	8,368	8,315	8,467
1568951_at	5,165	5,531	4,960	6,643	6,945	6,983
1568954_s_at	8,380	7,889	8,195	8,301	7,685	8,104
1568955_at	9,809	10,102	9,618	8,242	8,846	8,268
1568957_x_at	7,061	7,593	7,050	6,136	6,605	6,147
1568964_x_at	6,857	7,242	6,775	8,004	8,434	8,317
1568970_at	4,289	4,793	4,490	4,915	5,011	5,318
1568971_at	5,666	5,131	5,326	6,320	6,226	6,500
1568974_at	6,383	6,131	6,399	6,579	6,572	6,710
1568977_at	6,775	6,105	6,595	7,282	7,170	6,958
1568978_s_at	7,306	7,124	7,329	8,006	8,113	8,200
1568981_at	6,556	6,643	7,426	7,210	7,781	7,631
1568983_a_at	9,007	8,641	8,475	5,982	8,282	6,236
1568986_x_at	7,041	6,619	6,984	8,063	8,235	8,362
1568987_at	8,489	8,622	8,422	7,489	7,438	7,645
1568997_at	7,558	6,914	7,454	7,218	7,308	6,990
1568999_at	7,270	7,250	7,181	7,372	7,352	7,757
1569001_at	6,572	6,662	6,868	7,046	7,225	7,018
1569002_x_at	6,650	6,970	7,012	7,230	7,238	7,143
1569003_at	8,437	8,527	8,487	8,559	9,975	9,224
1569004_at	3,931	3,828	4,261	6,021	4,966	4,840
1569005_at	6,106	5,920	5,500	6,203	6,161	6,602
1569006_at	6,538	7,166	6,888	8,780	8,898	8,868
1569008_at	7,506	7,510	7,214	7,063	7,067	6,950
1569009_s_at	6,542	6,639	7,167	8,113	8,089	8,215
1569013_s_at	5,265	5,412	5,435	5,478	5,256	5,550
1569020_at	7,052	6,475	7,046	7,276	7,597	7,938
1569021_at	3,778	3,895	3,637	4,138	4,384	4,036
1569022_a_at	5,802	5,684	6,022	5,921	5,984	6,016
1569023_a_at	7,200	8,125	7,786	4,315	4,152	4,063
1569024_at	7,210	7,298	6,992	6,751	6,602	6,670
1569025_s_at	7,283	7,391	6,978	6,334	5,959	5,839
1569029_at	7,131	6,855	7,225	7,456	7,437	7,463
1569030_s_at	10,582	10,663	10,569	10,068	10,240	10,162
1569032_at	6,462	6,450	6,458	7,140	6,956	7,160
1569033_at	5,764	4,589	5,496	4,916	5,301	5,041
1569034_a_at	5,559	5,306	5,521	5,578	5,524	5,563
1569039_s_at	6,834	6,642	7,061	6,337	5,873	6,343
1569040_s_at	5,761	7,409	4,899	4,155	4,103	4,269
1569041_at	6,035	5,387	6,038	4,992	5,788	4,926
1569042_at	5,682	4,987	5,065	5,759	5,356	5,638
1569044_at	6,020	6,320	6,320	7,135	6,916	6,902
1569048_s_at	7,514	7,363	7,485	7,501	7,287	7,672
1569052_at	5,394	5,561	5,809	5,411	5,505	5,221
1569053_at	8,327	7,837	7,602	7,800	7,830	7,698
1569054_at	5,014	5,230	5,164	5,144	5,177	5,481
1569057_s_at	7,427	7,442	7,227	6,807	6,742	6,374
1569058_at	3,985	4,200	4,195	4,406	4,188	4,188
1569061_at	6,566	6,543	6,085	6,822	6,420	6,380
1569062_s_at	4,666	5,401	5,305	5,644	5,614	5,765
1569063_at	5,149	5,149	5,010	5,212	5,274	5,245
1569064_at	4,346	4,191	4,631	4,641	4,649	5,236
1569065_s_at	7,238	6,704	7,060	8,174	7,973	8,356
1569067_at	6,774	6,627	6,798	6,924	6,708	6,780

1569069_s_at	8,082	8,300	7,565	6,907	7,473	7,073
1569072_s_at	5,331	5,376	5,544	5,585	5,322	5,889
1569073_x_at	8,169	8,214	8,526	9,783	9,897	10,199
1569074_at	7,474	7,110	7,307	7,924	7,884	8,001
1569076_a_at	4,836	4,945	4,980	6,745	6,300	6,825
1569077_x_at	7,827	8,163	8,024	8,263	7,872	8,136
1569078_at	4,331	4,109	4,234	4,839	4,607	4,681
1569080_at	4,189	4,748	4,311	4,205	4,107	4,264
1569086_at	5,309	4,953	5,357	6,658	5,775	6,495
1569089_a_at	4,999	4,324	4,394	4,552	4,538	4,499
1569090_x_at	7,773	7,482	7,618	7,931	7,924	7,985
1569091_at	8,010	7,668	7,887	7,678	7,573	7,576
1569095_at	7,064	6,922	7,267	7,781	7,637	7,873
1569097_at	5,632	5,586	5,695	5,426	5,086	5,347
1569098_s_at	7,291	6,769	7,314	7,192	7,145	7,447
1569099_at	5,432	5,796	5,914	7,720	8,005	8,044
1569100_a_at	4,041	3,964	4,619	4,641	4,547	4,733
1569102_at	5,684	5,770	5,845	6,887	6,604	6,679
1569104_a_at	4,379	4,360	4,572	4,765	4,264	4,460
1569105_at	9,185	9,082	9,276	9,470	9,761	9,756
1569106_s_at	6,103	6,356	5,749	5,492	5,581	5,596
1569107_s_at	6,503	6,710	5,930	5,221	5,459	5,041
1569108_a_at	9,274	9,128	9,329	6,413	6,173	6,435
1569110_x_at	9,972	10,219	10,267	9,481	9,305	8,121
1569111_at	7,355	5,785	6,664	6,288	6,040	5,574
1569112_at	6,400	6,158	6,101	5,050	5,008	5,194
1569114_at	5,658	5,472	5,282	5,642	5,450	5,502
1569121_at	6,879	6,909	6,925	7,260	6,867	7,194
1569122_at	4,641	4,208	4,600	4,652	4,566	4,597
1569124_at	3,646	4,328	3,725	3,612	3,626	3,602
1569126_at	5,223	5,240	5,542	5,430	5,088	5,514
1569127_at	7,088	6,878	7,184	8,038	7,852	7,697
1569128_at	4,311	4,257	4,391	4,000	4,122	4,186
1569129_s_at	8,450	8,273	8,376	5,173	6,522	5,174
1569132_s_at	7,640	7,931	7,997	8,582	8,544	8,621
1569133_x_at	5,959	5,879	5,872	6,343	6,079	6,474
1569136_at	4,801	5,245	4,583	4,377	4,367	4,522
1569138_a_at	6,525	6,653	6,740	7,494	7,236	7,449
1569139_s_at	7,039	6,993	6,846	6,717	6,509	6,864
1569140_at	3,697	3,877	3,930	3,906	3,780	3,922
1569141_a_at	5,389	5,379	5,399	6,047	5,456	5,519
1569142_at	7,577	7,123	6,718	4,445	4,919	4,631
1569144_a_at	7,560	7,431	7,390	7,862	7,725	7,732
1569146_s_at	6,447	5,639	5,926	6,500	6,395	6,485
1569147_at	5,252	5,272	5,272	6,269	6,031	6,315
1569149_at	7,130	6,827	7,111	7,280	7,455	7,580
1569150_x_at	6,913	6,919	7,136	6,828	7,252	7,461
1569153_at	6,004	6,161	6,405	6,483	6,492	6,378
1569154_a_at	8,491	8,096	8,399	9,040	8,664	8,886
1569156_at	4,900	6,074	5,538	5,619	5,501	5,373
1569157_s_at	6,117	7,249	6,102	7,092	6,729	6,617
1569159_at	5,512	5,260	5,416	5,863	5,723	6,055
1569167_at	5,614	5,250	5,156	4,779	5,152	4,977
1569168_at	3,696	3,870	3,867	4,030	4,185	4,237
1569171_a_at	5,077	5,376	5,281	5,460	5,548	5,424
1569172_a_at	7,511	7,469	7,512	8,207	7,879	7,935

1569176_at	4,346	4,405	4,442	4,540	4,567	4,929
1569178_at	3,670	4,198	3,270	3,308	3,215	3,194
1569180_at	7,091	6,057	6,864	3,848	6,841	4,375
1569181_x_at	7,509	6,720	6,937	4,202	7,319	5,111
1569182_at	5,826	5,424	5,895	6,318	6,008	6,099
1569183_a_at	8,253	7,435	7,316	5,903	6,218	5,538
1569186_at	4,107	4,533	4,167	4,198	4,051	4,156
1569188_s_at	6,845	6,862	7,068	7,530	7,364	7,391
1569189_at	5,911	5,228	5,160	5,172	5,299	4,858
1569190_at	8,443	8,975	8,526	7,428	6,816	7,474
1569191_at	9,689	9,467	9,521	7,673	5,810	6,788
1569192_at	3,382	3,260	3,275	3,834	3,497	3,747
1569193_at	4,891	5,099	5,248	5,005	4,952	4,879
1569194_at	6,220	6,379	6,272	5,921	5,757	5,953
1569200_at	9,463	9,590	9,791	10,372	10,272	10,434
1569201_a_at	5,554	4,561	5,306	4,404	4,603	4,211
1569202_x_at	9,249	8,899	8,924	9,274	9,261	9,092
1569203_at	5,461	5,424	5,158	5,481	5,437	5,182
1569205_at	4,642	4,499	4,523	4,746	4,459	4,590
1569206_at	7,981	8,099	8,208	8,969	8,011	8,007
1569207_s_at	6,601	6,688	6,479	6,943	7,121	7,094
1569208_a_at	5,130	4,846	4,960	5,331	5,752	5,794
1569212_at	7,233	7,232	7,304	8,107	8,101	8,213
1569213_at	3,856	4,167	4,209	4,013	4,141	4,100
1569218_at	3,953	3,910	4,128	4,195	4,063	4,183
1569219_at	5,826	5,936	5,954	6,411	6,135	6,009
1569225_a_at	5,187	5,142	4,789	5,181	5,062	4,988
1569230_at	4,457	4,405	4,493	4,652	4,606	4,601
1569231_x_at	4,298	4,296	4,451	4,367	4,335	4,430
1569234_at	7,055	6,841	6,633	7,274	7,564	7,681
1569235_a_at	6,209	6,008	6,255	6,448	6,235	6,409
1569237_at	4,674	4,815	4,706	4,692	4,833	4,767
1569238_a_at	5,686	5,392	5,507	4,923	5,264	4,986
1569240_at	4,845	5,260	4,765	4,424	4,473	4,401
1569241_a_at	7,588	8,543	7,299	5,911	5,416	6,004
1569243_at	6,260	6,160	5,671	6,384	6,409	5,995
1569245_at	6,002	6,049	6,083	6,814	7,024	7,277
1569246_a_at	7,119	7,005	6,852	7,611	7,671	7,692
1569250_at	7,687	7,367	7,288	8,033	8,058	8,250
1569251_a_at	6,821	6,420	6,251	5,808	6,248	6,319
1569253_at	4,022	3,900	4,427	4,667	4,153	4,491
1569254_s_at	3,548	3,617	3,766	3,666	3,557	3,682
1569256_a_at	7,017	7,069	7,129	7,503	7,135	6,956
1569257_at	5,214	4,878	5,135	5,551	5,224	5,530
1569259_at	4,566	4,899	4,519	4,743	4,921	4,950
1569262_x_at	3,593	3,720	3,689	3,555	3,837	3,617
1569263_at	5,692	5,634	5,356	6,071	6,374	6,919
1569264_at	6,745	6,104	6,648	6,809	6,532	6,452
1569268_at	6,542	6,834	6,855	7,096	6,931	7,295
1569269_s_at	6,325	6,049	6,074	7,293	7,110	7,343
1569270_at	6,213	6,305	6,292	6,646	6,637	6,490
1569272_at	5,017	5,097	5,228	5,306	5,227	5,428
1569274_at	7,535	7,458	7,853	8,033	8,125	8,064
1569275_at	5,247	5,097	5,384	5,187	5,098	5,295
1569276_at	7,264	6,986	7,411	7,659	7,685	7,875
1569277_at	3,546	3,609	3,545	3,528	3,464	3,410

1569283_at	7,380	7,802	6,826	5,181	4,576	5,083
1569287_at	4,988	4,902	5,118	5,035	4,855	5,030
1569289_at	6,213	6,224	6,095	6,214	5,874	6,030
1569290_s_at	6,758	6,767	6,825	5,159	5,016	4,952
1569291_at	8,004	7,795	8,002	8,142	8,291	8,438
1569293_x_at	7,791	7,877	7,932	8,387	8,372	8,499
1569294_at	7,975	7,706	7,754	8,316	8,513	8,523
1569296_a_at	6,748	6,812	7,059	7,068	6,842	7,118
1569297_at	5,538	5,409	5,310	5,875	5,624	5,985
1569300_at	7,450	7,584	7,701	7,974	7,914	7,597
1569302_at	7,511	7,480	7,655	7,760	8,081	7,863
1569303_s_at	6,794	6,772	6,844	8,131	7,575	7,811
1569305_a_at	7,092	7,174	7,593	7,456	7,380	7,792
1569306_at	4,488	4,427	4,696	4,849	4,995	5,216
1569307_s_at	4,594	4,798	4,323	4,666	4,797	4,611
1569310_at	6,302	6,110	6,210	6,943	6,803	6,935
1569311_at	4,844	4,252	4,584	4,339	4,257	4,330
1569312_at	7,294	6,324	6,527	4,920	4,717	5,051
1569315_s_at	5,368	5,542	5,305	6,104	5,941	6,237
1569316_at	6,851	6,605	6,458	4,171	4,217	4,225
1569318_at	6,152	5,809	6,210	6,953	6,550	6,710
1569320_at	7,522	7,226	7,232	4,428	6,047	5,046
1569321_at	6,649	6,545	6,757	6,675	6,716	6,735
1569322_at	6,451	6,620	6,552	7,303	7,291	7,494
1569323_at	5,325	4,982	5,213	3,856	4,011	3,931
1569325_at	7,234	7,415	7,697	7,795	7,660	7,441
1569330_at	6,326	6,789	6,967	5,629	5,593	5,634
1569331_at	3,743	3,901	3,977	3,983	3,892	4,032
1569332_at	4,457	4,489	4,391	4,459	4,332	4,478
1569334_at	5,527	4,788	5,636	5,646	5,641	5,868
1569335_a_at	8,048	7,703	8,429	7,411	7,227	7,479
1569337_at	6,324	6,436	6,589	6,847	6,878	6,795
1569338_at	5,664	5,847	5,693	6,124	5,431	6,057
1569339_s_at	6,603	6,964	6,801	7,314	7,123	7,353
1569341_at	5,842	6,387	6,684	6,802	7,268	7,113
1569342_at	7,093	7,294	7,413	6,875	6,574	6,744
1569344_a_at	4,378	4,723	4,705	4,553	4,635	4,682
1569345_at	6,644	7,214	7,568	6,917	7,114	6,951
1569346_a_at	6,044	6,114	6,515	6,951	6,547	6,690
1569348_at	4,598	4,451	4,724	4,744	4,760	4,539
1569349_at	5,660	5,558	5,840	5,546	4,798	5,160
1569351_at	6,421	6,124	6,447	6,266	6,607	6,497
1569352_at	4,337	4,011	3,968	4,437	4,594	4,521
1569353_at	6,971	7,304	7,061	6,214	6,053	6,162
1569354_at	4,906	4,540	4,775	4,609	4,885	4,616
1569355_at	5,677	5,331	5,870	6,558	6,116	6,444
1569361_a_at	7,852	7,447	7,753	8,183	7,978	8,348
1569362_at	4,561	4,937	4,859	4,810	5,902	6,541
1569365_a_at	7,199	7,122	7,255	8,415	8,418	8,777
1569366_a_at	7,330	7,025	7,257	6,625	6,099	6,629
1569368_at	6,624	6,868	6,672	7,252	6,615	7,463
1569369_at	5,506	5,617	5,522	6,123	5,908	6,068
1569371_at	5,692	5,360	5,470	6,188	5,775	5,836
1569372_at	5,713	5,719	5,574	5,671	5,507	5,441
1569374_at	6,424	6,196	6,168	6,293	6,162	6,451
1569375_at	3,555	3,431	3,378	3,944	3,958	3,882

1569376_s_at	6,027	6,129	5,949	6,525	6,318	6,529
1569377_at	4,627	4,377	4,611	3,689	3,747	3,766
1569378_at	6,793	6,586	6,501	8,094	7,561	7,671
1569380_a_at	7,318	7,397	7,269	8,102	7,878	8,173
1569383_s_at	6,166	6,001	6,044	6,291	6,384	6,516
1569385_s_at	6,101	6,088	6,328	6,918	6,852	6,955
1569386_at	7,433	7,355	6,979	7,485	7,280	7,642
1569387_at	6,254	6,418	6,358	6,705	6,827	6,347
1569391_at	4,167	4,405	4,399	4,466	4,544	4,423
1569392_at	5,938	5,834	5,866	5,950	5,775	5,910
1569393_at	6,952	6,813	7,040	7,286	7,188	7,314
1569396_at	8,798	8,751	8,153	8,926	8,856	9,249
1569399_at	5,193	5,216	5,571	5,907	5,648	5,894
1569401_at	5,327	5,297	5,626	5,759	5,331	5,462
1569402_at	7,408	7,485	7,351	8,082	8,165	8,276
1569403_at	4,280	4,341	4,410	4,512	4,407	4,546
1569405_at	4,426	4,373	4,484	4,959	4,699	5,121
1569406_at	4,694	4,820	4,394	4,903	4,902	5,178
1569407_at	3,881	3,889	3,966	3,972	3,812	3,955
1569408_at	4,345	4,217	4,442	4,088	4,125	4,117
1569409_x_at	8,965	8,624	8,771	8,241	9,181	8,296
1569410_at	4,269	4,649	4,555	4,425	4,594	4,728
1569411_at	3,725	3,913	4,048	4,071	3,767	3,916
1569415_at	8,324	8,260	8,213	8,787	8,640	8,621
1569416_at	7,230	6,629	6,798	7,645	7,806	7,827
1569417_at	6,111	6,022	5,841	6,099	6,141	6,226
1569418_at	5,534	5,307	5,390	5,611	5,533	5,387
1569419_at	6,509	6,472	6,516	7,091	6,684	6,984
1569421_at	6,849	6,513	6,771	7,232	6,941	6,970
1569422_at	6,351	6,049	6,042	7,205	7,231	7,693
1569426_at	7,234	7,242	7,206	7,673	7,556	7,606
1569428_at	6,913	6,894	7,203	6,935	6,906	7,158
1569430_at	5,473	5,533	5,844	6,316	6,052	6,397
1569431_at	5,085	5,481	5,001	5,420	5,604	5,627
1569433_at	3,806	3,649	3,893	4,125	4,119	4,023
1569434_at	6,729	6,760	6,825	7,406	7,376	7,391
1569436_at	5,486	5,244	5,457	7,122	7,221	7,247
1569439_at	4,830	4,470	5,340	5,706	5,213	4,958
1569443_s_at	5,764	6,008	5,520	5,678	5,481	5,662
1569444_at	7,369	7,155	7,350	7,839	7,726	7,737
1569445_at	6,313	6,407	6,875	6,921	6,930	6,927
1569446_x_at	6,962	6,985	6,935	7,087	7,093	7,095
1569448_at	6,665	7,092	7,205	7,559	7,378	7,705
1569449_a_at	5,894	5,425	5,737	5,989	5,764	5,890
1569450_at	4,976	4,796	4,919	4,438	5,193	4,486
1569452_at	6,037	6,049	5,892	6,472	6,316	6,401
1569453_a_at	6,436	6,910	6,273	6,536	6,419	6,708
1569454_a_at	6,393	6,493	6,578	6,657	6,789	6,755
1569459_a_at	4,865	4,731	5,227	4,926	4,828	4,971
1569461_at	6,164	6,213	6,614	7,513	7,406	7,477
1569462_x_at	6,157	6,176	6,069	7,218	7,074	7,250
1569464_at	7,101	7,262	7,038	6,865	7,541	7,683
1569465_at	6,676	6,753	6,441	7,406	7,210	7,627
1569466_at	5,904	5,741	5,728	6,615	6,583	6,539
1569468_at	3,956	4,019	3,994	3,883	3,612	3,641
1569469_a_at	5,082	6,082	5,238	3,963	3,886	3,949

1569470_a_at	7,231	7,616	7,297	7,255	6,881	7,438
1569472_s_at	6,920	6,391	5,849	4,883	4,881	5,308
1569473_s_at	7,455	7,443	7,153	7,661	7,053	7,610
1569474_at	5,181	5,270	5,014	4,752	4,867	4,926
1569476_at	5,454	5,557	5,533	4,448	4,169	4,363
1569477_at	6,135	6,530	6,735	5,469	6,785	5,559
1569478_s_at	7,966	7,961	8,156	9,366	7,933	8,374
1569479_at	5,922	5,693	6,059	5,553	5,247	5,249
1569481_s_at	6,657	6,465	6,913	7,281	7,181	7,022
1569482_at	4,529	4,929	4,742	4,645	4,473	4,520
1569484_s_at	7,800	7,811	8,653	3,625	5,359	3,830
1569486_at	4,242	4,183	4,304	4,327	4,195	4,317
1569487_at	7,085	7,106	7,130	7,835	7,361	7,759
1569490_at	4,592	4,609	4,479	4,806	5,231	5,080
1569491_at	4,306	4,388	4,555	4,274	4,336	4,334
1569492_at	4,654	4,439	4,567	4,574	4,666	4,662
1569494_at	3,342	3,723	3,554	4,016	3,686	3,930
1569495_at	5,139	5,671	5,849	5,301	5,162	5,083
1569496_s_at	7,609	7,814	8,104	8,395	8,293	8,703
1569499_at	7,272	6,886	7,348	7,473	7,515	7,523
1569500_at	6,064	5,692	5,503	5,281	5,179	5,477
1569501_at	7,649	7,532	7,741	7,521	7,631	7,796
1569502_s_at	7,394	7,806	7,633	8,538	8,473	8,691
1569503_at	4,196	4,494	4,279	4,484	4,813	4,444
1569504_at	4,339	4,257	4,117	4,496	4,705	4,873
1569508_at	4,909	5,052	5,040	5,801	5,648	5,593
1569510_at	5,627	5,461	5,693	5,714	5,790	5,738
1569512_at	5,459	5,868	5,700	4,773	6,095	5,015
1569515_a_at	7,512	7,605	7,778	8,013	8,160	8,576
1569516_at	6,343	6,374	6,664	6,941	6,700	6,844
1569517_at	7,119	6,808	7,155	7,517	7,317	7,265
1569518_at	5,657	5,598	5,579	6,785	6,229	6,353
1569519_at	6,506	5,802	5,990	5,613	6,431	5,718
1569520_at	5,556	5,640	5,599	6,733	6,083	6,258
1569521_s_at	6,534	6,805	6,903	7,567	7,067	7,238
1569522_at	9,213	8,972	9,099	10,360	9,580	9,735
1569523_a_at	6,030	6,113	6,138	6,316	6,157	6,308
1569525_s_at	5,957	5,654	5,363	6,474	6,542	6,834
1569527_at	6,796	6,873	6,819	5,809	5,911	5,880
1569528_at	4,271	4,466	4,567	4,120	4,204	4,250
1569532_a_at	7,537	7,758	7,820	8,271	7,954	8,139
1569536_at	7,612	7,514	7,580	8,656	8,409	8,324
1569537_at	6,333	6,055	6,277	6,583	6,723	6,977
1569538_at	5,061	4,941	5,230	5,724	5,348	5,505
1569539_at	5,113	4,465	4,511	3,950	4,107	4,102
1569540_at	6,533	5,521	6,249	5,858	6,030	6,190
1569542_at	6,459	5,539	5,860	4,450	4,588	4,612
1569543_at	4,760	4,475	4,687	5,838	6,141	6,216
1569544_at	4,197	4,111	4,137	4,841	4,833	4,948
1569545_at	3,844	3,719	3,561	4,046	4,124	4,267
1569551_at	4,577	4,540	4,512	4,766	4,564	4,565
1569552_at	3,608	3,761	3,737	3,797	3,872	4,267
1569554_at	4,098	4,100	4,287	4,075	4,021	4,182
1569555_at	6,408	5,765	6,198	6,678	6,870	6,742
1569557_at	6,582	6,761	6,484	6,226	6,445	6,434
1569558_at	4,926	5,161	5,383	6,265	6,348	6,823

1569560_at	3,797	3,494	3,726	4,027	3,977	4,111
1569561_at	5,944	5,885	5,855	6,418	6,203	6,707
1569564_at	5,658	5,901	5,900	6,133	5,979	5,990
1569566_at	4,498	4,807	4,561	4,728	4,690	4,703
1569568_at	3,493	3,586	3,535	3,522	3,558	3,664
1569569_x_at	4,499	4,959	5,314	5,216	5,141	5,303
1569570_at	5,059	5,007	4,889	5,267	5,064	5,126
1569571_at	5,229	5,352	5,425	5,272	5,091	5,167
1569573_at	5,820	5,847	5,818	5,643	5,818	5,708
1569577_x_at	3,844	3,904	3,860	5,025	5,519	5,636
1569578_at	7,009	6,529	6,654	7,096	7,262	7,368
1569579_at	7,081	6,821	7,112	7,389	7,351	7,544
1569580_a_at	5,832	5,581	5,729	6,239	6,013	5,967
1569582_at	4,345	4,125	4,678	4,574	4,440	4,484
1569583_at	5,719	5,759	5,665	6,175	7,138	6,362
1569585_at	4,273	4,519	4,619	5,324	5,018	5,424
1569587_at	3,747	3,734	3,752	3,733	3,836	3,724
1569588_x_at	7,511	7,640	7,925	8,332	8,049	7,858
1569590_at	6,858	6,972	6,925	7,371	7,357	7,265
1569591_at	3,301	3,574	3,534	3,394	3,298	3,331
1569592_a_at	3,635	3,354	3,516	3,721	3,564	3,624
1569594_a_at	9,579	9,594	9,051	7,579	8,899	6,929
1569596_at	4,333	3,851	3,915	4,595	4,538	4,685
1569597_at	7,157	6,892	7,132	7,388	7,442	7,599
1569599_at	3,441	3,509	3,487	3,495	3,498	3,662
1569600_at	4,601	3,791	4,088	3,500	3,646	3,411
1569601_at	5,747	5,056	5,400	5,925	6,180	5,840
1569602_at	5,627	5,631	5,832	5,978	6,467	6,185
1569603_at	5,650	5,653	5,580	5,913	6,210	5,918
1569604_at	7,656	7,911	7,791	8,486	8,434	8,551
1569607_s_at	8,267	8,211	8,104	6,858	7,531	6,246
1569608_x_at	6,688	6,831	6,544	6,655	6,534	6,536
1569609_at	4,164	3,934	4,081	4,438	4,436	4,466
1569610_at	4,341	4,439	4,724	5,171	5,187	5,236
1569611_a_at	4,175	4,843	4,208	4,302	4,227	4,446
1569614_s_at	6,041	6,407	6,607	6,722	6,886	6,944
1569615_at	7,734	7,421	7,507	8,197	7,619	7,644
1569616_at	5,530	5,759	5,872	5,918	5,756	5,786
1569617_at	4,876	4,500	4,724	6,316	6,260	6,312
1569620_s_at	5,301	5,531	4,682	5,615	5,442	5,495
1569624_at	5,237	4,686	5,465	5,887	6,178	5,681
1569629_x_at	7,135	6,454	6,744	7,202	6,642	7,017
1569630_a_at	4,333	4,355	4,291	4,340	4,433	4,279
1569631_at	6,848	6,865	6,948	8,177	7,890	8,048
1569634_at	7,594	7,726	7,656	7,777	7,636	7,730
1569637_at	4,350	4,456	4,513	4,789	4,649	4,895
1569638_at	5,528	5,235	5,224	5,606	5,369	5,354
1569640_s_at	7,222	6,915	7,045	7,140	6,899	7,128
1569641_at	3,774	3,679	3,944	4,166	4,553	4,665
1569642_at	7,885	8,054	7,971	8,281	8,426	8,204
1569644_at	6,262	6,129	5,871	6,247	5,834	6,038
1569646_a_at	5,266	5,251	5,351	5,968	5,796	5,748
1569647_at	6,161	5,932	5,605	6,148	6,064	6,181
1569648_at	7,712	8,037	7,672	8,015	7,969	8,277
1569652_at	6,168	6,463	6,436	6,111	6,752	6,015
1569656_at	4,875	4,318	4,690	4,669	4,628	4,798

1569659_at	5,542	5,667	5,801	5,609	5,402	5,427
1569660_at	6,146	6,006	6,248	7,153	7,158	7,076
1569661_at	4,119	3,684	3,676	3,656	3,764	3,623
1569663_at	3,370	3,428	3,703	3,492	3,515	3,612
1569664_at	4,617	4,509	4,570	4,443	4,521	4,474
1569665_at	5,662	5,836	5,636	6,645	6,107	6,333
1569666_s_at	6,263	6,168	6,351	5,033	5,563	4,989
1569668_at	6,569	6,541	6,535	6,325	6,415	6,469
1569669_at	7,766	7,658	6,274	5,852	5,761	5,840
1569670_at	5,493	5,934	5,911	6,258	5,577	6,187
1569672_at	5,041	5,081	5,023	5,108	5,128	5,065
1569673_at	4,813	4,553	4,764	4,937	4,834	5,074
1569674_at	4,233	4,410	4,553	4,241	4,059	4,402
1569675_at	4,256	4,311	4,428	4,115	4,309	4,310
1569676_at	5,555	5,292	5,429	5,373	5,305	5,503
1569677_a_at	4,491	4,520	4,489	4,891	4,974	5,260
1569679_at	8,676	8,654	8,827	8,971	8,843	9,161
1569680_at	7,215	7,302	7,403	7,612	7,708	7,713
1569681_at	6,065	6,112	5,796	5,613	5,394	5,561
1569683_at	6,123	5,895	6,137	6,636	6,611	6,608
1569685_at	6,730	6,232	5,900	5,423	5,599	5,417
1569688_at	4,792	4,706	4,866	5,209	5,102	5,103
1569689_s_at	5,376	5,258	5,129	6,125	5,987	6,126
1569690_at	5,711	6,059	6,164	6,035	5,883	6,071
1569693_at	4,304	4,694	4,529	4,434	4,175	4,416
1569696_at	7,069	6,890	7,022	8,017	7,606	7,671
1569698_s_at	4,993	4,858	4,794	5,426	5,292	5,310
1569699_at	5,235	5,708	5,343	5,765	5,274	5,623
1569700_s_at	7,394	7,463	7,440	7,693	7,349	7,663
1569701_at	5,010	5,491	5,645	5,030	4,886	5,181
1569703_a_at	4,993	4,995	5,174	5,194	5,806	5,364
1569706_at	4,256	3,842	4,144	3,513	3,604	3,461
1569709_at	6,175	5,876	6,252	6,828	6,265	6,493
1569712_at	3,497	3,662	3,726	3,750	3,890	3,861
1569713_at	7,840	7,604	7,616	6,281	6,014	6,130
1569714_at	6,568	6,459	6,459	7,213	6,986	7,417
1569716_at	4,497	4,802	4,494	4,768	4,787	4,795
1569719_at	3,873	3,861	3,841	4,047	4,018	4,035
1569721_at	4,178	4,533	4,281	4,609	4,551	4,703
1569722_s_at	6,224	6,342	6,328	6,700	5,929	6,195
1569723_a_at	6,175	6,111	6,009	6,769	6,447	6,399
1569724_at	6,693	6,839	6,602	7,223	6,973	7,034
1569727_at	6,321	6,282	6,343	6,772	6,518	6,696
1569728_at	5,361	5,488	5,406	5,923	5,743	6,094
1569729_a_at	3,573	3,414	3,653	3,706	3,603	3,589
1569730_at	6,408	6,329	6,533	7,519	6,623	7,008
1569731_at	4,749	4,944	4,721	5,032	5,151	5,389
1569732_at	7,274	7,449	7,429	7,922	7,887	8,110
1569736_at	5,658	5,857	6,176	6,223	5,683	5,898
1569737_a_at	6,624	6,579	6,415	7,034	6,958	7,399
1569738_at	7,018	6,878	6,881	7,138	6,828	6,928
1569739_at	5,657	5,592	5,698	6,369	6,171	6,341
1569740_at	4,320	4,074	4,043	5,146	5,072	5,380
1569741_at	5,665	5,503	5,749	6,284	6,463	6,459
1569743_at	6,082	6,311	6,301	7,470	6,477	6,718
1569745_at	7,827	7,822	7,681	8,262	8,176	8,282

1569746_s_at	6,516	6,001	6,246	6,252	6,087	6,189
1569748_at	5,965	5,879	5,993	6,442	6,474	6,591
1569750_at	5,845	6,033	6,014	6,663	6,818	6,835
1569751_at	6,896	7,258	6,682	7,007	6,732	7,012
1569753_at	4,495	4,338	4,509	4,575	4,428	4,436
1569755_at	4,480	4,640	4,525	4,971	4,903	4,980
1569756_at	4,243	4,238	4,230	5,416	4,868	5,157
1569758_at	5,339	5,132	5,365	6,115	5,989	6,446
1569759_at	5,219	4,917	5,200	5,517	5,351	5,222
1569760_at	3,544	3,570	3,577	3,780	3,609	3,620
1569761_x_at	3,916	3,965	4,201	4,003	4,052	4,066
1569763_at	7,178	7,251	7,212	7,755	7,368	7,497
1569764_at	5,757	5,631	5,797	6,580	6,501	6,118
1569765_at	7,656	7,790	7,853	8,530	8,457	8,661
1569767_at	6,900	6,563	7,098	7,644	7,313	7,252
1569768_at	3,732	3,684	3,697	3,941	3,873	3,927
1569770_x_at	3,990	3,723	3,849	4,156	4,037	4,083
1569772_x_at	3,618	3,761	3,676	3,527	3,555	3,535
1569773_at	4,779	4,815	4,162	4,993	4,787	4,924
1569775_at	7,210	7,391	7,587	8,005	7,944	8,032
1569776_at	6,443	5,902	6,655	7,145	7,116	6,832
1569777_a_at	4,247	4,220	4,311	5,015	4,978	5,428
1569779_at	4,119	3,632	3,916	4,191	3,969	3,997
1569780_at	4,979	4,976	5,017	6,420	6,249	6,494
1569781_at	5,252	5,206	5,525	5,383	5,297	5,304
1569782_at	5,047	4,958	5,068	5,330	5,378	5,312
1569783_at	3,937	4,318	4,081	4,334	4,506	4,241
1569784_at	4,047	4,135	3,983	4,349	4,258	4,284
1569785_at	5,552	5,089	5,166	5,162	5,018	5,188
1569786_at	3,924	3,900	4,388	4,327	4,237	4,470
1569787_at	6,203	6,104	6,307	6,376	6,302	6,622
1569788_at	6,401	6,429	6,427	4,031	3,960	3,957
1569789_at	5,422	5,670	5,670	5,862	5,797	5,816
1569790_at	3,992	3,774	4,055	4,173	4,263	4,005
1569791_at	5,694	5,666	5,612	5,938	5,637	5,817
1569792_a_at	6,155	7,048	6,388	6,123	5,823	5,712
1569793_at	7,508	7,587	7,370	8,109	8,192	8,164
1569794_at	3,676	4,058	3,969	3,868	3,949	3,909
1569796_s_at	7,975	8,026	7,912	6,667	6,488	6,543
1569802_at	5,726	5,938	6,249	6,489	6,436	6,588
1569805_at	6,941	6,641	6,847	8,141	8,132	8,351
1569806_at	6,432	6,092	6,414	6,831	7,055	7,324
1569807_at	7,302	7,626	7,744	8,018	7,897	7,983
1569808_at	4,038	3,943	4,166	4,454	4,448	4,789
1569809_at	4,195	4,405	4,007	4,350	4,374	4,363
1569810_at	3,363	3,469	3,519	3,425	3,459	3,602
1569811_at	4,469	4,592	4,755	4,781	4,959	5,077
1569812_at	4,813	4,840	4,501	5,400	5,218	5,510
1569813_at	4,524	4,956	4,986	4,389	4,370	4,037
1569815_x_at	4,986	4,904	4,779	4,472	4,620	4,442
1569816_at	5,884	6,214	6,249	7,018	7,044	7,364
1569817_at	4,457	4,941	5,068	5,570	5,400	5,473
1569818_at	3,491	3,534	3,589	3,499	3,405	3,667
1569819_at	4,963	5,007	5,083	5,590	4,992	5,643
1569823_at	3,806	3,944	3,842	3,754	3,675	3,761
1569824_at	5,661	5,879	5,735	6,258	6,169	6,445

1569826_at	5,386	5,454	5,388	5,359	5,313	5,418
1569827_at	6,332	5,837	6,915	6,868	6,401	6,286
1569828_at	5,772	5,675	6,106	5,941	6,053	6,382
1569830_at	3,768	4,074	3,861	3,936	3,849	3,850
1569831_at	3,647	4,131	4,237	4,963	4,898	4,927
1569832_at	5,617	6,059	5,897	6,605	6,404	6,524
1569833_at	5,592	5,873	5,741	6,303	5,980	6,228
1569834_at	7,755	7,544	7,944	7,665	7,748	7,825
1569835_at	4,028	4,238	3,757	4,777	4,732	4,916
1569838_at	7,136	6,943	7,164	7,859	7,328	7,415
1569839_s_at	6,290	6,137	5,903	6,780	6,771	7,073
1569840_at	5,415	5,062	4,919	5,395	5,147	5,394
1569841_x_at	5,764	5,673	5,518	5,934	5,987	5,795
1569842_at	5,562	5,804	5,990	6,451	6,326	6,470
1569843_at	4,504	4,303	4,268	5,654	5,161	5,809
1569846_at	5,618	5,784	5,591	6,059	5,876	6,014
1569847_at	6,406	6,271	6,420	6,003	6,104	6,004
1569849_at	5,739	5,837	5,313	6,104	5,752	6,056
1569850_at	4,038	4,181	4,079	4,159	4,061	4,414
1569852_at	6,694	6,454	6,812	7,006	7,052	6,964
1569853_at	5,131	5,241	5,384	5,257	5,244	5,284
1569854_at	4,196	4,584	3,884	3,944	3,765	3,830
1569855_at	4,635	4,974	4,704	5,658	5,572	5,761
1569856_at	5,109	5,041	5,336	5,262	5,082	5,556
1569857_s_at	6,911	6,692	6,648	6,818	6,605	7,113
1569858_at	4,394	4,143	4,224	4,949	4,845	5,254
1569859_at	5,154	5,157	5,621	5,845	5,778	6,049
1569861_at	4,111	4,229	3,817	4,319	4,078	3,944
1569864_at	4,588	5,238	4,387	3,993	4,191	4,155
1569867_at	6,316	6,063	6,678	6,485	6,703	6,599
1569868_s_at	7,458	7,126	7,755	6,830	8,004	8,203
1569871_at	8,328	8,476	8,329	8,562	8,852	8,859
1569872_a_at	7,054	7,077	7,043	6,133	6,093	6,485
1569873_at	3,331	3,386	3,445	3,584	3,515	3,589
1569874_s_at	4,353	4,490	4,845	4,363	4,551	4,442
1569876_at	5,127	4,729	4,960	6,029	6,014	6,034
1569878_at	5,598	5,563	5,874	5,367	5,500	5,470
1569879_a_at	7,228	7,191	7,422	8,066	7,923	8,227
1569881_at	5,027	5,413	5,209	5,624	5,571	5,542
1569882_at	6,052	5,894	5,894	6,107	5,829	5,968
1569885_at	5,763	5,435	5,546	5,743	5,686	5,547
1569886_a_at	6,659	5,917	7,005	3,707	3,638	3,708
1569887_a_at	7,034	7,140	6,813	7,384	7,247	7,353
1569891_at	6,429	6,102	6,386	7,400	7,595	7,679
1569892_at	4,422	4,618	4,514	4,887	4,924	4,747
1569894_at	4,946	5,141	5,008	4,857	4,670	4,818
1569895_at	8,256	8,205	8,370	8,932	8,690	9,110
1569898_a_at	6,401	6,479	6,415	7,167	6,865	6,994
1569900_at	6,243	5,922	6,157	6,765	6,561	6,658
1569904_x_at	4,353	4,953	4,872	7,064	7,289	7,454
1569905_at	7,114	7,096	7,227	7,171	7,304	7,369
1569906_s_at	6,805	6,659	6,449	7,073	6,856	7,160
1569909_at	6,318	6,202	6,096	6,706	6,468	6,711
1569910_at	5,129	4,870	4,657	5,673	5,378	5,662
1569911_at	6,495	6,370	6,325	6,697	6,492	6,324
1569912_at	6,598	6,669	6,654	7,300	7,019	6,808

1569915_at	6,597	6,285	6,606	6,875	6,870	6,914
1569916_at	4,242	4,500	4,284	4,416	4,930	4,560
1569917_at	4,682	4,745	4,801	5,133	5,186	5,229
1569921_at	4,873	4,578	4,805	4,616	4,817	4,572
1569923_s_at	6,447	6,550	6,595	5,656	5,368	5,539
1569925_at	6,805	6,616	6,710	6,930	6,917	6,827
1569926_s_at	6,198	5,954	5,994	6,072	6,046	6,132
1569927_at	4,593	4,278	4,694	4,507	4,647	4,871
1569929_at	3,801	3,747	3,872	3,995	4,034	3,920
1569930_at	5,735	5,684	5,498	5,832	5,697	5,466
1569931_at	5,247	4,699	4,984	5,578	5,395	5,505
1569932_at	6,987	7,015	7,137	7,354	6,773	7,087
1569933_at	8,330	8,045	8,134	8,481	8,428	8,511
1569934_at	3,861	3,927	4,082	3,353	3,376	3,472
1569935_at	7,055	7,465	7,577	7,924	8,071	8,073
1569936_a_at	5,067	5,015	5,109	5,527	5,529	5,472
1569937_at	6,032	6,100	5,953	6,386	6,310	6,328
1569938_at	4,264	4,263	4,172	4,244	4,244	4,198
1569940_at	8,828	7,747	8,301	5,586	5,808	5,662
1569941_at	3,641	3,647	3,636	3,819	3,802	3,872
1569942_at	3,892	3,856	3,931	4,230	3,997	4,301
1569944_at	5,445	5,092	5,260	5,555	5,254	5,815
1569947_at	4,736	5,064	5,472	5,958	5,923	6,015
1569948_at	3,728	3,492	3,936	3,807	3,964	3,983
1569949_at	8,705	8,529	8,350	8,988	8,869	8,816
1569952_x_at	7,583	7,545	6,790	7,200	7,442	7,164
1569953_at	5,478	5,693	5,811	6,300	6,047	6,193
1569954_at	5,442	5,507	5,518	6,379	6,092	6,145
1569955_at	3,769	3,858	3,849	3,632	3,588	3,698
1569956_at	3,689	3,722	3,714	3,574	3,729	3,656
1569958_at	4,260	4,118	4,409	4,818	4,500	4,772
1569959_at	5,715	5,972	6,234	6,493	6,397	6,564
1569960_at	4,605	4,708	4,788	4,755	4,672	4,709
1569961_at	6,946	7,190	7,247	7,402	7,519	7,789
1569962_at	6,075	5,969	5,836	6,560	6,426	6,406
1569963_at	6,873	7,200	7,617	8,118	8,156	8,442
1569969_a_at	3,874	3,743	4,021	4,169	4,001	4,244
1569971_at	4,725	4,422	4,801	5,006	4,756	4,937
1569973_at	9,053	9,124	8,059	7,662	7,625	7,778
1569974_x_at	7,723	8,739	7,720	7,445	7,616	7,700
1569975_at	4,955	4,952	5,108	5,254	5,536	5,270
1569978_x_at	6,794	6,662	6,828	7,538	7,054	7,516
1569979_at	5,575	5,759	5,903	6,081	5,793	5,999
1569980_x_at	5,290	5,459	5,473	5,887	5,571	5,765
1569981_at	3,811	4,102	3,912	3,997	4,036	4,076
1569983_at	4,053	4,075	4,314	4,833	4,669	4,930
1569986_x_at	7,546	7,664	7,395	7,711	7,794	8,137
1569987_at	5,631	5,833	5,713	6,317	6,158	6,646
1569988_at	3,735	4,236	4,176	4,268	4,403	4,613
1569990_at	6,429	5,933	6,481	6,839	6,798	6,489
1569991_at	6,951	6,426	6,735	6,919	6,657	7,039
1569995_at	4,103	3,970	4,305	4,094	3,912	4,090
1569996_at	6,118	6,247	5,982	6,619	6,730	6,343
1569998_at	5,411	5,432	5,511	5,766	5,644	5,588
1569999_at	6,786	6,592	6,538	6,198	6,571	6,484
1570001_at	4,803	4,886	4,198	3,964	3,968	4,168

1570005_at	4,963	5,057	5,246	5,237	5,478	5,360
1570006_at	5,563	5,249	5,581	5,745	5,469	5,524
1570007_at	6,749	6,796	6,851	7,364	6,686	6,907
1570008_at	4,948	5,012	5,324	5,705	5,427	5,519
1570009_at	3,880	4,140	3,735	3,885	3,983	4,065
1570013_at	6,188	6,169	6,282	6,838	7,029	6,982
1570014_at	6,001	5,925	6,101	6,309	6,088	6,125
1570015_at	4,833	4,834	4,718	6,221	6,047	6,410
1570020_at	5,211	4,875	5,263	5,565	6,001	6,005
1570021_at	4,498	4,333	4,203	4,408	4,799	4,096
1570022_at	3,640	3,657	3,662	3,711	3,537	3,592
1570023_at	3,896	3,900	3,942	4,499	4,227	4,070
1570024_at	4,291	4,348	4,367	5,000	4,457	4,841
1570025_at	7,164	7,252	7,236	7,955	7,816	7,837
1570026_at	7,052	6,955	6,973	7,260	7,153	7,414
1570030_at	4,437	4,534	4,518	4,749	4,754	5,206
1570031_at	4,498	4,819	4,194	4,500	4,499	4,584
1570032_at	5,843	5,493	5,949	6,076	5,754	5,832
1570033_at	5,653	5,309	5,710	5,905	6,027	5,906
1570035_at	5,511	5,553	5,390	7,071	6,775	7,229
1570038_at	5,003	5,197	4,912	4,758	4,740	4,521
1570039_at	4,951	4,740	5,558	4,475	4,329	4,495
1570040_at	6,081	6,375	6,142	6,954	6,649	7,021
1570042_a_at	6,152	6,549	5,960	7,294	7,662	7,150
1570043_at	4,091	4,645	4,595	4,984	5,061	5,387
1570044_at	5,375	5,585	5,640	5,837	6,001	6,100
1570046_at	3,585	3,842	3,768	3,710	3,648	3,757
1570048_at	5,976	5,602	5,439	5,813	5,797	5,946
1570049_at	5,451	5,369	5,389	5,487	5,323	5,504
1570050_at	4,833	4,217	4,763	4,992	4,780	4,699
1570051_at	5,330	5,458	5,458	6,102	5,750	5,993
1570052_at	5,620	5,698	5,951	6,102	5,970	6,103
1570054_at	4,795	4,880	5,070	5,066	4,986	5,169
1570055_at	8,097	8,233	8,238	9,888	9,349	9,649
1570057_x_at	6,853	6,933	7,048	8,210	8,182	8,214
1570058_at	5,356	5,759	5,495	6,175	5,878	6,083
1570061_at	5,946	5,820	6,124	5,860	5,913	5,870
1570064_at	6,011	5,940	5,820	6,182	5,866	6,093
1570065_at	6,116	6,027	6,219	7,037	6,955	7,135
1570068_at	4,703	4,542	4,684	4,979	4,890	5,267
1570070_at	9,029	8,666	8,860	9,285	9,066	9,348
1570071_at	7,038	7,035	7,027	7,642	7,608	7,946
1570076_at	4,076	4,055	3,880	3,880	3,867	4,038
1570078_a_at	7,251	7,121	7,352	7,380	7,631	7,496
1570080_at	4,062	4,379	4,162	4,463	4,246	4,348
1570082_x_at	6,496	6,674	6,667	6,836	6,736	7,012
1570084_at	5,466	5,371	5,347	5,570	5,253	5,273
1570085_at	7,030	6,891	6,536	7,904	7,982	8,007
1570087_at	6,398	6,091	6,047	6,598	6,781	6,862
1570089_at	5,611	5,505	5,696	6,259	6,189	6,089
1570090_at	7,188	7,275	6,892	7,645	7,769	7,644
1570093_at	5,692	5,874	5,352	6,425	6,144	6,184
1570098_at	5,284	5,169	5,124	4,782	4,891	4,886
1570099_at	4,166	4,180	4,301	4,709	4,632	4,307
1570100_at	4,954	4,992	5,081	5,049	5,232	5,045
1570101_at	4,289	3,902	4,045	4,029	4,069	3,996

1570102_at	5,295	5,582	5,602	6,291	6,468	6,703
1570103_at	3,968	4,266	4,201	4,154	4,189	4,404
1570105_at	7,265	7,046	7,241	7,595	7,577	7,649
1570106_at	5,600	5,441	5,544	5,698	5,390	5,732
1570107_at	6,085	6,192	5,988	6,671	6,587	6,578
1570108_at	7,451	7,287	7,253	7,893	7,652	7,851
1570110_at	5,949	5,732	6,015	6,650	6,419	6,717
1570111_at	4,541	4,416	4,924	4,741	4,323	4,246
1570114_at	3,915	3,903	3,896	4,289	4,495	4,199
1570115_at	3,560	3,898	4,225	3,702	3,756	3,913
1570116_at	4,793	4,928	4,694	4,901	4,697	4,691
1570117_at	3,555	3,231	3,649	4,049	3,802	3,993
1570119_at	4,204	4,259	4,127	4,431	4,002	4,124
1570120_at	5,598	5,363	5,403	5,482	5,763	5,835
1570121_at	4,780	4,651	5,010	5,099	5,316	5,239
1570122_at	4,427	4,422	4,641	4,675	4,530	4,619
1570123_at	6,923	6,697	6,753	6,950	7,161	7,153
1570124_at	7,499	7,794	7,715	7,039	7,102	7,429
1570125_at	5,428	5,366	5,285	5,030	5,058	5,030
1570126_at	5,987	5,788	6,248	6,223	6,335	6,508
1570127_at	5,665	5,131	5,781	7,210	6,652	7,007
1570128_at	4,306	4,881	4,724	5,192	4,578	4,668
1570130_at	4,473	4,353	4,812	4,589	4,491	4,423
1570131_at	5,884	6,067	6,117	6,792	6,612	6,690
1570133_at	6,994	6,805	6,851	7,101	7,077	7,225
1570134_at	5,202	5,128	4,976	5,458	5,266	5,497
1570135_at	3,727	4,190	4,148	4,196	4,163	4,138
1570136_at	4,257	4,168	4,324	4,413	4,113	4,288
1570138_at	5,659	5,602	5,652	6,718	6,772	6,813
1570140_at	7,195	7,694	7,541	7,643	7,577	7,743
1570141_at	3,952	4,260	4,091	4,153	4,132	4,323
1570143_at	7,865	6,996	7,766	7,064	7,299	7,268
1570145_at	5,849	5,541	6,072	6,142	5,724	5,864
1570146_at	5,040	4,819	4,554	4,928	5,122	4,754
1570151_at	6,666	6,328	6,283	6,344	6,604	6,388
1570152_at	4,915	5,111	5,190	5,336	4,882	5,122
1570153_at	5,601	5,486	5,688	6,442	5,957	5,376
1570155_at	7,055	7,304	7,260	7,812	8,535	8,457
1570156_s_at	7,045	7,007	7,055	8,166	7,586	7,747
1570158_at	4,956	4,843	5,390	5,084	4,747	4,989
1570160_at	4,324	4,197	4,772	4,301	4,301	4,251
1570162_at	4,498	4,558	4,837	5,808	5,864	5,912
1570163_at	5,573	5,147	5,500	5,907	5,434	5,821
1570165_at	5,197	5,142	5,259	5,239	5,414	5,713
1570166_a_at	8,541	8,147	8,252	8,320	8,477	8,045
1570167_at	5,855	5,801	5,842	6,162	5,928	6,185
1570168_at	4,222	4,310	4,394	4,425	4,370	4,508
1570169_at	7,761	7,369	7,589	7,862	7,811	7,542
1570171_at	6,409	6,343	6,587	6,848	6,748	7,095
1570173_at	4,223	4,908	5,296	3,695	3,853	3,644
1570176_at	3,913	4,162	4,081	4,314	4,009	4,101
1570177_at	6,549	6,206	6,166	6,115	6,245	6,561
1570180_at	4,041	4,101	3,939	5,149	4,779	4,830
1570181_a_at	3,700	4,150	3,769	4,371	4,374	4,542
1570182_at	6,602	6,713	6,675	7,587	7,655	7,880
1570183_at	4,034	4,014	4,003	4,084	3,895	4,190

1570185_at	8,578	8,495	8,569	8,993	9,203	9,152
1570186_at	5,967	6,034	5,903	6,574	6,087	6,159
1570188_at	5,254	4,924	5,129	5,292	5,370	5,424
1570189_at	4,766	4,998	4,937	5,007	5,224	5,140
1570190_at	6,001	5,871	5,900	6,250	6,258	6,498
1570191_at	5,099	5,120	5,091	4,832	4,841	4,860
1570192_at	6,662	7,020	6,979	6,605	6,633	6,625
1570194_x_at	3,739	3,695	4,103	3,780	3,601	3,684
1570196_at	5,431	5,672	5,489	5,835	5,298	5,524
1570197_at	8,523	8,102	8,810	8,163	7,921	8,017
1570198_x_at	9,064	8,557	9,001	8,525	8,694	8,640
1570200_at	5,783	6,346	6,175	6,156	6,281	5,950
1570202_a_at	5,301	4,901	5,342	6,020	6,037	5,890
1570204_at	4,254	4,030	4,394	3,861	4,158	4,016
1570205_at	4,231	4,549	4,184	4,390	4,199	4,305
1570206_at	5,098	5,259	5,251	5,440	5,458	5,781
1570207_at	3,808	3,881	3,663	3,928	3,863	3,866
1570208_at	3,607	3,601	3,624	3,685	3,624	3,633
1570210_x_at	8,193	7,956	7,939	7,528	8,195	7,779
1570212_a_at	6,415	6,352	6,664	6,725	6,516	6,661
1570213_at	3,610	3,878	3,693	3,712	3,603	3,754
1570215_at	5,601	5,642	5,973	6,623	6,499	6,548
1570219_at	7,615	7,466	7,571	8,404	7,743	7,887
1570221_at	7,106	6,652	6,727	7,037	7,049	7,230
1570222_at	3,658	3,559	3,434	3,814	3,728	3,915
1570224_at	5,835	5,715	5,627	6,164	6,087	6,215
1570225_at	5,996	5,995	5,949	6,529	6,509	6,600
1570226_at	3,700	4,065	3,969	4,066	3,922	4,070
1570227_at	7,429	6,953	7,084	7,046	7,110	7,099
1570228_at	3,484	3,539	3,686	3,727	3,648	3,662
1570229_at	5,894	5,819	6,146	5,940	5,669	5,760
1570230_at	6,249	6,223	6,141	6,733	7,044	6,759
1570231_at	4,438	4,335	4,542	4,535	4,450	4,537
1570233_at	3,423	3,497	3,579	3,619	3,492	3,603
1570234_at	5,070	4,895	5,026	5,276	5,203	5,155
1570235_at	4,043	4,251	4,189	4,249	4,188	4,437
1570237_at	6,062	5,939	5,937	6,371	6,328	6,506
1570238_at	6,710	6,807	7,427	7,621	7,161	7,314
1570239_a_at	8,186	8,094	8,165	8,397	8,241	8,520
1570241_at	7,295	7,863	8,102	9,339	9,353	9,457
1570242_x_at	7,142	7,305	7,234	8,530	8,528	8,557
1570243_at	7,470	7,410	7,271	8,154	7,692	7,869
1570244_at	3,615	3,744	3,835	3,917	4,296	4,238
1570246_at	3,866	3,721	4,256	4,041	3,817	4,089
1570248_at	5,913	5,690	5,811	6,484	5,946	6,090
1570249_x_at	6,867	6,374	6,642	7,075	6,736	6,460
1570250_at	4,438	4,281	4,489	4,999	4,562	4,560
1570251_at	6,482	6,060	6,232	6,628	6,837	6,452
1570252_at	6,319	6,317	5,960	6,553	6,286	6,078
1570253_a_at	5,244	6,175	5,673	5,294	5,032	5,359
1570255_s_at	3,661	3,950	3,599	3,546	3,668	3,659
1570257_x_at	6,574	6,196	6,300	6,651	6,877	6,342
1570259_at	3,978	3,852	4,312	3,807	5,783	4,199
1570261_at	4,930	5,787	5,291	4,722	4,591	4,711
1570262_at	6,290	6,369	6,382	6,520	6,583	6,833
1570263_at	4,744	4,586	4,919	5,384	5,540	5,591

1570264_at	5,698	5,686	5,308	5,759	5,972	5,722
1570266_x_at	10,800	10,714	11,102	10,977	10,633	10,823
1570267_at	6,939	6,440	6,767	7,352	7,072	7,276
1570268_at	4,595	4,538	4,412	4,935	4,992	4,932
1570269_at	4,105	4,093	4,053	4,216	4,122	4,285
1570270_at	5,771	5,827	5,616	6,359	6,160	6,312
1570271_at	4,356	4,772	4,441	4,778	4,889	4,885
1570272_at	5,830	4,947	5,581	5,403	5,703	5,768
1570273_at	4,732	4,691	4,804	5,172	5,196	5,516
1570274_at	5,633	5,549	5,781	6,084	5,724	6,054
1570276_a_at	6,595	6,668	6,708	6,737	6,683	6,871
1570279_at	5,257	5,137	5,388	5,547	5,523	5,703
1570281_at	7,218	6,493	7,152	7,338	7,065	7,450
1570282_at	6,689	6,440	6,799	6,817	6,299	6,590
1570283_at	6,388	6,400	6,474	6,722	6,552	6,780
1570284_x_at	4,146	4,339	3,964	4,240	4,235	4,197
1570285_at	5,460	5,838	5,616	6,213	5,972	6,196
1570289_at	6,260	6,136	6,198	6,844	6,674	6,956
1570290_at	4,494	4,532	4,726	4,847	4,744	4,858
1570291_at	4,574	4,766	4,787	4,820	4,920	4,879
1570292_at	4,627	4,297	5,112	4,813	4,606	4,807
1570293_at	6,071	5,999	6,114	5,843	5,804	5,895
1570295_at	6,988	6,817	7,159	7,026	7,081	7,330
1570296_at	5,224	5,145	5,176	6,055	5,615	5,933
1570297_at	6,885	6,508	6,624	6,772	6,901	6,835
1570298_at	4,665	4,880	4,840	4,992	4,982	5,067
1570299_at	5,658	5,567	5,544	6,134	6,600	6,372
1570300_at	4,992	4,621	4,597	5,638	5,435	5,494
1570301_at	4,764	4,637	4,748	5,749	5,856	5,536
1570302_at	4,646	4,841	4,769	5,362	5,118	5,456
1570303_at	5,064	5,071	5,115	5,255	5,160	5,036
1570306_at	4,675	4,941	4,690	4,813	5,023	5,001
1570307_s_at	7,251	6,891	7,341	7,500	7,385	7,796
1570308_at	4,129	3,884	4,274	4,414	4,147	4,415
1570314_at	5,674	5,819	5,648	5,791	5,980	5,827
1570315_at	6,395	6,521	6,640	7,309	7,258	7,425
1570316_at	5,334	5,031	5,609	6,001	5,729	5,831
1570318_at	5,723	5,837	5,655	5,981	5,945	6,044
1570320_at	4,420	4,295	4,467	4,727	4,585	4,324
1570326_at	4,974	5,084	5,156	5,820	5,458	5,448
1570327_at	6,285	6,408	6,587	6,315	6,232	6,268
1570328_s_at	6,869	6,942	6,655	6,892	6,687	6,832
1570329_at	6,530	6,178	6,226	6,588	6,391	6,467
1570330_at	8,038	7,751	7,885	8,591	8,692	8,721
1570331_at	6,067	5,968	6,075	6,657	6,437	6,293
1570332_at	3,858	4,623	3,833	4,027	4,015	4,333
1570335_at	6,423	5,734	6,054	5,688	6,065	5,752
1570336_at	6,505	6,180	6,418	7,085	6,986	6,818
1570337_at	5,367	5,518	5,718	6,091	5,895	6,246
1570338_at	6,954	6,664	6,644	7,397	6,897	6,972
1570339_x_at	6,449	6,089	6,455	6,643	6,475	6,609
1570342_at	5,908	6,024	5,768	6,446	6,467	6,542
1570344_at	6,297	6,236	6,572	7,053	6,670	6,579
1570345_at	4,145	4,296	4,724	5,017	5,190	5,294
1570347_at	7,093	6,911	7,151	7,358	7,392	7,593
1570349_at	5,620	5,859	5,903	5,806	6,014	6,140

1570350_at	6,543	6,641	6,845	7,529	7,014	7,417
1570351_at	7,105	6,643	6,598	6,711	8,719	6,759
1570352_at	3,888	3,711	3,816	3,625	3,698	3,665
1570354_s_at	4,368	4,004	4,138	4,257	4,349	4,217
1570355_a_at	4,346	4,815	4,824	5,547	5,042	5,354
1570357_at	5,781	5,755	5,482	5,825	5,820	6,165
1570359_at	3,830	3,940	3,783	3,663	3,660	3,707
1570360_s_at	5,690	5,868	5,900	6,777	6,591	6,673
1570361_a_at	4,758	5,390	4,814	6,356	6,626	6,850
1570362_at	8,527	8,104	8,315	8,509	8,666	8,743
1570364_at	4,552	4,902	4,546	5,952	6,242	6,436
1570366_x_at	4,515	4,304	4,387	5,531	5,707	5,958
1570371_a_at	7,328	6,993	6,796	7,359	7,183	7,463
1570372_at	6,363	6,377	6,341	7,117	6,935	6,792
1570373_at	5,640	5,866	6,121	6,015	5,725	5,746
1570375_at	6,632	6,641	6,940	7,537	7,437	7,474
1570376_at	6,235	6,566	6,308	7,155	6,896	6,917
1570382_at	7,313	7,217	7,399	7,577	7,523	7,893
1570383_at	6,369	5,564	6,407	6,300	6,497	6,208
1570384_at	3,985	3,844	3,968	4,143	3,901	3,711
1570385_at	5,598	5,787	6,044	5,805	5,777	5,800
1570388_a_at	5,243	4,930	4,826	5,673	5,903	5,567
1570391_at	4,114	4,234	4,241	4,101	4,036	4,025
1570393_at	4,540	5,057	4,844	5,337	5,081	5,161
1570394_at	4,272	4,564	4,201	4,334	4,609	4,521
1570395_a_at	4,311	4,405	4,419	4,179	4,124	4,283
1570396_at	5,300	5,522	5,693	4,879	4,565	4,821
1570397_x_at	4,896	5,517	5,458	4,705	4,673	5,067
1570398_at	4,456	4,562	4,151	4,262	4,287	4,178
1570402_at	8,019	7,745	8,164	8,454	8,002	8,312
1570405_at	7,593	7,423	7,412	8,318	8,395	8,375
1570408_at	4,543	4,678	4,515	4,933	5,267	5,005
1570409_x_at	5,821	5,375	5,061	5,877	6,281	6,094
1570410_at	8,195	7,888	8,039	8,689	8,781	8,685
1570411_at	4,474	4,535	4,606	4,531	4,837	4,503
1570412_at	6,598	5,633	6,075	5,356	5,412	5,314
1570414_x_at	6,784	6,626	6,703	6,238	6,389	6,187
1570415_at	4,516	4,174	4,230	3,634	3,749	3,636
1570420_at	7,717	7,557	7,631	8,066	7,977	8,186
1570422_at	4,813	4,903	5,234	5,022	4,655	5,005
1570423_at	4,243	4,327	4,346	4,422	4,258	4,427
1570424_at	5,715	5,214	5,413	6,520	6,072	6,853
1570425_s_at	4,635	4,849	4,647	4,897	4,938	5,005
1570430_at	4,958	5,171	5,245	5,285	5,278	5,343
1570432_at	6,437	6,168	6,778	7,232	7,030	7,338
1570433_at	5,500	5,209	5,891	6,296	6,125	6,209
1570439_at	6,443	6,105	6,185	6,440	7,059	6,695
1570441_at	4,283	4,576	4,262	4,437	4,093	4,087
1570444_at	5,755	5,645	5,569	6,650	6,380	6,718
1570445_a_at	4,992	5,181	5,163	5,539	5,518	5,979
1570447_at	6,585	6,701	6,801	7,127	7,211	7,233
1570448_at	4,907	4,912	5,039	4,959	4,797	5,022
1570450_at	6,619	6,506	7,259	6,100	6,217	5,985
1570451_at	6,099	5,755	5,908	6,477	6,175	6,237
1570452_at	3,363	3,509	3,481	3,445	3,454	3,702
1570454_at	5,000	5,188	4,638	5,037	5,189	5,056

1570465_at	5,447	5,299	5,429	5,843	6,235	6,072
1570469_at	4,812	5,079	5,302	5,132	4,609	4,750
1570470_at	5,258	5,320	5,420	5,344	5,617	5,418
1570471_at	6,166	6,172	6,151	7,022	7,181	7,151
1570473_at	4,077	4,094	4,285	4,354	4,523	4,438
1570474_s_at	6,313	6,204	6,706	7,256	6,954	7,076
1570476_at	5,543	5,386	5,606	6,450	6,306	6,682
1570479_at	7,187	7,226	7,092	8,066	7,526	7,445
1570480_s_at	5,897	5,874	5,784	7,076	6,996	7,478
1570482_at	5,261	5,471	5,103	4,704	4,873	4,698
1570483_at	7,189	7,359	6,981	7,372	7,307	7,443
1570484_at	6,468	6,967	6,956	6,944	7,184	7,362
1570486_at	4,178	4,116	4,573	4,489	4,326	4,333
1570488_at	4,962	4,549	4,697	4,432	4,174	4,406
1570490_at	6,139	6,213	5,900	6,639	6,468	6,382
1570491_at	4,438	4,078	4,342	4,689	4,555	4,747
1570496_at	5,722	5,837	5,518	5,155	5,038	4,932
1570503_at	5,712	5,645	5,664	5,929	5,886	5,944
1570505_at	5,080	5,197	4,931	5,180	5,308	5,305
1570506_at	4,149	4,474	4,188	4,439	4,496	4,880
1570507_at	4,017	4,152	3,860	3,919	4,053	3,836
1570511_at	5,857	5,396	5,632	5,559	6,073	5,466
1570515_a_at	7,936	8,878	6,578	4,646	4,563	4,794
1570516_s_at	5,312	5,512	5,451	5,715	5,419	5,838
1570517_at	4,363	4,529	4,724	4,528	4,406	4,377
1570523_s_at	5,872	6,049	5,619	6,065	5,728	5,709
1570528_at	4,922	4,685	4,960	5,636	5,133	5,670
1570529_at	6,350	6,184	5,995	7,379	7,203	7,150
1570531_at	7,079	6,427	6,886	6,846	6,715	7,013
1570533_at	5,341	5,325	5,525	5,379	5,489	5,629
1570534_a_at	5,196	5,987	5,156	6,355	5,606	5,976
1570536_at	6,499	6,616	6,601	7,202	7,173	7,268
1570537_a_at	4,856	4,638	4,625	6,445	6,261	6,446
1570541_s_at	5,382	4,901	5,369	6,093	5,798	5,971
1570542_a_at	6,587	6,183	6,366	6,776	6,666	6,896
1570546_a_at	6,010	6,390	6,177	7,026	6,534	7,068
1570552_at	5,292	5,286	5,572	5,798	5,699	5,902
1570561_at	4,406	4,131	4,430	6,214	4,882	5,090
1570566_at	6,095	5,872	6,241	6,357	6,195	6,448
1570567_at	6,524	6,211	6,525	6,205	6,434	6,557
1570568_at	6,551	6,325	6,379	7,005	7,076	7,094
1570569_at	7,762	7,774	7,806	8,269	8,139	8,577
1570571_at	7,727	7,042	7,227	4,612	5,011	4,833
1570572_at	4,627	4,546	4,584	5,205	4,770	4,895
1570574_at	4,628	4,865	5,072	4,892	4,635	4,839
1570575_at	4,214	4,346	4,068	4,083	3,918	3,944
1570578_at	4,202	4,105	3,951	4,554	4,414	4,698
1570579_at	4,938	4,547	4,851	4,945	4,797	5,058
1570584_at	3,851	4,124	4,052	4,052	4,227	4,115
1570585_at	3,724	3,784	3,864	3,724	3,714	3,655
1570587_at	5,345	5,560	5,574	5,637	5,916	5,539
1570588_at	6,951	6,709	7,037	6,563	6,881	6,779
1570592_a_at	5,524	5,539	5,490	5,715	5,564	5,663
1570593_at	4,836	5,306	4,978	5,699	5,915	5,703
1570594_at	3,684	4,118	4,165	4,223	4,159	4,225
1570596_at	4,298	4,265	4,324	4,652	4,431	4,457

1570597_at	3,989	4,077	3,956	4,315	4,113	4,327
1570600_at	6,907	6,942	6,709	6,957	7,057	6,912
1570601_at	5,979	5,471	5,806	6,198	6,128	6,391
1570607_at	7,732	7,442	7,725	8,147	7,583	7,746
1570618_at	4,251	4,168	4,168	4,324	4,329	4,432
1570621_at	4,709	4,540	4,512	4,924	4,725	4,629
1570622_at	3,847	3,873	4,014	3,841	3,954	3,767
1570623_at	4,873	5,336	4,765	3,953	4,004	3,781
1570624_at	6,002	5,716	5,842	6,246	6,352	6,181
1570625_at	6,347	6,450	6,458	6,948	6,975	7,045
1570627_at	6,595	6,636	6,695	6,769	6,888	6,855
1570628_at	4,200	4,374	4,484	4,573	4,200	4,293
1570629_at	4,061	3,836	3,818	4,142	4,482	4,785
1570630_at	6,284	6,014	6,066	6,650	6,700	6,965
1570631_at	6,194	6,532	6,232	6,562	6,550	6,734
1570632_at	4,219	4,350	4,125	4,282	4,438	4,302
1570633_at	3,784	3,736	3,925	4,240	3,830	4,045
1570635_at	6,101	6,308	6,413	6,663	6,746	7,045
1570639_at	5,557	5,452	5,563	5,514	5,475	5,262
1570644_at	6,273	6,013	6,153	6,180	6,143	5,988
1570645_at	4,521	4,375	4,375	4,598	4,544	4,333
1570650_at	5,692	6,019	6,305	6,365	6,273	6,047
1570651_at	7,188	6,924	6,262	6,711	6,446	6,732
1570653_at	5,403	5,035	5,435	5,628	5,397	5,289
1598_g_at	9,741	10,015	9,692	11,175	11,004	11,898
160020_at	7,415	7,282	7,457	7,866	7,984	8,096
1729_at	7,500	7,595	7,585	9,511	8,932	9,644
1773_at	6,778	6,201	6,614	7,193	6,825	7,015
177_at	6,394	6,734	6,503	7,439	7,175	7,151
179_at	9,016	8,854	9,008	9,337	9,127	9,499
1861_at	7,247	6,764	6,938	8,202	7,485	7,979
200000_s_at	10,551	10,428	10,859	9,805	9,738	9,821
200001_at	9,914	9,803	9,970	11,086	10,920	11,486
200002_at	12,938	12,849	12,955	12,517	11,937	12,692
200003_s_at	12,188	12,126	12,300	12,516	12,080	12,902
200004_at	13,925	13,811	13,835	13,009	13,745	13,394
200005_at	11,877	12,110	12,070	11,954	11,685	12,024
200006_at	13,613	13,642	13,762	13,363	13,478	13,656
200007_at	12,705	12,592	12,402	12,512	12,277	12,826
200008_s_at	10,069	10,061	9,481	9,243	9,059	9,378
200009_at	13,270	13,226	12,952	12,476	12,888	12,783
200010_at	11,766	11,661	11,361	11,852	11,272	11,803
200011_s_at	11,029	10,670	11,226	10,126	9,831	10,570
200012_x_at	13,934	14,000	13,957	13,363	13,198	13,584
200013_at	13,286	13,332	13,134	12,686	12,391	12,958
200014_s_at	12,885	12,813	12,794	12,354	12,283	12,238
200015_s_at	13,499	13,497	13,252	12,023	12,973	12,477
200016_x_at	14,633	14,600	14,556	13,567	14,023	13,515
200017_at	13,637	13,690	13,523	13,100	13,018	13,361
200018_at	13,952	13,999	13,882	13,619	13,643	13,947
200019_s_at	13,671	13,638	13,639	12,841	12,262	12,980
200020_at	12,874	12,754	12,698	11,495	11,615	11,065
200021_at	14,177	14,141	14,342	13,963	13,908	14,037
200022_at	12,661	12,808	12,797	12,564	12,123	12,774
200023_s_at	13,253	13,592	13,387	13,049	13,004	13,384
200024_at	13,302	13,227	13,293	13,323	13,052	13,660

200025_s_at	13,893	14,020	13,968	13,430	13,642	13,785
200026_at	12,936	13,072	12,945	13,069	12,737	13,174
200027_at	12,978	13,220	13,050	12,199	12,738	12,019
200028_s_at	12,630	12,546	13,105	11,979	11,958	12,026
200029_at	13,050	13,367	13,237	12,980	12,908	13,202
200030_s_at	14,102	14,146	14,054	13,268	13,680	13,681
200031_s_at	14,039	13,966	14,058	13,401	13,540	13,873
200032_s_at	14,310	14,431	14,307	13,878	13,996	14,162
200033_at	13,350	13,412	13,090	12,176	13,016	12,181
200034_s_at	14,452	14,468	14,337	13,525	13,838	13,854
200035_at	9,402	9,509	9,607	9,644	9,269	9,610
200036_s_at	12,751	13,081	13,050	12,865	12,661	13,012
200037_s_at	13,734	13,823	13,521	12,401	12,885	12,497
200038_s_at	13,919	14,035	13,743	13,375	13,616	13,729
200039_s_at	12,478	12,484	12,771	11,681	11,124	11,911
200040_at	13,141	13,037	13,028	12,173	12,426	12,264
200041_s_at	13,167	12,388	13,148	11,552	11,078	11,658
200042_at	10,973	10,965	11,224	11,116	11,157	11,334
200043_at	13,238	12,648	12,667	11,811	11,617	11,963
200044_at	12,121	11,815	12,152	11,345	11,029	11,582
200045_at	11,431	11,176	11,556	10,492	10,257	10,438
200046_at	11,472	11,397	11,373	12,409	12,135	12,954
200047_s_at	12,686	12,715	12,710	12,114	12,619	12,539
200048_s_at	12,091	12,132	12,485	12,402	12,079	12,702
200049_at	10,623	10,151	10,438	8,088	7,964	8,002
200050_at	12,353	12,331	11,818	10,150	11,262	9,507
200051_at	9,305	8,847	9,220	8,849	8,405	8,924
200052_s_at	12,753	12,207	12,350	10,985	10,009	10,692
200053_at	10,567	10,727	10,555	10,313	9,955	10,453
200054_at	9,163	9,304	9,414	10,192	9,911	10,089
200055_at	10,149	10,402	10,279	10,851	10,079	11,138
200056_s_at	10,806	11,216	10,598	10,517	10,400	10,678
200057_s_at	13,956	13,847	13,963	12,434	12,606	12,651
200058_s_at	12,579	12,263	12,649	10,505	10,630	10,462
200059_s_at	13,049	12,936	12,931	12,516	12,622	13,157
200060_s_at	12,379	12,294	12,397	11,167	11,067	11,375
200061_s_at	13,498	13,268	13,166	12,764	12,845	13,191
200062_s_at	14,220	14,452	14,192	13,537	13,672	13,880
200063_s_at	14,441	14,457	14,400	13,916	14,358	14,199
200064_at	13,929	13,719	13,651	12,478	12,622	12,609
200065_s_at	12,566	12,426	12,377	12,392	11,975	12,627
200066_at	10,719	10,959	10,352	10,482	10,733	10,063
200067_x_at	11,275	11,682	11,720	12,697	13,070	13,266
200068_s_at	12,433	12,254	12,573	12,190	12,792	12,249
200069_at	11,973	11,677	12,052	9,009	8,638	8,276
200070_at	8,955	9,033	9,133	9,655	9,392	10,232
200071_at	11,947	12,042	12,135	11,295	11,147	11,195
200072_s_at	12,861	12,370	12,874	11,430	11,387	11,013
200073_s_at	12,965	12,907	12,968	12,128	12,260	12,069
200074_s_at	12,961	12,946	12,710	12,643	12,096	12,607
200075_s_at	10,246	10,207	10,383	11,406	10,975	11,746
200076_s_at	8,997	9,024	9,105	9,244	8,711	9,129
200077_s_at	13,755	13,740	13,806	13,566	13,612	13,851
200078_s_at	11,040	11,016	11,348	11,101	10,173	11,209
200079_s_at	12,121	12,325	12,411	11,678	11,516	11,654
200080_s_at	14,266	14,226	14,286	13,082	13,244	13,411

200081_s_at	14,168	14,053	13,893	13,029	12,464	13,365
200082_s_at	12,013	12,343	12,043	11,390	11,092	11,517
200083_at	11,146	11,082	11,475	10,197	10,297	10,469
200084_at	11,854	11,729	11,678	11,138	11,375	11,344
200085_s_at	10,981	11,022	11,183	10,732	10,797	11,313
200086_s_at	13,075	13,283	13,271	12,957	12,660	12,944
200087_s_at	12,008	11,685	12,047	11,699	12,021	12,129
200088_x_at	14,083	14,322	14,220	13,556	13,521	13,818
200089_s_at	13,900	14,043	13,852	13,306	13,295	13,502
200090_at	13,181	13,563	12,938	11,751	12,212	11,980
200091_s_at	13,055	13,144	12,899	13,005	12,896	13,232
200092_s_at	14,149	14,318	14,251	13,914	13,915	14,078
200093_s_at	12,242	12,474	12,495	12,327	12,208	12,595
200094_s_at	12,586	12,798	12,637	12,380	12,500	12,539
200095_x_at	14,116	14,402	14,365	13,876	13,792	14,018
200096_s_at	11,321	11,083	11,214	12,878	12,323	12,940
200097_s_at	13,045	12,900	12,858	11,768	12,455	12,060
200098_s_at	11,959	12,110	12,072	11,704	11,852	12,000
200099_s_at	14,372	14,537	14,285	13,683	13,981	14,036
200593_s_at	12,006	11,408	11,836	10,038	10,064	9,670
200594_x_at	13,260	12,467	13,022	11,250	11,744	11,033
200595_s_at	12,858	12,668	12,288	11,277	12,680	11,487
200596_s_at	10,960	11,093	10,776	9,993	10,735	9,658
200597_at	10,339	10,332	9,930	8,326	9,048	7,602
200598_s_at	10,830	10,722	10,908	10,639	10,092	10,271
200599_s_at	12,898	12,979	12,368	11,850	12,581	11,578
200600_at	11,438	11,158	11,099	10,940	11,008	11,603
200601_at	8,688	8,583	8,852	9,493	9,645	9,912
200602_at	10,976	11,205	11,043	10,159	11,256	10,775
200603_at	11,966	11,980	11,606	11,961	12,787	12,422
200604_s_at	10,052	9,863	10,213	9,564	9,510	9,436
200605_s_at	10,417	10,585	10,099	10,085	10,950	10,733
200606_at	10,572	10,947	10,772	4,867	7,685	9,400
200607_s_at	9,835	9,835	9,341	9,339	8,270	8,560
200608_s_at	12,142	12,220	11,734	10,773	11,460	10,363
200609_s_at	12,177	12,061	12,483	12,357	12,797	12,879
200610_s_at	13,131	12,974	12,821	12,271	12,574	11,915
200611_s_at	11,130	10,550	11,203	11,773	11,610	12,122
200612_s_at	8,878	8,876	8,817	7,889	8,547	8,248
200613_at	12,568	12,166	12,591	11,719	11,830	12,215
200614_at	13,266	13,489	13,339	12,448	13,424	12,741
200615_s_at	9,799	10,024	9,936	9,717	10,496	10,222
200616_s_at	12,016	11,550	12,093	11,361	10,816	11,422
200617_at	11,591	11,394	11,731	10,198	10,692	10,490
200618_at	10,655	10,711	10,998	11,880	12,180	12,630
200619_at	11,014	11,040	11,292	10,902	10,487	10,932
200620_at	11,729	11,990	11,638	11,290	11,842	11,869
200621_at	7,770	7,965	8,130	11,446	11,325	11,721
200622_x_at	9,126	9,039	9,078	9,807	8,771	9,765
200623_s_at	9,532	9,344	9,421	9,166	9,241	9,394
200624_s_at	11,528	11,567	11,008	9,372	10,272	8,851
200625_s_at	12,867	13,073	13,100	12,676	13,198	13,093
200626_s_at	12,709	12,773	12,474	10,711	11,578	10,697
200627_at	13,817	13,755	13,748	12,700	12,617	12,754
200628_s_at	9,182	9,067	9,395	9,122	8,953	9,497
200629_at	10,988	10,844	11,486	10,990	11,334	11,420

200630_x_at	12,534	12,365	12,586	11,185	11,422	11,541
200631_s_at	14,404	14,418	14,389	12,237	12,764	12,350
200632_s_at	8,554	9,095	8,096	9,868	9,825	9,450
200633_at	14,124	14,209	14,318	13,854	13,910	14,018
200634_at	10,089	9,346	9,789	9,954	9,174	10,396
200635_s_at	9,230	9,520	9,732	7,409	7,406	7,600
200636_s_at	11,713	11,778	11,856	7,793	7,339	7,796
200637_s_at	7,277	7,192	7,338	6,779	6,631	6,990
200638_s_at	12,298	11,947	11,687	11,400	11,679	11,788
200639_s_at	12,614	12,324	12,483	12,262	12,500	12,423
200640_at	12,423	12,333	12,412	11,673	11,961	11,831
200641_s_at	9,092	8,732	8,665	8,780	8,199	8,966
200642_at	13,802	13,958	13,636	13,018	12,868	13,218
200643_at	9,881	9,758	9,959	10,501	10,483	10,881
200644_at	12,794	12,287	12,624	7,159	6,867	7,166
200645_at	11,649	11,745	11,837	11,650	11,539	12,304
200646_s_at	6,602	6,394	6,611	7,795	7,435	7,770
200647_x_at	13,030	12,599	13,156	12,360	12,451	12,301
200648_s_at	9,670	9,496	9,593	7,883	6,937	7,593
200649_at	8,385	8,151	8,127	8,606	8,272	8,745
200650_s_at	13,005	12,914	13,132	13,684	14,126	14,073
200651_at	12,872	12,652	12,813	12,582	12,186	12,849
200652_at	12,302	12,146	12,234	12,305	11,948	12,565
200653_s_at	10,352	10,433	10,292	10,696	10,608	10,821
200654_at	10,632	10,579	11,013	12,182	12,237	12,788
200655_s_at	12,370	12,317	12,330	11,837	12,476	12,201
200656_s_at	8,260	8,038	8,261	9,685	9,322	10,328
200657_at	13,333	13,465	13,340	12,813	12,398	12,617
200658_s_at	10,789	10,572	10,807	10,963	10,227	11,045
200659_s_at	10,544	10,131	10,622	10,603	10,112	10,610
200660_at	9,118	9,141	9,314	10,602	10,259	11,041
200661_at	9,076	8,692	9,160	10,274	10,092	10,655
200662_s_at	13,543	13,730	13,153	11,231	12,162	11,140
200663_at	12,508	12,566	12,710	13,176	13,392	13,796
200664_s_at	8,638	9,001	8,867	8,922	8,320	8,566
200665_s_at	11,686	11,524	11,607	12,399	12,781	13,418
200666_s_at	10,174	10,163	10,170	9,454	8,925	9,514
200667_at	11,952	12,114	12,008	11,513	11,726	11,560
200668_s_at	11,992	11,883	11,978	12,304	12,525	12,532
200669_s_at	10,437	10,455	10,269	11,023	10,693	11,189
200670_at	8,936	9,559	9,543	9,949	10,193	10,635
200671_s_at	7,654	7,672	8,002	7,210	7,602	8,032
200672_x_at	7,288	7,168	7,423	7,442	7,300	6,986
200673_at	12,792	13,091	12,857	12,600	12,791	13,101
200674_s_at	13,904	14,268	14,235	13,455	13,522	13,712
200675_at	11,950	11,669	12,141	11,368	11,288	11,905
200676_s_at	8,874	8,898	9,175	8,321	8,310	8,522
200677_at	12,639	12,592	12,839	11,930	12,411	12,548
200678_x_at	9,093	9,282	9,464	10,156	9,701	10,523
200679_x_at	11,850	11,615	11,343	11,463	10,510	11,134
200680_x_at	13,563	13,595	13,352	12,786	12,662	12,683
200681_at	13,120	13,384	12,785	12,052	12,429	12,193
200682_s_at	10,251	10,314	10,379	11,497	11,312	11,545
200683_s_at	10,562	10,611	11,130	10,481	10,057	10,506
200684_s_at	9,866	9,838	10,367	9,332	9,186	9,472
200685_at	10,263	10,361	9,934	7,338	8,838	7,394

200686_s_at	13,427	13,291	13,058	11,628	12,717	11,478
200687_s_at	10,819	10,655	11,146	9,986	9,795	9,769
200688_at	9,177	8,887	9,185	8,516	8,691	8,450
200689_x_at	14,399	14,459	14,413	13,584	13,641	13,858
200690_at	9,887	9,939	9,810	9,084	9,602	8,722
200691_s_at	12,765	12,756	12,535	12,015	12,234	11,523
200692_s_at	11,497	11,390	10,900	10,763	10,551	10,267
200693_at	13,388	13,334	13,336	12,548	12,745	12,885
200694_s_at	10,631	10,480	10,531	10,360	10,553	10,468
200695_at	11,168	10,575	10,779	10,325	9,915	10,713
200696_s_at	9,377	9,608	9,275	9,254	9,611	10,230
200697_at	11,477	11,708	11,867	12,038	11,845	11,830
200698_at	10,569	10,349	10,553	11,024	11,301	11,565
200699_at	11,121	11,607	11,708	11,534	11,688	11,771
200700_s_at	11,249	11,148	11,411	12,523	12,792	13,098
200701_at	9,542	9,889	9,512	11,137	10,536	11,069
200702_s_at	9,106	9,265	9,370	9,993	9,569	9,892
200703_at	12,517	12,304	12,299	12,575	12,132	12,962
200704_at	13,616	13,704	13,742	10,087	10,339	10,913
200705_s_at	13,619	13,811	13,799	13,101	13,044	13,305
200706_s_at	11,873	11,449	11,731	8,399	7,902	9,196
200707_at	9,936	9,406	9,778	8,957	8,924	9,368
200708_at	12,691	12,628	13,056	12,310	12,005	12,250
200709_at	11,122	10,922	11,215	11,308	11,127	12,099
200710_at	9,565	9,375	9,300	10,286	10,441	10,755
200711_s_at	11,677	11,652	11,543	11,162	11,655	11,447
200712_s_at	9,325	9,532	9,045	9,665	9,568	9,253
200713_s_at	13,235	13,274	13,147	12,189	12,553	12,392
200714_x_at	10,174	10,215	10,463	9,953	10,314	10,233
200715_x_at	13,328	13,566	13,334	13,281	13,204	13,514
200716_x_at	13,984	13,850	13,596	13,115	12,725	13,510
200717_x_at	14,498	14,608	14,414	13,785	14,142	14,127
200718_s_at	13,692	13,783	13,717	13,394	13,842	13,736
200719_at	9,951	9,755	10,096	8,557	9,018	8,316
200720_s_at	8,782	8,855	8,679	9,776	9,282	10,174
200721_s_at	9,182	8,955	9,099	9,726	9,429	10,017
200722_s_at	8,852	8,795	8,535	8,486	8,398	8,426
200723_s_at	12,415	12,391	12,401	11,675	12,531	12,070
200724_at	7,490	7,631	7,777	8,286	8,082	8,337
200725_x_at	13,452	13,444	13,486	13,330	13,099	13,601
200726_at	13,545	13,823	13,705	11,951	12,595	11,933
200727_s_at	10,233	10,454	10,202	10,515	10,438	10,644
200728_at	11,778	11,942	11,226	10,747	11,743	11,138
200729_s_at	10,766	10,591	10,545	10,198	11,122	10,755
200730_s_at	8,543	8,257	8,323	8,460	9,558	9,271
200731_s_at	7,897	8,241	8,131	8,130	9,899	8,937
200732_s_at	10,124	10,428	10,240	10,264	12,031	10,942
200733_s_at	10,560	10,145	10,480	11,371	11,799	11,675
200734_s_at	10,599	10,515	11,083	11,134	10,851	11,394
200735_x_at	13,926	14,058	13,703	12,952	12,940	13,076
200736_s_at	12,467	12,082	12,283	12,663	11,960	12,484
200737_at	12,862	12,923	12,905	11,801	11,713	12,035
200738_s_at	13,782	13,748	13,841	13,059	13,101	13,390
200739_s_at	8,798	8,573	8,647	9,247	9,229	9,485
200740_s_at	13,480	13,400	13,801	12,570	12,500	12,728
200741_s_at	13,589	13,779	13,841	13,209	13,158	13,390

200742_s_at	8,590	8,540	9,179	8,407	8,005	8,458
200743_s_at	10,731	10,873	11,184	10,702	10,897	10,976
200744_s_at	9,989	9,975	10,136	11,048	10,515	11,004
200745_s_at	11,356	11,352	11,559	10,988	11,140	11,533
200746_s_at	12,079	12,091	12,147	11,495	11,779	11,716
200747_s_at	9,379	9,219	9,666	8,781	8,901	8,895
200748_s_at	12,479	12,397	12,276	13,681	13,491	14,040
200749_at	12,110	11,858	11,923	10,161	10,615	10,438
200750_s_at	13,927	13,867	13,773	13,210	12,773	13,220
200751_s_at	11,128	10,907	10,643	10,414	9,305	10,138
200752_s_at	9,222	9,101	9,361	8,585	8,119	8,555
200753_x_at	10,092	9,762	10,241	10,257	9,475	10,123
200754_x_at	13,033	12,998	13,032	11,987	11,910	12,021
200755_s_at	10,916	10,821	10,426	10,512	11,190	10,896
200756_x_at	8,565	8,596	8,249	9,099	8,929	9,046
200757_s_at	10,992	11,076	10,688	10,536	11,527	11,330
200758_s_at	9,808	9,633	9,938	10,145	10,478	10,615
200759_x_at	10,215	10,166	10,445	10,310	10,541	10,777
200760_s_at	9,126	9,316	8,418	10,306	10,875	11,551
200761_s_at	10,190	10,819	9,955	11,356	12,607	12,404
200762_at	13,016	12,828	13,205	11,497	12,225	12,221
200763_s_at	14,299	14,421	14,450	13,565	13,555	13,986
200764_s_at	9,553	9,744	9,416	9,360	9,582	9,447
200765_x_at	11,313	11,557	11,378	10,477	11,422	11,148
200766_at	8,470	8,220	8,276	8,734	8,427	9,275
200767_s_at	7,557	7,690	7,826	8,124	8,162	8,372
200768_s_at	11,951	11,356	11,754	11,030	11,034	10,740
200769_s_at	9,132	8,549	9,045	8,686	8,403	8,758
200770_s_at	9,697	9,828	9,799	10,367	10,835	10,720
200771_at	12,343	12,401	12,560	11,063	12,537	12,118
200772_x_at	11,732	11,382	11,326	10,718	10,360	10,586
200773_x_at	13,683	13,597	13,589	12,632	12,636	12,610
200774_at	12,548	12,391	12,700	12,742	13,352	13,211
200775_s_at	13,383	13,022	12,983	11,958	12,031	12,074
200776_s_at	11,209	10,718	10,727	10,934	11,965	11,267
200777_s_at	13,217	12,798	12,785	12,159	13,192	12,301
200778_s_at	11,667	11,725	11,653	10,937	11,421	11,217
200779_at	12,049	12,324	12,311	11,753	11,532	12,161
200780_x_at	12,866	13,067	13,327	12,264	12,667	13,001
200781_s_at	13,704	14,092	13,917	13,016	12,804	13,243
200782_at	11,700	11,589	12,035	12,631	12,961	13,304
200783_s_at	13,180	13,055	13,080	10,705	10,704	10,158
200784_s_at	7,481	7,550	7,656	7,774	7,848	8,389
200785_s_at	7,708	7,436	7,580	8,287	8,669	8,761
200786_at	12,502	12,421	12,836	12,429	11,919	12,449
200787_s_at	8,194	8,112	8,430	9,310	8,635	9,587
200788_s_at	10,518	10,205	10,666	11,242	11,199	11,705
200789_at	10,000	10,193	10,009	9,524	9,473	9,901
200790_at	13,575	12,967	13,728	13,171	13,095	13,116
200791_s_at	10,302	10,779	10,112	9,925	11,233	9,830
200792_at	13,106	12,972	13,010	12,258	12,119	12,293
200793_s_at	10,095	10,118	10,154	10,527	10,173	10,742
200794_x_at	10,635	11,098	10,675	11,635	11,630	11,976
200795_at	6,248	5,620	4,794	5,229	4,922	4,986
200796_s_at	5,538	5,156	5,294	5,996	5,104	5,533
200797_s_at	13,089	13,505	13,037	11,751	12,451	11,962

200798_x_at	10,791	10,469	10,758	9,820	9,224	9,121
200799_at	11,170	11,236	11,344	11,880	11,618	12,028
200800_s_at	10,076	9,925	9,966	10,566	10,003	10,634
200801_x_at	14,472	14,253	14,384	13,629	13,827	14,152
200802_at	10,847	11,184	11,131	10,918	10,489	11,317
200803_s_at	11,873	11,527	12,153	12,789	12,500	13,076
200804_at	12,385	12,352	12,446	11,508	11,730	11,858
200805_at	9,118	8,883	9,144	9,677	9,219	9,878
200806_s_at	12,357	12,238	11,788	10,542	10,436	9,904
200807_s_at	14,300	14,268	14,130	13,050	13,626	12,894
200808_s_at	8,704	8,509	8,789	8,713	8,869	9,613
200809_x_at	14,037	14,302	14,231	13,542	13,527	13,812
200810_s_at	12,890	13,101	13,227	11,860	12,518	12,442
200811_at	11,334	11,042	11,260	10,061	10,398	10,533
200812_at	12,882	12,898	13,023	12,488	12,155	12,383
200813_s_at	9,483	9,534	9,108	8,071	8,883	8,321
200814_at	9,520	10,163	9,950	10,970	10,707	11,135
200815_s_at	10,424	10,626	10,283	10,594	10,407	10,687
200816_s_at	10,743	10,512	10,362	8,453	9,949	8,823
200817_x_at	13,509	13,632	13,577	13,183	12,608	13,298
200818_at	13,749	13,797	13,746	12,835	12,498	13,038
200819_s_at	14,371	14,384	14,391	13,817	13,479	13,979
200820_at	10,639	10,591	10,823	11,832	11,165	11,969
200821_at	10,393	10,983	10,161	10,602	10,973	10,456
200822_x_at	13,565	13,371	13,750	12,525	12,207	12,674
200823_x_at	12,240	12,652	12,506	12,284	12,141	12,520
200824_at	10,738	10,513	10,956	10,250	9,108	10,343
200825_s_at	10,422	10,126	10,655	9,902	9,948	9,955
200826_at	12,198	12,356	12,180	12,047	11,813	12,175
200827_at	7,909	8,004	8,082	9,741	9,568	10,240
200828_s_at	11,602	11,461	11,367	10,700	10,856	10,704
200829_x_at	12,204	12,322	12,477	11,431	11,691	11,563
200830_at	12,694	12,644	13,011	12,725	12,607	12,829
200831_s_at	9,519	9,447	9,758	8,980	8,705	9,418
200832_s_at	12,363	12,358	12,224	9,688	9,925	10,029
200833_s_at	12,354	12,471	12,047	11,676	12,432	11,804
200834_s_at	13,751	13,918	14,007	13,487	13,436	13,633
200835_s_at	8,889	9,056	8,920	9,085	8,889	9,902
200836_s_at	8,795	8,724	8,913	9,414	9,263	9,481
200837_at	10,722	10,384	11,064	11,816	11,199	11,872
200838_at	9,209	9,459	9,364	11,162	11,483	11,557
200839_s_at	10,277	10,643	10,578	12,259	12,830	12,654
200840_at	13,707	13,766	13,858	13,075	13,258	13,272
200841_s_at	8,832	9,109	8,421	7,599	7,843	7,675
200842_s_at	11,180	11,697	10,931	10,332	11,129	10,165
200843_s_at	12,161	12,516	11,629	10,431	11,904	10,454
200844_s_at	12,897	12,868	12,904	12,155	12,203	12,512
200845_s_at	13,184	13,134	13,204	12,521	12,677	12,858
200846_s_at	11,084	10,516	10,959	10,563	9,799	10,726
200847_s_at	12,397	12,495	12,430	11,775	12,038	12,199
200848_at	10,799	10,730	10,454	9,659	9,404	9,666
200849_s_at	10,475	10,648	10,487	9,465	9,403	9,318
200850_s_at	11,475	11,657	11,390	10,349	10,503	10,216
200851_s_at	13,058	13,157	13,228	11,319	11,534	11,629
200852_x_at	9,617	9,389	9,434	10,090	9,521	10,255
200853_at	12,912	12,957	12,852	13,084	12,470	12,835

200854_at	9,005	8,785	9,190	7,986	8,675	7,898
200855_at	7,564	7,381	7,439	6,845	7,259	6,830
200856_x_at	7,164	7,617	6,977	6,074	6,536	5,650
200857_s_at	8,131	8,289	8,294	7,513	8,017	7,477
200858_s_at	13,976	14,107	13,912	13,418	13,136	13,651
200859_x_at	9,961	9,602	9,950	9,889	10,144	10,426
200860_s_at	12,118	12,399	12,217	10,288	11,180	9,957
200861_at	9,524	9,617	9,764	8,059	8,546	7,332
200862_at	11,891	11,507	12,085	11,043	10,380	11,223
200863_s_at	12,494	12,792	12,633	12,355	12,499	12,666
200864_s_at	10,565	10,622	10,068	9,419	9,677	9,481
200865_at	4,302	4,417	3,790	4,418	4,305	4,536
200866_s_at	9,691	9,885	9,716	9,222	8,686	9,700
200867_at	9,291	9,450	9,414	8,691	8,213	8,504
200868_s_at	8,824	9,191	8,825	9,209	8,577	9,013
200869_at	10,348	10,115	10,151	10,057	9,872	10,437
200870_at	14,053	13,926	14,054	12,750	13,039	13,165
200871_s_at	12,239	12,689	12,622	12,139	12,281	12,574
200872_at	10,893	11,284	11,326	12,336	13,038	12,955
200873_s_at	13,723	13,899	13,569	12,600	12,865	12,402
200874_s_at	9,495	9,034	9,571	8,443	7,594	8,254
200875_s_at	11,693	11,385	11,768	10,694	10,200	10,044
200876_s_at	12,633	12,540	12,566	12,344	11,866	12,256
200877_at	13,875	13,979	13,983	12,994	13,324	13,097
200878_at	6,481	7,080	6,782	11,384	11,392	11,131
200879_s_at	6,009	6,130	6,509	7,027	6,919	6,991
200880_at	12,150	11,716	11,760	10,316	10,862	10,244
200881_s_at	12,188	11,968	11,763	11,523	11,612	10,914
200882_s_at	12,780	12,917	12,876	11,951	11,624	11,933
200883_at	11,878	12,058	11,069	10,828	10,909	10,909
200884_at	10,505	10,366	9,985	8,668	7,432	7,978
200885_at	8,925	8,737	8,663	11,086	10,846	11,959
200886_s_at	13,813	13,908	14,083	13,471	13,707	13,736
200887_s_at	11,051	11,360	11,016	10,780	12,059	11,413
200888_s_at	10,934	10,559	10,298	10,851	10,695	11,024
200889_s_at	9,738	9,807	9,838	11,626	11,355	11,966
200890_s_at	9,208	9,084	8,932	9,770	10,294	10,368
200891_s_at	11,859	11,897	11,518	12,068	12,729	12,637
200892_s_at	11,928	11,870	11,659	10,935	10,593	10,548
200893_at	13,558	13,476	13,480	12,403	12,610	12,349
200894_s_at	10,528	10,161	10,368	8,168	7,339	7,563
200895_s_at	12,397	11,985	12,304	10,479	9,816	9,988
200896_x_at	11,621	11,592	12,152	11,440	10,588	11,148
200897_s_at	11,138	11,261	10,819	10,383	11,722	11,378
200898_s_at	8,974	9,272	8,806	7,993	9,082	7,858
200899_s_at	10,967	11,409	10,757	8,926	10,446	9,085
200900_s_at	11,449	11,071	11,295	10,075	8,958	10,016
200901_s_at	9,895	9,735	9,897	10,467	10,029	10,190
200902_at	11,527	11,654	11,488	11,585	12,023	11,933
200903_s_at	11,297	11,669	11,511	10,986	10,487	11,078
200904_at	7,904	8,470	8,100	9,322	9,484	9,775
200905_x_at	9,762	10,177	10,357	11,291	10,918	11,507
200906_s_at	8,079	8,027	8,216	8,185	8,424	8,906
200907_s_at	9,068	9,041	8,586	8,590	9,239	9,386
200908_s_at	7,521	7,842	7,871	7,179	7,296	7,351
200909_s_at	12,452	12,301	12,422	12,109	11,345	12,310

200910_at	12,847	12,735	12,951	12,000	11,622	12,048
200911_s_at	9,725	9,981	9,410	10,396	11,495	10,625
200912_s_at	13,883	14,174	13,714	12,002	12,917	12,531
200913_at	10,611	10,000	10,538	10,162	9,176	9,873
200914_x_at	9,391	9,158	9,118	8,911	9,937	8,276
200915_x_at	10,916	11,066	10,367	10,149	11,711	9,672
200916_at	11,696	11,368	11,756	11,995	10,939	11,546
200917_s_at	8,760	8,543	8,650	10,154	9,357	10,591
200918_s_at	10,564	10,738	11,005	11,536	11,706	12,009
200919_at	10,415	10,099	10,339	11,214	11,004	11,161
200920_s_at	9,362	9,845	9,595	8,833	9,948	9,768
200921_s_at	9,993	10,293	10,186	10,227	11,030	10,889
200922_at	7,270	7,381	7,198	7,808	6,797	8,247
200923_at	8,347	8,684	8,288	10,049	9,511	10,216
200924_s_at	10,552	10,869	10,894	10,256	9,771	10,832
200925_at	12,108	12,003	12,134	11,912	11,572	12,285
200926_at	14,327	14,415	14,286	13,799	13,938	14,075
200927_s_at	9,740	9,640	9,556	8,826	9,160	9,225
200928_s_at	6,481	6,972	6,512	6,383	6,734	6,301
200929_at	9,542	9,837	9,752	10,677	10,443	10,836
200930_s_at	5,198	4,997	5,579	5,722	5,613	5,808
200931_s_at	12,458	12,502	12,516	11,868	12,972	12,727
200932_s_at	10,160	10,412	10,170	10,095	9,870	10,322
200933_x_at	14,733	14,826	14,790	14,148	14,204	14,290
200934_at	13,045	13,008	12,566	11,577	12,452	10,886
200935_at	8,244	7,524	7,976	7,913	7,300	7,782
200936_at	13,773	13,754	13,721	13,242	12,514	13,457
200937_s_at	14,165	14,147	14,091	13,296	13,226	13,440
200938_s_at	5,888	6,074	6,219	7,017	7,027	7,239
200939_s_at	4,881	4,479	4,809	5,168	5,151	5,090
200940_s_at	10,237	10,066	10,518	9,117	10,365	10,239
200941_at	12,857	13,040	12,621	10,426	11,038	10,767
200942_s_at	10,751	11,043	10,478	10,237	9,757	10,323
200943_at	13,657	13,710	13,605	12,057	12,327	12,364
200944_s_at	14,076	13,954	14,016	12,680	12,983	12,908
200945_s_at	10,928	10,959	11,142	11,233	12,290	12,011
200946_x_at	9,573	9,438	9,442	9,145	9,233	9,578
200947_s_at	11,326	11,365	11,357	10,051	10,582	10,418
200948_at	11,914	11,766	12,193	10,977	9,919	11,098
200949_x_at	12,889	13,024	12,952	12,495	12,530	12,725
200950_at	10,959	10,855	10,877	11,285	11,268	11,677
200951_s_at	12,291	12,315	12,254	6,725	6,846	6,541
200952_s_at	9,011	9,043	8,864	7,110	7,228	7,166
200953_s_at	12,699	12,175	12,276	6,553	7,138	6,367
200954_at	9,307	8,767	9,194	9,539	9,619	9,900
200955_at	11,428	11,725	11,451	10,197	10,454	10,047
200956_s_at	10,267	10,167	10,426	10,023	8,730	9,151
200957_s_at	11,591	11,254	11,667	10,495	9,967	9,839
200958_s_at	12,751	13,089	12,484	12,465	13,122	12,478
200959_at	12,903	12,642	12,860	11,593	11,231	11,108
200960_x_at	11,917	11,923	12,163	12,071	11,481	12,058
200961_at	11,349	11,554	11,642	10,942	10,670	10,811
200962_at	10,845	12,029	10,292	8,314	8,024	8,367
200963_x_at	13,767	13,978	13,817	13,253	13,228	13,489
200964_at	11,471	11,193	11,322	10,539	10,080	10,895
200965_s_at	10,578	10,478	10,901	6,865	6,444	6,531

200966_x_at	12,733	12,614	13,083	12,868	12,647	13,227
200967_at	11,946	11,902	12,289	12,767	12,253	12,790
200968_s_at	11,108	10,937	11,281	11,670	11,178	11,817
200969_at	11,393	11,567	11,391	10,653	11,099	11,069
200970_s_at	11,583	11,608	11,345	10,700	11,125	11,484
200971_s_at	11,996	12,146	12,405	12,871	12,648	13,171
200972_at	12,022	11,992	12,064	11,413	11,066	11,343
200973_s_at	9,941	9,640	9,633	9,090	8,282	8,760
200974_at	7,199	6,710	6,969	9,976	10,479	10,522
200975_at	12,264	11,998	12,156	10,832	10,583	10,818
200976_s_at	11,833	12,373	11,336	9,922	11,128	9,720
200977_s_at	11,090	11,543	10,638	9,519	10,909	9,152
200978_at	12,926	13,353	12,743	12,623	12,624	12,966
200979_at	9,215	9,310	9,614	7,473	7,714	7,798
200980_s_at	11,857	11,833	11,586	11,042	10,379	10,845
200981_x_at	12,825	13,020	13,254	12,171	12,557	12,896
200982_s_at	8,473	8,475	8,353	9,970	9,578	9,987
200983_x_at	8,758	9,041	8,672	12,328	12,317	12,880
200984_s_at	8,971	9,229	9,040	11,899	12,109	12,288
200985_s_at	10,290	10,227	9,812	12,543	13,269	13,224
200986_at	9,395	9,454	9,748	7,722	7,966	8,474
200987_x_at	9,326	9,061	9,579	10,971	10,193	10,708
200988_s_at	9,023	8,708	9,307	8,803	8,532	8,649
200989_at	13,432	13,454	13,033	13,068	14,001	13,582
200990_at	10,560	9,977	10,680	10,113	9,148	9,946
200991_s_at	10,695	10,421	10,886	10,675	10,422	11,076
200992_at	12,168	12,114	11,570	10,757	11,610	10,418
200993_at	11,791	11,726	11,019	9,939	11,061	9,422
200994_at	12,389	12,252	11,828	10,594	11,567	10,256
200995_at	11,249	11,340	10,984	8,584	9,331	7,631
200996_at	12,317	12,217	12,072	12,072	12,616	12,476
200997_at	12,452	12,171	12,703	11,645	11,014	11,600
200998_s_at	10,773	10,700	10,891	11,857	11,913	12,384
200999_s_at	12,367	12,135	12,535	12,971	13,294	13,490
201000_at	11,774	12,179	11,898	10,409	10,371	10,547
201001_s_at	9,848	10,092	10,294	11,191	10,910	11,393
201002_s_at	11,458	11,211	11,627	10,535	10,866	10,906
201003_x_at	10,629	10,706	10,607	10,617	10,511	10,540
201004_at	10,291	10,093	10,431	10,880	10,921	11,600
201005_at	11,458	12,095	11,611	8,808	10,035	10,237
201006_at	7,430	7,648	7,971	8,029	7,787	8,071
201007_at	11,344	11,851	11,489	11,676	12,144	12,149
201008_s_at	8,319	9,191	8,862	6,808	6,113	7,791
201009_s_at	9,405	10,303	9,395	8,407	8,178	8,817
201010_s_at	10,336	11,609	10,780	8,870	8,454	9,067
201011_at	9,923	9,808	10,241	10,599	10,301	10,636
201012_at	7,534	7,034	6,813	12,721	13,241	12,832
201013_s_at	12,717	12,760	12,609	11,136	11,555	11,440
201014_s_at	12,812	12,995	12,422	11,769	11,554	11,453
201015_s_at	8,812	8,827	8,772	6,947	7,443	7,390
201016_at	10,945	10,703	10,920	11,406	10,848	10,877
201017_at	11,058	11,008	10,738	9,527	9,138	9,102
201018_at	11,577	11,960	11,681	10,298	10,526	9,714
201019_s_at	12,534	12,215	12,354	12,159	12,158	12,092
201020_at	9,573	9,051	9,006	8,123	8,075	8,399
201021_s_at	10,188	10,252	10,220	11,519	11,614	11,937

201022_s_at	12,558	12,612	12,780	12,734	13,427	13,405
201023_at	10,731	10,827	10,534	10,136	10,719	10,747
201024_x_at	11,738	11,910	11,301	10,540	11,450	10,041
201025_at	9,462	9,436	8,980	7,851	9,030	7,426
201026_at	8,794	8,554	8,231	7,485	8,525	6,720
201027_s_at	9,935	10,058	9,172	8,602	9,299	7,925
201028_s_at	7,762	8,267	8,032	9,542	9,617	10,383
201029_s_at	10,716	10,982	10,741	12,158	12,316	12,694
201030_x_at	14,911	15,007	14,780	13,540	13,701	13,632
201031_s_at	12,940	12,792	12,794	11,759	12,507	11,673
201032_at	10,626	10,647	10,952	10,754	10,538	11,317
201033_x_at	14,600	14,628	14,669	14,033	14,208	14,307
201034_at	10,602	11,070	9,985	9,223	11,947	10,281
201035_s_at	9,840	9,579	9,301	9,306	9,009	9,046
201036_s_at	11,273	11,352	11,470	10,411	10,221	10,225
201037_at	10,558	10,700	10,514	11,934	11,643	12,004
201038_s_at	11,154	10,719	10,870	9,232	9,423	8,965
201039_s_at	8,732	8,657	8,605	8,744	8,080	8,952
201040_at	9,924	9,681	9,465	10,060	9,392	10,546
201041_s_at	9,635	11,242	10,774	13,028	13,559	13,434
201042_at	6,014	6,952	6,728	8,781	7,953	8,707
201043_s_at	6,808	6,343	6,049	6,864	6,340	6,627
201044_x_at	7,203	7,097	7,469	8,568	8,225	8,307
201045_s_at	6,852	6,404	6,479	7,343	7,435	7,694
201046_s_at	10,175	10,485	10,442	10,506	9,941	10,371
201047_x_at	10,785	10,971	10,497	10,701	11,213	11,298
201048_x_at	5,710	6,144	6,243	6,765	6,775	6,909
201049_s_at	14,404	14,543	14,495	14,009	14,083	14,313
201050_at	8,557	8,418	8,631	8,226	7,894	8,820
201051_at	12,935	12,737	12,718	10,569	10,443	10,677
201052_s_at	9,016	8,565	9,193	9,679	9,106	9,638
201053_s_at	9,605	9,478	10,031	9,854	9,633	9,767
201054_at	12,365	11,861	12,322	10,620	10,637	10,879
201055_s_at	9,845	9,507	9,846	8,931	8,766	8,835
201056_at	8,830	9,233	8,491	8,121	8,980	7,932
201057_s_at	9,462	9,656	8,892	8,202	9,878	8,349
201058_s_at	7,859	7,513	7,502	8,694	9,671	10,856
201059_at	11,639	11,789	11,901	11,654	12,154	11,862
201060_x_at	10,391	10,559	10,669	10,340	10,131	10,401
201061_s_at	11,123	11,597	11,788	10,846	11,192	11,071
201062_at	6,024	5,840	6,049	6,074	6,296	6,138
201063_at	10,794	10,924	10,555	10,536	11,352	11,146
201064_s_at	11,335	11,010	11,758	10,618	10,732	10,916
201065_s_at	12,590	12,478	12,485	10,027	10,727	10,542
201066_at	11,044	11,332	11,372	11,774	11,329	11,866
201067_at	10,994	10,785	10,621	8,722	9,698	9,149
201068_s_at	13,233	13,104	12,941	12,645	12,906	12,617
201069_at	9,591	9,168	9,492	10,669	10,041	10,321
201070_x_at	10,434	10,667	10,521	9,135	9,668	8,725
201071_x_at	13,570	13,831	13,219	11,123	12,290	11,056
201072_s_at	10,023	9,894	10,229	7,969	7,620	7,868
201073_s_at	8,966	9,047	9,143	7,315	7,178	7,333
201074_at	12,520	12,424	12,662	9,822	10,388	9,855
201075_s_at	10,082	9,723	9,767	7,087	7,136	6,800
201076_at	11,477	11,246	11,544	11,126	10,690	11,362
201077_s_at	11,810	11,824	12,030	12,591	11,870	12,615

201078_at	11,142	11,012	11,173	11,287	11,423	11,742
201079_at	7,853	7,956	8,048	7,989	7,510	8,070
201080_at	8,949	8,654	8,944	8,388	8,294	8,484
201081_s_at	7,461	7,398	7,630	7,836	7,784	7,726
201082_s_at	8,443	7,814	8,292	8,263	8,348	8,679
201083_s_at	9,401	9,046	8,813	8,292	8,957	7,813
201084_s_at	12,666	12,286	12,301	11,102	12,349	10,556
201085_s_at	12,157	12,238	12,347	10,138	11,277	10,614
201086_x_at	12,187	12,032	12,011	10,017	11,209	10,431
201087_at	8,619	8,342	8,714	9,125	9,608	9,722
201088_at	13,148	13,124	13,072	12,368	12,633	12,378
201089_at	10,179	10,090	9,979	10,050	10,065	9,969
201090_x_at	14,238	14,143	14,138	13,721	13,682	14,025
201091_s_at	12,790	12,681	12,298	12,297	12,591	12,384
201092_at	12,970	12,998	12,834	12,032	12,001	11,705
201093_x_at	10,020	10,026	10,100	10,565	10,493	10,616
201094_at	14,071	14,238	14,218	13,774	13,912	14,047
201095_at	9,040	9,155	9,433	10,162	10,419	10,775
201096_s_at	10,955	10,890	11,075	11,467	11,235	11,878
201097_s_at	13,430	13,446	13,508	13,138	13,744	13,724
201098_at	11,713	12,128	11,545	11,309	11,824	11,074
201099_at	11,058	11,303	11,192	8,395	9,524	7,783
201100_s_at	12,236	12,378	12,263	10,286	11,546	9,979
201101_s_at	9,951	9,833	9,862	9,265	9,446	8,609
201102_s_at	9,278	9,266	9,040	9,113	9,039	9,254
201103_x_at	11,498	11,409	11,346	11,296	12,213	11,490
201104_x_at	9,558	9,342	9,279	8,984	9,989	9,425
201105_at	7,474	8,064	7,534	13,287	12,984	13,684
201106_at	11,624	11,983	11,767	11,650	11,040	11,782
201107_s_at	6,560	6,164	6,499	7,385	7,275	8,079
201108_s_at	7,192	7,321	6,527	8,609	8,950	9,722
201109_s_at	5,846	6,836	6,699	8,385	9,001	9,727
201110_s_at	6,906	8,182	7,557	9,460	10,697	11,481
201111_at	12,992	13,112	12,795	11,492	12,130	10,889
201112_s_at	13,387	13,548	12,980	11,969	12,378	11,173
201113_at	11,762	11,695	12,077	11,749	11,159	11,723
201114_x_at	12,524	12,667	12,944	12,325	11,988	12,365
201115_at	10,319	10,106	10,352	10,330	9,651	10,138
201116_s_at	8,171	8,320	8,111	6,508	6,294	6,483
201117_s_at	8,606	8,737	8,598	3,677	4,651	4,006
201118_at	10,278	9,920	10,136	10,504	10,292	10,172
201119_s_at	11,734	11,628	11,612	11,781	11,172	12,105
201120_s_at	10,813	10,180	10,096	9,455	8,784	9,679
201121_s_at	13,441	13,340	13,300	11,939	12,298	12,487
201122_x_at	9,215	8,932	9,320	9,745	10,267	10,496
201123_s_at	8,887	8,627	8,965	8,881	9,158	10,096
201124_at	7,785	8,438	8,111	7,213	8,325	8,285
201125_s_at	11,536	11,667	11,968	11,980	12,629	12,755
201126_s_at	9,272	9,044	9,363	10,270	9,974	10,557
201127_s_at	9,485	9,304	9,082	9,352	9,434	9,739
201128_s_at	12,294	12,128	12,158	12,082	12,514	12,665
201129_at	12,495	12,643	11,981	9,862	9,875	9,699
201130_s_at	5,056	5,154	5,039	6,165	5,574	5,792
201131_s_at	9,617	10,465	10,479	4,414	4,664	4,730
201132_at	11,545	11,269	10,892	9,855	10,430	9,414
201133_s_at	9,173	9,770	8,548	8,154	10,013	8,591

201134_x_at	12,901	13,272	12,894	12,422	12,263	12,557
201135_at	11,391	11,363	11,822	12,075	11,868	12,309
201136_at	7,448	7,642	7,885	11,658	10,848	11,676
201137_s_at	9,517	9,387	9,462	10,167	8,262	9,265
201138_s_at	10,555	10,632	9,800	9,282	9,810	9,713
201139_s_at	12,043	12,022	11,078	10,497	11,659	11,186
201140_s_at	7,789	7,398	7,955	8,410	8,136	8,432
201141_at	6,425	6,862	6,564	9,104	9,747	8,478
201142_at	9,625	9,523	9,363	9,252	10,333	9,278
201143_s_at	10,141	10,033	9,814	9,055	9,663	9,597
201144_s_at	11,823	11,721	11,724	12,170	12,190	11,922
201145_at	10,897	11,141	11,007	11,263	10,613	11,526
201146_at	10,972	11,362	10,676	10,339	11,590	10,530
201147_s_at	5,985	6,079	5,801	8,523	8,012	8,436
201148_s_at	7,915	7,905	7,865	9,225	9,192	9,401
201149_s_at	6,692	6,900	6,706	7,664	7,189	7,596
201150_s_at	5,827	5,936	6,146	8,004	8,629	8,662
201151_s_at	5,428	4,777	4,668	7,029	8,907	7,372
201152_s_at	8,462	8,343	8,238	9,702	11,537	10,226
201153_s_at	8,881	8,851	8,587	10,621	12,255	11,033
201154_x_at	14,190	14,314	14,133	13,467	13,460	13,629
201155_s_at	10,274	10,025	10,289	10,199	9,831	10,402
201156_s_at	7,733	7,614	7,450	8,950	8,262	8,689
201157_s_at	10,059	10,131	10,022	11,016	10,471	10,957
201158_at	8,160	8,349	8,583	7,694	7,629	7,745
201159_s_at	7,504	7,332	7,314	7,251	7,526	7,157
201160_s_at	12,447	12,576	12,709	11,888	11,988	11,940
201161_s_at	12,087	12,215	12,130	10,837	10,313	10,785
201162_at	7,193	7,592	7,614	9,414	10,183	11,246
201163_s_at	7,248	7,534	8,182	11,083	12,097	13,098
201164_s_at	11,050	11,268	11,086	8,502	9,151	9,129
201165_s_at	10,977	11,094	11,278	8,496	9,309	9,222
201166_s_at	13,263	13,186	13,177	10,913	11,903	11,315
201167_x_at	6,722	6,211	6,069	7,496	7,322	7,804
201168_x_at	8,896	8,640	9,204	10,218	9,874	10,147
201169_s_at	6,391	6,343	6,573	7,160	7,093	7,404
201170_s_at	7,147	8,057	7,954	9,677	12,044	10,549
201171_at	4,665	4,838	5,198	5,701	5,701	5,777
201172_x_at	11,246	11,148	11,400	12,461	12,321	12,837
201173_x_at	12,589	12,149	12,553	11,270	10,877	11,153
201174_s_at	9,913	9,806	9,547	9,747	9,771	10,281
201175_at	11,053	10,532	11,205	10,899	10,506	11,083
201176_s_at	10,894	10,989	10,879	11,097	11,505	11,353
201177_s_at	11,717	11,863	11,333	10,732	11,008	10,572
201178_at	9,179	9,316	9,611	9,663	9,155	9,729
201179_s_at	9,761	9,809	9,150	9,202	9,376	9,179
201180_s_at	13,347	13,391	13,133	12,254	12,883	12,632
201181_at	10,242	10,730	10,006	8,574	8,640	8,204
201182_s_at	10,225	9,747	10,072	8,751	8,457	8,776
201183_s_at	11,489	11,061	11,200	9,318	9,270	9,320
201184_s_at	11,283	11,579	11,736	9,620	10,180	9,754
201185_at	9,694	9,840	9,917	11,387	12,277	12,208
201186_at	9,405	9,367	9,780	10,208	10,256	10,371
201187_s_at	6,842	6,588	6,991	7,162	7,259	7,323
201188_s_at	7,257	7,516	7,407	8,550	8,075	8,362
201189_s_at	8,838	9,012	8,987	10,311	9,714	9,585

201190_s_at	10,412	10,326	10,580	9,729	10,252	10,109
201191_at	9,295	9,141	9,121	8,428	8,530	8,641
201192_s_at	9,298	8,988	9,104	9,430	8,958	9,344
201193_at	13,154	13,230	12,983	11,235	11,750	11,575
201194_at	11,296	11,019	11,133	10,604	10,043	10,911
201195_s_at	10,270	10,361	10,910	9,433	9,737	10,291
201196_s_at	10,223	10,256	10,103	11,011	10,749	10,716
201197_at	10,638	10,480	10,305	9,661	10,130	9,695
201198_s_at	11,810	11,725	11,414	10,457	11,008	10,262
201199_s_at	11,847	12,070	11,778	10,690	11,373	10,448
201200_at	9,907	10,336	9,407	10,510	10,712	10,724
201201_at	11,436	11,371	11,541	12,164	11,526	12,081
201202_at	12,786	12,706	12,489	12,111	11,935	11,983
201203_s_at	6,807	6,489	6,941	7,169	7,334	7,772
201204_s_at	7,360	7,381	7,516	8,315	8,944	8,814
201205_at	6,473	6,420	6,771	7,253	7,233	7,568
201206_s_at	7,912	8,035	8,073	8,717	8,874	9,645
201207_at	8,431	8,666	8,848	9,252	9,215	9,575
201208_s_at	7,361	7,609	7,681	8,895	8,539	8,892
201209_at	12,049	12,204	12,194	10,890	10,321	10,587
201210_at	13,206	13,078	12,838	11,466	12,252	11,081
201211_s_at	9,193	8,179	8,421	8,290	7,755	7,705
201212_at	8,913	9,098	8,822	7,755	8,048	8,732
201213_at	9,422	9,137	9,597	9,234	8,499	8,804
201214_s_at	10,177	10,072	10,260	10,280	10,349	10,418
201215_at	13,142	13,219	13,023	11,264	12,201	11,098
201216_at	10,892	11,089	11,276	10,580	9,791	10,447
201217_x_at	14,417	14,675	14,439	13,795	13,856	14,058
201218_at	11,858	11,678	11,807	10,448	10,492	10,399
201219_at	9,202	9,213	9,354	8,396	8,923	8,220
201220_x_at	11,880	11,999	12,191	11,450	11,361	11,300
201221_s_at	11,278	10,792	10,722	10,527	9,968	10,226
201222_s_at	11,620	11,212	11,382	10,328	10,699	11,081
201223_s_at	11,756	11,756	11,560	10,602	11,025	11,004
201224_s_at	9,780	9,774	10,286	9,293	8,564	9,410
201225_s_at	11,382	10,971	11,278	9,553	9,730	9,840
201226_at	11,657	11,447	11,476	11,712	11,134	11,736
201227_s_at	12,144	12,214	12,187	12,393	12,270	12,583
201228_s_at	8,443	8,413	9,129	8,283	7,382	7,747
201229_s_at	9,191	8,809	9,007	7,948	7,094	7,628
201230_s_at	10,881	11,112	11,160	9,119	9,452	9,226
201231_s_at	13,644	13,408	13,595	13,423	13,348	13,699
201232_s_at	11,281	11,083	11,263	11,124	10,572	11,223
201233_at	9,777	9,484	9,510	9,346	8,683	9,426
201234_at	10,385	10,286	10,541	10,898	10,510	11,095
201235_s_at	6,527	6,720	6,825	6,836	6,842	6,942
201236_s_at	8,536	9,661	9,439	8,104	8,207	8,019
201237_at	10,859	11,026	10,430	9,452	10,068	10,123
201238_s_at	12,112	12,409	11,776	11,355	12,058	12,140
201239_s_at	10,979	10,644	10,782	11,440	11,104	11,191
201240_s_at	12,607	12,466	12,698	12,365	12,503	12,363
201241_at	12,946	12,911	12,282	11,223	11,862	10,711
201242_s_at	8,820	8,769	8,508	9,609	9,808	8,758
201243_s_at	11,096	11,209	10,807	10,895	10,991	10,300
201244_s_at	11,213	11,098	11,254	10,220	10,141	10,302
201245_s_at	9,568	9,187	9,429	9,675	8,794	9,751

201246_s_at	8,540	7,913	8,378	7,899	7,043	7,476
201247_at	9,766	10,058	9,919	9,347	9,572	9,615
201248_s_at	8,045	8,252	8,148	7,953	7,879	7,963
201249_at	5,406	5,510	5,840	5,973	6,073	6,195
201250_s_at	9,539	9,330	10,153	9,099	8,694	9,435
201251_at	10,901	10,096	10,438	10,743	10,360	11,047
201252_at	10,064	9,767	10,252	10,473	9,665	10,335
201253_s_at	11,305	11,519	12,037	11,843	11,673	12,167
201254_x_at	14,468	14,482	14,394	13,451	13,502	13,848
201255_x_at	10,578	10,444	10,775	9,996	9,933	10,168
201256_at	12,704	13,217	12,903	12,749	12,552	12,999
201257_x_at	14,654	14,759	14,621	14,000	14,331	14,352
201258_at	12,933	13,072	12,933	12,544	12,039	12,751
201259_s_at	9,211	9,354	9,082	10,292	9,807	10,190
201260_s_at	11,139	11,318	11,020	11,596	12,043	11,959
201261_x_at	6,236	6,641	6,127	9,152	9,485	10,100
201262_s_at	6,256	6,106	6,262	7,197	6,741	7,073
201263_at	11,907	12,085	11,764	10,740	11,071	10,614
201264_at	9,439	9,175	9,085	9,450	9,225	9,765
201265_at	6,649	6,245	6,499	7,270	7,190	7,399
201266_at	11,959	11,999	11,691	11,846	12,815	12,291
201267_s_at	10,547	10,041	10,431	10,322	9,494	10,166
201268_at	11,789	11,982	12,002	12,176	11,711	12,284
201269_s_at	7,851	7,530	7,902	7,905	7,968	8,382
201270_x_at	11,034	10,805	11,232	10,252	10,105	10,372
201271_s_at	9,963	9,728	9,957	9,433	8,844	9,331
201272_at	12,526	12,517	12,521	13,409	12,838	13,622
201273_s_at	13,064	13,138	12,838	12,366	12,783	12,793
201274_at	12,087	12,380	12,337	12,173	11,949	12,334
201275_at	12,158	12,119	11,780	11,627	11,126	11,638
201276_at	8,178	8,240	8,156	7,260	7,540	7,808
201277_s_at	13,324	12,751	13,069	12,586	12,354	12,585
201278_at	7,735	7,878	7,163	8,669	10,548	9,362
201279_s_at	6,799	7,476	6,980	8,332	8,975	8,745
201280_s_at	6,935	6,908	6,475	8,101	9,920	9,098
201281_at	9,513	9,037	9,703	9,208	8,550	9,336
201282_at	7,859	7,420	7,739	8,054	8,030	8,157
201283_s_at	5,987	5,479	5,770	6,461	6,657	6,738
201284_s_at	9,803	9,963	9,805	9,820	9,160	9,670
201285_at	12,273	12,423	12,261	9,941	9,920	10,070
201286_at	9,417	9,047	9,643	7,652	7,116	7,343
201287_s_at	9,735	9,036	9,556	8,639	7,790	8,132
201288_at	7,030	7,363	7,299	9,452	9,133	9,696
201289_at	10,751	12,704	12,173	12,721	12,528	13,023
201290_at	13,780	13,962	13,754	13,182	13,193	13,283
201291_s_at	12,374	12,745	11,630	9,780	10,497	8,230
201292_at	11,731	12,193	10,906	9,386	10,304	7,931
201293_x_at	14,458	14,480	14,474	13,849	13,970	14,139
201294_s_at	9,026	8,904	7,388	7,026	8,629	8,211
201295_s_at	5,614	5,877	5,018	4,107	4,986	4,711
201296_s_at	10,457	10,820	9,889	8,796	10,524	9,598
201297_s_at	8,511	8,916	8,325	8,707	8,164	8,558
201298_s_at	10,134	10,532	10,249	11,178	10,472	11,109
201299_s_at	9,619	9,894	9,159	8,176	8,099	8,296
201300_s_at	10,561	10,667	10,666	11,787	12,138	12,113
201301_s_at	8,561	8,977	8,548	11,006	10,926	11,062

201302_at	8,546	8,774	8,129	9,633	10,254	10,216
201303_at	11,863	11,433	11,826	11,425	11,118	11,213
201304_at	11,642	11,654	11,162	10,699	10,934	11,196
201305_x_at	11,905	12,189	12,009	12,404	11,827	12,215
201306_s_at	12,761	13,072	12,866	12,668	12,878	12,819
201307_at	11,028	10,767	10,459	10,149	11,208	10,610
201308_s_at	6,512	5,968	5,685	5,549	5,469	5,736
201309_x_at	7,868	8,002	7,689	8,191	7,962	9,141
201310_s_at	13,189	13,065	13,353	11,446	12,198	12,898
201311_s_at	9,745	9,794	9,537	8,159	8,585	8,532
201312_s_at	12,144	12,019	11,748	10,103	11,598	11,132
201313_at	11,366	11,469	10,874	7,728	6,424	6,878
201314_at	9,344	9,312	9,496	8,940	8,250	9,123
201315_x_at	11,183	11,257	11,448	11,283	9,857	11,662
201316_at	11,541	11,670	11,424	9,410	9,580	9,516
201317_s_at	13,094	13,263	13,059	11,933	12,144	12,215
201318_s_at	11,979	12,010	11,402	12,632	13,082	13,152
201319_at	10,935	11,114	10,627	11,448	11,883	12,024
201320_at	8,756	8,364	8,837	8,502	7,673	8,019
201321_s_at	8,339	7,802	8,217	7,912	7,759	7,676
201322_at	13,798	13,780	13,912	12,877	12,829	13,195
201323_at	11,258	10,883	11,276	11,203	10,615	10,784
201324_at	5,593	5,287	4,968	12,166	12,657	12,371
201325_s_at	5,353	5,853	5,347	11,044	11,199	11,103
201326_at	12,611	12,741	12,489	11,209	11,714	10,926
201327_s_at	13,111	13,135	12,834	11,543	11,940	11,476
201328_at	9,193	9,496	9,745	7,491	7,702	6,643
201329_s_at	8,219	8,308	8,543	8,388	8,064	7,865
201330_at	11,413	11,483	11,342	10,825	11,433	10,618
201331_s_at	9,434	9,526	9,805	10,500	10,148	10,666
201332_s_at	6,954	6,868	6,872	7,407	6,998	7,329
201333_s_at	7,097	7,080	7,358	7,786	8,302	7,452
201334_s_at	10,390	10,193	10,237	9,949	11,594	10,011
201335_s_at	5,978	6,047	5,839	5,892	6,200	5,785
201336_at	10,532	10,601	10,522	11,568	11,856	11,856
201337_s_at	7,981	7,939	7,738	8,643	7,890	8,421
201338_x_at	13,316	13,408	13,607	13,126	13,254	13,129
201339_s_at	11,842	12,327	11,326	10,265	11,072	11,126
201340_s_at	9,502	9,083	9,572	7,827	7,723	8,960
201341_at	10,965	10,579	10,855	8,397	9,756	9,455
201342_at	10,361	10,125	10,244	10,451	9,886	10,466
201343_at	11,100	11,183	11,119	10,645	10,550	10,943
201344_at	8,588	8,456	8,992	7,259	7,188	7,424
201345_s_at	10,400	10,698	10,789	11,036	10,736	11,007
201346_at	11,964	11,869	12,098	9,967	10,239	9,792
201347_x_at	10,407	10,537	10,471	10,499	9,957	10,855
201348_at	8,448	8,450	8,784	6,798	7,851	8,258
201349_at	8,931	9,219	9,017	8,723	9,054	8,612
201350_at	10,048	9,847	10,151	8,829	8,657	9,379
201351_s_at	12,335	12,206	11,722	10,988	11,963	10,819
201352_at	12,427	12,457	12,108	11,166	12,314	11,290
201353_s_at	8,350	8,138	8,211	6,913	6,618	6,993
201354_s_at	8,400	8,554	8,329	7,654	7,543	7,552
201355_s_at	7,561	7,432	7,457	8,206	8,290	8,490
201356_at	11,383	11,500	11,846	10,645	10,489	10,642
201357_s_at	7,593	7,844	7,952	7,464	7,392	7,513

201358_s_at	11,985	12,491	11,940	12,311	13,114	12,642
201359_at	11,409	11,973	11,054	11,312	12,336	11,725
201360_at	8,600	8,610	8,546	8,756	8,765	9,376
201361_at	9,805	9,213	9,821	9,623	9,077	9,654
201362_at	11,067	11,321	10,267	8,473	8,606	7,712
201363_s_at	10,511	10,623	9,581	7,992	7,889	7,513
201364_s_at	10,229	10,287	10,619	8,522	7,918	8,515
201365_at	10,736	10,323	10,555	8,949	8,461	8,759
201366_at	10,866	11,300	11,090	11,775	12,334	12,319
201367_s_at	5,032	4,417	4,441	4,783	4,696	4,581
201368_at	11,442	11,337	11,457	9,819	12,007	11,341
201369_s_at	7,739	7,245	7,129	7,720	8,253	8,184
201370_s_at	10,087	9,952	9,738	7,362	7,899	6,717
201371_s_at	12,008	12,174	11,802	10,204	11,322	10,189
201372_s_at	5,948	6,553	6,040	4,955	4,724	4,853
201373_at	9,741	8,764	9,604	10,209	10,565	10,652
201374_x_at	5,392	5,147	5,183	5,793	5,929	5,874
201375_s_at	12,740	12,816	12,647	12,040	12,238	12,561
201376_s_at	9,135	9,071	9,033	9,728	8,870	9,075
201377_at	11,797	11,066	11,887	10,468	10,613	10,488
201378_s_at	11,124	10,228	11,143	9,733	9,533	9,638
201379_s_at	10,413	10,241	10,540	10,528	10,365	10,904
201380_at	8,064	8,159	8,674	9,735	8,902	9,555
201381_x_at	12,431	12,321	12,427	11,950	11,377	11,326
201382_at	7,857	7,606	7,609	8,546	7,802	7,893
201383_s_at	7,492	7,925	7,640	7,859	7,505	7,624
201384_s_at	9,712	10,157	9,426	9,603	10,449	9,733
201385_at	13,676	13,614	13,326	12,581	13,265	12,445
201386_s_at	12,599	12,608	12,298	10,608	11,352	10,332
201387_s_at	12,327	12,156	12,212	11,376	11,601	12,316
201388_at	9,022	8,734	8,916	8,974	8,132	8,844
201389_at	8,246	8,102	7,977	10,793	10,952	11,724
201390_s_at	10,701	10,309	10,780	11,739	11,256	11,929
201391_at	11,310	11,553	11,498	11,402	10,969	11,312
201392_s_at	7,262	7,237	7,747	8,552	8,755	8,638
201393_s_at	9,783	9,599	9,921	10,051	10,362	10,069
201394_s_at	9,905	9,727	9,398	7,829	8,288	7,914
201395_at	12,231	12,194	12,189	10,717	11,508	10,908
201396_s_at	9,467	9,114	9,545	9,320	8,826	9,130
201397_at	12,122	12,158	12,031	8,649	8,379	9,435
201398_s_at	11,166	11,307	11,011	11,776	12,847	12,849
201399_s_at	8,636	8,750	8,254	9,904	10,168	10,572
201400_at	11,612	11,355	11,619	11,534	11,118	11,869
201401_s_at	6,733	6,698	6,883	7,673	7,788	7,792
201402_at	8,753	8,654	8,806	8,927	8,984	9,022
201403_s_at	11,863	11,818	11,560	11,596	11,567	12,375
201404_x_at	8,212	7,919	8,258	8,492	8,386	8,555
201405_s_at	11,207	10,984	11,114	11,615	11,260	11,724
201406_at	12,825	13,246	12,941	12,036	11,265	12,064
201407_s_at	10,601	10,534	10,553	10,281	10,685	10,537
201408_at	10,194	10,632	10,127	8,856	9,660	8,745
201409_s_at	10,043	10,320	10,015	8,806	9,392	8,804
201410_at	12,114	12,383	12,421	11,643	12,075	11,955
201411_s_at	10,207	10,260	10,029	9,913	9,405	9,875
201412_at	6,519	7,497	6,797	9,962	10,092	10,616
201413_at	11,672	12,065	11,311	9,750	10,581	9,835

201414_s_at	11,001	10,761	10,713	9,999	9,754	9,367
201415_at	10,257	10,514	10,387	10,867	10,546	10,981
201416_at	10,619	10,846	11,045	6,097	7,568	7,048
201417_at	11,782	11,694	11,904	7,186	8,688	8,570
201418_s_at	9,550	9,553	9,772	8,434	8,112	8,252
201419_at	9,721	9,429	9,895	9,223	8,753	9,254
201420_s_at	10,216	9,675	10,715	10,244	9,402	10,205
201421_s_at	10,933	10,424	11,476	10,017	9,859	10,141
201422_at	9,327	9,448	9,311	10,546	9,474	10,150
201423_s_at	10,006	9,861	9,928	9,818	10,253	9,771
201424_s_at	10,639	10,377	10,391	10,216	10,863	10,098
201425_at	10,570	10,946	10,896	9,856	8,956	9,307
201426_s_at	12,359	12,476	12,361	13,773	13,991	14,156
201427_s_at	10,659	11,323	10,244	4,632	8,878	6,386
201428_at	6,683	7,095	6,770	7,255	7,022	7,289
201429_s_at	14,876	14,901	14,875	14,212	14,396	14,438
201430_s_at	7,973	7,592	8,464	6,761	6,375	6,555
201431_s_at	11,797	11,986	12,353	9,258	9,532	9,172
201432_at	6,171	5,740	5,896	11,303	11,529	11,960
201433_s_at	10,973	10,704	11,512	12,202	11,748	12,209
201434_at	10,059	10,114	10,117	9,830	10,005	9,831
201435_s_at	10,597	10,293	9,979	10,108	10,077	10,199
201436_at	10,452	10,548	10,205	9,374	9,298	8,941
201437_s_at	10,943	10,889	10,545	11,053	11,146	11,029
201438_at	6,809	7,135	7,146	12,876	13,915	13,797
201439_at	7,196	7,425	7,522	8,076	8,048	7,991
201440_at	10,764	10,349	10,766	9,504	9,077	9,230
201441_at	11,068	10,958	11,087	11,196	10,700	11,461
201442_s_at	6,347	6,188	6,314	6,626	6,590	6,888
201443_s_at	12,414	12,481	12,179	11,762	12,296	12,166
201444_s_at	9,569	9,658	9,159	9,702	9,181	9,791
201445_at	11,066	11,072	10,827	9,505	9,946	10,020
201446_s_at	10,297	10,631	10,024	7,997	8,483	7,795
201447_at	10,185	10,225	9,743	7,791	8,072	7,588
201448_at	11,588	11,582	10,944	7,769	8,889	7,773
201449_at	10,778	10,690	10,202	5,978	7,498	6,185
201450_s_at	10,851	10,905	10,515	8,382	9,315	8,307
201451_x_at	6,437	6,641	6,747	7,005	6,918	6,899
201452_at	5,773	6,025	5,932	6,316	6,021	6,216
201453_x_at	13,313	13,586	13,281	12,498	12,619	12,718
201454_s_at	10,938	11,194	11,015	10,091	11,254	10,672
201455_s_at	10,607	10,611	10,561	9,559	10,638	10,199
201456_s_at	9,945	9,928	9,492	9,663	9,504	9,510
201457_x_at	11,428	11,763	11,288	12,151	11,752	11,965
201458_s_at	10,744	10,758	10,422	10,017	10,500	10,253
201459_at	10,950	10,839	11,033	11,296	10,816	11,344
201460_at	9,498	9,698	10,038	9,332	9,300	9,505
201461_s_at	6,344	6,210	6,972	7,315	5,945	7,368
201462_at	11,219	11,162	10,831	9,313	10,096	10,027
201463_s_at	12,599	12,932	13,051	12,991	12,869	13,231
201464_x_at	9,647	11,142	10,879	10,796	11,218	10,428
201465_s_at	7,621	8,959	8,676	9,024	8,567	8,830
201466_s_at	8,775	10,734	10,428	10,382	10,058	9,514
201467_s_at	8,290	8,072	8,585	11,766	11,811	12,596
201468_s_at	9,902	9,732	10,595	13,063	13,707	13,842
201469_s_at	8,276	8,011	8,185	8,350	8,193	8,663

201470_at	11,198	11,760	11,957	13,296	12,933	13,231
201471_s_at	9,761	9,701	9,890	12,050	11,185	11,933
201472_at	12,354	12,781	12,266	11,533	11,851	11,736
201473_at	6,450	7,530	7,079	7,258	8,039	8,057
201474_s_at	7,508	6,706	7,095	9,665	9,127	10,153
201475_x_at	11,391	11,211	11,128	9,737	9,620	10,098
201476_s_at	9,500	9,760	9,073	8,615	8,170	8,074
201477_s_at	12,156	12,356	11,831	10,970	11,120	10,366
201478_s_at	11,573	11,358	11,357	10,350	10,081	9,903
201479_at	12,824	12,740	12,804	11,996	11,876	11,643
201480_s_at	9,376	9,099	9,600	8,991	8,960	9,261
201481_s_at	9,365	9,316	9,590	10,151	11,136	11,498
201482_at	9,205	9,239	9,001	10,423	10,726	10,881
201483_s_at	8,902	9,194	9,247	9,201	8,368	8,902
201484_at	9,388	9,270	9,670	8,830	8,480	9,026
201485_s_at	11,561	11,683	10,935	9,675	9,574	10,049
201486_at	12,601	12,796	12,261	11,507	11,573	11,865
201487_at	11,776	11,935	11,888	11,447	10,068	10,373
201488_x_at	11,811	11,977	11,592	11,150	11,033	11,188
201489_at	10,454	10,364	10,673	12,702	11,662	12,377
201490_s_at	8,536	8,280	8,303	10,706	9,024	10,202
201491_at	11,274	10,855	11,359	11,302	10,729	11,204
201492_s_at	14,956	15,035	15,005	14,346	14,537	14,616
201493_s_at	11,063	11,052	10,849	9,300	9,865	8,575
201494_at	9,725	10,069	9,840	9,823	10,319	10,378
201495_x_at	6,503	6,680	6,575	7,182	7,228	7,459
201496_x_at	6,075	6,417	5,784	7,239	7,253	7,639
201497_x_at	7,949	8,250	8,085	8,185	8,104	8,292
201498_at	11,426	11,022	11,342	9,564	10,132	9,261
201499_s_at	11,323	11,331	11,482	10,175	10,769	10,074
201500_s_at	9,769	9,906	10,495	11,021	10,768	11,265
201501_s_at	11,370	11,425	11,373	10,665	11,181	11,006
201502_s_at	9,164	9,864	9,568	11,009	10,597	10,363
201503_at	11,944	11,695	11,933	11,291	11,255	11,197
201504_s_at	9,212	9,197	9,139	8,992	8,230	8,762
201505_at	9,757	9,954	9,567	9,674	10,915	9,948
201506_at	9,101	9,312	9,225	13,722	13,224	13,460
201507_at	9,589	9,528	9,829	10,519	10,268	10,651
201508_at	7,561	7,325	7,266	9,396	10,230	10,556
201509_at	10,602	10,301	10,576	10,454	9,909	10,490
201510_at	6,672	7,531	6,890	6,998	7,090	7,114
201511_at	9,945	9,991	10,219	10,203	9,882	10,260
201512_s_at	9,941	10,016	10,053	9,951	9,885	10,256
201513_at	10,060	9,699	9,868	8,439	8,353	7,868
201514_s_at	10,976	10,290	10,440	10,398	9,745	10,074
201515_s_at	11,850	11,706	11,740	10,405	10,252	10,202
201516_at	9,368	9,146	9,718	10,781	10,053	10,985
201517_at	11,360	11,554	10,988	9,935	10,377	9,740
201518_at	12,114	12,097	11,791	10,131	10,265	9,925
201519_at	10,633	10,695	10,670	10,095	10,830	9,991
201520_s_at	10,240	10,597	10,297	10,647	10,333	10,563
201521_s_at	9,081	9,139	9,041	9,626	8,963	9,107
201522_x_at	13,428	13,224	13,371	9,954	9,992	10,735
201523_x_at	9,318	9,039	9,124	9,696	8,629	9,524
201524_x_at	11,221	10,934	11,163	11,212	10,953	11,174
201525_at	6,973	6,633	6,997	7,417	7,470	7,313

201526_at	9,810	9,525	9,527	9,221	8,727	9,273
201527_at	10,735	10,432	10,445	11,191	10,436	11,267
201528_at	12,557	12,585	12,327	11,272	11,373	10,879
201529_s_at	11,110	11,325	11,020	9,460	9,411	9,074
201530_x_at	13,414	13,299	13,394	13,276	12,965	13,291
201531_at	8,343	8,871	8,749	9,416	10,687	10,115
201532_at	12,265	12,526	11,937	11,833	11,639	12,023
201533_at	9,972	9,949	10,171	9,070	9,750	9,624
201534_s_at	8,381	8,718	8,112	7,503	8,754	8,149
201535_at	9,919	10,342	9,806	8,174	9,463	9,020
201536_at	8,663	9,118	8,871	8,774	8,666	8,875
201537_s_at	7,150	7,006	7,469	9,179	8,381	8,554
201538_s_at	7,368	7,258	6,901	8,399	7,688	8,260
201539_s_at	7,358	7,509	7,158	8,163	8,196	8,297
201540_at	11,884	12,008	11,902	10,589	12,132	12,093
201541_s_at	10,070	9,833	10,063	10,852	10,477	11,248
201542_at	11,211	11,289	11,168	10,413	12,069	11,367
201543_s_at	8,817	9,092	8,968	10,517	9,769	10,616
201544_x_at	12,160	12,124	12,189	11,232	10,736	11,073
201545_s_at	8,501	8,496	8,598	7,564	7,332	7,415
201546_at	11,378	11,407	11,236	10,725	11,699	10,557
201547_at	5,335	5,320	4,971	6,392	6,072	6,537
201548_s_at	11,112	11,395	11,350	8,114	8,302	8,647
201549_x_at	11,136	11,352	11,512	8,175	8,956	8,641
201550_x_at	14,411	14,474	14,539	14,014	14,360	14,459
201551_s_at	6,371	6,029	6,567	7,025	6,660	7,070
201552_at	10,104	9,705	10,365	11,077	10,836	11,241
201553_s_at	12,629	12,326	13,317	13,405	13,806	13,759
201554_x_at	9,699	9,834	9,730	9,720	9,223	10,086
201555_at	11,081	10,745	10,865	9,240	8,576	8,556
201556_s_at	6,494	6,279	6,867	6,304	6,057	6,671
201557_at	7,406	7,288	7,379	7,591	7,527	7,501
201558_at	11,554	11,725	12,168	11,474	11,202	11,037
201559_s_at	8,741	8,682	8,480	8,216	8,477	9,094
201560_at	12,442	12,636	12,312	10,890	12,539	12,235
201561_s_at	9,093	9,196	9,604	9,246	9,406	9,342
201562_s_at	8,940	8,858	9,109	8,916	7,797	8,552
201563_at	9,884	9,594	9,857	9,538	8,542	9,186
201564_s_at	9,517	9,007	9,344	8,379	8,089	8,994
201565_s_at	10,277	10,536	10,232	8,548	10,599	10,773
201566_x_at	9,921	10,486	10,339	7,696	10,876	10,514
201567_s_at	10,511	11,057	9,856	9,353	10,945	8,904
201568_at	10,210	10,291	10,587	11,566	11,434	11,724
201569_s_at	10,924	11,124	11,267	11,593	10,785	11,421
201570_at	10,302	10,176	10,391	10,756	10,046	10,591
201571_s_at	9,112	9,570	9,324	10,358	9,949	10,306
201572_x_at	10,505	10,370	10,718	10,836	10,377	10,784
201573_s_at	10,853	10,694	10,951	11,280	11,687	11,175
201574_at	11,695	11,319	11,184	10,806	11,782	11,013
201575_at	11,374	11,523	11,118	10,618	10,866	10,234
201576_s_at	10,236	10,656	10,712	10,852	10,676	11,224
201577_at	12,580	12,321	12,737	12,830	12,269	13,015
201578_at	12,642	12,454	12,680	6,892	7,152	6,983
201579_at	11,033	10,720	11,183	9,122	10,346	9,111
201580_s_at	7,812	7,805	8,020	8,712	8,440	8,515
201581_at	9,801	9,624	9,655	9,118	10,421	8,822

201582_at	9,189	9,211	9,414	8,421	8,676	8,178
201583_s_at	10,239	10,219	10,005	9,524	9,715	9,625
201584_s_at	11,600	11,320	12,221	11,684	11,132	11,315
201585_s_at	10,278	9,648	10,114	8,729	8,277	8,590
201586_s_at	11,800	11,038	11,637	10,328	10,156	10,066
201587_s_at	10,726	10,706	11,341	11,570	11,210	11,602
201588_at	11,819	12,024	12,098	12,098	11,874	12,157
201589_at	12,732	12,466	12,503	10,705	10,731	9,713
201590_x_at	12,111	12,566	12,770	14,141	14,526	14,529
201591_s_at	12,073	12,222	12,353	10,021	10,582	10,837
201592_at	12,604	13,129	12,493	11,728	12,089	11,817
201593_s_at	11,126	11,311	11,015	10,410	10,316	10,211
201594_s_at	11,540	11,378	11,755	11,014	11,510	11,158
201595_s_at	11,720	11,814	11,453	10,615	11,325	10,542
201596_x_at	12,066	12,245	12,542	7,827	7,621	7,976
201597_at	13,229	13,416	13,267	13,127	13,144	13,469
201598_s_at	8,482	8,409	8,879	8,226	8,443	8,785
201599_at	11,643	12,185	11,923	12,473	12,868	12,862
201600_at	13,787	13,797	13,923	12,629	12,299	12,664
201601_x_at	10,510	10,094	10,915	8,677	7,630	9,038
201602_s_at	8,321	8,719	8,487	7,446	8,803	7,976
201603_at	10,887	11,105	10,577	9,123	10,847	9,169
201604_s_at	9,107	9,257	8,803	7,049	8,949	7,457
201605_x_at	9,407	9,381	9,727	9,161	9,086	9,918
201606_s_at	11,847	11,783	11,748	11,846	11,896	11,835
201607_at	9,731	9,975	9,463	9,225	9,397	9,073
201608_s_at	11,261	11,343	11,236	11,019	11,210	10,898
201609_x_at	10,236	9,848	10,463	10,675	10,543	10,873
201610_at	5,234	5,585	5,624	6,216	6,196	6,382
201611_s_at	9,603	9,326	9,887	9,246	9,323	9,262
201612_at	12,216	12,503	12,348	11,706	11,808	11,991
201613_s_at	8,256	8,382	8,825	7,947	7,905	7,961
201614_s_at	11,427	10,843	11,245	10,090	9,493	9,491
201615_x_at	8,921	8,781	8,983	9,441	9,638	9,890
201616_s_at	9,842	9,506	8,916	8,369	9,103	8,827
201617_x_at	9,257	8,841	8,033	8,002	8,942	8,311
201618_x_at	9,081	8,832	9,238	10,183	9,810	10,264
201619_at	12,652	12,733	12,343	11,869	12,108	12,161
201620_at	9,760	9,709	9,603	8,778	9,037	9,333
201621_at	7,895	7,634	7,693	9,231	8,879	9,248
201622_at	10,676	10,793	10,786	10,312	10,440	10,746
201623_s_at	11,643	11,973	11,467	10,941	11,089	11,042
201624_at	9,047	9,331	8,937	8,745	8,736	8,372
201625_s_at	9,136	9,359	8,699	10,905	9,833	10,642
201626_at	11,421	11,466	10,751	11,937	11,983	12,183
201627_s_at	9,325	9,905	9,020	10,229	9,720	10,117
201628_s_at	10,098	10,146	10,067	11,378	11,266	11,999
201629_s_at	10,237	10,350	10,162	10,194	9,657	10,193
201630_s_at	12,625	12,737	12,501	11,231	11,255	11,308
201631_s_at	9,982	10,837	10,793	13,422	12,757	12,366
201632_at	10,589	10,463	10,528	10,327	10,119	10,295
201633_s_at	9,400	8,755	8,760	8,780	8,397	8,705
201634_s_at	10,830	10,403	10,877	10,767	10,321	10,684
201635_s_at	10,200	10,259	9,455	8,588	8,666	8,113
201636_at	11,223	11,302	10,324	7,965	8,894	7,845
201637_s_at	12,216	12,663	11,381	10,229	11,143	9,416

201638_s_at	6,373	6,237	6,159	6,564	6,517	6,651
201639_s_at	9,219	8,961	9,263	8,698	8,392	8,855
201640_x_at	8,532	8,209	8,579	8,986	8,692	8,897
201641_at	10,760	11,168	10,940	7,033	6,471	7,130
201642_at	10,753	11,003	10,905	10,999	10,745	11,075
201643_x_at	10,581	10,754	10,894	9,224	9,552	9,394
201644_at	8,738	8,459	8,904	9,199	8,692	9,404
201645_at	10,160	8,568	10,208	9,897	9,761	8,923
201646_at	7,057	7,294	6,700	6,729	7,461	6,922
201647_s_at	5,551	5,741	4,928	6,922	7,378	7,136
201648_at	9,385	9,921	9,561	10,788	11,658	10,867
201649_at	9,237	9,205	9,041	10,339	9,972	10,565
201650_at	7,836	8,138	7,778	6,003	7,639	7,159
201651_s_at	11,527	11,501	11,584	11,409	11,443	11,432
201652_at	11,422	11,637	11,523	10,872	11,115	11,014
201653_at	11,812	12,117	11,743	12,338	12,697	12,779
201654_s_at	6,791	6,406	6,811	7,040	6,991	7,042
201655_s_at	7,781	7,879	7,872	8,631	8,738	8,936
201656_at	11,125	11,456	10,901	6,319	6,557	5,899
201657_at	9,700	10,164	9,568	9,416	9,219	9,863
201658_at	10,404	10,823	10,260	10,158	10,665	10,582
201659_s_at	10,084	10,388	9,890	11,048	10,680	11,300
201660_at	10,867	11,112	10,551	9,768	10,765	9,348
201661_s_at	10,769	10,989	10,449	9,095	10,204	8,620
201662_s_at	10,242	10,196	9,757	10,033	10,508	9,454
201663_s_at	10,932	10,979	10,063	8,481	9,932	7,272
201664_at	12,296	12,302	11,802	9,821	11,050	8,712
201665_x_at	14,035	14,207	14,209	13,448	13,506	13,721
201666_at	9,510	9,679	9,756	12,346	12,340	12,806
201667_at	13,925	13,781	13,809	11,597	12,713	12,296
201668_x_at	7,971	8,044	8,109	7,664	7,546	8,246
201669_s_at	11,016	11,113	11,324	10,461	11,472	11,639
201670_s_at	9,116	8,792	8,667	7,538	7,914	8,801
201671_x_at	10,474	10,377	10,403	9,246	9,910	9,311
201672_s_at	11,841	11,941	12,214	12,165	12,500	12,206
201673_s_at	9,082	8,998	9,218	9,936	9,501	10,181
201674_s_at	9,834	10,015	10,404	8,781	8,465	8,611
201675_at	9,169	9,182	9,928	8,337	7,805	8,109
201676_x_at	13,181	13,353	13,188	12,971	12,892	13,080
201677_at	8,079	7,960	7,887	7,159	6,951	7,109
201678_s_at	10,079	10,179	10,183	9,669	9,772	9,825
201679_at	7,339	7,251	7,606	8,016	7,615	8,191
201680_x_at	10,950	10,643	11,194	10,234	9,898	9,912
201681_s_at	10,875	10,593	11,324	9,886	10,632	10,253
201682_at	11,789	11,936	11,455	10,590	10,872	10,127
201683_x_at	8,089	8,078	8,220	8,457	7,782	8,574
201684_s_at	9,469	9,417	9,615	10,156	10,058	10,355
201685_s_at	8,348	8,484	8,352	7,764	7,957	7,990
201686_x_at	8,707	8,438	8,386	7,665	7,902	7,562
201687_s_at	12,296	12,048	11,575	9,277	9,735	8,684
201688_s_at	7,963	8,079	7,891	5,360	5,360	5,241
201689_s_at	7,283	7,361	7,279	4,083	3,840	3,873
201690_s_at	9,530	9,950	9,822	6,750	6,272	6,151
201691_s_at	4,021	5,202	4,057	4,076	4,217	4,178
201692_at	9,597	9,475	9,756	10,624	10,045	10,561
201693_s_at	8,436	9,605	10,367	7,540	7,197	8,831

201694_s_at	10,645	11,594	11,842	10,193	10,348	10,818
201695_s_at	10,074	10,556	10,520	11,506	11,168	11,357
201696_at	11,858	11,781	11,965	10,272	10,127	10,000
201697_s_at	11,091	10,989	11,477	9,910	10,007	9,763
201698_s_at	12,458	11,748	12,012	10,741	10,136	10,966
201699_at	12,424	12,620	12,020	11,395	12,082	11,750
201700_at	9,230	8,993	9,383	10,847	10,667	11,373
201701_s_at	9,841	9,937	10,024	10,691	10,984	11,078
201702_s_at	8,846	8,693	9,231	8,531	8,496	8,857
201703_s_at	9,341	9,454	9,913	8,285	7,893	8,045
201704_at	9,647	9,740	10,129	10,879	11,036	11,416
201705_at	11,827	11,810	11,633	10,495	10,692	10,701
201706_s_at	10,039	10,155	10,237	9,536	8,982	9,515
201707_at	10,194	10,476	10,319	9,167	9,441	9,357
201708_s_at	9,741	9,695	9,363	8,362	7,771	8,463
201709_s_at	11,427	11,528	11,660	10,774	10,428	10,832
201710_at	9,771	9,215	9,563	8,668	8,250	8,489
201711_x_at	10,647	11,026	10,423	8,440	9,143	8,212
201712_s_at	10,328	10,264	9,921	7,774	8,302	7,096
201713_s_at	11,661	11,663	11,019	10,129	11,136	9,534
201714_at	9,943	9,689	9,810	10,392	9,162	9,992
201715_s_at	9,324	8,771	9,191	8,648	8,389	8,588
201716_at	9,539	9,302	9,230	9,706	9,338	9,857
201717_at	10,839	10,683	11,254	10,713	9,932	10,497
201718_s_at	7,960	7,899	7,720	6,707	6,612	6,072
201719_s_at	10,396	10,408	10,365	9,051	9,819	8,477
201720_s_at	7,481	7,360	7,112	9,571	8,142	8,353
201721_s_at	7,071	7,492	6,747	10,969	7,052	8,496
201722_s_at	10,673	10,699	10,317	9,643	11,993	10,479
201723_s_at	10,273	10,521	10,194	9,421	11,200	10,245
201724_s_at	10,247	10,107	9,842	8,827	11,268	9,404
201725_at	11,734	11,423	11,348	11,095	10,775	10,868
201726_at	12,449	12,099	12,314	11,001	10,996	11,047
201727_s_at	9,392	9,397	9,287	9,079	8,611	8,889
201728_s_at	8,485	8,242	8,608	8,784	8,239	9,146
201729_s_at	9,223	8,878	9,165	9,155	9,412	9,236
201730_s_at	10,414	10,484	9,645	7,873	9,517	7,303
201731_s_at	10,831	10,930	10,130	8,631	9,548	8,421
201732_s_at	7,657	8,181	7,539	7,677	8,055	7,964
201733_at	7,279	7,888	7,268	7,354	7,582	7,022
201734_at	9,886	10,428	9,703	10,111	10,965	9,949
201735_s_at	9,719	9,921	9,478	9,203	9,936	9,153
201736_s_at	10,082	10,347	10,246	10,400	10,936	10,761
201737_s_at	10,109	10,012	9,858	8,510	9,749	8,903
201738_at	12,310	12,573	12,307	12,047	11,532	12,052
201739_at	9,123	9,688	9,392	11,495	12,275	11,685
201740_at	11,547	11,310	11,508	11,020	10,063	11,039
201741_x_at	6,515	6,717	6,249	6,024	6,167	5,899
201742_x_at	9,758	8,980	9,108	8,999	8,307	8,665
201743_at	6,074	6,383	5,951	6,821	6,453	6,645
201744_s_at	3,633	3,735	4,158	8,219	10,862	8,502
201745_at	10,501	10,936	10,316	11,381	12,237	11,607
201746_at	8,291	8,295	8,836	6,641	6,079	6,447
201747_s_at	9,257	8,279	8,478	7,552	7,546	7,693
201748_s_at	10,775	9,862	10,507	8,798	8,763	8,545
201749_at	8,179	7,859	8,254	8,671	8,872	8,809

201750_s_at	7,350	7,180	7,369	7,893	7,506	7,840
201751_at	9,251	9,329	9,443	9,535	9,884	9,792
201752_s_at	9,411	9,689	9,068	8,305	10,292	9,068
201753_s_at	11,130	11,432	10,582	9,748	12,691	11,020
201754_at	11,594	11,895	11,580	11,592	11,310	11,847
201755_at	9,998	9,098	9,871	9,125	8,222	8,554
201756_at	11,501	11,320	11,543	9,632	8,680	9,463
201757_at	13,333	13,474	13,087	12,545	12,119	12,374
201758_at	11,059	11,257	11,212	11,094	11,108	11,219
201759_at	8,418	8,219	8,585	8,582	8,319	8,387
201760_s_at	10,685	10,878	10,940	11,442	11,805	11,822
201761_at	12,827	13,175	13,021	11,528	11,836	11,877
201762_s_at	9,642	9,812	9,610	10,969	10,560	10,911
201763_s_at	8,813	8,326	8,778	8,955	8,532	8,657
201764_at	9,175	9,337	9,257	10,117	8,945	9,472
201765_s_at	9,344	9,544	9,568	9,927	9,543	10,146
201766_at	6,364	5,980	6,142	7,188	6,705	6,790
201767_s_at	9,593	9,420	9,651	9,979	9,437	9,971
201768_s_at	8,881	8,994	8,806	8,770	8,869	8,694
201769_at	11,568	11,252	11,124	10,308	11,374	10,915
201770_at	10,773	10,840	11,148	10,313	9,644	10,264
201771_at	10,857	10,913	11,361	11,572	11,395	12,088
201772_at	9,792	9,919	9,928	9,667	9,530	9,631
201773_at	11,794	11,740	11,674	8,827	9,977	9,006
201774_s_at	11,730	11,952	11,905	9,821	8,515	8,954
201775_s_at	8,017	7,986	7,880	7,589	7,464	7,469
201776_s_at	8,618	8,425	8,543	8,119	7,794	7,569
201777_s_at	8,782	8,756	9,056	8,677	8,278	8,468
201778_s_at	10,944	11,097	11,107	9,548	10,702	10,061
201779_s_at	9,340	9,869	9,163	9,138	9,261	9,073
201780_s_at	8,748	9,123	8,574	8,916	9,003	8,054
201781_s_at	7,969	8,289	7,950	8,051	7,605	7,925
201782_s_at	10,136	10,210	9,949	9,902	9,306	9,996
201783_s_at	8,840	8,876	9,023	9,322	8,969	9,541
201784_s_at	12,081	11,888	11,555	11,967	12,045	11,954
201785_at	7,388	7,306	7,297	7,637	7,763	7,736
201786_s_at	12,440	12,343	12,665	10,551	11,116	10,693
201787_at	9,685	9,479	9,718	7,265	6,406	7,034
201788_at	11,351	11,061	11,041	9,122	9,904	9,180
201789_at	4,617	4,426	4,559	4,878	5,165	4,828
201790_s_at	9,462	9,326	9,191	9,356	8,742	9,452
201791_s_at	10,632	10,359	10,385	10,194	9,665	10,448
201792_at	8,841	8,859	8,642	8,487	8,948	9,248
201793_x_at	8,271	8,186	8,600	8,121	8,178	8,614
201794_s_at	9,455	9,346	9,697	7,278	7,298	7,405
201795_at	12,785	12,824	12,614	11,467	11,578	11,432
201796_s_at	7,722	7,818	7,748	7,700	7,787	7,877
201797_s_at	9,346	9,095	9,459	9,242	8,217	9,356
201798_s_at	5,716	5,312	6,041	11,458	12,931	12,040
201799_s_at	9,345	9,148	9,572	8,707	8,603	8,783
201800_s_at	10,082	10,400	10,274	9,412	10,033	9,631
201801_s_at	7,746	7,246	7,752	7,080	6,637	6,889
201802_at	9,466	9,342	9,266	9,058	8,689	8,822
201803_at	12,567	12,573	12,273	11,061	11,718	10,652
201804_x_at	10,732	10,476	10,512	10,848	9,986	11,079
201805_at	9,270	9,176	9,125	10,239	9,505	9,976

201806_s_at	7,440	7,771	7,655	7,361	6,916	7,269
201807_at	11,648	11,804	11,418	11,217	12,115	11,075
201808_s_at	7,155	6,719	6,825	7,444	7,333	7,455
201809_s_at	7,203	6,848	6,890	9,474	9,400	9,852
201810_s_at	9,150	9,425	9,327	9,087	9,556	9,004
201811_x_at	9,808	9,991	10,068	9,640	10,531	9,740
201812_s_at	12,134	12,392	12,252	11,629	11,555	11,472
201813_s_at	9,083	9,535	9,168	8,031	9,140	8,326
201814_at	10,045	10,402	10,095	9,060	9,887	9,149
201815_s_at	7,720	7,545	7,399	7,900	7,914	7,815
201816_s_at	11,458	12,156	11,158	9,825	10,332	9,898
201817_at	10,700	10,439	10,872	10,357	10,732	10,521
201818_at	10,743	10,634	11,173	10,601	10,485	10,783
201819_at	8,706	8,543	8,440	8,722	7,940	8,484
201820_at	7,445	7,423	6,901	7,989	7,954	8,121
201821_s_at	10,981	10,530	10,036	10,884	10,593	10,966
201822_at	7,819	7,685	7,856	7,615	7,397	7,034
201823_s_at	8,439	8,947	8,489	9,950	9,512	10,129
201824_at	8,001	8,397	8,121	8,311	8,387	8,688
201825_s_at	9,852	9,727	9,498	8,868	8,385	8,441
201826_s_at	9,148	9,100	9,156	8,146	8,155	7,907
201827_at	9,397	9,309	9,252	9,551	8,659	9,200
201828_x_at	9,830	9,832	10,337	11,479	11,548	12,129
201829_at	10,265	10,174	10,085	8,701	9,118	8,610
201830_s_at	10,300	10,026	10,215	8,794	8,145	7,994
201831_s_at	10,778	11,184	10,486	9,610	10,559	9,492
201832_s_at	12,462	12,983	12,187	10,886	12,084	10,822
201833_at	11,386	11,499	10,903	10,067	10,003	9,900
201834_at	8,757	8,892	9,240	7,680	7,378	7,792
201835_s_at	6,729	6,785	6,776	6,516	6,249	6,681
201836_s_at	8,099	8,228	8,318	6,394	6,975	6,784
201837_s_at	10,045	10,238	10,009	9,622	9,758	9,799
201838_s_at	7,649	7,308	7,480	6,785	6,900	6,782
201839_s_at	12,718	12,978	13,055	5,617	5,200	5,349
201840_at	10,390	10,014	10,113	10,478	10,077	10,687
201841_s_at	10,419	10,160	10,133	10,666	10,873	11,926
201842_s_at	8,543	9,239	8,628	7,755	9,588	9,503
201843_s_at	5,647	7,247	6,346	5,079	7,803	6,986
201844_s_at	10,839	10,913	10,907	8,766	9,611	8,576
201845_s_at	11,486	11,659	11,493	8,658	9,232	8,218
201846_s_at	10,679	10,911	10,759	9,213	9,771	8,996
201847_at	9,594	10,135	9,658	11,410	11,957	11,837
201848_s_at	7,452	8,304	7,717	8,741	8,159	8,776
201849_at	10,901	11,928	11,232	11,567	11,987	12,272
201850_at	8,204	8,820	8,194	8,977	7,965	8,625
201851_at	9,350	9,099	9,758	9,711	9,609	10,210
201852_x_at	4,992	5,281	5,151	8,120	11,375	9,805
201853_s_at	10,374	10,604	10,558	9,974	9,883	9,911
201854_s_at	10,676	10,751	10,897	9,338	9,646	9,445
201855_s_at	10,685	10,183	10,611	9,054	9,936	9,114
201856_s_at	7,998	8,374	7,892	6,724	7,076	6,817
201857_at	12,209	12,265	11,939	11,005	11,993	11,187
201858_s_at	5,081	5,734	4,952	11,640	11,963	12,816
201859_at	4,372	5,818	5,690	12,933	13,522	13,555
201860_s_at	9,026	8,904	8,948	6,729	8,789	9,252
201861_s_at	10,271	10,287	9,992	9,966	10,566	9,419

201862_s_at	8,652	8,372	7,974	7,561	8,178	6,570
201863_at	11,148	11,149	11,161	10,503	10,400	10,610
201864_at	10,321	10,292	10,132	10,727	10,725	11,035
201865_x_at	10,568	10,497	10,703	9,918	11,235	10,266
201866_s_at	7,142	7,536	7,468	8,018	8,739	8,144
201867_s_at	9,829	9,316	9,432	7,685	7,416	7,621
201868_s_at	7,364	6,605	6,839	5,665	5,437	5,401
201869_s_at	8,970	9,059	8,980	7,585	7,795	7,884
201870_at	9,835	9,817	9,815	10,418	9,833	10,397
201871_s_at	7,857	7,574	8,125	10,241	9,541	10,114
201872_s_at	10,753	10,991	10,663	10,985	11,508	10,694
201873_s_at	10,878	10,687	10,377	10,495	11,235	10,215
201874_at	9,696	9,624	9,657	9,497	9,812	9,640
201875_s_at	9,114	8,949	8,860	8,804	8,926	8,767
201876_at	10,418	10,434	10,174	10,870	10,948	11,334
201877_s_at	10,612	10,907	10,186	9,196	10,260	9,393
201878_at	9,121	9,275	8,839	7,838	8,297	7,784
201879_at	9,124	9,314	9,127	7,524	7,813	8,002
201880_at	9,801	9,917	9,725	7,936	8,652	7,905
201881_s_at	9,654	9,970	9,640	8,304	8,835	8,552
201882_x_at	6,914	7,016	6,974	7,324	7,229	7,405
201883_s_at	8,471	8,759	8,820	9,445	9,566	9,675
201884_at	5,811	6,001	5,857	6,470	6,404	6,403
201885_s_at	10,428	10,722	10,915	11,748	12,054	12,229
201886_at	8,796	9,134	9,017	8,577	8,147	8,558
201887_at	9,786	9,975	9,797	10,359	10,640	9,997
201888_s_at	7,250	7,182	7,450	8,617	8,736	8,340
201889_at	11,542	11,494	11,143	10,704	11,976	10,873
201890_at	12,972	12,551	13,000	12,360	12,482	12,106
201891_s_at	12,192	12,931	12,321	13,656	13,984	13,939
201892_s_at	12,625	12,869	12,821	11,845	11,588	11,956
201893_x_at	7,176	7,469	7,278	11,742	12,115	12,620
201894_s_at	10,477	10,382	10,001	10,328	10,941	11,008
201895_at	9,539	9,283	9,610	8,822	8,258	9,194
201896_s_at	10,667	10,446	10,757	8,840	8,214	8,762
201897_s_at	13,424	13,558	13,252	11,240	10,447	10,847
201898_s_at	9,370	9,577	9,573	9,699	9,069	9,775
201899_s_at	11,564	11,585	11,430	11,006	11,049	11,453
201900_s_at	11,442	12,077	11,788	11,104	10,898	11,357
201901_s_at	10,231	10,195	10,165	9,175	9,120	9,513
201902_s_at	7,435	7,397	7,698	7,303	7,333	7,122
201903_at	12,185	11,864	12,125	11,748	11,347	12,091
201904_s_at	9,476	9,495	9,574	8,684	8,608	8,987
201905_s_at	7,609	7,545	7,831	8,118	7,873	8,076
201906_s_at	10,379	10,766	10,816	8,727	8,982	9,089
201907_x_at	7,834	7,797	7,869	8,128	8,150	8,044
201908_at	9,568	9,218	9,711	7,562	8,200	8,329
201909_at	8,182	8,172	7,425	8,737	13,223	13,435
201910_at	10,079	9,997	10,193	8,887	8,782	9,047
201911_s_at	8,380	8,534	8,511	8,551	8,181	8,791
201912_s_at	10,863	10,518	10,505	10,721	10,634	10,662
201913_s_at	10,071	9,866	10,250	10,488	9,650	10,170
201914_s_at	10,434	10,385	9,835	9,121	10,028	8,723
201915_at	8,619	8,807	8,296	6,674	7,466	6,389
201916_s_at	10,961	11,014	10,654	9,475	10,826	9,163
201917_s_at	11,847	12,057	11,375	9,201	9,594	9,355

201918_at	10,310	10,719	10,125	8,504	9,091	8,587
201919_at	11,132	11,596	10,882	8,541	10,246	9,000
201920_at	11,727	11,407	11,517	11,723	12,380	12,525
201921_at	12,519	12,690	12,366	12,464	12,791	12,949
201922_at	11,370	11,530	11,154	11,403	11,850	11,662
201923_at	12,963	12,931	13,308	12,560	12,466	13,134
201924_at	9,976	10,348	10,392	8,888	9,606	8,791
201925_s_at	10,681	11,250	10,509	6,749	9,643	7,161
201926_s_at	9,427	9,632	9,263	6,938	7,860	7,090
201927_s_at	7,994	7,946	8,330	6,915	6,164	7,032
201928_at	10,607	10,740	10,656	9,294	9,558	9,243
201929_s_at	7,632	7,190	7,144	6,381	6,239	6,807
201930_at	12,782	12,658	12,676	10,824	11,380	10,481
201931_at	11,488	11,923	11,370	11,554	11,694	11,729
201932_at	9,922	9,841	10,121	9,855	9,221	10,030
201933_at	9,881	9,446	9,952	10,049	9,540	10,078
201934_at	12,032	11,899	12,191	10,739	10,928	10,775
201935_s_at	9,013	9,151	8,768	7,552	8,911	7,773
201936_s_at	8,562	8,598	8,565	7,735	8,885	7,678
201937_s_at	8,895	9,130	8,715	8,998	7,851	8,718
201938_at	13,040	12,779	13,191	12,794	13,018	13,326
201939_at	7,329	7,906	7,061	9,338	9,294	9,073
201940_at	8,875	9,234	8,899	7,632	8,486	7,264
201941_at	9,168	9,553	9,105	8,219	9,696	8,528
201942_s_at	7,064	7,689	7,297	7,091	7,028	6,903
201943_s_at	8,339	8,455	8,305	7,044	8,081	7,252
201944_at	9,988	10,925	10,035	11,169	11,276	10,954
201945_at	8,211	8,462	8,314	8,778	8,684	9,093
201946_s_at	11,079	10,868	10,855	10,059	9,352	9,879
201947_s_at	13,992	13,930	13,880	12,680	12,826	12,592
201948_at	11,582	11,696	10,931	9,942	10,490	9,128
201949_x_at	10,949	10,818	11,107	11,142	11,271	11,561
201950_x_at	9,439	9,206	9,510	10,074	9,386	10,232
201951_at	6,642	7,021	6,215	9,240	11,091	10,620
201952_at	8,854	9,460	8,716	11,016	13,092	12,430
201953_at	8,910	8,804	8,840	10,653	10,171	11,072
201954_at	9,976	9,792	9,676	11,444	11,047	11,646
201955_at	10,756	11,099	10,843	10,409	10,474	10,645
201956_s_at	11,760	11,613	11,510	10,112	10,403	10,156
201957_at	8,187	8,027	8,107	7,565	7,522	7,585
201958_s_at	5,874	6,244	6,345	6,674	6,434	6,460
201959_s_at	8,364	8,484	8,437	8,152	9,654	8,280
201960_s_at	10,032	10,177	9,697	9,684	11,320	9,523
201961_s_at	8,365	8,175	8,557	7,966	7,368	8,190
201962_s_at	9,215	9,216	9,348	7,700	7,411	8,032
201963_at	7,202	8,315	7,382	10,537	10,658	10,264
201964_at	10,419	10,076	9,941	7,422	8,677	7,004
201965_s_at	9,695	9,525	9,369	8,398	9,205	8,566
201966_at	12,622	12,710	12,734	12,212	11,661	12,178
201967_at	9,299	9,370	8,920	7,684	8,171	8,003
201968_s_at	12,294	12,826	12,717	11,601	11,763	12,120
201969_at	11,883	11,580	11,396	7,840	7,712	7,491
201970_s_at	13,404	13,186	12,648	10,214	9,599	8,863
201971_s_at	7,722	7,425	6,780	7,037	6,960	6,634
201972_at	12,235	12,228	12,208	11,347	12,369	11,543
201973_s_at	13,003	13,151	12,877	11,289	11,834	11,668

201974_s_at	7,528	7,369	7,099	8,031	8,284	7,813
201975_at	8,744	8,570	8,340	8,693	10,052	8,103
201976_s_at	11,749	11,918	12,172	8,737	10,633	9,276
201977_s_at	7,424	7,904	7,521	7,143	6,890	7,170
201978_s_at	8,283	8,456	8,450	8,382	8,346	8,321
201979_s_at	7,755	7,852	7,837	7,649	7,220	7,672
201980_s_at	9,111	9,276	9,492	10,885	10,694	11,250
201981_at	8,960	8,842	8,389	8,605	7,503	7,829
201982_s_at	7,701	7,866	7,403	7,566	7,550	7,613
201983_s_at	7,865	7,927	7,952	9,766	10,715	10,025
201984_s_at	8,343	8,657	8,676	9,878	10,006	9,636
201985_at	10,028	9,825	9,616	9,791	10,365	9,338
201986_at	9,435	9,423	9,237	7,807	8,596	7,575
201987_at	10,917	10,843	10,765	9,144	10,321	9,010
201988_s_at	8,627	8,961	8,618	7,897	7,595	7,646
201989_s_at	12,057	12,560	12,101	10,306	10,736	10,336
201990_s_at	9,841	10,387	9,721	7,506	7,879	7,006
201991_s_at	10,922	11,243	10,455	10,779	12,218	10,488
201992_s_at	7,133	7,277	6,593	7,768	8,619	7,448
201993_x_at	12,285	12,011	12,090	11,243	11,748	11,402
201994_at	13,877	13,940	13,729	13,058	13,490	13,513
201995_at	8,757	8,719	8,993	10,753	10,570	10,403
201996_s_at	8,748	8,173	9,292	7,304	7,757	7,599
201997_s_at	10,089	9,858	10,291	8,341	9,528	8,585
201998_at	10,686	10,976	11,465	8,461	8,017	8,198
201999_s_at	12,009	12,414	12,106	12,785	12,629	13,078
202000_at	10,575	10,573	10,650	9,876	9,275	9,604
202001_s_at	12,140	12,551	12,195	12,418	12,005	12,350
202002_at	7,037	7,222	7,038	7,125	6,890	7,060
202003_s_at	11,255	11,297	11,090	10,202	10,436	10,611
202004_x_at	10,442	10,024	10,375	10,116	9,781	10,284
202005_at	7,619	7,453	7,755	8,065	8,012	8,290
202006_at	11,892	12,198	11,700	10,853	11,762	10,448
202007_at	10,118	10,330	10,231	9,352	11,064	11,484
202008_s_at	7,800	7,798	7,776	7,245	7,796	8,221
202009_at	8,625	8,610	8,631	9,687	8,734	9,409
202010_s_at	8,734	8,884	8,891	9,279	9,239	9,377
202011_at	11,069	11,298	11,286	9,474	11,094	9,680
202012_s_at	10,136	10,492	10,359	10,742	10,209	10,685
202013_s_at	9,217	9,323	9,647	9,602	9,916	9,778
202014_at	6,650	7,076	7,430	8,483	7,955	8,576
202015_x_at	4,377	4,439	4,546	4,390	4,707	4,403
202016_at	11,287	10,632	10,020	10,604	11,835	10,210
202017_at	7,505	6,797	7,278	7,389	7,123	7,843
202018_s_at	6,990	7,208	7,563	7,344	7,216	7,589
202019_s_at	8,188	8,196	8,302	8,348	8,011	8,281
202020_s_at	11,059	11,597	10,919	10,080	10,527	9,605
202021_x_at	12,519	12,832	12,739	12,185	12,491	12,724
202022_at	8,803	9,063	8,858	8,427	8,525	8,697
202023_at	6,620	7,208	6,929	6,408	7,102	6,697
202024_at	10,528	10,319	10,545	10,434	9,573	10,318
202025_x_at	8,371	8,342	8,451	9,142	9,030	9,337
202026_at	10,802	10,890	10,800	11,465	11,352	11,537
202027_at	9,425	9,312	9,744	10,123	10,196	10,710
202028_s_at	9,585	9,945	10,085	10,000	10,257	9,988
202029_x_at	13,083	12,993	13,026	12,716	12,608	13,147

202030_at	8,574	8,485	8,745	9,144	8,415	9,120
202031_s_at	11,981	12,128	12,437	11,617	11,365	11,493
202032_s_at	8,819	8,646	8,769	8,555	8,507	8,855
202033_s_at	10,199	10,405	10,113	7,914	9,595	7,694
202034_x_at	10,411	10,599	9,899	8,812	9,943	7,875
202035_s_at	10,238	9,841	10,243	6,082	6,771	7,295
202036_s_at	11,725	11,285	11,788	6,609	7,769	8,089
202037_s_at	13,374	12,933	13,103	7,012	8,870	8,819
202038_at	10,856	10,887	10,829	10,136	11,085	10,395
202039_at	7,806	8,032	7,598	7,522	8,069	8,046
202040_s_at	10,398	10,324	10,414	7,577	8,943	7,399
202041_s_at	10,243	9,994	10,482	10,589	9,861	10,609
202042_at	10,255	9,860	10,374	11,270	11,056	11,355
202043_s_at	12,895	12,659	12,519	11,739	11,251	11,333
202044_at	7,982	7,936	7,917	8,373	8,436	8,585
202045_s_at	7,314	7,182	7,279	6,774	6,995	7,055
202046_s_at	6,441	6,093	6,260	6,794	6,454	6,794
202047_s_at	9,149	8,911	9,307	9,922	10,217	10,255
202048_s_at	9,709	9,285	9,730	10,042	10,733	10,501
202049_s_at	8,939	9,108	8,853	7,559	8,262	7,270
202050_s_at	9,783	9,797	9,800	7,946	9,027	7,992
202051_s_at	11,253	11,253	11,038	8,543	9,744	8,135
202052_s_at	10,717	10,488	10,241	10,613	11,951	10,697
202053_s_at	11,180	11,148	11,201	10,850	11,411	11,648
202054_s_at	10,109	9,947	10,366	9,402	10,670	9,849
202055_at	9,866	10,002	9,742	9,011	9,981	9,355
202056_at	9,397	9,558	9,508	8,069	9,164	8,479
202057_at	7,421	7,465	7,339	5,783	6,692	6,213
202058_s_at	7,715	7,774	7,540	7,601	7,097	7,739
202059_s_at	8,506	8,835	8,957	8,952	9,294	9,193
202060_at	10,662	10,844	10,324	9,540	10,420	9,080
202061_s_at	8,244	8,578	8,174	8,695	9,862	8,682
202062_s_at	8,327	8,112	7,447	7,446	8,257	8,048
202063_s_at	5,443	5,518	5,232	5,984	6,159	5,763
202064_s_at	7,026	6,810	7,307	7,283	7,596	7,217
202065_s_at	8,276	8,498	8,412	8,260	8,118	8,153
202066_at	10,807	11,093	10,907	9,485	10,620	9,533
202067_s_at	8,266	8,224	8,087	8,027	7,633	8,191
202068_s_at	11,165	11,421	11,363	10,088	10,292	10,579
202069_s_at	8,513	8,853	8,373	9,883	9,660	9,697
202070_s_at	7,988	8,287	7,983	8,833	8,719	8,837
202071_at	8,862	9,431	9,415	10,045	10,497	10,494
202072_at	9,338	9,323	9,407	9,085	8,666	9,380
202073_at	7,156	8,591	6,433	8,095	8,345	8,210
202074_s_at	7,914	8,991	7,071	10,019	10,560	10,045
202075_s_at	8,541	8,382	8,364	7,381	7,336	7,594
202076_at	11,721	12,191	11,547	11,484	12,215	11,311
202077_at	13,259	13,494	13,496	12,518	12,065	12,586
202078_at	10,796	10,639	10,812	10,365	9,988	10,141
202079_s_at	7,824	8,073	7,886	7,197	7,919	7,478
202080_s_at	10,404	10,639	10,540	9,013	10,082	9,758
202081_at	11,970	12,985	13,000	10,590	10,573	10,465
202082_s_at	8,856	9,070	8,992	9,113	9,045	10,042
202083_s_at	7,335	7,128	7,330	8,588	8,452	9,118
202084_s_at	10,653	10,296	10,382	9,746	10,431	10,730
202085_at	10,380	10,580	11,128	10,081	11,703	9,916

202086_at	7,592	7,527	7,969	7,660	7,294	7,458
202087_s_at	11,077	11,632	11,088	13,008	12,815	12,800
202088_at	12,123	11,941	11,880	11,651	12,370	11,732
202089_s_at	9,892	9,922	9,684	9,675	9,638	9,713
202090_s_at	11,991	12,273	12,293	12,382	12,257	12,717
202091_at	5,955	5,599	5,777	6,054	6,080	5,938
202092_s_at	8,511	8,292	8,594	9,201	8,798	9,667
202093_s_at	9,120	8,827	9,272	9,581	9,303	9,479
202094_at	9,920	9,774	10,424	9,033	8,782	8,405
202095_s_at	11,146	10,904	11,223	10,726	9,966	10,005
202096_s_at	7,820	8,627	8,132	11,280	10,841	11,948
202097_at	12,007	11,936	12,058	10,710	11,412	10,219
202098_s_at	9,901	9,867	10,018	9,239	8,858	9,531
202099_s_at	8,211	8,211	8,097	8,686	8,326	8,573
202100_at	10,496	10,787	10,154	11,422	11,792	11,406
202101_s_at	7,955	7,730	7,500	9,010	8,925	9,197
202102_s_at	10,091	9,673	10,268	8,596	8,818	8,958
202103_at	8,118	7,902	8,307	7,440	7,505	7,516
202104_s_at	9,722	9,558	9,653	8,895	9,171	9,370
202105_at	10,131	10,422	10,052	9,543	9,369	9,820
202106_at	8,947	8,974	9,094	9,152	9,689	9,568
202107_s_at	12,786	12,495	12,862	11,420	10,579	10,679
202108_at	9,654	9,749	9,979	10,098	9,276	9,869
202109_at	8,983	8,644	8,945	8,528	8,484	8,949
202110_at	12,154	12,440	12,232	12,011	11,685	12,192
202111_at	9,755	9,318	9,858	9,423	9,115	9,443
202112_at	7,348	7,945	7,240	7,221	7,408	7,805
202113_s_at	9,706	10,199	9,067	8,505	8,425	7,946
202114_at	9,827	10,338	9,562	9,047	9,093	8,490
202115_s_at	9,046	8,547	8,905	9,105	8,766	9,278
202116_at	10,125	9,996	10,403	8,762	9,818	9,622
202117_at	11,178	11,040	11,793	11,280	11,406	11,726
202118_s_at	8,359	8,599	8,162	9,659	9,808	9,734
202119_s_at	9,247	9,644	9,136	10,176	11,048	10,471
202120_x_at	9,986	9,766	9,983	10,362	9,932	10,545
202121_s_at	8,873	9,234	8,910	10,233	9,913	10,401
202122_s_at	9,574	9,809	9,611	10,959	10,299	11,290
202123_s_at	10,336	10,346	10,714	9,371	9,616	9,759
202124_s_at	8,351	8,114	8,201	8,720	9,470	8,916
202125_s_at	9,029	9,116	8,728	9,349	10,445	9,196
202126_at	10,447	10,179	9,896	8,896	9,769	8,344
202127_at	10,133	9,993	9,559	6,910	9,317	7,229
202128_at	9,260	9,053	9,479	8,344	8,328	8,571
202129_s_at	7,614	7,717	7,355	7,615	7,941	7,929
202130_at	10,019	10,352	10,078	9,599	10,710	9,929
202131_s_at	8,431	8,899	8,349	8,689	9,465	8,932
202132_at	7,655	8,111	7,979	8,499	8,945	8,920
202133_at	9,285	10,000	9,722	9,808	11,848	11,270
202134_s_at	7,456	7,436	7,910	9,338	8,596	9,251
202135_s_at	9,338	9,504	9,255	9,651	9,029	9,466
202136_at	10,081	10,253	10,155	10,108	11,096	10,201
202137_s_at	6,615	6,241	6,645	6,798	6,845	6,799
202138_x_at	12,604	12,466	12,996	12,062	11,498	11,844
202139_at	8,568	8,951	8,630	10,189	9,101	10,158
202140_s_at	8,520	8,559	8,359	8,760	8,109	8,478
202141_s_at	12,222	12,392	12,397	12,522	12,930	12,922

202142_at	10,470	10,539	10,372	10,782	10,852	11,042
202143_s_at	10,306	10,286	10,030	10,729	10,496	11,018
202144_s_at	12,307	12,529	12,780	12,087	11,841	12,073
202145_at	8,319	8,181	8,341	8,729	8,508	9,373
202146_at	11,703	11,554	11,585	8,599	10,364	9,634
202147_s_at	10,568	10,390	10,182	7,702	9,211	8,598
202148_s_at	9,846	9,781	9,974	8,987	9,269	10,356
202149_at	7,399	6,354	6,405	5,228	5,897	7,567
202150_s_at	7,356	7,063	7,229	7,715	7,556	8,326
202151_s_at	9,965	9,879	9,932	9,192	8,386	9,019
202152_x_at	8,729	8,520	8,642	9,392	8,794	9,012
202153_s_at	11,103	11,070	11,202	10,347	9,987	10,090
202154_x_at	12,018	11,804	11,701	11,678	11,224	11,920
202155_s_at	9,198	9,071	9,233	8,234	8,299	8,415
202156_s_at	7,767	7,684	7,844	5,866	5,713	5,634
202157_s_at	10,169	10,004	9,895	7,042	7,107	6,809
202158_s_at	8,754	8,253	8,208	5,009	5,520	5,076
202159_at	10,110	9,852	10,433	10,420	10,135	10,609
202160_at	10,525	10,189	10,792	8,328	9,934	8,972
202161_at	8,787	8,658	8,665	8,270	7,924	8,247
202162_s_at	8,766	8,946	8,513	9,229	8,740	8,942
202163_s_at	9,620	9,749	9,050	8,463	8,819	8,313
202164_s_at	9,418	9,632	8,772	7,431	7,695	7,401
202165_at	9,137	9,543	8,843	7,565	8,521	8,166
202166_s_at	8,463	9,004	8,627	8,716	8,480	9,237
202167_s_at	9,442	9,684	9,564	9,514	8,831	9,455
202168_at	10,362	10,386	10,143	10,660	10,834	10,899
202169_s_at	11,665	11,859	11,138	10,543	11,076	10,549
202170_s_at	10,284	10,659	10,218	10,796	10,484	10,395
202171_at	9,508	9,473	9,436	7,730	8,568	7,944
202172_at	10,167	9,835	9,855	8,333	8,913	8,055
202173_s_at	9,615	9,626	9,631	7,966	8,567	8,058
202174_s_at	11,076	11,116	10,506	8,606	10,273	7,620
202175_at	8,609	8,731	8,724	9,792	9,674	10,058
202176_at	9,633	9,467	9,553	8,382	7,816	8,004
202177_at	7,546	7,809	7,142	8,812	8,303	9,501
202178_at	8,719	9,005	8,781	6,338	6,194	6,372
202179_at	9,962	9,929	9,960	8,908	8,421	8,315
202180_s_at	7,479	7,828	7,455	9,764	9,663	10,377
202181_at	8,284	8,172	8,288	9,069	9,215	9,485
202182_at	8,721	8,656	8,874	8,727	8,493	8,953
202183_s_at	9,635	9,494	9,558	8,262	7,140	7,672
202184_s_at	12,003	12,280	12,008	9,592	9,954	8,693
202185_at	9,943	9,733	10,159	11,108	10,815	11,427
202186_x_at	6,562	6,306	6,306	6,838	6,906	6,967
202187_s_at	9,018	9,480	9,236	9,105	8,903	9,148
202188_at	11,509	11,472	11,628	10,462	9,735	10,072
202189_x_at	12,547	12,243	12,928	11,994	11,595	11,841
202190_at	10,378	10,672	10,424	9,415	9,129	9,152
202191_s_at	7,407	7,979	7,379	7,161	7,361	6,944
202192_s_at	8,140	8,233	8,150	8,236	8,029	8,284
202193_at	10,051	9,743	10,504	8,705	9,021	9,005
202194_at	11,365	11,553	11,005	11,186	12,474	11,724
202195_s_at	8,076	8,337	8,101	10,325	10,404	10,519
202196_s_at	6,932	6,951	6,260	6,473	5,144	6,599
202197_at	8,282	8,377	8,399	7,854	8,397	7,964

202198_s_at	6,115	6,191	5,744	6,268	6,091	6,260
202199_s_at	8,467	8,332	8,057	8,679	8,407	8,401
202200_s_at	10,925	10,971	10,773	10,119	10,993	9,731
202201_at	8,241	8,896	8,302	10,087	9,346	10,439
202202_s_at	5,776	5,242	5,232	10,469	12,032	11,205
202203_s_at	6,522	6,309	6,323	7,360	7,538	8,461
202204_s_at	7,996	8,276	8,128	8,438	7,842	8,452
202205_at	8,043	7,845	8,064	9,532	8,195	9,614
202206_at	8,998	8,841	8,935	6,315	8,003	7,080
202207_at	10,105	9,986	10,043	7,229	9,361	8,477
202208_s_at	8,701	8,581	8,487	7,771	7,961	7,760
202209_at	10,820	10,800	10,789	10,429	10,126	10,562
202210_x_at	8,344	8,275	8,666	8,538	8,208	8,295
202211_at	8,972	9,785	8,684	10,372	11,171	10,973
202212_at	9,885	9,548	9,957	10,159	9,588	10,210
202213_s_at	8,761	9,491	8,537	10,347	10,925	10,134
202214_s_at	11,307	11,895	11,162	10,731	11,900	10,926
202215_s_at	9,459	9,399	9,819	8,102	7,888	8,078
202216_x_at	8,518	8,528	8,625	8,257	7,953	8,043
202217_at	11,663	11,821	11,951	10,628	10,614	10,724
202218_s_at	9,298	9,125	9,344	9,073	8,675	8,925
202219_at	10,234	9,603	10,420	8,276	8,250	8,482
202220_at	11,667	11,809	11,603	9,920	10,401	9,897
202221_s_at	9,482	9,464	9,881	8,172	8,110	6,756
202222_s_at	7,796	7,785	7,633	8,229	8,549	8,574
202223_at	9,652	9,238	9,569	10,020	9,410	10,099
202224_at	8,755	8,580	8,518	8,247	8,534	8,510
202225_at	10,561	10,431	10,465	10,151	10,830	10,509
202226_s_at	7,841	7,561	7,508	7,625	7,476	7,566
202227_s_at	10,809	10,945	10,862	9,554	10,060	9,350
202228_s_at	12,872	12,924	12,923	12,357	12,676	12,514
202229_s_at	7,351	7,247	7,576	7,485	7,312	7,470
202230_s_at	11,913	11,665	12,318	10,499	10,387	10,622
202231_at	12,845	12,698	12,705	12,611	12,760	12,872
202232_s_at	11,919	11,845	11,470	11,620	11,494	11,925
202233_s_at	13,836	13,783	13,578	12,342	12,259	12,369
202234_s_at	11,110	11,427	11,487	9,133	9,515	9,214
202235_at	10,463	10,841	10,749	8,587	8,453	8,419
202236_s_at	11,277	10,506	10,655	8,320	8,421	8,474
202237_at	5,827	4,609	5,471	11,483	10,625	11,276
202238_s_at	6,749	6,775	6,757	10,408	9,570	10,469
202239_at	9,333	9,697	9,259	9,013	9,750	8,802
202240_at	9,419	9,484	9,990	8,843	8,034	8,137
202241_at	10,977	11,531	11,640	9,943	11,567	10,176
202242_at	9,928	10,470	9,716	6,534	6,546	7,159
202243_s_at	12,462	12,288	12,589	11,684	11,231	11,778
202244_at	13,568	13,609	13,889	12,820	12,521	12,831
202245_at	11,361	11,050	11,140	10,396	10,085	10,840
202246_s_at	11,851	11,592	12,262	11,303	9,945	10,723
202247_s_at	7,717	7,214	7,241	7,529	7,644	7,779
202248_at	8,123	7,816	7,920	7,886	7,611	7,780
202249_s_at	8,178	8,341	8,284	8,162	8,095	8,196
202250_s_at	8,594	8,677	8,529	7,323	7,472	7,644
202251_at	10,091	9,529	9,826	8,581	8,409	8,777
202252_at	13,016	13,256	13,063	13,125	13,208	12,985
202253_s_at	7,751	7,670	7,983	7,385	7,120	7,435

202254_at	7,552	7,814	7,674	6,513	7,322	7,007
202255_s_at	8,389	8,213	8,693	8,946	8,580	8,630
202256_at	10,059	9,972	10,235	9,668	9,582	9,662
202257_s_at	9,917	9,643	10,032	11,075	10,429	11,106
202258_s_at	11,231	12,042	10,710	9,944	10,484	9,693
202259_s_at	10,045	10,521	9,565	6,322	6,927	6,501
202260_s_at	10,498	10,867	10,795	10,558	10,397	10,608
202261_at	11,059	11,238	11,526	9,962	9,009	9,640
202262_x_at	8,443	8,163	7,953	8,413	8,167	8,525
202263_at	8,153	8,001	7,951	9,066	9,053	9,670
202264_s_at	9,603	9,047	9,635	9,826	9,220	9,527
202265_at	9,745	9,917	9,616	10,206	10,979	10,291
202266_at	11,052	11,232	10,919	11,430	11,707	11,060
202267_at	6,924	7,644	7,624	7,096	6,879	6,789
202268_s_at	13,330	13,185	12,678	11,177	11,631	10,581
202269_x_at	5,456	5,437	4,982	8,534	8,656	8,792
202270_at	5,276	5,703	5,220	9,426	10,603	9,480
202271_at	10,986	11,253	10,877	9,205	10,242	9,229
202272_s_at	11,185	11,116	11,314	9,646	9,622	9,375
202273_at	7,232	7,281	6,918	8,230	8,115	8,544
202274_at	6,598	6,410	6,286	7,249	6,746	7,346
202275_at	7,960	7,730	8,078	8,475	9,037	9,786
202276_at	10,959	10,950	10,738	10,188	10,276	10,374
202277_at	10,924	11,113	11,068	10,473	11,012	10,887
202278_s_at	8,180	7,849	7,417	6,768	6,766	7,369
202279_at	11,842	12,003	12,099	11,781	11,129	11,861
202280_at	5,577	5,342	5,214	5,965	5,457	5,646
202281_at	8,827	8,700	9,056	9,267	9,076	9,430
202282_at	12,622	12,334	12,830	11,473	11,006	11,666
202283_at	9,340	9,697	9,428	8,189	9,495	10,589
202284_s_at	7,791	8,165	8,013	11,242	11,213	11,926
202285_s_at	6,438	6,531	6,317	6,648	6,584	6,490
202286_s_at	5,588	5,222	5,535	7,101	5,625	6,682
202287_s_at	5,757	5,780	5,826	5,955	6,004	6,032
202288_at	8,733	9,035	9,036	7,717	8,094	7,976
202289_s_at	10,574	10,319	10,796	9,362	9,519	9,311
202290_at	7,775	7,736	7,909	8,326	7,571	8,060
202291_s_at	5,357	5,628	5,149	5,811	5,567	5,808
202292_x_at	10,230	9,937	10,448	10,104	9,829	10,214
202293_at	8,744	8,154	8,709	7,860	8,860	7,048
202294_at	9,094	8,740	9,308	8,436	9,251	7,821
202295_s_at	8,935	9,365	9,094	7,206	7,057	7,265
202296_s_at	9,417	9,001	9,410	11,155	10,532	11,480
202297_s_at	9,906	9,614	10,378	11,974	11,899	12,321
202298_at	12,449	12,575	12,157	11,583	11,310	11,804
202299_s_at	11,996	12,457	12,094	12,275	12,171	12,445
202300_at	11,591	11,824	11,449	11,639	11,537	11,813
202301_s_at	9,643	10,265	9,562	8,342	8,712	8,149
202302_s_at	11,990	12,438	11,601	9,838	11,262	9,706
202303_x_at	11,354	11,209	10,485	8,671	9,194	8,132
202304_at	9,468	10,093	9,518	9,007	10,596	9,151
202305_s_at	9,878	10,191	9,749	10,317	10,278	10,630
202306_at	12,402	12,105	12,746	11,779	11,691	12,011
202307_s_at	6,568	7,278	7,324	9,653	9,573	9,879
202308_at	8,786	8,513	8,938	8,157	8,041	8,191
202309_at	11,349	11,153	11,590	10,411	10,483	10,311

202310_s_at	6,262	5,837	6,163	10,070	11,277	11,840
202311_s_at	4,951	4,595	5,258	6,155	7,230	8,768
202312_s_at	7,165	6,912	6,925	7,630	7,878	7,904
202313_at	11,539	11,699	11,387	10,405	10,599	10,447
202314_at	10,563	10,447	10,281	10,900	11,099	10,861
202315_s_at	8,304	7,674	8,130	7,942	7,317	7,944
202316_x_at	8,264	8,065	8,245	7,880	7,775	7,962
202317_s_at	10,186	10,583	10,398	9,453	9,715	9,708
202318_s_at	9,530	9,481	9,214	8,219	9,499	7,532
202319_at	8,057	7,964	7,552	6,218	6,150	5,554
202320_at	9,348	9,079	9,575	8,307	8,630	8,792
202321_at	8,474	8,270	7,838	6,290	7,023	6,687
202322_s_at	10,747	10,963	10,310	8,694	9,356	8,989
202323_s_at	8,171	8,607	8,102	8,721	8,710	8,613
202324_s_at	11,314	11,757	10,860	10,395	11,392	10,650
202325_s_at	12,751	12,758	12,573	12,201	11,868	12,271
202326_at	9,581	9,519	9,933	8,223	7,710	8,212
202327_s_at	6,829	6,890	7,087	7,619	7,593	7,612
202328_s_at	8,461	8,515	8,697	8,857	9,165	9,428
202329_at	10,223	10,224	10,570	10,867	10,290	10,718
202330_s_at	11,104	10,961	10,886	9,420	8,265	8,948
202331_at	9,194	9,181	9,364	8,912	8,827	9,079
202332_at	9,801	10,069	10,092	9,383	9,079	9,575
202333_s_at	9,426	9,957	9,496	10,358	10,174	10,439
202334_s_at	8,788	8,745	8,490	9,571	9,296	9,252
202335_s_at	4,379	4,199	4,574	4,564	4,406	4,376
202336_s_at	10,540	10,479	10,247	10,870	12,401	11,461
202337_at	10,922	10,844	11,019	9,391	8,636	9,205
202338_at	8,792	8,544	8,979	10,100	9,360	9,484
202339_at	7,098	6,745	6,743	7,233	7,082	7,269
202340_x_at	8,079	8,635	8,122	8,019	8,225	8,269
202341_s_at	11,092	11,058	10,894	7,658	8,412	7,790
202342_s_at	11,682	11,000	10,850	5,445	7,723	6,393
202343_x_at	10,511	10,719	10,809	10,533	10,297	10,710
202344_at	8,801	8,526	8,776	8,580	8,389	8,734
202345_s_at	11,379	11,173	11,260	12,377	11,791	12,349
202346_at	10,700	10,654	10,349	10,001	10,574	10,242
202347_s_at	11,034	11,133	10,890	11,721	11,559	11,519
202348_s_at	9,885	9,613	9,966	10,576	9,803	10,334
202349_at	9,937	9,689	10,163	9,438	9,075	9,486
202350_s_at	7,669	7,845	7,642	5,880	5,385	5,275
202351_at	10,986	11,185	10,521	11,057	12,541	11,609
202352_s_at	11,186	11,101	10,776	11,417	11,659	11,100
202353_s_at	9,986	9,708	9,859	8,871	9,633	8,901
202354_s_at	8,039	7,769	8,066	8,388	7,178	8,381
202355_s_at	9,133	9,052	9,247	9,134	8,651	9,102
202356_s_at	9,276	8,982	9,486	8,911	8,209	9,070
202357_s_at	5,322	5,394	4,960	6,388	6,169	6,540
202358_s_at	8,695	8,259	8,544	9,544	9,490	9,552
202359_s_at	7,860	7,940	7,545	8,498	9,164	9,181
202360_at	9,913	9,583	10,063	8,317	8,491	8,441
202361_at	9,302	9,152	9,342	9,688	9,740	10,285
202362_at	9,676	10,233	9,855	10,749	10,871	10,781
202363_at	8,738	9,055	8,733	8,070	9,075	8,792
202364_at	8,733	9,302	8,606	7,227	7,872	7,804
202365_at	11,866	11,223	11,777	9,606	9,711	9,818

202366_at	7,730	7,767	7,785	8,580	8,306	8,509
202367_at	8,233	8,386	8,349	8,499	8,050	8,241
202368_s_at	7,835	7,583	7,867	9,086	10,402	10,383
202369_s_at	9,268	9,252	9,949	11,335	12,407	12,245
202370_s_at	11,206	11,427	11,327	10,542	11,244	10,799
202371_at	11,923	12,202	11,364	11,642	12,259	12,187
202372_at	10,783	10,862	10,544	8,657	9,961	8,270
202373_s_at	11,313	11,510	11,154	9,116	10,504	8,818
202374_s_at	9,580	9,589	9,157	7,752	8,819	7,775
202375_at	8,088	8,637	8,378	9,891	10,494	10,294
202376_at	6,112	6,288	6,207	7,015	6,969	6,870
202377_at	10,998	11,296	10,676	11,115	11,632	11,771
202378_s_at	11,531	11,668	11,122	11,006	11,247	11,751
202379_s_at	10,945	10,955	10,505	8,551	10,308	8,734
202380_s_at	10,110	9,906	9,514	8,278	9,437	8,073
202381_at	9,329	9,896	9,159	11,545	13,020	12,126
202382_s_at	11,377	11,459	11,760	11,371	10,102	10,563
202383_at	8,288	8,012	8,367	9,034	8,270	8,697
202384_s_at	8,736	8,557	8,857	8,490	8,145	8,439
202385_s_at	8,843	8,484	8,994	8,650	8,327	8,684
202386_s_at	8,237	8,695	8,316	7,144	8,001	7,023
202387_at	9,960	9,923	10,200	9,955	9,187	9,687
202388_at	11,223	12,216	11,155	7,931	11,780	10,459
202389_s_at	9,310	9,383	9,516	8,674	9,142	8,916
202390_s_at	9,610	9,486	9,954	8,372	8,221	8,423
202391_at	12,086	11,949	12,285	12,148	12,072	12,353
202392_s_at	8,834	8,703	8,802	9,274	9,065	9,404
202393_s_at	8,708	9,468	9,260	9,915	11,109	10,653
202394_s_at	8,611	8,209	8,490	8,934	8,359	8,711
202395_at	10,171	9,846	10,097	9,735	10,617	10,117
202396_at	11,431	11,310	11,056	9,457	10,654	9,454
202397_at	10,125	10,125	10,239	9,463	8,892	9,683
202398_at	8,568	8,928	9,178	7,969	7,802	8,006
202399_s_at	10,395	10,566	11,037	10,908	10,203	10,728
202400_s_at	6,395	6,616	6,319	7,021	6,685	6,856
202401_s_at	9,172	9,414	9,559	8,801	8,697	8,867
202402_s_at	9,700	9,944	9,860	9,414	9,018	9,583
202403_s_at	7,301	7,298	7,563	12,502	13,603	14,071
202404_s_at	5,366	6,161	5,955	11,832	13,464	13,692
202405_at	8,704	8,665	8,783	8,080	7,914	7,705
202406_s_at	11,185	11,363	11,318	11,846	11,649	11,693
202407_s_at	8,525	8,211	8,293	8,317	7,542	7,908
202408_s_at	10,273	10,112	10,438	10,708	10,158	10,413
202409_at	7,362	7,475	6,821	6,859	7,045	7,411
202410_x_at	7,037	7,062	6,989	7,221	7,101	7,433
202411_at	6,133	6,331	6,469	7,191	8,253	7,228
202412_s_at	11,103	11,035	10,905	8,584	8,946	7,700
202413_s_at	13,215	13,299	13,133	10,590	11,319	9,835
202414_at	8,602	8,708	8,437	8,660	9,807	8,584
202415_s_at	9,274	8,773	9,201	9,263	8,763	9,277
202416_at	9,401	8,783	8,950	8,243	8,294	7,797
202417_at	10,260	10,126	10,624	10,023	9,198	9,784
202418_at	10,787	11,264	11,355	12,253	12,078	12,762
202419_at	9,116	9,319	9,386	10,521	10,317	10,515
202420_s_at	11,286	10,914	10,977	9,677	9,967	9,340
202421_at	9,833	10,164	10,166	6,677	6,493	6,549

202422_s_at	9,060	8,997	8,461	9,035	9,410	8,445
202423_at	10,787	10,857	10,827	8,825	9,999	9,031
202424_at	8,888	8,991	9,035	9,320	8,731	9,439
202425_x_at	9,006	9,184	9,350	8,533	9,772	9,121
202426_s_at	7,385	7,330	7,297	8,001	7,703	7,680
202427_s_at	10,774	10,913	10,668	11,236	11,166	11,534
202428_x_at	13,487	13,534	13,308	13,318	13,076	13,485
202429_s_at	9,564	9,811	9,167	9,210	10,742	9,562
202430_s_at	7,938	8,094	7,994	8,145	7,951	7,606
202431_s_at	7,939	8,960	9,556	10,734	10,460	10,157
202432_at	11,039	11,293	11,178	10,739	11,518	11,179
202433_at	9,834	9,494	10,050	10,705	9,675	10,585
202434_s_at	5,149	5,641	5,414	6,256	5,841	6,334
202435_s_at	8,568	8,957	8,568	10,452	12,066	11,590
202436_s_at	9,060	9,360	8,924	10,828	13,078	12,225
202437_s_at	8,576	8,802	8,019	10,494	12,765	11,901
202438_x_at	5,497	6,131	5,738	6,332	6,530	6,429
202439_s_at	7,563	7,676	7,704	9,652	9,901	9,991
202440_s_at	9,143	8,844	9,255	8,674	8,265	8,414
202441_at	11,194	11,566	11,303	11,511	11,808	11,746
202442_at	11,796	12,082	11,806	12,194	12,298	12,618
202443_x_at	10,932	10,897	11,515	10,912	12,125	11,634
202444_s_at	6,524	6,420	6,272	7,926	7,351	7,346
202445_s_at	6,680	7,029	7,465	6,659	7,217	6,849
202446_s_at	10,196	10,650	10,360	10,402	9,980	9,588
202447_at	8,361	8,464	8,422	10,649	10,611	10,846
202448_s_at	6,571	6,482	6,664	7,094	6,518	6,612
202449_s_at	10,085	9,937	10,315	9,768	9,907	10,053
202450_s_at	8,488	8,812	8,756	10,697	10,319	9,845
202451_at	10,248	10,073	9,944	10,267	10,929	10,077
202452_at	7,881	7,769	7,983	7,826	7,971	8,050
202453_s_at	8,396	8,559	8,090	8,308	8,508	8,200
202454_s_at	8,014	8,484	8,160	4,941	4,959	4,959
202455_at	8,807	8,842	8,643	7,782	7,273	7,707
202456_s_at	7,457	7,559	7,485	7,979	7,739	8,161
202457_s_at	10,166	10,345	9,796	9,735	11,269	10,158
202458_at	8,680	8,400	8,455	11,098	12,042	11,649
202459_s_at	7,587	7,768	7,548	6,537	7,596	7,448
202460_s_at	8,871	9,169	8,807	8,602	9,598	9,211
202461_at	9,791	9,846	10,150	10,704	10,306	10,790
202462_s_at	11,909	11,725	11,236	10,170	11,219	9,384
202463_s_at	8,517	8,201	8,369	8,725	8,780	8,769
202464_s_at	9,906	9,514	10,058	9,350	10,069	9,504
202465_at	10,045	9,668	9,918	11,535	11,164	11,789
202466_at	10,498	10,613	10,812	9,891	10,198	9,666
202467_s_at	11,681	12,057	11,634	11,543	12,644	11,895
202468_s_at	11,097	11,292	10,598	10,081	11,297	10,257
202469_s_at	11,068	10,988	10,874	10,548	10,526	10,439
202470_s_at	9,197	9,079	8,982	7,191	7,152	6,916
202471_s_at	8,494	8,591	8,587	10,151	9,408	10,354
202472_at	8,033	8,272	8,174	8,128	7,529	8,131
202473_x_at	7,398	7,353	7,640	7,337	7,574	7,432
202474_s_at	10,366	9,836	10,075	9,879	9,985	10,014
202475_at	10,345	10,235	10,704	10,940	9,959	10,890
202476_s_at	8,018	8,253	8,245	8,665	8,143	8,744
202477_s_at	9,432	9,338	9,451	9,585	9,275	9,838

202478_at	10,368	10,365	10,366	6,811	7,049	7,104
202479_s_at	8,170	7,986	8,178	6,169	6,321	6,528
202480_s_at	8,421	8,070	8,431	7,820	6,870	7,587
202481_at	8,122	8,104	8,644	8,097	7,640	8,139
202482_x_at	7,066	6,994	6,821	6,973	6,881	6,853
202483_s_at	12,210	12,052	11,839	11,748	10,981	11,353
202484_s_at	5,186	5,318	5,289	10,884	10,852	10,794
202485_s_at	6,145	5,553	5,650	6,154	6,158	6,330
202486_at	10,164	10,187	10,053	9,538	9,167	9,067
202487_s_at	12,049	12,035	11,898	10,891	10,294	10,569
202488_s_at	8,176	7,895	8,229	8,609	8,477	8,726
202489_s_at	6,869	6,740	7,022	7,300	7,139	7,286
202490_at	6,679	6,104	6,909	5,398	5,981	5,319
202491_s_at	11,179	11,299	10,974	9,453	10,397	9,638
202492_at	8,842	8,678	9,205	9,293	9,056	9,570
202493_x_at	6,532	6,386	6,575	6,696	6,479	6,842
202494_at	10,037	10,114	9,920	10,020	9,327	9,723
202495_at	8,344	8,476	8,619	8,494	7,387	8,300
202496_at	8,502	8,481	8,661	8,252	7,987	8,446
202497_x_at	9,970	10,251	10,351	9,106	8,964	8,794
202498_s_at	10,554	10,464	10,410	7,124	7,057	7,161
202499_s_at	12,572	12,780	12,836	7,745	9,894	8,721
202500_at	8,120	8,388	8,264	8,335	8,407	8,611
202501_at	9,827	9,408	9,747	9,802	9,994	9,588
202502_at	11,378	11,397	10,656	9,182	10,356	8,810
202503_s_at	13,000	12,857	12,677	12,883	12,577	12,750
202504_at	6,293	6,068	6,461	6,668	6,621	6,738
202505_at	12,056	12,202	11,866	11,431	11,362	11,494
202506_at	8,153	8,309	7,467	8,141	9,430	8,544
202507_s_at	7,237	7,252	7,215	8,138	8,222	8,127
202508_s_at	5,856	6,397	5,667	6,250	7,708	7,478
202509_s_at	7,177	6,929	7,235	8,146	7,188	7,503
202510_s_at	6,794	7,249	6,515	7,098	6,545	6,909
202511_s_at	9,460	9,849	9,628	9,696	9,689	9,768
202512_s_at	8,895	9,753	8,884	7,149	7,974	7,369
202513_s_at	9,413	9,549	9,563	9,466	9,111	9,489
202514_at	9,250	9,495	8,851	6,748	7,510	6,549
202515_at	11,276	11,688	11,162	9,515	10,758	9,859
202516_s_at	7,551	7,349	6,976	5,292	5,636	5,365
202517_at	10,427	10,274	10,450	8,077	7,542	8,045
202518_at	9,163	8,946	9,097	9,408	9,130	9,638
202519_at	9,249	8,995	9,523	8,493	9,082	9,165
202520_s_at	11,080	11,066	11,030	10,320	10,521	10,254
202521_at	11,116	10,954	11,358	9,432	9,748	9,240
202522_at	11,759	11,714	11,735	11,412	12,098	11,504
202523_s_at	7,812	8,061	7,864	8,582	8,427	8,737
202524_s_at	6,543	6,511	6,660	7,474	7,190	7,458
202525_at	8,196	8,585	7,942	6,685	6,627	7,013
202526_at	5,132	5,042	5,507	4,107	4,278	4,086
202527_s_at	10,257	10,232	9,880	8,280	8,795	8,918
202528_at	7,894	7,923	8,156	9,656	8,651	9,184
202529_at	9,457	9,724	9,674	9,894	9,530	9,803
202530_at	9,631	9,895	9,822	9,634	10,148	9,648
202531_at	7,348	7,320	7,142	7,689	7,639	7,689
202532_s_at	10,635	10,499	10,226	8,894	9,096	8,712
202533_s_at	8,481	8,280	7,913	8,706	7,608	8,237

202534_x_at	11,189	10,790	11,002	9,496	9,541	9,073
202535_at	9,089	8,945	9,223	9,379	8,821	9,092
202536_at	10,945	11,469	10,902	10,250	10,599	10,249
202537_s_at	9,542	10,290	9,631	9,773	9,763	9,364
202538_s_at	9,739	10,240	9,756	9,185	9,544	9,314
202539_s_at	11,567	11,605	10,942	10,437	11,024	10,262
202540_s_at	12,195	12,027	11,488	10,712	10,999	10,287
202541_at	9,733	9,879	9,258	8,519	9,073	8,637
202542_s_at	10,415	10,812	10,118	10,416	10,833	10,618
202543_s_at	10,135	10,515	10,445	10,794	10,935	11,194
202544_at	11,854	12,136	11,718	11,060	12,070	11,778
202545_at	7,462	7,416	7,455	7,346	6,904	7,212
202546_at	6,794	7,501	7,128	7,611	7,515	8,778
202547_s_at	7,658	7,923	8,068	7,616	7,480	7,695
202548_s_at	10,910	11,005	11,188	9,290	10,177	9,774
202549_at	8,541	8,338	8,373	7,623	7,505	7,495
202550_s_at	10,307	10,626	10,544	9,755	10,131	10,062
202551_s_at	7,644	7,565	7,454	9,063	9,552	10,197
202552_s_at	8,090	8,371	8,177	10,195	11,500	11,416
202553_s_at	10,400	10,754	10,502	9,661	10,138	9,299
202554_s_at	8,905	9,053	8,530	8,456	10,537	10,703
202555_s_at	9,511	9,389	8,925	9,187	9,090	8,855
202556_s_at	9,784	9,459	9,653	9,215	8,142	9,018
202557_at	10,229	10,749	10,084	8,641	9,489	8,528
202558_s_at	10,304	10,585	10,288	9,196	9,675	9,271
202559_x_at	11,863	11,832	11,910	10,927	10,572	10,843
202560_s_at	11,823	11,629	12,018	9,903	9,390	9,831
202561_at	11,010	11,364	11,282	8,185	9,389	8,372
202562_s_at	10,782	10,642	10,818	10,738	10,080	10,583
202563_at	6,244	6,273	6,506	7,456	7,551	7,758
202564_x_at	9,992	9,997	9,713	9,163	8,530	8,741
202565_s_at	8,232	8,439	8,526	7,965	9,488	8,065
202566_s_at	7,500	7,342	7,050	7,187	7,194	7,283
202567_at	12,911	12,699	12,393	12,356	11,836	11,905
202568_s_at	9,370	9,390	9,165	8,415	9,279	8,793
202569_s_at	9,391	9,013	9,006	7,751	8,441	7,814
202570_s_at	8,033	8,307	8,143	8,676	8,815	9,069
202571_s_at	7,663	7,792	7,930	8,207	8,451	8,711
202572_s_at	7,863	7,856	7,928	7,973	8,105	7,866
202573_at	11,028	10,987	11,305	10,635	10,726	10,897
202574_s_at	8,303	8,130	8,317	8,001	7,717	8,110
202575_at	10,574	11,060	10,992	8,022	7,657	8,179
202576_s_at	8,840	8,946	8,990	8,900	8,244	8,744
202577_s_at	8,946	8,740	9,013	8,306	8,278	8,486
202578_s_at	8,710	8,332	8,711	8,202	7,465	8,165
202579_x_at	10,743	10,833	10,633	11,394	10,884	11,388
202580_x_at	11,546	11,131	11,750	8,876	8,104	8,310
202581_at	9,689	9,848	10,046	10,178	9,680	10,487
202582_s_at	11,343	11,468	11,124	10,836	11,655	11,158
202583_s_at	7,504	7,644	7,104	6,279	6,996	6,618
202584_at	8,406	8,412	8,490	7,938	8,417	8,050
202585_s_at	7,238	7,572	7,020	6,882	7,010	7,016
202586_at	7,374	6,806	7,560	8,178	7,993	8,191
202587_s_at	7,986	7,920	7,943	9,463	9,283	9,948
202588_at	8,431	8,208	8,317	8,862	9,060	9,234
202589_at	12,324	12,323	12,364	12,223	11,940	11,980

202590_s_at	7,674	7,713	7,632	7,235	7,320	7,592
202591_s_at	13,395	12,905	12,956	12,178	11,823	11,975
202592_at	8,998	9,205	9,094	9,214	8,838	9,307
202593_s_at	8,380	8,215	8,471	8,024	7,199	7,521
202594_at	11,635	11,644	11,358	9,113	9,120	9,331
202595_s_at	9,253	9,252	9,629	10,280	9,408	9,989
202596_at	11,645	11,301	11,569	10,609	10,112	10,280
202597_at	7,896	8,344	8,111	7,266	7,235	7,296
202598_at	9,528	9,687	9,595	9,884	9,230	9,940
202599_s_at	9,897	9,719	9,721	8,540	9,390	7,496
202600_s_at	9,416	8,632	8,583	8,014	9,431	7,261
202601_s_at	7,741	7,794	7,159	7,162	6,715	7,271
202602_s_at	11,601	11,467	10,942	10,012	10,633	9,909
202603_at	12,151	12,333	11,954	10,800	11,978	10,786
202604_x_at	7,695	7,654	7,426	7,381	8,371	7,436
202605_at	10,924	11,217	11,370	10,240	10,104	9,844
202606_s_at	11,067	11,016	10,841	8,504	9,948	8,312
202607_at	7,186	7,021	7,502	7,704	7,878	7,790
202608_s_at	7,060	7,048	6,998	7,902	7,216	7,984
202609_at	9,005	9,716	8,855	11,027	12,547	11,743
202610_s_at	9,949	10,174	10,186	8,567	8,800	8,485
202611_s_at	10,435	10,623	10,717	7,577	8,225	6,982
202612_s_at	5,311	5,013	5,074	5,337	5,082	5,339
202613_at	11,895	11,692	12,227	10,819	10,789	10,573
202614_at	11,205	11,431	10,747	10,168	10,771	9,859
202615_at	11,384	11,135	11,089	10,268	11,597	10,735
202616_s_at	7,042	6,719	7,288	7,051	6,958	6,850
202617_s_at	7,101	6,772	7,009	7,590	7,457	7,653
202618_s_at	7,732	7,396	7,610	8,207	8,057	8,284
202619_s_at	6,751	7,161	6,495	9,513	10,516	10,246
202620_s_at	9,055	9,749	9,022	11,831	12,971	12,476
202621_at	9,515	9,247	9,348	9,234	8,994	9,390
202622_s_at	9,052	9,366	9,249	7,773	8,431	8,638
202623_at	9,626	9,772	9,263	9,072	8,950	9,200
202624_s_at	7,725	7,697	7,843	7,887	7,909	7,871
202625_at	9,424	9,328	9,565	6,609	6,251	6,482
202626_s_at	10,755	10,589	10,575	7,532	7,249	7,225
202627_s_at	6,302	6,478	6,328	12,897	12,477	12,796
202628_s_at	7,173	6,671	7,246	12,838	12,545	12,854
202629_at	8,515	8,876	8,328	8,311	9,588	8,469
202630_at	8,090	8,178	7,736	6,699	8,573	6,909
202631_s_at	7,341	7,545	7,466	7,975	8,082	8,244
202632_at	8,629	8,494	8,750	10,055	9,257	9,932
202633_at	10,510	10,642	10,386	8,408	9,504	7,283
202634_at	10,130	10,314	10,149	10,679	10,032	10,664
202635_s_at	9,384	9,743	9,148	9,762	9,172	9,877
202636_at	9,261	9,743	9,263	8,860	9,519	9,164
202637_s_at	6,940	7,877	7,319	7,892	7,345	7,556
202638_s_at	5,658	6,241	5,927	6,758	6,212	6,629
202639_s_at	8,688	8,609	8,701	9,056	8,938	9,339
202640_s_at	9,050	8,941	9,235	8,659	8,582	8,847
202641_at	11,507	11,690	11,331	11,429	11,042	11,390
202642_s_at	10,086	9,772	10,299	7,938	8,387	7,943
202643_s_at	5,811	6,560	6,137	8,505	8,471	7,862
202644_s_at	7,372	8,440	7,817	10,645	10,822	9,636
202645_s_at	9,712	9,611	10,180	9,245	8,515	9,087

202646_s_at	12,314	12,284	12,135	10,028	11,078	10,209
202647_s_at	10,178	10,086	10,078	9,564	9,378	9,533
202648_at	9,000	9,160	9,318	8,275	8,925	8,778
202649_x_at	14,270	14,412	14,353	13,734	13,718	14,029
202650_s_at	8,301	8,142	8,234	7,675	7,444	7,817
202651_at	9,967	10,074	9,556	8,568	9,357	8,311
202652_at	7,473	7,409	7,114	7,350	7,155	7,354
202653_s_at	11,821	11,977	11,717	9,786	11,187	9,632
202654_x_at	9,895	10,040	9,417	7,591	8,712	7,403
202655_at	11,135	11,004	11,542	12,077	11,533	11,721
202656_s_at	9,424	9,406	9,304	8,290	10,129	9,604
202657_s_at	9,662	9,679	9,568	8,585	9,454	9,065
202658_at	8,928	9,243	9,043	9,645	8,896	9,749
202659_at	7,355	7,240	7,705	8,609	7,736	8,488
202660_at	7,230	7,272	6,683	5,340	6,515	4,889
202661_at	5,820	6,519	6,091	6,407	6,598	6,759
202662_s_at	6,747	7,211	6,598	6,603	6,635	6,525
202663_at	6,763	7,118	6,841	6,750	6,746	6,842
202664_at	9,329	9,749	9,316	8,149	8,810	8,434
202665_s_at	7,031	7,173	7,286	8,258	8,099	8,210
202666_s_at	11,379	11,021	10,941	10,721	10,391	10,406
202667_s_at	8,063	7,756	7,787	8,733	8,501	8,982
202668_at	10,500	10,316	10,324	9,882	10,391	9,326
202669_s_at	8,247	7,999	7,936	8,126	7,926	7,969
202670_at	11,544	11,944	11,782	11,130	10,931	11,365
202671_s_at	9,575	9,852	9,131	10,605	9,673	10,617
202672_s_at	10,173	11,725	10,933	7,821	9,649	9,408
202673_at	12,905	13,144	12,646	12,438	12,464	12,634
202674_s_at	5,150	5,459	5,280	5,331	5,802	5,942
202675_at	8,632	8,940	8,544	9,990	9,364	10,027
202676_x_at	9,382	9,199	9,338	9,605	9,144	9,620
202677_at	10,494	10,918	10,445	9,876	11,437	9,813
202678_at	10,516	10,530	10,201	10,263	9,971	10,528
202679_at	9,297	9,631	9,758	10,453	9,714	9,904
202680_at	10,217	10,926	10,194	10,223	10,370	10,188
202681_at	9,333	9,639	9,737	9,891	9,370	9,630
202682_s_at	9,525	9,764	9,840	9,860	9,736	9,923
202683_s_at	9,394	9,429	9,085	7,603	8,543	7,204
202684_s_at	6,821	6,909	6,849	7,302	8,225	7,421
202685_s_at	8,226	7,731	7,711	8,787	8,354	9,076
202686_s_at	9,124	9,312	9,299	11,091	11,646	11,839
202687_s_at	4,753	5,349	4,959	5,122	5,133	5,275
202688_at	4,408	4,650	4,568	4,764	4,770	5,059
202689_at	9,213	9,054	9,498	9,028	8,638	8,994
202690_s_at	11,597	11,481	10,931	11,146	10,433	10,612
202691_at	10,971	11,198	10,919	9,894	9,761	9,391
202692_s_at	8,191	8,033	8,450	8,257	8,124	8,265
202693_s_at	8,862	9,271	8,900	11,219	12,002	11,725
202694_at	6,390	6,634	6,484	6,926	7,396	7,095
202695_s_at	7,311	7,178	7,279	8,664	9,100	8,968
202696_at	10,824	10,970	10,674	9,522	10,557	9,777
202697_at	10,765	10,912	10,811	11,016	9,985	10,819
202698_x_at	13,397	13,559	13,594	13,053	12,918	13,127
202699_s_at	7,076	7,200	7,802	7,780	7,730	8,010
202700_s_at	7,304	7,421	7,792	7,565	7,296	7,393
202701_at	8,222	8,128	8,159	9,016	8,707	9,058

202702_at	8,877	8,588	9,294	9,091	8,531	8,957
202703_at	9,910	10,030	9,866	9,799	9,821	10,111
202704_at	8,915	9,475	8,584	8,465	10,367	9,844
202705_at	11,921	12,431	12,026	10,672	10,504	10,178
202706_s_at	10,566	10,320	10,468	10,121	9,408	9,654
202707_at	5,869	5,560	6,035	5,555	4,947	5,192
202708_s_at	7,633	8,921	8,707	7,017	8,219	7,990
202709_at	6,307	7,086	7,061	6,930	6,724	6,770
202710_at	10,177	10,469	9,826	10,144	9,933	10,176
202711_at	7,279	7,120	7,287	7,431	7,488	7,377
202712_s_at	8,961	9,080	8,879	7,593	7,457	7,705
202713_s_at	9,777	9,884	10,050	9,932	9,892	9,864
202714_s_at	6,306	6,172	6,132	6,268	6,150	6,228
202715_at	10,634	10,521	10,755	8,736	8,664	9,179
202716_at	9,446	9,244	9,656	9,207	9,816	10,109
202717_s_at	10,938	11,090	10,984	9,850	10,103	9,708
202718_at	8,745	8,689	8,834	7,199	9,246	10,035
202719_s_at	8,009	8,296	7,765	9,374	9,066	8,385
202720_at	9,198	9,793	9,286	10,568	10,599	9,938
202721_s_at	8,882	9,234	8,852	9,750	9,672	9,723
202722_s_at	9,975	10,597	10,081	10,496	11,044	10,853
202723_s_at	10,283	10,395	11,469	7,105	8,862	8,073
202724_s_at	9,867	9,892	10,687	6,262	8,000	7,408
202725_at	9,566	9,545	9,877	8,818	9,224	9,113
202726_at	9,105	8,722	9,129	8,487	8,081	8,362
202727_s_at	9,826	10,042	9,641	9,422	10,160	10,133
202728_s_at	9,034	8,865	8,889	9,879	10,960	11,234
202729_s_at	10,437	10,453	10,438	11,034	12,641	12,143
202730_s_at	7,513	8,111	6,677	7,148	7,605	7,472
202731_at	7,155	7,467	5,964	5,694	6,466	5,997
202732_at	9,069	9,266	9,733	10,080	9,411	9,682
202733_at	7,693	8,533	7,934	11,688	11,757	12,153
202734_at	8,357	8,972	8,604	8,468	7,717	8,058
202735_at	11,501	11,606	11,668	11,410	10,852	11,094
202736_s_at	9,543	9,206	9,261	8,710	7,882	8,559
202737_s_at	11,953	11,697	11,877	11,229	10,257	11,056
202738_s_at	10,028	10,189	9,300	8,913	9,908	8,693
202739_s_at	8,865	8,888	8,417	7,376	8,825	7,368
202740_at	9,502	9,368	9,337	9,149	8,534	9,047
202741_at	10,862	10,965	11,044	9,724	10,233	9,263
202742_s_at	8,382	8,271	8,087	6,921	6,740	6,337
202743_at	9,669	9,550	9,356	6,273	7,561	7,109
202744_at	7,803	7,261	7,793	7,568	7,488	7,696
202745_at	9,412	9,161	8,785	8,057	8,900	7,133
202746_at	8,265	8,603	8,498	3,875	3,844	3,717
202747_s_at	9,287	9,379	9,213	8,120	7,282	7,554
202748_at	7,058	6,695	6,851	7,518	7,703	7,671
202749_at	12,068	12,485	11,568	10,924	10,882	11,231
202750_s_at	8,884	8,723	8,914	9,042	8,602	8,935
202751_at	6,125	5,316	6,129	5,671	5,791	5,625
202752_x_at	9,682	9,264	9,192	8,246	8,112	8,443
202753_at	13,092	13,139	12,973	12,254	12,107	12,257
202754_at	10,585	10,368	10,827	9,445	10,130	9,520
202755_s_at	6,714	6,468	6,673	8,266	8,100	8,322
202756_s_at	9,365	9,037	9,267	10,088	10,761	11,202
202757_at	10,335	10,271	10,380	9,383	9,123	9,342

202758_s_at	8,920	8,679	8,945	9,477	8,869	9,512
202759_s_at	9,896	9,404	9,829	9,591	10,518	9,725
202760_s_at	11,237	10,693	11,120	10,618	11,535	10,888
202761_s_at	9,518	9,990	10,047	5,035	5,646	5,164
202762_at	9,105	9,335	8,746	7,676	9,432	7,578
202763_at	9,749	10,037	9,984	8,548	9,162	9,011
202764_at	7,050	7,062	6,851	7,473	7,234	7,408
202765_s_at	7,530	7,054	7,127	8,530	10,077	9,366
202766_s_at	9,020	8,948	8,894	10,514	13,024	11,323
202767_at	9,104	8,563	9,266	9,815	9,217	9,689
202768_at	7,550	10,033	9,680	9,216	10,670	10,539
202769_at	10,330	11,074	10,081	8,174	9,795	9,349
202770_s_at	7,231	7,921	6,899	5,231	6,352	6,037
202771_at	9,092	8,817	8,903	9,968	10,621	10,938
202772_at	9,513	10,024	9,512	10,420	9,956	10,560
202773_s_at	9,284	9,188	9,230	8,101	7,246	7,970
202774_s_at	9,813	9,882	9,842	8,742	8,597	8,772
202775_s_at	8,848	8,921	9,012	7,488	7,675	7,562
202776_at	11,677	11,572	11,028	9,929	10,641	8,396
202777_at	9,470	9,684	9,090	8,791	9,609	8,760
202778_s_at	10,842	11,198	10,467	7,641	9,452	7,754
202779_s_at	10,456	10,325	10,075	10,042	8,294	9,160
202780_at	8,945	8,814	8,764	9,396	10,594	9,688
202781_s_at	7,215	7,347	6,997	7,994	7,785	7,910
202782_s_at	7,727	7,744	7,814	8,353	7,880	8,203
202783_at	7,651	7,624	7,326	7,217	8,569	7,683
202784_s_at	7,237	7,437	6,819	6,859	7,689	7,180
202785_at	9,770	9,963	10,008	9,886	9,467	10,003
202786_at	10,615	10,953	10,612	9,956	10,719	10,204
202787_s_at	8,542	8,405	8,648	9,922	9,335	9,635
202788_at	10,593	10,655	11,193	11,312	11,140	11,126
202789_at	9,609	9,471	9,599	8,825	8,945	9,019
202790_at	7,477	7,429	7,658	7,036	6,808	6,984
202791_s_at	7,802	7,790	7,889	8,432	8,512	8,458
202792_s_at	7,432	6,931	7,018	6,760	7,050	6,917
202793_at	9,624	9,375	9,454	9,042	8,807	9,093
202794_at	7,769	8,428	8,247	10,166	9,668	9,807
202795_x_at	8,989	8,966	9,251	10,171	10,357	10,534
202796_at	7,044	6,950	6,921	7,649	7,761	7,994
202797_at	11,259	11,541	11,076	10,328	11,254	10,030
202798_at	9,697	10,260	9,727	9,379	10,583	9,767
202799_at	9,641	9,711	9,643	10,510	9,685	10,472
202800_at	8,071	7,597	7,908	7,155	6,784	7,190
202801_at	8,141	8,022	8,296	8,517	8,363	8,568
202802_at	9,418	9,742	9,204	9,004	8,453	9,089
202803_s_at	6,005	5,988	5,949	6,564	6,631	6,782
202804_at	10,242	10,548	10,640	9,924	11,271	10,932
202805_s_at	7,322	7,264	7,481	7,563	8,107	7,926
202806_at	11,230	10,881	11,749	10,611	10,425	10,920
202807_s_at	7,722	7,783	7,431	8,274	7,652	8,284
202808_at	8,930	9,283	9,436	9,410	10,050	10,188
202809_s_at	10,005	9,856	10,281	9,526	9,796	9,641
202810_at	11,414	11,483	11,840	11,360	11,238	11,519
202811_at	10,361	10,247	10,634	9,841	9,962	10,004
202812_at	8,876	8,825	9,031	8,175	8,156	8,430
202813_at	9,908	9,916	9,748	8,214	8,892	8,382

202814_s_at	10,231	10,186	10,039	9,877	10,359	10,144
202815_s_at	7,945	7,580	7,911	7,976	8,081	7,889
202816_s_at	7,252	7,149	7,263	8,898	9,285	9,070
202817_s_at	6,454	6,083	5,909	5,362	5,596	5,647
202818_s_at	9,225	8,933	9,409	9,668	8,891	9,614
202819_s_at	8,097	7,732	8,153	8,835	8,370	8,668
202820_at	6,158	6,687	6,173	7,961	9,436	8,339
202821_s_at	6,677	6,826	6,574	7,209	6,626	7,015
202822_at	9,838	9,819	10,097	9,156	10,717	9,228
202823_at	10,020	9,748	9,461	10,252	10,301	10,602
202824_s_at	12,449	12,525	12,287	12,903	12,620	12,918
202825_at	10,455	10,553	10,541	8,889	8,725	9,174
202826_at	7,864	7,813	8,019	7,793	7,745	8,150
202827_s_at	7,243	7,288	7,020	7,760	7,378	7,741
202828_s_at	7,970	8,092	7,981	8,522	8,641	8,207
202829_s_at	10,097	10,655	9,853	10,307	11,554	11,216
202830_s_at	9,178	9,305	9,203	8,960	8,181	8,511
202831_at	7,790	7,593	7,623	7,669	7,567	7,312
202832_at	9,858	10,185	9,596	8,333	9,813	7,949
202833_s_at	4,622	4,190	4,686	5,525	5,375	5,477
202834_at	6,867	7,032	7,015	7,434	7,445	7,484
202835_at	7,165	6,983	7,220	7,167	6,901	7,032
202836_s_at	12,158	12,139	12,388	12,475	11,913	12,263
202837_at	8,731	8,623	8,729	8,097	7,965	8,078
202838_at	8,079	9,023	8,457	7,641	7,775	7,709
202839_s_at	8,654	8,677	8,545	8,987	8,068	8,967
202840_at	9,438	9,151	9,461	9,071	9,546	9,001
202841_x_at	8,065	8,269	8,181	8,471	8,250	8,441
202842_s_at	9,698	10,211	9,648	9,557	10,387	10,203
202843_at	8,652	9,154	8,462	7,638	8,273	8,100
202844_s_at	8,705	8,923	8,834	9,046	8,916	8,878
202845_s_at	9,708	9,992	9,876	9,392	9,825	9,302
202846_s_at	10,762	10,819	11,164	10,035	9,099	9,686
202847_at	9,184	9,994	9,710	9,299	9,076	9,888
202848_s_at	8,033	8,391	8,142	7,596	7,245	7,594
202849_x_at	9,514	9,320	9,589	9,992	9,440	9,796
202850_at	11,511	11,585	11,082	10,458	11,174	10,449
202851_at	7,707	7,777	7,830	7,825	7,428	7,705
202852_s_at	10,738	10,576	10,543	9,311	8,916	8,734
202853_s_at	10,522	10,701	10,406	9,375	9,501	9,735
202854_at	11,472	11,684	11,256	11,855	11,546	11,639
202855_s_at	6,516	6,064	5,763	7,521	6,725	7,277
202856_s_at	5,518	6,016	4,954	9,822	8,986	9,833
202857_at	12,563	12,355	12,706	12,190	11,584	12,393
202858_at	13,643	13,219	13,279	11,711	10,957	11,330
202859_x_at	5,396	5,644	5,983	13,307	10,732	11,127
202860_at	10,260	10,245	10,537	8,977	8,718	9,129
202861_at	8,288	8,117	8,257	8,680	9,468	9,298
202862_at	7,219	7,488	7,444	8,742	8,627	9,100
202863_at	5,782	5,989	5,531	9,864	10,494	9,864
202864_s_at	6,192	6,649	6,920	9,561	9,904	9,125
202865_at	5,710	5,695	6,103	7,384	6,886	6,911
202866_at	9,180	9,002	9,341	10,333	10,065	10,320
202867_s_at	7,856	8,069	8,117	9,393	8,450	9,372
202868_s_at	9,642	9,779	9,732	11,180	10,430	11,261
202869_at	6,087	5,914	5,948	6,239	6,393	6,223

202870_s_at	12,042	12,008	12,379	11,127	9,953	10,358
202871_at	9,628	9,124	9,605	8,022	7,942	8,382
202872_at	8,871	9,352	8,677	10,064	9,944	9,864
202873_at	6,179	6,080	6,807	6,676	6,572	6,592
202874_s_at	8,496	9,019	8,019	8,849	8,900	8,790
202875_s_at	6,794	7,113	7,195	7,318	6,900	6,941
202876_s_at	8,289	8,349	8,377	8,160	8,343	8,296
202877_s_at	6,265	6,430	6,184	6,587	6,280	6,441
202878_s_at	5,831	6,268	5,791	6,009	5,697	6,015
202879_s_at	7,298	7,091	7,247	8,429	8,204	8,645
202880_s_at	7,536	8,002	7,565	7,513	7,870	7,901
202881_x_at	6,059	6,176	6,105	6,403	6,449	6,404
202882_x_at	11,203	11,350	11,172	11,803	11,474	11,682
202883_s_at	11,406	11,196	11,490	9,304	9,181	8,682
202884_s_at	8,574	8,521	7,887	8,208	8,216	7,961
202885_s_at	6,866	6,920	7,042	6,993	7,003	7,150
202886_s_at	8,758	8,726	8,566	8,886	9,129	8,510
202887_s_at	8,981	9,758	9,524	8,799	10,937	9,622
202888_s_at	6,842	7,268	6,627	12,159	12,091	12,208
202889_x_at	7,378	7,439	7,276	7,454	7,346	7,393
202890_at	8,006	8,578	7,926	5,017	5,465	5,325
202891_at	9,098	9,383	9,191	9,071	8,528	9,093
202892_at	10,482	10,822	10,626	9,725	9,823	9,459
202893_at	9,402	9,814	9,862	8,280	8,711	8,443
202894_at	9,918	9,623	10,218	8,580	8,007	8,228
202895_s_at	6,730	6,523	6,103	7,253	6,514	7,121
202896_s_at	6,911	7,154	7,350	6,992	7,165	7,812
202897_at	8,153	8,011	8,099	7,207	7,514	7,959
202898_at	8,683	8,409	8,395	7,443	7,409	8,164
202899_s_at	12,998	12,575	12,382	11,904	11,272	11,804
2028_s_at	8,604	8,188	8,507	8,638	8,399	8,590
202900_s_at	11,099	11,124	11,137	10,540	10,338	9,838
202901_x_at	3,748	3,643	3,784	6,455	3,829	3,478
202902_s_at	5,295	5,412	5,621	7,246	5,881	5,926
202903_at	10,756	10,610	10,979	7,426	7,221	6,822
202904_s_at	10,493	10,631	10,384	9,392	8,582	9,067
202905_x_at	7,725	7,659	7,384	8,508	8,754	8,180
202906_s_at	6,841	6,803	6,851	8,261	10,085	8,002
202907_s_at	9,348	9,345	9,013	9,638	9,969	8,677
202908_at	9,119	9,135	9,487	9,672	9,393	9,953
202909_at	9,195	9,261	8,872	7,923	8,673	8,081
202910_s_at	6,839	6,566	6,403	9,230	9,055	9,440
202911_at	12,926	12,949	12,615	10,216	10,757	9,539
202912_at	12,814	13,429	13,296	13,333	13,414	12,857
202913_at	7,938	8,002	8,227	6,918	6,820	7,285
202914_s_at	6,521	6,999	6,929	6,675	6,507	6,995
202915_s_at	8,496	8,430	8,591	7,623	7,830	7,812
202916_s_at	9,048	9,375	8,944	8,158	8,551	8,162
202917_s_at	4,543	4,310	4,921	4,384	4,077	4,248
202918_s_at	11,836	12,137	11,493	10,989	10,885	10,982
202919_at	10,612	11,172	10,475	9,096	9,548	9,315
202920_at	8,551	7,971	8,208	6,189	7,812	6,200
202921_s_at	6,449	6,388	6,593	6,671	6,585	6,558
202922_at	10,261	10,382	10,226	8,636	9,731	9,217
202923_s_at	9,769	9,916	9,953	9,735	9,987	9,713
202924_s_at	7,805	7,647	7,754	7,919	6,894	7,473

202925_s_at	8,665	8,182	8,792	6,914	6,186	6,216
202926_at	9,650	9,890	9,682	8,677	9,785	9,130
202927_at	8,918	8,764	9,117	9,064	8,478	8,963
202928_s_at	6,886	6,924	7,048	7,090	6,595	6,951
202929_s_at	8,248	8,474	8,338	9,180	8,282	9,074
202930_s_at	10,813	10,721	10,254	8,856	10,061	9,163
202931_x_at	9,407	9,406	9,329	9,615	10,018	9,547
202932_at	10,882	10,820	10,128	9,061	10,074	8,233
202933_s_at	11,482	11,577	10,878	10,678	11,519	9,914
202934_at	10,331	10,276	10,456	9,550	9,495	8,722
202935_s_at	6,315	6,440	6,629	5,786	6,474	6,710
202936_s_at	7,272	7,571	7,269	7,072	7,235	8,194
202937_x_at	8,605	8,376	8,724	10,615	8,326	9,384
202938_x_at	7,623	7,458	7,709	8,419	8,068	8,420
202939_at	11,786	11,367	11,837	10,821	11,758	11,085
202940_at	6,877	6,409	6,971	6,755	6,803	6,756
202941_at	9,888	10,170	9,948	10,809	10,242	10,421
202942_at	8,963	8,919	9,093	11,217	10,455	11,156
202943_s_at	7,899	8,270	8,137	8,595	8,246	9,030
202944_at	8,873	9,210	9,412	8,392	8,462	8,605
202945_at	9,354	9,189	9,275	9,535	9,238	9,602
202946_s_at	11,363	11,412	11,246	7,686	9,829	8,889
202947_s_at	8,551	8,284	8,470	9,310	9,082	9,372
202948_at	5,829	6,050	5,466	10,885	11,537	11,137
202949_s_at	10,614	10,587	10,652	12,109	12,500	12,725
202950_at	10,669	11,156	10,341	10,019	10,225	9,589
202951_at	8,683	8,899	8,458	8,292	8,750	8,525
202952_s_at	6,577	7,261	6,686	10,842	11,631	11,219
202953_at	6,041	5,417	5,900	6,828	6,487	6,951
202954_at	11,138	11,491	11,385	9,852	9,394	9,735
202955_s_at	9,010	9,407	8,873	7,629	7,920	7,591
202956_at	10,578	10,844	10,510	7,984	9,583	7,499
202957_at	4,405	4,938	4,921	6,631	7,470	6,431
202958_at	9,299	9,073	9,193	8,284	8,084	8,114
202959_at	8,932	9,383	8,686	7,710	8,180	7,165
202960_s_at	9,326	9,844	9,201	9,342	9,641	8,918
202961_s_at	12,668	12,478	12,595	12,278	12,273	12,671
202962_at	8,427	8,983	8,695	7,962	8,362	7,678
202963_at	10,551	10,437	10,358	8,861	8,663	8,441
202964_s_at	8,546	8,490	8,576	7,699	7,128	7,422
202965_s_at	8,277	7,628	7,041	5,193	4,933	5,090
202966_at	7,113	6,157	6,760	5,105	5,264	5,150
202967_at	11,548	11,951	11,454	10,301	10,976	11,114
202968_s_at	8,952	8,932	9,188	8,140	8,283	8,179
202969_at	10,347	10,184	10,114	7,984	8,835	7,369
202970_at	7,816	8,408	7,734	7,004	7,047	7,161
202971_s_at	9,545	9,151	9,190	6,656	7,400	6,893
202972_s_at	7,783	7,886	7,579	7,771	8,072	7,518
202973_x_at	9,587	9,824	9,459	8,287	9,761	8,069
202974_at	7,634	7,738	7,759	9,117	8,520	8,992
202975_s_at	9,532	9,442	9,148	9,170	9,745	9,553
202976_s_at	8,220	8,448	7,924	7,780	8,504	7,870
202977_s_at	7,476	7,324	7,358	8,506	8,736	8,657
202978_s_at	7,930	7,924	8,092	6,773	7,095	6,903
202979_s_at	8,934	8,821	8,627	6,963	7,365	7,248
202980_s_at	9,174	8,711	8,460	7,455	7,114	7,299

202981_x_at	12,473	12,547	12,612	9,611	9,926	9,663
202982_s_at	8,022	7,979	7,800	8,190	7,934	8,680
202983_at	11,458	12,091	10,808	8,080	9,231	7,468
202984_s_at	7,872	7,127	7,610	6,690	6,920	7,025
202985_s_at	9,980	9,790	10,272	9,300	9,807	9,311
202986_at	8,501	8,268	8,655	6,768	7,058	7,131
202987_at	4,135	4,090	4,330	4,594	4,459	4,556
202988_s_at	4,596	4,675	4,704	5,038	5,519	4,917
202989_at	3,580	3,458	3,531	3,722	3,932	3,936
202990_at	9,916	10,184	9,502	9,413	9,697	10,171
202991_at	7,824	7,638	7,658	8,165	7,872	8,242
202992_at	4,456	4,702	4,590	5,439	5,543	5,351
202993_at	9,061	8,944	9,016	9,367	8,632	9,552
202994_s_at	9,446	9,213	9,508	7,902	7,745	8,080
202995_s_at	8,468	8,202	8,247	5,934	5,622	6,248
202996_at	7,201	7,290	7,413	8,655	8,169	8,663
202997_s_at	6,867	6,552	6,816	7,047	6,930	7,071
202998_s_at	8,964	9,017	8,893	10,262	9,671	10,509
202999_s_at	6,948	7,149	7,084	7,504	7,241	7,353
203000_at	7,802	8,665	7,145	6,844	7,161	7,350
203001_s_at	6,648	6,568	5,769	6,539	6,594	6,652
203002_at	10,711	11,371	11,590	10,332	10,897	11,470
203003_at	7,688	7,640	7,214	7,467	7,482	7,614
203004_s_at	7,227	7,351	7,508	8,167	8,111	8,182
203005_at	7,600	7,589	7,546	9,606	9,446	9,973
203006_at	10,031	10,099	10,630	9,779	9,852	10,405
203007_x_at	11,204	11,514	11,089	10,143	9,987	10,605
203008_x_at	11,173	11,481	11,213	11,551	11,953	12,091
203009_at	7,012	7,106	7,218	7,574	7,561	7,406
203010_at	8,098	8,114	7,728	7,844	7,447	8,046
203011_at	10,423	11,173	9,838	10,260	11,155	10,784
203012_x_at	13,784	13,942	13,754	13,733	13,893	13,938
203013_at	9,539	9,765	9,502	9,589	9,771	9,239
203014_x_at	8,075	8,215	7,987	8,340	7,886	8,444
203015_s_at	8,321	8,198	7,910	7,255	7,142	6,669
203016_s_at	9,403	9,496	9,066	7,489	8,009	7,229
203017_s_at	9,147	9,123	8,600	6,898	7,240	5,891
203018_s_at	9,383	9,552	8,893	7,817	8,206	7,960
203019_x_at	8,129	8,373	7,777	5,861	6,289	5,779
203020_at	10,161	10,544	10,356	8,189	8,043	7,800
203021_at	7,372	7,845	7,977	8,377	8,175	8,584
203022_at	10,040	10,023	10,056	9,432	7,847	8,741
203023_at	8,942	8,630	8,698	10,024	8,231	9,160
203024_s_at	11,205	11,329	11,067	11,354	11,643	11,788
203025_at	9,203	9,152	9,512	10,727	9,629	10,403
203026_at	10,733	10,996	10,858	8,303	8,898	8,670
203027_s_at	7,602	7,604	7,424	7,487	7,490	7,740
203028_s_at	9,489	9,057	8,878	10,333	9,020	10,299
203029_s_at	8,751	8,597	8,084	6,926	6,618	6,704
203030_s_at	6,481	6,207	6,556	6,639	6,669	6,935
203031_s_at	9,002	8,986	9,065	8,552	8,452	8,916
203032_s_at	5,888	5,457	4,840	5,054	4,519	5,070
203033_x_at	10,904	11,116	10,783	10,772	10,644	10,749
203034_s_at	12,880	13,325	13,011	13,271	12,699	13,300
203035_s_at	9,729	9,783	9,810	8,937	9,249	9,798
203036_s_at	7,338	7,047	7,708	7,941	8,078	7,972

203037_s_at	8,376	8,827	8,896	8,885	10,692	9,556
203038_at	10,001	10,262	10,058	9,718	11,234	9,590
203039_s_at	10,470	10,660	10,391	10,219	10,568	10,103
203040_s_at	10,253	10,153	10,405	10,101	9,464	9,829
203041_s_at	9,628	9,783	9,896	10,972	11,209	11,367
203042_at	9,489	9,570	9,539	10,854	11,304	11,358
203043_at	10,090	9,702	10,294	8,891	8,143	8,495
203044_at	12,286	12,068	12,488	10,643	11,830	11,102
203045_at	8,562	8,752	8,718	8,705	8,222	8,614
203046_s_at	11,403	11,296	11,324	9,152	8,467	8,439
203047_at	7,521	7,663	7,605	9,050	9,063	9,231
203048_s_at	9,197	9,280	8,393	7,938	9,401	7,367
203049_s_at	9,996	9,882	9,061	9,639	11,241	9,022
203050_at	10,533	10,527	10,841	9,226	9,896	9,316
203051_at	8,645	8,244	8,182	8,066	7,763	8,349
203052_at	7,552	7,335	7,573	7,837	7,668	7,730
203053_at	11,779	12,022	11,523	10,555	10,962	10,560
203054_s_at	8,136	7,745	8,216	9,170	8,899	9,754
203055_s_at	8,626	8,534	8,693	9,142	9,193	8,935
203056_s_at	7,806	7,644	7,972	8,267	7,733	8,470
203057_s_at	9,291	8,990	9,143	8,128	9,752	8,338
203058_s_at	7,710	8,108	8,394	10,496	10,784	11,335
203059_s_at	7,144	7,524	7,300	8,328	8,030	8,580
203060_s_at	9,134	9,625	9,918	11,866	12,470	12,702
203061_s_at	7,965	7,538	7,646	7,572	7,167	7,543
203062_s_at	10,190	10,122	10,413	8,393	8,150	8,048
203063_at	8,950	8,844	9,010	9,100	9,378	9,565
203064_s_at	9,961	10,021	10,162	9,690	9,268	9,844
203065_s_at	7,319	7,901	7,946	12,829	13,587	13,478
203066_at	9,239	9,042	9,835	7,040	10,011	7,251
203067_at	10,313	10,611	10,316	10,110	10,219	9,850
203068_at	9,032	9,082	9,120	10,189	9,872	10,182
203069_at	9,977	9,791	10,163	7,972	7,344	7,785
203070_at	6,568	6,414	6,725	6,999	7,387	7,213
203071_at	7,731	7,780	7,663	8,074	8,229	8,186
203072_at	7,166	7,205	7,595	7,820	7,844	7,727
203073_at	10,439	10,588	10,677	8,844	9,080	8,925
203074_at	7,188	7,104	7,166	7,587	7,591	7,769
203075_at	10,669	10,697	10,638	10,113	10,608	10,157
203076_s_at	9,410	9,751	9,415	9,902	9,910	9,952
203077_s_at	8,974	9,243	8,934	9,090	9,224	9,062
203078_at	7,311	7,151	6,825	6,197	6,982	5,339
203079_s_at	9,429	9,808	9,273	8,731	9,920	8,134
203080_s_at	10,181	9,909	9,484	5,961	7,289	6,238
203081_at	9,239	9,529	9,614	8,631	8,112	8,327
203082_at	10,350	10,512	10,073	9,226	9,229	8,486
203083_at	9,149	9,118	10,095	9,771	10,422	8,783
203084_at	7,015	6,843	7,336	7,367	7,455	7,603
203085_s_at	6,029	5,748	5,589	6,600	6,453	6,882
203086_at	8,003	8,152	8,111	6,923	7,469	6,582
203087_s_at	10,750	10,802	10,340	8,966	9,502	8,450
203088_at	5,952	6,018	6,057	8,471	8,070	9,484
203089_s_at	9,180	9,071	9,131	10,141	8,549	10,110
203090_at	8,476	8,470	8,606	9,154	8,548	9,131
203091_at	9,531	9,136	9,220	8,531	8,453	8,606
203092_at	9,237	9,348	9,658	8,881	8,620	9,058

203093_s_at	9,465	9,278	9,493	8,694	8,381	8,813
203094_at	9,261	9,401	9,357	9,674	8,936	9,564
203095_at	11,441	11,682	11,134	10,260	10,695	9,322
203096_s_at	8,632	8,492	8,682	6,899	7,199	6,896
203097_s_at	10,532	10,733	10,617	8,851	10,759	8,443
203098_at	10,967	11,220	10,777	9,116	9,336	9,183
203099_s_at	9,435	9,442	9,299	8,504	8,201	8,548
203100_s_at	9,383	9,448	8,953	7,334	7,222	7,478
203101_s_at	6,429	6,010	5,933	7,012	7,261	7,490
203102_s_at	9,243	9,051	9,101	10,093	9,930	10,594
203103_s_at	10,695	9,821	10,488	9,945	8,986	9,270
203104_at	6,692	6,693	6,516	7,430	6,913	7,191
203105_s_at	11,050	11,411	10,554	9,192	9,699	8,592
203106_s_at	7,256	7,092	6,659	4,503	4,900	4,656
203107_x_at	14,690	14,735	14,724	14,078	14,262	14,400
203108_at	6,378	6,647	6,818	8,220	9,256	8,302
203109_at	8,482	8,210	8,241	9,366	8,476	9,283
203110_at	7,287	7,125	7,285	7,455	7,617	7,745
203111_s_at	7,848	7,505	8,017	8,521	8,409	8,480
203112_s_at	9,226	9,133	9,377	7,730	7,334	7,714
203113_s_at	12,286	12,114	12,103	12,490	11,611	12,437
203114_at	8,801	8,503	9,096	9,395	8,713	9,375
203115_at	8,728	8,714	8,697	8,004	7,640	7,852
203116_s_at	9,264	9,494	9,287	9,536	9,434	9,836
203117_s_at	8,599	8,700	8,596	7,054	7,131	7,045
203118_at	8,874	8,589	8,924	9,495	9,473	9,626
203119_at	10,157	9,884	10,745	11,276	10,348	10,699
203120_at	10,950	11,104	10,903	8,804	9,423	8,914
203122_at	8,974	8,857	9,183	8,874	8,675	9,067
203123_s_at	10,333	10,496	10,724	8,884	7,888	8,130
203124_s_at	10,856	11,161	11,187	9,832	9,402	8,624
203125_x_at	8,434	8,386	8,321	8,461	8,050	8,292
203126_at	10,378	10,324	10,207	8,657	8,461	8,688
203127_s_at	8,582	8,702	8,839	9,064	9,043	9,143
203128_at	7,288	7,152	7,184	7,372	7,302	7,235
203129_s_at	9,768	9,900	9,868	7,716	7,527	7,747
203130_s_at	11,370	11,827	11,521	5,879	5,898	5,979
203131_at	8,356	8,096	9,140	9,364	11,413	10,881
203132_at	10,141	10,128	9,889	10,037	10,678	9,647
203133_at	12,894	13,158	13,271	13,588	13,234	13,726
203134_at	5,823	6,053	5,678	5,818	5,724	5,760
203135_at	10,423	10,451	10,592	9,607	9,248	9,193
203136_at	9,706	9,843	9,670	11,003	10,147	11,597
203137_at	12,169	11,871	11,941	11,879	11,665	11,462
203138_at	12,126	12,235	11,556	10,790	10,884	10,903
203139_at	10,545	10,464	10,994	6,098	9,339	8,206
203140_at	7,945	8,960	8,342	9,450	10,797	9,930
203141_s_at	6,643	6,376	6,692	6,904	6,932	6,860
203142_s_at	10,000	9,780	9,553	9,106	9,769	8,980
203143_s_at	7,056	7,509	7,318	7,447	7,190	7,302
203144_s_at	7,380	7,058	7,151	7,675	8,093	8,375
203145_at	11,039	11,145	11,096	9,486	9,254	8,788
203146_s_at	8,093	7,608	8,152	7,582	7,494	7,390
203147_s_at	8,557	8,247	8,646	8,301	7,293	7,531
203148_s_at	10,074	9,983	10,673	9,876	8,245	8,535
203149_at	8,413	8,418	8,314	8,334	7,746	8,256

203150_at	9,664	9,666	10,055	10,247	9,659	10,112
203151_at	7,505	7,571	7,627	7,845	8,670	9,043
203152_at	8,913	9,029	9,274	10,445	9,763	10,139
203153_at	5,777	6,258	5,166	6,076	6,328	6,276
203154_s_at	8,666	8,704	8,987	8,762	8,195	8,777
203155_at	10,490	10,484	10,523	8,319	8,033	8,046
203156_at	9,659	9,514	9,116	8,331	9,634	7,869
203157_s_at	6,401	6,454	6,906	7,186	7,808	7,661
203158_s_at	7,281	6,864	7,002	7,924	8,169	8,309
203159_at	8,393	8,421	8,720	9,483	10,469	9,979
203160_s_at	9,848	9,684	9,443	9,060	8,647	8,728
203161_s_at	5,976	6,053	6,669	5,900	5,889	6,186
203162_s_at	9,546	9,461	9,849	10,083	9,038	9,743
203163_at	7,795	7,591	7,653	7,196	6,583	7,168
203164_at	9,191	9,184	9,220	9,052	9,690	9,302
203165_s_at	7,947	8,229	7,874	8,260	8,099	8,080
203166_at	10,693	10,828	10,036	8,897	8,685	8,519
203167_at	7,725	7,786	7,890	9,196	9,238	9,568
203168_at	7,316	7,199	7,443	7,725	7,718	7,697
203169_at	7,685	7,900	8,249	8,228	8,354	8,675
203170_at	4,954	4,513	4,845	4,536	4,393	4,564
203171_s_at	8,181	8,419	8,567	8,659	7,775	8,566
203172_at	8,264	8,470	8,380	8,497	8,093	8,359
203173_s_at	9,823	10,102	9,672	9,544	9,394	9,619
203174_s_at	8,363	8,145	8,299	8,517	8,220	8,331
203175_at	8,678	8,746	9,234	10,090	9,270	10,037
203176_s_at	8,511	8,570	8,381	8,487	6,954	7,472
203177_x_at	11,351	11,293	11,035	10,411	10,436	10,024
203178_at	8,619	9,092	8,398	3,375	3,276	3,354
203179_at	8,419	8,385	8,368	8,334	7,910	8,486
203180_at	8,867	9,547	9,465	12,521	11,489	11,815
203181_x_at	11,061	11,361	10,935	9,977	10,755	10,083
203182_s_at	9,851	10,232	9,615	7,955	8,759	8,318
203183_s_at	7,727	7,126	7,668	7,378	7,431	7,391
203184_at	9,923	9,518	9,650	7,370	8,448	9,501
203185_at	9,154	9,510	9,273	7,010	6,765	7,242
203186_s_at	7,718	7,952	7,497	7,633	10,213	7,731
203187_at	8,020	7,950	7,842	7,769	8,098	7,734
203188_at	10,057	10,109	10,302	9,686	9,106	9,323
203189_s_at	7,999	7,680	7,945	7,806	7,259	7,939
203190_at	9,724	9,771	10,154	10,052	9,386	10,195
203191_at	5,660	5,432	6,015	7,724	8,009	8,041
203192_at	8,677	8,722	8,616	8,721	8,438	8,908
203193_at	8,674	8,168	8,495	8,590	8,487	8,641
203194_s_at	8,909	8,767	9,276	8,076	8,007	7,858
203195_s_at	9,680	9,755	9,575	8,113	8,524	8,187
203196_at	9,348	9,374	9,180	8,900	9,974	8,871
203197_s_at	10,625	10,633	10,609	10,172	9,601	10,172
203198_at	9,198	8,932	8,914	8,169	7,099	8,000
203199_s_at	8,778	8,793	8,414	8,532	7,893	7,785
203200_s_at	9,421	9,554	9,354	9,723	10,512	10,023
203201_at	9,127	8,947	9,445	9,132	9,346	9,454
203202_at	10,265	10,476	10,053	9,104	9,785	9,029
203203_s_at	9,277	9,321	8,394	8,625	8,288	8,191
203204_s_at	10,183	10,252	10,632	8,364	7,973	8,594
203205_at	9,014	8,915	8,929	8,074	8,032	8,095

203206_at	8,808	8,699	8,855	8,931	8,815	9,018
203207_s_at	9,663	10,019	9,098	8,781	8,722	8,964
203208_s_at	11,579	12,121	11,368	10,907	10,728	10,977
203209_at	11,207	11,244	11,549	10,433	9,286	9,371
203210_s_at	8,536	8,227	8,439	7,684	6,614	7,013
203211_s_at	10,135	10,283	9,869	9,732	10,327	9,517
203212_s_at	9,833	9,845	9,744	8,764	9,341	8,495
203213_at	12,198	12,419	11,754	10,221	10,992	10,483
203214_x_at	10,425	10,780	9,673	8,475	8,659	8,200
203215_s_at	8,532	9,272	8,389	7,303	8,318	7,593
203216_s_at	9,335	10,338	9,181	7,261	9,579	7,828
203217_s_at	7,987	8,549	7,908	8,041	8,169	8,321
203218_at	9,527	9,816	9,644	9,598	9,663	9,434
203219_s_at	10,365	10,172	10,642	10,376	9,793	10,585
203220_s_at	7,192	7,249	7,386	7,429	7,523	7,642
203221_at	11,623	11,564	12,291	8,479	8,355	8,205
203222_s_at	9,916	9,848	10,609	7,246	6,598	6,821
203223_at	6,719	6,898	6,228	6,616	7,097	6,723
203224_at	10,566	11,127	10,370	10,833	11,041	11,086
203225_s_at	9,560	9,831	9,677	10,650	10,098	10,265
203226_s_at	8,063	8,032	7,764	7,650	7,817	8,287
203227_s_at	7,897	8,415	8,567	8,999	8,702	9,787
203228_at	10,452	10,454	10,714	9,350	8,390	8,998
203229_s_at	11,856	11,811	11,981	9,295	9,214	9,146
203230_at	8,609	8,903	9,161	9,777	9,677	9,985
203231_s_at	5,873	6,023	5,390	6,385	5,374	5,133
203232_s_at	6,927	7,577	7,005	6,577	8,032	6,541
203233_at	8,593	8,474	8,519	8,699	8,470	8,558
203234_at	8,907	9,211	9,362	8,996	8,695	9,124
203235_at	8,355	8,473	8,409	8,186	7,268	7,945
203236_s_at	6,490	6,383	6,924	7,465	7,478	7,413
203237_s_at	7,469	7,523	7,589	7,782	7,807	7,972
203238_s_at	7,443	7,442	7,776	6,635	7,473	7,791
203239_s_at	7,133	6,740	7,094	6,369	6,294	6,854
203240_at	6,919	6,961	7,038	7,498	7,776	7,571
203241_at	9,419	9,627	9,717	8,602	9,040	8,456
203242_s_at	6,179	6,210	6,185	8,113	8,187	8,685
203243_s_at	7,334	7,508	7,063	8,411	10,013	9,486
203244_at	10,307	10,482	10,476	8,512	8,207	8,953
203245_s_at	9,348	9,295	9,845	9,219	8,709	9,079
203246_s_at	9,583	9,569	9,471	9,573	8,595	9,318
203247_s_at	9,924	9,853	9,424	8,029	7,795	7,531
203248_at	7,408	7,117	7,054	7,027	7,025	6,710
203249_at	8,910	9,132	8,663	8,323	8,581	8,283
203250_at	10,390	10,489	10,587	8,527	9,416	8,640
203252_at	8,535	8,208	8,597	9,127	8,527	9,150
203253_s_at	10,167	10,211	9,703	8,430	9,822	8,242
203254_s_at	6,452	6,353	6,679	7,730	7,741	8,043
203255_at	8,668	8,903	8,762	6,978	8,453	6,864
203256_at	9,464	10,162	9,696	7,794	7,640	7,635
203257_s_at	9,441	9,445	9,447	8,893	8,734	8,988
203258_at	9,072	8,742	8,518	10,106	9,870	10,557
203259_s_at	9,988	10,149	9,844	10,342	9,554	9,808
203260_at	7,534	7,081	7,292	7,110	6,927	6,634
203261_at	9,920	10,123	9,513	9,795	9,642	9,826
203262_s_at	8,823	8,959	9,067	9,874	9,627	10,163

203263_s_at	9,480	9,555	9,574	7,315	7,368	7,627
203264_s_at	9,403	8,742	8,935	7,634	7,184	7,068
203265_s_at	7,659	8,093	7,665	7,797	7,778	7,915
203266_s_at	9,753	9,810	9,494	8,044	8,958	8,153
203267_s_at	7,761	7,771	7,836	8,141	7,861	8,201
203268_s_at	8,470	8,417	8,467	8,763	8,057	8,691
203269_at	11,510	11,338	11,586	11,561	11,619	11,417
203270_at	9,229	9,180	9,672	9,404	8,548	9,115
203271_s_at	8,865	8,970	8,875	8,160	8,135	8,281
203272_s_at	8,515	8,437	8,620	9,051	8,297	9,033
203273_s_at	9,111	8,946	9,266	8,864	8,645	8,829
203274_at	9,098	9,041	9,744	9,680	8,935	9,715
203275_at	6,485	6,882	6,774	6,659	5,906	5,852
203276_at	10,285	9,466	9,516	7,380	7,105	6,759
203277_at	8,704	8,588	8,650	8,397	7,909	8,275
203278_s_at	9,657	10,026	9,841	7,968	8,670	8,325
203279_at	8,525	8,944	8,630	9,183	9,615	9,189
203280_at	9,384	8,597	9,080	8,440	8,678	8,389
203281_s_at	7,254	7,354	7,132	7,981	7,796	8,192
203282_at	9,629	9,505	10,078	11,624	12,744	12,312
203283_s_at	9,431	9,022	8,886	9,044	9,570	9,006
203284_s_at	9,814	9,814	9,656	8,410	9,309	8,095
203285_s_at	9,218	9,082	8,869	7,836	8,701	7,431
203286_at	11,474	11,585	11,927	8,061	8,283	8,176
203287_at	7,471	7,418	7,885	8,241	7,943	7,992
203288_at	10,166	10,347	10,381	7,854	8,427	8,246
203289_s_at	7,188	7,053	7,081	7,936	7,862	8,225
203290_at	5,868	5,645	5,436	6,462	6,234	6,404
203291_at	9,278	9,020	9,570	8,455	8,637	8,376
203292_s_at	8,410	8,363	8,674	8,576	8,688	8,747
203293_s_at	6,924	7,200	6,872	6,624	7,137	7,188
203294_s_at	6,288	5,888	5,705	6,414	6,506	6,265
203295_s_at	8,056	8,211	7,821	7,004	6,315	6,496
203296_s_at	9,188	9,576	9,118	5,265	5,353	5,356
203297_s_at	10,246	10,354	10,498	8,529	9,559	8,737
203298_s_at	11,717	12,025	12,116	8,803	9,987	8,607
203299_s_at	11,108	10,405	11,276	8,491	8,550	8,967
203300_x_at	10,633	9,972	10,420	7,643	7,407	7,966
203301_s_at	10,526	10,473	9,893	8,054	9,518	8,213
203302_at	9,875	10,058	9,514	9,159	9,189	9,178
203303_at	9,852	10,487	9,797	10,516	11,375	11,222
203304_at	10,926	11,196	11,333	7,550	7,868	8,424
203305_at	5,636	5,694	5,513	5,782	5,913	5,718
203306_s_at	9,839	10,452	9,621	9,802	10,330	9,904
203307_at	8,671	8,550	8,606	9,563	9,676	9,680
203308_x_at	7,955	8,005	8,117	8,520	8,396	8,621
203309_s_at	6,858	6,538	7,011	7,223	7,013	7,424
203310_at	10,729	11,185	10,255	10,063	11,028	10,074
203311_s_at	7,784	7,709	7,800	8,205	7,468	8,106
203312_x_at	9,120	9,065	9,528	10,076	9,902	10,181
203313_s_at	10,998	11,438	11,331	8,773	8,552	8,885
203314_at	9,148	9,159	9,576	9,740	9,209	9,638
203315_at	9,786	9,951	10,135	9,153	8,401	8,844
203316_s_at	13,653	13,713	13,675	12,921	12,355	12,724
203317_at	6,194	6,342	6,098	6,614	6,210	6,340
203318_s_at	9,062	9,087	8,706	7,990	8,882	7,924

203319_s_at	8,779	8,691	8,179	6,369	7,335	6,273
203320_at	8,718	8,755	8,740	11,223	11,231	11,015
203321_s_at	10,622	10,721	10,643	8,607	8,912	8,638
203322_at	11,274	11,122	11,169	9,067	9,688	9,126
203323_at	4,859	5,754	4,662	9,408	11,280	10,790
203324_s_at	4,942	6,435	5,042	11,487	12,247	12,623
203325_s_at	7,733	7,539	7,679	9,411	10,167	10,750
203326_x_at	6,944	6,930	7,094	7,063	7,006	7,078
203327_at	10,084	10,308	10,191	9,037	10,190	9,130
203328_x_at	9,006	9,067	8,980	8,635	8,879	8,105
203329_at	8,109	8,390	7,941	7,862	9,800	8,932
203330_s_at	7,526	7,416	7,543	8,289	7,879	8,238
203331_s_at	6,239	6,068	6,430	6,791	7,063	7,222
203332_s_at	7,547	7,647	8,200	7,436	7,344	7,525
203333_at	8,049	8,537	7,894	8,344	8,780	7,941
203334_at	9,137	9,003	9,266	8,034	8,043	7,728
203335_at	10,699	11,506	10,604	10,708	10,475	10,980
203336_s_at	9,250	9,586	9,211	10,989	10,460	10,858
203337_x_at	8,605	8,575	8,312	8,794	9,092	8,709
203338_at	11,135	11,265	11,223	9,693	10,473	9,950
203339_at	8,857	8,974	9,098	9,668	9,176	8,266
203340_s_at	8,753	9,303	9,030	9,286	9,048	8,833
203341_at	11,433	11,769	11,057	9,262	9,570	8,124
203342_at	9,717	9,520	9,406	9,003	8,497	8,989
203343_at	10,911	10,712	10,975	12,077	12,873	12,316
203344_s_at	10,790	10,869	10,234	9,437	9,882	8,515
203345_s_at	12,254	12,608	11,719	8,745	8,786	8,240
203346_s_at	11,576	11,946	11,147	7,603	8,354	6,927
203347_s_at	8,801	8,852	8,184	5,278	5,825	4,982
203348_s_at	7,871	7,768	7,891	7,318	6,991	7,318
203349_s_at	11,085	11,003	11,370	9,026	9,606	9,753
203350_at	9,343	9,539	9,798	9,179	9,888	9,513
203351_s_at	11,337	12,062	11,458	10,179	10,439	10,212
203352_at	8,852	9,447	8,957	6,774	6,808	6,559
203353_s_at	10,047	9,925	10,221	10,442	10,357	10,660
203354_s_at	8,026	7,951	7,965	6,329	8,461	6,786
203355_s_at	9,936	9,753	9,513	8,486	11,122	7,888
203356_at	9,453	9,935	9,209	7,626	8,763	7,803
203357_s_at	8,042	8,339	7,607	6,558	6,923	6,505
203358_s_at	11,904	11,052	11,208	8,460	8,953	8,096
203359_s_at	10,793	10,578	10,527	10,223	10,187	10,058
203360_s_at	9,931	9,934	9,825	9,997	9,358	9,809
203361_s_at	8,958	9,106	8,989	9,035	8,899	8,642
203362_s_at	12,280	12,440	11,949	11,479	11,169	11,379
203363_s_at	8,807	8,642	8,743	8,971	8,855	9,155
203364_s_at	9,278	9,268	9,470	8,041	8,484	8,928
203365_s_at	8,398	8,340	8,591	8,209	8,317	8,237
203366_at	9,925	10,378	10,317	10,094	10,229	10,533
203367_at	8,547	8,543	8,923	10,238	9,898	10,421
203368_at	7,661	8,281	7,795	8,029	7,778	8,135
203369_x_at	8,089	7,612	7,497	8,678	8,636	8,688
203370_s_at	7,883	7,260	7,547	7,393	7,016	7,811
203371_s_at	10,349	10,533	10,081	10,562	10,439	10,672
203372_s_at	6,967	6,911	6,250	4,859	5,145	5,472
203373_at	11,534	11,701	11,853	7,729	9,080	9,152
203374_s_at	9,415	9,469	8,909	8,471	8,444	8,018

203375_s_at	9,949	9,903	9,560	8,041	8,566	7,681
203376_at	10,190	10,619	9,875	9,314	9,583	8,784
203377_s_at	6,972	7,386	6,509	6,899	6,862	6,346
203378_at	9,358	8,913	8,783	7,130	7,679	7,328
203379_at	8,745	8,808	8,699	7,455	6,790	6,956
203380_x_at	12,428	12,442	12,017	11,245	11,818	11,259
203381_s_at	10,034	10,325	9,907	7,998	8,370	8,490
203382_s_at	10,388	10,772	10,456	7,635	7,631	7,804
203383_s_at	8,150	8,294	8,148	8,126	8,571	8,032
203384_s_at	7,036	7,179	7,076	6,590	7,395	6,916
203385_at	7,667	7,478	7,461	7,925	8,235	8,380
203386_at	9,508	9,752	9,360	8,207	9,528	8,855
203387_s_at	8,086	7,893	7,920	6,930	7,383	7,084
203388_at	7,287	7,404	7,115	7,552	7,064	7,499
203389_at	9,059	8,970	9,191	8,242	7,887	8,453
203390_s_at	8,632	8,656	8,933	8,714	7,808	8,408
203391_at	9,098	9,003	9,256	9,501	9,032	9,401
203392_s_at	10,473	9,874	10,256	8,940	8,901	9,408
203393_at	7,045	6,587	6,623	6,881	6,828	6,667
203394_s_at	8,948	8,951	8,868	8,402	11,011	10,828
203395_s_at	9,606	9,456	9,431	8,689	10,960	10,636
203396_at	13,313	13,599	13,120	12,333	12,437	12,697
203397_s_at	4,331	4,421	4,659	6,150	7,734	7,827
203398_s_at	6,452	6,168	6,435	5,941	6,173	6,016
203399_x_at	6,158	5,669	5,934	7,005	6,541	6,933
203400_s_at	6,228	5,958	7,196	5,783	5,822	6,060
203401_at	10,711	10,664	10,301	10,281	10,348	10,029
203402_at	7,245	7,477	7,515	8,187	7,600	8,085
203403_s_at	9,498	9,797	8,935	9,043	10,478	8,494
203404_at	12,180	12,137	12,152	10,745	11,432	11,755
203405_at	12,018	11,806	12,135	11,955	11,278	11,287
203406_at	11,421	11,357	10,833	10,075	10,263	9,926
203407_at	9,822	10,399	10,190	8,009	8,661	7,966
203408_s_at	8,968	9,231	8,926	7,364	7,100	6,786
203409_at	8,230	8,537	8,031	8,758	8,528	8,764
203410_at	10,342	10,332	10,388	8,043	8,437	7,744
203411_s_at	8,379	8,321	8,624	11,338	11,435	11,907
203412_at	8,226	8,162	8,429	9,068	8,587	8,713
203413_at	11,686	11,707	11,263	6,925	6,761	6,860
203414_at	11,628	12,103	11,362	11,247	11,285	10,735
203415_at	10,243	10,450	10,521	10,408	9,775	10,400
203416_at	7,802	7,913	7,804	7,954	7,587	7,689
203417_at	10,200	9,917	10,282	8,380	8,419	8,890
203418_at	9,836	9,822	9,886	8,966	8,451	8,529
203419_at	9,017	8,649	9,233	8,578	8,640	8,366
203420_at	8,985	9,546	8,935	8,763	9,867	9,531
203421_at	8,106	7,692	8,053	7,107	7,399	7,215
203422_at	10,017	9,778	10,117	9,504	8,779	9,076
203423_at	8,378	8,222	8,466	7,696	7,446	7,597
203424_s_at	6,916	7,232	7,076	7,396	8,140	7,081
203425_s_at	6,570	6,333	6,857	7,040	7,194	7,354
203426_s_at	6,691	6,806	7,290	7,942	7,551	7,974
203427_at	8,941	9,064	8,318	8,411	9,134	8,955
203428_s_at	9,715	9,659	9,213	9,659	8,873	9,715
203429_s_at	10,469	10,898	9,987	8,707	9,348	8,414
203430_at	9,104	9,641	9,357	11,200	10,406	11,186

203431_s_at	8,876	8,804	9,003	6,713	7,002	6,699
203432_at	10,671	10,568	10,929	9,809	10,192	9,678
203433_at	8,262	8,391	8,422	8,523	8,087	8,191
203434_s_at	6,957	7,482	5,910	9,696	10,018	8,751
203435_s_at	7,983	8,127	7,741	9,353	10,032	9,041
203436_at	9,658	9,733	9,844	10,495	9,706	10,238
203437_at	8,472	8,800	9,028	9,377	8,462	9,202
203438_at	8,567	9,098	9,214	9,650	11,218	11,196
203439_s_at	6,477	7,019	7,431	10,000	10,713	11,191
203440_at	10,108	9,884	10,222	10,254	10,123	10,436
203441_s_at	7,790	7,505	7,696	7,811	7,721	7,878
203442_x_at	7,689	8,021	7,709	8,567	8,335	8,671
203443_at	6,873	6,950	6,961	7,450	7,188	7,279
203444_s_at	6,355	6,191	6,195	6,825	6,599	6,825
203445_s_at	11,690	11,855	12,076	10,483	10,495	10,674
203446_s_at	8,830	8,851	8,818	9,120	9,371	9,363
203447_at	10,617	10,630	10,377	10,281	10,623	10,503
203448_s_at	11,098	11,111	11,599	8,223	8,136	8,006
203449_s_at	11,668	11,607	12,017	7,714	8,417	7,549
203450_at	9,441	9,383	9,636	9,818	9,161	9,767
203451_at	8,303	8,043	8,255	7,766	7,385	7,804
203452_at	8,213	8,077	8,180	8,274	8,027	8,616
203453_at	10,618	11,043	11,662	6,451	6,319	6,265
203454_s_at	9,575	9,803	9,781	11,044	10,655	11,181
203455_s_at	10,691	11,491	10,925	12,638	12,609	12,648
203456_at	8,431	8,508	8,550	9,020	8,852	9,661
203457_at	6,624	7,086	6,617	7,320	7,140	7,098
203458_at	8,889	8,983	8,964	9,659	8,979	9,496
203459_s_at	10,060	10,200	10,207	9,612	9,509	9,780
203460_s_at	9,267	9,095	9,451	9,013	9,686	9,399
203461_at	5,653	5,516	5,744	5,459	6,068	5,803
203462_x_at	13,020	12,650	13,081	12,150	12,059	12,422
203463_s_at	8,098	8,062	8,147	8,760	8,238	8,672
203464_s_at	8,100	8,049	8,007	7,694	7,897	7,918
203465_at	10,607	10,567	10,430	10,265	10,134	10,529
203466_at	9,241	8,820	9,050	9,818	9,158	10,100
203467_at	7,426	7,795	7,418	8,831	8,518	9,132
203468_at	9,523	9,499	9,501	9,027	8,883	9,118
203469_s_at	8,561	8,473	8,326	7,752	7,474	7,719
203470_s_at	7,601	7,672	7,678	8,201	7,973	7,779
203471_s_at	6,018	6,017	6,210	6,685	6,369	6,273
203472_s_at	6,014	6,131	5,868	6,505	6,337	6,465
203473_at	6,706	6,952	7,265	7,674	7,445	7,677
203474_at	9,314	9,455	9,106	5,726	5,601	5,519
203475_at	5,435	5,283	5,174	6,588	6,209	6,113
203476_at	10,384	10,463	10,492	11,918	12,128	12,347
203477_at	4,589	4,508	4,454	4,115	4,001	4,027
203478_at	10,820	11,004	11,135	11,642	11,229	11,599
203479_s_at	8,628	8,692	8,801	8,055	8,346	8,190
203480_s_at	10,202	10,092	9,904	9,313	10,025	8,895
203481_at	8,510	8,128	7,778	7,166	8,370	6,823
203482_at	9,500	9,429	9,179	7,500	8,345	7,065
203483_at	6,893	6,364	6,643	7,413	7,058	7,077
203484_at	11,611	11,719	11,407	11,855	11,382	12,019
203485_at	9,755	9,713	9,883	4,947	5,337	4,922
203486_s_at	9,854	9,526	9,455	8,628	9,157	8,795

203487_s_at	9,467	8,921	9,261	7,100	8,560	7,386
203488_at	8,325	8,035	8,475	7,578	7,455	7,689
203489_at	8,334	8,070	8,404	8,823	8,402	8,950
203490_at	7,422	7,557	7,539	8,806	8,212	8,529
203491_s_at	8,552	9,002	8,174	6,773	6,162	6,159
203492_x_at	8,941	9,332	8,426	7,892	7,506	7,214
203493_s_at	10,099	10,102	9,306	6,848	7,515	5,878
203494_s_at	9,745	10,249	9,478	7,823	8,335	6,916
203495_at	7,568	7,405	7,590	7,426	7,647	7,748
203496_s_at	8,460	8,300	8,675	7,684	8,275	7,678
203497_at	10,760	10,333	10,367	8,879	10,183	8,622
203498_at	7,941	8,018	7,429	6,732	10,352	9,520
203499_at	8,650	8,384	8,683	7,967	8,167	8,635
203500_at	8,937	8,572	8,759	8,352	7,862	8,150
203501_at	6,004	5,886	5,778	7,771	8,015	8,283
203502_at	9,241	9,343	8,605	8,245	8,831	9,121
203503_s_at	8,575	8,448	8,783	8,806	8,133	8,394
203504_s_at	8,778	8,724	8,848	5,616	5,969	5,254
203505_at	10,385	10,649	10,402	7,074	7,200	6,770
203506_s_at	8,160	7,971	8,127	8,109	7,962	8,311
203507_at	6,640	6,861	6,706	8,026	7,914	7,853
203508_at	7,150	7,569	7,658	8,549	8,354	8,511
203509_at	9,258	9,360	9,982	7,866	7,620	7,921
203510_at	10,639	10,776	10,218	10,999	11,936	10,970
203511_s_at	10,160	10,697	10,377	10,033	8,592	10,551
203512_at	10,204	10,248	10,249	10,600	10,039	9,786
203513_at	10,419	11,108	10,215	8,264	9,727	8,419
203514_at	8,568	8,438	8,683	8,382	7,959	8,662
203515_s_at	9,156	8,830	8,857	8,543	8,178	8,708
203516_at	7,387	7,448	7,530	7,276	7,154	7,332
203517_at	11,181	11,300	10,563	9,734	8,991	9,564
203518_at	5,406	6,210	5,404	7,442	8,937	7,118
203519_s_at	9,731	9,660	9,099	8,401	9,680	7,908
203520_s_at	7,308	7,360	7,440	7,110	7,070	7,415
203521_s_at	9,308	9,188	9,355	7,297	7,833	6,907
203522_at	8,727	8,919	8,689	7,864	7,594	8,061
203523_at	7,260	7,247	7,189	7,237	7,103	7,320
203524_s_at	9,614	9,838	9,700	9,467	8,513	9,440
203525_s_at	8,835	8,979	8,308	8,362	10,027	7,794
203526_s_at	6,386	6,533	6,495	6,238	6,816	6,529
203527_s_at	8,070	7,524	7,933	8,440	8,553	8,165
203528_at	9,046	9,703	9,864	6,838	6,617	6,711
203529_at	10,832	10,920	11,104	10,586	10,360	10,488
203530_s_at	8,727	8,774	8,979	9,035	8,471	8,941
203531_at	10,819	11,269	10,455	9,071	10,467	8,888
203532_x_at	8,006	8,192	7,652	7,359	7,689	6,987
203533_s_at	8,612	8,685	8,074	7,187	7,816	7,061
203534_at	11,204	11,223	11,037	11,197	11,014	11,485
203535_at	6,205	6,039	5,830	6,756	6,722	6,918
203536_s_at	9,048	9,188	9,179	10,818	9,374	10,407
203537_at	10,497	10,810	10,483	9,480	9,678	9,555
203538_at	10,932	11,734	11,204	11,113	10,860	11,153
203539_s_at	7,305	7,145	7,375	7,339	7,168	7,524
203540_at	5,781	5,951	5,996	6,101	5,940	6,112
203541_s_at	6,695	6,199	6,598	6,698	6,289	6,313
203542_s_at	7,385	7,392	6,949	7,663	9,377	8,050

203543_s_at	5,441	6,603	5,419	6,088	9,831	7,822
203544_s_at	10,308	10,777	10,173	10,379	11,024	10,553
203545_at	11,309	10,871	11,382	10,872	10,561	10,512
203546_at	8,261	8,168	8,329	8,236	7,943	8,451
203547_at	8,131	7,936	7,879	8,648	8,411	8,709
203548_s_at	7,652	7,767	7,121	5,664	5,892	5,532
203549_s_at	8,645	8,917	8,627	7,165	7,763	7,239
203550_s_at	10,922	10,892	11,052	11,139	10,948	11,322
203551_s_at	8,770	9,059	8,842	8,333	8,372	8,500
203552_at	9,505	9,459	9,032	9,002	10,162	8,821
203553_s_at	8,307	8,444	7,718	8,286	8,883	8,203
203554_x_at	12,771	13,137	12,662	12,512	11,823	12,061
203555_at	8,382	8,090	8,458	9,102	8,849	9,122
203556_at	8,903	9,014	9,348	7,198	7,864	7,945
203557_s_at	8,040	7,870	7,775	8,330	8,071	8,591
203558_at	7,669	7,985	7,571	8,604	8,421	8,691
203559_s_at	5,667	5,244	5,674	7,227	6,674	6,863
203560_at	10,889	11,108	10,317	11,331	11,243	11,398
203561_at	5,861	5,860	5,231	6,737	6,349	6,749
203562_at	12,131	12,263	12,363	9,826	8,878	9,251
203563_at	6,664	6,755	6,702	5,296	6,814	7,104
203564_at	11,429	11,434	11,848	11,057	9,914	10,243
203565_s_at	8,809	9,083	8,679	8,661	8,999	8,826
203566_s_at	10,963	11,225	10,741	8,094	9,524	8,064
203567_s_at	8,141	8,219	8,316	8,419	9,219	8,947
203568_s_at	8,193	8,322	8,236	8,404	8,858	8,669
203569_s_at	9,477	9,774	9,351	7,497	9,139	8,543
203570_at	8,265	8,299	8,146	9,160	8,758	9,644
203571_s_at	7,462	7,262	7,133	7,960	8,374	8,240
203572_s_at	9,662	9,622	9,974	9,452	8,870	9,183
203573_s_at	7,870	7,942	8,023	8,672	8,003	8,723
203574_at	8,969	9,549	9,208	8,530	10,202	9,294
203575_at	9,268	9,428	10,066	9,453	9,223	9,449
203576_at	8,591	8,547	8,391	9,163	8,957	9,551
203577_at	8,136	8,175	8,150	7,764	7,238	7,633
203578_s_at	7,038	6,853	7,706	8,070	8,071	8,590
203579_s_at	7,253	6,847	7,459	7,082	7,411	7,502
203580_s_at	9,500	9,285	9,589	8,349	8,794	8,107
203581_at	10,742	11,114	10,842	10,238	10,214	10,434
203582_s_at	8,670	9,017	8,286	8,094	6,976	7,762
203583_at	10,766	11,103	10,670	11,156	10,539	11,002
203584_at	10,219	10,766	9,561	9,538	10,219	8,897
203585_at	8,320	8,749	8,208	8,667	9,190	8,774
203586_s_at	8,500	8,682	8,665	8,812	8,762	8,925
203587_at	5,457	5,604	5,283	5,735	5,566	5,631
203588_s_at	9,707	10,528	9,798	8,183	7,885	8,112
203589_s_at	9,186	9,811	9,072	6,085	6,302	6,323
203590_at	10,325	10,072	9,837	9,324	10,399	9,788
203591_s_at	6,008	6,046	6,023	6,224	6,089	5,961
203592_s_at	8,316	8,347	8,357	9,356	8,719	9,563
203593_at	10,118	10,391	9,931	7,587	9,101	7,323
203594_at	11,449	11,462	10,932	11,217	11,356	11,258
203595_s_at	7,407	7,368	7,220	9,305	9,439	8,890
203596_s_at	6,857	7,067	7,014	8,719	9,554	8,552
203597_s_at	8,188	8,414	7,742	6,923	7,289	6,605
203598_s_at	9,001	9,442	8,569	8,574	8,946	8,383

203599_s_at	9,254	9,317	8,313	7,104	7,814	7,052
203600_s_at	9,766	9,706	10,317	8,772	9,181	8,850
203601_s_at	7,044	7,267	7,158	8,358	8,718	8,721
203602_s_at	8,493	8,518	8,857	8,769	8,232	8,536
203603_s_at	8,017	7,634	7,084	6,971	9,003	6,752
203604_at	7,613	7,812	7,618	6,426	6,285	6,656
203605_at	11,062	11,207	10,195	10,224	11,123	10,482
203606_at	12,790	12,917	13,107	12,648	12,379	12,597
203607_at	9,284	9,767	8,976	8,155	9,195	8,250
203608_at	8,237	8,321	8,146	5,281	5,826	6,000
203609_s_at	7,035	6,998	7,194	7,823	7,980	8,034
203610_s_at	6,927	6,951	6,865	7,521	7,703	8,215
203611_at	8,838	8,658	8,987	8,024	7,844	8,259
203612_at	9,659	9,274	9,788	10,040	9,326	9,821
203613_s_at	12,198	12,351	12,101	12,420	11,971	12,298
203614_at	10,851	10,653	10,518	8,609	9,770	8,465
203615_x_at	8,485	8,600	8,555	7,392	7,102	7,683
203616_at	11,784	12,016	12,012	10,124	9,857	10,061
203617_x_at	9,566	9,638	9,621	10,289	9,825	9,912
203618_at	7,625	7,492	7,839	8,279	8,313	8,140
203619_s_at	6,007	6,378	6,377	6,629	6,074	6,583
203620_s_at	7,425	7,667	7,260	6,998	7,586	7,159
203621_at	12,158	12,553	12,147	11,581	11,675	11,892
203622_s_at	10,676	10,464	10,745	10,844	10,511	10,383
203623_at	7,396	7,218	7,381	8,017	8,092	8,612
203624_at	10,163	10,419	10,501	9,369	9,539	9,670
203625_x_at	11,060	10,662	10,904	9,219	8,643	8,309
203626_s_at	6,677	6,570	6,635	6,626	6,621	6,699
203627_at	8,355	7,650	8,594	6,751	8,256	8,302
203628_at	8,902	8,600	8,918	7,222	8,150	7,415
203629_s_at	7,892	8,475	8,164	7,835	8,723	8,410
203630_s_at	8,969	9,349	8,605	8,718	9,329	8,949
203631_s_at	7,114	6,735	6,997	7,070	6,608	6,964
203632_s_at	10,474	10,740	11,459	5,846	6,156	6,297
203633_at	6,562	7,087	7,182	9,128	9,211	9,194
203634_s_at	5,236	5,963	5,326	7,566	6,642	7,421
203635_at	9,614	9,963	9,939	9,402	9,100	9,221
203636_at	11,203	11,389	11,437	8,442	9,178	8,161
203637_s_at	10,993	11,152	11,234	8,807	9,474	8,595
203638_s_at	11,428	11,552	11,905	6,920	6,806	6,708
203639_s_at	9,091	9,071	8,976	6,481	6,050	6,487
203640_at	5,109	5,249	5,508	7,212	8,365	7,373
203641_s_at	6,502	6,459	6,398	6,414	6,582	6,682
203642_s_at	7,165	7,158	6,536	6,290	6,541	6,089
203643_at	7,064	6,964	7,226	7,641	7,161	7,166
203644_s_at	8,789	9,099	8,919	8,831	8,867	8,997
203645_s_at	6,161	6,011	5,870	6,248	6,278	6,535
203646_at	9,546	9,608	9,668	10,899	10,426	10,903
203647_s_at	10,321	10,487	10,517	11,725	11,138	11,641
203648_at	10,154	9,816	10,336	9,552	9,824	9,853
203649_s_at	6,778	7,807	7,434	7,416	7,286	7,415
203650_at	8,232	8,902	7,961	10,229	10,005	9,650
203651_at	9,657	10,097	9,168	7,492	8,996	7,134
203652_at	8,242	8,001	8,535	8,309	8,246	8,380
203653_s_at	7,343	7,021	7,054	7,476	6,934	7,003
203654_s_at	10,805	11,273	10,902	10,034	10,081	9,507

203655_at	8,106	8,200	8,319	7,919	7,488	7,825
203656_at	9,421	9,890	9,029	8,414	9,304	8,764
203657_s_at	7,279	7,468	7,174	7,894	7,976	8,216
203658_at	7,609	7,553	7,575	8,701	8,211	9,084
203659_s_at	10,845	11,249	10,865	9,739	9,356	9,494
203660_s_at	10,020	9,758	10,366	8,642	9,380	8,752
203661_s_at	8,136	8,313	8,235	8,508	8,389	8,672
203662_s_at	7,094	7,489	7,535	7,436	7,451	7,835
203663_s_at	13,024	13,194	13,232	12,873	12,587	12,883
203664_s_at	10,033	9,900	9,712	9,242	8,377	8,856
203665_at	7,952	7,876	7,962	11,901	10,703	12,016
203666_at	9,727	9,441	9,839	8,162	7,540	7,925
203667_at	12,653	12,961	12,956	12,885	12,757	13,017
203668_at	8,123	7,895	8,006	7,359	7,613	7,826
203669_s_at	7,887	7,845	7,754	8,179	7,495	8,327
203670_at	6,743	6,450	6,914	7,048	6,961	6,909
203671_at	7,725	7,632	7,811	8,341	8,758	8,220
203672_x_at	8,220	8,726	8,303	8,952	8,871	9,256
203673_at	6,929	7,171	7,222	7,898	7,456	7,478
203674_at	8,683	8,731	8,799	7,778	8,391	7,466
203675_at	7,820	7,833	7,108	8,587	9,290	8,205
203676_at	6,581	6,909	7,142	7,496	7,266	7,585
203677_s_at	9,793	9,631	9,959	9,732	8,695	9,527
203678_at	9,341	9,392	9,273	8,353	8,531	8,359
203679_at	9,307	9,437	9,264	9,963	9,045	9,671
203680_at	10,798	10,758	10,623	5,916	5,638	5,593
203681_at	5,606	5,509	5,731	6,189	5,530	5,912
203682_s_at	8,463	8,350	8,631	9,082	7,825	8,462
203683_s_at	7,587	7,160	7,222	7,777	7,258	7,681
203684_s_at	6,762	6,409	6,566	7,239	6,686	6,821
203685_at	6,036	5,417	5,669	5,187	5,077	5,173
203686_at	7,645	7,922	8,009	9,167	8,575	9,320
203687_at	7,425	7,705	7,724	7,543	7,242	7,727
203688_at	9,711	9,521	9,698	7,741	9,157	8,262
203689_s_at	8,281	8,291	8,115	6,870	7,425	6,507
203690_at	8,343	8,554	8,580	7,137	7,280	6,708
203691_at	6,205	6,404	6,196	7,214	6,913	7,171
203692_s_at	9,647	9,419	9,499	8,740	8,743	9,258
203693_s_at	11,223	10,690	11,087	8,698	9,683	9,324
203694_s_at	9,946	9,923	10,217	9,778	9,761	9,909
203695_s_at	9,439	9,984	9,656	10,444	9,864	10,054
203696_s_at	9,606	9,695	9,834	9,410	8,503	9,101
203697_at	8,587	7,942	7,946	5,770	5,446	5,401
203698_s_at	7,121	6,882	6,535	5,642	5,428	5,731
203699_s_at	6,389	6,014	5,942	5,678	6,021	5,801
203700_s_at	7,220	6,428	6,470	7,278	7,009	7,456
203701_s_at	10,488	10,102	10,574	9,708	9,194	9,951
203702_s_at	9,169	9,069	9,349	8,343	7,998	8,178
203703_s_at	7,688	7,221	7,903	6,799	7,022	7,003
203704_s_at	8,744	8,902	9,229	7,305	8,391	7,741
203705_s_at	10,721	10,733	11,055	7,581	8,983	9,374
203706_s_at	11,435	11,415	11,543	6,570	8,544	8,450
203707_at	10,252	10,423	10,698	8,838	8,760	8,842
203708_at	10,321	9,873	10,209	6,292	7,398	6,574
203709_at	8,529	8,032	8,354	8,014	7,697	8,056
203710_at	7,752	8,347	7,444	7,094	6,773	6,304

203711_s_at	7,753	7,630	6,961	7,085	7,155	6,257
203712_at	11,219	11,415	10,742	9,899	10,446	9,045
203713_s_at	8,132	8,237	8,208	7,769	7,872	8,039
203714_s_at	11,388	11,848	11,431	10,759	10,618	10,563
203715_at	8,897	9,059	8,791	8,108	7,618	7,771
203716_s_at	7,288	7,603	7,087	9,174	8,951	7,376
203717_at	7,666	7,914	7,282	9,438	9,485	8,160
203718_at	8,893	8,646	8,476	8,543	8,425	9,175
203719_at	8,306	8,299	8,817	10,376	10,094	10,606
203720_s_at	9,067	8,942	9,433	11,321	11,005	11,478
203721_s_at	12,344	12,534	12,310	11,653	11,305	11,124
203722_at	6,899	6,541	6,762	7,180	7,292	7,315
203723_at	8,557	8,403	8,727	7,142	6,887	7,339
203724_s_at	6,141	6,670	5,984	4,934	5,949	5,335
203725_at	10,004	10,579	10,128	10,556	10,632	11,257
203726_s_at	6,048	6,294	6,665	6,515	6,917	6,290
203727_at	8,845	8,707	8,712	9,041	8,767	9,132
203728_at	7,827	7,687	7,537	8,080	7,507	8,083
203729_at	7,223	7,253	6,700	10,273	9,794	10,688
203730_s_at	8,628	8,724	8,371	6,809	7,337	6,515
203731_s_at	8,526	8,394	8,676	8,328	8,359	8,461
203732_at	9,715	9,922	9,638	9,769	10,155	9,444
203733_at	8,528	8,740	8,879	8,861	8,238	8,946
203734_at	10,831	10,887	10,906	9,498	9,662	9,626
203735_x_at	8,439	8,819	8,720	8,643	8,867	8,607
203736_s_at	6,718	6,780	6,687	6,774	7,206	6,453
203737_s_at	9,506	9,385	10,056	8,381	8,258	8,520
203738_at	10,467	10,429	10,195	9,388	9,361	9,197
203739_at	10,766	10,617	10,353	7,422	8,024	7,277
203740_at	11,616	11,980	11,558	11,083	11,162	10,946
203741_s_at	7,498	7,782	7,401	7,915	9,020	8,817
203742_s_at	9,323	8,649	9,080	7,564	8,345	7,850
203743_s_at	12,981	12,701	12,694	10,757	11,367	10,975
203744_at	11,508	11,491	11,279	9,631	9,258	9,166
203745_at	10,603	10,492	10,642	9,177	8,782	9,154
203746_s_at	11,064	11,122	11,375	11,220	10,432	10,857
203747_at	7,060	7,006	7,224	7,372	7,012	7,336
203748_x_at	10,327	10,497	10,076	9,305	10,426	9,902
203749_s_at	6,651	6,415	7,068	7,134	7,183	7,533
203750_s_at	7,688	7,728	7,927	8,728	8,904	9,099
203751_x_at	6,947	7,013	6,888	7,084	7,129	7,119
203752_s_at	11,849	12,252	12,119	11,061	10,919	11,407
203753_at	9,904	9,828	9,892	7,735	8,265	8,127
203754_s_at	8,118	7,819	7,915	8,057	7,699	8,056
203755_at	12,424	12,687	11,974	10,513	10,796	9,067
203756_at	8,518	8,366	8,379	8,642	9,051	9,113
203757_s_at	5,569	5,440	5,369	6,069	5,789	5,938
203758_at	6,520	6,530	6,548	6,875	7,336	7,357
203759_at	7,014	6,977	6,996	8,371	7,478	7,718
203760_s_at	7,524	7,564	7,586	8,545	8,572	8,826
203761_at	7,408	7,054	7,481	7,943	7,420	7,420
203762_s_at	8,393	8,942	7,845	7,764	8,376	7,474
203763_at	9,316	9,937	8,800	8,151	9,138	8,335
203764_at	11,573	12,112	11,014	9,274	10,133	7,521
203765_at	9,057	10,052	8,905	6,436	6,863	6,241
203766_s_at	7,071	6,759	6,579	6,545	7,318	7,216

203767_s_at	6,420	5,940	7,074	7,498	8,889	7,529
203768_s_at	6,542	6,134	6,210	6,187	6,255	6,081
203769_s_at	6,343	6,101	6,496	6,451	8,022	7,161
203770_s_at	6,156	6,201	6,596	7,693	7,580	7,785
203771_s_at	8,380	8,390	8,039	8,201	7,691	8,000
203772_at	6,247	5,824	6,196	6,783	6,403	6,512
203773_x_at	10,500	10,803	10,582	10,766	10,341	10,479
203774_at	10,282	10,502	10,162	8,347	9,813	8,217
203775_at	11,806	11,275	11,705	10,392	9,749	9,318
203776_at	9,847	9,820	9,840	9,503	8,883	9,441
203777_s_at	8,966	8,966	9,287	9,215	8,984	9,389
203778_at	7,870	8,605	7,831	7,451	7,403	7,382
203779_s_at	4,707	4,615	5,136	4,998	4,891	4,771
203780_at	4,984	5,483	5,140	3,869	3,852	3,789
203781_at	10,833	11,052	10,700	11,861	11,319	11,677
203782_s_at	9,203	8,999	9,370	8,680	7,834	8,550
203783_x_at	6,808	6,832	6,908	6,941	6,732	6,901
203784_s_at	6,820	6,650	6,795	7,602	7,453	7,500
203785_s_at	8,668	8,369	8,769	8,055	6,797	7,379
203786_s_at	9,171	10,011	9,264	8,481	8,714	9,457
203787_at	9,865	10,251	9,704	8,929	9,422	9,748
203788_s_at	5,525	5,843	5,833	6,915	7,147	6,675
203789_s_at	4,414	4,337	4,410	9,293	11,304	9,418
203790_s_at	10,324	10,596	9,974	9,231	9,033	9,033
203791_at	8,870	9,551	8,581	7,211	9,472	6,999
203792_x_at	7,500	7,193	7,784	7,758	7,553	7,458
203793_x_at	8,387	8,398	8,451	8,103	7,831	7,954
203794_at	9,767	9,829	9,499	8,224	9,048	7,693
203795_s_at	7,803	7,785	7,824	7,736	7,387	7,668
203796_s_at	7,474	7,474	7,667	7,159	6,727	7,064
203797_at	9,133	9,494	9,694	7,157	6,898	6,896
203798_s_at	5,733	6,428	6,210	4,905	4,708	4,963
203799_at	7,154	8,117	6,813	6,501	6,844	6,322
203800_s_at	11,783	12,153	11,888	11,332	10,979	11,653
203801_at	7,943	7,858	7,203	5,773	6,558	6,094
203802_x_at	10,239	10,040	10,343	9,177	9,069	9,481
203803_at	8,302	8,364	8,043	7,463	8,123	8,037
203804_s_at	12,339	12,301	12,013	10,169	11,687	9,731
203805_s_at	8,431	8,406	8,278	8,054	7,861	8,081
203806_s_at	8,347	8,006	7,952	7,298	7,420	7,442
203807_x_at	6,378	6,477	6,707	6,974	6,879	6,969
203808_at	5,943	5,961	5,903	6,488	6,221	6,451
203809_s_at	6,194	5,920	6,087	6,513	6,538	6,682
203810_at	9,174	9,635	8,859	9,114	10,813	11,211
203811_s_at	7,568	7,833	6,687	7,118	8,030	8,795
203812_at	7,428	7,001	7,329	8,062	8,117	8,217
203813_s_at	6,778	6,520	6,712	6,518	6,299	7,018
203814_s_at	9,874	10,254	10,244	9,546	8,870	9,361
203815_at	7,801	8,231	7,852	6,729	8,319	6,654
203816_at	9,253	9,159	9,295	8,850	8,121	8,946
203817_at	7,348	7,719	6,962	7,441	7,003	6,728
203818_s_at	11,626	11,244	11,398	9,241	8,864	8,371
203819_s_at	12,560	12,964	12,220	10,105	11,153	10,231
203820_s_at	13,122	13,139	12,797	10,477	11,696	10,579
203821_at	8,244	7,739	8,150	9,712	9,417	10,078
203822_s_at	9,104	9,277	8,818	6,994	7,865	7,072

203823_at	8,834	9,294	9,031	10,027	9,463	9,501
203824_at	4,285	4,475	4,288	4,908	5,144	4,854
203825_at	11,177	10,744	11,474	8,246	8,418	8,335
203826_s_at	8,276	7,772	8,054	7,790	7,845	8,090
203827_at	6,567	7,374	6,362	9,657	9,101	9,730
203828_s_at	6,757	6,641	6,937	7,765	7,501	7,827
203829_at	9,310	9,886	9,293	9,314	8,637	8,946
203830_at	9,374	9,641	8,861	7,591	7,331	7,482
203831_at	8,732	9,162	9,020	8,538	8,662	8,636
203832_at	11,052	11,146	11,060	10,668	9,606	9,818
203833_s_at	8,028	8,134	8,208	8,761	8,707	9,452
203834_s_at	7,760	7,842	8,370	9,077	8,632	8,957
203835_at	7,344	7,484	8,049	8,003	7,630	8,701
203836_s_at	6,218	6,842	6,763	6,803	7,124	6,914
203837_at	7,433	7,675	7,163	7,936	9,651	8,185
203838_s_at	6,835	6,695	6,021	7,743	7,606	7,766
203839_s_at	8,032	8,017	8,153	8,467	8,632	8,494
203840_at	7,734	7,910	7,026	7,542	9,116	7,681
203841_x_at	5,658	5,725	6,129	6,607	6,150	6,469
203842_s_at	7,210	7,435	7,532	7,615	7,884	7,949
203843_at	8,502	8,513	8,194	8,368	8,701	7,734
203844_at	4,100	4,192	4,188	4,166	4,384	4,566
203845_at	7,077	7,055	6,263	7,505	8,758	7,645
203846_at	9,905	9,925	10,144	9,549	8,724	9,317
203847_s_at	9,841	9,871	10,170	10,085	9,779	9,993
203848_at	9,297	9,349	9,494	9,038	9,004	8,869
203849_s_at	7,672	7,611	7,902	6,820	6,567	6,985
203850_s_at	4,211	3,941	4,365	4,613	4,243	4,056
203851_at	7,282	7,347	7,512	10,603	10,591	10,946
203852_s_at	10,456	10,527	9,810	10,132	9,684	10,126
203853_s_at	9,161	9,245	9,073	8,645	8,565	8,837
203854_at	5,394	4,974	5,252	5,749	5,809	5,672
203855_at	8,981	9,273	8,651	7,615	8,633	7,720
203856_at	11,128	11,358	10,550	9,370	9,420	8,814
203857_s_at	8,886	9,453	9,048	10,271	9,722	10,155
203858_s_at	9,049	9,449	9,317	9,091	8,669	9,038
203859_s_at	8,128	8,214	8,335	8,430	8,019	8,607
203860_at	9,384	9,717	8,717	7,698	7,757	7,252
203861_s_at	7,795	7,765	7,694	7,780	7,810	7,758
203862_s_at	4,496	4,887	4,454	4,174	4,203	4,186
203863_at	4,729	5,398	5,026	4,603	4,463	4,716
203864_s_at	6,483	6,651	6,905	7,094	6,641	6,756
203865_s_at	8,683	8,800	8,848	5,933	6,790	6,932
203866_at	8,334	7,709	8,343	8,043	7,808	8,045
203867_s_at	9,032	9,075	9,333	9,605	8,519	9,352
203868_s_at	5,559	5,454	5,060	8,602	12,819	10,947
203869_at	9,367	9,230	9,154	7,859	7,545	7,334
203870_at	9,482	9,884	9,408	8,127	8,697	7,756
203871_at	8,889	8,849	9,069	9,344	8,577	9,026
203872_at	6,455	6,380	6,652	6,399	6,262	6,647
203873_at	5,052	4,945	5,167	4,880	4,813	4,898
203874_s_at	10,123	10,248	9,605	7,720	9,115	8,155
203875_at	11,484	11,571	10,974	8,996	10,230	9,340
203876_s_at	6,732	6,784	6,909	7,473	7,439	7,682
203877_at	6,668	6,500	6,619	6,681	6,767	7,086
203878_s_at	7,772	7,818	7,884	7,847	7,816	7,848

203879_at	7,742	8,711	8,035	8,952	7,628	7,727
203880_at	9,954	10,255	9,884	10,464	9,111	9,710
203881_s_at	9,685	9,569	9,043	4,641	6,480	6,183
203882_at	8,020	7,768	8,104	8,532	7,869	8,375
203883_s_at	9,009	9,077	8,704	7,412	8,055	7,251
203884_s_at	9,069	9,479	8,672	7,464	8,772	6,869
203885_at	10,785	10,708	10,662	9,661	9,978	9,841
203886_s_at	8,985	9,299	8,798	7,486	7,771	8,079
203887_s_at	4,888	4,983	4,880	9,396	10,790	8,720
203888_at	6,114	6,193	6,136	8,569	9,605	7,941
203889_at	11,600	11,838	11,448	9,352	9,258	9,275
203890_s_at	5,887	5,263	5,976	6,322	6,041	6,286
203891_s_at	8,595	8,460	8,582	8,732	8,602	8,778
203892_at	8,160	8,417	8,752	7,528	7,362	7,513
203893_at	10,813	11,017	10,480	10,902	11,073	11,198
203894_at	7,543	7,851	7,695	7,839	7,865	7,947
203895_at	8,859	9,452	8,261	7,425	8,392	6,539
203896_s_at	8,371	8,808	8,142	7,566	7,994	7,622
203897_at	10,943	11,036	10,665	10,471	10,736	10,922
203898_at	9,328	9,002	9,545	8,636	8,524	8,900
203899_s_at	9,082	8,992	9,188	8,756	8,496	8,986
203900_at	6,309	5,863	5,966	6,487	6,258	6,811
203901_at	6,763	6,740	6,410	7,004	6,787	7,188
203902_at	5,929	6,602	5,636	5,341	5,300	5,488
203903_s_at	9,958	10,677	10,009	5,853	7,788	7,042
203904_x_at	8,295	8,613	8,663	10,318	8,857	9,678
203905_at	10,873	10,923	11,095	9,482	9,651	9,171
203906_at	8,675	8,719	8,846	8,142	8,132	8,272
203907_s_at	8,573	8,477	8,567	7,779	7,966	7,814
203908_at	5,658	5,689	5,801	5,572	5,584	6,496
203909_at	9,679	9,864	9,795	9,135	9,960	9,164
203910_at	10,017	10,136	9,741	10,946	12,281	11,368
203911_at	9,562	9,616	9,484	9,390	9,182	9,463
203912_s_at	7,914	8,268	8,066	9,587	9,718	10,511
203913_s_at	3,685	4,007	3,936	3,761	3,727	3,705
203914_x_at	6,032	6,491	6,355	5,733	6,042	5,810
203915_at	6,097	6,073	5,779	7,669	7,310	7,471
203916_at	8,964	8,889	9,046	9,303	9,363	9,679
203917_at	13,802	14,020	13,592	6,870	6,844	7,052
203918_at	8,211	8,037	8,174	8,813	8,806	8,820
203919_at	8,795	8,923	8,878	6,851	6,677	7,176
203920_at	7,790	7,783	7,681	7,735	6,946	7,659
203921_at	9,728	10,068	9,990	10,669	10,530	11,050
203922_s_at	6,368	6,660	6,427	7,290	7,146	7,273
203923_s_at	6,575	6,440	6,831	6,470	6,255	6,535
203924_at	5,221	4,819	4,891	5,527	5,351	5,347
203925_at	9,247	9,392	9,681	12,378	11,998	12,335
203926_x_at	9,246	9,323	9,626	9,578	9,139	9,555
203927_at	8,580	9,116	8,859	9,009	8,673	8,914
203928_x_at	7,845	7,612	7,662	8,015	7,858	7,899
203929_s_at	6,617	7,124	6,518	6,267	6,490	6,397
203930_s_at	6,076	6,342	6,251	6,986	6,972	7,201
203931_s_at	9,472	9,097	9,754	10,290	8,919	10,183
203932_at	7,939	7,804	8,294	7,781	7,714	7,647
203933_at	8,750	8,226	8,455	8,751	8,604	8,985
203934_at	9,072	9,358	9,086	7,386	8,656	5,971

203935_at	10,073	9,995	10,211	9,791	10,414	10,471
203936_s_at	7,860	7,927	8,068	7,794	7,368	7,641
203937_s_at	7,394	7,297	7,382	8,049	7,358	7,833
203938_s_at	10,738	10,491	11,080	8,676	8,824	8,884
203939_at	5,075	5,124	4,584	10,686	11,397	10,609
203940_s_at	8,197	8,495	8,368	8,110	7,945	8,257
203941_at	8,779	8,804	9,099	7,911	7,240	7,724
203942_s_at	7,926	8,032	8,031	7,650	6,930	7,605
203943_at	8,649	8,194	8,415	7,842	8,629	7,702
203944_x_at	9,144	9,280	9,168	9,017	9,382	9,252
203945_at	7,945	8,190	8,361	7,498	7,261	7,440
203946_s_at	9,247	9,380	9,067	7,569	6,356	6,953
203947_at	11,042	10,799	10,679	10,279	11,062	10,258
203948_s_at	6,793	6,694	6,339	7,023	6,880	7,209
203949_at	6,206	5,923	6,192	6,420	6,486	6,641
203950_s_at	8,416	8,683	8,373	7,638	7,837	8,027
203951_at	8,329	8,502	7,786	7,983	7,443	7,847
203952_at	8,917	9,089	8,880	9,171	9,199	9,183
203953_s_at	5,542	6,035	5,368	6,549	6,317	6,675
203954_x_at	7,354	7,268	7,544	7,540	7,532	7,540
203955_at	10,385	10,462	10,471	8,551	8,572	8,318
203956_at	10,852	10,903	11,074	10,577	10,707	10,645
203957_at	10,611	10,879	10,954	9,047	8,773	8,912
203958_s_at	7,449	7,518	7,833	7,638	7,408	7,501
203959_s_at	8,958	9,026	9,509	8,340	8,774	8,276
203960_s_at	9,001	9,305	8,599	9,017	8,220	8,742
203961_at	9,072	9,248	9,135	5,602	5,609	5,825
203962_s_at	9,741	9,967	9,405	4,778	4,830	4,761
203963_at	8,161	8,073	8,041	10,967	11,656	11,739
203964_at	10,166	10,348	9,972	8,859	9,230	9,187
203965_at	8,923	8,549	8,698	8,004	8,188	8,431
203966_s_at	9,555	9,827	9,684	8,973	9,680	9,126
203967_at	9,087	8,452	9,117	7,763	6,831	6,712
203968_s_at	9,718	9,187	9,429	7,476	7,310	6,966
203969_at	5,563	5,989	5,612	6,834	6,697	6,640
203970_s_at	8,603	8,734	8,180	8,549	8,931	8,376
203971_at	10,603	10,728	11,074	11,176	10,993	11,310
203972_s_at	8,763	8,917	8,345	9,447	9,064	8,956
203973_s_at	7,033	7,566	7,543	9,775	11,196	10,760
203974_at	10,121	10,397	10,392	10,732	9,731	9,587
203975_s_at	8,894	8,598	8,823	8,200	8,050	8,218
203976_s_at	9,654	9,292	9,497	8,249	8,069	8,095
203977_at	7,522	7,597	7,500	7,594	7,379	7,728
203978_at	9,504	9,304	9,821	8,803	8,176	8,583
203979_at	8,969	9,127	8,878	8,987	8,813	9,140
203980_at	5,315	5,015	5,858	7,018	8,236	10,688
203981_s_at	8,985	9,042	8,857	8,423	7,765	8,091
203982_s_at	6,299	6,258	6,536	6,638	6,443	6,621
203983_at	11,161	11,083	10,593	9,551	10,267	9,579
203984_s_at	9,268	9,466	9,661	8,976	9,078	9,114
203985_at	9,267	9,351	9,412	7,716	7,576	7,815
203986_at	7,209	7,471	7,530	8,201	8,083	8,181
203987_at	8,876	9,151	8,424	6,639	7,549	7,600
203988_s_at	8,880	8,669	8,457	7,578	7,213	6,900
203989_x_at	8,163	7,899	7,876	4,692	7,395	5,084
203990_s_at	5,057	5,182	5,053	5,099	4,944	5,108

203991_s_at	8,470	8,633	8,174	6,402	6,192	5,583
203992_s_at	8,915	9,113	8,476	6,813	6,976	6,614
203993_x_at	6,696	6,865	6,778	6,608	6,519	6,562
203994_s_at	7,911	7,961	7,947	7,770	7,489	7,690
203995_at	7,481	7,391	7,379	7,507	7,655	7,798
203996_s_at	8,723	8,330	8,944	8,212	8,374	8,435
203997_at	8,252	8,954	8,079	7,928	7,806	7,668
203998_s_at	6,051	6,161	5,198	4,432	4,555	4,805
203999_at	8,953	9,234	8,543	5,756	6,572	5,982
204000_at	8,488	8,580	8,506	8,553	8,320	8,257
204001_at	9,070	9,009	8,812	8,473	8,985	8,512
204002_s_at	6,545	6,458	6,865	7,172	6,850	7,312
204003_s_at	9,816	9,558	9,131	8,590	8,422	8,505
204004_at	10,261	10,247	9,647	6,786	7,611	7,774
204005_s_at	9,110	9,233	8,906	8,215	7,896	9,266
204006_s_at	4,395	4,555	4,984	5,804	6,022	6,203
204007_at	5,676	5,837	5,906	5,984	5,919	5,963
204008_at	8,088	8,072	8,116	8,252	7,433	7,937
204009_s_at	13,224	13,217	13,014	9,709	10,438	9,494
204010_s_at	5,527	5,758	5,750	5,663	5,855	5,793
204011_at	10,207	10,690	10,765	9,884	10,337	10,217
204012_s_at	9,390	9,104	9,601	9,654	8,996	9,416
204013_s_at	5,997	5,955	6,062	5,789	5,666	5,578
204014_at	8,866	8,768	9,138	6,761	7,969	7,289
204015_s_at	8,508	8,150	8,177	8,369	8,274	7,979
204016_at	9,097	8,989	9,460	8,211	7,878	8,143
204017_at	8,500	9,305	8,477	12,442	12,955	13,524
204018_x_at	7,863	7,916	8,068	8,353	7,978	8,338
204019_s_at	8,545	9,005	8,327	8,462	9,088	8,417
204020_at	9,092	9,384	9,219	9,257	10,228	9,529
204021_s_at	7,701	7,772	7,511	8,344	8,024	7,970
204022_at	9,777	9,489	10,211	9,678	9,638	9,801
204023_at	11,829	11,894	11,670	9,888	9,662	9,248
204024_at	5,970	5,902	6,124	6,315	6,335	5,974
204025_s_at	8,714	8,902	9,260	9,821	9,133	9,663
204026_s_at	12,727	12,325	12,653	12,048	11,634	11,658
204027_s_at	9,416	8,903	9,810	9,240	8,496	9,127
204028_s_at	10,544	10,759	10,493	8,471	9,203	8,487
204029_at	9,644	9,234	9,776	6,857	6,744	6,858
204030_s_at	10,789	10,983	10,632	9,136	9,587	9,721
204031_s_at	12,726	12,817	13,001	12,010	11,526	12,079
204032_at	8,770	8,864	8,779	9,189	8,567	9,773
204033_at	10,761	10,744	10,765	10,697	10,400	10,260
204034_at	8,231	8,566	8,307	9,645	8,774	9,894
204035_at	8,169	7,906	7,412	6,710	6,840	6,728
204036_at	7,756	7,857	7,889	9,953	11,222	11,021
204037_at	7,516	7,928	7,357	9,376	10,904	10,438
204038_s_at	8,529	8,334	7,972	9,340	9,590	9,996
204039_at	7,526	7,688	7,542	8,001	7,666	7,918
204040_at	9,864	9,880	10,020	6,369	6,195	6,093
204041_at	8,624	9,197	9,203	7,482	7,601	7,375
204042_at	10,429	10,644	10,213	8,444	9,123	8,421
204043_at	8,496	8,413	8,415	7,479	7,493	7,809
204044_at	10,467	10,634	10,117	7,253	6,923	8,016
204045_at	8,503	8,937	8,193	10,146	10,144	10,690
204046_at	7,501	7,449	7,716	7,347	7,182	7,252

204047_s_at	8,124	7,915	7,935	7,446	7,040	7,193
204048_s_at	9,468	9,133	9,472	7,623	8,838	8,380
204049_s_at	9,981	9,564	9,831	7,350	9,643	8,606
204050_s_at	11,262	11,196	11,437	11,261	10,820	11,329
204051_s_at	6,285	6,049	6,156	5,162	5,459	5,378
204052_s_at	4,733	4,621	4,661	4,826	4,646	4,768
204053_x_at	9,143	9,958	9,297	10,036	10,381	10,071
204054_at	7,368	7,712	7,510	6,814	7,201	6,837
204055_s_at	6,843	6,092	4,978	4,253	4,430	4,104
204056_s_at	8,910	8,555	8,554	8,862	8,655	8,904
204057_at	7,267	7,886	7,662	7,327	7,476	7,221
204058_at	4,155	4,134	3,507	9,566	10,288	9,905
204059_s_at	6,166	6,588	5,361	11,120	11,532	11,506
204060_s_at	10,249	10,156	10,260	7,546	8,512	7,611
204061_at	9,756	9,623	9,721	6,672	6,902	6,576
204062_s_at	7,468	8,049	7,222	6,227	6,477	6,351
204063_s_at	7,868	8,524	7,697	6,560	6,567	6,500
204064_at	9,846	9,790	9,610	9,241	9,388	8,470
204065_at	8,778	8,808	8,840	7,977	7,827	7,683
204066_s_at	9,609	9,521	9,758	8,872	8,488	9,204
204067_at	8,571	8,399	8,773	8,226	7,804	8,208
204068_at	8,442	9,136	8,212	7,937	8,721	7,919
204069_at	5,286	5,696	4,888	6,401	6,987	6,202
204070_at	6,586	6,790	6,728	7,434	7,249	7,676
204071_s_at	9,963	9,620	9,197	7,283	7,598	7,004
204072_s_at	7,405	7,856	7,529	4,932	5,653	5,399
204073_s_at	8,317	7,967	8,024	7,382	7,089	7,259
204074_s_at	8,638	8,466	8,421	9,043	8,741	8,991
204075_s_at	6,160	6,179	5,993	6,026	6,402	6,576
204076_at	10,671	11,079	10,512	9,318	10,699	9,212
204077_x_at	8,071	7,689	7,979	7,746	7,785	7,746
204078_at	8,268	8,443	8,512	9,522	9,441	9,838
204079_at	9,277	9,692	9,592	9,878	9,625	10,559
204080_at	9,940	9,746	10,350	9,668	8,661	9,066
204081_at	7,646	7,544	7,952	7,856	7,499	7,759
204082_at	11,200	11,665	11,564	8,256	8,899	8,242
204083_s_at	10,896	10,582	10,754	12,339	11,929	12,929
204084_s_at	7,586	7,848	7,705	9,521	9,509	10,048
204085_s_at	6,860	7,185	6,613	7,360	8,312	8,141
204086_at	5,886	5,968	5,898	6,263	6,101	6,156
204087_s_at	10,489	9,703	10,556	9,383	8,450	9,051
204088_at	7,817	7,772	7,487	8,062	7,047	7,850
204089_x_at	10,420	10,087	10,519	9,349	10,140	8,913
204090_at	7,695	7,609	7,760	8,116	7,979	8,411
204091_at	9,538	9,908	9,702	9,418	8,470	9,159
204092_s_at	11,629	11,878	11,736	10,892	10,267	10,139
204093_at	9,963	10,509	9,522	10,939	11,096	11,199
204094_s_at	9,958	9,655	9,802	7,975	8,867	8,960
204095_s_at	6,579	6,713	6,830	7,364	7,117	7,125
204096_s_at	7,717	7,184	7,508	7,200	6,971	7,294
204097_s_at	8,555	8,416	8,508	8,592	7,995	8,389
204098_at	9,639	9,801	9,721	9,563	8,980	9,242
204099_at	8,285	8,519	8,471	7,852	8,340	8,841
204100_at	7,368	7,383	7,386	7,927	7,712	8,132
204101_at	7,599	7,939	7,256	6,926	7,203	6,438
204102_s_at	13,389	13,479	13,392	12,738	12,429	12,952

204103_at	7,366	7,346	7,153	7,805	7,579	7,844
204104_at	7,378	7,209	7,452	8,090	7,861	7,985
204105_s_at	9,529	9,395	9,097	7,584	7,395	7,291
204106_at	8,183	8,096	8,288	8,320	7,947	8,520
204107_at	6,898	7,392	7,060	6,615	6,522	6,536
204108_at	8,813	8,487	8,281	7,314	7,613	7,201
204109_s_at	6,794	6,770	6,466	7,944	7,154	7,728
204110_at	5,441	5,654	5,778	5,844	5,990	5,678
204111_at	4,964	4,804	5,018	4,948	4,857	4,996
204112_s_at	9,369	9,091	8,700	8,855	9,415	8,866
204113_at	8,258	8,316	8,642	8,250	7,719	7,965
204114_at	7,551	8,428	7,853	10,648	12,208	11,680
204115_at	7,677	7,927	7,784	12,235	11,887	11,730
204116_at	7,370	7,125	7,315	7,565	7,614	7,617
204117_at	9,817	9,933	9,505	9,473	9,328	9,503
204118_at	6,672	6,892	6,876	7,402	7,143	7,244
204119_s_at	8,834	9,144	8,648	10,418	10,268	10,429
204120_s_at	5,659	6,018	5,717	9,619	9,609	9,629
204121_at	8,466	9,095	8,866	7,110	7,203	7,311
204122_at	5,651	5,329	5,693	6,728	6,716	6,783
204123_at	9,575	9,204	9,413	7,782	7,443	7,618
204124_at	5,693	5,908	5,914	5,576	5,696	5,557
204125_at	10,004	10,190	9,891	10,802	9,918	10,161
204126_s_at	8,984	8,784	8,590	8,911	8,192	8,583
204127_at	12,222	12,231	12,054	10,508	10,389	9,834
204128_s_at	9,569	9,505	8,838	6,725	6,225	6,372
204129_at	8,881	8,750	9,074	7,220	7,144	7,251
204130_at	8,077	7,892	8,126	6,019	5,932	6,081
204131_s_at	9,876	10,097	10,272	9,318	11,057	10,326
204132_s_at	7,788	7,282	7,551	7,466	7,501	7,564
204133_at	9,238	8,760	9,173	9,122	8,101	8,438
204134_at	8,793	9,273	8,813	7,945	7,708	8,140
204135_at	5,783	5,789	5,359	6,937	7,697	6,894
204136_at	7,423	7,389	7,780	8,045	8,459	8,534
204137_at	7,876	8,429	7,681	8,155	7,886	8,070
204138_s_at	7,351	7,177	7,430	8,131	8,166	8,168
204139_x_at	7,424	7,012	7,355	7,763	7,855	7,922
204140_at	9,596	9,963	9,593	10,666	10,443	10,515
204141_at	12,217	12,263	12,883	12,076	12,321	13,085
204142_at	10,033	9,949	9,974	10,813	10,765	11,018
204143_s_at	9,604	9,485	9,575	9,993	9,995	10,275
204144_s_at	6,594	6,551	6,937	6,857	6,524	6,991
204145_at	10,059	10,327	9,970	10,373	10,241	10,030
204146_at	13,119	13,227	12,825	9,943	9,997	9,422
204147_s_at	7,686	7,283	7,649	7,689	7,355	7,540
204148_s_at	8,427	8,758	8,566	8,232	8,011	8,250
204149_s_at	5,243	5,289	4,979	5,112	4,659	5,188
204150_at	7,120	7,578	7,229	7,834	7,848	7,942
204151_x_at	7,790	8,168	8,120	13,749	13,653	13,737
204152_s_at	6,643	7,005	6,705	7,221	7,093	7,167
204153_s_at	6,050	6,444	6,089	6,014	6,026	6,166
204154_at	9,555	9,602	9,372	7,453	7,816	8,483
204155_s_at	7,739	7,656	8,160	7,325	7,626	7,166
204156_at	6,964	6,663	6,801	5,248	5,775	5,868
204157_s_at	7,013	7,131	7,527	7,862	7,632	7,527
204158_s_at	8,071	8,244	7,985	10,046	9,972	10,427

204159_at	5,157	4,905	5,026	10,332	9,569	9,811
204160_s_at	7,799	8,087	7,738	6,540	7,375	6,532
204161_s_at	6,477	7,254	6,336	5,608	5,535	5,378
204162_at	10,994	11,563	10,433	9,875	10,183	8,186
204163_at	6,429	5,897	5,580	8,627	8,185	9,033
204164_at	7,486	7,551	7,443	7,459	6,939	7,419
204165_at	11,617	11,469	11,698	8,911	9,333	9,329
204166_at	7,724	7,660	7,765	7,775	7,635	7,860
204167_at	7,625	7,842	7,802	7,927	7,732	7,827
204168_at	8,617	8,981	8,851	9,333	8,318	9,042
204169_at	9,165	9,115	9,410	9,142	8,888	9,432
204170_s_at	13,406	13,881	13,292	13,046	12,803	13,221
204171_at	9,184	9,197	8,535	8,293	9,089	7,957
204172_at	9,725	10,000	9,582	9,685	9,508	9,469
204173_at	10,524	10,649	10,636	9,537	9,056	9,370
204174_at	6,898	6,704	6,774	7,194	7,105	7,401
204175_at	8,610	8,858	8,791	9,488	8,484	9,225
204176_at	8,819	8,727	8,449	8,025	8,755	8,005
204177_s_at	9,276	9,225	8,797	7,977	8,599	8,464
204178_s_at	10,868	10,372	10,701	9,580	9,404	9,606
204179_at	7,259	8,163	7,363	7,911	7,327	7,668
204180_s_at	8,176	8,164	7,893	7,748	7,672	7,330
204181_s_at	9,106	8,624	8,692	8,572	8,971	8,655
204182_s_at	7,210	6,923	6,796	6,062	5,698	5,621
204183_s_at	7,855	7,362	7,379	7,820	7,448	7,781
204184_s_at	8,187	7,767	8,299	7,186	6,949	6,981
204185_x_at	9,661	9,892	9,798	10,019	9,573	9,614
204186_s_at	9,813	9,888	9,676	9,641	9,045	9,182
204187_at	7,138	7,474	7,124	6,844	6,887	7,094
204188_s_at	7,705	7,634	7,778	8,137	7,991	8,216
204189_at	8,310	8,423	8,439	8,422	8,070	8,249
204190_at	9,783	10,082	9,264	7,908	9,099	7,661
204191_at	7,217	7,303	6,915	6,059	6,199	6,189
204192_at	5,588	5,485	5,292	6,080	5,957	5,690
204193_at	8,237	8,505	8,479	8,669	8,736	9,220
204194_at	10,614	10,902	10,280	7,849	10,620	8,258
204195_s_at	7,608	7,856	8,300	8,420	7,983	8,441
204196_x_at	5,939	6,043	6,192	6,384	5,993	5,981
204197_s_at	6,954	7,844	7,291	6,541	6,787	6,795
204198_s_at	6,877	7,337	7,185	6,536	6,967	6,565
204199_at	8,165	8,460	7,871	6,500	6,198	6,233
204200_s_at	6,109	6,444	6,316	6,403	6,393	6,437
204201_s_at	8,963	8,600	8,218	6,738	7,819	6,721
204202_at	8,307	7,786	8,091	7,027	7,267	7,538
204203_at	8,299	8,964	8,564	9,063	7,807	8,306
204204_at	7,375	7,620	7,399	9,528	9,238	9,070
204205_at	7,570	8,436	7,848	8,800	7,755	8,159
204206_at	9,050	9,381	9,124	8,763	9,544	9,468
204207_s_at	8,233	8,402	8,150	8,022	8,159	7,815
204208_at	8,909	9,441	8,486	8,108	8,838	8,046
204209_at	7,570	7,593	7,694	7,565	7,574	7,859
204210_s_at	5,957	5,699	5,740	7,316	6,276	6,996
204211_x_at	7,235	7,432	7,296	7,269	7,319	6,788
204212_at	8,760	9,131	9,286	8,855	8,676	9,152
204213_at	7,198	7,114	7,326	7,864	7,705	7,563
204214_s_at	8,615	8,775	8,550	10,511	10,127	10,363

204215_at	8,753	8,857	8,471	8,964	8,437	9,373
204216_s_at	10,065	10,200	9,879	9,906	9,621	9,304
204217_s_at	7,556	7,307	7,412	7,916	7,906	8,100
204218_at	8,906	8,783	9,120	9,056	8,580	9,152
204219_s_at	12,611	12,455	12,733	12,635	12,602	12,542
204220_at	6,890	6,591	6,860	8,490	8,356	8,572
204221_x_at	6,487	6,868	6,739	9,260	11,492	11,271
204222_s_at	5,212	5,605	5,856	11,518	12,400	12,606
204223_at	6,815	6,655	6,751	7,591	6,950	7,133
204224_s_at	9,339	10,029	9,416	7,386	7,181	6,687
204225_at	9,290	8,674	9,133	5,882	6,436	6,279
204226_at	9,615	9,964	9,511	7,028	8,267	7,021
204227_s_at	7,929	8,020	7,917	7,926	7,743	7,861
204228_at	12,532	12,389	12,696	11,456	10,156	10,833
204229_at	7,684	8,015	7,627	7,914	7,860	7,726
204230_s_at	7,914	7,741	7,741	7,993	7,392	7,741
204231_s_at	7,467	7,125	7,784	8,501	7,966	8,591
204232_at	6,667	6,541	6,815	7,157	6,865	7,093
204233_s_at	8,160	8,218	8,646	7,197	6,938	6,782
204234_s_at	9,944	9,997	9,584	6,281	7,235	5,960
204235_s_at	9,386	9,423	9,036	7,371	9,800	7,859
204236_at	5,877	6,181	5,996	7,127	6,169	7,120
204237_at	9,737	9,711	9,070	7,525	10,003	7,774
204238_s_at	7,850	7,895	7,627	7,810	7,193	7,830
204239_s_at	7,563	7,269	7,429	8,081	7,894	8,232
204240_s_at	10,142	10,410	9,859	8,965	9,863	7,903
204241_at	7,506	8,052	8,023	8,845	8,779	9,382
204242_s_at	7,975	8,285	7,863	8,560	7,923	8,820
204243_at	10,450	10,558	9,910	7,369	8,841	6,995
204244_s_at	11,271	11,098	10,650	8,732	8,952	7,633
204245_s_at	9,605	9,481	9,466	8,865	7,927	8,475
204246_s_at	9,929	9,944	10,018	11,249	10,970	11,502
204247_s_at	9,025	8,892	9,297	8,842	7,781	8,790
204248_at	6,949	7,182	7,378	8,054	7,263	8,124
204249_s_at	6,717	7,197	7,058	6,644	6,927	6,801
204250_s_at	6,852	6,588	6,839	7,666	7,570	7,748
204251_s_at	8,128	8,234	8,016	8,323	8,453	8,291
204252_at	9,238	9,373	9,311	8,541	7,689	8,196
204253_s_at	7,373	7,594	7,570	7,903	7,971	7,939
204254_s_at	5,223	6,171	5,710	9,118	8,661	8,717
204255_s_at	5,502	5,843	6,087	7,637	7,110	7,384
204256_at	10,570	10,464	10,268	10,603	11,546	10,621
204257_at	8,459	8,770	8,904	10,412	9,643	10,572
204258_at	10,082	10,127	9,719	7,908	9,490	7,023
204259_at	6,668	6,738	6,526	7,275	7,334	7,255
204260_at	9,035	7,737	8,557	6,068	6,123	6,228
204261_s_at	5,574	5,724	6,026	7,450	7,651	7,620
204262_s_at	9,184	9,318	9,143	8,901	8,074	8,671
204263_s_at	8,765	9,044	8,953	7,901	7,478	7,669
204264_at	11,020	10,960	11,259	9,471	9,032	9,306
204265_s_at	7,945	8,063	8,139	7,843	7,828	7,759
204266_s_at	9,163	9,175	9,198	7,526	7,570	7,545
204267_x_at	9,132	8,659	8,906	9,063	8,858	9,053
204268_at	6,654	7,012	6,814	8,900	8,004	8,189
204269_at	8,695	8,618	9,118	7,382	7,064	6,931
204270_at	6,858	6,945	7,626	7,031	7,080	7,815

204271_s_at	5,220	5,592	5,790	9,432	7,672	9,300
204272_at	6,838	6,189	6,728	6,992	6,826	7,202
204273_at	3,240	3,465	3,536	4,998	4,791	5,152
204274_at	9,874	10,122	9,673	9,689	9,510	9,586
204275_at	8,013	7,630	8,208	8,033	8,178	8,141
204276_at	7,677	7,583	7,758	7,870	8,081	8,299
204277_s_at	7,028	7,078	7,182	8,015	8,046	8,370
204278_s_at	8,455	8,628	8,181	8,075	7,141	7,729
204279_at	7,045	6,375	6,787	10,610	9,152	9,433
204280_at	6,634	6,772	6,263	7,176	6,995	7,395
204281_at	9,675	9,593	9,826	9,126	8,893	9,043
204282_s_at	8,883	9,109	9,425	10,083	9,732	9,782
204283_at	8,688	8,557	8,756	9,602	8,335	8,947
204284_at	7,857	8,148	7,879	8,606	10,080	9,934
204285_s_at	11,759	12,319	11,882	10,248	9,408	9,726
204286_s_at	11,225	11,508	11,131	9,114	8,577	8,916
204287_at	8,622	8,792	8,980	8,233	7,461	7,933
204288_s_at	7,484	7,147	7,556	7,846	8,121	7,861
204289_at	5,305	5,296	5,208	5,578	5,690	5,824
204290_s_at	8,799	9,182	8,578	7,230	7,771	7,579
204291_at	9,691	9,596	8,855	5,073	6,453	4,518
204292_x_at	7,850	7,659	7,644	8,350	8,136	8,153
204293_at	7,959	7,913	8,077	9,104	8,912	9,048
204294_at	8,837	8,979	8,963	8,150	7,956	8,112
204295_at	9,877	9,767	10,156	9,905	9,228	9,908
204296_at	4,316	4,477	4,632	4,655	4,633	4,516
204297_at	9,663	10,134	9,605	9,484	10,315	9,291
204298_s_at	5,506	5,940	5,676	8,168	8,697	10,217
204299_at	11,483	11,339	11,040	8,511	9,380	8,172
204300_at	8,685	8,784	8,720	8,830	8,414	8,723
204301_at	9,161	9,462	9,154	7,043	7,489	7,631
204302_s_at	8,073	7,867	8,331	8,770	8,580	8,916
204303_s_at	7,684	7,689	7,814	8,322	8,257	8,708
204304_s_at	10,926	10,542	10,964	4,925	4,775	4,603
204305_at	9,349	9,391	9,543	10,048	9,386	9,140
204306_s_at	8,414	8,333	8,746	9,998	9,916	10,261
204307_at	7,949	7,987	8,046	7,831	7,969	7,825
204308_s_at	8,031	8,299	8,820	8,425	8,534	8,602
204309_at	5,950	6,098	5,884	5,912	5,703	5,853
204310_s_at	6,762	6,708	6,618	6,524	6,660	6,795
204311_at	7,009	7,380	7,386	7,011	6,662	6,789
204312_x_at	8,593	8,204	7,779	7,492	7,257	7,404
204313_s_at	10,923	10,752	10,595	9,578	10,087	9,794
204314_s_at	10,313	9,688	9,793	7,484	8,743	7,897
204315_s_at	9,774	9,576	9,870	8,984	8,188	8,642
204316_at	7,068	7,378	7,602	8,101	7,904	8,071
204317_at	8,369	8,343	8,510	7,813	7,649	7,999
204318_s_at	8,837	8,592	8,845	7,868	7,271	7,617
204319_s_at	7,771	8,422	7,755	9,213	8,476	8,983
204320_at	8,204	8,516	7,374	5,553	7,108	6,278
204321_at	9,536	9,466	9,793	8,599	8,561	8,902
204322_at	8,058	7,600	7,343	8,046	7,581	7,755
204323_x_at	6,480	6,376	6,327	6,880	6,622	6,754
204324_s_at	7,688	7,529	7,482	6,912	7,425	7,233
204325_s_at	6,206	6,273	6,320	6,014	6,250	6,137
204326_x_at	10,653	10,261	11,576	11,910	11,208	12,185

204327_s_at	9,427	9,135	9,422	6,886	6,713	6,673
204328_at	7,630	7,605	7,307	6,974	6,872	6,888
204329_s_at	9,078	8,446	9,253	7,240	6,789	6,718
204330_s_at	5,485	5,504	5,242	5,500	5,148	5,510
204331_s_at	8,201	7,717	8,007	9,273	7,816	9,169
204332_s_at	9,052	9,372	9,000	9,547	9,338	9,627
204333_s_at	8,058	8,375	7,466	6,837	7,258	7,511
204334_at	8,850	8,754	8,960	8,531	9,470	8,750
204335_at	8,815	8,123	8,837	8,322	7,810	8,418
204336_s_at	8,847	9,043	9,277	9,621	8,867	9,575
204337_at	8,331	8,168	7,644	10,462	11,279	12,437
204338_s_at	5,619	5,967	5,690	7,248	7,162	8,208
204339_s_at	7,034	6,530	6,604	7,835	7,935	9,481
204340_at	7,545	8,162	7,960	8,884	8,417	8,890
204341_at	7,390	7,778	7,748	10,271	9,331	10,191
204342_at	11,164	11,295	10,997	9,676	10,478	9,874
204343_at	7,904	8,143	8,215	7,559	7,755	7,653
204344_s_at	7,824	7,964	7,466	8,392	8,615	8,785
204345_at	6,919	6,960	7,051	8,477	9,027	9,345
204346_s_at	8,992	9,031	8,918	9,666	9,005	9,633
204347_at	8,885	9,217	9,787	7,377	7,907	7,649
204348_s_at	9,477	9,594	10,150	7,496	8,470	8,370
204349_at	8,985	9,349	8,953	9,421	9,394	9,706
204350_s_at	8,964	8,980	8,775	8,920	8,623	9,105
204351_at	6,657	6,764	6,484	7,173	6,939	7,110
204352_at	9,535	9,838	9,176	5,822	7,105	5,754
204353_s_at	9,228	9,848	8,673	6,604	7,474	6,788
204354_at	10,617	11,331	10,230	8,325	9,226	7,977
204355_at	9,713	9,488	9,687	9,022	8,240	8,845
204356_at	6,970	6,835	6,933	7,519	7,262	7,379
204357_s_at	8,645	8,608	9,038	8,601	8,539	8,549
204358_s_at	7,775	7,370	7,671	7,653	7,777	7,905
204359_at	8,553	8,143	8,668	8,062	8,715	7,725
204360_s_at	8,146	8,295	8,385	8,768	8,666	8,707
204361_s_at	4,978	5,320	5,105	7,076	6,869	7,108
204362_at	6,534	6,375	6,431	9,206	9,460	9,207
204363_at	8,557	8,449	8,584	9,924	10,115	8,661
204364_s_at	7,841	8,032	7,237	4,612	4,925	5,092
204365_s_at	7,769	8,047	7,991	6,686	6,682	6,432
204366_s_at	11,727	11,494	12,273	10,717	10,580	10,703
204367_at	8,780	8,831	9,170	8,357	8,303	8,443
204368_at	7,137	7,101	7,184	7,238	7,027	7,158
204369_at	8,262	8,570	8,071	6,585	7,930	6,868
204370_at	10,481	10,391	10,789	9,983	8,999	9,568
204371_s_at	7,322	7,161	7,308	7,326	6,993	7,225
204372_s_at	11,336	10,746	11,484	10,405	10,156	10,162
204373_s_at	10,491	10,373	10,009	8,091	9,882	7,053
204374_s_at	6,189	6,020	6,231	6,940	6,410	6,760
204375_at	6,649	6,925	6,688	6,541	6,636	6,494
204376_at	8,236	8,289	8,326	5,912	6,017	5,642
204377_s_at	8,328	8,421	8,324	7,173	7,648	7,399
204378_at	5,674	6,159	5,673	5,943	5,739	5,871
204379_s_at	11,593	11,562	11,952	6,201	6,033	6,306
204380_s_at	4,866	4,928	5,326	5,487	5,307	5,487
204381_at	7,787	7,800	8,120	7,953	7,641	8,001
204382_at	8,551	8,678	8,616	8,768	8,402	8,741

204383_at	7,898	7,392	7,475	8,344	7,398	8,195
204384_at	7,576	8,199	7,512	8,052	8,181	8,705
204385_at	7,469	7,507	7,516	10,029	9,733	8,177
204386_s_at	10,834	11,157	11,427	12,188	11,533	11,947
204387_x_at	9,788	9,494	9,853	9,821	9,580	9,161
204388_s_at	6,532	7,404	6,501	8,340	10,171	9,737
204389_at	7,508	7,814	7,418	8,135	9,036	8,731
204390_at	5,966	6,124	6,285	6,946	6,817	7,071
204391_x_at	10,989	11,416	10,748	7,605	8,064	7,369
204392_at	7,857	7,290	7,259	7,745	7,469	7,901
204393_s_at	5,627	5,405	5,451	6,884	6,272	6,529
204394_at	7,490	7,638	7,590	7,778	7,332	7,639
204395_s_at	7,803	7,656	7,862	8,416	8,385	8,581
204396_s_at	7,100	7,565	7,067	8,777	8,644	8,479
204397_at	7,178	7,275	7,210	7,538	7,546	7,758
204398_s_at	8,296	8,582	8,217	8,702	8,430	8,541
204399_s_at	6,148	6,638	6,614	7,214	7,056	7,305
204400_at	9,623	9,504	9,811	5,314	6,004	8,174
204401_at	7,105	6,784	6,911	7,301	7,857	8,110
204402_at	9,334	9,035	9,441	8,776	8,448	8,708
204403_x_at	8,703	8,325	7,899	7,334	8,363	7,848
204404_at	7,465	7,248	7,694	8,227	8,195	7,531
204405_x_at	10,885	10,882	11,401	11,547	11,383	11,669
204406_at	4,900	4,918	5,084	4,902	5,081	5,042
204407_at	9,537	9,329	9,258	9,326	8,980	8,454
204408_at	9,366	9,159	9,283	9,457	8,871	9,106
204409_s_at	10,325	10,570	10,071	4,340	10,137	9,866
204410_at	9,850	10,101	10,030	4,917	9,301	8,923
204411_at	7,764	7,596	7,780	6,819	7,235	7,093
204412_s_at	8,999	7,728	8,916	7,064	7,507	7,482
204413_at	7,868	7,980	7,853	7,597	7,087	7,315
204414_at	5,039	5,286	4,857	5,844	5,998	6,066
204415_at	7,373	7,656	7,473	7,889	7,841	8,141
204416_x_at	8,295	8,643	8,699	7,793	7,506	7,674
204417_at	8,847	9,576	8,836	7,760	8,826	8,266
204418_x_at	9,361	9,443	9,349	8,838	8,998	8,859
204419_x_at	6,744	6,687	6,680	7,019	6,675	7,096
204420_at	7,165	6,941	7,379	9,307	8,331	9,042
204421_s_at	9,250	9,506	9,128	10,755	9,686	9,205
204422_s_at	8,013	8,085	7,734	9,537	8,893	7,328
204423_at	9,778	9,740	9,543	8,974	8,976	8,435
204424_s_at	10,769	10,217	9,599	6,469	7,281	6,409
204425_at	7,897	7,631	7,631	8,329	8,401	8,517
204426_at	7,269	7,096	6,480	7,616	7,230	7,523
204427_s_at	7,882	7,342	7,087	7,792	7,589	7,688
204428_s_at	7,064	6,724	7,163	7,342	7,387	7,605
204429_s_at	7,142	7,091	7,202	8,079	7,859	8,205
204430_s_at	6,539	5,817	6,457	6,741	6,700	6,662
204431_at	5,811	6,176	5,927	6,340	6,647	6,424
204432_at	9,182	9,129	9,496	7,268	7,730	7,664
204433_s_at	8,855	8,526	9,165	7,428	6,442	7,356
204434_at	8,801	8,788	8,985	7,142	7,108	7,144
204435_at	10,075	9,930	9,425	7,418	9,055	7,344
204436_at	9,087	8,942	9,273	8,842	8,592	8,860
204437_s_at	6,436	7,208	6,282	6,892	6,287	6,646
204438_at	4,982	5,864	5,042	4,763	4,607	4,636

204439_at	4,566	4,527	4,793	7,665	8,954	7,244
204440_at	9,067	9,285	9,299	9,219	8,360	8,815
204441_s_at	10,877	10,525	10,472	9,522	8,897	9,160
204442_x_at	7,740	7,631	7,539	8,176	8,341	8,393
204443_at	6,914	6,923	6,951	7,524	7,260	7,530
204444_at	10,436	10,552	10,060	8,247	9,258	6,824
204445_s_at	6,925	6,585	6,885	7,527	7,462	7,803
204446_s_at	4,933	6,075	5,816	4,322	4,373	4,321
204447_at	7,975	8,041	8,111	7,491	6,657	7,259
204448_s_at	9,959	9,949	9,981	9,285	8,591	8,904
204449_at	10,173	10,384	9,816	8,572	9,062	9,012
204450_x_at	6,808	6,696	6,935	7,373	7,116	7,365
204451_at	10,073	9,659	9,828	8,752	9,131	9,251
204452_s_at	6,147	6,192	6,393	6,573	6,344	6,482
204453_at	10,662	10,694	10,220	7,478	8,049	7,576
204454_at	8,588	8,397	8,926	9,881	9,443	10,290
204455_at	4,598	4,748	4,066	4,530	5,552	4,408
204456_s_at	5,954	5,231	5,410	6,277	6,178	6,702
204457_s_at	10,435	10,567	10,779	7,261	9,791	8,245
204458_at	7,574	7,872	7,972	8,310	8,132	8,678
204459_at	10,494	10,333	10,359	9,853	9,277	9,704
204460_s_at	8,233	8,035	8,093	8,753	7,447	8,202
204461_x_at	10,331	10,088	10,194	9,254	9,117	8,908
204462_s_at	8,996	8,751	9,282	7,456	7,207	8,312
204463_s_at	4,718	5,473	5,222	4,969	5,160	5,131
204464_s_at	7,816	8,208	8,007	5,732	7,228	7,005
204465_s_at	10,602	10,105	10,527	6,843	6,903	7,275
204466_s_at	9,249	9,598	8,700	6,590	6,437	6,535
204467_s_at	7,837	8,169	7,298	7,295	6,771	6,826
204468_s_at	6,095	6,200	5,952	5,947	5,511	5,984
204469_at	12,947	12,548	12,087	3,915	3,695	3,981
204470_at	5,467	6,166	5,801	12,418	9,687	8,360
204471_at	10,335	10,110	10,346	5,594	5,366	6,496
204472_at	8,268	9,215	8,031	9,834	9,359	8,227
204473_s_at	8,109	7,668	8,623	7,691	7,468	7,841
204474_at	8,619	8,188	8,767	8,281	8,286	8,590
204475_at	4,535	4,407	4,359	13,487	6,807	11,107
204476_s_at	7,971	7,737	7,962	7,042	7,239	7,451
204477_at	8,248	8,332	8,414	8,097	8,198	8,652
204478_s_at	8,809	8,796	9,064	10,194	9,013	9,796
204479_at	8,372	8,695	8,516	10,046	10,071	10,273
204480_s_at	7,454	7,347	7,335	6,705	6,376	7,192
204481_at	8,711	8,511	8,938	8,024	7,976	8,166
204482_at	6,238	6,407	6,398	7,251	7,105	7,184
204483_at	8,380	8,550	8,080	7,377	7,022	7,511
204484_at	10,347	9,756	10,371	8,177	7,592	7,759
204485_s_at	8,391	8,726	8,249	7,516	8,334	7,589
204486_at	5,563	5,171	5,433	7,032	7,125	7,250
204487_s_at	7,494	7,361	7,638	7,602	7,488	7,844
204488_at	9,150	8,738	9,452	9,630	8,902	9,498
204489_s_at	5,840	7,199	7,017	11,557	10,978	11,216
204490_s_at	6,777	6,505	6,136	10,243	9,253	9,820
204491_at	7,341	6,592	6,856	6,706	7,749	6,759
204492_at	7,573	7,470	7,263	6,399	7,038	5,511
204493_at	8,040	7,921	8,375	9,433	8,231	8,992
204494_s_at	8,541	8,825	8,882	8,679	8,800	8,807

204495_s_at	8,896	8,588	8,740	7,981	7,879	8,216
204496_at	10,091	10,194	9,490	8,881	10,326	8,744
204497_at	8,589	8,648	8,987	8,209	9,281	9,326
204498_s_at	6,916	6,905	7,154	6,951	6,882	7,155
204499_at	8,557	8,795	7,753	6,408	6,909	6,475
204500_s_at	10,629	10,961	9,788	7,345	8,883	6,525
204501_at	6,611	6,903	6,874	7,638	7,351	7,599
204502_at	8,644	9,395	9,101	7,965	7,604	7,291
204503_at	6,810	6,618	6,922	7,615	7,689	7,667
204504_s_at	9,229	9,089	9,475	9,082	8,809	8,864
204505_s_at	7,967	7,288	8,279	9,147	8,650	9,248
204506_at	7,999	8,165	8,068	7,624	7,816	7,423
204507_s_at	7,383	7,229	7,793	7,967	7,857	8,005
204508_s_at	6,798	6,845	6,492	8,403	8,678	9,051
204509_at	6,851	7,370	7,285	7,924	7,796	8,093
204510_at	11,808	11,645	11,066	8,065	8,032	7,000
204511_at	7,208	6,813	7,266	7,303	6,826	7,204
204512_at	7,800	7,954	7,747	6,308	6,100	5,935
204513_s_at	7,424	7,692	7,478	7,000	6,712	6,854
204514_at	10,455	9,750	10,866	9,558	8,688	9,187
204515_at	4,917	5,029	5,189	5,337	5,067	5,316
204516_at	9,046	8,693	8,615	6,956	8,565	7,237
204517_at	9,827	10,635	10,194	11,677	11,391	12,033
204518_s_at	6,735	7,547	6,968	8,724	7,948	8,730
204519_s_at	7,255	8,261	8,096	6,845	6,576	6,660
204520_x_at	9,967	9,550	10,090	8,131	8,326	8,537
204521_at	10,769	10,996	10,476	9,943	9,533	9,956
204522_at	7,451	7,061	7,381	8,411	8,213	8,431
204523_at	7,932	8,298	7,775	6,925	6,902	6,489
204524_at	9,648	9,347	9,740	8,780	9,644	9,405
204525_at	5,996	5,746	4,954	3,495	3,603	3,733
204526_s_at	8,075	8,419	8,597	8,124	9,621	8,719
204527_at	6,711	7,078	6,519	6,916	8,380	6,945
204528_s_at	12,932	13,081	12,867	11,460	11,785	11,400
204529_s_at	7,709	8,338	8,157	7,357	7,601	8,716
204530_s_at	6,291	6,036	6,042	6,923	7,045	7,286
204531_s_at	9,214	9,350	9,103	8,029	8,237	7,338
204532_x_at	7,863	7,741	7,886	7,838	8,037	7,893
204533_at	6,186	6,015	5,935	6,165	6,047	6,099
204534_at	7,034	6,867	7,179	7,278	7,775	7,706
204535_s_at	9,502	9,385	9,535	9,915	9,725	9,906
204536_s_at	6,160	6,182	6,446	6,672	6,200	6,752
204537_s_at	6,130	6,302	6,228	6,045	6,786	6,560
204538_x_at	10,583	10,447	10,507	9,920	9,608	9,585
204539_s_at	6,368	6,207	6,315	6,779	6,851	6,997
204540_at	6,906	7,204	7,196	7,324	7,184	7,550
204541_at	6,304	6,171	6,439	8,065	8,121	8,334
204542_at	6,169	6,296	5,956	6,583	6,279	6,616
204543_at	6,633	6,351	6,836	7,326	7,154	7,256
204544_at	9,214	9,442	9,200	8,556	9,190	8,960
204545_at	7,817	7,588	7,555	8,263	7,749	7,811
204546_at	8,661	8,678	8,987	7,874	8,865	8,823
204547_at	6,896	7,394	6,811	8,207	7,437	7,999
204548_at	9,469	9,594	9,482	6,501	6,374	6,224
204549_at	8,279	8,174	8,314	8,621	8,414	8,634
204550_x_at	8,375	8,260	8,220	7,687	7,729	7,838

204551_s_at	6,203	6,501	6,367	6,646	6,645	6,662
204552_at	8,354	8,186	7,886	5,320	5,940	5,663
204553_x_at	7,395	7,443	7,436	7,331	7,238	7,536
204554_at	5,980	6,559	6,272	6,468	5,955	6,384
204555_s_at	7,064	7,228	6,992	7,055	6,640	7,216
204556_s_at	9,138	9,238	8,931	8,220	8,834	7,943
204557_s_at	8,904	9,036	8,135	5,605	7,265	5,472
204558_at	10,438	10,488	10,540	8,554	7,979	8,089
204559_s_at	10,861	10,432	10,476	9,813	8,920	9,558
204560_at	7,018	7,003	6,758	7,160	7,554	7,693
204561_x_at	7,935	7,639	7,523	8,804	8,509	8,734
204562_at	7,526	7,618	7,768	7,711	7,730	7,658
204563_at	5,049	4,967	5,362	5,114	5,080	5,073
204564_at	7,703	7,851	7,280	8,158	7,878	8,011
204565_at	8,957	9,551	8,993	9,107	8,505	9,534
204566_at	9,152	9,301	9,097	7,376	7,617	7,611
204567_s_at	6,578	7,894	6,708	4,009	3,790	4,123
204568_at	9,015	9,150	9,212	7,796	8,797	8,068
204569_at	7,268	7,367	6,948	5,934	7,964	6,500
204570_at	6,738	7,290	6,739	8,593	9,495	10,038
204571_x_at	11,747	12,019	11,669	10,498	10,912	10,809
204572_s_at	8,334	8,387	8,182	7,303	7,328	7,260
204573_at	7,736	7,516	7,540	8,149	8,706	7,844
204574_s_at	7,815	7,762	7,818	8,311	8,372	8,488
204575_s_at	6,884	6,718	7,124	8,208	8,590	8,814
204576_s_at	9,645	9,727	9,396	8,870	8,580	8,695
204577_s_at	6,579	6,316	6,061	6,175	6,204	6,387
204578_at	7,076	7,492	7,202	6,871	7,095	7,060
204579_at	8,741	8,935	9,091	7,289	7,060	7,324
204580_at	3,901	4,509	4,176	4,288	4,258	4,297
204581_at	6,525	6,306	6,553	7,193	6,592	6,811
204582_s_at	6,745	6,935	6,875	8,015	7,725	7,784
204583_x_at	7,278	6,726	6,821	7,814	7,558	7,935
204584_at	8,588	8,642	8,778	6,605	6,429	6,467
204585_s_at	7,745	7,681	7,662	8,203	7,599	8,229
204586_at	5,928	5,382	5,945	5,265	5,058	5,139
204587_at	9,684	9,565	9,860	10,287	9,842	9,956
204588_s_at	8,477	8,495	8,332	7,384	7,640	7,496
204589_at	9,192	9,397	9,607	6,596	6,320	6,580
204590_x_at	7,815	7,975	8,152	7,657	7,479	7,572
204591_at	4,229	4,863	4,727	4,430	4,424	4,427
204592_at	6,061	6,357	6,141	6,076	5,726	5,808
204593_s_at	8,821	8,574	8,968	9,436	9,033	9,114
204594_s_at	9,270	8,987	9,388	8,842	8,637	8,760
204595_s_at	8,579	8,053	8,342	12,109	12,112	11,122
204596_s_at	5,722	5,708	5,757	8,251	7,369	6,909
204597_x_at	7,279	6,894	7,214	9,918	9,929	8,989
204598_at	8,384	8,746	8,208	8,308	8,206	8,364
204599_s_at	10,147	9,901	10,110	9,938	9,851	10,100
204600_at	8,319	8,035	8,229	8,234	8,342	8,588
204601_at	9,014	9,169	8,998	7,321	7,586	7,534
204602_at	6,328	5,928	5,209	11,157	11,625	11,919
204603_at	9,876	9,752	9,442	8,186	8,187	7,765
204604_at	8,936	8,446	8,654	7,467	8,625	7,883
204605_at	8,811	9,433	8,811	8,616	8,511	8,712
204606_at	6,793	6,497	6,558	7,853	8,063	7,960

204607_at	7,325	7,207	7,338	8,309	7,581	7,563
204608_at	8,919	9,173	9,022	9,935	9,383	10,245
204609_at	5,520	5,281	5,210	6,157	6,011	5,812
204610_s_at	8,920	8,542	8,769	9,456	8,560	9,572
204611_s_at	7,786	8,044	8,095	8,742	8,594	8,893
204612_at	8,838	8,744	8,728	9,555	9,149	8,815
204613_at	9,137	9,470	9,434	8,705	8,039	8,274
204614_at	5,671	5,957	6,204	12,920	11,417	12,677
204615_x_at	12,118	12,318	11,484	11,214	11,873	11,805
204616_at	10,102	10,407	10,137	10,946	11,117	11,021
204617_s_at	10,227	10,029	10,458	9,967	9,223	9,537
204618_s_at	9,612	9,428	9,012	7,287	8,020	7,359
204619_s_at	10,144	9,330	9,833	5,577	10,057	8,417
204620_s_at	10,721	10,022	10,540	7,257	10,837	8,901
204621_s_at	5,821	6,493	6,118	7,061	8,910	7,887
204622_x_at	7,487	7,845	8,080	6,598	11,011	9,350
204623_at	6,745	6,871	6,688	7,263	7,517	7,363
204624_at	8,033	8,448	8,233	7,166	7,320	7,299
204625_s_at	6,657	6,747	6,715	7,057	6,892	7,082
204626_s_at	7,039	6,862	7,040	7,866	7,442	7,332
204627_s_at	5,474	5,518	5,327	7,893	7,051	7,068
204628_s_at	7,044	6,615	6,808	9,042	7,988	8,213
204629_at	6,955	6,960	6,799	8,014	7,217	7,333
204630_s_at	10,547	10,585	10,677	9,897	10,290	10,224
204631_at	7,083	7,977	7,276	7,015	6,962	7,031
204632_at	8,074	8,160	8,028	8,420	7,929	8,179
204633_s_at	6,878	7,318	6,493	5,915	6,307	6,027
204634_at	10,246	10,298	9,794	8,665	8,970	8,037
204635_at	7,118	6,770	6,363	6,287	6,108	6,415
204636_at	7,027	6,460	6,895	7,488	7,477	7,448
204637_at	6,224	6,221	6,094	6,288	6,116	6,259
204638_at	6,615	7,225	6,791	7,293	6,973	7,140
204639_at	8,599	9,727	9,265	9,135	7,988	8,123
204640_s_at	8,762	8,735	8,493	8,942	8,625	8,887
204641_at	11,132	11,550	10,832	8,423	8,120	7,553
204642_at	6,784	6,788	6,739	6,322	6,485	6,387
204643_s_at	8,572	8,700	8,494	8,129	8,056	7,725
204644_at	7,125	7,057	7,128	6,896	6,733	6,373
204645_at	9,476	9,683	9,074	6,444	7,813	6,134
204646_at	3,890	3,900	4,287	8,302	9,231	7,891
204647_at	9,360	9,066	8,869	10,091	8,852	9,359
204648_at	8,001	8,092	7,938	8,447	8,374	8,578
204649_at	9,832	9,759	10,282	8,686	8,348	8,360
204650_s_at	7,718	7,672	7,743	7,198	7,212	7,304
204651_at	9,552	9,020	9,564	7,418	6,988	6,856
204652_s_at	8,712	8,703	8,945	7,950	7,743	7,571
204653_at	7,413	6,897	6,771	5,706	5,628	5,727
204654_s_at	6,128	6,290	6,250	6,942	6,624	7,066
204655_at	6,783	6,672	6,992	7,269	7,114	7,457
204656_at	8,259	8,067	8,270	8,597	8,417	8,636
204657_s_at	7,507	7,331	7,599	7,942	7,940	7,843
204658_at	10,168	9,971	9,282	8,664	9,212	8,546
204659_s_at	7,925	8,257	8,109	8,542	8,066	8,344
204660_at	7,929	7,961	8,111	8,667	8,295	8,862
204661_at	7,348	7,491	7,403	7,150	7,217	7,092
204662_at	9,754	9,949	9,309	7,961	8,519	7,353

204663_at	7,632	7,081	6,948	7,960	7,909	8,019
204664_at	6,025	5,598	5,830	7,095	7,154	7,300
204665_at	5,474	5,400	5,249	5,742	5,346	5,461
204666_s_at	6,337	6,026	6,625	5,578	5,290	5,303
204667_at	5,547	5,809	5,714	5,944	5,753	6,091
204668_at	8,390	8,457	9,134	8,149	8,398	8,699
204669_s_at	9,537	9,187	9,650	8,728	8,923	9,107
204670_x_at	7,815	8,032	7,836	8,224	7,884	8,290
204671_s_at	9,354	9,239	8,962	5,919	6,079	5,939
204672_s_at	7,729	7,616	6,746	4,739	4,765	4,999
204673_at	7,051	7,158	7,053	7,992	7,756	7,930
204674_at	7,264	7,620	7,584	7,839	7,501	7,811
204675_at	10,487	10,462	10,889	10,062	9,838	10,474
204676_at	9,877	10,145	10,011	9,725	9,044	9,577
204677_at	6,888	6,534	6,942	7,393	6,949	7,073
204678_s_at	7,741	8,135	7,536	6,695	6,625	7,148
204679_at	9,789	10,315	9,949	7,618	8,572	8,830
204680_s_at	6,653	6,931	6,833	6,487	6,422	6,403
204681_s_at	7,726	8,209	8,060	5,996	5,984	5,866
204682_at	7,208	7,612	7,965	9,187	9,664	11,031
204683_at	7,011	6,512	6,761	8,207	7,961	7,862
204684_at	6,217	6,533	6,389	6,839	9,872	9,002
204685_s_at	6,882	7,009	6,666	7,006	6,581	6,728
204686_at	9,784	10,013	10,107	11,618	11,651	11,316
204687_at	7,543	7,453	7,659	7,250	7,206	7,538
204688_at	9,993	10,228	9,866	9,381	9,448	8,976
204689_at	5,002	5,137	4,824	5,709	4,967	5,336
204690_at	9,453	10,141	9,602	9,576	9,275	9,701
204691_x_at	8,210	8,204	8,232	8,088	7,961	7,978
204692_at	7,953	7,787	8,089	7,794	7,908	7,946
204693_at	7,099	7,066	7,029	7,661	7,405	7,323
204694_at	5,810	5,900	6,042	6,691	7,015	7,187
204695_at	11,361	10,817	11,005	9,340	8,492	8,476
204696_s_at	8,892	7,974	8,358	7,821	7,378	7,622
204697_s_at	7,071	7,282	7,350	7,530	6,954	7,505
204698_at	7,157	7,118	7,162	6,600	6,523	6,601
204699_s_at	9,394	9,695	9,597	9,438	9,049	8,712
204700_x_at	10,151	10,302	10,578	9,842	9,130	8,695
204701_s_at	7,159	7,403	7,298	8,786	8,098	8,746
204702_s_at	10,585	11,141	10,697	6,065	6,113	6,788
204703_at	8,818	8,964	8,506	8,424	8,774	8,541
204704_s_at	3,487	3,694	3,979	4,048	3,869	3,994
204705_x_at	7,049	6,559	6,692	7,084	7,076	7,003
204706_at	9,909	10,156	10,052	9,685	9,459	9,567
204707_s_at	5,940	6,260	6,314	6,752	6,443	6,899
204708_at	5,961	6,049	5,905	6,102	6,420	6,253
204709_s_at	10,613	10,825	10,097	8,368	9,076	7,585
204710_s_at	10,039	10,143	10,259	10,126	9,448	9,854
204711_at	9,035	8,833	9,089	8,204	8,569	8,099
204712_at	7,339	6,818	8,072	5,326	5,301	5,201
204713_s_at	7,051	7,168	6,979	7,811	7,703	7,850
204714_s_at	6,728	5,735	6,694	6,496	6,593	6,574
204715_at	8,101	8,142	8,255	7,516	7,662	8,207
204716_at	8,207	7,933	8,345	7,393	7,437	7,867
204717_s_at	8,426	8,231	8,266	7,170	7,084	7,050
204718_at	7,672	7,531	7,503	7,867	7,879	8,133

204719_at	4,576	4,421	4,770	5,228	7,696	6,892
204720_s_at	8,706	9,203	8,621	7,943	9,038	8,538
204721_s_at	4,834	4,748	4,787	5,246	4,972	4,896
204722_at	5,561	4,993	5,616	5,601	5,160	5,259
204723_at	6,106	5,725	5,772	6,331	5,929	6,026
204724_s_at	7,419	7,744	7,135	6,247	6,452	6,331
204725_s_at	9,291	9,541	8,781	8,169	8,406	8,125
204726_at	7,114	6,899	7,407	7,509	8,186	6,477
204727_at	9,101	9,392	8,311	5,214	5,456	4,600
204728_s_at	9,799	10,241	9,459	6,594	7,013	5,723
204729_s_at	8,183	8,043	8,130	9,116	8,737	8,995
204730_at	9,714	10,362	9,924	6,608	6,384	6,333
204731_at	8,911	9,088	9,049	8,182	9,745	8,760
204732_s_at	7,249	7,615	7,331	6,165	6,702	6,519
204733_at	6,898	6,976	7,074	6,989	6,813	7,088
204734_at	6,521	6,484	6,560	8,101	7,822	8,100
204735_at	8,032	8,218	8,052	7,894	7,414	7,782
204736_s_at	6,274	7,015	6,462	7,202	7,472	7,238
204737_s_at	7,037	6,686	6,321	5,604	5,942	6,040
204738_s_at	7,328	7,561	7,167	7,088	7,141	7,136
204739_at	8,790	9,185	7,805	5,931	7,276	5,105
204740_at	8,101	8,160	8,073	7,122	6,903	7,407
204741_at	9,153	9,196	9,098	8,945	8,723	8,803
204742_s_at	9,051	8,869	8,450	5,374	6,470	5,512
204743_at	8,557	8,718	8,417	8,729	8,040	8,448
204744_s_at	12,921	13,171	12,705	11,735	12,584	12,085
204745_x_at	10,657	10,557	11,898	12,711	12,249	12,824
204746_s_at	7,725	7,867	7,837	7,562	7,481	7,580
204747_at	6,858	6,611	6,626	7,007	7,318	7,130
204748_at	4,802	5,693	5,104	9,686	10,077	7,722
204749_at	10,427	10,492	10,209	5,787	8,180	7,682
204750_s_at	5,660	5,554	5,639	5,398	5,231	5,283
204751_x_at	7,444	6,875	6,252	5,132	4,553	4,807
204752_x_at	10,115	10,096	9,695	9,658	9,164	8,862
204753_s_at	5,529	5,645	5,325	5,256	5,340	5,413
204754_at	6,028	5,879	6,045	6,246	6,060	5,938
204755_x_at	4,550	4,528	4,171	4,419	4,337	4,432
204756_at	9,015	8,607	8,822	8,307	8,571	8,516
204757_s_at	7,935	7,621	7,982	8,913	8,514	8,847
204758_s_at	5,547	5,568	5,610	5,847	6,045	5,836
204759_at	10,282	9,977	10,208	7,503	8,816	8,193
204760_s_at	6,363	6,517	6,782	6,743	7,663	7,300
204761_at	7,964	8,156	8,399	6,611	7,097	6,251
204762_s_at	6,483	5,943	6,147	7,049	6,574	6,815
204763_s_at	7,855	7,788	8,009	8,204	7,659	7,981
204764_at	7,260	6,645	7,040	7,238	7,264	7,701
204765_at	9,101	9,095	9,282	9,071	9,174	9,182
204766_s_at	10,713	10,348	10,534	9,476	8,412	8,928
204767_s_at	13,081	12,792	13,084	12,170	11,416	11,256
204768_s_at	11,352	10,647	10,931	10,160	8,788	8,908
204769_s_at	6,794	6,625	6,667	7,790	7,537	7,907
204770_at	6,171	6,428	6,181	7,197	6,999	6,905
204771_s_at	10,034	9,876	9,780	7,888	8,259	7,534
204772_s_at	9,205	8,986	8,464	7,018	6,479	6,161
204773_at	7,675	7,671	7,055	8,016	7,853	8,415
204774_at	5,067	5,496	5,065	8,718	7,192	7,149

204775_at	9,142	9,070	8,960	8,776	8,604	8,669
204776_at	8,337	8,380	8,175	7,457	7,067	7,019
204777_s_at	6,574	6,464	6,534	6,506	6,336	6,437
204778_x_at	6,473	6,641	6,613	7,104	6,922	7,516
204779_s_at	6,173	5,978	6,208	6,263	6,174	9,475
204780_s_at	5,609	6,662	6,367	9,132	10,658	10,045
204781_s_at	6,558	5,787	5,799	8,755	9,906	9,018
204782_at	4,473	4,748	4,153	4,582	4,617	4,628
204783_at	7,869	8,882	7,629	5,935	6,247	6,139
204784_s_at	8,181	8,710	7,995	7,632	8,148	7,818
204785_x_at	9,045	9,268	9,138	9,126	7,965	8,590
204786_s_at	8,343	8,862	8,420	8,247	8,192	8,431
204787_at	6,599	6,403	6,613	6,874	6,844	6,896
204788_s_at	9,080	8,964	8,958	8,280	7,920	8,226
204789_at	6,493	6,165	6,128	6,644	6,628	6,806
204790_at	8,312	8,277	9,129	7,189	8,118	8,288
204791_at	9,251	9,868	9,306	7,303	7,878	7,295
204792_s_at	8,281	8,419	8,031	6,731	6,923	6,969
204793_at	7,837	7,856	7,421	5,963	6,277	5,659
204794_at	8,889	9,880	9,710	7,495	6,986	7,314
204795_at	8,974	8,314	8,511	8,091	7,812	8,426
204796_at	8,567	8,432	8,571	8,521	9,076	7,369
204797_s_at	9,257	9,028	9,412	9,610	9,711	8,454
204798_at	8,190	7,897	7,927	6,724	6,631	6,863
204799_at	9,019	8,545	8,922	6,884	6,935	6,993
204800_s_at	7,519	8,009	7,569	7,085	7,042	7,156
204801_s_at	6,788	6,921	6,932	7,014	6,929	7,053
204802_at	5,361	4,987	5,661	6,686	6,222	6,503
204803_s_at	7,134	6,660	6,727	8,320	8,708	8,771
204804_at	7,579	7,804	7,570	7,951	7,512	7,885
204805_s_at	10,128	10,171	9,978	7,428	6,827	7,235
204806_x_at	8,972	9,351	8,927	9,585	9,070	9,866
204807_at	9,388	9,354	9,301	9,173	9,104	9,109
204808_s_at	10,280	10,530	10,190	10,808	10,267	10,604
204809_at	10,172	10,735	9,892	9,254	9,742	8,824
204810_s_at	5,740	5,979	5,370	6,444	5,681	6,153
204811_s_at	9,190	9,318	9,483	7,152	7,219	7,075
204812_at	9,847	9,680	9,495	8,862	8,945	8,276
204813_at	9,022	8,792	8,422	7,976	7,831	7,478
204814_at	4,791	4,813	5,377	3,983	3,936	4,169
204815_s_at	6,895	6,478	6,520	7,525	7,384	7,505
204816_s_at	5,915	6,092	6,237	6,219	6,491	6,199
204817_at	10,525	10,324	10,940	8,723	8,513	8,474
204818_at	5,057	4,909	5,045	5,478	5,133	5,157
204819_at	8,740	8,682	8,760	7,932	7,734	7,814
204820_s_at	5,776	5,481	5,508	9,825	9,783	9,366
204821_at	4,913	5,335	5,283	6,934	7,075	7,139
204822_at	10,515	11,095	10,527	9,062	9,634	7,626
204823_at	7,000	6,835	6,544	10,177	11,368	9,347
204824_at	8,349	8,556	8,646	8,863	8,074	8,620
204825_at	12,317	12,298	12,154	11,124	11,294	10,753
204826_at	10,930	10,916	11,390	9,604	9,148	9,393
204827_s_at	8,467	8,505	9,058	6,892	6,308	6,575
204828_at	8,180	7,637	8,102	6,838	6,369	6,794
204829_s_at	7,487	7,424	7,512	8,228	7,823	8,120
204830_x_at	5,719	5,453	5,483	5,632	5,824	5,964

204831_at	10,614	11,071	10,574	9,616	10,225	9,705
204832_s_at	10,465	10,592	10,424	8,018	8,963	8,017
204833_at	7,561	7,837	7,462	8,612	8,421	8,568
204834_at	4,683	4,804	4,367	4,814	4,736	4,735
204835_at	10,644	10,360	10,649	8,044	8,250	7,481
204836_at	10,613	10,592	10,926	9,358	9,199	8,477
204837_at	9,858	9,886	9,655	7,958	8,904	8,218
204838_s_at	8,744	8,935	8,454	7,606	8,503	7,080
204839_at	9,722	9,522	9,856	10,139	9,178	10,095
204840_s_at	5,066	5,371	4,422	5,192	5,762	5,043
204841_s_at	5,397	5,929	5,310	5,515	6,330	4,944
204842_x_at	9,363	9,633	9,628	8,788	9,191	9,121
204843_s_at	7,155	7,203	7,203	7,909	7,755	7,964
204844_at	4,290	4,536	4,856	4,890	6,432	5,902
204845_s_at	4,224	3,770	4,227	5,568	6,025	6,066
204846_at	6,164	5,590	5,947	6,045	5,401	5,586
204847_at	9,152	9,302	8,716	8,093	9,003	7,728
204848_x_at	7,168	7,156	7,131	7,428	6,988	7,287
204849_at	10,087	10,341	9,942	8,293	8,129	8,642
204850_s_at	9,182	9,079	8,885	6,037	5,929	5,828
204851_s_at	8,792	8,320	7,897	4,852	4,543	4,472
204852_s_at	6,784	6,841	6,744	6,674	6,560	6,736
204853_at	10,264	10,169	9,800	8,694	8,551	8,152
204854_at	8,739	8,642	8,686	8,837	8,708	9,129
204855_at	5,398	4,857	5,691	6,155	5,921	6,251
204856_at	7,701	7,517	7,387	7,705	7,587	7,699
204857_at	8,158	7,965	8,243	8,052	7,667	8,006
204858_s_at	7,781	7,926	7,930	8,330	7,841	8,262
204859_s_at	9,091	9,519	8,588	7,392	8,359	7,463
204860_s_at	5,697	5,327	5,207	5,895	6,238	5,950
204861_s_at	6,201	6,008	5,958	5,497	5,874	5,396
204862_s_at	9,251	9,244	9,259	9,131	8,971	9,614
204863_s_at	6,253	5,582	6,028	8,221	8,698	7,968
204864_s_at	5,840	5,699	5,434	6,124	6,342	6,375
204865_at	3,687	4,052	3,797	4,070	4,203	4,132
204866_at	9,969	9,912	10,251	7,042	7,038	6,892
204867_at	7,627	7,746	7,620	7,136	7,020	7,090
204868_at	8,893	8,929	9,025	9,734	8,586	9,330
204869_at	4,040	4,032	4,014	3,968	4,118	4,252
204870_s_at	6,173	5,879	6,179	5,995	5,615	5,938
204871_at	9,326	9,417	9,041	8,584	9,216	8,525
204872_at	10,944	10,923	10,660	8,104	9,332	8,748
204873_at	9,332	9,419	8,755	6,626	7,892	6,226
204874_x_at	6,808	6,877	6,659	6,616	6,530	6,659
204875_s_at	8,206	8,321	8,483	8,641	7,649	8,306
204876_at	6,797	6,916	6,864	7,004	6,925	7,018
204877_s_at	6,968	7,237	7,060	7,932	7,884	8,328
204878_s_at	7,445	7,298	7,413	7,509	7,035	7,153
204879_at	7,612	8,263	8,249	7,145	6,547	6,757
204880_at	7,189	7,467	6,918	8,206	7,458	8,411
204881_s_at	10,007	10,455	9,766	11,387	11,897	12,229
204882_at	6,768	7,548	7,247	6,897	7,384	7,188
204883_s_at	9,615	9,665	9,867	10,469	9,747	10,315
204884_s_at	4,296	4,248	4,501	3,948	3,846	4,019
204885_s_at	6,224	6,383	6,309	7,061	7,453	7,419
204886_at	8,652	8,853	7,897	6,002	6,596	5,012

204887_s_at	10,212	10,354	9,354	8,375	8,666	7,908
204888_s_at	5,468	5,717	5,537	6,273	6,305	6,382
204889_s_at	7,444	7,351	7,487	7,840	7,710	7,926
204890_s_at	7,316	6,871	6,639	6,376	6,408	6,524
204891_s_at	8,326	8,762	8,482	6,690	7,065	6,772
204892_x_at	14,941	15,073	14,913	14,289	14,597	14,593
204893_s_at	8,595	8,543	8,594	7,858	7,746	7,779
204894_s_at	5,192	5,300	5,137	5,613	4,992	5,133
204895_x_at	6,199	6,300	6,380	6,485	6,451	6,650
204896_s_at	6,593	6,562	6,375	6,552	6,263	6,790
204897_at	4,750	5,894	5,517	8,498	11,686	10,861
204898_at	6,122	6,169	6,326	7,364	7,098	7,354
204899_s_at	7,870	7,483	7,504	8,816	8,425	8,583
204900_x_at	9,979	10,244	9,901	10,607	9,787	9,765
204901_at	7,742	7,908	7,770	8,196	8,206	7,967
204902_s_at	9,538	9,394	9,372	8,146	8,039	8,375
204903_x_at	10,253	10,383	10,519	10,220	9,801	10,422
204904_at	6,658	6,752	6,899	7,018	6,944	6,820
204905_s_at	11,083	11,393	10,790	10,853	10,800	11,044
204906_at	7,826	7,783	8,006	9,191	9,874	9,842
204907_s_at	7,237	7,059	6,943	7,675	7,248	7,324
204908_s_at	7,398	7,630	7,157	8,747	8,714	8,674
204909_at	7,631	7,321	7,270	7,463	6,825	7,277
204910_s_at	5,965	6,177	6,152	7,013	6,698	6,803
204911_s_at	5,326	5,307	5,247	5,485	5,416	5,746
204912_at	6,566	6,580	6,596	6,903	6,813	6,862
204913_s_at	9,287	8,671	8,915	5,143	4,941	5,029
204914_s_at	10,056	9,858	9,933	6,764	6,213	6,400
204915_s_at	10,592	10,262	10,304	5,832	5,513	5,916
204916_at	7,274	7,291	7,552	7,629	8,130	8,506
204917_s_at	7,912	8,141	7,040	7,224	7,528	7,109
204918_s_at	6,928	7,159	6,406	6,157	6,340	5,825
204919_at	6,862	6,817	6,903	7,207	7,101	7,332
204920_at	9,154	9,406	9,056	7,893	7,701	7,822
204921_at	8,762	8,522	8,855	8,688	8,995	8,866
204922_at	8,543	8,293	8,510	6,987	7,073	7,209
204923_at	7,062	7,003	7,124	7,124	7,050	6,926
204924_at	6,399	6,367	6,389	8,388	7,886	6,914
204925_at	7,509	7,210	7,831	8,700	7,212	8,023
204926_at	5,518	5,676	5,759	6,290	6,171	6,421
204927_at	8,570	8,302	8,457	8,509	8,300	8,540
204928_s_at	7,385	7,277	7,668	9,255	8,806	9,470
204929_s_at	6,752	6,835	7,236	9,406	9,166	9,563
204930_s_at	8,397	8,432	8,472	9,297	8,714	9,244
204931_at	5,476	6,005	5,705	5,593	5,457	5,510
204932_at	5,423	5,202	5,771	4,544	5,155	5,595
204933_s_at	5,524	5,821	5,633	6,590	6,574	7,002
204934_s_at	6,076	6,897	5,549	6,125	6,020	6,429
204935_at	5,823	6,031	5,678	5,615	5,137	5,245
204936_at	6,731	6,475	6,636	7,090	7,058	7,257
204937_s_at	9,932	9,967	10,227	9,346	9,667	9,517
204938_s_at	3,766	3,814	3,874	3,934	3,758	3,977
204939_s_at	5,999	6,310	6,253	6,618	6,434	6,337
204940_at	3,600	3,706	3,481	3,691	3,497	3,624
204941_s_at	6,001	5,914	5,626	5,857	5,970	6,146
204942_s_at	4,436	4,177	4,606	4,878	4,845	4,913

204943_at	6,794	7,010	7,107	8,211	8,030	8,265
204944_at	8,515	8,607	8,688	7,784	8,634	8,016
204945_at	7,387	7,435	7,401	7,879	7,590	8,043
204946_s_at	7,807	7,602	8,108	7,731	7,376	7,424
204947_at	8,636	8,256	8,805	8,415	7,811	8,039
204948_s_at	10,830	10,408	10,910	10,342	10,272	9,504
204949_at	7,523	7,390	7,760	9,369	8,473	9,611
204950_at	6,687	6,958	7,061	7,379	7,250	7,539
204951_at	7,778	7,626	7,897	8,062	7,749	8,120
204952_at	7,573	8,062	7,894	7,710	7,721	7,809
204953_at	5,702	5,752	5,861	5,092	5,300	5,127
204954_s_at	6,915	7,079	7,023	7,744	7,771	7,877
204955_at	8,862	9,349	8,980	11,752	11,452	11,560
204956_at	7,888	7,455	7,655	6,720	7,007	6,580
204957_at	9,693	9,547	9,638	10,329	10,001	9,855
204958_at	7,658	7,584	7,758	8,118	7,331	8,004
204959_at	5,325	4,540	4,885	5,894	5,669	6,035
204960_at	8,309	8,414	8,592	9,028	9,051	9,019
204961_s_at	5,602	5,794	6,045	6,553	6,842	6,847
204962_s_at	11,001	11,398	11,139	10,122	9,076	9,010
204963_at	8,266	8,543	7,813	5,977	7,024	6,548
204964_s_at	7,110	7,324	6,710	6,967	6,624	7,106
204965_at	4,817	4,722	4,540	4,941	4,746	4,865
204966_at	6,366	6,463	6,997	6,464	6,401	6,692
204967_at	9,510	9,730	10,299	7,961	9,416	8,334
204968_at	8,306	8,192	8,352	8,422	8,428	8,449
204969_s_at	5,822	6,210	5,409	5,691	5,782	5,622
204970_s_at	9,241	9,351	9,437	9,315	9,185	9,571
204971_at	5,387	6,382	4,849	8,392	9,154	8,746
204972_at	5,485	5,700	5,388	6,512	6,326	6,259
204973_at	7,775	7,974	8,223	8,332	8,207	8,276
204974_at	7,022	6,634	6,994	7,100	6,634	6,757
204975_at	9,563	9,471	9,609	10,247	9,657	9,929
204976_s_at	10,848	10,622	10,764	8,136	9,708	8,849
204977_at	10,037	10,325	9,746	10,251	11,080	9,502
204978_at	9,774	9,418	9,774	9,705	9,592	9,395
204979_s_at	7,480	8,176	7,120	6,900	6,915	6,924
204980_at	8,258	8,320	8,177	7,968	8,819	7,204
204981_at	8,049	8,442	7,600	8,342	8,418	8,892
204982_at	6,982	8,549	6,994	6,774	6,978	6,715
204983_s_at	9,756	9,318	10,029	8,187	8,271	8,954
204984_at	10,016	10,113	10,612	6,556	8,185	8,207
204985_s_at	8,708	9,189	8,749	8,184	7,895	7,967
204986_s_at	7,062	6,802	7,083	7,081	6,746	7,027
204987_at	6,614	6,418	6,589	6,553	6,567	6,594
204988_at	6,386	6,246	6,296	6,742	6,751	6,784
204989_s_at	6,753	6,496	6,160	7,409	7,101	7,228
204990_s_at	7,415	7,124	7,279	7,399	7,665	7,624
204991_s_at	6,844	6,950	6,567	8,172	7,719	8,151
204992_s_at	12,419	12,514	12,274	11,847	12,366	12,328
204993_at	9,153	9,635	9,347	8,137	7,948	8,228
204994_at	9,992	9,883	10,101	7,854	7,601	7,881
204995_at	8,769	8,479	8,901	5,573	4,639	5,103
204996_s_at	7,553	7,004	7,088	7,580	7,590	7,513
204997_at	6,579	6,527	6,505	7,047	6,713	6,970
204998_s_at	7,091	7,662	7,520	8,349	7,737	7,878

204999_s_at	5,278	5,898	5,813	6,717	6,239	6,530
205000_at	11,289	11,802	10,960	4,971	11,933	9,864
205001_s_at	8,482	8,281	7,853	6,855	8,304	7,579
205002_at	7,664	7,762	7,520	7,703	7,353	8,042
205003_at	8,230	8,413	8,127	7,662	9,094	6,821
205004_at	9,444	9,189	9,552	8,602	8,742	8,617
205005_s_at	9,052	9,289	8,754	8,510	9,562	9,702
205006_s_at	8,196	8,449	8,181	8,292	9,189	9,085
205007_s_at	7,951	8,291	8,119	7,380	6,704	7,023
205008_s_at	9,037	9,300	9,131	8,070	7,791	8,511
205009_at	6,774	6,902	7,042	7,541	7,450	7,352
205010_at	8,486	8,004	8,355	7,479	7,267	7,606
205011_at	7,595	7,950	7,790	7,383	7,472	7,438
205012_s_at	8,815	8,916	8,950	9,114	8,483	8,913
205013_s_at	7,329	7,185	7,563	7,151	6,928	7,175
205014_at	6,423	5,924	6,400	6,775	6,763	6,853
205015_s_at	6,315	5,802	5,984	7,081	7,051	6,983
205016_at	5,936	6,100	6,250	4,809	4,894	5,050
205017_s_at	6,699	6,578	6,233	6,832	6,555	6,672
205018_s_at	4,496	5,133	4,904	5,402	5,450	5,746
205019_s_at	7,728	7,384	7,562	7,471	7,207	7,256
205020_s_at	10,771	11,386	10,920	10,440	10,136	10,341
205021_s_at	8,922	8,749	8,752	9,168	8,748	8,998
205022_s_at	9,372	9,322	9,759	7,933	8,475	8,393
205023_at	6,466	6,613	6,931	5,770	5,803	5,731
205024_s_at	10,924	10,755	11,010	9,893	9,084	9,615
205025_at	7,674	7,507	7,518	6,917	6,376	7,037
205026_at	6,385	6,514	6,535	7,202	6,740	6,971
205027_s_at	5,836	7,437	6,181	4,815	6,758	3,936
205028_at	7,702	7,559	7,083	7,146	7,299	7,409
205029_s_at	6,516	5,989	5,252	5,685	6,053	6,030
205030_at	8,779	7,371	6,913	5,523	5,492	5,297
205031_at	8,655	8,707	8,761	6,727	6,708	6,968
205032_at	6,631	7,018	6,663	7,769	7,828	7,771
205033_s_at	6,562	6,821	6,407	7,219	7,264	7,403
205034_at	7,400	7,130	7,163	7,563	7,988	7,644
205035_at	8,336	8,230	8,779	8,234	8,163	8,212
205036_at	10,984	10,966	10,748	10,355	9,528	9,813
205037_at	8,507	8,733	8,594	8,351	8,000	8,370
205038_at	5,747	5,485	5,458	5,306	5,160	5,287
205039_s_at	6,684	6,442	6,763	6,566	6,381	6,684
205040_at	6,571	6,018	5,829	7,350	7,346	7,323
205041_s_at	4,091	4,450	3,701	4,518	4,617	5,019
205042_at	9,542	10,086	9,603	8,674	8,830	8,735
205043_at	7,006	7,327	6,958	6,421	6,639	6,594
205044_at	6,186	7,100	6,366	6,304	6,144	6,432
205045_at	6,906	6,722	6,672	6,713	6,803	6,890
205046_at	9,235	9,690	8,659	7,015	8,511	5,939
205047_s_at	11,441	12,620	11,632	9,145	10,068	10,764
205048_s_at	7,474	7,256	7,360	6,491	6,617	9,231
205049_s_at	6,905	6,978	7,077	7,215	6,773	6,873
205050_s_at	6,488	6,760	6,880	6,534	6,222	6,300
205051_s_at	10,014	10,285	9,636	5,563	5,760	6,024
205052_at	9,961	10,468	9,865	8,078	7,928	7,226
205053_at	12,140	12,510	11,909	9,479	9,071	8,774
205054_at	5,752	6,145	5,518	6,268	6,083	6,194

205055_at	10,673	10,466	10,513	11,273	10,823	11,362
205056_s_at	7,381	7,409	7,309	6,920	6,814	7,067
205057_s_at	6,358	5,976	5,900	7,019	6,697	6,933
205058_at	6,550	6,249	6,007	7,281	7,176	7,241
205059_s_at	6,641	7,000	6,669	6,875	7,206	7,590
205060_at	10,422	10,604	10,119	8,296	9,039	8,252
205061_s_at	10,547	10,656	9,988	10,148	9,647	9,526
205062_x_at	7,220	7,504	7,022	6,175	6,535	5,922
205063_at	8,671	8,789	8,215	6,493	5,600	6,243
205064_at	4,933	5,086	4,988	5,123	5,211	5,383
205065_at	4,794	4,782	5,012	4,945	4,876	5,039
205066_s_at	9,095	9,489	9,275	8,626	8,861	8,934
205067_at	7,578	7,346	7,557	10,475	8,822	9,023
205068_s_at	7,903	8,157	8,222	7,342	7,637	7,622
205069_s_at	5,659	5,383	5,604	6,046	6,008	5,736
205070_at	10,020	10,142	9,849	8,488	9,030	8,294
205071_x_at	8,071	8,515	7,746	7,174	7,082	7,244
205072_s_at	7,563	8,617	7,973	7,766	7,862	7,349
205073_at	6,517	6,651	6,592	6,946	6,531	6,498
205074_at	6,655	6,980	6,767	6,805	6,656	6,710
205075_at	6,097	6,439	6,396	6,360	5,991	6,338
205076_s_at	6,550	7,163	6,799	7,521	7,537	7,465
205077_s_at	10,776	11,174	10,722	10,796	10,723	11,060
205078_at	9,821	10,184	9,639	9,666	9,394	10,070
205079_s_at	9,649	10,013	9,373	7,552	8,122	7,596
205080_at	6,781	7,054	6,722	7,421	7,406	7,570
205081_at	6,946	6,793	6,441	7,766	7,756	7,821
205082_s_at	7,962	8,124	7,371	9,120	9,813	8,570
205083_at	9,343	9,491	8,741	11,232	12,287	10,740
205084_at	6,512	6,738	6,461	8,240	8,503	8,678
205085_at	9,782	9,823	10,201	7,514	6,906	7,181
205086_s_at	7,971	7,915	7,900	8,178	7,802	8,060
205087_at	8,765	9,341	8,560	8,223	7,815	7,984
205088_at	7,053	7,246	7,200	6,894	8,240	7,441
205089_at	9,077	9,389	9,206	7,983	8,153	8,012
205090_s_at	8,212	7,996	8,740	8,795	8,432	8,699
205091_x_at	10,201	10,407	9,667	9,326	10,489	8,836
205092_x_at	4,270	3,960	4,321	5,163	6,636	5,431
205093_at	7,454	6,980	7,035	6,865	6,813	7,303
205094_at	8,317	8,480	8,342	8,294	8,122	8,380
205095_s_at	7,734	7,917	7,998	8,084	8,110	8,149
205096_at	7,226	6,895	6,901	7,279	7,122	7,281
205097_at	8,458	8,112	8,236	8,042	9,014	8,517
205098_at	6,077	6,089	6,115	6,540	6,369	6,330
205099_s_at	5,960	6,271	6,206	6,404	6,164	6,342
205100_at	10,081	10,562	10,598	11,403	11,186	10,891
205101_at	6,225	5,753	6,393	6,729	6,515	6,712
205102_at	7,545	7,638	7,547	7,406	7,317	7,395
205103_at	8,402	8,899	8,198	7,564	7,679	7,516
205104_at	7,266	7,189	7,553	7,539	7,473	7,846
205105_at	10,435	10,682	10,231	8,147	10,551	8,456
205106_at	5,904	6,233	6,044	6,472	6,322	6,589
205107_s_at	9,349	8,999	9,164	7,349	7,282	7,486
205108_s_at	4,813	5,281	5,378	5,420	5,539	5,694
205109_s_at	8,359	8,555	8,561	7,608	7,628	8,064
205110_s_at	8,629	9,102	9,223	6,155	6,216	6,148

205111_s_at	5,498	5,943	5,460	6,053	6,665	6,361
205112_at	5,085	5,866	5,125	5,987	6,685	6,340
205113_at	9,305	8,998	8,854	7,023	6,290	6,923
205114_s_at	5,737	6,560	6,353	6,349	6,584	6,577
205115_s_at	8,115	7,636	8,784	7,343	7,799	7,901
205116_at	6,676	6,670	6,147	6,963	7,061	6,891
205117_at	7,643	7,446	7,567	7,085	8,210	7,762
205118_at	5,229	5,132	5,372	5,573	5,572	5,651
205119_s_at	6,898	7,018	7,062	9,002	7,523	8,306
205120_s_at	9,609	9,643	9,684	11,322	11,579	11,340
205121_at	5,409	4,930	4,880	5,552	5,184	5,684
205122_at	11,711	11,400	10,939	7,506	7,676	7,397
205123_s_at	9,585	9,921	8,720	4,611	4,747	4,390
205124_at	7,103	7,074	6,590	7,578	7,156	7,337
205125_at	9,253	9,260	8,946	9,474	9,396	9,832
205126_at	8,281	8,505	8,274	8,838	8,786	8,642
205127_at	6,829	7,127	6,540	7,365	7,299	7,514
205128_x_at	7,816	8,217	7,733	9,558	11,441	11,275
205129_at	8,098	8,140	8,419	9,284	8,473	9,173
205130_at	6,628	6,798	6,608	9,971	9,675	10,633
205131_x_at	7,514	6,558	6,829	7,552	7,727	8,645
205132_at	7,753	7,679	7,902	7,441	6,888	6,900
205133_s_at	12,626	12,653	12,412	12,101	11,347	11,532
205134_s_at	8,676	8,939	8,549	7,467	7,404	7,279
205135_s_at	9,317	9,505	9,345	7,867	8,117	7,940
205136_s_at	9,086	9,579	9,462	6,886	6,727	6,916
205137_x_at	7,281	7,110	7,035	7,674	7,280	7,612
205138_s_at	6,797	7,043	6,743	7,738	7,009	7,582
205139_s_at	8,239	8,379	8,317	7,339	6,996	7,474
205140_at	8,736	8,955	8,331	6,837	7,369	6,844
205141_at	6,316	6,782	6,209	7,462	6,826	7,852
205142_x_at	7,665	6,901	7,665	7,961	7,434	7,824
205143_at	7,879	7,667	7,460	6,624	6,830	6,809
205144_at	7,608	7,571	7,492	8,282	7,993	8,055
205145_s_at	6,958	6,734	7,046	8,135	7,494	7,816
205146_x_at	7,267	7,248	7,260	7,267	7,146	7,347
205147_x_at	5,178	5,031	5,533	6,668	6,376	6,557
205148_s_at	7,613	7,995	7,980	7,224	7,006	7,092
205149_s_at	6,222	6,002	6,243	6,476	5,962	6,242
205150_s_at	8,729	8,792	9,063	6,031	5,835	5,910
205151_s_at	8,254	8,282	8,806	6,018	6,079	6,071
205152_at	4,414	5,345	5,391	4,613	4,682	4,843
205153_s_at	7,247	7,301	7,116	7,268	7,282	7,512
205154_at	6,993	6,440	7,240	7,006	6,750	7,090
205155_s_at	8,036	7,626	8,089	8,064	8,050	7,989
205156_s_at	7,508	7,408	7,458	8,042	7,781	7,894
205157_s_at	6,781	6,268	6,710	7,161	7,077	7,046
205158_at	5,086	5,477	5,036	6,163	6,411	7,380
205159_at	4,424	4,420	4,623	5,140	5,140	4,876
205160_at	8,260	8,049	8,014	7,740	8,173	8,027
205161_s_at	6,250	6,397	6,347	6,658	6,273	6,883
205162_at	8,821	8,913	8,713	7,863	8,000	8,367
205163_at	7,631	7,276	7,669	7,718	7,523	7,837
205164_at	8,383	8,708	8,740	9,389	8,464	9,308
205165_at	8,480	7,951	8,130	6,547	6,321	6,789
205166_at	6,609	6,487	6,420	6,815	6,474	6,914

205167_s_at	8,509	8,932	8,489	7,387	6,991	7,050
205168_at	7,327	7,087	7,531	8,730	8,837	8,332
205169_at	8,026	8,134	7,980	6,605	6,594	6,735
205170_at	6,098	5,692	5,660	6,211	6,323	6,509
205171_at	9,531	9,644	9,222	7,241	7,869	7,155
205172_x_at	8,293	8,202	8,345	9,513	9,082	9,568
205173_x_at	8,266	8,510	8,073	9,522	9,362	9,631
205174_s_at	4,794	5,542	5,112	8,189	6,592	6,238
205175_s_at	6,647	6,598	6,761	7,111	6,739	6,867
205176_s_at	11,034	11,621	10,675	9,921	9,502	9,503
205177_at	6,343	5,923	5,836	6,569	6,250	6,482
205178_s_at	9,976	10,291	9,747	8,005	9,339	7,688
205179_s_at	6,571	6,379	6,719	7,942	7,841	7,918
205180_s_at	6,963	7,236	6,882	7,241	7,149	7,382
205181_at	8,207	8,343	8,088	6,794	6,336	7,177
205182_s_at	8,043	7,597	7,993	7,726	7,472	7,805
205183_at	6,086	6,302	5,732	6,557	6,716	6,741
205184_at	8,859	8,981	9,018	6,559	6,453	6,649
205185_at	7,145	7,110	7,083	6,035	5,960	6,209
205186_at	6,153	6,102	6,146	7,374	6,771	7,209
205187_at	6,651	6,664	6,059	6,167	5,660	6,299
205188_s_at	7,790	7,676	7,437	7,242	7,038	7,232
205189_s_at	8,132	7,785	8,105	7,957	7,757	8,147
205190_at	10,379	11,217	10,098	6,023	6,408	5,669
205191_at	7,816	8,166	7,495	7,271	7,874	7,241
205192_at	6,200	6,662	6,565	6,952	6,916	7,009
205193_at	7,213	7,652	7,646	8,956	8,824	9,401
205194_at	10,071	10,256	10,509	8,629	8,600	9,567
205195_at	7,094	6,831	6,725	6,992	7,149	7,332
205196_s_at	8,038	7,752	7,408	8,307	8,404	8,547
205197_s_at	7,945	7,510	7,210	8,370	8,050	8,490
205198_s_at	6,629	6,933	6,452	7,096	7,600	7,313
205199_at	8,181	7,738	7,889	8,862	8,595	8,761
205200_at	6,915	6,472	7,115	7,419	8,575	7,893
205201_at	9,439	9,151	9,462	7,858	8,143	8,191
205202_at	11,226	11,367	11,179	11,347	11,381	11,484
205203_at	6,955	7,360	6,846	7,696	7,592	7,401
205204_at	8,901	9,339	9,371	9,229	8,618	9,274
205205_at	6,542	6,705	6,834	7,595	7,089	7,510
205206_at	11,732	11,774	11,700	6,059	5,504	5,677
205207_at	6,571	6,690	6,821	9,836	10,249	8,605
205208_at	7,939	8,181	8,176	8,194	8,332	8,645
205209_at	7,850	7,571	7,939	7,765	7,666	7,845
205210_at	9,132	8,916	9,311	8,650	8,295	8,641
205211_s_at	7,637	7,509	7,489	8,700	8,567	8,677
205212_s_at	6,528	6,829	6,677	6,659	6,480	6,488
205213_at	6,827	6,891	6,800	6,984	6,425	6,679
205214_at	7,091	7,462	6,521	7,735	8,096	8,440
205215_at	8,962	9,060	9,009	7,220	7,400	7,166
205216_s_at	5,543	5,809	5,370	6,199	6,355	6,257
205217_at	10,622	10,575	10,432	9,833	8,920	9,221
205218_at	7,556	7,776	7,438	7,320	7,198	7,033
205219_s_at	7,266	7,471	7,023	7,674	7,533	7,419
205220_at	5,389	5,527	5,420	5,275	5,382	5,503
205221_at	6,142	6,084	6,466	6,748	6,616	6,669
205222_at	8,275	8,494	7,459	6,661	6,920	6,560

205223_at	8,236	8,326	8,106	7,844	7,913	7,705
205224_at	8,203	7,806	8,368	8,436	7,910	7,919
205225_at	5,236	5,787	5,129	5,621	5,759	5,746
205226_at	8,767	9,467	9,104	7,306	7,643	7,787
205227_at	8,080	8,298	7,386	7,665	8,667	7,869
205228_at	7,594	7,464	7,605	8,277	8,030	8,388
205229_s_at	9,618	10,100	9,595	6,679	6,458	6,238
205230_at	7,648	7,640	8,069	7,970	7,636	7,874
205231_s_at	7,814	7,644	7,467	7,159	6,985	6,971
205232_s_at	6,970	6,604	6,578	6,776	6,452	6,697
205233_s_at	8,295	8,423	8,739	7,374	6,956	7,147
205234_at	4,957	4,862	4,631	7,918	9,635	9,133
205235_s_at	9,317	9,457	8,718	6,940	7,796	6,279
205236_x_at	7,898	7,571	7,650	8,546	7,781	8,059
205237_at	5,805	5,807	5,849	6,362	6,485	6,318
205238_at	8,342	8,212	8,359	8,070	7,907	7,673
205239_at	5,524	5,327	5,981	11,439	11,357	10,761
205240_at	8,835	9,466	8,254	7,741	8,692	7,689
205241_at	8,774	9,003	9,125	10,815	10,048	11,017
205242_at	4,113	4,181	4,297	4,550	4,250	4,352
205243_at	7,961	7,912	8,516	7,423	7,237	7,338
205244_s_at	8,998	8,373	8,961	7,713	7,471	7,583
205245_at	8,130	7,974	8,054	8,080	8,028	8,146
205246_at	8,248	8,345	8,053	8,144	7,717	8,047
205247_at	6,897	6,909	7,231	7,500	7,606	7,759
205248_at	5,729	5,863	6,193	6,203	6,227	6,561
205249_at	7,781	9,945	9,443	7,648	7,106	8,214
205250_s_at	8,898	9,283	8,206	7,336	9,259	7,250
205251_at	9,556	9,246	9,733	6,500	7,213	6,413
205252_at	8,045	7,899	8,113	7,376	7,105	7,169
205253_at	9,321	9,511	9,701	7,654	7,232	7,723
205254_x_at	6,682	6,416	6,523	7,541	7,581	7,866
205255_x_at	9,198	8,918	9,188	8,334	9,180	8,888
205256_at	8,421	8,159	8,900	7,089	7,022	7,175
205257_s_at	8,655	8,769	8,791	7,276	7,110	7,249
205258_at	6,713	6,284	6,398	6,772	6,665	6,681
205259_at	5,656	6,344	5,919	6,074	6,718	6,085
205260_s_at	9,266	9,737	8,778	9,458	8,804	8,614
205261_at	7,279	7,347	7,346	7,618	7,410	7,575
205262_at	6,798	6,771	6,550	6,132	6,657	6,593
205263_at	11,213	11,352	11,397	11,185	11,510	11,396
205264_at	8,821	8,786	9,275	8,781	7,910	8,237
205265_s_at	8,824	8,879	8,921	8,492	9,004	8,998
205266_at	6,194	6,155	6,267	9,855	9,087	9,265
205267_at	5,511	5,422	5,380	5,639	5,579	5,289
205268_s_at	9,838	10,000	10,290	7,028	7,284	6,966
205269_at	3,848	3,897	4,234	4,234	3,948	4,081
205270_s_at	6,692	6,829	6,664	7,031	6,616	6,883
205271_s_at	7,616	7,547	7,530	7,749	7,524	7,987
205272_s_at	7,168	7,110	7,464	8,109	8,147	8,333
205273_s_at	8,849	8,683	8,714	10,376	10,733	10,464
205274_at	7,170	6,938	7,076	7,569	7,378	7,303
205275_at	6,423	6,231	6,576	7,359	7,181	7,362
205276_s_at	7,785	7,347	7,939	8,325	8,298	8,340
205277_at	6,366	6,850	7,067	7,349	7,026	7,044
205278_at	7,880	8,463	8,387	4,388	4,436	4,644

205279_s_at	5,758	6,560	5,400	6,979	7,307	6,993
205280_at	5,563	6,472	4,960	6,902	8,207	7,784
205281_s_at	8,850	9,213	8,468	7,026	7,937	6,925
205282_at	9,974	10,546	10,030	8,908	8,590	8,582
205283_at	8,398	8,450	8,246	7,974	8,791	7,028
205284_at	11,011	10,499	11,316	8,871	8,728	8,745
205285_s_at	6,198	5,985	5,846	6,975	7,016	6,881
205286_at	9,642	9,928	10,077	6,454	6,263	6,388
205287_s_at	6,180	5,873	6,225	5,760	5,433	6,017
205288_at	6,067	6,636	5,719	4,881	5,008	4,705
205289_at	6,005	6,008	5,887	6,621	5,693	5,703
205290_s_at	6,813	7,256	6,808	7,119	5,995	6,412
205291_at	8,420	8,398	8,631	8,448	8,591	8,641
205292_s_at	13,870	13,609	13,369	12,312	12,518	11,774
205293_x_at	7,546	7,437	7,812	8,232	8,039	8,268
205294_at	7,974	7,884	7,616	8,406	8,176	8,468
205295_at	5,699	6,205	5,836	5,870	6,142	6,027
205296_at	8,028	7,421	7,342	6,843	7,295	5,904
205297_s_at	7,460	7,126	7,164	7,990	7,855	8,173
205298_s_at	8,875	9,228	9,125	8,663	8,774	8,976
205299_s_at	6,539	6,391	6,768	5,857	5,869	5,929
205300_s_at	9,406	9,303	9,199	9,421	8,980	9,166
205301_s_at	7,470	7,188	7,557	7,902	7,264	7,652
205302_at	4,340	4,625	4,670	5,160	10,993	6,968
205303_at	9,324	9,528	9,515	7,241	6,543	7,118
205304_s_at	8,473	9,062	8,730	7,000	6,566	7,186
205305_at	7,126	7,148	7,300	7,887	7,805	7,860
205306_x_at	5,158	5,552	5,271	5,405	5,579	5,279
205307_s_at	5,089	5,117	5,373	5,760	5,636	5,424
205308_at	8,237	8,673	8,001	8,334	8,877	8,703
205309_at	7,307	7,293	8,004	5,842	5,568	5,657
205310_at	9,217	9,319	9,592	7,435	7,499	7,278
205311_at	7,711	7,084	7,248	7,094	6,858	6,686
205312_at	6,293	5,948	6,219	6,510	6,567	6,862
205313_at	8,550	8,436	8,264	9,172	8,981	9,129
205314_x_at	6,593	6,693	6,357	5,851	5,947	5,989
205315_s_at	9,257	9,211	8,992	8,919	9,645	9,309
205316_at	6,544	6,729	7,013	5,771	5,808	5,955
205317_s_at	6,116	5,968	6,212	6,244	6,117	5,944
205318_at	6,553	6,682	6,859	6,498	6,741	6,567
205319_at	6,498	6,121	6,582	7,484	7,808	7,830
205320_at	6,978	6,724	7,064	7,401	7,398	7,492
205321_at	9,840	9,355	9,250	7,662	7,549	7,582
205322_s_at	8,807	8,617	8,502	8,637	8,705	8,607
205323_s_at	8,813	9,093	8,978	8,530	8,569	8,366
205324_s_at	9,975	9,831	10,206	10,367	9,973	10,494
205325_at	6,597	6,739	6,730	7,091	7,258	6,852
205326_at	6,998	6,716	7,104	7,146	6,860	7,103
205327_s_at	9,481	9,495	9,492	9,565	9,342	9,231
205328_at	8,986	9,362	8,878	6,324	6,223	6,404
205329_s_at	9,125	9,645	8,838	8,631	8,689	8,612
205330_at	8,183	8,062	8,313	5,116	6,155	5,467
205331_s_at	8,195	8,147	8,286	8,203	8,341	8,413
205332_at	8,151	7,545	7,902	8,304	8,484	8,700
205333_s_at	8,176	7,563	8,471	7,228	7,120	7,437
205334_at	6,556	7,302	7,144	7,869	7,822	7,654

205335_s_at	11,029	11,036	10,951	11,457	11,467	11,456
205336_at	6,027	6,585	6,163	6,441	6,219	6,298
205337_at	4,860	4,788	4,339	4,502	4,376	4,509
205338_s_at	5,895	6,170	5,777	6,094	6,004	6,186
205339_at	10,888	11,268	10,950	9,384	9,914	8,655
205340_at	9,756	9,652	9,664	8,419	8,263	7,985
205341_at	7,741	7,814	7,745	8,387	8,213	8,420
205342_s_at	6,281	7,095	5,636	5,313	5,147	5,300
205343_at	5,306	5,324	5,363	5,267	5,087	5,351
205344_at	7,899	7,858	7,586	7,823	7,475	7,541
205345_at	10,928	11,101	10,496	8,106	8,885	8,188
205346_at	7,796	8,122	8,059	7,150	6,717	7,011
205347_s_at	13,903	13,953	13,732	6,485	6,639	6,591
205348_s_at	8,278	8,640	7,916	8,355	8,569	8,895
205349_at	6,042	5,884	6,191	6,252	6,166	6,281
205350_at	9,678	9,924	9,569	6,817	6,902	6,969
205351_at	6,967	6,720	7,343	8,186	7,752	8,613
205352_at	7,837	8,682	7,972	7,187	9,080	7,814
205353_s_at	11,177	11,544	11,614	10,314	9,443	10,311
205354_at	7,947	7,572	7,418	7,966	7,578	7,849
205355_at	6,576	7,414	6,628	7,354	7,640	7,117
205356_at	10,564	10,489	10,471	8,994	9,227	8,926
205357_s_at	5,878	5,641	5,637	8,275	10,501	10,622
205358_at	4,924	4,748	4,959	5,292	5,123	5,228
205359_at	5,711	6,000	5,936	6,721	6,903	6,201
205360_at	7,417	7,457	7,145	7,820	7,644	7,735
205361_s_at	12,025	12,276	11,803	11,283	10,439	11,010
205362_s_at	9,447	9,831	9,408	9,304	9,022	9,378
205363_at	4,685	4,500	5,148	5,398	5,322	5,700
205364_at	8,200	8,510	8,306	7,925	7,761	7,681
205365_at	5,397	5,371	5,475	5,896	5,534	5,648
205366_s_at	6,281	6,089	6,350	6,835	6,715	7,549
205367_at	9,002	8,985	9,100	8,544	8,792	8,544
205368_at	9,080	8,820	8,745	6,100	6,327	6,572
205369_x_at	7,492	8,038	7,410	6,727	6,675	6,612
205370_x_at	11,587	11,518	11,333	9,730	11,126	10,087
205371_s_at	7,033	7,031	6,280	5,069	5,471	5,148
205372_at	9,141	9,589	8,768	6,344	7,369	6,182
205373_at	8,577	9,060	7,995	5,603	5,635	5,628
205374_at	6,536	6,622	6,563	6,902	6,749	6,905
205375_at	7,064	7,280	7,343	7,517	7,314	7,515
205376_at	4,060	4,812	4,546	8,864	7,776	7,915
205377_s_at	6,937	6,824	7,179	7,592	7,519	7,150
205378_s_at	5,636	4,887	5,462	6,201	6,120	6,290
205379_at	7,813	8,225	8,470	9,593	8,543	8,800
205380_at	4,953	5,970	4,677	4,486	4,829	4,689
205381_at	6,361	6,380	6,252	6,699	8,884	9,522
205382_s_at	7,181	7,568	7,078	6,597	7,238	7,228
205383_s_at	7,574	7,265	7,431	8,042	9,232	8,041
205384_at	7,142	7,048	7,019	7,859	7,636	7,840
205385_at	5,682	5,477	5,806	6,448	6,391	6,246
205386_s_at	5,147	4,704	5,249	4,575	4,932	4,784
205387_s_at	7,753	7,588	7,737	8,553	8,350	8,498
205388_at	6,106	6,597	6,326	6,482	6,194	6,634
205389_s_at	5,686	6,253	5,696	5,615	5,826	5,808
205390_s_at	6,163	6,434	6,439	6,302	6,576	6,580

205391_x_at	7,051	6,758	6,859	7,763	7,784	7,689
205392_s_at	7,133	6,955	7,591	7,431	7,298	7,266
205393_s_at	10,104	10,218	10,044	8,429	8,453	8,140
205394_at	10,276	10,124	9,958	8,929	9,169	8,520
205395_s_at	8,840	9,025	8,342	7,574	8,041	6,408
205396_at	6,805	6,647	6,662	7,407	7,415	7,113
205397_x_at	6,450	6,625	6,498	7,267	7,056	7,360
205398_s_at	7,447	7,728	7,921	9,144	9,137	9,008
205399_at	12,590	12,843	12,718	5,601	6,944	6,520
205400_at	6,593	6,573	6,798	6,735	6,754	6,866
205401_at	9,511	9,555	8,952	8,693	8,585	8,220
205402_x_at	7,635	6,914	7,144	9,379	9,117	9,111
205403_at	5,988	6,204	6,148	7,200	7,005	7,213
205404_at	6,032	6,065	6,227	6,967	6,642	7,064
205405_at	7,886	7,712	7,820	7,956	8,047	7,875
205406_s_at	9,384	9,629	9,169	9,380	8,595	8,964
205407_at	8,706	8,833	8,386	6,590	9,316	8,830
205408_at	6,521	6,769	6,579	5,681	6,359	5,887
205409_at	6,347	5,765	6,171	7,694	7,405	7,882
205410_s_at	5,441	5,619	5,636	5,777	5,767	5,908
205411_at	6,458	6,407	6,412	6,790	6,666	6,534
205412_at	10,511	11,083	10,356	10,573	10,529	10,284
205413_at	7,284	7,129	7,622	3,964	4,040	4,060
205414_s_at	8,081	8,708	8,156	5,929	7,442	6,632
205415_s_at	8,868	9,379	8,238	8,086	8,023	7,254
205416_s_at	8,288	8,946	7,934	7,029	6,960	7,014
205417_s_at	11,548	11,314	11,817	10,214	10,808	10,736
205418_at	6,955	6,875	6,825	7,207	6,879	7,067
205419_at	5,236	5,354	5,480	5,535	6,262	5,433
205420_at	8,082	8,758	7,864	8,657	8,419	9,179
205421_at	6,336	5,304	6,351	4,186	4,284	4,114
205422_s_at	4,214	4,734	4,297	8,588	10,043	9,420
205423_at	8,923	8,295	8,732	9,053	9,030	9,376
205424_at	7,019	6,827	7,040	7,310	7,377	7,345
205425_at	7,130	6,841	7,350	6,460	6,022	6,389
205426_s_at	6,257	6,057	6,361	6,239	5,658	5,933
205427_at	8,315	8,863	7,883	6,838	6,855	6,320
205428_s_at	7,396	7,591	7,350	7,922	7,664	8,011
205429_s_at	10,167	10,083	9,972	7,809	8,307	7,560
205430_at	3,544	4,020	3,656	4,089	4,245	4,053
205431_s_at	6,310	6,453	6,490	6,536	6,600	6,686
205432_at	7,329	7,477	6,833	7,433	7,104	7,379
205433_at	5,992	6,550	4,743	4,090	4,119	4,173
205434_s_at	8,761	8,454	8,459	8,318	8,779	8,646
205435_s_at	6,351	6,145	6,255	6,536	6,535	6,656
205436_s_at	9,729	9,722	10,127	9,769	8,604	9,265
205437_at	8,542	8,723	8,682	7,712	7,831	7,285
205438_at	6,368	6,037	6,458	6,721	6,131	6,638
205439_at	6,585	6,660	6,579	8,926	6,829	10,460
205440_s_at	4,948	6,192	4,802	4,337	4,340	4,315
205441_at	8,194	8,729	8,370	8,184	8,125	8,166
205442_at	8,020	8,290	7,598	6,247	6,716	6,147
205443_at	8,737	9,252	8,249	9,904	9,567	9,916
205444_at	6,191	6,274	6,515	6,477	6,211	6,352
205445_at	5,682	5,926	6,227	6,745	6,416	6,239
205446_s_at	8,633	8,921	8,093	7,534	7,720	7,496

205447_s_at	7,355	7,387	7,684	8,207	7,823	8,183
205448_s_at	7,558	7,736	8,083	7,238	7,392	7,214
205449_at	10,006	9,821	9,951	10,109	9,091	9,652
205450_at	8,827	9,329	9,057	8,154	8,430	8,398
205451_at	7,806	7,454	7,718	6,620	6,750	6,680
205452_at	8,651	8,817	8,246	9,329	9,550	9,350
205453_at	7,764	8,021	7,933	7,527	7,307	10,636
205454_at	7,052	7,223	6,953	7,488	7,135	7,507
205455_at	7,125	7,174	6,981	7,245	7,072	7,320
205456_at	7,233	7,034	7,052	7,489	7,268	7,539
205457_at	8,822	9,286	8,669	8,725	8,569	9,039
205458_at	7,240	7,162	7,418	7,471	7,194	7,406
205459_s_at	7,068	7,383	7,385	8,243	7,928	7,898
205460_at	6,605	6,131	5,924	7,057	7,334	7,241
205461_at	5,966	5,739	5,713	5,879	5,789	5,929
205462_s_at	6,641	6,665	6,641	8,387	8,198	8,840
205463_s_at	9,429	9,585	9,896	8,103	7,472	7,666
205464_at	6,248	6,207	6,466	6,895	6,741	6,759
205465_x_at	7,034	7,072	7,471	7,698	7,450	7,916
205466_s_at	4,452	4,919	4,884	4,232	4,119	4,293
205467_at	6,961	7,040	7,157	7,276	7,189	7,194
205468_s_at	7,023	6,692	7,128	7,284	7,183	7,504
205469_s_at	7,834	7,923	7,829	8,719	8,218	8,663
205470_s_at	6,991	6,784	6,858	6,764	6,731	6,808
205471_s_at	4,588	4,437	4,955	5,201	4,891	4,937
205472_s_at	4,647	4,611	5,043	4,667	4,642	4,781
205473_at	6,257	6,487	6,327	6,380	6,369	6,264
205474_at	9,847	10,361	9,742	9,264	9,113	9,151
205475_at	6,112	6,384	6,486	7,368	7,206	7,406
205476_at	5,144	5,183	4,468	11,193	7,866	8,056
205477_s_at	6,449	6,695	6,728	6,248	6,107	6,460
205478_at	7,819	7,628	7,159	7,293	7,183	7,297
205479_s_at	8,489	9,189	8,780	9,847	9,080	9,007
205480_s_at	12,744	13,239	12,865	11,355	12,562	12,151
205481_at	6,951	7,232	6,850	7,553	7,290	7,484
205482_x_at	8,303	8,493	8,876	8,745	8,456	8,795
205483_s_at	6,936	7,648	7,324	8,863	8,684	9,092
205484_at	7,132	7,090	6,559	7,980	7,381	7,777
205485_at	6,039	5,819	6,045	6,051	5,811	6,093
205486_at	8,911	9,853	9,021	8,008	7,916	8,016
205487_s_at	6,829	6,586	6,534	6,710	6,395	6,733
205488_at	4,938	4,991	5,033	5,275	5,303	5,714
205489_at	9,500	9,855	9,910	8,702	8,762	8,702
205490_x_at	7,075	7,252	7,431	7,782	7,996	7,670
205491_s_at	6,340	6,065	6,316	7,092	6,879	7,073
205492_s_at	6,707	6,810	6,359	6,907	6,551	6,991
205493_s_at	8,111	7,832	8,206	6,980	6,959	7,255
205494_at	7,389	7,463	7,616	7,534	7,319	7,489
205495_s_at	6,019	5,835	6,055	6,819	6,846	6,800
205496_at	4,366	4,566	4,195	4,206	4,201	4,161
205497_at	9,278	9,302	8,868	8,133	8,482	8,496
205498_at	4,505	4,849	4,480	6,328	8,154	7,205
205499_at	6,149	6,164	6,035	9,363	10,116	10,054
205500_at	7,968	8,540	6,800	6,491	6,537	6,206
205501_at	6,407	6,099	5,718	4,411	4,715	4,861
205502_at	6,019	6,186	6,258	6,763	6,632	6,484

205503_at	7,123	7,557	7,296	7,203	7,297	7,081
205504_at	6,679	6,441	6,818	6,536	6,461	6,411
205505_at	8,072	8,949	8,543	8,453	9,102	8,819
205506_at	6,951	6,821	6,850	7,208	7,015	7,225
205507_at	7,293	7,233	7,165	7,928	7,614	7,883
205508_at	7,451	7,410	7,548	7,555	7,012	7,204
205509_at	5,593	5,302	5,630	6,087	5,850	6,170
205510_s_at	7,543	7,934	7,863	7,216	6,403	6,637
205511_at	7,418	7,524	7,686	5,853	5,981	5,860
205512_s_at	10,677	10,309	10,237	9,965	9,770	9,639
205513_at	5,945	6,019	5,827	6,200	6,077	6,291
205514_at	9,292	9,699	9,205	4,765	7,016	5,700
205515_at	5,463	5,757	5,453	5,453	5,589	5,878
205516_x_at	7,668	7,409	7,969	8,049	7,901	8,157
205517_at	7,373	6,785	7,182	7,978	7,403	8,096
205518_s_at	4,797	5,046	5,146	5,236	5,045	5,125
205519_at	7,316	7,303	6,811	6,957	6,600	6,725
205520_at	5,567	5,728	5,553	6,055	5,863	6,053
205521_at	9,180	8,692	9,213	8,908	8,934	8,816
205522_at	6,620	6,710	6,857	7,062	6,717	6,831
205523_at	4,905	4,625	4,382	4,767	4,533	4,349
205524_s_at	7,924	7,301	7,709	7,537	7,801	7,801
205525_at	7,563	7,245	7,531	5,979	8,351	6,191
205526_s_at	9,860	10,325	9,804	8,779	8,707	8,419
205527_s_at	9,388	9,210	9,840	9,730	8,847	9,097
205528_s_at	5,694	6,190	5,828	4,452	4,754	5,840
205529_s_at	7,035	7,239	6,988	4,877	5,108	5,478
205530_at	7,236	7,347	6,832	7,587	8,462	7,535
205531_s_at	7,553	8,390	8,014	7,564	7,645	7,857
205532_s_at	7,354	6,936	7,030	5,376	5,233	5,334
205533_s_at	4,646	4,592	4,761	4,452	4,191	4,172
205534_at	6,220	5,956	6,311	5,297	6,392	5,785
205535_s_at	5,626	5,820	5,739	5,765	6,105	5,803
205536_at	7,121	6,972	7,060	7,465	7,243	7,340
205537_s_at	7,768	7,192	7,504	8,247	8,475	8,578
205538_at	6,895	7,348	7,418	6,186	6,158	6,427
205539_at	5,395	5,296	5,169	5,370	5,331	5,424
205540_s_at	6,974	7,065	6,865	5,758	5,461	5,682
205541_s_at	11,821	11,829	11,830	8,803	8,994	8,973
205542_at	9,110	9,640	9,039	13,073	12,522	12,319
205543_at	6,208	6,605	6,166	5,493	6,394	5,777
205544_s_at	6,624	6,541	6,270	4,869	4,754	4,833
205545_x_at	12,192	12,468	12,451	11,031	11,141	11,077
205546_s_at	8,379	8,542	8,560	9,622	9,539	9,927
205547_s_at	8,069	7,772	9,214	7,581	10,043	12,088
205548_s_at	10,702	11,163	10,314	10,103	9,638	9,683
205549_at	6,654	6,699	6,926	7,631	7,800	7,810
205550_s_at	10,056	10,260	10,438	10,274	9,677	9,868
205551_at	6,479	6,458	6,793	6,316	5,795	6,058
205552_s_at	5,667	5,142	5,906	5,698	5,557	5,624
205553_s_at	5,239	5,202	5,423	5,873	5,724	5,991
205554_s_at	5,998	6,500	6,402	6,851	6,696	6,971
205555_s_at	6,401	6,692	6,696	6,270	6,419	6,423
205556_at	4,478	4,609	4,520	5,038	4,825	4,923
205557_at	6,065	6,122	6,400	7,179	6,842	7,103
205558_at	8,532	8,489	8,579	7,590	7,523	7,582

205559_s_at	8,430	8,700	8,040	7,270	8,482	8,114
205560_at	7,031	7,424	7,104	6,836	7,164	7,286
205561_at	7,783	7,810	7,771	8,467	8,386	8,384
205562_at	8,710	8,868	8,873	8,876	8,216	8,559
205563_at	5,524	5,444	5,375	6,478	6,187	6,473
205564_at	6,016	6,081	6,135	7,056	7,411	6,997
205565_s_at	8,927	8,723	9,242	9,266	8,588	9,036
205566_at	7,558	7,330	7,486	9,581	8,849	9,217
205567_at	8,572	8,972	8,223	8,660	7,970	8,617
205568_at	6,218	6,589	6,268	6,681	7,236	6,509
205569_at	5,366	5,955	5,666	5,625	5,351	5,110
205570_at	7,110	7,116	7,207	7,596	7,236	7,776
205571_at	9,642	9,883	9,422	8,300	8,025	7,891
205572_at	5,559	5,338	5,286	6,042	5,974	6,326
205573_s_at	10,538	11,059	10,424	11,237	11,421	11,392
205574_x_at	6,619	6,853	7,017	8,272	8,281	8,651
205575_at	7,284	7,591	7,340	7,589	7,186	6,976
205576_at	4,701	5,142	4,690	7,388	6,219	6,266
205577_at	7,165	7,599	7,028	6,650	6,435	6,735
205578_at	7,155	6,785	6,734	5,539	5,670	5,782
205579_at	5,933	6,122	6,269	8,127	8,888	8,186
205580_s_at	7,066	6,880	6,865	7,596	7,438	7,545
205581_s_at	6,989	6,562	6,705	6,816	6,759	7,009
205582_s_at	7,002	6,818	7,188	8,271	8,230	8,433
205583_s_at	10,103	10,564	9,467	6,969	8,781	7,707
205584_at	9,488	9,749	8,600	6,676	8,246	7,160
205585_at	7,569	7,223	7,304	7,297	7,354	7,284
205586_x_at	6,953	6,972	7,350	7,645	7,802	7,951
205587_at	5,679	5,501	5,716	5,719	5,572	5,969
205588_s_at	8,662	8,381	8,318	8,842	8,781	8,444
205589_at	7,426	7,466	7,248	8,242	7,865	8,168
205590_at	5,704	6,935	6,456	5,046	4,927	4,907
205591_at	7,329	6,993	7,418	6,506	6,486	6,300
205592_at	7,651	7,580	7,614	7,945	7,849	7,865
205593_s_at	10,532	10,458	10,262	7,173	7,228	7,022
205594_at	7,511	7,694	7,616	6,159	6,363	5,712
205595_at	6,225	6,182	6,244	6,792	6,625	6,608
205596_s_at	9,458	9,574	9,582	10,267	11,040	10,693
205597_at	5,869	5,592	5,791	6,901	6,196	6,404
205598_at	9,070	8,670	9,070	7,977	7,841	7,950
205599_at	6,490	6,612	6,677	7,544	7,214	7,554
205600_x_at	6,246	6,173	6,395	7,314	7,094	7,071
205601_s_at	7,254	7,232	7,671	7,875	7,733	8,085
205602_x_at	6,882	7,035	6,762	7,608	7,307	7,712
205603_s_at	10,134	10,482	10,279	6,526	7,564	6,230
205604_at	6,415	6,488	6,216	7,050	6,810	7,078
205605_at	6,698	6,173	6,668	6,594	6,476	6,342
205606_at	9,953	9,927	9,528	8,616	8,618	8,873
205607_s_at	8,960	9,282	9,095	7,604	7,474	7,354
205608_s_at	6,594	6,287	7,092	7,628	8,313	8,366
205609_at	6,875	5,662	6,313	7,535	9,778	8,738
205610_at	7,088	7,063	7,017	7,463	7,115	7,167
205611_at	6,765	6,529	6,468	7,078	7,003	7,010
205612_at	3,706	3,923	3,846	3,903	3,781	4,105
205613_at	7,717	7,901	7,715	8,022	7,841	8,167
205614_x_at	7,985	8,153	8,143	8,690	8,593	8,775

205615_at	4,864	4,875	5,243	5,584	5,435	5,571
205616_at	7,311	7,621	6,996	7,062	6,976	7,068
205617_at	6,993	6,942	7,179	7,132	7,097	7,159
205618_at	9,250	9,843	9,001	8,507	9,162	8,685
205619_s_at	6,508	5,599	6,292	7,012	7,215	7,032
205620_at	5,948	6,323	6,212	6,785	6,240	6,752
205621_at	9,396	9,310	9,303	8,651	8,261	8,560
205622_at	8,020	8,297	8,450	8,873	7,977	8,682
205623_at	7,006	7,034	7,096	7,504	7,351	7,541
205624_at	5,157	5,588	5,478	5,446	5,687	5,693
205625_s_at	4,780	5,079	5,197	4,755	4,836	4,767
205626_s_at	6,082	6,947	6,679	5,269	5,355	5,439
205627_at	6,993	7,139	7,182	8,436	7,755	8,343
205628_at	6,748	6,450	6,591	6,939	6,834	6,390
205629_s_at	7,466	7,244	7,436	8,014	8,145	8,067
205630_at	5,339	5,356	5,571	5,557	5,454	5,490
205631_at	8,558	8,969	8,573	8,390	8,787	8,044
205632_s_at	6,614	6,993	7,051	4,812	4,802	4,637
205633_s_at	10,595	10,464	10,558	11,375	10,564	11,240
205634_x_at	7,702	8,002	8,010	9,176	8,392	8,971
205635_at	5,878	5,972	6,178	5,904	6,068	6,152
205636_at	6,385	7,063	6,622	7,265	7,279	7,470
205637_s_at	9,371	9,843	8,812	7,573	7,114	7,387
205638_at	4,703	4,800	4,822	5,233	4,860	5,073
205639_at	6,762	6,918	6,674	7,571	7,350	7,761
205640_at	6,645	6,844	6,634	9,010	8,699	8,979
205641_s_at	8,422	8,309	8,301	9,843	9,398	9,903
205642_at	8,382	8,476	7,431	6,499	6,482	6,060
205643_s_at	5,096	5,322	5,365	5,355	5,160	5,201
205644_s_at	13,783	13,807	13,575	13,290	12,671	13,152
205645_at	5,680	5,758	5,664	6,038	6,206	6,134
205646_s_at	8,072	7,821	7,625	8,384	8,235	8,493
205647_at	8,109	8,373	8,031	6,404	6,592	6,455
205648_at	6,621	6,324	6,557	6,541	6,535	6,308
205649_s_at	3,589	3,612	3,353	3,591	3,619	3,742
205650_s_at	6,102	6,407	6,567	7,252	6,426	6,909
205651_x_at	4,138	4,455	4,103	3,908	3,847	3,877
205652_s_at	7,593	8,058	7,590	7,132	7,240	7,873
205653_at	7,623	7,384	7,706	8,071	7,764	8,175
205654_at	6,732	6,727	6,434	7,181	7,333	7,307
205655_at	4,748	4,405	5,237	4,237	4,153	4,227
205656_at	8,072	8,472	8,435	6,567	6,628	6,606
205657_at	7,526	7,443	7,614	7,926	7,688	7,910
205658_s_at	8,015	7,620	8,240	7,324	7,328	7,647
205659_at	10,185	10,591	10,152	9,335	7,969	7,268
205660_at	6,288	6,556	6,393	7,368	7,479	7,566
205661_s_at	9,561	9,203	9,405	8,953	8,351	8,595
205662_at	8,308	8,628	8,535	8,897	8,506	8,877
205663_at	7,717	7,772	7,343	7,745	7,691	7,649
205664_at	9,744	9,795	9,267	8,146	8,603	7,827
205665_at	7,576	7,182	7,766	7,402	6,864	7,323
205666_at	6,394	6,817	6,550	6,727	6,840	6,848
205667_at	10,134	10,247	9,517	8,526	9,698	7,896
205668_at	5,589	6,900	5,009	4,301	6,446	4,514
205669_at	5,388	5,168	5,266	5,484	5,530	5,699
205670_at	7,800	7,874	7,586	7,677	7,419	7,636

205671_s_at	6,886	7,092	7,018	7,128	6,912	6,842
205672_at	9,159	9,257	9,045	8,866	8,986	8,517
205673_s_at	8,046	8,038	7,803	6,312	5,960	6,018
205674_x_at	6,887	6,863	6,913	6,762	6,752	6,930
205675_at	4,404	5,030	4,875	4,252	4,219	4,403
205676_at	7,534	7,527	8,076	6,683	6,593	6,800
205677_s_at	10,221	10,060	9,682	9,320	8,905	9,138
205678_at	6,374	6,966	6,453	6,675	6,827	6,938
205679_x_at	7,128	6,805	6,759	7,644	7,907	7,779
205680_at	5,103	5,241	5,339	4,985	4,951	5,267
205681_at	4,490	4,554	4,458	6,151	5,042	5,185
205682_x_at	7,046	7,181	7,341	7,496	7,273	7,410
205683_x_at	6,921	7,085	6,836	7,144	7,128	7,158
205684_s_at	7,777	8,137	7,566	6,482	7,091	6,576
205685_at	7,040	7,000	6,989	7,379	6,883	6,995
205686_s_at	5,468	4,736	5,629	5,359	5,404	5,324
205687_at	9,942	9,806	10,075	9,238	8,681	9,443
205688_at	7,875	7,887	7,952	7,838	7,665	7,603
205689_at	8,346	7,696	7,714	7,317	7,243	7,125
205690_s_at	10,703	10,762	10,944	10,909	10,608	10,877
205691_at	10,634	10,643	11,000	6,020	5,717	5,882
205692_s_at	7,731	7,784	7,518	8,141	8,288	8,230
205693_at	6,270	6,204	6,233	7,273	7,080	7,420
205694_at	3,878	3,688	3,832	4,104	3,970	4,202
205695_at	7,223	7,728	7,590	7,731	7,791	8,191
205696_s_at	6,676	6,589	6,758	5,791	6,260	6,026
205697_at	6,270	6,149	6,086	7,102	6,980	6,931
205698_s_at	9,898	10,127	9,709	7,793	7,523	7,416
205699_at	5,056	4,953	4,900	4,960	4,898	4,994
205700_at	7,824	8,035	7,924	5,588	5,100	5,813
205701_at	7,402	7,120	7,148	7,158	7,194	7,274
205702_at	9,299	9,361	9,102	7,803	8,299	8,011
205703_at	4,631	4,748	4,798	5,402	5,337	5,213
205704_s_at	9,351	9,163	9,505	8,432	7,967	8,137
205705_at	7,436	7,715	7,128	5,778	6,231	5,814
205706_s_at	7,048	7,904	6,928	7,123	8,058	7,182
205707_at	8,119	7,866	8,529	7,963	7,876	8,147
205708_s_at	7,668	7,603	7,535	7,743	7,734	7,883
205709_s_at	7,774	8,405	7,812	4,907	5,026	5,122
205710_at	5,603	5,809	5,379	5,394	5,358	5,384
205711_x_at	12,535	13,041	12,827	12,678	12,849	12,959
205712_at	7,007	7,545	7,021	5,811	5,507	5,562
205713_s_at	6,481	6,407	6,031	6,741	6,813	6,926
205714_s_at	7,302	7,046	7,202	7,788	8,047	8,047
205715_at	6,643	7,249	6,868	7,006	6,686	6,668
205716_at	6,292	6,468	6,322	6,763	6,303	6,342
205717_x_at	8,580	8,391	8,490	9,032	9,460	9,629
205718_at	8,161	8,000	7,826	8,349	8,335	8,297
205719_s_at	4,449	5,158	4,396	4,652	4,490	4,594
205720_at	7,180	6,973	6,707	6,965	6,737	6,789
205721_at	6,867	6,742	7,025	6,869	6,715	6,950
205722_s_at	7,333	7,018	7,065	7,474	7,562	7,715
205723_at	7,733	7,570	7,675	8,497	8,367	8,643
205724_at	6,338	6,108	6,434	6,873	6,916	6,854
205725_at	6,299	5,987	6,176	5,967	5,919	6,027
205726_at	9,127	9,288	9,339	7,352	8,759	7,248

205727_at	5,764	5,330	5,488	5,927	5,768	6,066
205728_at	4,280	4,474	4,724	4,431	4,282	4,455
205729_at	4,635	4,969	4,885	6,407	6,533	6,320
205730_s_at	6,664	5,723	6,583	9,195	8,936	8,779
205731_s_at	5,787	6,126	6,029	6,663	6,194	6,561
205732_s_at	4,347	4,257	4,052	4,229	4,077	4,068
205733_at	10,427	10,296	10,106	8,395	8,299	7,419
205734_s_at	6,307	5,830	6,311	7,116	7,241	7,231
205735_s_at	5,999	6,002	6,033	6,926	6,970	6,795
205736_at	6,171	6,008	6,151	6,622	6,378	6,657
205737_at	8,157	7,882	7,699	6,714	6,031	6,408
205738_s_at	7,491	7,772	7,501	6,856	6,992	7,536
205739_x_at	10,432	9,979	9,917	5,886	6,928	5,602
205740_s_at	9,115	8,922	9,303	9,286	8,776	9,398
205741_s_at	8,958	9,204	9,564	9,002	7,969	8,119
205742_at	7,208	7,646	6,915	6,747	6,335	6,710
205743_at	9,216	9,076	9,275	7,146	7,842	7,699
205744_at	7,725	7,647	7,483	8,024	7,745	8,000
205745_x_at	9,470	9,343	9,120	8,175	9,153	8,568
205746_s_at	6,047	5,692	5,376	5,560	5,817	5,458
205747_at	9,121	8,936	9,533	6,736	6,362	6,534
205748_s_at	9,045	8,615	8,539	9,151	8,469	8,855
205749_at	7,462	7,923	7,390	7,615	7,794	7,959
205750_at	8,464	8,405	8,541	7,767	7,380	7,648
205751_at	7,083	7,942	7,356	3,996	3,923	3,918
205752_s_at	7,516	7,964	7,922	8,293	8,111	7,994
205753_at	6,310	5,915	5,949	7,086	6,930	7,062
205754_at	7,221	7,049	7,134	7,593	7,360	7,303
205755_at	6,236	5,567	6,083	6,626	6,695	6,905
205756_s_at	6,250	6,597	6,476	7,154	6,994	7,314
205757_at	6,738	6,421	6,133	7,714	7,418	7,578
205758_at	6,462	6,808	6,586	6,997	6,514	6,695
205759_s_at	6,470	6,902	6,757	6,792	6,863	6,680
205760_s_at	9,120	8,868	9,032	8,649	8,167	8,682
205761_s_at	8,635	8,820	8,445	7,506	6,869	6,647
205762_s_at	7,810	7,682	7,450	6,831	6,698	6,165
205763_s_at	10,547	10,717	10,162	8,675	8,747	7,683
205764_at	4,866	4,839	5,110	5,068	4,805	5,087
205765_at	4,786	5,056	4,827	5,168	5,124	5,069
205766_at	7,830	8,007	8,102	8,602	8,247	8,372
205767_at	3,945	4,078	4,154	10,862	12,758	9,850
205768_s_at	7,341	8,039	7,996	7,038	7,003	6,957
205769_at	6,065	6,178	6,086	5,349	5,196	5,295
205770_at	8,936	9,235	9,097	9,146	8,398	8,914
205771_s_at	10,545	10,955	10,503	7,450	7,568	7,004
205772_s_at	4,702	4,619	4,472	5,138	5,217	4,979
205773_at	4,464	5,341	4,848	4,115	4,642	4,248
205774_at	7,783	8,595	7,980	7,995	7,702	8,025
205775_at	8,514	8,422	8,530	9,551	9,472	9,698
205776_at	6,128	6,513	6,363	5,881	5,543	5,532
205777_at	6,757	6,757	6,785	7,044	6,838	6,607
205778_at	7,066	7,366	6,666	7,569	7,562	7,552
205779_at	7,473	7,379	7,488	7,789	7,223	7,482
205780_at	7,358	7,098	7,024	6,123	6,157	6,380
205781_at	7,178	7,338	7,660	8,292	8,437	8,478
205782_at	4,391	5,171	4,555	5,296	5,177	4,629

205783_at	7,004	6,823	6,874	7,012	6,951	7,094
205784_x_at	8,396	8,413	8,495	8,535	8,453	8,338
205785_at	7,260	7,012	7,150	7,521	7,349	7,571
205786_s_at	7,233	7,392	7,090	6,767	6,858	6,941
205787_x_at	8,574	9,071	8,648	7,677	7,913	7,759
205788_s_at	12,588	12,901	12,234	10,677	11,979	10,685
205789_at	5,722	6,241	6,598	5,945	5,430	5,806
205790_at	7,049	7,288	7,028	7,196	7,194	7,267
205791_x_at	7,348	7,547	6,940	6,789	6,101	6,348
205792_at	6,934	6,725	6,171	7,432	7,246	7,189
205793_x_at	7,091	6,968	6,978	7,962	7,653	7,898
205794_s_at	8,851	8,291	8,638	6,624	7,643	5,995
205795_at	6,470	6,621	7,473	5,868	6,109	5,870
205796_at	7,106	7,298	7,249	8,797	8,349	8,889
205797_s_at	7,393	7,460	7,705	8,113	7,813	8,183
205798_at	6,799	6,336	6,809	8,452	8,130	8,116
205799_s_at	5,025	5,392	5,307	4,681	4,786	4,686
205800_at	5,219	4,990	5,398	6,395	6,044	6,117
205801_s_at	4,943	5,229	5,339	5,751	5,781	5,599
205802_at	9,221	9,630	8,893	7,673	8,697	7,568
205803_s_at	9,130	9,519	8,705	6,797	8,242	6,715
205804_s_at	5,032	5,166	5,337	5,588	5,379	5,605
205805_s_at	10,189	10,080	10,226	7,968	9,234	8,825
205806_at	7,101	6,657	7,257	7,992	7,672	7,819
205807_s_at	9,349	9,757	9,864	7,203	8,199	7,803
205808_at	6,033	6,306	6,132	7,072	7,204	7,157
205809_s_at	9,327	9,418	9,616	8,236	7,761	8,310
205810_s_at	7,038	6,774	6,701	7,163	6,923	7,079
205811_at	8,988	9,090	8,571	8,079	8,053	7,933
205812_s_at	12,202	12,535	12,742	12,841	13,049	13,098
205813_s_at	7,838	7,866	7,986	8,594	8,291	8,383
205814_at	7,795	7,116	7,344	6,429	6,499	6,573
205815_at	6,063	5,887	6,104	6,404	6,213	6,468
205816_at	5,461	5,495	5,623	5,832	5,794	5,764
205817_at	6,895	6,442	6,550	7,931	8,272	8,421
205818_at	9,018	9,080	10,007	7,571	7,546	8,333
205819_at	6,407	6,455	6,536	6,881	6,846	6,917
205820_s_at	7,201	7,389	7,327	8,029	7,440	7,886
205821_at	6,787	6,363	6,388	7,199	7,199	6,857
205822_s_at	6,687	6,266	5,807	6,293	6,118	6,139
205823_at	8,095	7,811	7,812	7,717	7,702	7,829
205824_at	7,158	7,159	7,350	8,161	8,224	8,384
205825_at	5,070	5,018	4,982	4,667	5,249	4,998
205826_at	6,304	6,095	6,088	7,085	6,524	7,133
205827_at	6,527	6,546	6,180	6,910	7,013	7,326
205828_at	5,477	5,400	4,959	13,082	7,581	6,018
205829_at	5,398	5,236	5,125	4,760	4,660	4,934
205830_at	9,541	10,062	8,696	4,524	4,873	4,932
205831_at	6,965	6,600	6,659	7,283	7,115	7,351
205832_at	6,847	7,130	6,980	8,343	8,680	7,875
205833_s_at	6,723	6,879	7,103	7,488	7,660	7,801
205834_s_at	6,752	6,364	6,536	6,891	7,104	7,110
205835_s_at	4,763	5,161	4,053	5,016	5,351	4,995
205836_s_at	7,265	7,252	6,270	6,382	7,969	6,524
205837_s_at	3,387	3,589	3,336	3,485	3,429	3,539
205838_at	5,295	5,460	5,113	5,847	5,341	5,703

205839_s_at	7,272	7,485	7,325	7,103	7,047	6,767
205840_x_at	6,461	6,514	6,433	6,673	6,494	6,998
205841_at	5,519	5,958	5,246	4,872	6,082	5,447
205842_s_at	6,542	6,254	6,077	6,511	7,030	6,302
205843_x_at	8,346	8,611	8,650	9,183	9,400	9,520
205844_at	5,566	5,361	5,577	6,026	5,821	6,088
205845_at	7,499	7,440	7,641	7,332	7,133	7,368
205846_at	6,695	5,859	6,118	6,938	6,627	6,785
205847_at	7,247	7,508	7,600	7,797	7,558	7,817
205848_at	4,256	4,329	4,365	4,102	4,036	3,879
205849_s_at	12,349	12,705	11,985	12,260	12,117	12,573
205850_s_at	7,321	6,355	7,415	5,890	5,713	5,752
205851_at	7,581	7,347	7,584	7,953	7,060	7,458
205852_at	5,613	5,281	5,177	6,530	6,435	6,450
205853_at	6,942	6,758	6,717	7,534	7,228	7,402
205854_at	10,358	10,578	10,728	9,486	9,322	9,757
205855_at	7,495	7,835	7,439	7,458	7,743	7,756
205856_at	4,430	4,602	4,320	8,420	6,984	5,372
205857_at	6,322	7,386	5,949	5,976	5,938	6,108
205858_at	6,321	6,050	5,976	6,557	6,246	6,486
205859_at	6,591	6,541	6,412	6,836	6,987	6,752
205860_x_at	5,465	6,025	5,166	5,574	5,426	5,612
205861_at	7,135	6,852	7,314	7,315	7,263	7,489
205862_at	8,475	8,168	8,437	3,522	3,409	3,591
205863_at	5,500	5,403	5,487	5,741	5,431	5,851
205864_at	7,396	6,975	6,927	7,125	6,845	6,983
205865_at	8,877	8,673	8,598	7,164	6,745	6,854
205866_at	6,083	5,679	6,307	6,954	6,537	6,820
205867_at	4,963	5,203	5,072	4,678	4,997	4,682
205868_s_at	6,910	6,624	6,797	7,406	7,397	7,427
205869_at	5,696	5,648	5,885	6,759	5,780	5,755
205870_at	6,231	6,732	6,589	6,964	7,032	7,087
205871_at	6,161	5,217	5,849	4,380	4,346	4,421
205872_x_at	7,048	7,161	6,741	11,541	9,572	9,968
205873_at	6,928	7,368	7,084	7,502	7,423	7,531
205874_at	7,092	6,683	7,022	7,655	7,475	7,748
205875_s_at	8,148	7,558	7,914	9,586	8,788	9,066
205876_at	4,931	4,951	4,880	5,808	6,918	6,216
205877_s_at	7,896	7,758	8,097	7,922	8,194	8,347
205878_at	7,317	7,825	7,993	7,239	6,884	7,189
205879_x_at	8,700	8,671	8,613	8,973	8,857	9,220
205880_at	7,717	7,747	7,802	7,874	8,067	7,760
205881_at	7,460	7,448	7,470	7,014	6,113	6,747
205882_x_at	9,708	9,931	9,168	8,384	10,568	9,164
205883_at	6,827	6,664	6,508	7,225	7,808	8,028
205884_at	5,097	4,714	4,689	6,215	6,108	5,397
205885_s_at	4,153	4,208	4,419	6,273	6,655	5,989
205886_at	6,267	6,404	5,997	6,772	6,575	6,627
205887_x_at	7,541	7,523	7,484	6,564	7,628	6,388
205888_s_at	8,111	8,510	7,258	6,363	6,244	6,066
205889_s_at	5,523	5,300	4,912	4,345	4,486	4,374
205890_s_at	5,083	5,124	5,078	5,279	5,228	5,569
205891_at	7,953	8,614	8,400	10,384	9,681	9,533
205892_s_at	4,521	4,729	4,311	4,585	4,434	4,661
205893_at	7,405	7,378	6,447	4,685	5,025	5,370
205894_at	8,482	8,177	9,000	7,224	6,840	7,191

205895_s_at	9,935	9,610	9,997	9,198	8,885	8,654
205896_at	6,092	5,334	5,973	9,643	8,697	8,883
205897_at	7,264	7,056	7,399	7,172	6,812	7,156
205898_at	5,133	5,407	5,301	5,014	5,045	5,083
205899_at	5,676	6,603	5,734	4,597	4,411	4,572
205900_at	6,536	6,653	6,363	6,766	6,927	7,119
205901_at	5,960	5,888	5,835	5,949	5,860	5,920
205902_at	7,337	6,817	6,786	7,441	6,937	6,861
205903_s_at	6,344	6,203	5,543	6,613	6,418	6,309
205904_at	8,817	9,305	8,874	10,345	10,356	10,925
205905_s_at	8,693	8,783	8,788	10,135	9,380	10,455
205906_at	7,419	6,936	7,112	7,905	7,721	7,711
205907_s_at	3,827	4,143	4,185	4,258	4,258	4,480
205908_s_at	4,417	4,560	4,727	4,961	4,817	5,105
205909_at	11,079	11,263	10,899	10,144	10,028	9,508
205910_s_at	7,265	6,737	6,851	5,127	4,823	5,096
205911_at	7,550	7,958	7,937	7,708	7,352	7,663
205912_at	5,692	5,923	6,017	5,825	5,766	5,888
205913_at	7,173	7,258	7,230	7,496	7,184	7,356
205914_s_at	6,443	6,436	5,812	6,796	6,518	6,611
205915_x_at	6,601	6,863	6,832	6,972	6,647	6,651
205916_at	6,534	6,551	6,672	6,993	6,976	7,227
205917_at	8,389	8,093	8,241	6,814	6,938	6,866
205918_at	7,646	7,388	7,485	7,417	7,425	7,777
205919_at	7,017	6,719	7,240	7,779	7,644	7,657
205920_at	5,702	5,975	5,996	6,460	6,103	6,439
205921_s_at	5,492	4,918	4,801	5,877	5,395	5,459
205922_at	6,541	6,923	6,407	7,499	7,161	7,174
205923_at	6,239	5,810	6,298	6,021	7,417	6,388
205924_at	8,358	8,249	8,237	7,130	6,843	7,384
205925_s_at	7,543	7,483	7,501	6,810	6,616	6,898
205926_at	7,094	6,995	6,930	7,413	7,217	7,381
205927_s_at	6,729	7,012	6,777	7,145	6,929	7,202
205928_at	9,478	9,671	9,288	7,069	6,785	6,404
205929_at	7,451	7,315	7,497	7,350	7,247	7,409
205930_at	9,961	10,191	10,188	8,374	7,990	7,809
205931_s_at	7,542	7,507	7,151	5,955	6,729	5,765
205932_s_at	6,509	6,720	6,567	8,844	8,252	8,558
205933_at	7,641	7,214	7,807	6,427	6,107	5,987
205934_at	5,312	5,538	5,421	5,192	5,535	5,420
205935_at	6,434	6,472	6,501	6,314	6,310	6,393
205936_s_at	7,122	7,076	6,993	7,589	7,771	7,996
205937_at	6,255	6,183	5,855	6,739	6,978	7,204
205938_at	6,753	6,847	6,702	6,075	5,831	5,957
205939_at	5,361	5,883	6,011	6,276	6,186	6,081
205940_at	6,371	5,232	5,969	5,926	5,369	5,858
205941_s_at	7,103	7,054	7,076	7,380	7,297	7,482
205942_s_at	5,129	6,721	5,228	5,599	5,350	5,855
205943_at	6,042	5,142	5,635	5,446	5,053	5,316
205944_s_at	8,377	8,542	8,458	8,089	8,031	8,290
205945_at	5,658	6,163	6,258	6,864	6,918	6,686
205946_at	8,520	8,476	8,587	8,599	8,490	8,650
205947_s_at	6,360	6,426	6,345	5,705	5,637	5,795
205948_at	7,018	6,727	6,941	7,338	6,984	6,880
205949_at	4,977	4,909	5,133	5,263	5,122	5,326
205950_s_at	7,745	7,769	7,490	7,584	7,532	7,609

205951_at	4,239	4,567	4,293	4,729	5,058	5,167
205952_at	4,909	4,854	5,138	5,716	5,418	5,801
205953_at	8,808	8,768	8,391	7,431	7,643	7,235
205954_at	5,461	5,268	5,008	6,124	6,045	5,757
205955_at	5,549	5,359	5,253	5,590	5,116	5,635
205956_x_at	9,048	8,954	8,975	8,838	8,786	8,555
205957_at	6,435	6,644	6,505	7,638	7,239	7,159
205958_x_at	6,240	6,397	6,522	6,680	6,672	6,979
205959_at	5,280	5,833	5,992	5,717	5,712	5,741
205960_at	5,610	5,762	5,974	6,436	6,784	6,479
205961_s_at	11,828	12,050	11,447	7,793	8,491	6,591
205962_at	5,439	5,403	5,431	5,579	5,418	5,761
205963_s_at	10,982	11,044	11,402	10,969	10,843	10,851
205964_at	6,658	6,440	6,841	5,092	5,204	5,061
205965_at	7,154	7,247	7,222	7,917	8,289	8,731
205966_at	5,024	5,056	5,084	6,687	6,205	7,313
205967_at	13,465	13,334	13,318	12,978	12,358	12,700
205968_at	8,747	9,330	9,153	6,868	6,012	7,044
205969_at	5,670	5,980	5,939	6,126	6,536	6,172
205970_at	7,445	7,463	7,376	8,412	8,395	8,641
205971_s_at	5,661	5,579	5,367	6,182	5,944	6,123
205972_at	6,573	6,464	6,416	6,895	6,771	6,795
205973_at	7,225	7,093	7,405	7,562	7,515	7,362
205974_at	4,085	4,078	4,008	3,999	3,899	4,178
205975_s_at	7,210	7,597	7,815	7,342	6,823	6,877
205976_at	6,155	6,558	6,464	4,565	6,294	4,591
205977_s_at	9,826	9,910	9,877	8,314	7,849	8,063
205978_at	3,661	3,869	3,835	3,833	3,973	3,783
205979_at	4,590	4,468	4,210	5,185	4,733	4,997
205980_s_at	7,646	7,794	7,560	4,862	4,637	4,838
205981_s_at	9,146	9,529	8,971	9,099	8,751	8,728
205982_x_at	7,165	7,199	7,332	7,592	7,472	7,542
205983_at	7,482	7,262	7,633	7,738	7,837	8,234
205984_at	5,768	5,559	5,899	6,080	6,212	6,025
205985_x_at	7,114	6,995	7,153	7,558	7,374	7,695
205986_at	7,959	7,860	8,032	7,703	7,336	7,610
205987_at	7,568	7,619	7,778	8,494	8,538	8,576
205988_at	5,598	5,627	5,843	5,982	5,864	5,699
205989_s_at	4,539	4,410	4,742	4,397	4,451	4,220
205990_s_at	10,220	9,849	10,356	11,360	12,071	11,675
205991_s_at	6,359	5,929	6,133	7,541	7,823	7,666
205992_s_at	4,698	5,315	4,491	8,226	7,785	8,052
205993_s_at	7,164	7,070	6,869	7,709	7,439	7,720
205994_at	5,482	6,026	5,610	5,532	5,310	5,359
205995_x_at	10,465	10,737	9,947	8,556	8,495	8,339
205996_s_at	9,103	8,690	9,121	8,834	8,116	8,444
205997_at	5,531	5,746	5,257	5,605	5,546	5,639
205998_x_at	7,966	7,600	7,884	7,681	8,100	7,617
205999_x_at	5,164	5,258	5,548	5,770	5,377	5,709
206000_at	5,877	5,616	5,360	6,014	6,163	5,864
206001_at	7,086	6,624	6,965	7,138	7,226	7,241
206002_at	9,367	9,709	10,173	6,580	7,110	7,276
206003_at	9,192	9,256	8,448	7,935	8,359	7,629
206004_at	6,556	6,408	6,374	7,264	6,813	7,157
206005_s_at	7,538	7,465	7,115	6,665	7,209	6,250
206006_s_at	8,105	7,500	7,394	5,921	5,912	5,351

206007_at	3,794	4,055	4,200	4,253	8,618	4,157
206008_at	7,475	7,053	7,310	7,832	7,469	7,951
206009_at	6,797	6,718	6,925	6,963	7,003	7,008
206010_at	6,977	6,724	7,015	7,524	7,344	7,638
206011_at	4,667	4,728	4,612	5,604	5,558	5,208
206012_at	7,425	7,552	10,020	7,182	7,334	7,366
206013_s_at	5,426	5,579	5,639	6,164	5,864	6,002
206014_at	7,182	6,960	7,103	7,863	7,726	8,055
206015_s_at	11,994	12,071	12,069	9,806	10,592	10,057
206016_at	7,884	7,938	7,934	8,492	6,968	8,077
206017_at	6,313	6,124	6,095	6,210	6,126	6,153
206018_at	6,871	7,397	7,115	6,395	6,935	6,610
206019_at	7,963	7,550	7,841	7,108	7,470	7,261
206020_at	8,467	8,255	8,518	8,400	8,440	8,453
206021_at	5,781	5,442	5,839	5,751	5,658	5,935
206022_at	6,609	5,879	6,453	6,108	6,410	5,437
206023_at	9,055	9,294	8,573	6,320	6,181	6,171
206024_at	7,091	7,023	7,040	6,221	5,923	6,010
206025_s_at	6,146	7,013	5,996	9,232	8,432	8,667
206026_s_at	5,181	6,219	4,883	8,945	8,122	7,942
206027_at	5,839	5,782	5,961	8,155	6,828	6,837
206028_s_at	8,314	8,932	7,781	5,081	5,102	5,086
206029_at	5,244	5,440	5,444	5,916	6,421	6,271
206030_at	4,966	5,265	4,737	5,438	5,569	5,490
206031_s_at	8,747	8,441	8,760	8,151	8,056	8,145
206032_at	5,542	5,690	5,718	5,027	5,133	5,021
206033_s_at	4,519	4,483	4,537	4,423	4,447	4,542
206034_at	6,690	6,267	6,724	9,207	8,308	8,666
206035_at	4,361	4,660	4,412	4,587	4,553	4,549
206036_s_at	7,232	7,494	6,602	6,388	6,350	6,686
206037_at	8,812	9,276	8,784	7,837	7,711	7,796
206038_s_at	7,081	7,271	7,012	6,907	6,715	6,928
206039_at	6,793	6,969	6,712	6,029	5,571	6,119
206040_s_at	6,205	5,905	5,938	6,768	6,828	6,899
206042_x_at	10,565	10,280	10,416	8,449	8,500	8,960
206043_s_at	6,653	6,152	6,468	7,076	6,703	7,081
206044_s_at	8,618	8,744	8,781	7,782	7,460	7,802
206045_s_at	5,562	5,401	5,142	5,325	5,653	5,352
206046_at	8,062	7,880	7,809	6,522	6,519	6,766
206047_at	9,128	8,990	8,943	8,464	8,637	8,543
206048_at	4,422	4,732	4,688	4,646	4,645	4,760
206049_at	7,126	6,900	7,094	7,324	7,290	7,400
206050_s_at	10,105	10,247	10,201	12,304	11,723	12,620
206051_at	7,055	7,210	6,532	7,654	7,143	7,417
206052_s_at	11,036	10,915	10,838	10,474	9,955	10,359
206053_at	7,751	8,091	7,503	7,559	7,586	7,513
206054_at	5,594	6,306	5,692	6,033	5,324	5,754
206055_s_at	12,708	12,481	12,123	11,958	12,258	11,858
206056_x_at	9,219	8,691	8,811	9,086	9,763	9,562
206057_x_at	7,821	7,621	7,818	8,385	8,163	8,439
206058_at	6,698	6,294	6,255	5,857	5,826	5,920
206059_at	7,947	8,026	7,366	4,807	6,080	4,612
206060_s_at	4,855	4,190	5,164	5,889	5,224	5,201
206061_s_at	8,880	8,610	8,432	6,382	7,484	6,760
206062_at	8,917	9,001	9,017	7,825	7,386	7,658
206063_x_at	8,499	8,495	8,674	8,667	8,931	8,841

206064_s_at	7,188	6,578	7,483	7,829	7,577	7,853
206065_s_at	5,304	5,438	5,275	5,861	5,477	5,701
206066_s_at	7,568	7,333	7,222	8,387	7,865	8,022
206067_s_at	5,783	7,232	7,017	4,894	5,043	4,913
206068_s_at	5,867	6,330	6,103	5,847	5,315	5,491
206069_s_at	5,999	5,946	5,841	5,804	5,768	6,028
206070_s_at	3,445	3,704	3,347	3,705	4,883	3,859
206071_s_at	6,188	6,424	6,431	6,868	6,546	6,660
206072_at	6,646	7,023	7,060	5,972	5,757	6,134
206073_at	6,233	6,584	6,210	6,836	6,907	7,230
206074_s_at	12,527	12,301	12,776	11,906	12,042	12,145
206075_s_at	9,677	9,413	9,260	7,991	7,724	8,045
206076_at	6,886	6,933	6,811	7,425	7,356	7,726
206077_at	7,822	7,473	7,604	8,041	7,746	7,876
206078_at	5,626	5,658	5,591	5,973	5,747	5,942
206079_at	6,367	6,116	5,799	6,262	6,718	6,259
206080_at	7,490	7,144	7,403	7,300	7,631	7,660
206081_at	8,535	8,704	8,592	7,558	8,056	7,691
206082_at	7,067	7,696	8,195	6,881	6,467	6,804
206083_at	7,546	7,276	7,571	8,107	8,081	8,048
206084_at	5,288	5,915	6,215	4,672	4,261	4,546
206085_s_at	7,603	9,210	7,620	6,329	6,511	6,446
206086_x_at	7,321	7,188	7,185	7,827	7,503	7,787
206087_x_at	6,422	6,843	6,811	7,762	7,676	8,016
206088_at	5,444	5,219	5,495	5,641	5,775	5,607
206089_at	6,787	6,793	6,689	7,289	7,142	7,303
206090_s_at	7,110	7,849	7,553	7,110	7,177	6,778
206091_at	5,816	6,125	5,601	4,454	3,875	4,847
206092_x_at	7,880	8,002	8,150	8,209	8,075	7,919
206093_x_at	7,732	8,154	7,853	8,134	8,938	8,280
206094_x_at	7,168	7,325	7,066	7,519	7,750	7,863
206095_s_at	12,237	12,438	11,760	11,253	11,355	10,945
206096_at	8,338	8,026	8,497	6,881	6,948	6,805
206097_at	7,733	8,079	8,008	8,078	7,778	8,403
206098_at	5,775	5,425	5,182	4,197	4,054	4,114
206099_at	5,332	5,795	5,916	5,930	5,578	5,845
206100_at	4,633	4,885	4,540	7,334	7,155	8,396
206101_at	4,624	4,713	4,655	5,730	6,759	7,284
206102_at	11,728	11,432	11,956	10,592	9,415	9,444
206103_at	6,804	6,218	6,253	7,155	6,772	6,971
206104_at	5,408	5,320	5,529	6,493	6,335	6,060
206105_at	7,702	7,428	7,635	7,866	7,695	7,890
206106_at	7,785	7,948	7,915	8,455	7,406	8,439
206107_at	6,114	5,741	5,888	5,760	5,820	5,678
206108_s_at	7,880	7,418	8,093	8,416	6,009	8,259
206109_at	7,034	7,783	7,685	6,359	6,162	6,252
206110_at	7,551	7,590	7,804	7,136	6,783	7,069
206111_at	6,597	6,834	6,802	7,068	7,092	7,160
206112_at	4,196	4,345	4,066	4,334	4,398	4,681
206113_s_at	8,214	8,275	7,685	7,743	7,641	7,977
206114_at	8,193	8,393	8,211	7,426	7,734	8,112
206115_at	7,610	9,937	9,656	4,649	6,413	6,022
206116_s_at	10,129	9,776	10,188	9,448	8,673	9,914
206117_at	9,308	9,525	10,130	8,014	7,554	8,525
206118_at	5,859	6,305	5,910	6,899	6,622	6,634
206119_at	7,499	7,635	6,971	6,320	6,640	6,253

206120_at	6,449	6,142	6,306	6,772	6,446	6,626
206121_at	5,188	5,684	5,546	5,213	5,060	5,281
206122_at	7,897	7,696	7,931	8,404	8,570	8,672
206123_at	8,356	8,250	8,221	9,160	8,381	9,117
206124_s_at	7,730	7,561	7,689	7,787	7,959	7,924
206125_s_at	7,414	7,654	7,590	7,116	7,051	7,177
206126_at	5,921	6,491	6,286	6,239	6,283	6,134
206127_at	6,542	6,434	6,241	6,753	6,786	6,946
206128_at	7,665	7,807	7,506	7,991	8,129	8,287
206129_s_at	5,388	5,203	6,157	6,263	6,265	6,205
206130_s_at	7,346	7,201	7,144	7,953	7,613	7,747
206131_at	7,258	7,001	7,158	7,345	7,410	7,451
206132_at	7,111	6,929	6,943	6,742	7,206	6,599
206133_at	6,609	6,542	6,494	7,652	7,520	7,822
206134_at	4,415	4,402	4,363	4,331	4,277	4,405
206135_at	4,109	4,228	4,563	4,898	4,254	4,403
206136_at	8,100	7,677	7,531	7,078	6,855	6,845
206137_at	7,939	8,032	7,791	7,688	7,314	7,628
206138_s_at	10,090	9,848	10,250	10,882	11,002	11,188
206139_at	8,282	8,076	8,568	9,240	8,991	9,131
206140_at	10,672	10,355	10,403	4,779	4,648	4,687
206141_at	7,474	7,146	7,628	7,667	7,029	7,307
206142_at	6,853	6,661	7,232	6,158	5,672	5,784
206143_at	3,744	3,830	3,659	4,137	4,237	4,178
206144_at	8,889	8,087	7,878	5,852	5,986	5,930
206145_at	3,807	4,265	3,871	3,744	3,663	3,725
206146_s_at	6,613	6,349	6,480	6,745	6,689	6,850
206147_x_at	11,598	11,883	11,503	9,643	10,682	9,870
206148_at	7,547	8,032	7,565	8,477	8,250	8,185
206149_at	6,611	6,384	6,672	6,806	6,887	6,753
206150_at	7,289	7,373	7,142	7,737	7,865	7,759
206151_x_at	7,098	6,833	6,846	7,571	7,497	7,817
206152_at	7,427	7,585	7,252	7,861	7,583	7,634
206153_at	5,095	4,834	5,011	5,234	5,450	5,663
206154_at	6,729	6,157	6,283	6,431	6,369	6,455
206155_at	5,975	6,006	5,636	6,236	6,041	6,150
206156_at	6,125	6,134	6,173	6,647	6,410	6,568
206157_at	9,106	8,943	8,098	13,599	13,812	13,890
206158_s_at	12,936	12,889	12,558	11,931	11,878	12,023
206159_at	4,069	4,273	3,856	4,134	3,978	4,186
206160_at	7,103	7,019	6,924	7,985	7,563	7,834
206161_s_at	6,409	6,297	6,414	6,739	6,743	6,971
206162_x_at	5,856	5,773	5,426	6,768	6,729	6,963
206163_at	4,618	4,842	4,710	5,230	5,302	5,204
206164_at	4,414	4,292	4,564	5,064	4,852	5,022
206165_s_at	3,843	3,877	4,170	4,164	4,348	3,928
206166_s_at	5,521	5,675	5,612	6,015	5,917	5,922
206167_s_at	6,793	6,887	6,987	6,699	8,698	8,380
206168_at	6,850	6,472	6,802	7,093	6,765	7,035
206169_x_at	8,908	8,587	8,683	8,262	9,341	8,545
206170_at	5,870	5,957	6,019	7,214	7,949	7,639
206171_at	6,228	5,936	6,101	6,321	6,478	6,620
206172_at	5,977	5,666	5,363	10,973	10,844	8,793
206173_x_at	8,408	8,519	8,329	8,631	8,534	8,302
206174_s_at	9,676	9,513	9,608	9,736	9,224	9,439
206175_x_at	7,959	8,149	7,550	6,637	6,436	6,562

206176_at	7,406	7,452	7,194	7,839	7,811	7,583
206177_s_at	5,382	5,409	5,823	5,546	5,499	5,633
206178_at	6,950	6,954	7,069	7,239	7,029	7,407
206179_s_at	5,952	6,168	6,659	6,524	6,552	6,776
206180_x_at	8,350	8,190	8,524	8,115	7,944	8,213
206181_at	5,077	5,347	4,901	6,244	5,814	6,024
206182_at	9,147	9,055	9,112	7,558	7,410	7,168
206183_s_at	5,527	5,721	5,807	6,633	6,944	6,787
206184_at	6,574	6,220	6,673	6,943	6,699	6,881
206185_at	6,445	6,430	6,668	7,116	7,161	7,510
206186_at	7,787	7,625	7,655	7,735	7,733	7,922
206187_at	7,784	7,617	7,878	8,558	8,458	8,402
206188_at	7,938	7,618	7,983	6,808	6,509	6,597
206189_at	4,552	4,961	4,889	5,012	4,898	5,134
206190_at	4,665	4,949	4,448	4,999	4,956	5,365
206191_at	6,267	6,246	6,526	6,831	6,716	6,938
206192_at	6,836	7,041	7,290	7,287	7,067	7,071
206193_s_at	6,156	6,176	6,205	6,135	6,090	6,239
206194_at	7,618	8,041	7,604	9,341	8,891	9,212
206195_x_at	6,221	6,371	6,365	6,082	5,945	6,003
206196_s_at	6,498	7,067	7,288	7,021	6,879	7,084
206197_at	6,698	7,163	6,742	6,996	6,630	7,378
206198_s_at	4,178	4,024	3,903	4,581	4,494	4,692
206199_at	5,423	5,717	5,681	6,193	6,012	5,974
206200_s_at	9,335	9,833	9,508	11,080	11,613	11,756
206201_s_at	4,026	4,067	4,663	4,200	6,034	6,404
206202_at	4,043	4,056	4,347	3,881	3,977	3,901
206203_at	6,817	6,757	6,839	7,383	7,379	7,460
206204_at	8,246	8,719	7,423	6,510	7,734	6,664
206205_at	9,593	10,159	8,690	7,600	7,993	6,310
206206_at	7,450	7,428	7,352	7,686	7,840	7,722
206207_at	5,562	5,406	5,757	6,044	5,697	6,198
206208_at	6,745	7,236	6,766	8,094	8,033	8,285
206209_s_at	7,021	7,501	6,993	6,859	6,613	6,762
206210_s_at	5,741	5,752	5,266	6,433	6,108	5,906
206211_at	5,047	5,213	5,368	5,395	5,260	5,402
206212_at	7,028	6,882	6,948	6,806	6,438	6,706
206213_at	6,919	7,067	7,080	7,201	7,073	7,084
206214_at	5,692	5,660	5,073	4,003	4,102	4,132
206215_at	6,637	6,945	6,632	7,048	7,013	7,181
206216_at	7,679	7,666	7,684	8,451	8,763	8,755
206217_at	7,969	8,031	7,870	7,720	7,631	7,312
206218_at	5,160	5,215	5,040	5,544	5,368	5,536
206219_s_at	6,186	6,363	6,314	6,542	6,526	6,698
206220_s_at	7,631	7,406	7,662	8,259	8,267	8,509
206221_at	5,930	5,204	5,581	6,427	6,067	6,497
206222_at	6,893	6,505	6,745	7,438	7,107	6,903
206223_at	6,446	5,904	5,897	7,115	6,799	6,727
206224_at	5,116	4,878	5,108	5,856	5,502	5,765
206225_at	6,621	6,850	6,934	7,353	6,947	7,168
206226_at	5,710	5,614	6,142	6,876	6,901	6,763
206227_at	7,483	7,621	7,486	7,305	7,321	7,537
206228_at	6,256	5,617	5,803	5,708	5,549	5,974
206229_x_at	7,277	7,543	7,651	8,039	7,780	7,897
206230_at	6,529	6,573	6,624	6,440	6,530	6,586
206231_at	8,901	8,681	8,997	8,733	8,443	8,612

206232_s_at	5,605	5,542	5,795	5,405	5,153	5,272
206233_at	7,580	7,903	7,278	5,817	5,874	5,970
206234_s_at	7,647	7,206	7,475	7,913	7,903	7,890
206235_at	6,585	6,321	6,449	6,556	6,494	6,638
206236_at	7,177	7,084	6,990	7,614	7,480	7,609
206237_s_at	7,246	7,345	7,479	8,874	8,352	8,628
206238_s_at	6,385	6,524	6,744	7,043	7,097	7,221
206239_s_at	5,411	5,488	5,590	5,419	5,791	5,538
206240_s_at	8,510	8,922	8,187	6,608	7,042	6,795
206241_at	5,961	6,336	5,864	5,784	5,683	5,682
206242_at	6,487	6,785	6,603	6,506	6,329	6,606
206243_at	9,116	8,260	10,025	8,319	7,761	8,688
206244_at	7,094	7,239	7,092	7,164	7,252	7,225
206245_s_at	12,131	12,523	11,346	10,695	10,263	10,035
206246_at	6,668	6,600	6,856	7,303	7,323	6,963
206247_at	10,564	11,187	10,864	11,487	10,560	11,016
206248_at	5,821	5,825	6,249	6,498	6,230	6,564
206249_at	6,909	6,616	6,818	7,396	7,416	7,445
206250_x_at	6,764	6,839	6,622	7,897	7,375	7,627
206251_s_at	4,015	3,994	4,327	4,656	4,567	4,442
206252_s_at	6,503	6,542	6,759	6,776	6,561	6,659
206253_at	5,191	5,124	5,175	5,081	5,015	5,067
206254_at	7,375	7,932	6,488	5,860	5,992	6,022
206255_at	5,497	5,243	5,400	6,148	5,773	6,305
206256_at	6,927	6,900	6,989	7,144	7,224	7,417
206257_at	8,060	8,324	8,204	8,512	8,158	8,328
206258_at	6,799	6,910	7,161	7,492	7,316	7,534
206259_at	6,227	6,319	6,617	6,693	6,398	6,371
206260_at	5,663	5,447	5,823	6,589	6,617	6,514
206261_at	9,105	8,854	9,128	7,737	7,777	7,380
206262_at	5,238	5,214	5,545	5,970	5,839	6,341
206263_at	6,876	6,779	6,742	6,921	6,727	6,972
206264_at	5,755	5,519	6,151	5,728	5,761	5,902
206265_s_at	5,444	4,604	5,152	5,218	5,075	5,304
206266_at	6,533	6,420	6,595	7,274	7,278	7,317
206267_s_at	7,830	7,961	7,571	7,211	7,181	7,273
206268_at	7,194	7,449	9,329	6,825	6,908	6,939
206269_at	4,927	4,953	5,052	4,935	4,757	4,916
206270_at	7,100	6,706	6,980	7,040	6,833	7,083
206271_at	5,936	5,844	5,223	4,871	5,647	4,848
206272_at	9,731	10,238	10,121	7,917	7,778	8,160
206273_at	8,000	7,773	7,958	8,362	8,055	8,358
206274_s_at	7,180	7,205	7,165	7,085	7,018	7,351
206275_s_at	6,291	5,987	6,782	7,634	7,866	7,965
206276_at	6,505	6,229	6,598	7,746	7,449	7,600
206277_at	6,630	6,369	6,755	7,388	7,042	7,200
206278_at	5,938	5,717	5,954	6,000	5,632	5,925
206279_at	7,364	7,266	7,128	6,301	7,030	6,530
206280_at	6,773	6,615	6,829	7,212	7,059	7,143
206281_at	5,399	5,209	5,416	5,511	5,484	5,441
206282_at	3,788	3,830	3,902	3,873	3,639	3,891
206283_s_at	5,988	5,476	5,418	5,303	5,417	5,585
206284_x_at	8,145	7,859	8,071	9,268	8,898	9,477
206285_at	5,691	5,484	5,756	5,643	5,784	5,655
206286_s_at	13,239	13,385	13,856	6,369	6,134	6,491
206287_s_at	6,846	6,475	6,671	7,285	7,114	7,399

206288_at	6,745	7,012	7,105	7,105	6,357	6,495
206289_at	6,813	6,756	6,886	7,489	7,373	7,772
206290_s_at	6,930	7,564	7,040	4,865	5,729	6,068
206291_at	5,571	6,220	5,583	5,146	5,160	5,208
206292_s_at	5,341	5,493	5,667	6,078	5,641	5,923
206293_at	4,011	4,240	4,390	4,274	4,147	4,038
206294_at	5,179	5,052	5,417	5,857	5,823	6,003
206295_at	4,174	4,078	4,263	3,818	4,286	4,052
206296_x_at	8,009	8,305	8,111	7,694	7,440	7,518
206297_at	7,298	7,244	7,147	8,281	7,899	8,280
206298_at	7,532	7,299	7,858	9,418	8,801	9,140
206299_at	9,110	9,001	9,508	8,118	7,665	7,683
206300_s_at	4,969	4,594	4,811	5,727	5,234	5,210
206301_at	7,409	7,515	7,291	7,482	7,416	7,429
206302_s_at	9,813	9,879	10,015	10,584	9,928	10,632
206303_s_at	9,186	9,309	9,331	9,626	8,669	9,603
206304_at	6,355	6,641	6,557	6,947	6,899	7,075
206305_s_at	6,266	6,199	6,370	6,954	6,550	6,903
206306_at	4,849	5,213	5,026	4,055	4,210	4,337
206307_s_at	8,175	8,449	8,549	10,280	8,630	10,110
206308_at	9,328	9,404	8,760	7,017	7,591	6,973
206309_at	8,419	8,107	8,606	6,898	6,809	6,694
206310_at	7,218	7,648	7,584	7,555	7,299	7,524
206311_s_at	4,174	3,877	4,192	5,021	4,886	5,282
206312_at	5,702	5,729	5,323	5,848	5,977	5,903
206313_at	7,150	6,701	6,838	7,697	7,297	7,928
206314_at	9,825	9,884	9,996	6,437	6,603	6,659
206315_at	7,902	7,988	7,878	7,774	8,002	8,236
206316_s_at	10,327	10,603	9,688	6,999	7,802	6,772
206317_s_at	7,204	7,037	7,084	7,162	6,620	7,352
206318_at	4,399	4,568	4,410	4,357	4,388	4,575
206319_s_at	4,758	5,202	4,938	5,380	4,988	5,398
206320_s_at	5,990	6,065	5,904	6,215	6,312	6,418
206321_at	5,867	5,527	5,766	5,766	5,887	6,116
206322_at	6,727	7,010	6,383	6,424	6,444	6,581
206323_x_at	10,118	10,161	10,403	10,075	10,415	10,028
206324_s_at	8,148	7,970	8,306	8,541	8,382	8,496
206325_at	5,724	5,844	5,796	5,950	6,007	5,938
206326_at	5,881	6,003	6,018	6,340	6,227	6,393
206327_s_at	6,698	6,447	6,812	7,589	7,608	7,910
206328_at	6,057	6,168	6,395	6,922	6,941	7,014
206329_at	6,414	6,320	6,370	6,729	6,758	6,843
206330_s_at	7,108	7,015	7,143	8,084	7,817	8,029
206331_at	5,223	5,692	5,733	5,477	5,533	5,694
206332_s_at	8,886	9,269	8,414	10,037	10,206	9,003
206333_at	7,222	7,078	7,528	7,741	7,296	7,730
206334_at	5,071	4,976	4,961	5,187	5,026	5,351
206335_at	8,735	8,706	8,995	8,920	8,931	9,228
206336_at	6,264	6,734	5,880	10,514	7,253	6,425
206337_at	6,005	5,917	5,565	6,996	6,704	6,602
206338_at	7,044	7,238	7,370	7,646	7,677	7,671
206339_at	7,252	7,020	7,198	7,535	7,453	7,639
206340_at	5,383	5,552	5,453	5,688	5,414	5,506
206341_at	6,617	6,541	6,387	6,658	6,678	6,434
206342_x_at	7,927	8,079	8,228	9,357	9,171	9,623
206343_s_at	9,055	9,334	8,847	11,038	8,682	9,882

206344_at	4,778	5,079	4,687	5,368	5,156	5,445
206345_s_at	6,060	6,114	5,816	6,131	6,195	6,277
206346_at	4,148	4,006	3,853	4,217	3,845	4,017
206347_at	6,064	6,236	6,536	5,892	5,487	5,815
206348_s_at	9,126	9,505	8,945	6,628	6,485	6,481
206349_at	7,725	7,910	6,567	4,692	4,704	4,593
206350_at	6,674	6,702	6,744	7,339	7,309	7,443
206351_s_at	6,718	6,263	6,306	7,233	7,289	7,257
206352_s_at	8,022	8,158	8,560	8,595	8,202	8,642
206353_at	6,367	5,794	6,073	6,278	6,215	6,383
206354_at	5,034	5,116	5,399	5,210	5,161	5,226
206355_at	4,573	4,754	4,530	4,064	4,088	4,178
206356_s_at	6,745	7,031	6,688	6,281	6,277	6,372
206357_at	6,443	6,286	6,155	6,920	7,040	6,996
206358_at	7,207	7,205	7,298	8,026	7,717	7,843
206359_at	6,182	6,427	5,922	7,530	7,418	7,564
206360_s_at	6,904	6,769	6,776	6,982	6,796	6,902
206361_at	6,990	7,282	7,513	7,709	7,754	7,919
206362_x_at	8,559	8,512	8,450	8,948	9,072	9,093
206363_at	9,562	9,257	9,552	6,711	7,125	7,205
206364_at	11,468	11,420	10,742	7,954	9,177	7,088
206365_at	4,569	4,602	4,368	5,159	4,665	4,946
206366_x_at	6,298	6,353	6,004	7,505	6,978	7,050
206367_at	6,265	6,355	6,101	6,585	5,987	6,454
206368_at	6,681	6,887	6,651	7,031	6,991	7,094
206369_s_at	5,070	4,768	4,556	5,148	4,871	5,111
206370_at	3,495	3,611	3,650	3,733	3,768	3,814
206371_at	6,289	5,897	6,152	6,511	6,407	6,544
206372_at	6,713	7,171	6,678	7,708	7,603	7,813
206373_at	6,734	6,515	6,525	6,218	7,349	7,738
206374_at	7,593	7,804	7,626	7,000	7,310	7,500
206375_s_at	5,842	5,973	5,489	6,839	6,845	6,759
206376_at	8,287	8,855	8,405	10,716	10,778	10,646
206377_at	6,115	6,494	6,021	7,669	7,307	8,053
206378_at	3,678	3,826	3,952	3,940	4,496	4,421
206379_at	7,721	7,304	7,576	7,574	7,714	7,603
206380_s_at	5,565	5,202	5,113	5,300	4,927	5,202
206381_at	6,558	6,414	6,359	6,955	6,533	6,888
206382_s_at	7,287	7,504	7,516	5,642	6,800	8,325
206383_s_at	8,802	8,544	8,578	7,986	7,648	8,128
206384_at	5,936	6,181	6,311	6,706	6,686	6,892
206385_s_at	10,022	11,012	9,668	4,719	11,002	6,118
206386_at	6,129	6,168	5,900	6,472	6,401	6,906
206387_at	7,098	6,903	7,097	7,025	6,976	7,050
206388_at	7,403	7,103	7,586	7,764	7,901	7,589
206389_s_at	6,407	6,334	6,605	6,841	6,645	6,896
206390_x_at	7,044	7,078	6,774	8,029	8,661	9,167
206391_at	5,701	5,606	6,235	7,134	6,987	7,003
206392_s_at	5,831	5,968	6,020	6,148	6,452	5,862
206393_at	6,081	6,000	6,237	6,605	6,386	6,529
206394_at	6,020	5,871	5,907	6,077	5,768	6,144
206395_at	6,838	6,531	6,772	7,440	7,172	6,970
206396_at	6,610	6,295	6,363	7,429	7,480	7,625
206397_x_at	8,166	7,814	8,043	7,442	7,282	7,665
206398_s_at	6,106	6,060	6,287	6,792	6,541	6,837
206399_x_at	7,555	7,970	7,506	8,250	8,293	8,443

206400_at	6,729	6,271	6,249	6,985	7,171	6,862
206401_s_at	7,264	7,759	7,178	7,962	7,570	8,064
206402_s_at	8,084	8,092	8,208	8,293	8,072	8,097
206403_at	7,130	7,582	6,520	6,966	7,085	7,064
206404_at	6,714	7,456	7,104	6,594	6,827	6,659
206405_x_at	9,077	9,220	8,828	8,578	9,389	7,845
206406_at	6,396	6,354	6,720	7,569	7,038	7,243
206407_s_at	6,649	6,429	6,712	7,142	6,967	7,328
206408_at	4,498	4,911	4,945	5,490	5,950	5,216
206409_at	6,955	6,963	7,205	7,725	7,374	7,694
206410_at	7,271	7,289	7,599	8,199	7,735	7,870
206411_s_at	7,325	7,401	7,216	7,487	7,610	7,561
206412_at	8,181	8,190	8,087	8,140	8,463	7,839
206413_s_at	8,138	8,107	8,152	7,848	7,545	7,826
206414_s_at	9,950	9,921	9,594	8,665	10,603	9,100
206415_at	4,097	4,373	4,284	4,125	4,155	4,046
206416_at	7,325	7,414	7,706	7,727	7,793	7,501
206417_at	6,785	7,006	6,622	6,712	6,078	6,657
206418_at	3,607	3,544	3,712	3,607	3,702	3,690
206419_at	7,203	7,143	7,295	7,369	7,104	7,364
206420_at	5,977	6,034	6,107	6,670	6,205	6,552
206421_s_at	5,436	6,540	6,207	9,993	7,999	8,831
206422_at	5,173	5,525	4,994	5,445	5,201	5,492
206423_at	6,249	6,121	6,052	6,570	6,520	6,746
206424_at	10,651	10,543	11,720	7,813	7,570	7,691
206425_s_at	5,853	5,862	5,424	6,306	6,028	6,147
206426_at	4,109	3,797	4,060	4,080	4,007	4,135
206427_s_at	6,507	6,492	6,689	6,972	6,715	7,017
206428_s_at	5,917	5,887	5,977	6,108	5,704	5,908
206429_at	6,550	6,744	6,744	7,221	6,805	6,924
206430_at	8,405	8,207	8,361	8,308	8,181	8,429
206431_x_at	8,705	8,718	9,063	8,932	8,969	9,254
206432_at	5,920	6,377	6,446	9,989	8,847	10,407
206433_s_at	4,669	5,254	5,402	5,682	5,547	5,819
206434_at	3,736	3,641	3,821	3,535	3,599	3,604
206435_at	8,122	7,846	8,358	8,068	7,907	8,312
206436_at	7,342	7,182	7,399	7,724	7,464	7,455
206437_at	8,259	8,263	8,396	8,669	8,620	8,809
206438_x_at	8,369	8,440	8,551	8,520	8,798	8,852
206439_at	3,949	4,085	4,118	4,020	3,949	3,979
206440_at	6,558	7,068	6,504	7,408	7,791	8,049
206441_s_at	9,918	9,263	9,849	9,415	8,637	9,150
206442_at	5,280	6,310	6,111	4,607	4,754	4,670
206443_at	6,002	6,585	6,416	6,708	6,663	6,636
206444_at	7,449	7,166	7,590	7,572	7,378	7,386
206445_s_at	10,549	10,150	10,456	10,535	9,286	10,013
206446_s_at	5,676	5,800	5,784	6,987	6,559	6,920
206447_at	5,176	5,134	5,466	5,689	5,715	6,108
206448_at	6,692	6,589	6,688	7,516	7,212	7,302
206449_s_at	5,553	5,683	5,900	6,117	5,876	6,068
206450_at	7,118	7,005	7,045	7,814	7,935	8,047
206451_at	8,119	8,205	8,112	8,600	8,121	8,314
206452_x_at	10,009	10,003	10,296	9,252	9,251	9,488
206453_s_at	8,730	8,778	8,551	8,415	8,308	7,950
206454_s_at	6,729	6,530	6,807	7,546	7,143	7,656
206455_s_at	8,054	7,895	8,154	8,308	8,073	7,981

206456_at	7,769	7,942	7,466	3,898	4,214	4,308
206457_s_at	4,499	4,738	4,700	4,476	4,357	4,580
206458_s_at	6,345	6,573	6,354	6,622	6,977	7,353
206459_s_at	6,026	5,858	5,932	6,189	6,223	6,467
206460_at	6,990	7,144	7,910	6,219	6,664	6,447
206461_x_at	11,335	11,095	12,555	13,476	13,205	13,722
206462_s_at	5,649	5,717	5,748	6,227	5,822	6,038
206463_s_at	7,331	7,242	7,399	8,066	8,075	7,929
206464_at	5,551	5,228	5,514	5,870	5,658	5,775
206465_at	7,654	7,955	7,864	5,145	5,241	5,568
206466_at	6,321	6,266	6,179	6,417	6,300	6,459
206467_x_at	7,924	7,604	7,807	7,698	7,368	7,520
206468_s_at	9,799	9,321	9,513	8,511	7,347	8,079
206469_x_at	7,662	7,680	7,422	8,875	8,011	8,619
206470_at	5,897	6,367	5,676	6,011	5,802	6,018
206471_s_at	4,183	4,360	4,104	3,997	3,855	4,142
206472_s_at	7,283	7,552	7,222	6,844	6,483	6,930
206473_at	6,130	6,085	6,327	6,286	6,250	6,372
206474_at	6,118	6,293	6,263	6,348	6,551	6,248
206475_x_at	6,379	6,719	6,632	6,595	6,591	6,642
206476_s_at	5,249	5,629	5,251	5,829	5,994	6,203
206477_s_at	7,560	7,531	7,681	8,122	8,313	8,389
206478_at	5,692	5,372	5,590	6,049	5,990	5,604
206479_at	4,264	4,265	4,267	4,794	4,508	4,703
206480_at	7,241	7,069	7,159	7,291	7,079	7,458
206481_s_at	8,961	9,687	9,142	5,323	5,768	6,516
206482_at	4,279	4,405	4,765	4,419	4,346	4,435
206483_at	7,280	7,342	7,511	7,459	7,572	7,338
206484_s_at	6,335	6,156	6,473	6,806	6,443	6,771
206485_at	8,281	8,389	8,573	9,042	8,958	8,925
206486_at	6,870	7,315	6,867	6,527	6,394	6,434
206487_at	7,026	6,578	6,493	6,100	6,279	6,313
206488_s_at	5,447	4,965	5,296	5,989	6,741	7,205
206489_s_at	4,937	4,677	4,767	4,752	4,650	4,701
206490_at	5,490	5,271	5,535	5,892	5,861	6,155
206491_s_at	8,519	8,509	8,628	9,415	8,953	9,733
206492_at	5,932	6,132	5,831	5,892	5,663	5,527
206493_at	7,393	7,378	7,305	7,709	7,757	7,597
206494_s_at	5,670	6,092	5,297	5,207	4,933	5,535
206495_s_at	9,408	9,591	9,841	8,290	7,766	8,243
206496_at	5,688	5,434	6,327	6,053	6,699	6,181
206497_at	7,040	6,834	6,527	5,383	5,230	4,969
206498_at	8,395	8,343	8,380	7,840	7,710	8,016
206499_s_at	9,692	9,091	9,531	9,190	8,310	8,506
206500_s_at	7,801	8,172	7,604	6,472	6,350	6,087
206501_x_at	7,959	8,103	8,075	7,117	6,958	6,847
206502_s_at	7,321	8,095	8,118	4,639	4,454	4,549
206503_x_at	7,679	7,828	7,657	8,312	7,801	8,165
206504_at	3,833	3,858	4,160	4,027	3,974	3,959
206505_at	3,426	3,523	3,593	3,399	3,435	3,445
206506_s_at	8,681	9,105	8,632	7,794	7,895	7,845
206507_at	8,407	8,554	8,661	7,316	7,054	7,196
206508_at	7,892	7,881	8,155	8,383	7,950	8,142
206509_at	7,213	6,845	6,929	7,697	7,960	8,205
206510_at	6,960	7,452	7,317	8,917	8,836	10,002
206511_s_at	6,279	6,326	6,575	7,128	6,991	7,619

206512_at	6,973	6,641	7,110	6,953	6,986	6,736
206513_at	7,463	7,522	7,418	7,013	6,802	6,771
206514_s_at	6,257	6,312	6,428	6,664	6,693	6,844
206515_at	4,291	4,399	4,932	4,715	4,761	4,649
206516_at	8,584	8,009	8,102	7,388	7,542	7,413
206517_at	7,382	7,008	7,239	8,022	7,091	7,695
206518_s_at	6,341	5,837	5,867	6,677	6,778	6,972
206519_x_at	4,210	3,984	4,175	4,105	3,976	4,013
206520_x_at	5,884	5,320	5,990	6,705	6,780	6,921
206521_s_at	8,468	8,576	8,295	8,523	8,791	8,753
206522_at	5,530	5,045	4,860	8,825	5,926	6,282
206523_at	5,757	5,796	6,205	6,697	6,744	6,852
206524_at	7,677	7,781	7,972	7,942	8,123	8,063
206525_at	5,347	5,432	5,542	4,862	4,648	4,646
206526_at	6,956	6,629	6,452	7,043	6,702	6,762
206527_at	7,212	7,361	7,257	7,610	7,405	7,841
206528_at	4,040	4,190	4,244	4,354	4,377	4,415
206529_x_at	4,666	5,249	5,060	4,890	5,503	5,139
206530_at	6,150	6,362	6,071	6,422	6,246	6,414
206531_at	7,339	7,322	7,255	7,726	7,465	7,756
206532_at	5,357	5,558	5,359	5,526	5,644	5,516
206533_at	6,698	6,724	6,507	5,558	5,618	5,555
206534_at	6,939	6,768	6,831	7,447	7,163	7,653
206535_at	4,161	3,766	4,081	4,128	4,056	4,213
206536_s_at	6,402	6,648	6,616	6,674	6,671	7,003
206537_at	6,146	6,413	6,265	6,390	6,447	6,567
206538_at	7,490	7,445	7,532	8,374	8,190	8,636
206539_s_at	7,743	7,377	7,456	7,841	7,890	7,899
206540_at	6,968	7,182	7,001	8,043	7,544	7,916
206541_at	8,378	8,765	8,836	7,424	7,352	7,475
206542_s_at	8,244	7,864	8,120	7,453	8,399	7,470
206543_at	3,642	3,809	3,629	4,136	4,276	4,413
206544_x_at	6,918	6,480	6,403	6,208	6,188	6,349
206545_at	3,971	3,900	4,031	4,234	4,004	4,112
206546_at	3,772	3,838	3,777	4,096	3,946	4,067
206547_s_at	5,165	4,976	4,921	5,100	5,211	5,329
206548_at	8,062	6,953	7,508	5,237	5,776	5,625
206549_at	6,004	5,923	6,236	6,274	6,524	6,456
206550_s_at	10,186	10,298	10,045	9,172	9,451	8,454
206551_x_at	8,896	8,904	8,962	7,954	8,986	8,327
206552_s_at	6,788	8,372	6,592	3,590	3,692	4,836
206553_at	6,391	6,083	6,105	6,983	6,800	6,995
206554_x_at	8,622	8,343	8,409	7,252	6,852	6,654
206555_s_at	11,367	11,558	10,772	9,194	10,181	8,443
206556_at	5,290	5,330	5,524	6,429	5,888	6,202
206557_at	6,221	6,827	5,912	4,067	5,405	4,310
206558_at	6,570	6,673	6,846	7,182	7,533	7,395
206559_x_at	14,964	14,959	14,985	14,268	14,583	14,602
206560_s_at	7,492	7,493	7,541	8,004	7,970	8,045
206561_s_at	6,300	6,361	6,736	7,738	8,231	8,243
206562_s_at	9,477	9,350	9,221	9,578	9,318	9,680
206563_s_at	6,922	6,355	6,154	7,916	7,679	8,022
206564_at	7,281	6,996	6,835	7,903	7,623	7,964
206565_x_at	7,216	6,260	6,935	6,817	7,055	7,010
206566_at	5,286	5,424	5,565	5,624	5,299	5,524
206567_s_at	9,492	9,720	9,774	7,973	8,508	7,956

206568_at	6,319	6,235	6,288	7,116	7,299	7,095
206569_at	5,922	5,922	5,997	7,752	6,657	7,266
206570_s_at	6,339	6,423	6,396	6,904	6,699	6,918
206571_s_at	10,314	9,749	10,243	10,821	11,443	11,018
206572_x_at	9,188	9,636	9,159	7,120	8,258	7,066
206573_at	6,428	6,252	6,685	7,578	7,149	7,475
206574_s_at	8,551	8,483	8,193	8,019	7,764	7,978
206575_at	6,362	6,213	6,556	7,298	7,259	7,411
206576_s_at	6,551	6,489	6,242	7,227	7,045	7,320
206577_at	3,792	3,915	4,230	3,867	3,754	3,854
206578_at	8,290	7,919	8,112	7,693	7,637	7,781
206579_at	4,436	4,306	4,322	4,500	4,371	4,653
206580_s_at	8,197	7,923	7,878	9,985	10,331	10,847
206581_at	6,026	5,877	5,908	7,487	6,877	6,920
206582_s_at	7,056	6,952	6,924	7,719	7,702	7,848
206583_at	9,358	9,496	9,317	7,426	7,556	7,281
206584_at	3,835	3,353	3,578	9,541	9,163	9,689
206585_at	5,805	5,551	5,458	6,812	6,848	6,472
206586_at	7,180	7,053	7,434	7,608	7,230	7,498
206587_at	8,054	8,621	8,146	6,240	6,827	6,668
206588_at	5,936	6,375	5,865	4,253	4,095	4,135
206589_at	6,302	6,096	6,210	6,762	6,508	6,626
206590_x_at	7,082	7,053	7,047	7,145	7,077	7,408
206591_at	7,254	7,517	6,551	6,256	6,338	6,155
206592_s_at	11,833	11,789	12,204	11,489	11,618	11,795
206593_s_at	9,606	9,230	9,891	7,761	7,281	7,826
206594_at	7,067	6,958	7,220	6,838	6,784	6,799
206595_at	6,614	5,692	6,429	7,169	7,815	7,981
206596_s_at	7,001	7,077	7,001	6,990	6,657	7,014
206597_at	6,183	6,181	6,128	6,689	6,444	6,722
206598_at	5,447	5,401	5,697	6,609	6,186	6,373
206599_at	7,870	8,451	8,047	7,669	7,310	7,815
206600_s_at	8,725	9,318	9,051	7,317	7,653	8,191
206601_s_at	5,716	5,127	5,390	5,407	5,250	5,456
206602_s_at	5,513	5,171	4,783	5,413	4,930	5,547
206603_at	5,203	5,200	5,286	5,832	5,612	5,996
206604_at	7,123	7,369	6,687	7,085	6,920	7,059
206605_at	5,569	5,887	5,940	6,464	6,337	6,378
206606_at	5,601	5,537	5,372	5,749	5,841	6,000
206607_at	6,076	6,188	6,339	6,604	6,342	6,577
206608_s_at	7,137	6,914	7,110	7,506	7,340	7,370
206609_at	6,025	5,766	5,788	6,301	6,430	6,407
206610_s_at	7,560	7,485	7,249	8,174	8,069	8,132
206611_at	7,508	7,471	7,497	7,533	7,381	7,471
206612_at	7,186	7,068	7,117	7,377	7,216	7,429
206613_s_at	10,072	10,345	9,550	6,364	5,896	5,157
206614_at	8,070	7,980	8,094	8,321	9,241	9,168
206615_s_at	4,401	4,431	4,468	4,144	4,132	4,030
206616_s_at	6,639	6,488	6,340	8,118	8,091	8,299
206617_s_at	6,895	6,505	6,591	7,103	6,324	6,723
206618_at	6,036	5,618	5,558	5,526	6,075	5,050
206619_at	6,438	6,522	6,709	7,900	7,502	7,733
206620_at	5,485	5,343	5,176	6,186	5,868	6,370
206621_s_at	12,502	12,110	12,231	11,662	11,762	11,966
206622_at	6,930	7,261	7,008	7,494	7,621	7,835
206623_at	6,362	6,290	6,390	7,329	7,283	7,432

206624_at	7,573	6,956	6,027	4,446	5,743	4,858
206625_at	6,449	6,584	6,465	7,051	6,978	6,987
206626_x_at	5,833	5,326	5,454	5,967	5,606	5,962
206627_s_at	4,155	4,089	3,917	4,692	4,397	4,785
206628_at	5,973	6,179	6,129	6,598	6,731	6,808
206629_at	6,429	6,430	6,405	6,083	5,929	6,190
206630_at	7,031	7,053	6,922	7,251	7,122	7,432
206631_at	5,309	6,008	6,327	8,523	10,240	10,718
206632_s_at	4,989	6,069	5,400	10,696	10,136	9,892
206633_at	5,044	5,202	5,432	5,398	5,318	5,596
206634_at	6,597	6,137	6,340	7,343	7,354	7,528
206635_at	8,172	8,181	8,605	8,590	8,750	8,836
206636_at	7,251	7,240	6,091	6,289	6,404	6,331
206637_at	3,671	3,503	3,858	3,831	3,919	3,981
206638_at	4,692	4,587	4,512	4,657	4,598	4,419
206639_x_at	4,625	4,471	4,641	4,636	4,541	4,706
206640_x_at	6,102	6,091	5,784	6,305	5,980	5,992
206641_at	4,178	4,745	4,375	4,597	4,293	4,605
206642_at	4,435	4,478	4,164	4,427	4,370	4,285
206643_at	5,457	5,285	5,581	5,276	5,341	5,056
206644_at	4,467	4,971	4,540	5,667	5,077	5,519
206645_s_at	6,394	6,801	6,808	7,705	7,243	7,849
206646_at	6,642	6,733	6,702	6,460	6,132	6,235
206647_at	6,835	6,568	6,529	6,824	6,588	6,861
206648_at	6,388	7,663	6,709	4,740	4,987	4,681
206649_s_at	8,302	8,225	8,363	8,384	7,740	8,372
206650_at	6,911	6,744	7,019	7,541	7,757	7,864
206651_s_at	4,583	4,609	4,450	4,873	4,636	4,583
206652_at	5,122	4,714	4,446	5,181	4,883	5,045
206653_at	11,001	11,412	11,002	8,330	8,927	7,642
206654_s_at	8,766	9,358	8,782	8,596	8,253	8,668
206655_s_at	6,513	6,932	6,698	7,135	7,402	7,381
206656_s_at	11,790	11,818	12,007	12,083	12,265	12,628
206657_s_at	7,735	7,419	8,004	8,137	8,070	8,105
206658_at	6,405	6,399	6,502	6,552	6,305	6,516
206659_at	6,536	6,078	6,196	6,764	6,520	6,766
206660_at	6,515	6,419	6,569	7,112	7,158	7,535
206661_at	5,840	5,422	5,433	5,923	5,738	6,051
206662_at	9,768	10,026	9,641	11,987	11,954	12,316
206663_at	7,346	7,516	7,295	6,183	5,856	5,722
206664_at	3,811	3,604	3,745	4,277	4,107	4,104
206665_s_at	6,794	6,539	6,900	7,911	7,936	8,090
206666_at	6,066	6,187	6,212	6,165	5,613	5,900
206667_s_at	5,987	6,364	5,632	6,289	6,145	6,485
206668_s_at	7,974	8,151	7,614	6,837	7,249	7,037
206669_at	6,816	6,989	7,598	7,067	6,995	7,057
206670_s_at	7,145	7,260	7,748	6,120	6,053	6,201
206671_at	7,135	6,510	6,982	7,247	6,967	7,044
206672_at	7,755	7,779	7,823	8,232	7,869	8,368
206673_at	5,716	5,783	5,950	6,426	6,161	6,474
206674_at	7,453	7,559	7,202	7,034	6,969	7,004
206675_s_at	6,592	6,937	6,527	5,800	5,962	6,142
206676_at	3,461	3,794	3,648	3,765	3,714	3,728
206677_at	5,637	5,497	5,551	6,389	6,228	6,486
206678_at	6,088	6,200	5,941	6,814	6,420	6,600
206679_at	5,677	5,733	5,573	6,175	5,930	5,760

206680_at	6,679	6,673	7,052	7,330	7,019	7,197
206681_x_at	6,312	6,056	6,280	7,170	6,836	7,105
206682_at	6,167	6,211	6,134	6,788	6,533	6,926
206683_at	6,633	8,419	7,045	4,399	4,388	4,618
206684_s_at	6,092	5,909	6,206	6,077	5,953	5,810
206685_at	5,154	5,307	5,204	5,912	5,724	5,852
206686_at	7,495	7,259	7,157	7,586	7,703	7,703
206687_s_at	8,208	8,264	8,204	8,509	8,610	8,885
206688_s_at	11,345	11,305	11,453	10,881	10,424	10,558
206689_x_at	8,782	8,729	9,028	8,263	7,595	8,059
206690_at	6,931	6,373	6,096	6,449	6,193	6,299
206691_s_at	6,430	6,227	6,368	7,004	6,962	7,167
206692_at	4,979	4,912	4,641	5,492	5,449	5,466
206693_at	3,678	4,440	4,003	5,842	5,066	4,788
206694_at	6,505	6,935	7,037	6,822	6,635	6,363
206695_x_at	8,561	9,453	8,158	6,288	6,692	6,348
206696_at	8,285	8,610	8,827	6,294	6,214	6,226
206697_s_at	6,745	6,241	6,796	8,077	7,916	8,031
206698_at	9,475	9,331	9,183	4,299	4,446	4,524
206699_x_at	7,720	7,599	7,569	7,808	7,953	8,202
206700_s_at	8,892	9,034	9,097	6,088	8,435	8,061
206701_x_at	4,492	4,685	5,273	6,651	6,307	6,639
206702_at	10,655	10,258	10,200	6,612	6,144	6,741
206703_at	6,147	6,133	5,959	6,060	5,801	6,002
206704_at	7,330	7,395	7,141	7,317	7,073	6,809
206705_at	6,481	6,495	6,394	6,835	6,302	6,608
206706_at	8,720	8,545	8,455	8,413	8,675	8,555
206707_x_at	6,718	6,619	6,877	7,323	7,336	7,554
206708_at	5,907	6,294	5,900	6,042	6,097	6,071
206709_x_at	7,902	8,140	7,950	7,765	7,657	8,108
206710_s_at	8,054	8,476	7,651	4,357	6,504	4,324
206711_at	5,564	5,505	5,670	6,016	6,324	5,944
206712_at	7,262	7,550	7,531	6,994	6,978	6,878
206713_at	5,427	5,091	4,886	5,598	5,501	5,723
206714_at	6,519	6,119	6,417	6,718	6,287	6,593
206715_at	4,641	4,789	4,535	5,283	5,277	5,279
206716_at	6,769	6,747	6,744	6,729	7,232	7,045
206717_at	6,259	6,333	6,401	6,825	6,479	6,789
206718_at	6,892	6,534	6,359	7,097	6,679	6,705
206719_at	6,595	6,560	6,288	6,838	6,504	6,839
206720_at	6,716	6,963	7,072	7,445	7,060	7,449
206721_at	8,704	9,060	8,435	5,785	6,230	5,813
206722_s_at	7,555	7,360	7,530	7,631	7,495	7,568
206723_s_at	8,972	8,971	9,314	7,287	7,137	7,268
206724_at	5,748	5,160	5,299	5,709	5,759	5,753
206725_x_at	7,742	7,783	7,955	8,094	7,723	8,413
206726_at	6,222	6,391	6,204	6,946	6,786	6,581
206727_at	4,122	4,313	3,895	4,530	4,334	4,402
206728_at	7,490	7,291	7,530	7,440	7,314	7,337
206729_at	7,465	7,793	8,002	7,520	7,307	7,541
206730_at	5,559	5,838	5,788	6,677	6,806	7,023
206731_at	5,057	5,306	5,085	5,193	5,061	5,069
206732_at	5,188	4,707	5,206	5,671	5,610	5,300
206733_at	5,497	5,626	5,385	5,687	5,812	5,910
206734_at	6,872	6,262	5,507	7,213	7,885	6,803
206735_at	7,290	7,516	7,628	8,466	8,415	8,278

206736_x_at	6,517	6,131	6,263	6,294	6,557	6,611
206737_at	6,262	6,427	6,500	7,202	6,986	6,888
206738_at	7,629	7,835	7,580	8,351	8,206	8,257
206739_at	7,235	7,085	7,314	8,296	8,055	8,397
206740_x_at	5,501	5,684	5,529	5,696	5,511	5,785
206741_at	6,405	6,061	6,453	6,758	6,670	6,812
206742_at	5,325	5,442	5,526	6,002	5,988	6,044
206743_s_at	7,118	7,034	6,873	6,755	6,592	6,980
206744_s_at	4,448	5,070	4,729	4,667	4,953	4,830
206745_at	7,374	7,484	7,500	8,327	7,598	8,086
206746_at	8,064	8,269	8,297	7,760	7,318	7,711
206747_at	8,373	7,740	8,189	7,597	7,655	7,592
206748_s_at	6,331	6,729	6,619	7,571	7,805	7,264
206749_at	6,128	6,143	6,160	6,502	6,628	6,410
206750_at	6,873	6,744	6,659	7,628	7,530	7,891
206751_s_at	7,188	5,895	7,416	5,455	5,528	5,683
206752_s_at	7,254	7,505	7,875	7,720	7,068	7,362
206753_at	8,039	7,454	7,604	8,657	7,925	8,282
206754_s_at	7,069	6,737	7,230	7,646	7,624	7,561
206755_at	6,357	6,305	6,335	7,226	7,727	7,551
206756_at	9,160	9,588	10,111	8,952	8,461	9,575
206757_at	4,525	4,564	4,206	5,061	4,840	4,680
206758_at	6,842	6,613	6,917	7,656	7,819	7,962
206759_at	6,960	7,094	6,742	7,136	7,098	7,165
206760_s_at	6,853	6,676	6,806	7,487	7,444	7,825
206761_at	6,235	6,400	6,466	6,621	6,722	6,797
206762_at	9,406	9,103	9,346	7,261	7,289	7,338
206763_at	7,265	7,243	7,570	7,771	7,652	7,795
206764_x_at	8,860	8,436	8,907	9,007	9,373	9,520
206765_at	6,032	6,263	5,257	8,104	7,174	6,880
206766_at	6,838	6,622	6,593	8,649	9,280	9,747
206767_at	6,714	6,543	6,804	7,286	6,930	6,787
206768_at	6,898	6,530	6,768	7,324	6,914	7,067
206769_at	7,716	7,960	7,794	8,946	8,884	9,137
206770_s_at	8,677	8,809	8,261	8,618	9,795	9,911
206771_at	6,476	6,373	6,288	7,026	7,060	7,176
206772_at	4,939	5,209	5,172	5,366	5,378	5,393
206773_at	6,155	5,924	5,971	6,120	6,423	6,212
206774_at	6,385	6,139	6,517	6,660	6,654	6,695
206775_at	6,064	5,894	6,124	6,118	5,969	6,134
206776_x_at	6,098	6,180	6,471	6,372	6,194	6,417
206777_s_at	6,488	6,240	6,174	6,455	6,602	6,467
206778_at	7,527	7,315	6,993	7,990	8,056	8,083
206779_s_at	5,119	5,436	5,109	5,921	5,392	5,647
206780_at	5,814	6,241	6,503	6,619	6,553	6,502
206781_at	6,347	6,658	6,469	7,251	7,305	7,229
206782_s_at	7,007	7,019	6,869	6,932	7,074	7,205
206783_at	9,999	10,182	10,630	7,023	6,946	7,131
206784_at	6,444	6,297	6,088	6,910	6,719	7,153
206785_s_at	4,550	4,554	4,545	5,517	5,226	5,743
206786_at	4,063	4,333	4,551	4,531	4,363	4,379
206787_at	4,911	4,747	4,867	5,414	5,630	5,203
206788_s_at	8,515	8,026	8,076	9,173	8,559	8,978
206789_s_at	10,036	9,703	9,874	7,544	7,521	7,857
206790_s_at	12,711	12,776	12,598	12,872	12,531	12,988
206791_s_at	8,158	7,928	7,790	7,559	7,195	7,595

206792_x_at	11,081	10,678	10,843	9,861	11,038	10,270
206793_at	7,717	7,632	7,834	7,951	7,696	8,034
206794_at	6,327	6,286	5,984	6,402	6,201	6,356
206795_at	5,050	4,917	5,527	5,570	5,736	5,675
206796_at	5,643	5,766	5,533	6,564	6,003	6,295
206797_at	6,169	6,403	6,441	6,377	6,276	6,119
206798_x_at	8,200	7,697	7,860	8,513	8,056	8,026
206799_at	5,673	5,781	5,380	5,676	5,424	5,662
206800_at	6,812	6,656	6,702	7,224	6,729	6,885
206801_at	7,506	6,472	7,351	7,965	7,954	8,045
206802_at	7,295	7,374	7,455	7,698	7,643	7,428
206803_at	6,281	6,754	6,315	5,862	5,859	6,016
206804_at	6,694	6,599	6,744	6,565	6,404	6,715
206805_at	9,450	9,202	8,328	6,382	7,289	6,719
206806_at	6,732	5,761	6,179	5,518	5,653	6,095
206807_s_at	8,030	8,066	8,126	8,131	8,408	8,109
206808_at	6,187	6,197	6,192	5,598	5,714	5,647
206809_s_at	11,238	10,946	11,007	9,635	9,838	9,024
206810_at	5,072	4,986	4,954	6,208	6,254	6,130
206811_at	5,646	5,437	5,496	6,641	6,091	6,544
206812_at	6,971	7,055	6,993	7,352	7,148	7,141
206813_at	6,381	6,171	6,403	7,258	6,957	6,913
206814_at	6,675	7,004	6,875	7,252	6,547	6,597
206815_at	5,833	5,936	6,441	7,870	7,890	8,321
206816_s_at	6,733	6,888	6,852	8,344	8,175	8,344
206817_x_at	7,628	7,552	7,774	7,944	7,637	7,661
206818_s_at	7,568	8,292	7,967	6,776	6,438	6,532
206819_at	5,300	4,980	5,738	5,624	6,383	6,388
206820_at	6,703	6,665	6,727	7,151	6,813	6,836
206821_x_at	7,205	6,972	7,190	8,064	7,723	7,944
206822_s_at	6,239	6,680	6,547	6,489	6,241	6,516
206823_at	7,120	6,924	7,017	7,776	7,131	7,293
206824_at	6,968	6,902	6,781	7,700	8,194	8,131
206825_at	6,078	6,533	6,415	6,224	6,518	6,169
206826_at	4,071	3,924	3,962	4,772	4,127	4,899
206827_s_at	7,058	6,886	7,072	7,148	7,400	7,356
206828_at	4,975	5,116	5,134	5,059	5,024	5,076
206829_x_at	9,005	9,199	8,418	7,262	8,025	6,977
206830_at	3,901	3,741	3,733	3,715	3,587	3,766
206831_s_at	6,033	5,802	6,329	6,662	6,529	6,674
206832_s_at	6,047	6,139	5,511	6,032	5,788	6,162
206833_s_at	7,393	7,738	7,187	8,065	7,735	8,346
206834_at	6,338	6,353	6,249	6,980	6,854	7,118
206835_at	3,977	4,369	4,314	4,524	4,349	4,848
206836_at	7,642	7,661	7,876	7,858	7,492	7,639
206837_at	9,051	9,614	9,198	7,618	7,478	8,609
206838_at	7,129	7,249	7,370	7,402	7,571	7,602
206839_at	7,091	7,571	7,182	7,750	7,811	7,827
206840_at	4,510	4,609	4,930	4,903	4,680	4,739
206841_at	4,254	4,299	4,149	4,231	4,103	4,376
206842_at	7,402	7,077	7,342	7,388	7,224	7,367
206843_at	6,309	6,597	5,963	6,515	6,571	6,622
206844_at	6,776	6,616	6,925	7,230	6,875	7,331
206845_s_at	8,826	8,404	8,996	8,761	8,443	8,840
206846_s_at	9,249	9,186	8,750	8,842	8,249	8,543
206847_s_at	5,980	6,243	5,906	8,223	7,626	7,489

206848_at	9,421	8,802	8,709	5,975	6,404	5,838
206849_at	6,490	6,716	6,511	5,933	5,884	6,029
206850_at	7,665	7,609	7,685	7,592	7,268	7,470
206851_at	7,817	7,856	7,929	7,750	7,723	7,812
206852_at	3,609	3,805	3,757	4,002	3,959	4,260
206853_s_at	9,461	9,542	9,258	7,645	8,702	7,284
206854_s_at	10,188	10,184	9,614	8,479	9,259	8,489
206855_s_at	9,301	9,176	9,338	8,812	8,188	8,733
206856_at	6,366	6,700	6,640	7,087	7,148	7,493
206857_s_at	11,718	12,276	11,977	10,014	9,498	10,051
206858_s_at	5,968	6,440	5,552	10,614	10,229	10,466
206859_s_at	5,457	5,311	4,850	5,687	5,443	5,724
206860_s_at	10,919	11,196	10,673	9,802	11,242	10,170
206861_s_at	9,148	9,442	9,323	9,981	8,857	9,228
206862_at	7,083	7,869	7,685	5,279	5,804	5,164
206863_x_at	5,759	5,741	6,123	6,857	6,555	6,947
206864_s_at	6,569	6,835	6,786	6,818	6,793	7,024
206865_at	6,216	6,196	5,938	6,758	6,485	6,722
206866_at	7,217	7,324	7,172	6,827	6,840	6,943
206867_at	6,506	6,540	6,703	6,664	6,614	6,657
206868_at	7,109	7,641	7,669	7,459	7,165	7,225
206869_at	7,048	6,721	6,838	7,469	7,206	7,315
206870_at	5,103	5,387	5,229	6,210	6,206	6,086
206871_at	5,887	5,556	5,898	6,106	6,225	6,305
206872_at	6,700	6,316	6,616	7,507	6,972	7,121
206873_at	6,699	6,509	6,588	6,794	6,674	6,705
206874_s_at	9,923	10,405	9,723	8,840	10,174	8,369
206875_s_at	9,624	10,037	9,332	9,403	10,703	8,562
206876_at	5,843	6,084	5,924	6,020	5,932	5,776
206877_at	6,104	5,704	6,057	6,610	6,708	6,954
206878_at	6,599	6,509	6,434	7,081	7,168	7,101
206879_s_at	6,196	6,049	6,156	6,366	6,790	6,707
206880_at	7,350	7,120	7,332	7,737	7,739	7,675
206881_s_at	6,600	6,695	6,799	7,764	7,757	7,801
206882_at	6,654	6,123	6,475	6,744	6,427	6,974
206883_x_at	7,504	7,435	7,521	7,681	7,668	7,870
206884_s_at	4,548	4,430	4,382	4,567	4,364	4,383
206885_x_at	7,042	7,067	7,143	6,949	6,879	6,950
206886_x_at	6,528	6,525	6,316	6,627	6,094	6,659
206887_at	6,774	6,846	6,974	7,650	7,301	7,581
206888_s_at	7,391	6,837	7,390	7,480	7,264	7,589
206889_at	7,394	7,456	7,359	7,548	7,607	7,478
206890_at	7,320	7,430	7,447	8,694	8,452	8,648
206891_at	6,687	6,909	6,926	7,652	7,132	7,069
206892_at	7,122	6,950	6,966	7,396	7,339	7,507
206893_at	9,321	9,130	9,491	5,499	4,072	4,640
206894_at	7,167	7,109	7,445	7,817	8,144	8,032
206895_at	4,414	4,463	4,358	4,403	4,481	4,442
206896_s_at	7,740	7,847	7,717	7,527	7,476	7,486
206897_at	6,015	5,961	5,671	6,420	6,352	6,482
206898_at	4,258	4,265	3,889	4,701	4,330	4,337
206899_at	6,239	6,348	6,388	6,721	6,352	6,916
206900_x_at	7,609	7,232	6,950	6,278	5,941	5,788
206901_at	7,948	7,745	7,842	8,180	7,646	7,858
206902_s_at	6,873	6,710	6,883	7,004	6,847	6,898
206903_at	7,398	7,142	7,308	8,034	7,802	8,006

206904_at	6,667	6,527	6,514	7,000	7,038	7,292
206905_s_at	6,295	6,669	6,740	7,107	7,128	7,032
206906_at	6,226	5,745	5,911	6,298	5,786	6,117
206907_at	6,813	7,413	6,987	6,715	6,719	6,543
206908_s_at	7,178	6,926	7,050	7,706	6,970	7,485
206909_at	4,705	4,600	4,849	4,763	4,595	4,692
206910_x_at	5,849	5,502	6,178	6,344	6,653	6,300
206911_at	7,508	7,167	7,366	8,039	8,081	8,265
206912_at	4,956	4,829	5,186	4,932	4,928	4,875
206913_at	6,778	6,476	6,551	6,893	7,152	6,906
206914_at	4,022	4,052	4,690	4,507	4,748	4,594
206915_at	4,553	4,304	4,472	4,650	4,591	4,666
206916_x_at	7,152	6,656	7,083	7,143	7,075	7,175
206917_at	6,011	5,778	5,872	6,472	6,442	6,324
206918_s_at	9,663	9,695	9,937	9,927	9,486	10,136
206919_at	5,190	5,750	5,543	5,561	5,471	5,188
206920_s_at	7,653	7,674	7,590	7,562	7,402	7,535
206921_at	5,864	6,168	6,128	6,186	5,770	6,123
206922_at	7,372	7,209	7,402	7,674	7,476	7,497
206923_at	5,889	5,651	5,760	6,568	6,502	6,716
206924_at	6,528	6,941	6,567	6,962	6,986	6,899
206925_at	4,583	4,903	4,688	5,024	5,248	5,292
206926_s_at	8,011	7,841	7,993	8,407	8,115	8,045
206927_s_at	5,869	6,387	6,085	7,010	7,114	7,123
206928_at	9,237	9,066	9,008	6,498	6,265	6,105
206929_s_at	7,375	7,182	7,102	8,966	9,080	9,286
206930_at	5,285	5,402	5,551	5,621	5,572	5,711
206931_at	6,838	6,586	6,362	4,178	4,317	4,117
206932_at	5,146	5,526	5,254	5,787	6,872	6,517
206933_s_at	6,956	6,643	6,706	7,157	7,200	7,168
206934_at	5,870	5,963	6,181	6,730	6,261	6,472
206935_at	9,005	9,680	10,037	3,653	3,504	3,455
206936_x_at	7,939	8,451	8,481	8,039	8,356	8,517
206937_at	5,056	5,205	4,863	5,385	5,211	5,340
206938_at	6,347	6,307	6,210	6,453	6,523	6,380
206939_at	5,452	5,625	5,944	6,118	6,060	6,076
206940_s_at	5,407	5,173	5,584	6,168	5,842	6,112
206941_x_at	3,796	3,861	3,900	3,829	3,886	3,805
206942_s_at	6,504	6,910	6,605	6,635	6,448	6,839
206943_at	5,982	6,254	6,014	6,325	6,000	6,220
206944_at	7,573	7,240	7,511	8,119	8,097	8,073
206945_at	6,037	5,923	5,708	6,512	6,621	6,706
206946_at	6,579	6,598	6,583	6,771	6,452	6,731
206947_at	5,670	5,592	5,818	5,896	5,592	5,590
206948_at	6,520	6,524	6,434	6,782	6,587	6,634
206949_s_at	10,690	10,451	10,898	10,073	9,517	10,064
206950_at	5,187	4,989	5,237	4,799	5,924	6,107
206951_at	6,924	6,924	6,865	7,120	6,702	6,736
206952_at	3,784	3,808	3,704	3,988	3,842	3,984
206953_s_at	11,541	11,679	11,585	8,736	10,428	8,658
206954_at	6,146	6,367	5,954	6,963	6,491	6,458
206955_at	7,155	7,004	7,386	7,840	8,317	8,339
206956_at	9,012	9,056	8,775	7,130	7,269	7,516
206957_at	4,918	5,035	5,080	5,222	4,907	4,851
206958_s_at	11,242	11,217	10,526	8,867	9,188	8,857
206959_s_at	8,831	8,774	7,984	7,365	7,316	7,299

206960_at	8,686	8,714	7,993	4,630	5,237	5,089
206961_s_at	6,601	6,448	6,506	6,996	7,204	7,100
206962_x_at	5,967	6,419	5,922	5,950	5,766	5,891
206963_s_at	6,937	6,578	6,579	6,880	6,953	7,080
206964_at	6,451	6,823	6,506	6,980	7,219	7,155
206965_at	5,604	4,997	5,602	5,155	6,677	5,514
206966_s_at	7,343	7,037	7,104	7,652	8,030	7,840
206967_at	9,180	9,096	9,221	7,724	7,851	7,764
206968_s_at	7,617	7,559	7,681	7,413	7,529	7,585
206969_at	5,894	6,089	6,088	10,819	8,043	10,188
206970_at	7,099	7,049	7,496	7,763	7,720	7,506
206971_at	7,319	6,990	7,090	7,594	7,373	7,424
206972_s_at	7,584	7,325	7,317	7,293	7,324	7,267
206973_at	7,139	7,246	6,717	7,338	7,264	7,227
206974_at	5,994	6,157	6,106	7,066	7,012	7,137
206975_at	6,480	6,505	6,281	7,047	7,045	6,922
206976_s_at	12,393	12,132	12,021	11,252	11,935	10,456
206977_at	3,751	3,649	3,805	4,091	3,897	4,046
206978_at	5,197	5,168	5,738	6,245	5,859	6,126
206979_at	6,753	6,887	6,544	7,325	7,049	7,318
206980_s_at	6,683	7,122	7,005	7,910	8,089	8,153
206981_at	6,713	6,615	6,584	6,586	6,400	6,498
206982_at	6,961	6,803	5,450	5,423	5,260	5,410
206983_at	4,940	4,323	4,844	4,916	4,871	4,898
206984_s_at	5,049	6,513	5,529	4,331	4,216	4,167
206985_at	6,232	5,983	6,098	6,262	6,015	6,158
206986_at	6,579	6,043	6,629	7,197	7,172	7,285
206987_x_at	6,634	6,528	6,628	6,317	6,598	6,618
206988_at	6,088	5,968	6,327	6,812	6,276	6,759
206989_s_at	8,944	9,133	8,815	7,961	9,141	7,429
206990_at	5,649	5,350	5,643	6,267	5,899	5,976
206991_s_at	7,694	7,599	7,601	8,421	8,482	8,312
206992_s_at	8,742	8,744	8,519	8,870	7,766	8,306
206993_at	7,779	7,591	7,859	8,743	7,641	8,167
206994_at	7,097	6,667	7,051	7,722	7,595	7,793
206995_x_at	4,087	4,037	3,962	4,192	4,108	4,442
206996_x_at	6,413	5,820	6,798	7,461	7,248	7,522
206997_s_at	7,160	7,019	7,228	7,578	7,788	7,663
206998_x_at	8,031	7,892	7,941	8,351	8,287	8,413
206999_at	5,989	6,097	6,429	6,018	6,277	6,086
207000_s_at	7,815	8,106	7,040	8,887	8,806	8,790
207001_x_at	6,729	6,605	6,694	6,737	6,729	6,922
207002_s_at	8,591	7,085	7,163	9,027	10,156	9,792
207003_at	7,191	7,157	7,361	7,693	7,665	7,679
207004_at	6,523	6,761	6,760	7,431	7,649	7,503
207005_s_at	5,692	5,730	5,977	6,367	6,139	6,220
207006_s_at	7,504	7,837	7,974	7,773	7,781	7,776
207007_at	5,965	5,898	6,210	6,486	6,423	6,585
207008_at	4,235	4,334	4,884	5,241	4,902	5,162
207009_at	5,867	5,715	5,988	6,285	6,177	6,380
207010_at	4,458	4,849	4,806	4,847	4,850	4,729
207011_s_at	7,124	7,203	7,254	7,497	7,587	7,557
207012_at	6,438	6,329	5,992	5,537	6,362	5,942
207013_s_at	6,307	6,218	6,284	7,071	7,217	7,378
207014_at	4,991	5,134	4,535	5,439	5,176	5,199
207015_s_at	7,413	7,411	7,353	7,293	7,163	7,475

207016_s_at	6,021	5,974	6,298	5,716	5,777	5,725
207017_at	4,806	4,806	5,060	6,571	6,258	6,519
207018_s_at	4,017	3,974	3,867	6,409	5,281	4,907
207019_s_at	6,477	6,678	6,121	7,548	7,330	7,320
207020_at	7,550	7,643	7,735	7,256	7,299	7,582
207021_at	5,518	5,812	5,348	6,239	6,257	6,139
207022_s_at	6,519	6,485	6,501	7,128	6,963	7,034
207023_x_at	10,067	9,915	10,325	11,555	11,747	11,991
207024_at	6,294	6,035	5,926	6,661	6,296	6,685
207025_at	8,130	8,023	8,033	8,590	8,509	8,380
207026_s_at	6,654	6,496	6,550	6,983	6,824	6,831
207027_at	6,495	6,393	6,756	7,338	6,879	7,333
207028_at	6,623	6,797	6,907	7,209	6,669	6,803
207029_at	4,414	3,952	4,041	4,740	4,569	5,022
207030_s_at	13,145	13,266	13,111	9,745	8,882	10,071
207031_at	8,403	8,999	8,441	6,884	6,556	6,945
207032_s_at	5,152	5,120	5,031	5,786	5,531	5,659
207033_at	4,540	4,799	4,676	5,756	5,347	5,574
207034_s_at	9,139	8,519	9,597	6,245	5,507	5,668
207035_at	9,166	9,119	9,034	8,909	8,942	9,038
207036_x_at	6,783	6,626	6,618	6,533	6,276	6,373
207037_at	6,452	6,563	7,378	5,810	5,640	5,526
207038_at	5,865	5,809	5,706	9,416	8,440	8,002
207039_at	5,417	5,565	5,175	6,951	6,841	7,606
207040_s_at	11,901	12,263	12,083	11,812	11,867	11,811
207041_at	6,497	6,710	6,827	7,505	7,533	7,561
207042_at	6,727	6,331	6,765	6,905	6,518	6,735
207043_s_at	5,774	5,260	5,118	6,292	5,979	6,151
207044_at	6,549	6,813	6,854	7,559	7,200	7,687
207045_at	5,274	4,960	5,390	4,780	4,981	4,946
207046_at	6,225	6,098	6,475	5,880	5,884	6,084
207047_s_at	6,169	5,720	5,894	6,789	6,812	6,679
207048_at	7,382	7,360	7,729	8,454	8,626	8,814
207049_at	6,923	6,997	6,635	6,822	7,110	7,205
207050_at	7,046	7,170	7,288	7,658	7,800	7,678
207051_at	6,484	6,635	6,682	7,495	7,137	7,156
207052_at	4,873	4,488	4,351	5,234	4,819	4,991
207053_at	4,264	3,784	4,233	4,130	4,063	4,319
207054_at	4,078	4,141	4,336	4,516	4,655	4,597
207055_at	6,907	6,704	6,992	7,653	7,675	7,874
207056_s_at	6,473	6,995	6,699	4,990	4,691	4,821
207057_at	6,347	6,322	6,000	8,433	9,549	7,835
207058_s_at	5,330	4,713	5,106	5,705	5,536	5,590
207059_at	7,150	7,094	6,993	7,345	7,340	7,671
207060_at	5,956	5,487	5,755	6,126	6,339	5,958
207061_at	7,723	7,192	7,556	7,904	7,843	8,089
207062_at	5,661	6,458	5,700	6,079	6,165	5,966
207063_at	7,157	6,692	7,155	6,956	7,616	7,403
207064_s_at	6,225	6,201	6,498	7,180	6,568	6,782
207065_at	6,823	6,912	6,899	7,308	7,077	7,244
207066_at	7,044	7,542	6,996	7,311	7,396	7,341
207067_s_at	6,679	6,756	6,556	7,799	7,640	7,423
207068_at	8,606	9,092	8,410	6,383	6,485	6,667
207069_s_at	8,441	8,719	8,796	7,044	7,435	7,575
207070_at	6,971	6,443	6,327	7,642	7,333	7,483
207071_s_at	9,842	10,257	10,027	10,789	10,692	10,709

207072_at	6,194	5,943	6,109	6,729	6,585	6,596
207073_at	5,820	6,267	5,877	6,601	6,368	6,832
207074_s_at	5,297	5,645	5,268	6,667	6,113	6,186
207075_at	5,048	5,235	5,324	5,685	5,393	5,749
207076_s_at	9,602	10,231	10,316	10,621	10,619	11,641
207077_at	5,419	5,552	5,693	6,242	6,249	6,255
207078_at	7,784	8,004	8,104	4,905	6,960	5,459
207079_s_at	8,385	8,510	8,113	6,800	6,806	6,692
207080_s_at	5,622	5,879	5,581	6,183	6,436	6,429
207081_s_at	8,581	8,350	8,599	8,266	8,011	8,313
207082_at	6,650	6,690	6,700	7,539	7,303	7,422
207083_s_at	5,522	5,755	5,572	6,369	6,240	6,160
207084_at	6,214	6,086	5,861	6,911	6,949	7,236
207085_x_at	5,652	5,492	5,479	5,836	5,268	5,489
207086_x_at	6,784	7,110	6,766	6,963	6,931	7,000
207087_x_at	6,869	6,885	7,033	7,760	7,646	7,769
207088_s_at	8,581	8,416	8,540	8,948	8,108	8,976
207089_at	4,794	4,464	5,008	5,946	5,990	5,887
207090_x_at	7,170	7,206	7,230	6,601	6,411	6,522
207091_at	5,549	5,544	5,727	5,932	5,815	5,806
207092_at	6,142	6,082	6,324	6,716	6,643	6,972
207093_s_at	7,272	7,153	7,352	7,970	7,958	8,377
207094_at	8,248	8,292	8,244	9,073	8,718	8,894
207095_at	5,557	6,168	5,606	5,707	5,400	5,703
207096_at	7,451	7,204	7,625	8,155	7,898	8,312
207097_s_at	5,960	6,214	5,602	6,327	6,186	6,135
207098_s_at	9,556	10,069	9,279	9,148	9,957	8,744
207099_s_at	5,143	5,090	5,112	5,553	5,480	5,546
207100_s_at	8,764	9,084	8,725	7,432	7,553	7,879
207101_at	7,454	7,701	7,729	7,687	7,852	8,196
207102_at	3,905	3,791	4,033	3,919	3,950	4,176
207103_at	6,886	6,826	6,958	5,540	5,633	5,510
207104_x_at	7,239	6,950	6,793	8,105	8,175	8,326
207105_s_at	7,394	7,173	7,227	7,006	7,120	7,024
207106_s_at	6,038	6,182	6,282	6,270	6,232	5,922
207107_at	3,859	3,926	3,990	3,923	3,661	3,723
207108_s_at	7,399	7,313	6,883	6,397	6,530	5,752
207109_at	4,434	4,453	4,575	4,063	4,048	3,970
207110_at	6,398	6,258	6,157	5,269	4,963	5,084
207111_at	6,956	6,821	6,905	8,352	7,700	7,790
207112_s_at	5,546	5,637	5,356	6,080	6,068	6,278
207113_s_at	6,741	6,506	6,501	7,106	6,935	7,194
207114_at	6,989	6,823	7,065	7,413	7,243	7,442
207115_x_at	6,098	5,787	5,837	5,406	5,929	5,600
207116_s_at	5,457	5,095	5,631	5,568	5,496	5,636
207117_at	4,655	4,987	4,868	3,939	3,770	3,823
207118_s_at	6,162	6,194	5,901	6,223	6,269	6,207
207119_at	4,790	4,990	4,769	4,473	4,101	4,415
207120_at	7,102	7,417	6,705	4,972	5,396	5,045
207121_s_at	11,479	11,656	11,159	11,024	12,460	10,999
207122_x_at	8,187	8,283	8,238	8,060	8,018	8,238
207123_s_at	7,351	7,219	7,282	7,311	7,113	7,501
207124_s_at	8,031	8,165	8,225	8,107	7,297	8,003
207125_at	6,778	6,747	5,347	5,077	5,481	5,646
207126_x_at	7,704	7,792	7,909	7,775	8,063	7,988
207127_s_at	11,008	10,873	10,257	9,461	9,693	8,991

207128_s_at	7,820	7,675	7,344	5,953	5,751	5,832
207129_at	6,713	6,569	6,890	6,619	6,284	6,363
207130_at	7,186	7,132	7,153	6,100	6,033	6,123
207131_x_at	8,159	7,987	8,339	7,862	7,472	7,703
207132_x_at	10,995	11,014	10,817	10,653	10,051	10,837
207133_x_at	8,689	8,526	8,517	8,357	8,873	8,462
207134_x_at	6,821	6,856	6,850	6,848	7,155	6,895
207135_at	6,092	6,204	6,434	6,316	6,232	6,372
207136_at	7,878	7,699	7,811	8,329	8,382	8,631
207137_at	6,406	6,645	6,954	7,422	7,334	7,446
207138_at	7,294	6,993	6,900	7,745	7,444	7,591
207139_at	7,420	7,151	7,599	7,753	7,890	7,784
207140_at	6,766	6,223	6,426	7,316	6,960	6,930
207141_s_at	6,268	6,537	6,678	6,959	6,692	6,863
207142_at	4,690	4,526	4,493	5,164	4,987	4,777
207143_at	7,740	7,263	7,551	8,555	8,093	8,023
207144_s_at	7,106	7,131	7,329	6,674	7,075	6,982
207145_at	3,721	3,697	3,851	3,600	3,687	3,626
207146_at	6,776	7,126	7,048	7,067	7,284	7,062
207147_at	5,542	5,739	5,496	7,863	8,998	8,318
207148_x_at	4,780	5,252	5,032	5,368	5,685	5,548
207149_at	4,066	3,902	4,206	5,087	4,860	4,989
207150_at	6,123	5,942	6,051	6,918	7,169	7,024
207151_at	6,418	6,553	6,557	6,864	6,629	6,981
207152_at	7,363	7,469	7,131	7,755	7,388	7,583
207153_s_at	10,089	10,065	9,681	8,735	9,080	8,498
207154_at	7,053	7,163	7,070	7,267	7,272	7,123
207155_at	5,112	5,288	5,649	6,386	6,430	6,211
207156_at	6,441	6,800	7,242	5,504	5,482	5,741
207157_s_at	12,705	12,763	12,999	12,582	12,305	12,772
207158_at	6,542	6,590	6,546	6,852	6,923	7,581
207159_x_at	8,328	8,244	8,222	8,415	8,320	8,524
207160_at	5,396	5,723	5,419	6,175	6,241	6,110
207161_at	4,060	4,108	4,119	4,438	4,157	4,317
207162_s_at	6,594	6,888	6,809	6,822	6,615	6,966
207163_s_at	8,797	8,698	8,534	9,066	8,203	8,999
207164_s_at	4,586	4,691	4,566	4,626	4,585	4,649
207165_at	12,628	13,073	12,065	10,887	11,566	9,424
207166_at	4,305	4,566	4,576	4,904	4,743	4,814
207167_at	5,706	5,667	5,717	6,252	5,950	6,313
207168_s_at	12,319	12,038	12,515	12,185	11,804	11,971
207169_x_at	8,928	8,441	8,781	7,974	8,143	8,158
207170_s_at	9,839	10,403	9,966	9,195	9,434	9,629
207172_s_at	7,138	7,048	6,814	7,559	7,485	8,106
207173_x_at	8,999	8,363	8,682	7,843	10,797	10,790
207174_at	4,734	4,815	4,494	4,987	4,938	5,019
207175_at	4,258	4,389	4,560	4,588	4,539	4,605
207176_s_at	6,331	6,241	6,236	6,589	6,214	6,605
207177_at	6,027	6,094	4,572	7,362	7,410	6,579
207178_s_at	4,136	4,001	4,252	3,904	3,778	3,988
207179_at	6,643	6,676	6,947	7,390	7,457	7,671
207180_s_at	9,588	10,189	9,812	10,260	9,536	10,144
207181_s_at	8,402	8,391	7,980	9,315	9,583	9,218
207182_at	4,333	4,236	4,434	4,657	4,607	4,505
207183_at	10,615	10,650	10,745	5,833	5,258	5,562
207184_at	7,345	6,902	7,100	7,541	6,951	6,895

207185_at	6,398	6,509	6,400	6,497	6,486	6,474
207186_s_at	10,803	10,565	10,672	7,722	9,453	7,646
207187_at	7,161	7,258	7,474	7,738	7,638	7,697
207188_at	7,728	7,841	7,592	7,171	6,785	7,298
207189_s_at	7,205	7,195	7,177	7,511	7,749	7,620
207190_at	5,368	5,698	5,491	6,223	6,225	6,376
207191_s_at	6,079	6,132	5,999	7,015	5,696	6,503
207192_at	7,072	6,950	7,142	7,567	7,558	7,587
207193_at	7,314	7,171	7,298	7,855	7,761	7,728
207194_s_at	5,475	6,177	5,674	5,255	5,212	5,045
207195_at	4,414	4,211	4,143	4,270	4,431	4,387
207196_s_at	8,404	8,724	8,770	10,676	9,410	9,803
207197_at	10,408	10,403	10,348	5,993	5,762	5,776
207198_s_at	9,334	9,167	9,060	10,037	9,363	10,042
207199_at	7,799	7,226	7,674	8,186	8,093	8,125
207200_at	4,995	5,239	5,233	5,353	4,940	5,073
207201_s_at	5,445	5,472	6,101	6,346	6,531	6,580
207202_s_at	5,333	5,779	5,423	6,176	5,786	6,117
207203_s_at	7,520	7,163	7,614	8,315	7,776	7,686
207204_at	6,362	5,947	6,515	6,901	6,779	6,996
207205_at	6,808	6,499	6,700	7,032	6,598	6,692
207206_s_at	5,838	5,553	5,665	5,341	5,055	5,181
207207_at	6,178	6,119	5,708	5,923	5,751	5,954
207208_at	5,812	5,920	5,620	5,947	5,688	5,767
207209_at	7,406	7,178	7,421	7,909	7,608	7,661
207210_at	5,354	4,953	5,098	5,641	5,682	5,470
207211_at	6,579	6,347	6,261	6,618	6,598	6,649
207212_at	6,407	6,506	6,547	7,124	6,988	7,161
207213_s_at	6,507	6,183	6,212	6,192	5,971	6,197
207214_at	7,309	6,797	6,943	7,442	6,965	7,631
207215_at	7,968	7,781	7,651	8,403	8,407	8,325
207216_at	6,682	6,578	7,204	7,443	7,364	7,668
207217_s_at	7,152	7,194	7,641	7,958	7,670	7,889
207218_at	4,662	5,202	5,129	5,232	4,764	5,185
207219_at	7,721	7,845	7,712	5,141	4,996	5,006
207220_at	5,024	5,532	5,141	5,677	5,698	5,673
207221_at	5,806	6,198	6,359	6,652	6,478	6,630
207222_at	7,576	7,563	7,596	8,327	7,933	8,159
207223_s_at	6,719	6,936	6,707	6,482	5,970	6,460
207224_s_at	5,549	5,571	5,560	5,527	5,690	5,653
207225_at	5,574	5,508	5,552	6,418	6,447	6,666
207226_at	5,785	5,752	5,735	6,873	6,813	7,161
207227_x_at	6,760	6,674	6,847	6,872	6,702	6,448
207228_at	7,472	7,350	7,450	7,919	7,882	7,852
207229_at	5,959	4,977	4,743	4,100	3,812	3,800
207230_at	7,275	6,971	6,869	7,438	7,535	7,666
207231_at	8,067	8,088	7,537	4,709	6,072	4,655
207232_s_at	8,735	9,097	8,078	6,563	6,801	6,571
207233_s_at	6,931	7,336	7,249	7,958	8,973	8,132
207234_at	6,316	6,454	6,638	6,207	6,053	6,255
207235_s_at	5,373	5,148	5,390	5,501	5,356	5,146
207236_at	5,305	5,091	4,729	4,187	4,077	3,866
207237_at	6,205	6,263	6,210	6,437	6,617	6,395
207238_s_at	5,029	5,222	5,168	5,478	5,351	5,539
207239_s_at	8,774	8,595	8,543	8,160	6,873	7,851
207240_s_at	4,573	4,920	4,807	4,875	5,012	4,829

207241_at	5,072	5,131	4,851	5,936	5,536	5,645
207242_s_at	6,194	6,145	6,135	6,544	6,799	6,717
207243_s_at	14,226	14,417	14,194	13,440	13,940	13,890
207244_x_at	8,063	8,005	8,210	8,268	8,414	8,410
207245_at	3,888	4,195	3,889	3,928	3,824	3,847
207246_at	4,600	4,745	4,687	4,936	4,704	4,798
207247_s_at	5,533	5,403	5,324	5,222	5,029	4,554
207248_at	7,383	7,391	7,281	7,645	7,816	7,822
207249_s_at	6,377	6,026	6,417	6,358	6,485	6,352
207250_at	6,105	5,673	5,633	5,879	5,572	5,550
207251_at	5,627	5,554	5,636	6,700	6,294	6,529
207252_at	7,380	7,252	7,305	7,896	7,690	7,929
207253_s_at	9,765	9,663	9,727	9,530	9,784	9,511
207254_at	5,589	5,429	5,578	6,130	6,240	6,415
207255_at	6,297	6,278	6,659	6,707	6,620	6,825
207256_at	3,916	3,880	4,212	4,072	4,201	4,245
207257_at	6,423	6,725	6,521	7,622	7,552	7,996
207258_at	7,003	7,009	7,344	7,027	6,622	6,863
207259_at	5,477	5,853	5,364	5,479	5,510	5,825
207260_at	7,232	7,364	7,483	7,791	7,590	7,535
207261_at	5,116	4,601	5,110	5,390	4,850	5,053
207262_at	5,146	5,488	4,606	5,195	5,037	5,286
207263_x_at	6,062	6,117	6,326	5,795	6,245	5,950
207264_at	6,216	6,427	5,786	9,477	9,862	10,259
207265_s_at	7,105	7,558	6,638	10,696	10,963	11,898
207266_x_at	10,354	10,535	10,030	9,297	10,463	9,900
207267_s_at	5,569	6,922	6,092	5,056	5,237	5,351
207268_x_at	9,346	9,517	9,444	9,566	9,629	9,790
207269_at	6,581	6,343	6,421	7,134	7,003	7,265
207270_x_at	6,782	7,042	6,680	7,419	7,320	7,276
207271_x_at	6,170	5,785	5,938	6,547	6,547	6,303
207272_at	3,874	4,172	3,819	4,064	3,812	3,951
207273_at	4,870	4,888	4,925	5,313	5,133	5,331
207274_at	6,762	6,949	6,889	7,843	7,841	7,925
207275_s_at	6,829	7,708	6,387	8,521	8,628	8,470
207276_at	4,243	4,394	4,629	6,323	6,280	6,517
207277_at	7,097	6,831	7,157	7,374	7,075	7,287
207278_s_at	7,478	7,301	7,510	7,893	7,986	8,164
207279_s_at	6,729	7,025	6,743	6,953	6,709	6,811
207280_at	6,852	6,950	6,908	7,389	7,297	7,383
207281_x_at	6,901	6,997	6,959	7,278	7,507	7,297
207282_s_at	7,276	7,393	7,494	7,869	7,378	7,488
207283_at	6,957	6,726	7,118	7,754	7,786	7,686
207284_s_at	4,875	5,320	5,016	6,003	7,042	6,526
207285_x_at	5,949	6,265	6,158	6,798	6,785	6,898
207286_at	4,622	4,121	4,296	3,826	3,967	3,912
207287_at	6,255	6,133	6,311	6,226	6,777	6,568
207288_at	6,646	6,637	6,426	6,712	6,519	6,772
207289_at	7,389	7,386	7,246	8,463	8,497	8,508
207290_at	5,688	5,382	5,445	5,881	6,203	6,525
207291_at	5,451	5,317	5,550	6,171	6,296	5,958
207292_s_at	7,981	7,671	8,212	7,966	7,823	8,103
207293_s_at	5,248	4,733	5,788	6,053	5,684	5,711
207294_at	5,072	4,962	5,217	5,392	5,338	5,524
207295_at	5,315	5,373	5,428	6,221	5,825	5,799
207296_at	4,981	4,976	4,863	5,262	5,073	5,315

207298_at	6,094	5,912	5,926	5,918	5,879	5,720
207299_s_at	4,739	4,942	4,744	5,026	4,825	5,131
207300_s_at	7,629	7,166	7,787	8,182	8,201	8,418
207301_at	6,558	6,008	6,495	6,815	6,709	6,646
207302_at	5,811	6,252	6,052	6,334	6,199	6,118
207303_at	6,344	6,238	5,790	6,838	6,671	6,527
207304_at	7,688	7,530	7,302	5,717	6,091	5,648
207305_s_at	10,192	10,732	10,019	8,843	10,386	8,765
207306_at	6,993	7,487	7,457	7,525	7,508	7,438
207307_at	5,088	5,616	5,155	4,624	4,478	4,429
207308_at	7,614	7,785	7,418	4,303	4,118	4,121
207309_at	6,545	6,530	6,667	6,987	6,982	7,035
207310_s_at	7,279	6,629	6,770	7,315	7,070	7,099
207311_at	7,463	7,421	7,453	7,494	7,595	7,768
207312_at	7,591	7,265	6,709	7,628	7,315	7,471
207313_x_at	6,165	6,440	6,479	7,465	7,358	7,519
207314_x_at	5,793	5,697	5,821	6,599	6,435	6,580
207315_at	5,155	4,969	5,066	5,671	6,023	5,805
207316_at	7,380	7,952	7,648	11,333	8,612	8,612
207317_s_at	6,473	6,632	6,501	6,927	6,779	6,728
207318_s_at	10,229	10,062	10,596	8,120	8,741	8,359
207319_s_at	6,432	5,952	6,379	5,519	5,541	5,933
207320_x_at	12,312	12,354	12,625	11,379	11,760	11,577
207321_s_at	6,794	6,748	7,005	7,374	7,466	7,412
207322_at	5,497	5,602	5,771	5,852	5,694	5,768
207323_s_at	7,882	7,514	7,822	8,130	7,942	8,019
207324_s_at	4,781	4,369	4,404	5,235	4,961	5,319
207325_x_at	5,631	5,422	5,990	6,062	5,871	6,322
207326_at	4,207	4,396	4,572	4,587	4,429	4,321
207327_at	5,494	5,213	5,039	6,822	6,633	6,536
207328_at	5,384	5,837	5,291	5,096	5,220	5,356
207329_at	5,971	5,817	5,978	6,047	6,161	5,886
207330_at	6,478	6,261	6,249	7,202	7,102	7,228
207331_at	6,675	6,492	6,212	4,029	4,476	4,185
207332_s_at	13,271	13,374	13,073	12,614	13,228	12,177
207333_at	4,283	4,326	4,350	4,723	4,672	5,093
207334_s_at	6,291	6,159	6,165	7,143	7,208	7,130
207335_x_at	10,960	10,869	11,344	11,598	11,163	11,601
207336_at	5,553	5,773	5,580	5,421	5,204	5,242
207337_at	6,919	6,927	7,359	7,821	7,959	7,942
207338_s_at	9,778	9,883	9,816	7,428	7,183	6,808
207339_s_at	6,817	6,682	6,943	7,159	7,198	7,270
207341_at	4,989	5,325	4,867	5,363	5,535	5,509
207342_at	4,232	4,076	4,206	4,360	4,203	4,237
207343_at	6,801	6,504	6,199	6,986	6,700	7,067
207344_at	7,200	7,305	7,646	6,071	6,200	6,326
207345_at	8,651	7,766	8,536	7,704	7,870	7,018
207346_at	7,524	7,679	7,794	8,352	8,468	8,619
207347_at	6,691	6,918	6,383	6,684	7,229	6,868
207348_s_at	7,999	7,799	7,853	7,026	6,722	6,963
207349_s_at	5,729	5,295	5,532	6,744	6,255	6,554
207350_s_at	7,079	7,576	7,121	8,288	7,776	7,847
207351_s_at	8,182	8,133	8,339	7,533	7,040	6,703
207352_s_at	7,318	6,793	6,945	7,885	7,738	7,595
207353_s_at	8,082	7,788	8,011	8,138	8,108	8,108
207354_at	5,927	5,836	6,025	6,436	6,233	6,266

207355_at	7,673	7,527	7,464	8,066	8,245	8,379
207356_at	7,445	7,148	7,206	7,860	7,703	7,748
207357_s_at	6,791	7,252	7,181	7,750	7,986	8,611
207358_x_at	10,598	10,379	10,503	8,818	10,241	8,584
207359_at	5,583	5,574	5,613	6,485	6,584	6,539
207360_s_at	7,582	7,401	7,377	7,723	7,372	7,618
207361_at	3,891	4,139	3,871	3,981	3,948	4,076
207362_at	4,501	4,739	4,372	4,863	5,031	5,135
207363_at	4,814	5,202	4,784	5,923	5,843	6,181
207364_at	6,935	6,853	6,792	7,017	6,763	6,909
207365_x_at	10,446	9,978	10,175	8,628	10,140	9,076
207366_at	7,378	7,525	7,450	7,885	7,648	7,765
207367_at	6,813	6,952	7,080	7,751	7,040	7,441
207368_at	6,186	6,104	6,665	6,175	6,016	6,237
207369_at	6,810	6,795	6,598	7,499	7,048	7,033
207370_at	6,771	7,085	7,232	7,916	8,056	8,200
207371_at	4,431	4,979	4,611	4,668	4,486	4,651
207372_s_at	7,024	7,126	6,680	7,635	7,412	7,481
207373_at	5,703	5,192	5,796	6,197	5,975	5,924
207374_at	5,532	5,435	5,801	6,022	5,673	5,850
207375_s_at	7,531	7,429	7,617	8,243	7,913	8,191
207376_at	8,242	8,382	8,678	6,916	6,821	7,008
207377_at	6,709	6,568	6,538	7,277	6,716	6,829
207378_at	5,932	5,820	6,606	6,895	6,954	7,032
207379_at	5,780	5,840	5,890	6,546	6,673	7,036
207380_x_at	6,528	6,508	6,463	7,089	7,022	7,247
207381_at	6,094	5,610	5,934	6,482	6,303	6,484
207382_at	7,061	6,862	6,964	7,648	7,543	7,395
207383_s_at	6,090	6,055	5,841	6,041	5,756	6,149
207384_at	7,494	7,283	7,197	8,139	7,942	8,308
207385_at	6,009	6,143	6,160	6,855	6,852	7,062
207386_at	4,946	5,445	5,186	5,315	4,845	4,992
207387_s_at	7,128	7,232	6,187	7,120	5,845	5,810
207388_s_at	7,030	6,959	6,971	7,743	7,372	7,624
207389_at	6,544	6,763	6,585	7,241	7,277	7,232
207390_s_at	7,063	6,927	7,346	9,292	8,873	9,494
207391_s_at	10,066	9,821	9,571	9,219	9,204	9,121
207392_x_at	6,701	6,647	6,784	6,825	6,831	6,867
207393_at	6,801	6,787	6,320	7,032	7,088	7,171
207394_at	7,832	7,284	6,848	5,093	5,427	5,251
207395_at	5,297	5,288	5,574	5,505	5,599	5,930
207396_s_at	8,963	8,896	9,399	9,701	8,473	9,415
207397_s_at	5,186	5,478	5,225	5,434	5,040	5,104
207398_at	5,637	5,603	5,492	5,578	5,464	5,540
207399_at	7,154	7,102	7,212	7,508	7,377	7,663
207400_at	5,994	5,072	5,699	6,190	6,173	6,135
207401_at	5,442	5,569	5,269	5,298	5,317	5,055
207402_at	6,540	6,089	6,536	6,218	5,805	5,775
207403_at	7,203	7,195	7,229	7,944	7,604	7,623
207404_s_at	6,429	6,250	6,676	7,402	7,249	7,402
207405_s_at	10,063	10,469	9,648	8,622	9,335	8,086
207406_at	3,948	3,947	3,654	3,589	3,584	3,726
207407_x_at	5,656	5,828	5,908	6,346	6,275	6,170
207408_at	8,044	7,974	8,104	7,694	7,800	7,787
207409_at	5,641	5,323	5,348	5,505	5,465	5,415
207410_s_at	5,451	5,187	5,585	6,618	6,140	6,478

207412_x_at	7,433	7,072	7,402	7,625	7,336	7,670
207413_s_at	7,603	7,251	7,671	7,718	7,255	7,269
207414_s_at	7,776	7,535	7,940	6,739	6,625	6,594
207415_at	4,514	4,322	4,431	5,081	5,144	5,022
207416_s_at	7,950	7,744	8,123	7,129	7,186	7,286
207417_s_at	7,702	7,815	7,258	7,361	6,878	6,983
207418_s_at	6,154	5,920	6,184	6,493	6,447	6,322
207419_s_at	6,497	6,224	6,536	8,334	7,461	8,285
207420_at	5,106	5,064	5,390	5,376	5,323	5,346
207421_at	6,323	6,037	6,235	6,843	6,493	6,634
207422_at	6,628	6,821	6,452	7,006	6,700	7,075
207423_s_at	6,239	6,035	6,522	6,591	6,325	6,394
207424_at	5,236	5,185	5,344	5,614	5,438	5,637
207425_s_at	6,923	6,598	6,842	7,346	7,253	7,312
207426_s_at	7,071	6,895	7,234	5,996	6,571	6,203
207427_at	6,503	6,625	6,583	6,794	6,592	6,869
207428_x_at	9,233	9,085	8,945	8,441	8,265	8,288
207429_at	6,560	6,603	6,633	7,517	6,935	7,298
207430_s_at	3,872	4,376	4,133	4,797	4,728	4,763
207431_s_at	9,154	8,880	9,181	9,948	9,365	10,255
207432_at	7,197	7,151	7,150	7,533	7,047	7,028
207433_at	5,014	4,895	5,075	4,989	4,733	4,921
207434_s_at	6,806	7,117	7,159	7,059	7,033	7,085
207435_s_at	10,355	9,891	10,288	9,245	9,143	9,092
207436_x_at	9,148	8,941	9,118	9,153	9,723	9,232
207437_at	5,163	5,085	5,523	4,102	4,326	3,431
207438_s_at	9,130	9,279	9,510	9,229	8,827	9,013
207439_s_at	5,529	5,432	5,293	5,860	5,567	5,775
207440_at	5,468	5,108	6,030	6,692	6,408	6,948
207441_at	5,972	6,086	6,187	6,632	5,982	6,189
207442_at	7,026	6,841	6,884	10,101	7,741	7,966
207443_at	5,109	5,213	5,445	5,830	5,606	5,488
207444_at	6,468	5,823	5,906	6,270	6,199	6,586
207445_s_at	4,727	4,639	4,660	4,842	4,758	4,744
207446_at	5,820	5,782	6,085	6,503	6,672	6,302
207447_s_at	7,672	7,486	6,850	3,768	3,535	3,506
207448_at	5,717	6,241	5,836	6,069	6,187	6,459
207449_s_at	7,016	6,974	7,144	7,700	7,600	7,741
207450_s_at	6,261	6,034	5,942	6,369	6,222	6,293
207451_at	6,787	7,172	7,083	7,517	7,712	7,500
207452_s_at	4,719	5,002	4,801	5,303	5,415	5,202
207453_s_at	6,038	6,059	6,099	6,642	6,430	6,675
207454_at	5,166	5,029	5,112	5,460	5,514	5,662
207455_at	5,698	5,507	5,473	5,228	4,969	4,891
207456_at	3,872	4,266	4,055	4,090	4,285	4,299
207457_s_at	7,095	6,923	6,722	7,601	7,531	7,759
207458_at	7,117	7,215	7,222	7,217	7,353	7,435
207459_x_at	6,566	6,631	6,630	7,199	6,902	6,935
207460_at	7,534	7,094	7,157	7,339	7,293	7,146
207461_at	3,552	3,778	3,860	3,711	3,462	3,682
207462_at	7,373	7,051	7,285	8,316	8,064	8,410
207463_x_at	6,954	6,872	6,643	10,302	9,215	8,953
207464_at	5,342	5,287	5,354	5,513	5,559	5,492
207465_at	3,848	3,823	3,752	3,675	3,740	3,642
207466_at	6,283	6,528	6,503	7,045	6,904	7,121
207467_x_at	8,334	8,672	7,924	10,706	10,926	10,686

207468_s_at	6,342	6,239	6,003	6,609	6,695	6,694
207469_s_at	9,853	9,966	9,525	10,017	8,675	9,072
207470_at	5,635	5,634	5,566	5,710	5,819	6,023
207471_at	4,008	4,080	3,939	4,151	4,002	4,191
207472_at	4,583	4,639	4,388	4,452	4,377	4,419
207473_at	6,646	6,735	6,860	7,317	7,005	7,202
207474_at	5,306	5,235	5,326	4,320	4,532	4,473
207475_at	6,773	6,692	6,645	6,863	6,879	6,795
207476_at	6,027	6,008	6,303	8,071	7,965	8,280
207477_at	4,796	5,113	4,918	5,196	4,622	4,772
207478_at	4,322	4,667	4,450	4,199	4,424	4,346
207479_at	4,489	3,951	4,663	5,572	4,889	5,264
207480_s_at	8,526	8,799	9,007	9,296	10,062	9,697
207481_at	4,169	3,902	4,272	4,324	4,343	4,422
207482_at	7,112	6,669	7,222	7,862	7,525	7,529
207483_s_at	11,443	11,467	11,084	9,525	10,510	9,094
207484_s_at	7,903	7,802	8,315	7,813	7,594	8,056
207485_x_at	6,468	6,787	6,963	7,735	7,343	7,495
207486_x_at	6,614	6,529	6,546	6,868	7,173	7,046
207487_at	3,767	3,883	3,851	3,637	3,967	3,924
207488_at	6,407	6,251	6,329	6,808	6,890	6,660
207489_at	5,134	5,421	5,348	5,284	5,336	5,187
207490_at	8,683	8,740	8,647	9,949	9,376	9,858
207491_at	6,429	6,698	6,716	7,019	6,789	6,917
207492_at	6,713	6,800	6,791	6,534	6,887	6,600
207493_x_at	7,048	7,071	6,771	7,196	6,867	7,177
207494_s_at	7,673	7,708	7,855	7,931	7,737	7,970
207495_at	7,484	7,775	7,380	7,534	7,605	7,586
207496_at	4,748	4,469	4,692	4,656	4,648	4,955
207497_s_at	5,063	5,255	4,871	5,706	5,500	5,785
207498_s_at	7,554	7,215	7,374	7,624	7,495	7,637
207499_x_at	7,938	7,575	7,588	7,509	7,971	7,550
207500_at	6,350	6,012	6,510	6,636	6,595	6,535
207501_s_at	6,550	6,696	6,358	5,689	5,556	5,760
207502_at	6,639	6,530	6,565	7,330	6,818	7,209
207503_at	6,768	6,871	7,132	6,915	6,916	6,992
207504_at	7,513	7,839	7,604	8,298	8,312	8,458
207505_at	6,702	7,128	7,168	7,406	7,020	7,351
207506_at	6,971	6,883	7,052	7,301	7,105	7,095
207507_s_at	12,380	12,374	12,482	12,553	12,031	12,702
207508_at	12,551	12,679	12,794	12,613	12,167	12,618
207509_s_at	8,145	8,054	8,128	8,455	8,070	8,199
207510_at	6,386	6,788	6,573	9,259	8,582	8,751
207511_s_at	6,778	6,483	6,310	6,967	6,580	7,369
207513_s_at	9,949	9,822	9,624	7,875	8,689	8,138
207514_s_at	6,330	6,526	6,516	6,748	6,610	6,690
207515_s_at	8,986	8,441	8,653	8,681	7,950	8,427
207516_at	5,753	5,643	5,693	5,809	5,798	5,969
207517_at	5,719	5,320	5,427	6,088	6,746	6,508
207518_at	5,542	5,667	5,646	5,890	5,864	5,577
207519_at	6,179	6,266	6,292	6,528	6,406	6,409
207520_at	5,269	4,968	5,127	5,024	4,911	5,064
207521_s_at	6,589	6,140	6,487	6,924	6,672	7,040
207522_s_at	6,853	6,641	6,441	7,121	7,072	7,178
207523_at	4,178	4,222	4,299	4,392	4,149	4,299
207524_at	7,799	7,909	8,154	8,207	8,016	8,105

207525_s_at	9,071	9,136	9,129	9,699	9,684	9,829
207526_s_at	6,589	6,610	6,731	7,497	7,769	7,786
207527_at	6,673	6,684	6,730	7,635	7,386	7,428
207528_s_at	6,262	6,574	7,051	8,835	8,928	9,627
207529_at	5,224	5,719	5,289	6,360	6,111	6,038
207530_s_at	6,435	6,492	6,266	6,745	6,475	6,442
207531_at	7,075	7,321	7,171	7,379	7,488	7,457
207532_at	7,337	7,075	7,423	7,704	7,523	7,704
207533_at	6,467	6,360	6,748	6,980	7,248	7,102
207534_at	4,617	4,240	4,322	4,707	4,687	4,898
207535_s_at	6,454	6,213	6,085	6,462	6,683	6,273
207536_s_at	6,000	5,970	6,148	6,355	6,192	6,514
207537_at	7,504	7,621	7,512	7,654	7,729	7,746
207538_at	4,714	4,405	4,914	5,222	4,917	5,031
207539_s_at	5,932	5,528	6,009	6,930	6,187	6,550
207540_s_at	7,333	7,466	7,653	7,391	7,589	7,560
207541_s_at	10,094	9,965	10,069	9,667	9,840	9,722
207542_s_at	7,333	6,997	7,100	7,523	7,637	7,345
207543_s_at	9,247	9,756	9,169	9,949	10,825	10,680
207544_s_at	4,384	5,029	5,056	5,251	5,273	5,303
207545_s_at	7,277	7,654	7,018	8,766	8,850	8,723
207546_at	5,735	5,113	5,781	6,692	6,467	6,670
207547_s_at	7,300	7,387	7,000	7,131	7,062	7,034
207548_at	4,452	4,310	4,414	4,516	4,407	4,475
207549_x_at	9,461	10,080	8,695	7,991	7,973	8,001
207550_at	5,615	5,945	5,759	5,634	5,238	5,396
207551_s_at	9,591	9,207	9,660	9,362	9,541	9,547
207552_at	7,355	6,975	6,825	7,662	7,211	7,501
207553_at	7,762	7,489	7,124	6,380	6,334	6,248
207554_x_at	6,780	7,052	6,895	7,266	6,903	7,338
207555_s_at	7,922	7,783	8,130	9,395	9,374	9,773
207556_s_at	8,705	8,807	8,822	8,881	8,682	8,988
207557_s_at	6,149	6,009	6,527	6,388	6,167	6,489
207558_s_at	4,309	3,910	4,222	3,853	3,878	7,626
207559_s_at	11,163	11,584	11,460	8,684	8,530	8,798
207560_at	6,146	6,254	6,169	6,784	7,057	6,802
207561_s_at	5,830	5,409	5,244	6,236	6,139	6,205
207562_at	6,574	6,580	6,603	6,696	6,423	6,654
207563_s_at	8,794	8,833	8,610	7,515	8,279	7,773
207564_x_at	8,946	9,200	8,869	6,714	7,856	6,978
207565_s_at	5,122	5,610	5,801	7,962	8,362	8,951
207566_at	6,407	6,430	6,659	7,422	7,471	7,630
207567_at	5,703	5,695	5,687	5,615	5,425	5,469
207568_at	6,209	6,338	6,450	6,458	6,432	6,432
207569_at	6,462	6,580	6,433	7,133	6,833	7,054
207570_at	6,100	6,205	5,949	6,204	6,100	6,214
207571_x_at	8,484	8,629	8,327	7,575	7,268	7,209
207572_at	5,616	5,392	5,333	5,542	5,507	5,592
207573_x_at	11,923	12,137	12,019	12,073	11,612	12,118
207574_s_at	7,958	9,749	9,224	10,076	11,926	11,409
207575_at	5,651	6,091	5,561	5,133	5,126	5,281
207576_x_at	7,468	7,327	6,792	8,223	7,932	7,984
207577_at	5,412	5,278	5,394	5,443	5,286	5,299
207578_s_at	8,231	8,300	8,483	8,867	8,706	9,055
207579_at	4,452	4,469	4,617	4,841	4,908	4,964
207580_at	3,757	3,901	3,780	3,795	3,884	3,818

207581_s_at	5,346	5,275	5,239	6,294	5,767	5,689
207582_at	7,174	7,228	7,358	7,739	7,226	7,597
207583_at	4,074	3,887	4,081	4,115	4,299	4,330
207584_at	4,680	4,857	4,888	4,764	4,768	4,645
207585_s_at	10,739	11,012	10,465	11,630	11,491	11,560
207586_at	5,666	5,642	5,644	5,930	5,751	5,835
207587_at	5,988	6,113	5,988	6,098	5,992	6,401
207588_at	3,764	3,938	3,651	4,356	4,234	4,235
207589_at	5,776	5,911	6,268	6,192	6,165	6,282
207590_s_at	8,416	8,566	7,896	7,015	7,019	6,504
207591_s_at	7,821	7,877	8,165	8,836	8,531	8,631
207592_s_at	6,430	6,563	6,510	6,835	6,872	7,225
207593_at	8,381	8,103	8,456	8,857	8,781	8,697
207594_s_at	5,497	5,643	5,513	5,942	5,545	5,801
207595_s_at	7,690	7,454	7,699	8,465	8,499	9,003
207596_at	3,944	3,680	4,022	3,906	3,847	3,894
207597_at	5,577	5,582	5,570	6,386	5,793	5,923
207598_x_at	8,854	7,871	8,265	7,645	8,000	7,728
207599_at	4,571	5,209	4,767	5,353	4,832	4,770
207600_at	8,390	8,046	8,237	8,666	8,295	8,412
207601_at	6,739	7,001	6,948	7,992	10,542	8,112
207602_at	6,899	7,094	6,774	7,450	7,391	7,596
207603_at	4,123	4,421	4,606	4,912	4,730	4,958
207604_s_at	9,529	9,738	9,296	9,123	10,293	9,401
207605_x_at	7,551	7,923	7,040	5,450	5,466	5,268
207606_s_at	8,606	8,830	8,128	7,092	8,682	6,562
207607_at	3,594	3,495	3,766	3,716	3,638	3,558
207608_x_at	7,713	7,481	7,782	7,766	7,563	7,579
207609_s_at	7,078	6,905	6,693	7,687	7,149	7,480
207610_s_at	5,938	5,977	5,525	7,391	6,204	6,829
207611_at	5,702	5,570	5,528	5,808	5,678	5,851
207612_at	6,729	6,894	6,878	7,028	6,799	6,895
207613_s_at	6,682	6,720	6,617	7,013	6,747	6,938
207614_s_at	11,003	10,617	10,555	9,010	9,686	8,728
207615_s_at	5,598	5,396	5,748	6,942	6,610	6,876
207616_s_at	8,870	9,480	8,691	9,107	10,272	9,335
207617_at	6,581	6,516	6,441	7,413	7,303	7,520
207618_s_at	9,587	9,251	9,571	10,453	9,656	10,142
207619_at	5,235	5,220	5,183	5,364	5,315	5,336
207620_s_at	8,239	8,314	7,753	6,742	6,908	6,628
207621_s_at	8,760	8,640	9,044	9,728	8,264	9,658
207622_s_at	9,253	8,714	9,298	9,299	8,997	9,117
207623_at	6,780	6,594	6,718	7,383	7,284	7,044
207624_s_at	7,654	7,800	6,968	6,841	7,412	6,658
207625_s_at	8,698	8,744	8,823	7,957	7,845	8,272
207626_s_at	4,749	4,837	5,234	5,540	5,273	5,496
207627_s_at	8,509	8,026	8,015	7,347	7,178	7,724
207628_s_at	10,193	9,452	9,859	9,939	9,265	9,984
207629_s_at	7,981	7,655	7,668	7,173	6,794	7,130
207630_s_at	8,721	8,919	8,555	8,068	7,750	7,852
207631_at	7,290	7,107	7,328	7,062	7,218	7,372
207632_at	5,476	5,429	5,569	5,894	5,569	5,667
207633_s_at	5,869	5,875	6,042	6,153	5,815	5,882
207634_at	7,245	7,573	7,543	7,602	7,506	7,478
207635_s_at	5,575	5,943	5,810	6,154	6,629	6,417
207636_at	5,326	5,547	5,801	6,073	6,147	5,857

207637_at	6,784	6,707	6,981	7,201	7,153	7,289
207638_at	3,876	4,109	3,880	3,870	3,741	3,808
207639_at	7,208	7,078	7,043	7,181	6,875	7,309
207640_x_at	6,485	6,803	6,348	6,800	7,044	7,064
207641_at	6,691	6,817	6,984	7,057	7,081	7,118
207642_at	6,507	6,293	6,107	6,324	5,998	6,240
207643_s_at	9,845	9,659	9,831	9,523	9,664	10,152
207644_at	7,324	7,358	7,527	7,137	6,742	6,812
207645_s_at	6,650	6,615	6,371	6,467	6,229	6,358
207646_s_at	5,871	5,467	5,636	6,067	5,813	5,815
207647_at	3,861	3,802	3,699	3,968	3,761	3,943
207648_at	7,432	7,334	7,228	7,666	7,354	7,662
207649_at	5,774	6,089	6,090	6,515	6,691	6,889
207650_x_at	7,017	7,208	7,139	7,833	8,140	8,110
207651_at	5,279	4,519	5,166	5,541	5,150	5,588
207652_s_at	5,676	5,976	5,843	6,799	6,648	6,889
207653_at	5,679	5,153	5,196	5,679	6,099	6,242
207654_x_at	9,249	9,251	9,247	9,795	9,234	9,970
207655_s_at	5,032	5,210	5,452	5,451	5,626	5,731
207656_s_at	6,407	6,181	6,682	7,000	6,866	6,963
207657_x_at	11,213	11,060	10,867	10,781	12,041	10,856
207658_s_at	6,680	6,597	6,501	6,941	6,972	6,878
207659_s_at	5,585	5,498	5,515	5,894	5,654	5,883
207660_at	5,919	6,699	6,240	6,133	6,024	5,973
207661_s_at	5,011	5,156	5,063	4,716	4,862	4,906
207662_at	6,596	6,615	6,598	7,554	7,394	7,760
207663_x_at	6,725	6,691	6,598	7,030	6,775	6,937
207664_at	4,396	4,273	4,140	4,584	4,642	4,610
207665_at	5,905	6,234	5,724	5,785	6,112	5,998
207666_x_at	5,634	5,371	5,408	5,621	5,819	5,881
207667_s_at	7,685	7,695	7,782	7,990	7,999	8,372
207668_x_at	12,122	11,975	11,833	11,513	11,579	11,563
207669_at	7,402	7,266	7,350	7,789	7,438	8,054
207670_at	7,373	7,041	7,331	7,900	7,690	7,768
207671_s_at	7,113	7,197	6,977	7,559	7,598	7,477
207672_at	4,429	4,906	4,554	4,509	4,256	4,386
207673_at	6,374	6,171	6,164	6,413	6,500	6,535
207674_at	3,935	3,901	4,324	3,867	4,088	4,086
207675_x_at	7,333	6,816	7,037	8,563	8,473	8,610
207676_at	6,636	6,366	6,236	7,033	7,347	7,627
207677_s_at	4,871	4,953	5,058	6,026	6,019	6,151
207678_s_at	5,130	5,617	5,749	4,592	4,619	4,790
207679_at	5,542	5,759	5,816	6,477	6,198	6,437
207680_x_at	5,815	5,771	6,341	6,772	6,786	6,927
207681_at	6,220	6,282	6,198	6,670	6,318	6,345
207682_s_at	5,951	6,232	6,155	6,313	6,360	6,580
207683_at	6,395	5,993	6,373	6,739	6,685	6,949
207684_at	7,245	6,993	7,040	7,184	7,115	7,146
207685_at	7,193	7,294	7,121	7,864	7,589	7,660
207686_s_at	6,171	6,551	6,586	6,397	6,258	6,478
207687_at	6,630	6,350	6,803	7,501	6,707	7,093
207688_s_at	6,355	7,014	6,797	6,485	6,745	6,280
207689_at	5,560	5,466	5,916	6,760	6,454	6,714
207690_at	6,852	6,633	7,064	7,665	7,215	7,288
207691_x_at	5,532	5,572	5,450	6,503	6,609	6,741
207692_s_at	6,919	6,533	6,784	7,395	7,113	7,318

207693_at	7,075	6,814	7,031	7,560	7,469	7,449
207694_at	6,230	6,277	6,386	7,686	7,546	7,631
207695_s_at	7,292	7,406	7,672	6,733	6,280	6,724
207696_at	4,263	4,101	3,936	4,026	4,148	4,216
207697_x_at	7,091	7,126	6,886	7,343	7,271	7,464
207698_at	6,268	6,556	6,299	6,772	6,638	6,838
207699_at	7,564	7,425	7,511	8,495	8,495	8,492
207700_s_at	8,649	8,708	8,640	7,267	8,670	7,712
207701_at	6,504	6,328	6,623	6,564	7,089	7,039
207702_s_at	6,149	6,287	6,400	6,744	6,351	6,648
207703_at	7,759	8,195	7,986	4,997	6,286	5,497
207704_s_at	6,253	5,906	6,228	6,355	6,109	6,211
207705_s_at	9,506	9,684	9,468	7,686	7,757	7,873
207706_at	3,810	3,892	3,845	3,897	3,745	3,808
207707_s_at	11,866	11,967	12,184	12,235	11,828	12,507
207708_at	6,921	6,993	7,182	7,369	7,334	7,369
207709_at	7,166	6,989	6,858	6,219	6,245	5,864
207710_at	5,360	5,458	5,481	5,916	6,065	6,041
207711_at	7,154	6,447	6,501	5,962	6,378	6,022
207712_at	4,304	4,566	4,591	4,329	4,194	4,450
207713_s_at	7,874	7,819	8,103	8,134	8,135	8,486
207714_s_at	10,616	10,380	10,498	10,882	9,887	11,575
207715_at	6,829	7,082	6,962	6,974	6,847	6,991
207716_at	7,246	7,341	7,205	7,337	7,476	7,350
207717_s_at	7,865	8,175	7,707	3,689	3,847	3,486
207718_x_at	6,505	6,582	6,956	7,033	7,298	7,279
207719_x_at	11,431	11,387	10,896	9,996	10,946	9,819
207720_at	7,083	7,182	7,192	7,199	7,074	7,184
207721_x_at	12,322	12,554	12,515	12,330	12,261	12,589
207722_s_at	7,822	7,562	7,614	7,508	7,482	7,514
207723_s_at	3,863	3,773	4,113	4,306	4,346	4,395
207724_s_at	6,558	6,568	6,160	4,691	5,416	5,066
207725_at	5,030	5,091	5,196	5,336	5,064	5,090
207726_at	5,999	5,940	6,244	7,002	6,976	6,782
207727_s_at	11,207	11,090	11,293	9,505	8,402	9,027
207728_at	5,706	5,432	5,340	4,993	5,458	5,367
207729_at	8,757	9,023	8,147	4,833	4,655	4,730
207730_x_at	10,178	9,740	10,110	8,977	10,211	9,118
207731_at	4,836	5,059	4,642	4,647	4,680	4,698
207732_s_at	8,122	8,131	7,835	6,694	6,618	6,933
207733_x_at	6,766	6,642	6,580	6,897	6,806	6,736
207734_at	5,723	5,709	5,752	6,031	6,053	5,932
207735_at	6,801	6,690	6,771	6,955	6,738	6,857
207736_s_at	6,010	5,692	5,849	6,738	6,546	6,983
207737_at	4,063	3,944	4,100	3,850	3,765	4,084
207738_s_at	11,969	11,857	11,787	11,408	12,169	11,356
207739_s_at	5,960	5,974	6,279	7,935	7,947	8,009
207740_s_at	7,671	6,976	7,673	7,382	6,908	7,293
207741_x_at	6,993	7,020	7,283	7,558	7,428	7,703
207742_s_at	7,149	7,249	7,120	6,930	6,600	6,823
207743_at	6,165	6,563	6,222	5,938	6,160	6,027
207744_at	3,761	3,952	3,999	3,685	3,560	3,724
207745_at	6,445	6,047	6,145	6,809	6,848	6,731
207746_at	9,210	8,589	9,070	6,104	7,040	6,220
207747_s_at	7,567	7,102	7,555	8,267	7,743	8,066
207748_at	7,496	7,491	7,368	7,954	7,731	8,109

207749_s_at	9,075	9,545	9,276	8,448	8,881	8,580
207750_at	3,830	3,695	3,708	4,008	4,108	3,924
207751_at	6,010	6,131	6,047	6,588	6,648	6,809
207752_x_at	8,072	7,707	7,809	8,892	8,504	8,784
207753_at	7,963	7,282	7,428	5,888	6,168	5,224
207754_at	7,879	8,019	7,671	7,840	7,725	8,060
207755_at	6,025	5,862	5,871	6,988	6,861	6,409
207756_at	5,652	5,576	6,067	5,769	6,066	5,565
207757_at	5,719	5,951	5,510	5,843	5,379	5,388
207758_at	6,887	6,271	6,770	8,520	8,806	8,723
207759_s_at	4,468	4,217	4,631	4,619	4,372	4,668
207760_s_at	10,954	10,805	11,101	9,293	9,879	10,034
207761_s_at	10,658	10,332	11,727	9,293	10,179	9,745
207762_at	6,208	6,172	6,082	6,804	6,342	6,512
207763_at	7,254	7,240	7,230	8,345	8,126	8,279
207764_s_at	5,498	5,686	5,358	5,754	5,843	5,612
207765_s_at	7,011	6,902	6,881	8,021	7,986	8,252
207766_at	6,342	6,131	6,148	7,629	7,218	7,415
207767_s_at	7,098	7,428	7,425	7,617	7,265	7,632
207768_at	4,963	7,371	6,978	5,448	5,354	5,727
207769_s_at	9,797	9,473	9,717	9,428	8,680	9,412
207770_x_at	7,457	7,398	7,428	7,586	7,051	7,341
207771_at	6,999	6,959	7,222	7,355	7,097	7,499
207772_s_at	6,500	6,055	6,020	6,092	6,048	6,220
207773_x_at	4,818	4,630	4,915	5,501	5,323	5,422
207774_at	7,109	6,889	7,278	7,300	7,291	7,354
207775_at	3,818	3,641	4,495	3,906	4,149	4,015
207776_s_at	6,310	6,415	6,255	5,922	5,952	6,072
207777_s_at	4,654	4,414	5,053	5,963	5,861	5,918
207778_at	6,474	6,232	6,246	6,604	6,067	6,289
207779_at	4,599	4,815	5,039	4,541	4,431	4,552
207780_at	5,543	5,368	5,394	5,735	5,548	5,776
207781_s_at	6,055	5,887	4,845	4,223	4,007	4,135
207782_s_at	8,082	7,600	7,617	7,461	7,587	7,739
207783_x_at	14,375	14,493	14,428	14,018	14,218	14,276
207784_at	7,527	7,665	7,729	8,052	7,927	8,011
207785_s_at	10,793	10,796	10,608	7,940	9,567	8,264
207786_at	7,215	7,327	7,167	6,607	6,815	6,771
207787_at	6,928	6,782	7,409	7,472	7,217	7,500
207788_s_at	7,249	7,122	7,184	8,247	8,447	8,357
207789_s_at	7,994	7,761	8,125	7,074	6,875	7,127
207790_at	5,497	5,514	5,905	6,059	6,150	5,962
207791_s_at	10,434	10,408	9,639	10,461	10,578	10,364
207792_at	6,524	6,557	6,716	7,109	7,051	7,053
207793_s_at	3,606	3,743	3,821	3,643	3,569	3,618
207794_at	4,806	4,164	5,016	5,093	5,128	5,117
207795_s_at	6,744	6,577	6,732	6,901	6,997	6,976
207796_x_at	4,944	5,058	4,849	4,985	4,899	5,042
207797_s_at	5,862	5,792	5,234	5,442	5,893	5,743
207798_s_at	6,473	6,375	7,104	6,599	6,586	6,765
207799_x_at	5,886	6,054	6,124	6,336	6,092	5,740
207800_at	5,966	6,077	6,217	6,672	6,400	6,375
207801_s_at	9,406	9,383	9,532	8,986	8,966	9,262
207802_at	4,034	3,754	3,746	4,351	4,103	4,133
207803_s_at	5,853	5,584	6,111	6,366	5,673	6,030
207804_s_at	5,650	5,885	5,835	6,582	6,367	6,717

207805_s_at	10,509	10,618	10,837	11,771	11,056	11,690
207808_s_at	8,639	8,875	7,969	9,161	10,448	8,985
207809_s_at	9,397	9,105	9,547	10,046	9,485	10,230
207810_at	4,215	4,265	4,514	4,901	4,559	4,626
207811_at	5,769	5,383	5,430	6,583	6,240	6,219
207812_s_at	11,499	11,617	11,763	11,182	11,493	11,795
207813_s_at	7,018	7,139	6,939	7,766	7,249	7,652
207814_at	6,650	6,640	6,728	7,030	7,092	6,975
207815_at	3,495	4,024	3,815	4,862	4,731	4,931
207816_at	5,834	5,946	6,000	6,771	6,729	6,370
207817_at	5,721	5,680	5,801	6,146	6,188	6,186
207818_s_at	5,647	5,280	5,408	6,285	5,828	6,237
207819_s_at	6,421	6,859	6,227	6,210	5,870	6,255
207820_at	5,950	5,879	6,007	6,492	6,202	6,334
207821_s_at	8,816	8,746	8,415	7,540	7,972	7,708
207822_at	8,010	7,488	7,184	6,094	6,151	6,220
207823_s_at	6,112	5,589	6,012	6,542	6,227	6,200
207824_s_at	6,249	6,164	6,410	6,942	6,642	7,027
207825_s_at	7,454	7,347	7,085	7,576	7,402	7,488
207826_s_at	9,877	10,077	9,714	9,298	10,373	10,956
207827_x_at	8,714	8,858	8,814	9,286	9,518	9,547
207828_s_at	12,818	12,927	12,296	9,483	10,506	8,395
207829_s_at	7,283	7,359	7,066	7,667	7,358	7,852
207830_s_at	12,203	11,957	11,802	10,215	10,054	10,195
207831_x_at	10,191	10,350	9,906	9,603	8,609	9,497
207832_at	7,120	7,430	7,242	8,280	8,295	8,511
207833_s_at	7,481	6,460	7,107	7,237	7,165	7,381
207834_at	7,197	6,965	7,125	7,736	7,545	7,493
207835_at	6,641	6,722	6,816	7,085	6,653	6,874
207836_s_at	10,840	11,500	11,467	9,671	9,038	9,733
207837_at	6,142	6,516	6,316	6,015	6,053	5,913
207838_x_at	7,185	7,196	6,927	7,583	7,369	7,772
207839_s_at	7,974	8,512	7,960	8,420	8,047	8,330
207840_at	6,324	6,523	6,634	7,137	7,001	7,003
207841_at	6,090	5,701	6,219	6,578	6,290	6,037
207842_s_at	9,484	9,733	9,835	8,802	8,852	9,033
207843_x_at	9,929	10,321	9,999	10,381	10,590	10,936
207844_at	6,882	6,894	6,712	7,355	7,408	7,596
207845_s_at	9,004	9,471	9,211	9,676	8,963	9,372
207846_at	3,406	3,415	3,401	3,922	3,731	4,109
207847_s_at	8,257	8,143	8,346	8,400	8,503	8,406
207848_at	6,846	6,981	6,868	7,054	6,811	6,881
207849_at	3,803	4,141	4,512	4,415	4,438	4,614
207850_at	6,399	7,192	6,616	13,012	10,372	10,134
207851_s_at	6,679	7,076	6,736	8,081	7,827	7,874
207852_at	3,538	3,719	3,683	8,686	3,996	4,314
207853_s_at	7,857	7,544	7,425	8,032	8,020	8,054
207854_at	4,766	5,012	5,204	5,474	5,648	5,459
207855_s_at	8,087	8,145	7,536	8,351	8,353	8,258
207856_s_at	10,233	9,847	10,292	8,939	8,821	9,059
207857_at	5,189	5,105	4,802	5,900	5,907	6,175
207858_s_at	6,488	5,976	6,100	6,838	6,786	6,561
207859_s_at	5,134	5,459	5,246	5,859	5,570	5,854
207860_at	4,844	4,972	5,039	5,263	5,421	5,303
207861_at	7,666	7,492	7,495	7,821	8,070	8,030
207862_at	6,427	6,343	6,803	6,955	6,722	6,827

207863_at	6,796	6,393	7,010	6,900	6,512	6,772
207864_at	4,360	4,815	4,599	4,852	4,788	4,966
207865_s_at	6,408	6,256	6,387	6,455	6,097	6,032
207866_at	6,626	6,469	6,767	6,590	6,404	6,687
207867_at	3,706	3,628	3,788	3,544	3,520	3,471
207868_at	8,500	8,558	8,744	9,400	9,228	9,240
207869_s_at	4,194	4,258	4,035	3,732	3,638	3,735
207870_at	4,498	4,260	4,415	4,798	4,879	5,009
207871_s_at	7,889	7,643	8,363	8,126	7,746	7,733
207872_s_at	6,227	5,877	5,898	7,238	6,953	6,960
207873_x_at	6,853	6,999	6,834	7,155	7,019	7,081
207874_s_at	7,063	7,372	6,698	6,418	6,188	6,513
207875_at	4,226	4,482	4,791	4,847	4,542	4,433
207876_s_at	9,761	9,520	9,796	10,135	9,976	11,008
207877_s_at	10,593	10,452	10,437	8,156	8,378	8,099
207878_at	6,438	6,388	6,473	7,473	7,021	7,286
207879_at	3,665	3,807	3,797	4,539	4,259	4,412
207880_at	7,094	7,163	7,203	7,369	7,037	7,103
207881_at	4,544	4,540	4,369	4,658	4,351	4,606
207882_at	6,903	6,710	6,572	7,149	7,136	7,059
207883_s_at	7,277	7,530	7,608	7,530	7,365	7,494
207884_at	7,123	7,211	7,319	7,355	7,186	7,359
207885_at	5,392	5,241	5,393	6,362	6,064	6,144
207886_s_at	5,257	5,535	5,488	5,710	5,582	5,825
207887_s_at	6,348	6,290	6,598	6,658	6,542	6,955
207888_at	4,962	4,702	4,719	5,529	5,508	5,900
207889_at	5,558	5,391	5,390	5,649	5,170	5,384
207890_s_at	7,481	7,434	7,212	6,900	6,469	6,659
207891_s_at	6,872	6,437	6,342	6,631	5,585	6,281
207892_at	6,810	6,830	6,895	7,082	6,892	7,091
207893_at	7,155	7,117	7,017	7,664	7,163	7,551
207894_s_at	6,793	6,829	6,521	7,818	7,677	7,560
207895_at	7,143	6,972	7,118	7,834	7,695	7,738
207896_s_at	7,353	7,155	7,058	7,477	7,254	7,500
207897_at	5,873	6,528	5,900	6,434	6,461	6,396
207899_at	6,445	6,506	6,379	6,815	7,016	7,070
207900_at	6,990	6,736	6,659	7,499	7,548	7,373
207901_at	3,947	4,268	4,210	4,110	4,202	4,446
207902_at	6,838	6,641	6,765	7,078	7,079	7,069
207904_s_at	4,814	4,926	5,039	6,845	7,627	6,846
207906_at	4,811	5,049	4,820	5,986	5,860	5,961
207907_at	6,060	5,715	5,837	7,396	7,251	7,263
207908_at	6,643	6,480	6,865	7,173	6,924	6,876
207909_x_at	3,870	4,236	3,881	4,502	4,585	4,466
207910_at	4,861	4,657	4,756	5,065	4,963	4,834
207911_s_at	8,409	7,999	8,372	8,821	8,713	8,406
207912_s_at	4,173	4,651	3,868	3,541	3,468	3,520
207913_at	5,121	4,985	4,937	4,956	4,723	4,824
207914_x_at	6,772	6,834	6,993	7,017	6,960	6,700
207915_at	6,320	5,998	6,060	6,694	6,212	6,439
207916_at	6,130	6,168	6,411	6,482	6,428	6,439
207917_at	6,275	6,375	6,356	7,422	7,603	7,526
207918_s_at	4,841	4,839	5,140	5,616	5,490	5,742
207919_at	7,450	6,821	7,225	7,892	7,582	7,839
207920_x_at	7,084	7,331	7,060	6,831	6,848	6,342
207921_x_at	6,734	6,764	6,889	6,803	6,700	7,177

207922_s_at	11,207	11,092	11,445	11,322	11,244	11,446
207923_x_at	6,582	6,900	6,645	7,043	7,175	7,124
207924_x_at	4,890	4,738	4,717	5,795	5,794	5,825
207925_at	6,269	6,389	6,221	6,602	6,527	6,376
207926_at	4,941	5,076	5,352	5,332	5,352	5,256
207927_at	6,938	7,140	7,063	8,292	8,133	8,315
207928_s_at	3,359	3,685	3,578	3,586	3,468	3,511
207929_at	7,473	7,555	7,649	7,956	7,419	7,865
207930_at	6,664	6,440	6,472	7,382	7,345	7,524
207931_s_at	7,353	7,283	7,239	7,684	7,519	7,707
207932_at	4,064	3,996	3,880	4,524	4,168	4,587
207933_at	6,655	6,480	6,623	7,066	6,879	7,139
207934_at	3,674	3,621	3,806	3,746	3,695	3,665
207935_s_at	5,095	5,236	5,354	6,289	5,654	5,893
207936_x_at	6,743	7,010	6,604	6,870	6,774	6,972
207937_x_at	8,820	8,685	8,777	8,700	8,567	8,488
207938_at	6,767	6,712	6,598	6,683	6,430	6,533
207939_x_at	10,492	10,399	10,647	9,532	9,780	9,869
207940_x_at	6,673	6,537	6,684	7,277	7,186	7,219
207941_s_at	12,551	12,897	12,495	10,444	11,838	10,691
207943_x_at	7,892	6,468	6,610	8,144	9,025	8,779
207944_at	6,061	6,493	6,263	6,797	6,542	6,613
207945_s_at	8,521	8,402	8,883	9,831	9,176	9,641
207946_at	6,610	6,629	6,902	6,785	6,748	6,819
207949_s_at	7,796	8,345	7,824	8,190	8,082	8,141
207950_s_at	6,374	6,897	6,484	6,231	6,281	6,212
207951_at	5,220	5,097	5,537	5,166	5,052	5,139
207952_at	4,377	4,746	4,842	5,104	5,054	5,353
207953_at	7,617	7,441	7,387	7,981	8,778	8,121
207954_at	7,350	7,149	7,174	8,010	8,011	8,042
207955_at	5,750	5,291	5,517	6,232	6,506	6,451
207956_x_at	10,506	10,435	10,209	8,087	9,238	7,158
207957_s_at	6,665	7,043	6,630	4,837	4,983	4,897
207958_at	4,178	4,315	4,227	4,906	4,620	4,829
207959_s_at	6,100	6,500	6,710	7,389	7,784	7,834
207960_at	7,062	6,899	6,645	6,934	6,904	7,114
207961_x_at	7,291	7,876	7,698	8,281	8,289	8,393
207962_at	6,614	6,374	6,108	6,759	6,232	6,556
207963_at	7,673	7,592	7,331	7,561	7,425	7,340
207964_x_at	5,704	5,896	5,884	6,498	6,465	6,510
207965_at	6,096	6,332	5,963	6,616	6,708	6,920
207966_s_at	10,843	10,793	11,046	10,367	10,960	10,995
207967_at	7,737	7,721	7,307	8,299	8,299	8,656
207968_s_at	6,029	6,024	6,036	6,565	5,961	6,034
207969_x_at	6,510	6,407	6,601	6,939	6,707	6,737
207971_s_at	8,280	8,397	8,719	7,517	7,608	7,695
207972_at	5,345	4,766	5,240	5,553	5,423	5,548
207973_x_at	6,169	5,997	6,421	6,636	6,369	6,825
207974_s_at	11,229	11,294	10,924	11,339	11,543	11,451
207976_at	6,359	6,233	6,707	6,284	6,461	6,106
207977_s_at	6,087	6,330	6,936	7,499	7,270	7,457
207978_s_at	5,004	4,559	5,485	5,803	6,254	5,859
207979_s_at	7,296	7,310	7,282	7,950	7,766	7,953
207980_s_at	6,359	7,353	6,691	7,972	8,666	9,664
207981_s_at	6,173	7,501	5,154	4,345	3,927	4,019
207982_at	6,853	6,943	6,819	7,294	7,511	7,463

207983_s_at	9,714	9,962	9,104	7,449	8,524	6,917
207984_s_at	7,883	7,863	8,111	6,691	6,624	6,620
207985_at	6,385	6,228	6,340	6,508	6,186	6,437
207986_x_at	8,924	8,826	8,860	8,850	8,601	8,958
207987_s_at	5,902	6,437	5,398	4,461	5,427	4,553
207988_s_at	11,921	11,948	11,980	12,107	12,099	12,448
207989_at	4,451	4,425	4,467	4,590	4,677	4,674
207990_x_at	6,566	6,272	6,304	6,781	6,550	6,803
207991_x_at	6,082	5,998	6,357	6,360	6,547	6,510
207992_s_at	7,542	7,705	7,566	8,802	8,301	8,056
207993_s_at	6,469	6,579	6,270	7,024	6,630	6,905
207994_s_at	5,257	5,353	5,202	5,628	5,603	5,732
207995_s_at	6,005	6,040	5,812	6,336	6,326	6,198
207996_s_at	4,791	5,107	5,385	3,880	3,853	3,842
207998_s_at	6,546	6,660	6,787	7,237	7,033	7,174
207999_s_at	7,044	7,053	7,228	7,530	7,598	7,625
208000_at	6,282	6,242	6,260	6,136	6,363	5,916
208002_s_at	9,404	9,443	9,570	10,450	9,684	10,347
208003_s_at	7,699	7,969	7,425	6,942	7,406	7,128
208004_at	5,114	5,438	5,927	6,008	6,045	6,237
208005_at	5,942	5,651	5,776	6,329	6,005	6,081
208006_at	5,510	5,681	5,518	5,763	5,703	5,466
208007_at	6,978	6,266	6,717	7,103	7,020	7,133
208008_at	6,716	6,947	6,711	7,618	7,595	7,847
208009_s_at	8,548	8,450	8,561	8,339	8,068	8,486
208010_s_at	4,084	4,264	3,989	4,216	4,141	4,186
208011_at	4,030	4,054	3,930	4,031	3,974	3,941
208012_x_at	8,018	8,170	8,480	8,809	8,669	8,789
208013_s_at	6,165	6,049	6,043	6,527	6,661	6,542
208014_x_at	5,495	5,923	5,956	5,903	5,566	5,822
208015_at	6,823	6,403	6,626	6,218	6,415	6,128
208016_s_at	5,611	5,476	5,852	6,289	6,834	7,465
208017_s_at	5,205	5,936	5,390	4,912	4,953	5,168
208018_s_at	7,592	7,476	7,403	7,755	7,476	7,865
208019_at	4,202	4,240	4,140	3,901	3,862	3,885
208020_s_at	6,730	6,519	6,771	7,524	7,275	7,221
208021_s_at	9,797	10,097	9,488	8,016	8,913	7,488
208022_s_at	9,878	10,237	9,961	7,559	8,328	7,740
208023_at	5,883	6,057	6,112	5,932	6,058	6,118
208024_s_at	7,911	7,606	7,861	9,354	8,520	9,375
208025_s_at	12,948	12,249	12,356	10,512	11,416	9,976
208026_at	5,585	5,697	5,742	6,491	5,842	5,762
208027_s_at	5,525	5,053	5,400	5,815	5,828	5,864
208028_s_at	6,317	6,164	6,163	6,651	6,586	6,509
208029_s_at	13,570	13,602	13,786	10,787	10,174	10,303
208030_s_at	10,262	10,246	10,720	10,325	10,813	10,902
208031_s_at	8,284	7,635	7,921	8,826	8,386	8,735
208032_s_at	6,011	5,739	6,214	6,474	6,599	6,785
208033_s_at	6,492	6,148	6,216	7,026	7,140	7,325
208034_s_at	7,297	7,023	7,279	7,683	7,659	7,748
208035_at	6,920	7,176	6,772	7,528	7,479	7,401
208036_at	6,217	6,074	6,317	6,751	6,471	6,349
208037_s_at	7,321	7,671	7,405	7,867	7,752	8,024
208038_at	6,536	6,205	6,458	6,675	6,819	6,830
208039_at	7,585	7,337	7,394	7,841	7,721	7,671
208040_s_at	8,104	7,824	7,870	8,716	8,661	8,805

208041_at	6,591	6,416	6,530	6,963	7,070	7,222
208042_at	4,729	4,458	4,859	6,405	6,187	6,154
208043_at	5,836	5,498	6,050	6,136	6,226	6,290
208044_s_at	7,410	7,234	7,786	8,220	7,937	8,210
208045_at	4,238	4,546	4,299	4,516	4,511	4,595
208046_at	5,400	6,019	5,737	6,064	5,768	6,153
208047_s_at	4,390	4,539	3,884	3,918	3,805	4,300
208048_at	5,268	5,102	4,956	5,881	5,723	5,922
208049_s_at	5,793	5,690	6,051	6,492	6,221	6,277
208050_s_at	7,417	7,219	7,777	7,119	6,668	7,063
208051_s_at	11,528	11,306	11,274	8,386	8,664	8,674
208052_x_at	7,924	7,677	7,982	8,518	8,339	8,501
208053_at	5,604	5,542	5,835	5,662	5,634	5,582
208054_at	5,064	4,735	4,708	4,636	6,058	4,780
208055_s_at	6,593	6,465	6,034	6,557	7,412	6,683
208056_s_at	7,416	7,339	7,207	7,391	7,176	7,149
208057_s_at	5,502	5,519	5,484	6,684	6,759	6,606
208058_s_at	6,410	6,393	6,636	6,822	6,953	7,087
208059_at	6,777	6,449	6,659	7,357	7,223	7,092
208060_at	6,729	6,789	6,643	7,231	6,902	6,832
208061_at	6,194	6,226	5,820	6,541	6,507	6,619
208062_s_at	5,398	5,257	4,980	5,463	4,786	4,818
208063_s_at	7,043	6,916	7,149	7,013	6,892	7,152
208064_s_at	5,933	5,800	6,073	5,741	5,652	6,217
208065_at	6,407	5,891	7,115	5,465	5,266	5,587
208066_s_at	10,605	10,873	10,429	9,990	10,396	10,301
208067_x_at	7,221	7,163	7,141	7,417	7,493	7,431
208068_x_at	7,266	7,323	7,286	7,450	7,222	7,421
208069_x_at	7,308	7,443	7,293	6,818	6,748	7,031
208070_s_at	8,774	8,785	8,455	7,702	10,240	7,432
208071_s_at	7,238	7,328	7,181	8,461	8,335	8,086
208072_s_at	9,386	9,272	9,323	9,172	9,300	9,385
208073_x_at	11,359	11,600	10,662	9,485	11,680	10,014
208074_s_at	9,892	9,742	9,827	10,504	9,748	10,733
208075_s_at	5,945	6,277	6,274	7,606	7,097	6,652
208076_at	5,811	5,509	5,954	5,791	5,239	5,372
208077_at	5,648	5,116	5,749	5,132	5,171	4,943
208078_s_at	8,809	8,895	8,880	7,423	9,699	8,852
208079_s_at	12,176	12,602	12,365	11,218	11,102	10,668
208080_at	6,360	6,191	6,249	6,641	6,124	6,387
208081_s_at	5,833	6,612	5,952	6,021	5,659	5,535
208082_x_at	9,260	8,823	9,045	7,705	8,671	7,746
208083_s_at	4,728	4,651	4,724	5,584	5,669	5,937
208084_at	5,562	5,442	5,682	6,063	5,672	5,981
208085_s_at	6,305	6,109	6,095	6,587	6,466	6,377
208086_s_at	6,512	6,582	6,313	6,523	6,178	6,445
208087_s_at	6,407	6,556	6,538	6,887	6,941	6,894
208088_s_at	4,195	4,333	4,242	4,420	4,135	4,313
208089_s_at	7,910	8,160	6,993	6,448	6,734	6,227
208090_s_at	7,038	6,732	6,567	7,708	7,893	8,055
208091_s_at	11,792	11,732	12,137	11,366	11,232	11,712
208092_s_at	6,542	7,263	6,683	6,604	7,536	6,729
208093_s_at	9,407	9,687	9,494	10,194	10,866	10,994
208094_s_at	9,157	9,237	9,424	9,429	9,249	9,625
208095_s_at	11,244	11,406	11,089	10,883	11,803	11,047
208096_s_at	6,925	6,994	6,645	6,270	7,084	6,619

208097_s_at	9,721	9,222	8,898	8,709	7,954	8,655
208098_at	4,163	4,004	4,461	4,797	4,808	4,569
208099_x_at	7,982	7,413	7,148	7,253	7,274	7,497
208100_x_at	7,899	7,681	7,876	8,122	7,902	8,070
208101_s_at	9,465	9,192	9,722	9,592	9,603	9,691
208102_s_at	6,388	6,560	6,375	6,540	6,475	6,484
208103_s_at	11,366	11,114	10,807	10,125	10,341	9,443
208104_s_at	6,726	6,971	6,697	7,866	7,754	7,822
208105_at	6,273	6,235	6,161	6,449	6,088	6,312
208106_x_at	7,098	6,956	7,099	7,228	7,334	7,224
208107_s_at	9,148	9,571	8,954	8,485	8,401	8,225
208108_s_at	6,347	6,505	6,710	7,415	6,966	7,622
208109_s_at	7,073	6,754	6,549	5,681	6,815	5,668
208110_x_at	7,951	7,856	8,093	8,112	8,026	8,221
208111_at	6,650	6,801	6,308	7,469	7,371	7,572
208112_x_at	9,096	8,866	9,198	10,426	10,652	10,812
208113_x_at	13,301	13,237	13,416	12,554	13,112	12,965
208114_s_at	9,804	9,387	9,990	9,140	7,935	8,805
208115_x_at	6,697	6,869	6,746	6,606	6,812	6,802
208116_s_at	5,144	5,824	5,513	8,164	8,081	7,440
208117_s_at	10,533	10,180	10,586	9,345	8,937	9,041
208118_x_at	8,607	8,447	8,187	8,049	8,052	8,000
208119_s_at	9,316	9,332	8,944	6,800	6,971	6,869
208120_x_at	10,066	9,626	9,760	8,955	9,624	9,359
208121_s_at	6,542	5,931	5,996	5,624	5,531	5,666
208122_x_at	6,334	6,278	6,375	7,238	6,904	7,072
208123_at	5,575	5,772	5,390	5,942	5,804	5,968
208124_s_at	6,721	6,775	6,838	7,146	6,890	6,926
208126_s_at	5,590	5,012	5,955	6,329	6,024	6,103
208127_s_at	8,606	8,789	8,561	8,518	9,830	9,681
208128_x_at	7,547	7,547	7,830	8,439	8,384	8,463
208129_x_at	6,704	6,857	6,892	7,412	7,523	7,665
208130_s_at	7,174	6,578	6,826	7,389	7,035	7,345
208131_s_at	9,821	10,030	9,975	7,287	6,598	7,083
208132_x_at	9,047	8,611	9,072	8,358	8,363	8,410
208133_at	6,794	6,523	6,631	6,916	6,837	6,896
208134_x_at	5,138	6,024	5,693	6,399	6,036	6,192
208135_at	6,915	6,929	6,939	8,131	8,076	8,040
208136_s_at	5,239	5,045	5,166	5,142	5,106	5,333
208137_x_at	9,880	9,504	9,432	8,536	9,441	8,673
208138_at	7,578	7,266	7,674	8,170	8,256	8,296
208139_s_at	4,815	5,011	4,741	5,483	5,123	5,458
208140_s_at	5,913	5,267	5,237	6,092	5,583	5,867
208141_s_at	8,426	8,193	8,139	8,506	8,102	8,308
208142_at	4,553	4,687	4,514	4,656	4,268	4,619
208143_s_at	3,888	4,090	3,945	4,117	4,054	4,214
208144_s_at	4,980	5,708	5,615	5,068	5,049	5,070
208145_at	6,269	6,452	6,504	6,942	7,045	7,117
208146_s_at	10,028	10,127	10,293	6,594	6,369	6,415
208147_s_at	3,835	3,990	3,935	3,965	4,011	3,922
208148_at	5,072	5,095	4,635	5,816	5,589	5,997
208149_x_at	11,367	10,851	11,114	8,747	8,786	8,568
208151_x_at	9,858	9,370	9,491	8,985	8,510	8,660
208152_s_at	12,768	12,640	12,267	11,857	12,698	11,246
208153_s_at	5,991	5,968	6,166	5,915	5,709	5,941
208154_at	5,904	5,262	5,254	4,156	4,125	4,047

208155_x_at	6,718	6,586	6,735	7,052	6,689	6,647
208156_x_at	6,710	6,813	6,928	6,541	6,010	6,267
208157_at	6,562	6,395	6,247	6,806	6,650	6,539
208158_s_at	10,167	10,653	9,962	9,389	9,920	9,906
208159_x_at	10,615	10,367	10,957	8,976	8,872	8,874
208160_at	7,794	6,695	7,692	7,755	7,851	8,210
208161_s_at	6,463	7,408	7,005	10,333	10,608	10,934
208162_s_at	5,594	5,676	5,568	6,491	6,188	6,386
208163_s_at	6,512	6,658	6,381	7,102	7,245	7,185
208164_s_at	6,936	7,071	7,150	7,655	7,659	7,767
208165_s_at	6,238	7,012	7,238	5,580	5,471	5,701
208166_at	4,566	4,696	4,684	5,112	5,164	5,329
208167_s_at	5,090	5,148	4,937	5,717	5,568	5,677
208168_s_at	5,564	5,552	5,709	6,333	6,127	6,565
208169_s_at	6,181	5,940	6,101	6,488	6,420	6,554
208170_s_at	5,344	4,895	5,257	5,653	5,533	5,490
208172_s_at	6,692	6,182	6,288	7,404	6,972	7,262
208173_at	6,271	5,627	5,837	6,735	6,427	6,639
208174_x_at	8,743	8,670	8,541	8,309	8,160	8,155
208175_s_at	4,571	4,254	4,234	4,124	4,134	3,989
208176_at	7,541	7,600	7,814	8,286	7,995	8,221
208177_at	6,159	6,609	6,587	7,058	7,448	7,065
208178_x_at	7,373	6,945	7,547	8,015	8,856	7,465
208179_x_at	6,470	6,129	6,313	7,033	6,931	6,818
208180_s_at	6,469	6,540	6,172	6,636	6,670	7,278
208181_at	4,089	3,948	3,943	4,082	4,020	4,251
208182_x_at	5,149	4,962	5,212	5,599	5,576	5,699
208183_at	6,258	6,235	5,868	6,263	6,390	6,485
208184_s_at	8,279	8,254	8,530	8,052	8,093	8,027
208185_x_at	8,088	7,923	7,999	7,783	7,982	8,319
208186_s_at	7,700	7,644	7,790	8,241	7,573	7,736
208187_s_at	6,416	6,739	6,411	6,327	6,435	6,422
208188_at	7,669	7,508	7,795	8,492	8,141	8,155
208189_s_at	7,327	7,331	7,483	8,031	7,887	8,115
208190_s_at	8,505	8,123	8,318	7,264	6,400	6,997
208191_x_at	6,796	6,145	6,327	6,953	6,916	7,550
208192_at	3,755	3,663	3,630	3,767	3,646	3,643
208193_at	5,209	4,993	4,916	5,570	5,506	5,546
208194_s_at	7,044	7,290	7,033	7,776	7,640	7,482
208195_at	6,717	6,872	6,819	6,940	6,795	7,154
208196_x_at	7,250	7,517	7,247	8,028	7,908	8,006
208198_x_at	7,497	7,341	7,396	7,886	7,445	7,754
208199_s_at	6,447	6,245	6,266	7,227	7,452	7,598
208200_at	7,223	7,053	7,152	5,925	6,249	6,099
208201_at	6,544	6,547	6,366	7,466	7,688	7,847
208202_s_at	6,777	6,722	6,929	7,091	7,176	7,123
208203_x_at	7,021	6,790	7,049	7,631	7,384	7,424
208204_s_at	7,860	7,835	7,994	7,990	8,011	8,205
208205_at	5,864	6,278	6,280	5,846	5,765	5,974
208206_s_at	7,267	6,889	7,155	7,041	6,768	7,277
208207_at	6,234	6,175	5,773	6,648	6,389	6,401
208208_at	6,190	5,949	6,327	7,181	7,456	7,600
208209_s_at	6,244	6,657	6,693	7,677	7,671	7,673
208210_at	6,347	6,525	6,327	6,973	6,760	6,964
208211_s_at	6,292	6,040	6,246	6,059	5,916	6,068
208212_s_at	7,229	7,406	7,182	7,237	7,220	7,240

208213_s_at	6,395	6,262	6,367	6,399	6,598	6,402
208214_at	6,042	6,095	5,906	6,624	6,218	6,288
208215_x_at	6,440	6,695	6,509	6,443	6,261	6,444
208216_at	6,597	6,440	6,636	7,025	6,941	6,790
208217_at	7,062	7,044	7,218	7,501	7,379	7,569
208218_s_at	6,289	6,366	6,323	6,807	6,325	6,514
208219_at	6,601	6,452	6,581	6,874	6,566	6,646
208220_x_at	5,967	6,343	6,129	6,453	6,529	6,473
208221_s_at	6,234	6,205	6,095	6,645	6,279	6,632
208222_at	6,235	5,893	6,000	5,945	5,898	5,991
208223_s_at	5,920	5,942	5,630	6,082	5,650	6,114
208224_at	6,973	7,171	7,070	7,137	7,246	7,286
208225_at	5,884	6,176	6,233	6,517	6,374	6,381
208226_x_at	7,218	7,249	6,995	7,313	6,816	6,954
208227_x_at	7,743	7,478	7,850	7,180	7,332	7,427
208228_s_at	10,079	10,263	10,228	7,587	7,656	7,756
208229_at	5,617	5,946	6,380	6,160	5,709	6,012
208230_s_at	6,725	6,589	6,516	7,558	7,172	7,636
208231_at	4,154	4,014	4,138	4,306	4,351	4,397
208232_x_at	7,464	7,864	7,816	8,225	8,168	8,385
208233_at	5,221	5,642	5,369	5,486	5,146	5,206
208234_x_at	8,228	7,738	7,934	8,314	8,063	8,242
208235_x_at	5,920	6,026	5,696	6,354	5,932	6,268
208237_x_at	6,869	6,615	6,938	6,869	6,493	6,494
208238_x_at	9,595	9,469	9,593	9,219	10,251	9,664
208239_at	5,833	5,571	5,257	5,370	5,013	5,192
208240_s_at	6,231	6,024	5,856	6,548	6,519	6,663
208241_at	6,728	7,011	6,466	6,845	7,370	6,974
208242_at	7,601	7,470	7,574	7,492	7,451	7,397
208243_s_at	6,171	6,165	6,101	7,283	6,950	7,137
208244_at	6,213	6,250	6,214	6,387	6,033	6,210
208245_at	3,751	3,874	4,409	4,558	4,389	4,594
208246_x_at	10,381	9,958	10,203	8,898	10,173	9,261
208247_at	6,697	6,307	6,662	6,685	6,700	6,893
208248_x_at	11,340	11,178	11,049	9,737	10,784	10,324
208249_s_at	9,215	9,492	8,766	8,283	8,219	7,960
208250_s_at	8,030	8,364	8,423	8,609	8,101	8,677
208251_at	6,648	6,822	6,598	7,514	7,322	7,443
208252_s_at	7,098	6,903	6,474	7,920	7,613	8,047
208253_at	5,510	5,779	5,518	6,722	6,291	6,764
208254_at	6,384	6,206	6,600	6,775	6,435	6,481
208255_s_at	7,487	7,449	7,401	7,379	7,026	7,343
208256_at	6,468	6,013	6,250	6,708	6,482	6,594
208257_x_at	7,894	7,998	8,194	8,386	8,081	8,448
208258_s_at	7,329	7,317	7,386	8,239	7,927	8,359
208259_x_at	6,106	6,069	6,173	6,606	6,524	6,542
208260_at	8,380	8,507	8,175	8,593	8,485	8,528
208261_x_at	5,261	5,008	5,304	5,539	5,546	5,682
208262_x_at	6,023	6,400	6,498	6,143	6,218	6,547
208263_at	4,251	4,439	4,566	4,134	4,001	4,108
208264_s_at	9,503	9,522	9,040	8,999	9,745	8,728
208265_at	5,160	5,211	5,238	5,440	5,383	5,181
208266_at	5,467	5,333	5,706	5,551	5,479	5,641
208267_at	7,122	7,144	7,013	7,528	7,211	7,372
208268_at	4,355	4,609	4,512	4,458	4,341	4,690
208269_s_at	7,348	7,106	7,257	7,689	7,427	7,636

208270_s_at	10,625	10,723	10,646	10,385	10,314	10,501
208271_at	4,670	4,665	4,874	5,538	5,131	5,599
208272_at	7,301	7,279	7,528	7,990	7,553	7,887
208273_at	5,356	5,545	5,431	4,525	4,589	4,491
208274_at	5,731	6,331	6,011	6,423	6,313	6,432
208275_x_at	7,249	7,171	7,481	6,720	6,334	6,705
208276_at	5,946	5,951	5,844	6,150	6,232	6,299
208277_at	7,534	7,490	7,550	7,868	7,380	7,701
208278_s_at	6,017	5,945	5,873	6,809	6,545	6,718
208279_s_at	6,995	6,697	6,806	7,232	7,070	7,312
208280_at	4,860	5,094	5,246	5,149	4,988	5,056
208281_x_at	5,255	5,405	5,409	5,831	5,587	5,520
208282_x_at	4,377	4,538	4,585	4,834	4,858	4,820
208283_at	5,761	5,999	5,747	6,733	6,105	6,443
208284_x_at	8,346	8,177	8,312	7,937	7,387	7,764
208285_at	6,337	6,235	6,164	6,652	6,537	6,715
208286_x_at	10,820	10,864	11,070	8,391	8,385	8,418
208287_at	6,910	6,956	6,839	6,922	7,046	7,402
208288_at	4,457	4,727	4,743	4,925	4,462	4,637
208289_s_at	10,136	10,106	10,517	10,499	9,966	10,408
208290_s_at	10,399	10,143	10,254	9,819	9,667	9,349
208291_s_at	6,764	6,645	6,262	7,053	6,679	7,103
208292_at	6,151	5,930	5,939	6,730	6,427	6,845
208293_x_at	6,395	6,224	6,386	6,676	6,578	6,983
208294_x_at	5,951	6,589	6,491	6,539	6,355	6,615
208295_x_at	6,396	6,791	6,730	6,745	6,729	6,914
208296_x_at	9,116	9,354	9,158	8,895	8,462	7,961
208297_s_at	6,418	6,735	6,391	6,587	6,597	6,619
208298_at	5,335	5,316	5,576	5,928	5,728	6,060
208299_at	7,187	6,865	6,930	7,622	7,753	7,710
208300_at	6,508	6,377	6,121	6,860	6,722	6,698
208301_at	7,131	6,504	6,878	7,631	7,471	7,702
208302_at	5,336	4,946	4,877	6,281	5,906	6,388
208303_s_at	6,128	6,318	6,529	6,398	6,416	6,429
208304_at	5,524	4,920	5,280	5,782	5,819	6,092
208305_at	4,465	4,783	4,798	4,922	5,052	5,096
208306_x_at	8,049	8,265	8,145	8,118	7,771	8,085
208307_at	5,915	5,671	5,789	6,455	6,722	6,308
208308_s_at	10,549	10,233	10,763	11,418	10,857	11,663
208309_s_at	7,190	7,888	6,749	6,511	6,430	6,363
208310_s_at	10,460	10,201	9,668	8,231	8,152	8,359
208311_at	6,341	6,388	6,145	6,912	6,647	6,700
208312_s_at	6,672	6,694	6,741	7,116	6,865	6,881
208313_s_at	11,415	11,211	11,477	10,223	10,290	10,208
208314_at	5,570	5,618	6,287	6,273	5,883	6,209
208315_x_at	7,330	7,499	7,702	7,816	7,452	7,887
208316_s_at	7,176	7,138	7,319	7,559	7,627	7,722
208317_at	5,731	5,456	5,962	6,067	5,901	6,268
208318_s_at	6,806	6,830	6,671	6,905	6,449	6,965
208319_s_at	9,800	9,408	9,695	10,485	10,182	10,505
208320_at	6,354	6,110	5,724	6,206	5,964	6,357
208321_s_at	7,154	6,993	7,017	7,225	6,911	7,010
208322_s_at	7,324	7,151	7,399	8,665	8,625	8,817
208323_s_at	6,855	6,472	6,304	6,445	6,314	6,746
208324_at	4,657	4,238	4,555	4,972	4,781	5,152
208325_s_at	7,416	7,406	7,614	7,160	6,864	7,087

208327_at	6,867	6,403	6,793	6,570	6,503	6,581
208328_s_at	6,792	6,846	6,793	5,710	7,154	6,498
208329_at	4,562	4,548	4,517	5,027	4,872	4,933
208330_at	7,345	7,188	7,053	7,330	7,515	7,524
208331_at	5,381	5,127	5,186	5,840	5,724	5,736
208332_at	7,396	7,320	7,398	8,253	8,051	8,181
208333_at	7,775	7,289	7,578	7,937	7,479	7,678
208334_at	6,820	6,498	6,504	7,511	7,242	7,454
208335_s_at	6,459	6,415	6,403	6,917	7,256	7,033
208336_s_at	10,559	10,281	10,624	9,801	9,482	10,323
208337_s_at	5,684	6,305	6,140	5,903	6,040	6,136
208338_at	5,819	5,444	5,919	7,008	7,114	6,940
208339_at	4,345	4,469	4,513	4,657	4,450	4,573
208340_at	4,779	4,396	4,491	5,472	5,405	5,572
208341_x_at	6,409	6,645	6,632	6,988	6,607	6,864
208342_x_at	6,795	6,584	6,747	6,675	6,484	6,716
208343_s_at	5,046	4,894	5,312	4,023	3,961	4,197
208344_x_at	7,075	6,983	7,317	8,132	7,393	7,643
208345_s_at	6,920	7,039	7,124	6,683	6,333	6,244
208346_at	5,564	5,398	5,286	5,810	5,757	5,938
208347_at	7,348	6,950	7,453	7,836	7,166	7,334
208348_s_at	4,389	4,461	4,292	4,622	4,768	4,793
208349_at	5,453	5,443	5,307	5,393	5,408	5,524
208350_at	5,001	5,079	5,186	5,386	6,024	5,364
208351_s_at	7,226	7,339	7,017	7,571	7,183	7,821
208352_x_at	7,608	6,854	7,289	8,308	8,054	8,307
208353_x_at	6,236	6,698	6,840	7,512	7,394	7,704
208354_s_at	6,529	6,387	6,461	7,270	6,983	7,007
208356_x_at	6,393	6,695	6,608	6,934	6,338	6,784
208357_x_at	6,500	6,784	6,825	6,676	6,601	6,550
208358_s_at	6,461	6,506	5,801	5,336	5,283	5,216
208359_s_at	6,222	6,690	6,111	5,843	5,442	5,723
208360_s_at	7,453	7,089	7,998	7,209	7,098	7,192
208361_s_at	9,481	9,352	9,598	9,180	8,673	9,409
208363_s_at	6,771	6,552	7,008	6,883	6,645	6,882
208364_at	6,540	6,530	6,223	7,094	6,905	7,071
208365_s_at	7,044	7,054	6,619	6,604	6,397	6,667
208366_at	5,403	5,208	6,019	6,219	5,917	6,201
208367_x_at	5,959	5,957	6,343	6,509	6,168	6,473
208368_s_at	7,236	7,151	6,368	5,717	6,020	5,204
208369_s_at	8,770	8,622	8,657	8,053	7,842	7,731
208370_s_at	9,218	9,257	8,886	9,000	9,233	9,598
208371_s_at	8,910	8,617	8,800	9,537	9,571	10,002
208372_s_at	6,358	6,089	6,324	7,291	7,214	7,240
208373_s_at	6,365	6,650	6,436	6,900	6,886	6,897
208374_s_at	12,766	12,798	12,752	12,504	13,148	13,004
208375_at	4,026	4,038	4,521	4,402	4,637	4,645
208376_at	5,384	5,794	5,675	6,038	5,745	5,801
208377_s_at	7,467	7,476	7,257	7,499	7,623	7,617
208378_x_at	6,492	6,661	6,173	7,287	8,192	8,274
208379_x_at	6,106	6,000	6,232	6,672	6,857	6,620
208380_at	6,426	5,871	6,323	6,567	5,841	6,518
208381_s_at	6,180	6,160	6,210	6,641	6,359	6,668
208382_s_at	5,973	6,168	6,001	5,847	5,529	5,492
208383_s_at	4,326	4,755	4,367	4,572	4,724	4,538
208384_s_at	8,130	8,119	8,127	9,418	9,059	9,343

208385_at	6,227	6,151	6,521	6,349	6,311	6,380
208386_x_at	6,803	6,585	6,570	6,407	6,726	6,402
208387_s_at	6,449	6,725	6,659	7,145	6,964	7,042
208388_at	6,621	6,310	6,321	6,604	6,719	6,665
208389_s_at	7,469	6,924	6,855	7,331	7,105	7,239
208390_s_at	7,757	7,745	7,773	8,476	7,715	8,097
208391_s_at	6,570	5,981	6,323	6,982	6,839	6,688
208392_x_at	7,040	7,484	7,222	7,877	8,113	7,935
208393_s_at	9,431	10,135	9,007	8,164	9,263	7,048
208394_x_at	4,296	5,128	4,453	7,466	6,606	5,785
208395_s_at	6,895	6,859	6,981	6,585	6,401	6,790
208396_s_at	5,487	5,337	5,415	5,773	8,099	5,603
208397_x_at	8,141	7,901	8,222	9,257	8,662	8,946
208398_s_at	10,708	10,860	10,648	10,320	10,430	10,249
208399_s_at	6,132	6,257	6,224	5,645	5,546	5,664
208400_at	6,438	6,406	6,569	7,323	7,282	7,376
208401_s_at	6,609	6,815	7,053	8,163	8,176	8,389
208402_at	6,252	6,223	6,315	6,880	6,730	6,915
208403_x_at	6,926	6,825	6,982	7,147	7,040	7,338
208404_x_at	7,020	6,910	6,919	7,041	7,130	7,192
208405_s_at	11,284	11,531	11,078	12,208	12,939	12,541
208406_s_at	7,070	7,094	6,866	7,870	7,593	7,820
208407_s_at	9,294	9,857	9,497	8,368	9,659	9,111
208408_at	6,368	6,421	6,421	6,722	6,323	6,688
208409_at	6,651	6,530	6,574	6,779	6,385	6,635
208410_x_at	6,727	6,817	6,851	7,275	7,113	7,065
208411_x_at	3,553	3,805	3,610	3,749	3,695	3,854
208412_s_at	5,794	5,998	6,420	6,852	6,696	6,753
208413_at	4,781	4,783	4,901	4,884	4,857	4,931
208414_s_at	6,565	6,855	6,773	7,485	7,148	7,192
208415_x_at	9,211	9,447	9,298	8,699	8,441	8,531
208416_s_at	7,277	7,206	6,983	7,776	7,768	7,959
208417_at	7,425	7,216	7,697	8,208	7,850	8,311
208420_x_at	8,432	8,097	8,616	8,141	8,303	8,327
208421_at	5,586	5,248	5,441	5,900	5,482	5,712
208422_at	4,414	4,260	4,451	5,024	4,986	4,840
208423_s_at	5,693	5,557	5,327	5,982	5,801	5,768
208424_s_at	10,594	10,794	11,167	10,253	9,890	9,892
208425_s_at	5,771	5,744	6,082	6,064	6,637	6,579
208426_x_at	6,083	6,245	6,047	6,374	6,268	6,405
208427_s_at	5,166	5,428	5,229	4,979	4,654	4,631
208428_at	5,588	5,486	5,465	5,784	5,308	5,654
208429_x_at	6,482	6,682	6,552	7,534	6,609	7,389
208430_s_at	6,581	6,477	6,413	6,653	6,606	6,657
208431_s_at	6,895	6,672	6,691	7,331	7,137	7,356
208432_s_at	6,994	6,364	6,167	7,149	7,140	7,205
208433_s_at	9,828	9,405	9,128	8,482	8,336	8,770
208434_at	6,131	5,970	6,261	6,156	5,835	5,777
208435_s_at	6,894	6,459	6,494	6,988	7,051	7,102
208436_s_at	6,237	6,312	6,533	7,575	7,200	7,487
208437_at	5,776	6,391	5,981	7,066	7,290	7,420
208438_s_at	6,178	6,374	6,171	6,466	6,292	6,286
208439_s_at	6,631	6,590	6,514	7,092	7,051	7,112
208440_at	7,165	6,690	6,873	7,385	7,359	7,437
208441_at	6,702	6,711	6,645	6,954	6,719	6,798
208442_s_at	6,586	6,070	6,022	6,550	6,658	6,799

208443_x_at	5,675	6,184	6,963	7,868	7,591	7,457
208445_s_at	10,333	10,040	10,148	8,907	9,438	8,412
208446_s_at	6,035	5,787	5,943	5,970	6,054	6,320
208447_s_at	9,922	9,678	9,440	8,419	7,444	8,224
208448_x_at	4,533	4,445	4,485	5,074	4,975	5,087
208449_s_at	6,725	6,168	6,552	7,331	7,247	7,707
208450_at	6,490	6,480	6,717	6,830	6,714	6,814
208451_s_at	7,545	7,431	7,436	7,695	7,595	7,893
208452_x_at	7,097	6,738	7,083	8,223	7,485	7,873
208453_s_at	8,708	8,647	8,625	8,409	8,390	9,048
208454_s_at	7,652	7,545	7,274	8,124	8,368	8,628
208455_at	6,204	6,148	6,383	7,044	7,037	7,116
208456_s_at	8,492	8,083	8,037	8,416	8,818	9,722
208457_at	6,622	6,923	6,775	6,853	6,954	6,857
208458_at	6,797	6,725	6,824	7,265	6,777	7,138
208459_s_at	8,515	8,185	7,451	6,825	6,841	7,012
208460_at	8,368	8,153	8,300	7,503	6,937	6,976
208461_at	5,552	5,589	5,604	6,031	5,864	5,919
208462_s_at	5,231	5,497	5,342	5,295	5,458	5,057
208463_at	5,775	5,223	5,807	6,231	5,970	6,143
208464_at	6,418	5,830	6,603	7,754	7,268	7,384
208465_at	7,233	7,012	7,239	6,926	6,714	7,030
208466_at	7,741	7,702	7,604	8,156	7,980	8,074
208467_at	5,913	5,417	5,778	6,169	6,033	6,134
208468_at	6,524	6,458	6,512	6,630	6,263	6,365
208469_s_at	6,937	6,311	6,840	7,300	7,395	7,410
208470_s_at	5,595	5,644	5,561	6,530	6,739	6,709
208471_at	6,901	6,941	7,007	7,658	7,492	7,871
208472_at	7,659	8,206	8,195	8,165	8,127	7,882
208473_s_at	6,421	6,103	6,218	6,940	6,452	6,768
208474_at	9,299	8,735	9,203	6,875	6,812	7,029
208475_at	7,215	7,178	7,590	7,211	7,482	7,055
208476_s_at	6,998	6,921	7,329	7,276	7,336	7,332
208477_at	6,884	6,404	6,397	6,374	6,057	6,418
208478_s_at	6,754	6,888	6,957	7,335	6,612	6,992
208479_at	6,710	6,722	6,598	7,005	6,833	6,853
208480_s_at	6,668	6,345	6,380	7,038	7,114	7,154
208481_at	4,896	4,809	4,707	5,763	5,423	5,498
208482_at	6,048	6,358	6,112	7,249	6,947	7,249
208483_x_at	6,289	5,998	6,097	7,789	7,354	7,745
208484_at	6,298	6,316	6,141	7,572	7,173	7,660
208485_x_at	6,590	6,306	6,387	6,855	6,906	7,047
208486_at	6,657	6,880	6,796	7,135	7,044	6,929
208487_at	7,289	7,165	7,024	8,017	7,657	7,951
208488_s_at	7,858	7,703	7,714	8,371	8,476	8,641
208489_at	6,718	6,442	6,490	7,491	7,208	7,672
208490_x_at	7,359	7,360	7,154	7,276	7,289	7,702
208491_s_at	7,024	7,042	6,652	7,089	7,023	7,037
208492_at	7,139	6,930	6,547	6,702	6,684	6,473
208493_at	5,991	5,658	5,809	7,010	6,739	6,942
208494_at	7,030	6,929	7,011	7,227	7,281	7,272
208495_at	6,388	6,000	6,310	6,760	6,100	6,515
208496_x_at	7,504	7,791	7,444	7,238	7,246	7,214
208497_x_at	7,253	6,654	6,870	7,804	7,610	8,049
208498_s_at	5,265	5,901	4,880	6,132	6,490	6,049
208499_s_at	6,838	7,100	7,066	8,698	7,952	7,922

208500_x_at	5,161	5,564	5,446	5,430	5,116	5,355
208501_at	5,546	5,592	5,979	6,210	5,584	5,994
208502_s_at	6,091	5,919	5,808	6,748	6,457	6,784
208503_s_at	8,768	8,733	8,495	8,245	7,538	7,953
208504_x_at	7,671	7,176	7,394	6,911	6,693	6,693
208505_s_at	6,573	6,616	6,744	6,699	6,642	6,942
208506_at	4,471	4,444	4,384	4,593	4,273	4,571
208507_at	7,425	7,284	7,290	7,819	7,729	7,756
208508_s_at	3,980	4,158	4,031	3,958	3,845	3,971
208509_s_at	5,699	6,422	6,006	6,296	6,159	6,169
208510_s_at	6,239	6,445	5,811	8,777	8,879	9,151
208511_at	7,785	7,915	8,093	8,968	7,989	8,074
208512_s_at	5,514	5,648	5,453	6,651	6,621	6,767
208513_at	6,319	5,847	5,524	6,289	5,797	5,857
208514_at	6,236	6,128	6,194	7,064	6,373	6,887
208515_at	4,233	3,900	4,054	4,775	4,948	4,775
208516_at	6,634	6,588	6,525	6,805	6,815	6,779
208517_x_at	13,235	13,436	13,221	12,721	12,641	12,726
208518_s_at	6,815	6,704	6,718	7,059	6,803	6,735
208519_x_at	7,819	7,194	7,632	8,248	8,473	8,435
208520_at	5,664	6,002	5,946	6,243	6,203	6,193
208521_at	5,661	5,763	5,555	6,077	5,970	5,605
208522_s_at	6,466	6,367	6,592	7,039	6,924	6,995
208523_x_at	6,813	5,948	6,488	6,981	6,760	7,234
208524_at	5,887	5,835	6,039	5,976	5,748	6,175
208525_s_at	4,867	4,816	5,130	5,250	5,153	5,466
208526_at	6,398	6,347	6,567	6,880	6,522	7,004
208527_x_at	7,155	6,771	7,343	7,124	7,160	7,802
208528_x_at	7,270	7,453	7,213	7,295	7,183	7,327
208529_at	4,821	4,996	4,773	6,083	6,482	6,570
208530_s_at	6,946	6,952	6,948	7,992	7,542	8,005
208531_at	5,100	5,320	5,393	5,782	5,166	5,370
208532_x_at	8,535	8,753	8,635	8,957	8,879	8,910
208533_at	5,597	5,468	5,636	6,508	6,550	6,717
208534_s_at	6,295	6,193	6,218	7,544	7,371	7,426
208535_x_at	8,171	7,824	7,773	8,470	8,208	8,516
208536_s_at	5,326	5,376	5,696	5,555	5,255	5,574
208537_at	6,665	6,495	6,705	7,016	6,988	7,286
208538_at	5,424	5,284	5,333	5,836	5,773	5,865
208539_x_at	7,241	7,156	7,403	7,870	7,873	7,815
208540_x_at	8,811	8,688	8,745	9,729	9,214	9,959
208541_x_at	8,043	8,489	8,249	8,382	8,271	8,344
208542_x_at	6,943	7,234	6,626	4,426	4,348	4,725
208543_at	4,649	4,932	4,645	4,797	4,757	4,767
208544_at	7,220	7,319	7,464	7,559	7,543	7,679
208545_x_at	6,690	6,484	6,976	7,616	7,809	7,926
208546_x_at	6,305	5,803	5,752	5,534	5,613	6,424
208547_at	5,072	5,432	5,001	5,892	5,629	5,924
208548_at	5,894	5,982	6,063	6,184	6,183	6,352
208549_x_at	11,744	11,472	11,439	11,609	11,101	11,037
208550_x_at	5,451	6,131	5,573	6,992	7,185	7,397
208551_at	6,531	6,243	6,406	6,275	6,213	6,317
208552_at	6,217	6,131	6,492	7,108	7,197	7,305
208553_at	7,383	7,027	7,031	7,035	6,787	7,028
208554_at	5,782	5,065	5,725	6,361	6,204	6,670
208555_x_at	6,741	6,532	6,495	7,213	6,980	7,496

208556_at	5,768	5,953	5,978	6,227	6,076	6,049
208557_at	6,638	6,798	6,888	7,650	7,402	7,545
208558_at	7,090	7,124	7,235	8,366	8,619	8,414
208559_at	6,695	6,472	6,691	7,195	6,994	7,402
208560_at	6,215	6,396	6,440	7,175	7,035	7,389
208561_at	4,434	4,470	4,423	5,495	5,177	5,437
208562_s_at	6,228	5,852	6,045	6,891	6,954	7,079
208563_x_at	5,636	5,544	5,462	5,853	5,669	5,722
208564_at	6,379	6,566	6,922	6,720	6,458	6,569
208565_at	4,933	4,682	5,112	5,345	5,203	5,142
208566_at	4,357	4,399	4,311	4,963	4,784	4,979
208567_s_at	5,869	5,349	5,522	5,720	5,654	5,775
208568_at	7,693	7,518	7,492	8,034	7,939	7,901
208569_at	7,290	7,064	7,297	7,633	7,440	7,673
208570_at	6,731	6,373	6,434	6,527	6,193	6,471
208571_at	5,624	5,401	5,510	6,254	5,723	5,626
208572_at	6,204	6,323	6,383	6,541	6,255	6,466
208573_s_at	7,150	6,770	7,152	7,258	7,297	7,194
208574_at	5,497	5,414	5,155	6,128	6,253	6,152
208575_at	4,281	4,571	4,535	4,751	4,659	5,095
208576_s_at	6,146	5,986	5,892	6,097	5,702	6,066
208577_at	6,895	6,822	6,847	7,018	6,696	6,742
208578_at	6,860	6,563	6,861	7,403	7,070	6,921
208579_x_at	7,037	6,722	6,737	7,720	7,969	8,539
208580_x_at	8,400	8,686	8,485	7,740	6,997	7,153
208581_x_at	11,461	11,079	12,642	13,445	13,049	13,776
208582_s_at	5,973	5,030	5,294	6,552	6,643	6,513
208583_x_at	8,416	8,861	8,647	8,645	8,458	8,994
208584_at	6,261	6,374	6,469	8,346	8,331	8,568
208585_at	7,096	7,141	6,738	7,771	7,137	7,296
208586_s_at	4,062	3,962	4,301	4,621	4,548	4,888
208587_s_at	7,202	7,092	7,664	8,266	7,570	7,913
208588_at	5,880	5,787	5,454	6,496	6,367	6,754
208589_at	5,894	6,407	6,058	6,582	6,408	6,507
208590_x_at	7,462	7,071	7,313	7,620	7,631	7,843
208591_s_at	7,601	7,681	7,915	7,449	7,815	7,693
208592_s_at	6,711	6,775	6,586	6,796	6,635	6,625
208593_x_at	6,480	6,839	6,951	7,513	7,538	7,544
208594_x_at	6,555	6,376	6,472	6,797	6,712	6,856
208595_s_at	8,899	8,858	9,117	9,615	9,590	9,490
208596_s_at	6,730	6,420	6,784	7,685	7,350	7,699
208597_at	5,999	6,160	5,774	6,417	6,282	6,181
208598_s_at	10,342	10,512	10,772	8,738	9,591	9,208
208599_at	6,937	7,081	7,041	7,179	7,006	7,268
208600_s_at	5,821	5,853	5,712	5,823	6,012	5,825
208601_s_at	6,817	6,948	6,703	7,007	6,915	6,831
208602_x_at	5,925	5,409	5,901	6,572	6,700	6,939
208603_s_at	7,343	7,133	7,394	7,868	7,742	7,950
208604_s_at	7,246	7,279	7,202	7,767	7,638	7,798
208605_s_at	5,647	5,471	5,256	5,757	5,883	6,090
208606_s_at	6,637	6,597	6,495	6,894	6,414	6,650
208607_s_at	5,192	6,024	5,828	6,376	6,120	6,373
208608_s_at	6,186	5,934	6,210	6,503	6,353	6,426
208609_s_at	6,985	6,976	7,497	7,718	7,810	7,999
208610_s_at	11,354	10,996	11,565	9,494	9,635	10,201
208611_s_at	9,603	9,611	9,701	8,160	9,172	8,902

208612_at	11,159	10,921	10,838	11,080	11,012	11,381
208613_s_at	8,336	8,604	8,560	9,272	9,062	9,461
208614_s_at	10,219	10,710	10,550	9,763	9,901	9,960
208615_s_at	10,991	10,855	11,052	10,316	10,854	10,981
208616_s_at	12,984	13,053	13,022	12,943	12,998	13,121
208617_s_at	12,305	12,216	12,203	10,929	11,686	11,425
208619_at	10,869	10,860	10,881	9,676	9,584	10,007
208620_at	12,179	12,136	12,407	12,073	11,249	11,917
208621_s_at	8,204	8,219	8,724	7,398	6,690	7,709
208622_s_at	9,502	9,544	9,787	8,418	8,468	8,435
208623_s_at	10,015	9,541	9,638	8,452	8,598	8,737
208624_s_at	10,330	9,947	10,878	9,826	9,531	10,190
208625_s_at	9,694	9,243	9,797	9,514	9,509	9,818
208626_s_at	10,168	10,439	10,287	10,609	10,583	10,826
208627_s_at	13,404	13,184	12,934	12,190	11,491	12,494
208628_s_at	14,261	13,968	13,865	13,159	13,163	13,463
208629_s_at	9,348	9,513	9,742	9,879	9,593	10,330
208630_at	10,812	10,939	11,120	10,780	11,278	11,230
208631_s_at	9,999	10,149	10,024	9,699	10,239	10,187
208632_at	9,744	9,632	10,104	9,139	9,610	9,577
208633_s_at	8,838	8,406	8,454	7,491	8,173	7,733
208634_s_at	11,545	11,484	11,744	9,509	11,036	9,305
208635_x_at	13,914	14,041	13,690	12,956	12,939	13,061
208636_at	11,555	11,509	12,024	12,231	12,124	12,608
208637_x_at	9,527	9,355	9,540	10,770	10,032	11,271
208638_at	12,751	12,851	12,823	12,216	12,490	12,302
208639_x_at	12,092	11,966	11,842	11,438	11,496	11,461
208640_at	14,161	14,240	14,238	12,979	13,553	13,394
208641_s_at	12,834	12,884	12,973	12,215	12,163	12,460
208642_s_at	12,898	12,834	12,530	11,353	11,679	10,909
208643_s_at	13,088	12,870	12,736	10,204	11,078	9,788
208644_at	11,510	11,558	11,615	9,687	9,299	9,322
208645_s_at	13,613	13,711	13,697	13,079	12,955	13,427
208646_at	12,408	12,799	12,685	12,303	12,228	12,461
208647_at	13,596	13,964	13,658	12,822	12,588	12,936
208648_at	11,607	11,613	12,013	10,877	10,606	10,877
208649_s_at	11,770	11,563	11,732	11,130	10,809	11,121
208650_s_at	11,570	11,553	11,897	4,453	4,422	4,484
208651_x_at	12,255	11,948	12,157	5,863	5,923	6,188
208652_at	11,172	10,641	10,599	10,330	10,403	10,182
208653_s_at	6,686	6,716	6,528	9,984	9,628	10,128
208654_s_at	8,821	8,859	8,366	10,155	10,534	10,368
208655_at	12,193	12,313	12,188	11,521	11,639	11,761
208656_s_at	11,437	11,737	11,665	10,830	10,965	11,218
208657_s_at	8,376	8,020	8,328	7,889	8,262	8,191
208658_at	10,728	10,681	11,347	9,888	9,808	9,865
208659_at	11,941	12,204	12,119	13,017	12,559	13,141
208660_at	12,081	12,026	12,448	11,966	11,826	12,107
208661_s_at	11,033	11,299	10,504	9,333	11,308	10,143
208662_s_at	11,262	11,280	10,504	9,552	11,623	10,055
208663_s_at	10,044	10,367	9,402	8,167	9,787	8,524
208664_s_at	8,142	8,668	8,042	7,464	7,717	7,753
208666_s_at	10,147	10,128	10,061	8,681	8,311	8,157
208667_s_at	11,249	11,536	11,321	10,408	10,717	9,700
208668_x_at	13,948	13,608	14,025	12,998	12,978	12,953
208669_s_at	3,495	4,359	3,774	10,573	11,123	11,213

208670_s_at	6,095	5,633	6,564	9,173	8,937	9,406
208671_at	9,870	9,986	9,250	9,719	11,338	10,091
208672_s_at	13,211	13,084	13,001	12,160	12,274	12,313
208673_s_at	11,090	10,872	10,781	9,714	9,928	9,874
208674_x_at	11,151	11,361	11,629	11,561	11,433	11,731
208675_s_at	10,686	10,574	10,746	10,829	10,227	11,095
208676_s_at	10,939	10,585	11,010	10,701	10,310	10,616
208677_s_at	10,498	10,590	10,778	9,502	8,971	9,787
208678_at	10,679	11,203	10,660	11,411	11,658	11,699
208679_s_at	12,228	11,920	11,935	12,337	12,042	12,781
208680_at	14,196	14,195	14,113	13,033	13,026	13,534
208682_s_at	10,715	10,953	10,661	10,435	10,277	11,060
208683_at	11,406	11,901	11,664	12,716	13,820	13,268
208684_at	10,885	11,081	10,900	10,679	10,750	11,005
208685_x_at	10,074	9,916	10,088	9,604	9,542	10,260
208686_s_at	7,727	7,690	7,684	6,781	7,227	7,423
208687_x_at	13,548	13,330	13,634	13,358	13,329	13,295
208688_x_at	13,007	12,557	12,988	12,246	12,277	12,489
208689_s_at	11,466	11,595	11,733	10,945	11,169	11,505
208690_s_at	10,371	10,559	10,715	11,966	11,254	12,287
208691_at	13,896	13,869	13,755	13,207	13,944	13,177
208692_at	14,105	14,252	14,100	13,628	13,509	13,783
208693_s_at	13,562	13,836	13,803	12,547	13,109	13,152
208694_at	10,960	10,531	10,495	8,047	9,202	7,999
208695_s_at	14,619	14,637	14,603	13,897	14,094	14,182
208696_at	12,327	12,264	12,148	11,700	12,075	11,551
208697_s_at	13,625	14,034	13,536	12,445	13,121	13,077
208698_s_at	10,760	10,449	10,343	9,822	8,881	9,258
208699_x_at	10,845	10,817	10,953	10,238	9,555	10,249
208700_s_at	12,116	12,528	12,504	11,496	11,058	11,638
208701_at	6,130	6,350	6,491	6,110	6,061	6,285
208702_x_at	9,512	9,195	9,274	8,620	9,730	9,044
208703_s_at	9,918	9,567	9,409	8,114	8,898	8,446
208704_x_at	11,408	11,177	11,104	9,866	10,747	10,434
208705_s_at	11,437	11,435	11,315	10,683	11,445	10,542
208706_s_at	11,988	11,848	11,412	10,545	11,378	10,186
208707_at	8,665	9,062	8,655	6,712	7,841	7,000
208708_x_at	11,607	11,442	11,551	11,443	11,474	11,091
208709_s_at	11,394	11,378	11,079	9,599	10,374	9,659
208710_s_at	10,054	9,855	10,508	10,238	9,694	10,570
208711_s_at	9,326	8,708	8,951	9,166	9,234	10,712
208712_at	11,366	10,894	11,196	10,553	11,385	11,635
208713_at	9,717	9,627	10,174	9,629	9,334	9,496
208714_at	11,057	10,678	10,872	10,984	10,600	11,217
208715_at	9,886	9,887	9,930	8,765	9,770	9,671
208716_s_at	11,408	11,360	11,532	11,077	11,694	11,935
208717_at	9,788	9,843	9,789	10,635	10,039	10,346
208718_at	13,879	13,829	13,974	12,427	12,913	12,594
208719_s_at	6,953	6,501	6,183	5,840	5,484	5,825
208720_s_at	11,423	11,638	11,115	9,942	11,184	10,318
208721_s_at	6,993	7,012	6,799	7,277	6,646	7,116
208722_s_at	10,267	9,913	10,051	9,498	9,491	9,831
208723_at	10,970	10,668	11,002	9,149	8,749	9,422
208724_s_at	13,347	13,448	13,114	13,006	13,624	13,343
208725_at	9,617	10,102	10,020	8,804	8,677	8,539
208726_s_at	12,304	12,854	12,374	11,413	12,183	11,619

208727_s_at	9,424	9,586	9,470	11,030	10,853	11,183
208728_s_at	10,383	10,604	10,435	10,745	10,868	10,952
208729_x_at	9,168	9,381	9,119	10,462	9,776	10,322
208730_x_at	9,452	9,588	9,814	10,520	10,776	10,897
208731_at	10,634	10,950	10,596	11,173	11,323	11,633
208732_at	9,348	9,646	9,592	9,242	9,766	9,649
208733_at	6,202	6,890	6,269	6,452	6,260	6,072
208734_x_at	10,021	10,322	10,033	11,203	10,459	11,280
208735_s_at	9,246	9,203	9,629	9,632	8,908	9,322
208736_at	10,590	10,535	10,024	11,591	11,294	11,916
208737_at	11,921	12,196	11,808	11,073	10,471	11,301
208738_x_at	12,755	12,690	12,453	12,240	12,176	12,459
208739_x_at	11,925	11,551	11,303	10,851	9,835	10,865
208740_at	9,904	9,989	9,698	9,216	9,647	9,064
208741_at	8,071	8,314	8,387	7,858	8,387	7,887
208742_s_at	11,964	12,144	11,913	12,169	12,022	12,400
208743_s_at	10,858	10,692	10,876	11,320	11,103	11,346
208744_x_at	10,308	10,018	10,204	9,369	8,944	8,751
208745_at	10,957	11,348	10,889	11,249	10,961	11,179
208746_x_at	12,327	12,496	12,492	12,465	12,063	12,529
208747_s_at	8,835	9,328	8,523	8,649	9,146	8,689
208748_s_at	6,001	6,006	6,256	6,183	5,965	6,217
208749_x_at	10,248	10,437	10,387	10,321	10,229	10,829
208750_s_at	9,562	9,593	9,293	9,883	9,003	9,954
208751_at	7,696	7,692	7,641	8,444	7,771	8,612
208752_x_at	13,658	13,934	13,504	12,129	12,333	12,145
208753_s_at	11,766	11,481	11,713	10,132	10,501	9,802
208754_s_at	12,417	12,457	12,179	10,142	10,707	10,098
208755_x_at	12,985	13,023	13,088	11,694	11,606	11,731
208756_at	12,638	12,731	12,793	12,217	11,726	12,383
208757_at	9,762	9,813	9,830	10,451	10,317	10,689
208758_at	12,368	12,404	12,553	11,399	10,866	11,183
208759_at	9,592	9,544	9,794	9,632	9,707	9,928
208760_at	10,678	10,471	10,795	7,753	8,362	8,536
208761_s_at	12,157	11,929	11,323	10,212	10,098	10,172
208762_at	9,820	9,541	9,086	7,544	7,496	7,469
208763_s_at	9,647	9,847	9,870	9,277	10,195	10,558
208764_s_at	10,858	10,510	10,679	10,403	9,833	10,543
208765_s_at	11,239	10,847	10,398	10,009	9,429	9,312
208766_s_at	13,425	13,361	13,217	12,138	12,524	11,830
208767_s_at	12,017	11,561	11,703	10,139	9,028	9,059
208768_x_at	13,149	13,408	12,967	13,106	13,120	13,193
208769_at	7,311	7,315	7,477	7,101	6,649	6,795
208770_s_at	11,766	11,581	11,639	9,918	9,379	9,783
208771_s_at	12,629	13,050	12,464	10,717	10,881	10,543
208772_at	10,399	10,417	10,255	7,924	9,391	7,847
208773_s_at	10,287	10,274	9,917	7,603	9,184	7,935
208774_at	10,433	10,088	10,479	8,945	9,360	9,349
208775_at	13,707	13,918	13,523	11,868	12,910	11,702
208776_at	9,600	10,002	10,051	8,027	8,558	7,971
208777_s_at	11,274	10,735	11,348	10,819	10,561	10,977
208778_s_at	12,833	12,638	12,683	12,207	12,323	11,863
208779_x_at	8,892	8,593	8,305	8,051	7,492	8,175
208780_x_at	11,809	12,213	12,105	12,660	12,789	12,975
208781_x_at	10,354	10,603	10,448	11,122	11,031	11,614
208782_at	12,158	12,544	12,215	11,444	12,259	11,992

208783_s_at	11,924	12,347	11,508	10,303	11,617	10,884
208784_s_at	9,381	9,149	9,074	9,035	8,535	9,072
208785_s_at	8,967	9,050	9,084	9,737	9,524	9,889
208786_s_at	10,890	10,923	10,962	9,956	11,106	11,005
208787_at	13,301	13,369	13,508	12,732	12,742	12,821
208788_at	12,724	12,949	12,701	12,200	12,776	12,634
208789_at	8,539	8,870	9,079	10,771	11,072	11,566
208790_s_at	6,782	6,440	6,036	7,823	7,077	8,656
208791_at	10,903	10,971	10,861	7,651	8,470	8,614
208792_s_at	11,094	11,177	11,155	7,862	8,812	8,884
208793_x_at	8,749	8,459	8,588	8,356	8,223	8,288
208794_s_at	11,752	11,570	11,969	10,486	10,377	10,548
208795_s_at	12,289	11,988	12,157	10,804	10,194	10,394
208796_s_at	12,000	12,660	11,825	10,700	11,821	11,517
208797_s_at	7,048	6,904	6,084	7,128	6,968	6,981
208798_x_at	10,655	10,556	10,041	5,887	9,889	7,312
208799_at	12,368	11,788	12,093	12,144	11,940	12,525
208800_at	10,346	10,407	9,763	9,654	10,424	9,899
208801_at	12,204	12,338	12,015	11,529	12,433	11,700
208802_at	10,653	10,685	10,316	9,705	10,753	9,887
208803_s_at	7,992	8,268	7,919	7,955	7,922	7,714
208804_s_at	11,455	11,692	11,807	10,383	11,304	10,583
208805_at	13,143	13,371	13,233	13,158	13,020	13,307
208806_at	7,519	7,484	7,437	7,468	7,318	7,707
208807_s_at	7,719	7,521	7,255	7,889	7,741	7,955
208808_s_at	13,903	13,940	13,646	12,437	12,512	12,216
208809_s_at	11,018	11,050	10,739	10,186	10,762	10,566
208810_at	10,707	10,618	10,017	9,326	8,969	9,260
208811_s_at	10,688	10,528	9,906	9,060	8,929	9,108
208812_x_at	10,633	10,975	10,833	12,113	11,383	12,046
208813_at	9,193	9,449	9,345	10,040	9,233	9,842
208814_at	10,601	10,472	10,180	8,114	9,428	8,416
208815_x_at	11,757	11,828	11,521	10,893	11,497	10,817
208816_x_at	11,093	11,344	11,591	13,034	13,487	13,404
208817_at	8,343	8,243	8,438	9,482	9,053	10,280
208818_s_at	8,884	8,868	9,701	10,665	10,605	11,461
208819_at	9,822	9,480	9,982	9,328	9,315	9,365
208820_at	11,050	11,324	10,782	9,722	10,744	9,640
208821_at	12,818	12,702	12,831	12,208	11,609	12,092
208822_s_at	12,004	12,255	12,098	11,765	11,448	11,692
208823_s_at	9,390	9,594	9,947	9,450	9,479	9,441
208824_x_at	8,980	8,937	8,924	8,489	7,248	8,290
208825_x_at	14,008	14,014	14,193	13,857	14,084	14,069
208826_x_at	12,716	12,988	12,989	12,727	12,691	13,067
208827_at	11,783	11,992	12,163	11,864	11,101	11,924
208828_at	11,034	10,654	11,255	10,919	10,153	10,778
208829_at	7,407	7,280	7,353	8,954	9,032	9,115
208830_s_at	7,480	7,254	7,663	7,840	8,240	8,301
208831_x_at	8,466	8,206	8,549	8,008	8,298	8,181
208832_at	9,417	9,529	9,440	8,667	8,602	8,701
208833_s_at	12,682	12,711	12,557	11,653	12,104	12,024
208834_x_at	13,972	14,012	14,112	13,840	14,060	14,153
208835_s_at	11,727	11,879	11,301	9,492	10,591	9,177
208836_at	13,055	13,391	13,412	12,819	12,974	12,992
208837_at	9,347	10,108	9,889	10,878	9,975	10,956
208838_at	11,809	11,840	11,785	8,582	9,755	8,320

208839_s_at	11,237	11,275	11,003	8,117	8,727	7,523
208840_s_at	9,989	9,674	9,952	9,492	9,756	9,412
208841_s_at	12,207	12,169	11,850	10,910	11,774	11,025
208842_s_at	11,253	11,318	11,355	11,234	11,249	11,632
208843_s_at	11,385	11,558	11,739	10,949	11,440	11,767
208844_at	6,697	6,827	6,630	7,071	6,698	7,024
208845_at	13,191	13,429	13,268	13,068	13,025	13,172
208846_s_at	11,811	11,766	11,444	10,810	10,259	10,780
208847_s_at	12,561	12,732	12,654	12,237	12,298	12,507
208848_at	11,525	11,773	11,498	9,195	9,781	9,987
208849_at	5,072	5,167	5,934	5,954	5,559	5,857
208850_s_at	9,696	9,340	9,568	9,719	8,387	10,574
208851_s_at	9,375	9,170	9,451	9,259	9,061	9,856
208852_s_at	9,657	9,451	9,584	9,701	10,386	9,747
208853_s_at	9,031	8,592	8,543	8,980	9,114	8,788
208854_s_at	9,539	9,424	9,399	9,068	8,743	9,038
208855_s_at	12,389	12,432	12,738	12,204	12,516	12,396
208856_x_at	14,628	14,730	14,677	14,035	14,208	14,305
208857_s_at	10,003	9,905	9,565	10,825	10,678	11,002
208858_s_at	10,424	10,022	10,557	9,764	9,813	10,177
208859_s_at	10,052	10,075	9,562	6,452	7,765	5,866
208860_s_at	10,335	10,181	9,506	6,964	9,032	7,031
208861_s_at	11,210	11,439	10,854	8,022	10,513	8,120
208862_s_at	10,110	10,072	10,132	8,648	8,931	8,809
208863_s_at	11,147	10,516	10,793	9,924	9,831	9,683
208864_s_at	12,919	13,193	12,939	13,056	13,028	13,413
208865_at	10,032	10,323	10,208	10,919	11,676	11,411
208866_at	9,100	8,550	8,696	8,301	9,578	8,451
208867_s_at	7,141	6,858	6,935	7,000	6,659	6,789
208868_s_at	8,948	9,087	8,495	7,440	7,696	7,696
208869_s_at	10,268	11,107	10,415	7,253	8,261	8,070
208870_x_at	12,662	13,042	12,853	12,685	12,833	12,967
208871_at	6,504	6,735	6,567	6,858	7,068	7,231
208872_s_at	8,674	8,787	9,078	11,121	10,464	11,262
208873_s_at	10,624	10,843	10,362	10,685	11,536	10,729
208874_x_at	10,490	9,961	10,069	9,817	9,772	10,033
208875_s_at	8,504	8,589	8,836	8,261	8,490	8,015
208876_s_at	6,481	6,235	6,162	6,231	6,669	6,653
208877_at	10,610	10,451	10,428	9,641	10,809	10,112
208878_s_at	8,583	8,479	8,404	8,206	8,988	8,633
208879_x_at	7,257	7,191	7,599	7,622	7,199	7,736
208880_s_at	10,115	9,867	10,109	9,623	9,094	9,801
208881_x_at	12,116	12,382	11,435	11,323	12,008	11,855
208882_s_at	10,645	10,692	10,461	8,836	10,063	8,244
208883_at	8,780	8,891	8,756	6,592	8,072	6,332
208884_s_at	9,303	9,056	8,725	7,264	8,858	7,153
208885_at	7,432	7,678	7,712	7,735	7,654	8,034
208886_at	10,429	10,351	10,382	7,752	7,568	7,367
208887_at	11,509	11,390	11,777	11,570	11,051	11,590
208888_s_at	6,784	6,532	6,631	7,441	7,287	7,476
208889_s_at	6,854	6,632	6,688	7,170	6,981	7,184
208890_s_at	8,769	8,665	9,009	9,855	10,070	10,860
208891_at	10,127	10,976	11,960	11,734	11,120	11,520
208892_s_at	9,974	10,670	11,564	11,503	10,899	11,474
208893_s_at	9,735	10,222	10,978	10,659	9,616	10,668
208894_at	6,263	6,196	6,299	6,829	6,840	6,705

208895_s_at	10,138	10,297	9,699	9,042	8,706	8,423
208896_at	11,068	10,868	10,443	9,167	9,913	7,973
208897_s_at	11,677	11,742	11,210	10,425	10,364	10,054
208898_at	11,267	11,547	11,335	11,275	11,770	11,660
208899_x_at	9,797	10,057	9,708	10,346	10,199	10,425
208900_s_at	10,074	9,995	10,186	10,226	10,134	9,882
208901_s_at	12,044	11,718	11,668	11,093	12,001	11,071
208902_s_at	7,411	7,538	7,754	8,066	7,887	8,308
208903_at	9,124	9,011	9,467	8,720	8,091	8,384
208904_s_at	14,284	14,455	14,458	13,768	13,659	14,081
208905_at	13,911	13,897	13,755	12,888	13,400	13,122
208906_at	9,988	9,518	10,110	8,653	8,283	8,840
208907_s_at	8,469	8,263	8,393	8,946	7,874	8,912
208908_s_at	7,298	7,504	7,167	9,164	9,192	9,063
208909_at	12,462	12,566	12,451	12,383	11,747	12,469
208910_s_at	12,581	12,460	12,643	12,282	11,164	11,936
208911_s_at	11,277	11,489	11,006	10,198	10,046	10,239
208912_s_at	9,798	9,745	9,969	10,585	10,508	10,954
208913_at	10,930	11,000	10,827	8,203	7,789	8,295
208914_at	8,356	8,374	8,529	6,674	7,024	6,993
208915_s_at	8,909	8,794	8,839	8,814	8,790	8,990
208916_at	9,215	9,021	9,109	9,166	8,692	9,446
208917_x_at	7,458	7,416	7,427	7,827	7,440	7,955
208918_s_at	9,408	9,489	9,729	11,580	10,716	11,104
208919_s_at	10,191	10,255	10,314	9,900	9,664	10,170
208920_at	7,214	7,149	6,957	6,537	7,026	6,530
208921_s_at	12,173	12,275	12,150	12,254	11,915	12,287
208922_s_at	9,323	9,082	9,963	8,987	9,057	9,353
208923_at	11,946	12,111	12,283	12,067	12,547	12,503
208924_at	11,010	11,289	10,864	10,977	11,554	11,715
208925_at	10,027	10,120	9,350	7,512	7,932	7,710
208926_at	8,546	8,793	8,595	10,081	8,452	9,525
208927_at	8,217	8,273	8,018	8,181	7,898	8,179
208928_at	8,931	8,796	8,977	8,702	8,331	9,089
208929_x_at	12,666	12,890	12,714	12,398	12,096	12,700
208930_s_at	11,330	10,985	11,093	10,473	9,567	10,277
208931_s_at	11,154	10,311	10,818	9,266	8,563	8,697
208932_at	11,519	11,283	11,440	11,048	10,782	11,278
208933_s_at	7,383	7,683	7,233	6,911	8,549	7,002
208934_s_at	7,916	8,172	7,571	9,885	9,323	9,254
208935_s_at	6,362	6,579	6,256	6,142	7,654	6,477
208936_x_at	8,552	8,520	8,467	9,457	9,522	8,937
208937_s_at	9,444	10,068	9,491	9,223	9,301	9,887
208938_at	8,453	7,754	8,241	7,806	7,284	8,116
208939_at	12,230	11,992	12,547	9,005	8,981	8,906
208940_at	11,626	11,284	12,193	8,162	7,785	7,717
208941_s_at	11,085	10,733	11,082	10,508	9,794	10,040
208942_s_at	10,351	10,659	9,969	10,332	10,904	9,966
208943_s_at	10,880	10,884	10,320	9,877	11,111	9,818
208944_at	8,055	7,793	7,627	10,916	11,712	11,240
208945_s_at	8,334	8,363	8,255	8,980	8,470	8,904
208946_s_at	8,894	9,021	9,209	9,730	9,708	9,696
208947_s_at	7,768	7,467	7,862	7,478	7,507	7,735
208948_s_at	12,168	12,364	12,669	11,268	11,760	11,568
208949_s_at	8,446	9,266	8,639	12,968	13,166	13,498
208950_s_at	11,211	11,364	10,916	9,507	9,103	8,946

208951_at	10,256	10,245	9,670	8,558	7,855	7,748
208952_s_at	8,406	8,423	8,823	8,160	8,427	8,111
208953_at	7,726	7,685	7,793	6,963	7,171	6,878
208954_s_at	9,397	9,431	9,298	8,186	8,891	8,226
208955_at	9,814	9,952	10,026	9,545	9,484	8,994
208956_x_at	12,595	12,747	12,589	12,627	12,398	12,502
208957_at	7,975	8,163	8,050	8,002	8,065	7,598
208958_at	7,167	7,097	6,986	5,462	5,748	5,513
208959_s_at	10,151	10,411	10,377	10,489	10,564	10,499
208960_s_at	6,977	7,245	6,819	7,610	7,874	7,672
208961_s_at	7,819	8,273	8,182	9,040	9,225	9,568
208962_s_at	10,733	10,809	10,628	10,570	9,430	10,500
208963_x_at	10,453	10,375	10,053	9,779	9,171	9,952
208964_s_at	10,649	10,546	10,519	10,034	9,673	10,370
208965_s_at	8,917	9,337	8,603	9,216	8,672	8,370
208966_x_at	8,880	9,352	8,630	9,851	10,108	9,003
208967_s_at	9,836	9,729	9,938	10,537	9,764	10,449
208968_s_at	11,303	10,971	11,626	10,170	10,029	10,294
208969_at	11,875	11,885	12,636	11,551	11,202	11,535
208970_s_at	11,425	11,217	11,710	12,236	11,902	12,276
208971_at	9,718	9,354	9,492	10,182	9,574	10,161
208972_s_at	10,332	10,500	10,847	11,860	11,349	11,773
208973_at	9,122	9,040	9,107	8,510	8,251	8,557
208974_x_at	11,912	11,286	11,611	11,079	11,161	10,862
208975_s_at	9,247	8,909	9,274	9,080	9,398	8,549
208977_x_at	11,961	11,681	11,746	12,256	11,536	12,230
208978_at	6,829	6,936	6,284	7,866	7,909	8,535
208979_at	10,189	10,119	10,490	9,761	10,335	9,897
208980_s_at	13,088	13,400	13,128	13,250	13,436	13,647
208981_at	6,387	6,248	6,450	6,914	7,057	6,863
208982_at	7,126	7,441	6,782	7,723	7,858	7,875
208983_s_at	6,145	6,212	5,638	6,973	6,616	6,833
208984_x_at	9,989	9,705	10,197	8,933	8,079	8,404
208985_s_at	9,741	9,754	9,786	10,288	10,666	10,042
208986_at	11,392	11,754	11,075	8,759	9,863	8,608
208987_s_at	9,788	9,600	9,960	9,467	9,498	9,834
208988_at	10,799	10,575	11,011	8,812	8,892	9,003
208989_s_at	9,154	9,043	9,437	8,421	8,648	8,587
208990_s_at	12,683	12,935	11,822	10,073	10,781	9,679
208991_at	9,579	9,476	9,750	8,440	8,771	9,124
208992_s_at	9,048	8,921	9,031	8,957	8,362	9,008
208993_s_at	10,163	10,388	9,658	8,040	8,695	7,073
208994_s_at	9,633	9,990	9,190	7,952	8,331	7,473
208995_s_at	9,928	9,922	9,188	8,476	9,241	7,210
208996_s_at	10,637	10,511	10,928	10,541	10,244	10,747
208997_s_at	8,146	8,017	8,134	8,639	7,879	8,336
208998_at	10,178	10,436	10,793	7,940	7,905	8,321
208999_at	10,052	9,972	9,892	9,365	10,168	9,949
209000_s_at	7,240	7,174	6,939	7,979	8,043	8,299
209001_s_at	11,999	12,142	11,939	12,028	12,457	12,643
209002_s_at	7,319	7,481	7,355	8,392	7,987	8,315
209003_at	9,112	9,129	9,194	9,977	8,876	9,775
209004_s_at	9,524	9,978	9,686	9,587	9,741	9,659
209005_at	8,868	9,111	8,915	8,532	8,891	8,252
209006_s_at	8,264	8,555	8,100	5,591	7,510	6,737
209007_s_at	10,202	10,606	10,080	8,836	10,457	9,707

209008_x_at	10,007	9,484	9,611	7,508	7,542	7,784
209009_at	10,739	11,172	10,437	11,837	11,992	12,160
209010_s_at	5,643	5,397	5,896	6,497	6,556	6,481
209011_at	8,557	8,402	8,494	8,767	9,110	8,628
209012_at	9,359	9,227	9,207	9,224	10,278	9,361
209013_x_at	7,646	7,324	7,880	8,376	9,068	7,836
209014_at	13,050	13,177	13,254	12,579	13,128	13,429
209015_s_at	9,899	9,259	10,346	8,064	7,759	8,498
209016_s_at	6,699	6,417	6,501	7,156	7,307	7,651
209017_s_at	10,590	10,606	10,785	9,668	8,943	9,301
209018_s_at	8,716	8,732	8,631	9,696	9,265	9,566
209019_s_at	8,549	8,890	8,213	9,639	9,419	9,742
209020_at	8,916	9,680	9,376	9,166	9,475	9,212
209021_x_at	9,719	9,551	9,797	9,619	9,347	9,708
209022_at	11,362	11,758	11,034	9,385	10,719	8,528
209023_s_at	10,932	11,028	10,318	9,394	10,571	8,363
209024_s_at	11,695	11,695	11,682	11,267	10,704	11,012
209025_s_at	12,944	12,369	12,760	11,566	12,325	11,549
209026_x_at	12,799	12,652	12,720	12,649	12,144	12,854
209027_s_at	9,444	9,583	9,336	9,460	9,553	9,779
209028_s_at	10,131	10,585	9,986	9,392	10,288	9,756
209029_at	10,219	10,022	10,130	10,157	9,428	10,316
209030_s_at	8,994	8,699	8,399	7,270	7,479	7,272
209031_at	10,323	10,256	9,467	5,420	5,538	5,162
209032_s_at	8,569	8,431	7,955	6,641	6,362	6,526
209033_s_at	11,580	11,652	11,581	8,421	9,937	8,919
209034_at	8,396	9,066	8,506	8,346	9,320	9,526
209035_at	10,176	9,848	9,829	7,615	7,152	7,630
209036_s_at	12,711	12,791	12,867	12,271	12,153	12,471
209037_s_at	5,632	5,361	4,880	8,198	7,634	8,070
209038_s_at	6,777	7,091	7,122	8,013	7,961	7,825
209039_x_at	9,027	8,967	9,230	10,365	10,236	10,586
209040_s_at	6,786	7,455	6,501	10,170	8,852	9,261
209041_s_at	7,701	7,708	7,586	7,812	7,288	7,802
209042_s_at	10,775	10,664	10,893	9,746	9,719	9,663
209043_at	11,348	11,529	11,095	10,578	10,697	11,059
209044_x_at	10,794	10,277	11,298	10,527	9,727	10,316
209045_at	10,676	10,904	10,799	11,144	11,584	11,845
209046_s_at	11,661	11,779	11,433	11,503	10,968	11,463
209047_at	5,490	5,871	6,070	5,883	6,305	5,877
209048_s_at	11,245	11,290	11,225	8,179	9,375	8,426
209049_s_at	11,469	11,436	11,572	8,816	9,810	9,211
209050_s_at	8,095	8,209	8,413	7,321	6,811	6,991
209051_s_at	7,709	7,414	7,529	7,369	7,239	7,382
209052_s_at	8,006	7,889	7,781	7,384	7,349	7,379
209053_s_at	10,049	9,793	10,227	9,093	8,472	8,321
209054_s_at	10,259	10,169	10,525	8,995	8,996	8,423
209055_s_at	8,243	8,396	7,415	6,814	6,816	6,622
209056_s_at	10,759	11,102	10,450	9,435	10,233	9,008
209057_x_at	7,471	7,923	7,952	7,629	7,655	8,141
209058_at	11,454	11,444	11,700	11,469	11,188	11,818
209059_s_at	10,523	10,317	10,556	10,591	10,107	11,041
209060_x_at	7,573	7,467	7,541	7,343	7,708	7,391
209061_at	9,327	9,476	9,438	7,901	9,818	8,193
209062_x_at	7,353	6,928	7,169	7,972	8,226	8,064
209063_x_at	9,964	10,093	9,684	9,797	9,761	10,009

209064_x_at	11,097	11,050	11,063	8,315	8,742	8,686
209065_at	9,119	9,254	9,259	9,033	8,786	9,281
209066_x_at	11,633	11,910	11,073	11,566	11,488	11,837
209067_s_at	12,734	12,439	12,242	11,384	11,567	11,166
209068_at	10,022	10,070	9,773	8,342	8,711	8,391
209069_s_at	12,529	12,489	11,822	12,249	11,595	12,161
209070_s_at	8,060	7,903	8,185	7,508	7,244	7,424
209071_s_at	8,356	8,349	8,132	6,303	6,524	6,449
209072_at	7,992	7,756	8,003	7,978	7,705	8,010
209073_s_at	8,063	8,240	7,997	9,301	9,119	9,244
209074_s_at	7,062	6,693	6,596	6,981	6,802	6,745
209075_s_at	11,116	11,328	11,220	11,730	11,290	11,657
209076_s_at	12,449	12,427	12,390	11,651	11,849	11,845
209077_at	9,550	9,715	10,075	10,461	10,278	10,681
209078_s_at	8,492	8,449	8,581	9,232	8,912	9,360
209079_x_at	8,743	8,441	8,558	9,973	9,634	9,691
209080_x_at	10,270	10,302	10,331	10,859	9,937	10,730
209081_s_at	10,071	10,143	10,154	8,667	8,835	8,677
209082_s_at	9,117	8,786	9,087	8,700	8,814	8,846
209083_at	7,480	7,299	7,212	7,566	7,040	7,531
209084_s_at	9,754	9,986	9,114	7,367	7,289	7,028
209085_x_at	9,923	10,046	9,196	9,475	9,817	8,728
209086_x_at	7,973	7,946	8,138	9,017	7,003	8,516
209087_x_at	9,060	8,963	9,135	10,387	8,703	9,782
209088_s_at	10,606	10,469	10,877	10,224	10,166	10,466
209089_at	11,956	12,100	11,875	11,292	11,908	11,363
209090_s_at	9,920	10,335	9,981	9,159	10,329	9,319
209091_s_at	9,296	9,659	9,370	10,496	10,794	10,964
209092_s_at	10,508	10,186	10,227	10,122	9,707	9,913
209093_s_at	7,777	7,661	7,739	8,986	8,348	9,037
209094_at	11,422	11,695	11,779	11,413	12,801	12,685
209095_at	12,012	12,218	11,511	10,978	11,737	10,505
209096_at	11,761	11,730	11,110	10,715	11,098	10,763
209097_s_at	6,683	6,663	6,452	6,736	6,835	6,795
209098_s_at	8,060	7,237	7,471	6,158	7,860	7,173
209099_x_at	10,499	10,496	10,677	7,887	11,328	10,040
209100_at	10,020	9,816	10,415	10,897	10,003	10,452
209101_at	9,308	10,683	10,647	10,608	11,612	12,342
209102_s_at	9,483	9,879	9,433	7,706	9,038	8,169
209103_s_at	10,670	10,216	10,647	11,337	10,591	11,355
209104_s_at	12,454	12,505	12,717	12,766	12,018	12,586
209105_at	6,899	6,980	6,893	5,676	6,447	5,464
209106_at	8,751	9,067	9,007	5,900	7,184	5,921
209107_x_at	8,292	8,633	8,759	8,078	8,454	8,043
209108_at	12,274	12,375	12,345	9,532	9,190	9,795
209109_s_at	12,020	11,906	11,818	10,176	9,799	10,242
209110_s_at	8,565	8,845	8,715	8,627	8,037	8,742
209111_at	10,193	9,978	10,013	9,855	9,005	9,776
209112_at	11,652	11,954	11,574	8,475	9,720	8,620
209113_s_at	8,185	8,220	7,903	7,756	7,524	7,961
209114_at	6,968	6,825	7,196	7,705	7,498	7,658
209115_at	11,877	12,073	11,469	10,328	11,005	10,571
209116_x_at	6,286	6,474	6,523	7,122	7,101	7,431
209117_at	8,239	8,306	8,223	8,534	7,857	8,193
209118_s_at	14,033	14,143	13,960	13,009	13,517	13,722
209119_x_at	6,247	5,838	6,552	8,406	8,452	8,415

209120_at	7,275	5,189	4,550	11,257	11,891	11,807
209121_x_at	7,601	7,492	7,104	9,838	10,137	10,136
209122_at	11,990	11,961	12,199	13,122	13,543	13,508
209123_at	11,002	11,277	11,301	11,168	10,807	11,185
209124_at	10,461	10,011	10,966	10,785	10,266	10,711
209125_at	4,204	4,182	4,625	4,847	4,738	4,861
209126_x_at	7,952	7,734	7,848	8,553	8,431	8,597
209127_s_at	11,653	11,664	11,706	10,263	9,541	9,928
209128_s_at	11,412	11,275	11,325	9,753	9,754	9,599
209129_at	10,625	10,587	10,791	11,058	10,748	11,272
209130_at	11,019	11,193	11,200	10,411	10,943	10,897
209131_s_at	5,753	6,267	5,937	5,818	5,827	6,387
209132_s_at	10,268	9,629	9,984	9,955	8,883	9,726
209133_s_at	7,453	6,993	7,543	7,594	7,469	7,441
209134_s_at	14,341	14,258	14,105	13,316	13,075	13,669
209135_at	11,034	11,336	10,635	12,358	13,305	12,907
209136_s_at	9,293	9,228	9,319	8,506	7,707	8,315
209137_s_at	10,909	10,801	11,156	10,215	10,239	10,323
209138_x_at	7,356	7,524	7,311	8,032	8,118	8,315
209139_s_at	10,788	11,139	10,605	9,538	9,632	9,957
209140_x_at	10,481	10,900	10,555	11,955	10,934	11,793
209141_at	11,461	11,613	11,696	11,038	11,014	11,195
209142_s_at	10,443	10,310	10,400	10,387	9,829	10,369
209143_s_at	12,362	12,312	12,114	11,425	10,799	11,287
209144_s_at	6,935	7,287	7,387	6,834	6,821	7,015
209145_s_at	6,642	6,990	6,687	7,338	6,870	7,170
209146_at	12,020	12,426	11,592	11,901	11,872	12,320
209147_s_at	9,437	10,038	9,738	9,315	9,518	9,951
209148_at	8,357	8,494	8,483	8,955	8,897	8,863
209149_s_at	7,680	7,495	8,036	10,041	9,063	10,125
209150_s_at	9,851	9,812	10,211	10,921	10,370	10,934
209151_x_at	5,719	5,887	6,059	6,164	6,452	6,555
209152_s_at	8,626	8,466	8,401	8,006	7,802	8,004
209153_s_at	12,229	12,288	12,780	9,721	9,662	9,959
209154_at	11,990	11,934	12,620	13,190	13,402	13,667
209155_s_at	9,343	9,325	9,541	8,996	8,973	9,056
209156_s_at	7,678	8,179	7,616	10,611	10,446	11,201
209157_at	9,788	9,954	9,881	10,032	9,623	9,659
209158_s_at	8,944	8,918	8,911	8,582	8,163	8,705
209159_s_at	8,364	8,307	7,809	7,625	7,448	7,180
209160_at	5,077	6,634	5,184	11,531	11,814	12,119
209161_at	10,986	10,907	11,069	10,211	10,037	9,885
209162_s_at	9,101	8,688	8,649	8,076	7,248	7,944
209163_at	8,210	8,311	8,869	8,685	8,184	8,771
209164_s_at	8,354	8,239	8,314	8,871	7,682	8,909
209165_at	9,793	9,348	9,812	9,426	9,222	9,328
209166_s_at	8,961	8,934	9,078	8,612	8,167	9,035
209167_at	11,123	11,036	11,217	11,867	11,437	11,774
209168_at	9,915	10,188	10,131	9,577	9,158	8,905
209169_at	10,500	10,364	10,345	9,514	8,617	8,636
209170_s_at	11,235	10,991	11,157	12,246	11,877	12,166
209171_at	10,244	9,751	10,380	10,534	9,841	10,469
209172_s_at	11,850	11,774	10,754	7,960	8,696	6,884
209173_at	5,781	5,466	5,772	5,749	5,536	5,920
209174_s_at	11,249	11,058	11,056	10,233	10,212	10,285
209175_at	10,165	9,844	9,564	8,675	9,519	8,953

209176_at	8,507	8,567	8,478	8,439	9,025	8,632
209177_at	8,033	8,036	8,175	9,614	9,016	9,865
209178_at	9,149	9,162	9,246	9,064	9,228	9,153
209179_s_at	9,029	8,689	9,267	9,311	9,376	9,522
209180_at	11,746	12,040	11,348	10,563	10,711	10,255
209181_s_at	13,068	13,098	12,588	12,369	12,919	12,387
209182_s_at	7,259	6,939	7,268	7,831	7,780	7,885
209183_s_at	7,488	8,277	7,612	8,192	9,042	9,002
209184_s_at	9,254	9,757	9,509	8,118	10,067	9,565
209185_s_at	10,683	11,201	11,044	9,370	11,656	11,216
209186_at	10,802	10,584	10,892	10,074	10,447	10,191
209187_at	10,787	11,003	10,510	9,635	10,028	10,207
209188_x_at	9,576	9,701	9,842	10,095	9,615	10,366
209189_at	9,340	11,845	11,459	10,642	10,819	11,238
209190_s_at	10,756	10,703	10,911	10,277	11,103	10,675
209191_at	11,132	10,997	10,990	13,071	12,535	13,009
209192_x_at	8,925	8,682	9,077	8,272	7,528	8,302
209193_at	8,848	9,273	9,161	8,084	8,275	8,451
209194_at	10,786	10,959	10,612	11,319	11,534	11,480
209195_s_at	7,843	7,532	7,735	7,094	6,915	7,397
209196_at	9,316	8,846	9,346	9,682	9,013	9,678
209197_at	9,403	9,417	9,452	7,968	8,347	8,041
209198_s_at	8,870	8,532	9,042	8,352	8,606	8,541
209199_s_at	6,733	6,999	6,346	6,748	7,196	6,775
209200_at	6,502	6,718	6,251	6,612	7,356	6,537
209201_x_at	7,286	6,998	6,919	6,804	6,689	6,494
209202_s_at	8,826	8,489	9,168	8,146	7,966	8,233
209203_s_at	8,056	7,972	7,905	8,458	7,729	8,480
209204_at	7,334	7,117	6,989	7,166	6,837	7,175
209205_s_at	9,727	9,746	9,722	8,658	7,570	8,385
209206_at	9,325	9,378	9,203	11,042	11,083	11,441
209207_s_at	8,181	7,931	8,257	8,785	8,863	9,383
209208_at	8,412	8,146	8,293	9,277	8,553	9,230
209209_s_at	8,655	8,887	8,087	8,141	9,588	8,811
209210_s_at	12,325	12,337	11,941	11,235	13,270	12,480
209211_at	5,407	6,376	5,581	5,726	6,004	5,701
209212_s_at	6,483	6,498	6,531	6,749	6,849	6,967
209213_at	5,999	5,281	5,348	9,564	8,905	9,535
209214_s_at	10,401	10,119	10,448	10,163	9,990	9,933
209215_at	9,198	9,146	9,388	9,447	9,245	9,771
209216_at	7,816	7,980	8,418	8,739	8,262	8,939
209217_s_at	7,486	7,497	7,913	8,330	7,516	8,281
209218_at	12,394	12,499	12,063	11,854	11,797	11,987
209219_at	10,663	10,898	10,982	11,466	11,095	11,595
209220_at	9,520	9,535	9,281	6,403	6,673	6,387
209221_s_at	7,884	8,314	8,060	7,862	7,489	7,811
209222_s_at	8,779	9,041	8,781	8,363	8,507	8,482
209223_at	5,739	5,724	5,390	5,733	5,587	6,000
209224_s_at	10,384	10,389	10,432	10,238	10,093	10,520
209225_x_at	11,624	11,302	11,080	11,336	12,476	11,290
209226_s_at	10,856	10,618	10,289	10,173	11,613	10,209
209227_at	8,021	7,949	7,745	6,654	6,732	6,887
209228_x_at	10,416	10,187	10,188	10,219	10,226	10,736
209229_s_at	8,699	8,663	8,979	8,678	8,687	9,120
209230_s_at	5,991	6,475	6,249	7,725	7,565	8,190
209231_s_at	8,770	8,311	9,028	9,256	8,773	9,249

209232_s_at	10,197	10,036	10,305	9,031	9,007	9,261
209233_at	12,948	12,624	13,148	11,898	11,006	11,684
209234_at	10,687	10,961	10,592	8,026	8,980	7,425
209235_at	8,886	8,599	8,673	8,989	8,948	8,939
209236_at	7,663	7,540	8,048	7,058	7,015	6,956
209237_s_at	7,490	7,549	7,639	7,917	7,962	8,147
209238_at	11,192	11,742	11,192	9,870	9,119	8,722
209239_at	7,963	7,946	8,075	8,844	8,591	8,578
209240_at	12,251	12,130	12,368	9,357	11,268	9,815
209241_x_at	7,606	7,521	7,795	7,779	7,583	7,809
209242_at	12,349	11,696	11,647	5,323	6,312	5,845
209243_s_at	9,813	8,849	8,481	4,974	4,777	4,940
209244_s_at	8,159	7,841	8,216	9,659	9,673	9,859
209245_s_at	6,771	6,828	6,434	6,375	6,276	6,012
209246_at	9,148	8,760	9,299	8,745	8,379	8,465
209247_s_at	10,127	9,810	10,523	10,542	10,303	10,317
209248_at	11,673	11,680	11,800	11,552	11,506	11,820
209249_s_at	11,748	11,744	11,803	12,267	11,932	12,414
209250_at	11,179	11,200	11,753	11,992	12,344	12,528
209251_x_at	14,425	14,394	14,374	14,042	14,044	14,271
209252_at	9,924	9,877	9,979	9,661	9,539	9,513
209253_at	8,498	8,308	8,791	8,971	8,538	8,967
209254_at	6,956	6,864	7,311	8,922	8,196	8,573
209255_at	7,178	7,245	6,925	6,751	7,082	6,931
209256_s_at	7,344	7,410	7,432	7,171	8,088	7,351
209257_s_at	10,021	10,161	9,203	7,709	7,362	6,946
209258_s_at	10,048	10,270	9,345	7,975	7,614	6,523
209259_s_at	12,315	12,181	11,146	9,037	10,077	7,387
209260_at	7,912	7,405	7,683	8,436	7,904	8,322
209261_s_at	7,268	7,284	7,163	7,011	6,740	7,062
209262_s_at	10,623	10,619	11,052	10,122	9,368	9,945
209263_x_at	9,222	9,188	9,327	10,382	10,114	10,411
209264_s_at	7,519	7,389	7,086	8,591	7,844	8,500
209265_s_at	11,302	11,379	11,446	10,660	10,319	10,490
209266_s_at	6,146	5,865	6,175	6,176	5,782	5,878
209267_s_at	8,801	8,704	8,548	8,437	8,298	8,127
209268_at	9,384	9,375	9,359	8,561	8,908	8,372
209269_s_at	6,112	6,315	6,320	6,837	7,034	7,240
209270_at	5,902	6,309	5,703	7,114	7,003	7,340
209271_at	9,875	9,776	10,109	7,747	8,635	7,407
209272_at	9,755	9,756	9,175	9,013	8,329	7,449
209273_s_at	11,423	11,876	11,708	11,508	11,050	11,287
209274_s_at	12,394	12,460	12,377	10,607	11,184	11,020
209275_s_at	8,985	8,610	8,750	8,573	7,901	8,576
209276_s_at	6,926	7,367	6,896	9,666	9,191	9,969
209277_at	6,899	7,179	7,556	13,005	13,281	13,183
209278_s_at	7,443	7,377	8,204	13,844	13,714	13,987
209279_s_at	8,818	8,594	8,595	9,464	8,462	8,948
209280_at	7,951	7,873	8,366	8,824	9,072	9,002
209281_s_at	9,600	9,483	9,025	10,573	10,338	9,234
209282_at	8,255	8,281	8,225	7,717	7,375	7,942
209283_at	6,574	6,963	6,701	7,113	8,396	8,932
209284_s_at	10,515	10,573	10,001	8,669	10,293	8,215
209285_s_at	9,041	9,442	8,487	7,306	9,009	7,122
209286_at	7,564	7,377	7,900	7,538	9,072	9,332
209287_s_at	7,699	8,124	8,130	8,153	7,585	8,550

209288_s_at	10,057	10,080	10,271	9,889	10,629	11,440
209289_at	8,426	8,147	8,366	7,959	10,125	8,125
209290_s_at	7,394	7,523	7,292	6,656	8,879	7,150
209291_at	10,006	10,580	9,860	4,937	10,734	10,177
209292_at	7,862	8,745	8,026	4,395	7,642	6,184
209293_x_at	8,726	8,992	8,385	8,274	9,020	9,126
209294_x_at	7,996	7,819	7,631	8,815	8,103	8,259
209295_at	10,825	11,270	11,283	11,429	11,731	11,634
209296_at	11,829	12,123	11,779	8,796	9,183	8,324
209297_at	11,972	12,161	12,025	9,267	10,095	9,112
209298_s_at	11,183	11,183	11,122	7,558	8,476	7,345
209299_x_at	8,561	8,307	8,787	8,595	8,407	8,743
209300_s_at	10,649	10,621	10,478	9,603	9,761	10,093
209301_at	6,369	6,740	6,330	4,964	5,085	5,125
209302_at	11,696	11,860	12,066	11,640	10,937	11,383
209303_at	9,008	9,526	9,041	9,722	9,422	9,882
209304_x_at	7,487	9,193	8,702	9,474	10,517	10,166
209305_s_at	7,546	8,045	7,564	8,121	9,172	9,497
209306_s_at	9,686	10,280	9,477	9,098	9,845	8,754
209307_at	9,986	10,376	9,615	7,477	7,857	6,814
209308_s_at	9,812	9,910	9,537	8,427	9,729	8,570
209309_at	4,349	4,281	4,311	5,274	5,507	5,207
209310_s_at	5,979	5,591	5,178	9,619	9,323	8,804
209311_at	8,663	8,856	8,207	8,841	9,212	9,423
209312_x_at	7,021	7,654	6,930	7,806	7,545	7,800
209313_at	11,039	11,248	11,332	11,056	11,007	10,965
209314_s_at	8,991	9,017	8,082	7,950	8,701	7,257
209315_at	7,526	6,841	6,689	5,849	6,377	6,058
209316_s_at	10,528	10,550	9,868	9,739	10,349	9,454
209317_at	10,932	10,747	10,948	11,075	10,390	10,703
209318_x_at	8,229	7,109	7,253	8,272	9,446	9,077
209320_at	7,601	7,913	7,953	8,408	8,324	8,165
209321_s_at	6,978	6,852	6,891	7,981	7,262	7,685
209322_s_at	7,803	7,963	8,189	8,127	8,063	8,121
209323_at	10,738	10,684	10,268	9,691	10,444	9,611
209324_s_at	8,320	8,091	8,488	7,893	8,039	8,169
209325_s_at	8,750	8,343	8,776	7,628	7,496	7,842
209326_at	9,700	9,756	9,895	10,067	10,022	10,563
209327_s_at	7,087	7,046	7,519	7,461	7,492	7,294
209328_x_at	6,898	6,763	7,060	7,243	6,837	7,180
209329_x_at	9,893	9,967	10,073	10,566	9,732	10,623
209330_s_at	13,104	12,838	12,702	11,505	11,211	11,024
209331_s_at	8,646	8,110	8,500	9,081	8,605	8,483
209332_s_at	10,880	10,503	11,029	11,684	11,330	11,694
209333_at	8,124	8,065	8,230	7,129	7,274	7,010
209334_s_at	7,903	7,837	8,182	7,293	7,213	7,544
209335_at	5,317	5,036	5,073	9,730	9,665	10,191
209336_at	10,126	9,944	10,441	9,039	8,478	8,725
209337_at	12,474	12,736	11,890	8,092	8,985	6,813
209338_at	9,468	9,447	9,370	8,577	8,233	8,081
209339_at	8,848	8,879	9,050	8,675	8,285	8,659
209340_at	10,542	11,102	10,305	11,658	12,584	12,146
209341_s_at	9,401	9,372	9,451	7,736	8,501	8,285
209342_s_at	7,190	7,303	7,145	6,582	6,432	6,518
209343_at	8,886	9,473	8,860	6,754	6,973	7,064
209344_at	9,443	9,331	9,393	10,258	9,958	11,025

209345_s_at	8,195	8,319	8,350	8,928	8,543	8,708
209346_s_at	7,900	7,571	7,875	8,274	7,983	8,412
209347_s_at	5,870	5,409	5,329	6,642	6,968	6,986
209348_s_at	9,777	9,886	9,818	5,153	7,824	5,663
209349_at	6,759	6,887	6,659	6,156	6,768	6,162
209350_s_at	8,338	7,965	8,484	8,468	7,999	8,530
209351_at	6,171	5,801	5,636	6,883	6,654	6,728
209352_s_at	8,566	8,516	8,492	7,332	7,310	7,707
209353_s_at	7,196	7,011	7,314	6,717	6,279	6,650
209354_at	7,164	7,267	7,005	8,598	8,381	8,592
209355_s_at	9,728	9,701	9,642	7,915	9,823	10,361
209356_x_at	7,603	7,631	7,440	9,135	8,931	10,053
209357_at	8,287	8,820	8,742	9,328	10,575	11,198
209358_at	10,210	10,298	10,225	10,057	9,587	9,942
209359_x_at	6,731	6,781	6,003	6,672	6,534	6,531
209360_s_at	7,299	7,040	7,571	8,795	9,373	8,808
209361_s_at	8,578	8,190	8,366	8,417	8,043	8,363
209362_at	11,026	11,314	11,075	9,529	9,416	9,879
209363_s_at	9,391	9,690	9,331	8,971	7,767	8,405
209364_at	6,631	6,612	6,836	7,844	7,349	7,650
209365_s_at	7,000	6,903	6,722	7,700	7,892	7,947
209366_x_at	10,118	10,420	10,086	10,612	10,888	11,058
209367_at	8,030	7,571	7,781	7,918	8,004	8,011
209368_at	7,119	7,676	6,882	5,597	5,353	5,471
209369_at	6,447	7,238	8,310	4,609	4,451	4,630
209370_s_at	8,635	8,338	8,479	8,200	7,790	8,256
209371_s_at	7,736	7,715	7,616	7,608	7,697	7,711
209372_x_at	9,606	9,190	9,254	9,261	8,678	9,384
209373_at	6,698	6,594	6,682	6,840	6,868	6,981
209374_s_at	7,360	7,282	7,556	8,237	8,209	8,460
209375_at	9,607	9,514	9,671	8,478	8,884	8,707
209376_x_at	9,738	9,614	9,230	8,444	9,010	8,419
209377_s_at	13,211	13,297	12,904	11,883	12,097	12,008
209378_s_at	8,450	8,280	8,061	8,514	9,008	8,601
209379_s_at	8,242	8,369	7,683	8,099	9,309	7,995
209380_s_at	10,441	10,326	10,645	8,506	8,887	8,600
209381_x_at	8,429	8,175	8,507	8,079	7,503	7,961
209382_at	9,210	9,248	9,705	9,284	8,671	9,000
209383_at	9,396	10,575	9,682	9,069	9,075	9,274
209384_at	11,171	10,716	11,325	10,225	10,799	10,653
209385_s_at	10,381	9,978	10,389	9,840	9,491	10,003
209386_at	6,611	6,420	7,105	12,783	12,672	13,027
209387_s_at	5,447	5,392	5,575	11,837	11,168	11,762
209388_at	10,199	10,930	9,728	10,821	10,608	10,576
209389_x_at	11,437	11,426	11,118	11,210	10,710	11,344
209390_at	9,952	10,257	10,083	8,091	9,208	8,539
209391_at	8,928	8,445	9,039	9,007	8,399	9,050
209392_at	8,505	8,851	8,160	9,971	10,584	10,270
209393_s_at	11,878	11,869	11,796	11,389	10,923	11,522
209394_at	9,985	10,638	9,508	8,766	8,604	9,322
209395_at	6,907	6,579	5,831	8,747	6,340	7,621
209396_s_at	6,700	6,023	5,531	8,187	6,995	7,592
209397_at	10,944	11,301	10,846	10,700	10,856	10,523
209398_at	9,996	10,090	9,913	9,284	8,442	9,936
209399_at	8,643	8,953	8,498	8,022	8,026	8,080
209400_at	6,241	6,442	6,271	6,653	6,549	6,860

209401_s_at	5,930	5,674	6,203	6,333	6,245	6,405
209402_s_at	8,512	8,650	8,577	9,258	8,810	9,337
209403_at	8,833	8,621	8,460	8,078	8,565	8,013
209404_s_at	8,402	8,614	8,532	10,158	10,282	10,509
209405_s_at	7,301	7,638	7,907	8,375	8,162	8,216
209406_at	9,044	9,140	9,097	9,583	9,776	10,052
209407_s_at	8,131	7,729	7,610	6,711	6,500	6,814
209408_at	12,319	12,713	12,389	10,383	9,641	9,599
209409_at	11,842	11,742	12,141	9,534	9,843	9,580
209410_s_at	8,327	8,294	8,307	8,234	7,525	8,063
209411_s_at	9,322	9,173	9,496	8,266	8,042	8,806
209412_at	9,459	9,284	9,391	7,135	7,950	6,908
209413_at	8,547	8,641	8,730	8,557	8,165	8,456
209414_at	7,100	7,026	7,769	7,007	6,868	7,200
209415_at	8,673	8,548	8,651	8,868	8,840	8,974
209416_s_at	7,802	7,673	7,824	7,923	7,639	7,869
209417_s_at	6,808	6,500	6,827	8,772	8,086	8,513
209418_s_at	9,995	9,982	10,323	9,892	9,791	9,889
209419_at	4,865	5,070	5,257	5,403	5,023	5,369
209420_s_at	7,408	7,411	7,724	9,281	9,315	9,714
209421_at	12,469	12,517	11,709	8,269	8,981	7,245
209422_at	11,125	11,475	11,197	9,059	10,413	8,811
209423_s_at	7,113	6,928	7,034	7,367	7,200	7,480
209424_s_at	7,177	7,829	6,765	7,254	7,285	7,003
209425_at	7,774	8,296	7,954	6,741	6,568	6,094
209426_s_at	7,579	7,989	7,976	8,000	7,786	7,539
209427_at	7,219	7,110	7,258	9,186	8,648	9,454
209428_s_at	9,226	8,710	8,841	9,245	8,972	9,471
209429_x_at	10,736	10,628	11,236	10,904	10,617	11,064
209430_at	10,093	10,060	9,594	7,931	9,989	7,744
209431_s_at	9,672	8,888	9,619	6,727	6,307	6,536
209432_s_at	9,382	9,382	9,600	10,447	10,079	11,033
209433_s_at	11,719	11,635	11,020	9,004	8,826	8,342
209434_s_at	11,491	11,537	10,848	9,311	9,427	8,581
209435_s_at	9,849	9,772	9,928	9,795	8,704	9,324
209436_at	8,578	8,757	8,556	7,003	6,679	7,052
209437_s_at	7,727	7,466	7,297	7,511	7,406	7,515
209438_at	7,397	7,878	7,906	7,217	7,331	7,250
209439_s_at	8,844	8,968	8,771	8,350	8,395	8,774
209440_at	12,276	12,130	12,139	9,826	9,870	9,896
209441_at	7,658	7,492	7,642	7,854	7,712	7,867
209442_x_at	8,253	9,338	8,010	7,511	9,306	7,736
209443_at	7,069	7,232	6,743	7,613	7,803	7,575
209444_at	10,012	9,886	10,001	10,771	11,350	10,976
209445_x_at	10,232	10,872	9,899	9,563	8,779	9,349
209446_s_at	6,504	6,221	6,389	5,414	6,448	5,988
209447_at	6,368	6,248	6,594	6,016	7,821	7,048
209448_at	11,331	11,872	11,665	12,085	11,592	11,992
209449_at	9,147	8,948	9,097	9,076	8,057	8,612
209450_at	8,590	8,600	8,944	8,674	7,990	8,254
209451_at	8,746	9,117	8,123	9,320	10,719	9,366
209452_s_at	9,385	9,349	8,997	10,094	9,656	10,251
209453_at	8,444	8,293	8,645	9,062	8,843	9,225
209454_s_at	6,984	6,886	6,925	7,948	7,570	8,204
209455_at	10,015	9,972	9,853	9,031	9,856	9,221
209456_s_at	6,880	6,842	6,567	7,413	7,190	7,283

209457_at	8,732	9,215	9,236	10,034	10,505	10,757
209458_x_at	7,711	7,957	7,779	8,327	8,196	8,184
209459_s_at	8,945	9,515	9,134	4,760	6,452	6,348
209460_at	8,422	9,063	8,552	6,183	6,317	6,152
209461_x_at	9,904	9,532	9,744	9,276	8,460	9,231
209462_at	8,290	8,057	7,893	7,991	7,836	8,108
209463_s_at	9,085	9,010	8,815	8,292	7,852	8,500
209464_at	9,769	9,617	9,795	8,349	7,348	7,616
209465_x_at	9,802	9,170	8,921	6,507	6,674	6,386
209466_x_at	9,835	9,832	9,577	8,041	8,043	8,212
209467_s_at	9,247	9,379	9,260	8,853	9,135	9,265
209468_at	6,990	6,606	6,778	6,519	6,068	6,640
209469_at	6,680	7,292	5,983	4,471	4,545	4,486
209470_s_at	6,104	6,310	5,458	4,277	4,448	4,703
209471_s_at	10,691	10,856	9,964	8,463	8,627	8,995
209472_at	10,953	11,396	10,686	10,432	10,878	10,541
209473_at	6,769	7,156	6,797	7,215	7,062	7,288
209474_s_at	4,694	4,914	4,828	5,452	5,481	5,772
209475_at	8,624	8,717	8,397	7,255	8,225	6,951
209476_at	12,253	12,107	12,044	11,767	12,230	12,109
209477_at	8,373	8,477	8,539	8,017	7,835	8,247
209478_at	10,678	10,343	10,599	11,584	10,484	11,184
209479_at	8,134	8,374	8,086	7,795	7,850	7,402
209480_at	4,570	5,167	4,922	4,719	4,782	4,724
209481_at	10,190	9,985	10,202	7,026	8,368	7,161
209482_at	10,642	10,473	10,897	11,068	10,182	10,492
209483_s_at	5,779	7,362	6,421	5,228	5,207	5,456
209484_s_at	12,301	12,475	12,168	10,535	10,385	10,537
209485_s_at	9,120	9,430	8,971	7,868	8,030	8,597
209486_at	9,874	10,061	9,802	11,123	10,627	10,593
209487_at	10,997	11,246	11,746	11,262	11,097	11,529
209488_s_at	9,658	10,084	10,361	9,268	8,432	9,452
209489_at	11,891	12,003	12,065	9,756	10,537	10,086
209490_s_at	9,148	8,950	9,330	8,995	9,071	9,276
209491_s_at	6,713	6,817	6,536	7,743	7,320	7,500
209492_x_at	11,231	11,307	11,533	11,719	11,348	11,778
209493_at	7,208	8,130	8,166	5,085	4,570	4,692
209494_s_at	7,507	7,191	7,972	6,923	6,947	7,085
209495_at	8,567	8,348	8,281	8,277	8,270	8,248
209496_at	11,266	11,158	11,082	7,775	7,823	8,011
209497_s_at	8,732	8,385	8,704	7,831	7,197	7,626
209498_at	6,037	6,365	6,241	5,985	5,961	5,759
209499_x_at	7,030	7,032	7,017	7,222	7,120	7,203
209500_x_at	6,005	5,683	6,114	6,896	6,399	6,529
209501_at	9,902	10,250	10,157	9,709	10,056	9,865
209502_s_at	7,134	7,290	7,216	7,528	7,616	7,726
209503_s_at	12,024	11,614	11,973	11,780	11,462	11,643
209504_s_at	7,416	7,039	6,418	5,638	5,672	5,661
209505_at	5,871	4,747	4,960	8,818	10,961	10,674
209506_s_at	6,726	6,887	6,851	8,009	8,338	8,681
209507_at	11,808	12,077	11,590	10,783	9,889	10,076
209508_x_at	7,176	7,101	7,229	7,934	8,246	7,907
209509_s_at	9,180	8,870	9,084	9,639	8,484	9,774
209510_at	10,050	10,593	10,120	9,691	10,171	10,310
209511_at	9,971	9,519	9,872	9,963	9,449	10,007
209512_at	8,321	8,331	7,804	7,341	8,398	7,062

209513_s_at	9,400	9,939	9,078	9,319	10,431	8,985
209514_s_at	7,803	7,711	7,756	10,113	9,587	9,459
209515_s_at	6,983	7,051	6,685	6,958	7,883	7,158
209516_at	8,421	8,091	8,412	7,904	7,738	8,170
209517_s_at	11,995	12,201	12,156	10,385	10,578	10,258
209518_at	10,348	9,927	10,644	8,742	8,551	8,598
209519_at	4,770	4,333	4,346	3,976	3,988	3,862
209520_s_at	9,446	9,188	9,132	8,299	8,037	7,592
209521_s_at	6,336	6,033	6,774	6,437	6,250	6,557
209522_s_at	8,384	8,553	8,544	9,289	9,560	9,742
209523_at	9,867	9,881	9,315	9,074	10,590	9,018
209524_at	11,187	11,105	10,883	9,912	9,730	9,879
209525_at	7,281	7,153	7,202	5,190	5,170	4,946
209526_s_at	10,546	10,296	10,328	8,972	8,411	8,892
209527_at	10,438	10,258	10,745	9,817	8,819	8,873
209528_s_at	8,164	7,952	8,051	8,139	8,114	8,503
209529_at	9,596	9,783	9,932	7,148	6,843	6,976
209530_at	8,716	8,475	8,739	8,444	8,402	8,717
209531_at	7,822	8,174	7,758	8,745	8,332	8,479
209532_at	8,102	8,004	7,546	6,934	7,168	7,048
209533_s_at	10,384	10,319	10,435	9,378	9,617	9,332
209534_x_at	8,319	8,080	8,301	8,159	7,849	8,037
209535_s_at	7,659	8,108	7,893	7,831	8,248	7,532
209536_s_at	9,277	9,393	9,301	9,487	9,085	9,336
209537_at	11,123	11,255	11,010	9,380	10,828	10,365
209538_at	9,207	9,354	9,382	9,133	8,313	8,638
209539_at	8,322	8,988	8,585	8,274	8,292	8,259
209540_at	5,878	5,841	5,938	6,059	6,015	6,474
209541_at	5,812	5,167	5,474	6,234	5,500	5,563
209542_x_at	6,924	7,023	7,154	7,912	7,761	8,116
209543_s_at	7,631	7,842	7,703	7,902	8,087	7,712
209544_at	6,192	6,711	6,294	8,056	7,592	7,609
209545_s_at	8,219	8,586	8,338	10,161	10,367	9,997
209546_s_at	7,435	7,473	7,476	7,922	7,964	7,777
209547_s_at	9,583	9,392	9,790	9,524	9,236	9,576
209549_s_at	11,372	11,231	11,404	10,848	10,518	10,873
209550_at	11,349	11,220	11,616	9,263	8,736	10,508
209551_at	7,322	7,639	7,088	8,043	7,959	8,384
209552_at	6,804	7,199	7,272	8,342	8,553	8,665
209553_at	9,010	9,334	8,819	7,596	8,468	7,386
209554_at	5,148	5,598	5,678	5,979	5,725	6,110
209555_s_at	4,463	4,480	4,405	4,493	5,929	7,029
209556_at	8,900	8,550	9,289	8,504	7,990	8,493
209557_s_at	7,796	7,646	7,985	8,099	8,117	8,130
209558_s_at	8,232	8,073	8,289	8,299	8,460	8,543
209559_at	7,532	7,109	7,490	7,506	7,124	7,509
209560_s_at	10,798	10,471	9,974	6,819	6,550	6,592
209561_at	7,920	7,737	7,580	7,697	7,737	7,736
209563_x_at	12,006	11,877	11,952	11,820	12,082	12,183
209565_at	9,249	9,289	9,132	8,990	8,261	8,820
209566_at	8,640	9,025	8,166	7,718	8,263	7,708
209567_at	10,928	10,432	10,916	10,340	9,427	9,937
209568_s_at	9,043	8,822	9,176	10,212	10,646	10,069
209569_x_at	7,792	8,123	7,728	5,973	5,727	5,896
209570_s_at	9,911	10,213	10,259	8,061	7,927	8,271
209571_at	7,699	8,110	7,315	8,032	8,165	8,427

209572_s_at	11,284	11,484	10,975	10,544	10,284	10,190
209573_s_at	7,815	7,360	7,949	8,263	7,898	8,346
209574_s_at	6,885	6,765	6,591	5,734	5,960	5,836
209575_at	7,469	7,714	7,368	9,361	8,464	9,435
209576_at	9,014	9,376	8,635	8,995	9,244	9,354
209577_at	8,793	8,514	8,862	9,649	9,312	9,771
209578_s_at	8,568	8,682	8,884	8,674	9,011	9,420
209579_s_at	10,999	11,293	10,761	10,154	10,106	9,822
209580_s_at	9,603	9,994	9,333	8,465	8,303	7,851
209581_at	9,461	10,163	9,651	10,286	9,654	9,995
209582_s_at	8,252	8,139	8,054	5,325	5,323	5,662
209583_s_at	10,603	10,488	10,596	4,740	4,850	4,803
209584_x_at	7,615	7,586	7,352	8,011	7,755	7,818
209585_s_at	9,989	10,305	9,951	10,185	10,660	10,457
209586_s_at	11,021	11,313	11,504	9,486	9,120	9,217
209587_at	6,845	6,670	6,790	7,541	7,061	7,320
209588_at	8,038	7,653	7,753	8,177	8,012	8,102
209589_s_at	7,442	7,311	7,250	6,755	6,626	6,858
209590_at	7,977	8,311	7,792	7,002	6,848	7,014
209591_s_at	6,420	5,888	6,248	5,371	4,765	5,133
209592_s_at	8,114	8,345	8,028	8,397	7,584	8,356
209593_s_at	9,781	9,654	9,952	9,521	9,840	10,060
209594_x_at	6,696	6,288	6,567	6,810	6,784	6,610
209595_at	7,956	8,267	7,831	8,166	8,127	8,108
209596_at	8,709	8,251	8,781	7,247	7,278	7,357
209597_s_at	6,962	6,671	6,756	6,943	7,174	7,248
209598_at	11,248	11,459	10,907	7,239	9,171	8,141
209599_s_at	5,939	5,926	5,975	5,671	5,285	5,777
209600_s_at	8,329	8,712	8,606	9,255	9,483	9,581
209601_at	5,056	5,249	5,664	5,329	5,267	5,404
209602_s_at	4,993	5,117	5,295	4,045	3,848	3,973
209603_at	6,269	5,741	5,379	5,421	5,179	5,354
209604_s_at	7,055	7,614	6,660	6,707	6,364	6,637
209605_at	10,201	10,427	10,784	10,385	9,991	11,024
209606_at	5,646	6,054	5,144	5,822	7,058	7,083
209607_x_at	8,667	8,384	8,504	7,150	7,207	7,756
209608_s_at	11,764	11,407	11,566	11,162	10,800	10,842
209609_s_at	11,831	11,821	12,262	11,816	11,103	11,701
209610_s_at	7,485	7,427	8,039	6,446	7,337	8,124
209611_s_at	6,579	6,966	7,183	7,375	6,896	7,175
209612_s_at	4,445	4,670	4,480	4,962	5,407	4,931
209613_s_at	3,586	3,695	3,600	3,492	3,890	3,379
209614_at	6,258	6,498	6,750	7,536	7,761	7,790
209615_s_at	8,294	8,134	8,363	6,735	6,150	6,453
209616_s_at	5,729	5,962	6,278	8,384	7,218	7,053
209617_s_at	6,497	6,799	6,595	5,483	5,527	5,577
209618_at	5,613	6,086	5,834	4,105	3,975	4,185
209619_at	7,011	7,272	7,332	7,859	7,562	7,808
209620_s_at	10,528	11,207	10,208	8,561	8,355	8,239
209621_s_at	7,477	8,203	7,409	6,449	6,110	6,539
209622_at	6,982	6,719	7,202	7,371	7,146	7,478
209623_at	10,711	10,747	11,019	9,770	9,707	9,807
209624_s_at	7,157	7,331	7,163	7,384	6,840	7,226
209625_at	6,717	6,545	6,317	8,883	8,151	9,006
209626_s_at	7,648	7,696	7,301	6,730	7,769	7,288
209627_s_at	9,158	9,147	8,889	6,444	7,640	6,814

209628_at	10,840	11,158	10,608	9,047	9,454	9,058
209629_s_at	7,177	7,328	6,932	6,849	6,399	6,847
209630_s_at	10,976	10,890	10,800	8,629	9,537	8,531
209631_s_at	6,025	7,839	6,931	4,669	5,086	5,052
209632_at	8,205	8,223	8,144	7,944	8,678	8,109
209633_at	10,041	9,966	9,777	9,645	10,601	9,448
209635_at	8,872	9,073	8,890	9,384	9,340	9,560
209636_at	6,617	7,034	6,433	7,332	7,259	7,596
209637_s_at	6,436	6,348	6,255	6,171	6,280	6,294
209638_x_at	7,239	7,259	7,264	7,593	7,725	7,382
209639_s_at	7,076	6,967	7,136	7,107	7,117	7,106
209640_at	6,171	6,060	6,250	6,596	6,065	6,624
209641_s_at	6,671	6,547	6,688	8,518	8,652	9,038
209642_at	11,394	11,492	10,845	8,612	8,061	6,857
209643_s_at	8,725	8,537	8,786	9,160	9,062	9,279
209644_x_at	7,265	6,874	7,009	8,913	8,556	8,947
209645_s_at	7,517	7,163	7,481	8,314	8,024	8,268
209646_x_at	7,476	7,768	8,224	8,135	7,561	8,323
209647_s_at	10,564	10,652	10,709	9,673	10,023	9,636
209648_x_at	8,640	8,708	8,766	8,478	10,071	9,583
209649_at	8,596	8,771	8,220	7,963	9,086	8,219
209650_s_at	8,548	8,444	8,260	9,036	8,465	9,001
209651_at	6,128	6,733	6,813	10,248	9,380	10,663
209652_s_at	6,634	6,819	6,598	7,260	7,518	7,557
209653_at	8,551	8,344	8,442	8,303	8,665	8,335
209654_at	9,911	9,811	9,936	7,861	9,478	7,348
209655_s_at	7,503	7,560	6,993	7,165	8,543	9,030
209656_s_at	9,579	9,578	8,923	8,702	11,134	10,878
209657_s_at	9,514	9,692	9,019	7,223	7,599	7,162
209658_at	10,793	11,069	11,012	10,641	11,089	10,677
209659_s_at	11,843	11,802	11,623	10,822	11,360	10,875
209660_at	7,919	7,788	8,025	8,423	8,102	8,290
209661_at	7,328	6,795	7,302	8,785	7,840	8,150
209662_at	11,418	12,304	11,075	10,605	10,062	10,220
209663_s_at	7,438	7,208	7,200	7,118	7,416	7,578
209664_x_at	6,321	6,400	6,603	7,202	7,004	7,241
209665_at	8,639	8,391	8,707	9,883	8,936	10,196
209666_s_at	9,778	9,741	9,602	9,627	10,123	9,224
209667_at	8,155	7,999	8,720	7,997	7,947	8,278
209668_x_at	8,371	8,014	8,284	8,081	8,195	8,202
209669_s_at	13,500	13,560	13,383	12,308	11,822	12,029
209670_at	7,738	7,771	7,655	8,111	8,272	8,061
209671_x_at	6,796	6,867	6,880	8,016	8,076	8,170
209672_s_at	6,545	6,427	6,772	6,272	7,390	6,249
209674_at	11,230	11,555	11,075	8,486	9,805	8,802
209675_s_at	8,607	8,272	8,339	8,641	8,501	8,651
209676_at	5,307	5,657	5,102	8,128	9,675	8,805
209677_at	5,099	4,989	4,969	4,622	4,855	4,893
209678_s_at	11,521	11,525	10,740	8,984	9,499	8,641
209679_s_at	8,755	9,261	9,100	10,487	9,841	10,510
209680_s_at	8,754	8,774	8,558	8,846	8,702	8,649
209681_at	10,104	10,257	9,925	8,115	9,291	8,197
209682_at	9,100	9,868	9,297	8,527	9,928	8,717
209683_at	5,916	6,176	5,467	5,499	6,298	5,491
209684_at	7,368	7,646	7,010	9,848	10,128	9,875
209685_s_at	7,484	7,870	7,496	6,460	6,228	6,433

209686_at	5,616	5,854	5,754	6,769	6,477	7,009
209687_at	10,017	10,300	10,297	6,191	6,062	6,069
209688_s_at	9,169	9,543	9,032	7,437	8,272	7,442
209689_at	7,580	7,678	7,222	7,143	7,202	7,100
209690_s_at	7,296	7,392	7,092	8,408	8,018	8,385
209691_s_at	7,987	7,632	7,516	7,707	7,608	7,996
209692_at	7,020	7,303	6,765	5,498	6,987	5,404
209693_at	8,732	8,651	8,015	7,478	7,212	7,478
209694_at	10,341	10,586	9,992	11,717	11,003	11,628
209695_at	8,290	8,525	8,598	5,896	6,106	6,373
209696_at	6,642	6,841	6,630	6,675	6,320	6,168
209697_at	6,274	6,217	6,026	6,523	6,181	6,431
209698_at	8,974	8,901	8,736	8,711	8,461	8,591
209699_x_at	7,943	8,344	8,134	13,702	13,619	13,780
209700_x_at	4,060	4,704	4,067	7,280	5,929	4,760
209701_at	6,324	6,989	6,103	7,144	7,051	7,246
209702_at	9,369	9,485	9,844	9,280	9,367	9,565
209703_x_at	8,021	7,446	8,323	7,358	7,831	7,380
209704_at	11,001	11,071	10,565	6,515	7,102	5,904
209705_at	10,604	11,158	10,057	6,230	6,770	6,246
209706_at	7,610	7,748	8,052	8,183	7,455	7,572
209707_at	9,302	9,703	9,201	9,211	9,714	9,848
209708_at	7,559	7,788	7,049	7,841	9,623	7,791
209709_s_at	10,899	11,373	10,417	8,741	9,159	7,225
209710_at	8,181	8,668	8,226	7,956	8,023	7,928
209711_at	8,299	8,030	7,976	8,713	9,239	8,448
209712_at	8,295	7,939	8,147	7,906	8,422	7,927
209713_s_at	5,892	6,168	6,495	7,969	7,320	7,446
209714_s_at	9,325	9,698	9,100	9,517	9,052	8,919
209715_at	10,911	10,749	10,420	9,719	8,947	9,131
209716_at	7,647	7,656	8,018	8,137	8,102	8,273
209717_at	8,137	8,331	8,149	7,326	8,924	7,434
209718_at	8,008	7,996	8,193	8,276	7,970	8,365
209719_x_at	5,185	5,254	5,461	5,343	5,080	5,291
209720_s_at	3,851	4,232	4,193	4,081	4,129	4,076
209721_s_at	9,880	9,891	9,935	9,726	9,810	9,995
209722_s_at	5,865	5,665	5,897	5,837	5,390	5,761
209723_at	9,984	10,405	11,479	5,688	5,972	4,436
209724_s_at	8,622	8,754	8,460	7,514	7,164	6,969
209725_at	9,884	9,275	9,822	8,609	8,541	8,343
209726_at	9,230	8,770	9,713	7,253	7,123	7,105
209727_at	8,047	7,928	8,007	8,519	8,285	8,495
209728_at	6,878	6,920	6,825	6,866	6,495	7,268
209729_at	7,568	7,815	8,219	8,985	8,924	9,298
209730_at	7,577	7,923	7,573	7,877	7,790	7,977
209731_at	9,511	9,210	9,565	8,656	8,181	8,603
209732_at	6,218	5,804	5,940	6,948	6,629	6,730
209733_at	7,218	7,603	6,868	7,044	7,391	7,269
209734_at	7,093	6,982	7,234	8,305	7,982	8,167
209735_at	8,940	9,600	9,049	7,632	6,565	6,878
209736_at	8,360	8,466	8,596	7,593	7,436	7,819
209737_at	7,154	7,439	7,841	5,745	6,135	6,107
209738_x_at	5,770	5,502	5,596	5,888	5,898	5,860
209739_s_at	9,928	10,019	9,891	9,571	8,495	9,044
209740_s_at	8,152	8,396	8,463	6,380	5,754	5,902
209741_x_at	6,835	7,746	6,308	5,587	6,115	5,301

209742_s_at	5,705	5,837	5,492	6,430	6,611	6,518
209743_s_at	6,556	6,675	6,715	6,170	6,078	5,933
209744_x_at	8,502	8,764	8,462	8,318	8,513	8,155
209745_at	8,643	8,779	8,917	7,984	7,699	7,939
209746_s_at	8,307	8,360	8,037	7,936	7,712	7,934
209747_at	6,802	7,059	7,052	8,047	7,830	7,975
209748_at	10,118	10,188	10,044	7,838	8,691	7,478
209749_s_at	5,488	4,989	5,274	5,733	5,721	5,899
209750_at	9,813	10,348	9,580	7,736	9,246	7,111
209751_s_at	8,407	8,774	8,405	9,196	8,043	9,331
209752_at	6,810	7,028	7,039	7,503	7,308	7,680
209753_s_at	7,811	7,749	7,742	7,580	6,783	6,899
209754_s_at	9,141	8,053	8,756	5,895	5,533	4,843
209755_at	8,561	8,960	8,679	7,155	6,987	7,281
209756_s_at	7,463	7,459	7,434	8,054	8,174	8,492
209757_s_at	10,601	10,362	10,806	6,229	6,150	6,429
209758_s_at	6,401	6,392	6,134	6,384	6,026	6,154
209759_s_at	10,357	10,707	10,588	9,411	8,717	8,984
209760_at	8,918	8,974	9,006	7,948	8,118	8,033
209761_s_at	6,921	6,538	6,870	7,393	7,051	7,690
209762_x_at	7,628	8,040	8,217	8,369	8,446	8,456
209763_at	10,513	10,559	10,151	6,557	7,279	6,631
209764_at	7,692	7,557	7,655	7,823	7,683	7,728
209765_at	8,503	8,144	8,138	8,038	8,024	8,857
209766_at	7,205	7,057	7,239	7,349	6,863	6,739
209767_s_at	7,530	7,678	7,635	8,163	8,284	8,470
209768_s_at	6,640	6,693	6,716	7,755	7,927	7,931
209769_s_at	7,754	7,847	7,703	8,686	8,745	8,741
209770_at	6,742	6,994	6,682	7,775	7,236	7,365
209771_x_at	13,545	13,438	13,599	6,854	7,389	6,815
209772_s_at	8,591	8,229	8,585	5,641	5,666	5,850
209773_s_at	12,218	11,921	12,467	12,834	12,504	12,410
209774_x_at	4,817	5,418	4,880	11,475	10,063	8,111
209775_x_at	6,029	5,981	6,291	6,719	6,340	6,618
209776_s_at	6,227	6,155	6,403	6,751	6,906	6,984
209777_s_at	8,274	8,005	8,561	8,345	7,942	8,268
209778_at	6,705	7,249	6,552	6,625	8,172	6,278
209779_at	7,231	7,297	6,763	6,976	6,727	7,067
209780_at	10,252	10,288	9,794	9,510	10,603	8,838
209781_s_at	8,479	8,850	8,620	8,617	8,153	8,027
209782_s_at	6,951	7,396	6,925	6,859	6,703	6,694
209783_at	6,467	6,374	6,581	7,058	7,430	7,348
209784_s_at	8,701	9,115	8,934	8,395	8,195	7,949
209785_s_at	6,478	6,860	6,485	6,443	5,996	6,343
209786_at	10,804	11,013	10,728	11,398	11,053	11,352
209787_s_at	11,406	11,542	11,423	11,778	11,523	11,804
209788_s_at	7,287	7,552	7,702	7,049	8,045	7,166
209789_at	7,503	6,913	7,628	8,117	8,090	8,526
209790_s_at	8,862	8,890	8,720	8,836	7,973	8,584
209791_at	7,662	8,023	7,750	7,862	7,319	7,458
209792_s_at	7,656	8,500	7,708	7,044	6,769	6,768
209793_at	6,309	6,474	6,277	6,057	5,862	5,626
209794_at	8,277	8,227	8,248	7,694	7,801	8,050
209795_at	4,157	4,569	4,261	4,152	4,241	4,358
209796_s_at	9,865	9,758	10,001	10,029	8,508	9,970
209797_at	9,518	9,494	9,937	10,423	9,628	10,456

209798_at	9,980	10,166	9,684	7,683	8,415	6,432
209799_at	7,038	7,605	6,939	7,902	8,690	7,815
209800_at	6,356	5,973	6,563	7,924	7,691	7,861
209802_at	6,453	6,505	6,730	7,448	7,852	7,113
209803_s_at	10,577	11,148	11,196	12,471	12,744	12,803
209804_at	9,525	9,785	9,096	7,790	7,836	7,356
209805_at	8,598	8,757	8,442	8,064	8,369	7,939
209806_at	8,426	8,557	9,583	10,095	10,670	11,278
209807_s_at	6,822	6,238	6,700	7,751	7,941	8,418
209808_x_at	8,399	8,666	8,626	8,078	7,680	7,944
209810_at	7,475	7,710	7,556	7,655	7,723	7,631
209811_at	7,550	6,723	7,906	5,894	5,499	5,830
209812_x_at	7,584	7,060	7,142	7,746	7,597	7,868
209813_x_at	5,030	5,002	4,828	5,597	5,397	5,674
209814_at	11,142	11,716	10,557	10,513	10,648	10,382
209815_at	12,144	11,827	12,016	5,686	6,827	6,339
209816_at	9,039	8,776	8,696	5,584	5,445	5,277
209817_at	9,325	9,722	9,847	10,034	10,192	10,357
209818_s_at	7,430	7,646	7,029	7,270	7,497	7,279
209819_at	5,118	5,304	4,942	5,806	6,557	6,619
209820_s_at	8,871	8,788	8,874	8,438	7,980	8,373
209821_at	3,551	3,851	4,002	3,721	3,640	3,717
209822_s_at	8,922	9,546	9,271	5,145	5,301	5,417
209823_x_at	7,223	7,139	7,265	7,497	6,989	7,260
209824_s_at	7,835	8,156	7,815	8,783	9,334	8,993
209825_s_at	10,677	10,263	10,413	10,593	9,599	10,429
209826_at	7,320	7,301	7,424	7,098	7,215	7,167
209827_s_at	7,364	7,423	7,481	7,934	7,660	8,093
209828_s_at	6,662	6,472	6,407	7,343	7,384	7,504
209829_at	3,956	3,996	3,921	3,876	3,802	3,878
209830_s_at	5,157	5,598	5,395	6,566	6,556	6,734
209831_x_at	8,782	8,910	8,586	8,960	8,783	8,970
209832_s_at	8,626	8,976	8,450	7,344	6,882	7,359
209833_at	8,703	8,987	8,791	9,088	8,570	9,363
209834_at	7,932	7,677	7,945	7,826	8,447	8,449
209835_x_at	4,977	5,542	5,138	9,723	8,845	9,117
209836_x_at	10,318	10,439	10,699	11,039	10,069	10,628
209837_at	8,281	8,122	8,274	7,811	7,363	8,185
209838_at	9,313	9,630	9,381	7,836	8,645	7,340
209839_at	5,781	6,127	5,438	5,248	5,172	5,101
209840_s_at	9,044	8,611	8,100	6,297	6,850	5,732
209841_s_at	9,839	8,827	8,382	7,179	7,531	6,976
209842_at	6,794	6,606	6,774	7,517	7,502	7,464
209843_s_at	7,622	7,441	7,583	7,929	7,635	7,773
209844_at	6,058	5,653	6,110	6,882	6,336	6,785
209845_at	10,077	10,424	10,218	8,538	7,768	8,660
209846_s_at	5,326	5,823	5,366	8,121	6,826	7,190
209847_at	5,787	5,840	5,788	6,026	5,910	5,864
209848_s_at	9,177	9,234	8,615	7,247	6,803	6,899
209849_s_at	9,794	9,771	9,439	9,802	9,750	9,213
209850_s_at	9,181	8,958	9,211	10,117	9,928	10,124
209851_at	7,217	6,950	6,917	7,479	7,420	7,375
209852_x_at	11,169	10,506	11,403	11,220	10,277	10,995
209853_s_at	9,414	9,049	9,455	10,981	10,358	10,643
209854_s_at	6,346	5,951	6,341	7,708	7,780	7,744
209855_s_at	6,832	7,055	6,820	7,667	7,819	7,821

209856_x_at	9,015	8,903	8,915	9,038	8,871	9,109
209857_s_at	7,991	7,837	8,032	7,767	7,465	7,659
209858_x_at	8,907	9,093	9,109	9,442	9,790	10,013
209859_at	7,451	7,482	7,383	7,259	7,292	7,341
209860_s_at	10,405	10,692	10,450	10,860	11,166	11,425
209861_s_at	12,171	12,106	11,260	10,487	11,242	10,065
209862_s_at	8,438	8,697	8,167	7,102	6,855	6,620
209863_s_at	5,309	5,513	6,249	6,138	5,701	5,953
209864_at	10,235	10,166	10,448	8,413	7,540	7,955
209865_at	6,945	7,332	6,361	5,696	6,012	5,824
209866_s_at	7,483	6,988	6,717	6,012	5,818	5,938
209867_s_at	8,554	8,447	8,647	7,232	6,850	7,230
209868_s_at	9,448	9,492	9,115	8,222	9,163	8,483
209869_at	6,903	7,222	7,600	6,350	6,057	6,318
209870_s_at	8,919	8,731	9,229	9,224	8,812	9,142
209871_s_at	9,257	9,402	9,592	8,274	7,680	8,184
209872_s_at	6,194	6,261	6,085	7,092	7,013	7,003
209873_s_at	7,618	7,971	7,988	8,351	8,240	8,335
209874_x_at	8,551	8,916	8,997	8,377	8,223	8,215
209875_s_at	10,695	11,269	10,598	6,574	7,801	7,727
209876_at	6,645	6,755	6,927	7,142	7,118	7,472
209877_at	7,211	6,980	7,266	7,464	7,235	7,638
209878_s_at	7,168	7,236	7,448	7,851	7,425	7,862
209879_at	6,980	7,017	7,004	7,411	7,590	7,916
209880_s_at	7,617	7,523	7,671	7,779	7,897	7,956
209881_s_at	8,122	7,802	7,811	8,118	8,283	8,312
209882_at	9,781	10,080	9,462	9,849	8,710	9,482
209883_at	8,389	8,116	8,491	6,988	7,339	7,962
209884_s_at	9,854	10,135	9,367	8,686	10,031	8,690
209885_at	7,381	7,417	7,783	7,192	7,576	7,740
209886_s_at	6,852	6,391	6,479	7,479	7,684	7,545
209887_at	5,654	5,307	5,462	6,035	6,179	6,399
209888_s_at	6,488	5,990	6,799	7,755	7,390	7,281
209889_at	6,984	7,346	7,305	7,260	6,771	6,995
209890_at	9,780	9,878	10,016	11,354	11,915	12,004
209891_at	9,852	9,850	9,342	8,908	7,786	7,646
209892_at	8,360	8,233	8,323	6,867	6,986	7,400
209893_s_at	7,613	7,780	8,271	8,640	8,514	8,701
209894_at	8,425	9,317	7,959	6,651	10,702	8,591
209895_at	8,882	8,920	8,537	8,078	7,940	7,604
209896_s_at	9,706	9,849	9,496	8,936	9,020	8,535
209897_s_at	8,572	8,634	8,323	9,805	11,279	10,379
209898_x_at	9,509	10,005	9,496	8,603	9,861	8,139
209899_s_at	10,436	9,904	10,200	10,687	9,987	10,635
209900_s_at	11,276	11,392	11,056	9,369	9,794	9,473
209901_x_at	7,403	7,452	7,394	7,411	7,531	7,385
209902_at	9,477	9,753	9,067	7,693	9,178	7,003
209903_s_at	10,816	11,046	10,507	9,429	10,820	8,744
209904_at	5,907	5,967	6,598	7,002	6,929	7,130
209905_at	6,131	5,867	5,651	10,346	9,850	10,009
209906_at	6,220	6,179	6,284	8,064	7,775	8,285
209907_s_at	7,146	7,419	6,989	6,902	7,875	6,539
209908_s_at	3,861	3,768	3,759	4,545	4,762	4,595
209909_s_at	4,580	5,676	5,187	6,349	7,071	6,612
209910_at	6,757	7,144	7,117	6,867	7,311	7,440
209911_x_at	8,351	8,876	8,756	7,392	7,795	8,280

209912_s_at	7,783	7,258	7,775	8,213	8,162	8,397
209913_x_at	10,748	10,320	11,583	7,602	7,793	8,238
209914_s_at	6,616	6,728	6,417	7,206	6,704	7,024
209915_s_at	5,002	5,009	5,098	5,271	4,887	5,257
209916_at	9,238	9,527	9,284	7,493	6,991	7,213
209917_s_at	6,409	7,155	6,545	7,219	7,119	7,554
209918_at	7,229	7,268	7,227	7,844	7,705	7,695
209919_x_at	8,261	8,141	8,429	7,956	7,532	8,073
209920_at	7,349	7,377	7,251	7,839	8,111	8,080
209921_at	9,931	10,355	10,162	10,441	11,991	10,471
209922_at	7,192	7,596	7,580	7,099	6,563	6,793
209923_s_at	9,055	9,093	9,141	9,003	9,224	9,155
209924_at	6,784	6,918	6,958	7,442	7,313	7,536
209925_at	6,333	6,733	6,331	5,850	5,668	5,856
209926_at	8,012	8,296	8,192	8,644	8,295	8,535
209927_s_at	8,659	8,716	8,366	8,090	7,701	7,717
209928_s_at	6,996	6,969	6,622	6,983	6,714	6,908
209929_s_at	9,103	9,031	9,269	9,679	9,587	9,995
209930_s_at	5,781	5,320	5,872	6,338	6,322	6,624
209931_s_at	8,673	8,985	8,217	7,356	6,907	7,414
209932_s_at	11,136	11,270	10,890	11,355	10,877	11,222
209933_s_at	6,915	6,769	6,853	7,171	7,502	7,630
209934_s_at	8,129	8,499	8,241	8,603	9,270	8,861
209935_at	8,234	8,537	8,277	8,783	9,818	9,189
209936_at	5,948	5,320	5,582	5,692	5,616	5,733
209937_at	6,610	6,356	6,545	6,965	6,781	7,220
209938_at	7,894	7,561	7,817	7,637	7,455	7,189
209939_x_at	8,659	8,703	8,598	9,183	9,333	10,268
209940_at	7,412	7,533	7,457	8,581	8,682	8,902
209941_at	7,511	7,314	7,450	7,756	7,778	7,465
209942_x_at	4,931	5,609	5,942	5,694	5,607	6,043
209943_at	7,154	7,565	7,159	7,576	7,329	7,519
209944_at	10,147	10,148	10,220	11,096	10,668	10,888
209945_s_at	8,749	8,922	8,956	8,772	8,778	8,748
209946_at	7,121	7,993	7,418	11,356	11,919	11,109
209947_at	9,145	9,084	9,195	8,626	8,375	8,303
209948_at	7,209	6,810	7,156	7,263	6,919	7,218
209949_at	5,409	5,990	5,900	6,377	6,414	6,300
209950_s_at	6,604	6,637	6,482	7,412	7,201	7,372
209951_s_at	5,272	5,613	5,627	6,641	6,431	6,923
209952_s_at	6,425	6,345	6,656	7,230	7,004	7,196
209953_s_at	9,719	8,944	8,982	9,280	8,661	9,605
209954_x_at	7,835	7,448	7,512	9,106	8,391	8,713
209955_s_at	5,436	6,140	5,676	8,805	9,931	8,523
209956_s_at	4,945	5,313	5,046	5,214	5,084	5,109
209957_s_at	7,735	7,438	7,434	7,875	7,967	7,891
209958_s_at	8,160	8,527	7,607	7,709	7,985	7,924
209959_at	7,211	7,351	7,195	8,394	9,552	8,473
209960_at	3,962	4,866	4,311	9,411	10,747	7,338
209961_s_at	6,812	6,602	6,988	7,849	8,100	7,779
209962_at	6,861	7,253	6,756	7,129	6,850	7,104
209963_s_at	7,226	7,296	7,132	7,835	8,056	8,046
209964_s_at	7,379	7,164	7,438	7,331	7,617	7,170
209965_s_at	8,061	7,789	8,210	8,108	7,872	7,921
209966_x_at	5,738	6,511	5,563	5,478	5,384	5,438
209967_s_at	9,314	9,573	9,746	8,748	8,268	8,351

209968_s_at	7,373	7,034	7,200	8,295	8,123	8,333
209969_s_at	7,822	8,226	7,782	9,491	10,248	9,633
209970_x_at	6,810	6,486	6,027	8,699	8,389	7,697
209971_x_at	12,758	12,572	13,150	12,125	11,502	11,933
209972_s_at	8,289	8,300	8,510	7,834	7,843	8,014
209973_at	7,372	7,500	7,594	8,273	8,233	8,485
209974_s_at	11,942	12,119	11,768	12,166	12,185	12,169
209975_at	7,986	8,268	8,567	7,276	7,001	6,587
209976_s_at	6,258	6,257	6,592	6,529	6,148	6,258
209977_at	6,340	6,483	6,382	6,715	6,484	6,667
209978_s_at	6,433	6,069	6,397	6,447	6,516	6,584
209979_at	6,905	7,184	6,882	7,544	7,621	7,516
209980_s_at	8,224	8,307	8,030	7,188	7,214	7,143
209981_at	7,122	6,977	7,053	7,130	7,043	6,955
209982_s_at	7,424	6,817	6,442	6,088	6,369	6,406
209983_s_at	6,902	6,595	6,580	6,922	6,713	6,796
209984_at	8,261	8,880	8,289	7,263	7,911	7,267
209985_s_at	6,571	6,571	6,676	7,546	7,680	7,525
209986_at	5,417	5,252	5,093	5,904	5,485	5,866
209987_s_at	5,335	5,242	5,295	5,356	5,322	5,523
209988_s_at	6,239	7,211	6,650	5,081	5,201	5,044
209989_at	9,353	9,342	8,783	6,126	6,932	6,231
209990_s_at	7,101	7,309	7,978	4,897	4,817	6,342
209991_x_at	5,656	5,646	5,581	6,175	5,974	6,253
209992_at	6,774	7,069	6,488	5,753	5,547	5,663
209993_at	5,598	5,432	5,591	5,607	5,352	5,303
209994_s_at	7,679	8,086	6,931	7,366	7,441	7,865
209995_s_at	7,514	7,464	7,718	7,455	7,499	7,872
209996_x_at	7,441	6,911	6,936	7,421	7,539	7,815
209997_x_at	6,171	6,049	5,900	6,382	6,115	5,961
209998_at	7,951	8,117	8,340	8,604	7,830	8,486
209999_x_at	6,778	6,769	6,618	7,092	6,991	7,536
210000_s_at	5,352	5,231	4,802	5,672	5,267	5,386
210001_s_at	7,892	7,770	7,824	6,589	6,632	6,613
210002_at	4,150	4,536	4,444	4,685	7,816	6,468
210004_at	4,022	3,933	4,418	4,363	4,286	4,298
210005_at	9,112	9,274	9,117	8,300	7,766	8,276
210006_at	6,546	6,706	6,767	7,861	7,375	7,930
210007_s_at	6,572	6,439	6,288	6,590	7,555	7,018
210008_s_at	7,136	7,063	7,222	8,078	8,180	8,306
210009_s_at	7,018	7,182	7,419	8,805	8,284	8,727
210010_s_at	8,773	8,625	8,676	9,556	8,994	9,858
210011_s_at	9,762	9,384	9,662	9,300	8,618	8,902
210012_s_at	6,945	6,843	6,923	5,758	6,114	5,673
210013_at	7,538	7,249	7,682	7,729	7,621	7,710
210014_x_at	11,567	11,451	11,752	11,109	10,597	11,142
210015_s_at	8,758	8,789	8,790	6,383	6,519	6,620
210016_at	4,427	4,771	5,043	5,272	5,062	5,500
210017_at	7,241	7,513	7,353	6,523	6,764	6,360
210018_x_at	7,752	8,190	7,673	7,189	7,100	6,858
210019_at	6,092	6,273	5,985	6,651	6,475	6,519
210020_x_at	6,174	6,019	6,453	7,057	7,227	7,352
210021_s_at	6,412	6,406	6,411	7,246	7,045	7,000
210022_at	8,873	9,071	9,113	9,228	9,194	9,306
210023_s_at	8,748	8,955	8,917	8,681	8,104	8,585
210024_s_at	12,054	12,143	12,147	11,806	12,560	12,244

210025_s_at	8,387	8,107	8,307	8,519	8,451	8,495
210026_s_at	8,940	9,080	9,334	9,238	9,966	9,709
210027_s_at	12,867	12,771	13,156	12,395	11,996	12,342
210028_s_at	10,256	10,759	10,051	8,775	9,663	9,134
210029_at	8,690	9,199	8,408	7,402	6,870	7,404
210030_at	6,307	6,092	6,332	6,175	5,670	5,910
210031_at	5,581	5,768	5,726	6,223	5,906	6,035
210032_s_at	5,535	5,576	5,773	6,074	5,821	6,145
210033_s_at	5,281	6,006	5,399	5,401	5,269	5,083
210034_s_at	7,309	7,506	7,231	5,377	6,093	5,357
210035_s_at	5,004	4,953	5,230	5,832	5,445	5,696
210036_s_at	8,458	8,063	8,159	7,338	7,653	7,828
210037_s_at	6,413	6,624	6,126	6,755	6,487	6,753
210038_at	8,334	8,318	8,145	6,239	6,280	6,267
210039_s_at	7,672	7,823	7,576	7,600	7,361	7,507
210040_at	6,163	6,000	5,931	5,964	5,574	5,940
210041_s_at	8,665	9,468	8,262	10,026	10,194	10,044
210042_s_at	7,216	7,115	6,853	8,817	8,617	8,916
210043_at	6,701	6,509	6,790	6,814	6,582	6,608
210044_s_at	7,276	7,235	7,272	7,423	7,169	7,350
210045_at	8,250	8,035	8,036	7,020	6,884	7,090
210046_s_at	9,760	9,885	9,630	8,902	8,343	8,986
210047_at	7,297	7,215	7,224	7,997	7,107	7,097
210048_at	8,002	7,910	7,401	8,377	7,996	8,401
210049_at	5,440	5,374	5,186	5,543	5,481	5,494
210050_at	7,152	6,886	7,604	5,912	5,648	5,884
210051_at	6,728	6,860	7,034	7,913	8,086	8,101
210052_s_at	11,438	11,390	11,350	10,070	9,896	9,370
210053_at	10,561	10,905	10,288	7,653	7,453	6,974
210054_at	9,525	9,887	9,269	7,998	8,864	7,867
210055_at	4,516	4,461	4,439	4,834	4,888	4,915
210056_at	8,502	8,790	8,564	7,260	6,958	7,137
210057_at	6,843	6,482	6,249	4,031	4,814	4,056
210058_at	6,406	6,861	6,534	8,548	7,992	8,288
210059_s_at	6,459	6,473	6,931	8,520	8,182	8,442
210060_at	7,577	7,226	7,599	8,008	8,219	8,462
210061_at	8,255	7,337	7,564	6,954	6,651	6,991
210062_s_at	8,609	7,895	8,156	5,638	5,577	5,691
210063_at	7,181	7,262	7,077	7,639	7,349	7,454
210064_s_at	6,388	6,440	6,436	6,500	6,269	6,247
210065_s_at	6,333	6,818	6,598	6,411	6,803	6,764
210066_s_at	4,055	4,023	4,611	4,224	4,092	4,007
210067_at	3,785	3,960	3,863	4,167	4,027	4,305
210068_s_at	5,448	5,472	5,599	5,931	5,633	5,466
210069_at	8,372	8,339	8,503	6,474	6,648	6,574
210070_s_at	7,526	7,599	7,348	6,788	6,784	6,770
210072_at	6,177	6,344	6,006	6,606	6,504	6,807
210073_at	7,395	7,723	6,780	5,910	5,507	5,820
210074_at	10,969	11,068	10,611	8,254	7,748	7,851
210075_at	8,105	8,473	7,807	7,057	7,310	7,524
210076_x_at	10,471	10,397	10,381	9,929	10,061	9,753
210077_s_at	5,707	6,121	5,553	6,411	6,398	6,711
210078_s_at	5,246	5,101	4,944	4,808	4,882	5,017
210079_x_at	6,900	6,725	7,074	6,917	7,032	6,994
210080_x_at	6,163	6,052	6,171	6,234	6,063	6,399
210081_at	5,831	5,868	5,949	7,054	6,947	6,914

210082_at	7,583	7,627	7,502	7,848	7,834	7,841
210083_at	6,897	7,183	7,284	7,171	6,887	7,198
210084_x_at	6,738	6,544	6,621	7,678	7,339	7,592
210085_s_at	6,537	6,879	6,895	7,319	7,074	7,604
210086_at	7,319	7,471	7,313	7,985	8,168	8,014
210087_s_at	7,313	7,288	7,079	9,589	8,792	9,382
210088_x_at	7,413	7,038	7,202	7,466	7,029	7,465
210089_s_at	6,122	5,968	5,687	9,078	8,827	9,168
210090_at	7,142	7,426	6,984	6,599	6,933	7,060
210091_s_at	6,126	7,128	6,115	5,705	5,648	5,462
210092_at	11,151	10,941	10,535	9,575	8,438	8,939
210093_s_at	11,775	11,508	11,289	10,325	9,835	9,960
210094_s_at	9,417	9,791	9,798	8,269	8,383	8,066
210095_s_at	7,415	7,663	8,026	7,858	13,126	11,575
210096_at	5,125	5,376	5,348	5,387	5,263	5,439
210097_s_at	11,107	11,267	11,110	11,941	11,674	11,854
210098_s_at	7,157	7,262	7,060	7,475	7,416	7,720
210099_at	6,957	6,454	6,659	7,478	7,172	7,216
210100_s_at	7,045	6,782	6,778	8,026	7,896	8,251
210101_x_at	9,131	9,496	9,409	10,292	10,706	10,684
210102_at	9,042	9,407	8,674	8,236	8,212	7,782
210103_s_at	6,811	6,313	6,688	7,343	6,842	6,949
210104_at	9,001	8,729	8,361	8,560	7,479	7,963
210105_s_at	9,995	10,263	10,164	10,128	10,113	9,649
210106_at	8,518	8,124	8,401	8,873	9,035	9,451
210107_at	5,826	5,778	5,593	6,089	5,797	6,123
210108_at	4,851	4,837	5,022	4,768	4,786	4,838
210109_at	7,977	7,421	7,656	7,174	7,413	7,362
210110_x_at	10,779	10,839	10,309	9,355	9,759	9,036
210111_s_at	8,958	9,034	9,118	9,583	10,817	9,778
210112_at	7,926	7,884	8,193	9,626	8,531	8,848
210113_s_at	6,754	6,382	6,551	7,293	7,160	7,163
210114_at	7,357	8,014	7,205	7,237	6,760	6,805
210115_at	9,955	10,240	9,750	8,153	7,463	7,841
210116_at	5,254	5,144	5,567	5,758	5,775	5,719
210117_at	4,942	5,628	4,968	5,133	5,397	4,601
210118_s_at	6,373	6,986	6,845	6,084	5,721	5,662
210119_at	6,147	5,689	5,407	8,410	7,532	7,161
210120_s_at	8,017	7,958	8,359	7,587	7,041	7,145
210121_at	3,733	3,926	3,918	3,848	4,173	4,386
210122_at	5,778	5,688	5,989	6,847	6,825	7,215
210123_s_at	7,698	8,119	7,789	7,576	7,844	7,740
210124_x_at	8,919	9,269	8,945	8,996	8,845	8,963
210125_s_at	11,464	11,009	11,206	10,793	10,204	10,849
210126_at	6,937	7,066	7,261	7,788	7,583	7,886
210127_at	7,542	7,425	7,745	6,889	6,416	6,912
210128_s_at	7,849	7,733	7,910	8,104	8,473	8,124
210129_s_at	6,758	6,589	7,060	7,469	7,740	7,708
210130_s_at	8,106	8,005	8,009	7,124	6,869	7,014
210131_x_at	10,699	10,537	10,600	10,321	10,063	10,545
210132_at	8,077	8,457	8,142	7,758	7,832	8,100
210133_at	5,580	5,623	5,406	6,229	5,986	6,349
210134_x_at	6,869	7,003	7,230	7,494	7,249	7,517
210135_s_at	4,634	5,115	4,982	6,844	7,568	5,081
210136_at	7,803	8,416	7,824	6,547	6,892	7,236
210137_s_at	10,629	10,680	10,916	10,913	10,407	10,961

210138_at	6,713	7,674	6,983	10,546	10,750	10,241
210139_s_at	8,750	9,336	8,713	11,615	12,343	12,275
210140_at	6,594	6,451	6,224	6,992	6,726	6,857
210141_s_at	6,260	6,311	6,434	6,336	6,480	6,544
210142_x_at	10,271	10,445	10,557	10,340	10,149	10,839
210143_at	6,488	6,369	6,351	8,695	8,791	8,994
210144_at	7,703	7,641	7,990	8,078	7,761	7,957
210145_at	6,966	8,310	6,127	10,191	10,424	8,436
210146_x_at	3,852	4,065	4,066	4,594	4,566	4,850
210147_at	5,672	4,817	6,236	3,861	3,893	3,885
210148_at	4,300	4,627	4,498	5,030	4,959	4,888
210149_s_at	10,261	10,534	10,307	10,836	10,375	10,957
210150_s_at	8,904	9,246	8,945	7,828	8,707	7,819
210151_s_at	8,867	8,604	8,698	9,112	8,847	8,853
210152_at	6,022	5,928	6,371	7,530	7,640	7,773
210153_s_at	9,822	10,058	9,543	9,201	9,043	8,924
210154_at	8,661	8,926	8,305	7,681	7,406	7,421
210155_at	7,447	7,364	7,451	7,376	7,275	7,219
210156_s_at	10,208	10,386	9,971	11,189	10,515	11,129
210157_at	8,703	8,899	8,585	7,657	7,566	7,479
210158_at	6,966	6,863	6,954	7,111	6,805	6,962
210159_s_at	6,078	5,783	6,004	7,050	6,797	6,941
210160_at	6,945	6,832	7,113	8,006	7,349	7,822
210161_at	6,990	7,149	7,261	7,742	7,722	7,588
210162_s_at	7,590	7,762	7,804	7,517	7,175	7,375
210163_at	3,699	3,809	4,106	3,946	3,874	3,927
210164_at	5,141	5,130	4,987	5,514	5,246	5,330
210165_at	6,853	6,930	6,799	6,867	6,792	7,064
210166_at	5,955	6,271	6,403	6,855	6,723	6,696
210167_s_at	5,370	4,950	5,512	5,968	5,278	5,642
210168_at	5,534	5,350	5,600	6,051	5,947	6,227
210169_at	6,893	7,449	6,994	6,939	6,586	6,481
210170_at	4,646	4,595	4,320	4,539	4,644	4,687
210171_s_at	5,477	4,983	5,208	6,134	5,767	5,501
210172_at	8,130	8,293	8,261	6,315	6,584	6,751
210173_at	5,126	5,160	5,464	5,368	5,255	5,088
210174_at	5,865	6,787	6,247	5,797	5,511	5,828
210175_at	8,216	8,264	8,329	9,616	8,453	9,012
210176_at	4,556	4,207	4,634	4,352	4,443	4,308
210177_at	7,999	8,108	8,258	8,516	8,369	8,539
210178_x_at	10,121	10,178	9,905	8,863	8,538	8,556
210179_at	4,457	5,085	4,506	5,346	5,683	5,899
210180_s_at	8,740	9,038	8,877	8,227	7,852	8,108
210181_s_at	6,399	5,941	5,691	6,560	5,790	6,131
210182_at	7,538	7,628	7,463	8,324	7,925	8,241
210183_x_at	11,249	11,123	11,203	11,633	11,462	12,078
210184_at	6,605	6,909	6,977	7,146	6,919	7,173
210185_at	8,303	8,268	8,406	9,142	9,066	9,339
210186_s_at	8,905	8,679	8,657	9,565	8,995	9,616
210187_at	8,421	8,938	8,901	9,851	8,792	8,868
210188_at	7,987	8,260	7,744	6,804	6,919	6,722
210189_at	6,885	7,117	7,240	7,133	6,611	6,993
210190_at	6,017	5,913	5,964	6,939	6,661	6,959
210191_s_at	9,525	9,280	8,966	6,714	7,011	6,301
210192_at	6,100	6,191	5,716	6,292	6,166	6,136
210193_at	5,357	5,481	5,361	5,679	5,430	5,674

210194_at	3,955	3,786	4,162	4,729	4,662	4,628
210195_s_at	5,266	5,279	5,255	5,336	5,546	5,441
210196_s_at	4,116	4,229	4,174	4,482	4,421	4,596
210197_at	6,325	6,539	6,517	7,170	6,824	7,196
210198_s_at	9,359	9,315	9,094	4,843	4,672	4,518
210199_at	7,282	7,235	6,670	7,483	7,167	7,520
210200_at	8,985	8,699	8,366	8,566	8,559	8,645
210201_x_at	9,726	9,822	9,605	10,031	10,507	10,004
210202_s_at	8,304	8,280	8,014	9,034	9,104	8,996
210203_at	6,993	6,879	6,219	4,866	4,946	4,473
210204_s_at	6,281	7,091	6,386	6,624	6,769	6,917
210205_at	6,902	7,334	6,699	7,438	7,497	7,400
210206_s_at	7,223	7,278	7,115	6,968	6,947	7,468
210208_x_at	10,494	10,503	10,963	10,054	9,883	10,232
210210_at	8,316	7,846	8,026	6,838	7,754	6,940
210211_s_at	14,138	14,036	13,695	13,001	13,643	12,641
210212_x_at	8,297	8,583	8,396	9,508	9,221	9,374
210213_s_at	9,663	9,197	9,866	10,948	10,720	11,341
210214_s_at	7,080	6,969	7,290	7,177	7,130	7,290
210215_at	6,925	7,232	6,799	6,346	6,014	6,372
210216_x_at	8,597	8,432	8,555	8,835	7,888	8,502
210218_s_at	4,617	5,136	5,014	7,105	7,634	6,950
210219_at	5,714	6,153	5,852	7,164	7,681	6,729
210220_at	10,943	10,682	10,880	8,066	7,617	8,534
210221_at	7,327	7,791	7,102	6,697	6,459	6,650
210222_s_at	7,267	7,527	6,881	6,148	6,094	6,222
210223_s_at	6,243	6,168	5,900	8,624	8,972	9,357
210224_at	6,520	6,569	6,700	7,769	8,133	8,047
210225_x_at	7,713	7,271	7,694	8,440	8,340	8,487
210226_at	8,099	8,129	7,952	8,659	8,615	8,591
210227_at	5,047	4,688	4,600	4,657	4,446	4,447
210228_at	6,808	6,446	6,711	7,520	7,210	7,123
210229_s_at	6,692	6,631	6,666	7,321	6,852	7,014
210230_at	7,668	7,368	7,386	6,715	7,601	7,090
210231_x_at	10,717	10,498	10,781	9,210	9,273	9,595
210232_at	6,614	6,962	6,635	6,914	6,740	7,094
210233_at	7,395	7,902	6,711	6,313	6,946	7,198
210234_at	7,329	7,410	7,297	7,168	7,306	7,302
210235_s_at	8,143	8,434	8,441	7,572	7,438	6,976
210236_at	7,486	7,781	8,158	7,942	7,982	7,513
210237_at	7,683	7,232	7,555	8,056	7,915	7,935
210239_at	6,745	6,666	6,325	6,754	6,817	6,942
210240_s_at	6,513	6,597	6,594	6,865	6,890	6,877
210241_s_at	6,198	6,800	6,591	7,595	7,435	7,576
210242_x_at	9,575	9,211	10,205	10,144	9,157	9,631
210243_s_at	9,907	9,954	10,224	10,073	9,502	9,985
210244_at	7,286	7,371	7,494	7,868	7,666	8,018
210245_at	6,874	6,659	6,599	7,909	7,422	7,880
210246_s_at	6,733	6,930	6,630	7,477	6,954	7,289
210247_at	8,043	8,796	8,199	6,867	6,761	6,994
210248_at	6,692	6,332	6,890	7,238	7,226	7,540
210249_s_at	8,601	8,726	8,706	8,058	8,341	7,978
210250_x_at	11,640	11,861	12,088	11,319	10,670	11,170
210251_s_at	8,137	8,444	7,826	6,987	7,587	7,305
210252_s_at	9,277	9,506	9,458	8,835	9,077	9,032
210253_at	8,150	8,778	8,168	8,907	7,942	8,423

210254_at	5,725	6,129	6,013	6,050	5,964	5,869
210255_at	5,645	6,123	5,864	5,933	5,686	5,796
210256_s_at	7,739	7,624	7,180	7,679	7,542	7,655
210257_x_at	7,753	8,612	7,360	8,555	9,356	8,526
210258_at	3,751	3,794	3,619	3,691	3,684	3,741
210259_s_at	5,623	5,753	5,580	5,735	5,630	5,686
210260_s_at	9,985	10,211	9,858	9,552	8,966	8,650
210261_at	5,737	5,979	5,907	5,760	6,223	5,837
210262_at	4,182	4,328	4,436	4,575	4,677	4,597
210263_at	7,387	7,477	7,796	8,974	9,238	9,240
210264_at	7,069	6,580	6,902	7,216	6,716	7,232
210265_x_at	10,153	10,092	10,223	6,500	6,644	6,774
210266_s_at	11,537	11,432	11,291	8,116	9,026	8,166
210267_at	5,644	5,592	6,125	6,408	5,928	6,174
210268_at	4,406	4,544	4,681	4,170	4,429	4,350
210269_s_at	7,256	7,126	7,263	7,480	7,231	7,225
210270_at	6,743	6,052	6,288	7,111	7,004	7,072
210271_at	6,621	7,038	7,104	7,238	6,923	7,009
210272_at	7,475	7,511	7,324	7,739	7,467	7,937
210273_at	6,606	6,665	6,775	6,636	6,235	6,475
210274_at	5,522	5,133	5,390	5,627	5,535	5,581
210275_s_at	13,031	13,093	12,971	10,864	11,974	11,324
210276_s_at	8,145	8,141	8,575	9,732	9,531	10,197
210277_at	8,226	7,879	8,378	7,738	7,574	7,811
210278_s_at	7,545	7,144	7,475	7,475	7,186	7,533
210279_at	4,617	4,579	4,861	4,954	4,886	4,810
210280_at	6,422	6,073	6,024	6,771	6,452	6,677
210281_s_at	8,259	8,449	7,677	5,661	7,515	6,735
210282_at	8,137	7,989	7,784	4,225	6,966	5,311
210283_x_at	9,329	9,433	8,995	9,334	9,311	9,515
210284_s_at	8,322	8,161	8,101	7,408	7,249	7,461
210285_x_at	8,684	8,432	8,327	8,580	8,063	8,114
210286_s_at	4,649	6,227	5,026	5,548	7,538	5,373
210287_s_at	5,485	5,916	5,379	5,361	5,061	5,096
210288_at	8,467	7,769	8,189	7,237	6,854	7,093
210289_at	5,856	5,639	5,721	6,540	6,647	6,798
210290_at	7,375	7,353	8,111	8,023	7,132	7,821
210291_s_at	8,173	7,893	7,938	7,996	7,917	8,051
210292_s_at	4,925	5,317	5,239	4,505	4,504	4,803
210293_s_at	10,710	10,708	10,807	11,141	11,264	11,120
210294_at	5,794	6,220	5,840	6,932	7,003	7,160
210295_at	6,147	6,334	6,363	6,668	6,191	6,156
210296_s_at	10,035	10,459	9,829	9,615	9,832	10,170
210297_s_at	4,428	4,306	4,207	4,894	4,672	4,758
210298_x_at	7,568	7,772	7,184	7,264	7,585	7,942
210299_s_at	8,495	8,516	8,362	7,241	8,196	8,581
210300_at	7,015	7,477	7,418	8,051	8,125	7,997
210301_at	7,920	7,955	7,660	8,052	7,618	7,990
210302_s_at	7,777	6,382	6,121	6,702	8,311	6,063
210303_at	5,798	5,564	5,475	5,862	6,132	5,724
210304_at	5,296	5,626	5,545	5,679	5,745	5,863
210305_at	5,284	5,219	5,355	11,169	7,100	7,532
210306_at	6,488	7,738	6,201	5,299	5,489	5,486
210307_s_at	8,542	8,058	8,701	8,507	8,614	8,566
210309_at	6,682	6,348	6,388	6,546	6,655	6,365
210310_s_at	6,743	6,754	6,849	7,820	8,087	8,261

210311_at	6,771	6,307	6,605	7,110	8,174	7,940
210312_s_at	8,944	9,337	9,004	10,453	10,103	10,712
210313_at	6,283	6,231	6,649	8,278	8,299	8,595
210314_x_at	5,903	6,076	6,297	6,739	6,227	6,726
210315_at	7,207	7,657	7,315	6,697	6,761	6,866
210316_at	7,223	7,299	7,386	7,580	7,329	7,561
210317_s_at	8,709	8,279	7,733	6,990	6,640	7,131
210318_at	7,037	6,781	7,231	7,064	7,342	7,667
210319_x_at	5,468	5,556	5,382	4,456	4,881	4,423
210320_s_at	9,833	9,367	9,538	8,442	8,751	8,159
210321_at	5,997	6,056	6,007	7,001	6,927	7,054
210322_x_at	6,015	6,154	6,224	6,443	6,693	6,569
210323_at	6,631	6,535	6,156	6,987	7,009	7,177
210324_at	6,231	6,496	6,263	6,609	6,695	6,869
210325_at	6,817	7,091	6,712	6,720	6,582	6,610
210326_at	6,978	6,876	6,877	7,344	7,339	7,486
210327_s_at	7,143	7,026	7,203	7,430	7,586	7,675
210328_at	5,953	5,840	5,538	6,362	6,122	6,236
210329_s_at	7,030	7,461	7,423	7,712	8,192	8,112
210330_at	4,911	4,875	4,838	5,569	5,743	5,747
210331_at	7,110	7,205	7,345	7,164	7,437	7,426
210332_at	7,351	6,972	7,263	7,518	7,399	7,329
210333_at	6,185	6,211	5,738	6,369	6,181	6,417
210334_x_at	8,056	8,067	8,096	8,656	7,387	8,252
210335_at	5,594	6,243	5,411	5,347	5,352	5,639
210336_x_at	8,688	8,516	8,669	9,156	9,196	9,271
210337_s_at	10,245	9,923	9,333	9,335	9,248	9,927
210338_s_at	13,361	12,895	13,176	12,608	12,775	12,907
210339_s_at	6,484	6,730	6,744	7,548	7,312	7,469
210340_s_at	5,829	5,706	5,798	5,940	5,822	6,009
210341_at	7,008	7,279	6,966	6,988	6,954	6,924
210342_s_at	5,817	5,770	6,168	6,204	5,967	6,155
210343_s_at	6,417	6,199	6,489	7,429	7,523	7,716
210344_at	6,956	6,926	6,774	7,652	7,501	7,731
210345_s_at	6,474	6,649	6,276	6,992	7,275	7,106
210346_s_at	8,591	8,933	8,267	7,687	9,877	8,002
210347_s_at	10,141	10,120	10,756	7,904	7,452	7,654
210348_at	7,109	7,249	7,568	7,436	7,273	7,568
210349_at	5,528	4,883	4,920	5,484	5,367	5,393
210350_x_at	7,217	7,646	6,877	7,112	6,903	6,922
210352_at	5,360	5,418	5,479	5,373	5,543	5,204
210353_s_at	6,158	6,177	6,336	6,475	6,261	6,255
210354_at	5,007	4,933	5,234	5,435	5,275	5,798
210355_at	4,225	4,347	4,162	4,170	4,361	4,578
210356_x_at	6,040	6,083	6,288	6,399	6,507	6,398
210357_s_at	9,304	9,004	9,204	9,850	8,876	9,257
210358_x_at	7,001	7,376	7,244	7,221	7,164	7,024
210359_at	6,748	6,703	6,490	7,667	7,444	7,531
210360_s_at	4,955	4,815	4,663	4,958	4,554	4,827
210361_s_at	7,548	7,634	6,988	5,929	6,548	6,036
210362_x_at	6,429	6,572	6,492	6,588	6,501	6,930
210363_s_at	6,244	6,139	6,490	6,927	6,568	7,281
210364_at	6,698	6,779	6,127	6,940	6,845	6,752
210365_at	5,570	5,770	5,888	6,821	7,686	7,241
210366_at	7,332	7,177	6,464	5,935	5,606	5,880
210367_s_at	7,063	7,292	7,240	9,263	7,491	7,432

210368_at	6,225	6,168	5,968	6,002	5,976	5,916
210369_at	8,136	7,846	8,350	6,886	7,060	6,963
210370_s_at	6,091	5,688	5,405	5,991	6,030	5,890
210371_s_at	12,261	12,157	11,813	8,969	8,974	9,249
210372_s_at	6,426	6,343	5,861	6,739	6,840	7,007
210373_at	7,432	7,450	7,867	7,529	7,583	7,657
210374_x_at	6,252	6,082	6,195	6,229	6,317	6,176
210375_at	3,708	3,832	3,720	3,940	3,906	4,264
210376_x_at	6,477	6,359	6,878	7,606	7,115	7,248
210377_at	4,663	5,244	4,340	4,755	4,425	4,651
210378_s_at	8,942	8,650	8,979	9,557	8,580	9,498
210379_s_at	5,944	5,680	5,693	5,617	5,801	5,586
210380_s_at	6,794	6,930	6,471	6,704	6,562	6,672
210381_s_at	10,021	10,090	11,067	9,094	8,562	8,683
210382_at	7,109	7,083	6,864	7,498	7,269	7,534
210383_at	3,831	3,576	3,617	3,675	3,557	3,649
210384_at	5,911	5,432	5,186	6,175	6,534	6,484
210385_s_at	6,691	6,823	6,688	7,151	7,524	7,825
210386_s_at	9,878	10,148	10,343	10,381	9,842	10,421
210387_at	7,159	7,410	7,677	5,135	5,866	5,489
210388_at	6,847	6,818	7,040	8,030	8,195	7,999
210389_x_at	8,571	8,953	8,037	7,354	6,991	7,167
210390_s_at	4,727	4,646	4,787	4,614	4,445	4,523
210391_at	7,362	7,431	7,619	7,327	7,026	7,221
210392_x_at	7,822	7,535	7,561	8,222	7,334	7,717
210393_at	6,123	6,248	6,054	6,407	6,465	6,687
210394_x_at	7,028	7,368	7,066	6,316	6,316	6,375
210395_x_at	7,482	7,176	7,123	7,415	7,019	7,588
210396_s_at	11,452	11,482	11,502	11,706	11,701	11,727
210397_at	6,616	6,907	6,623	7,233	7,209	6,989
210398_x_at	5,540	5,062	5,276	5,738	5,570	5,676
210399_x_at	8,339	8,145	8,260	8,869	8,384	8,509
210400_at	7,200	6,751	6,849	7,376	7,278	7,489
210401_at	7,722	7,681	7,813	8,236	8,101	8,332
210402_at	5,228	5,177	5,293	5,070	5,240	5,285
210403_s_at	5,386	5,479	5,750	6,164	6,083	6,149
210404_x_at	6,370	6,377	5,934	6,633	6,518	6,688
210405_x_at	7,799	7,605	7,740	8,827	7,731	8,225
210406_s_at	10,674	10,929	10,526	10,710	11,182	11,317
210407_at	7,133	7,441	7,216	5,856	6,364	6,020
210408_s_at	6,184	6,168	6,559	6,811	6,958	6,989
210409_at	6,602	7,005	5,998	5,224	4,872	5,074
210410_s_at	8,264	8,295	8,054	7,701	7,851	7,565
210411_s_at	5,739	6,033	6,188	6,565	6,530	6,735
210412_at	5,963	5,840	6,323	5,887	6,012	6,005
210413_x_at	5,779	5,890	5,926	6,396	5,783	5,934
210414_at	7,454	7,392	7,337	7,353	7,048	7,297
210415_s_at	7,930	7,876	7,629	8,036	7,690	7,961
210416_s_at	10,454	10,803	10,301	8,006	8,019	7,916
210417_s_at	8,205	8,045	8,247	8,415	8,381	8,524
210418_s_at	10,908	10,842	11,073	10,386	9,834	10,394
210419_at	8,038	7,850	8,072	9,064	9,066	9,161
210420_at	7,410	7,168	7,654	8,217	7,979	8,273
210421_s_at	6,513	7,055	6,773	6,938	7,001	7,065
210422_x_at	7,507	7,469	7,450	8,018	7,960	8,118
210423_s_at	6,855	6,682	6,513	7,123	7,131	7,327

210424_s_at	6,879	6,819	6,968	7,223	7,284	7,139
210425_x_at	8,885	8,875	8,242	6,367	7,557	6,479
210426_x_at	7,134	8,045	7,163	5,764	6,049	6,458
210427_x_at	12,100	12,483	12,749	14,098	14,547	14,494
210428_s_at	9,230	8,924	9,327	9,735	9,261	9,792
210429_at	7,251	6,207	8,623	5,108	5,255	5,352
210430_x_at	8,608	8,413	8,710	7,716	7,691	8,156
210431_at	7,188	7,119	7,536	8,285	8,574	8,503
210432_s_at	4,967	4,706	4,343	4,558	5,299	4,616
210433_at	6,277	6,381	6,066	6,583	6,773	6,810
210434_x_at	12,574	12,662	12,975	12,710	12,624	13,110
210435_at	4,523	5,203	4,254	4,364	4,703	4,545
210436_at	6,524	6,967	6,793	5,099	5,319	5,347
210437_at	6,736	6,871	7,001	7,230	7,017	7,044
210438_x_at	11,643	11,503	11,091	9,049	11,018	9,801
210439_at	6,295	6,030	6,280	7,366	7,028	7,290
210440_s_at	6,151	6,042	6,205	6,693	6,151	6,444
210441_at	4,938	5,471	5,062	5,704	5,204	5,704
210442_at	6,883	6,828	6,740	7,752	7,611	7,653
210443_x_at	8,248	8,355	8,195	8,447	8,090	8,551
210444_at	7,477	7,451	7,724	7,592	7,514	7,554
210445_at	6,406	6,496	6,771	7,059	7,029	7,187
210446_at	6,519	6,528	6,746	7,015	6,746	6,942
210447_at	4,475	4,460	4,540	4,151	4,103	4,037
210448_s_at	9,052	8,617	9,142	8,828	8,496	9,347
210449_x_at	6,580	6,790	6,431	6,984	6,531	6,726
210450_at	5,475	5,700	5,456	5,913	5,952	6,046
210451_at	6,483	6,251	6,293	6,537	6,461	6,659
210452_x_at	6,013	5,716	6,146	6,824	6,563	6,810
210453_x_at	12,384	12,503	12,475	12,495	12,013	12,522
210454_s_at	6,638	6,404	6,579	7,302	7,475	7,660
210455_at	4,524	5,056	4,824	5,063	4,674	4,989
210456_at	7,565	7,203	7,646	7,905	7,982	8,149
210457_x_at	7,719	7,457	7,429	7,818	7,622	7,705
210458_s_at	6,367	6,560	6,307	7,381	6,780	6,985
210459_at	6,297	6,452	6,301	7,371	7,181	7,453
210460_s_at	12,668	12,671	12,591	11,785	10,947	11,495
210461_s_at	8,203	8,565	8,398	7,844	7,580	7,766
210462_at	4,776	5,138	5,815	5,215	5,470	5,448
210463_x_at	10,201	9,938	10,356	9,657	9,044	9,826
210464_at	4,571	4,688	4,592	4,706	4,661	4,873
210465_s_at	8,747	8,490	8,155	8,326	8,237	8,213
210466_s_at	13,158	13,032	13,012	12,227	12,587	12,275
210467_x_at	5,599	5,696	5,381	5,772	5,681	5,644
210469_at	7,292	6,606	7,166	6,434	6,854	6,521
210470_x_at	10,935	10,670	10,770	10,069	9,519	9,618
210471_s_at	5,683	6,000	5,900	5,714	5,464	5,574
210472_at	5,696	5,937	5,818	6,653	6,661	6,697
210473_s_at	10,074	9,696	9,933	7,601	7,348	7,235
210474_s_at	8,948	8,706	8,650	8,190	8,275	8,144
210475_at	7,626	7,266	7,614	7,735	7,219	7,353
210476_s_at	7,198	6,744	7,222	8,083	8,248	8,121
210477_x_at	6,309	5,984	6,000	6,542	6,489	6,461
210479_s_at	6,963	7,999	6,954	5,527	5,994	6,118
210480_s_at	7,526	7,998	7,386	7,033	7,769	7,359
210481_s_at	5,523	5,616	5,882	5,907	5,650	5,733

210482_x_at	8,370	8,501	8,212	7,006	7,139	7,556
210483_at	8,171	8,338	8,040	9,039	9,099	9,137
210484_s_at	6,948	6,718	6,694	6,736	7,208	6,765
210486_at	7,211	7,114	7,221	7,593	7,393	7,338
210487_at	6,738	6,493	6,669	7,085	6,672	6,846
210491_at	5,432	5,187	5,112	6,073	6,398	6,741
210492_at	5,053	5,177	5,059	6,183	5,506	5,860
210493_s_at	5,819	6,020	5,947	5,792	5,485	5,883
210495_x_at	10,142	10,293	10,757	13,495	14,579	14,445
210496_at	5,512	5,963	5,741	6,038	6,120	5,902
210497_x_at	6,625	7,006	6,724	7,063	6,917	7,001
210498_at	3,758	3,785	3,804	3,598	3,825	3,737
210499_s_at	8,178	7,937	8,060	8,586	8,299	8,591
210500_at	4,921	4,693	4,898	4,770	4,604	4,631
210501_x_at	10,380	10,541	10,721	11,002	10,352	11,091
210502_s_at	9,647	9,543	9,254	9,106	8,279	8,924
210503_at	5,109	5,109	5,253	5,325	5,441	5,342
210504_at	6,748	6,806	6,603	7,212	6,744	6,901
210505_at	6,489	6,492	6,038	7,175	6,959	7,072
210506_at	5,610	5,660	5,849	6,128	5,764	5,807
210507_s_at	6,332	6,543	6,691	7,369	7,091	7,121
210508_s_at	9,421	9,398	8,733	6,453	6,419	6,326
210510_s_at	7,458	7,439	7,505	9,805	9,791	9,907
210511_s_at	6,021	6,054	5,897	8,290	9,273	8,790
210512_s_at	9,069	9,497	9,337	10,981	10,767	10,330
210513_s_at	6,802	6,635	7,019	8,289	7,712	8,221
210514_x_at	8,354	8,577	8,515	8,977	8,886	9,149
210515_at	8,517	8,375	8,483	8,694	8,736	8,854
210516_at	4,702	4,381	4,323	4,634	4,934	4,763
210517_s_at	11,331	11,145	10,976	12,272	13,319	12,304
210518_at	6,542	6,726	6,492	7,402	7,187	7,362
210519_s_at	9,622	9,460	9,929	13,115	13,206	13,720
210520_at	6,299	6,645	6,598	7,113	7,144	7,176
210521_s_at	4,130	4,560	4,234	5,462	5,133	5,216
210523_at	5,313	4,761	5,131	5,534	5,051	5,316
210524_x_at	10,523	10,204	11,176	10,868	10,003	10,486
210525_x_at	3,742	3,802	3,693	3,901	3,741	3,885
210527_x_at	9,845	9,771	9,615	9,830	9,628	9,976
210528_at	4,068	3,892	4,143	3,966	4,083	4,061
210529_s_at	8,165	7,826	7,969	8,330	8,030	8,327
210530_s_at	7,888	8,024	7,845	7,202	6,739	6,760
210531_at	5,560	5,320	5,140	5,069	4,831	4,876
210532_s_at	11,574	11,868	11,455	11,935	11,737	12,146
210533_at	4,513	4,850	4,379	4,954	4,795	5,145
210534_s_at	7,375	7,571	7,120	7,546	7,093	7,746
210535_at	6,597	6,669	6,916	7,510	7,459	7,759
210536_s_at	6,029	5,837	6,042	6,758	6,503	6,390
210537_s_at	7,378	7,608	7,643	6,906	6,285	6,833
210538_s_at	5,074	5,761	5,088	6,550	6,969	6,153
210539_at	7,232	6,821	6,720	7,189	7,315	7,214
210540_s_at	8,577	8,985	8,043	7,292	7,382	7,191
210541_s_at	7,969	7,835	7,585	8,016	7,088	7,913
210542_s_at	7,882	7,807	7,942	8,167	8,276	8,112
210543_s_at	10,179	9,923	9,511	7,464	7,812	7,399
210544_s_at	8,890	8,753	8,461	8,117	8,166	7,852
210545_at	6,198	6,262	6,271	7,233	7,103	7,088

210546_x_at	6,694	6,487	6,612	7,052	6,863	7,295
210547_x_at	7,774	7,937	7,820	6,942	6,977	6,619
210548_at	5,516	5,309	5,515	6,001	5,825	5,782
210549_s_at	3,834	4,115	3,814	4,751	4,429	4,741
210550_s_at	5,918	5,986	5,600	6,264	5,931	6,066
210551_s_at	6,720	6,744	6,617	7,104	6,701	7,052
210552_s_at	6,065	6,718	6,159	6,619	6,759	6,731
210553_x_at	6,639	6,505	6,737	6,368	6,457	6,490
210554_s_at	10,857	10,984	10,960	10,817	10,289	10,744
210555_s_at	9,264	8,985	9,075	6,725	6,940	7,060
210556_at	6,562	5,950	6,249	4,707	4,916	4,680
210557_x_at	6,744	6,917	6,907	7,541	7,364	7,645
210558_at	5,776	5,449	5,196	7,433	7,351	7,767
210559_s_at	11,122	11,541	10,426	9,454	9,689	9,171
210560_at	6,076	6,392	6,427	7,230	7,148	7,349
210561_s_at	10,306	10,618	9,737	9,014	10,338	9,523
210562_at	5,052	4,976	5,087	5,274	5,299	5,517
210563_x_at	8,876	8,783	8,517	9,091	9,290	10,218
210564_x_at	8,018	7,872	7,695	8,318	8,355	9,215
210565_at	7,669	7,268	7,481	8,141	8,099	8,240
210567_s_at	10,515	10,944	10,264	8,201	6,584	7,289
210568_s_at	9,663	9,897	9,119	8,936	9,803	8,423
210569_s_at	6,505	6,502	6,623	6,840	6,725	6,945
210570_x_at	7,783	8,034	7,600	7,727	7,794	7,500
210571_s_at	5,072	5,778	5,449	5,920	5,540	6,046
210572_at	4,296	4,208	4,214	4,891	4,898	4,829
210573_s_at	8,393	8,200	8,581	8,185	7,694	8,050
210574_s_at	12,689	12,025	12,516	11,209	10,871	11,106
210575_at	6,497	5,934	6,081	6,323	5,986	6,149
210576_at	5,161	4,678	4,980	5,963	6,114	6,349
210577_at	7,438	7,376	7,069	7,619	7,623	7,712
210578_at	5,632	5,509	5,924	6,418	6,060	6,387
210579_s_at	5,915	6,073	5,872	6,011	6,204	6,027
210580_x_at	9,511	9,289	9,419	7,795	8,229	8,201
210581_x_at	6,882	6,335	6,688	6,274	6,256	6,374
210582_s_at	8,581	8,302	8,599	7,728	7,324	7,773
210583_at	6,732	7,036	6,629	7,034	6,753	6,846
210584_s_at	6,188	6,052	6,062	6,156	6,200	6,231
210585_s_at	5,138	5,044	5,157	5,375	5,148	5,740
210586_x_at	8,594	8,352	8,811	7,886	7,694	8,200
210587_at	7,157	8,702	8,366	6,234	6,554	6,600
210588_x_at	11,050	10,871	10,294	9,471	9,821	9,122
210589_s_at	8,467	8,094	8,628	9,876	9,472	10,060
210592_s_at	10,137	11,022	10,458	12,477	12,099	12,243
210593_at	5,932	6,134	6,081	6,651	6,524	6,745
210594_x_at	8,957	8,287	8,403	9,628	9,190	9,648
210595_at	5,198	5,219	5,160	6,131	5,606	5,764
210596_at	7,947	7,970	8,203	7,453	8,011	7,612
210597_x_at	8,204	7,377	7,733	8,806	8,616	8,678
210598_at	7,031	6,712	6,716	6,706	6,847	6,685
210599_at	5,855	6,033	5,738	5,950	5,764	5,905
210600_s_at	3,803	3,907	3,819	4,866	4,174	4,530
210601_at	7,988	8,091	7,848	7,764	7,570	7,591
210602_s_at	6,099	6,262	6,047	6,741	6,685	6,786
210603_at	5,466	5,654	5,768	5,589	5,367	5,642
210604_at	5,589	5,571	5,749	6,211	6,205	5,924

210605_s_at	9,234	9,507	9,170	8,182	8,317	8,239
210606_x_at	6,464	6,450	6,759	7,259	7,035	6,963
210607_at	5,544	5,328	6,085	6,891	7,049	7,250
210608_s_at	7,286	6,965	7,300	7,196	7,280	7,221
210609_s_at	10,372	9,736	10,609	10,451	10,111	10,904
210610_at	6,919	7,182	7,111	7,344	7,345	7,259
210611_s_at	5,014	5,295	5,041	4,502	4,238	4,445
210612_s_at	5,695	5,994	6,393	7,602	7,759	6,999
210613_s_at	8,135	8,156	8,348	8,213	8,292	8,373
210614_at	4,395	4,322	4,509	4,819	4,861	4,878
210615_at	6,335	6,501	6,370	6,719	6,552	6,630
210616_s_at	9,296	9,244	9,458	9,837	10,572	10,585
210617_at	6,003	6,119	6,387	6,722	6,269	6,811
210618_at	7,293	7,082	6,994	6,881	7,017	6,642
210619_s_at	7,544	7,448	7,184	7,422	7,602	7,570
210620_s_at	7,761	7,658	7,847	7,527	7,153	7,326
210621_s_at	9,332	9,597	9,002	8,422	10,079	8,331
210622_x_at	8,386	7,880	7,654	7,308	6,700	7,370
210623_at	6,600	6,537	6,764	6,883	7,080	7,049
210624_s_at	9,983	10,000	10,133	10,231	9,653	10,459
210625_s_at	7,747	7,641	8,024	7,029	6,604	6,703
210626_at	6,222	6,045	6,236	6,858	6,504	6,536
210627_s_at	9,083	8,966	8,938	8,813	8,274	9,199
210628_x_at	7,886	7,706	7,110	8,017	8,099	8,448
210629_x_at	6,156	5,988	6,276	6,701	6,632	6,876
210630_s_at	8,304	8,286	7,808	5,854	5,506	5,614
210631_at	5,316	5,459	5,343	6,037	5,847	5,978
210632_s_at	5,920	5,787	5,492	7,088	7,069	7,090
210633_x_at	11,178	11,175	11,510	12,138	12,406	12,571
210634_at	5,497	6,192	6,227	5,922	5,924	6,012
210635_s_at	7,174	7,148	6,931	6,739	6,789	6,827
210636_at	5,490	5,902	6,153	6,957	6,660	7,071
210637_at	6,565	6,577	6,705	7,097	7,071	7,226
210638_s_at	10,546	10,332	10,910	10,714	10,951	10,652
210639_s_at	8,360	8,267	7,784	7,208	7,176	7,393
210640_s_at	7,427	7,355	7,363	8,231	8,422	8,183
210641_at	5,570	5,763	5,794	5,786	5,821	5,817
210642_at	6,723	6,541	7,381	7,397	7,370	7,440
210643_at	6,353	7,535	6,909	4,076	4,196	3,940
210644_s_at	6,797	7,084	6,979	7,182	7,113	7,339
210645_s_at	11,377	11,466	10,648	9,307	11,592	9,917
210646_x_at	14,364	14,321	14,233	13,546	13,455	13,871
210647_x_at	8,258	8,326	8,278	8,355	8,274	8,452
210648_x_at	11,874	12,238	12,385	12,990	13,269	13,490
210649_s_at	9,256	8,655	9,298	6,416	6,212	6,497
210650_s_at	4,529	4,799	4,353	4,347	4,712	4,646
210651_s_at	7,554	7,763	7,825	8,320	8,132	8,146
210652_s_at	8,772	9,300	9,035	7,639	7,375	7,692
210653_s_at	8,991	9,349	8,508	7,362	7,217	7,972
210654_at	6,164	5,646	6,209	7,126	6,865	7,084
210655_s_at	7,457	7,392	7,429	7,111	6,984	7,210
210656_at	5,417	6,200	6,249	4,310	5,379	4,783
210657_s_at	5,723	5,351	5,581	6,021	5,990	5,802
210658_s_at	9,275	8,931	9,102	7,449	7,198	7,679
210659_at	5,973	6,189	6,542	7,176	7,212	7,559
210660_at	5,161	5,228	5,276	5,749	5,668	5,698

210661_at	3,685	3,799	3,911	3,826	3,798	3,838
210662_at	4,498	4,461	4,245	6,800	6,106	5,675
210663_s_at	6,191	6,545	5,974	8,614	8,002	7,095
210664_s_at	7,782	8,269	6,744	11,080	12,227	11,398
210665_at	6,257	7,071	5,996	9,524	11,059	9,805
210666_at	7,361	7,365	7,900	8,022	8,266	8,397
210667_s_at	7,902	7,929	7,903	7,567	6,837	7,435
210669_at	5,409	5,592	5,526	5,398	5,433	5,342
210670_at	5,803	5,423	5,842	5,996	5,679	5,657
210671_x_at	5,973	6,168	5,953	6,617	6,364	6,401
210672_s_at	8,997	9,116	9,456	8,962	8,557	9,005
210673_x_at	7,236	6,705	7,204	8,120	7,787	8,141
210674_s_at	7,992	8,236	8,320	4,263	4,059	4,178
210675_s_at	4,773	4,767	4,586	4,260	4,175	4,212
210676_x_at	8,957	9,686	8,672	7,207	7,759	6,525
210677_at	7,042	7,097	6,825	7,935	7,596	7,948
210678_s_at	8,004	7,709	8,149	8,427	8,180	8,659
210679_x_at	9,012	8,740	8,761	7,770	8,734	7,915
210680_s_at	7,190	7,007	7,222	7,504	7,352	7,560
210681_s_at	9,581	9,493	9,192	8,586	10,003	8,155
210682_at	6,299	6,161	6,046	7,166	6,687	6,994
210683_at	6,485	7,013	6,161	4,752	4,784	4,828
210684_s_at	6,386	6,661	6,739	7,053	6,859	6,745
210685_s_at	7,991	8,001	8,246	7,252	7,133	7,313
210686_x_at	9,033	9,029	9,174	8,323	8,996	8,785
210687_at	5,205	5,317	5,174	6,725	6,902	6,998
210688_s_at	6,621	6,622	6,380	8,219	8,006	8,162
210689_at	6,473	6,770	6,691	6,573	6,699	6,685
210690_at	3,753	3,388	3,574	4,031	3,805	3,919
210691_s_at	11,004	10,894	10,688	10,507	9,675	9,950
210692_s_at	7,078	6,217	6,688	8,406	6,548	7,035
210693_at	7,315	7,352	7,273	7,271	7,137	7,499
210694_s_at	8,205	8,471	8,068	6,793	7,097	6,605
210695_s_at	8,730	9,027	8,874	8,801	8,406	8,746
210697_at	8,450	8,439	7,974	4,781	5,086	4,802
210698_at	4,333	4,629	4,801	4,581	4,665	4,742
210699_at	4,489	4,660	4,780	4,788	4,552	4,384
210701_at	8,296	7,463	8,624	6,377	6,692	6,672
210702_s_at	5,552	5,855	6,278	6,310	5,720	5,974
210703_at	3,780	3,709	3,728	3,631	3,752	3,790
210704_at	5,541	5,815	5,888	6,175	6,191	6,090
210705_s_at	8,075	8,107	8,065	8,339	8,925	9,284
210706_s_at	9,152	8,913	9,247	9,276	8,997	9,413
210707_x_at	8,333	8,070	8,113	7,922	7,474	8,048
210708_x_at	6,970	6,850	7,191	7,147	6,570	6,933
210709_at	4,226	4,051	4,130	4,190	4,350	4,135
210710_at	5,664	5,492	5,733	6,919	6,914	6,827
210711_at	6,293	6,169	6,196	6,088	6,240	6,047
210712_at	4,773	4,337	4,158	4,853	4,368	4,333
210713_at	6,875	6,827	6,921	6,271	6,057	5,912
210714_at	6,273	6,375	6,598	6,153	6,048	6,113
210715_s_at	10,018	10,173	9,865	7,238	7,469	7,198
210716_s_at	6,745	6,979	5,892	5,307	6,349	5,267
210717_at	5,990	5,339	5,600	5,012	5,404	4,983
210718_s_at	5,920	5,899	5,659	5,238	5,324	5,202
210719_s_at	9,053	9,104	8,638	8,432	7,857	8,295

210720_s_at	8,141	8,477	7,937	7,763	7,635	8,022
210721_s_at	4,479	4,576	4,505	3,908	3,748	3,705
210722_at	5,025	4,934	5,062	5,426	5,533	5,440
210723_x_at	7,121	6,950	6,982	7,469	7,409	7,409
210724_at	5,256	5,460	5,272	6,402	6,220	6,311
210726_at	7,253	7,186	7,159	7,685	7,457	7,299
210727_at	5,651	5,842	5,531	6,210	6,205	6,726
210728_s_at	6,777	6,726	6,796	7,728	7,793	8,060
210729_at	4,114	4,253	4,400	3,906	4,011	4,082
210730_s_at	5,961	5,912	6,047	6,523	6,680	6,743
210731_s_at	7,066	7,252	7,258	6,927	7,099	6,832
210732_s_at	5,276	5,738	5,140	7,005	6,583	6,829
210733_at	7,888	8,149	7,994	8,651	9,729	9,761
210734_x_at	6,938	6,832	6,353	7,663	7,529	7,824
210735_s_at	7,115	6,636	6,772	8,214	8,054	8,698
210736_x_at	7,457	7,533	7,345	7,880	7,957	7,956
210737_at	7,028	6,681	6,769	8,001	7,873	8,199
210738_s_at	3,582	3,623	3,688	3,767	3,752	3,740
210739_x_at	6,730	6,560	6,696	6,917	6,643	6,883
210740_s_at	9,882	9,824	10,498	9,804	9,563	9,876
210741_at	4,157	4,597	4,476	4,609	4,724	4,911
210742_at	6,875	6,704	6,800	5,719	5,526	5,546
210743_s_at	5,811	5,311	5,672	4,891	4,618	4,738
210744_s_at	7,680	7,738	7,567	8,133	7,694	7,744
210745_at	5,996	6,013	6,078	6,507	6,380	6,643
210746_s_at	5,830	5,879	6,024	6,281	6,503	6,669
210747_at	4,821	4,693	4,461	5,166	5,015	5,182
210748_at	5,717	5,675	5,777	5,932	5,747	5,957
210749_x_at	8,934	8,600	9,090	8,223	7,930	8,449
210750_s_at	7,301	6,950	6,961	7,574	7,137	7,286
210751_s_at	7,576	6,760	7,204	8,762	7,778	8,403
210752_s_at	6,464	6,574	7,040	7,930	7,749	7,982
210753_s_at	7,802	7,586	8,138	7,662	7,412	7,905
210754_s_at	9,806	9,700	9,585	7,524	7,405	7,425
210755_at	4,883	4,376	5,259	6,806	7,376	5,865
210756_s_at	7,659	7,500	7,656	8,026	7,567	8,055
210757_x_at	7,240	7,952	7,233	9,488	10,052	9,845
210758_at	10,842	10,864	10,404	7,591	7,465	7,117
210759_s_at	12,221	12,248	12,137	11,644	11,651	11,932
210760_x_at	7,211	7,047	7,050	7,240	7,289	7,085
210761_s_at	8,200	8,157	8,345	8,361	7,907	8,327
210762_s_at	7,585	8,002	7,274	10,576	11,726	11,325
210763_x_at	6,225	6,500	6,451	6,620	6,809	6,733
210764_s_at	8,314	9,530	9,084	9,884	9,210	10,238
210765_at	7,981	8,390	8,394	6,966	7,726	6,867
210766_s_at	12,372	12,379	11,891	10,770	11,413	9,750
210767_at	5,010	4,881	5,004	7,023	6,832	7,206
210768_x_at	9,019	9,168	9,135	8,722	9,431	9,714
210769_at	7,250	7,304	7,591	8,449	8,720	8,743
210770_s_at	6,336	6,354	6,371	7,358	7,129	7,323
210771_at	5,684	5,545	5,688	6,576	6,216	6,572
210772_at	5,723	5,846	5,832	5,887	5,587	5,906
210773_s_at	6,574	6,977	6,720	6,683	6,434	6,577
210774_s_at	12,187	12,469	12,413	12,422	13,090	12,850
210775_x_at	8,206	8,073	7,751	8,064	8,011	8,385
210776_x_at	10,525	10,534	10,918	9,871	9,885	10,272

210778_s_at	7,900	7,923	7,910	8,756	9,123	9,714
210779_x_at	8,707	8,551	7,998	7,232	7,070	7,138
210780_at	6,753	6,598	7,088	7,476	7,021	7,084
210781_x_at	7,246	7,084	7,200	7,214	6,850	7,192
210782_x_at	7,383	7,352	7,750	8,110	8,142	8,461
210783_x_at	7,832	7,679	7,462	7,359	7,309	8,264
210784_x_at	7,973	7,604	7,867	8,878	8,459	8,835
210785_s_at	7,751	7,611	7,384	6,987	6,649	6,779
210786_s_at	5,362	5,354	5,458	5,923	5,683	5,715
210787_s_at	5,928	5,705	5,932	6,357	5,777	6,372
210788_s_at	8,593	8,837	8,792	9,274	8,972	9,592
210789_x_at	7,317	7,128	7,215	7,761	7,629	7,719
210790_s_at	7,541	7,595	7,609	9,087	8,241	8,876
210791_s_at	7,836	7,734	7,499	7,163	7,281	7,133
210792_x_at	9,303	9,116	9,586	10,086	9,040	9,627
210793_s_at	9,930	9,519	10,153	9,356	9,169	8,807
210794_s_at	7,141	7,032	6,771	7,971	8,892	8,887
210795_s_at	7,791	7,863	7,820	8,185	8,149	8,189
210796_x_at	5,854	6,389	6,210	6,894	6,805	6,708
210797_s_at	6,995	7,082	7,330	7,652	7,509	7,949
210798_x_at	6,949	6,888	6,380	7,452	6,960	7,101
210799_at	6,306	6,242	6,512	6,287	6,388	6,383
210800_at	3,964	4,041	4,049	3,854	4,158	3,954
210801_at	4,572	4,614	4,623	5,065	4,530	5,030
210802_s_at	8,528	8,371	8,394	8,731	8,103	8,662
210803_at	7,217	7,358	7,278	7,822	7,579	7,713
210804_x_at	6,838	6,979	7,093	8,230	7,806	8,194
210805_x_at	5,670	5,320	5,524	6,145	5,902	5,932
210806_at	5,771	6,142	5,787	6,678	7,068	6,954
210807_s_at	5,634	6,186	5,978	7,029	7,840	6,938
210808_s_at	6,944	7,151	7,017	7,550	7,659	7,841
210809_s_at	7,625	7,705	7,943	7,278	6,977	9,673
210810_s_at	6,418	6,681	6,484	6,412	6,446	6,422
210811_s_at	7,913	7,221	8,077	8,080	8,112	8,384
210812_at	6,177	6,506	6,492	6,776	6,779	6,881
210813_s_at	7,261	8,019	6,797	6,788	6,688	6,493
210814_at	4,861	4,532	4,697	4,615	4,413	4,751
210815_s_at	5,047	4,998	5,150	5,219	5,355	5,263
210816_s_at	6,801	7,005	6,767	6,986	7,015	7,064
210817_s_at	8,864	9,336	8,189	9,269	9,705	9,496
210818_s_at	5,308	6,174	5,714	6,234	6,486	6,186
210819_x_at	7,499	7,423	7,485	7,488	7,311	7,538
210820_x_at	8,508	8,803	8,683	8,092	7,441	7,876
210821_x_at	8,092	8,214	7,920	7,719	6,858	7,436
210822_at	7,767	7,840	7,882	7,651	7,291	7,766
210823_s_at	6,439	6,318	6,636	6,740	6,593	7,009
210824_at	6,257	6,214	6,054	6,518	6,211	6,494
210825_s_at	12,571	12,847	13,053	11,756	11,498	11,608
210826_x_at	9,311	9,794	8,902	7,771	8,233	7,693
210827_s_at	7,597	7,791	7,811	7,665	7,743	7,753
210828_s_at	7,481	7,179	6,992	7,457	7,494	7,582
210829_s_at	9,042	9,314	8,841	7,996	8,360	8,579
210830_s_at	9,233	9,259	8,764	8,759	8,951	9,498
210831_s_at	6,014	6,162	6,132	6,186	6,150	6,243
210832_x_at	5,896	5,915	5,925	6,424	6,018	6,221
210833_at	4,205	4,306	4,476	4,312	4,395	4,412

210834_s_at	5,455	5,479	5,925	5,710	5,590	5,671
210835_s_at	11,901	12,070	12,089	11,066	11,138	11,066
210836_x_at	5,030	5,408	5,678	5,532	5,615	5,584
210837_s_at	4,690	4,766	4,858	5,053	5,342	5,074
210838_s_at	6,435	6,566	6,864	7,465	7,427	7,740
210839_s_at	7,716	8,104	7,096	8,589	9,049	8,925
210840_s_at	9,595	9,686	9,168	9,487	10,253	9,702
210841_s_at	7,623	7,518	7,624	7,927	7,581	7,839
210842_at	6,035	6,871	6,806	6,980	6,060	6,467
210843_s_at	5,501	5,316	5,476	5,357	5,282	5,407
210844_x_at	11,833	11,997	11,834	11,304	12,006	11,737
210845_s_at	8,335	8,201	8,384	10,360	9,505	9,984
210846_x_at	6,559	6,277	6,295	6,324	5,814	5,884
210847_x_at	7,201	6,538	6,753	6,470	6,409	6,318
210848_at	6,620	6,343	6,535	6,852	6,953	7,098
210849_s_at	9,844	9,949	9,681	8,476	9,543	8,172
210850_s_at	6,885	6,593	6,645	6,632	6,019	6,346
210852_s_at	8,890	8,788	8,472	6,133	6,073	6,095
210853_at	4,842	5,039	4,856	4,817	4,741	4,794
210854_x_at	8,639	7,904	9,196	7,593	7,378	7,887
210855_at	7,530	7,176	7,303	7,906	7,660	7,730
210858_x_at	7,850	7,602	7,612	7,221	7,238	7,091
210859_x_at	8,493	8,264	8,522	8,089	7,425	8,041
210861_s_at	4,347	4,521	4,821	6,717	5,678	6,834
210862_s_at	5,638	5,346	5,190	5,599	5,355	5,699
210864_x_at	7,339	7,316	7,335	8,002	7,864	8,092
210865_at	6,193	6,131	6,137	6,436	6,199	5,991
210866_s_at	7,064	6,468	6,497	6,122	5,981	6,006
210867_at	6,948	7,286	6,385	6,511	6,628	6,330
210868_s_at	8,949	8,752	8,487	8,722	9,331	8,502
210869_s_at	8,112	8,122	8,084	9,153	6,969	8,946
210870_s_at	6,961	7,115	6,852	6,582	6,540	6,677
210871_x_at	8,560	8,956	8,168	6,331	6,559	6,239
210872_x_at	5,953	5,985	6,358	6,458	6,202	5,902
210873_x_at	4,331	3,896	4,186	4,675	4,561	4,892
210874_s_at	7,520	7,889	7,501	8,027	7,787	8,210
210875_s_at	5,073	5,145	4,714	5,167	5,735	5,178
210876_at	6,551	6,773	7,182	9,092	8,916	8,928
210878_s_at	9,874	9,583	9,853	7,656	7,802	7,482
210879_s_at	8,780	8,729	8,540	9,196	9,152	9,813
210880_s_at	8,142	7,942	7,907	7,363	6,997	7,207
210881_s_at	7,233	7,394	7,434	7,793	7,576	7,754
210882_s_at	8,787	8,936	8,809	6,455	6,381	6,622
210883_x_at	6,893	6,743	6,653	7,679	7,329	7,457
210884_s_at	7,158	7,338	7,360	8,412	7,984	8,108
210885_s_at	7,079	7,225	6,731	7,296	7,356	7,796
210886_x_at	7,168	6,813	7,032	7,315	7,208	7,605
210887_s_at	7,563	7,442	7,639	8,320	8,096	8,308
210888_s_at	6,041	6,349	6,601	6,730	6,434	6,586
210889_s_at	5,677	5,794	6,051	6,505	6,731	6,100
210890_x_at	7,054	7,147	7,205	7,741	7,733	8,023
210891_s_at	13,057	13,005	12,952	10,257	11,208	10,515
210892_s_at	6,210	6,515	6,184	6,399	5,686	6,242
210893_at	4,044	3,689	3,769	3,840	3,629	3,760
210894_s_at	7,122	7,009	7,110	7,658	7,394	7,956
210895_s_at	4,625	4,825	4,652	4,384	3,994	4,560

210896_s_at	9,166	9,257	8,486	10,594	12,165	11,390
210904_s_at	6,220	6,388	6,116	6,699	6,281	6,369
210905_x_at	10,723	10,570	10,728	6,973	7,152	7,439
210906_x_at	5,143	5,075	5,268	5,428	5,233	5,121
210907_s_at	11,726	12,037	11,410	11,142	11,789	11,599
210908_s_at	10,944	10,960	10,815	10,629	10,047	10,990
210909_x_at	5,860	5,741	5,975	6,473	6,536	6,585
210910_s_at	7,916	8,321	8,193	7,230	6,699	7,639
210911_at	6,987	7,232	6,964	7,286	7,822	7,584
210912_x_at	7,318	7,828	7,377	7,142	7,124	7,162
210913_at	6,053	5,785	5,821	6,251	6,041	6,163
210914_at	4,769	4,753	4,670	5,117	4,899	5,097
210915_x_at	7,366	7,288	7,085	7,440	7,217	7,350
210916_s_at	6,843	6,798	6,792	8,683	7,711	8,059
210917_at	4,682	4,628	4,687	4,840	5,119	4,706
210918_at	5,776	5,961	5,607	5,956	5,864	5,875
210919_at	3,657	3,419	3,483	3,647	3,883	3,744
210920_x_at	6,258	6,174	6,042	6,284	7,056	6,510
210921_at	6,154	5,789	6,154	6,274	5,880	6,050
210922_at	6,853	6,792	7,118	7,442	7,010	6,882
210923_at	6,091	6,389	6,457	7,161	6,925	6,874
210924_at	8,057	7,814	8,018	8,262	8,023	8,139
210925_at	6,704	6,950	6,970	6,775	6,809	7,197
210926_at	8,089	7,735	7,865	7,783	7,236	8,212
210927_x_at	12,314	12,342	12,666	12,476	12,483	12,762
210928_at	3,951	4,108	4,505	4,261	4,497	4,439
210929_s_at	6,585	6,241	6,948	7,008	6,504	6,842
210930_s_at	6,073	5,707	6,003	6,572	6,498	6,950
210931_at	4,177	4,116	4,403	4,715	4,667	4,801
210932_s_at	4,402	4,405	4,613	5,366	5,065	5,742
210933_s_at	8,572	8,214	8,673	8,284	8,309	8,860
210934_at	6,466	6,557	6,791	6,831	6,881	6,941
210935_s_at	7,887	7,174	7,754	7,810	7,434	8,206
210936_at	6,086	5,907	6,316	6,732	7,180	7,246
210937_s_at	6,508	6,008	6,992	7,839	7,783	8,037
210938_at	6,858	6,524	6,725	6,748	6,716	6,705
210939_s_at	6,406	6,601	6,239	7,317	7,500	7,675
210940_s_at	7,027	6,675	6,825	7,475	7,379	7,596
210941_at	4,094	3,828	4,313	4,049	4,109	4,113
210942_s_at	7,172	8,184	7,183	5,022	4,795	4,815
210943_s_at	6,674	6,882	6,694	7,830	8,061	7,707
210944_s_at	6,914	7,020	6,974	6,946	7,055	6,739
210945_at	6,448	6,560	6,440	5,753	5,322	5,490
210946_at	10,003	10,789	10,704	10,618	11,056	11,356
210947_s_at	8,251	8,733	7,845	7,938	7,582	7,488
210948_s_at	5,587	5,462	5,659	6,305	5,875	5,997
210949_s_at	13,122	12,836	13,254	12,368	12,505	12,403
210950_s_at	12,466	12,405	11,840	11,219	10,262	11,311
210951_x_at	8,231	8,135	7,970	10,404	10,179	9,924
210952_at	7,279	7,202	7,371	7,322	7,377	7,351
210953_at	4,366	4,409	4,297	4,416	4,370	4,339
210954_s_at	4,728	5,088	5,504	4,957	5,062	4,987
210955_at	7,334	6,849	7,163	8,100	7,932	7,749
210956_at	6,356	6,333	6,491	6,743	6,431	6,637
210957_s_at	6,257	5,704	5,819	6,149	6,490	6,091
210958_s_at	7,938	7,671	7,489	8,168	7,839	8,187

210959_s_at	7,445	7,374	7,499	7,942	7,365	8,151
210960_at	6,181	6,061	6,191	6,712	6,389	6,915
210961_s_at	5,194	4,707	5,098	5,980	5,598	6,012
210962_s_at	9,894	10,040	9,171	8,283	9,943	8,099
210963_s_at	8,225	7,859	8,111	6,811	6,369	6,685
210964_s_at	10,030	9,543	9,896	6,451	6,744	6,684
210965_x_at	5,531	4,694	4,943	4,513	4,886	4,675
210966_x_at	10,292	9,868	10,046	9,471	9,706	9,602
210967_x_at	7,541	7,113	7,583	8,364	7,986	8,490
210968_s_at	12,806	12,636	12,448	13,240	13,614	13,722
210969_at	3,950	3,741	4,085	3,859	3,907	3,914
210970_s_at	9,895	10,186	9,581	9,975	10,886	9,457
210971_s_at	5,899	6,624	5,885	6,541	6,062	6,313
210972_x_at	7,265	6,883	7,035	8,245	7,659	8,093
210973_s_at	9,568	9,173	9,386	8,692	8,755	9,000
210974_s_at	8,382	8,208	7,951	7,908	7,500	8,252
210975_x_at	9,109	9,028	9,196	9,627	9,134	9,661
210976_s_at	11,198	11,661	11,205	11,037	10,583	10,458
210977_s_at	8,258	8,165	8,371	8,482	8,342	8,423
210978_s_at	8,641	8,288	8,563	8,893	8,437	8,945
210979_at	4,301	4,537	4,850	4,979	4,791	5,071
210980_s_at	9,486	10,086	9,390	9,961	10,683	9,832
210981_s_at	8,661	8,516	8,739	8,797	8,464	9,001
210982_s_at	6,097	5,923	6,406	6,097	6,079	6,275
210983_s_at	11,683	10,818	10,917	9,947	8,754	9,135
210984_x_at	7,194	7,147	7,383	8,005	7,261	7,890
210985_s_at	5,657	5,654	5,257	6,438	6,660	6,156
210986_s_at	12,723	12,384	13,206	12,339	12,479	12,947
210987_x_at	11,669	11,480	11,880	10,917	10,876	11,761
210988_s_at	7,607	7,581	7,843	6,570	6,343	6,255
210989_at	4,671	4,545	4,850	5,965	5,784	6,238
210990_s_at	6,393	6,106	6,360	7,211	7,108	7,806
210991_s_at	7,005	6,738	6,959	7,920	7,357	7,599
210992_x_at	7,190	7,065	6,882	8,138	8,045	8,474
210993_s_at	9,506	9,118	9,220	7,817	7,725	7,916
210994_x_at	6,774	6,730	6,485	6,750	7,051	7,269
210995_s_at	6,693	6,389	6,171	6,722	6,570	6,450
210996_s_at	10,674	10,367	10,505	9,935	10,345	10,292
210997_at	4,055	3,860	4,366	10,697	10,069	9,181
210998_s_at	6,277	6,536	6,610	9,070	8,681	8,050
210999_s_at	9,084	9,479	9,419	9,066	8,600	8,660
211000_s_at	5,865	6,514	6,011	7,928	8,612	7,851
211001_at	7,297	7,135	7,502	8,532	8,574	8,651
211002_s_at	6,664	6,704	6,764	7,701	7,338	7,425
211003_x_at	6,862	7,102	6,921	7,749	7,296	7,568
211004_s_at	7,776	7,753	8,062	8,800	9,072	9,205
211005_at	7,770	7,654	7,887	8,036	7,870	8,115
211006_s_at	5,316	5,661	5,566	5,653	5,746	5,366
211008_s_at	7,404	7,690	7,521	7,774	7,618	7,738
211009_s_at	8,213	7,870	7,707	6,995	7,636	5,973
211010_s_at	6,349	6,245	6,137	6,364	6,318	6,513
211011_at	5,910	6,045	6,202	6,537	6,199	6,048
211012_s_at	8,009	8,226	8,424	8,761	8,217	8,817
211013_x_at	8,195	7,952	8,195	8,370	7,866	8,579
211014_s_at	8,077	7,937	8,043	7,692	7,181	7,491
211015_s_at	10,539	10,459	10,062	9,709	10,013	9,589

211016_x_at	7,291	7,172	7,135	6,350	6,441	6,327
211017_s_at	4,743	4,595	4,476	5,156	4,946	5,510
211018_at	7,912	8,216	7,809	7,649	7,506	8,392
211019_s_at	7,105	7,138	6,913	7,441	6,991	6,998
211020_at	6,604	6,566	6,339	5,677	5,847	5,936
211021_s_at	6,633	6,538	6,344	7,593	7,986	7,820
211022_s_at	6,146	5,617	5,350	5,944	5,601	5,772
211023_at	12,515	12,528	12,453	12,021	12,157	12,153
211024_s_at	4,853	4,969	5,054	5,335	5,113	5,121
211025_x_at	10,570	10,637	10,683	10,572	10,424	10,759
211026_s_at	6,037	6,208	6,008	9,943	9,455	10,065
211027_s_at	4,869	4,992	4,724	5,561	5,084	5,424
211028_s_at	7,905	7,974	7,788	7,372	7,063	7,318
211029_x_at	6,807	6,707	6,784	6,578	6,596	6,677
211030_s_at	6,627	6,911	7,186	7,712	7,007	7,696
211031_s_at	7,216	7,334	7,046	7,944	7,668	8,173
211032_at	3,917	3,845	3,805	4,446	4,190	4,165
211033_s_at	9,152	9,818	9,291	8,840	8,571	9,131
211034_s_at	9,658	9,375	9,866	8,662	9,493	9,039
211036_x_at	11,846	11,782	12,046	11,758	11,638	11,880
211037_s_at	8,448	8,385	8,809	8,608	8,257	8,415
211038_s_at	9,330	9,113	9,295	8,228	7,615	8,060
211039_at	5,942	5,916	6,090	6,147	6,105	6,260
211040_x_at	9,794	9,748	9,814	9,639	9,961	9,564
211042_x_at	10,953	10,544	10,836	9,325	8,806	8,760
211043_s_at	8,992	8,934	9,001	10,238	9,595	10,232
211044_at	5,041	5,246	5,528	5,414	5,508	5,220
211045_s_at	5,895	5,831	5,781	6,446	6,516	6,578
211046_at	3,929	4,121	3,952	4,598	4,692	4,667
211047_x_at	11,009	10,788	11,171	11,597	11,279	11,829
211048_s_at	10,724	10,387	10,605	10,217	9,546	9,772
211049_at	6,824	6,695	6,973	7,728	7,601	7,910
211050_x_at	5,993	5,606	5,756	6,183	5,712	5,750
211051_s_at	8,299	8,099	8,395	8,227	7,866	7,984
211052_s_at	9,470	9,553	9,217	9,218	8,763	9,161
211053_at	7,633	7,662	7,843	7,748	7,597	7,783
211054_at	3,665	3,703	3,785	3,702	3,515	3,662
211055_s_at	7,043	7,295	7,512	6,021	5,950	5,545
211056_s_at	8,935	9,000	9,187	8,439	8,339	8,774
211057_at	8,828	9,381	8,868	7,103	8,504	7,577
211058_x_at	14,578	14,468	14,449	14,100	14,086	14,339
211059_s_at	7,902	8,390	8,077	8,875	8,616	9,404
211060_x_at	9,287	8,998	9,248	10,151	9,842	10,249
211061_s_at	9,105	9,245	9,109	9,753	9,491	9,997
211062_s_at	7,739	8,151	8,258	8,102	7,681	7,832
211063_s_at	9,832	10,292	9,698	8,798	9,943	9,054
211064_at	7,207	7,252	7,258	5,760	6,072	5,915
211065_x_at	9,207	9,324	9,420	9,118	8,798	9,188
211066_x_at	8,716	8,565	8,730	9,606	9,839	9,981
211067_s_at	7,431	7,065	7,666	7,557	6,820	6,914
211068_x_at	8,801	8,765	8,917	8,357	9,381	8,597
211069_s_at	12,961	12,881	12,539	11,733	11,922	11,788
211070_x_at	11,942	12,014	11,826	11,742	11,373	11,853
211071_s_at	12,998	12,573	12,755	11,294	11,457	12,041
211072_x_at	14,571	14,510	14,523	14,123	14,114	14,391
211073_x_at	14,458	14,717	14,490	13,822	13,887	14,069

211074_at	8,134	8,765	9,372	9,906	9,312	9,859
211075_s_at	10,366	10,347	10,026	11,993	11,439	11,913
211076_x_at	6,171	6,038	6,284	6,607	6,626	6,748
211077_s_at	6,522	6,654	6,641	6,180	5,943	6,144
211078_s_at	3,997	4,349	4,064	4,089	4,188	4,091
211079_s_at	6,229	5,987	6,172	6,229	6,269	6,591
211080_s_at	8,722	8,921	7,598	6,268	6,019	6,315
211081_s_at	6,836	6,958	6,944	6,471	5,743	6,389
211082_x_at	5,640	5,486	5,948	5,665	5,629	5,615
211083_s_at	4,477	4,334	4,903	5,282	5,098	5,398
211084_x_at	9,418	9,149	8,834	6,614	7,612	6,759
211085_s_at	7,205	6,839	6,730	6,032	5,873	6,071
211086_x_at	6,951	7,040	6,383	6,116	6,558	6,040
211087_x_at	5,818	6,204	5,892	7,006	6,813	7,354
211088_s_at	4,344	4,098	3,847	4,251	4,259	4,144
211089_s_at	6,909	6,614	6,381	5,742	5,447	5,543
211090_s_at	9,310	9,258	8,621	6,918	7,938	6,557
211091_s_at	6,103	5,705	6,145	7,110	6,738	7,511
211092_s_at	5,923	5,943	6,044	6,646	6,502	6,665
211093_at	3,683	3,711	3,630	3,891	3,834	3,903
211094_s_at	6,647	6,855	6,887	7,017	7,212	7,242
211095_at	4,039	4,360	4,589	4,387	4,748	4,415
211096_at	7,004	6,676	7,033	7,862	8,048	7,881
211097_s_at	6,615	6,776	6,886	7,085	7,040	6,996
211098_x_at	9,225	9,014	9,098	8,838	9,107	9,763
211099_s_at	6,762	6,230	6,372	6,628	6,697	6,830
211100_x_at	6,886	7,145	7,222	8,004	7,462	7,758
211101_x_at	7,064	7,293	7,228	8,075	7,816	7,827
211102_s_at	5,770	5,320	5,473	6,297	6,398	6,596
211103_at	6,556	6,397	6,440	6,304	6,628	6,895
211104_s_at	6,140	5,549	6,124	7,099	7,016	7,154
211105_s_at	9,037	9,638	9,439	7,287	7,432	7,449
211106_at	5,754	5,892	6,230	6,381	6,395	6,453
211107_s_at	9,950	9,846	10,218	8,593	8,360	8,326
211108_s_at	4,413	4,340	4,625	4,707	4,629	4,528
211109_at	5,880	5,837	6,206	6,354	6,055	6,186
211110_s_at	7,388	7,360	7,542	7,237	7,393	7,560
211111_at	7,026	7,009	6,886	7,554	7,435	7,575
211112_at	7,959	8,206	8,555	8,960	8,946	9,177
211113_s_at	6,609	6,952	6,663	6,881	6,766	6,821
211114_x_at	8,311	8,825	8,126	7,124	7,132	7,011
211115_x_at	9,394	9,555	8,937	7,758	7,355	8,045
211116_at	5,326	5,398	5,716	6,363	6,095	6,274
211117_x_at	5,917	5,712	5,759	7,189	6,698	6,968
211118_x_at	6,283	6,014	6,184	6,602	6,706	6,881
211119_at	4,317	4,014	4,032	4,776	4,563	4,774
211120_x_at	6,049	5,988	5,530	6,824	6,826	6,842
211121_s_at	7,385	7,527	7,435	8,796	8,091	8,677
211122_s_at	3,679	3,736	3,563	3,866	3,674	3,703
211123_at	8,087	8,200	8,283	8,598	8,387	8,669
211124_s_at	4,048	4,147	4,523	4,732	4,689	4,926
211125_x_at	7,210	6,943	7,079	7,064	6,830	6,979
211126_s_at	12,079	12,318	11,913	8,808	8,368	9,192
211127_x_at	5,208	4,886	5,177	4,868	4,888	4,785
211128_at	5,347	4,959	5,469	5,870	5,613	5,673
211129_x_at	6,388	6,141	6,402	5,919	5,864	6,068

211130_x_at	6,880	6,891	7,120	6,831	7,077	6,972
211131_s_at	4,769	4,844	4,841	4,964	5,496	5,350
211132_at	6,580	6,526	6,789	7,533	7,282	7,156
211133_x_at	7,720	7,236	7,723	8,548	8,233	8,380
211135_x_at	7,978	7,579	7,720	8,983	8,891	8,858
211136_s_at	8,323	7,900	8,647	8,930	8,042	9,241
211137_s_at	10,323	10,347	10,062	8,768	9,968	8,670
211138_s_at	5,052	5,142	5,514	5,450	5,436	5,457
211139_s_at	7,455	7,159	7,125	7,217	7,323	7,163
211140_s_at	6,974	6,634	6,735	7,387	6,970	7,147
211141_s_at	5,810	5,002	5,207	5,887	5,811	6,015
211142_x_at	7,957	8,046	7,973	8,966	8,933	9,448
211143_x_at	7,470	8,013	7,692	7,890	7,723	7,710
211144_x_at	5,262	5,087	5,182	5,774	5,806	5,780
211145_x_at	5,905	6,003	6,143	6,666	6,579	6,798
211146_at	5,319	5,376	5,949	6,088	5,606	5,729
211147_s_at	6,384	6,487	6,279	6,803	6,800	6,684
211148_s_at	5,886	5,432	6,043	6,978	6,771	7,084
211149_at	3,878	4,070	3,864	3,842	4,176	4,106
211150_s_at	9,474	9,495	9,330	9,316	8,728	9,001
211151_x_at	5,550	5,951	5,878	5,684	5,375	5,627
211152_s_at	8,978	8,791	8,974	9,707	8,973	9,693
211153_s_at	5,747	6,073	5,801	5,859	5,372	5,771
211154_at	4,878	5,152	4,479	5,285	5,370	5,108
211155_s_at	3,970	4,161	4,119	4,513	4,456	4,719
211156_at	5,337	5,396	5,666	5,442	5,470	5,351
211157_at	7,343	6,992	7,285	7,605	7,418	7,695
211159_s_at	8,625	8,650	8,497	8,908	8,352	8,978
211160_x_at	9,025	8,925	9,053	10,372	9,724	10,884
211161_s_at	4,627	4,957	5,053	8,478	12,582	11,041
211162_x_at	7,752	7,390	7,730	7,489	7,196	7,526
211163_s_at	6,708	6,797	5,939	6,748	6,844	6,853
211164_at	3,752	3,886	4,154	3,894	3,762	3,966
211165_x_at	7,917	7,791	7,468	6,965	7,019	7,260
211166_at	7,665	7,344	7,493	8,213	7,695	8,139
211167_s_at	6,500	6,984	6,828	7,160	6,946	7,150
211168_s_at	10,061	9,853	10,279	9,057	8,908	9,005
211169_s_at	3,939	4,120	3,758	4,241	4,106	4,137
211170_s_at	5,326	5,923	5,693	5,766	5,797	6,516
211171_s_at	6,041	5,787	6,086	6,473	6,140	6,436
211172_x_at	7,137	6,964	6,958	7,600	7,370	7,577
211173_at	7,231	7,763	7,547	7,654	7,496	7,766
211174_s_at	6,837	6,630	6,851	7,171	7,239	7,431
211175_at	6,588	6,777	6,472	7,009	6,866	7,280
211176_s_at	6,698	6,773	7,005	7,348	6,972	7,499
211177_s_at	8,370	8,188	8,387	8,583	8,162	8,711
211178_s_at	6,016	5,597	5,893	6,651	7,108	6,874
211179_at	4,129	4,112	3,775	4,284	4,137	4,160
211180_x_at	7,232	6,744	6,850	7,223	7,105	7,145
211181_x_at	6,685	6,909	7,165	7,091	7,129	7,326
211182_x_at	6,961	6,910	6,424	7,108	7,233	7,235
211184_s_at	5,921	6,388	6,437	6,713	6,552	6,713
211185_s_at	13,287	13,328	12,995	10,995	12,195	10,882
211186_s_at	7,181	6,820	6,990	8,061	7,727	7,466
211187_at	3,716	3,565	3,746	3,796	3,792	3,813
211188_at	6,235	5,999	6,247	6,228	6,004	6,139

211189_x_at	6,099	6,474	6,477	7,127	6,642	7,067
211190_x_at	6,156	5,968	6,254	6,633	6,499	6,414
211191_at	5,175	5,241	5,326	6,054	6,033	6,010
211192_s_at	5,648	4,834	5,575	6,058	5,748	5,969
211193_at	5,776	6,009	6,268	6,346	6,415	6,485
211194_s_at	5,669	5,510	5,399	5,826	5,818	5,845
211195_s_at	6,375	6,350	6,637	6,498	6,533	6,555
211196_at	4,959	5,379	5,147	5,759	5,375	5,468
211197_s_at	7,657	7,661	7,807	7,692	7,836	7,822
211198_s_at	7,809	7,821	7,990	8,028	7,962	8,273
211199_s_at	5,422	5,473	5,591	5,836	5,467	5,776
211200_s_at	9,289	9,592	8,855	8,354	8,391	8,596
211201_at	5,433	4,842	5,492	5,909	5,671	5,786
211202_s_at	11,155	11,560	11,607	8,331	9,299	8,831
211203_s_at	6,496	6,547	6,733	7,384	7,195	7,600
211204_at	6,596	6,305	6,449	7,064	7,051	7,161
211205_x_at	7,381	7,042	6,855	6,853	7,043	6,885
211207_s_at	3,275	3,924	3,392	3,958	3,810	3,886
211208_s_at	10,886	11,317	10,760	8,896	9,521	8,715
211209_x_at	6,413	6,400	6,299	7,053	7,040	7,188
211210_x_at	7,275	7,277	7,144	8,070	8,104	8,203
211211_x_at	6,345	6,288	6,085	7,006	7,196	7,385
211212_s_at	7,625	7,396	6,979	7,440	6,624	7,172
211213_at	4,228	4,492	4,389	4,602	4,642	4,714
211214_s_at	5,483	5,584	5,579	5,543	5,326	5,415
211215_x_at	7,308	7,278	7,094	8,011	7,260	7,582
211217_s_at	7,888	7,536	7,771	8,261	8,146	8,413
211218_at	4,862	5,010	4,883	5,574	5,345	5,320
211219_s_at	6,840	6,840	6,628	6,280	6,102	6,318
211220_s_at	5,672	5,716	5,097	5,144	5,018	5,354
211221_at	6,421	6,466	6,444	6,659	6,395	6,674
211222_s_at	7,489	7,583	7,762	7,999	7,835	8,016
211223_at	6,729	6,754	6,550	7,430	7,329	7,686
211224_s_at	6,637	6,607	6,498	6,985	6,667	6,993
211225_at	7,601	7,585	7,914	8,285	8,159	8,342
211226_at	5,410	5,279	5,856	6,138	5,747	6,275
211227_s_at	3,947	3,780	3,978	4,031	4,147	4,228
211228_s_at	8,906	9,078	8,235	6,993	7,596	6,893
211230_s_at	6,076	6,207	6,327	6,426	6,383	6,453
211231_x_at	7,365	7,226	7,441	7,720	7,767	7,985
211232_x_at	6,877	6,735	6,916	7,649	7,588	7,710
211233_x_at	6,905	6,485	6,601	7,460	7,432	7,539
211234_x_at	7,197	7,112	7,339	7,424	7,463	7,483
211235_s_at	7,071	6,843	7,087	7,444	7,177	7,579
211237_s_at	8,029	8,050	7,591	7,540	7,575	7,674
211238_at	5,660	5,903	6,022	6,043	5,990	6,264
211239_s_at	3,718	3,649	4,013	3,912	3,830	3,759
211240_x_at	10,172	9,938	10,141	8,369	8,712	8,661
211241_at	6,876	6,728	7,441	8,765	8,751	8,877
211242_x_at	6,511	6,314	6,066	6,969	7,047	7,206
211245_x_at	6,240	6,112	6,327	6,609	6,518	6,515
211248_s_at	6,687	6,690	6,518	7,288	7,152	7,225
211249_at	6,190	6,297	6,554	6,419	6,765	6,701
211250_s_at	7,366	7,252	7,422	7,761	7,077	7,676
211251_x_at	8,554	8,761	8,593	8,317	8,016	8,453
211252_x_at	8,303	8,498	8,312	9,083	8,946	9,176

211253_x_at	8,091	8,067	7,980	8,408	8,049	8,497
211254_x_at	6,679	6,343	6,579	6,895	6,723	6,886
211255_x_at	8,609	8,128	7,723	7,527	7,178	7,369
211256_x_at	8,602	8,777	8,660	8,326	8,787	8,597
211257_x_at	10,800	11,061	10,246	7,809	9,405	7,584
211258_s_at	7,246	7,099	7,248	7,110	6,885	7,117
211259_s_at	7,225	6,995	7,292	7,525	7,055	7,225
211260_at	7,583	7,615	7,647	7,480	7,565	7,833
211261_at	8,654	8,477	8,447	9,192	9,194	9,418
211262_at	5,195	5,097	5,513	5,683	5,703	5,665
211263_s_at	6,748	6,659	7,053	7,475	7,511	7,738
211264_at	3,715	3,842	3,531	3,663	3,652	3,697
211265_at	4,113	4,286	4,104	4,948	4,889	4,870
211266_s_at	7,374	7,036	7,200	8,092	7,533	7,747
211267_at	8,902	8,345	8,498	5,891	5,993	6,124
211269_s_at	6,738	6,938	7,033	7,071	6,867	7,056
211270_x_at	13,124	12,892	13,575	12,498	12,257	12,429
211271_x_at	11,753	11,561	11,984	10,832	10,627	10,863
211272_s_at	5,091	5,314	5,026	5,613	5,686	5,909
211273_s_at	5,253	5,033	4,977	5,532	5,555	5,589
211274_at	6,487	6,837	6,377	7,129	6,784	7,085
211275_s_at	10,688	11,124	11,498	11,599	11,461	11,738
211276_at	8,690	8,903	8,594	5,520	5,507	5,512
211277_x_at	9,062	8,624	9,050	8,296	8,719	8,402
211279_at	6,673	6,628	7,042	7,355	7,219	7,116
211280_s_at	7,245	6,989	6,681	6,419	6,339	6,567
211282_x_at	7,934	7,432	7,430	8,198	7,966	8,301
211284_s_at	8,799	8,897	9,029	9,840	9,432	10,109
211285_s_at	10,551	10,437	10,259	9,248	10,049	9,243
211286_x_at	6,332	6,112	6,351	6,671	6,397	6,115
211287_x_at	6,069	6,348	6,516	6,906	6,648	6,629
211289_x_at	9,342	9,061	8,954	8,511	8,555	8,509
211295_x_at	6,951	6,446	6,954	7,033	7,387	7,240
211296_x_at	13,865	14,173	14,009	13,802	14,095	14,148
211297_s_at	10,557	10,619	10,088	10,107	10,184	10,334
211298_s_at	4,336	4,496	4,369	5,288	5,030	5,367
211299_s_at	6,893	6,819	6,799	6,904	6,505	6,877
211300_s_at	6,339	6,564	6,241	6,020	5,821	6,007
211301_at	6,637	6,708	6,777	6,925	6,849	7,068
211302_s_at	7,549	7,414	6,995	6,750	6,519	6,617
211303_x_at	5,485	5,725	5,679	5,643	5,781	5,783
211304_x_at	7,910	7,945	8,009	8,551	8,897	8,906
211305_x_at	6,803	6,918	6,855	6,974	6,853	6,889
211306_s_at	5,020	5,278	4,908	6,407	6,451	6,629
211307_s_at	5,924	5,894	5,777	7,137	6,844	7,485
211310_at	4,309	4,540	4,394	4,107	3,921	3,991
211312_s_at	6,486	5,952	6,131	6,778	6,196	6,814
211313_s_at	6,458	6,272	6,203	7,334	6,893	7,312
211314_at	6,441	6,391	6,388	6,771	6,617	6,647
211315_s_at	5,878	5,924	5,807	6,514	6,584	6,630
211316_x_at	7,612	7,531	7,622	7,918	8,550	8,249
211317_s_at	5,694	5,397	5,349	6,088	5,959	5,852
211318_s_at	10,191	10,120	10,708	9,920	9,560	9,746
211319_at	5,235	5,570	5,369	5,596	5,313	5,520
211320_s_at	8,343	8,263	8,317	8,368	8,203	8,622
211322_s_at	6,276	6,582	5,846	6,802	6,883	6,891

211323_s_at	6,345	7,092	6,464	6,425	6,181	6,433
211324_s_at	5,100	5,131	5,374	5,382	5,913	5,354
211325_x_at	7,629	7,705	7,727	8,004	8,053	8,070
211326_x_at	7,119	7,140	7,040	7,972	7,760	7,790
211327_x_at	6,421	6,453	6,436	6,956	6,725	7,017
211328_x_at	7,478	7,317	7,314	8,162	7,866	8,122
211329_x_at	6,469	6,288	6,508	7,407	7,216	7,571
211330_s_at	5,465	5,626	5,533	7,089	6,628	7,180
211331_x_at	7,294	7,178	7,301	7,593	7,535	7,788
211332_x_at	7,821	7,734	7,862	8,686	8,616	9,037
211333_s_at	5,179	5,116	5,223	5,150	5,500	5,230
211334_at	6,343	6,090	6,393	6,700	6,673	6,816
211336_x_at	7,489	7,013	7,202	8,109	8,039	8,415
211337_s_at	8,184	8,352	8,340	8,925	7,700	8,129
211338_at	4,009	4,075	4,446	4,965	4,845	4,881
211339_s_at	6,409	6,386	6,172	5,916	5,818	5,983
211340_s_at	9,045	8,861	9,119	10,041	8,358	9,707
211341_at	4,968	5,054	4,770	5,259	4,988	4,918
211342_x_at	8,343	8,383	8,339	7,817	7,936	8,200
211343_s_at	9,237	9,202	8,380	9,505	8,200	8,406
211345_x_at	14,378	14,414	14,401	13,568	13,577	13,829
211347_at	6,630	6,813	6,801	7,272	7,073	7,006
211348_s_at	5,911	5,886	5,834	6,107	5,942	6,280
211349_at	6,522	6,380	6,495	7,189	6,913	6,869
211350_s_at	6,476	6,262	6,255	6,844	6,506	6,780
211351_at	4,081	4,070	4,008	4,639	4,610	4,800
211352_s_at	6,762	6,637	6,424	5,727	6,422	5,774
211353_at	5,744	5,226	5,441	5,998	5,973	6,191
211354_s_at	6,108	6,357	5,740	5,601	7,075	5,864
211355_x_at	5,959	6,338	5,892	5,807	7,060	5,968
211356_x_at	6,188	6,576	6,294	6,034	7,194	6,039
211357_s_at	4,941	5,011	5,386	5,475	5,376	5,505
211358_s_at	7,886	7,322	7,400	8,168	8,129	8,435
211359_s_at	7,032	7,039	6,925	7,139	6,775	6,874
211360_s_at	7,662	7,565	7,764	7,953	7,696	7,582
211361_s_at	3,723	3,645	3,729	3,665	3,518	3,691
211362_s_at	5,525	5,562	5,291	5,917	5,518	5,813
211363_s_at	7,248	6,782	6,598	6,620	6,581	6,610
211364_at	6,664	6,612	6,584	7,202	7,199	7,410
211365_s_at	5,334	4,993	5,124	5,792	5,705	6,148
211366_x_at	6,658	6,629	6,694	8,292	8,027	7,699
211367_s_at	4,811	4,784	4,684	6,570	6,155	5,934
211368_s_at	5,426	4,732	4,379	8,313	7,720	6,585
211369_at	4,967	5,262	5,186	5,447	5,280	5,516
211370_s_at	8,514	8,181	8,497	7,772	7,515	7,936
211371_at	4,633	4,601	4,576	4,564	4,503	4,819
211372_s_at	5,988	6,215	6,215	6,492	6,002	6,482
211373_s_at	9,265	9,615	10,011	8,923	8,474	8,686
211374_x_at	7,566	7,327	7,404	7,019	7,773	6,828
211375_s_at	12,629	12,687	12,656	11,899	11,737	11,899
211376_s_at	8,711	9,009	8,166	8,004	7,033	7,171
211377_x_at	7,270	7,065	7,293	7,971	8,049	7,809
211378_x_at	14,392	14,353	14,383	13,807	13,950	14,148
211379_x_at	7,193	7,245	7,104	7,318	7,093	6,088
211380_s_at	6,195	6,229	6,448	6,619	6,594	6,670
211381_x_at	5,878	6,024	5,720	6,317	5,974	6,328

211382_s_at	9,446	8,968	9,671	8,302	8,326	8,311
211383_s_at	9,109	9,072	9,272	8,515	9,112	8,913
211384_s_at	6,993	6,993	6,943	7,016	6,972	7,174
211385_x_at	8,395	8,371	8,423	7,729	7,613	7,749
211386_at	5,177	5,098	4,958	4,224	4,920	4,255
211387_x_at	7,775	7,185	7,487	6,930	7,215	6,807
211388_s_at	6,562	6,647	6,677	7,679	7,447	7,543
211389_x_at	5,958	5,734	6,303	6,600	6,699	6,753
211390_at	4,485	4,275	4,660	5,482	5,322	5,325
211391_s_at	6,257	5,999	6,037	6,381	6,368	6,488
211392_s_at	7,790	7,614	7,658	8,051	8,226	8,252
211393_at	6,543	6,401	6,548	6,895	6,367	6,500
211395_x_at	6,151	6,185	6,364	7,360	7,378	7,528
211396_at	7,018	6,910	7,326	7,981	8,012	8,371
211397_x_at	6,546	5,695	6,627	7,488	7,036	7,404
211398_at	6,234	6,367	6,450	6,559	6,307	6,396
211399_at	7,165	6,876	7,456	8,772	8,393	8,717
211400_at	6,302	6,628	6,303	6,873	6,482	6,748
211401_s_at	7,627	7,554	7,413	6,887	6,759	6,905
211402_x_at	7,935	7,860	7,711	8,206	7,808	7,932
211403_x_at	7,258	7,176	7,155	7,464	7,284	7,620
211404_s_at	9,265	9,114	9,094	8,503	9,499	8,813
211405_x_at	6,958	6,886	6,877	7,816	8,040	8,084
211406_at	6,106	6,644	6,706	7,803	7,323	7,808
211407_at	6,727	5,942	6,056	6,983	7,196	7,476
211410_x_at	6,777	6,733	6,971	7,460	7,581	7,608
211411_at	6,904	7,103	6,877	7,569	7,629	7,640
211412_at	3,333	3,815	3,671	4,243	4,137	3,970
211413_s_at	6,057	5,904	5,100	6,498	6,831	6,971
211414_at	4,451	4,667	4,442	5,209	4,829	5,110
211416_x_at	7,471	7,272	7,451	7,440	7,558	7,654
211417_x_at	8,202	8,200	8,160	7,663	7,295	7,652
211419_s_at	5,514	5,588	5,528	6,156	6,060	5,836
211421_s_at	10,087	9,015	10,016	5,308	5,465	5,428
211422_at	4,836	5,154	5,386	5,614	5,818	5,637
211423_s_at	10,959	11,135	10,446	9,586	10,332	10,020
211424_x_at	5,274	4,697	5,071	5,006	5,374	5,016
211425_x_at	6,834	7,168	7,104	5,915	5,759	6,192
211426_x_at	7,431	7,333	7,503	7,843	7,289	7,568
211427_s_at	3,896	3,705	4,096	4,055	4,301	4,178
211428_at	3,991	4,170	4,460	4,105	4,012	4,073
211429_s_at	7,268	7,281	7,197	8,217	8,271	8,440
211430_s_at	6,378	6,211	6,298	7,547	7,323	7,654
211431_s_at	5,884	5,392	5,458	5,384	5,230	5,441
211432_s_at	10,067	9,706	9,954	8,698	8,232	8,736
211433_x_at	8,696	8,461	8,713	9,354	9,395	9,688
211434_s_at	6,940	7,055	6,886	7,367	7,446	7,460
211435_at	6,723	6,404	6,913	6,738	6,495	6,540
211436_at	4,419	4,609	4,513	5,055	4,774	4,652
211437_at	4,305	4,412	4,081	5,042	4,973	5,152
211438_at	5,510	5,402	5,663	6,531	6,349	6,530
211439_at	6,932	6,611	6,457	7,155	6,744	6,931
211440_x_at	5,971	5,966	5,952	6,251	6,093	6,400
211441_x_at	4,903	4,998	5,190	5,857	5,955	5,776
211442_x_at	5,380	5,405	5,450	6,075	5,910	6,164
211444_at	7,849	7,410	7,532	7,041	6,752	6,946

211445_x_at	12,170	11,898	12,255	11,638	12,106	11,775
211446_at	6,556	6,549	6,598	6,764	7,038	6,855
211447_s_at	6,471	6,082	5,925	7,143	7,053	7,170
211448_s_at	6,294	6,372	6,363	6,470	6,545	6,526
211449_at	5,212	5,254	5,248	5,095	5,067	5,025
211450_s_at	9,337	9,076	8,444	5,666	5,735	5,245
211451_s_at	6,688	6,244	6,455	7,234	7,115	7,028
211452_x_at	10,908	10,763	10,461	9,709	10,898	9,630
211453_s_at	7,588	7,216	7,495	8,008	7,878	8,147
211454_x_at	8,577	8,007	8,260	7,925	8,232	7,682
211455_at	7,875	7,607	7,694	8,471	8,050	8,453
211456_x_at	11,472	11,225	12,414	13,348	12,966	13,562
211457_at	5,065	5,414	5,547	5,412	5,463	5,289
211458_s_at	12,256	13,019	12,655	8,999	10,553	9,995
211459_at	5,073	5,079	4,928	5,113	5,263	5,197
211460_at	6,497	6,442	6,598	6,812	6,467	6,588
211461_at	7,556	7,309	7,468	7,753	7,768	8,006
211462_s_at	6,316	5,712	6,328	5,647	5,618	5,694
211463_at	4,816	4,754	5,307	5,263	5,034	5,019
211464_x_at	9,096	8,888	8,822	9,096	9,320	8,746
211465_x_at	8,730	8,107	8,635	8,741	8,426	8,497
211466_at	5,828	6,175	5,661	5,749	5,881	5,803
211467_s_at	5,883	5,881	6,224	6,822	6,613	6,842
211468_s_at	6,521	7,021	6,581	8,236	7,886	8,052
211469_s_at	7,151	6,995	6,952	7,725	7,339	7,416
211470_s_at	7,061	8,074	6,896	4,587	4,493	4,518
211471_s_at	7,561	7,396	7,684	8,609	8,309	8,307
211472_at	7,116	7,199	7,116	7,739	7,467	7,795
211473_s_at	6,639	6,105	6,465	5,890	5,985	5,974
211474_s_at	8,891	9,116	9,134	10,696	10,317	10,624
211475_s_at	9,160	8,880	9,232	8,955	8,319	8,894
211476_at	5,249	5,076	5,027	5,426	5,336	5,432
211478_s_at	6,087	6,531	5,915	7,318	7,266	5,995
211479_s_at	4,147	4,094	4,361	4,843	4,633	4,937
211480_s_at	8,030	8,499	7,694	4,745	4,929	5,038
211481_at	6,388	6,075	5,937	5,428	5,474	5,357
211483_x_at	7,281	7,012	6,915	8,158	7,766	8,095
211484_s_at	7,853	7,646	7,603	8,301	7,741	7,803
211485_s_at	6,474	6,645	6,162	6,837	6,394	6,849
211486_s_at	7,263	7,391	7,371	7,433	7,665	7,708
211487_x_at	13,994	14,170	14,153	13,416	13,468	13,686
211488_s_at	6,104	6,236	6,288	7,129	6,545	6,458
211489_at	5,503	5,236	5,668	6,360	5,851	5,965
211490_at	5,317	5,059	5,228	5,435	5,503	5,655
211491_at	5,943	6,049	5,749	6,641	6,690	6,768
211492_s_at	4,898	4,787	5,186	5,472	5,108	5,386
211493_x_at	7,127	7,163	7,182	7,887	7,880	7,700
211494_s_at	6,486	6,824	6,877	6,745	6,949	6,755
211495_x_at	6,920	6,855	6,886	7,252	7,021	7,136
211496_s_at	4,785	5,110	5,060	5,104	5,183	5,169
211497_x_at	6,944	6,871	6,997	7,347	6,973	7,287
211498_s_at	6,781	7,169	7,021	8,037	7,472	7,616
211499_s_at	7,133	7,090	7,166	7,502	7,280	7,656
211500_at	7,643	7,804	7,780	8,490	8,353	8,485
211501_s_at	11,085	10,624	10,843	10,089	10,064	10,198
211502_s_at	7,429	7,203	7,376	7,332	7,572	7,390

211503_s_at	10,049	9,962	9,900	10,307	9,861	10,279
211504_x_at	7,488	7,546	7,604	7,498	7,393	7,136
211505_s_at	9,563	9,343	9,705	8,679	8,738	8,941
211506_s_at	5,705	5,890	5,457	9,449	7,320	7,544
211507_s_at	5,793	6,100	6,327	6,351	6,277	6,625
211508_s_at	4,943	4,630	4,862	4,967	4,864	5,168
211509_s_at	13,461	13,348	13,271	13,504	14,004	13,990
211510_s_at	5,510	5,741	5,750	5,783	5,595	6,053
211512_s_at	7,576	7,549	7,524	7,466	7,071	7,640
211513_s_at	6,461	6,093	6,294	6,857	6,852	6,795
211514_at	4,896	4,873	4,960	5,180	4,777	5,269
211515_s_at	6,041	5,988	6,039	6,451	5,952	6,265
211516_at	4,423	4,381	4,293	4,494	4,521	4,608
211517_s_at	4,491	4,810	4,679	5,437	5,746	5,831
211518_s_at	8,738	8,967	9,124	7,419	7,035	7,419
211519_s_at	9,594	9,885	9,322	7,803	7,060	7,395
211520_s_at	3,899	4,379	4,262	5,026	5,202	4,832
211521_s_at	7,281	7,192	7,333	7,883	7,980	8,004
211522_s_at	4,268	4,346	4,653	4,428	4,102	4,333
211523_at	5,663	5,521	6,042	6,122	6,069	6,092
211524_at	4,294	4,286	4,845	4,243	4,434	4,161
211525_s_at	4,403	4,104	3,988	4,841	4,614	4,936
211526_s_at	5,906	6,100	5,631	6,455	6,584	6,812
211527_x_at	6,378	6,502	6,493	7,744	7,494	7,378
211528_x_at	8,956	8,820	8,759	9,421	9,239	9,614
211529_x_at	9,211	9,218	9,184	9,879	9,571	9,961
211530_x_at	9,114	9,095	9,175	9,580	9,056	9,499
211531_x_at	8,187	7,949	8,012	9,001	8,534	8,751
211532_x_at	6,055	6,044	6,150	6,825	6,641	6,735
211533_at	4,652	4,472	4,546	5,183	4,930	5,139
211534_x_at	6,862	6,631	6,973	7,381	7,190	7,661
211535_s_at	11,330	11,253	11,566	10,064	10,124	10,593
211536_x_at	7,443	7,318	7,055	7,027	6,915	6,948
211537_x_at	8,306	8,321	7,212	6,833	7,089	6,999
211538_s_at	8,979	9,494	8,764	5,882	6,205	6,235
211540_s_at	6,313	6,281	6,430	6,303	6,146	6,614
211541_s_at	7,484	7,356	7,648	7,676	7,731	7,988
211542_x_at	13,598	13,677	13,648	13,211	12,592	13,317
211543_s_at	8,040	8,138	7,994	7,873	8,013	8,183
211544_s_at	6,940	6,463	6,752	6,586	6,266	6,666
211545_at	6,419	6,235	6,760	6,510	6,335	6,593
211546_x_at	8,334	8,871	8,086	9,043	9,046	9,228
211547_s_at	6,729	6,883	6,043	6,159	6,252	5,938
211548_s_at	4,875	5,336	5,020	4,813	4,959	4,726
211549_s_at	5,627	5,806	5,820	6,843	6,777	7,113
211550_at	6,703	6,440	6,459	7,237	7,027	7,278
211551_at	6,506	6,801	6,898	7,085	6,894	7,208
211552_s_at	8,016	7,776	8,156	7,993	8,058	8,391
211553_x_at	6,644	6,855	6,636	7,773	7,477	7,926
211554_s_at	7,392	7,574	7,414	7,165	7,199	7,131
211555_s_at	5,657	5,879	5,883	6,558	6,385	6,596
211556_at	4,257	4,345	4,033	4,330	4,259	4,282
211557_x_at	7,790	7,393	7,491	8,453	8,227	8,384
211558_s_at	9,840	9,858	9,410	9,278	8,793	9,356
211559_s_at	6,487	7,067	6,273	6,055	6,043	6,154
211560_s_at	6,730	6,414	6,705	7,321	6,810	6,887

211561_x_at	7,352	7,485	7,240	7,753	7,450	7,641
211562_s_at	6,211	6,121	6,191	5,995	6,158	6,182
211563_s_at	8,884	9,047	8,511	7,712	8,140	7,933
211564_s_at	6,802	6,487	6,445	8,836	7,858	8,957
211565_at	4,955	4,953	4,978	5,030	4,982	4,802
211566_x_at	9,812	9,880	9,858	9,639	9,062	9,219
211567_at	4,904	4,471	4,270	5,679	5,610	5,440
211568_at	4,111	3,662	3,704	4,125	4,405	4,102
211569_s_at	9,787	10,066	9,916	8,510	8,010	8,210
211570_s_at	5,846	6,022	5,845	6,329	6,245	6,400
211571_s_at	7,932	7,236	7,527	6,550	7,303	6,477
211572_s_at	6,799	7,138	7,098	7,759	6,676	7,437
211573_x_at	6,774	6,561	6,891	7,870	7,217	7,633
211574_s_at	9,009	9,024	8,277	7,962	8,153	7,954
211575_s_at	8,930	8,831	8,720	8,072	8,352	7,740
211576_s_at	9,335	8,946	9,859	10,132	9,002	9,268
211577_s_at	7,449	7,255	7,762	8,399	8,214	8,326
211578_s_at	8,052	8,057	7,442	7,330	7,625	7,273
211579_at	4,719	4,978	5,274	5,891	6,123	5,835
211580_s_at	6,857	6,696	6,573	6,778	6,781	6,891
211581_x_at	7,067	7,209	7,001	7,493	7,387	7,679
211582_x_at	6,422	6,385	6,195	7,156	6,968	7,268
211583_x_at	6,333	6,883	6,831	7,291	7,339	7,227
211584_s_at	7,077	6,970	6,602	6,150	5,736	5,682
211585_at	6,104	5,974	5,814	5,766	5,797	5,754
211586_s_at	6,191	5,837	5,779	6,008	5,543	5,552
211587_x_at	7,773	7,634	7,694	8,193	8,153	8,237
211588_s_at	7,154	6,868	6,816	8,100	7,299	7,448
211589_at	7,297	7,021	7,522	7,279	7,214	7,119
211590_x_at	7,171	7,236	7,526	7,592	7,608	7,612
211591_s_at	5,592	5,803	5,643	5,811	5,729	5,872
211592_s_at	5,028	5,715	5,372	5,814	5,728	5,776
211593_s_at	8,385	8,189	8,109	7,982	8,145	8,174
211594_s_at	10,704	10,734	10,982	10,506	9,877	10,524
211595_s_at	9,969	9,524	10,035	10,649	9,788	10,643
211596_s_at	10,891	10,699	10,915	7,600	7,474	7,742
211597_s_at	6,207	6,466	6,576	7,902	8,078	8,368
211598_x_at	7,822	7,605	7,927	6,714	6,967	6,847
211599_x_at	7,057	7,129	6,822	7,394	7,358	7,189
211600_at	10,929	11,196	12,259	10,776	11,879	11,390
211601_at	3,993	4,127	4,237	4,215	4,077	4,164
211602_s_at	5,444	5,619	4,841	5,145	5,076	4,903
211603_s_at	9,218	8,982	9,233	7,953	7,796	7,913
211604_x_at	8,476	8,390	8,420	8,772	8,832	8,975
211605_s_at	6,630	6,253	6,795	6,951	6,812	6,763
211606_at	5,185	5,917	5,458	5,527	5,443	5,466
211607_x_at	7,107	7,520	7,176	7,755	7,338	7,604
211608_at	5,647	5,588	5,665	6,448	6,692	6,771
211609_x_at	12,547	12,589	12,432	11,593	10,966	11,478
211610_at	6,591	6,670	6,862	7,068	6,904	6,787
211611_s_at	6,530	6,020	6,442	6,628	6,899	6,790
211612_s_at	6,889	6,569	7,085	7,988	7,868	7,093
211613_s_at	6,029	5,835	5,963	6,308	6,339	6,639
211614_at	5,262	5,376	5,721	5,451	5,499	5,474
211615_s_at	12,362	12,712	12,195	10,041	10,665	9,245
211616_s_at	5,349	5,238	5,488	5,938	5,800	6,011

211617_at	7,021	7,152	6,952	7,877	7,905	7,829
211618_s_at	6,467	6,592	6,980	7,461	7,315	7,562
211619_s_at	7,455	7,429	7,739	8,669	8,515	8,647
211620_x_at	6,719	6,830	6,835	6,703	6,628	6,945
211621_at	8,811	8,082	8,087	7,534	8,057	7,816
211622_s_at	9,321	8,987	9,127	9,086	8,353	9,144
211623_s_at	12,653	12,600	12,656	12,307	11,525	11,853
211624_s_at	7,313	7,106	7,188	7,540	7,606	7,749
211625_s_at	5,541	5,685	5,693	6,599	6,096	6,500
211626_x_at	5,838	5,473	5,607	5,811	5,999	5,774
211627_x_at	3,789	3,905	3,602	3,872	3,926	3,875
211628_x_at	13,424	13,399	13,440	13,951	14,045	14,232
211630_s_at	9,371	9,329	9,283	9,854	9,475	9,967
211631_x_at	6,853	6,780	6,546	7,708	7,348	7,715
211632_at	4,795	5,047	4,931	5,285	5,070	5,400
211633_x_at	6,382	6,021	6,091	6,847	6,384	6,551
211634_x_at	7,200	7,169	7,046	7,729	7,574	7,747
211635_x_at	7,483	7,405	7,727	7,670	7,677	7,551
211636_at	4,987	5,188	5,199	5,242	5,201	5,327
211637_x_at	6,704	6,853	6,398	7,396	7,184	7,319
211638_at	5,114	4,936	5,087	5,763	5,272	5,411
211639_x_at	6,719	6,631	6,443	7,746	7,299	7,551
211640_x_at	6,283	6,284	6,685	6,916	6,811	6,920
211641_x_at	6,029	5,727	5,967	7,281	7,178	7,334
211642_at	5,193	4,968	5,315	5,965	6,145	6,223
211643_x_at	7,199	6,863	7,141	7,453	7,425	7,567
211644_x_at	6,442	6,642	6,226	7,303	7,026	6,975
211645_x_at	5,277	5,184	5,066	5,360	5,163	5,203
211646_at	4,065	3,960	4,079	4,587	4,481	5,044
211647_x_at	5,295	5,427	5,755	6,026	5,843	6,077
211648_at	5,784	5,316	5,396	5,703	5,754	5,594
211649_x_at	6,871	6,472	7,060	7,198	7,003	7,142
211650_x_at	6,682	6,325	6,435	7,188	6,880	7,099
211651_s_at	8,644	8,682	8,292	7,940	9,140	8,677
211652_s_at	7,684	7,298	7,286	7,442	7,502	7,420
211653_x_at	6,521	7,076	6,926	12,586	12,145	12,420
211654_x_at	7,317	7,071	7,401	8,024	7,750	7,694
211655_at	4,414	4,748	4,405	4,809	4,525	4,375
211656_x_at	7,303	7,049	7,393	7,783	7,547	7,422
211657_at	6,672	6,714	7,021	7,330	7,218	7,106
211658_at	10,197	10,408	10,297	9,689	9,487	10,054
211659_at	6,368	6,426	6,948	6,781	6,511	6,698
211660_at	7,623	7,784	7,555	8,009	7,743	7,877
211661_x_at	7,977	7,915	7,928	8,002	8,075	8,056
211662_s_at	12,158	12,349	12,382	12,093	12,003	12,353
211663_x_at	7,885	7,497	7,490	7,696	7,897	7,685
211665_s_at	5,619	5,502	5,847	5,728	5,745	5,723
211666_x_at	13,547	13,750	13,302	12,638	12,133	12,768
211667_x_at	6,323	6,356	6,458	7,007	6,906	6,819
211668_s_at	6,799	6,865	6,479	6,779	6,308	6,580
211670_x_at	6,966	6,653	7,087	7,730	7,383	7,625
211671_s_at	10,086	9,888	10,168	9,296	10,574	9,560
211672_s_at	6,984	6,600	6,750	6,925	6,486	7,211
211673_s_at	7,382	7,739	7,711	7,380	7,110	7,548
211674_x_at	6,119	5,991	6,255	6,266	6,152	6,516
211675_s_at	9,286	9,260	8,935	10,013	10,864	10,278

211676_s_at	8,552	8,509	7,938	7,206	7,891	7,917
211677_x_at	7,418	7,457	7,614	8,070	7,921	7,913
211678_s_at	11,505	11,615	11,714	10,902	10,624	10,949
211679_x_at	8,003	7,913	8,199	8,383	8,353	8,506
211680_at	3,629	3,763	3,756	3,762	3,695	3,803
211681_s_at	6,864	6,822	7,140	7,426	7,435	7,915
211682_x_at	6,273	6,169	6,545	6,891	6,611	7,017
211684_s_at	11,881	12,098	11,302	11,138	11,853	11,518
211685_s_at	7,358	7,299	6,954	6,842	6,576	6,717
211686_s_at	11,289	11,410	11,236	9,807	10,185	9,551
211687_x_at	6,406	6,566	6,470	7,057	7,002	7,100
211688_x_at	6,752	6,511	6,113	7,273	7,147	7,346
211689_s_at	6,053	6,440	6,715	5,994	5,521	6,041
211690_at	5,055	5,420	5,170	5,829	5,615	5,640
211691_x_at	7,571	7,642	7,489	8,188	7,804	8,247
211692_s_at	7,390	7,947	7,865	7,826	7,741	7,851
211693_at	7,354	7,341	7,664	8,497	8,709	8,724
211694_at	5,806	5,778	5,504	6,327	6,249	6,435
211695_x_at	6,542	6,291	6,694	7,163	6,599	7,132
211696_x_at	6,969	6,841	7,048	7,128	6,963	7,185
211697_x_at	9,459	9,711	9,816	10,339	10,129	10,243
211698_at	4,089	3,914	4,008	11,466	11,865	11,873
211699_x_at	7,999	8,239	7,842	8,246	8,245	8,267
211700_s_at	10,558	10,737	10,431	8,033	8,548	8,654
211701_s_at	9,198	9,077	8,914	9,235	9,237	9,006
211702_s_at	8,464	9,102	8,776	8,981	9,525	8,911
211703_s_at	9,101	9,969	8,903	9,216	8,794	9,311
211704_s_at	8,709	8,568	8,667	8,610	8,038	8,463
211705_s_at	6,354	6,455	6,781	7,243	6,996	7,418
211706_s_at	6,658	6,291	6,217	6,749	6,678	6,784
211707_s_at	10,190	10,387	9,737	8,070	8,397	8,002
211708_s_at	8,080	7,791	8,244	7,998	7,383	7,653
211709_s_at	7,971	7,584	7,733	7,710	8,697	9,921
211710_x_at	14,137	14,267	14,072	13,456	13,453	13,637
211711_s_at	7,857	8,195	7,888	7,832	8,277	8,350
211712_s_at	5,072	5,365	5,157	5,284	5,507	5,240
211713_x_at	8,934	8,458	8,493	8,419	8,346	8,431
211714_x_at	13,412	13,303	13,341	13,262	12,758	13,487
211715_s_at	8,883	8,509	8,906	8,520	7,391	7,454
211716_x_at	9,052	8,637	9,373	9,997	9,595	10,014
211717_at	8,673	8,814	8,648	8,660	7,948	8,741
211718_at	5,149	4,757	5,559	5,306	5,373	5,217
211719_x_at	9,850	9,965	10,544	13,344	14,425	14,358
211720_x_at	14,619	14,747	14,684	14,028	14,207	14,298
211721_s_at	8,789	8,380	8,169	5,639	5,710	5,389
211722_s_at	6,025	6,399	6,466	6,603	6,138	6,584
211724_x_at	11,168	11,510	11,191	10,608	11,788	10,882
211725_s_at	8,752	8,811	8,918	10,677	9,509	10,205
211726_s_at	6,370	6,430	6,220	7,123	6,591	6,799
211727_s_at	10,771	10,893	10,498	10,972	10,989	11,366
211728_s_at	6,671	6,540	6,446	7,093	6,907	7,317
211729_x_at	10,441	10,842	10,582	10,832	10,558	10,573
211730_s_at	11,038	10,284	10,423	11,825	11,574	12,509
211731_x_at	6,293	6,207	6,505	7,007	7,131	7,161
211732_x_at	5,772	6,060	5,513	6,873	6,590	6,449
211733_x_at	12,119	12,452	11,682	10,954	11,829	11,742

211734_s_at	3,618	3,642	3,774	3,952	3,970	3,976
211735_x_at	7,095	7,103	7,350	7,577	7,462	7,707
211736_at	3,932	4,713	4,193	4,776	4,551	4,712
211737_x_at	10,592	10,595	10,353	7,715	7,244	8,088
211738_x_at	6,589	6,659	6,827	7,054	6,968	7,172
211739_x_at	6,148	6,692	6,551	6,651	6,542	6,554
211740_at	3,740	3,633	3,839	4,077	3,802	3,936
211741_x_at	6,667	6,166	6,368	7,126	6,832	6,951
211742_s_at	4,254	4,562	4,226	7,426	5,507	5,117
211743_s_at	6,359	6,142	6,248	6,713	6,449	6,564
211744_s_at	8,207	8,330	8,024	9,084	8,755	9,078
211745_x_at	7,873	7,766	8,136	8,313	8,236	8,327
211746_x_at	13,193	13,354	13,201	12,839	12,940	13,090
211747_s_at	11,747	11,943	11,662	11,421	10,294	10,671
211748_x_at	7,953	8,008	7,925	8,700	8,759	8,802
211749_s_at	9,111	8,971	8,989	9,955	9,773	9,942
211750_x_at	14,340	14,257	14,186	13,852	13,848	14,134
211751_at	4,158	4,014	3,822	4,716	4,520	4,100
211752_s_at	9,334	9,347	9,372	9,290	8,401	9,352
211753_s_at	5,948	6,222	5,824	6,281	6,133	6,292
211754_s_at	9,844	10,000	10,145	10,582	10,196	10,534
211755_s_at	13,721	13,856	13,706	13,337	13,614	13,470
211756_at	4,182	4,462	4,403	4,561	4,346	4,352
211758_x_at	11,276	11,623	11,439	11,633	12,103	12,192
211759_x_at	10,689	10,496	10,583	10,829	10,084	11,137
211760_s_at	7,686	8,460	7,538	8,506	7,986	8,305
211761_s_at	12,955	12,971	12,731	11,186	11,238	11,074
211762_s_at	13,614	13,739	13,551	12,214	12,290	11,994
211763_s_at	9,082	9,246	8,921	9,040	8,788	8,644
211764_s_at	10,410	10,357	10,305	10,037	9,597	10,091
211765_x_at	14,452	14,481	14,464	13,818	13,928	14,127
211766_s_at	7,093	7,805	6,989	5,636	5,681	5,720
211767_at	8,403	7,850	8,221	7,747	6,934	7,161
211768_at	7,316	7,146	7,419	7,556	7,719	7,515
211769_x_at	9,415	9,746	9,591	10,251	11,067	11,094
211771_s_at	7,241	7,080	7,377	7,811	7,759	7,851
211772_x_at	6,076	6,041	6,198	6,475	6,191	6,244
211773_s_at	7,719	7,829	7,907	7,660	7,338	7,367
211774_s_at	8,541	8,353	8,945	8,326	7,375	7,940
211775_x_at	3,889	4,116	3,889	3,644	3,748	3,631
211776_s_at	6,650	7,602	5,766	5,275	6,000	5,024
211778_s_at	6,171	6,036	5,928	6,335	6,222	6,246
211779_x_at	8,827	8,635	8,912	8,429	8,218	8,526
211780_x_at	9,947	9,608	10,023	9,757	9,761	10,204
211781_x_at	10,718	10,754	11,056	7,567	7,679	7,652
211782_at	4,424	4,367	3,956	4,935	4,660	5,118
211783_s_at	10,891	10,964	11,071	9,381	8,990	9,597
211784_s_at	13,288	12,898	13,316	12,131	12,152	11,960
211786_at	4,947	4,951	4,594	5,590	5,554	5,558
211787_s_at	13,471	13,296	13,444	13,219	13,000	13,248
211788_s_at	6,228	5,931	6,322	6,740	6,492	6,787
211789_s_at	4,459	4,242	4,610	4,841	4,724	4,658
211790_s_at	5,627	5,317	5,393	5,481	5,096	5,447
211791_s_at	6,815	6,926	6,943	7,644	7,072	7,532
211792_s_at	7,467	7,229	7,909	8,322	8,192	8,095
211793_s_at	9,592	9,445	9,505	9,694	9,422	9,626

211794_at	5,912	6,309	6,305	6,469	6,579	6,449
211795_s_at	5,398	5,049	5,346	5,790	5,612	5,636
211796_s_at	6,597	6,420	6,336	6,769	6,747	6,913
211797_s_at	7,935	7,750	7,730	7,576	7,094	7,459
211798_x_at	6,826	6,833	6,804	7,695	7,543	7,713
211799_x_at	8,454	8,518	8,466	9,725	8,674	9,459
211800_s_at	9,464	9,692	9,765	9,707	9,610	9,725
211801_x_at	7,781	7,889	6,979	7,105	7,265	6,604
211802_x_at	8,120	8,109	8,339	8,970	8,927	9,070
211803_at	6,222	6,138	6,298	6,040	5,883	5,860
211804_s_at	6,358	6,314	6,567	6,297	5,945	6,393
211805_s_at	7,204	7,323	7,448	8,605	8,152	8,341
211806_s_at	6,709	6,819	6,629	7,800	7,868	7,768
211807_x_at	7,094	7,810	7,585	7,761	7,803	7,734
211808_s_at	6,985	6,733	6,876	7,658	7,460	7,639
211809_x_at	8,135	7,871	7,873	8,394	8,269	8,538
211810_s_at	6,998	6,992	6,880	6,775	6,447	6,819
211811_s_at	5,010	4,545	5,039	5,284	4,950	5,154
211812_s_at	6,739	6,620	6,917	7,781	7,109	6,635
211813_x_at	6,782	6,736	6,828	10,993	11,141	11,718
211814_s_at	5,272	5,837	5,298	6,249	6,236	5,553
211815_s_at	7,222	7,244	7,147	7,791	7,772	7,855
211816_x_at	6,323	6,166	6,580	7,338	7,183	7,542
211817_s_at	6,909	7,221	7,087	7,701	7,696	7,961
211818_s_at	7,458	7,224	7,309	7,558	7,622	7,857
211819_s_at	6,150	5,976	6,085	6,375	6,065	6,344
211820_x_at	5,993	6,620	6,292	6,471	6,116	6,071
211821_x_at	4,896	5,251	5,458	5,857	5,826	5,599
211822_s_at	6,786	6,396	6,406	7,197	7,307	7,251
211823_s_at	6,870	6,569	6,756	6,981	6,753	7,009
211824_x_at	5,831	6,088	6,042	6,751	6,431	6,863
211825_s_at	6,489	6,114	6,449	6,726	6,189	6,332
211826_s_at	6,153	6,676	6,659	6,462	6,528	6,565
211827_s_at	6,740	6,818	7,038	7,708	7,338	7,653
211828_s_at	6,676	6,641	6,158	6,130	5,928	5,753
211829_s_at	4,992	4,329	5,306	5,832	5,773	6,120
211830_s_at	7,377	7,259	7,499	8,281	8,119	8,616
211831_s_at	5,509	5,627	5,945	6,771	6,389	6,728
211832_s_at	5,418	5,279	5,518	5,824	5,872	5,766
211833_s_at	7,472	6,900	7,050	7,923	6,390	7,415
211834_s_at	5,749	5,488	5,665	6,413	6,352	6,631
211835_at	6,948	6,606	6,836	6,876	6,500	6,841
211836_s_at	5,467	5,700	6,355	6,425	6,266	6,388
211837_s_at	7,200	6,993	7,392	8,071	8,287	8,504
211838_x_at	7,337	6,926	7,084	7,602	7,319	7,862
211839_s_at	6,992	7,144	7,104	7,355	7,540	7,539
211840_s_at	5,986	6,046	6,162	6,476	6,635	6,752
211841_s_at	7,158	7,091	7,184	7,677	7,491	7,683
211842_s_at	5,598	5,890	5,780	5,516	5,792	5,597
211843_x_at	4,850	5,044	4,990	5,288	5,233	5,411
211844_s_at	6,061	5,878	6,327	7,057	6,356	6,735
211845_at	7,093	7,058	7,015	7,383	7,113	7,481
211846_s_at	7,184	6,872	7,061	7,962	7,954	8,110
211848_s_at	5,401	5,531	5,690	6,012	6,062	6,374
211849_s_at	6,103	5,971	5,923	6,260	5,999	6,342
211851_x_at	7,737	7,674	7,370	7,491	7,846	7,846

211852_s_at	7,361	7,428	7,672	7,000	7,346	7,332
211855_s_at	8,296	8,251	8,222	8,323	7,989	8,096
211856_x_at	6,209	6,151	6,397	6,545	6,354	6,565
211858_x_at	12,540	12,814	13,004	12,050	12,376	12,711
211861_x_at	4,438	4,130	4,122	4,438	4,166	4,324
211862_x_at	6,571	6,452	6,434	6,985	7,213	7,155
211863_x_at	7,305	7,071	7,187	7,873	7,645	7,843
211864_s_at	5,125	4,724	5,239	9,109	10,882	9,951
211865_s_at	6,568	6,462	6,325	7,500	7,245	7,592
211866_x_at	7,046	6,799	7,248	7,484	7,387	7,697
211867_s_at	6,405	6,625	6,566	6,772	6,713	6,839
211868_x_at	6,993	6,676	6,993	7,235	6,897	7,096
211869_at	5,668	5,971	5,900	6,883	6,904	7,014
211870_s_at	6,869	6,606	6,682	7,053	6,757	7,210
211871_x_at	7,807	8,190	7,885	7,938	7,587	7,899
211872_s_at	6,982	6,864	7,031	7,443	7,286	7,398
211873_s_at	6,495	6,642	6,864	7,037	6,931	6,865
211874_s_at	6,924	6,697	6,917	7,338	7,120	7,366
211875_x_at	6,542	6,204	6,639	6,096	6,383	6,622
211876_x_at	6,499	6,432	6,444	6,872	6,561	6,825
211877_s_at	5,785	5,807	5,477	5,751	5,807	5,955
211878_at	5,505	5,266	5,409	5,703	5,910	5,930
211879_x_at	6,656	6,383	6,894	7,178	6,581	7,225
211880_x_at	6,037	5,837	6,392	7,104	6,892	6,940
211881_x_at	6,686	7,115	7,104	7,681	7,339	7,815
211882_x_at	6,458	6,174	6,529	6,626	6,441	6,297
211883_x_at	5,813	5,404	5,368	5,950	6,227	6,063
211884_s_at	6,430	6,362	6,564	6,893	7,064	6,949
211885_x_at	6,180	5,849	6,340	7,196	6,684	6,524
211886_s_at	6,209	6,919	6,838	6,992	6,611	6,828
211887_x_at	5,999	5,850	5,365	6,208	5,729	6,063
211888_x_at	7,113	6,730	7,007	6,985	6,786	7,060
211889_x_at	5,787	5,921	5,410	6,332	6,204	6,254
211890_x_at	5,731	6,077	5,628	6,585	6,476	6,683
211891_s_at	6,215	6,044	6,410	6,644	6,439	6,691
211892_s_at	6,294	6,638	6,692	6,781	6,607	6,939
211893_x_at	6,338	6,333	6,252	7,392	7,530	7,846
211894_x_at	6,828	6,922	6,917	7,082	7,235	7,255
211896_s_at	4,566	5,280	4,961	10,908	11,355	11,756
211897_s_at	5,870	6,456	5,911	6,331	6,162	6,281
211898_s_at	6,465	6,306	6,577	6,855	6,825	6,997
211899_s_at	6,449	6,410	6,305	6,484	6,460	6,395
211900_x_at	7,095	6,678	7,118	8,037	8,102	8,108
211901_s_at	7,257	7,232	7,398	7,698	7,558	7,586
211902_x_at	6,673	6,797	6,799	7,654	7,497	7,471
211903_s_at	5,743	5,699	5,365	6,701	6,464	6,532
211904_x_at	7,082	6,999	6,818	8,056	7,928	7,916
211905_s_at	5,828	6,202	5,801	7,564	7,716	7,784
211906_s_at	3,956	4,075	4,093	4,275	3,932	4,150
211907_s_at	5,616	5,795	5,807	6,359	6,389	6,523
211908_x_at	7,031	6,743	6,815	7,411	7,101	7,312
211909_x_at	5,058	5,508	5,658	8,826	9,204	9,355
211910_at	8,006	7,818	7,857	8,440	8,672	8,606
211911_x_at	9,175	9,518	9,142	10,656	9,522	10,392
211912_at	5,732	5,526	6,004	5,823	5,440	5,770
211913_s_at	7,551	7,749	7,104	6,672	6,492	6,551

211914_x_at	6,356	6,175	6,588	6,606	6,777	6,733
211915_s_at	6,630	6,195	6,566	7,310	7,265	7,673
211916_s_at	6,218	6,651	6,622	7,315	7,351	7,370
211917_s_at	4,046	4,337	3,895	4,489	4,003	4,053
211918_x_at	7,319	7,223	7,323	7,899	7,830	7,960
211919_s_at	7,329	7,081	7,062	6,965	6,893	7,075
211920_at	5,906	6,441	6,502	7,238	7,280	7,189
211921_x_at	13,814	13,780	13,636	13,127	12,722	12,735
211922_s_at	5,909	6,127	5,911	8,175	8,172	8,444
211923_s_at	4,557	4,455	4,889	4,989	4,566	4,733
211924_s_at	7,894	7,741	7,832	9,468	9,019	9,443
211925_s_at	6,818	6,675	6,393	7,003	7,380	7,027
211926_s_at	8,299	8,067	8,119	9,062	9,475	9,563
211927_x_at	14,320	14,466	14,406	13,569	13,610	13,871
211928_at	10,389	10,346	10,305	10,018	10,897	10,354
211929_at	12,464	12,493	12,034	9,178	10,453	8,499
211930_at	10,706	10,722	10,055	7,284	8,320	7,518
211931_s_at	13,203	12,728	12,424	12,008	12,362	11,715
211932_at	12,102	11,821	11,808	10,295	10,847	9,610
211933_s_at	12,727	12,630	12,432	11,476	11,806	11,066
211934_x_at	9,157	8,770	9,049	9,029	8,790	9,234
211935_at	13,126	13,209	12,993	11,189	11,876	11,577
211936_at	12,137	11,973	12,260	12,529	12,526	12,598
211937_at	11,730	11,854	11,523	10,361	10,228	10,644
211938_at	11,396	11,738	11,451	10,168	10,524	10,214
211939_x_at	13,389	13,527	13,427	12,837	13,004	12,952
211940_x_at	13,500	13,775	13,820	11,736	11,562	11,822
211941_s_at	11,010	10,965	11,046	10,529	9,540	10,618
211942_x_at	12,999	12,550	12,516	12,136	11,254	12,582
211943_x_at	14,062	14,124	13,927	13,569	13,687	13,972
211944_at	9,755	9,691	9,898	8,456	8,694	8,121
211945_s_at	12,348	12,450	12,195	12,965	13,861	13,518
211946_s_at	13,349	13,312	13,320	11,919	12,756	11,728
211947_s_at	10,525	10,308	10,380	8,760	9,398	8,690
211948_x_at	9,549	9,056	9,109	8,201	8,310	7,833
211949_s_at	8,904	8,727	8,801	8,465	7,961	8,583
211950_at	11,506	11,369	11,838	9,920	11,175	10,714
211951_at	13,055	13,050	13,260	12,094	12,466	11,737
211952_at	8,783	8,678	8,485	6,707	7,339	6,747
211953_s_at	11,380	11,222	11,255	10,017	11,033	10,056
211954_s_at	11,902	11,836	11,616	10,398	11,070	10,369
211955_at	11,352	11,532	11,063	10,507	10,817	10,367
211956_s_at	13,171	13,412	13,296	13,647	13,885	14,015
211958_at	6,174	6,502	6,639	7,028	7,097	6,954
211959_at	5,749	5,880	4,665	5,379	7,861	5,891
211960_s_at	9,569	9,401	9,575	10,435	9,772	10,149
211961_s_at	10,568	10,845	10,570	10,983	10,865	10,998
211962_s_at	11,178	11,437	11,741	11,125	11,142	11,508
211963_s_at	12,512	12,595	12,355	12,317	12,994	12,872
211964_at	10,322	10,232	10,514	11,106	11,039	11,629
211965_at	4,839	4,604	4,877	4,726	4,517	4,491
211966_at	7,392	7,049	7,010	7,421	7,448	7,487
211967_at	12,526	12,575	11,993	11,237	12,033	11,055
211968_s_at	13,586	13,554	12,841	12,613	12,773	11,637
211969_at	14,096	14,116	13,696	13,222	13,840	12,667
211970_x_at	14,432	14,446	14,515	14,027	14,347	14,409

211971_s_at	12,589	12,941	12,113	10,404	11,394	9,786
211972_x_at	14,473	14,489	14,489	13,830	13,911	14,158
211973_at	7,057	7,110	7,074	7,071	6,891	6,966
211974_x_at	12,944	12,956	13,193	10,436	11,920	10,460
211975_at	10,167	9,880	10,184	9,693	9,254	9,789
211976_at	9,838	9,348	9,762	8,294	8,759	8,283
211977_at	9,212	9,506	9,644	8,746	9,509	9,396
211978_x_at	14,452	14,387	14,445	13,874	13,983	14,202
211979_at	8,920	9,135	9,124	8,344	8,936	8,571
211980_at	10,576	10,460	10,585	10,932	11,282	11,550
211981_at	7,519	7,571	7,279	8,065	8,007	8,515
211982_x_at	10,537	10,433	10,719	10,299	10,207	10,445
211983_x_at	14,399	14,405	14,499	14,073	14,416	14,476
211984_at	10,728	10,420	10,602	9,807	10,586	10,111
211985_s_at	10,555	10,433	10,349	9,276	10,026	9,628
211986_at	7,975	7,896	7,897	9,926	11,560	10,841
211987_at	12,259	12,350	11,847	9,568	11,119	9,127
211988_at	11,572	11,446	11,393	10,494	11,169	10,529
211989_at	10,827	10,994	10,586	10,391	10,102	9,843
211990_at	7,931	8,175	8,596	8,865	8,685	8,016
211991_s_at	7,382	6,789	7,222	8,475	8,107	8,413
211992_at	10,680	10,267	10,260	8,899	9,675	9,122
211993_at	10,292	10,112	10,282	8,977	8,482	8,827
211994_at	10,875	10,711	10,692	8,805	9,804	9,097
211995_x_at	14,179	14,088	14,061	13,902	14,157	14,369
211996_s_at	9,700	8,897	9,108	8,321	9,116	8,537
211997_x_at	12,538	12,479	11,969	12,370	11,603	12,227
211998_at	9,870	10,777	10,224	8,340	9,355	8,249
211999_at	13,224	13,420	13,234	12,654	12,631	12,378
212000_at	8,150	7,545	7,322	7,372	7,308	7,215
212001_at	9,361	9,522	9,274	8,701	9,534	8,930
212002_at	9,757	9,180	9,534	9,652	9,315	10,173
212003_at	7,594	7,125	7,546	8,651	8,064	8,436
212004_at	9,295	9,372	9,650	10,254	9,959	10,392
212005_at	8,713	8,167	8,724	8,068	8,318	8,724
212006_at	11,163	11,295	10,464	9,291	10,427	9,287
212007_at	10,394	10,740	10,236	10,018	9,828	9,717
212008_at	8,611	8,461	8,063	7,527	8,263	7,301
212009_s_at	8,650	8,661	8,719	8,600	7,453	8,321
212010_s_at	12,972	13,277	13,091	12,341	12,996	12,489
212012_at	11,839	11,950	12,337	11,032	12,397	12,231
212013_at	10,240	10,070	10,555	9,455	10,878	11,022
212014_x_at	5,472	5,988	6,129	9,444	8,638	8,903
212015_x_at	11,233	11,391	11,455	10,851	10,545	10,821
212016_s_at	8,864	8,663	8,806	8,400	8,006	8,235
212017_at	11,360	11,008	11,207	9,996	10,681	10,323
212018_s_at	10,340	10,253	9,819	9,437	8,968	8,682
212019_at	6,712	6,407	6,752	5,005	5,524	5,290
212020_s_at	8,603	8,464	8,703	8,194	8,712	7,897
212021_s_at	8,839	9,035	8,410	8,497	9,462	8,080
212022_s_at	8,756	9,384	8,530	8,840	9,364	8,611
212023_s_at	9,729	9,300	9,488	7,442	7,884	7,043
212024_x_at	9,574	9,548	9,701	10,054	9,821	10,568
212025_s_at	8,249	8,281	8,120	8,664	8,061	8,850
212026_s_at	6,563	6,964	6,460	7,857	7,612	8,012
212027_at	10,717	11,045	10,281	10,129	10,519	9,873

212028_at	9,794	9,935	9,798	9,124	8,756	8,852
212029_s_at	6,475	6,405	6,401	6,310	6,266	6,193
212030_at	10,558	10,577	9,939	8,905	9,559	8,719
212031_at	10,728	10,819	10,442	9,527	10,671	9,388
212032_s_at	8,898	8,828	8,879	8,574	7,914	8,745
212033_at	11,280	11,169	10,763	8,789	9,884	8,693
212034_s_at	9,590	9,356	9,991	9,386	9,654	9,914
212035_s_at	7,100	6,931	7,398	7,705	7,930	8,045
212036_s_at	11,352	11,503	10,560	9,296	10,435	8,638
212037_at	11,784	11,668	11,299	9,505	9,895	8,472
212038_s_at	11,395	11,109	11,033	11,240	11,257	11,484
212039_x_at	14,479	14,705	14,580	13,944	14,049	14,136
212040_at	8,227	8,037	8,050	7,411	8,233	7,760
212041_at	9,674	9,331	9,892	9,970	9,402	10,126
212042_x_at	14,033	14,137	13,953	13,494	13,760	13,767
212043_at	11,265	11,103	11,256	11,081	12,141	11,685
212044_s_at	8,971	9,282	9,784	8,824	9,629	9,569
212045_at	9,708	9,591	9,719	8,956	9,240	9,282
212046_x_at	9,238	9,033	9,238	9,713	9,379	9,623
212047_s_at	10,142	9,977	10,077	9,998	9,768	10,111
212048_s_at	12,252	12,711	12,707	11,807	11,502	11,750
212049_at	8,618	8,487	8,682	8,052	8,382	8,421
212050_at	9,012	8,885	9,113	8,058	8,947	8,896
212051_at	8,487	8,667	9,126	8,210	8,627	8,469
212052_s_at	9,208	9,046	9,302	9,496	9,490	9,785
212053_at	9,781	9,891	9,931	8,889	9,423	9,266
212054_x_at	8,714	8,701	8,874	8,817	9,042	9,217
212055_at	10,116	9,812	10,169	10,390	9,803	10,484
212056_at	9,971	9,644	9,827	7,646	7,967	7,907
212057_at	9,401	9,179	9,290	6,508	6,941	7,251
212058_at	11,199	11,123	10,395	8,932	9,869	8,262
212059_s_at	8,758	8,858	8,814	8,911	8,717	9,157
212060_at	10,717	10,876	10,172	8,646	9,656	7,884
212061_at	10,379	10,130	9,768	6,542	7,485	5,769
212062_at	8,765	9,021	8,498	7,910	9,806	9,405
212063_at	6,377	6,481	6,929	12,247	12,437	12,401
212064_x_at	9,162	9,272	9,731	9,058	8,221	9,287
212065_s_at	8,337	8,684	8,156	6,314	7,774	6,398
212066_s_at	11,138	11,394	10,895	9,513	11,205	9,532
212067_s_at	8,143	8,426	8,288	8,103	7,390	7,589
212068_s_at	10,212	9,997	10,491	8,819	9,136	9,092
212069_s_at	8,701	8,163	8,745	7,013	7,256	7,638
212070_at	7,154	7,127	6,684	9,348	8,230	8,456
212071_s_at	10,802	10,804	10,981	8,835	10,613	9,924
212072_s_at	11,994	12,063	12,018	10,629	10,327	10,650
212073_at	11,074	11,107	11,241	9,401	9,629	9,624
212074_at	11,123	11,494	11,348	9,652	10,273	9,913
212075_s_at	9,953	9,966	10,050	9,242	8,809	9,277
212076_at	8,439	8,161	8,364	7,537	7,952	7,733
212077_at	12,471	12,350	11,972	11,351	12,583	11,961
212078_s_at	8,783	8,454	8,456	7,993	8,134	7,984
212079_s_at	7,422	6,848	7,135	5,743	5,988	5,861
212080_at	8,266	8,227	8,573	7,037	7,608	7,088
212081_x_at	8,784	8,376	8,649	7,908	7,880	8,035
212082_s_at	13,767	13,631	13,663	13,341	13,481	13,810
212083_at	10,018	9,594	10,597	11,478	11,187	11,401

212084_at	8,075	7,982	8,421	8,340	8,429	8,711
212085_at	12,695	13,049	12,683	12,782	11,904	12,675
212086_x_at	7,928	8,280	8,562	10,505	10,814	10,929
212087_s_at	8,573	8,246	8,796	8,622	8,041	8,380
212088_at	10,961	10,915	11,087	10,776	10,482	11,040
212089_at	9,128	9,074	9,213	10,190	10,436	10,614
212090_at	7,672	7,827	7,880	8,370	7,845	8,458
212091_s_at	6,911	6,841	6,795	8,312	7,975	8,525
212092_at	9,206	8,903	8,738	7,186	7,301	7,107
212093_s_at	7,284	6,746	6,845	6,442	6,265	6,567
212094_at	11,692	10,871	10,736	6,149	8,331	6,525
212095_s_at	8,435	7,546	7,641	7,011	6,879	6,609
212096_s_at	10,106	9,584	9,982	6,314	6,309	6,053
212097_at	8,073	8,523	8,692	13,341	14,036	13,876
212098_at	10,613	10,449	10,964	10,273	10,870	10,679
212099_at	7,111	7,672	8,098	7,923	9,016	9,174
212100_s_at	9,983	9,905	10,022	9,135	8,820	9,411
212101_at	10,912	10,867	11,088	9,605	10,003	10,021
212102_s_at	7,498	7,132	7,469	7,573	7,161	7,492
212103_at	9,881	9,705	10,014	8,650	8,443	8,887
212104_s_at	11,050	11,029	11,003	10,607	11,079	10,926
212105_s_at	8,595	8,170	8,389	7,597	6,810	6,851
212106_at	8,727	8,668	8,690	9,071	8,740	9,290
212107_s_at	11,272	11,117	11,368	9,655	8,868	9,454
212108_at	10,316	10,239	10,571	9,997	10,828	10,244
212109_at	9,498	8,845	9,444	8,054	8,012	7,801
212110_at	11,826	11,849	12,287	12,298	11,843	11,992
212111_at	9,511	10,215	9,444	9,796	10,256	9,894
212112_s_at	9,150	9,711	9,047	10,093	10,315	10,432
212113_at	8,459	8,187	8,362	7,523	7,341	8,007
212114_at	10,621	10,097	10,739	9,363	9,223	9,676
212115_at	13,081	12,884	13,321	11,390	11,091	11,267
212116_at	9,691	9,668	10,100	10,544	10,139	10,489
212117_at	9,199	9,352	9,197	9,306	9,863	9,592
212118_at	10,076	10,202	10,521	9,688	9,028	9,454
212119_at	10,558	10,728	10,399	10,809	11,013	10,801
212120_at	10,304	10,624	10,190	9,867	10,778	10,109
212121_at	9,167	9,405	9,388	10,114	9,901	9,896
212122_at	8,502	8,657	8,486	7,655	8,684	7,468
212123_at	9,355	9,504	9,622	9,926	9,615	9,763
212124_at	10,306	10,287	11,005	9,925	10,719	10,173
212125_at	8,182	8,178	8,165	8,766	8,046	8,748
212126_at	11,900	11,479	11,663	8,771	9,521	8,948
212127_at	9,438	9,361	9,670	10,251	9,650	10,069
212128_s_at	7,784	7,674	7,930	7,694	7,575	7,790
212129_at	11,212	11,106	11,477	11,251	11,718	11,481
212130_x_at	12,543	12,827	12,713	12,178	12,468	12,719
212131_at	11,655	11,666	11,425	9,311	10,149	9,321
212132_at	11,040	11,148	10,702	9,747	10,269	9,719
212133_at	8,957	8,981	9,299	9,022	8,630	8,556
212134_at	8,285	8,342	8,499	9,171	9,425	9,437
212135_s_at	8,954	9,133	8,662	9,290	10,507	9,769
212136_at	9,799	10,002	9,255	10,546	11,776	11,298
212137_at	11,552	11,363	11,791	11,070	11,475	11,265
212138_at	10,775	10,885	10,475	10,261	11,229	9,365
212139_at	10,686	10,806	11,021	10,483	10,906	11,163

212140_at	10,244	10,318	9,855	9,259	10,252	8,569
212141_at	9,981	9,659	10,185	8,980	8,258	8,365
212142_at	7,284	7,473	7,420	6,634	6,349	6,749
212143_s_at	6,925	7,162	6,716	8,105	9,930	8,957
212144_at	8,461	8,771	8,601	9,696	9,790	9,866
212145_at	10,093	10,265	10,438	10,163	9,896	10,217
212146_at	7,331	7,173	7,578	7,879	7,713	8,125
212147_at	9,241	8,902	9,393	8,753	8,514	8,781
212148_at	11,572	11,474	11,508	5,495	6,729	6,012
212149_at	9,837	9,983	9,885	11,269	12,058	11,328
212150_at	9,014	9,225	9,101	10,003	11,352	9,845
212151_at	11,635	11,648	11,684	7,613	8,606	7,865
212152_x_at	10,737	10,131	11,043	8,091	8,336	8,212
212153_at	11,697	11,783	11,840	9,055	9,323	8,657
212154_at	8,347	8,366	7,912	8,489	9,229	9,961
212155_at	10,914	11,075	11,176	9,682	8,902	9,850
212156_at	9,152	8,913	9,104	8,391	8,752	8,682
212157_at	8,786	8,819	8,423	7,822	8,237	8,293
212158_at	9,911	9,944	9,783	8,014	10,070	10,509
212159_x_at	8,913	8,740	8,980	8,478	8,329	8,448
212160_at	12,154	12,185	11,883	11,067	11,732	11,022
212161_at	7,450	7,092	7,408	7,263	7,312	7,562
212162_at	8,182	8,160	7,772	7,562	7,527	7,481
212163_at	10,799	10,644	10,391	8,334	10,246	8,598
212164_at	7,962	8,156	8,332	6,458	6,925	7,087
212165_at	11,898	11,867	12,004	10,781	9,876	10,860
212166_at	10,527	10,510	10,356	8,986	8,823	8,825
212167_s_at	8,483	8,483	8,730	8,713	8,097	8,628
212168_at	11,995	11,631	11,743	10,383	10,978	10,658
212169_at	8,482	8,700	8,009	9,598	10,272	10,458
212170_at	10,482	10,651	10,164	7,143	7,885	7,315
212171_x_at	8,433	8,626	8,415	9,443	9,336	9,270
212172_at	7,592	7,472	7,250	6,226	6,495	6,341
212173_at	9,083	8,769	9,146	7,884	8,028	7,838
212174_at	9,688	9,323	10,027	9,480	9,041	9,156
212175_s_at	11,322	11,455	11,586	10,422	9,838	10,141
212176_at	11,381	11,587	10,380	8,289	10,301	8,160
212177_at	10,023	10,311	9,102	6,945	8,330	6,579
212178_s_at	8,776	8,421	9,006	8,166	8,054	8,512
212179_at	11,193	11,424	10,301	6,419	7,603	5,881
212180_at	11,033	11,103	11,248	9,115	9,260	9,117
212181_s_at	10,182	10,197	10,099	8,611	9,200	8,829
212182_at	4,626	4,099	4,855	3,838	4,019	3,836
212183_at	9,160	9,350	9,158	7,795	8,435	8,544
212184_s_at	10,036	10,356	9,893	8,560	9,216	8,708
212185_x_at	11,951	11,653	13,173	14,129	13,777	14,328
212186_at	10,617	10,630	10,607	8,770	9,483	9,011
212187_x_at	7,828	7,603	7,334	8,724	8,457	8,742
212188_at	8,320	8,617	8,748	11,476	13,024	12,702
212189_s_at	9,766	9,642	10,047	9,085	8,898	9,266
212190_at	10,544	10,652	10,934	11,887	13,784	13,793
212191_x_at	12,058	12,324	12,092	12,208	11,435	12,479
212192_at	8,734	8,577	8,556	10,523	13,005	12,363
212193_s_at	9,165	8,660	8,792	7,584	8,122	8,092
212194_s_at	8,603	8,403	8,686	9,040	8,849	9,527
212195_at	8,678	9,058	8,148	11,541	12,890	11,600

212196_at	6,879	6,935	6,694	8,978	10,271	8,734
212197_x_at	10,712	10,537	10,934	11,437	11,983	12,124
212198_s_at	8,791	8,727	8,711	8,681	8,554	8,872
212199_at	11,916	11,523	11,075	9,817	9,427	9,289
212200_at	9,912	9,758	10,017	8,838	9,368	9,318
212201_at	8,361	8,365	8,278	7,436	7,523	7,812
212202_s_at	9,736	9,694	9,426	8,457	9,272	9,059
212203_x_at	11,783	11,720	12,080	11,389	10,358	11,683
212204_at	9,930	10,193	9,925	9,492	10,222	9,766
212205_at	11,685	11,231	11,203	8,883	9,399	7,740
212206_s_at	10,285	9,791	9,875	7,130	7,831	7,091
212207_at	8,723	8,726	8,752	7,169	9,159	7,829
212208_at	9,285	9,211	9,307	8,703	9,204	8,943
212209_at	8,196	7,989	7,924	6,845	8,141	7,231
212210_at	6,751	6,486	6,738	8,307	7,810	8,241
212211_at	10,142	9,938	10,053	8,509	9,426	8,629
212212_s_at	9,682	9,366	9,813	9,040	8,800	9,169
212213_x_at	10,862	11,232	10,430	9,239	10,287	8,424
212214_at	9,201	9,543	8,685	6,947	8,080	6,684
212215_at	12,197	12,630	11,857	10,654	11,735	10,622
212216_at	7,987	7,956	7,745	7,926	9,057	8,005
212217_at	8,976	9,212	8,592	7,863	9,301	8,210
212218_s_at	8,908	8,981	9,085	8,513	8,012	9,025
212219_at	10,650	10,517	10,589	9,710	10,313	9,117
212220_at	7,659	7,238	7,403	6,554	6,522	6,230
212221_x_at	8,970	8,994	8,815	9,467	9,662	9,254
212222_at	9,652	9,621	9,281	7,852	8,617	7,617
212223_at	8,079	8,387	8,013	8,471	9,318	8,770
212224_at	4,842	4,468	4,866	5,444	5,472	5,847
212225_at	6,396	7,359	6,718	6,156	7,099	6,941
212226_s_at	11,111	11,255	11,323	9,380	11,420	11,842
212227_x_at	12,402	12,811	12,700	12,220	12,466	12,764
212228_s_at	9,827	9,830	10,053	10,108	9,303	10,025
212229_s_at	11,479	11,404	11,241	8,585	8,758	8,823
212230_at	10,996	11,021	11,142	9,220	11,222	11,228
212231_at	10,982	11,244	11,023	8,763	9,160	8,816
212232_at	10,801	10,875	10,171	7,952	9,684	8,106
212233_at	10,919	10,639	10,693	9,998	11,564	10,741
212234_at	8,236	7,919	8,150	7,603	8,437	8,091
212235_at	8,175	8,144	7,453	8,711	8,317	8,524
212236_x_at	7,266	6,975	6,865	7,453	7,492	7,814
212237_at	8,497	8,018	8,495	8,247	8,305	7,747
212238_at	8,180	7,546	7,980	7,817	8,966	8,128
212239_at	8,414	8,266	8,096	7,683	9,860	8,104
212240_s_at	8,548	8,229	7,755	7,882	8,814	7,991
212241_at	8,016	8,581	8,329	7,609	7,700	7,872
212242_at	10,416	10,638	10,755	12,531	11,969	12,115
212243_at	9,947	10,131	9,915	8,160	8,395	8,031
212244_at	10,634	10,645	10,583	8,894	9,304	8,923
212245_at	11,266	11,302	11,200	11,265	12,077	11,906
212246_at	7,952	7,844	8,151	10,530	9,688	10,639
212247_at	11,721	11,724	11,541	9,205	9,741	8,606
212248_at	9,452	9,147	9,461	10,546	11,307	10,186
212249_at	5,537	5,852	5,711	5,036	6,106	5,405
212250_at	10,476	10,733	10,165	11,401	11,878	11,234
212251_at	10,411	10,655	10,353	11,431	12,013	11,241

212252_at	8,953	8,748	8,942	8,844	8,848	9,083
212253_x_at	7,459	7,464	7,464	7,920	9,420	7,936
212254_s_at	9,490	9,381	9,061	9,155	11,421	8,925
212255_s_at	9,210	9,483	9,066	8,950	9,644	8,774
212256_at	7,724	7,874	7,720	8,179	8,886	8,978
212257_s_at	5,891	5,920	5,996	6,206	6,177	5,786
212258_s_at	6,834	7,256	7,109	7,808	7,960	8,037
212259_s_at	8,031	8,029	7,767	7,836	7,962	7,791
212260_at	8,813	8,881	8,630	7,647	8,195	7,426
212261_at	9,558	9,486	9,634	8,893	9,118	8,668
212262_at	8,411	8,511	8,270	7,719	8,529	8,057
212263_at	10,271	10,053	9,984	8,549	9,269	9,120
212264_s_at	9,667	9,639	9,429	9,396	10,119	8,671
212265_at	12,816	12,853	12,666	11,300	12,238	11,604
212266_s_at	11,579	11,535	11,033	10,401	10,915	10,447
212267_at	9,897	9,943	9,633	7,988	9,155	7,394
212268_at	7,987	8,999	8,201	9,144	9,001	9,516
212269_s_at	9,759	9,634	10,065	8,894	9,507	8,935
212270_x_at	13,636	13,849	13,479	13,119	13,338	13,518
212271_at	9,276	9,194	9,197	10,011	10,433	10,659
212272_at	6,750	6,374	6,154	6,452	6,312	6,359
212273_x_at	12,908	13,015	13,347	12,194	12,654	12,964
212274_at	9,750	10,389	9,864	9,063	9,903	9,130
212275_s_at	7,953	7,822	8,046	8,124	8,149	8,429
212276_at	9,529	10,061	9,452	8,240	9,313	8,558
212277_at	8,935	8,925	8,982	8,359	8,318	7,865
212278_x_at	6,543	6,227	6,219	6,988	6,940	7,197
212279_at	9,840	9,571	9,955	9,509	7,598	8,155
212280_x_at	10,290	10,382	10,450	10,077	9,897	10,388
212281_s_at	11,945	11,728	12,113	11,216	9,910	10,136
212282_at	11,992	11,476	11,897	11,048	9,765	10,056
212283_at	7,594	7,545	7,351	8,192	8,034	7,956
212284_x_at	14,572	14,599	14,493	14,053	14,360	14,472
212285_s_at	9,355	9,561	8,992	8,892	8,622	8,478
212286_at	8,177	7,845	7,744	7,091	8,975	6,711
212287_at	10,800	11,057	10,696	9,689	10,818	9,166
212288_at	10,993	11,176	11,239	8,616	10,245	9,807
212289_at	7,485	7,326	6,915	6,607	7,692	5,964
212290_at	8,589	8,446	8,744	9,118	8,733	9,362
212291_at	8,818	8,190	8,548	7,140	7,616	7,343
212292_at	7,362	7,053	7,163	8,064	7,914	8,076
212293_at	10,455	10,433	10,548	7,568	9,106	8,496
212294_at	10,006	10,170	9,649	10,767	12,181	11,765
212295_s_at	11,518	11,416	12,026	11,168	11,276	11,244
212296_at	13,369	13,445	13,247	12,129	12,550	12,388
212297_at	10,943	10,952	10,678	11,275	12,302	10,845
212298_at	5,742	5,378	5,603	10,514	11,266	10,441
212299_at	10,891	10,987	10,987	8,796	9,897	9,321
212300_at	9,117	8,617	8,839	7,552	7,576	8,150
212301_at	10,326	10,161	9,938	8,864	8,975	8,380
212302_at	9,456	9,208	9,389	8,420	8,475	8,170
212303_x_at	8,897	8,715	9,001	8,949	8,656	8,552
212305_s_at	8,856	8,916	8,638	7,828	8,128	7,747
212306_at	9,842	10,397	10,243	8,766	9,929	8,255
212307_s_at	9,650	9,747	9,113	7,260	9,089	7,865
212308_at	8,196	8,379	7,900	4,516	5,944	4,355

212309_at	9,567	9,845	9,495	7,859	8,668	7,682
212310_at	7,769	8,058	7,571	5,650	7,059	6,130
212311_at	8,866	8,580	8,606	7,601	8,012	8,277
212312_at	9,812	9,876	9,759	10,546	10,364	10,512
212313_at	10,383	10,516	10,433	10,232	9,935	10,160
212314_at	11,387	11,430	11,218	9,933	10,728	10,918
212315_s_at	8,407	8,215	8,921	7,363	7,582	7,631
212316_at	8,557	8,427	7,978	7,212	7,061	7,307
212317_at	9,875	9,768	9,942	8,100	8,830	8,195
212318_at	10,319	10,206	10,310	8,594	8,878	8,667
212319_at	8,111	7,650	7,890	7,207	7,825	7,748
212320_at	13,034	12,767	13,200	12,402	12,160	12,661
212321_at	9,628	9,816	10,034	7,743	8,338	8,135
212322_at	9,676	9,506	10,178	9,266	9,100	9,295
212323_s_at	8,525	8,396	8,633	8,317	8,948	7,948
212324_s_at	7,012	7,020	7,156	7,397	7,414	7,292
212325_at	5,748	6,192	6,707	6,051	6,058	6,066
212326_at	8,114	7,924	8,276	7,668	7,870	7,404
212327_at	6,110	6,184	6,971	5,690	5,864	5,784
212328_at	6,419	6,796	7,848	5,551	5,976	5,962
212329_at	10,263	10,064	10,390	9,480	8,885	9,547
212330_at	10,974	10,762	11,074	11,135	10,990	10,791
212331_at	9,697	10,269	9,622	9,169	9,808	9,005
212332_at	7,000	7,187	7,163	7,144	6,932	7,331
212333_at	10,964	10,723	10,821	11,062	11,803	11,399
212334_at	10,170	10,385	10,740	11,149	11,417	11,384
212335_at	7,269	7,671	7,994	9,377	9,618	9,627
212336_at	5,699	6,622	5,816	6,848	7,001	7,176
212337_at	8,927	8,738	8,376	6,761	7,776	6,874
212338_at	5,064	5,106	5,526	5,722	5,149	5,825
212339_at	7,692	7,174	7,147	6,427	6,012	5,828
212340_at	9,126	9,893	9,081	8,302	8,909	8,436
212341_at	11,025	11,282	10,725	9,964	10,433	10,002
212342_at	9,793	9,901	9,372	7,208	8,160	7,080
212343_at	9,739	9,791	9,650	7,330	8,404	7,515
212344_at	6,424	6,525	6,685	7,009	7,212	7,246
212345_s_at	9,286	9,644	9,686	10,479	11,349	10,886
212346_s_at	8,204	7,957	8,061	8,267	8,263	8,891
212347_x_at	7,703	7,819	7,841	9,570	9,510	10,464
212348_s_at	10,418	10,377	10,487	8,910	8,691	8,734
212349_at	8,161	7,711	8,124	8,762	8,851	8,923
212350_at	8,443	8,686	8,246	8,407	8,607	8,412
212351_at	10,051	10,084	10,490	9,760	9,343	9,473
212352_s_at	11,911	12,065	11,916	11,825	12,364	12,166
212353_at	7,467	7,360	6,982	4,960	7,817	5,078
212354_at	7,571	7,903	7,328	7,210	8,102	7,341
212355_at	8,195	8,321	8,664	9,965	9,221	9,633
212356_at	9,129	9,237	9,232	8,557	8,453	8,419
212357_at	8,274	8,175	8,409	7,222	7,961	7,988
212358_at	8,212	8,715	8,147	8,598	8,996	9,313
212359_s_at	7,178	7,003	6,921	7,588	7,665	8,064
212360_at	9,864	9,859	10,062	10,039	9,456	10,186
212361_s_at	11,082	10,828	11,150	9,088	9,863	9,162
212362_at	7,363	7,105	7,202	7,863	7,707	7,685
212363_x_at	14,245	14,103	14,341	13,905	14,210	14,299
212364_at	10,734	10,137	9,793	10,416	10,934	9,600

212365_at	9,827	9,773	9,463	10,124	10,173	9,326
212366_at	8,078	7,515	6,858	5,215	6,690	5,031
212367_at	11,233	11,119	10,830	7,852	8,455	7,458
212368_at	9,992	9,853	9,342	6,433	8,270	6,647
212369_at	10,558	10,264	10,302	9,731	9,195	9,329
212370_x_at	9,096	9,093	9,357	8,879	9,635	8,961
212371_at	12,277	12,290	12,214	9,197	9,926	9,632
212372_at	13,207	13,006	13,151	9,556	10,438	9,378
212373_at	8,494	8,455	8,155	6,284	7,149	6,306
212374_at	8,339	9,054	8,957	7,417	8,133	7,757
212375_at	10,661	10,756	10,831	8,375	9,341	8,508
212376_s_at	9,015	8,823	9,366	8,736	8,980	8,875
212377_s_at	10,583	10,542	11,150	10,312	11,673	11,074
212378_at	11,314	11,350	11,395	10,229	10,156	9,907
212379_at	10,401	10,395	10,399	9,555	8,850	9,309
212380_at	8,509	8,526	8,386	8,326	8,205	8,475
212381_at	9,165	9,251	9,119	8,011	8,995	7,649
212382_at	9,328	9,265	8,650	7,605	7,696	7,531
212383_at	9,294	9,291	9,777	9,276	9,399	9,543
212384_at	7,882	8,157	8,104	6,513	7,206	7,121
212385_at	8,491	8,412	8,383	6,797	7,020	6,918
212386_at	9,696	9,719	9,579	6,199	7,460	6,621
212387_at	9,338	9,330	8,939	7,157	7,164	6,831
212388_at	9,911	10,132	9,690	7,485	9,164	7,361
212389_at	6,938	6,438	6,870	7,558	7,497	7,747
212390_at	6,581	6,743	6,345	9,710	10,667	9,322
212391_x_at	14,613	14,737	14,586	13,985	14,303	14,324
212392_s_at	5,430	5,335	5,449	5,918	6,011	6,062
212393_at	7,975	7,286	7,465	7,482	7,443	7,975
212394_at	7,200	7,290	7,781	7,242	7,272	7,713
212395_s_at	8,603	8,616	9,054	9,469	9,772	9,893
212396_s_at	8,884	8,646	9,219	9,819	9,448	10,287
212397_at	12,008	12,256	11,607	11,292	12,231	11,240
212398_at	7,841	7,862	7,454	7,571	7,993	7,501
212399_s_at	9,816	9,922	10,148	8,660	9,387	9,100
212400_at	8,636	8,730	8,989	8,058	8,057	8,093
212401_s_at	10,177	10,037	10,411	9,786	10,172	10,055
212402_at	11,315	11,321	10,692	8,946	10,066	8,627
212403_at	9,260	9,334	9,587	7,749	7,999	7,924
212404_s_at	6,893	6,514	6,743	6,671	6,515	6,634
212405_s_at	10,724	10,354	10,996	9,869	8,945	9,447
212406_s_at	10,309	10,587	10,186	7,172	8,234	7,178
212407_at	10,632	10,537	11,237	9,245	9,354	9,084
212408_at	10,786	10,758	10,524	10,514	11,063	10,509
212409_s_at	7,588	7,954	7,614	8,728	8,440	8,202
212410_at	11,120	11,308	10,551	10,392	10,706	10,375
212411_at	10,048	9,646	10,211	11,081	10,247	10,951
212412_at	10,405	10,575	10,378	11,116	12,677	11,992
212413_at	8,568	8,999	7,970	6,406	6,648	6,549
212414_s_at	9,921	10,246	9,819	7,033	6,831	6,972
212415_at	9,124	8,641	9,113	6,546	5,873	6,310
212416_at	10,311	10,830	10,125	9,826	10,447	9,637
212417_at	9,689	10,035	9,550	8,768	8,868	8,690
212418_at	8,203	8,779	8,241	7,773	8,973	8,232
212419_at	8,512	8,558	8,424	8,857	9,490	9,681
212420_at	9,189	9,632	8,996	8,934	9,156	9,591

212421_at	7,446	7,481	7,699	6,862	7,036	7,160
212422_at	9,380	9,120	9,588	8,957	9,135	9,016
212423_at	7,975	7,904	7,707	8,307	9,087	9,589
212424_at	9,677	9,648	10,109	9,474	9,645	9,659
212425_at	8,123	8,423	7,778	7,521	7,055	7,123
212426_s_at	13,874	13,706	13,921	13,234	13,738	13,599
212427_at	8,567	8,944	9,050	5,952	6,589	6,317
212428_at	11,078	11,148	10,842	8,791	9,544	8,786
212429_s_at	11,648	11,440	12,369	10,654	10,493	10,652
212430_at	8,167	7,965	8,385	7,861	7,850	8,069
212431_at	8,953	8,935	9,082	9,436	8,926	9,140
212432_at	10,048	9,844	10,096	11,721	10,542	10,895
212433_x_at	14,397	14,512	14,378	13,821	13,981	14,109
212434_at	10,138	10,022	9,960	10,674	10,088	10,261
212435_at	11,522	11,516	11,315	8,277	9,236	7,904
212436_at	10,216	10,221	10,220	7,406	8,620	7,138
212437_at	7,772	7,280	7,542	8,353	7,924	8,319
212438_at	9,816	10,190	9,814	10,745	9,977	10,687
212439_at	9,280	9,073	9,684	8,870	8,708	8,707
212440_at	10,755	10,924	10,812	10,613	10,882	11,039
212441_at	10,853	10,696	10,475	8,338	9,552	8,360
212442_s_at	9,370	9,264	9,304	8,387	8,904	8,673
212443_at	8,418	8,377	8,492	8,892	9,025	9,242
212444_at	7,868	7,254	7,449	7,946	9,504	7,988
212445_s_at	8,710	8,943	8,800	8,350	7,506	7,619
212446_s_at	9,833	9,564	9,800	8,322	8,696	8,136
212447_at	11,110	11,040	10,816	8,824	9,780	9,000
212448_at	7,894	8,161	8,234	6,481	6,200	6,518
212449_s_at	12,489	12,465	12,299	11,789	12,194	12,278
212450_at	8,404	8,566	7,887	7,273	9,020	7,444
212451_at	7,129	7,366	7,165	7,037	7,046	6,788
212452_x_at	7,787	7,614	7,795	7,799	7,497	7,666
212453_at	9,350	9,397	9,367	9,722	10,116	9,949
212454_x_at	10,585	10,349	9,443	8,292	10,367	9,224
212455_at	11,883	11,786	11,369	9,652	10,901	9,565
212456_at	9,856	9,803	10,337	10,792	10,467	10,557
212457_at	10,394	10,527	10,753	10,319	10,291	10,600
212458_at	9,211	9,215	9,696	8,029	8,263	8,093
212459_x_at	10,261	10,688	9,900	10,271	10,428	10,522
212460_at	10,090	9,998	9,717	10,041	9,827	9,983
212461_at	12,005	12,228	12,201	10,482	11,528	10,542
212462_at	9,249	9,234	9,231	7,326	8,842	7,339
212463_at	9,055	9,194	8,958	10,366	11,945	11,264
212464_s_at	9,766	9,879	10,043	13,420	14,430	14,387
212465_at	9,967	10,111	9,987	10,011	10,063	9,663
212466_at	6,637	6,830	6,629	7,172	7,254	7,193
212467_at	9,345	9,505	9,181	9,135	10,469	8,714
212468_at	7,932	8,135	7,648	6,869	8,036	7,061
212469_at	9,721	9,956	9,507	8,404	8,857	8,188
212470_at	9,937	10,072	9,502	9,406	10,692	9,020
212471_at	8,158	8,550	8,289	8,173	8,161	8,158
212472_at	6,281	5,562	6,287	8,634	8,433	9,027
212473_s_at	5,689	5,831	5,632	11,149	11,010	11,380
212474_at	10,915	10,993	10,779	8,535	9,555	8,353
212475_at	9,597	10,172	9,917	8,276	8,760	8,196
212476_at	9,388	9,892	8,930	8,535	10,072	8,508

212477_at	4,196	4,463	4,107	4,276	3,910	4,185
212478_at	7,570	8,112	8,389	6,963	6,874	7,065
212479_s_at	8,253	8,362	8,524	7,913	7,632	7,887
212480_at	10,015	9,643	9,846	8,492	9,502	8,869
212481_s_at	9,680	9,710	9,528	11,242	11,070	11,493
212482_at	10,705	10,750	10,560	7,107	7,795	7,499
212483_at	9,469	9,709	9,117	6,320	7,916	6,477
212484_at	8,079	7,603	8,094	8,296	8,143	8,650
212485_at	9,607	9,624	9,596	8,744	9,126	8,804
212486_s_at	8,140	8,111	8,383	7,252	7,774	6,963
212487_at	9,550	9,522	9,662	8,272	8,782	8,385
212488_at	7,711	7,907	7,906	8,984	10,086	10,742
212489_at	6,446	6,580	6,627	8,571	8,982	9,818
212490_at	8,854	8,847	9,265	8,652	8,633	8,657
212491_s_at	11,231	11,594	11,530	10,346	10,277	10,252
212492_s_at	8,262	8,209	8,657	7,023	6,520	7,554
212493_s_at	10,667	10,767	10,845	8,895	9,938	8,379
212494_at	6,595	6,902	6,799	7,782	7,851	7,803
212495_at	8,026	7,531	8,071	8,598	8,953	8,652
212496_s_at	9,592	9,537	9,964	8,347	8,595	9,030
212497_at	6,564	6,835	7,248	6,881	6,534	7,188
212498_at	11,379	11,245	11,257	9,842	11,056	10,114
212499_s_at	10,641	10,240	10,391	10,395	10,278	10,566
212500_at	9,546	9,522	9,654	8,578	8,872	9,134
212501_at	10,106	10,886	10,896	12,169	11,952	11,645
212502_at	10,361	10,147	10,323	9,958	9,150	9,815
212503_s_at	8,277	8,408	8,294	7,183	8,570	8,389
212504_at	8,996	9,148	9,018	8,180	9,188	8,956
212505_s_at	8,887	8,793	9,201	8,554	8,282	8,457
212506_at	10,173	10,128	9,819	10,141	11,057	10,516
212507_at	9,843	9,732	10,250	9,441	11,044	10,054
212508_at	9,492	10,340	10,074	8,752	8,958	8,962
212509_s_at	10,389	10,531	10,391	12,239	12,635	12,818
212510_at	7,960	8,117	7,897	8,299	8,799	8,459
212511_at	9,492	9,671	9,054	8,040	9,231	8,366
212512_s_at	8,576	8,552	9,120	8,177	8,201	8,331
212513_s_at	11,838	12,048	11,709	10,206	10,838	9,901
212514_x_at	9,281	8,966	8,759	9,136	8,606	8,062
212515_s_at	10,813	10,676	9,953	8,238	8,884	7,587
212516_at	7,742	7,795	7,808	8,290	8,140	8,334
212517_at	8,800	8,750	8,781	7,765	8,407	8,068
212518_at	9,013	9,023	9,324	8,909	9,026	9,206
212519_at	12,516	12,534	12,243	12,047	11,877	12,004
212520_s_at	9,835	9,446	9,950	7,850	7,528	8,126
212521_s_at	8,569	8,630	8,289	8,310	8,333	8,436
212522_at	8,842	9,352	8,642	9,016	9,563	9,157
212523_s_at	9,159	8,912	8,512	6,369	6,519	6,474
212524_x_at	6,432	6,793	6,762	7,159	6,915	6,735
212525_s_at	7,790	6,877	7,223	7,314	7,163	7,222
212526_at	10,004	10,429	9,684	10,089	10,645	9,791
212527_at	6,846	7,259	7,650	9,705	7,951	9,186
212528_at	9,275	8,713	9,330	9,874	9,549	9,773
212529_at	7,647	7,711	7,792	7,694	7,125	7,623
212530_at	9,043	9,976	8,988	9,709	11,272	10,790
212531_at	6,484	6,015	6,104	6,843	6,611	6,634
212532_s_at	10,524	10,590	10,929	10,346	10,036	10,419

212533_at	12,144	12,217	12,280	8,496	9,152	8,492
212534_at	11,019	11,363	10,778	9,094	8,965	8,419
212535_at	8,886	9,088	8,819	7,050	9,664	7,996
212536_at	9,494	10,243	9,285	9,356	10,227	9,003
212537_x_at	13,580	13,802	13,381	13,028	13,285	13,450
212538_at	7,640	7,971	7,257	6,844	8,035	6,114
212539_at	11,978	12,051	11,842	10,568	10,624	10,474
212540_at	9,662	9,181	9,408	9,634	8,736	9,355
212541_at	10,171	9,830	10,570	8,975	8,194	8,871
212542_s_at	10,011	10,150	9,545	6,801	8,272	6,102
212543_at	6,093	6,985	6,729	9,252	10,718	9,322
212544_at	10,464	10,898	10,279	9,593	9,111	9,871
212545_s_at	6,447	7,004	7,121	7,798	7,714	7,808
212546_s_at	6,521	6,803	6,278	5,258	6,988	5,532
212547_at	11,426	11,141	11,677	8,879	8,983	9,138
212548_s_at	8,162	8,547	7,974	7,199	8,936	7,057
212549_at	9,449	9,473	9,389	9,068	8,879	9,170
212550_at	7,591	7,632	7,558	7,383	7,131	7,554
212551_at	9,369	9,635	9,224	7,530	7,010	8,042
212552_at	8,666	8,794	9,086	11,256	11,378	11,486
212553_at	10,257	9,124	10,018	9,232	9,002	9,240
212554_at	10,375	10,721	10,265	7,365	8,139	8,313
212555_at	7,746	7,564	7,629	8,072	7,447	8,124
212556_at	9,156	9,075	9,553	9,519	9,485	9,675
212557_at	10,217	10,654	9,561	7,967	8,947	7,494
212558_at	9,470	9,634	9,865	6,354	6,963	7,244
212559_at	8,199	7,551	8,295	7,227	7,171	7,371
212560_at	9,721	10,070	9,969	4,758	4,797	4,713
212561_at	10,606	10,675	10,488	9,909	10,454	10,595
212562_s_at	7,775	7,761	7,720	8,031	8,022	7,941
212563_at	9,262	8,671	9,476	9,576	8,492	9,585
212564_at	8,722	8,697	8,757	8,296	8,339	8,508
212565_at	9,818	10,294	9,397	8,119	8,501	7,711
212566_at	11,521	11,886	12,069	11,160	12,053	12,003
212567_s_at	8,807	9,058	8,983	8,966	9,452	9,271
212568_s_at	10,161	10,022	10,024	9,595	9,259	9,150
212569_at	9,419	9,764	8,988	7,757	9,308	6,834
212570_at	7,190	7,429	7,473	8,929	8,625	9,526
212571_at	8,699	8,195	8,611	7,722	7,812	7,552
212572_at	10,920	11,115	10,512	7,152	8,600	7,233
212573_at	7,287	7,585	7,452	9,301	11,211	10,511
212574_x_at	8,060	7,223	7,652	7,845	7,390	8,038
212575_at	6,420	6,115	6,509	7,239	7,332	7,543
212576_at	9,470	9,177	9,665	8,878	8,883	9,062
212577_at	8,464	8,747	8,039	7,285	8,393	7,197
212578_x_at	13,993	14,156	14,128	13,405	13,453	13,658
212579_at	9,302	9,407	8,576	7,185	8,454	6,162
212580_at	5,392	5,978	5,383	5,004	5,074	4,709
212581_x_at	14,828	14,760	14,873	14,046	14,222	14,416
212582_at	11,112	11,267	10,841	9,542	11,154	8,479
212583_at	8,283	8,190	8,464	6,444	6,962	6,266
212584_at	9,813	10,035	9,416	8,321	8,787	7,841
212585_at	10,523	10,774	10,251	9,236	10,858	9,322
212586_at	9,618	10,044	9,150	10,839	11,692	10,681
212587_s_at	6,036	5,993	5,596	6,240	5,805	5,917
212588_at	3,892	4,026	3,849	3,783	3,731	3,611

212589_at	11,575	11,848	11,847	10,647	11,963	11,973
212590_at	9,964	9,955	10,151	10,557	11,376	11,792
212591_at	11,715	11,955	11,417	10,011	10,586	9,926
212592_at	4,172	4,619	4,137	4,333	4,289	4,448
212593_s_at	9,287	9,906	8,800	8,982	10,026	9,073
212594_at	6,661	7,309	6,094	6,388	7,425	6,458
212595_s_at	8,052	8,109	8,070	8,901	8,446	9,084
212596_s_at	9,580	9,713	9,448	7,814	8,386	7,863
212597_s_at	10,036	10,120	9,903	8,920	9,220	8,558
212598_at	7,517	7,193	7,414	7,372	7,967	7,046
212599_at	12,353	12,058	12,498	5,570	7,293	6,593
212600_s_at	12,780	13,027	12,290	12,022	12,164	12,075
212601_at	7,877	7,767	8,116	8,255	8,721	8,333
212602_at	8,595	8,490	8,882	8,153	9,152	8,106
212603_at	10,060	10,377	10,150	9,877	9,281	8,941
212604_at	9,541	10,060	9,653	9,328	8,352	8,544
212605_s_at	8,521	8,286	8,470	6,256	6,613	5,979
212606_at	8,886	8,888	8,758	6,284	8,293	6,513
212607_at	9,974	10,286	9,686	9,350	9,936	8,780
212608_s_at	10,514	10,216	10,547	8,887	9,716	9,057
212609_s_at	9,432	9,789	9,242	7,923	8,343	7,067
212610_at	12,878	12,881	12,716	12,004	13,031	11,940
212611_at	7,725	7,249	7,795	6,620	5,714	6,288
212612_at	9,930	10,371	9,731	8,592	9,661	9,055
212613_at	6,063	6,084	6,249	8,330	8,043	7,643
212614_at	7,537	7,947	7,265	8,928	11,438	10,219
212615_at	9,604	9,429	9,155	7,926	9,630	7,792
212616_at	10,369	10,341	9,844	9,088	10,522	8,587
212617_at	8,005	7,809	8,127	7,126	6,953	7,198
212618_at	7,726	7,299	7,470	6,408	6,735	6,684
212619_at	6,647	6,783	6,146	6,332	6,196	6,450
212620_at	9,044	8,783	9,120	7,850	7,882	8,185
212621_at	10,118	10,242	9,909	7,704	8,174	7,180
212622_at	9,622	9,747	9,881	9,778	10,386	10,053
212623_at	11,901	12,281	12,171	9,111	9,220	9,248
212624_s_at	12,422	12,160	12,059	10,025	10,551	9,535
212625_at	8,939	8,528	8,728	9,346	8,513	9,182
212626_x_at	12,481	12,384	12,226	11,631	11,418	11,696
212627_s_at	11,517	11,663	11,136	10,581	9,935	10,282
212628_at	10,329	10,498	9,995	8,692	10,081	8,326
212629_s_at	9,941	10,236	9,553	8,491	9,359	8,352
212630_at	9,115	8,998	9,314	9,845	9,867	9,978
212631_at	9,062	9,654	8,977	9,196	9,672	9,339
212632_at	8,705	9,101	8,401	7,815	8,780	8,292
212633_at	8,894	9,420	8,103	9,565	10,776	8,992
212634_at	7,050	7,720	6,513	7,329	8,074	6,929
212635_at	11,416	11,615	11,348	10,402	12,119	10,780
212636_at	9,094	9,005	8,932	7,918	9,203	8,412
212637_s_at	8,601	8,615	7,781	7,809	8,437	7,260
212638_s_at	9,525	9,709	8,864	8,390	9,391	7,955
212639_x_at	14,359	14,324	14,278	13,758	13,786	14,091
212640_at	10,714	10,415	10,302	10,054	9,474	9,757
212641_at	6,629	6,828	6,509	6,374	6,852	6,583
212642_s_at	7,532	7,898	7,568	8,263	8,764	8,355
212643_at	12,408	12,408	12,583	12,254	12,154	12,499
212644_s_at	10,012	9,718	9,908	9,557	9,389	9,930

212645_x_at	10,037	9,912	10,176	9,865	9,492	9,661
212646_at	6,094	7,184	6,741	9,751	9,584	9,906
212647_at	6,682	7,850	6,464	9,391	9,365	10,272
212648_at	10,414	10,597	10,228	9,739	10,963	8,833
212649_at	6,197	6,407	5,772	6,011	6,441	6,127
212650_at	9,064	8,986	8,475	7,739	7,468	7,300
212651_at	8,354	8,422	8,197	6,651	7,729	7,281
212652_s_at	10,288	10,565	10,139	7,842	9,076	8,792
212653_s_at	11,043	11,035	10,458	9,620	10,529	9,042
212654_at	8,312	8,565	8,430	8,717	8,040	9,000
212655_at	9,679	9,799	9,798	7,702	7,872	7,315
212656_at	9,828	9,861	10,002	10,061	9,128	9,744
212657_s_at	5,753	5,592	5,919	6,452	6,071	5,953
212658_at	9,172	9,145	9,178	8,729	10,053	8,472
212659_s_at	7,546	7,518	7,418	7,518	7,531	7,665
212660_at	9,566	9,361	10,415	8,686	9,140	9,269
212661_x_at	14,498	14,544	14,500	13,873	14,005	14,193
212662_at	7,562	7,248	7,490	8,810	7,903	8,699
212663_at	8,907	8,854	8,976	8,921	8,632	8,821
212664_at	7,435	7,475	7,227	6,635	6,614	6,532
212665_at	9,540	10,295	9,520	10,492	12,046	10,815
212666_at	9,181	9,055	9,102	7,769	8,930	8,272
212667_at	9,294	9,937	9,502	10,355	11,739	11,747
212668_at	6,203	6,085	6,271	7,173	6,658	7,325
212669_at	7,406	7,671	7,608	6,912	7,057	7,441
212670_at	7,798	7,812	8,055	8,418	8,339	8,435
212671_s_at	4,247	4,880	4,736	5,595	5,499	5,376
212672_at	8,901	8,786	8,635	7,432	9,230	7,243
212673_at	11,230	11,624	11,042	10,882	11,064	10,843
212674_s_at	10,399	10,292	10,505	9,792	8,697	9,511
212675_s_at	10,663	10,530	10,651	7,220	7,826	7,140
212676_at	9,512	9,703	9,512	8,372	10,197	8,451
212677_s_at	10,176	9,948	10,271	6,800	7,397	7,101
212678_at	8,073	7,845	7,414	6,251	7,387	6,558
212679_at	6,491	6,706	6,603	6,444	6,228	6,182
212680_x_at	10,493	10,318	10,604	10,773	10,139	10,681
212681_at	8,095	8,471	7,770	4,601	7,208	4,244
212682_s_at	8,446	8,254	8,743	9,008	8,737	9,358
212683_at	9,355	8,980	9,485	8,569	8,553	8,718
212684_at	10,158	9,783	9,922	7,435	7,942	7,698
212685_s_at	10,254	10,159	10,393	10,973	10,246	10,905
212686_at	10,058	10,233	10,496	6,218	6,991	7,305
212687_at	10,809	10,659	10,712	11,850	12,221	11,830
212688_at	9,895	10,727	10,159	8,761	9,789	8,621
212689_s_at	10,530	10,808	10,402	7,230	8,960	7,123
212690_at	10,022	10,538	9,441	7,101	8,862	7,520
212691_at	9,328	9,223	9,774	8,757	8,669	8,815
212692_s_at	9,719	10,082	9,496	8,204	9,635	7,983
212693_at	11,610	11,123	11,496	8,412	9,279	8,316
212694_s_at	10,350	10,646	10,331	10,159	9,724	10,386
212695_at	9,225	9,057	8,970	8,266	8,209	8,414
212696_s_at	12,041	11,961	12,181	10,896	11,143	11,288
212697_at	9,195	9,280	9,427	9,344	8,706	9,458
212698_s_at	12,197	12,370	11,854	11,096	12,062	11,334
212699_at	8,986	9,372	9,112	8,134	7,465	7,653
212700_x_at	6,375	6,303	6,598	6,987	7,167	7,277

212701_at	8,243	8,419	8,652	6,643	7,166	6,717
212702_s_at	9,188	8,896	9,124	9,294	9,251	9,236
212703_at	8,493	8,323	8,793	7,482	7,598	7,698
212704_at	10,554	10,707	9,996	7,874	8,870	7,621
212705_x_at	7,365	7,401	7,586	7,905	7,973	7,989
212706_at	5,521	6,270	5,539	4,918	5,190	5,070
212707_s_at	7,650	7,922	7,773	8,025	7,738	8,102
212708_at	9,186	9,066	9,246	8,654	8,186	8,512
212709_at	10,709	10,423	10,216	8,163	8,761	7,406
212710_at	10,127	9,927	10,171	8,684	8,784	8,449
212711_at	9,369	9,143	9,643	7,806	8,572	8,291
212712_at	8,941	8,419	9,468	7,979	8,149	8,138
212713_at	7,641	7,266	7,306	7,528	7,479	7,677
212714_at	10,985	11,102	10,553	9,438	10,406	9,122
212715_s_at	6,856	7,187	7,128	6,942	7,004	7,185
212716_s_at	11,011	11,112	11,400	11,728	11,088	11,890
212717_at	8,106	8,460	8,442	9,495	9,514	9,690
212718_at	11,674	11,719	11,392	10,595	11,357	10,017
212719_at	7,168	7,355	7,431	5,416	5,578	5,337
212720_at	10,496	10,279	10,084	9,143	9,099	8,406
212721_at	10,833	10,422	10,208	8,370	10,395	7,919
212722_s_at	8,168	8,077	8,094	8,988	8,470	9,137
212723_at	9,145	9,265	9,454	10,722	10,523	10,968
212724_at	10,016	10,531	9,827	12,278	12,450	11,804
212725_s_at	10,282	9,985	9,939	8,280	9,873	8,215
212726_at	8,443	8,027	8,360	7,394	7,458	7,100
212727_at	10,292	10,245	10,116	8,616	8,971	8,871
212728_at	8,912	8,993	8,675	7,618	7,762	7,383
212729_at	8,595	8,424	8,763	6,923	6,584	6,917
212730_at	9,512	9,231	9,427	7,845	8,359	8,183
212731_at	10,331	10,775	10,345	7,422	7,790	7,875
212732_at	6,045	5,691	5,827	7,196	7,702	7,334
212733_at	8,624	8,852	9,129	8,814	8,816	9,239
212734_x_at	12,740	13,082	12,829	12,678	12,330	13,001
212735_at	7,453	7,801	7,832	7,220	7,560	7,439
212736_at	8,684	8,681	8,525	8,234	8,592	8,312
212737_at	9,613	10,146	10,029	10,330	10,010	10,079
212738_at	10,801	11,463	10,719	8,885	8,970	8,446
212739_s_at	10,522	10,532	10,611	9,715	9,059	10,318
212740_at	10,222	10,551	10,372	9,254	9,597	8,854
212741_at	8,201	8,871	7,758	8,010	10,267	9,458
212742_at	9,207	8,870	9,203	8,983	8,888	9,531
212743_at	6,107	6,159	6,200	4,695	4,770	4,763
212744_at	10,475	10,797	10,220	8,733	8,529	8,603
212745_s_at	11,018	11,324	10,718	8,644	8,516	8,843
212746_s_at	10,021	10,149	9,530	7,960	9,168	8,040
212747_at	9,620	9,858	10,303	8,416	8,665	8,580
212748_at	8,150	8,096	8,264	8,097	8,027	8,429
212749_s_at	10,517	10,574	10,560	9,033	9,126	8,930
212750_at	7,410	8,444	8,182	6,618	6,276	6,553
212751_at	11,818	11,374	11,319	9,038	9,060	9,368
212752_at	9,835	9,938	10,117	8,284	9,582	8,811
212753_at	9,645	9,776	9,769	8,472	9,109	8,949
212754_s_at	9,187	9,547	8,793	8,264	9,670	8,380
212755_at	7,264	7,681	7,189	7,260	7,075	7,215
212756_s_at	8,545	8,895	8,569	8,391	8,934	8,148

212757_s_at	9,105	9,092	8,936	7,998	8,054	8,385
212758_s_at	8,370	8,545	7,719	9,380	9,983	9,429
212759_s_at	8,383	8,242	8,414	7,760	8,000	8,076
212760_at	8,821	9,238	8,848	7,321	8,675	7,391
212761_at	12,680	12,641	12,793	10,225	11,146	10,764
212762_s_at	9,787	9,336	9,804	6,928	7,439	6,825
212763_at	7,579	7,759	7,604	7,782	7,326	7,485
212764_at	8,236	8,784	7,720	9,257	10,447	9,365
212765_at	10,822	10,813	10,540	9,086	10,334	8,859
212766_s_at	11,885	11,408	12,114	10,550	10,129	10,364
212767_at	9,988	9,502	10,367	10,084	9,109	9,765
212768_s_at	3,656	3,596	3,883	3,990	4,297	4,428
212769_at	7,930	8,046	8,275	7,798	7,517	7,774
212770_at	8,180	8,111	8,280	7,535	7,380	7,420
212771_at	10,973	10,686	11,182	9,722	10,380	10,579
212772_s_at	6,758	6,700	6,654	6,846	6,827	7,189
212773_s_at	10,981	11,023	10,765	10,460	9,857	10,401
212774_at	11,326	11,141	10,609	8,023	8,670	8,396
212775_at	9,480	9,594	9,844	7,944	7,673	8,458
212776_s_at	9,223	9,208	9,130	7,420	7,123	8,104
212777_at	7,231	7,213	7,433	7,013	6,930	6,904
212778_at	8,114	8,218	8,064	7,662	7,542	7,761
212779_at	9,183	9,634	8,759	7,249	9,965	7,392
212780_at	8,569	8,825	8,850	7,263	8,410	7,225
212781_at	10,702	10,633	10,053	8,496	10,210	8,011
212782_x_at	8,963	8,781	9,118	9,497	8,537	9,241
212783_at	10,321	10,595	9,952	7,902	9,245	7,527
212784_at	7,800	7,605	7,853	7,718	7,720	7,982
212785_s_at	9,985	10,972	9,364	8,218	9,141	8,201
212786_at	7,665	7,531	7,608	6,797	6,619	6,803
212787_at	10,450	10,233	10,401	9,159	9,824	9,280
212788_x_at	13,307	13,616	13,585	14,041	14,061	14,304
212789_at	9,323	9,531	9,248	9,361	9,313	8,941
212790_x_at	14,160	14,162	14,090	13,404	13,291	13,726
212791_at	9,520	9,330	9,694	9,774	9,237	9,486
212792_at	8,964	8,892	8,698	7,690	8,610	8,125
212793_at	6,668	6,945	6,828	7,616	7,424	8,108
212794_s_at	10,199	10,486	10,047	8,711	9,777	8,414
212795_at	10,592	11,098	10,331	9,386	11,070	9,078
212796_s_at	9,241	9,015	9,205	9,007	9,075	8,841
212797_at	4,955	5,376	5,107	5,624	5,564	5,768
212798_s_at	10,439	11,000	10,014	9,036	9,998	8,679
212799_at	10,508	10,848	10,450	8,528	9,557	9,115
212800_at	7,523	7,999	7,801	6,459	6,356	6,624
212801_at	9,235	9,806	9,131	8,381	8,558	8,245
212802_s_at	9,647	10,016	9,900	8,945	9,893	9,056
212803_at	9,167	8,998	9,043	8,884	8,715	8,897
212804_s_at	8,985	9,225	9,199	8,437	8,827	8,194
212805_at	8,228	8,987	7,864	6,537	8,720	6,876
212806_at	6,355	6,733	5,987	5,761	6,772	5,777
212807_s_at	9,057	9,111	9,319	8,196	9,798	10,311
212808_at	8,946	8,579	8,553	7,166	7,246	7,447
212809_at	10,068	9,782	10,067	9,843	9,421	9,786
212810_s_at	8,497	8,486	8,882	6,996	7,929	7,944
212811_x_at	7,819	8,098	8,307	7,800	8,441	8,702
212812_at	9,433	10,005	9,959	7,957	8,858	8,548

212813_at	10,114	10,264	10,209	9,971	10,257	10,103
212814_at	9,281	9,431	9,540	7,318	7,855	7,553
212815_at	8,650	8,983	8,281	9,077	10,351	8,371
212816_s_at	10,877	10,236	10,444	7,886	6,946	8,131
212817_at	7,862	8,045	7,878	7,909	7,741	7,505
212818_s_at	9,406	9,313	9,543	8,436	8,372	8,401
212819_at	7,158	6,500	6,687	5,050	5,135	5,378
212820_at	10,315	10,473	9,353	7,320	9,504	7,165
212821_at	7,210	7,122	7,312	7,786	7,426	7,561
212822_at	8,807	8,364	8,529	9,006	9,652	9,197
212823_s_at	7,839	7,674	7,870	7,742	7,614	7,764
212824_at	10,977	10,839	10,915	10,015	10,418	10,197
212825_at	11,040	10,936	11,026	8,433	8,254	7,692
212826_s_at	13,121	13,361	13,133	13,042	12,398	13,045
212827_at	6,873	7,114	6,920	7,675	7,581	7,496
212828_at	8,253	8,314	8,386	8,800	9,227	7,856
212829_at	9,964	10,210	10,263	9,454	10,180	10,114
212830_at	6,322	6,818	6,178	6,534	7,397	6,712
212831_at	6,049	6,525	6,277	7,146	7,008	7,230
212832_s_at	11,957	12,264	11,599	9,948	10,598	9,714
212833_at	9,476	9,582	9,500	10,193	10,861	10,615
212834_at	8,986	8,874	9,653	8,501	9,090	8,056
212835_at	8,675	8,843	8,342	7,904	7,623	7,552
212836_at	10,742	10,487	10,169	9,084	8,860	8,395
212837_at	9,718	10,142	9,415	9,213	9,830	9,031
212838_at	9,118	9,350	9,435	8,159	9,184	8,829
212839_s_at	5,876	6,531	6,057	5,894	6,281	6,201
212840_at	9,455	9,429	9,029	8,264	8,600	8,242
212841_s_at	8,844	8,912	8,996	7,188	6,921	6,667
212842_x_at	11,331	11,703	10,952	8,952	10,418	8,626
212843_at	7,286	7,163	6,945	5,621	5,696	5,706
212844_at	7,354	7,495	7,881	7,757	7,829	7,991
212845_at	9,263	9,323	9,028	9,076	10,704	9,733
212846_at	12,812	12,613	13,046	10,931	11,194	10,760
212847_at	10,748	10,627	10,538	5,595	8,402	6,295
212848_s_at	8,833	8,864	8,857	8,511	8,862	9,071
212849_at	8,818	8,714	8,987	8,166	7,638	8,054
212850_s_at	10,064	10,406	10,550	7,512	7,269	7,158
212851_at	6,507	6,787	6,066	7,632	9,134	8,925
212852_s_at	12,102	12,033	11,935	10,436	12,144	11,030
212853_at	6,375	6,416	6,707	6,980	7,272	7,348
212854_x_at	10,972	10,943	11,061	10,727	11,433	10,681
212855_at	10,221	10,085	9,945	8,120	9,992	8,872
212856_at	7,501	7,777	7,274	7,110	6,867	7,452
212857_x_at	12,598	12,767	12,564	12,855	12,927	13,132
212858_at	9,151	8,934	8,985	8,792	8,625	8,679
212859_x_at	11,094	10,861	12,762	12,911	12,552	13,333
212860_at	8,882	8,858	9,287	8,514	8,287	8,661
212861_at	9,560	9,084	9,571	10,000	9,303	9,991
212862_at	8,171	8,224	8,325	8,345	7,630	8,041
212863_x_at	11,202	10,974	11,177	10,318	10,579	10,774
212864_at	9,445	9,636	10,178	9,236	8,726	8,945
212865_s_at	5,435	5,572	5,505	4,487	4,875	4,638
212866_at	8,277	7,925	8,523	8,273	7,354	8,062
212867_at	10,543	10,397	10,501	7,273	9,347	7,580
212868_x_at	7,174	7,501	7,471	7,795	7,502	7,798

212869_x_at	14,561	14,622	14,549	14,057	14,332	14,427
212870_at	8,144	8,290	8,066	7,517	8,696	7,541
212871_at	9,248	9,207	9,197	7,603	7,377	7,631
212872_s_at	10,660	10,784	10,932	9,062	8,863	9,303
212873_at	9,141	8,965	9,213	7,479	7,332	7,585
212874_at	6,559	6,314	6,938	6,628	6,528	6,640
212875_s_at	10,911	10,850	10,719	9,655	10,422	10,053
212876_at	9,362	10,001	9,253	9,150	9,275	9,173
212877_at	8,881	8,902	8,660	9,040	9,253	9,539
212878_s_at	8,939	8,811	8,647	8,418	8,576	8,849
212879_x_at	8,376	7,968	8,109	7,430	7,286	7,553
212880_at	8,367	8,697	8,543	7,799	8,595	7,819
212881_at	8,673	8,628	8,954	7,590	6,637	7,062
212882_at	9,660	9,659	10,207	9,467	9,698	9,700
212883_at	6,577	7,477	7,666	6,019	5,901	5,918
212884_x_at	9,956	10,154	9,861	9,422	9,719	9,609
212885_at	10,420	10,708	10,073	9,294	9,686	8,554
212886_at	7,015	7,014	7,083	6,464	6,539	6,417
212887_at	10,273	10,673	10,090	11,411	12,355	12,089
212888_at	9,604	9,485	9,180	7,309	8,611	7,153
212889_x_at	6,714	6,685	6,771	6,657	6,393	6,623
212890_at	9,200	8,847	9,144	9,603	9,577	10,224
212891_s_at	8,619	8,801	8,836	9,408	8,823	9,362
212892_at	8,717	8,562	8,884	8,320	7,763	8,308
212893_at	11,159	11,384	10,962	9,501	10,625	9,128
212894_at	9,652	9,578	9,727	8,781	8,619	8,435
212895_s_at	9,446	9,832	9,528	9,517	9,583	9,589
212896_at	10,953	11,255	10,578	9,292	9,977	8,699
212897_at	7,472	7,441	7,448	7,948	7,411	7,548
212898_at	10,555	10,361	10,483	9,018	8,978	8,983
212899_at	8,287	8,408	8,281	6,855	7,873	6,671
212900_at	9,822	10,291	9,850	9,724	10,807	10,183
212901_s_at	8,582	7,850	8,283	8,521	8,105	8,299
212902_at	7,041	7,388	6,982	7,682	8,780	8,336
212904_at	12,116	12,471	12,768	11,650	11,204	11,866
212905_at	8,874	8,629	8,697	7,958	8,730	8,200
212906_at	8,957	8,951	8,935	8,625	8,409	8,905
212907_at	11,664	11,435	10,961	9,149	10,639	10,254
212908_at	8,981	9,040	9,313	8,294	8,632	8,529
212909_at	6,853	6,444	7,219	6,680	6,203	6,524
212910_at	10,241	10,090	10,625	9,568	9,061	9,303
212911_at	7,032	6,692	7,285	6,500	6,827	6,556
212912_at	6,996	7,103	7,297	8,385	10,502	9,865
212913_at	8,496	8,319	8,060	6,319	7,051	6,223
212914_at	7,516	7,728	7,527	7,481	7,236	7,621
212915_at	7,836	7,972	7,783	9,168	8,399	8,048
212916_at	9,694	9,369	9,539	7,358	7,724	7,554
212917_x_at	11,230	11,525	10,526	9,820	11,421	9,648
212918_at	12,167	12,379	11,686	10,805	12,304	10,375
212919_at	11,925	12,014	11,604	7,978	8,658	7,177
212920_at	9,377	9,132	9,059	7,357	8,513	6,940
212921_at	6,632	6,733	6,993	5,594	5,581	5,479
212922_s_at	9,418	9,323	9,876	9,921	9,562	9,952
212923_s_at	7,762	8,288	8,029	10,497	10,711	10,917
212924_s_at	6,698	6,796	6,882	6,835	6,602	6,622
212925_at	8,167	7,970	8,166	8,686	8,674	8,639

212926_at	8,597	8,769	7,958	6,844	7,685	6,662
212927_at	8,839	9,104	8,447	7,736	8,842	6,985
212928_at	10,344	10,257	10,636	9,631	9,892	10,239
212929_s_at	9,433	9,599	9,723	9,569	10,356	9,604
212930_at	8,782	8,975	8,021	8,832	8,469	6,971
212931_at	9,475	9,141	9,190	8,989	9,173	9,086
212932_at	8,327	8,440	7,994	7,337	7,693	6,704
212933_x_at	13,163	13,586	13,521	13,291	13,048	13,406
212934_at	10,375	10,433	10,481	7,662	8,494	7,721
212935_at	8,711	8,326	8,679	7,621	7,488	7,655
212936_at	7,852	8,189	7,812	7,448	7,949	7,484
212937_s_at	6,713	6,756	6,448	7,634	7,724	7,839
212938_at	6,665	6,976	6,349	7,661	7,327	7,845
212939_at	5,637	5,963	6,023	6,885	6,669	6,984
212940_at	6,897	6,855	6,866	7,761	7,887	8,147
212942_s_at	5,748	5,990	6,003	6,028	7,304	7,683
212943_at	12,677	12,897	12,302	9,382	10,351	8,605
212944_at	10,046	9,938	9,553	10,616	12,362	9,542
212945_s_at	8,247	7,982	7,896	6,798	6,884	6,071
212946_at	8,985	9,468	9,384	7,573	9,152	7,897
212947_at	7,877	7,972	7,985	7,062	6,997	7,365
212948_at	6,713	7,179	6,731	7,311	7,471	7,759
212949_at	10,523	10,308	10,015	8,583	7,940	7,613
212950_at	4,330	4,203	4,141	4,842	4,741	4,908
212951_at	5,994	5,656	5,892	5,972	5,774	6,089
212952_at	10,866	11,228	11,849	11,553	11,947	11,883
212953_x_at	8,820	8,522	9,046	8,573	9,174	8,846
212954_at	9,274	9,325	9,105	9,018	8,447	8,926
212955_s_at	10,017	9,920	10,178	9,780	9,214	9,606
212956_at	8,154	7,980	7,683	8,770	9,855	8,857
212957_s_at	8,155	8,324	8,664	7,071	6,982	7,375
212958_x_at	10,328	10,151	9,865	10,546	11,995	11,124
212959_s_at	8,801	9,518	9,365	8,254	8,902	7,708
212960_at	6,960	6,817	6,927	8,051	8,014	7,810
212961_x_at	9,333	9,475	9,516	10,298	9,799	10,521
212962_at	6,458	6,445	6,624	6,596	6,335	6,631
212963_at	6,145	6,520	6,413	6,376	6,144	6,315
212964_at	10,428	10,140	10,657	7,174	7,979	7,647
212965_at	8,181	7,898	8,102	7,055	7,016	6,971
212966_at	9,509	8,863	9,038	6,227	6,362	6,445
212967_x_at	13,529	13,779	13,355	12,150	12,243	12,100
212968_at	7,615	7,849	8,103	8,669	8,240	9,161
212969_x_at	7,083	7,336	7,119	7,876	7,542	7,731
212970_at	8,924	8,878	8,801	8,121	9,377	8,759
212971_at	12,602	12,930	13,022	11,833	12,026	12,336
212972_x_at	6,512	6,415	6,736	6,537	6,871	6,856
212973_at	10,673	10,725	10,754	10,344	9,709	9,893
212974_at	5,840	5,736	5,750	6,443	6,438	6,529
212975_at	7,715	7,913	7,727	7,966	8,497	8,277
212976_at	8,070	7,861	7,647	5,368	6,028	5,244
212977_at	9,250	9,289	9,304	5,934	7,800	6,235
212978_at	9,847	9,799	9,466	7,870	9,142	8,109
212979_s_at	8,079	7,942	7,242	6,400	6,777	6,845
212980_at	8,027	7,451	7,210	5,597	6,529	5,716
212981_s_at	10,076	10,042	9,154	9,178	10,338	9,394
212982_at	10,914	11,120	10,286	8,010	9,465	7,469

212983_at	9,213	8,397	8,882	10,009	8,984	9,851
212984_at	10,181	10,490	9,523	7,904	8,415	7,408
212985_at	9,006	8,991	8,677	8,095	9,374	8,605
212986_s_at	9,498	9,432	9,286	8,098	8,067	8,057
212987_at	11,322	11,153	11,380	10,737	11,391	11,113
212988_x_at	14,368	14,382	14,471	14,034	14,362	14,403
212989_at	7,528	7,623	7,538	8,478	9,124	8,596
212990_at	8,415	8,727	8,261	7,391	8,938	7,545
212991_at	7,154	7,124	6,965	5,945	6,150	5,865
212992_at	5,921	6,313	5,936	7,888	9,821	8,243
212993_at	9,325	8,823	9,253	8,311	8,854	8,925
212994_at	8,626	8,777	8,210	7,508	7,928	7,250
212995_x_at	10,142	10,385	10,127	10,648	10,257	10,977
212996_s_at	9,034	8,766	9,798	7,587	7,949	7,527
212997_s_at	10,126	10,113	9,872	8,186	9,157	8,346
212998_x_at	5,820	5,796	5,685	6,387	6,098	6,310
212999_x_at	3,819	3,688	3,797	4,002	3,978	4,165
213000_at	10,430	10,794	9,954	8,629	9,617	8,021
213001_at	8,549	8,329	8,790	7,411	7,297	7,377
213002_at	8,230	8,651	8,778	8,047	8,614	8,971
213003_s_at	6,609	6,417	6,773	7,257	7,271	7,178
213004_at	7,605	7,530	8,019	7,904	7,695	7,684
213005_s_at	9,054	9,339	9,093	8,133	8,454	8,552
213006_at	4,655	4,776	4,420	4,866	6,848	6,118
213007_at	11,044	11,059	10,456	8,621	9,505	7,801
213008_at	10,482	10,488	9,651	7,768	8,064	7,198
213009_s_at	12,228	12,317	11,955	9,823	10,865	9,903
213010_at	8,302	8,840	9,370	10,088	9,771	10,298
213011_s_at	13,662	13,178	13,462	12,487	11,998	12,564
213012_at	7,403	7,762	6,996	9,717	10,079	8,799
213013_at	8,488	8,443	8,527	8,636	8,558	8,478
213014_at	6,699	6,241	7,193	7,440	7,349	7,258
213015_at	9,589	9,723	8,895	8,718	9,844	8,525
213016_at	9,516	9,411	8,613	8,087	9,021	7,620
213017_at	8,953	8,921	8,400	8,142	7,928	8,384
213018_at	8,732	8,896	8,250	7,043	7,242	7,161
213019_at	9,282	8,665	8,984	8,579	9,514	7,637
213020_at	9,183	9,212	9,217	8,239	8,679	8,299
213021_at	8,866	8,656	8,861	7,769	8,036	7,796
213022_s_at	7,455	7,417	7,592	7,810	7,870	7,747
213023_at	6,719	6,446	6,665	7,096	6,855	7,293
213024_at	8,075	8,502	7,657	7,453	8,857	6,596
213025_at	10,805	11,019	10,356	8,632	9,434	7,792
213026_at	10,389	10,365	10,035	9,921	10,784	10,146
213027_at	10,281	10,207	9,727	7,434	9,708	7,653
213028_at	10,069	10,125	10,348	9,277	9,339	9,360
213029_at	7,979	8,066	8,156	7,406	9,149	7,887
213030_s_at	7,142	6,847	7,301	7,727	7,999	8,434
213031_s_at	9,737	9,191	10,138	9,436	9,437	9,622
213032_at	6,024	5,450	5,431	4,892	6,917	5,682
213033_s_at	4,984	5,232	5,356	5,196	5,529	5,325
213034_at	9,024	8,881	9,562	8,049	8,725	8,236
213035_at	9,200	9,550	9,148	8,310	9,762	8,129
213036_x_at	7,425	7,436	7,450	7,654	7,625	7,805
213037_x_at	12,958	13,088	13,313	12,027	12,456	12,264
213038_at	6,926	6,838	6,433	6,860	6,543	6,908

213039_at	10,199	10,154	10,664	9,656	9,777	10,279
213040_s_at	6,905	6,796	6,721	6,779	6,786	6,999
213041_s_at	9,965	9,950	10,366	10,472	9,625	10,298
213042_s_at	5,159	5,576	4,708	5,116	4,959	4,910
213043_s_at	9,057	8,879	9,264	8,742	8,135	8,523
213044_at	11,486	11,703	10,962	10,380	12,130	10,251
213045_at	8,327	8,769	8,689	8,321	8,322	8,526
213046_at	8,952	8,557	8,678	6,712	7,126	7,381
213047_x_at	13,295	13,270	13,249	11,002	11,284	11,014
213048_s_at	11,416	11,740	11,622	10,189	10,927	10,514
213049_at	9,143	9,310	8,983	7,037	9,137	6,722
213050_at	9,454	10,079	10,255	6,446	6,414	6,471
213051_at	10,141	10,255	10,313	9,171	10,405	9,364
213052_at	9,073	9,373	9,276	9,142	9,485	9,488
213053_at	8,409	8,474	8,470	8,562	8,331	8,344
213054_at	7,259	6,780	7,349	6,218	5,898	6,124
213055_at	7,374	7,130	7,190	7,019	7,147	7,059
213056_at	6,665	7,619	6,248	3,853	4,127	3,824
213057_at	8,219	8,120	8,329	8,618	8,880	9,085
213058_at	8,746	8,575	8,950	6,918	8,413	7,127
213059_at	7,903	7,875	7,771	8,526	8,494	9,153
213060_s_at	6,176	7,002	6,555	7,059	6,590	6,803
213061_s_at	9,447	9,930	9,687	9,893	9,196	10,114
213062_at	10,104	10,660	10,281	10,366	10,020	10,471
213063_at	9,239	9,407	9,159	8,137	8,766	7,818
213064_at	9,807	9,560	9,381	8,446	9,020	7,802
213065_at	9,689	9,812	9,387	7,698	9,858	7,742
213066_at	7,968	8,108	8,236	8,405	8,435	8,785
213067_at	10,325	10,017	10,022	6,572	6,831	6,863
213068_at	5,103	5,376	5,156	6,195	5,662	6,018
213069_at	10,211	10,137	10,469	9,418	11,159	9,815
213070_at	10,478	10,457	10,052	8,778	10,559	8,371
213071_at	5,550	5,320	5,671	5,907	5,758	6,180
213072_at	7,242	6,964	7,185	7,431	7,526	7,875
213073_at	8,347	8,260	8,673	7,728	8,142	7,746
213074_at	10,992	11,247	10,617	7,985	9,741	7,506
213075_at	9,238	9,205	9,019	7,692	7,764	7,653
213076_at	7,472	7,308	7,353	7,661	7,494	7,871
213077_at	9,731	9,694	9,080	8,070	9,631	7,873
213078_x_at	7,275	7,273	7,504	7,788	7,536	7,935
213079_at	10,079	9,548	10,107	9,613	9,253	9,528
213080_x_at	14,651	14,679	14,641	13,963	14,186	14,197
213081_at	8,060	8,120	8,052	8,340	8,592	8,710
213082_s_at	8,201	8,505	8,045	8,758	7,938	8,276
213083_at	9,252	9,842	9,604	9,836	9,300	9,580
213084_x_at	13,952	14,091	13,972	13,814	14,027	14,124
213085_s_at	9,333	9,355	9,929	8,060	7,440	7,647
213086_s_at	11,520	11,402	11,353	10,645	11,377	11,139
213087_s_at	6,766	7,988	7,949	7,798	7,703	7,150
213088_s_at	11,068	11,014	10,974	11,086	10,515	10,397
213089_at	10,322	9,780	9,519	8,392	9,019	8,374
213090_s_at	10,276	10,466	10,907	8,099	8,553	8,137
213091_at	8,350	8,190	8,516	8,382	8,135	8,334
213092_x_at	10,038	10,108	9,899	9,573	9,814	8,753
213093_at	8,851	9,278	9,535	10,076	11,074	10,266
213094_at	5,230	5,895	5,424	4,898	5,012	5,080

213095_x_at	7,867	7,680	7,930	7,535	7,229	7,404
213096_at	8,253	8,139	8,352	8,541	8,631	8,523
213097_s_at	11,453	11,648	11,328	9,556	11,034	8,813
213098_at	9,012	8,818	8,916	8,838	8,142	8,619
213099_at	9,308	9,194	9,269	8,621	8,540	8,614
213100_at	8,791	8,721	9,053	8,354	8,192	8,188
213101_s_at	13,606	13,604	13,479	13,234	13,918	13,678
213102_at	11,425	11,280	11,239	11,882	12,626	12,452
213103_at	8,113	8,359	8,083	8,971	9,868	9,551
213104_at	8,657	8,489	8,616	9,606	8,096	9,497
213105_s_at	9,360	9,042	9,292	10,333	9,751	10,372
213106_at	8,360	9,094	7,661	5,565	5,435	5,431
213107_at	9,119	8,855	8,681	7,175	7,176	7,282
213108_at	7,328	7,251	7,506	7,957	7,638	7,817
213109_at	6,161	6,285	5,964	6,036	5,695	5,672
213110_s_at	10,981	10,879	10,507	7,231	8,154	5,504
213111_at	10,594	10,929	9,959	7,614	9,425	7,286
213112_s_at	8,140	8,364	8,209	10,787	9,928	10,347
213113_s_at	8,904	8,449	8,758	10,822	8,761	9,252
213114_at	6,746	6,588	6,705	7,066	7,228	6,915
213115_at	8,111	8,333	8,149	8,199	8,170	7,946
213116_at	10,263	10,151	9,691	8,042	8,335	7,993
213117_at	8,748	8,346	8,399	8,712	9,360	9,199
213118_at	9,589	10,066	9,285	8,837	10,246	8,338
213119_at	10,060	9,930	10,313	9,758	9,850	9,906
213120_at	5,502	5,179	5,317	4,901	4,730	4,626
213121_at	7,293	6,796	7,013	7,176	7,310	7,181
213122_at	6,390	6,345	6,540	7,965	8,601	9,301
213123_at	9,642	9,530	9,444	8,300	9,392	8,595
213124_at	8,496	8,530	8,802	7,196	6,796	6,370
213125_at	6,724	6,916	6,619	7,642	6,640	6,534
213126_at	7,745	7,580	7,555	7,615	7,515	8,234
213127_s_at	8,777	8,920	9,031	9,187	9,096	9,639
213128_s_at	9,543	9,401	9,238	8,757	9,458	8,334
213129_s_at	12,552	12,724	12,409	11,692	11,520	11,818
213130_at	7,835	7,797	8,129	7,497	7,205	7,239
213131_at	11,225	11,305	11,463	5,292	5,437	5,345
213132_s_at	8,656	8,176	8,550	9,515	8,661	9,198
213133_s_at	11,542	11,834	11,582	10,616	9,763	10,226
213134_x_at	10,998	11,454	10,519	10,578	10,092	10,069
213135_at	9,526	9,934	9,960	6,353	8,582	7,430
213136_at	9,796	10,198	9,914	9,425	9,546	8,916
213137_s_at	8,832	9,306	9,125	8,863	8,583	8,766
213138_at	7,983	8,175	8,436	7,569	7,900	7,865
213139_at	5,641	6,092	6,058	11,896	12,472	12,251
213140_s_at	9,979	9,940	9,380	7,036	8,163	7,639
213141_at	8,644	8,477	8,552	8,020	7,820	7,994
213142_x_at	7,295	7,421	6,625	8,161	8,024	7,016
213143_at	7,233	7,418	7,350	7,046	7,206	7,196
213144_at	8,350	7,868	8,436	8,325	7,823	8,432
213145_at	11,140	11,067	11,341	9,144	9,586	8,994
213146_at	6,621	6,933	6,943	6,478	6,364	6,440
213147_at	8,043	8,006	7,906	9,232	9,428	9,684
213148_at	6,981	7,379	7,450	6,664	6,914	6,978
213149_at	9,180	9,285	8,998	8,872	9,148	8,712
213150_at	5,306	5,740	5,793	8,255	8,451	8,793

213151_s_at	13,208	13,450	13,046	11,693	12,788	12,048
213152_s_at	8,376	8,657	8,556	9,319	9,209	9,646
213153_at	10,976	10,348	11,306	9,055	9,768	9,611
213154_s_at	10,326	10,241	10,352	10,673	10,569	10,715
213155_at	9,975	10,123	9,894	6,473	6,735	6,464
213156_at	4,301	4,902	4,045	4,640	8,100	5,644
213157_s_at	8,529	8,595	8,844	8,546	8,464	8,545
213158_at	4,620	5,369	4,377	5,467	7,826	5,866
213159_at	7,892	7,912	7,709	7,187	7,668	7,339
213160_at	6,074	6,492	6,536	6,940	6,761	6,706
213161_at	10,585	10,611	10,486	7,643	8,151	7,479
213162_at	5,808	5,970	6,029	6,112	6,053	6,249
213164_at	10,440	9,992	9,736	10,067	12,176	9,450
213165_at	10,865	10,886	10,560	9,267	10,523	8,117
213166_x_at	10,046	10,245	10,235	10,347	9,943	10,676
213167_s_at	5,581	5,510	5,512	5,680	5,758	5,408
213168_at	11,345	11,290	10,947	9,665	9,842	8,971
213169_at	7,830	8,018	7,697	8,279	9,021	8,131
213170_at	9,083	9,330	9,273	8,093	8,371	8,005
213171_s_at	7,927	8,160	8,557	7,058	6,957	6,963
213172_at	7,471	6,764	6,671	3,807	3,764	3,888
213173_at	6,566	6,370	6,449	5,885	7,057	6,447
213174_at	7,244	7,036	7,172	7,133	6,942	7,120
213175_s_at	13,623	13,301	13,496	12,558	12,086	12,576
213176_s_at	7,722	7,557	7,447	8,071	8,191	8,366
213177_at	7,795	7,895	8,257	8,609	8,591	8,370
213178_s_at	8,136	8,143	8,343	8,100	8,256	8,105
213179_at	10,275	10,224	10,661	9,091	9,067	9,276
213180_s_at	7,565	7,712	7,613	8,118	7,859	8,272
213181_s_at	7,327	7,387	7,350	7,839	7,294	7,641
213182_x_at	8,599	8,645	8,465	6,738	6,859	6,674
213183_s_at	7,961	8,935	8,632	6,540	5,581	5,947
213184_at	9,494	9,674	9,698	9,239	9,407	8,917
213185_at	8,262	8,060	8,740	7,422	7,528	7,700
213186_at	10,317	10,639	9,582	6,915	7,971	6,536
213187_x_at	12,633	12,698	12,623	13,626	13,302	13,944
213188_s_at	9,502	9,565	9,638	9,963	10,258	9,784
213189_at	8,651	8,674	8,568	9,211	8,805	9,071
213190_at	9,000	9,384	9,105	8,668	8,274	8,557
213191_at	6,720	6,728	6,959	7,835	7,636	7,998
213192_at	7,974	7,888	7,990	8,640	8,275	8,458
213193_x_at	6,964	7,041	7,055	7,341	7,326	7,122
213194_at	11,684	11,530	11,310	9,717	10,756	9,072
213195_at	7,841	8,350	8,049	8,099	8,239	8,054
213196_at	10,039	9,434	10,092	8,777	8,785	8,903
213197_at	7,378	6,084	6,894	4,787	4,360	4,857
213198_at	10,196	10,094	10,607	8,672	8,777	8,863
213199_at	8,094	8,013	8,252	7,299	7,778	7,280
213200_at	7,465	7,280	7,606	8,426	7,952	8,017
213201_s_at	9,201	9,418	9,292	6,629	6,335	6,402
213202_at	7,199	7,609	7,796	7,391	7,186	7,103
213203_at	10,693	11,003	10,343	10,002	9,792	9,684
213204_at	7,395	7,025	7,060	7,689	7,734	7,666
213205_s_at	8,895	8,829	9,136	7,941	8,278	8,368
213206_at	6,594	6,548	7,516	7,330	7,088	7,385
213207_s_at	7,675	7,128	7,795	8,450	7,671	7,891

213208_at	8,469	8,138	8,598	6,066	6,543	5,982
213209_at	7,156	6,812	6,863	7,047	6,531	6,898
213210_at	7,542	7,354	7,439	7,758	6,986	7,351
213211_s_at	7,170	7,054	6,872	7,527	7,017	7,267
213212_x_at	9,084	8,998	8,376	4,777	7,757	5,567
213213_at	8,874	8,446	9,098	7,531	8,382	7,528
213214_x_at	14,254	14,303	14,371	13,965	14,279	14,359
213215_at	9,359	9,701	9,605	8,029	8,179	8,173
213216_at	9,237	9,136	8,658	6,770	7,168	6,669
213217_at	8,714	8,995	9,124	7,328	6,905	7,150
213218_at	8,162	8,270	8,167	7,551	7,442	7,184
213219_at	5,840	5,287	5,849	5,930	6,124	6,116
213220_at	9,312	9,995	9,163	10,408	10,045	10,555
213221_s_at	7,849	7,648	7,874	7,447	8,140	7,737
213222_at	10,406	10,837	9,912	5,459	10,639	8,271
213223_at	10,128	10,325	10,562	9,048	8,715	9,323
213224_s_at	8,033	8,513	7,968	7,511	7,559	7,365
213225_at	10,730	10,956	10,426	7,960	9,059	8,503
213226_at	10,461	10,536	10,506	9,516	9,612	9,163
213227_at	10,104	10,143	9,890	9,314	9,853	9,499
213228_at	6,042	6,318	6,047	5,935	6,244	5,964
213229_at	11,107	10,589	10,540	8,760	10,292	8,691
213230_at	8,486	8,430	8,679	9,457	9,122	9,504
213231_at	6,577	6,495	6,681	6,689	6,240	6,744
213232_at	5,756	6,159	6,057	6,462	5,979	6,249
213233_s_at	10,345	10,274	10,262	10,152	10,975	10,488
213234_at	10,117	10,454	10,454	8,717	8,186	8,268
213235_at	10,206	10,040	10,506	9,288	8,911	9,256
213236_at	8,919	9,308	8,782	8,059	9,448	8,467
213237_at	10,192	10,281	10,432	7,985	7,034	7,151
213238_at	6,889	7,042	6,852	8,669	10,014	8,952
213239_at	7,278	8,224	6,977	6,229	7,312	6,122
213240_s_at	6,166	6,113	6,318	6,960	6,311	6,066
213241_at	9,006	9,112	8,501	5,803	6,062	5,517
213242_x_at	9,051	8,756	9,253	9,679	10,279	10,002
213243_at	7,930	7,945	7,790	6,698	9,092	7,248
213244_at	7,939	7,868	8,091	8,176	7,720	8,064
213245_at	9,501	9,666	9,634	6,442	6,033	6,185
213246_at	9,024	9,213	8,643	9,410	7,981	8,828
213247_at	8,114	8,405	8,114	8,340	9,324	8,907
213248_at	6,858	7,263	6,679	6,930	8,254	8,083
213249_at	8,983	8,854	8,910	8,015	8,531	8,274
213250_at	7,485	7,614	7,642	7,392	7,512	7,397
213251_at	11,486	11,249	10,874	8,266	9,445	7,852
213252_at	6,698	6,375	6,694	7,366	7,435	7,581
213253_at	9,327	9,410	8,771	7,469	7,969	6,738
213254_at	7,975	8,063	7,998	8,204	8,151	7,911
213256_at	8,706	9,126	9,746	7,322	7,508	7,405
213257_at	7,163	6,972	7,261	7,189	6,612	6,772
213258_at	6,974	7,436	6,289	10,469	12,744	11,442
213259_s_at	7,791	7,290	7,964	8,329	8,129	7,988
213260_at	8,288	8,499	8,367	9,642	9,260	9,319
213261_at	7,862	8,161	7,611	7,816	7,808	8,038
213262_at	11,334	10,855	10,820	10,595	12,011	9,843
213263_s_at	9,577	9,356	9,963	8,405	8,355	8,413
213264_at	9,842	9,764	10,358	8,882	8,818	8,892

213265_at	6,624	6,773	6,965	7,057	7,195	7,047
213266_at	9,872	9,628	9,679	8,136	8,064	7,944
213267_at	5,146	4,940	4,947	4,859	5,345	4,804
213268_at	8,681	8,882	7,896	5,840	5,561	5,712
213269_at	8,604	8,971	8,159	7,956	8,221	8,114
213270_at	7,904	7,542	7,765	7,340	7,225	7,327
213271_s_at	7,781	7,998	7,655	7,576	8,203	7,252
213272_s_at	7,823	7,561	7,400	7,540	7,239	7,392
213273_at	8,263	7,538	8,453	8,056	7,786	8,111
213274_s_at	7,257	7,532	7,797	9,518	9,481	9,849
213275_x_at	7,876	8,245	8,623	10,507	11,150	10,910
213276_at	6,569	6,351	6,298	6,785	6,833	6,962
213277_at	6,353	5,826	6,185	6,611	6,000	5,920
213278_at	9,733	9,662	9,434	7,741	8,633	7,382
213279_at	8,009	8,114	8,097	9,454	8,695	9,785
213280_at	9,591	9,935	10,200	9,370	8,246	7,898
213281_at	7,342	7,887	7,860	8,044	8,159	7,493
213282_at	9,621	9,933	9,236	8,634	9,852	8,566
213283_s_at	12,598	12,451	13,060	7,919	7,878	7,953
213284_at	5,736	5,675	5,878	6,172	6,009	6,006
213285_at	6,034	7,104	6,359	5,527	5,757	5,441
213286_at	8,947	9,179	8,876	8,418	8,123	8,344
213287_s_at	10,904	10,811	11,101	12,261	12,409	12,722
213288_at	8,674	8,807	8,696	8,238	8,130	8,217
213289_at	8,080	8,323	7,391	6,969	7,406	6,648
213290_at	6,928	7,147	7,316	9,323	9,172	9,463
213291_s_at	9,742	9,632	9,625	9,095	9,857	8,888
213292_s_at	8,878	9,277	8,523	7,865	8,965	7,667
213293_s_at	9,337	9,251	9,011	8,637	9,923	9,304
213294_at	10,086	10,161	9,801	8,352	9,426	7,632
213295_at	7,423	7,718	7,060	7,098	8,193	7,286
213296_at	8,319	8,212	8,542	8,745	8,731	8,987
213297_at	7,578	7,592	7,723	7,864	7,657	7,783
213298_at	6,537	6,774	6,982	8,001	8,235	8,339
213299_at	6,465	6,432	6,827	6,946	6,864	7,222
213300_at	8,233	8,418	8,741	8,824	8,888	8,898
213301_x_at	11,469	11,799	11,223	8,041	8,557	7,714
213302_at	10,315	9,872	11,072	9,904	8,784	9,296
213303_x_at	5,619	5,697	6,262	7,701	7,969	7,598
213304_at	8,530	8,535	8,293	7,271	8,762	6,904
213305_s_at	8,441	8,709	7,969	8,217	7,724	8,042
213306_at	10,914	10,955	10,226	8,542	9,613	7,820
213307_at	6,724	6,801	7,441	5,007	5,366	5,170
213308_at	7,813	7,719	7,754	5,338	6,166	6,053
213309_at	9,690	9,900	9,594	7,775	8,195	7,597
213310_at	8,884	8,983	9,346	8,365	9,077	8,391
213311_s_at	9,355	8,985	9,093	9,597	9,162	10,152
213312_at	7,843	8,396	7,508	6,822	6,176	6,713
213313_at	10,874	11,162	10,752	8,698	9,989	8,675
213314_at	6,281	6,462	6,351	5,910	5,960	5,809
213315_x_at	9,170	9,259	9,196	10,152	9,603	10,274
213316_at	5,921	6,180	6,659	6,414	6,460	6,474
213317_at	3,800	3,975	4,653	3,859	3,894	4,019
213318_s_at	10,725	10,637	10,996	10,117	9,649	10,292
213319_s_at	7,108	6,979	7,152	7,412	6,963	7,166
213320_at	10,222	10,402	10,034	9,364	9,800	9,173

213321_at	8,889	9,140	8,682	7,773	8,354	8,167
213322_at	11,184	11,565	10,976	9,487	8,933	9,049
213323_s_at	7,721	7,576	8,150	8,134	8,074	8,143
213324_at	8,196	7,897	8,016	8,068	7,526	7,814
213325_at	9,286	9,459	9,045	8,681	10,065	9,977
213326_at	9,939	10,269	10,390	7,899	8,413	8,870
213327_s_at	9,480	9,474	9,208	9,433	10,039	8,802
213328_at	8,431	8,590	7,920	7,879	8,340	7,564
213329_at	9,124	9,136	9,254	7,350	7,214	7,318
213330_s_at	9,941	9,664	9,808	9,646	9,235	9,472
213331_s_at	8,812	9,063	8,254	7,899	8,934	7,395
213332_at	7,173	7,697	7,057	7,314	7,562	7,294
213333_at	9,709	9,549	9,970	8,422	8,391	8,760
213334_x_at	8,424	8,149	8,161	8,473	7,320	7,981
213335_s_at	5,158	5,142	5,366	5,722	5,558	5,969
213336_at	8,004	7,827	7,258	7,305	6,974	6,990
213337_s_at	7,184	7,186	7,199	7,144	7,184	7,240
213338_at	9,938	9,967	10,548	10,598	9,783	10,483
213339_at	6,934	7,284	7,106	6,805	6,898	7,031
213340_s_at	9,478	9,785	9,785	8,435	8,510	8,216
213341_at	8,311	7,803	7,967	7,900	8,902	7,850
213342_at	8,676	8,704	9,186	9,981	10,192	9,991
213343_s_at	8,124	8,058	7,872	7,886	7,735	7,852
213344_s_at	7,444	7,236	7,488	7,858	7,601	7,594
213345_at	8,573	8,414	8,090	8,598	8,402	8,755
213346_at	8,393	9,025	8,303	9,307	8,649	8,761
213347_x_at	14,668	14,650	14,671	14,165	14,109	14,221
213348_at	9,590	10,390	10,145	5,509	5,269	5,029
213349_at	8,308	8,462	8,821	7,716	7,990	7,659
213350_at	9,018	9,764	10,078	9,299	9,717	9,346
213351_s_at	8,717	8,856	8,894	5,174	6,828	5,872
213352_at	7,917	7,896	8,003	4,824	5,015	4,750
213353_at	6,222	6,042	6,363	6,540	7,634	6,496
213354_s_at	5,548	5,643	5,569	5,700	5,694	5,600
213355_at	8,890	8,612	8,216	5,620	5,274	5,681
213356_x_at	14,243	14,197	14,115	13,194	13,675	13,283
213357_at	10,578	10,863	10,105	10,839	10,651	11,035
213358_at	10,224	9,994	10,451	8,105	9,000	8,222
213359_at	9,393	10,423	10,126	6,417	9,479	7,211
213360_s_at	10,893	10,420	11,073	10,299	10,459	10,522
213361_at	8,381	8,944	7,758	8,131	8,563	8,089
213362_at	6,911	6,624	6,677	4,689	4,724	4,696
213363_at	8,321	7,987	8,067	8,068	7,813	8,104
213364_s_at	9,709	9,464	8,816	6,276	7,678	6,691
213365_at	9,742	9,738	9,625	8,542	8,960	8,078
213366_x_at	12,608	13,029	12,758	12,752	12,854	13,006
213367_at	8,856	8,791	8,731	8,841	8,970	8,882
213368_x_at	7,525	7,572	7,701	7,215	7,092	7,198
213369_at	6,732	6,979	6,900	6,804	6,822	6,863
213370_s_at	9,109	8,983	8,891	8,327	7,897	7,899
213371_at	5,633	6,000	5,679	6,016	5,663	5,848
213372_at	10,923	11,064	10,181	9,528	10,050	9,097
213373_s_at	8,212	8,591	8,593	9,758	9,710	9,643
213374_x_at	9,320	9,279	8,980	9,205	9,731	8,661
213375_s_at	7,370	8,987	7,392	4,927	4,841	4,464
213376_at	9,361	9,415	9,039	7,684	8,770	7,800

213377_x_at	14,214	14,404	14,268	13,685	13,622	13,949
213378_s_at	8,955	8,456	8,740	7,492	6,854	7,424
213379_at	9,076	9,497	9,277	9,578	8,448	9,213
213380_x_at	7,043	7,182	6,688	7,508	7,429	7,639
213381_at	6,754	6,910	6,592	7,720	7,450	7,757
213382_at	4,060	3,846	4,284	4,676	4,572	4,841
213383_at	7,162	7,083	7,474	7,116	6,560	6,940
213384_x_at	8,582	8,444	8,630	8,786	9,073	9,142
213385_at	6,906	7,421	6,647	5,517	5,295	5,651
213386_at	7,757	7,583	7,737	6,850	6,618	7,137
213387_at	9,670	9,498	9,119	5,701	6,999	5,878
213388_at	8,700	8,978	8,491	7,056	8,868	6,728
213389_at	7,900	7,788	8,349	7,936	8,206	8,043
213390_at	11,296	10,688	11,779	9,544	10,315	9,897
213391_at	9,409	9,844	9,209	8,201	9,379	8,522
213392_at	8,868	8,984	8,591	8,267	8,197	7,955
213393_at	7,626	7,686	7,589	6,605	6,923	6,765
213394_at	7,480	7,329	7,476	8,176	8,347	8,615
213395_at	6,569	6,407	6,227	6,439	5,825	6,336
213396_s_at	8,536	8,632	8,143	7,498	8,215	7,657
213397_x_at	4,718	6,423	5,160	7,432	7,973	8,891
213398_s_at	9,186	9,575	9,507	9,660	9,275	9,813
213399_x_at	13,056	13,148	13,380	12,513	12,851	12,997
213400_s_at	11,514	11,397	11,744	9,379	9,628	9,175
213401_s_at	6,707	6,757	6,776	6,232	6,421	6,249
213402_at	8,133	8,050	7,791	8,046	7,777	8,191
213403_at	8,617	8,292	8,339	7,160	6,838	7,236
213404_s_at	12,258	12,430	12,225	11,724	11,796	12,039
213405_at	8,675	8,550	8,511	8,064	9,029	7,802
213406_at	5,555	5,247	5,390	5,405	5,285	5,577
213407_at	9,719	9,473	9,326	8,091	8,555	7,920
213408_s_at	9,705	9,832	9,807	9,057	9,106	9,365
213409_s_at	6,006	6,218	5,951	5,351	5,645	5,578
213410_at	11,189	11,124	10,819	8,683	9,568	8,140
213411_at	8,187	8,466	7,627	5,985	5,852	5,980
213412_at	7,752	8,019	7,900	7,707	7,535	7,312
213413_at	9,052	9,602	8,763	6,180	7,271	6,986
213414_s_at	14,268	14,368	14,273	13,587	13,472	13,870
213415_at	4,364	4,677	4,375	4,327	4,201	4,099
213416_at	7,556	7,507	6,579	8,528	9,024	7,923
213417_at	7,100	6,909	6,926	8,395	8,350	8,470
213418_at	6,606	6,720	6,741	7,355	7,240	7,497
213419_at	8,242	8,542	8,377	7,921	8,578	8,555
213420_at	8,548	8,459	8,125	7,747	7,783	7,704
213421_x_at	7,439	7,450	7,561	10,455	9,604	9,428
213422_s_at	9,156	8,915	9,174	9,978	10,701	11,494
213423_x_at	11,079	10,939	10,806	10,975	11,244	11,455
213424_at	5,115	5,697	4,637	4,807	4,929	4,457
213425_at	9,396	9,246	9,101	10,166	10,875	10,180
213426_s_at	6,510	5,914	6,226	6,383	6,552	6,587
213427_at	9,675	9,996	10,035	10,680	10,317	10,563
213428_s_at	9,782	9,969	9,734	12,469	12,841	12,859
213429_at	5,529	5,776	5,370	8,064	8,779	7,133
213430_at	5,839	6,008	5,921	5,733	5,686	5,478
213431_x_at	8,101	8,170	8,171	8,530	8,637	8,654
213432_at	6,443	6,630	6,598	7,035	6,889	7,156

213433_at	7,798	7,690	7,635	7,471	7,532	7,313
213434_at	10,040	10,168	9,855	9,379	9,997	9,372
213435_at	8,448	8,602	8,653	7,615	8,411	7,639
213436_at	5,109	5,373	5,617	4,859	7,480	6,160
213437_at	8,086	8,145	7,763	7,584	8,478	7,833
213438_at	6,417	6,490	6,474	5,633	6,014	6,556
213439_x_at	7,262	7,179	7,255	7,993	7,979	8,109
213440_at	10,336	10,518	10,123	9,621	10,320	9,524
213441_x_at	8,073	8,107	8,336	8,801	8,892	8,999
213442_x_at	8,763	8,536	8,753	8,999	8,766	8,856
213443_at	6,036	6,435	6,415	6,319	6,228	6,286
213444_at	9,069	8,997	9,112	8,830	8,981	9,035
213445_at	7,410	7,146	7,137	7,452	7,186	7,417
213446_s_at	10,168	10,415	9,695	10,184	10,917	10,262
213447_at	12,014	11,907	12,034	8,738	9,276	8,831
213448_at	8,140	7,749	8,491	8,082	7,899	8,334
213449_at	9,116	9,113	9,285	8,154	7,874	7,819
213450_s_at	8,575	8,476	8,585	8,707	8,618	8,704
213451_x_at	7,709	7,866	7,992	8,281	8,548	8,333
213452_at	8,755	9,262	8,596	6,808	6,820	6,134
213453_x_at	14,784	14,754	14,811	13,839	14,060	14,236
213454_at	9,743	9,664	9,897	9,978	9,135	9,279
213455_at	8,741	9,374	8,797	10,529	11,701	11,559
213456_at	5,457	5,427	5,089	4,431	4,598	4,510
213457_at	9,011	8,811	9,200	7,138	7,407	7,258
213458_at	4,646	4,319	4,834	4,660	4,636	4,886
213459_at	9,701	9,262	9,253	5,410	6,821	5,267
213460_x_at	10,164	10,009	10,102	8,669	8,781	9,056
213461_at	9,113	8,858	8,906	7,547	7,312	7,450
213462_at	6,713	6,851	7,020	7,948	8,468	8,195
213463_s_at	8,003	8,169	7,747	6,874	7,555	7,611
213464_at	8,127	8,411	8,195	7,839	7,542	7,476
213465_s_at	10,372	10,372	10,399	10,889	10,631	10,951
213466_at	8,249	8,364	8,435	8,553	8,682	8,746
213467_at	8,845	8,776	8,568	7,271	7,211	7,531
213468_at	7,442	7,757	7,571	8,436	8,029	8,619
213469_at	9,340	9,550	8,567	5,447	6,719	5,452
213470_s_at	7,636	6,689	6,588	5,805	5,576	5,833
213471_at	7,286	7,481	7,599	7,417	7,189	7,343
213472_at	4,611	4,195	4,181	4,407	4,351	4,121
213473_at	9,224	9,229	8,935	6,829	7,152	6,818
213474_at	8,436	8,330	8,117	7,661	7,878	7,733
213475_s_at	6,443	6,253	6,462	6,745	6,633	6,705
213476_x_at	12,117	11,713	11,783	12,169	11,461	12,201
213477_x_at	14,757	14,770	14,759	14,214	14,563	14,550
213478_at	9,545	9,530	9,787	8,827	9,462	9,813
213479_at	10,523	10,100	9,734	6,762	6,905	5,947
213480_at	8,991	9,495	8,817	8,914	8,892	8,792
213481_at	7,936	8,181	8,429	7,930	7,474	7,641
213482_at	7,092	7,282	7,175	6,645	6,335	6,392
213483_at	9,308	9,665	8,984	7,875	8,613	7,096
213484_at	10,425	9,656	10,755	7,085	6,748	7,115
213485_s_at	8,602	8,666	9,089	9,330	9,404	10,059
213486_at	6,397	5,598	6,559	6,403	6,302	6,577
213487_at	6,428	6,769	6,592	6,598	6,840	6,886
213488_at	7,400	7,281	7,412	7,724	7,685	7,933

213489_at	8,443	8,311	8,305	7,705	8,073	7,421
213490_s_at	7,107	7,160	6,898	7,091	6,431	6,811
213491_x_at	12,876	13,055	13,168	12,244	12,658	12,745
213492_at	7,113	6,433	6,356	6,236	6,292	6,003
213493_at	5,609	5,565	5,508	6,579	6,591	6,685
213494_s_at	8,202	8,345	8,287	7,547	7,534	7,841
213495_s_at	5,293	5,511	5,238	5,376	5,160	5,320
213496_at	4,534	5,035	4,758	4,421	4,682	4,446
213497_at	8,523	8,446	8,729	7,664	7,458	7,727
213498_at	6,336	5,893	6,292	6,769	6,496	6,611
213499_at	7,813	7,546	7,475	7,779	7,766	7,857
213500_at	9,171	9,371	8,949	7,666	8,590	7,531
213501_at	7,409	8,033	7,605	8,434	8,464	8,349
213502_x_at	7,864	7,913	7,941	8,071	8,036	8,309
213503_x_at	12,026	12,374	12,717	14,040	14,468	14,424
213504_at	10,218	9,964	10,620	10,908	10,072	10,712
213505_s_at	11,141	11,456	11,089	9,674	10,338	9,973
213506_at	9,768	10,642	10,097	6,375	7,564	6,831
213507_s_at	12,572	12,534	12,548	11,578	12,386	11,545
213508_at	9,253	9,569	9,374	8,127	8,030	7,878
213509_x_at	8,659	8,600	8,955	8,790	8,927	8,703
213510_x_at	8,773	8,920	8,351	6,416	8,872	6,467
213511_s_at	9,429	9,439	9,505	9,839	10,338	9,737
213512_at	7,643	7,196	7,557	7,993	7,903	7,878
213513_x_at	12,107	11,980	12,168	12,209	12,366	12,498
213514_s_at	8,381	8,358	8,834	8,459	8,126	8,447
213515_x_at	6,026	5,787	6,719	6,503	6,349	6,779
213516_at	6,492	6,109	5,693	6,665	6,309	6,376
213517_at	9,828	9,220	9,399	7,214	8,477	8,114
213518_at	10,845	10,949	10,355	7,635	8,650	7,269
213519_s_at	7,209	7,362	6,717	7,388	7,504	7,616
213520_at	8,832	8,620	9,096	8,645	8,278	8,578
213521_at	8,549	8,501	8,394	9,593	8,925	9,349
213522_s_at	6,140	5,845	6,116	6,510	6,388	6,344
213523_at	10,761	10,753	10,915	9,915	8,699	8,989
213524_s_at	7,286	7,800	7,694	9,489	7,557	8,145
213525_at	7,297	7,302	7,477	7,662	7,753	8,071
213526_s_at	6,653	6,572	6,831	8,029	6,900	8,061
213527_s_at	7,180	7,572	7,668	7,791	7,516	7,898
213528_at	9,803	9,816	9,357	7,332	7,337	7,417
213529_at	7,479	7,612	7,433	8,455	8,181	8,186
213530_at	9,162	9,037	8,856	7,419	8,427	7,410
213531_s_at	11,664	11,984	11,443	10,696	11,503	10,706
213532_at	10,101	10,528	10,185	8,745	9,633	8,669
213533_at	7,483	7,532	7,789	7,479	7,349	7,344
213534_s_at	9,215	8,960	8,842	6,904	6,266	6,303
213535_s_at	11,664	11,381	11,620	10,773	10,357	10,518
213536_s_at	6,839	6,655	7,163	6,989	7,000	6,699
213537_at	5,507	5,422	5,900	5,870	6,118	5,786
213538_at	9,807	10,109	10,209	8,272	9,462	8,728
213539_at	7,615	7,249	7,937	8,144	7,992	7,978
213540_at	8,351	8,432	7,991	8,150	8,326	8,275
213541_s_at	5,294	5,238	5,105	5,423	5,493	5,088
213542_at	8,552	8,377	8,554	8,966	8,959	8,911
213543_at	6,113	6,084	6,439	6,618	6,130	6,203
213544_at	6,161	6,559	6,352	6,368	6,076	5,872

213545_x_at	10,236	10,944	10,674	11,789	12,106	12,312
213546_at	9,337	9,774	9,321	6,490	7,939	6,418
213547_at	9,930	10,412	10,081	5,707	6,887	7,381
213548_s_at	6,345	6,832	6,478	6,768	6,730	6,916
213549_at	8,110	8,253	8,111	7,083	7,932	6,526
213550_s_at	6,857	7,434	7,522	6,675	6,619	6,629
213551_x_at	9,167	9,176	9,419	8,677	8,201	8,465
213552_at	7,329	7,649	6,504	6,685	7,269	6,263
213553_x_at	8,225	8,258	8,526	7,500	7,634	7,652
213554_s_at	9,167	9,520	9,459	9,963	9,387	9,959
213555_at	7,964	8,549	7,794	7,411	7,312	7,636
213556_at	7,155	7,636	7,493	8,293	7,961	7,731
213557_at	7,361	7,415	7,174	7,010	6,705	6,697
213558_at	6,112	5,853	6,120	5,439	5,728	5,547
213559_s_at	7,644	7,557	7,584	7,942	7,462	7,755
213560_at	6,533	7,088	7,064	7,645	7,726	7,874
213561_at	6,294	6,585	6,374	6,834	6,781	6,877
213562_s_at	8,306	8,328	7,671	7,006	6,729	7,003
213563_s_at	7,573	7,462	7,446	8,786	8,662	8,900
213564_x_at	14,947	14,952	14,856	13,684	13,967	13,903
213565_s_at	7,733	7,764	7,700	7,854	7,799	7,760
213566_at	5,156	4,767	4,979	5,544	5,435	5,722
213567_at	10,218	10,210	10,252	8,034	8,851	7,498
213568_at	4,311	4,624	4,226	7,791	8,258	7,325
213569_at	6,681	6,903	6,630	6,817	6,652	6,615
213570_at	7,142	7,168	7,435	7,549	7,378	7,353
213571_s_at	11,704	11,967	11,989	11,525	11,264	11,792
213572_s_at	6,391	7,477	6,642	7,115	6,902	7,169
213573_at	10,795	10,608	10,550	9,761	10,884	9,807
213574_s_at	11,923	12,051	11,897	11,271	11,897	11,313
213575_at	7,782	7,392	7,452	6,723	7,026	7,040
213577_at	10,025	9,914	9,844	9,968	9,972	10,490
213578_at	11,203	11,057	11,151	8,389	9,261	8,515
213579_s_at	8,528	8,695	8,596	7,050	8,425	7,817
213580_at	3,671	3,818	3,879	3,791	3,761	3,720
213581_at	9,727	9,732	9,846	10,132	9,525	9,745
213582_at	5,092	5,307	5,382	5,317	5,250	5,303
213583_x_at	14,767	14,704	14,698	14,093	14,452	14,446
213584_s_at	6,913	6,577	6,744	7,018	6,987	6,882
213585_s_at	6,989	7,160	6,981	7,614	7,362	7,503
213586_at	6,090	6,310	6,039	6,547	6,550	6,940
213587_s_at	8,698	8,685	8,755	9,141	8,500	8,685
213588_x_at	14,177	14,340	14,320	14,083	14,079	14,160
213589_s_at	7,499	7,576	7,385	8,488	7,737	8,109
213590_at	8,083	8,408	8,109	7,596	7,466	7,701
213591_at	8,564	8,881	8,313	6,638	7,050	6,804
213592_at	8,112	7,165	7,081	7,250	7,141	7,118
213593_s_at	9,278	9,228	8,824	6,014	8,376	7,607
213594_x_at	10,031	9,793	9,642	8,826	9,000	8,816
213595_s_at	7,981	7,919	8,107	8,115	8,640	8,196
213596_at	4,991	4,942	5,478	6,088	6,855	6,226
213597_s_at	5,285	5,071	5,242	5,066	5,167	5,200
213598_at	8,381	8,765	8,427	7,759	8,106	7,560
213599_at	10,485	10,598	10,305	8,871	7,828	8,072
213600_at	6,897	6,925	7,562	7,153	7,215	7,125
213601_at	7,530	7,717	7,842	7,725	7,614	7,640

213602_s_at	7,000	6,887	7,124	7,898	7,949	7,949
213603_s_at	8,281	8,559	8,737	11,823	11,360	11,914
213604_at	9,281	9,134	9,287	9,452	9,448	9,472
213605_s_at	9,782	9,380	9,158	6,397	8,515	7,063
213606_s_at	5,586	4,524	5,552	6,055	6,111	6,474
213607_x_at	8,155	8,126	7,743	9,046	8,597	8,756
213608_s_at	8,917	8,559	8,799	9,452	8,515	9,042
213609_s_at	6,522	6,495	6,508	6,791	6,317	6,549
213610_s_at	9,852	9,780	9,529	5,869	5,648	5,376
213611_at	6,697	6,782	6,698	7,282	6,713	6,952
213612_x_at	11,512	11,530	11,420	11,367	12,276	11,364
213613_s_at	6,542	6,630	6,576	7,117	7,207	7,361
213614_x_at	14,629	14,733	14,595	14,089	14,441	14,460
213615_at	10,127	9,837	10,734	7,691	7,884	7,623
213616_at	8,167	8,842	8,433	9,481	8,848	9,234
213617_s_at	9,304	9,410	9,165	9,518	8,396	9,420
213618_at	4,625	4,748	4,625	4,501	4,858	4,241
213619_at	12,247	12,594	12,739	11,715	12,619	11,511
213620_s_at	6,033	6,238	6,096	9,042	8,364	8,132
213621_s_at	6,841	7,114	7,151	7,723	8,295	7,850
213622_at	6,488	6,352	6,244	6,556	6,085	6,300
213623_at	7,633	8,162	6,902	6,357	7,248	5,911
213624_at	6,876	7,255	6,400	7,969	8,738	8,454
213625_at	8,731	8,705	8,831	7,778	7,329	7,048
213626_at	9,299	10,079	8,972	7,268	8,199	7,357
213627_at	9,949	10,352	10,309	10,089	10,274	10,666
213628_at	7,870	8,074	8,036	7,666	8,319	7,980
213629_x_at	10,695	10,572	11,287	11,642	10,925	11,774
213630_at	8,376	8,495	7,696	8,194	7,876	8,097
213631_x_at	6,473	6,081	7,013	6,687	6,227	6,234
213632_at	8,985	9,030	8,987	8,550	8,017	8,449
213633_at	7,084	7,205	7,383	7,492	7,297	7,303
213634_s_at	8,641	8,439	9,012	8,795	8,299	8,641
213635_s_at	7,089	6,539	6,861	6,754	6,958	6,845
213636_at	7,167	7,477	7,187	7,589	7,589	7,802
213637_at	8,675	8,263	8,889	7,416	8,423	7,164
213638_at	6,918	7,361	6,658	6,513	6,374	6,368
213639_s_at	7,775	7,410	7,607	7,295	7,285	7,495
213640_s_at	6,164	5,917	6,056	6,748	6,764	6,935
213641_at	7,398	7,017	7,215	7,193	7,102	7,221
213642_at	8,605	8,674	9,010	8,044	9,253	8,811
213643_s_at	8,050	8,358	7,481	6,404	6,063	6,328
213644_at	5,983	6,588	6,142	6,511	7,024	6,630
213645_at	8,172	8,074	7,994	7,454	8,584	7,567
213646_x_at	14,341	14,256	14,208	13,831	13,801	14,085
213647_at	10,270	10,020	9,615	6,902	7,409	6,102
213648_at	9,408	8,864	9,447	8,790	8,411	8,519
213649_at	10,299	10,576	10,039	7,383	9,237	8,255
213650_at	9,367	9,025	8,874	8,637	8,737	8,514
213651_at	7,985	7,692	7,720	7,666	7,571	7,809
213652_at	7,229	7,181	6,666	5,930	7,120	6,922
213653_at	9,878	9,490	9,268	7,380	8,839	7,922
213654_at	9,538	9,132	9,623	8,981	8,824	9,088
213655_at	13,047	13,431	13,446	12,698	12,956	12,981
213656_s_at	11,135	11,056	11,056	10,710	11,370	11,241
213657_s_at	9,287	9,494	9,212	7,958	8,201	8,383

213658_at	7,904	8,320	7,932	6,880	6,879	6,802
213659_at	6,906	7,399	7,120	5,955	6,442	5,732
213660_s_at	7,886	7,609	7,616	7,702	7,669	8,011
213661_at	8,855	9,091	8,717	8,660	9,717	9,378
213662_at	6,258	6,311	6,042	6,649	6,547	6,644
213663_s_at	4,006	4,090	4,595	4,044	3,997	4,045
213664_at	8,018	8,473	8,116	9,629	11,308	10,713
213665_at	6,321	6,116	6,567	6,175	6,245	6,417
213666_at	8,451	9,278	8,573	6,799	6,792	6,722
213667_at	6,980	6,882	7,262	7,055	6,895	6,839
213668_s_at	9,141	9,040	9,228	6,987	6,992	7,508
213669_at	7,788	7,914	7,610	8,280	8,380	8,297
213670_x_at	9,816	9,630	9,837	8,510	8,591	8,772
213671_s_at	11,891	11,901	11,866	10,612	10,322	10,853
213672_at	7,947	7,635	7,877	6,197	6,189	6,156
213673_x_at	7,563	7,427	7,568	7,795	7,678	7,906
213674_x_at	6,418	5,749	6,019	6,894	7,001	7,089
213675_at	7,141	6,672	7,388	7,197	8,216	7,658
213676_at	8,506	8,604	8,874	8,030	7,700	7,772
213677_s_at	11,105	11,283	10,595	8,118	9,346	7,325
213678_at	6,528	6,963	7,083	6,998	6,819	6,969
213679_at	6,405	6,016	6,367	5,517	5,378	5,671
213680_at	5,203	5,546	5,480	5,451	5,461	5,327
213681_at	7,869	7,771	7,771	7,925	7,823	8,016
213682_at	11,124	11,073	11,019	9,740	10,486	9,201
213683_at	5,192	5,286	5,434	6,187	5,433	5,608
213684_s_at	6,194	5,837	6,122	5,957	8,816	7,234
213685_at	7,832	8,144	7,918	7,658	7,510	7,651
213686_at	9,574	9,917	9,117	6,789	8,366	6,859
213687_s_at	12,978	13,158	12,827	12,217	12,048	12,570
213688_at	6,563	6,474	6,757	6,603	6,427	6,627
213689_x_at	8,602	9,053	8,393	8,617	9,780	9,011
213690_s_at	7,938	7,855	7,885	8,272	8,567	8,393
213691_at	8,220	7,866	8,161	8,588	8,719	8,701
213692_s_at	6,858	7,404	7,578	7,870	7,782	7,789
213693_s_at	8,243	8,242	8,302	8,484	8,261	8,091
213694_at	10,316	10,554	9,929	6,805	6,736	6,297
213695_at	6,654	6,343	6,173	6,687	6,751	6,620
213696_s_at	9,628	10,048	9,893	10,511	9,783	10,428
213697_at	5,277	5,041	5,257	5,550	5,279	5,470
213698_at	9,094	9,567	9,401	9,555	8,727	9,567
213699_s_at	14,102	13,909	14,064	13,376	13,796	13,820
213700_s_at	8,264	7,586	7,783	7,611	8,069	7,531
213701_at	9,542	10,029	9,381	8,697	9,425	9,293
213702_x_at	11,205	11,878	11,122	11,161	12,085	11,200
213703_at	6,416	5,645	5,169	6,668	7,501	6,722
213704_at	10,875	11,168	10,509	8,966	9,505	9,434
213705_at	6,042	6,261	6,661	5,909	6,776	6,103
213706_at	7,071	6,791	7,065	7,554	7,558	7,729
213707_s_at	6,309	5,319	6,038	6,735	6,343	6,470
213708_s_at	9,520	9,589	10,075	11,039	10,477	10,953
213709_at	7,515	7,572	7,235	6,116	6,347	6,174
213710_s_at	7,965	7,910	8,126	8,281	8,374	8,303
213711_at	7,210	6,930	7,225	7,072	7,273	7,445
213712_at	6,533	6,461	6,801	5,398	4,934	5,042
213713_s_at	7,568	7,539	7,629	7,251	6,939	7,172

213714_at	6,264	6,255	6,141	4,825	4,600	4,874
213715_s_at	6,628	7,062	6,771	7,657	7,605	7,879
213716_s_at	7,106	6,700	7,329	8,805	8,135	8,638
213717_at	7,580	7,701	7,635	8,276	7,889	7,944
213718_at	9,274	9,152	9,066	7,398	7,948	7,213
213719_s_at	6,833	5,763	6,661	6,446	6,471	6,628
213720_s_at	12,365	12,018	12,566	10,379	10,535	10,612
213721_at	11,898	11,646	11,059	7,613	7,725	7,824
213722_at	8,688	8,332	8,182	7,143	7,169	7,121
213723_s_at	7,128	6,910	7,000	8,207	7,855	8,201
213724_s_at	7,550	6,956	6,949	7,694	7,657	7,769
213725_x_at	9,612	9,685	10,011	10,259	11,319	11,106
213726_x_at	12,327	12,155	12,273	12,774	12,009	12,781
213727_x_at	8,588	8,651	8,761	9,359	9,825	9,947
213728_at	8,834	8,663	9,432	9,851	10,144	9,868
213729_at	11,604	11,721	11,119	9,326	9,994	8,751
213730_x_at	8,646	8,888	9,095	8,453	8,556	8,880
213731_s_at	7,528	7,387	7,466	8,107	8,292	8,212
213732_at	7,569	7,232	7,587	7,610	7,728	7,494
213733_at	7,166	6,939	7,124	7,293	6,879	7,117
213734_at	9,740	9,804	9,908	10,414	10,463	10,639
213735_s_at	11,695	12,114	12,089	11,637	11,117	11,800
213736_at	7,502	7,776	7,788	7,679	7,625	7,814
213737_x_at	9,673	10,206	9,160	7,143	9,567	7,961
213738_s_at	14,339	14,538	14,443	13,635	14,024	13,956
213739_at	7,190	7,041	7,059	7,053	6,801	6,865
213740_s_at	6,744	6,906	7,068	7,587	7,572	7,636
213741_s_at	8,605	8,555	8,474	8,588	8,598	8,597
213742_at	9,786	9,785	9,306	8,330	8,486	8,075
213743_at	10,320	10,523	9,974	6,709	7,791	6,480
213744_at	6,036	6,209	6,136	6,584	6,108	6,069
213745_at	4,712	4,781	4,661	4,027	4,776	4,151
213746_s_at	8,495	7,794	8,363	7,959	8,099	8,760
213747_at	8,700	9,279	9,336	8,541	9,128	8,366
213748_at	7,127	6,649	7,228	6,326	6,474	6,358
213749_at	7,299	7,553	7,632	7,965	8,169	8,391
213750_at	10,389	10,372	10,644	7,829	9,299	8,493
213751_at	6,628	6,787	6,644	7,159	6,934	7,487
213752_at	6,548	6,315	6,650	7,185	7,334	7,606
213753_x_at	8,999	9,035	9,416	9,637	10,334	10,413
213754_s_at	9,557	9,582	9,059	9,872	10,032	10,001
213755_s_at	7,896	7,728	7,798	8,432	8,575	8,742
213756_s_at	5,861	5,758	6,068	6,466	6,384	6,810
213757_at	12,093	12,343	12,656	12,433	11,313	11,379
213758_at	8,561	8,766	8,669	7,606	7,984	7,448
213759_at	6,976	7,124	7,302	7,157	6,888	6,869
213760_s_at	8,467	8,224	8,138	9,284	8,775	9,037
213761_at	8,893	8,990	8,236	5,760	7,211	5,609
213762_x_at	11,986	12,160	12,329	11,401	11,362	11,341
213763_at	6,598	6,583	6,546	7,385	7,704	7,937
213764_s_at	4,536	4,747	4,399	4,293	4,209	4,397
213765_at	4,628	5,202	4,762	4,677	4,914	4,657
213766_x_at	9,109	9,143	9,641	10,256	10,030	10,456
213767_at	7,108	6,705	7,222	7,614	7,631	7,918
213768_s_at	6,952	6,804	7,017	7,085	6,967	7,061
213769_at	6,869	6,644	6,997	7,678	7,812	7,927

213770_at	7,363	7,249	7,515	7,884	7,307	7,563
213771_at	7,876	7,792	7,874	8,531	8,638	8,754
213772_s_at	9,618	9,464	9,405	7,637	7,638	7,943
213773_x_at	10,818	10,562	10,914	9,805	9,895	10,042
213774_s_at	5,414	5,223	5,489	5,665	5,462	5,656
213775_x_at	11,145	11,387	10,699	8,541	9,779	8,055
213776_at	7,378	7,419	7,309	7,909	7,783	8,003
213777_s_at	6,381	6,070	6,368	6,153	5,976	6,358
213778_x_at	7,095	7,012	7,345	7,885	7,691	8,208
213779_at	7,673	7,640	7,805	8,083	7,887	8,010
213780_at	5,905	5,428	6,382	5,122	5,257	5,166
213781_at	5,970	5,565	6,091	6,538	6,236	6,454
213782_s_at	4,866	4,633	5,096	5,178	5,262	5,473
213783_at	6,617	6,421	6,405	6,747	6,416	6,655
213784_at	6,838	6,864	6,929	6,307	6,491	6,393
213785_at	9,605	9,210	8,751	6,982	7,628	7,325
213786_at	9,550	9,595	8,705	7,298	8,737	6,926
213787_s_at	11,167	10,987	10,994	10,699	10,291	10,472
213788_s_at	9,464	9,497	9,732	8,103	8,031	7,990
213789_at	8,034	8,778	9,067	8,712	8,499	8,429
213790_at	4,727	5,072	4,762	8,080	9,737	8,240
213791_at	7,292	8,048	7,972	9,784	9,380	10,910
213792_s_at	10,613	11,029	11,110	7,522	7,591	7,292
213793_s_at	10,450	10,334	10,350	7,922	8,285	7,089
213794_s_at	10,133	10,240	10,213	9,714	9,064	9,321
213795_s_at	9,060	8,891	9,032	7,712	7,831	7,794
213796_at	5,030	5,184	5,441	5,740	5,503	5,749
213797_at	5,961	6,287	5,925	6,832	6,598	6,708
213798_s_at	13,001	13,113	13,067	12,850	13,059	13,291
213799_s_at	7,372	7,532	7,398	7,443	7,390	7,790
213800_at	5,358	6,064	5,636	6,848	8,756	8,513
213801_x_at	14,746	14,875	14,775	14,058	14,126	14,291
213802_at	6,169	6,536	6,180	5,628	7,697	7,377
213803_at	10,896	10,592	10,661	10,227	10,906	10,334
213804_at	8,320	8,411	8,078	7,941	7,572	7,367
213805_at	7,182	7,216	7,425	6,340	6,297	5,754
213806_at	6,467	6,435	6,704	6,767	6,737	6,956
213807_x_at	6,092	6,103	6,067	6,146	6,361	6,454
213808_at	9,104	8,343	8,205	5,423	5,621	5,527
213809_x_at	7,254	7,503	7,275	7,167	6,964	6,969
213810_s_at	6,364	6,343	6,334	6,525	6,529	6,475
213811_x_at	11,167	11,083	11,642	10,496	10,588	10,947
213812_s_at	10,367	10,254	10,369	11,266	11,313	11,423
213813_x_at	8,159	8,570	8,453	7,347	8,870	8,143
213814_s_at	5,615	5,359	5,350	5,501	5,329	5,433
213815_x_at	7,649	7,432	7,495	7,821	7,837	8,157
213816_s_at	5,300	4,907	5,342	6,674	6,176	6,577
213817_at	5,334	4,552	4,729	5,606	6,140	4,694
213818_x_at	6,511	6,706	6,659	7,267	7,026	7,154
213819_s_at	7,638	7,832	7,702	8,073	7,936	8,384
213820_s_at	6,976	7,067	7,257	7,615	7,923	7,838
213821_s_at	6,559	6,373	6,178	6,977	7,056	6,818
213822_s_at	8,094	8,748	8,484	9,600	9,012	9,415
213823_at	7,074	7,019	6,977	8,805	8,067	8,314
213824_at	5,823	5,741	6,294	6,152	6,029	6,472
213825_at	8,946	9,464	8,729	6,650	6,122	6,091

213826_s_at	8,242	8,386	8,303	6,821	6,894	6,912
213827_at	8,156	8,031	8,156	7,978	8,216	8,243
213828_x_at	13,659	13,905	13,883	11,912	11,801	11,954
213829_x_at	8,365	8,348	8,492	8,557	8,453	8,647
213830_at	6,812	6,430	6,641	7,111	7,232	7,141
213831_at	5,310	5,236	4,512	5,340	5,312	5,284
213832_at	6,072	6,215	6,171	6,499	6,216	6,367
213833_x_at	5,682	5,884	6,016	5,557	5,583	5,716
213834_at	7,012	7,083	7,026	7,199	7,431	7,502
213835_x_at	9,915	9,897	10,276	9,988	9,460	9,516
213836_s_at	7,513	8,769	7,185	10,938	10,646	11,066
213837_at	7,308	7,680	7,191	6,720	6,765	6,828
213838_at	9,571	9,509	9,485	9,896	10,116	9,828
213839_at	7,631	8,047	7,190	6,816	7,140	6,940
213840_s_at	7,248	7,168	7,067	7,997	7,851	8,016
213841_at	5,688	5,594	5,993	6,178	6,068	6,206
213842_x_at	10,515	10,261	10,438	8,797	8,980	9,232
213843_x_at	8,404	7,649	9,279	7,721	7,540	7,788
213844_at	5,465	6,301	6,598	6,861	7,414	8,734
213845_at	5,518	5,879	5,717	6,289	6,715	6,334
213846_at	9,235	9,653	9,322	9,278	8,515	9,165
213847_at	8,324	8,460	8,333	8,789	8,337	8,688
213848_at	8,926	8,905	9,508	9,339	8,720	9,188
213849_s_at	9,249	8,977	9,768	5,815	5,657	5,914
213850_s_at	10,829	10,828	10,445	9,197	10,298	9,001
213851_at	7,961	7,860	7,900	8,688	8,474	8,788
213852_at	11,475	11,416	11,925	8,887	9,012	8,780
213853_at	9,034	9,406	8,937	8,859	9,352	9,238
213854_at	7,252	7,331	7,216	7,667	7,196	7,417
213855_s_at	6,663	6,349	6,439	6,768	6,826	6,949
213856_at	5,099	5,315	4,226	4,307	4,535	4,228
213857_s_at	10,670	10,997	10,500	12,294	12,049	12,187
213858_at	6,931	7,176	7,098	6,991	6,679	6,975
213859_x_at	9,403	9,452	8,887	7,332	8,191	6,877
213860_x_at	12,119	11,940	11,965	11,148	11,721	11,650
213861_s_at	7,725	7,923	7,915	7,941	7,588	7,764
213862_at	6,158	6,027	6,020	6,319	6,063	6,504
213863_s_at	5,843	6,082	6,388	6,210	6,021	6,157
213864_s_at	13,641	13,839	13,373	12,117	12,317	12,124
213865_at	7,483	7,232	7,220	9,032	9,118	9,810
213866_at	7,034	7,005	6,915	7,411	7,284	7,494
213867_x_at	14,417	14,206	14,310	13,537	13,797	14,029
213868_s_at	5,012	4,841	4,979	6,016	5,980	6,138
213869_x_at	9,619	9,419	10,121	9,532	8,998	10,086
213870_at	7,394	7,421	7,346	7,329	7,163	7,443
213871_s_at	7,717	7,623	7,729	8,723	8,000	8,311
213872_at	11,431	11,589	11,467	10,934	12,190	11,429
213873_at	5,664	6,055	5,919	6,381	6,706	6,807
213874_at	6,846	6,616	7,078	7,085	7,234	7,172
213875_x_at	7,503	7,661	7,557	7,283	7,724	7,572
213876_x_at	9,152	9,264	8,960	8,688	8,575	8,526
213877_x_at	9,113	8,787	9,194	9,193	9,065	9,405
213878_at	9,184	9,673	9,078	8,113	9,527	8,267
213879_at	8,749	9,101	9,366	9,464	9,603	9,587
213880_at	4,516	4,149	3,971	3,981	4,133	3,857
213881_x_at	13,366	13,346	13,397	12,602	12,708	12,916

213882_at	6,943	6,985	6,704	4,912	5,842	4,776
213883_s_at	10,526	11,222	10,282	11,030	10,992	11,333
213884_s_at	6,150	6,132	6,101	6,327	6,065	6,242
213885_at	6,888	6,879	7,299	8,186	8,111	8,263
213887_s_at	9,383	8,890	9,270	9,568	8,820	9,604
213888_s_at	6,039	5,828	5,684	6,356	6,288	6,235
213889_at	7,803	8,002	7,595	8,059	7,357	8,191
213890_x_at	14,474	14,567	14,515	13,945	14,018	14,254
213891_s_at	9,546	9,561	9,219	6,322	7,827	6,808
213892_s_at	10,636	10,376	10,893	10,503	9,931	10,710
213893_x_at	9,348	9,240	9,404	9,052	8,568	8,962
213894_at	6,808	6,204	6,165	4,914	4,772	4,821
213895_at	4,665	5,124	5,110	7,635	7,967	7,283
213896_x_at	8,005	8,489	8,178	7,492	7,544	7,674
213897_s_at	9,672	9,588	9,697	10,204	8,638	9,919
213898_at	6,311	5,913	5,854	6,033	6,029	5,959
213899_at	9,227	9,043	8,482	6,844	7,321	6,228
213900_at	8,417	8,498	9,038	7,667	7,515	7,629
213901_x_at	7,633	7,540	7,154	7,471	7,607	7,879
213902_at	11,378	12,053	11,494	11,576	11,470	11,616
213903_s_at	6,748	6,629	6,925	6,717	6,810	6,889
213904_at	7,921	8,432	7,835	4,839	4,833	4,953
213905_x_at	6,181	5,738	6,080	8,461	9,227	10,215
213906_at	8,663	8,925	7,776	7,351	8,501	7,341
213907_at	6,525	6,541	6,546	6,576	6,458	6,069
213908_at	5,954	6,559	6,207	6,888	7,578	6,879
213909_at	7,914	7,608	7,767	7,706	7,613	7,718
213910_at	4,206	4,452	3,874	4,240	4,449	4,709
213911_s_at	13,883	13,770	13,763	13,525	13,443	13,650
213912_at	7,113	6,784	6,520	7,127	6,455	6,510
213913_s_at	6,675	7,096	7,599	5,769	5,823	5,699
213914_s_at	6,821	6,866	6,993	6,985	6,735	6,874
213915_at	4,926	5,067	4,963	5,375	5,588	5,450
213916_at	8,606	9,164	8,509	6,393	7,060	6,138
213917_at	7,458	7,340	7,536	7,925	7,905	7,883
213918_s_at	9,440	9,116	9,122	7,892	9,644	6,738
213919_at	6,650	6,681	7,102	7,620	7,634	7,603
213920_at	8,215	6,795	7,553	5,857	5,920	5,779
213921_at	6,065	6,160	6,223	6,600	6,435	7,018
213922_at	6,639	6,994	6,898	4,294	5,180	4,458
213923_at	11,295	11,255	11,567	10,313	10,205	10,529
213924_at	9,834	10,064	9,325	7,481	7,278	7,318
213925_at	6,661	7,134	6,920	6,776	6,956	6,695
213926_s_at	7,952	8,069	7,852	8,914	8,216	8,442
213927_at	8,730	8,705	8,809	6,433	6,473	6,546
213928_s_at	5,734	5,616	6,080	6,053	5,936	5,700
213929_at	4,787	4,910	4,916	4,640	4,545	4,459
213930_at	6,683	6,655	6,637	4,932	6,892	5,332
213931_at	8,331	9,083	8,387	6,616	11,345	10,345
213932_x_at	11,803	12,148	12,225	12,266	12,317	12,637
213933_at	5,375	5,893	5,693	5,405	5,307	5,360
213934_s_at	9,477	9,847	9,020	7,473	8,243	6,492
213935_at	8,036	8,013	7,821	7,826	7,950	7,782
213936_x_at	8,959	8,658	8,932	8,347	8,807	8,391
213937_s_at	8,380	8,164	8,302	9,008	8,258	8,876
213938_at	7,456	8,032	7,826	6,121	5,790	6,134

213939_s_at	8,799	8,547	8,503	5,901	9,158	6,793
213940_s_at	9,555	9,810	9,604	8,464	8,869	9,073
213941_x_at	14,314	14,466	14,408	13,713	13,870	13,949
213942_at	6,983	6,945	7,100	7,514	7,439	7,695
213943_at	7,334	7,974	7,662	11,589	11,173	11,900
213944_x_at	9,040	8,953	9,472	10,065	9,983	10,280
213945_s_at	6,505	6,641	6,630	6,110	6,088	6,265
213946_s_at	7,040	7,012	6,907	7,597	7,312	7,695
213947_s_at	9,886	9,946	10,008	7,203	7,201	7,539
213948_x_at	6,670	6,624	6,486	6,937	6,621	6,775
213949_s_at	6,368	6,819	6,686	7,890	7,741	8,059
213950_s_at	5,856	5,676	5,635	5,841	5,849	6,011
213951_s_at	9,629	9,228	9,168	8,605	8,640	8,155
213952_s_at	5,262	5,034	5,196	5,426	5,430	5,451
213953_at	5,253	5,207	5,458	5,736	5,429	5,489
213954_at	8,546	8,719	8,318	4,070	3,986	4,151
213955_at	8,683	8,050	8,429	7,397	7,342	7,618
213956_at	9,153	8,852	8,823	7,861	8,757	7,871
213957_s_at	9,008	8,873	8,548	7,022	7,425	6,567
213958_at	7,961	7,952	8,120	8,289	8,201	8,196
213959_s_at	7,075	7,356	6,512	6,609	7,538	5,682
213960_at	4,585	4,771	4,975	5,662	5,188	5,224
213961_s_at	5,825	5,320	5,622	5,792	5,730	5,995
213962_s_at	6,003	6,322	6,039	6,678	6,722	6,684
213963_s_at	5,458	5,957	5,815	5,816	5,565	5,617
213964_x_at	3,638	3,543	4,069	4,051	4,194	4,006
213965_s_at	6,514	6,070	6,553	7,395	7,313	7,616
213966_at	7,097	6,909	6,918	7,758	7,116	7,737
213967_at	6,274	6,416	6,222	6,423	6,278	6,442
213968_at	5,319	5,301	5,788	5,946	5,808	5,909
213969_x_at	13,253	13,579	13,435	13,169	13,137	13,439
213970_at	7,730	7,908	7,734	7,568	7,696	7,271
213971_s_at	8,747	9,091	8,746	7,356	6,669	6,899
213972_at	6,147	6,193	6,087	6,767	6,537	6,898
213973_at	6,743	6,401	6,783	7,520	7,437	7,236
213974_at	5,598	5,791	5,687	5,839	5,528	5,568
213975_s_at	4,826	4,864	5,033	5,074	4,861	5,025
213976_at	6,187	6,614	6,464	6,621	6,800	6,772
213977_s_at	8,196	8,085	8,152	8,054	7,533	8,398
213978_at	6,504	6,491	6,411	7,007	6,903	7,086
213979_s_at	9,958	9,950	10,444	10,620	9,550	10,044
213980_s_at	9,486	9,413	9,798	8,961	9,336	9,262
213981_at	7,558	6,963	7,680	8,480	8,490	9,084
213982_s_at	8,142	8,907	8,660	7,744	9,349	7,937
213983_s_at	8,129	8,337	7,656	7,854	8,024	7,811
213984_at	8,398	8,659	7,800	8,460	8,657	8,508
213985_s_at	5,825	6,407	6,408	7,495	7,337	7,473
213986_s_at	7,290	7,112	6,938	7,617	6,948	7,358
213987_s_at	7,046	7,467	6,866	7,271	6,890	7,469
213988_s_at	8,721	9,143	8,456	10,507	9,459	10,168
213989_x_at	8,947	8,751	9,039	8,910	8,673	8,860
213990_s_at	7,346	7,012	7,093	7,151	7,101	7,146
213991_s_at	6,476	6,730	6,647	7,113	6,742	6,938
213992_at	9,233	9,463	9,544	5,689	6,396	5,133
213993_at	5,396	6,012	5,535	5,488	5,098	5,438
213994_s_at	6,881	7,915	6,975	4,483	4,623	4,373

213995_at	9,185	9,230	9,074	9,029	8,151	8,692
213996_at	7,215	6,627	6,473	6,125	6,077	6,180
213997_at	6,542	6,462	6,538	6,439	6,275	6,343
213998_s_at	8,954	8,428	8,440	6,887	7,146	7,156
213999_at	5,909	6,112	6,287	6,599	6,396	6,518
214000_s_at	5,927	6,204	5,790	7,078	6,724	6,981
214001_x_at	8,616	8,658	8,938	8,168	9,467	9,032
214002_at	8,295	8,284	8,034	8,402	8,154	8,367
214003_x_at	14,641	14,714	14,682	14,155	14,258	14,346
214004_s_at	10,068	9,868	10,084	9,528	9,832	9,691
214005_at	9,315	9,862	9,889	11,237	10,339	11,000
214006_s_at	7,466	7,516	7,586	8,561	7,821	8,595
214007_s_at	6,534	6,707	6,363	6,936	6,443	7,114
214008_at	5,127	5,201	5,544	5,482	5,530	5,519
214009_at	6,379	6,497	6,687	7,081	7,005	6,964
214010_s_at	8,042	8,068	8,141	8,085	8,176	8,252
214011_s_at	10,531	10,038	10,430	11,201	9,988	10,724
214012_at	6,086	6,694	5,112	3,981	4,045	5,066
214013_s_at	5,938	5,944	5,932	6,385	6,017	6,043
214014_at	7,883	7,470	7,830	8,314	8,048	8,371
214015_at	6,653	6,226	6,387	6,836	6,249	6,380
214016_s_at	12,089	12,265	11,627	8,878	11,108	9,131
214017_s_at	7,984	7,734	8,064	8,251	8,263	8,421
214018_at	5,819	5,954	5,864	6,280	5,930	6,102
214019_at	7,122	6,778	6,862	7,568	7,082	7,190
214020_x_at	10,564	10,483	11,013	11,993	12,260	12,427
214021_x_at	5,342	5,150	5,168	5,548	6,646	6,437
214022_s_at	11,306	11,706	12,130	6,285	5,872	6,492
214023_x_at	12,941	12,788	12,873	5,178	5,072	5,400
214024_s_at	7,462	7,326	7,545	8,329	8,415	8,545
214025_at	6,760	6,863	6,546	6,811	6,595	6,741
214026_s_at	4,558	4,380	4,731	5,111	5,036	5,055
214027_x_at	8,090	8,422	8,495	8,486	8,470	8,452
214028_x_at	7,443	7,736	7,414	7,285	7,531	7,306
214029_at	5,502	5,344	5,351	5,446	5,330	5,411
214030_at	8,911	8,709	8,497	8,407	9,916	8,288
214031_s_at	5,228	5,729	5,520	5,260	5,330	5,372
214032_at	6,870	6,431	6,537	6,824	6,639	6,879
214033_at	7,702	7,612	7,335	6,368	6,104	6,316
214034_at	4,929	4,934	4,624	4,913	4,912	4,934
214035_x_at	10,045	9,832	9,993	9,034	9,217	9,314
214036_at	9,452	9,149	9,387	8,499	9,030	8,527
214037_s_at	7,838	7,492	7,421	8,018	7,308	7,576
214038_at	4,535	4,437	4,309	4,701	4,833	4,764
214039_s_at	13,828	13,694	13,954	11,749	11,267	11,426
214040_s_at	7,666	8,023	8,098	8,406	8,403	8,419
214041_x_at	6,709	7,257	7,299	6,876	8,250	7,450
214042_s_at	11,035	11,148	10,637	10,987	11,159	10,836
214043_at	9,994	9,927	9,792	6,149	6,527	5,902
214044_at	5,460	5,171	5,380	4,377	4,422	4,513
214045_at	8,446	8,494	8,517	7,423	6,422	6,806
214046_at	7,499	7,107	6,248	4,427	4,426	4,311
214047_s_at	10,506	10,793	10,321	9,306	9,513	8,819
214048_at	7,543	7,110	6,478	5,549	6,765	5,578
214049_x_at	7,451	7,005	7,398	7,858	7,681	8,131
214050_at	7,196	7,113	7,234	7,512	7,294	7,425

214051_at	9,388	9,580	9,485	7,605	7,615	7,672
214052_x_at	8,443	8,205	8,501	6,215	6,973	6,281
214053_at	5,722	5,864	5,438	5,533	5,507	5,411
214054_at	8,089	7,800	7,662	8,267	8,393	8,303
214055_x_at	10,574	10,343	10,358	9,022	9,822	9,107
214056_at	6,360	6,632	7,051	6,076	5,808	5,958
214057_at	7,471	8,193	7,771	7,196	7,418	7,385
214058_at	8,652	8,710	8,953	6,488	6,452	6,072
214059_at	5,219	5,370	5,428	6,036	6,669	6,098
214060_at	7,462	7,786	7,511	5,973	7,108	6,062
214061_at	9,398	9,717	8,829	6,591	7,511	6,083
214062_x_at	9,154	8,920	8,880	9,367	9,137	9,463
214063_s_at	7,276	7,243	7,837	7,036	6,999	7,169
214064_at	7,320	7,394	7,419	7,964	8,017	7,939
214065_s_at	6,281	6,332	6,240	6,705	6,791	6,892
214066_x_at	8,267	8,177	8,154	8,288	8,524	8,911
214067_at	6,752	6,606	6,496	7,356	7,277	7,433
214068_at	5,308	5,513	5,308	5,433	5,222	5,231
214069_at	7,463	7,241	7,273	7,815	7,711	7,794
214070_s_at	5,952	6,278	5,936	6,689	6,410	6,696
214071_at	6,282	6,248	6,028	4,016	4,134	4,104
214072_x_at	7,956	8,115	8,110	8,119	8,106	8,219
214073_at	5,958	6,924	6,515	9,202	8,184	8,751
214074_s_at	6,270	6,323	6,379	6,889	6,250	6,831
214075_at	9,284	9,534	9,523	10,098	9,350	9,797
214076_at	8,043	7,851	8,225	8,256	8,211	8,248
214077_x_at	6,225	6,915	6,191	7,900	8,193	8,125
214078_at	7,823	7,483	7,022	3,510	3,740	3,791
214079_at	4,656	4,469	4,682	4,390	4,519	4,626
214080_x_at	10,161	9,636	10,108	9,867	9,615	9,989
214081_at	6,017	6,254	6,479	6,461	6,279	6,496
214082_at	6,585	6,624	6,729	7,110	6,633	6,702
214083_at	7,321	7,617	7,404	8,760	8,291	8,785
214084_x_at	6,746	6,548	6,305	7,075	7,302	6,845
214085_x_at	7,009	7,022	6,417	10,288	12,391	12,237
214086_s_at	9,878	9,771	9,223	8,623	8,345	7,787
214087_s_at	5,624	5,359	5,544	5,848	5,724	5,920
214088_s_at	6,771	6,605	6,756	7,077	6,710	7,085
214089_at	6,252	6,300	6,512	6,717	6,431	6,577
214090_at	5,966	6,074	6,171	6,355	6,421	6,469
214091_s_at	8,130	7,845	8,380	8,137	8,177	8,500
214092_x_at	10,735	10,718	10,855	9,932	10,378	10,014
214093_s_at	10,063	10,264	9,900	6,439	8,373	7,084
214094_at	6,812	6,712	6,834	7,203	7,040	6,953
214095_at	9,912	9,943	10,016	9,086	8,370	9,012
214096_s_at	12,266	12,344	12,320	11,977	11,289	11,954
214097_at	11,206	11,392	11,813	10,789	10,979	11,224
214098_at	7,221	8,476	6,536	6,077	5,306	6,056
214099_s_at	6,997	7,127	7,204	8,250	9,066	8,129
214100_x_at	10,474	10,278	10,409	9,129	9,473	9,586
214101_s_at	6,986	7,369	7,614	7,045	7,472	7,131
214102_at	11,320	11,446	11,497	6,917	8,314	6,953
214103_s_at	6,536	6,311	6,844	6,912	7,018	7,152
214104_at	9,012	8,970	8,843	7,999	7,072	7,723
214105_at	5,830	5,709	6,000	6,338	6,265	6,313
214106_s_at	9,002	8,840	9,040	9,523	9,287	9,357

214107_x_at	8,316	8,398	8,174	8,842	9,760	9,401
214108_at	6,803	6,503	6,809	7,730	7,357	7,865
214109_at	9,411	9,564	9,124	7,127	8,852	6,630
214110_s_at	7,253	7,844	7,467	7,562	7,748	7,552
214111_at	5,662	6,062	6,490	6,937	6,161	6,686
214112_s_at	9,323	9,268	9,483	10,193	9,830	10,316
214113_s_at	11,577	11,210	11,722	9,445	9,598	9,525
214114_x_at	10,068	9,825	10,159	10,246	9,824	10,526
214115_at	6,573	6,432	6,956	7,592	7,967	7,727
214116_at	7,455	7,685	7,481	7,816	7,315	7,496
214117_s_at	7,249	7,053	7,013	6,930	6,598	6,901
214118_x_at	9,227	9,259	9,070	8,226	8,879	6,983
214119_s_at	8,922	8,770	9,130	10,144	9,526	10,271
214120_at	5,424	5,381	5,332	3,903	4,003	4,004
214121_x_at	8,044	7,965	8,174	8,457	8,521	8,823
214122_at	7,500	7,522	7,609	7,984	8,034	8,168
214123_s_at	6,192	5,807	6,304	6,749	6,824	6,953
214124_x_at	9,465	9,656	8,885	9,301	8,535	7,922
214125_s_at	7,401	7,606	7,523	7,627	7,508	7,557
214126_at	8,504	8,684	8,561	8,008	8,651	8,097
214127_s_at	6,663	6,735	7,025	7,167	7,000	6,998
214128_at	7,394	7,315	7,593	7,339	7,266	7,637
214129_at	9,403	9,171	9,319	10,101	10,440	9,751
214130_s_at	7,359	7,258	7,015	7,317	7,545	6,833
214131_at	9,271	8,770	8,170	5,312	7,492	5,892
214132_at	7,657	7,043	6,961	6,636	7,371	6,515
214133_at	6,744	6,966	6,847	7,019	7,001	6,983
214134_at	6,318	6,266	6,138	6,876	6,932	7,271
214135_at	7,637	7,807	7,949	7,805	7,708	8,023
214136_at	5,834	6,037	5,836	6,121	6,164	6,132
214137_at	5,837	6,081	6,106	5,886	5,841	6,070
214138_at	7,176	6,857	7,187	7,762	7,490	7,544
214139_at	5,929	5,876	5,953	5,888	6,120	6,026
214140_at	6,645	7,157	6,973	6,893	7,609	7,450
214141_x_at	13,007	12,799	12,171	11,797	11,140	11,443
214142_at	7,254	7,053	7,603	7,567	7,191	7,054
214143_x_at	14,219	14,424	14,306	13,860	13,855	14,023
214144_at	9,229	8,843	9,431	8,106	7,762	7,921
214145_s_at	7,114	7,265	7,050	7,631	7,705	7,610
214146_s_at	4,933	4,950	4,919	5,783	5,113	5,341
214147_at	5,443	5,634	5,283	5,809	6,083	5,800
214148_at	7,609	8,052	7,707	7,404	7,083	7,406
214149_s_at	7,082	6,945	7,494	7,834	8,901	8,545
214150_x_at	11,448	11,562	11,857	13,015	13,102	13,307
214151_s_at	6,389	7,342	6,579	6,457	8,023	6,807
214152_at	6,791	7,724	6,673	6,529	8,063	6,819
214153_at	6,876	6,702	6,815	6,538	6,406	6,754
214154_s_at	5,816	5,888	6,190	6,991	6,956	7,051
214155_s_at	9,305	8,984	9,005	8,334	8,900	7,749
214156_at	6,122	7,092	7,257	4,253	4,041	4,043
214157_at	6,795	6,744	7,530	5,417	4,922	5,603
214158_s_at	7,155	7,182	7,080	7,188	6,899	7,371
214159_at	5,557	5,346	5,146	6,393	5,422	5,750
214160_at	5,068	5,217	5,339	5,081	5,199	5,322
214161_at	4,879	4,777	5,005	5,295	5,308	5,188
214162_at	4,714	4,687	4,861	4,657	4,433	4,599

214163_at	6,444	7,144	5,636	5,237	5,280	5,114
214164_x_at	8,303	8,682	8,573	10,775	11,257	11,243
214165_s_at	5,618	5,288	5,518	5,996	5,736	5,925
214166_at	6,849	6,849	7,009	7,225	7,381	7,327
214167_s_at	13,842	13,830	13,750	12,967	12,870	13,466
214168_s_at	7,703	7,897	8,088	7,741	8,412	7,749
214169_at	6,354	6,980	7,160	7,272	6,778	6,850
214170_x_at	11,697	11,891	11,691	11,374	11,707	11,566
214171_s_at	6,433	6,593	6,904	6,591	6,560	6,541
214172_x_at	9,064	8,949	8,547	7,798	7,694	7,834
214173_x_at	10,594	10,591	9,911	9,094	9,068	8,920
214174_s_at	7,358	7,233	7,302	8,611	8,690	8,813
214175_x_at	8,485	8,312	8,344	11,152	10,582	11,155
214176_s_at	7,560	7,714	7,620	7,213	7,286	7,271
214177_s_at	7,939	8,148	8,116	7,581	7,449	7,652
214178_s_at	6,310	6,236	6,598	6,562	6,373	6,476
214179_s_at	9,762	9,634	9,934	9,700	9,760	10,198
214180_at	7,558	7,563	7,612	6,527	6,709	6,853
214181_x_at	6,266	6,241	6,324	6,741	6,527	6,770
214182_at	8,060	8,214	8,415	8,640	9,311	9,150
214183_s_at	5,912	6,020	6,071	5,894	5,546	5,768
214184_at	6,453	6,459	6,764	6,409	6,432	6,419
214185_at	9,355	9,610	8,719	7,181	7,151	7,108
214186_s_at	7,309	7,232	7,223	7,576	7,397	7,619
214187_x_at	6,027	6,357	6,543	6,253	5,996	6,275
214188_at	6,325	6,543	6,866	7,133	6,958	7,312
214189_s_at	6,107	6,352	6,354	6,488	6,657	6,794
214190_x_at	6,271	6,364	6,022	6,755	6,740	6,763
214191_at	5,849	5,792	5,573	6,324	6,585	6,580
214192_at	6,466	6,926	6,711	7,339	7,110	7,036
214193_s_at	9,965	9,746	10,114	9,688	8,992	8,591
214194_at	8,242	8,461	8,261	7,330	7,656	7,293
214195_at	7,393	7,421	7,643	7,863	7,825	7,878
214196_s_at	6,765	6,379	6,061	6,406	6,752	6,678
214197_s_at	7,538	7,513	7,433	7,391	7,303	7,484
214198_s_at	9,682	9,456	10,139	9,800	9,827	10,192
214199_at	6,699	6,546	6,598	7,301	7,193	7,177
214200_s_at	6,535	6,042	6,567	7,141	7,228	7,351
214201_x_at	9,197	9,146	9,526	7,908	8,228	8,479
214202_at	8,439	8,426	7,843	6,221	7,435	6,238
214203_s_at	8,520	8,898	8,924	7,421	7,269	7,614
214204_at	4,694	4,560	4,829	5,297	5,099	5,346
214205_x_at	9,456	9,428	9,796	11,093	9,808	10,602
214206_at	6,978	6,816	7,030	7,652	7,378	7,308
214207_s_at	6,260	5,645	6,498	7,342	7,531	7,366
214208_at	6,874	6,579	6,823	7,857	7,348	7,523
214209_s_at	7,167	6,343	6,714	8,175	7,721	8,000
214210_at	8,595	8,717	8,768	8,199	8,589	8,410
214211_at	11,288	11,810	11,837	13,181	13,266	13,587
214212_x_at	8,092	8,305	7,993	8,203	8,717	8,633
214213_x_at	8,445	8,413	8,604	8,790	8,856	8,951
214214_s_at	12,298	12,229	12,541	12,197	11,349	11,809
214215_s_at	5,461	5,384	5,688	5,976	5,487	5,728
214216_s_at	5,505	5,407	5,588	6,154	6,115	6,270
214217_at	5,653	5,906	5,852	6,107	6,015	6,049
214218_s_at	4,127	4,447	4,308	5,946	4,188	4,222

214219_x_at	7,870	8,145	7,818	7,506	7,303	7,455
214220_s_at	10,088	9,710	9,342	6,519	7,548	6,461
214221_at	10,523	10,159	10,037	7,234	8,063	6,237
214222_at	6,557	6,579	6,185	7,115	7,184	7,275
214223_at	7,634	7,744	7,831	8,477	8,465	8,593
214224_s_at	13,015	13,019	12,797	11,582	12,048	11,911
214225_at	6,034	6,153	5,544	5,186	4,991	4,864
214226_at	7,858	7,771	7,876	7,576	7,526	7,800
214227_at	6,162	6,135	6,249	6,257	5,855	5,951
214228_x_at	6,407	6,681	6,437	6,900	6,934	6,976
214229_at	7,388	7,245	7,170	8,394	8,211	8,274
214230_at	7,489	7,805	7,706	8,324	8,352	8,125
214231_s_at	8,521	9,005	8,407	6,666	6,850	6,514
214232_at	8,538	8,477	8,737	7,805	7,927	7,811
214233_at	7,046	7,337	7,423	7,816	7,240	7,809
214234_s_at	6,698	6,720	6,803	7,382	7,289	7,305
214235_at	5,248	5,037	5,268	5,573	5,459	5,624
214236_at	6,216	6,322	6,512	6,363	6,708	6,333
214237_x_at	7,543	7,626	7,482	7,916	7,959	8,141
214238_at	7,507	7,429	7,634	7,280	7,270	7,372
214239_x_at	10,326	10,363	10,799	9,144	8,658	8,987
214240_at	8,599	8,899	9,196	7,640	7,342	7,506
214241_at	7,478	7,589	7,373	7,100	7,020	7,264
214242_at	6,202	6,342	6,585	7,222	6,458	6,388
214243_s_at	6,569	6,276	6,527	6,872	6,475	7,136
214244_s_at	5,830	5,969	6,347	7,374	7,099	7,224
214245_at	6,239	6,013	6,273	6,606	6,231	6,336
214246_x_at	9,099	9,172	9,710	9,280	9,717	9,766
214247_s_at	9,948	10,503	9,859	9,651	9,224	10,037
214248_s_at	7,701	7,822	8,199	7,632	7,489	7,808
214249_at	6,553	5,711	6,154	6,387	6,220	6,387
214250_at	6,714	6,263	6,598	6,965	6,761	6,867
214251_s_at	6,049	5,432	5,895	6,215	6,407	6,620
214252_s_at	6,143	7,173	6,536	7,578	7,314	7,625
214253_s_at	8,251	8,313	8,257	7,893	7,920	8,095
214254_at	4,395	4,423	4,617	4,413	4,260	4,406
214255_at	7,458	7,521	7,783	8,117	8,665	9,252
214256_at	7,221	7,357	7,442	7,787	7,539	7,438
214257_s_at	10,786	10,765	10,840	12,052	12,296	12,083
214258_x_at	8,798	8,625	8,971	8,356	7,656	8,118
214259_s_at	9,017	9,583	9,522	11,432	10,756	11,340
214260_at	7,867	8,242	8,257	8,252	8,482	8,295
214261_s_at	5,458	5,471	5,361	5,897	6,118	6,607
214262_at	7,609	7,595	7,642	8,504	7,955	8,218
214263_x_at	10,330	10,179	10,452	10,486	10,167	10,566
214264_s_at	9,141	9,585	8,817	9,152	8,186	8,675
214265_at	5,994	6,099	6,129	6,211	6,607	6,783
214266_s_at	6,401	6,475	6,377	7,109	6,417	7,096
214267_s_at	6,709	6,697	6,977	7,719	7,921	7,969
214268_s_at	9,889	10,128	10,044	10,015	10,032	9,508
214269_at	5,466	6,311	6,825	6,821	6,317	7,143
214270_s_at	5,363	5,389	5,394	6,326	5,797	6,206
214271_x_at	14,007	14,265	14,181	13,455	13,549	13,715
214272_at	6,706	6,927	6,864	7,325	7,369	7,217
214273_x_at	7,059	7,289	7,202	7,296	6,597	7,139
214274_s_at	8,453	8,265	8,766	9,946	9,693	10,042

214275_at	6,377	6,366	6,373	6,790	6,489	6,701
214276_at	7,672	7,589	7,991	8,329	8,484	8,708
214277_at	4,993	4,946	4,724	6,704	6,467	7,065
214278_s_at	5,915	5,712	5,901	6,989	6,761	6,682
214279_s_at	6,138	5,539	5,996	6,578	6,204	6,069
214280_x_at	13,303	13,289	12,780	10,519	11,471	10,691
214281_s_at	11,035	11,066	11,359	10,212	10,129	9,926
214282_at	3,402	3,711	3,578	3,490	3,511	3,479
214283_at	8,998	8,802	8,992	8,144	8,104	8,135
214284_s_at	5,218	5,547	6,003	5,462	5,626	5,595
214285_at	7,855	8,352	8,326	7,062	7,947	8,289
214286_at	6,214	5,786	6,156	6,545	6,443	6,630
214287_s_at	6,783	6,581	6,567	6,697	6,045	6,555
214288_s_at	13,747	13,862	13,903	13,440	13,400	13,452
214289_at	6,457	7,749	7,386	6,058	7,132	6,305
214290_s_at	8,176	8,095	8,332	8,897	8,661	9,216
214291_at	8,897	8,809	8,562	8,109	8,445	8,062
214292_at	6,209	6,105	6,082	6,549	6,446	6,613
214293_at	7,858	7,442	7,382	8,252	9,491	9,276
214294_at	6,809	6,990	7,127	7,131	7,740	7,267
214295_at	7,181	6,516	6,744	4,963	6,187	5,277
214296_x_at	6,982	7,292	7,412	8,062	7,812	8,087
214297_at	7,110	7,275	6,934	7,516	8,170	7,598
214298_x_at	8,712	8,937	8,532	5,854	5,635	6,052
214299_at	8,332	8,141	8,683	8,035	7,948	7,859
214300_s_at	5,402	5,341	5,946	6,344	5,931	6,005
214301_s_at	7,197	7,362	7,523	7,583	7,591	7,869
214302_x_at	7,657	7,635	7,626	7,658	7,778	7,809
214303_x_at	6,778	6,343	6,630	7,464	7,496	7,614
214304_x_at	5,544	5,912	5,274	5,689	5,770	6,189
214305_s_at	11,056	11,129	11,075	9,421	10,013	9,053
214306_at	9,400	10,024	8,973	7,151	8,547	6,239
214307_at	5,487	5,441	5,085	5,821	5,804	5,794
214308_s_at	7,076	6,982	7,211	7,306	7,435	7,285
214309_s_at	6,715	6,164	6,398	6,695	6,462	6,501
214310_s_at	5,898	5,543	6,024	6,189	6,081	6,463
214311_at	7,651	7,504	7,599	8,083	8,197	8,327
214312_at	6,880	7,056	6,770	6,914	6,852	6,981
214313_s_at	6,661	6,650	6,006	4,662	6,160	4,115
214314_s_at	9,408	9,461	8,523	7,578	8,901	6,787
214315_x_at	7,690	7,383	7,665	6,214	7,806	7,353
214316_x_at	9,037	9,222	9,544	11,202	11,327	11,247
214317_x_at	13,246	13,452	13,403	12,941	12,935	13,126
214318_s_at	5,771	6,342	6,271	6,091	5,617	6,059
214319_at	7,115	7,654	7,301	6,546	6,567	6,565
214320_x_at	6,502	6,160	6,604	6,569	6,721	6,849
214321_at	5,295	5,886	5,870	7,207	7,609	7,243
214322_at	6,577	6,401	6,512	6,780	6,978	6,975
214323_s_at	12,312	12,479	12,033	10,374	11,192	10,678
214324_at	6,876	6,495	6,744	7,180	6,983	7,201
214325_at	6,460	6,754	6,699	7,150	7,197	7,413
214326_x_at	5,363	5,225	4,960	5,667	5,677	5,983
214327_x_at	13,919	14,161	14,054	13,570	13,779	13,961
214328_s_at	14,187	14,200	13,978	13,400	14,085	13,109
214329_x_at	4,094	4,452	4,327	4,068	4,419	4,067
214330_at	7,630	6,940	7,448	7,313	7,194	7,155

214331_at	8,766	8,789	8,713	7,110	6,423	7,024
214332_s_at	8,077	8,226	8,150	7,391	7,145	7,538
214333_x_at	8,054	8,084	8,190	9,413	8,939	9,451
214334_x_at	10,872	11,699	11,278	11,795	11,928	12,130
214335_at	7,695	7,739	8,154	7,322	7,660	7,853
214336_s_at	6,933	6,928	6,948	6,782	6,565	7,105
214337_at	5,661	5,796	5,751	7,059	6,626	7,378
214338_at	8,302	8,131	8,445	8,721	8,739	8,769
214339_s_at	7,819	7,885	7,952	7,372	7,211	7,356
214340_at	6,898	6,919	6,881	7,276	7,031	7,050
214341_at	6,574	6,165	6,735	7,512	7,413	7,328
214342_at	6,705	6,375	6,644	6,936	6,929	7,129
214343_s_at	5,459	6,043	5,318	6,346	6,224	6,325
214344_at	6,153	6,168	6,255	6,335	6,524	6,382
214345_at	6,911	7,163	7,119	7,662	7,854	7,914
214346_at	5,857	5,631	5,275	5,945	5,501	6,453
214347_s_at	6,687	6,619	6,262	7,883	7,926	8,271
214348_at	6,804	6,808	6,678	7,354	7,424	7,502
214349_at	7,351	7,289	7,401	10,047	9,031	8,657
214350_at	6,474	6,251	6,235	6,900	6,756	6,892
214351_x_at	12,422	12,716	12,529	12,228	11,823	12,562
214352_s_at	12,767	12,622	12,146	9,071	9,849	9,139
214353_at	6,939	7,246	7,163	7,080	7,024	7,337
214354_x_at	7,096	7,045	7,203	7,500	7,125	7,041
214355_x_at	9,080	9,050	9,164	7,927	8,453	8,125
214356_s_at	11,021	11,181	11,151	9,605	10,429	9,782
214357_at	6,533	6,171	6,393	7,482	6,555	6,956
214358_at	8,840	8,887	8,733	8,336	7,658	8,031
214359_s_at	12,719	12,244	12,028	11,024	10,925	10,964
214360_at	6,386	5,901	6,305	6,705	6,216	6,730
214361_s_at	7,190	7,045	7,202	7,382	6,764	7,136
214362_at	6,319	6,339	6,300	7,557	7,182	7,720
214363_s_at	13,834	13,952	13,704	12,049	13,193	12,128
214364_at	7,783	7,315	7,240	7,045	6,663	7,105
214365_at	6,239	6,375	6,583	6,668	6,749	6,953
214366_s_at	5,564	5,581	5,481	5,820	5,751	5,590
214367_at	6,993	6,458	6,918	7,961	7,771	7,849
214368_at	6,412	6,459	6,903	6,217	6,205	6,214
214369_s_at	6,671	6,260	6,313	6,110	6,254	5,940
214370_at	3,940	3,708	4,011	4,087	3,993	4,045
214371_at	6,147	5,643	5,999	6,785	6,438	6,585
214372_x_at	8,201	8,466	8,592	8,234	9,037	8,707
214373_at	8,581	8,722	7,994	7,153	7,378	7,140
214374_s_at	8,759	8,717	8,348	8,096	8,475	8,457
214375_at	7,893	7,677	8,001	6,741	8,674	7,612
214376_at	6,485	6,127	5,812	6,195	6,068	6,128
214377_s_at	7,196	7,092	7,390	7,138	6,825	7,030
214378_at	4,786	4,953	4,641	6,399	7,556	7,479
214379_at	6,728	6,760	6,682	6,972	6,789	6,986
214380_at	6,016	6,393	6,276	6,832	6,862	6,852
214381_at	8,909	8,787	9,124	7,069	6,883	6,807
214382_at	5,236	5,853	5,537	5,172	5,160	5,247
214383_x_at	9,268	9,212	9,217	8,460	8,126	8,625
214384_s_at	7,018	7,071	7,527	7,215	6,879	7,034
214385_s_at	6,516	6,340	6,443	7,185	7,026	7,012
214386_at	7,071	6,566	7,241	7,278	7,309	7,390

214387_x_at	7,178	7,123	7,171	7,458	7,697	7,611
214388_at	7,228	7,175	7,174	7,313	7,725	7,451
214389_at	4,226	4,502	4,622	3,678	3,764	3,914
214390_s_at	9,304	9,292	9,178	8,140	7,598	8,029
214391_x_at	8,374	8,356	8,485	8,121	7,877	8,182
214392_at	6,884	6,757	6,473	7,177	7,058	7,243
214393_at	6,993	6,848	6,920	6,762	6,550	6,634
214394_x_at	13,925	13,825	13,941	13,680	13,484	13,824
214395_x_at	7,814	7,652	7,983	7,400	7,265	7,560
214396_s_at	9,342	9,614	9,546	7,388	7,054	6,995
214397_at	10,044	10,562	10,462	4,365	4,342	4,286
214398_s_at	7,385	7,427	7,497	8,689	8,475	8,598
214399_s_at	7,267	7,310	7,145	7,314	7,394	7,461
214400_at	5,577	5,586	5,693	6,851	6,629	6,808
214401_at	6,546	6,443	6,592	7,132	6,889	7,028
214402_s_at	6,220	6,342	7,076	7,117	7,324	7,117
214403_x_at	8,399	8,468	8,641	9,033	9,533	9,251
214404_x_at	8,066	8,104	8,054	8,748	8,918	9,031
214405_at	4,972	4,469	4,912	4,433	4,509	4,483
214406_s_at	6,082	5,966	5,959	5,759	5,861	5,841
214407_x_at	6,956	6,744	6,605	7,322	7,061	7,243
214408_s_at	5,388	5,442	5,390	5,868	5,742	5,938
214409_at	4,955	5,023	5,056	3,981	3,740	3,884
214410_at	4,418	4,579	4,114	4,599	4,288	4,410
214411_x_at	6,842	7,019	7,036	7,666	7,696	7,713
214412_at	6,748	6,305	6,568	6,746	6,973	6,926
214413_at	6,325	5,976	6,265	6,615	6,693	6,684
214414_x_at	8,011	8,041	8,102	8,067	7,664	8,012
214415_at	4,654	4,419	4,647	4,092	4,245	4,191
214416_at	5,127	5,196	4,584	6,143	5,901	5,608
214417_s_at	6,151	5,993	6,107	7,003	7,078	7,105
214418_at	5,952	6,201	6,123	5,950	6,020	5,801
214419_s_at	6,553	6,671	6,575	6,987	7,144	7,003
214420_s_at	5,333	5,406	5,748	5,884	5,866	5,697
214421_x_at	6,224	5,966	5,990	6,858	6,315	6,512
214422_at	5,017	4,985	4,924	4,160	4,569	4,183
214423_x_at	7,615	7,332	7,434	8,350	8,155	8,429
214424_s_at	5,444	5,133	5,320	5,954	5,627	5,850
214425_at	6,575	6,125	6,713	6,914	6,944	7,080
214426_x_at	11,289	10,724	11,434	9,173	8,990	9,068
214427_at	11,145	11,015	11,582	10,439	9,980	10,351
214428_x_at	7,775	7,465	7,751	8,743	8,429	8,290
214429_at	9,205	9,656	9,285	10,148	10,851	10,153
214430_at	10,775	10,871	11,134	10,995	10,750	11,495
214431_at	12,118	12,333	12,208	10,946	11,140	10,560
214432_at	6,093	6,112	6,423	7,045	6,710	6,587
214433_s_at	9,533	9,342	9,344	8,156	8,648	8,616
214434_at	8,168	8,363	8,039	7,472	7,693	7,229
214435_x_at	9,975	10,200	9,917	11,546	9,965	10,868
214436_at	7,958	8,503	7,375	8,242	8,600	8,656
214437_s_at	10,566	10,298	10,101	9,971	8,707	9,927
214438_at	7,252	7,359	7,693	9,971	8,972	9,530
214439_x_at	9,600	9,750	9,496	9,889	10,256	9,821
214440_at	8,383	9,097	8,173	8,789	9,055	8,981
214441_at	6,838	6,933	7,372	7,238	7,127	7,189
214442_s_at	8,623	9,141	8,988	8,764	8,446	8,626

214443_at	6,290	6,174	6,276	6,242	6,053	6,265
214444_s_at	7,633	7,512	7,933	8,557	8,217	8,324
214445_at	4,985	4,698	5,097	5,352	5,358	5,639
214446_at	6,254	6,633	6,430	8,128	8,183	8,477
214447_at	7,308	7,358	7,315	7,978	7,758	7,973
214448_x_at	7,625	7,290	7,503	8,226	7,798	8,200
214449_s_at	7,295	7,411	6,720	7,619	7,145	7,727
214450_at	7,267	7,099	6,917	7,615	7,009	7,704
214451_at	4,852	4,880	4,966	6,019	5,815	5,806
214452_at	10,088	10,211	10,281	8,895	7,910	8,248
214453_s_at	4,842	4,996	5,070	10,069	9,675	8,889
214454_at	7,149	7,134	7,083	7,901	7,932	8,384
214455_at	3,704	4,095	4,048	4,523	5,784	5,774
214456_x_at	6,741	6,316	6,938	7,188	7,004	7,122
214457_at	4,865	5,260	5,024	5,360	5,349	5,773
214458_at	6,618	6,375	6,931	7,452	7,408	7,819
214459_x_at	10,562	10,905	10,683	11,600	10,985	11,601
214460_at	7,649	7,089	7,369	7,372	7,131	7,364
214461_at	6,535	6,775	6,909	6,400	6,163	6,380
214462_at	6,183	6,377	6,321	6,362	6,064	6,057
214463_x_at	9,718	9,635	9,751	8,769	8,164	8,291
214464_at	10,454	10,196	10,138	7,882	9,299	7,678
214465_at	5,905	5,994	5,783	6,103	6,038	5,849
214466_at	5,821	5,539	5,769	6,069	6,098	6,142
214467_at	5,187	5,155	5,051	5,351	5,269	5,414
214468_at	10,878	10,569	10,206	8,379	8,285	8,339
214469_at	5,890	6,039	5,921	6,108	5,649	5,733
214470_at	5,454	5,446	5,636	6,122	5,843	6,062
214471_x_at	7,547	7,559	7,608	7,309	7,195	7,105
214472_at	5,564	5,691	5,515	5,639	5,831	5,633
214473_x_at	9,158	8,949	9,034	8,776	8,374	8,836
214474_at	7,733	8,281	8,214	6,880	6,823	7,135
214475_x_at	4,823	5,215	4,874	5,616	5,651	5,891
214476_at	6,528	6,414	6,469	7,235	7,184	7,045
214477_at	6,638	6,485	6,102	6,877	6,852	6,987
214478_at	6,574	6,623	6,758	7,720	7,195	7,651
214479_at	7,152	7,053	7,377	7,737	7,688	7,639
214480_at	6,562	6,519	6,128	6,740	6,310	6,474
214481_at	5,977	5,677	6,327	6,672	6,331	6,795
214482_at	7,146	7,143	7,520	7,681	7,905	7,108
214483_s_at	6,461	6,848	6,623	6,954	6,714	7,098
214484_s_at	7,587	7,452	7,933	8,903	8,147	8,976
214485_at	6,754	6,841	6,706	7,437	7,228	7,025
214486_x_at	7,165	7,600	7,603	7,736	7,818	7,687
214487_s_at	7,112	6,977	6,791	6,953	6,485	6,957
214488_at	4,636	4,773	5,174	5,073	5,020	5,128
214489_at	6,652	6,941	6,813	7,346	7,033	6,944
214490_at	7,122	6,994	6,824	7,324	7,182	7,320
214491_at	7,360	7,279	7,257	8,151	7,935	7,880
214492_at	7,026	6,890	7,329	7,946	7,851	7,801
214493_s_at	6,644	7,418	6,542	7,986	8,462	8,112
214494_s_at	10,459	10,371	10,572	9,937	10,474	10,503
214495_at	5,995	6,339	6,166	6,761	6,836	6,788
214496_x_at	6,945	6,873	6,806	6,908	6,827	6,884
214497_s_at	6,290	6,395	6,162	6,807	6,931	6,725
214498_at	5,937	6,191	6,289	6,722	6,531	6,787

214499_s_at	9,526	9,179	9,227	8,908	9,453	8,162
214500_at	6,011	6,394	5,880	7,312	6,130	7,473
214501_s_at	10,673	10,264	10,815	10,963	10,024	10,820
214502_at	5,627	5,260	5,780	6,636	6,563	6,842
214503_x_at	4,585	4,299	4,524	4,923	4,521	4,722
214504_at	7,675	7,250	7,690	7,649	7,627	7,782
214505_s_at	7,647	7,839	7,865	7,466	7,446	7,718
214506_at	7,394	7,425	7,400	7,521	7,453	7,745
214507_s_at	8,476	7,976	8,220	8,393	7,491	7,834
214508_x_at	8,476	8,570	8,576	8,354	7,988	8,151
214509_at	5,160	5,461	5,206	5,819	5,297	5,364
214510_at	6,950	7,162	6,957	6,625	6,844	6,874
214511_x_at	5,297	5,447	5,738	6,157	5,770	5,961
214512_s_at	9,005	9,365	8,875	9,757	9,176	9,784
214513_s_at	8,272	8,226	7,722	7,422	7,147	7,298
214514_at	4,302	4,619	4,460	4,395	4,322	4,412
214515_at	4,297	4,543	4,364	4,774	4,666	4,742
214516_at	5,314	5,542	5,326	6,032	5,460	5,829
214517_at	6,515	6,587	6,434	6,812	6,407	6,745
214518_at	6,140	6,201	6,229	7,370	6,862	7,380
214519_s_at	4,835	4,881	5,093	5,187	5,160	5,227
214520_at	5,020	5,084	5,086	5,095	4,939	5,369
214521_at	5,687	5,592	5,984	6,301	5,818	6,123
214522_x_at	6,748	7,001	6,668	7,327	7,306	7,291
214523_at	6,709	6,733	6,937	7,763	7,791	8,050
214524_at	6,466	6,638	6,226	6,882	7,029	7,152
214525_x_at	8,151	8,293	8,139	7,704	7,799	7,650
214526_x_at	8,968	8,750	8,884	8,589	7,985	8,657
214527_s_at	9,724	9,130	9,505	9,128	7,950	8,999
214528_s_at	4,643	5,171	4,621	5,393	5,383	5,448
214529_at	5,574	5,376	5,578	6,015	6,320	6,491
214530_x_at	6,508	6,232	5,900	6,434	6,570	6,332
214531_s_at	8,895	8,729	8,420	8,781	8,475	9,016
214532_x_at	11,639	11,543	11,749	8,395	8,310	8,383
214533_at	5,547	5,461	5,328	6,454	6,491	6,299
214534_at	6,780	7,099	6,923	6,581	6,158	6,397
214535_s_at	7,229	7,435	7,533	7,642	7,814	8,187
214536_at	6,386	6,091	6,344	7,443	7,401	7,766
214537_at	6,811	6,823	7,056	6,803	6,175	6,802
214538_x_at	6,489	6,392	6,526	7,089	6,901	6,993
214539_at	6,247	5,738	5,930	6,284	6,395	6,426
214540_at	4,842	4,657	5,186	4,813	4,522	4,726
214541_s_at	5,448	5,156	5,379	5,346	5,506	5,634
214542_x_at	8,013	7,807	8,160	8,118	7,970	8,236
214543_x_at	7,970	7,712	7,700	7,439	7,290	7,482
214544_s_at	6,317	6,234	5,801	6,092	6,102	6,380
214545_s_at	8,397	7,888	8,204	7,457	6,536	7,402
214546_s_at	7,143	6,832	7,044	7,611	7,680	7,698
214547_at	4,208	4,082	4,400	4,261	4,344	4,275
214548_x_at	12,844	13,094	13,329	12,179	12,493	12,947
214549_x_at	7,425	7,018	7,183	8,179	7,918	8,143
214550_s_at	9,999	9,810	9,764	7,952	8,099	8,067
214551_s_at	5,692	5,582	5,913	7,298	7,320	7,376
214552_s_at	7,873	8,037	7,510	8,004	8,152	8,020
214553_s_at	8,510	8,614	8,165	7,837	7,250	7,945
214554_at	4,364	4,073	4,763	4,224	3,875	3,805

214555_at	5,833	5,825	5,784	7,012	6,917	6,938
214556_at	6,542	6,264	6,718	7,053	7,222	7,345
214557_at	4,708	4,810	4,763	5,044	4,948	5,126
214558_at	6,310	6,100	6,256	6,595	6,407	6,484
214559_at	4,961	5,213	4,724	5,209	5,351	5,310
214560_at	5,986	6,479	6,040	6,153	5,729	5,619
214561_at	6,181	5,940	6,327	6,451	6,420	6,885
214562_at	5,277	5,799	5,947	5,820	5,533	5,995
214563_at	6,717	6,837	6,729	7,051	7,117	7,063
214564_s_at	5,408	5,108	5,306	5,966	5,947	5,944
214565_s_at	5,195	5,119	5,194	5,247	5,163	5,170
214566_at	4,929	4,898	5,358	6,360	5,980	6,054
214567_s_at	5,091	4,691	4,721	6,598	6,102	6,801
214568_at	6,548	6,020	6,229	7,171	7,024	7,235
214569_at	3,704	3,930	3,716	3,815	3,830	3,780
214570_x_at	7,591	7,360	7,217	8,005	7,746	8,026
214571_at	6,410	6,106	6,246	6,777	6,577	6,723
214572_s_at	5,974	5,974	5,874	6,755	6,726	6,929
214574_x_at	6,615	6,603	6,571	6,710	6,439	7,006
214575_s_at	6,560	6,972	6,026	6,822	6,670	7,131
214576_at	5,935	5,219	6,036	6,209	6,312	6,171
214577_at	8,818	8,248	8,473	8,498	8,687	8,866
214578_s_at	8,230	8,415	7,705	7,747	8,548	7,823
214579_at	7,082	7,296	6,887	7,354	7,628	7,778
214580_x_at	6,411	6,483	6,852	6,755	6,366	6,304
214581_x_at	8,387	7,874	8,778	6,492	6,314	6,887
214582_at	4,944	4,882	4,959	3,759	3,942	3,747
214583_at	8,160	7,826	8,051	7,492	7,707	6,924
214584_x_at	7,018	6,530	6,948	7,376	7,354	7,375
214585_s_at	10,375	10,136	10,697	10,452	10,327	10,636
214586_at	3,726	4,270	3,967	3,891	4,039	4,165
214587_at	4,780	4,454	4,720	5,812	6,731	7,128
214588_s_at	6,195	6,467	6,209	6,776	6,925	7,028
214589_at	5,847	5,879	5,857	5,484	5,100	5,115
214590_s_at	6,470	6,765	6,209	6,347	5,375	6,264
214591_at	7,041	7,240	6,830	4,762	5,430	4,952
214592_s_at	6,746	6,933	6,744	7,699	7,115	7,467
214593_at	3,944	3,909	3,883	3,922	3,921	3,934
214594_x_at	9,153	8,638	8,968	8,058	9,629	8,234
214595_at	8,538	8,768	9,028	8,058	8,156	9,147
214596_at	8,181	8,364	7,938	5,396	5,499	5,390
214597_at	5,752	5,780	6,017	5,939	5,664	5,727
214598_at	4,808	4,922	4,660	4,833	4,660	4,812
214599_at	6,793	6,509	6,626	7,468	7,256	7,217
214600_at	7,098	6,618	6,689	7,074	7,638	7,455
214601_at	3,964	3,760	3,876	3,819	3,740	3,869
214602_at	5,538	6,641	5,604	5,100	4,913	5,274
214603_at	4,692	4,688	4,431	4,718	5,142	4,838
214604_at	6,406	6,863	6,520	6,337	6,146	6,193
214605_x_at	7,857	7,756	8,057	7,250	9,390	8,557
214606_at	4,836	4,669	4,710	4,616	4,764	4,503
214607_at	8,767	8,841	8,767	7,541	7,144	7,228
214608_s_at	7,837	8,011	7,877	7,137	7,202	7,500
214609_at	7,188	7,588	7,065	7,359	7,217	7,290
214610_at	4,434	4,413	4,657	4,793	4,688	4,626
214611_at	6,325	6,500	6,557	7,278	7,398	7,393

214612_x_at	4,597	5,015	4,979	5,599	5,033	5,198
214613_at	7,297	7,143	7,263	6,769	6,482	6,761
214614_at	6,483	6,371	6,698	6,601	6,110	6,403
214615_at	4,334	4,300	4,524	4,376	4,287	4,513
214616_at	6,103	6,030	6,098	6,471	6,415	6,352
214617_at	7,668	7,363	7,807	8,148	8,036	7,932
214618_at	5,003	4,926	4,946	4,938	4,805	4,948
214619_at	7,018	7,094	6,955	7,060	6,921	7,156
214620_x_at	8,261	8,115	7,867	8,381	9,812	8,988
214621_at	3,890	3,951	3,789	3,943	3,895	3,889
214622_at	6,350	6,173	6,558	7,101	6,781	7,081
214623_at	7,515	6,764	7,102	6,818	6,517	6,593
214624_at	6,375	6,533	6,425	6,519	6,099	6,334
214625_s_at	8,070	7,965	8,064	8,777	8,715	8,626
214626_s_at	9,952	9,740	9,962	9,532	9,499	9,830
214627_at	6,280	6,165	6,273	6,436	6,423	6,175
214628_at	8,173	7,694	7,786	8,021	7,733	7,808
214629_x_at	13,612	13,477	13,396	13,548	14,065	14,067
214630_at	6,369	6,568	6,848	6,445	6,217	6,480
214631_at	3,719	3,777	3,909	3,768	3,612	3,644
214632_at	7,183	6,839	7,343	7,080	5,779	5,959
214633_at	7,389	7,050	7,155	5,169	5,301	5,469
214634_at	7,181	7,249	7,241	7,384	7,170	7,307
214635_at	7,131	7,081	7,170	7,854	7,990	7,772
214636_at	6,571	7,742	6,831	5,864	5,756	6,011
214637_at	7,228	7,524	7,370	7,964	8,146	8,170
214638_s_at	6,103	6,620	6,004	6,160	6,414	6,269
214639_s_at	5,187	5,291	5,732	7,927	7,760	7,737
214640_at	6,100	5,878	5,873	6,466	6,172	6,470
214641_at	6,752	6,434	6,964	7,514	7,134	7,275
214642_x_at	5,698	5,962	5,890	6,247	6,250	6,542
214643_x_at	8,281	8,101	7,930	8,744	8,626	8,520
214644_at	4,837	4,642	4,783	5,272	5,043	5,113
214645_at	4,241	4,293	4,325	4,359	4,346	4,515
214646_at	5,982	6,143	6,206	6,201	6,187	6,563
214647_s_at	4,437	4,480	4,724	5,556	4,886	5,682
214648_at	5,196	5,215	5,167	5,418	5,244	5,301
214649_s_at	9,108	9,125	8,638	8,772	9,050	8,585
214650_x_at	5,730	5,991	6,058	5,069	5,720	5,315
214651_s_at	4,564	4,859	4,777	11,236	11,043	11,093
214652_at	4,984	4,929	5,052	5,458	5,345	5,308
214655_at	5,667	6,029	5,688	6,133	6,014	6,202
214656_x_at	10,068	9,848	10,024	10,732	11,185	11,399
214657_s_at	5,143	5,124	4,871	6,474	8,756	6,993
214658_at	10,009	9,994	9,929	11,338	11,890	11,839
214659_x_at	9,681	9,572	9,669	8,550	7,924	8,883
214660_at	4,227	4,266	4,543	4,710	4,725	4,797
214661_s_at	9,817	9,553	10,263	9,426	8,976	8,919
214662_at	9,883	9,900	9,798	8,794	8,995	8,471
214663_at	8,220	7,851	8,024	7,014	7,197	6,877
214664_at	4,939	4,735	5,641	4,666	4,461	4,562
214665_s_at	10,067	10,388	10,523	9,888	9,999	10,170
214666_x_at	7,524	6,694	6,688	5,896	5,993	6,065
214667_s_at	7,185	6,872	6,974	7,252	7,060	7,515
214668_at	5,470	5,487	5,012	6,348	5,955	5,881
214669_x_at	6,992	6,213	6,355	7,330	7,381	7,619

214670_at	8,280	8,225	8,181	5,759	7,899	6,233
214671_s_at	6,441	6,628	6,401	7,187	7,409	7,486
214672_at	8,678	8,573	8,903	8,211	8,326	7,994
214673_s_at	6,855	7,053	6,916	6,890	6,490	7,086
214674_at	8,114	8,099	8,379	7,720	7,583	7,733
214675_at	6,355	6,323	6,318	6,047	6,379	6,167
214676_x_at	7,200	7,070	7,105	7,389	7,289	7,344
214677_x_at	6,754	6,578	7,202	7,429	7,371	7,524
214678_x_at	8,230	7,613	7,692	8,057	8,730	7,242
214679_x_at	8,724	8,644	8,899	9,776	9,172	9,748
214680_at	5,715	5,388	5,282	6,485	6,434	6,599
214681_at	8,063	8,345	7,807	8,023	7,687	7,084
214682_at	6,836	6,888	6,779	6,779	6,736	6,714
214683_s_at	9,056	8,809	7,930	5,789	8,902	7,439
214684_at	8,448	8,843	8,616	7,941	9,787	8,328
214685_at	4,404	4,691	4,495	5,112	4,976	4,880
214686_at	10,645	10,545	10,458	8,147	8,724	8,027
214687_x_at	12,845	12,679	13,221	12,843	12,624	13,233
214688_at	6,783	6,733	7,010	7,326	7,283	7,248
214689_at	6,081	6,302	6,427	6,841	6,428	6,547
214690_at	7,257	7,143	6,752	5,727	6,617	5,746
214691_x_at	6,235	6,268	5,972	5,515	5,462	5,187
214692_s_at	9,088	8,981	9,016	9,016	8,946	9,084
214693_x_at	9,322	9,482	9,095	9,304	10,086	9,510
214694_at	6,758	6,777	7,104	7,343	7,250	7,114
214695_at	8,930	7,844	9,168	7,663	7,569	8,008
214696_at	7,843	9,266	8,273	10,134	11,072	11,258
214697_s_at	8,212	8,395	8,479	7,209	7,384	7,469
214698_at	7,763	7,953	8,075	7,816	8,000	7,706
214699_x_at	8,185	8,168	8,403	8,761	8,555	8,535
214700_x_at	10,297	10,413	9,882	7,687	9,087	6,564
214701_s_at	6,238	6,201	6,226	9,039	9,904	10,976
214702_at	4,764	4,880	4,513	9,132	10,112	10,738
214703_s_at	7,472	7,646	7,797	8,025	8,687	9,057
214704_at	7,223	7,201	7,488	7,381	7,358	7,613
214705_at	5,832	5,606	5,249	4,041	3,880	3,842
214706_at	10,151	9,814	10,368	7,468	6,290	6,720
214707_x_at	8,816	8,550	8,865	7,795	8,860	7,995
214708_at	5,650	5,305	5,780	6,804	6,760	6,767
214709_s_at	9,903	9,938	9,448	9,271	10,860	8,751
214710_s_at	12,505	12,577	12,435	12,118	11,693	11,642
214711_at	7,544	7,178	7,910	7,451	7,096	7,496
214712_at	4,006	3,711	4,177	3,662	3,710	3,652
214713_at	8,022	8,097	8,436	7,826	7,848	7,895
214714_at	8,160	8,462	8,282	8,096	7,349	7,601
214715_x_at	10,708	10,222	10,356	9,449	10,724	9,795
214716_at	4,741	5,007	4,488	4,806	5,247	4,433
214717_at	8,240	6,766	7,765	6,667	6,561	6,524
214718_at	8,279	8,351	8,310	7,540	6,880	7,435
214719_at	6,260	6,258	5,905	5,846	6,688	7,256
214720_x_at	8,868	9,207	8,496	8,262	8,493	8,240
214721_x_at	8,239	8,228	8,427	8,626	8,137	8,651
214722_at	10,891	10,592	10,418	9,380	11,955	9,728
214723_x_at	7,689	7,767	7,112	5,199	7,350	6,206
214724_at	8,030	8,187	7,613	6,848	7,307	7,690
214725_at	3,837	3,760	4,083	3,956	4,006	3,741

214726_x_at	9,500	9,155	9,128	9,967	9,716	10,119
214727_at	5,721	4,909	5,031	4,227	4,319	4,203
214728_x_at	11,723	11,636	11,884	10,214	10,199	10,422
214729_at	4,774	4,810	4,501	4,394	4,646	4,518
214730_s_at	10,881	10,633	10,977	10,049	10,663	10,519
214731_at	8,765	8,626	8,904	7,020	8,274	7,329
214732_at	6,119	6,131	6,253	6,028	5,863	6,084
214733_s_at	9,281	9,354	9,517	8,695	8,275	8,762
214734_at	5,497	5,653	5,648	5,488	5,608	5,719
214735_at	6,630	6,495	6,756	6,679	6,581	6,492
214736_s_at	9,675	9,621	10,001	9,506	9,939	10,004
214737_x_at	13,331	13,303	13,262	12,488	12,345	12,605
214738_s_at	7,850	7,790	7,846	8,326	7,966	8,457
214739_at	8,458	8,380	8,747	7,584	7,506	7,408
214740_at	6,821	6,657	6,928	7,147	7,163	7,361
214741_at	10,148	10,181	9,619	8,209	8,714	8,096
214742_at	8,381	8,527	8,505	8,103	8,088	8,338
214743_at	9,463	9,262	9,831	6,956	9,240	8,074
214744_s_at	8,667	8,947	9,162	9,505	9,180	9,382
214745_at	9,361	9,362	9,708	6,178	5,943	6,257
214746_s_at	7,509	7,155	6,900	7,464	6,865	7,018
214747_at	6,318	6,175	5,973	6,357	6,351	6,326
214748_at	6,403	6,310	6,777	6,953	8,331	7,373
214749_s_at	12,057	12,240	12,578	12,427	12,342	12,959
214750_at	7,739	7,576	7,808	8,185	8,025	8,357
214751_at	8,888	8,911	8,412	6,930	7,526	6,207
214752_x_at	9,900	9,496	9,849	9,712	9,995	10,305
214753_at	9,124	9,044	8,564	6,474	7,459	6,895
214754_at	7,725	7,473	7,338	7,810	7,265	7,320
214755_at	5,488	6,049	5,423	6,757	6,325	6,984
214756_x_at	8,898	8,887	9,091	8,873	8,441	8,862
214757_at	7,396	6,767	7,331	6,915	7,078	7,060
214758_at	6,779	7,003	6,810	7,245	7,153	7,340
214759_at	6,379	6,280	6,044	6,211	6,487	6,123
214760_at	8,459	8,607	8,360	8,263	8,416	8,385
214761_at	11,597	11,466	12,113	7,837	8,079	8,166
214762_at	5,926	6,525	6,035	6,675	6,799	7,046
214763_at	7,673	7,898	7,937	7,946	7,680	7,869
214764_at	8,948	8,590	8,532	5,505	6,609	5,671
214765_s_at	7,509	7,721	7,702	8,202	8,014	8,155
214766_s_at	9,976	9,913	9,450	8,594	9,619	7,707
214767_s_at	6,758	6,740	6,500	6,889	6,809	6,961
214768_x_at	6,168	6,363	6,301	6,799	6,704	6,751
214769_at	8,999	9,071	8,785	7,297	7,541	6,914
214770_at	3,789	3,794	4,045	3,796	3,894	4,078
214771_x_at	10,782	10,380	10,904	11,215	11,814	11,956
214772_at	6,597	6,654	6,900	6,147	6,661	7,057
214773_x_at	9,740	10,227	9,372	8,069	8,401	8,636
214774_x_at	9,900	10,103	10,216	3,928	4,003	3,957
214775_at	7,543	7,761	7,599	7,129	7,060	7,066
214776_x_at	4,090	4,192	4,159	5,713	5,235	5,706
214777_at	6,198	5,874	5,791	6,268	6,453	6,341
214778_at	7,593	7,329	7,340	6,872	6,840	7,302
214779_s_at	8,253	8,562	8,175	8,483	8,220	8,598
214780_s_at	7,943	7,871	8,111	7,574	7,974	8,016
214781_at	6,318	5,919	6,054	6,323	6,332	6,045

214782_at	5,970	5,864	5,675	5,338	6,028	5,528
214783_s_at	7,242	7,351	7,279	8,243	8,555	8,664
214784_x_at	10,729	10,573	10,820	10,589	10,502	10,570
214785_at	9,190	9,710	8,314	6,124	8,256	6,179
214786_at	5,223	5,609	5,949	5,134	4,887	5,015
214787_at	4,914	6,162	4,924	5,455	6,490	5,181
214788_x_at	7,049	7,023	6,966	7,175	7,421	7,442
214789_x_at	10,298	10,326	10,462	10,704	10,781	10,906
214790_at	7,499	8,849	7,296	5,954	6,312	5,844
214791_at	8,984	9,308	9,041	8,830	9,344	9,362
214792_x_at	6,191	5,818	6,180	6,743	5,740	6,306
214793_at	6,552	6,583	6,811	7,355	7,279	7,240
214794_at	8,609	7,925	8,700	7,300	6,374	6,986
214795_at	7,315	7,531	7,683	7,986	7,998	7,993
214796_at	6,275	6,094	6,211	6,446	6,310	6,369
214797_s_at	7,813	7,933	7,742	8,021	8,229	8,388
214798_at	6,920	6,863	6,751	6,768	6,840	6,735
214799_at	6,621	6,338	6,643	6,636	6,884	6,747
214800_x_at	13,171	13,098	13,226	12,907	13,117	13,117
214801_at	9,874	9,594	9,997	9,400	9,319	9,464
214802_at	8,254	7,580	7,870	7,889	7,736	7,724
214803_at	7,914	7,196	7,489	7,059	6,766	6,833
214804_at	9,034	9,228	8,644	7,275	7,529	7,170
214805_at	9,682	9,985	9,734	7,814	8,170	8,549
214806_at	9,508	9,690	9,735	7,621	7,623	7,312
214807_at	6,020	5,932	5,776	5,504	5,605	5,590
214808_at	8,052	8,034	7,863	7,914	8,154	8,262
214809_at	6,682	6,505	6,501	7,012	6,904	6,701
214811_at	6,343	5,735	6,210	5,958	5,805	5,877
214812_s_at	11,133	10,989	10,553	9,376	10,316	9,428
214813_at	6,012	6,190	5,811	5,824	5,497	5,691
214814_at	5,921	6,260	5,974	5,334	5,742	5,179
214815_at	7,076	5,519	6,130	4,514	4,931	4,602
214816_x_at	7,049	6,857	6,673	7,751	7,980	8,061
214817_at	8,158	7,841	7,948	7,166	7,338	7,390
214818_at	6,774	7,053	7,104	7,254	7,148	7,045
214819_at	7,195	7,175	7,411	7,712	7,784	7,831
214820_at	8,067	8,257	7,587	5,420	5,339	5,186
214821_at	5,804	5,641	5,549	5,510	5,357	5,436
214822_at	5,808	6,127	6,210	6,931	6,751	6,773
214823_at	6,562	8,220	6,100	4,363	4,326	4,128
214824_at	7,178	6,923	7,117	7,505	7,136	7,358
214825_at	6,377	6,660	6,732	5,629	6,072	5,786
214826_at	6,577	6,625	6,387	6,788	6,894	7,016
214827_at	4,794	4,627	4,730	5,212	4,998	5,047
214828_s_at	7,784	7,500	7,688	7,945	7,983	8,031
214829_at	8,683	8,487	8,320	6,118	6,192	5,622
214830_at	8,244	8,470	8,216	10,238	9,966	10,530
214831_at	7,336	7,564	7,174	7,067	7,084	6,659
214832_at	7,064	6,488	6,711	7,324	6,889	7,374
214833_at	5,598	6,115	6,129	5,775	6,091	6,000
214834_at	6,279	5,993	5,614	3,922	3,991	3,868
214835_s_at	10,476	10,789	9,950	10,508	10,711	10,876
214836_x_at	7,152	6,930	6,907	8,036	7,888	8,296
214837_at	5,257	5,417	5,564	5,712	5,507	5,676
214838_at	7,605	7,621	7,987	9,255	9,022	9,306

214839_at	8,220	8,276	7,713	7,041	7,343	7,134
214840_at	8,313	7,923	8,335	8,744	8,918	8,901
214841_at	8,870	8,758	9,655	6,573	6,251	6,563
214842_s_at	4,590	4,766	4,544	5,228	5,094	5,206
214843_s_at	10,792	11,384	10,690	9,674	9,931	9,697
214844_s_at	8,801	9,375	9,187	6,291	8,250	7,093
214845_s_at	8,449	8,396	7,932	9,132	9,145	9,161
214846_s_at	8,496	8,247	8,653	7,604	7,701	7,627
214847_s_at	7,423	7,649	7,722	7,762	7,384	7,779
214848_at	9,041	9,225	9,393	8,781	9,258	8,635
214849_at	7,281	7,602	7,413	7,857	7,490	7,721
214850_at	6,970	6,680	6,965	6,975	7,084	6,994
214851_at	6,527	6,383	6,287	6,759	6,711	6,529
214852_x_at	6,118	5,883	5,863	6,062	6,352	5,882
214853_s_at	11,548	11,534	11,897	12,161	12,646	12,888
214854_at	6,645	6,684	6,900	7,414	7,630	7,727
214855_s_at	8,660	8,635	7,995	6,193	7,852	6,636
214856_at	5,651	4,959	5,107	6,059	5,833	5,699
214857_at	7,402	7,317	7,396	6,773	6,726	6,803
214858_at	6,363	6,319	6,294	6,987	7,013	7,127
214859_at	6,628	6,591	6,805	7,102	7,144	7,145
214860_at	4,878	5,115	5,346	5,800	5,998	5,922
214861_at	6,342	6,511	6,815	5,844	6,721	5,827
214862_x_at	8,612	8,083	8,086	5,979	6,075	6,026
214863_at	5,041	4,963	5,207	5,153	4,996	5,211
214864_s_at	10,447	10,543	10,481	10,437	10,045	10,878
214865_at	6,110	6,128	6,017	6,494	6,425	6,758
214866_at	6,877	6,628	6,779	8,788	8,086	8,439
214867_at	7,883	7,843	7,903	7,886	7,831	7,960
214868_at	4,585	4,709	4,966	5,101	5,179	5,261
214869_x_at	8,888	9,024	9,196	8,600	8,621	8,666
214870_x_at	10,304	10,240	10,319	8,934	9,299	9,350
214871_x_at	6,646	6,560	6,594	7,856	7,666	7,710
214872_at	5,360	5,366	5,665	5,728	6,008	5,995
214873_at	6,982	6,743	7,050	7,161	6,998	7,128
214874_at	7,476	7,442	7,337	5,225	5,149	5,002
214875_x_at	9,706	9,478	9,350	8,772	9,818	9,191
214876_s_at	8,048	8,244	7,302	6,591	6,626	6,295
214877_at	7,498	7,361	7,306	6,706	6,423	6,746
214878_at	5,363	5,396	5,340	4,664	5,112	4,725
214879_x_at	8,878	8,650	8,688	9,743	9,349	9,422
214880_x_at	6,309	6,246	6,535	7,263	6,220	6,896
214881_s_at	6,322	5,742	6,286	6,043	5,504	5,940
214882_s_at	10,727	10,643	10,648	10,503	9,703	10,006
214883_at	6,695	6,588	6,465	7,575	7,582	7,641
214884_at	4,332	5,106	5,458	5,186	5,596	5,424
214885_at	7,646	7,541	7,692	7,953	7,648	7,516
214886_s_at	6,194	6,276	6,220	7,329	7,113	7,230
214887_at	4,056	4,923	4,081	4,292	4,343	4,244
214888_at	6,929	6,948	7,153	7,116	7,405	7,251
214889_at	7,169	7,808	7,674	7,112	6,447	6,845
214890_s_at	8,247	8,451	8,305	7,745	7,285	7,583
214891_at	7,219	7,406	7,576	7,206	7,334	7,434
214892_x_at	8,145	8,155	7,930	7,904	7,884	8,027
214893_x_at	7,817	7,783	7,891	8,335	8,330	8,613
214894_x_at	9,253	8,963	9,084	7,441	8,797	7,340

214895_s_at	7,713	7,836	6,972	6,426	7,421	6,593
214896_at	6,407	6,755	6,908	6,924	7,000	7,093
214897_at	5,480	4,758	5,174	4,512	4,578	4,522
214898_x_at	7,670	7,744	7,903	7,717	7,552	7,810
214899_at	3,610	3,787	3,746	3,604	3,470	3,626
214900_at	7,891	7,524	7,155	7,243	7,107	7,521
214901_at	8,807	9,079	9,297	8,231	8,186	8,173
214902_x_at	9,121	8,949	9,037	8,352	9,312	8,502
214903_at	8,939	8,795	9,047	8,907	8,984	8,837
214904_at	5,334	5,242	5,121	5,107	5,053	5,025
214905_at	6,266	6,438	6,472	6,673	6,567	6,711
214906_x_at	6,937	7,495	6,900	7,391	7,172	7,317
214907_at	5,423	5,446	5,484	6,263	6,209	6,287
214908_s_at	6,957	6,725	6,985	6,021	5,532	6,078
214909_s_at	8,395	8,115	7,843	7,899	7,960	7,970
214910_s_at	6,550	6,953	6,884	6,941	6,625	7,037
214911_s_at	10,252	10,300	10,356	9,858	9,892	10,535
214912_at	5,658	5,308	5,533	5,306	5,491	5,291
214913_at	6,952	7,034	6,598	4,632	4,960	5,076
214914_at	3,990	3,947	3,934	4,141	4,078	4,035
214915_at	6,790	6,695	6,665	6,638	6,282	6,442
214916_x_at	6,682	6,772	6,859	7,008	6,914	6,988
214917_at	6,978	6,641	6,860	4,539	6,397	5,063
214918_at	5,781	6,110	6,072	4,913	4,803	4,829
214919_s_at	9,524	9,105	9,250	8,774	8,942	8,612
214920_at	6,653	5,460	5,022	4,011	3,979	3,984
214921_at	5,688	5,581	5,646	5,824	5,777	5,548
214922_at	6,774	6,254	6,039	5,989	5,916	6,232
214923_at	6,796	6,479	6,661	6,642	6,588	6,418
214924_s_at	8,368	8,277	8,480	8,911	9,580	9,125
214925_s_at	6,753	6,472	6,688	6,881	6,888	6,549
214926_at	6,291	6,638	6,975	7,174	6,451	7,135
214927_at	5,740	5,880	5,968	8,938	11,738	9,630
214928_at	6,106	6,361	6,344	6,803	6,849	6,884
214929_s_at	6,681	6,433	6,556	6,956	6,817	6,818
214930_at	9,885	9,705	9,687	6,160	6,626	6,099
214931_s_at	7,227	7,421	7,128	7,207	7,075	7,159
214932_at	4,812	4,624	4,745	5,616	5,249	5,300
214933_at	8,202	8,334	7,873	7,928	7,447	7,631
214934_at	7,730	7,871	8,151	7,376	7,659	7,245
214935_at	6,630	6,618	6,600	7,614	7,574	7,798
214936_at	5,416	5,601	5,245	4,828	4,882	4,898
214937_x_at	10,687	10,677	10,346	8,611	10,137	7,606
214938_x_at	13,871	13,833	13,672	12,816	13,037	12,896
214939_x_at	6,463	7,012	6,855	7,637	7,394	7,576
214940_s_at	7,476	7,256	7,636	7,032	6,819	6,918
214941_s_at	9,611	9,200	8,975	8,792	8,468	8,524
214942_at	4,316	4,815	4,459	5,663	5,275	5,489
214943_s_at	8,138	7,783	7,722	6,985	6,468	6,714
214944_at	6,418	6,343	6,180	6,285	5,713	6,128
214945_at	6,240	6,307	6,327	5,999	6,104	6,040
214946_x_at	8,635	8,726	8,890	8,375	9,170	8,535
214947_at	6,108	5,908	6,015	6,246	6,074	6,115
214948_s_at	11,914	11,785	12,236	9,518	10,544	9,787
214949_at	11,200	11,014	11,421	8,584	9,776	9,022
214950_at	8,003	7,861	7,943	7,905	7,853	8,022

214951_at	8,847	8,340	8,405	7,391	7,541	7,545
214952_at	6,800	6,550	6,775	7,478	7,582	7,764
214953_s_at	10,452	10,345	10,142	8,938	9,785	9,418
214954_at	5,466	5,467	5,581	4,964	6,628	5,535
214955_at	7,866	7,647	7,746	7,923	7,618	7,901
214956_at	5,573	5,821	6,104	5,840	6,235	6,099
214957_at	7,702	7,581	7,668	7,809	7,771	7,359
214958_s_at	7,294	7,311	7,162	7,408	7,482	7,666
214959_s_at	8,017	7,696	7,458	6,591	6,749	6,309
214960_at	8,491	8,573	8,172	8,880	9,007	8,161
214961_at	4,559	4,688	4,721	5,214	4,852	5,019
214962_s_at	6,798	6,190	6,729	6,158	5,775	5,906
214963_at	7,297	6,694	7,429	7,317	6,534	6,672
214964_at	6,759	7,269	7,017	7,677	7,902	7,734
214965_at	8,223	7,812	8,082	8,443	8,232	8,562
214966_at	6,213	6,680	5,964	7,041	7,188	7,026
214967_at	5,419	5,015	4,394	5,314	5,841	5,077
214968_at	7,125	6,879	6,911	7,181	7,169	7,448
214969_at	6,986	7,041	7,110	7,296	7,233	7,406
214970_s_at	5,156	5,126	5,476	5,228	5,234	5,378
214971_s_at	5,510	5,376	5,372	5,300	5,236	5,175
214972_at	6,456	6,465	6,508	6,577	6,331	6,417
214973_x_at	6,956	6,462	6,971	8,213	8,303	8,408
214974_x_at	4,640	5,083	4,426	13,699	12,256	12,359
214975_s_at	5,003	4,688	5,308	4,833	4,530	4,547
214976_at	6,993	6,930	7,181	6,903	7,007	7,027
214977_at	5,628	6,012	6,061	5,944	5,690	5,880
214978_s_at	6,811	6,664	6,329	6,458	6,773	6,471
214979_at	6,055	6,183	6,289	6,532	6,920	6,459
214980_at	7,320	7,312	7,397	7,312	7,560	7,531
214981_at	3,621	3,567	3,715	3,682	3,536	3,951
214982_at	4,414	4,333	4,387	4,379	4,236	4,434
214983_at	9,639	9,606	9,530	6,528	7,851	7,589
214984_at	4,855	4,326	4,384	3,805	3,764	3,878
214985_at	6,153	6,336	6,210	5,442	6,031	5,548
214986_x_at	8,661	8,697	8,868	8,693	8,564	8,763
214987_at	6,677	6,924	6,902	7,482	7,214	7,428
214988_s_at	12,834	12,719	12,726	10,874	12,121	11,156
214989_x_at	8,676	8,309	8,472	6,897	7,858	7,279
214990_at	7,511	7,673	7,805	8,681	8,222	8,542
214991_s_at	7,433	7,790	7,379	8,107	7,780	8,102
214992_s_at	6,921	7,087	7,000	7,402	7,280	7,477
214993_at	8,126	7,978	8,136	7,927	7,577	7,952
214994_at	6,885	6,819	7,376	8,869	8,794	8,821
214995_s_at	6,756	6,438	6,502	6,934	6,632	6,885
214996_at	7,714	7,395	8,068	7,073	7,174	7,407
214997_at	6,009	6,267	5,818	4,175	4,302	4,227
214998_at	4,896	5,512	5,004	5,039	5,010	5,080
214999_s_at	7,817	7,755	7,347	8,948	8,437	8,778
215000_s_at	11,723	12,122	11,684	11,849	12,308	12,497
215001_s_at	13,410	13,546	13,487	11,306	11,817	11,623
215002_at	4,066	4,123	4,288	4,866	4,970	4,554
215003_at	7,274	7,183	7,240	7,162	7,086	7,385
215004_s_at	9,650	9,016	9,429	8,373	7,939	8,532
215005_at	8,661	8,654	8,675	8,932	8,820	8,850
215006_at	9,314	9,098	9,448	7,517	7,763	7,419

215008_at	4,465	4,097	4,591	4,185	4,071	4,101
215009_s_at	11,113	11,229	10,316	8,544	10,143	7,864
215010_s_at	5,894	5,961	5,438	6,359	5,958	6,149
215011_at	8,820	8,522	9,020	6,954	6,584	6,998
215012_at	6,490	5,934	5,821	5,770	5,843	5,277
215013_s_at	5,744	5,602	5,712	5,802	5,861	5,689
215014_at	3,374	3,674	3,708	3,848	3,988	3,965
215015_at	6,831	6,629	6,811	7,451	7,439	7,691
215016_x_at	9,605	9,612	9,347	9,590	11,938	9,472
215017_s_at	11,942	12,051	11,470	6,332	7,717	6,842
215018_at	4,925	4,436	4,527	3,946	4,125	4,173
215019_x_at	7,305	7,562	7,693	7,016	6,981	7,578
215020_at	6,561	6,493	6,557	6,558	6,457	6,397
215021_s_at	6,433	6,516	6,578	7,292	7,401	7,945
215022_x_at	8,155	7,893	7,795	6,956	6,904	7,073
215023_s_at	8,351	8,836	7,889	6,563	6,992	6,048
215024_at	7,437	7,033	7,488	6,563	6,332	6,414
215025_at	7,212	7,153	7,262	7,385	7,199	7,224
215026_x_at	7,976	7,820	8,044	8,154	8,169	8,198
215027_at	6,842	6,579	6,864	7,357	6,813	7,014
215028_at	9,421	8,833	9,217	4,866	5,056	5,333
215029_at	8,498	8,362	7,988	4,514	4,423	4,945
215030_at	9,774	9,663	9,934	6,843	7,287	6,867
215031_x_at	8,682	8,534	8,523	8,671	7,954	8,505
215032_at	7,378	6,886	7,479	7,357	7,583	7,361
215033_at	4,684	5,092	4,794	5,680	6,092	5,550
215034_s_at	4,993	4,726	5,357	11,987	11,325	11,798
215035_at	6,417	6,288	6,492	7,104	6,977	6,802
215036_at	6,830	6,557	6,630	7,376	7,602	7,534
215037_s_at	6,323	5,913	6,766	7,053	6,686	7,186
215038_s_at	8,679	8,578	8,556	6,226	7,096	6,195
215039_at	5,573	5,428	5,270	6,094	5,671	6,047
215040_at	7,868	7,467	8,088	7,370	7,617	7,557
215041_s_at	5,442	5,320	5,247	5,570	5,377	5,728
215042_at	5,628	5,950	5,996	6,700	6,436	6,568
215043_s_at	5,045	4,944	4,494	5,112	5,021	4,806
215044_s_at	6,140	6,526	6,649	7,611	7,625	7,163
215045_at	6,344	6,552	6,742	5,860	5,693	5,599
215046_at	5,234	5,365	5,322	5,017	5,139	5,129
215047_at	4,450	4,880	4,725	3,647	3,776	3,587
215048_at	7,646	7,858	7,507	7,711	7,610	7,716
215049_x_at	6,022	6,365	5,981	6,369	6,182	6,224
215050_x_at	7,180	6,774	7,243	7,654	7,279	7,417
215051_x_at	7,902	7,942	7,887	7,453	7,208	7,664
215052_at	6,244	6,140	6,017	6,205	6,447	6,218
215053_at	5,979	5,711	5,835	6,090	6,186	6,079
215054_at	6,770	7,278	7,059	7,083	6,453	7,059
215055_at	7,323	7,411	7,101	8,456	8,719	8,737
215056_at	7,094	6,873	7,349	6,700	6,325	6,664
215057_at	6,868	7,126	6,984	7,318	7,025	6,966
215058_at	9,042	8,671	8,198	5,652	5,957	5,749
215059_at	5,126	5,335	5,091	6,486	6,452	6,118
215060_at	5,514	5,595	5,941	5,867	5,907	5,867
215061_at	6,994	6,817	7,165	7,807	7,799	8,011
215062_at	8,893	8,299	7,988	6,973	6,885	6,423
215063_x_at	7,613	7,466	7,624	6,985	7,499	6,898

215064_at	4,640	4,707	4,696	5,490	4,840	4,979
215065_at	4,811	4,975	5,230	4,973	4,968	4,987
215066_at	7,036	7,034	7,078	7,266	7,099	7,236
215067_x_at	9,332	9,001	9,082	7,707	8,564	8,070
215068_s_at	6,990	6,745	7,280	6,637	6,380	6,704
215069_at	6,265	6,386	6,171	6,092	6,272	6,163
215070_x_at	6,076	5,751	5,771	5,502	5,458	5,540
215071_s_at	9,348	9,863	10,030	8,192	9,481	9,677
215072_x_at	6,310	6,610	6,453	6,346	6,565	6,492
215073_s_at	4,122	4,008	4,529	7,696	7,698	8,093
215074_at	5,429	5,418	5,478	5,657	5,560	5,673
215075_s_at	8,964	8,899	9,292	10,349	9,462	10,445
215076_s_at	5,075	4,833	4,969	9,392	12,815	11,480
215077_at	6,402	6,398	6,331	6,730	6,494	7,070
215078_at	4,504	4,238	4,152	6,052	5,591	5,298
215079_at	6,357	6,102	6,346	6,616	6,517	6,596
215080_s_at	5,709	5,015	5,899	6,598	6,104	6,432
215081_at	5,707	5,355	5,537	5,129	5,145	5,132
215082_at	6,300	7,024	6,666	7,750	7,289	7,494
215083_at	8,228	7,601	8,148	5,681	6,719	5,545
215084_s_at	9,285	9,620	9,374	8,513	8,029	8,532
215085_x_at	7,792	7,760	7,481	7,818	7,251	7,503
215086_at	3,720	3,759	4,081	3,804	3,851	3,961
215087_at	7,290	6,744	7,649	7,367	7,238	7,291
215088_s_at	11,359	11,360	11,559	11,264	11,178	11,539
215089_s_at	10,557	10,274	10,759	9,415	8,547	9,012
215090_x_at	8,641	8,714	8,563	9,299	10,024	9,778
215091_s_at	12,783	12,989	13,035	12,590	12,656	12,600
215092_s_at	7,887	8,260	7,718	6,424	7,128	6,132
215093_at	8,893	8,887	9,260	10,376	9,712	10,105
215094_at	5,278	5,063	5,349	4,962	4,660	4,680
215095_at	4,969	4,683	4,311	4,376	4,628	4,335
215096_s_at	10,838	11,314	10,875	12,400	12,543	12,613
215097_at	5,305	5,571	5,278	5,589	5,447	5,330
215098_at	6,562	6,495	6,855	6,730	6,584	6,802
215099_s_at	5,152	5,386	5,290	5,532	5,277	5,481
215100_at	5,756	5,426	5,510	6,263	6,234	6,021
215101_s_at	4,292	4,130	4,076	11,955	6,297	7,431
215102_at	5,423	5,719	5,498	5,576	5,359	5,305
215103_at	4,541	4,396	4,358	4,465	4,431	4,513
215104_at	6,770	7,071	6,464	7,507	7,488	7,561
215105_at	6,477	6,856	6,560	5,813	5,725	5,638
215106_at	5,971	5,990	5,719	6,514	6,272	6,634
215107_s_at	4,749	4,851	4,638	5,111	5,106	5,525
215108_x_at	9,588	9,631	9,908	4,111	3,997	4,032
215109_at	6,569	6,272	6,874	5,253	5,802	5,242
215110_at	6,389	6,226	6,249	7,031	6,966	7,159
215111_s_at	12,643	12,994	12,818	11,729	12,755	12,366
215112_x_at	3,521	3,682	3,693	3,380	3,467	3,499
215113_s_at	7,601	7,500	7,322	7,027	6,610	6,896
215114_at	7,469	6,853	7,310	6,551	6,782	6,767
215115_x_at	6,522	6,624	6,265	7,069	6,789	7,091
215116_s_at	9,704	9,572	9,509	7,130	6,956	7,089
215117_at	3,855	3,745	4,026	4,033	4,003	4,241
215118_s_at	5,686	5,068	5,464	6,264	6,383	6,396
215119_at	6,291	6,410	6,276	6,522	6,681	7,042

215120_s_at	5,260	5,197	5,280	5,723	5,779	5,662
215121_x_at	7,568	7,755	7,608	7,790	8,112	8,146
215122_at	5,840	5,938	6,233	6,872	6,944	6,898
215123_at	8,466	7,681	8,330	6,830	7,878	6,234
215124_at	5,784	5,718	5,949	6,352	6,351	6,485
215125_s_at	6,474	6,438	6,631	6,660	6,681	6,640
215126_at	5,305	5,351	5,562	5,754	5,660	5,701
215127_s_at	11,432	11,343	11,093	10,859	12,276	11,315
215128_at	9,261	8,670	9,412	6,409	6,622	6,299
215129_at	5,811	5,603	5,451	6,142	5,871	5,922
215130_s_at	7,680	7,107	7,377	7,431	7,026	7,440
215131_at	6,545	6,518	6,117	6,670	6,581	6,656
215132_at	4,522	4,706	4,472	4,666	4,415	4,397
215133_s_at	4,999	4,829	4,842	5,207	5,478	5,229
215134_at	6,871	6,817	7,308	7,712	7,336	7,584
215135_at	7,491	7,457	7,663	7,096	7,440	7,178
215136_s_at	11,062	11,374	10,701	10,363	9,957	9,409
215137_at	7,766	7,674	7,766	8,396	8,563	8,580
215138_s_at	7,203	6,721	6,563	7,290	7,127	7,489
215139_at	5,910	5,476	5,326	4,702	4,666	4,721
215140_at	7,157	7,186	6,848	7,486	7,443	7,579
215141_at	6,561	6,311	6,806	7,118	7,160	7,469
215142_at	6,189	6,533	6,440	6,667	6,679	6,403
215143_at	7,508	7,147	6,841	5,250	5,271	5,081
215144_at	5,974	6,142	5,844	6,764	6,652	7,036
215145_s_at	8,306	8,271	8,220	6,380	6,091	6,075
215146_s_at	8,468	8,592	8,971	5,445	8,152	6,736
215147_at	6,635	6,576	6,899	6,991	6,872	7,088
215148_s_at	8,048	8,147	8,593	8,747	8,456	8,917
215149_at	6,171	6,643	6,925	6,785	6,781	6,750
215150_at	6,573	6,059	6,129	5,048	4,617	4,718
215151_at	5,115	4,853	4,778	5,553	6,608	5,730
215152_at	3,976	4,198	4,259	4,129	4,149	4,122
215153_at	8,223	8,094	8,237	8,727	8,545	8,415
215154_at	6,424	7,047	6,628	6,722	6,315	6,657
215155_at	7,856	8,037	7,913	7,712	8,105	7,913
215156_at	7,298	7,470	7,220	7,254	7,368	7,218
215157_x_at	13,942	13,937	14,054	12,786	13,286	13,225
215158_s_at	10,049	9,975	10,203	9,222	8,717	9,124
215159_s_at	7,154	7,126	6,579	7,383	7,273	7,625
215160_x_at	9,041	9,408	8,573	9,217	8,378	8,455
215161_at	6,833	7,233	6,847	7,188	6,722	6,946
215162_at	5,270	4,899	5,323	5,484	5,670	5,470
215163_at	7,346	6,842	7,152	5,779	6,189	6,132
215164_at	5,334	5,611	5,791	5,130	4,944	5,220
215165_x_at	11,069	10,902	11,237	10,781	10,019	10,303
215166_at	5,596	5,787	5,906	6,484	6,486	6,549
215167_at	7,315	8,084	7,669	6,884	6,691	6,928
215168_at	3,535	3,526	3,663	3,807	3,667	3,708
215169_at	8,240	8,138	8,012	6,318	6,883	7,116
215170_s_at	6,500	6,239	5,419	5,380	5,848	5,489
215171_s_at	12,818	13,001	12,860	12,617	12,990	12,891
215172_at	5,382	5,243	5,183	5,778	6,819	6,530
215173_at	6,655	6,414	6,304	7,278	7,269	7,307
215174_at	5,295	5,436	5,971	5,984	5,756	5,662
215175_at	6,028	6,062	5,816	4,705	5,719	4,836

215176_x_at	5,632	5,488	5,034	5,814	6,082	6,258
215177_s_at	8,907	8,970	8,574	6,755	6,436	6,594
215178_x_at	7,765	7,721	7,779	7,882	7,511	7,795
215179_x_at	10,287	9,991	10,136	9,011	10,253	9,297
215180_at	7,040	7,003	6,736	7,318	7,369	7,229
215181_at	7,228	7,322	7,472	7,875	7,837	7,932
215182_x_at	6,728	6,745	6,795	7,404	7,314	7,796
215183_at	5,811	6,473	6,108	6,583	6,494	6,811
215184_at	7,170	7,205	6,719	6,883	6,788	6,487
215185_at	5,334	5,019	4,702	6,219	5,797	6,298
215186_at	6,168	6,074	6,345	7,087	6,920	7,132
215187_at	4,362	4,286	4,226	4,491	4,658	4,692
215188_at	6,592	5,837	6,581	5,750	6,637	6,086
215189_at	5,753	6,315	6,248	6,354	6,272	6,084
215190_at	7,394	7,950	7,986	5,261	5,125	5,212
215191_at	7,443	5,892	6,961	4,420	5,437	4,615
215192_at	4,793	4,660	4,819	6,245	5,386	5,487
215193_x_at	6,458	6,477	6,598	7,692	7,284	7,615
215194_at	6,405	5,993	6,744	6,961	7,055	7,072
215195_at	7,346	7,521	7,720	8,558	8,412	8,400
215196_at	6,985	6,909	6,995	7,083	7,272	6,976
215197_at	5,189	4,984	5,337	5,239	5,347	5,356
215198_s_at	7,538	7,072	7,266	7,881	8,097	7,756
215199_at	6,052	6,258	6,298	6,955	6,638	6,610
215200_x_at	8,710	8,860	8,956	8,418	8,818	8,603
215201_at	5,002	4,654	5,008	4,649	5,015	4,776
215202_at	7,886	7,739	7,877	8,175	8,082	8,115
215203_at	7,703	7,806	7,500	6,618	8,156	6,640
215204_at	6,674	6,439	7,159	5,221	6,385	5,057
215205_x_at	7,095	6,936	6,900	7,321	7,302	7,322
215206_at	7,984	7,696	7,919	7,411	8,875	6,927
215207_x_at	7,612	7,659	7,083	7,801	7,641	7,600
215208_x_at	8,124	7,953	8,259	8,099	8,849	8,400
215209_at	5,265	5,687	5,501	5,472	7,314	5,784
215210_s_at	8,262	8,311	8,450	8,703	8,577	8,697
215211_at	7,445	7,111	7,280	5,555	5,887	5,662
215212_at	4,080	3,928	3,652	4,002	4,031	4,211
215213_at	3,775	3,857	4,034	3,633	3,908	3,693
215214_at	7,279	7,674	7,298	8,079	8,226	8,526
215215_s_at	7,950	8,325	7,608	7,457	7,248	7,497
215216_at	6,368	6,199	6,011	6,133	6,505	6,255
215217_at	7,790	7,828	7,768	8,123	8,030	8,187
215218_s_at	7,930	7,795	7,876	8,442	8,324	8,587
215219_at	4,826	4,472	4,801	5,064	4,968	4,933
215220_s_at	7,758	7,616	6,799	5,765	5,574	5,435
215221_at	8,474	8,115	8,218	8,185	7,804	7,832
215222_x_at	9,157	8,856	8,869	7,787	8,387	7,760
215223_s_at	10,107	9,923	9,990	11,498	8,871	9,419
215224_at	6,801	6,661	6,796	5,447	5,703	5,740
215225_s_at	6,022	5,669	6,089	6,547	6,345	6,278
215226_at	6,567	6,488	6,365	6,430	6,405	6,530
215227_x_at	11,199	11,382	11,238	11,255	10,884	11,234
215228_at	6,918	7,040	6,986	4,728	4,697	4,692
215229_at	4,794	5,192	4,880	4,642	4,560	4,700
215230_x_at	12,774	12,647	13,208	12,370	12,501	12,389
215231_at	4,526	4,336	4,605	4,606	4,817	4,587

215232_at	7,018	6,948	7,230	6,946	7,321	7,062
215233_at	6,500	6,481	6,803	7,265	7,048	7,212
215234_at	6,186	6,514	6,681	6,220	6,474	6,391
215235_at	10,157	10,259	10,535	8,633	10,183	9,569
215236_s_at	7,003	6,857	6,462	6,010	6,611	6,342
215237_at	5,539	5,826	6,009	6,309	6,142	6,177
215238_s_at	6,567	7,160	6,942	7,518	7,668	7,479
215239_x_at	8,287	8,525	7,920	4,758	5,486	4,979
215240_at	4,026	4,383	4,117	4,849	4,374	4,352
215241_at	5,243	4,673	5,422	5,454	5,779	5,326
215242_at	5,417	5,187	4,998	5,983	5,686	6,067
215243_s_at	7,659	7,376	7,543	8,167	8,315	8,361
215244_at	7,002	7,150	6,820	7,541	7,715	8,072
215245_x_at	9,285	9,334	9,126	8,787	9,434	7,686
215246_at	5,910	5,670	6,028	5,590	5,671	5,491
215247_at	6,154	6,051	6,289	7,092	6,821	6,989
215248_at	7,890	7,331	7,479	6,787	6,619	6,671
215249_at	4,727	5,320	4,757	5,066	4,718	4,821
215250_at	5,324	4,893	5,213	5,149	5,014	5,157
215251_at	6,646	6,293	6,129	7,081	6,798	6,983
215252_at	8,255	7,461	8,571	6,414	7,406	6,156
215253_s_at	7,204	6,792	7,046	7,665	6,897	6,939
215254_at	4,978	5,142	5,188	5,567	5,816	5,441
215255_at	6,426	6,070	6,542	7,128	7,287	7,409
215256_x_at	6,940	6,905	7,202	7,796	7,373	7,337
215257_at	4,217	4,526	4,450	4,735	4,451	4,321
215258_at	6,819	6,817	6,886	7,425	7,310	7,550
215259_s_at	5,809	5,855	5,817	5,825	5,970	5,790
215260_s_at	8,434	8,409	8,703	8,024	7,710	7,728
215261_at	6,967	6,862	7,323	7,513	7,240	7,715
215262_at	5,406	5,596	4,965	4,338	4,520	4,201
215263_at	6,630	7,268	7,314	6,837	6,876	7,020
215264_at	5,824	5,968	6,078	6,589	6,023	6,131
215265_at	7,129	7,232	7,494	7,934	7,768	8,021
215266_at	7,335	7,340	7,504	7,966	7,446	7,347
215267_s_at	7,188	7,006	7,280	7,443	7,152	7,271
215268_at	7,157	6,202	7,314	4,902	5,401	5,292
215269_at	8,506	7,743	8,289	6,349	6,897	6,349
215270_at	7,081	7,352	6,956	6,987	6,757	6,942
215271_at	6,411	6,563	6,532	6,179	5,940	5,988
215272_at	6,624	7,180	6,500	7,116	7,056	6,997
215273_s_at	9,709	9,682	9,841	9,430	9,021	9,359
215274_at	6,584	6,886	6,925	7,328	7,096	7,138
215275_at	6,022	6,286	5,947	6,142	6,269	6,278
215276_at	6,937	6,832	7,646	8,215	7,995	8,111
215277_at	6,643	6,075	6,485	6,401	6,172	6,277
215278_at	6,113	6,093	6,029	6,939	6,875	6,469
215279_at	6,538	6,293	6,521	7,205	6,768	6,896
215280_s_at	8,292	8,517	8,064	7,117	6,908	7,138
215281_x_at	7,333	7,104	7,367	6,581	6,910	6,684
215282_at	4,513	4,819	5,099	4,968	4,825	4,835
215283_at	8,512	7,943	8,356	7,216	7,953	7,562
215284_at	5,698	5,787	6,174	4,860	6,643	4,961
215285_s_at	8,535	8,330	8,182	7,729	7,600	7,487
215286_s_at	6,724	7,490	6,328	5,905	6,070	5,971
215287_at	9,239	8,991	8,867	6,780	7,909	6,592

215288_at	6,426	6,097	6,895	6,977	7,153	7,221
215289_at	4,357	4,247	4,426	5,042	4,875	5,094
215290_at	4,573	4,314	4,780	5,188	4,929	5,378
215291_at	6,457	6,765	6,659	8,065	7,841	8,233
215292_s_at	7,703	7,391	7,822	8,523	8,731	8,794
215293_s_at	9,667	9,421	10,165	9,145	8,965	9,378
215294_s_at	8,824	8,830	7,726	5,769	6,406	6,236
215295_at	5,322	5,260	5,140	5,479	5,595	5,449
215296_at	7,930	7,425	7,927	7,260	7,794	7,270
215297_at	7,348	7,144	6,135	5,592	5,128	5,366
215298_at	6,772	6,472	6,654	6,789	7,097	6,871
215299_x_at	8,491	8,638	8,512	7,161	7,683	7,875
215300_s_at	4,618	5,135	5,401	4,279	4,314	4,628
215301_at	4,901	4,236	4,555	3,581	3,665	3,604
215302_at	5,834	5,755	5,949	5,631	5,834	6,026
215303_at	8,396	8,524	8,396	4,687	4,998	4,979
215304_at	7,620	7,671	7,565	7,174	7,922	7,076
215305_at	7,741	7,677	8,611	8,137	8,002	8,427
215306_at	9,040	8,919	8,672	5,577	6,613	5,661
215307_at	7,814	7,877	7,424	5,122	5,348	5,483
215308_at	6,026	5,098	6,123	4,965	5,195	5,014
215309_at	3,474	3,404	3,466	3,308	3,255	3,357
215310_at	6,783	6,708	6,674	6,243	6,506	6,460
215311_at	4,736	5,234	4,966	5,353	5,307	5,431
215312_at	3,827	3,754	3,672	4,016	3,936	3,842
215313_x_at	11,644	12,042	12,040	11,991	11,917	12,507
215314_at	6,522	6,886	5,945	4,727	6,631	4,657
215315_at	4,733	4,267	4,481	4,730	4,518	4,563
215316_at	5,198	5,402	4,766	4,679	4,713	4,666
215317_at	5,344	5,651	5,557	6,176	5,717	5,848
215318_at	5,063	4,994	5,154	4,918	4,935	4,975
215319_at	5,615	5,391	5,292	5,958	5,725	5,704
215320_at	6,284	5,888	6,221	6,504	6,465	6,556
215321_at	5,676	6,300	5,569	5,209	4,919	5,004
215322_at	9,130	9,177	8,498	5,800	6,281	6,099
215323_at	7,315	7,583	7,313	8,228	8,235	7,938
215324_at	4,869	5,411	5,242	6,081	6,000	6,145
215325_x_at	6,381	6,311	6,210	7,742	8,079	8,254
215326_at	5,973	5,569	6,040	6,214	5,914	5,995
215327_at	5,947	5,969	6,371	6,501	6,280	6,353
215328_at	7,675	7,386	7,528	7,620	7,953	7,585
215329_s_at	8,342	8,352	8,432	8,635	8,546	8,732
215330_at	4,489	4,399	4,420	4,121	5,633	4,107
215331_at	5,380	5,425	5,434	6,124	6,241	6,241
215332_s_at	4,708	4,591	4,685	4,777	4,724	4,821
215333_x_at	8,866	8,934	9,008	9,062	8,932	8,844
215334_at	7,122	6,972	6,971	7,658	7,401	7,407
215335_at	6,686	6,653	6,861	7,195	7,272	7,139
215336_at	6,689	6,453	6,663	6,990	6,997	7,019
215337_at	6,175	5,879	5,913	6,272	6,084	6,256
215338_s_at	9,535	9,412	8,874	6,759	8,757	7,372
215339_at	4,717	5,111	4,677	4,248	4,636	4,416
215340_at	6,649	6,512	6,299	6,726	6,782	7,093
215341_at	7,414	7,144	7,340	8,062	8,086	8,254
215342_s_at	6,407	6,821	6,427	5,483	5,538	5,513
215343_at	4,888	5,369	5,242	4,841	4,528	4,645

215344_at	7,325	7,307	7,315	7,750	7,521	7,495
215345_x_at	6,667	6,857	7,089	7,372	7,261	7,054
215346_at	7,526	7,126	7,761	7,433	7,494	7,786
215347_at	5,557	5,468	5,775	6,078	6,150	6,517
215348_at	6,904	6,601	6,726	7,122	7,513	7,395
215349_at	5,483	6,033	5,425	4,477	4,754	4,624
215350_at	3,862	3,972	4,092	4,154	4,281	4,273
215351_at	5,257	5,326	4,846	4,592	5,187	4,573
215352_at	3,990	4,540	4,503	4,632	4,481	4,871
215353_at	7,836	7,605	8,000	8,779	8,936	8,992
215354_s_at	8,388	8,531	8,750	8,956	8,952	9,187
215355_at	4,191	4,539	4,476	4,482	4,374	4,518
215356_at	11,916	11,833	12,124	7,046	6,777	6,926
215357_s_at	7,300	6,874	7,021	7,105	6,981	7,214
215358_x_at	7,255	7,012	7,240	6,510	7,003	6,664
215359_x_at	9,084	9,250	8,740	6,831	8,239	6,875
215360_at	6,386	6,234	6,139	6,309	6,271	6,409
215361_at	4,338	4,561	4,230	4,453	4,298	4,343
215362_at	6,431	6,367	6,375	6,848	6,847	7,020
215363_x_at	6,591	6,942	6,134	6,811	6,299	6,462
215364_s_at	7,830	7,686	8,062	7,627	7,497	7,649
215365_at	6,597	6,465	6,490	6,507	6,465	6,343
215366_at	6,761	6,771	7,262	7,296	7,276	7,340
215367_at	7,412	7,406	7,420	7,674	7,802	7,780
215368_at	5,312	5,295	5,212	5,628	5,654	5,696
215369_at	6,747	6,961	6,714	6,993	7,054	7,207
215370_at	6,968	6,886	6,926	7,428	6,879	7,257
215371_at	7,110	6,703	7,107	7,306	7,445	7,693
215372_x_at	8,777	8,420	8,586	8,055	8,709	8,587
215373_x_at	9,782	9,344	9,525	8,852	10,092	9,380
215374_at	6,413	5,789	6,100	5,254	5,552	5,381
215375_x_at	5,134	5,618	5,404	5,459	6,151	5,622
215376_at	6,814	6,690	6,916	6,894	6,472	6,838
215377_at	5,933	5,804	6,241	6,477	6,829	6,663
215378_at	3,919	4,199	4,070	4,925	4,734	5,050
215379_x_at	7,161	7,261	7,134	7,535	7,641	7,679
215380_s_at	13,259	13,471	13,194	12,208	11,799	12,003
215381_at	6,688	6,827	6,663	6,994	7,103	6,945
215382_x_at	7,171	6,737	6,972	7,770	7,835	7,946
215383_x_at	8,848	8,695	8,772	8,761	8,988	8,499
215384_s_at	5,893	5,488	5,693	7,821	8,361	8,160
215385_at	7,071	6,576	6,502	5,249	7,061	5,619
215386_at	8,082	7,648	8,111	6,843	6,953	6,679
215387_x_at	8,303	7,831	8,188	7,621	8,432	7,989
215388_s_at	5,438	5,178	5,457	6,739	9,497	7,934
215389_s_at	7,010	6,671	6,925	7,253	7,266	7,524
215390_at	7,151	6,029	6,251	5,066	5,011	5,177
215391_at	5,284	4,953	5,397	5,132	5,397	5,469
215392_at	6,228	5,851	6,027	3,780	5,846	4,004
215393_s_at	5,829	5,672	5,866	6,075	5,827	6,232
215394_at	7,004	7,236	6,881	7,362	6,859	7,191
215395_x_at	7,161	7,175	7,222	8,805	8,027	8,188
215396_at	4,956	5,759	5,414	4,558	4,436	4,330
215397_x_at	7,045	6,973	7,324	7,205	7,378	7,133
215398_at	7,810	7,762	7,805	7,863	8,122	7,646
215399_s_at	9,469	9,299	9,156	8,698	8,848	8,863

215400_x_at	7,269	7,300	7,303	7,462	7,230	7,578
215401_at	7,402	7,274	7,513	7,274	7,540	7,322
215402_at	5,438	5,291	5,058	5,598	5,558	5,855
215403_at	6,299	6,292	6,311	6,836	6,838	7,208
215404_x_at	8,318	7,902	8,139	7,674	8,188	7,753
215405_at	4,320	4,203	4,425	4,627	4,432	4,736
215406_at	5,970	5,912	5,945	6,103	6,326	6,728
215407_s_at	8,155	8,441	8,213	7,310	7,408	7,636
215408_at	4,956	5,331	5,325	5,372	5,244	5,420
215409_at	6,146	6,461	6,648	6,611	6,624	6,601
215410_at	6,047	5,764	6,249	7,746	7,906	8,069
215411_s_at	8,407	8,924	8,744	9,931	9,484	9,649
215412_x_at	7,410	7,274	7,573	7,325	6,931	7,342
215413_at	6,774	6,960	6,789	7,181	7,123	7,531
215414_at	6,495	6,407	6,431	7,057	6,838	6,781
215415_s_at	6,026	5,803	6,036	6,538	6,248	6,421
215416_s_at	10,647	10,415	10,892	10,816	9,560	10,316
215417_at	5,777	6,121	5,646	5,874	5,934	5,733
215418_at	6,363	6,268	6,333	6,885	7,434	7,206
215419_at	7,310	7,543	7,807	7,854	7,723	7,933
215420_at	8,229	7,799	7,711	8,776	8,607	8,783
215421_at	6,741	6,294	6,757	7,225	6,924	7,036
215422_at	5,006	4,575	4,692	4,783	4,604	4,815
215423_at	6,867	6,852	7,131	6,684	6,552	6,575
215424_s_at	9,645	9,395	8,987	7,940	7,882	7,928
215425_at	7,030	6,264	6,044	5,102	4,786	4,685
215426_at	6,839	5,982	6,585	7,553	7,479	7,666
215427_s_at	6,149	6,605	6,629	6,641	6,600	6,711
215428_at	7,118	6,646	6,926	6,716	7,347	7,074
215429_s_at	7,658	7,474	7,678	7,598	7,543	7,704
215430_at	5,610	5,952	5,828	6,527	6,447	6,384
215431_at	5,391	5,476	5,743	6,101	5,910	6,001
215432_at	6,642	6,396	6,904	6,860	6,543	7,071
215433_at	6,529	6,433	6,282	5,877	6,179	6,015
215434_x_at	9,287	9,212	9,005	9,615	9,917	9,635
215435_at	4,321	4,644	4,913	4,358	6,159	4,452
215436_at	5,860	6,244	5,976	5,618	5,633	5,686
215437_x_at	5,946	6,028	6,047	5,892	5,927	5,802
215438_x_at	12,336	12,244	12,343	12,148	12,231	12,112
215439_x_at	7,240	7,083	7,368	7,134	7,543	7,265
215440_s_at	11,506	11,280	10,829	8,120	6,299	6,533
215441_at	4,007	3,979	4,127	4,070	3,915	4,333
215442_s_at	5,468	5,809	5,747	6,781	6,127	6,538
215443_at	5,443	5,269	4,976	5,681	5,509	5,515
215444_s_at	6,924	6,480	6,565	7,001	6,967	7,113
215445_x_at	5,868	5,618	6,121	5,683	6,253	5,883
215446_s_at	6,099	6,793	5,996	8,571	11,359	11,761
215447_at	4,475	4,579	4,302	5,928	8,613	6,424
215448_at	5,908	5,934	6,010	6,346	6,563	6,838
215449_at	7,204	7,075	7,095	7,760	7,620	7,617
215450_at	8,037	8,304	8,311	7,551	7,420	8,021
215451_s_at	6,399	6,492	6,504	6,741	6,689	6,704
215452_x_at	10,092	9,785	9,867	9,565	8,953	9,628
215454_x_at	7,055	6,937	7,384	8,080	8,004	8,104
215455_at	7,322	6,811	7,347	6,101	6,349	5,737
215456_at	5,745	5,787	5,820	5,813	5,797	5,686

215457_at	3,844	3,782	3,656	3,709	3,957	3,926
215458_s_at	7,107	7,188	7,248	7,783	7,273	7,642
215459_at	5,268	5,264	5,578	6,148	6,353	5,834
215460_x_at	9,132	8,869	9,345	7,377	7,667	7,799
215461_at	7,281	7,435	7,215	8,065	7,901	8,055
215462_at	7,604	7,657	7,832	8,392	8,552	8,105
215463_at	5,198	5,275	5,321	5,475	5,503	5,503
215464_s_at	8,602	8,197	8,473	9,851	9,306	10,265
215465_at	4,256	4,181	4,346	4,714	4,532	4,985
215466_at	6,373	6,305	6,287	6,417	6,340	6,151
215467_x_at	8,219	8,066	8,077	7,076	7,772	7,507
215468_at	4,448	4,471	4,481	3,647	3,762	3,656
215469_at	4,768	5,390	3,850	3,825	3,779	3,906
215470_at	7,445	7,523	6,937	5,517	6,347	5,733
215471_s_at	8,201	8,061	7,910	8,189	8,323	8,173
215472_at	6,406	6,330	6,294	7,451	7,064	7,237
215473_at	5,334	5,055	4,931	4,447	4,319	4,644
215474_at	4,878	4,915	4,850	5,364	5,270	5,465
215475_at	3,989	4,312	4,227	4,388	4,435	4,673
215476_at	7,648	7,163	7,450	7,714	7,702	7,713
215477_at	5,124	5,182	5,253	5,150	5,061	5,056
215478_at	4,613	4,848	4,675	4,937	5,017	5,342
215479_at	8,028	7,719	7,901	7,576	7,423	7,488
215480_at	5,674	5,659	5,759	6,369	6,361	6,442
215481_s_at	6,677	5,967	5,738	6,089	6,132	6,417
215482_s_at	10,174	10,108	10,584	10,250	9,837	10,337
215483_at	6,390	6,131	5,156	4,222	5,769	4,303
215484_at	6,348	6,701	6,620	6,815	6,629	6,948
215485_s_at	6,653	6,844	6,761	7,494	7,225	7,266
215486_at	6,148	6,018	5,696	6,037	5,755	5,782
215487_x_at	6,471	6,335	6,490	6,860	6,996	7,028
215488_at	7,966	7,860	7,951	8,219	8,435	8,299
215489_x_at	7,581	7,236	7,661	8,271	7,995	7,991
215490_at	6,528	6,758	6,920	7,250	7,526	7,614
215491_at	8,014	8,092	8,232	7,391	7,154	7,302
215492_x_at	8,623	8,724	8,774	8,897	9,016	8,850
215493_x_at	9,151	9,050	9,035	8,878	9,148	9,023
215494_at	5,053	5,095	5,183	5,831	5,854	5,856
215495_s_at	6,049	6,131	6,263	7,797	7,484	7,759
215496_at	6,281	6,136	6,527	6,493	5,768	6,211
215497_s_at	8,085	8,528	8,389	7,921	7,874	8,340
215498_s_at	9,366	9,369	9,684	10,550	10,796	11,110
215499_at	10,112	10,295	10,604	11,125	11,608	11,537
215500_at	6,671	6,600	6,440	6,546	6,955	6,527
215501_s_at	6,914	7,492	6,903	7,749	7,757	7,930
215502_at	7,622	7,318	7,544	7,685	7,828	7,919
215503_at	5,137	5,369	5,472	5,826	5,951	5,741
215504_x_at	9,857	9,785	10,097	8,856	10,162	9,227
215505_s_at	7,566	7,890	7,311	6,905	6,599	7,379
215506_s_at	5,714	5,856	6,032	6,833	6,847	7,195
215507_x_at	6,749	6,538	6,726	7,143	7,420	7,182
215508_at	6,125	6,500	5,849	4,736	4,490	4,616
215509_s_at	8,732	8,719	8,138	6,829	6,653	6,624
215510_at	5,810	5,680	5,597	6,374	5,936	6,000
215511_at	7,089	7,270	6,693	7,332	7,277	7,577
215512_at	4,709	4,922	5,210	4,831	4,690	4,771

215513_at	6,517	5,724	5,458	5,814	6,935	6,024
215514_at	3,477	3,451	3,403	3,522	3,551	3,581
215515_at	8,405	7,930	8,116	7,811	7,893	7,750
215516_at	3,645	3,557	3,744	4,129	4,134	4,141
215517_at	7,287	7,106	6,752	6,800	6,717	6,520
215518_at	6,209	6,020	6,320	6,123	5,863	5,945
215519_x_at	7,850	8,003	7,818	8,019	7,940	8,324
215520_at	4,377	4,641	4,252	4,342	4,365	4,292
215521_at	6,551	6,715	6,455	6,548	6,442	6,518
215522_at	4,446	5,136	4,703	4,747	4,878	5,034
215523_at	5,055	5,758	4,947	5,051	4,555	4,845
215524_x_at	4,443	4,260	4,492	5,426	5,048	5,424
215525_at	7,353	6,942	7,060	6,792	6,386	6,550
215526_at	7,368	6,720	6,925	7,412	7,213	7,266
215527_at	6,719	6,579	6,598	7,311	7,488	7,486
215528_at	7,895	7,339	7,603	6,764	7,061	6,659
215529_x_at	10,527	10,132	10,078	9,812	10,281	9,741
215530_at	7,481	7,118	7,321	7,854	8,140	8,194
215531_s_at	5,007	4,452	4,641	6,165	5,924	6,264
215532_x_at	9,105	9,152	9,015	4,109	3,916	3,973
215533_s_at	7,923	8,032	8,199	8,026	7,748	7,903
215534_at	6,447	5,700	6,084	6,118	5,765	5,877
215535_s_at	9,456	9,202	9,661	8,905	8,536	8,910
215536_at	6,090	6,077	6,120	6,949	6,951	7,245
215537_x_at	8,564	8,235	7,991	8,146	8,048	8,504
215538_at	6,764	6,152	6,038	7,317	7,310	7,449
215539_at	4,194	4,339	4,313	4,565	4,636	4,711
215540_at	6,585	6,636	6,952	6,578	6,089	6,404
215541_s_at	9,212	8,986	9,220	9,691	9,454	9,458
215542_at	4,239	4,148	4,290	4,281	4,467	4,222
215543_s_at	8,886	8,688	8,855	8,421	7,787	7,988
215544_s_at	8,215	8,297	8,591	8,435	8,027	8,312
215545_at	6,767	6,089	6,501	6,059	6,166	6,056
215546_at	6,218	6,121	6,641	7,310	7,196	6,856
215547_at	5,854	6,158	5,695	6,206	6,120	6,347
215548_s_at	9,822	10,186	9,287	8,839	10,114	8,803
215549_x_at	8,018	8,159	8,116	6,953	7,416	6,836
215550_at	6,260	6,454	6,908	6,704	6,575	6,757
215551_at	5,020	5,015	5,162	4,951	4,949	4,993
215552_s_at	5,969	5,846	6,016	6,653	6,692	6,511
215553_x_at	8,860	8,415	8,673	8,226	9,022	8,191
215554_at	6,260	5,381	6,148	3,886	3,802	3,845
215555_at	7,067	7,193	6,936	5,086	5,115	5,093
215556_at	7,058	6,950	7,017	7,190	7,144	7,510
215557_at	6,464	6,451	6,150	7,105	6,816	6,729
215558_at	4,205	4,118	4,564	4,238	4,332	4,310
215559_at	6,117	5,877	5,620	6,045	6,118	6,089
215560_x_at	7,165	7,361	7,139	6,635	7,268	6,750
215561_s_at	5,852	5,779	5,490	7,613	7,512	7,655
215562_at	6,607	6,593	6,740	6,889	7,003	7,094
215563_s_at	4,750	4,857	4,411	4,878	4,738	4,884
215564_at	3,821	3,851	4,183	3,838	3,709	3,766
215565_at	5,644	5,485	5,331	5,968	6,009	6,005
215566_x_at	10,350	10,089	10,488	10,087	9,958	10,543
215567_at	7,103	6,776	6,630	4,763	5,388	5,057
215568_x_at	10,210	10,187	10,549	10,239	10,073	10,508

215569_at	5,770	5,934	5,913	5,971	5,933	6,020
215570_s_at	4,375	4,507	4,078	4,001	3,916	3,999
215571_at	5,075	4,880	4,724	4,880	4,610	4,860
215572_at	5,336	6,318	5,848	5,900	5,951	6,214
215573_at	4,749	4,707	4,777	4,842	4,990	4,849
215574_at	7,230	6,995	7,036	7,011	7,154	7,014
215575_at	5,326	5,678	5,591	4,839	5,528	4,836
215576_at	7,499	7,165	7,325	8,600	8,241	8,410
215577_at	7,894	7,251	7,563	6,145	6,729	6,087
215578_at	4,178	4,089	4,014	3,848	4,361	4,042
215579_at	7,653	7,372	7,809	8,358	7,975	7,760
215580_at	6,631	6,596	6,629	7,162	6,893	7,225
215581_s_at	4,940	5,274	5,358	5,322	5,211	5,108
215582_x_at	8,001	7,963	7,994	7,736	8,295	8,073
215583_at	7,173	7,216	7,446	7,886	7,769	7,965
215584_at	6,803	7,182	6,977	7,502	7,491	7,568
215585_at	4,504	4,418	4,702	4,752	4,928	4,669
215586_at	3,831	4,522	4,083	4,021	3,990	3,908
215587_x_at	7,205	6,952	7,109	7,008	7,480	7,457
215588_x_at	9,475	9,076	9,410	8,400	9,743	8,950
215589_at	5,029	4,828	5,135	5,015	5,318	5,325
215590_x_at	6,349	6,562	6,400	6,132	6,094	5,930
215591_at	3,809	3,892	4,102	4,273	4,411	4,366
215592_at	7,601	7,558	7,930	6,073	6,040	6,058
215593_at	6,213	6,237	6,428	6,342	6,169	6,363
215594_at	10,588	10,240	11,217	7,906	7,928	8,271
215595_x_at	8,893	8,545	8,641	9,175	9,550	9,232
215596_s_at	10,263	10,670	9,681	8,269	9,749	8,088
215597_x_at	6,009	5,655	6,213	5,609	5,808	5,433
215598_at	4,880	4,902	4,752	5,029	5,248	5,173
215599_at	6,139	5,776	5,138	4,188	4,743	4,256
215600_x_at	9,890	9,649	9,961	8,898	10,157	9,282
215601_at	4,955	4,888	4,972	5,219	5,175	5,202
215602_at	4,985	5,076	5,656	6,264	6,170	6,172
215603_x_at	7,783	8,056	7,933	7,752	7,561	7,791
215604_x_at	9,284	8,842	9,026	8,430	9,533	9,002
215605_at	7,609	7,773	7,887	7,591	7,801	7,765
215606_s_at	7,211	6,952	6,972	6,910	7,000	6,860
215607_x_at	7,891	7,986	8,038	7,581	7,992	7,579
215608_at	7,469	7,182	7,506	8,144	7,916	8,321
215609_at	8,158	7,817	8,204	8,667	9,321	9,083
215610_at	6,853	7,083	7,058	7,137	7,259	7,355
215611_at	5,923	5,315	5,909	4,634	4,727	4,700
215612_at	6,211	6,573	6,646	5,887	6,720	5,563
215613_at	6,898	6,689	6,456	6,756	7,618	6,909
215614_at	6,665	6,563	6,463	6,992	6,797	7,068
215615_x_at	6,890	6,561	7,236	6,700	6,842	6,519
215616_s_at	7,415	7,502	7,729	8,084	7,929	8,061
215617_at	3,992	4,549	3,881	4,896	5,746	5,618
215618_at	6,841	6,679	6,642	7,187	7,189	6,831
215619_at	3,937	3,742	3,392	4,595	4,323	4,472
215620_at	5,467	5,578	5,837	5,303	5,751	5,254
215621_s_at	6,944	6,930	7,066	7,363	6,920	7,374
215622_x_at	6,787	7,124	7,278	7,803	7,504	7,726
215623_x_at	7,456	6,969	5,943	5,339	6,126	5,148
215624_at	5,557	5,277	5,384	6,220	6,049	6,346

215625_at	6,057	6,028	6,183	6,619	6,744	7,122
215626_at	3,664	3,645	3,794	4,116	4,152	4,166
215627_at	4,318	4,278	4,666	4,564	4,905	4,688
215628_x_at	8,900	8,596	8,694	7,803	8,884	8,069
215629_s_at	9,382	8,713	9,180	8,391	8,392	8,137
215630_at	5,856	5,524	5,849	6,390	6,601	6,737
215631_s_at	8,850	8,456	9,031	9,424	8,757	9,210
215632_at	5,001	4,748	4,592	6,069	6,056	6,130
215633_x_at	6,244	6,087	6,398	6,808	6,786	7,006
215634_at	4,211	4,064	4,273	4,500	3,981	4,209
215635_at	6,385	6,614	6,565	5,002	5,405	5,237
215636_at	6,125	5,879	6,178	6,545	6,392	6,628
215637_at	6,246	6,132	6,246	6,530	6,131	6,229
215638_at	6,125	6,332	6,357	6,722	6,470	6,677
215639_at	5,315	5,402	5,361	5,558	5,292	5,718
215640_at	8,038	7,794	7,986	8,071	7,979	7,420
215641_at	4,435	4,220	4,636	4,686	5,013	4,631
215642_at	6,753	6,604	6,713	6,680	6,580	6,571
215643_at	4,191	4,176	4,229	4,333	4,076	4,156
215644_at	7,506	7,463	7,479	7,807	7,852	7,904
215645_at	7,455	7,003	7,295	8,061	8,118	7,916
215646_s_at	8,430	7,501	7,930	5,040	8,070	6,153
215647_at	3,634	3,744	3,706	3,810	3,857	4,045
215648_at	5,626	5,486	5,749	5,707	5,754	5,277
215649_s_at	7,402	7,435	7,198	8,037	7,257	7,697
215650_at	6,432	5,717	6,209	6,573	6,532	6,896
215651_at	6,267	6,925	6,783	7,124	7,246	7,511
215652_at	6,183	6,526	6,034	6,198	5,718	5,904
215653_at	3,673	3,540	3,727	3,492	3,590	3,719
215654_at	7,090	7,257	7,185	7,868	8,130	8,133
215655_at	3,314	3,509	3,574	3,464	3,395	3,350
215656_at	5,353	5,175	5,169	5,784	5,714	5,866
215657_at	3,795	3,742	3,604	3,931	3,909	3,894
215658_at	5,527	5,922	5,619	6,624	6,271	6,406
215659_at	5,887	5,749	5,674	6,149	5,727	5,782
215660_s_at	6,788	6,905	6,728	6,618	6,385	6,599
215661_at	5,630	6,193	6,011	6,281	6,263	6,541
215662_at	7,999	8,068	8,015	8,503	8,559	8,861
215663_at	3,835	3,843	4,077	4,368	4,195	4,358
215664_s_at	4,851	4,570	4,572	5,864	5,486	6,186
215665_at	4,749	4,787	5,074	5,595	5,793	5,459
215666_at	4,405	4,918	4,467	5,040	4,886	4,872
215667_x_at	9,704	9,397	9,624	9,106	8,619	9,202
215668_s_at	6,962	7,194	7,238	7,664	7,683	7,878
215669_at	6,842	6,670	6,791	6,719	6,665	6,699
215670_s_at	7,740	7,867	7,946	8,198	7,843	8,035
215671_at	3,964	3,803	3,558	3,830	3,618	3,699
215672_s_at	7,229	7,535	7,368	7,165	7,338	7,362
215673_at	3,922	3,846	3,936	4,049	4,135	3,963
215674_at	3,544	3,562	3,445	3,785	3,936	3,628
215675_at	7,012	6,499	6,842	7,619	7,238	7,416
215676_at	7,163	6,944	7,322	7,948	7,634	7,836
215677_s_at	6,861	6,788	7,025	7,793	7,891	8,177
215678_at	7,061	7,191	7,182	7,926	7,769	7,784
215679_at	4,579	4,599	4,637	4,978	4,899	5,120
215680_at	6,986	6,900	6,993	7,183	7,311	7,354

215681_at	6,734	6,311	6,778	6,881	6,637	6,656
215682_at	7,164	7,163	7,256	7,588	7,589	7,804
215683_at	7,592	7,375	7,808	7,680	7,763	7,790
215684_s_at	7,606	7,085	7,344	8,040	7,803	8,172
215685_s_at	7,529	7,296	7,530	8,205	8,359	8,434
215686_x_at	4,042	3,786	4,265	4,467	4,380	4,616
215687_x_at	6,808	6,551	6,569	7,166	6,799	6,939
215688_at	6,774	6,697	6,702	6,995	7,146	6,937
215689_s_at	7,660	7,664	7,666	8,272	8,084	8,230
215690_x_at	9,333	9,199	9,601	10,205	10,171	10,505
215691_x_at	12,458	12,779	12,252	12,435	12,068	12,379
215692_s_at	5,336	4,781	5,257	5,433	5,138	5,112
215693_x_at	10,938	10,809	11,245	10,665	10,765	10,659
215694_at	5,577	4,866	5,179	4,341	4,474	4,339
215695_s_at	10,467	10,424	10,697	7,258	6,916	7,188
215696_s_at	9,964	10,188	10,676	9,927	10,727	10,731
215697_at	7,680	7,713	7,590	8,551	8,572	8,689
215698_at	7,840	7,797	7,862	6,059	6,643	6,220
215699_x_at	8,062	8,107	8,191	8,373	8,550	8,482
215700_x_at	7,317	7,540	7,761	7,897	7,850	7,813
215701_at	7,435	7,508	7,586	7,578	7,626	7,422
215702_s_at	4,788	4,524	4,836	4,918	4,720	4,782
215703_at	4,379	4,604	4,436	4,687	4,714	4,566
215704_at	4,214	4,614	4,561	4,343	4,706	4,864
215705_at	8,095	7,948	8,425	8,161	8,033	8,330
215706_x_at	9,208	8,991	9,222	9,326	9,399	10,212
215707_s_at	7,754	7,720	7,511	8,314	8,163	8,364
215708_s_at	7,150	6,803	6,474	6,986	6,194	6,276
215709_at	7,319	7,055	7,240	7,961	7,907	8,141
215710_at	5,775	5,611	5,942	6,939	6,742	6,993
215711_s_at	8,653	8,961	8,490	6,613	6,400	6,595
215712_s_at	6,656	6,416	6,728	7,346	7,137	7,050
215713_at	4,374	4,038	4,172	4,637	4,360	4,558
215714_s_at	10,459	10,145	10,337	8,996	8,291	8,938
215715_at	7,505	7,209	7,237	7,093	7,333	7,617
215716_s_at	9,785	9,546	9,327	11,273	11,467	10,023
215717_s_at	5,579	6,942	6,127	4,876	5,145	5,326
215718_s_at	7,226	7,436	7,041	7,767	8,119	8,087
215719_x_at	4,043	4,265	4,146	5,561	6,990	6,304
215720_s_at	6,489	6,210	6,129	7,383	7,791	7,672
215721_at	6,023	6,012	6,110	6,146	5,817	5,938
215722_s_at	10,823	10,757	10,065	9,883	9,794	9,645
215723_s_at	4,680	4,657	4,716	5,285	5,568	5,133
215724_at	3,943	3,828	4,286	5,102	5,236	4,945
215725_at	6,987	6,864	7,040	8,260	8,547	8,428
215726_s_at	10,134	10,311	10,126	10,762	10,908	11,225
215727_x_at	6,396	6,254	6,318	6,905	6,596	6,444
215728_s_at	7,288	7,368	7,485	8,118	7,545	7,908
215729_s_at	4,380	3,836	4,011	4,701	4,747	4,954
215730_at	5,947	5,712	6,000	6,133	5,989	6,030
215731_s_at	9,742	9,781	9,288	5,958	6,756	5,364
215732_s_at	7,436	7,437	7,750	7,911	7,546	7,623
215733_x_at	6,162	6,390	6,274	6,098	6,100	6,099
215734_at	6,642	6,191	6,015	6,838	6,053	6,407
215735_s_at	8,436	8,202	8,639	7,096	7,008	7,672
215736_at	5,471	5,554	5,708	5,502	5,539	5,220

215737_x_at	8,798	8,734	9,195	9,582	9,263	9,387
215738_at	6,508	6,196	6,363	6,884	6,464	6,611
215739_s_at	4,631	4,468	4,647	4,448	4,646	4,372
215740_at	6,710	6,579	6,561	6,764	6,831	6,890
215741_x_at	8,109	8,088	7,705	7,891	7,900	8,064
215742_at	4,938	5,376	5,600	5,781	5,825	5,561
215743_at	6,967	7,105	7,116	5,803	6,805	6,990
215744_at	7,054	7,841	7,654	7,286	7,126	7,291
215745_at	6,711	6,397	6,967	6,876	7,248	7,093
215746_at	6,235	5,953	6,106	6,506	6,418	6,600
215747_s_at	8,613	7,964	8,266	8,368	8,011	8,000
215748_at	6,758	6,707	6,798	6,817	6,677	6,818
215749_s_at	8,887	9,040	9,064	8,508	8,291	8,676
215750_at	4,970	4,755	5,390	4,459	4,545	4,542
215751_at	3,635	3,876	4,089	4,184	4,077	3,950
215752_at	5,258	5,425	5,263	5,560	5,291	5,484
215753_at	6,602	6,460	6,229	6,792	6,611	6,908
215754_at	7,393	7,249	7,410	6,958	7,720	7,386
215755_at	5,568	5,651	5,524	6,250	6,328	6,500
215756_at	6,762	6,702	6,761	7,183	6,663	6,791
215757_at	6,388	6,008	6,112	6,253	6,172	6,049
215758_x_at	9,675	9,637	9,201	6,997	7,072	6,801
215759_at	5,293	4,880	5,112	6,890	6,916	6,955
215760_s_at	7,511	7,303	7,707	8,328	8,460	8,766
215761_at	6,915	6,057	6,384	5,996	5,498	5,900
215762_at	5,159	5,373	5,593	5,862	5,994	5,764
215763_at	4,777	4,680	4,622	4,737	4,539	4,578
215764_x_at	8,526	8,434	8,426	8,372	8,376	8,631
215765_at	6,876	6,375	7,451	6,221	5,517	5,658
215766_at	7,126	7,616	7,224	6,936	6,893	6,626
215767_at	5,428	5,348	5,390	5,887	8,014	5,882
215768_at	9,180	7,942	8,764	4,873	5,338	4,764
215769_at	5,054	5,308	5,326	5,526	5,616	5,697
215770_at	3,924	3,979	3,939	3,999	3,844	3,980
215771_x_at	8,653	8,197	8,218	9,031	8,708	8,948
215772_x_at	10,387	10,707	10,012	10,358	10,430	10,548
215773_x_at	10,134	10,185	9,752	9,698	9,123	8,813
215774_s_at	4,659	4,519	4,689	4,898	4,805	4,507
215775_at	6,002	6,154	6,264	6,541	6,376	6,355
215776_at	6,071	6,163	6,318	6,924	6,795	7,340
215777_at	6,840	6,769	7,040	7,359	7,634	7,424
215778_x_at	8,480	8,614	9,049	10,068	10,519	10,385
215779_s_at	5,650	5,705	5,832	6,441	6,286	6,306
215780_s_at	9,811	9,013	9,086	6,591	6,088	7,020
215781_s_at	6,970	6,594	6,834	6,709	6,360	6,986
215782_at	6,583	6,343	6,358	6,367	6,106	6,135
215783_s_at	8,118	7,876	8,087	7,415	6,703	7,053
215784_at	4,845	4,815	4,895	4,978	4,878	4,928
215785_s_at	10,016	10,238	10,279	7,696	8,112	7,812
215786_at	6,060	5,208	5,949	4,414	4,591	4,351
215787_at	6,022	5,913	5,868	6,546	6,019	6,028
215788_at	7,787	7,787	7,838	8,447	8,115	8,400
215789_s_at	6,944	7,164	7,129	6,970	6,751	6,899
215790_at	5,501	4,748	6,035	4,926	4,940	5,005
215791_at	7,982	7,551	8,551	5,001	5,573	5,102
215792_s_at	10,335	10,132	10,478	9,834	9,656	9,804

215793_at	4,766	4,990	4,540	5,092	4,960	4,947
215794_x_at	9,164	8,996	8,799	8,306	8,201	8,563
215795_at	5,370	5,485	5,090	5,483	5,396	5,444
215796_at	4,567	4,636	4,588	5,163	4,606	4,553
215797_at	4,799	5,015	4,801	5,343	5,132	5,128
215798_at	7,422	7,729	7,803	8,269	8,243	8,205
215799_at	6,883	6,910	6,851	7,901	7,712	7,694
215800_at	5,775	5,393	5,785	6,347	6,267	6,244
215801_at	4,266	4,548	5,039	4,425	4,608	4,578
215802_at	7,411	7,759	7,793	6,042	6,142	6,057
215803_at	3,603	3,706	3,707	3,726	3,580	3,722
215804_at	7,317	7,195	7,400	8,325	8,126	8,307
215805_at	5,660	5,494	5,452	5,696	5,637	5,758
215806_x_at	5,374	5,237	5,739	5,909	5,841	5,958
215807_s_at	8,059	7,831	8,169	7,615	7,731	7,659
215808_at	6,282	6,395	6,032	6,215	6,295	6,301
215809_at	7,725	7,680	7,795	8,357	8,189	8,440
215810_x_at	5,054	5,198	5,594	5,206	5,379	5,103
215811_at	7,215	7,199	7,147	6,427	6,046	6,083
215812_s_at	8,990	8,823	9,214	8,954	8,652	8,981
215813_s_at	7,684	8,010	7,836	9,244	10,889	11,016
215814_at	4,853	4,696	4,968	5,129	4,976	5,026
215815_at	6,121	6,159	6,414	6,799	6,698	6,948
215816_at	6,477	6,701	6,921	6,570	6,914	6,956
215817_at	5,975	5,481	5,800	6,710	5,892	6,002
215818_at	6,551	6,529	6,623	7,038	6,716	7,128
215819_s_at	7,788	7,647	8,090	6,663	6,491	7,016
215820_x_at	6,823	7,021	6,447	6,569	6,501	6,670
215821_x_at	6,567	6,276	6,595	7,250	7,105	7,176
215822_x_at	7,698	7,634	8,006	8,325	8,042	8,332
215823_x_at	13,582	13,795	13,788	12,566	13,111	12,986
215824_at	4,932	4,890	4,788	4,996	4,988	4,669
215825_at	7,129	6,943	7,023	6,965	6,830	7,014
215826_x_at	6,810	7,283	7,254	6,388	6,223	6,616
215827_x_at	7,559	7,442	7,419	8,061	8,068	8,323
215828_at	7,687	7,334	7,318	7,635	7,763	7,778
215829_at	6,058	5,685	5,877	6,981	6,710	6,815
215830_at	5,979	5,837	6,123	6,511	6,569	6,321
215831_at	4,564	4,444	4,676	3,981	4,017	3,917
215832_x_at	7,649	7,782	7,507	8,060	7,583	7,766
215833_s_at	6,850	6,934	7,199	7,357	7,117	7,452
215834_x_at	6,542	6,706	6,462	7,232	6,888	7,048
215835_at	5,975	6,093	5,832	6,052	5,770	5,864
215836_s_at	8,465	8,216	8,465	9,327	9,523	9,580
215837_x_at	5,061	4,949	5,098	5,141	5,219	5,294
215838_at	5,216	5,061	5,256	6,008	5,586	5,746
215839_at	6,660	6,530	6,383	7,831	7,601	7,655
215840_at	6,500	6,694	6,793	6,898	7,013	6,634
215841_at	7,265	7,482	7,493	8,295	8,049	8,300
215842_s_at	7,066	7,275	7,495	7,179	6,978	7,133
215843_s_at	7,396	7,240	7,680	6,959	6,661	6,939
215844_at	7,182	7,201	7,374	7,104	7,527	7,481
215845_x_at	6,681	6,374	6,809	6,524	6,946	6,414
215846_at	4,898	4,502	4,748	4,736	4,791	4,895
215847_at	6,368	7,044	6,825	4,932	4,728	4,907
215848_at	6,829	6,332	6,514	6,596	6,385	6,465

215849_x_at	7,869	7,989	7,908	8,037	7,938	7,888
215850_s_at	4,174	4,412	4,561	3,898	3,774	3,996
215851_at	4,637	4,848	4,671	4,746	4,730	4,680
215852_x_at	6,548	6,111	6,744	6,151	6,339	6,187
215853_at	3,990	4,124	4,089	3,855	3,973	3,960
215854_at	6,193	5,766	5,791	5,240	5,026	5,145
215855_s_at	7,584	7,615	7,738	7,671	8,184	7,896
215856_at	7,607	7,571	7,499	7,429	7,461	7,436
215857_at	6,614	6,602	6,490	7,752	7,524	7,560
215858_at	6,938	6,902	7,083	7,000	7,019	6,870
215859_at	6,004	5,664	5,786	6,609	6,269	6,585
215860_at	6,498	6,669	6,853	7,014	6,763	6,786
215861_at	6,586	6,443	6,761	7,218	7,357	7,269
215862_at	6,560	6,207	6,759	6,916	6,858	7,170
215863_at	6,762	6,595	6,468	6,554	6,975	6,442
215864_at	5,226	5,171	5,216	5,783	5,741	5,654
215865_at	8,090	8,206	8,155	8,021	7,915	8,171
215866_at	7,030	6,412	6,869	6,574	6,325	6,557
215867_x_at	8,308	8,522	8,504	10,744	11,212	11,255
215868_x_at	6,833	6,427	6,924	7,080	7,056	7,040
215869_at	6,519	6,867	6,856	7,559	7,875	7,839
215870_s_at	4,873	5,074	5,245	5,193	5,431	5,419
215871_at	5,603	5,376	5,406	6,632	6,774	6,900
215872_at	5,428	5,248	5,438	5,975	5,898	5,817
215873_x_at	8,478	8,291	8,606	8,805	8,947	9,403
215874_at	6,111	5,355	6,187	6,272	6,183	6,129
215875_at	6,741	6,852	6,722	7,159	7,194	7,279
215876_at	6,446	6,190	6,439	6,813	6,152	6,607
215877_at	6,120	6,382	6,042	6,544	6,342	6,380
215878_at	3,890	4,013	4,107	4,238	4,248	4,229
215879_at	3,692	3,762	3,645	3,615	3,928	3,622
215880_at	6,605	6,316	6,418	7,188	6,952	7,192
215881_x_at	6,449	6,436	6,537	7,161	7,255	7,471
215882_at	5,948	5,796	5,966	6,301	5,841	5,887
215883_at	7,306	7,045	7,305	7,416	7,499	7,516
215884_s_at	11,008	10,908	11,060	10,350	10,769	10,794
215885_at	5,605	5,388	5,598	5,996	5,832	6,110
215886_x_at	5,534	5,782	5,505	6,045	5,156	5,554
215887_at	7,276	7,255	7,297	7,330	7,328	7,243
215888_at	7,388	7,508	8,071	5,564	6,153	5,629
215889_at	5,149	5,260	5,390	4,986	5,042	4,971
215890_at	6,387	6,520	6,680	7,163	7,123	7,104
215891_s_at	5,582	5,911	5,855	6,133	5,745	6,290
215892_at	4,467	4,327	4,718	4,510	4,713	4,655
215893_x_at	5,200	5,141	5,329	5,713	5,648	5,362
215894_at	4,660	5,233	4,725	6,574	5,034	5,030
215895_x_at	5,970	5,512	6,051	5,324	5,552	5,217
215896_at	5,633	5,551	5,789	5,787	5,751	5,882
215897_at	4,394	4,480	4,527	4,521	4,442	4,830
215898_at	6,562	5,968	6,129	4,138	5,755	4,363
215899_at	6,773	6,505	7,133	7,973	7,551	8,038
215900_at	5,620	5,424	5,025	5,147	5,133	5,185
215901_at	3,695	4,014	4,122	4,443	4,329	4,340
215902_at	4,717	5,006	5,079	4,976	5,166	5,175
215903_s_at	8,944	9,125	9,346	9,038	9,088	9,219
215904_at	5,663	5,692	5,553	6,569	6,401	6,875

215905_s_at	10,234	9,329	9,842	7,935	7,294	7,327
215906_at	6,954	6,646	6,588	7,280	7,329	7,224
215907_at	8,170	8,051	7,917	7,651	8,315	7,842
215908_at	7,722	7,334	7,601	7,832	8,011	7,975
215909_x_at	7,757	7,542	7,768	7,663	7,874	7,691
215910_s_at	6,808	6,907	7,223	7,961	8,208	7,869
215911_x_at	4,759	5,341	5,284	5,475	5,524	5,596
215912_at	5,596	5,708	5,864	6,099	5,852	5,962
215913_s_at	8,223	8,145	7,349	7,436	7,686	7,193
215914_at	5,422	5,323	5,592	6,413	6,162	6,228
215915_at	4,605	4,805	5,083	5,433	5,324	5,292
215916_at	6,551	6,776	6,729	7,076	6,604	6,717
215917_at	3,758	3,842	4,092	4,620	4,122	4,705
215918_s_at	6,292	6,714	6,626	7,071	6,840	7,166
215919_s_at	6,344	5,801	5,749	5,656	5,407	5,852
215920_s_at	6,429	6,493	6,436	7,211	7,300	7,475
215921_at	6,016	5,637	5,118	5,075	4,907	4,827
215922_at	4,616	4,569	4,506	4,474	4,717	4,437
215923_s_at	7,042	6,582	6,909	7,556	6,802	7,170
215924_at	3,695	3,525	3,621	3,836	3,744	3,599
215925_s_at	6,535	6,357	5,959	6,878	6,707	7,033
215926_x_at	8,248	7,885	8,418	7,840	8,239	8,515
215927_at	6,823	6,528	6,906	7,913	7,822	8,097
215928_at	6,867	6,385	6,217	7,232	7,189	7,735
215929_at	3,797	3,838	3,933	3,829	3,777	3,813
215930_s_at	6,933	7,310	7,231	7,463	7,927	7,583
215931_s_at	6,568	6,245	6,855	6,063	6,173	5,636
215932_at	5,650	5,146	4,628	6,075	5,440	5,554
215933_s_at	5,101	5,130	5,131	5,914	5,260	5,556
215934_at	5,326	5,492	5,331	6,229	6,139	6,170
215935_at	6,528	6,495	6,376	6,815	7,102	6,880
215936_s_at	8,740	9,017	8,472	7,129	8,048	6,726
215937_at	4,985	4,767	4,755	5,398	5,636	5,576
215938_s_at	7,286	7,191	7,162	7,207	7,248	7,218
215939_at	6,815	6,448	6,802	6,613	6,605	6,463
215940_at	4,423	4,203	4,378	4,553	4,536	4,547
215941_at	5,214	5,393	5,351	6,173	5,690	5,879
215942_s_at	7,329	7,340	7,545	7,248	6,973	7,214
215943_at	5,824	5,567	5,578	5,424	5,260	5,564
215944_at	4,082	3,739	4,092	4,420	4,636	4,583
215945_s_at	9,839	9,528	9,550	7,558	7,585	7,523
215946_x_at	6,400	6,435	6,557	7,163	7,241	7,087
215947_s_at	11,366	11,079	11,549	10,188	9,808	9,631
215948_x_at	6,853	7,435	7,094	7,205	7,427	6,968
215949_x_at	7,583	7,470	7,783	8,323	8,130	8,335
215951_at	6,763	6,757	6,760	7,363	7,266	7,465
215952_s_at	12,255	11,900	11,861	12,458	11,849	12,656
215953_at	5,911	5,647	5,804	6,202	6,024	6,058
215954_s_at	7,913	7,272	7,948	7,061	6,816	6,837
215955_x_at	5,439	6,151	6,329	6,648	5,997	6,680
215956_at	6,247	6,071	6,123	6,012	6,631	6,011
215957_at	3,824	3,894	3,853	4,145	4,118	4,214
215958_at	4,615	4,588	4,888	4,786	4,631	4,621
215959_at	5,794	5,767	5,900	6,377	6,025	6,085
215960_at	5,432	5,673	5,649	5,882	5,985	5,751
215961_at	6,184	6,314	6,087	6,931	6,820	6,823

215962_at	3,752	3,744	3,778	3,965	3,902	3,865
215963_x_at	14,125	14,364	14,046	13,699	13,309	13,797
215964_at	5,393	5,020	5,326	5,884	5,694	6,030
215965_at	5,930	5,815	5,908	5,765	5,671	6,112
215966_x_at	5,786	5,742	5,399	6,533	6,012	6,433
215967_s_at	6,081	6,312	6,279	6,157	6,133	6,237
215968_at	4,457	4,586	4,521	4,378	4,378	4,631
215969_at	5,467	5,816	5,874	5,682	5,754	5,411
215970_at	6,944	6,453	6,555	7,252	7,238	7,180
215971_at	5,476	5,592	5,497	5,833	5,665	5,590
215972_at	5,728	5,432	5,945	7,080	7,015	7,094
215973_at	6,481	6,589	6,758	6,679	6,568	6,490
215974_at	6,433	6,574	6,681	7,278	6,795	7,157
215975_x_at	6,394	6,265	6,461	6,551	6,791	6,884
215976_at	5,587	5,550	5,873	4,989	4,808	4,987
215977_x_at	7,349	7,241	7,045	8,210	7,439	7,400
215978_x_at	11,432	11,032	11,080	10,067	10,946	10,343
215979_s_at	6,066	6,328	6,160	7,299	7,483	7,289
215980_s_at	7,560	7,518	7,434	7,758	7,514	7,652
215981_at	4,761	4,859	4,909	4,796	4,836	4,782
215982_s_at	8,414	8,337	8,710	9,005	8,236	8,614
215983_s_at	8,042	7,620	8,133	8,590	8,271	8,467
215984_s_at	6,028	6,229	6,210	7,051	6,715	6,905
215985_at	7,300	7,243	7,279	6,339	6,206	6,449
215986_at	4,368	4,974	4,583	4,273	4,236	4,191
215987_at	5,428	5,559	5,330	5,746	5,895	5,825
215988_s_at	5,861	6,278	6,347	5,930	5,731	5,816
215989_at	6,547	6,210	6,446	7,093	6,832	7,028
215990_s_at	5,548	5,567	5,949	5,687	5,364	5,523
215991_s_at	5,271	5,692	5,459	5,556	5,481	6,203
215992_s_at	7,384	7,523	7,101	4,743	6,145	4,972
215993_at	4,002	4,244	4,216	4,057	4,100	4,076
215994_x_at	9,082	9,199	9,383	9,404	9,366	9,781
215995_x_at	5,895	5,739	5,749	6,315	6,314	6,459
215996_at	7,015	7,157	7,363	7,429	7,248	7,363
215997_s_at	7,328	8,080	7,112	8,437	9,003	8,295
215998_at	5,476	5,302	5,358	5,996	5,652	5,872
215999_at	6,461	6,557	6,838	6,348	6,290	6,675
216000_at	8,473	7,981	8,359	6,375	6,271	6,591
216001_at	6,627	6,675	6,897	7,321	7,440	7,347
216002_at	5,088	4,497	4,881	5,127	5,046	5,137
216003_at	4,967	4,949	5,051	5,681	5,255	5,307
216004_s_at	5,146	5,694	5,900	5,986	5,927	5,798
216005_at	5,758	4,904	6,333	5,345	7,400	5,466
216006_at	6,260	6,329	6,688	5,502	6,290	5,854
216007_at	4,271	4,024	4,081	4,159	4,334	4,167
216008_s_at	8,908	8,804	8,717	8,727	8,589	8,707
216009_at	6,695	6,896	6,539	6,318	6,081	6,599
216010_x_at	5,722	5,678	5,660	6,517	6,395	6,851
216011_at	6,141	6,101	6,288	6,615	6,331	6,398
216012_at	4,583	4,797	4,499	4,736	5,204	4,715
216013_at	5,070	4,928	5,230	5,563	5,775	5,795
216014_s_at	6,495	6,703	6,764	7,060	6,738	6,774
216015_s_at	4,912	4,489	4,740	4,769	4,815	4,831
216016_at	3,715	3,675	3,818	4,362	4,230	4,337
216017_s_at	6,897	6,875	7,122	7,149	6,866	7,302

216018_at	6,268	6,683	6,431	7,177	6,945	7,086
216019_x_at	7,596	7,377	7,616	8,417	8,574	8,520
216020_at	4,021	4,645	4,137	4,496	4,413	4,299
216021_s_at	4,769	4,561	5,036	4,670	4,518	4,537
216022_at	7,123	6,756	7,115	5,080	6,399	5,046
216023_at	7,030	7,146	7,215	7,338	7,167	7,345
216024_at	6,809	6,579	6,761	7,260	6,908	7,158
216025_x_at	6,856	6,642	6,702	7,218	6,995	6,914
216026_s_at	9,268	8,735	9,236	8,052	8,182	8,279
216027_at	4,307	4,333	4,489	5,805	5,245	5,260
216028_at	8,835	8,587	8,589	7,432	8,420	8,791
216029_at	6,668	6,528	6,621	6,793	6,554	6,649
216030_s_at	4,319	4,219	4,331	5,368	5,276	5,434
216031_x_at	6,281	6,497	6,563	6,605	6,263	6,324
216032_s_at	10,077	10,032	10,183	10,015	9,544	10,503
216033_s_at	8,492	8,538	8,367	8,132	8,032	7,757
216034_at	6,796	6,997	6,948	6,906	6,813	6,898
216035_x_at	10,473	10,425	10,648	7,938	8,476	8,225
216036_x_at	8,618	8,483	8,675	8,653	8,536	8,820
216037_x_at	10,097	9,951	10,083	7,483	8,173	7,631
216038_x_at	8,339	8,082	8,307	8,464	8,392	8,373
216039_at	4,454	4,451	4,875	4,281	4,225	4,303
216040_x_at	6,567	6,366	6,647	7,963	8,175	7,936
216041_x_at	9,035	8,827	9,068	9,836	9,193	10,080
216042_at	8,256	8,233	8,390	8,504	8,459	8,697
216043_x_at	7,335	7,420	7,475	7,904	7,902	7,788
216044_x_at	7,803	8,528	7,655	7,964	9,065	8,399
216045_at	5,067	4,815	5,015	5,166	5,119	5,321
216046_at	7,566	7,241	7,612	8,057	8,114	8,296
216047_x_at	6,991	6,992	7,169	7,399	7,154	7,356
216048_s_at	7,191	6,659	6,779	6,015	6,503	6,656
216049_at	5,296	5,044	6,068	5,439	5,345	5,531
216050_at	3,761	3,739	3,883	3,749	4,014	3,733
216051_x_at	7,993	7,758	7,928	8,243	8,291	8,380
216052_x_at	7,130	6,893	7,230	7,969	8,285	8,271
216053_x_at	6,071	5,807	5,744	6,808	6,601	6,854
216054_x_at	6,811	6,501	6,252	7,114	6,787	7,344
216055_at	6,542	7,084	6,721	7,438	7,354	7,866
216056_at	5,611	5,692	5,542	6,166	5,902	5,965
216057_at	4,543	4,645	4,311	4,898	5,212	5,228
216058_s_at	5,362	5,144	5,600	5,843	5,679	5,747
216059_at	6,225	6,527	6,194	6,888	6,517	6,836
216060_s_at	8,994	9,212	8,379	5,621	7,086	6,239
216061_x_at	7,694	7,950	7,904	7,881	7,952	7,573
216062_at	5,696	5,526	5,888	6,089	5,854	6,398
216063_at	5,258	5,507	5,087	5,977	5,941	6,092
216064_s_at	7,806	8,099	7,357	7,244	7,092	7,426
216065_at	5,299	5,320	4,977	5,433	5,266	5,779
216066_at	7,503	7,008	6,991	7,411	7,519	7,558
216067_at	6,239	6,089	6,536	5,811	6,002	5,996
216068_at	3,769	3,714	3,848	3,443	3,679	3,567
216069_at	4,716	4,272	4,402	4,046	4,282	4,159
216070_at	4,414	4,469	4,556	4,182	4,163	4,131
216071_x_at	8,032	7,852	8,035	7,704	7,395	7,873
216072_at	6,662	6,695	6,711	7,129	7,123	6,953
216073_at	5,459	5,144	5,704	5,651	5,461	5,604

216074_x_at	7,870	7,897	8,166	6,597	6,372	6,651
216075_at	5,822	5,827	5,715	6,389	5,884	6,133
216076_at	6,779	6,446	7,019	7,241	7,257	7,338
216077_s_at	6,149	6,578	7,338	7,449	7,490	7,662
216078_at	6,808	6,636	6,630	6,518	6,447	6,571
216079_at	7,182	6,891	7,409	7,511	7,416	7,564
216080_s_at	7,651	7,794	7,700	9,573	8,361	9,791
216081_at	6,247	5,697	5,975	6,089	6,081	6,396
216082_at	7,409	7,210	7,260	7,772	7,413	7,734
216083_s_at	7,937	7,665	8,000	8,322	7,934	8,368
216084_at	4,841	5,270	5,384	6,031	5,886	5,996
216085_at	5,339	5,336	5,688	5,612	5,694	5,818
216086_at	6,194	5,542	5,957	4,818	4,638	4,760
216087_at	4,027	3,832	4,046	4,415	4,478	4,610
216088_s_at	10,443	10,660	10,756	10,623	9,790	10,507
216089_at	4,177	4,335	3,797	4,758	4,484	4,838
216090_x_at	5,622	5,561	5,840	6,036	6,367	6,053
216091_s_at	6,978	7,734	7,209	7,716	7,491	7,772
216092_s_at	9,578	9,499	9,553	7,814	7,640	7,904
216093_at	4,955	4,855	4,637	6,022	5,639	5,928
216094_at	6,688	6,871	6,923	4,739	5,435	4,821
216095_x_at	9,956	9,956	9,977	10,400	10,713	10,314
216096_s_at	4,858	4,426	4,877	5,726	4,962	5,042
216097_at	3,721	3,548	3,859	3,669	3,601	3,680
216098_s_at	4,454	4,222	4,406	4,895	4,694	4,854
216099_at	5,800	5,814	5,434	6,088	5,872	6,190
216100_s_at	7,001	6,411	6,101	6,333	6,038	5,771
216101_at	6,951	6,733	6,716	6,209	6,704	6,248
216102_at	7,134	6,899	7,266	7,549	7,355	7,759
216103_at	4,760	4,975	5,043	5,414	5,312	5,182
216104_at	4,848	4,890	4,557	4,976	5,108	5,169
216105_x_at	9,950	9,493	9,668	9,430	9,271	9,488
216106_at	6,480	6,022	6,194	6,909	6,862	7,062
216107_at	6,514	6,382	6,531	6,529	6,241	6,592
216108_at	4,436	4,419	4,630	5,148	4,913	4,824
216109_at	5,939	6,388	6,327	4,110	6,088	4,823
216110_x_at	6,766	6,695	6,757	5,867	6,533	5,883
216111_x_at	7,550	7,496	7,567	7,563	7,487	7,616
216112_at	6,855	6,905	6,911	6,664	6,786	6,722
216113_at	5,696	4,795	5,326	4,261	4,313	4,490
216114_at	7,201	6,974	7,328	7,044	7,182	7,107
216115_at	6,728	6,523	6,772	5,787	6,662	5,883
216116_at	6,972	6,973	6,752	6,955	6,859	6,969
216117_at	7,046	7,007	6,993	7,402	7,206	7,489
216118_at	5,598	5,511	5,657	5,511	5,669	5,478
216119_s_at	6,779	7,043	6,561	7,208	7,508	7,574
216120_s_at	5,569	5,781	5,631	5,733	5,653	5,755
216121_at	5,132	5,458	5,491	4,657	5,038	4,929
216122_at	5,810	5,516	5,701	6,185	5,921	6,082
216123_x_at	6,116	5,823	6,129	6,229	6,508	6,529
216124_at	4,492	4,439	4,381	4,706	4,509	4,528
216125_s_at	7,678	8,031	7,560	7,172	7,011	7,265
216126_at	6,490	6,325	6,464	6,723	7,034	7,100
216127_at	5,865	6,049	6,524	6,949	6,739	6,594
216128_at	7,261	7,331	7,106	7,565	7,608	7,480
216129_at	5,882	6,343	5,848	6,199	6,550	6,232

216130_at	7,054	6,867	6,989	7,448	7,466	7,225
216131_at	5,352	5,470	5,436	5,397	5,209	5,108
216132_at	5,795	5,955	6,173	6,089	6,160	6,344
216133_at	6,528	6,333	6,425	7,337	7,094	7,360
216134_at	5,314	4,982	4,870	5,391	5,286	5,466
216135_at	5,090	4,800	4,929	5,094	5,146	5,062
216136_at	4,060	4,267	3,941	4,462	4,523	4,328
216137_s_at	6,619	6,552	6,600	7,392	7,032	7,588
216138_at	7,694	7,744	7,699	8,455	8,195	8,312
216139_s_at	7,058	6,917	7,395	8,380	8,301	8,511
216140_at	6,052	6,088	6,202	6,253	6,120	6,372
216141_at	7,048	6,895	7,202	7,243	7,184	7,310
216142_at	6,831	6,596	6,922	7,265	7,503	7,358
216143_at	7,240	7,071	7,474	7,750	7,624	7,845
216144_at	6,055	6,089	6,190	6,500	7,217	6,908
216145_at	7,153	7,354	7,301	7,144	6,837	7,322
216146_at	6,045	5,921	6,161	6,902	6,835	7,029
216147_at	6,302	6,560	6,501	5,609	7,881	6,197
216148_at	6,112	5,480	5,776	6,133	6,095	5,977
216149_at	5,674	6,156	6,032	5,309	5,562	5,502
216150_at	5,870	6,500	6,501	6,965	7,055	7,017
216151_at	3,742	3,529	3,724	3,827	4,146	4,130
216152_at	7,032	6,646	7,183	7,408	7,609	7,677
216153_x_at	8,494	8,417	8,316	8,109	8,446	8,336
216154_at	5,142	5,741	5,457	5,820	5,692	5,791
216155_at	5,295	4,748	4,915	5,168	5,329	5,277
216156_at	5,295	6,023	5,576	5,824	5,677	5,765
216157_at	6,194	5,846	5,991	6,873	6,716	6,803
216158_at	3,994	3,960	4,032	4,431	4,290	4,434
216159_s_at	7,233	7,001	7,185	7,680	7,323	7,398
216160_at	5,657	5,765	5,890	6,040	6,205	6,150
216161_at	5,808	5,783	5,749	5,479	5,485	5,206
216162_at	4,955	4,974	4,819	5,110	5,173	5,141
216163_at	6,435	6,271	6,612	6,954	6,612	6,684
216164_at	7,347	7,516	7,655	7,811	7,831	7,980
216165_at	5,137	5,034	5,194	4,935	5,058	5,116
216166_at	6,082	6,131	6,287	6,148	5,971	6,362
216167_at	4,716	4,460	4,593	4,439	4,326	4,485
216168_at	5,870	6,008	5,731	6,549	5,852	6,145
216169_at	4,795	4,815	4,892	5,095	4,957	5,083
216170_at	6,535	6,150	6,628	5,129	5,320	4,834
216171_at	4,100	4,294	3,998	4,516	4,436	4,660
216172_at	6,754	6,520	6,760	7,414	7,335	7,559
216173_at	4,581	4,203	4,880	3,904	4,080	3,855
216174_at	8,345	7,888	7,999	7,875	7,875	7,957
216175_at	7,976	7,821	8,042	7,510	7,714	7,695
216176_at	7,751	7,717	7,451	6,241	6,417	5,993
216177_at	7,593	7,568	7,485	8,826	8,009	8,481
216178_x_at	5,567	4,828	5,518	6,166	6,246	6,161
216179_x_at	5,514	5,208	5,804	5,799	5,630	5,808
216180_s_at	6,974	7,559	6,775	8,017	7,632	7,664
216181_at	7,473	7,558	7,627	7,821	8,000	8,047
216182_at	6,697	6,798	6,710	7,331	6,935	7,137
216183_at	7,229	7,467	7,340	7,711	7,251	7,295
216184_s_at	5,898	6,121	5,919	6,704	6,459	6,722
216185_at	5,598	5,541	5,542	5,091	4,864	5,091

216186_at	8,719	8,549	8,710	9,603	8,786	9,252
216187_x_at	10,020	9,376	9,594	8,712	9,751	8,954
216188_at	6,395	5,626	5,949	6,242	6,234	6,113
216189_at	4,840	4,300	5,625	4,342	4,508	4,419
216190_x_at	4,875	5,001	4,329	5,368	5,328	5,183
216191_s_at	4,048	4,014	4,244	5,008	4,395	4,467
216192_at	5,266	5,219	5,251	5,776	5,481	5,535
216193_at	6,527	6,751	6,581	6,931	6,681	6,805
216194_s_at	10,534	10,340	10,205	10,671	9,852	10,908
216195_at	6,732	6,524	6,642	5,894	5,813	5,601
216196_at	5,773	6,046	6,097	5,825	5,768	6,095
216197_at	6,369	6,496	6,318	4,497	5,028	4,650
216198_at	5,721	5,454	5,949	5,190	5,424	5,218
216199_s_at	10,479	10,259	10,381	9,273	10,197	8,715
216200_at	4,745	5,376	4,716	5,971	5,887	5,328
216201_at	4,583	4,676	4,881	4,894	4,507	4,875
216202_s_at	7,677	7,831	7,768	7,582	7,322	7,828
216203_at	4,747	4,991	5,137	4,819	4,912	5,139
216204_at	7,863	7,388	7,640	8,167	8,179	8,271
216205_s_at	7,045	6,783	7,111	7,082	7,019	7,162
216206_x_at	6,717	6,832	6,757	6,419	6,639	6,044
216207_x_at	7,444	7,365	7,341	7,937	7,678	8,024
216208_s_at	7,219	6,511	6,736	6,948	7,130	7,560
216209_at	6,533	7,241	6,759	7,670	7,515	7,610
216210_x_at	9,292	9,173	9,443	10,501	10,725	10,815
216211_at	6,588	5,924	6,597	5,931	6,413	6,099
216212_s_at	10,411	10,454	10,799	10,019	9,193	9,722
216213_at	5,191	5,395	5,606	5,307	5,403	5,411
216214_at	4,824	4,641	4,806	5,114	5,048	5,140
216215_s_at	11,038	11,113	10,968	9,547	10,719	9,990
216216_at	6,041	5,845	6,296	6,968	6,953	7,248
216217_at	6,795	6,595	6,671	7,316	6,947	7,082
216218_s_at	7,457	7,827	7,306	6,311	6,298	6,413
216219_at	7,974	7,984	7,959	8,302	8,572	8,393
216220_s_at	7,663	7,296	7,781	8,961	8,777	8,760
216221_s_at	11,244	11,516	11,390	8,888	10,265	8,678
216222_s_at	9,013	9,020	9,414	7,384	7,287	7,590
216223_at	5,513	5,102	5,069	6,031	5,896	6,069
216224_s_at	7,823	8,014	7,995	7,894	7,799	7,808
216225_at	5,915	5,755	5,751	6,221	6,132	6,033
216226_at	6,424	6,769	6,877	7,077	6,672	6,727
216227_at	6,076	6,266	6,158	6,756	6,714	6,622
216228_s_at	9,993	10,329	9,143	7,031	7,646	5,919
216229_x_at	8,085	8,048	7,936	7,648	8,457	7,685
216230_x_at	5,957	6,072	5,639	6,455	5,825	6,515
216231_s_at	10,025	10,462	9,485	12,410	12,144	12,663
216232_s_at	9,200	9,179	9,190	8,370	8,327	8,806
216233_at	4,596	4,971	4,740	5,403	4,856	5,093
216234_s_at	6,201	6,340	5,946	7,261	6,723	7,042
216235_s_at	6,125	6,045	5,837	6,619	6,414	6,684
216236_s_at	10,427	10,399	10,411	8,922	8,552	8,721
216237_s_at	10,766	10,368	11,037	10,282	9,279	9,603
216238_s_at	4,520	4,917	4,472	5,089	4,724	5,141
216239_at	4,540	4,530	4,328	4,624	4,953	4,517
216240_at	6,042	5,968	6,281	6,588	6,297	6,366
216241_s_at	12,992	13,492	13,172	10,703	11,359	11,194

216242_x_at	7,905	7,516	7,667	8,856	8,661	8,792
216243_s_at	6,328	6,410	6,392	6,844	6,710	6,633
216244_at	4,543	4,333	4,927	4,980	4,800	4,756
216245_at	5,875	5,815	6,421	6,234	6,226	6,553
216246_at	9,142	9,571	9,689	9,135	9,444	9,029
216247_at	6,625	5,950	7,720	4,775	5,424	5,279
216248_s_at	6,678	7,299	7,275	5,978	10,651	8,747
216249_at	5,974	5,898	6,139	6,817	6,626	6,559
216250_s_at	7,362	7,804	7,853	11,449	9,178	10,610
216251_s_at	9,696	9,461	10,316	9,507	9,090	9,183
216252_x_at	4,544	4,998	5,515	5,590	6,600	6,226
216253_s_at	5,995	6,187	6,363	7,934	7,967	8,335
216254_at	5,252	5,113	5,488	5,749	5,615	5,599
216255_s_at	5,726	5,598	5,194	5,468	5,342	5,628
216256_at	6,647	6,651	6,144	5,739	5,702	5,527
216257_at	5,849	5,824	5,896	6,578	6,436	6,417
216258_s_at	3,952	3,624	4,029	4,134	3,997	4,184
216259_at	5,865	5,884	5,497	6,346	6,864	6,453
216260_at	5,839	5,964	6,084	5,514	5,979	5,778
216261_at	5,398	5,488	5,530	6,322	6,436	6,254
216262_s_at	10,022	9,937	10,055	8,583	8,636	8,775
216263_s_at	5,476	5,544	6,138	5,526	5,464	5,691
216264_s_at	8,404	8,355	8,541	8,881	9,193	9,755
216265_x_at	6,871	7,159	7,139	6,513	6,479	6,444
216266_s_at	7,730	8,065	7,712	5,450	6,197	5,254
216267_s_at	8,066	7,650	8,162	8,799	7,910	8,703
216268_s_at	10,694	10,619	10,802	7,857	11,462	10,094
216269_s_at	6,694	6,898	6,645	7,961	7,925	8,069
216270_at	5,830	6,219	5,890	6,840	6,835	6,683
216271_x_at	7,444	7,111	7,207	7,708	6,907	7,243
216272_x_at	7,979	7,951	8,132	8,714	8,851	9,132
216273_at	6,539	6,900	6,833	7,075	7,149	7,261
216274_s_at	12,515	12,609	12,139	11,821	11,280	11,867
216275_at	6,305	6,162	6,314	6,915	6,661	6,696
216276_s_at	5,538	5,284	5,391	5,973	5,492	5,969
216277_at	4,815	5,054	4,687	4,829	4,648	4,642
216278_at	4,813	4,797	4,711	4,994	5,369	5,072
216279_at	4,731	4,658	4,711	4,805	4,605	4,656
216280_s_at	5,348	5,458	5,468	5,753	5,769	5,692
216281_at	5,642	5,650	5,578	5,513	5,580	5,703
216282_x_at	9,338	9,310	9,328	9,274	9,250	9,538
216283_s_at	6,325	5,965	6,233	6,580	6,657	6,667
216284_at	6,657	6,254	6,497	6,206	6,472	6,219
216285_at	6,614	6,668	6,315	7,448	7,058	7,035
216286_at	4,896	4,546	4,960	4,152	4,047	4,238
216287_at	4,754	5,211	4,954	4,921	4,774	4,700
216288_at	4,543	4,381	4,465	4,739	5,040	5,018
216289_at	7,154	7,085	7,275	7,525	7,564	8,019
216290_x_at	6,076	5,978	6,027	5,644	6,010	5,566
216291_at	5,744	5,778	5,457	6,770	6,259	6,629
216292_at	7,236	7,147	7,170	7,746	7,532	7,742
216293_at	9,088	8,797	9,063	10,125	10,080	10,158
216294_s_at	6,097	5,964	6,222	6,176	6,231	6,223
216295_s_at	12,007	12,074	12,252	12,237	11,508	12,204
216296_at	6,773	7,493	6,867	6,807	6,648	6,555
216297_at	4,463	4,568	4,701	4,769	4,732	4,660

216298_at	4,723	4,888	4,765	5,713	5,178	5,550
216299_s_at	7,145	7,147	7,340	8,494	8,244	8,433
216300_x_at	7,125	7,199	7,744	7,695	7,918	7,844
216301_at	7,421	7,227	7,561	8,437	8,448	8,389
216302_at	6,915	6,983	7,046	6,829	6,674	7,111
216303_s_at	6,137	6,155	5,802	6,872	6,241	6,712
216304_x_at	11,379	11,454	11,248	10,709	11,577	10,683
216305_s_at	8,877	9,159	8,501	7,172	7,490	6,444
216306_x_at	12,399	12,263	12,690	11,662	11,242	11,592
216307_at	4,862	5,008	4,886	5,553	5,950	5,784
216308_x_at	10,358	10,546	10,460	10,440	9,885	10,841
216309_x_at	9,010	8,602	8,898	8,780	8,744	8,791
216310_at	8,772	8,543	8,610	8,123	8,786	8,535
216311_at	7,080	7,033	6,928	7,048	6,624	6,849
216312_at	5,979	6,031	6,253	6,246	6,204	6,275
216313_at	4,758	4,969	5,149	5,476	5,391	5,852
216314_at	3,848	3,892	3,878	3,799	3,775	3,700
216315_x_at	8,047	8,182	8,261	8,898	8,486	9,169
216316_x_at	6,545	6,792	6,288	6,733	6,409	6,343
216317_x_at	7,468	7,507	7,897	7,006	7,258	7,403
216318_at	7,323	7,066	7,278	7,933	7,991	8,042
216319_at	5,295	6,778	6,940	4,755	4,663	4,827
216320_x_at	8,072	8,598	8,309	8,743	8,651	8,843
216321_s_at	10,221	10,097	10,172	9,492	10,873	9,640
216322_at	3,448	3,649	3,641	3,918	3,723	3,861
216323_x_at	9,001	9,329	8,693	9,678	9,242	9,785
216324_at	6,167	5,914	6,164	6,664	6,472	6,744
216325_x_at	6,925	6,947	6,801	7,521	7,598	7,808
216326_s_at	10,539	10,363	10,694	9,979	9,471	9,949
216327_s_at	5,764	5,763	5,629	5,893	5,864	6,145
216328_at	4,723	4,753	4,574	5,405	5,448	5,633
216329_at	6,287	6,077	6,521	7,556	7,595	7,761
216330_s_at	7,402	7,267	7,403	7,443	7,325	7,471
216331_at	8,944	8,851	8,812	8,537	9,159	9,138
216332_at	7,352	7,194	7,449	7,678	7,358	7,677
216333_x_at	7,321	7,761	7,478	8,289	8,733	8,379
216334_s_at	5,021	5,086	5,035	7,066	7,046	7,545
216336_x_at	10,489	10,068	11,436	12,725	11,872	12,948
216337_at	6,906	7,088	7,040	6,836	6,854	6,820
216338_s_at	8,088	8,044	8,332	8,833	8,380	9,426
216339_s_at	6,281	6,177	5,919	6,735	6,591	7,029
216340_s_at	7,317	7,060	7,424	7,389	7,481	7,540
216341_s_at	5,320	5,405	4,793	5,334	5,298	5,162
216342_x_at	13,734	13,790	13,799	13,235	13,054	13,161
216343_at	5,934	5,542	5,817	6,587	6,327	6,594
216344_at	6,320	6,741	6,376	6,675	6,113	6,205
216345_at	6,807	6,617	6,920	7,526	7,391	7,299
216346_at	5,494	5,280	5,498	5,752	5,850	5,999
216347_s_at	7,105	7,086	7,102	7,578	7,223	7,711
216348_at	9,820	10,073	10,313	10,528	9,926	10,547
216349_at	7,776	7,890	7,562	8,517	8,393	8,548
216350_s_at	7,420	6,807	6,925	6,065	6,968	6,249
216351_x_at	4,414	4,670	4,580	4,758	4,656	4,914
216352_x_at	6,178	6,405	6,185	6,980	6,727	6,907
216353_s_at	4,263	4,477	4,358	4,541	4,445	4,807
216354_at	7,321	7,444	7,481	8,128	7,754	7,955

216355_at	6,580	6,372	6,525	6,592	6,605	6,552
216356_x_at	8,076	7,609	7,917	8,267	8,197	7,965
216357_at	5,861	5,354	5,921	6,365	6,403	6,263
216358_at	6,143	6,033	6,324	7,893	8,126	8,347
216359_at	5,574	5,809	5,996	6,163	5,887	6,344
216360_x_at	7,358	6,993	7,353	7,750	7,531	7,759
216361_s_at	5,552	5,782	5,554	5,364	5,253	5,929
216362_at	6,129	6,463	6,664	6,303	6,007	6,143
216363_at	7,404	7,348	7,078	7,887	7,892	7,884
216364_s_at	6,020	5,530	6,129	6,454	6,170	6,523
216365_x_at	6,822	6,750	6,746	7,488	7,621	7,727
216366_x_at	7,884	7,970	8,064	8,264	8,022	8,216
216367_at	4,953	5,082	5,474	5,507	5,294	5,477
216368_s_at	6,444	6,602	6,070	6,689	6,821	6,996
216369_at	4,895	4,999	5,225	5,183	5,259	5,355
216370_s_at	6,014	6,029	6,004	6,283	6,472	6,576
216371_at	7,573	7,760	7,612	8,455	8,125	8,456
216372_at	5,629	5,235	5,594	6,156	6,225	6,299
216373_at	4,267	4,265	4,518	4,217	3,985	3,956
216374_at	6,013	5,677	5,803	6,680	6,777	6,933
216375_s_at	7,377	7,249	7,050	7,489	7,393	7,489
216376_x_at	6,488	6,656	6,811	6,467	6,567	6,536
216377_x_at	7,167	6,923	7,284	7,803	7,342	7,755
216378_at	4,134	4,327	3,996	4,460	4,321	4,633
216379_x_at	13,468	13,447	13,573	6,947	7,127	6,818
216380_x_at	9,478	9,650	9,728	9,417	8,907	9,561
216381_x_at	7,710	7,628	7,603	8,734	7,881	8,492
216382_s_at	7,818	8,107	7,875	7,663	8,042	8,352
216383_at	7,877	8,072	8,377	8,684	7,955	8,763
216384_x_at	10,579	10,588	10,486	10,288	9,966	9,845
216385_at	7,360	7,220	7,512	7,724	7,525	7,815
216386_at	5,037	5,174	5,205	5,941	5,880	6,058
216387_x_at	9,759	9,917	9,453	9,643	9,593	9,629
216388_s_at	6,666	6,694	6,505	6,333	6,448	6,374
216389_s_at	7,392	7,404	7,468	7,521	7,212	7,459
216390_at	6,443	6,630	6,760	6,827	6,942	7,083
216391_s_at	7,328	7,335	7,478	7,870	7,627	7,860
216392_s_at	8,575	8,558	8,522	8,357	8,330	8,480
216393_at	5,626	5,842	5,829	5,721	5,888	5,800
216394_x_at	7,173	7,170	7,227	7,891	7,645	7,898
216395_at	7,251	7,340	7,508	7,895	8,067	8,110
216396_s_at	11,117	10,903	11,435	11,028	10,406	11,065
216397_s_at	8,439	8,246	8,411	9,247	9,386	9,729
216398_at	6,281	6,159	6,877	6,981	6,759	7,063
216399_s_at	6,516	7,531	6,075	5,654	6,097	5,939
216400_at	5,975	5,771	6,120	5,891	5,765	5,738
216401_x_at	6,224	5,787	5,824	6,453	6,275	6,561
216402_at	6,081	5,866	6,001	6,552	6,332	6,388
216403_at	4,875	4,723	4,416	5,394	5,160	4,997
216404_at	6,410	6,187	6,426	6,431	6,441	6,349
216405_at	5,701	5,368	6,438	8,092	8,322	8,406
216406_at	4,128	4,388	4,422	4,665	4,633	4,713
216407_at	6,831	6,640	7,086	7,199	7,462	7,554
216408_at	4,274	4,797	4,440	5,342	5,154	5,368
216409_at	6,633	6,206	6,673	7,204	7,011	7,104
216410_at	5,513	5,470	5,324	6,069	6,015	6,184

216411_s_at	6,245	6,592	6,472	6,864	6,853	6,819
216412_x_at	6,838	6,706	6,947	6,975	7,238	7,264
216413_at	5,708	5,664	5,650	6,323	5,993	6,175
216414_at	5,606	5,723	5,664	6,000	6,042	5,944
216415_at	3,786	3,899	3,906	4,117	3,853	3,929
216416_at	6,406	6,379	6,652	7,146	6,913	7,184
216417_x_at	6,600	6,556	6,922	7,256	6,816	7,480
216418_at	7,478	7,156	7,403	7,569	7,525	7,626
216419_at	7,624	7,684	7,471	8,565	7,898	8,094
216420_at	5,100	5,316	5,181	5,614	5,529	5,672
216421_at	6,000	5,945	6,303	6,697	6,135	6,438
216422_at	6,421	6,097	6,573	7,413	6,879	7,450
216423_at	6,650	6,248	6,488	6,807	6,726	6,789
216424_at	4,887	5,157	5,068	4,905	4,724	4,868
216425_at	6,682	7,350	7,050	7,923	8,119	7,534
216426_at	7,216	7,106	7,301	8,661	8,292	8,445
216427_at	6,070	6,359	6,211	7,275	7,051	7,344
216428_x_at	6,490	6,155	6,172	6,181	6,082	6,144
216429_at	5,999	5,935	6,205	7,170	6,845	7,129
216430_x_at	6,904	6,853	6,803	6,932	6,996	6,944
216431_at	5,991	6,323	6,625	6,511	6,269	6,431
216432_at	5,943	5,891	6,215	6,258	6,231	6,083
216433_s_at	5,429	5,132	5,565	6,045	6,258	6,320
216434_at	7,913	7,282	7,668	8,792	8,599	8,701
216435_at	3,525	3,755	3,788	3,870	3,677	3,650
216436_at	4,413	4,450	4,127	4,683	4,803	4,596
216437_at	4,746	4,586	4,931	5,241	5,048	5,066
216438_s_at	13,242	13,328	13,159	13,414	13,601	14,062
216439_at	7,040	7,086	7,302	7,194	7,322	7,308
216440_at	4,968	5,147	5,154	5,299	5,164	5,167
216441_at	3,820	3,847	3,937	4,247	4,048	4,009
216442_x_at	9,863	10,136	10,711	13,499	14,551	14,458
216443_at	4,517	4,746	4,764	4,840	4,736	4,938
216444_at	4,641	4,713	4,792	5,048	4,891	4,968
216445_at	4,594	4,965	4,510	5,030	5,046	4,907
216446_at	6,757	6,864	7,208	6,856	7,111	7,081
216447_at	4,239	4,167	4,257	4,568	4,553	4,695
216448_at	6,653	6,882	6,494	6,835	7,108	7,054
216449_x_at	9,337	9,600	9,435	9,586	9,589	9,559
216450_x_at	8,148	8,137	8,299	7,714	7,973	7,642
216451_at	7,193	7,211	7,321	8,039	7,664	7,822
216452_at	4,875	5,128	5,259	5,809	6,266	5,983
216453_at	5,737	5,471	5,888	5,794	5,669	5,605
216454_at	6,862	6,701	6,825	6,874	6,963	7,053
216455_at	7,527	7,283	7,633	7,720	7,728	7,821
216456_at	4,197	4,578	4,116	4,343	4,633	4,395
216457_s_at	11,261	11,113	11,750	10,733	10,274	10,481
216458_at	7,795	7,438	7,901	7,801	7,652	7,933
216459_x_at	6,450	6,362	6,240	6,144	6,765	6,284
216460_at	7,355	7,267	7,246	7,687	7,722	7,705
216461_at	6,714	6,492	7,008	7,547	7,497	7,408
216462_at	4,508	4,378	4,527	4,998	4,969	4,977
216463_at	4,944	5,432	5,124	5,250	5,555	5,448
216464_x_at	6,787	6,829	6,654	7,505	7,489	7,598
216465_at	5,694	5,851	5,460	6,340	6,603	6,575
216466_at	3,958	3,822	3,936	4,252	5,596	4,207

216467_s_at	5,545	6,033	5,177	5,840	5,832	5,885
216468_s_at	6,899	7,203	6,174	5,007	5,168	5,177
216469_at	4,311	4,797	4,510	4,677	4,726	4,765
216470_x_at	6,087	6,725	6,925	8,778	7,897	8,133
216471_x_at	6,453	6,295	6,271	7,010	7,029	7,129
216472_at	8,344	7,857	8,349	7,168	7,379	6,999
216473_x_at	7,964	8,054	7,951	7,943	8,038	8,013
216474_x_at	7,230	7,091	7,228	7,427	7,266	7,428
216475_at	6,842	6,860	6,859	7,582	7,280	7,406
216476_at	6,937	6,675	6,750	7,051	6,942	6,842
216477_at	5,299	5,291	5,325	5,582	5,605	5,782
216478_at	5,165	5,026	5,293	5,235	5,358	5,281
216479_at	6,676	7,053	6,413	7,816	7,320	7,576
216480_x_at	4,699	4,540	5,155	5,009	5,070	4,726
216481_at	7,989	7,839	7,784	7,946	7,593	7,617
216482_x_at	5,979	6,168	5,620	6,424	6,431	6,398
216483_s_at	8,738	8,363	8,574	10,273	9,812	10,570
216484_x_at	10,873	10,719	11,364	10,910	9,815	10,498
216485_s_at	7,031	6,764	7,042	7,151	6,909	7,011
216486_x_at	7,718	7,272	7,429	7,252	7,149	7,274
216487_at	5,000	5,015	5,175	5,301	5,463	5,444
216488_s_at	7,331	6,930	7,071	7,878	7,930	8,071
216489_at	5,612	5,731	5,393	6,320	6,193	6,043
216490_x_at	8,056	7,544	8,092	9,105	8,788	8,935
216491_x_at	6,340	6,049	6,169	7,265	7,303	7,387
216492_at	7,335	7,129	7,369	7,717	7,600	7,750
216493_s_at	9,283	9,694	8,737	6,845	6,544	6,846
216494_at	6,353	6,027	6,147	6,111	6,321	6,238
216495_x_at	8,515	8,284	8,520	9,250	9,059	9,313
216496_s_at	6,155	6,455	6,425	6,711	6,827	6,606
216497_at	9,361	9,201	9,572	8,948	8,825	8,122
216498_at	5,648	5,681	5,400	6,350	6,187	6,112
216499_at	7,549	7,253	7,329	8,018	8,293	8,346
216500_at	7,386	7,398	7,371	7,911	7,542	7,616
216501_at	5,839	5,701	5,736	6,485	6,263	6,564
216502_at	5,668	5,692	5,993	5,738	5,791	5,600
216503_s_at	7,927	7,760	7,720	7,248	6,778	7,295
216504_s_at	4,274	4,361	4,558	4,623	4,530	4,802
216505_x_at	10,044	10,441	10,306	10,602	9,491	10,394
216506_x_at	7,196	7,246	7,335	7,085	7,224	7,278
216507_at	6,669	6,113	6,400	7,228	6,853	6,950
216508_x_at	9,953	9,854	9,785	9,798	8,890	9,538
216509_x_at	6,923	6,691	6,799	6,485	6,985	6,481
216510_x_at	5,800	5,813	5,932	6,806	6,495	6,610
216511_s_at	9,978	9,803	10,092	7,189	7,871	7,387
216512_s_at	6,465	6,467	6,257	6,933	6,816	7,073
216513_at	3,691	4,229	3,690	4,813	4,552	4,569
216514_at	5,811	5,839	5,581	6,329	6,021	6,238
216515_x_at	11,573	11,540	11,869	11,057	10,984	11,034
216516_at	5,330	5,781	5,919	5,901	5,677	5,766
216517_at	4,989	5,346	4,884	6,192	5,507	5,449
216518_at	3,956	4,090	4,005	3,867	3,874	3,660
216519_s_at	5,689	5,419	5,458	5,649	5,504	5,565
216520_s_at	13,566	13,753	13,478	13,134	12,595	13,600
216521_s_at	7,083	6,183	6,466	6,510	6,120	5,849
216522_at	5,833	5,623	5,701	6,142	6,150	6,076

216524_x_at	9,198	9,093	9,117	8,393	9,314	8,681
216525_x_at	9,258	8,975	9,063	8,761	8,320	8,779
216526_x_at	11,128	11,594	11,652	11,886	11,625	12,134
216527_at	6,647	6,134	6,312	5,500	6,327	5,546
216528_at	6,829	6,889	6,510	7,079	7,189	7,252
216529_at	3,721	3,971	4,031	3,896	3,992	3,986
216530_at	7,358	7,409	7,300	7,387	7,404	7,225
216531_at	6,589	6,529	6,496	6,821	6,703	6,963
216532_x_at	8,687	8,488	8,669	9,043	8,349	8,733
216533_at	3,521	3,500	3,626	3,890	3,669	3,597
216534_at	5,722	6,227	6,187	5,795	5,821	6,006
216535_at	7,211	6,875	6,979	6,910	6,736	7,067
216536_at	5,708	5,692	5,558	5,786	6,103	5,984
216537_s_at	7,652	7,458	7,700	7,886	7,964	7,864
216538_at	6,959	7,139	6,650	6,784	7,262	7,167
216539_at	4,877	4,294	4,910	4,991	4,777	4,584
216540_at	6,793	6,621	6,562	7,374	6,858	7,268
216541_x_at	6,391	6,410	6,427	6,441	6,461	6,520
216542_x_at	7,310	7,495	7,564	8,311	8,234	8,221
216543_at	5,219	5,539	5,442	5,748	5,507	5,612
216544_at	5,192	5,025	5,189	6,043	6,025	5,777
216545_at	6,604	6,440	7,071	7,337	6,822	7,132
216546_s_at	5,974	5,692	5,860	6,494	6,449	6,396
216547_at	9,492	9,576	9,762	10,048	9,217	10,074
216548_x_at	8,904	8,766	8,582	8,206	7,913	7,938
216549_s_at	5,051	5,110	5,161	5,829	5,775	5,958
216550_x_at	8,547	8,724	7,596	6,850	9,324	6,652
216551_x_at	8,258	8,203	8,597	8,468	8,419	8,722
216552_x_at	6,181	5,951	6,209	7,164	6,867	6,879
216553_x_at	4,678	5,095	5,599	6,464	6,226	6,490
216554_s_at	7,893	8,004	7,970	8,063	7,970	8,341
216555_at	7,872	7,845	7,848	7,239	7,163	7,339
216556_x_at	4,536	4,815	4,273	4,958	5,018	5,171
216557_x_at	6,322	6,477	6,085	7,035	7,124	7,222
216558_x_at	4,100	4,447	4,389	5,016	4,754	5,133
216559_x_at	12,654	12,533	12,154	11,236	11,175	10,992
216560_x_at	6,985	7,033	6,691	7,361	7,142	7,495
216561_x_at	5,847	5,755	5,581	6,952	6,406	6,561
216562_at	6,974	6,810	7,106	7,697	7,680	7,696
216563_at	6,944	7,475	6,705	5,483	7,652	5,584
216564_at	6,729	6,666	6,668	7,386	7,026	7,264
216565_x_at	8,375	8,492	8,666	8,695	8,201	8,727
216566_at	5,075	5,272	5,560	5,609	5,298	5,560
216567_at	6,280	6,353	6,460	6,729	6,696	7,036
216568_x_at	7,473	7,492	7,083	7,645	7,392	7,691
216569_at	6,632	6,498	6,420	7,026	6,860	7,115
216570_x_at	10,121	10,405	10,211	10,740	10,297	10,687
216571_at	7,683	7,293	7,629	7,917	7,919	8,025
216572_at	6,098	5,996	6,433	6,883	6,500	6,500
216573_at	4,837	5,150	4,683	5,265	4,925	5,323
216574_s_at	8,437	8,525	8,284	8,895	8,544	8,717
216575_at	3,917	3,602	3,690	4,096	3,970	4,179
216576_x_at	6,247	6,046	6,218	7,000	6,066	6,470
216577_at	6,959	7,096	7,073	7,566	7,130	7,591
216578_at	5,639	5,837	6,463	6,756	6,774	6,822
216579_at	7,143	6,945	7,162	7,676	7,588	7,487

216580_at	5,515	5,502	5,358	6,678	6,224	6,363
216581_at	6,722	6,472	6,964	6,989	6,723	6,943
216582_at	6,757	6,458	6,568	7,158	6,990	7,220
216583_x_at	8,670	8,651	8,620	8,940	8,416	8,865
216584_at	7,709	7,414	7,568	8,450	8,394	8,593
216585_at	6,991	6,685	6,792	7,511	7,414	7,673
216586_at	7,025	7,157	7,143	7,383	6,775	7,131
216587_s_at	5,884	6,051	6,285	6,405	6,145	6,328
216588_at	5,279	5,426	5,318	5,566	5,633	5,621
216589_at	5,585	5,871	6,018	6,478	5,779	6,430
216590_at	4,273	4,131	4,048	4,370	4,382	4,572
216591_s_at	8,221	7,967	8,167	8,374	7,688	8,192
216592_at	4,709	4,860	4,828	5,166	4,828	4,759
216593_s_at	9,645	9,564	9,297	8,494	7,700	8,137
216594_x_at	7,539	7,979	7,807	13,209	12,699	12,990
216595_at	4,186	4,397	4,276	4,029	3,967	3,996
216596_at	6,562	6,719	6,572	6,554	6,665	6,862
216597_at	7,351	7,347	7,632	8,044	7,805	8,216
216598_s_at	6,574	6,577	6,388	7,411	6,586	7,229
216599_x_at	6,482	6,753	6,270	6,909	7,406	7,118
216600_x_at	7,467	6,935	7,675	8,273	8,173	8,391
216601_at	7,640	7,584	7,613	8,124	8,244	8,159
216602_s_at	9,422	8,650	9,061	9,394	8,323	9,343
216603_at	8,666	8,842	8,873	6,951	7,054	6,949
216604_s_at	9,279	9,528	9,796	8,144	7,859	7,768
216605_s_at	5,678	5,610	5,407	5,980	5,992	6,415
216606_x_at	9,357	8,879	9,390	8,868	8,848	9,269
216607_s_at	9,164	9,175	9,016	10,025	9,499	9,797
216608_at	7,221	6,848	7,418	7,472	7,403	7,648
216609_at	7,787	8,233	8,024	8,012	8,782	8,921
216610_at	5,324	5,010	4,924	5,254	5,173	5,408
216611_s_at	6,156	6,127	6,356	6,360	6,482	6,305
216612_x_at	5,632	5,764	5,869	6,336	6,721	6,676
216613_at	4,237	4,329	4,643	5,073	5,018	5,184
216614_at	6,508	6,188	6,463	6,605	6,530	6,570
216615_s_at	8,795	8,813	8,497	7,502	7,322	7,441
216616_at	6,101	5,529	5,977	6,707	6,361	6,680
216617_s_at	5,999	5,718	5,856	7,608	7,720	7,876
216618_at	4,847	4,482	5,087	5,227	4,969	4,636
216619_at	4,172	4,306	3,975	4,325	4,234	4,290
216620_s_at	10,554	10,834	10,793	8,664	9,942	9,291
216621_at	6,347	5,701	5,646	5,797	5,835	5,818
216622_at	5,972	5,832	5,924	6,649	6,348	6,225
216623_x_at	10,215	10,403	10,589	4,234	4,123	4,066
216624_s_at	6,403	6,125	5,951	6,354	5,993	6,382
216625_at	3,515	3,668	3,392	4,126	4,087	4,436
216626_at	6,175	6,380	6,410	6,382	6,491	6,239
216627_s_at	4,298	5,062	4,525	5,449	5,093	5,035
216628_at	7,046	7,037	7,068	5,289	5,684	5,347
216629_at	5,685	5,485	5,583	6,360	6,301	6,442
216630_at	6,542	6,390	6,574	6,763	6,670	6,784
216631_s_at	6,439	6,641	6,521	6,919	6,751	6,768
216632_at	5,095	5,226	5,348	5,355	5,143	5,209
216633_s_at	7,067	6,760	7,151	7,221	7,107	7,367
216634_at	4,379	4,335	4,199	4,379	4,135	4,264
216635_at	6,623	6,832	6,183	7,217	7,238	7,113

216636_at	5,664	5,688	5,631	5,742	5,711	5,762
216637_at	7,640	7,413	7,796	7,725	7,736	7,552
216638_s_at	4,553	4,673	4,204	5,110	5,082	5,150
216639_at	3,716	3,755	3,604	4,033	3,912	4,134
216640_s_at	11,690	11,557	11,301	11,198	10,954	11,052
216641_s_at	6,562	6,579	6,803	6,954	6,877	7,031
216642_at	4,872	4,888	4,949	4,912	5,090	5,141
216643_at	4,602	4,570	4,392	4,644	4,402	4,490
216644_at	5,505	5,407	5,801	6,244	5,757	6,099
216645_at	9,173	8,876	9,144	9,065	9,179	8,937
216646_at	7,328	7,231	6,978	7,317	7,181	7,481
216647_at	7,550	7,510	7,683	8,374	8,485	8,735
216648_s_at	6,643	6,453	6,783	7,115	7,193	7,342
216649_at	5,347	5,300	5,504	6,374	6,434	6,513
216650_at	7,566	7,313	7,522	8,277	7,811	8,243
216651_s_at	6,102	6,186	6,426	6,521	6,533	6,706
216652_s_at	8,902	9,060	9,111	9,665	8,894	9,852
216653_at	4,147	4,723	3,881	3,981	3,971	4,010
216654_at	6,693	6,681	6,790	7,128	6,877	6,829
216655_s_at	7,096	6,947	6,501	7,337	7,302	7,325
216656_at	7,236	6,970	6,730	7,465	7,636	7,708
216657_at	5,319	5,064	5,140	5,112	5,294	5,358
216658_at	6,959	6,543	6,570	8,171	8,127	8,420
216659_at	3,665	3,836	3,591	4,188	4,343	4,424
216660_at	5,326	5,400	5,440	5,604	5,872	5,510
216661_x_at	6,407	5,849	6,430	7,028	6,939	7,179
216662_at	6,834	6,844	6,814	6,613	6,348	6,442
216663_s_at	6,218	6,151	6,283	6,496	6,620	6,750
216664_at	5,507	5,100	5,091	5,467	5,535	5,501
216665_s_at	4,331	4,105	4,242	4,896	4,794	4,948
216666_at	4,496	4,939	4,863	5,279	5,029	5,029
216667_at	7,153	7,150	7,023	7,427	7,584	7,670
216668_at	7,556	7,347	7,552	7,971	7,720	7,689
216669_at	4,774	5,004	5,084	5,206	4,909	5,124
216670_at	6,634	6,438	6,841	7,592	7,321	7,503
216671_x_at	8,647	8,804	8,974	9,231	8,965	9,595
216672_s_at	3,848	4,117	3,798	3,917	3,882	4,142
216673_at	5,880	6,307	6,378	7,158	7,150	7,201
216674_at	5,758	5,782	5,326	6,233	6,261	6,609
216675_at	6,884	6,800	6,902	6,759	6,905	6,929
216676_x_at	6,195	5,934	6,513	7,408	7,206	7,095
216677_at	6,149	5,878	5,887	6,480	6,183	6,217
216678_at	8,397	8,075	8,106	8,980	8,299	8,804
216679_at	7,655	7,305	7,251	8,123	8,266	8,172
216680_s_at	7,850	7,900	8,191	7,538	7,321	7,589
216681_at	4,714	4,593	4,702	5,257	4,800	5,163
216682_s_at	8,611	7,825	7,710	6,326	6,284	6,274
216683_at	4,826	4,720	4,616	4,702	4,832	4,659
216684_s_at	6,018	5,887	6,016	7,519	7,659	7,813
216685_s_at	7,556	7,516	7,614	7,627	7,330	7,800
216686_at	4,276	4,051	4,170	4,718	4,803	4,711
216687_x_at	5,627	5,843	5,581	5,804	5,525	5,609
216688_at	6,659	6,367	6,651	7,071	7,092	7,141
216689_x_at	7,753	7,811	7,862	8,464	8,163	8,507
216690_at	5,388	5,390	5,599	5,879	5,886	5,851
216691_at	5,163	4,971	5,390	5,638	5,466	5,794

216692_at	6,393	6,796	6,575	6,451	6,996	6,804
216693_x_at	10,507	10,478	10,161	8,993	8,661	9,062
216694_at	7,560	7,350	7,016	7,746	7,620	7,583
216695_s_at	5,912	5,885	5,938	5,978	6,345	6,472
216696_s_at	7,375	7,151	7,314	7,936	7,632	7,889
216697_at	3,987	4,155	4,196	4,059	4,204	4,263
216698_x_at	7,623	7,963	8,128	8,640	7,890	8,808
216699_s_at	7,234	7,613	7,448	7,897	7,355	7,724
216700_at	6,862	6,924	6,945	7,282	7,401	7,139
216701_at	6,205	6,792	6,929	7,084	6,793	6,950
216702_x_at	7,472	7,474	7,594	7,821	8,059	8,160
216703_at	4,679	4,809	5,058	5,115	4,774	5,000
216704_at	6,458	5,875	6,569	7,490	7,759	7,851
216705_s_at	7,620	8,373	7,824	7,417	6,975	7,397
216706_x_at	6,142	6,412	6,425	7,115	7,164	7,245
216707_at	7,390	7,188	7,473	8,129	8,272	8,389
216708_x_at	6,240	6,254	6,003	6,673	6,882	6,924
216709_at	4,154	4,040	4,154	4,490	4,410	4,353
216710_x_at	6,301	6,464	6,378	6,468	6,280	6,480
216711_s_at	7,482	7,347	7,077	7,294	7,133	7,379
216712_at	4,047	4,029	4,254	4,430	4,290	4,474
216713_at	8,440	8,314	7,695	7,199	8,056	6,573
216714_at	6,325	6,253	6,332	6,675	6,716	6,952
216715_at	7,481	6,999	7,599	8,466	8,560	8,728
216716_at	5,191	5,741	5,731	6,848	6,785	7,221
216717_at	5,466	4,927	5,327	6,021	6,050	6,152
216718_at	4,121	4,631	5,153	5,645	5,897	5,957
216719_s_at	4,284	4,336	4,186	4,519	4,442	4,451
216720_at	5,096	4,609	4,721	5,213	5,227	5,283
216721_at	6,347	6,131	6,290	6,814	7,066	7,039
216722_at	3,791	3,976	3,997	3,971	3,994	3,958
216723_at	5,108	5,363	4,801	5,729	5,677	5,690
216724_at	4,880	5,014	5,701	6,430	6,614	6,357
216725_at	6,894	6,668	7,054	7,586	7,446	7,644
216726_at	6,355	5,999	5,740	6,521	6,103	6,398
216727_at	6,153	6,406	6,229	6,387	6,150	6,216
216728_at	4,757	5,054	5,117	5,134	5,163	4,758
216729_at	6,851	6,765	6,688	7,709	7,911	7,744
216730_at	7,458	7,578	7,430	8,279	8,093	8,102
216731_s_at	4,973	4,835	4,784	5,452	5,608	5,775
216732_at	5,522	6,052	5,402	5,730	5,379	5,722
216733_s_at	9,101	9,955	8,770	5,078	5,007	4,928
216734_s_at	6,817	7,022	6,859	8,086	8,067	8,165
216735_x_at	5,053	5,011	5,257	5,193	5,139	4,838
216736_at	6,967	6,660	6,771	7,646	7,402	7,241
216737_at	3,534	3,607	3,566	3,552	3,618	3,634
216738_at	7,106	6,948	7,098	7,068	6,962	6,900
216739_at	7,240	7,182	7,146	7,416	7,648	7,561
216740_at	4,804	4,795	5,020	5,042	4,969	5,092
216741_at	6,686	6,756	7,002	7,268	7,326	7,250
216742_at	5,211	4,870	5,309	5,818	5,303	5,493
216743_at	6,521	6,332	6,608	6,746	6,412	6,489
216744_at	5,840	5,785	5,932	5,927	5,939	5,865
216745_x_at	8,768	8,516	8,691	7,859	9,124	8,325
216746_at	4,816	4,398	4,960	4,545	4,374	4,433
216747_at	7,403	7,184	7,315	8,175	8,153	8,116

216748_at	5,171	5,342	5,705	5,461	5,311	5,555
216749_at	5,631	5,923	5,858	6,035	5,732	5,768
216750_at	6,785	6,500	6,862	7,706	7,474	7,565
216751_at	7,119	7,263	7,120	6,844	7,080	6,891
216752_at	3,919	3,930	3,870	4,311	4,386	4,478
216753_at	5,640	5,712	5,892	5,533	5,299	5,298
216754_at	6,506	6,029	6,268	6,594	6,054	6,550
216755_at	7,583	7,760	7,715	7,796	8,104	7,637
216756_at	5,867	5,931	6,029	6,130	6,974	6,552
216757_at	5,478	5,557	5,842	5,380	5,525	5,314
216758_at	3,889	4,046	4,270	4,100	4,325	4,040
216759_at	6,793	6,818	6,854	7,200	7,075	7,198
216760_at	6,715	6,744	6,770	7,444	7,217	7,397
216761_at	6,196	5,842	6,790	6,002	6,261	6,060
216762_at	7,346	7,000	6,942	7,138	7,241	7,027
216763_at	5,389	5,418	5,715	5,977	5,931	6,094
216764_at	5,261	5,504	5,284	5,601	5,462	5,359
216765_at	4,787	4,802	4,687	3,746	4,039	3,934
216766_at	4,767	4,942	4,627	4,478	4,469	4,358
216767_at	4,897	4,882	5,051	4,922	4,757	5,074
216768_x_at	7,723	7,786	7,353	7,799	7,931	7,794
216769_x_at	5,949	6,365	5,910	6,746	7,379	7,200
216770_at	5,351	6,025	5,084	6,238	6,069	6,331
216771_at	5,326	5,461	5,539	5,743	5,889	5,818
216772_at	5,256	5,506	5,749	5,802	5,825	5,814
216773_at	4,832	4,942	4,972	5,081	5,097	5,031
216774_at	6,267	6,567	6,327	7,022	6,771	6,631
216775_at	3,647	4,079	3,687	3,691	3,747	3,860
216776_at	6,711	6,803	6,758	6,902	6,819	6,935
216777_at	5,179	4,970	5,458	5,499	5,545	5,534
216778_s_at	4,491	4,525	4,724	4,797	4,616	4,702
216779_at	5,884	6,124	5,833	6,418	6,187	6,418
216780_at	4,578	4,670	4,880	4,918	4,945	5,039
216781_at	6,488	6,530	6,414	6,705	6,671	6,696
216782_at	6,077	5,667	5,636	5,821	5,692	5,772
216783_at	4,799	4,815	4,747	4,626	4,892	4,890
216784_at	7,707	7,504	7,958	9,126	8,854	9,180
216785_at	5,728	5,792	5,749	6,140	5,997	6,099
216786_at	5,454	5,220	4,985	5,513	5,515	5,522
216787_at	5,822	5,674	6,321	6,467	6,478	6,481
216788_at	5,184	5,073	5,341	5,175	5,063	4,987
216789_at	7,642	7,637	7,871	7,863	7,637	7,603
216790_at	6,843	6,581	6,605	7,096	6,784	6,964
216791_at	8,004	8,014	8,109	8,294	8,194	8,177
216792_at	6,145	6,158	6,125	6,581	6,533	6,690
216793_x_at	4,427	4,324	4,366	4,179	4,225	4,198
216794_at	7,006	7,043	7,158	7,464	7,451	7,457
216795_at	4,086	4,030	4,226	4,107	4,268	4,208
216796_s_at	5,119	5,645	4,724	6,161	6,053	6,089
216797_at	4,947	5,086	5,225	5,793	6,053	5,764
216798_at	6,100	5,974	5,933	6,296	5,893	5,765
216799_at	6,508	5,777	6,445	6,362	6,555	6,587
216800_at	6,292	6,121	6,103	6,802	6,223	6,441
216801_at	6,758	7,034	6,365	7,763	7,465	7,908
216802_at	4,303	4,141	4,343	4,913	4,637	4,767
216803_at	5,192	5,269	5,353	5,571	5,793	5,603

216804_s_at	7,465	7,424	7,357	8,930	9,370	9,478
216805_at	4,281	4,546	4,577	4,695	4,636	4,796
216806_at	10,295	10,563	10,576	10,274	9,686	10,176
216807_at	6,414	6,420	6,547	6,916	6,704	6,692
216808_at	5,896	5,944	5,617	6,531	6,809	6,782
216809_at	6,067	6,171	6,115	6,076	6,191	6,092
216810_at	3,950	4,048	3,821	4,128	3,930	4,143
216811_at	6,887	6,723	6,536	7,152	7,546	7,521
216812_at	5,160	5,419	5,301	5,835	5,666	5,434
216813_at	5,251	4,758	5,116	5,572	6,010	5,513
216814_at	4,669	4,819	4,555	5,362	5,045	5,064
216815_at	5,566	5,551	5,680	6,027	5,720	6,222
216816_at	6,476	6,375	6,322	7,097	6,800	6,841
216817_s_at	6,574	6,224	6,375	7,298	7,474	7,172
216818_s_at	5,667	5,399	5,797	6,261	6,151	6,047
216819_at	4,648	4,605	4,742	4,898	4,889	4,796
216820_at	6,393	6,278	6,136	6,792	6,787	6,991
216821_at	6,323	6,566	6,576	7,406	7,375	7,609
216822_x_at	7,779	7,654	7,664	7,758	7,741	7,691
216823_at	7,607	7,466	7,260	7,782	7,432	7,819
216824_at	6,652	6,803	6,619	7,031	6,872	7,115
216825_s_at	5,369	5,585	5,652	5,327	5,340	5,566
216826_at	5,980	6,114	6,179	6,429	6,299	6,298
216827_at	4,873	4,818	4,806	4,935	4,694	4,639
216828_at	5,692	5,431	5,083	6,207	5,899	5,566
216829_at	5,367	5,448	5,552	6,169	5,847	6,120
216830_at	5,682	5,988	5,930	5,968	5,999	6,381
216831_s_at	6,136	6,206	6,198	6,874	6,904	7,241
216832_at	6,507	6,608	6,740	7,274	7,229	7,315
216833_x_at	6,405	6,346	6,371	6,977	7,069	7,061
216834_at	3,663	3,707	3,798	3,575	3,775	3,664
216835_s_at	8,656	8,630	8,537	9,827	9,618	10,198
216836_s_at	9,891	9,501	10,124	8,305	8,538	8,813
216837_at	6,383	6,385	6,742	6,483	6,598	6,466
216838_at	6,377	6,535	6,130	6,546	6,057	6,157
216839_at	3,766	3,766	3,760	3,871	3,758	3,787
216840_s_at	7,117	6,999	6,974	6,391	6,367	6,499
216841_s_at	8,881	8,900	8,859	12,049	9,956	10,174
216842_x_at	7,132	7,176	7,213	7,076	7,172	6,963
216843_x_at	8,916	8,668	8,945	8,671	8,133	8,623
216844_at	5,257	5,269	5,300	6,593	6,590	6,748
216845_x_at	8,265	8,020	8,256	8,889	8,863	8,940
216846_at	7,455	7,287	7,627	7,134	7,051	6,791
216847_at	5,948	6,084	6,291	6,762	6,960	7,278
216848_at	6,306	6,401	6,524	6,949	7,087	7,123
216849_at	7,816	7,460	7,616	8,188	7,835	8,136
216850_at	6,844	6,686	6,769	5,535	5,504	5,772
216851_at	6,249	6,395	6,296	5,845	5,854	5,596
216852_x_at	7,496	7,233	7,593	7,674	7,439	7,860
216853_x_at	7,010	7,449	7,488	7,884	7,832	8,024
216854_at	6,527	6,850	6,630	6,798	6,770	6,667
216855_s_at	7,141	6,730	7,102	7,112	7,115	7,498
216856_s_at	5,893	5,289	5,689	5,914	5,672	5,962
216857_at	5,822	6,038	5,906	5,953	5,900	6,205
216858_x_at	9,531	9,097	9,186	8,784	9,423	9,016
216859_x_at	8,089	7,635	7,593	7,765	8,045	7,720

216860_s_at	8,023	8,576	8,687	7,783	7,445	7,870
216861_at	5,658	5,573	5,039	5,662	5,556	5,719
216862_s_at	8,995	9,029	8,750	9,821	9,455	9,628
216863_s_at	8,740	8,771	8,876	7,981	7,825	7,776
216864_at	5,120	5,272	5,201	5,148	4,926	5,137
216865_at	5,044	4,844	5,036	5,216	4,967	5,213
216866_s_at	6,658	5,932	5,996	6,876	6,813	6,468
216867_s_at	8,021	8,312	8,495	7,542	7,095	7,436
216868_s_at	4,344	4,648	4,244	4,482	4,313	4,256
216869_at	6,640	6,661	6,744	7,649	7,748	7,702
216870_x_at	10,010	9,422	9,868	8,426	8,782	8,528
216871_at	4,581	4,873	4,775	5,039	4,862	5,128
216872_at	4,448	4,309	4,569	4,539	4,227	4,575
216873_s_at	8,366	8,012	8,528	9,324	9,223	9,611
216874_at	5,965	6,146	5,944	4,793	4,899	4,791
216875_x_at	7,946	7,860	8,194	9,485	9,727	9,798
216876_s_at	3,946	4,458	4,073	4,579	4,490	4,372
216877_at	6,544	6,414	6,814	6,044	5,929	5,911
216878_x_at	6,506	6,441	6,557	7,551	7,518	7,681
216879_at	6,398	6,117	6,018	6,459	6,194	6,330
216880_at	5,257	4,815	5,216	6,380	5,430	5,431
216881_x_at	7,483	7,042	7,146	7,559	7,360	7,553
216882_s_at	7,399	7,689	7,595	7,772	7,818	7,597
216883_x_at	8,756	8,747	8,730	8,512	8,147	8,542
216884_at	4,792	4,885	4,922	5,073	5,073	4,695
216885_s_at	7,642	8,025	7,706	7,262	7,095	7,093
216886_at	6,228	6,146	6,151	6,733	6,484	6,672
216887_s_at	5,753	5,953	5,801	6,222	6,202	6,432
216888_at	7,733	7,214	7,345	8,150	8,381	8,360
216889_s_at	7,693	7,770	7,829	8,381	8,403	8,679
216890_at	6,857	6,893	7,068	7,591	7,622	7,547
216891_at	5,533	5,436	5,747	6,218	6,337	6,298
216892_at	7,297	7,127	7,536	8,069	8,224	8,154
216893_s_at	4,172	4,333	4,306	4,222	4,187	4,173
216894_x_at	8,593	8,824	8,216	6,550	6,449	6,756
216895_at	5,468	5,217	5,683	6,096	5,901	5,967
216896_at	4,639	4,512	4,605	5,091	4,840	5,235
216897_s_at	7,105	7,256	7,400	7,264	6,846	7,207
216898_s_at	6,734	6,645	6,777	7,293	7,033	7,207
216899_s_at	5,561	5,493	5,453	7,034	6,845	7,080
216900_s_at	5,427	5,719	5,512	6,192	6,153	6,213
216901_s_at	4,358	5,063	4,560	5,489	5,538	5,693
216902_s_at	7,759	7,840	7,317	7,166	7,609	7,184
216903_s_at	8,925	9,105	8,974	9,204	8,859	9,236
216904_at	6,502	6,205	6,629	6,722	6,657	6,909
216905_s_at	5,517	5,255	5,857	5,807	5,808	5,918
216906_at	6,072	6,260	6,256	6,976	6,757	6,972
216907_x_at	6,327	6,101	6,365	7,163	7,094	7,005
216908_x_at	7,998	7,849	7,924	7,642	7,545	7,431
216909_at	6,974	6,685	6,995	7,362	7,122	7,439
216910_at	6,433	6,370	6,336	6,481	6,714	6,711
216911_s_at	7,134	6,929	7,392	8,152	8,186	8,249
216912_at	6,838	6,647	6,536	7,229	7,142	6,930
216913_s_at	8,599	8,667	9,063	8,749	8,607	8,763
216914_at	5,854	6,122	5,946	6,388	6,057	6,423
216915_s_at	5,326	6,093	5,214	5,452	6,044	5,817

216916_s_at	6,947	6,614	6,762	7,408	7,026	7,285
216917_s_at	4,107	3,853	3,904	3,965	3,837	4,288
216918_s_at	7,543	7,856	7,655	8,230	8,064	8,022
216919_at	7,071	6,744	6,942	7,385	7,068	7,316
216920_s_at	5,740	6,068	6,349	7,130	6,961	7,101
216921_s_at	4,913	4,753	4,900	5,034	5,198	5,063
216922_x_at	4,666	4,694	4,613	5,096	4,998	5,051
216923_at	6,143	6,139	6,164	6,519	6,340	6,654
216924_s_at	7,285	7,724	7,842	8,210	8,199	8,110
216925_s_at	7,388	7,262	7,569	8,316	7,893	8,290
216926_s_at	5,761	5,584	5,276	6,504	6,196	6,290
216927_at	5,591	5,945	5,898	6,371	5,905	6,042
216928_at	6,344	6,155	6,415	6,578	6,347	6,672
216929_x_at	7,447	7,172	7,530	9,515	9,645	9,691
216930_at	6,309	6,121	6,075	7,057	7,049	7,065
216931_at	6,351	6,396	6,591	6,826	6,404	6,611
216932_at	6,771	6,688	6,708	7,525	7,301	7,018
216933_x_at	3,873	3,654	3,770	3,785	4,025	4,024
216934_at	5,083	5,664	5,328	6,145	5,613	6,038
216935_at	6,576	6,589	6,544	7,016	7,255	7,485
216936_at	7,052	6,762	6,597	7,461	7,293	7,564
216937_s_at	6,773	6,311	6,422	7,651	7,073	8,020
216938_x_at	7,196	7,245	7,095	7,272	7,275	7,464
216939_s_at	5,253	4,988	4,501	5,531	5,579	5,531
216940_x_at	6,008	6,122	6,114	6,970	7,060	7,068
216941_s_at	9,396	9,511	9,074	8,044	8,286	8,096
216942_s_at	7,682	7,439	7,123	8,214	7,699	8,279
216943_at	6,947	6,769	6,839	7,568	7,328	7,588
216944_s_at	6,666	6,724	6,158	6,258	6,114	5,999
216945_x_at	9,603	9,533	9,676	7,005	7,157	6,778
216946_at	6,128	6,247	6,152	7,047	7,128	6,727
216947_at	3,919	4,292	3,978	4,247	4,318	4,020
216948_at	4,155	4,333	3,994	4,395	4,414	4,575
216949_s_at	7,358	7,421	7,404	7,994	7,756	8,229
216950_s_at	6,166	6,290	6,063	7,043	7,064	7,071
216951_at	4,934	5,297	5,427	5,311	5,159	5,300
216952_s_at	11,628	11,008	11,461	9,665	8,966	9,556
216953_s_at	5,955	5,774	5,740	6,574	6,341	6,534
216954_x_at	11,579	11,697	11,604	11,209	11,133	11,194
216955_at	7,078	7,172	7,298	7,555	7,466	7,532
216956_s_at	6,466	6,250	6,219	6,321	6,298	6,475
216957_at	6,239	5,610	5,769	5,606	5,649	5,518
216958_s_at	8,484	8,486	9,146	9,455	8,866	9,120
216959_x_at	6,276	6,315	6,183	6,911	6,817	6,942
216960_s_at	8,042	8,414	8,257	8,171	8,173	8,174
216961_s_at	6,345	6,311	6,616	6,822	5,856	6,165
216962_at	6,112	6,288	6,513	6,402	6,348	6,488
216963_s_at	8,331	7,734	8,027	8,167	8,275	8,227
216964_at	5,983	6,148	6,257	6,891	6,624	6,800
216965_x_at	6,851	6,939	7,062	7,342	7,054	7,394
216966_at	3,975	4,437	4,182	4,415	4,295	4,354
216967_at	6,866	6,988	7,285	5,089	4,804	4,798
216968_at	5,731	5,740	5,859	6,073	6,149	6,181
216969_s_at	7,684	7,621	7,774	8,204	8,140	8,340
216970_at	5,583	5,431	5,510	5,789	5,781	5,912
216971_s_at	6,478	6,308	6,317	7,046	7,071	7,345

216972_at	4,133	4,800	4,245	4,628	4,464	4,786
216973_s_at	6,412	6,135	6,319	6,951	6,660	9,339
216974_at	4,196	4,077	4,148	4,587	4,707	4,878
216975_x_at	7,535	7,567	7,270	7,246	7,121	7,407
216976_s_at	8,898	8,715	8,227	7,717	7,583	7,819
216977_x_at	11,132	11,068	10,348	9,929	9,896	9,644
216978_x_at	4,460	4,450	4,574	4,647	4,616	4,693
216979_at	5,898	5,794	5,794	6,785	6,993	6,380
216980_s_at	6,489	6,744	6,644	7,350	6,782	7,328
216981_x_at	7,854	7,644	7,801	8,579	8,290	8,527
216982_x_at	5,979	6,416	6,353	6,606	6,668	6,763
216983_s_at	6,068	6,012	5,700	6,372	6,567	6,357
216984_x_at	7,011	7,129	7,324	7,573	7,538	7,606
216985_s_at	5,414	5,969	5,232	5,458	5,395	5,491
216986_s_at	6,552	6,458	6,293	7,052	7,031	7,144
216987_at	3,448	3,679	3,503	3,573	3,472	3,600
216988_s_at	11,005	10,887	10,865	11,424	10,894	11,497
216989_at	4,955	4,772	4,845	4,985	4,704	4,815
216990_at	4,028	3,721	4,109	4,116	3,952	3,951
216991_at	6,542	6,695	6,467	6,816	6,811	6,922
216992_s_at	5,570	5,698	5,266	5,298	5,345	5,168
216993_s_at	8,227	8,166	8,147	8,905	8,755	8,712
216994_s_at	6,535	6,669	6,579	6,699	6,876	6,767
216995_x_at	6,898	7,029	7,025	8,040	7,711	8,240
216996_s_at	10,015	10,283	9,903	9,355	9,665	9,157
216997_x_at	6,219	6,440	6,723	5,235	5,354	5,355
216998_s_at	5,752	5,542	5,511	6,459	5,854	6,555
216999_at	4,378	4,377	4,315	4,398	4,195	4,172
217000_at	5,817	4,880	5,728	5,982	5,777	6,053
217001_x_at	7,877	7,531	7,780	7,817	7,677	7,758
217002_s_at	7,341	7,281	7,106	6,621	6,200	6,588
217003_s_at	5,862	5,584	6,157	5,708	5,609	5,957
217004_s_at	7,391	7,513	7,422	7,771	7,698	7,701
217005_at	6,897	7,188	6,825	7,585	7,379	7,449
217006_x_at	6,511	6,320	6,221	7,024	7,239	7,454
217007_s_at	6,869	6,553	6,598	7,275	7,207	7,345
217008_s_at	5,216	5,086	5,035	5,415	5,690	5,442
217009_at	6,449	6,322	6,456	6,788	6,559	6,731
217010_s_at	6,483	6,578	5,984	6,591	5,973	6,000
217011_at	7,633	7,597	7,591	7,892	7,689	7,739
217012_at	4,538	5,372	4,543	5,413	5,669	5,774
217013_at	6,354	6,336	6,421	6,849	6,484	6,893
217014_s_at	6,329	6,679	6,686	7,352	7,623	7,399
217015_at	4,783	4,655	4,709	5,216	5,249	5,266
217016_x_at	7,551	7,382	7,461	7,563	8,063	7,276
217017_at	6,895	6,516	6,717	7,265	6,831	7,044
217018_at	6,325	6,241	5,696	6,574	6,567	7,010
217019_at	8,284	8,246	8,128	9,219	8,170	8,754
217020_at	5,985	5,717	6,129	6,449	6,446	6,458
217021_at	5,564	5,501	5,642	5,535	5,523	5,733
217022_s_at	6,093	6,087	6,273	6,679	6,793	6,909
217023_x_at	6,979	6,839	7,156	8,155	8,315	8,431
217024_x_at	6,793	6,487	6,924	7,937	7,737	7,919
217025_s_at	9,012	8,675	9,106	8,443	7,847	8,410
217026_at	5,530	5,434	5,503	6,073	5,715	5,622
217027_x_at	8,897	8,610	8,628	8,556	8,576	8,367

217028_at	8,261	8,419	8,208	4,556	4,604	5,071
217029_at	6,869	6,966	7,126	7,218	7,176	7,300
217030_at	6,557	6,571	6,866	6,945	6,974	7,129
217031_at	5,122	4,918	5,039	5,310	5,183	5,323
217032_at	6,492	6,434	6,313	6,388	5,715	6,192
217033_x_at	6,682	6,782	6,730	7,162	6,812	7,062
217034_at	6,407	6,487	6,744	6,938	6,958	6,944
217035_at	6,914	7,144	7,142	8,002	7,432	8,054
217036_at	5,259	5,539	5,248	5,786	5,880	5,926
217037_at	4,408	3,979	4,297	4,811	4,644	5,088
217038_at	6,225	5,883	6,161	6,076	5,949	6,395
217039_x_at	7,294	7,543	7,526	8,057	7,534	7,545
217040_x_at	7,235	7,422	7,182	7,781	7,332	7,320
217041_at	7,518	7,006	7,208	7,855	8,053	8,112
217042_at	6,066	6,367	6,434	6,371	6,249	6,138
217043_s_at	9,253	9,639	8,659	8,487	9,337	8,344
217044_s_at	6,778	7,043	6,432	7,070	7,054	7,067
217045_x_at	7,849	7,976	7,969	8,645	8,676	8,679
217046_s_at	6,723	6,984	6,744	7,549	7,478	7,771
217047_s_at	9,230	9,356	9,068	7,983	9,364	7,982
217048_at	6,617	6,270	6,413	6,817	6,958	7,253
217049_x_at	5,154	5,371	5,384	4,778	5,225	4,934
217050_at	5,402	5,488	5,560	6,270	6,052	6,054
217051_s_at	7,248	7,016	7,225	7,497	7,731	7,820
217052_x_at	6,923	7,322	7,167	7,472	7,830	7,856
217053_x_at	8,130	8,750	8,523	7,610	7,544	7,556
217054_at	6,785	6,981	6,874	8,344	8,512	8,606
217055_x_at	8,010	7,941	8,016	8,595	8,616	8,517
217056_at	5,129	5,770	4,909	6,016	6,130	5,996
217057_s_at	5,606	5,906	6,110	5,918	5,411	5,895
217058_at	7,332	7,146	7,446	8,076	8,218	8,285
217059_at	5,295	4,982	5,585	5,644	5,471	5,509
217060_at	5,528	4,885	5,414	6,268	6,072	5,827
217061_s_at	8,058	8,153	8,305	7,677	7,758	7,743
217062_at	6,972	7,157	6,613	7,555	7,635	7,783
217063_x_at	5,575	5,394	5,765	6,082	5,743	5,830
217065_at	5,072	5,400	4,950	5,462	5,474	5,073
217066_s_at	7,438	7,193	7,421	7,883	7,616	7,787
217067_s_at	4,912	4,970	4,971	5,953	5,773	6,168
217068_at	5,658	5,658	5,819	5,917	5,884	5,875
217069_at	6,522	6,431	6,932	7,323	7,630	7,614
217070_at	7,936	7,625	8,026	8,592	8,395	8,265
217071_s_at	6,934	6,846	7,337	7,901	7,971	8,129
217072_at	6,427	6,180	6,400	7,020	7,044	7,196
217073_x_at	6,757	6,523	6,582	7,234	7,013	7,463
217074_at	7,013	6,860	7,406	7,040	6,971	7,293
217075_x_at	4,102	3,541	3,845	3,942	3,787	3,774
217076_s_at	4,770	4,645	4,272	4,826	4,977	4,900
217077_s_at	6,302	6,600	6,744	7,365	7,330	8,054
217078_s_at	5,314	5,197	5,140	5,992	6,137	6,312
217079_at	6,187	6,256	6,131	7,191	7,326	7,020
217080_s_at	7,522	7,440	7,358	6,605	5,941	6,513
217081_at	5,601	6,137	6,080	6,845	6,679	6,698
217082_at	4,980	4,940	5,029	5,732	5,812	5,746
217083_at	5,060	4,958	5,018	5,251	5,267	5,446
217084_at	4,242	4,160	3,988	4,701	4,295	4,167

217085_at	3,616	3,606	3,775	3,494	3,319	3,457
217086_at	5,298	5,524	4,755	5,749	5,396	5,706
217087_at	7,308	7,251	7,305	8,035	7,637	7,985
217088_s_at	7,159	6,583	6,671	7,484	7,271	7,462
217090_at	4,366	4,568	4,461	4,794	4,864	5,091
217091_at	4,000	3,934	4,081	4,029	3,733	3,928
217092_x_at	12,673	12,578	12,386	12,499	12,712	12,721
217093_at	4,626	4,346	4,365	4,898	4,484	4,587
217094_s_at	8,229	8,516	7,959	7,823	8,189	7,879
217095_x_at	7,191	7,117	7,107	7,755	7,619	7,791
217096_at	6,101	5,726	6,327	6,542	6,470	6,523
217097_s_at	5,668	6,008	5,990	6,239	5,933	5,960
217098_s_at	4,957	4,966	4,736	5,609	5,274	5,432
217099_s_at	9,256	8,693	9,374	9,008	8,215	8,805
217100_s_at	9,279	9,223	9,103	6,156	6,544	5,674
217101_at	6,223	5,560	5,749	7,004	7,455	7,145
217102_at	6,903	6,687	6,822	7,207	7,007	7,196
217103_at	4,605	5,165	4,592	5,121	4,808	4,800
217104_at	6,542	6,551	6,508	6,147	6,008	5,288
217105_at	3,990	3,814	3,940	3,945	3,672	3,733
217106_x_at	11,035	10,934	11,376	11,548	11,323	11,666
217107_at	8,997	8,633	8,824	9,226	8,706	8,769
217108_at	5,204	4,953	5,026	5,616	5,425	5,599
217109_at	8,069	8,000	8,206	7,169	7,258	7,067
217110_s_at	5,010	5,072	5,263	4,334	4,097	4,116
217111_at	6,468	6,299	5,996	7,228	6,833	6,944
217112_at	6,041	6,125	6,168	6,311	6,086	6,072
217113_at	6,430	6,751	6,513	6,939	6,751	6,990
217114_at	6,360	6,514	6,657	6,586	6,779	6,977
217115_at	7,128	7,101	7,418	8,017	7,736	7,876
217117_x_at	8,011	7,824	7,936	7,891	7,612	7,940
217118_s_at	9,365	9,486	9,324	10,431	9,607	9,971
217119_s_at	7,403	7,529	7,498	7,220	7,091	7,344
217120_s_at	6,657	6,321	6,744	6,739	6,502	6,567
217121_at	5,820	5,603	5,900	5,773	5,809	5,870
217122_s_at	11,287	11,134	11,420	9,502	10,681	10,229
217123_x_at	4,195	4,364	4,502	4,482	4,394	4,331
217124_at	5,805	5,905	6,145	6,994	6,018	6,496
217125_at	6,312	6,233	6,023	6,737	6,352	6,630
217126_at	4,456	4,052	4,341	4,452	4,408	4,395
217127_at	9,170	10,351	9,519	7,766	8,550	8,149
217128_s_at	5,720	5,621	5,904	6,394	6,568	6,303
217129_at	6,971	7,175	6,999	7,401	7,073	7,445
217130_at	7,223	7,075	7,303	6,695	6,887	6,724
217131_at	4,050	4,198	4,361	4,736	4,274	4,696
217132_at	3,611	3,919	3,999	3,546	3,430	3,427
217133_x_at	5,291	5,415	5,483	5,165	5,190	5,238
217134_at	6,185	5,995	6,065	6,749	6,577	6,974
217135_x_at	6,880	6,976	7,072	8,542	8,135	8,445
217136_at	5,007	5,013	5,049	5,582	4,922	5,537
217137_x_at	7,993	7,099	7,226	7,528	7,849	7,431
217138_x_at	6,123	6,181	6,122	7,570	7,395	7,424
217139_at	5,952	5,872	6,047	6,479	6,013	6,493
217140_s_at	9,301	9,182	9,148	9,734	8,999	9,819
217141_at	6,125	5,904	6,147	6,626	7,190	6,335
217142_at	8,415	8,865	8,461	8,871	9,021	9,112

217143_s_at	6,130	6,557	6,007	6,674	6,621	6,739
217144_at	9,776	9,590	9,721	10,463	9,584	10,194
217145_at	6,516	6,496	6,742	6,188	6,101	6,205
217146_at	6,669	5,879	6,244	7,288	7,102	7,272
217147_s_at	3,956	4,115	3,893	4,007	3,843	4,054
217148_x_at	7,748	7,765	7,774	7,719	7,424	7,441
217149_x_at	7,678	7,449	7,628	8,169	7,957	8,294
217150_s_at	7,052	6,913	7,050	8,776	7,775	8,154
217151_at	4,666	4,967	4,862	5,166	5,248	5,373
217152_at	7,598	6,926	6,533	6,429	7,055	6,758
217153_at	6,597	6,873	7,040	7,598	7,248	7,395
217154_s_at	5,948	5,809	6,026	6,338	6,257	6,343
217155_at	4,011	4,350	4,546	4,965	5,204	5,133
217156_at	7,370	6,761	7,121	7,193	7,183	7,465
217157_x_at	6,281	6,028	5,955	6,596	6,585	6,719
217158_at	6,889	6,718	6,971	7,286	7,258	7,482
217159_x_at	7,134	6,982	7,576	7,701	7,655	7,863
217160_at	7,479	7,163	7,277	7,695	8,112	7,940
217161_x_at	7,642	7,415	7,351	8,048	8,118	8,205
217162_at	7,624	7,388	7,711	7,722	7,052	7,539
217163_at	5,902	5,484	5,746	6,375	6,437	6,457
217164_at	8,532	7,689	7,625	4,398	4,909	4,293
217165_x_at	10,910	10,813	12,121	12,720	12,147	13,159
217166_at	5,788	5,588	5,236	6,326	5,458	5,932
217167_x_at	7,641	7,641	7,330	8,335	7,529	7,626
217168_s_at	10,769	11,522	11,555	11,629	12,053	12,223
217169_at	5,149	6,056	5,766	6,557	5,933	6,293
217170_at	6,239	6,090	5,701	6,304	6,204	6,393
217171_at	6,896	7,192	6,934	7,616	7,526	7,799
217172_at	6,077	6,037	5,815	6,275	5,927	6,328
217173_s_at	8,137	7,838	7,903	7,985	8,206	8,187
217174_s_at	6,258	6,188	6,327	7,222	6,554	6,782
217175_at	4,337	4,663	4,449	4,669	4,705	4,866
217176_s_at	4,736	4,826	4,745	4,623	4,627	4,452
217177_s_at	5,341	5,828	5,429	5,572	5,310	5,298
217178_at	6,222	6,105	5,787	6,752	6,553	6,581
217179_x_at	7,459	7,160	7,649	8,211	7,647	7,819
217180_at	6,490	6,494	6,609	7,420	7,268	7,467
217181_at	6,521	6,137	6,432	6,516	6,205	6,770
217182_at	7,090	7,435	7,336	8,570	8,856	8,724
217183_at	7,347	7,725	7,211	8,131	7,792	7,740
217184_s_at	6,915	6,753	6,960	7,033	7,088	7,005
217185_s_at	7,723	7,634	7,543	8,289	7,952	8,100
217186_at	6,263	6,472	5,913	7,281	6,869	7,387
217187_at	6,932	7,310	7,754	7,992	7,896	8,146
217188_s_at	7,566	7,609	7,572	7,392	6,577	7,295
217189_s_at	7,410	7,359	7,616	7,403	6,964	7,364
217190_x_at	6,717	6,983	6,799	8,698	8,611	8,505
217191_x_at	7,799	7,516	7,770	8,213	7,783	8,035
217192_s_at	6,505	6,497	6,540	7,668	7,266	7,514
217193_x_at	7,618	7,411	7,221	7,952	8,014	7,992
217194_at	3,585	3,783	3,594	3,807	3,965	3,941
217195_at	6,734	6,799	6,395	7,237	6,991	7,031
217196_s_at	9,861	9,785	9,664	8,235	9,590	7,547
217197_x_at	6,115	6,490	5,974	6,520	6,263	6,555
217198_x_at	6,788	6,827	7,222	7,781	7,636	7,629

217199_s_at	7,274	7,273	7,207	7,237	7,149	7,219
217200_x_at	8,994	8,949	9,190	9,219	8,705	9,349
217201_at	5,158	5,064	5,188	5,138	5,273	5,243
217202_s_at	9,632	9,125	9,066	7,737	7,573	7,890
217203_at	5,257	5,772	5,326	5,701	5,262	5,776
217204_at	4,419	4,766	4,967	4,405	4,339	4,466
217205_at	8,048	7,950	8,019	8,232	7,573	7,855
217206_at	4,623	4,736	4,469	5,281	5,183	5,534
217207_s_at	7,920	8,013	8,101	8,179	8,028	8,094
217208_s_at	6,669	7,152	7,073	6,531	6,156	6,457
217209_at	6,850	6,355	6,976	7,111	6,821	7,003
217210_at	6,447	6,526	6,353	7,583	7,331	7,712
217211_at	8,182	7,946	8,134	8,196	7,804	8,523
217212_s_at	6,897	6,876	6,993	7,997	7,967	8,179
217213_at	3,889	4,075	4,100	4,777	4,636	4,894
217214_s_at	4,973	5,079	5,232	5,263	5,036	4,892
217215_s_at	5,541	5,794	5,170	5,804	5,628	5,911
217216_x_at	8,226	8,426	7,901	7,368	7,561	7,196
217217_at	7,041	7,004	6,870	7,075	7,215	7,187
217218_at	5,290	5,325	5,136	5,583	5,422	5,176
217219_at	8,636	8,389	8,483	8,831	8,430	8,634
217220_at	5,279	5,575	5,497	5,921	5,647	5,567
217221_x_at	9,535	9,233	9,684	8,383	7,584	7,822
217222_at	6,238	6,254	5,816	6,579	6,421	6,852
217223_s_at	7,698	7,242	7,742	7,548	7,427	7,569
217224_at	6,816	6,746	6,820	7,230	7,289	7,103
217225_x_at	12,403	12,373	12,610	11,822	12,426	12,534
217226_s_at	5,894	6,587	6,363	8,782	8,925	8,970
217227_x_at	6,857	7,114	7,038	7,390	7,745	7,643
217228_s_at	5,539	5,159	5,657	5,887	5,841	5,874
217229_at	4,118	4,146	4,083	4,081	4,143	4,114
217230_at	5,556	5,733	6,089	6,175	6,097	6,059
217231_s_at	7,023	6,864	6,917	6,670	6,800	6,956
217232_x_at	7,210	7,091	7,311	7,334	7,242	7,516
217233_at	5,910	5,029	5,467	6,674	6,138	6,186
217234_s_at	9,292	9,245	9,520	8,423	7,568	8,668
217235_x_at	6,506	6,621	6,895	7,567	7,002	7,243
217236_x_at	5,302	5,210	5,718	6,042	5,831	6,236
217237_at	7,792	7,258	7,416	7,596	7,310	7,400
217238_s_at	4,264	4,333	4,166	4,677	4,721	4,944
217239_x_at	5,817	5,645	5,941	6,792	6,733	6,759
217240_at	7,008	6,870	6,996	7,715	7,461	7,587
217242_at	5,900	6,131	5,626	6,062	6,058	5,952
217244_at	5,637	5,537	5,370	5,655	5,710	5,748
217245_at	5,305	5,334	4,948	5,148	4,959	4,890
217246_s_at	4,005	4,195	4,169	4,031	4,282	4,214
217247_at	6,433	6,378	6,058	6,746	6,419	6,927
217248_s_at	5,287	5,233	5,170	5,816	5,662	5,634
217249_x_at	10,755	10,956	10,914	11,383	10,722	11,190
217250_s_at	4,685	4,393	4,665	5,024	4,539	4,824
217251_x_at	6,550	6,367	6,564	7,215	7,153	7,156
217252_at	6,383	6,267	5,861	6,773	6,396	6,483
217253_at	8,326	8,536	8,371	7,378	7,520	7,458
217254_s_at	6,864	6,687	6,549	6,588	6,544	6,602
217255_at	6,996	6,657	6,842	7,145	7,159	7,148
217256_x_at	11,119	11,331	11,143	10,914	10,358	10,909

217257_at	3,896	4,000	4,271	4,330	4,351	4,225
217258_x_at	7,095	6,751	6,968	7,392	7,430	7,701
217259_at	5,412	5,560	5,693	5,060	5,450	4,923
217260_x_at	5,825	5,676	5,863	6,768	6,489	6,632
217261_at	6,771	6,021	6,931	6,882	6,823	6,759
217262_s_at	5,353	5,550	5,458	6,377	6,029	6,030
217263_x_at	6,070	6,109	6,134	7,074	7,106	7,057
217264_s_at	7,369	7,090	7,583	8,664	8,569	8,720
217265_at	5,706	5,603	5,560	5,426	5,077	5,509
217266_at	10,010	10,099	10,077	9,985	9,534	10,059
217267_s_at	3,994	3,935	4,090	4,154	4,146	4,116
217268_at	3,710	4,078	3,577	4,045	4,299	4,323
217269_s_at	7,279	7,145	6,982	7,858	7,478	7,698
217270_s_at	6,414	6,850	6,685	7,158	7,349	7,194
217271_at	5,800	5,642	5,707	6,274	6,085	6,112
217272_s_at	4,494	4,703	4,804	4,813	4,747	4,896
217273_at	6,598	6,926	6,926	7,549	7,388	7,663
217274_x_at	7,268	7,172	7,110	7,854	7,940	7,904
217275_at	7,759	7,901	7,969	8,121	8,173	8,010
217276_x_at	7,032	7,074	6,597	7,940	7,574	8,028
217277_at	6,512	6,381	6,494	7,682	7,422	7,730
217278_x_at	5,605	5,320	5,752	5,844	5,750	6,045
217279_x_at	7,982	8,038	8,094	8,014	7,963	8,272
217280_x_at	5,549	6,154	6,156	5,641	5,735	5,706
217281_x_at	6,952	6,857	6,755	7,712	7,676	7,694
217282_at	5,162	4,850	4,753	4,523	4,730	4,483
217283_at	6,168	5,979	5,958	6,474	6,736	6,985
217284_x_at	6,867	6,948	6,874	7,666	7,487	8,014
217285_at	7,409	7,300	7,480	7,923	7,854	7,977
217286_s_at	9,708	10,253	10,389	10,592	10,393	10,622
217287_s_at	6,402	6,616	6,507	6,913	6,898	6,790
217288_at	6,073	5,396	5,621	5,611	5,473	5,370
217289_s_at	8,231	8,295	8,147	7,728	7,397	7,625
217290_at	6,231	6,129	5,908	6,760	6,314	6,717
217291_at	8,115	8,028	8,132	8,580	8,446	8,919
217292_at	5,072	4,991	4,895	4,914	5,085	4,891
217293_at	6,649	6,814	6,522	6,528	6,771	6,756
217294_s_at	9,382	8,831	9,020	9,143	8,013	9,236
217295_at	6,989	6,998	7,144	7,863	7,506	8,046
217296_at	5,968	5,762	5,839	6,476	6,188	6,205
217297_s_at	6,963	6,893	7,013	7,339	7,321	7,333
217298_at	5,658	5,518	5,788	5,962	6,053	6,118
217299_s_at	6,040	6,158	5,807	7,692	8,401	7,217
217300_at	5,411	5,836	5,714	5,374	5,496	5,457
217301_x_at	11,021	10,842	10,747	8,368	8,471	8,417
217302_at	4,989	5,142	5,211	5,671	5,511	5,759
217303_s_at	6,602	6,646	6,545	6,589	6,554	6,661
217304_at	5,589	5,564	5,420	6,172	6,305	6,249
217305_s_at	6,291	6,409	6,510	7,193	7,203	7,220
217306_at	4,544	4,727	4,624	5,349	5,563	5,701
217307_at	6,902	6,705	7,008	7,844	7,308	7,792
217308_at	6,123	6,089	5,769	6,847	6,729	7,061
217309_s_at	8,176	8,122	7,613	8,032	7,559	7,943
217310_s_at	6,129	6,577	6,248	6,354	6,169	5,998
217311_at	6,495	6,116	6,210	6,576	6,534	6,569
217312_s_at	7,251	7,422	7,453	7,733	7,910	8,362

217313_at	9,376	9,604	9,609	10,004	9,459	10,177
217314_at	6,649	6,821	6,418	7,534	7,385	7,472
217315_s_at	6,217	6,530	6,630	6,901	6,746	6,745
217316_at	4,585	4,658	4,941	4,901	4,568	4,772
217317_s_at	10,148	10,023	9,756	8,371	9,900	8,816
217318_x_at	6,010	5,506	6,151	7,140	6,735	7,269
217319_x_at	5,854	5,918	5,720	6,213	6,077	6,358
217320_at	4,897	4,980	4,891	5,218	5,047	5,521
217321_x_at	5,009	4,998	4,618	5,187	5,161	5,179
217322_x_at	9,398	9,623	9,566	9,482	9,730	9,262
217323_at	5,809	5,914	5,801	6,676	6,541	6,744
217324_at	7,611	7,878	7,624	7,925	7,659	7,759
217325_at	6,597	6,342	6,109	7,683	7,488	7,775
217326_x_at	7,058	7,257	6,936	7,615	7,549	7,631
217327_at	5,750	5,878	5,644	6,500	6,222	6,361
217328_at	6,295	6,242	6,229	7,145	7,041	7,429
217329_x_at	9,495	9,680	9,536	9,867	9,405	9,992
217330_at	6,324	6,266	6,485	6,717	6,225	6,404
217331_at	6,298	6,237	6,504	6,229	6,445	6,313
217332_at	5,973	5,885	6,114	6,266	6,130	6,366
217333_at	5,930	5,255	5,275	6,511	6,452	6,576
217334_at	5,227	5,038	5,169	5,593	5,379	5,489
217335_at	5,109	5,199	5,844	6,108	6,167	5,981
217336_at	7,238	7,404	7,203	8,134	7,743	8,144
217337_at	6,136	6,070	6,034	6,968	6,771	7,034
217338_at	5,768	5,718	5,787	6,309	6,062	6,161
217339_x_at	6,615	6,503	6,788	7,085	7,305	6,972
217340_at	5,109	5,327	5,691	5,725	5,524	5,715
217341_at	7,182	7,157	7,002	7,764	7,578	7,839
217342_x_at	6,076	5,941	6,042	6,028	6,253	6,109
217343_at	4,109	4,248	4,084	4,338	4,525	4,206
217344_at	6,754	6,272	5,963	7,024	6,811	6,987
217345_at	4,326	4,295	4,047	4,480	4,286	4,365
217346_at	8,230	8,287	8,269	8,838	8,239	8,793
217347_at	8,446	8,195	8,269	8,608	7,973	8,694
217348_x_at	7,128	6,999	7,032	7,471	7,176	7,252
217349_s_at	6,911	6,797	6,567	7,372	7,500	7,261
217350_at	5,255	5,243	5,715	6,208	5,970	6,228
217351_at	4,866	5,012	5,054	5,296	5,301	5,535
217352_at	7,274	7,659	7,086	7,713	7,638	7,937
217353_at	8,912	8,678	8,857	8,858	8,801	8,207
217354_s_at	6,785	6,689	6,713	7,712	7,246	7,812
217355_at	6,933	6,931	7,111	7,559	7,555	7,872
217356_s_at	10,662	10,920	10,647	10,325	9,683	10,455
217357_at	7,512	7,055	7,051	7,850	7,301	7,617
217358_at	8,052	7,665	7,844	8,431	8,574	8,419
217359_s_at	5,189	4,983	4,894	5,661	5,658	5,505
217360_x_at	5,405	5,461	5,533	5,993	5,991	6,005
217361_at	6,017	5,803	5,981	6,498	6,212	6,188
217362_x_at	6,568	6,949	6,634	7,120	7,060	7,305
217363_x_at	8,697	8,858	8,491	9,236	8,993	9,406
217364_x_at	6,526	6,440	6,375	6,557	6,415	6,485
217365_at	7,226	7,214	7,419	8,621	8,097	8,406
217366_at	6,147	6,309	6,111	6,551	6,483	6,588
217367_s_at	6,729	6,863	6,693	7,315	7,947	7,705
217368_at	6,606	5,799	6,115	7,095	6,378	6,718

217369_at	7,456	8,154	8,124	8,839	8,605	9,156
217370_x_at	8,588	8,620	8,686	8,416	8,352	8,540
217371_s_at	4,638	4,697	4,564	5,488	5,483	5,569
217372_at	5,297	5,174	4,802	5,129	4,997	5,261
217373_x_at	5,440	5,311	5,296	5,885	6,053	6,428
217374_x_at	5,985	5,890	6,175	7,014	6,347	6,682
217375_at	4,876	4,596	4,748	4,863	4,983	4,692
217376_at	6,142	6,435	6,589	6,869	6,801	7,211
217377_x_at	6,691	6,514	6,619	7,049	6,739	7,025
217378_x_at	6,302	6,888	6,879	7,836	7,823	7,565
217379_at	10,718	10,630	10,741	11,350	10,602	11,403
217380_s_at	4,543	4,845	4,641	4,736	5,174	4,679
217381_s_at	6,753	6,909	6,820	6,539	6,606	6,444
217382_at	6,233	6,216	6,303	7,754	7,441	7,882
217383_at	5,749	6,141	6,363	6,266	5,996	6,092
217384_x_at	5,468	5,140	4,923	5,426	5,326	5,537
217385_at	5,781	5,233	5,515	6,289	6,037	6,309
217386_at	5,149	5,177	5,458	5,970	5,750	6,335
217387_at	4,495	3,960	3,925	4,610	4,663	4,938
217388_s_at	4,749	5,165	4,851	11,219	11,152	7,571
217389_s_at	6,916	6,701	6,951	7,169	7,273	7,143
217390_x_at	7,590	7,649	7,639	8,321	8,250	8,546
217391_x_at	7,030	6,817	6,888	6,880	7,046	7,006
217392_at	4,085	4,122	4,136	4,466	4,136	4,578
217393_x_at	10,924	10,689	10,808	11,320	10,874	11,200
217394_at	4,020	3,689	3,974	4,168	4,011	4,014
217395_at	7,714	7,634	8,042	9,047	8,869	8,991
217396_at	6,596	6,349	6,678	8,102	7,977	7,876
217397_at	5,690	5,933	5,923	6,339	5,906	6,134
217398_x_at	14,663	14,589	14,661	13,899	14,067	14,294
217399_s_at	6,322	6,698	5,932	6,459	6,091	6,302
217400_at	3,980	4,412	4,173	4,414	4,193	4,291
217401_at	5,461	5,510	5,594	6,292	5,911	6,323
217402_at	4,916	4,456	4,579	5,785	5,956	6,143
217403_s_at	8,523	8,588	7,711	5,826	5,323	5,863
217404_s_at	5,880	5,824	5,530	6,075	6,032	6,201
217405_x_at	6,095	5,663	6,079	6,515	6,002	6,378
217406_at	6,555	6,164	6,508	6,914	6,422	6,960
217407_x_at	8,701	8,576	8,669	8,726	8,505	8,753
217408_at	11,058	11,273	11,503	11,942	11,150	11,398
217409_at	6,136	5,672	6,138	6,327	6,327	6,246
217410_at	6,662	6,654	6,979	7,435	7,308	7,406
217411_s_at	6,120	6,140	5,900	7,631	7,411	7,808
217412_at	4,854	4,862	4,874	5,777	5,423	5,555
217413_s_at	7,261	7,924	7,896	8,419	8,438	8,655
217414_x_at	7,770	8,107	7,908	8,159	7,921	8,329
217415_at	5,360	5,617	5,770	6,341	6,138	6,455
217416_x_at	5,070	5,515	4,853	5,584	5,326	5,186
217417_at	4,439	4,477	4,311	4,372	4,389	4,379
217418_x_at	6,138	5,781	6,141	6,070	5,956	5,980
217419_x_at	8,353	8,488	8,359	8,289	8,046	8,109
217420_s_at	5,452	5,603	5,730	6,218	6,289	6,290
217421_at	7,190	7,216	6,913	7,098	7,112	6,886
217422_s_at	5,217	5,515	5,233	5,579	5,322	5,402
217423_at	5,638	5,570	6,053	5,826	5,870	5,929
217424_at	5,350	5,112	5,475	5,371	5,275	5,327

217425_at	5,424	5,432	5,605	5,627	5,556	5,912
217426_at	5,627	5,645	5,638	5,659	5,430	5,826
217427_s_at	9,128	9,014	9,367	8,670	8,459	8,658
217428_s_at	4,060	4,014	3,821	4,580	4,070	4,580
217429_at	5,640	5,698	5,324	5,736	5,929	5,869
217430_x_at	8,711	8,632	8,951	9,967	9,642	10,019
217431_x_at	4,418	4,706	4,704	5,380	5,058	5,471
217432_s_at	6,776	6,736	6,991	7,763	7,962	7,939
217433_at	5,440	5,499	5,321	6,084	5,691	5,941
217434_at	4,540	4,639	4,816	4,869	4,842	5,062
217435_x_at	6,049	6,212	6,327	7,659	7,579	7,796
217436_x_at	8,765	8,733	8,759	9,285	8,846	9,640
217437_s_at	8,368	8,409	7,830	8,588	9,371	8,491
217438_at	5,742	5,071	4,931	6,234	6,275	6,281
217439_at	6,485	6,471	6,567	7,498	7,173	7,304
217440_at	3,562	3,733	3,846	3,770	3,695	3,849
217441_at	5,215	5,685	5,496	4,795	4,670	4,859
217442_at	6,171	5,911	6,078	6,541	6,348	6,454
217443_at	3,821	3,765	4,152	4,398	3,968	4,137
217444_at	6,615	6,849	6,958	7,557	7,563	7,782
217445_s_at	7,421	7,421	7,075	6,398	6,223	6,287
217446_x_at	7,402	7,278	7,612	7,611	8,129	7,350
217447_at	6,346	6,164	6,129	6,852	6,504	6,507
217448_s_at	6,876	6,695	6,367	7,518	7,416	7,499
217449_at	6,682	6,533	6,778	7,331	7,370	7,420
217450_at	6,654	6,400	6,478	6,412	6,441	6,505
217451_at	4,794	4,712	4,802	5,544	4,871	5,078
217452_s_at	3,624	3,744	3,883	4,099	4,182	4,603
217453_at	7,380	7,008	7,114	7,444	7,183	7,368
217454_at	5,486	5,206	5,487	6,841	6,396	6,702
217455_s_at	6,457	6,069	6,654	6,055	6,083	5,974
217456_x_at	8,948	9,196	9,235	10,122	9,741	10,241
217457_s_at	9,098	8,839	8,781	9,433	10,220	9,965
217458_at	7,042	6,892	7,008	7,212	7,071	6,750
217459_at	6,084	6,249	6,285	6,523	6,460	6,471
217460_at	4,188	4,453	4,067	4,124	4,044	4,132
217461_x_at	8,080	8,253	7,866	8,784	8,545	8,802
217462_at	6,159	6,440	6,066	7,834	8,024	8,032
217463_s_at	5,185	5,344	5,407	6,568	6,837	6,935
217464_at	7,245	7,228	7,189	7,390	7,206	7,381
217465_at	5,326	5,702	5,684	4,993	4,901	4,881
217466_x_at	12,520	12,768	12,334	11,918	11,807	12,269
217467_at	5,785	6,041	6,051	6,533	6,057	6,421
217468_at	7,859	8,166	8,074	8,592	8,649	9,019
217469_at	6,619	6,403	6,495	7,406	7,172	7,268
217470_at	4,619	4,478	4,508	4,664	4,383	4,735
217471_at	6,518	6,675	6,587	6,865	7,379	7,029
217472_at	6,469	7,394	6,771	7,091	7,148	7,234
217473_x_at	7,616	7,526	7,304	7,898	8,013	8,087
217474_at	5,768	6,301	5,910	5,875	5,580	6,093
217475_s_at	4,938	5,094	5,565	5,990	5,696	5,755
217476_at	6,218	6,593	6,492	6,799	6,785	6,938
217477_at	6,093	5,886	6,210	6,337	6,291	6,296
217478_s_at	8,640	8,842	8,853	7,018	6,161	7,313
217479_at	3,919	3,766	4,002	4,728	4,416	4,559
217480_x_at	7,809	7,633	8,072	8,157	7,839	8,118

217481_x_at	6,327	6,501	6,470	5,791	5,729	5,812
217482_at	9,080	7,486	8,442	6,674	7,145	6,711
217483_at	5,525	5,714	5,561	5,976	5,940	6,188
217484_at	5,982	6,644	6,443	6,709	6,511	6,835
217485_x_at	9,027	8,804	9,099	8,970	8,616	9,046
217486_s_at	6,281	5,974	5,755	5,878	5,943	6,209
217487_x_at	6,690	6,450	6,377	7,184	7,307	7,383
217488_x_at	5,491	5,447	5,518	5,433	5,625	5,518
217489_s_at	5,993	5,835	5,958	6,992	6,312	6,516
217490_at	6,898	6,671	6,906	7,300	7,459	7,374
217491_x_at	12,505	12,684	12,496	12,119	11,952	12,253
217492_s_at	9,571	10,223	9,436	9,926	10,489	10,259
217493_x_at	7,999	7,773	7,912	8,479	8,689	8,762
217494_s_at	4,353	4,177	4,496	4,699	4,652	4,640
217495_x_at	7,117	6,963	6,879	7,862	7,848	8,068
217496_s_at	9,251	9,414	9,298	8,802	9,041	8,433
217497_at	6,260	6,221	6,390	7,159	7,326	7,518
217498_at	7,030	6,697	6,967	6,765	6,817	6,998
217499_x_at	8,859	9,128	9,193	9,503	8,721	9,916
217500_at	6,609	6,409	6,762	6,884	6,769	6,897
217501_at	8,688	8,748	8,991	7,979	8,320	7,931
217502_at	6,671	7,032	6,825	7,204	7,252	7,339
217503_at	5,321	6,049	5,627	5,863	5,822	5,941
217504_at	5,238	5,145	4,844	5,382	5,446	5,393
217505_at	3,856	3,862	4,201	3,908	4,115	4,008
217506_at	5,713	6,305	6,060	5,172	5,326	5,316
217507_at	6,201	6,421	6,186	6,993	6,953	7,047
217508_s_at	6,846	6,643	7,052	7,795	6,868	7,118
217509_x_at	9,352	8,936	9,162	9,814	9,473	9,422
217510_at	6,315	6,972	6,425	6,735	6,818	6,961
217511_at	6,730	6,642	6,964	7,658	7,306	7,261
217512_at	5,329	5,522	5,476	6,154	5,673	5,974
217513_at	5,155	6,138	6,219	7,665	8,014	8,205
217514_at	6,676	6,738	6,928	7,107	7,003	7,076
217515_s_at	7,452	7,257	6,974	7,888	7,802	7,848
217516_x_at	8,381	8,354	8,509	8,317	8,279	8,320
217517_x_at	6,805	6,380	6,678	7,007	7,261	7,268
217518_at	6,061	6,298	6,248	6,450	8,040	6,714
217519_at	5,458	5,712	5,326	5,121	5,124	5,111
217520_x_at	7,670	7,958	8,039	8,346	6,534	7,986
217521_at	7,031	7,129	7,225	7,136	6,550	6,969
217522_at	5,016	4,677	5,301	5,232	5,133	5,138
217523_at	5,020	5,289	5,299	5,507	7,763	5,728
217524_x_at	3,990	3,883	3,998	3,851	3,780	3,905
217525_at	4,286	4,178	4,301	4,264	4,208	4,178
217526_at	10,171	9,821	10,005	7,463	8,343	7,825
217527_s_at	10,589	10,318	10,275	8,989	9,837	9,260
217528_at	4,469	4,293	4,520	4,491	4,592	4,598
217529_at	8,050	7,900	8,048	7,358	7,403	7,918
217530_at	5,780	6,426	6,122	6,459	6,100	6,432
217531_at	5,403	5,040	5,455	6,146	5,971	6,255
217532_x_at	5,318	5,408	5,117	5,500	5,614	5,556
217533_x_at	5,132	5,205	5,424	5,826	5,741	5,712
217534_at	4,526	4,540	4,503	4,630	4,944	4,469
217535_at	4,848	5,015	4,970	5,527	5,467	5,324
217536_x_at	5,993	5,071	5,326	4,714	4,602	4,698

217537_x_at	3,794	4,021	3,858	4,010	4,284	4,206
217538_at	6,840	6,796	7,064	6,650	7,026	6,746
217539_at	5,532	5,804	5,627	6,096	6,060	6,134
217540_at	8,042	8,077	7,903	7,824	8,276	7,249
217541_x_at	6,099	6,343	6,178	5,997	6,275	6,349
217542_at	8,140	7,935	8,241	7,793	7,593	7,557
217543_s_at	9,226	9,040	8,893	8,744	9,063	9,065
217544_at	5,257	5,464	5,648	5,102	5,326	5,126
217545_at	7,150	7,361	7,350	7,890	7,813	7,833
217546_at	6,845	6,260	9,852	7,561	7,266	7,557
217547_x_at	6,221	7,030	5,844	3,886	4,091	3,871
217548_at	7,704	7,662	7,477	7,709	7,792	7,385
217549_at	7,538	7,582	7,443	8,005	8,144	8,051
217550_at	8,189	8,557	7,994	7,001	7,464	6,796
217551_at	6,062	5,842	6,072	6,093	5,842	5,884
217552_x_at	5,401	5,699	5,663	3,593	3,730	3,765
217553_at	9,149	9,657	9,353	11,899	10,957	11,376
217554_at	7,168	7,538	7,139	6,398	6,033	6,219
217555_at	8,215	7,971	7,450	6,851	7,021	6,620
217556_at	5,906	6,100	5,940	6,294	5,789	5,980
217557_s_at	7,795	7,693	8,411	9,460	9,500	9,583
217558_at	4,212	4,321	4,326	4,418	4,323	4,295
217559_at	7,584	7,755	7,756	8,724	7,873	8,630
217560_at	7,977	8,043	7,851	7,677	7,789	7,815
217561_at	5,139	5,152	5,191	5,193	5,106	5,131
217562_at	3,800	3,850	3,923	3,888	4,074	4,146
217563_at	6,611	6,313	6,797	6,432	6,407	6,402
217564_s_at	6,851	7,028	6,790	6,169	6,121	5,500
217565_at	5,364	5,850	5,397	5,382	5,262	5,356
217566_s_at	4,955	5,614	5,437	6,250	6,464	6,547
217567_at	5,897	5,689	6,098	6,487	6,269	6,625
217568_at	4,653	4,567	4,888	4,345	4,428	4,701
217569_x_at	6,644	6,340	6,463	6,581	6,234	5,902
217570_x_at	5,753	6,187	6,150	6,141	5,955	6,118
217571_at	4,443	4,122	4,384	4,495	4,612	4,517
217572_at	6,057	5,438	5,773	6,438	5,921	6,191
217573_at	7,372	7,558	7,586	7,931	7,839	8,425
217574_at	5,251	5,764	5,509	5,669	5,397	5,552
217575_s_at	3,969	4,078	4,185	4,099	4,231	4,193
217576_x_at	7,001	7,029	6,997	6,930	6,556	6,649
217577_at	3,797	3,970	4,283	4,394	4,100	4,436
217578_at	4,336	4,203	4,040	3,997	4,148	4,022
217579_x_at	8,244	7,790	7,982	7,448	7,757	7,752
217580_x_at	6,984	7,356	7,229	7,387	7,643	7,991
217581_at	6,567	6,045	5,997	7,051	7,014	7,296
217582_at	5,584	5,523	6,083	6,530	6,525	6,585
217583_at	4,504	4,057	4,578	4,200	4,189	4,270
217584_at	5,323	5,287	4,920	5,331	5,113	5,257
217585_at	6,543	6,614	6,232	6,173	5,921	6,005
217586_x_at	8,739	8,143	8,238	8,052	8,259	7,811
217587_at	5,071	4,948	5,276	5,084	5,163	5,189
217588_at	6,948	7,081	6,864	6,967	7,225	7,025
217589_at	4,333	4,622	4,337	5,050	5,212	5,032
217590_s_at	6,507	6,701	6,247	6,737	6,827	6,616
217591_at	7,918	7,680	8,010	5,829	6,565	6,348
217592_at	6,307	5,987	6,756	6,588	6,258	6,485

217593_at	8,101	7,717	8,064	7,273	7,265	7,632
217594_at	4,336	3,797	3,937	3,746	3,890	3,765
217595_at	5,209	5,546	5,475	4,817	5,072	4,848
217596_at	6,673	6,620	6,520	7,012	7,222	6,990
217597_x_at	7,288	7,397	7,292	8,940	8,957	9,026
217598_at	6,704	6,759	7,432	6,983	6,031	6,660
217599_s_at	6,553	6,355	6,095	8,894	8,244	8,806
217600_at	6,347	5,788	6,334	6,404	6,390	6,843
217601_at	6,315	5,678	6,021	6,313	6,098	6,128
217602_at	7,760	7,589	7,184	5,250	5,222	5,526
217603_at	6,771	6,308	6,815	6,336	6,285	6,629
217604_at	7,859	8,048	8,181	7,386	7,616	7,342
217605_at	6,577	6,244	6,507	6,812	6,554	6,737
217606_at	6,791	6,905	7,034	7,765	7,660	7,642
217607_x_at	5,580	5,834	5,740	5,864	5,709	5,535
217608_at	6,913	7,022	6,494	6,987	7,623	7,024
217609_at	7,325	7,550	7,192	6,842	6,382	6,645
217610_at	8,975	8,034	8,093	7,242	7,824	7,112
217611_at	7,666	7,211	7,444	7,024	7,144	7,014
217612_at	7,846	7,547	7,854	7,901	7,151	7,654
217613_at	4,175	4,483	4,185	4,470	4,175	4,250
217614_at	6,768	6,622	6,725	6,412	6,452	6,593
217615_at	6,761	6,898	7,131	7,338	7,346	7,596
217616_at	6,076	5,571	5,431	5,982	5,784	5,802
217617_at	8,361	7,741	8,365	6,080	5,904	5,890
217618_x_at	7,580	7,545	7,613	7,282	7,017	7,392
217619_x_at	8,272	8,101	8,288	7,889	8,255	7,857
217620_s_at	5,627	5,454	5,245	4,876	4,813	5,075
217621_at	4,158	4,591	4,270	4,082	4,176	4,013
217622_at	8,107	8,075	7,876	7,897	7,896	8,268
217623_at	4,807	4,678	4,641	5,078	5,175	5,371
217624_at	5,388	5,335	5,517	5,753	5,535	5,726
217625_x_at	6,383	6,311	6,222	6,009	5,755	6,040
217626_at	6,307	6,686	6,229	8,023	8,450	8,250
217627_at	8,558	8,153	8,584	5,831	6,714	5,991
217628_at	6,449	6,719	6,501	7,202	7,063	7,141
217629_at	6,368	6,547	6,500	6,686	6,500	6,702
217630_at	7,699	8,096	7,748	6,808	6,584	6,673
217631_at	7,116	6,990	6,944	7,001	7,306	7,348
217632_at	4,974	4,370	5,166	4,111	4,011	4,097
217633_at	6,172	6,197	6,900	7,158	7,425	6,585
217634_at	6,914	6,960	7,016	7,708	7,476	7,643
217635_s_at	7,368	7,614	7,501	7,987	7,766	7,798
217636_at	7,047	7,093	6,908	6,907	6,852	7,209
217637_at	7,091	6,626	6,902	7,464	7,568	7,558
217638_at	7,114	6,486	6,955	7,365	7,294	7,790
217639_at	5,764	5,579	5,537	5,632	5,808	6,204
217640_x_at	5,756	5,829	5,165	4,688	4,700	4,638
217641_at	6,072	6,396	6,555	6,578	7,160	6,805
217642_at	7,216	7,041	7,205	7,400	7,205	7,359
217643_x_at	7,347	6,751	7,197	6,625	7,135	6,579
217644_s_at	5,663	6,025	5,990	5,728	5,578	5,892
217645_at	8,511	8,600	8,739	8,370	8,201	7,925
217646_at	7,117	6,674	6,562	6,240	6,049	6,332
217647_at	7,315	7,376	7,585	7,663	7,252	7,411
217648_at	5,208	5,503	5,055	5,276	5,288	5,162

217649_at	6,287	7,224	6,818	5,869	7,780	6,241
217650_x_at	8,984	8,804	9,172	8,610	8,468	8,839
217651_at	6,361	5,912	6,851	4,949	4,813	4,759
217652_at	6,181	5,854	6,462	6,679	6,943	6,955
217653_x_at	7,282	6,750	6,872	6,183	7,787	6,507
217654_at	5,036	4,907	5,230	5,532	5,458	5,441
217655_at	6,205	5,757	5,818	5,988	5,657	5,779
217656_at	8,681	7,610	8,335	6,868	6,909	7,088
217657_at	5,951	5,830	5,996	7,158	7,051	7,120
217658_at	6,551	6,434	6,385	7,628	7,553	7,550
217659_at	6,253	5,001	5,722	4,859	4,749	4,508
217660_at	6,631	6,221	6,244	7,458	7,701	7,850
217661_x_at	5,879	5,873	5,913	6,330	6,211	6,247
217662_x_at	7,565	7,238	7,112	6,763	7,734	6,795
217663_at	5,774	5,667	6,186	5,514	5,649	5,651
217664_at	5,530	5,215	5,351	5,487	5,578	5,682
217665_at	7,773	7,878	7,672	6,142	5,814	5,834
217666_at	5,938	5,860	5,949	6,140	5,897	6,268
217667_at	6,970	6,109	6,461	6,051	6,207	6,033
217668_at	5,343	5,470	5,602	5,859	5,673	5,685
217669_s_at	5,914	6,045	6,115	6,580	6,517	6,686
217670_at	5,425	5,202	5,467	5,512	5,403	5,205
217671_at	7,656	6,719	7,260	5,395	5,880	5,519
217672_x_at	7,441	7,105	7,418	7,796	7,577	8,084
217673_x_at	9,800	9,783	10,086	9,543	9,535	9,969
217674_at	5,002	4,940	5,280	5,599	5,430	5,406
217675_at	5,667	5,060	5,100	5,705	5,364	5,885
217676_at	6,660	6,311	6,580	6,867	6,133	6,962
217677_at	6,232	6,499	6,252	5,409	5,628	5,965
217678_at	7,835	8,246	7,497	8,220	10,098	7,876
217679_x_at	9,526	9,166	9,268	9,029	9,977	8,928
217680_x_at	6,660	6,827	6,841	7,006	6,783	6,867
217681_at	7,541	6,638	6,820	7,584	7,381	7,727
217682_at	8,312	8,480	8,529	7,504	7,542	7,660
217683_at	6,503	5,978	6,582	7,339	6,244	6,427
217684_at	6,036	5,982	5,971	6,345	6,103	6,565
217685_at	6,547	6,265	6,254	8,374	8,585	8,694
217686_at	6,454	6,352	6,726	6,903	6,628	6,666
217687_at	6,091	6,664	6,555	6,859	6,663	6,759
217688_at	6,179	5,746	6,071	7,833	7,802	7,764
217689_at	6,466	6,100	6,300	7,027	6,861	7,083
217690_at	7,625	7,087	7,505	6,778	7,395	6,583
217691_x_at	8,606	8,599	9,038	10,048	9,770	10,198
217692_at	6,347	6,007	5,664	5,485	5,408	5,299
217693_x_at	8,102	7,951	7,836	7,718	6,968	7,092
217694_at	3,460	3,417	3,687	3,883	3,676	3,689
217695_x_at	6,614	6,879	7,210	6,987	6,961	7,122
217696_at	8,040	7,713	8,024	8,286	8,174	7,838
217697_at	4,469	4,694	4,694	4,986	4,896	4,960
217698_at	3,461	3,536	3,665	3,673	3,474	3,619
217699_at	4,870	5,006	5,330	5,742	5,698	5,765
217700_at	7,292	7,041	7,359	7,192	7,320	7,278
217701_x_at	4,857	4,207	4,769	4,099	4,907	4,055
217702_at	5,710	5,839	5,709	6,755	6,671	6,729
217703_x_at	9,069	8,974	9,231	9,034	9,690	9,394
217704_x_at	6,095	5,682	5,801	5,544	5,775	5,620

217705_at	4,110	4,387	3,867	4,212	4,329	4,276
217706_at	5,509	5,349	5,744	7,142	7,248	7,141
217707_x_at	8,978	8,640	9,183	9,570	10,219	9,370
217708_x_at	7,302	7,060	7,293	7,689	7,608	7,989
217709_at	3,914	3,595	3,827	4,092	4,155	4,122
217710_x_at	7,385	7,318	7,412	8,043	7,902	7,979
217711_at	7,445	7,204	7,603	8,696	8,299	8,428
217712_at	7,168	6,057	6,890	4,125	3,768	4,164
217713_x_at	8,143	7,798	7,897	7,077	7,939	7,269
217714_x_at	7,907	7,668	8,052	8,777	9,074	8,926
217715_x_at	9,693	9,042	9,504	8,876	9,460	9,138
217716_s_at	10,991	10,660	11,298	11,695	11,997	12,250
217717_s_at	10,679	10,801	10,953	10,674	10,953	10,898
217718_s_at	13,047	13,035	13,179	12,500	13,160	12,863
217719_at	13,526	13,685	13,581	12,777	13,012	13,060
217720_at	12,661	12,828	12,950	12,091	11,834	12,392
217721_at	8,520	8,820	8,375	7,307	7,703	7,039
217722_s_at	12,544	12,560	12,767	11,402	11,181	11,634
217723_x_at	7,496	7,631	7,952	7,835	7,558	7,926
217724_at	14,020	14,006	13,911	12,113	12,997	12,072
217725_x_at	12,626	12,174	12,360	11,061	11,216	10,851
217726_at	11,205	11,371	11,504	11,319	11,173	11,539
217727_x_at	12,163	12,284	12,045	11,740	12,346	11,559
217728_at	6,928	6,149	6,578	11,352	11,127	11,600
217729_s_at	8,481	8,430	8,287	8,296	8,505	8,938
217730_at	8,335	8,928	8,576	11,044	10,951	11,452
217731_s_at	12,535	12,271	12,556	11,171	11,992	12,156
217732_s_at	12,064	11,996	12,496	11,750	11,864	12,214
217733_s_at	12,850	12,613	12,628	13,561	13,755	14,028
217734_s_at	10,536	10,179	10,662	9,105	9,144	9,564
217735_s_at	7,823	7,317	7,698	6,876	7,172	6,665
217736_s_at	11,373	11,248	11,424	10,430	10,496	10,476
217737_x_at	9,721	9,776	10,056	9,675	9,751	10,065
217738_at	9,524	10,040	9,146	8,582	8,939	7,218
217739_s_at	10,874	11,115	10,606	10,864	11,273	9,814
217740_x_at	14,398	14,646	14,595	13,868	14,084	14,159
217741_s_at	10,167	11,022	10,762	10,456	11,603	11,001
217742_s_at	10,014	10,125	9,936	8,258	8,978	8,008
217743_s_at	9,375	9,614	8,883	10,299	11,074	10,535
217744_s_at	8,681	8,325	8,758	7,974	5,965	7,816
217745_s_at	12,840	12,526	12,196	11,426	12,035	11,141
217746_s_at	11,008	11,171	11,068	11,177	11,882	11,402
217747_s_at	12,805	12,757	12,687	12,468	11,822	12,648
217748_at	10,357	10,244	10,409	10,062	9,471	10,069
217749_at	8,769	8,551	8,332	8,451	8,427	8,906
217750_s_at	10,125	9,843	9,983	10,285	10,412	10,679
217751_at	8,828	8,825	8,543	9,870	9,624	10,387
217752_s_at	11,502	11,072	11,879	11,539	11,331	11,429
217753_s_at	12,987	13,009	13,155	12,078	12,037	12,954
217754_at	9,109	8,727	9,371	9,343	8,924	9,260
217755_at	10,088	9,883	10,266	10,808	10,152	10,927
217756_x_at	11,353	10,986	11,198	11,701	11,513	12,240
217757_at	6,791	6,804	6,684	6,578	6,700	6,881
217758_s_at	10,705	10,776	10,692	10,920	11,767	10,958
217759_at	10,079	9,962	9,989	8,774	10,226	9,428
217760_at	9,865	9,823	9,894	8,385	9,460	8,686

217761_at	10,083	9,776	10,551	11,884	11,347	12,198
217762_s_at	9,839	9,870	9,732	9,453	9,137	8,636
217763_s_at	8,427	8,615	8,336	9,164	8,617	8,868
217764_s_at	10,427	10,549	10,351	10,045	10,318	9,583
217765_at	9,401	9,264	9,567	9,252	8,491	9,087
217766_s_at	11,342	11,292	11,366	9,978	10,457	10,373
217767_at	7,298	7,236	7,226	9,382	7,393	7,468
217768_at	13,274	13,629	13,270	12,936	12,909	13,179
217769_s_at	12,548	12,488	12,572	12,710	12,844	12,999
217770_at	9,117	9,134	9,412	9,280	9,496	9,599
217771_at	10,996	10,795	10,812	9,126	9,615	8,784
217772_s_at	12,717	12,709	12,971	12,691	12,004	12,672
217773_s_at	13,303	13,352	13,282	12,125	12,132	12,582
217774_s_at	12,800	12,868	12,730	12,163	11,700	12,408
217775_s_at	9,425	9,702	9,402	9,995	9,915	10,258
217776_at	11,369	11,490	11,040	11,566	12,089	11,873
217777_s_at	10,350	10,417	10,269	9,180	8,447	8,770
217778_at	8,593	8,608	9,196	8,306	7,754	8,226
217779_s_at	13,200	13,249	12,756	10,859	11,605	10,429
217780_at	9,925	9,667	10,144	10,379	10,177	10,663
217781_s_at	9,239	9,278	9,063	8,513	9,465	8,475
217782_s_at	8,986	8,735	8,845	8,938	8,324	9,088
217783_s_at	10,373	11,181	10,368	9,222	10,315	10,115
217784_at	10,105	9,715	9,996	9,581	9,142	9,736
217785_s_at	7,208	6,701	7,162	8,638	7,752	8,225
217786_at	11,460	11,101	11,475	11,338	10,806	11,247
217787_s_at	8,002	7,514	7,903	8,643	9,160	9,193
217788_s_at	11,811	11,803	11,724	11,135	11,445	11,598
217789_at	10,804	11,005	10,495	9,929	10,816	10,058
217790_s_at	7,922	7,941	7,859	8,307	8,829	7,817
217791_s_at	10,332	10,030	10,309	10,142	10,230	10,570
217792_at	9,679	9,780	9,750	9,209	9,056	9,326
217793_at	6,955	7,227	6,919	8,048	7,971	7,986
217794_at	10,015	10,188	10,126	9,598	9,552	9,793
217795_s_at	9,821	9,972	10,014	10,449	10,539	10,822
217796_s_at	10,130	9,846	10,260	9,575	9,346	9,844
217797_at	11,606	11,510	11,552	11,148	11,001	11,463
217798_at	10,471	10,696	10,222	9,202	9,867	8,889
217799_x_at	9,134	9,001	9,092	9,602	9,232	9,393
217800_s_at	9,041	8,945	9,159	9,152	9,162	9,483
217801_at	10,628	10,928	10,588	12,287	12,017	12,606
217802_s_at	12,200	12,204	12,214	9,888	10,651	9,867
217803_at	10,685	10,440	10,188	9,988	10,169	10,506
217804_s_at	11,343	10,916	11,202	9,435	9,545	9,654
217805_at	9,878	9,683	9,443	9,144	8,596	9,090
217806_s_at	9,837	9,386	9,868	10,331	9,667	10,351
217807_s_at	13,389	13,656	13,600	12,282	11,858	12,275
217808_s_at	9,024	8,639	9,093	9,001	8,558	8,817
217809_at	11,666	11,725	11,577	11,456	11,431	11,459
217810_x_at	11,136	11,386	10,799	9,138	10,508	9,477
217811_at	9,043	8,734	8,447	9,301	9,129	9,483
217812_at	13,088	13,165	13,109	11,463	11,626	11,692
217813_s_at	9,431	9,406	9,415	8,161	8,115	8,447
217814_at	10,382	9,702	9,775	9,169	10,243	8,936
217815_at	11,341	11,133	10,774	9,321	9,787	8,666
217816_s_at	12,113	12,235	11,808	10,481	11,305	10,689

217817_at	10,129	9,696	10,150	9,539	9,700	10,015
217818_s_at	8,066	8,032	8,195	7,765	7,452	7,831
217819_at	11,543	11,684	11,311	10,612	10,874	10,848
217820_s_at	12,416	12,098	11,920	9,691	11,349	10,488
217821_s_at	10,950	10,824	10,706	8,320	8,189	8,208
217822_at	13,068	12,920	13,191	11,158	11,392	11,079
217823_s_at	9,106	9,140	8,899	9,990	10,387	10,204
217824_at	5,876	5,968	5,833	6,147	6,223	6,241
217825_s_at	8,229	8,108	7,971	8,688	8,869	8,771
217826_s_at	7,344	7,266	7,081	8,652	7,825	8,616
217827_s_at	11,721	11,939	11,870	11,441	10,814	11,394
217828_at	12,054	11,854	11,845	9,366	10,258	8,610
217829_s_at	10,484	10,235	10,468	9,580	9,220	9,591
217830_s_at	9,945	9,909	10,431	8,384	8,758	8,896
217831_s_at	9,019	9,024	9,200	8,917	8,359	8,840
217832_at	10,840	10,457	10,764	10,144	10,615	9,935
217833_at	10,565	10,718	10,003	8,278	9,484	8,182
217834_s_at	10,769	10,199	10,798	11,281	11,518	10,787
217835_x_at	12,387	12,375	12,261	13,055	12,577	13,118
217836_s_at	10,618	10,622	10,957	9,711	9,643	9,706
217837_s_at	9,616	9,894	9,969	10,324	11,367	11,001
217838_s_at	8,734	8,814	8,710	8,257	8,149	8,567
217839_at	12,356	12,496	12,579	12,316	12,792	12,912
217840_at	10,209	10,053	10,411	10,444	10,059	10,508
217841_s_at	9,769	9,678	10,008	10,224	10,005	10,380
217842_at	11,010	11,023	10,945	9,328	9,970	8,889
217843_s_at	11,540	11,708	11,066	9,961	10,346	9,739
217844_at	8,941	8,871	9,114	8,871	8,674	9,092
217845_x_at	12,810	12,973	12,733	12,230	12,843	12,733
217846_at	11,763	12,117	12,098	11,443	11,142	11,649
217847_s_at	7,035	7,208	6,948	6,605	6,452	6,454
217848_s_at	12,993	13,011	12,861	12,597	13,116	12,968
217849_s_at	7,588	7,637	7,800	7,719	8,053	8,210
217850_at	11,420	11,630	10,595	9,938	10,446	9,548
217851_s_at	10,284	10,235	9,975	9,859	10,270	9,551
217852_s_at	11,425	11,616	11,480	11,371	11,577	11,195
217853_at	10,622	10,417	11,081	10,051	11,250	10,664
217854_s_at	11,475	10,985	11,441	11,885	11,124	11,460
217855_x_at	9,484	9,187	9,773	10,330	10,219	10,652
217856_at	7,203	6,904	7,527	5,678	5,437	5,557
217857_s_at	9,656	9,070	9,370	8,015	8,128	8,288
217858_s_at	10,937	11,258	10,779	10,247	11,261	10,761
217859_s_at	5,812	5,905	5,913	6,379	6,545	6,692
217860_at	10,619	10,657	10,550	10,514	9,811	10,405
217861_s_at	9,295	9,155	9,227	9,512	8,700	9,533
217862_at	9,555	9,717	9,087	6,983	7,999	7,226
217863_at	9,524	10,166	9,464	7,108	8,774	7,099
217864_s_at	10,280	10,478	9,765	9,015	9,495	8,805
217865_at	9,598	9,789	9,529	7,887	7,368	8,366
217866_at	10,916	10,562	10,854	9,158	9,542	9,497
217867_x_at	9,433	9,680	9,485	8,058	8,330	8,859
217868_s_at	12,212	12,484	12,294	12,442	12,761	12,658
217869_at	12,449	12,739	12,374	11,712	12,482	11,932
217870_s_at	11,069	11,533	10,854	11,360	12,101	11,817
217871_s_at	10,553	10,308	10,772	10,884	10,023	11,457
217872_at	11,005	10,708	10,713	9,827	9,307	9,848

217873_at	11,281	11,519	11,024	10,027	11,330	9,998
217874_at	11,274	11,726	11,602	12,287	11,769	12,269
217875_s_at	7,528	7,469	7,648	7,327	7,419	7,666
217876_at	8,098	7,979	8,156	7,883	7,435	7,831
217877_s_at	9,650	9,859	9,403	7,870	8,142	8,053
217878_s_at	9,531	9,701	9,287	10,251	10,156	9,824
217879_at	9,521	9,779	9,227	9,999	10,742	9,960
217880_at	9,928	10,257	9,775	10,607	11,025	10,287
217881_s_at	7,834	8,022	7,530	7,726	8,226	7,440
217882_at	9,604	9,697	10,024	10,909	10,894	11,402
217883_at	13,267	13,568	13,212	12,826	12,956	13,194
217884_at	10,377	10,398	10,908	10,384	10,093	10,034
217885_at	11,436	11,133	11,182	9,699	9,904	9,816
217886_at	10,220	10,817	9,937	7,655	9,329	7,720
217887_s_at	9,928	10,386	9,762	9,321	9,802	8,733
217888_s_at	9,799	9,857	9,858	9,754	9,688	10,191
217889_s_at	7,454	7,315	6,744	8,757	7,846	9,037
217890_s_at	8,685	8,585	8,867	9,519	9,846	10,147
217891_at	7,608	7,567	7,553	7,445	6,896	7,538
217892_s_at	9,895	10,578	9,625	8,496	10,563	9,951
217893_s_at	11,044	11,084	11,195	9,217	8,686	9,250
217894_at	10,421	10,271	9,669	8,240	8,639	7,569
217895_at	10,906	11,032	10,379	9,711	10,078	9,110
217896_s_at	10,118	10,116	10,351	9,204	9,397	9,224
217897_at	9,306	9,004	8,791	7,027	7,144	7,232
217898_at	10,406	10,672	10,574	11,739	11,541	11,928
217899_at	8,679	8,401	8,440	9,492	9,690	9,825
217900_at	12,399	12,510	12,122	11,137	11,533	10,934
217901_at	11,795	11,665	10,923	5,593	5,582	5,540
217902_s_at	9,302	9,393	9,838	8,273	9,384	8,737
217903_at	7,503	7,740	8,022	7,564	7,220	7,456
217904_s_at	7,675	7,148	7,209	7,816	8,159	7,899
217905_at	9,036	8,913	9,065	8,391	8,534	7,865
217906_at	10,280	10,626	10,462	9,624	9,590	9,966
217907_at	11,095	11,040	11,071	11,573	10,550	11,273
217908_s_at	9,299	9,533	8,667	8,876	9,647	9,227
217909_s_at	8,640	8,712	8,854	10,473	9,470	10,235
217910_x_at	9,000	9,292	9,634	10,574	10,054	10,536
217911_s_at	8,889	9,228	9,063	10,709	10,889	11,348
217912_at	9,261	9,087	9,592	9,761	9,457	9,934
217913_at	9,770	9,673	10,165	10,248	9,894	10,348
217914_at	8,217	8,434	8,447	8,420	8,228	8,382
217915_s_at	11,132	11,576	10,564	10,310	10,321	10,659
217916_s_at	10,430	10,486	10,226	10,025	9,830	9,676
217917_s_at	10,946	11,090	11,109	11,179	10,815	11,416
217918_at	10,286	10,493	10,752	10,568	10,301	10,898
217919_s_at	10,966	11,311	10,867	10,653	10,244	10,715
217920_at	7,403	7,666	7,335	7,616	8,065	7,688
217921_at	6,248	5,901	6,113	6,137	7,269	5,935
217922_at	10,643	10,743	10,466	9,503	10,590	9,115
217923_at	10,310	10,276	10,495	10,440	9,862	10,500
217924_at	7,497	6,976	7,802	7,692	7,130	7,650
217925_s_at	9,466	9,282	9,502	9,352	9,322	9,459
217926_at	10,732	10,435	10,741	11,422	10,673	11,481
217927_at	13,205	13,188	13,379	12,872	12,995	13,155
217928_s_at	9,024	8,946	8,680	8,341	8,454	8,259

217929_s_at	8,865	8,957	9,032	8,380	7,950	8,298
217930_s_at	7,826	7,726	7,807	8,926	7,911	8,833
217931_at	8,911	8,695	8,974	8,687	8,437	9,008
217932_at	9,608	9,417	9,638	9,646	8,964	9,649
217933_s_at	12,030	11,684	11,508	11,043	11,311	11,105
217934_x_at	10,064	9,898	10,320	11,180	10,765	11,480
217935_s_at	9,754	9,775	9,897	9,683	9,890	10,048
217936_at	8,154	8,101	7,464	5,190	5,949	4,738
217937_s_at	7,607	7,456	7,811	7,513	7,452	7,708
217938_s_at	12,180	12,664	12,634	12,046	12,157	12,356
217939_s_at	9,283	9,749	9,085	8,493	8,699	8,423
217940_s_at	10,422	10,210	10,763	10,104	9,969	10,324
217941_s_at	9,598	9,430	9,066	9,368	11,001	9,933
217942_at	12,027	12,410	12,200	10,698	11,017	10,999
217943_s_at	9,592	9,268	9,440	9,625	9,196	9,918
217944_at	9,016	9,199	9,179	9,771	9,067	9,736
217945_at	11,592	11,970	11,546	11,128	11,911	11,670
217946_s_at	11,236	11,011	11,096	10,339	10,090	10,263
217947_at	11,267	11,237	10,942	10,294	11,147	10,934
217948_at	7,844	7,683	7,977	8,495	8,004	8,581
217949_s_at	10,322	10,296	10,293	10,665	9,955	11,079
217950_at	9,547	9,480	9,746	9,658	9,046	9,717
217951_s_at	8,071	7,945	7,695	7,157	6,778	6,372
217952_x_at	9,675	9,597	9,024	8,331	9,411	7,721
217953_at	4,170	4,307	4,334	4,052	4,110	3,945
217954_s_at	10,527	10,590	9,836	8,403	9,425	6,946
217955_at	9,423	9,734	9,794	9,875	10,227	10,292
217956_s_at	11,569	11,718	11,384	11,018	11,040	10,859
217957_at	12,032	11,963	12,137	11,088	10,660	10,904
217958_at	10,756	10,613	10,873	11,623	12,044	11,997
217959_s_at	10,072	9,758	9,910	10,849	11,344	11,309
217960_s_at	8,483	8,375	8,630	8,873	8,167	8,545
217961_at	10,541	10,630	11,129	10,111	9,345	9,963
217962_at	10,943	11,004	11,159	11,692	11,420	11,942
217963_s_at	14,346	14,368	14,416	13,547	13,928	13,886
217964_at	11,341	11,482	11,231	9,136	9,790	9,560
217965_s_at	9,206	9,223	9,322	10,144	9,412	9,955
217966_s_at	8,410	8,601	8,445	6,717	7,217	6,548
217967_s_at	8,319	8,591	8,535	5,246	6,857	5,052
217968_at	9,217	9,528	9,416	9,740	9,082	9,399
217969_at	10,721	10,848	11,160	10,331	10,049	10,702
217970_s_at	10,262	9,919	10,171	8,444	9,162	7,985
217971_at	10,293	10,845	10,137	10,778	10,720	11,076
217972_at	10,790	10,730	10,893	10,107	9,824	9,854
217973_at	9,285	9,377	9,379	9,366	8,657	9,399
217974_at	9,372	9,264	9,125	6,819	6,800	6,596
217975_at	11,776	12,011	11,091	11,588	12,316	12,130
217976_s_at	10,449	10,534	9,818	9,074	9,360	9,117
217977_at	9,854	9,757	9,944	9,830	8,852	9,726
217978_s_at	10,629	10,555	10,978	10,214	10,140	10,306
217979_at	11,064	11,064	10,898	9,808	9,662	10,773
217980_s_at	10,810	10,868	11,117	10,759	9,655	10,540
217981_s_at	9,059	8,924	9,383	9,709	8,889	9,462
217982_s_at	14,059	14,061	14,003	12,881	13,457	13,391
217983_s_at	9,229	9,881	9,543	9,588	9,305	9,523
217984_at	9,333	10,093	9,641	9,845	9,843	9,779

217985_s_at	8,251	8,044	7,698	8,143	8,844	7,579
217986_s_at	9,064	8,984	8,592	8,285	9,254	7,385
217987_at	12,343	12,464	11,892	10,322	10,792	10,385
217988_at	11,271	12,067	11,477	9,321	8,855	9,198
217989_at	10,356	10,961	10,334	8,183	8,817	8,667
217990_at	9,872	9,691	9,467	9,531	8,881	9,403
217991_x_at	9,447	9,158	8,832	7,775	7,121	7,511
217992_s_at	9,211	9,175	9,262	10,361	10,058	10,277
217993_s_at	11,954	12,036	11,741	11,366	11,286	11,241
217994_x_at	9,315	9,154	9,441	9,624	9,394	9,762
217995_at	6,320	6,515	6,678	12,224	12,135	12,544
217996_at	8,511	8,457	8,655	11,730	11,085	11,257
217997_at	8,407	8,309	8,730	11,290	10,791	10,712
217998_at	8,342	8,188	8,443	9,640	8,962	8,790
217999_s_at	6,631	6,448	6,662	8,634	8,581	8,038
218000_s_at	6,427	6,589	6,461	8,004	8,005	7,906
218001_at	9,885	9,727	9,903	9,903	8,362	9,379
218002_s_at	6,173	6,709	6,319	5,953	5,814	5,981
218003_s_at	11,505	11,444	11,085	10,922	11,089	10,905
218004_at	9,639	9,725	9,789	9,239	9,014	9,621
218005_at	10,327	10,458	10,176	8,859	8,400	8,669
218006_s_at	9,364	9,089	8,638	7,145	5,846	7,123
218007_s_at	11,350	11,923	11,233	12,970	12,967	13,178
218008_at	10,303	10,259	10,400	10,640	10,271	10,713
218009_s_at	11,323	11,592	11,130	10,469	10,714	9,542
218010_x_at	7,570	7,490	7,702	7,307	7,492	7,565
218011_at	11,726	11,764	11,719	11,737	11,585	12,123
218012_at	9,429	9,539	9,497	9,109	9,439	9,147
218013_x_at	8,033	7,975	7,742	7,381	7,379	7,701
218014_at	10,473	10,378	10,679	10,106	9,228	9,567
218015_s_at	7,063	6,459	6,649	7,373	7,249	7,215
218016_s_at	10,947	10,923	11,086	10,210	9,608	9,896
218017_s_at	8,718	8,300	9,114	9,136	9,653	9,679
218018_at	9,671	10,090	9,874	11,031	10,679	11,137
218019_s_at	10,014	9,889	10,078	8,640	8,603	8,881
218020_s_at	8,507	8,845	8,646	8,082	8,483	8,477
218021_at	9,001	8,800	8,825	10,279	9,885	10,304
218022_at	9,295	9,330	9,239	9,409	8,673	9,305
218023_s_at	10,742	10,850	11,088	9,752	9,602	10,051
218024_at	8,151	8,281	8,447	10,195	9,838	10,489
218025_s_at	10,488	10,538	10,238	11,185	11,240	11,261
218026_at	9,896	9,737	10,166	10,777	9,902	10,773
218027_at	11,898	11,943	12,287	12,133	11,469	11,736
218028_at	7,790	7,553	7,780	9,331	8,824	9,505
218029_at	8,089	8,439	8,023	9,200	8,966	9,594
218030_at	8,749	8,649	8,995	9,744	9,477	9,596
218031_s_at	9,378	9,138	9,289	5,749	6,691	6,805
218032_at	11,357	11,102	11,377	9,179	9,128	9,378
218033_s_at	6,496	6,563	6,528	6,904	6,286	6,807
218034_at	10,289	10,221	10,006	10,543	9,854	10,746
218035_s_at	9,383	9,832	9,926	5,815	5,667	5,847
218036_x_at	9,070	9,285	8,372	8,131	8,203	8,373
218037_at	9,633	9,688	9,955	9,385	8,984	9,496
218038_at	9,439	9,326	9,800	9,046	8,758	9,185
218039_at	12,748	12,958	12,371	11,034	11,120	10,200
218040_at	10,277	10,145	9,668	8,221	8,899	7,878

218041_x_at	12,033	12,467	12,048	11,337	13,175	12,395
218042_at	11,004	11,289	11,037	10,800	11,483	11,498
218043_s_at	9,041	9,256	8,697	8,885	9,699	8,940
218044_x_at	7,968	8,228	7,991	7,909	7,937	7,926
218045_x_at	7,619	7,815	7,706	7,691	7,622	7,534
218046_s_at	9,367	9,477	9,326	10,633	9,644	10,360
218047_at	11,753	12,163	11,320	9,788	9,885	9,092
218048_at	8,959	9,334	8,588	10,854	10,698	10,919
218049_s_at	10,325	10,595	10,150	10,520	10,123	10,381
218050_at	10,382	10,402	9,789	10,006	10,913	10,405
218051_s_at	10,501	10,126	10,266	9,430	8,335	9,886
218052_s_at	9,597	9,279	10,209	9,898	9,597	10,102
218053_at	12,610	12,667	12,204	10,825	11,674	10,381
218054_s_at	7,131	7,287	7,422	7,333	7,327	7,154
218055_s_at	7,866	8,480	7,772	9,203	10,195	9,477
218056_at	10,328	10,508	10,601	9,899	9,344	9,907
218057_x_at	10,814	10,703	11,188	11,224	10,531	10,997
218058_at	9,085	8,352	9,237	8,636	8,171	8,449
218059_at	10,215	10,316	10,165	10,909	10,118	10,657
218060_s_at	9,052	8,941	9,337	9,226	8,466	8,857
218061_at	10,474	10,242	10,650	11,068	10,068	11,022
218062_x_at	8,272	8,403	8,380	8,694	8,651	9,004
218063_s_at	6,416	6,525	6,839	6,958	6,761	6,835
218064_s_at	8,736	9,018	8,230	7,504	7,110	7,566
218065_s_at	9,026	9,427	9,265	11,102	11,439	11,399
218066_at	9,723	9,397	9,490	9,142	8,947	9,398
218067_s_at	11,766	11,725	11,353	10,148	11,368	10,199
218068_s_at	9,595	9,416	9,714	8,761	8,239	8,713
218069_at	11,528	11,535	11,767	11,398	10,496	11,024
218070_s_at	8,288	8,399	8,513	9,628	9,155	9,796
218071_s_at	9,322	9,391	9,168	8,960	8,957	9,283
218072_at	9,108	8,582	9,177	9,728	8,790	9,658
218073_s_at	9,031	9,057	9,210	8,471	7,712	7,540
218074_at	10,653	10,840	10,850	10,972	10,235	10,885
218075_at	9,118	9,120	9,111	8,851	8,258	8,553
218076_s_at	10,047	9,813	10,383	9,535	9,943	9,842
218077_s_at	8,114	7,928	8,202	8,752	8,412	8,787
218078_s_at	9,754	9,040	9,799	9,128	9,066	9,717
218079_s_at	9,959	10,066	9,678	9,040	10,011	8,897
218080_x_at	10,517	10,566	10,318	9,504	9,178	9,153
218081_at	8,996	8,890	9,182	9,328	9,110	9,188
218082_s_at	11,690	11,338	11,951	10,108	10,467	10,034
218083_at	9,300	9,103	9,240	9,790	9,558	9,701
218084_x_at	8,619	8,870	8,975	10,117	9,353	10,107
218085_at	12,227	12,553	12,034	11,859	12,121	12,289
218086_at	8,605	8,308	8,518	8,819	9,191	9,468
218087_s_at	6,668	7,033	6,164	3,685	3,755	3,610
218088_s_at	10,661	11,159	11,081	10,776	11,180	10,780
218089_at	9,898	9,768	10,161	10,075	9,772	10,112
218090_s_at	10,414	10,702	10,208	9,491	10,635	9,632
218091_at	10,844	11,087	10,833	9,961	10,585	9,923
218092_s_at	10,084	10,182	10,098	10,278	10,727	10,542
218093_s_at	9,383	9,289	9,562	7,555	7,920	6,670
218094_s_at	9,190	9,181	9,303	8,726	8,265	8,671
218095_s_at	10,483	10,526	10,733	11,796	12,093	12,336
218096_at	12,043	11,797	11,843	10,153	10,431	9,444

218097_s_at	10,007	9,891	9,983	10,213	9,654	10,485
218098_at	9,092	9,540	9,436	7,913	9,333	7,801
218099_at	8,559	8,465	8,570	8,748	9,156	9,274
218100_s_at	9,904	10,152	9,717	9,650	9,801	9,752
218101_s_at	12,060	11,963	12,055	12,172	11,867	12,409
218102_at	12,612	13,022	12,612	10,715	11,182	11,064
218103_at	10,703	10,303	10,805	10,380	9,810	10,111
218104_at	11,593	11,347	11,694	10,341	10,219	10,006
218105_s_at	9,382	9,261	9,498	9,501	8,356	8,947
218106_s_at	10,293	10,288	10,125	9,753	10,211	9,316
218107_at	11,454	11,288	11,505	10,391	11,036	10,618
218108_at	10,251	10,092	10,087	9,214	9,648	9,137
218109_s_at	9,801	9,728	9,732	11,497	11,943	11,801
218110_at	7,597	7,571	7,230	7,264	7,385	7,584
218111_s_at	11,167	11,187	10,192	8,902	8,338	8,702
218112_at	9,407	9,276	9,498	9,425	8,654	9,045
218113_at	9,488	8,749	8,796	7,959	8,539	8,241
218114_at	8,225	8,480	8,322	8,249	8,081	8,453
218115_at	9,705	9,387	9,610	8,690	8,298	8,329
218116_at	9,324	9,238	9,353	8,714	8,787	8,362
218117_at	11,447	11,585	11,301	11,783	11,047	11,819
218118_s_at	10,833	10,227	10,411	10,720	10,087	10,742
218119_at	9,477	9,099	9,080	8,936	8,390	9,065
218120_s_at	8,995	8,873	8,806	9,484	8,883	9,493
218121_at	8,612	8,526	8,700	9,811	9,122	9,374
218122_s_at	8,819	8,773	8,449	7,956	8,184	7,893
218123_at	11,966	12,066	12,032	10,739	10,080	10,414
218124_at	7,909	8,259	7,974	9,474	8,884	9,428
218125_s_at	10,117	10,383	9,951	8,498	8,909	8,494
218126_at	9,078	8,738	9,479	9,877	9,714	9,977
218127_at	11,212	11,718	11,050	7,702	7,857	7,750
218128_at	10,110	10,468	9,980	6,768	6,972	6,711
218129_s_at	8,237	8,708	8,059	5,784	4,727	5,307
218130_at	7,993	7,941	8,529	8,950	8,388	9,009
218131_s_at	10,371	9,962	10,861	9,821	9,713	9,702
218132_s_at	9,164	9,388	9,588	10,621	10,131	10,680
218133_s_at	11,774	11,965	11,843	10,658	9,939	10,198
218134_s_at	10,407	10,764	10,862	10,673	10,678	10,496
218135_at	11,127	11,600	10,932	10,003	10,791	10,470
218136_s_at	7,681	7,271	7,109	6,974	7,323	7,377
218137_s_at	10,125	10,404	10,290	9,683	10,223	10,005
218138_at	10,618	10,700	10,624	9,336	9,535	8,735
218139_s_at	10,278	10,078	10,143	8,535	9,763	9,135
218140_x_at	10,877	10,770	11,525	11,765	12,013	12,187
218141_at	8,319	8,232	8,508	7,934	7,454	7,822
218142_s_at	9,756	10,192	9,476	8,002	8,360	8,160
218143_s_at	9,459	9,363	9,783	9,771	9,637	10,160
218144_s_at	7,967	7,939	8,082	8,209	8,260	8,600
218145_at	8,303	9,303	9,212	9,546	9,180	9,736
218146_at	10,450	10,706	10,364	10,752	11,389	10,979
218147_s_at	8,846	9,201	8,631	9,604	10,148	9,796
218148_at	7,499	7,360	7,694	7,185	6,657	7,300
218149_s_at	10,836	11,147	11,108	8,480	9,106	9,101
218150_at	11,862	11,866	11,678	11,153	11,513	11,449
218151_x_at	9,180	8,958	9,502	10,413	9,666	10,612
218152_at	10,322	10,631	10,243	8,171	8,269	8,181

218153_at	9,846	9,774	10,207	11,445	10,904	11,048
218154_at	7,327	7,370	7,501	8,729	8,078	8,164
218155_x_at	8,716	8,617	8,824	7,758	8,592	7,810
218156_s_at	10,701	9,928	10,561	9,787	9,712	9,525
218157_x_at	9,939	10,127	10,491	9,776	9,956	9,815
218158_s_at	9,067	9,300	8,868	8,057	8,386	7,286
218159_at	9,262	9,425	9,739	10,152	9,729	10,382
218160_at	11,404	11,772	11,858	11,620	11,258	11,627
218161_s_at	7,816	7,783	8,339	7,879	7,108	7,399
218162_at	10,900	11,245	11,931	8,080	8,665	9,653
218163_at	11,421	11,676	11,778	12,240	11,733	12,121
218164_at	8,557	8,656	8,524	9,789	9,134	10,155
218165_at	10,637	10,581	10,385	9,816	8,933	9,616
218166_s_at	9,097	9,349	8,600	6,102	7,763	5,523
218167_at	11,775	12,102	11,450	11,537	11,395	11,651
218168_s_at	9,658	9,872	9,897	8,939	8,133	8,611
218169_at	8,862	8,720	9,061	8,573	8,122	8,435
218170_at	11,142	11,060	10,871	10,632	10,078	10,256
218171_at	10,792	11,048	10,737	10,583	11,444	10,431
218172_s_at	9,169	8,921	8,770	8,339	8,461	8,855
218173_s_at	8,285	8,372	8,231	6,806	6,642	6,493
218174_s_at	7,612	7,633	7,493	7,015	6,994	7,286
218175_at	6,983	7,440	7,095	8,791	9,713	9,899
218176_at	10,034	10,289	10,118	8,586	7,907	8,180
218177_at	7,020	7,122	7,021	8,020	8,758	8,558
218178_s_at	8,092	7,843	7,988	8,520	8,685	8,744
218179_s_at	8,285	8,792	7,696	7,377	8,487	7,087
218180_s_at	7,638	7,868	7,816	8,355	8,670	8,817
218181_s_at	10,163	9,972	9,893	8,507	10,206	8,558
218182_s_at	5,357	5,216	5,341	5,882	5,426	6,143
218183_at	7,680	7,687	8,100	7,478	7,709	7,921
218184_at	7,846	7,752	8,152	6,171	7,397	6,725
218185_s_at	10,257	10,170	9,967	8,903	8,734	9,329
218186_at	8,277	9,503	8,667	6,622	6,779	6,826
218187_s_at	9,408	9,336	9,929	9,363	8,956	9,281
218188_s_at	10,856	10,496	11,269	11,115	10,691	10,843
218189_s_at	10,357	10,525	10,686	10,859	10,448	11,186
218190_s_at	10,485	10,637	10,582	11,028	10,577	11,142
218191_s_at	9,372	9,739	9,041	8,885	9,440	9,341
218192_at	11,079	11,161	11,281	9,184	9,545	9,868
218193_s_at	9,538	9,710	9,553	10,620	10,651	10,824
218194_at	12,026	12,028	12,097	12,589	12,732	12,781
218195_at	11,284	11,313	10,833	9,981	10,426	10,300
218196_at	9,549	10,168	9,537	9,411	9,821	9,748
218197_s_at	6,432	7,050	5,740	5,407	6,579	5,918
218198_at	10,245	10,444	10,399	9,937	9,903	9,796
218199_s_at	8,931	8,573	9,144	9,177	8,913	9,136
218200_s_at	12,932	13,020	13,095	12,686	12,587	12,905
218201_at	11,917	11,935	12,095	12,079	11,690	12,145
218202_x_at	7,215	7,602	7,693	8,231	7,325	7,920
218203_at	11,042	11,195	11,134	11,515	11,571	11,509
218204_s_at	8,424	8,664	8,786	7,510	9,126	8,512
218205_s_at	11,992	12,068	12,298	10,969	11,375	11,619
218206_x_at	9,415	9,057	9,452	10,116	9,488	10,020
218207_s_at	5,871	5,980	5,962	6,559	6,179	6,821
218208_at	8,383	8,273	9,226	9,226	8,943	9,422

218209_s_at	9,928	10,117	9,957	9,577	10,195	9,935
218210_at	9,621	9,379	9,424	9,899	9,539	9,672
218211_s_at	7,665	8,032	8,055	10,902	10,767	11,249
218212_s_at	8,308	8,189	7,920	9,131	8,697	9,130
218213_s_at	12,731	12,632	12,876	12,562	11,920	12,558
218214_at	10,427	10,383	10,597	10,622	10,144	10,848
218215_s_at	7,899	7,288	7,649	7,987	7,214	8,089
218216_x_at	10,323	10,292	10,343	10,564	9,853	10,532
218217_at	8,626	8,688	8,455	8,672	8,646	9,132
218218_at	8,214	8,211	7,798	6,631	7,320	6,634
218219_s_at	9,237	8,758	9,379	9,456	8,745	8,957
218220_at	9,395	9,267	9,668	9,976	8,795	9,747
218221_at	8,478	8,898	8,399	6,971	7,276	7,495
218222_x_at	8,110	8,350	8,223	8,320	8,043	8,219
218223_s_at	9,032	8,455	8,573	8,507	7,448	8,064
218224_at	10,277	10,092	10,288	10,725	10,901	11,034
218225_at	8,884	8,640	9,052	9,342	8,703	9,048
218226_s_at	10,905	11,129	10,971	10,911	10,456	10,959
218227_at	8,495	8,387	9,058	9,547	8,747	9,432
218228_s_at	9,490	9,605	8,928	6,842	8,181	7,223
218229_s_at	10,416	10,088	10,541	8,281	8,412	8,189
218230_at	10,739	10,836	10,454	10,147	10,835	10,095
218231_at	9,536	9,315	9,742	10,321	9,959	10,784
218232_at	5,733	6,014	6,356	6,520	6,436	6,756
218233_s_at	11,988	11,967	12,110	12,663	12,235	12,653
218234_at	9,110	8,916	9,007	8,380	7,895	8,338
218235_s_at	10,634	10,442	10,116	9,881	9,845	9,545
218236_s_at	10,140	9,860	9,542	7,890	9,080	7,014
218237_s_at	12,242	11,809	12,030	8,333	10,177	8,040
218238_at	11,443	11,546	10,903	10,688	10,930	10,323
218239_s_at	11,935	12,031	11,625	11,339	11,530	11,082
218240_at	9,549	9,384	9,731	9,028	8,954	9,306
218241_at	9,917	10,631	9,667	9,516	10,465	9,491
218242_s_at	9,766	9,411	9,393	6,184	7,942	6,653
218243_at	9,400	9,563	9,672	9,073	9,486	9,308
218244_at	8,619	8,630	8,278	7,623	8,102	6,638
218245_at	8,685	8,538	8,841	8,699	8,603	9,082
218246_at	9,667	9,454	9,895	10,261	9,858	10,437
218247_s_at	10,968	10,973	10,876	9,218	9,818	9,595
218248_at	8,227	8,400	7,808	8,876	9,140	8,255
218249_at	10,260	9,958	10,279	10,394	9,793	10,065
218250_s_at	11,957	11,794	11,593	10,858	10,534	10,839
218251_at	8,290	8,211	8,436	7,977	7,130	7,799
218252_at	11,190	11,174	10,734	8,949	9,691	8,097
218253_s_at	9,357	9,964	9,545	10,165	9,861	10,260
218254_s_at	7,864	8,207	7,865	10,478	10,629	10,994
218255_s_at	7,459	7,592	7,488	8,937	8,422	8,672
218256_s_at	11,776	11,899	11,365	10,453	10,840	10,084
218257_s_at	9,389	9,033	9,158	8,704	9,062	8,733
218258_at	11,128	11,582	11,614	10,800	9,716	10,669
218259_at	10,173	9,971	10,211	8,347	9,536	7,861
218260_at	7,446	7,391	7,405	7,899	7,814	8,320
218261_at	8,219	8,304	7,753	7,092	6,712	7,079
218262_at	8,248	8,506	8,567	7,966	7,721	7,810
218263_s_at	11,130	11,097	10,906	9,114	10,675	9,338
218264_at	9,120	9,393	8,595	8,721	8,373	8,161

218265_at	9,975	10,372	9,752	7,914	8,566	7,925
218266_s_at	7,058	6,642	7,152	7,865	7,101	7,833
218267_at	8,170	7,766	7,799	8,575	8,175	8,475
218268_at	9,695	10,200	9,164	8,285	9,329	7,580
218269_at	11,430	11,474	11,524	10,357	10,712	10,386
218270_at	10,845	10,512	10,593	11,112	10,408	11,068
218271_s_at	11,618	11,384	11,477	10,894	10,394	10,898
218272_at	9,243	9,287	9,218	9,186	8,628	9,278
218273_s_at	6,476	7,211	6,473	6,815	6,762	7,414
218274_s_at	8,790	8,633	8,604	8,402	8,430	8,541
218275_at	8,334	8,051	8,185	8,470	7,856	8,332
218276_s_at	9,189	9,334	9,162	7,478	7,888	7,378
218277_s_at	10,108	10,210	9,836	8,581	9,544	8,558
218278_at	10,263	9,870	10,145	10,773	10,259	10,528
218279_s_at	6,858	7,315	7,637	7,568	7,898	7,952
218280_x_at	8,837	9,190	9,217	9,126	9,075	9,353
218281_at	9,544	9,661	9,517	9,297	8,624	9,058
218282_at	7,919	8,306	8,210	8,542	8,375	8,706
218283_at	11,672	11,897	11,873	11,332	10,715	11,267
218284_at	9,316	9,316	9,449	9,183	10,164	9,186
218285_s_at	9,685	10,076	9,389	9,647	9,655	9,822
218286_s_at	10,259	10,546	10,396	10,547	9,663	10,344
218287_s_at	11,146	11,179	11,754	9,271	9,804	9,287
218288_s_at	11,050	11,471	11,074	10,851	11,071	11,291
218289_s_at	10,031	10,181	9,569	8,657	9,485	8,514
218290_at	10,264	9,966	10,176	9,495	8,683	9,602
218291_at	8,063	8,025	8,349	8,699	8,120	8,777
218292_s_at	7,740	7,980	7,521	8,374	7,723	8,625
218293_x_at	5,301	5,475	5,665	5,553	5,249	5,379
218294_s_at	10,246	9,722	9,679	7,732	8,357	7,624
218295_s_at	7,907	7,922	7,800	8,327	8,325	7,967
218296_x_at	8,435	8,167	8,740	8,532	7,869	8,490
218297_at	8,326	8,833	8,092	9,492	9,941	9,842
218298_s_at	9,407	9,421	9,664	8,723	8,325	8,372
218299_at	8,176	8,141	8,450	8,484	8,174	8,690
218300_at	9,544	9,089	9,370	9,130	8,196	8,855
218301_at	7,243	7,319	7,300	7,686	7,743	7,712
218302_at	8,563	8,423	8,649	9,448	8,729	9,603
218303_x_at	10,278	10,432	9,955	8,843	9,145	9,554
218304_s_at	10,857	10,786	10,703	8,172	8,709	7,579
218305_at	9,737	9,045	10,016	10,167	9,572	10,118
218306_s_at	9,588	9,559	9,428	8,317	9,527	7,964
218307_at	8,761	8,986	8,806	9,225	8,491	8,897
218308_at	10,490	10,316	10,433	9,491	8,770	8,956
218309_at	9,238	9,527	9,097	11,508	11,027	11,543
218310_at	9,931	10,066	9,772	9,229	10,248	9,539
218311_at	10,266	10,370	10,240	7,595	9,624	7,519
218312_s_at	10,034	10,008	10,019	8,532	8,662	9,259
218313_s_at	10,026	10,013	10,025	8,263	9,770	8,113
218314_s_at	8,961	8,863	8,706	7,383	8,293	7,484
218315_s_at	9,577	9,753	9,907	9,760	9,163	9,514
218316_at	9,196	9,649	9,469	9,491	9,020	9,220
218317_x_at	9,417	9,245	9,673	9,744	9,014	9,528
218318_s_at	8,293	7,893	7,957	6,998	6,709	6,826
218319_at	10,171	10,233	10,126	5,193	5,974	5,358
218320_s_at	11,373	11,276	11,622	10,620	9,825	10,798

218321_x_at	9,066	9,281	9,121	9,548	9,078	9,862
218322_s_at	5,948	5,902	6,163	7,371	6,857	7,017
218323_at	10,730	11,226	10,446	9,150	10,352	9,151
218324_s_at	9,571	9,730	9,730	8,266	8,351	8,462
218325_s_at	9,034	9,378	9,258	8,061	8,346	8,332
218326_s_at	10,507	10,621	10,479	7,281	7,980	6,847
218327_s_at	7,933	8,371	7,887	10,146	8,976	9,665
218328_at	7,964	7,815	8,351	8,990	8,240	8,684
218329_at	9,331	9,332	9,390	9,335	9,623	9,114
218330_s_at	10,683	10,419	10,871	9,274	10,570	9,384
218331_s_at	10,331	10,240	10,071	9,094	10,628	8,706
218332_at	13,634	13,587	13,626	12,870	12,951	13,234
218333_at	9,836	9,648	9,956	10,938	10,430	10,930
218334_at	11,135	11,333	11,140	11,165	11,173	11,101
218335_x_at	9,510	9,466	9,771	9,608	9,048	9,545
218336_at	11,788	12,012	11,467	11,651	10,718	11,288
218337_at	9,179	8,884	9,112	8,652	8,617	9,106
218338_at	13,008	13,141	13,212	8,179	8,175	8,328
218339_at	10,092	10,183	10,015	10,658	10,118	10,412
218340_s_at	7,435	7,790	6,705	6,898	7,413	6,922
218341_at	9,456	10,192	9,631	9,515	8,557	9,448
218342_s_at	9,010	9,641	9,266	8,482	8,939	8,627
218343_s_at	10,069	10,812	9,675	8,889	9,522	8,176
218344_s_at	8,596	8,798	8,719	6,300	7,267	6,708
218345_at	7,368	6,928	7,406	8,007	7,709	8,009
218346_s_at	8,541	9,025	8,646	7,501	8,349	7,949
218347_at	10,187	10,297	10,176	8,907	9,194	8,814
218348_s_at	9,813	9,973	9,513	8,987	9,718	9,432
218349_s_at	10,415	10,174	9,951	8,270	8,497	7,654
218350_s_at	12,139	12,373	12,101	11,317	11,241	10,785
218351_at	10,270	11,119	9,722	11,210	11,870	11,928
218352_at	10,487	10,640	10,358	8,484	9,549	8,553
218353_at	6,983	6,715	6,548	5,489	5,532	5,738
218354_at	9,127	8,750	9,156	9,388	8,186	9,224
218355_at	11,021	11,287	11,086	9,180	9,107	8,289
218356_at	11,283	10,946	11,270	10,187	9,459	9,546
218357_s_at	11,460	11,402	11,765	12,110	11,569	12,099
218358_at	10,078	9,970	10,735	11,940	11,297	11,625
218359_at	7,294	7,105	7,549	7,567	7,108	7,459
218360_at	8,558	9,101	8,435	9,180	9,326	9,438
218361_at	10,543	10,779	10,497	8,511	8,852	8,653
218362_s_at	6,253	6,630	5,935	5,259	5,694	4,725
218363_at	9,418	9,554	9,452	9,531	9,434	9,802
218364_at	8,300	8,139	8,056	7,734	8,378	7,883
218365_s_at	8,717	8,738	8,814	7,852	7,412	7,664
218366_x_at	11,191	10,951	11,424	10,507	9,711	10,343
218367_x_at	9,103	9,167	9,263	8,259	7,612	8,094
218368_s_at	8,830	8,973	8,961	10,894	9,685	11,177
218369_s_at	5,205	5,554	5,804	6,317	6,542	6,724
218370_s_at	11,214	11,770	11,537	8,210	8,703	7,895
218371_s_at	8,638	8,615	8,734	7,605	8,105	7,285
218372_at	7,973	8,174	8,185	7,774	7,280	7,448
218373_at	9,327	9,686	9,068	8,630	8,807	8,847
218374_s_at	9,225	9,411	8,678	7,942	8,706	7,815
218375_at	10,165	10,406	10,126	10,322	10,283	9,852
218376_s_at	9,225	9,264	9,041	8,880	8,946	9,215

218377_s_at	9,308	9,259	9,573	8,802	9,395	9,536
218378_s_at	9,865	9,405	9,732	8,519	8,880	8,855
218379_at	10,318	10,354	9,814	8,918	9,051	8,925
218380_at	8,770	8,796	8,366	7,570	7,365	7,148
218381_s_at	11,019	10,601	11,026	11,381	11,021	11,484
218382_s_at	7,391	7,479	7,713	7,452	7,344	7,720
218383_at	9,105	9,714	9,028	8,764	8,059	8,443
218384_at	8,651	8,456	8,463	8,814	8,192	8,394
218385_at	9,655	9,951	10,287	10,535	9,925	10,308
218386_x_at	10,679	10,689	10,263	9,420	10,166	8,773
218387_s_at	9,573	9,913	10,036	9,505	9,211	9,931
218388_at	8,658	8,743	8,770	8,850	8,245	9,056
218389_s_at	9,000	8,983	9,233	8,765	8,137	8,664
218390_s_at	5,977	5,913	6,053	5,761	5,857	5,837
218391_at	9,863	9,585	9,940	10,089	9,323	10,010
218392_x_at	9,951	10,146	10,188	10,051	10,010	10,074
218393_s_at	9,828	9,787	9,198	8,160	8,307	8,264
218394_at	7,071	6,831	6,790	7,057	6,467	6,884
218395_at	10,643	11,316	10,405	9,086	9,205	9,097
218396_at	9,286	9,850	8,722	8,108	10,419	7,954
218397_at	11,959	12,045	11,567	8,824	9,149	8,171
218398_at	10,757	11,079	11,029	10,891	10,076	10,435
218399_s_at	10,907	10,908	11,024	10,620	9,948	10,398
218400_at	7,705	7,694	7,554	7,821	7,443	7,397
218401_s_at	10,558	10,597	10,707	7,741	7,977	8,162
218402_s_at	9,208	9,159	8,876	8,440	8,018	8,255
218403_at	11,748	11,663	11,647	11,415	11,014	11,304
218404_at	10,945	11,940	10,565	5,837	6,456	6,439
218405_at	8,643	8,526	8,682	9,209	8,105	8,943
218406_x_at	7,484	7,321	7,566	7,985	7,810	8,061
218407_x_at	9,274	9,617	9,612	9,868	9,369	9,981
218408_at	10,544	10,220	10,471	10,541	10,013	10,838
218409_s_at	7,702	7,971	7,918	8,823	9,003	9,050
218410_s_at	8,529	8,311	8,532	7,982	7,655	7,977
218411_s_at	8,605	8,533	8,174	7,704	7,449	7,831
218412_s_at	9,757	9,930	10,262	8,803	9,024	9,356
218413_s_at	7,477	7,808	7,558	7,294	7,470	7,263
218414_s_at	8,631	8,747	9,069	8,273	8,030	8,129
218415_at	9,030	9,327	9,134	8,774	8,471	8,770
218416_s_at	6,503	6,733	6,677	7,093	6,997	7,295
218417_s_at	6,389	6,867	6,333	7,589	7,026	7,610
218418_s_at	9,890	9,890	9,829	9,949	10,739	10,367
218419_s_at	7,574	7,780	7,944	8,750	8,378	8,802
218420_s_at	7,681	7,660	7,708	6,467	6,457	6,337
218421_at	10,160	10,029	10,582	9,140	8,782	9,113
218422_s_at	10,403	10,077	9,973	8,463	9,800	8,544
218423_x_at	10,243	10,664	10,131	9,005	9,820	8,206
218424_s_at	9,082	8,939	9,176	10,036	10,143	9,934
218425_at	8,160	8,023	8,064	7,163	7,128	7,329
218426_s_at	8,062	8,217	8,279	8,096	7,968	8,322
218427_at	11,016	10,782	11,142	9,911	9,445	9,784
218428_s_at	9,681	9,918	9,232	7,832	8,706	7,342
218429_s_at	8,082	8,397	8,241	8,483	8,108	8,545
218430_s_at	8,229	8,340	8,047	5,950	6,091	5,721
218431_at	9,116	9,211	9,336	8,899	8,710	9,105
218432_at	9,099	9,003	8,414	7,891	8,742	7,465

218433_at	7,960	8,001	7,747	7,150	7,140	7,183
218434_s_at	9,929	10,335	10,104	9,849	9,534	10,057
218435_at	7,204	6,615	6,062	11,149	11,058	11,531
218436_at	7,528	7,978	8,075	9,951	9,934	10,388
218437_s_at	8,765	9,195	8,595	7,331	7,899	6,993
218438_s_at	9,716	9,949	9,608	9,976	8,650	9,728
218439_s_at	9,783	10,197	9,721	9,040	8,762	9,204
218440_at	9,290	9,536	9,055	9,120	9,869	9,135
218441_s_at	9,693	9,565	9,904	8,373	8,378	8,460
218442_at	10,628	10,475	10,647	10,248	9,789	10,035
218443_s_at	10,927	10,831	11,159	9,841	10,143	10,318
218444_at	7,016	7,008	7,720	7,746	7,962	8,095
218445_at	11,217	10,649	10,786	9,258	9,214	9,151
218446_s_at	9,342	9,916	9,336	10,306	11,116	11,027
218447_at	11,164	11,521	11,280	11,898	11,191	11,772
218448_at	9,634	9,774	9,962	9,137	9,102	9,087
218449_at	9,120	9,487	8,856	9,707	9,782	9,251
218450_at	11,233	11,844	11,535	11,943	11,977	12,148
218451_at	8,467	8,508	8,714	9,100	8,548	8,522
218452_at	9,081	9,403	9,179	8,757	8,520	8,828
218453_s_at	7,830	8,223	7,988	7,284	7,239	7,494
218454_at	10,120	10,411	10,117	6,799	7,130	7,500
218455_at	9,365	9,665	9,558	9,397	9,317	9,800
218456_at	10,658	10,947	9,993	7,400	9,182	8,104
218457_s_at	10,011	9,837	10,325	7,305	7,357	7,803
218458_at	9,181	9,476	8,881	6,110	6,364	6,138
218459_at	8,455	9,056	9,179	9,282	8,527	8,836
218460_at	11,757	11,675	11,907	10,502	10,070	10,340
218461_at	11,089	11,520	10,867	10,309	9,972	10,097
218462_at	12,613	12,664	12,432	11,141	11,105	10,913
218463_s_at	9,307	9,232	9,387	9,457	9,007	9,462
218464_s_at	10,116	10,180	10,698	8,812	9,435	9,704
218465_at	10,674	10,327	10,656	10,299	10,427	10,174
218466_at	7,837	7,667	7,692	7,479	7,266	7,447
218467_at	11,210	11,680	11,412	11,746	11,335	11,819
218468_s_at	7,212	6,131	6,210	11,879	12,755	11,330
218469_at	7,439	6,721	6,836	12,068	12,768	11,145
218470_at	10,389	10,349	10,449	9,995	9,676	9,888
218471_s_at	9,359	9,087	9,676	7,374	7,390	7,898
218472_s_at	8,480	8,734	8,561	10,098	9,777	10,337
218473_s_at	9,601	9,769	9,816	10,640	10,182	10,752
218474_s_at	10,193	9,771	10,161	9,909	9,573	10,796
218475_at	8,247	8,107	8,095	8,091	7,649	8,265
218476_at	8,118	8,004	7,968	8,070	8,338	8,660
218477_at	10,351	10,779	10,068	11,429	10,955	11,491
218478_s_at	10,572	10,807	10,545	8,763	9,429	8,710
218479_s_at	8,354	8,719	8,060	7,725	8,008	7,044
218480_at	10,029	10,238	10,232	8,901	8,612	8,877
218481_at	9,948	9,635	9,934	9,666	8,705	9,266
218482_at	11,346	11,340	10,838	11,182	10,743	11,118
218483_s_at	8,518	8,481	8,568	9,311	9,000	9,505
218484_at	7,642	7,559	7,765	8,085	7,888	7,930
218485_s_at	8,459	8,289	8,249	9,071	8,616	8,897
218486_at	10,051	10,752	10,328	6,667	7,512	7,260
218487_at	7,863	7,879	7,638	7,979	8,053	8,479
218488_at	10,196	10,574	10,289	10,064	9,844	9,753

218489_s_at	7,262	7,361	7,469	7,850	7,115	7,501
218490_s_at	9,238	9,567	9,050	7,035	7,765	6,637
218491_s_at	10,061	10,282	9,967	9,395	8,620	9,204
218492_s_at	8,694	8,408	8,766	9,050	8,541	8,849
218493_at	11,084	10,964	11,574	10,864	10,163	10,896
218494_s_at	7,872	7,887	7,818	7,803	7,365	7,559
218495_at	10,647	10,912	11,039	10,806	9,853	10,708
218496_at	11,136	10,983	11,441	10,114	10,534	10,248
218497_s_at	10,617	10,544	10,700	10,898	10,482	10,494
218498_s_at	7,641	7,784	7,234	8,227	8,645	7,858
218499_at	12,473	12,672	12,041	10,331	10,546	9,805
218500_at	7,635	7,336	7,747	8,108	7,914	8,430
218501_at	7,670	8,301	8,020	5,808	5,591	5,536
218502_s_at	6,062	6,260	5,974	5,856	7,904	5,599
218503_at	9,682	9,839	9,794	8,925	9,522	8,902
218504_at	9,162	9,682	9,300	9,506	8,697	9,490
218505_at	11,010	11,137	11,037	8,954	8,992	9,042
218506_x_at	10,200	9,838	9,892	10,475	9,521	9,750
218507_at	10,133	10,216	10,709	9,134	8,941	9,023
218508_at	9,434	9,534	9,279	7,611	7,873	8,053
218509_at	7,265	6,980	7,205	7,743	7,325	7,936
218510_x_at	6,022	6,348	6,172	6,203	6,400	6,395
218511_s_at	8,234	8,244	8,650	9,411	9,238	9,610
218512_at	12,130	12,480	12,041	11,096	10,773	10,666
218513_at	8,985	8,654	8,240	8,180	8,137	7,943
218514_at	10,040	10,192	10,234	9,469	9,336	9,633
218515_at	12,785	12,873	12,580	8,576	10,166	8,368
218516_s_at	8,500	8,398	8,466	9,696	9,196	9,661
218517_at	11,580	11,530	11,654	7,976	9,304	8,635
218518_at	9,457	9,548	9,196	7,783	9,473	7,919
218519_at	9,879	10,191	10,066	8,586	9,226	8,549
218520_at	10,647	10,656	10,124	8,337	9,513	7,806
218521_s_at	8,889	8,902	8,516	7,165	7,394	7,405
218522_s_at	8,614	8,270	8,586	8,873	8,993	9,147
218523_at	7,341	7,687	7,748	6,700	6,857	7,123
218524_at	8,704	8,974	9,209	8,758	8,416	8,813
218525_s_at	6,381	6,430	6,627	6,487	6,375	6,611
218526_s_at	8,941	9,053	9,382	9,340	8,492	9,034
218527_at	10,341	10,114	10,619	9,679	9,109	9,182
218528_s_at	11,705	11,750	11,748	8,223	8,780	8,219
218529_at	9,544	9,608	9,913	9,402	8,848	9,451
218530_at	8,487	8,563	8,530	9,074	8,538	9,199
218531_at	8,745	9,263	9,046	8,302	7,491	8,319
218532_s_at	6,284	6,875	6,042	5,926	6,505	5,658
218533_s_at	9,687	9,682	10,113	9,444	8,836	9,480
218534_s_at	4,568	5,270	4,797	8,109	8,655	7,430
218535_s_at	9,785	10,263	9,532	9,611	10,149	8,892
218536_at	11,041	11,679	11,722	8,675	8,991	8,772
218537_at	8,410	8,181	8,368	8,632	8,331	8,387
218538_s_at	10,467	10,993	10,799	8,184	8,202	8,575
218539_at	9,682	9,968	9,766	8,757	9,165	8,581
218540_at	7,825	7,381	7,841	8,494	7,647	7,906
218541_s_at	3,861	4,469	3,524	3,478	3,648	3,517
218542_at	10,061	9,919	9,019	9,040	9,685	7,852
218543_s_at	7,280	8,119	7,598	7,786	7,089	6,784
218544_s_at	9,985	10,011	10,225	9,149	9,333	9,303

218545_at	9,567	9,948	9,103	7,303	8,173	6,634
218546_at	8,590	9,015	8,991	6,265	5,912	6,160
218547_at	9,139	8,857	9,215	8,929	9,224	9,682
218548_x_at	7,979	7,861	7,924	9,007	8,563	9,122
218549_s_at	8,366	9,014	8,336	10,454	10,150	10,403
218550_s_at	8,339	7,858	8,037	7,369	7,622	7,588
218551_at	8,375	8,311	8,499	8,795	8,712	8,723
218552_at	7,633	7,852	7,280	8,052	8,047	8,235
218553_s_at	8,322	7,968	8,695	7,666	7,441	7,636
218554_s_at	6,125	6,028	6,318	6,181	6,189	5,814
218555_at	7,627	7,422	7,604	7,934	7,731	8,058
218556_at	9,045	8,886	9,086	10,400	9,647	10,780
218557_at	10,332	10,594	10,250	10,594	10,095	10,845
218558_s_at	12,147	12,389	11,632	11,035	10,584	10,634
218559_s_at	8,670	9,927	9,743	7,814	8,326	7,620
218560_s_at	8,107	7,922	8,860	8,099	7,560	8,234
218561_s_at	10,017	10,212	10,221	9,734	9,202	9,611
218562_s_at	8,023	8,052	7,847	7,408	7,930	7,790
218563_at	10,071	10,065	10,186	10,340	10,260	10,645
218564_at	9,890	9,478	9,949	8,413	7,650	8,181
218565_at	7,933	7,913	8,093	8,391	7,445	7,818
218566_s_at	10,167	9,928	9,717	10,246	10,528	10,266
218567_x_at	9,947	9,918	10,043	9,413	8,930	9,696
218568_at	9,561	9,910	9,741	8,876	8,966	8,972
218569_s_at	8,581	8,536	8,724	8,795	8,071	8,747
218570_at	8,920	9,083	9,286	9,066	8,581	9,059
218571_s_at	10,692	10,910	11,087	11,231	11,522	11,439
218572_at	8,730	8,762	8,946	8,662	8,988	9,008
218573_at	9,498	9,700	9,444	9,974	9,839	10,143
218574_s_at	9,234	9,323	9,784	7,733	7,738	8,185
218575_at	11,612	11,994	11,501	10,041	10,433	9,392
218576_s_at	11,427	11,337	11,335	10,058	9,685	9,893
218577_at	12,031	12,259	11,489	9,284	10,205	9,530
218578_at	9,491	9,332	9,430	9,341	9,948	9,828
218579_s_at	9,238	9,012	8,827	7,507	7,255	7,290
218580_x_at	10,217	10,111	10,422	11,438	10,394	11,124
218581_at	9,586	9,377	9,805	9,488	9,283	9,758
218582_at	8,878	9,267	8,771	9,478	9,556	9,700
218583_s_at	10,370	10,488	10,177	8,924	9,843	8,790
218584_at	8,930	8,742	8,783	8,458	8,392	8,772
218585_s_at	12,352	12,319	12,259	9,973	9,858	9,003
218586_at	8,521	8,191	8,288	8,535	7,740	8,186
218587_s_at	9,195	9,046	8,782	8,534	8,637	8,658
218588_s_at	7,510	7,448	7,072	6,905	7,488	7,088
218589_at	8,434	9,472	8,815	6,236	8,035	8,183
218590_at	10,067	9,780	10,354	9,169	8,708	8,572
218591_s_at	6,766	6,999	6,178	6,293	6,102	6,368
218592_s_at	10,593	10,757	11,108	10,318	9,298	9,917
218593_at	10,679	10,839	10,484	9,819	9,816	9,529
218594_at	11,438	11,580	11,811	10,256	10,716	9,771
218595_s_at	12,497	12,574	12,667	11,378	11,591	10,913
218596_at	8,507	8,436	8,885	8,414	8,255	8,653
218597_s_at	10,133	10,495	10,286	10,819	10,692	10,908
218598_at	9,521	9,406	9,140	9,163	9,953	9,061
218599_at	8,925	8,765	8,826	8,721	8,584	8,620
218600_at	6,643	6,381	6,693	7,065	7,026	7,305

218601_at	9,567	9,442	9,673	9,894	9,294	9,742
218602_s_at	8,290	8,134	7,488	5,814	6,306	5,168
218603_at	8,469	9,222	8,548	8,307	9,653	8,559
218604_at	11,498	11,521	11,676	9,675	10,591	10,172
218605_at	10,908	10,978	10,893	10,582	10,544	10,474
218606_at	10,457	10,327	10,427	9,440	10,194	10,020
218607_s_at	8,730	8,681	8,557	7,287	7,894	7,424
218608_at	8,072	8,067	8,082	8,939	8,644	8,770
218609_s_at	8,488	8,529	8,419	8,897	8,358	9,201
218610_s_at	5,401	5,405	5,390	6,786	7,138	7,153
218611_at	9,895	10,426	10,243	10,129	10,243	10,575
218612_s_at	8,556	8,316	8,643	8,781	8,297	8,734
218613_at	9,452	9,349	8,976	8,499	10,491	7,815
218614_at	10,837	11,152	10,410	6,972	7,303	6,042
218615_s_at	9,411	9,601	9,419	8,536	8,292	8,670
218616_at	9,091	9,171	8,747	9,356	9,417	9,327
218617_at	10,533	10,290	10,133	9,047	8,802	8,593
218618_s_at	9,516	9,529	9,484	9,624	10,339	9,380
218619_s_at	9,517	9,354	9,605	9,129	8,231	8,609
218620_s_at	8,487	8,755	8,838	9,323	8,425	9,382
218621_at	7,798	7,995	7,375	8,066	8,180	7,976
218622_at	11,886	12,151	11,680	11,298	10,590	10,785
218623_at	6,999	6,909	7,037	7,137	6,927	7,099
218624_s_at	9,247	9,043	9,230	9,662	9,157	9,723
218625_at	5,918	6,083	5,688	9,001	7,578	9,084
218626_at	9,658	9,795	9,972	8,692	8,572	8,745
218627_at	7,212	7,870	7,436	7,379	7,884	7,362
218628_at	7,635	8,122	7,514	9,255	8,760	9,190
218629_at	8,437	8,293	8,309	7,944	7,317	7,612
218630_at	8,043	7,917	7,904	7,777	7,815	7,843
218631_at	8,100	8,214	8,324	9,157	9,496	9,602
218632_at	7,786	7,621	8,027	8,569	7,844	8,415
218633_x_at	10,400	10,840	10,295	9,585	9,968	9,901
218634_at	7,994	8,110	8,144	8,750	8,351	8,653
218636_s_at	9,063	9,170	9,406	9,643	9,439	10,197
218637_at	10,047	10,347	9,821	7,919	8,776	7,785
218638_s_at	7,755	7,907	7,859	6,784	7,865	7,549
218639_s_at	9,052	8,816	9,351	8,271	8,281	8,362
218640_s_at	7,837	8,164	7,627	6,978	6,953	7,339
218641_at	11,100	10,743	11,220	8,458	8,504	8,617
218642_s_at	10,717	10,867	10,565	9,757	10,504	10,271
218643_s_at	9,045	9,327	8,834	8,940	8,770	9,365
218644_at	8,053	7,828	8,074	10,335	8,847	9,330
218645_at	8,655	9,618	8,351	8,129	8,768	8,036
218646_at	11,226	11,703	10,965	10,623	10,922	10,211
218647_s_at	11,754	12,104	12,069	10,868	10,372	11,117
218648_at	9,159	9,197	9,598	9,341	9,285	9,549
218649_x_at	10,616	10,933	10,069	9,440	11,108	8,848
218650_at	8,104	7,968	8,840	7,282	7,741	8,226
218651_s_at	9,493	10,214	9,949	9,671	8,982	9,680
218652_s_at	10,316	10,030	10,637	10,067	10,181	10,468
218653_at	10,614	10,358	10,635	9,422	8,526	9,038
218654_s_at	10,999	11,221	10,930	11,038	10,230	10,745
218655_s_at	8,445	8,825	8,423	8,418	8,389	8,432
218656_s_at	7,777	7,019	8,216	10,422	10,945	10,733
218657_at	7,952	7,739	7,637	7,503	7,589	7,532

218658_s_at	8,605	8,288	8,568	6,582	7,018	6,877
218659_at	9,939	9,826	9,931	8,470	9,315	8,269
218660_at	8,697	8,694	8,956	8,095	8,220	8,622
218661_at	8,575	8,583	9,168	7,627	7,514	7,874
218662_s_at	10,597	10,538	10,043	9,218	9,856	8,480
218663_at	10,407	10,461	9,784	8,548	8,832	6,679
218664_at	8,219	8,505	8,487	8,079	7,652	8,107
218665_at	8,253	8,270	8,426	8,195	7,816	8,014
218666_s_at	7,892	7,931	7,901	6,884	7,017	6,741
218667_at	11,570	11,437	11,676	9,876	9,530	10,228
218668_s_at	9,816	10,012	9,863	9,686	10,236	9,949
218669_at	10,037	10,319	10,162	9,739	10,314	9,927
218670_at	10,499	10,263	10,579	9,831	9,241	9,510
218671_s_at	12,345	12,450	12,368	11,432	11,380	11,649
218672_at	9,915	9,463	10,089	9,692	9,104	9,678
218673_s_at	8,174	8,063	8,341	9,080	8,459	8,847
218674_at	7,756	7,916	7,092	5,928	7,278	5,642
218675_at	7,952	8,013	7,927	7,795	7,607	8,091
218676_s_at	9,668	9,788	9,906	9,415	9,076	9,438
218677_at	6,527	6,538	6,593	6,689	6,611	6,628
218678_at	11,310	10,792	11,138	7,606	7,295	7,406
218679_s_at	9,315	9,426	9,278	10,385	9,687	10,593
218680_x_at	10,358	9,759	9,951	10,540	10,534	10,604
218681_s_at	8,674	8,573	8,985	10,629	9,018	9,537
218682_s_at	8,784	9,020	8,586	8,041	8,498	7,931
218683_at	11,335	11,133	11,082	7,845	10,267	8,240
218684_at	10,223	10,159	10,160	9,644	10,167	9,707
218685_s_at	9,069	8,976	9,049	7,951	6,950	7,910
218686_s_at	8,775	8,951	8,995	9,005	9,584	9,727
218687_s_at	6,601	6,338	6,050	7,394	7,280	7,633
218688_at	7,351	7,097	7,306	7,054	7,322	7,572
218689_at	9,202	8,850	9,586	7,683	6,905	7,119
218690_at	8,362	7,899	8,305	8,373	8,390	8,332
218691_s_at	6,382	6,241	6,574	9,158	8,124	8,822
218692_at	6,229	6,265	6,744	5,269	6,836	5,592
218693_at	5,724	5,927	5,724	5,526	5,148	5,604
218694_at	11,529	12,181	11,836	12,088	11,920	12,004
218695_at	9,516	8,933	9,608	10,145	9,241	10,125
218696_at	9,348	9,743	9,321	7,510	9,146	8,031
218697_at	8,796	8,895	9,139	8,456	8,041	8,485
218698_at	10,892	11,557	11,075	11,020	10,472	10,920
218699_at	10,062	10,619	10,152	10,121	10,032	9,767
218700_s_at	6,540	7,517	6,515	6,301	5,353	5,562
218701_at	9,540	9,951	9,257	8,135	8,577	9,669
218702_at	8,090	8,254	8,442	8,548	7,763	8,084
218703_at	8,236	8,974	8,394	8,928	8,412	8,586
218704_at	7,617	7,568	7,492	7,956	7,690	8,029
218705_s_at	8,686	9,042	8,646	8,041	8,207	8,195
218706_s_at	6,044	6,336	6,529	7,497	8,358	7,962
218707_at	8,398	8,219	8,790	8,922	8,839	8,814
218708_at	9,687	9,742	10,034	10,366	9,397	9,899
218709_s_at	10,202	10,407	10,063	8,902	8,985	9,183
218710_at	9,861	9,935	9,787	9,422	9,992	9,188
218711_s_at	5,564	5,779	5,540	6,419	6,366	6,388
218712_at	10,429	10,408	10,169	8,836	7,949	8,402
218713_at	10,007	10,336	9,536	7,820	8,459	6,790

218714_at	8,240	8,332	8,214	8,965	8,601	8,857
218715_at	10,924	10,959	10,694	9,905	10,104	9,657
218716_x_at	9,528	9,701	9,524	9,153	9,603	9,095
218717_s_at	8,730	8,735	8,910	8,854	9,530	11,194
218718_at	7,561	8,283	7,773	10,574	11,677	11,258
218719_s_at	9,253	9,231	9,148	8,728	8,103	8,339
218720_x_at	7,308	8,174	7,720	6,697	6,352	6,868
218721_s_at	9,048	9,302	8,834	8,764	9,382	8,755
218722_s_at	9,938	9,971	10,606	9,779	9,124	9,377
218723_s_at	5,584	6,039	6,110	5,862	8,445	7,357
218724_s_at	9,395	9,313	9,833	7,638	6,991	7,723
218725_at	9,774	9,199	9,602	9,420	9,285	9,498
218726_at	10,637	11,006	10,524	8,925	8,786	8,858
218727_at	7,915	7,589	8,311	7,778	7,415	7,904
218728_s_at	12,020	12,281	12,107	11,550	11,036	11,429
218729_at	7,969	8,411	7,607	8,925	10,471	9,467
218730_s_at	4,203	3,845	4,223	4,102	4,058	4,166
218731_s_at	7,121	6,960	7,497	7,687	7,663	7,672
218732_at	10,253	10,853	10,419	11,673	11,324	11,453
218733_at	8,417	7,738	7,936	5,890	5,651	5,702
218734_at	7,988	7,835	8,176	7,183	7,146	7,136
218735_s_at	10,202	9,920	10,443	8,418	8,982	8,049
218736_s_at	4,788	4,544	4,691	4,758	6,031	4,667
218737_at	9,024	8,659	8,423	7,203	7,711	6,314
218738_s_at	11,712	11,780	11,707	9,746	9,788	9,347
218739_at	8,903	8,774	9,474	10,380	9,552	9,417
218740_s_at	10,240	10,164	9,804	9,252	9,107	9,329
218741_at	9,375	9,219	9,477	9,515	8,421	9,010
218742_at	8,797	8,174	8,554	8,967	9,006	9,165
218743_at	8,454	8,202	8,622	8,970	8,529	8,934
218744_s_at	7,788	7,654	7,779	7,206	6,700	7,065
218745_x_at	9,241	9,282	9,458	9,234	8,747	9,314
218746_at	5,774	5,741	6,035	6,959	7,043	6,937
218747_s_at	6,576	6,410	6,569	7,535	7,747	7,918
218748_s_at	6,347	6,545	6,021	5,367	5,906	5,381
218749_s_at	7,377	7,344	7,334	7,621	7,428	7,683
218750_at	9,119	8,917	8,628	7,511	8,368	7,285
218751_s_at	8,644	8,410	8,316	7,534	8,095	7,682
218752_at	7,212	7,419	7,466	7,667	7,429	7,910
218753_at	9,783	9,645	9,941	9,254	8,795	9,377
218754_at	9,532	9,444	10,110	9,145	9,355	9,247
218755_at	11,365	11,643	11,410	9,956	9,504	9,388
218756_s_at	7,827	7,785	7,649	7,267	6,471	7,231
218757_s_at	12,286	12,494	11,729	8,586	9,538	7,408
218758_s_at	9,641	9,199	10,080	9,773	8,907	9,514
218759_at	7,037	7,145	7,144	6,380	6,271	6,358
218760_at	7,453	7,360	7,573	7,942	7,323	7,857
218761_at	10,128	10,215	10,351	8,380	8,857	8,567
218762_at	8,339	8,489	8,827	8,040	7,370	7,717
218763_at	10,281	9,989	9,915	9,532	9,314	9,318
218764_at	7,148	8,436	7,531	7,330	7,749	7,180
218765_at	8,329	8,382	8,620	8,610	8,902	9,271
218766_s_at	8,566	8,615	8,708	8,019	7,440	7,592
218767_at	9,447	9,312	9,546	9,402	8,341	8,772
218768_at	12,444	12,616	12,044	10,165	10,434	8,984
218769_s_at	9,087	9,804	9,195	7,941	8,428	8,337

218770_s_at	9,399	8,863	9,806	8,904	8,006	8,663
218771_at	8,595	8,673	8,570	8,328	7,779	8,295
218772_x_at	9,474	9,991	9,602	9,799	9,122	9,541
218773_s_at	9,636	9,838	9,615	9,157	8,284	9,257
218774_at	8,987	9,072	9,077	9,597	8,870	9,489
218775_s_at	8,204	8,124	7,946	6,842	7,581	6,250
218776_s_at	7,632	7,683	7,612	9,109	8,438	8,716
218777_at	9,373	9,409	9,534	9,367	8,990	9,200
218778_x_at	7,291	7,302	7,282	8,040	8,157	8,049
218779_x_at	7,445	7,313	7,395	7,146	6,902	7,222
218780_at	7,821	7,986	7,907	7,351	7,641	7,868
218781_at	10,592	10,772	10,268	8,231	9,207	7,427
218782_s_at	8,408	8,508	7,870	7,239	8,093	6,036
218783_at	10,319	10,282	10,357	7,880	8,189	7,719
218784_s_at	7,957	7,967	7,986	8,103	7,917	8,013
218785_s_at	7,265	6,754	7,168	7,191	6,548	7,417
218786_at	8,243	8,135	8,561	7,298	7,691	7,135
218787_x_at	8,392	8,171	8,309	8,425	7,960	8,514
218788_s_at	10,188	10,369	10,082	9,705	9,027	9,402
218789_s_at	7,551	7,498	7,954	6,715	6,444	6,464
218790_s_at	5,809	5,942	5,887	6,371	6,106	6,399
218791_s_at	7,464	8,260	7,260	6,439	6,615	6,735
218792_s_at	6,473	6,979	6,875	6,556	6,485	6,373
218793_s_at	8,386	8,960	7,886	8,294	9,906	8,526
218794_s_at	8,368	8,179	8,630	8,368	7,722	7,863
218795_at	9,252	9,809	9,688	8,724	8,707	9,006
218796_at	9,056	8,730	9,219	8,580	8,064	8,942
218797_s_at	8,851	8,506	8,675	8,977	8,428	9,145
218798_at	8,645	8,814	8,976	8,481	8,189	8,420
218799_at	8,639	8,387	8,817	9,086	8,424	9,024
218800_at	6,711	5,947	6,617	5,691	5,341	5,637
218801_at	8,337	7,966	7,916	8,938	9,361	8,360
218802_at	10,392	10,323	10,403	10,426	10,209	10,531
218803_at	9,714	9,586	10,103	9,246	9,698	9,830
218804_at	4,452	5,414	4,573	3,962	3,943	3,954
218805_at	7,598	7,298	7,535	8,121	7,628	8,089
218806_s_at	5,909	6,736	6,173	5,666	5,257	5,384
218807_at	8,531	8,737	8,663	7,060	6,458	6,616
218808_at	6,685	6,616	6,819	8,065	8,055	8,313
218809_at	11,612	11,579	11,382	11,085	10,717	10,541
218810_at	7,426	7,550	7,602	9,143	7,860	7,743
218811_at	7,233	7,294	7,008	7,869	7,366	7,793
218812_s_at	7,195	6,639	6,792	7,902	7,177	7,666
218813_s_at	7,732	7,837	7,314	7,347	7,184	7,432
218814_s_at	7,549	7,474	7,449	7,880	7,540	7,963
218815_s_at	7,458	7,891	7,955	7,789	7,690	7,746
218816_at	8,003	8,726	8,214	7,062	7,219	7,385
218817_at	8,423	8,501	8,476	9,446	9,213	9,687
218818_at	8,118	7,831	8,220	8,154	8,088	8,227
218819_at	9,942	9,751	9,715	8,573	9,507	8,436
218820_at	10,844	10,437	11,181	8,837	8,950	8,344
218821_at	8,110	8,070	7,846	7,506	7,243	7,655
218822_s_at	7,864	7,886	7,887	8,021	7,748	8,200
218823_s_at	9,285	9,498	8,977	9,100	10,504	9,158
218824_at	11,128	11,432	11,311	6,203	6,951	6,766
218825_at	7,659	7,676	7,861	7,942	7,820	7,945

218826_at	8,922	8,943	9,565	9,452	9,099	8,807
218827_s_at	8,647	9,060	8,327	7,241	8,228	6,440
218828_at	8,818	8,462	8,831	9,545	9,413	9,986
218829_s_at	10,382	10,185	10,449	5,600	6,033	5,454
218830_at	11,126	11,605	11,505	12,140	11,698	12,090
218831_s_at	7,516	7,563	7,614	7,851	7,511	7,660
218832_x_at	6,429	6,900	6,619	6,691	6,444	6,453
218833_at	7,475	6,898	7,214	7,453	7,135	6,647
218834_s_at	8,656	8,722	9,024	8,662	8,321	8,274
218835_at	5,775	5,887	5,522	6,944	6,819	6,848
218836_at	8,381	8,415	8,716	8,231	7,857	8,335
218837_s_at	8,519	8,730	8,704	8,764	8,301	8,752
218838_s_at	9,376	9,475	9,654	8,997	8,506	9,001
218839_at	7,382	7,604	7,542	8,166	9,947	8,911
218840_s_at	8,005	8,273	8,335	8,954	9,100	9,346
218841_at	6,539	6,476	6,964	8,317	7,542	7,894
218842_at	9,908	10,177	8,867	7,960	8,991	6,781
218843_at	8,632	8,735	8,813	8,864	8,672	8,980
218844_at	7,776	8,223	7,450	8,036	7,778	8,202
218845_at	10,297	10,679	10,499	10,021	10,094	9,966
218846_at	9,066	9,419	8,674	7,874	9,173	8,140
218847_at	11,225	11,383	11,469	9,670	10,193	10,137
218848_at	8,524	8,219	8,147	7,118	6,926	6,973
218849_s_at	7,555	8,093	8,033	7,437	7,143	7,352
218850_s_at	6,917	6,778	6,759	6,571	6,575	6,621
218851_s_at	7,910	8,933	7,942	6,216	6,137	5,937
218852_at	10,613	10,911	10,148	8,963	10,075	9,322
218853_s_at	9,195	9,577	8,947	9,136	9,567	9,664
218854_at	10,170	10,502	10,021	10,563	10,632	10,570
218855_at	8,440	8,366	8,133	8,631	7,964	8,466
218856_at	10,786	10,774	11,452	8,926	8,509	9,373
218857_s_at	10,775	11,154	11,092	8,366	7,938	8,309
218858_at	7,058	8,087	8,038	6,064	6,150	6,328
218859_s_at	10,703	11,146	10,176	8,773	9,559	7,659
218860_at	9,039	8,916	9,322	8,727	7,977	8,380
218861_at	7,796	7,398	7,480	7,913	7,528	7,794
218862_at	9,951	9,682	10,187	9,679	8,990	9,194
218863_s_at	6,135	6,008	5,983	6,317	6,118	6,335
218864_at	6,514	6,293	6,491	7,053	6,864	7,217
218865_at	11,122	10,905	11,235	8,679	8,562	9,132
218866_s_at	9,766	9,545	9,668	9,300	8,088	8,638
218867_s_at	9,367	9,404	9,579	9,586	9,244	9,178
218868_at	10,012	9,969	9,761	7,169	7,046	7,090
218869_at	8,446	8,462	8,513	7,861	7,781	8,118
218870_at	6,588	6,517	6,320	5,992	5,753	5,667
218871_x_at	9,412	9,502	9,399	10,724	12,066	11,018
218872_at	7,909	8,128	7,627	7,154	6,926	7,054
218873_at	10,149	10,311	10,470	8,170	8,514	8,324
218874_s_at	8,131	7,747	8,027	8,038	8,319	8,400
218875_s_at	10,625	10,946	10,349	8,173	8,209	7,504
218876_at	7,032	6,937	6,655	6,901	6,768	6,950
218877_s_at	10,347	10,931	9,958	8,425	9,121	8,269
218878_s_at	10,805	11,052	10,928	7,698	8,736	7,455
218879_s_at	7,261	7,219	7,253	6,723	6,567	6,890
218880_at	6,069	6,076	5,540	7,849	6,796	7,425
218881_s_at	6,839	6,760	6,992	7,673	7,521	7,576

218882_s_at	11,795	11,610	11,527	10,133	10,291	9,416
218883_s_at	11,223	11,526	10,777	8,872	9,906	8,000
218884_s_at	7,506	7,883	7,228	7,924	7,831	7,318
218885_s_at	11,217	11,580	10,769	7,324	6,973	5,864
218886_at	9,963	9,684	9,345	9,512	9,062	9,410
218887_at	8,007	8,112	8,117	8,324	7,567	8,285
218888_s_at	11,630	10,998	11,770	9,436	11,216	11,313
218889_at	10,516	10,866	10,253	9,913	10,414	8,956
218890_x_at	9,133	9,156	8,871	9,781	9,201	10,077
218891_at	8,990	8,869	9,260	8,903	8,887	8,686
218892_at	6,853	6,757	6,842	7,742	7,375	7,569
218893_at	7,966	7,969	7,917	8,990	8,108	8,559
218894_s_at	9,914	9,520	9,711	9,153	8,238	8,813
218895_at	8,977	9,046	8,921	8,542	7,947	8,285
218896_s_at	10,337	10,208	10,267	8,472	9,017	8,334
218897_at	10,103	9,896	10,123	9,342	7,730	8,501
218898_at	9,317	9,015	9,252	10,535	9,735	10,807
218899_s_at	5,615	5,968	5,349	7,515	9,415	10,057
218900_at	8,851	9,444	9,242	8,077	7,644	8,164
218901_at	6,812	7,899	6,886	6,717	8,746	8,470
218902_at	9,374	9,346	9,642	8,339	8,291	8,532
218903_s_at	8,476	8,116	8,333	9,024	8,503	8,888
218904_s_at	9,780	9,819	9,495	8,402	7,618	8,030
218905_at	10,917	11,439	10,827	9,384	10,026	9,068
218906_x_at	8,985	8,697	9,154	8,960	8,616	9,076
218907_s_at	9,176	8,847	9,247	9,035	8,940	8,953
218908_at	7,692	7,495	7,330	7,288	7,042	7,450
218909_at	10,441	10,637	10,102	8,126	9,411	8,456
218910_at	9,429	9,346	9,289	9,694	9,538	9,826
218911_at	9,889	9,836	9,378	8,085	7,349	7,889
218912_at	8,924	8,548	8,745	8,307	8,165	8,297
218913_s_at	7,791	7,529	8,037	8,041	7,884	8,339
218914_at	7,727	7,999	7,734	7,613	7,513	7,573
218915_at	9,165	9,121	9,529	9,599	9,686	9,900
218916_at	8,238	8,641	8,615	8,243	7,915	8,062
218917_s_at	11,991	11,609	12,244	8,364	9,711	8,966
218918_at	9,098	9,524	9,555	6,861	6,869	6,885
218919_at	9,178	9,640	8,973	8,746	9,506	9,451
218920_at	9,108	8,404	8,856	8,679	8,384	8,516
218921_at	7,284	7,605	7,865	7,534	7,381	7,569
218922_s_at	7,499	7,788	7,610	7,516	7,650	7,849
218923_at	8,069	9,002	7,237	6,929	8,218	7,550
218924_s_at	8,556	9,348	8,224	7,957	8,257	8,292
218925_s_at	4,456	4,357	4,655	4,467	4,438	4,477
218926_at	9,428	9,734	9,513	7,904	8,496	8,082
218927_s_at	9,188	9,178	9,426	9,333	9,201	9,443
218928_s_at	8,617	8,201	8,260	7,656	7,275	7,405
218929_at	8,453	8,886	8,500	6,663	6,550	6,684
218930_s_at	10,022	10,166	9,951	9,455	10,072	10,080
218931_at	6,944	7,167	7,191	7,469	7,356	7,156
218932_at	9,941	10,105	9,704	8,954	9,662	9,053
218933_at	9,076	9,039	9,113	9,157	9,168	9,182
218934_s_at	6,023	6,036	6,122	6,237	7,764	7,737
218935_at	9,217	9,189	9,462	8,708	7,778	8,637
218936_s_at	10,301	10,904	10,250	9,619	9,846	9,444
218937_at	8,851	8,834	9,053	7,399	7,107	7,224

218938_at	7,616	7,835	7,590	8,062	7,457	8,052
218939_at	8,742	8,413	8,971	9,221	8,696	9,238
218940_at	9,219	9,385	9,185	7,636	8,138	8,229
218941_at	8,822	8,389	8,734	8,612	8,612	8,485
218942_at	10,441	10,294	10,658	9,387	9,508	9,925
218943_s_at	6,839	7,328	6,596	7,208	7,783	6,620
218944_at	7,499	7,291	7,579	8,153	7,758	8,347
218945_at	9,446	9,761	9,656	9,865	8,899	10,020
218946_at	10,317	10,878	10,383	10,378	9,954	10,192
218947_s_at	9,064	9,101	8,862	7,817	7,497	7,321
218948_at	5,882	5,681	5,746	5,519	5,985	5,316
218949_s_at	8,724	8,909	8,723	7,635	7,041	7,237
218950_at	9,159	9,184	9,565	8,399	7,826	8,126
218951_s_at	11,223	11,346	11,431	9,205	8,627	8,746
218952_at	7,525	7,369	7,713	7,230	7,059	7,269
218953_s_at	8,708	9,054	8,743	8,974	8,486	8,645
218954_s_at	8,215	8,343	8,358	8,141	7,829	8,314
218955_at	9,143	9,164	9,502	8,987	8,684	9,226
218956_s_at	9,615	9,522	9,849	9,629	9,454	9,731
218957_s_at	9,719	10,179	9,874	9,654	8,843	9,437
218958_at	8,392	8,319	8,325	8,135	7,578	8,184
218959_at	6,770	6,940	7,006	9,037	8,815	9,222
218960_at	6,973	6,817	6,857	7,431	7,484	7,402
218961_s_at	8,334	8,461	8,320	9,298	8,953	9,512
218962_s_at	9,524	9,656	9,030	8,483	9,601	8,571
218963_s_at	5,752	5,474	5,518	7,167	7,269	7,060
218964_at	10,206	10,279	10,836	7,465	7,508	7,346
218965_s_at	8,221	8,160	8,337	8,861	9,009	9,191
218966_at	8,912	8,857	8,812	6,684	6,712	6,747
218967_s_at	8,407	8,361	8,080	7,854	7,631	6,833
218968_s_at	10,517	10,451	10,899	9,405	8,933	9,406
218969_at	8,253	8,382	8,594	8,551	8,136	8,734
218970_s_at	8,951	9,259	8,626	8,748	8,305	8,518
218971_s_at	8,605	8,822	8,778	8,790	8,534	8,810
218972_at	9,075	9,655	9,514	8,287	8,544	8,220
218973_at	9,852	10,120	9,751	9,097	9,322	8,655
218974_at	9,755	9,350	9,890	6,951	6,853	6,735
218975_at	7,857	8,123	8,056	7,775	8,169	8,344
218976_at	6,579	7,108	6,513	6,823	6,667	7,367
218977_s_at	8,826	9,132	9,085	8,815	8,010	8,612
218978_s_at	7,112	7,157	6,542	7,123	7,039	6,958
218979_at	10,776	10,542	10,290	7,983	8,412	7,637
218980_at	8,733	8,182	8,106	7,972	6,677	6,919
218981_at	8,224	8,162	7,536	8,740	8,255	8,831
218982_s_at	10,791	11,065	10,766	10,919	10,009	10,387
218983_at	6,509	6,954	5,838	6,165	6,148	6,589
218984_at	10,596	9,994	9,924	8,454	8,961	7,775
218985_at	8,282	8,031	8,314	8,007	8,359	7,929
218986_s_at	5,658	6,944	4,889	4,736	5,950	4,562
218987_at	9,938	10,147	9,899	7,479	8,295	7,652
218988_at	10,130	10,049	10,330	10,113	10,208	10,128
218989_x_at	9,499	9,992	9,208	10,097	10,449	10,148
218990_s_at	6,447	6,282	6,467	6,783	6,716	6,539
218991_at	8,786	9,082	9,116	9,227	9,313	9,243
218992_at	9,383	10,082	9,580	9,416	8,431	9,456
218993_at	9,637	9,500	9,885	10,125	9,473	9,813

218994_s_at	7,017	7,270	6,927	6,178	6,090	6,360
218995_s_at	6,507	7,006	7,085	7,414	7,234	8,004
218996_at	8,134	7,690	8,147	9,218	7,748	8,644
218997_at	9,024	9,099	9,195	9,085	8,878	9,201
218998_at	9,676	9,910	9,841	9,754	9,865	9,526
218999_at	6,332	6,029	6,418	7,129	7,026	7,276
219000_s_at	10,037	10,157	9,765	8,473	7,903	8,144
219001_s_at	8,022	8,016	7,735	6,981	7,602	7,177
219002_at	11,298	11,741	11,030	9,498	9,399	9,248
219003_s_at	6,646	6,906	6,129	7,964	7,594	7,874
219004_s_at	11,285	11,410	10,861	9,646	8,335	8,419
219005_at	7,412	7,101	7,266	7,365	7,360	7,352
219006_at	10,185	10,331	10,014	11,438	10,733	10,852
219007_at	10,817	10,945	11,143	9,632	9,690	9,778
219008_at	10,005	10,422	9,688	8,464	8,773	8,054
219009_at	6,384	6,502	6,786	6,298	5,929	5,707
219010_at	10,435	10,268	10,961	6,835	6,537	6,824
219011_at	7,320	7,465	7,428	8,056	7,334	7,492
219012_s_at	8,332	8,212	8,200	6,445	6,360	6,476
219013_at	11,273	11,750	11,171	10,400	10,460	10,577
219014_at	6,162	6,148	6,288	5,999	5,787	6,248
219015_s_at	6,976	6,775	6,613	7,473	6,675	7,185
219016_at	9,965	9,800	9,923	8,895	8,946	8,716
219017_at	11,140	11,440	10,986	9,856	10,523	10,045
219018_s_at	7,099	7,249	7,527	7,310	7,213	7,269
219019_at	8,296	8,340	8,426	8,017	8,048	8,267
219020_at	9,071	9,349	9,097	9,775	9,045	9,484
219021_at	8,674	8,404	8,853	7,849	7,317	8,222
219022_at	9,529	9,345	9,729	9,323	8,360	9,042
219023_at	8,871	8,950	8,272	7,482	6,901	7,361
219024_at	8,736	9,340	8,616	7,480	8,139	7,825
219025_at	7,621	7,869	7,850	8,634	10,368	10,470
219026_s_at	6,833	6,619	7,208	7,059	7,235	6,681
219027_s_at	9,429	9,639	9,396	9,057	9,790	8,823
219028_at	7,438	7,252	7,117	8,170	9,261	8,484
219029_at	6,967	7,415	7,031	7,685	8,849	8,697
219030_at	10,786	10,813	10,578	10,168	9,709	9,784
219031_s_at	10,773	10,326	10,360	10,582	9,645	10,089
219032_x_at	12,472	12,429	12,404	10,785	10,497	10,935
219033_at	7,740	8,205	7,633	7,378	7,309	7,084
219034_at	8,626	8,494	8,761	8,485	8,043	8,365
219035_s_at	10,885	10,757	11,115	9,646	9,395	9,627
219036_at	9,708	10,337	8,995	7,213	7,504	6,580
219037_at	10,443	10,231	10,159	9,672	10,022	9,180
219038_at	6,925	7,585	6,555	7,309	8,688	8,133
219039_at	8,862	8,947	9,003	8,276	7,846	8,054
219040_at	7,783	7,717	7,546	7,673	7,357	7,497
219041_s_at	11,081	10,957	11,553	10,299	9,975	10,106
219042_at	7,605	7,263	7,807	7,295	6,982	7,531
219043_s_at	10,099	10,309	10,144	9,676	8,526	9,353
219044_at	7,556	7,407	7,421	7,502	7,547	7,673
219045_at	7,620	7,635	7,807	7,101	7,012	7,347
219046_s_at	8,237	8,334	8,211	8,212	8,171	7,936
219047_s_at	7,729	7,745	8,108	7,914	7,272	7,730
219048_at	8,674	9,363	9,037	7,265	8,326	7,530
219049_at	5,060	5,202	5,799	5,743	7,481	5,769

219050_s_at	7,267	7,037	7,168	7,041	6,326	6,749
219051_x_at	6,707	6,946	6,474	7,534	6,882	7,190
219052_at	8,512	8,263	8,484	8,741	8,379	8,887
219053_s_at	10,814	10,884	11,316	9,941	9,993	10,053
219054_at	6,793	7,269	7,245	8,771	10,935	10,124
219055_at	9,294	9,574	8,717	7,400	7,970	6,451
219056_at	9,146	9,237	9,396	7,534	7,177	6,990
219057_at	7,581	7,515	7,787	7,811	7,560	7,765
219058_x_at	7,629	8,174	8,009	8,150	8,309	8,435
219059_s_at	6,901	6,920	6,802	7,517	7,278	7,403
219060_at	9,731	10,348	9,557	9,871	8,931	9,430
219061_s_at	9,916	10,009	10,151	9,361	8,684	9,455
219062_s_at	8,551	8,685	8,454	7,550	8,777	7,287
219063_at	9,433	9,346	9,455	8,834	8,730	8,902
219064_at	6,813	6,513	7,023	7,081	6,888	6,751
219065_s_at	10,510	10,574	10,569	10,666	10,713	10,850
219066_at	8,601	8,686	8,891	8,553	7,785	8,384
219067_s_at	9,605	9,942	9,389	9,335	8,694	8,628
219068_x_at	9,039	9,005	9,245	9,406	9,398	9,212
219069_at	9,098	9,343	9,220	7,845	7,952	8,201
219070_s_at	8,652	8,452	8,517	9,240	8,579	8,933
219071_x_at	9,464	9,107	9,753	9,779	9,150	9,700
219072_at	7,969	8,037	7,913	8,677	7,778	8,516
219073_s_at	10,142	10,094	10,308	8,559	9,216	8,546
219074_at	8,804	8,887	9,369	9,547	9,525	9,662
219075_at	6,745	6,888	6,945	7,712	7,731	7,873
219076_s_at	9,705	9,576	10,192	9,155	8,722	9,052
219077_s_at	9,025	9,486	9,548	6,916	6,894	7,058
219078_at	8,051	7,559	8,269	7,129	7,745	7,008
219079_at	7,802	8,166	7,705	7,825	8,131	8,002
219080_s_at	9,091	9,171	9,164	8,170	7,680	7,962
219081_at	9,626	10,061	9,388	9,526	8,819	8,754
219082_at	7,326	7,251	7,481	8,530	8,062	8,557
219083_at	11,959	11,804	12,056	10,524	10,649	10,321
219084_at	8,172	8,223	8,281	7,541	7,778	7,603
219085_s_at	7,861	7,741	7,752	8,106	8,263	8,139
219086_at	8,288	7,960	8,241	7,152	7,382	7,200
219087_at	6,108	6,053	6,204	6,435	6,425	6,171
219088_s_at	7,710	7,344	7,718	8,043	7,497	8,095
219089_s_at	8,530	8,253	8,923	8,581	8,110	8,451
219090_at	6,004	6,173	6,201	6,648	6,473	6,711
219091_s_at	6,117	6,064	6,079	6,281	6,073	6,497
219092_s_at	8,836	8,640	8,958	8,638	8,637	8,655
219093_at	6,852	7,138	6,904	10,499	8,582	8,029
219094_at	9,145	9,426	8,584	6,135	7,277	5,987
219095_at	8,005	7,858	8,030	7,843	7,776	7,972
219096_at	8,561	8,753	8,553	8,794	8,836	8,997
219097_x_at	10,553	10,540	10,730	10,858	10,366	10,585
219098_at	8,529	8,101	8,604	8,866	8,846	9,029
219099_at	10,691	10,733	10,651	9,798	10,192	9,821
219100_at	8,098	8,932	8,357	9,346	9,151	9,219
219101_x_at	7,556	7,041	7,487	7,425	7,400	7,615
219102_at	7,513	7,822	7,507	10,124	9,530	10,652
219103_at	7,615	8,137	7,602	8,109	7,917	8,104
219104_at	8,368	8,389	7,959	8,435	8,055	8,840
219105_x_at	11,261	11,317	11,229	10,041	9,570	9,555

219106_s_at	4,058	4,752	3,905	3,804	3,682	3,679
219107_at	7,017	6,914	6,890	7,496	7,299	7,675
219108_x_at	10,957	10,796	11,362	10,581	10,740	10,654
219109_at	8,192	8,850	8,407	7,523	7,781	7,638
219110_at	10,993	10,763	10,846	10,742	10,631	10,483
219111_s_at	9,195	9,161	9,360	9,247	8,800	9,107
219112_at	8,992	9,263	8,784	7,910	9,149	7,725
219113_x_at	7,392	7,468	7,478	6,631	6,824	6,810
219114_at	8,667	8,658	8,704	8,117	7,839	8,161
219115_s_at	4,950	5,284	5,383	4,247	4,273	4,144
219116_s_at	9,149	9,021	8,712	8,407	8,710	8,587
219117_s_at	10,167	9,545	10,047	12,556	12,317	12,788
219118_at	7,726	7,349	7,350	9,652	9,325	9,857
219119_at	9,801	10,132	9,336	9,623	8,583	8,899
219120_at	9,353	8,861	8,971	7,604	7,238	7,217
219121_s_at	8,707	9,323	9,176	6,038	5,933	5,656
219122_s_at	8,454	8,549	8,753	9,332	8,584	8,963
219123_at	11,311	10,963	11,357	8,474	7,651	8,076
219124_at	9,125	9,177	9,482	8,675	7,672	8,302
219125_s_at	8,039	7,953	8,068	8,306	8,002	8,367
219126_at	9,069	8,898	9,442	8,147	7,849	7,747
219127_at	6,488	6,500	6,716	6,627	6,981	6,675
219128_at	8,456	8,457	8,779	8,326	8,252	8,389
219129_s_at	7,054	7,779	7,317	8,223	7,051	7,778
219130_at	9,834	10,001	9,327	7,745	8,543	7,206
219131_at	7,915	7,690	7,959	9,015	8,293	8,441
219132_at	9,783	9,967	9,667	6,174	6,122	5,930
219133_at	7,604	7,697	7,617	9,759	8,940	9,313
219134_at	5,058	4,824	4,343	10,267	10,607	9,250
219135_s_at	7,196	7,311	6,932	8,280	8,161	8,544
219136_s_at	7,605	7,854	7,887	6,951	7,107	7,407
219137_s_at	10,602	10,616	10,650	10,037	10,586	10,236
219138_at	8,610	8,927	9,104	8,813	8,875	8,688
219139_s_at	6,188	5,832	5,633	6,289	6,048	5,956
219140_s_at	6,239	6,589	6,709	8,497	7,126	8,961
219141_s_at	10,315	10,176	10,427	9,787	9,841	9,991
219142_at	9,071	9,450	9,721	7,071	7,041	7,018
219143_s_at	7,619	7,589	7,697	7,664	6,797	7,372
219144_at	7,632	7,737	7,831	7,720	7,734	7,991
219145_at	8,039	7,706	7,594	7,336	7,481	7,579
219146_at	9,340	9,592	9,397	9,687	9,898	10,155
219147_s_at	8,048	8,841	7,861	9,147	9,351	8,436
219148_at	10,055	10,779	9,745	10,346	10,684	10,102
219149_x_at	9,463	9,426	9,172	8,278	8,146	8,512
219150_s_at	7,723	7,565	7,723	7,386	7,097	7,126
219151_s_at	8,704	8,419	9,010	7,725	6,080	7,531
219152_at	7,021	6,472	6,517	5,805	5,938	6,192
219153_s_at	6,069	6,951	5,989	6,279	5,912	6,182
219154_at	8,497	8,564	8,512	7,432	7,349	7,424
219155_at	8,939	9,145	9,346	10,332	9,341	9,161
219156_at	8,562	8,411	8,035	7,394	8,036	7,900
219157_at	8,927	9,473	8,615	8,542	9,266	8,154
219158_s_at	10,376	10,127	9,602	9,408	9,650	8,462
219159_s_at	5,715	5,456	6,068	6,086	5,993	6,058
219160_s_at	5,486	5,957	5,369	4,876	4,677	4,593
219161_s_at	8,592	8,877	8,519	9,962	8,943	9,706

219162_s_at	10,286	9,999	10,262	9,522	8,588	9,313
219163_at	9,792	9,600	9,808	7,770	8,317	7,820
219164_s_at	7,350	7,814	7,226	7,109	7,734	6,247
219165_at	9,021	9,220	8,955	10,279	10,235	10,963
219166_at	7,249	7,265	7,017	6,410	5,836	6,061
219167_at	8,569	8,317	8,264	6,850	6,358	6,661
219168_s_at	7,592	7,763	7,322	8,003	7,371	7,929
219169_s_at	9,670	10,043	9,781	10,283	9,220	9,203
219170_at	9,569	9,531	9,064	8,204	7,992	8,164
219171_s_at	7,348	7,799	7,309	5,497	5,534	5,002
219172_at	7,709	7,679	7,907	8,721	7,864	8,530
219173_at	7,160	7,224	7,305	7,432	7,352	7,742
219174_at	8,739	9,497	8,374	6,776	7,931	6,200
219175_s_at	9,010	9,329	9,260	8,763	8,553	8,998
219176_at	10,964	11,149	11,093	11,019	10,656	11,021
219177_at	9,329	9,659	8,996	8,668	8,752	8,115
219178_at	9,335	8,893	9,081	7,418	7,633	6,987
219179_at	7,670	7,462	7,772	5,414	5,462	6,174
219180_s_at	8,016	7,962	8,395	8,360	7,939	7,855
219181_at	7,200	7,596	6,748	5,311	4,641	4,287
219182_at	8,549	8,500	8,519	8,739	8,214	8,455
219183_s_at	7,497	7,681	7,894	8,178	8,174	8,290
219184_x_at	8,812	8,557	9,115	8,895	8,877	8,933
219185_at	8,747	9,014	8,917	8,748	8,379	8,707
219186_at	7,291	6,940	7,261	6,962	6,943	7,156
219187_at	8,754	8,214	8,619	9,138	8,491	8,956
219188_s_at	7,935	8,446	7,750	7,879	7,328	8,060
219189_at	6,967	6,457	6,237	6,986	6,376	7,050
219190_s_at	6,965	7,728	7,202	6,996	7,220	7,272
219191_s_at	8,122	8,205	8,320	8,559	8,645	8,574
219192_at	10,902	10,901	11,388	10,226	10,444	10,294
219193_at	8,941	8,882	8,730	8,004	8,105	8,353
219194_at	7,022	7,013	7,024	7,320	6,978	6,953
219195_at	6,596	7,064	6,214	5,643	5,948	5,768
219196_at	8,095	7,976	7,867	3,823	3,835	3,887
219197_s_at	6,474	6,240	6,225	5,712	5,671	6,111
219198_at	9,199	9,297	9,357	8,606	8,237	8,485
219199_at	5,884	6,249	5,891	6,443	6,910	6,248
219200_at	9,749	9,706	9,611	9,664	9,330	9,210
219201_s_at	7,723	7,445	7,217	8,128	8,457	7,915
219202_at	8,620	8,566	8,754	8,565	8,313	8,543
219203_at	9,480	9,392	9,525	9,570	8,533	9,138
219204_s_at	6,807	7,291	6,774	6,863	6,055	6,242
219205_at	7,487	7,835	7,493	7,864	7,544	7,601
219206_x_at	10,500	10,197	9,837	9,773	10,794	10,566
219207_at	8,431	8,413	8,638	8,334	7,744	8,244
219208_at	10,318	10,134	10,380	6,592	7,196	6,588
219209_at	4,747	5,342	4,731	5,736	6,540	5,416
219210_s_at	8,140	8,037	7,922	8,517	8,726	8,363
219211_at	7,617	7,492	7,727	6,846	7,006	7,463
219212_at	11,154	11,589	11,183	10,390	10,325	9,952
219213_at	9,638	9,650	9,549	7,647	7,318	7,430
219214_s_at	8,530	8,477	8,567	8,785	8,391	8,749
219215_s_at	8,246	8,189	8,455	8,485	7,865	8,731
219216_at	9,086	9,986	8,733	7,152	7,940	7,211
219217_at	10,766	10,949	10,521	9,391	9,206	9,027

219218_at	7,220	7,260	7,088	7,541	7,748	7,845
219219_at	7,090	7,162	7,100	7,580	7,174	7,323
219220_x_at	10,972	11,280	11,109	10,987	10,808	10,862
219221_at	8,648	8,533	8,002	9,687	10,190	8,903
219222_at	7,622	7,938	7,942	8,679	7,911	8,147
219223_at	8,662	8,455	8,817	8,496	8,512	8,653
219224_x_at	9,717	9,721	9,912	9,308	9,280	9,410
219225_at	9,850	9,798	9,962	6,353	5,935	6,072
219226_at	8,673	8,147	8,606	8,242	8,030	8,183
219227_at	7,517	7,298	7,842	6,959	7,015	6,926
219228_at	8,500	8,413	8,415	7,782	9,257	7,688
219229_at	8,132	8,196	8,247	8,327	8,118	8,469
219230_at	7,231	7,491	6,799	8,881	9,618	9,352
219231_at	10,544	10,647	10,566	8,473	8,644	7,793
219232_s_at	7,468	7,641	7,897	5,482	5,101	5,269
219233_s_at	8,504	8,232	7,732	7,127	7,087	7,312
219234_x_at	6,698	7,232	6,570	4,559	5,316	5,077
219235_s_at	8,775	8,833	9,142	6,743	6,905	6,882
219236_at	8,587	8,810	8,265	7,761	7,493	7,833
219237_s_at	8,729	8,915	8,298	8,118	9,567	8,529
219238_at	9,797	10,015	10,010	9,399	8,804	9,412
219239_s_at	6,963	7,331	6,342	6,531	6,915	6,713
219240_s_at	8,480	8,610	8,312	7,052	7,094	7,540
219241_x_at	8,612	8,795	8,800	8,267	8,193	8,538
219242_at	9,367	9,819	9,126	7,619	8,340	7,173
219243_at	4,027	3,960	3,874	3,970	4,043	3,957
219244_s_at	10,151	10,482	10,645	10,431	9,432	9,928
219245_s_at	7,711	7,570	7,806	8,561	7,999	8,426
219246_s_at	7,141	6,767	6,802	7,534	7,184	7,449
219247_s_at	8,914	8,880	9,090	9,435	9,718	9,141
219248_at	6,125	6,530	6,146	8,472	8,524	7,963
219249_s_at	7,754	7,476	7,681	8,259	8,379	8,293
219250_s_at	9,731	9,571	8,820	8,457	8,376	8,167
219251_s_at	9,057	9,355	9,104	8,581	9,107	8,408
219252_s_at	7,287	7,819	7,638	7,257	6,678	6,866
219253_at	8,610	8,505	8,456	9,019	8,135	8,732
219254_at	7,381	7,421	7,297	7,655	7,219	7,739
219255_x_at	8,868	8,802	8,941	6,774	6,527	6,870
219256_s_at	6,678	6,150	6,412	6,837	6,543	7,300
219257_s_at	7,825	7,531	7,358	10,956	9,312	11,046
219258_at	10,126	10,151	10,043	9,667	9,231	9,370
219259_at	7,510	7,673	7,873	7,558	7,335	7,304
219260_s_at	8,178	7,651	7,890	9,052	9,019	7,479
219261_at	9,208	9,171	9,369	8,818	8,200	8,708
219262_at	4,908	4,703	4,240	4,125	3,948	3,977
219263_at	6,229	7,938	6,236	7,131	9,392	9,125
219264_s_at	7,308	7,425	7,338	6,259	6,012	5,947
219265_at	8,350	8,464	8,924	8,226	8,240	8,161
219266_at	7,511	7,441	7,494	7,187	7,628	7,509
219267_at	6,905	6,941	7,306	8,476	8,074	8,712
219268_at	7,420	7,816	7,651	7,362	7,329	7,273
219269_at	9,486	9,190	9,163	8,251	8,755	8,128
219270_at	7,700	8,128	8,595	7,798	7,450	8,267
219271_at	8,241	8,456	8,218	7,209	5,649	5,868
219272_at	7,678	7,820	7,958	7,743	7,756	7,998
219273_at	6,940	6,897	7,108	7,352	6,800	7,277

219274_at	9,241	9,813	9,323	6,070	6,546	6,586
219275_at	8,242	8,081	7,910	9,509	8,916	9,290
219276_x_at	10,479	10,594	10,348	8,006	8,547	8,244
219277_s_at	7,812	8,415	8,063	7,302	7,103	7,219
219278_at	7,015	7,272	7,047	8,042	7,835	8,281
219279_at	6,522	6,422	6,352	9,269	10,257	8,995
219280_at	8,213	8,603	7,900	6,235	6,534	6,404
219281_at	8,215	8,425	8,931	9,537	9,367	9,701
219282_s_at	7,450	6,883	6,647	10,787	10,183	11,205
219283_at	9,909	10,040	9,491	10,662	10,489	10,767
219284_at	8,915	9,065	8,828	7,440	7,207	6,821
219285_s_at	5,109	5,370	5,442	5,795	5,673	5,606
219286_s_at	12,221	12,179	12,418	9,687	10,024	9,212
219287_at	9,493	10,038	9,856	7,187	6,782	6,896
219288_at	10,239	10,447	10,211	9,732	9,879	10,296
219289_at	8,423	8,267	8,517	8,256	7,579	8,021
219290_x_at	7,409	6,992	7,389	7,084	7,608	6,825
219291_at	7,581	7,815	7,445	8,748	7,854	7,438
219292_at	9,378	9,663	9,447	8,075	8,056	8,376
219293_s_at	12,120	12,186	12,023	11,658	11,339	11,333
219294_at	8,866	9,173	8,420	6,908	7,384	6,113
219295_s_at	9,441	9,553	9,353	6,478	9,789	9,411
219296_at	8,458	8,872	8,431	7,223	7,040	7,285
219297_at	9,873	10,460	10,056	8,319	9,356	8,852
219298_at	9,348	9,875	9,818	7,737	7,430	7,621
219299_at	6,288	6,308	6,222	8,712	8,281	8,594
219300_s_at	8,343	8,524	8,816	5,492	5,584	5,622
219301_s_at	8,940	8,735	8,667	6,015	6,073	6,480
219302_s_at	7,863	8,031	7,990	4,941	4,769	4,805
219303_at	5,497	5,517	4,914	8,662	9,088	7,562
219304_s_at	7,337	7,637	7,280	6,054	6,437	6,190
219305_x_at	7,463	7,959	7,466	7,321	7,497	7,400
219306_at	10,806	10,835	9,989	6,521	7,487	5,132
219307_at	9,009	9,372	9,372	8,947	9,708	9,136
219308_s_at	6,429	6,744	6,840	7,774	7,330	8,846
219309_at	7,130	6,790	6,949	6,821	6,840	7,029
219310_at	8,010	7,942	8,186	7,666	6,827	6,951
219311_at	9,596	9,923	9,545	8,299	8,012	7,989
219312_s_at	7,801	7,796	7,623	4,091	4,712	4,064
219313_at	6,780	8,688	6,287	5,252	5,102	4,747
219314_s_at	9,222	9,164	9,309	8,914	8,800	8,898
219315_s_at	6,493	6,293	6,103	9,467	7,967	9,005
219316_s_at	7,275	7,748	7,658	8,693	8,742	8,022
219317_at	8,205	8,698	7,795	6,293	7,342	5,791
219318_x_at	6,612	6,886	6,872	7,365	6,848	7,261
219319_at	7,099	7,377	7,232	7,160	7,142	7,496
219320_at	8,754	8,374	8,773	8,784	8,224	8,688
219321_at	7,015	6,734	6,949	7,133	7,442	6,821
219322_s_at	9,013	9,024	9,040	9,265	8,703	9,075
219323_s_at	7,193	7,046	7,160	7,453	7,303	7,422
219324_at	7,781	7,305	7,713	8,864	8,312	8,671
219325_s_at	6,412	5,974	6,744	6,353	6,156	6,351
219326_s_at	7,955	7,531	7,385	6,647	6,250	6,741
219327_s_at	8,071	7,903	7,593	7,265	6,825	7,070
219328_at	9,446	9,494	9,378	8,839	8,568	8,544
219329_s_at	12,661	12,987	13,027	12,236	11,936	12,258

219330_at	9,409	9,523	9,856	8,760	8,180	8,362
219331_s_at	6,387	6,389	6,248	6,838	6,545	6,730
219332_at	8,050	7,565	7,853	8,226	8,294	8,771
219333_s_at	8,731	8,746	8,439	8,387	8,316	8,394
219334_s_at	5,881	6,015	5,543	6,407	6,390	6,639
219335_at	8,692	9,092	8,709	7,701	8,282	7,824
219336_s_at	9,382	9,838	9,405	9,542	9,538	9,412
219337_at	7,380	7,522	7,506	7,769	7,643	7,557
219338_s_at	9,245	9,785	8,714	7,645	7,702	7,400
219339_s_at	7,166	6,873	6,900	7,056	7,078	7,320
219340_s_at	7,791	7,850	7,819	8,108	7,769	8,346
219341_at	6,150	6,504	5,916	6,204	6,095	6,488
219342_at	9,585	9,632	9,178	6,532	7,426	6,467
219343_at	8,722	8,846	8,236	7,201	7,315	7,502
219344_at	8,239	8,501	8,602	7,991	7,728	8,116
219345_at	6,674	6,606	6,661	7,570	7,328	7,860
219346_at	6,885	6,891	7,466	6,223	5,934	6,576
219347_at	10,211	10,435	10,233	9,578	8,809	9,108
219348_at	7,649	7,496	7,614	8,911	8,881	9,266
219349_s_at	8,755	9,177	8,436	8,014	8,280	7,689
219350_s_at	11,308	11,260	11,358	10,962	10,312	10,982
219351_at	8,321	8,175	8,767	6,891	7,497	7,394
219352_at	6,938	7,728	7,297	6,536	6,404	6,505
219353_at	9,161	8,780	8,869	7,994	8,648	7,292
219354_at	8,258	8,042	8,102	7,272	7,185	7,143
219355_at	9,591	9,896	8,620	7,098	6,911	5,774
219356_s_at	10,892	11,325	10,603	10,710	10,756	11,129
219357_at	9,414	9,684	9,856	8,336	8,396	8,665
219358_s_at	7,519	8,017	7,820	7,944	7,854	7,851
219359_at	8,603	8,625	8,216	8,100	8,198	8,420
219360_s_at	7,464	7,306	7,623	7,921	7,815	8,191
219361_s_at	11,208	11,039	11,383	10,068	10,064	10,139
219362_at	9,775	9,966	9,430	8,463	8,854	8,414
219363_s_at	9,692	10,087	9,644	9,897	9,758	9,500
219364_at	6,972	6,873	6,828	7,161	7,203	7,094
219365_s_at	8,631	8,563	8,713	7,361	7,377	7,488
219366_at	8,849	8,984	9,206	9,654	9,467	9,774
219367_s_at	7,095	7,150	6,942	7,071	7,060	7,112
219368_at	9,610	9,551	8,747	4,287	5,056	4,458
219369_s_at	7,001	7,634	7,267	6,886	6,463	6,894
219370_at	8,080	8,464	8,620	7,042	6,572	6,769
219371_s_at	7,838	7,873	8,371	9,131	9,101	9,518
219372_at	10,737	10,479	9,753	6,918	8,505	6,488
219373_at	9,831	10,176	9,530	10,335	9,992	10,457
219374_s_at	8,407	8,165	8,502	9,138	8,320	8,900
219375_at	8,740	9,036	8,832	8,642	8,721	8,749
219376_at	7,719	7,998	7,297	5,890	5,585	5,559
219377_at	8,174	8,237	8,330	8,402	8,852	8,306
219378_at	11,084	10,794	10,498	7,945	8,907	7,393
219379_x_at	8,123	8,186	8,136	8,228	8,128	8,391
219380_x_at	5,420	5,230	5,721	8,008	8,436	8,389
219381_at	6,919	7,279	6,346	5,631	6,109	5,585
219382_at	8,227	8,592	8,320	8,113	7,486	7,776
219383_at	6,053	6,087	6,025	6,135	5,555	5,780
219384_s_at	8,776	8,556	8,659	7,935	8,025	8,024
219385_at	6,863	6,742	6,943	7,621	7,674	7,764

219386_s_at	4,627	4,668	4,751	6,053	6,113	6,094
219387_at	8,854	9,142	8,426	6,329	7,039	6,029
219388_at	5,919	5,995	6,195	6,275	6,139	6,120
219389_at	7,941	7,874	8,193	8,568	8,195	8,497
219390_at	7,888	8,590	7,809	8,757	9,545	8,866
219391_at	4,321	4,064	4,801	5,191	5,170	5,482
219392_x_at	11,532	11,180	11,227	10,497	11,300	10,510
219393_s_at	4,952	4,732	5,166	5,596	5,060	5,477
219394_at	9,223	8,948	9,222	9,097	8,858	8,732
219395_at	7,041	7,177	7,535	6,393	6,436	6,318
219396_s_at	7,505	7,509	7,601	7,140	7,018	7,011
219397_at	9,030	9,816	9,172	9,501	9,902	9,513
219398_at	7,510	7,466	7,501	8,046	8,275	9,166
219399_at	8,772	9,073	8,731	8,706	8,943	8,654
219400_at	8,878	8,991	8,852	8,886	8,912	8,912
219401_at	8,161	8,579	8,314	8,872	8,347	8,855
219402_s_at	8,956	8,793	9,131	11,287	10,480	11,082
219403_s_at	5,962	6,282	6,013	6,389	6,106	6,152
219404_at	6,572	6,292	5,951	6,881	6,624	6,730
219405_at	8,476	8,979	9,043	7,800	7,835	7,828
219406_at	8,025	8,335	8,540	8,771	8,355	8,884
219407_s_at	7,378	7,518	7,564	7,249	7,195	7,443
219408_at	9,092	8,906	9,111	8,654	7,974	8,480
219409_at	8,942	9,070	8,737	7,939	7,478	7,776
219410_at	8,788	9,314	8,677	10,412	10,549	11,445
219411_at	6,897	6,297	6,203	6,590	6,328	6,395
219412_at	8,792	9,629	9,209	10,278	8,533	8,268
219413_at	7,017	6,947	7,129	7,256	7,016	7,326
219414_at	5,531	5,497	6,055	6,013	5,904	5,974
219415_at	7,049	6,841	7,284	7,029	6,756	7,109
219416_at	7,708	7,738	7,731	8,642	9,196	9,238
219417_s_at	7,205	7,770	7,544	8,009	8,043	7,915
219418_at	8,896	9,110	9,202	8,596	8,091	8,377
219419_at	8,836	9,131	9,041	9,387	9,009	9,464
219420_s_at	10,733	10,545	10,851	9,550	8,743	9,150
219421_at	7,772	8,423	7,529	6,777	6,816	6,919
219422_at	7,418	7,511	7,737	8,379	8,281	8,486
219423_x_at	7,220	6,413	6,821	6,368	6,029	6,433
219424_at	6,466	6,356	6,116	7,085	6,912	7,003
219425_at	7,759	7,728	8,225	7,633	7,396	7,766
219426_at	8,878	8,728	8,510	6,333	7,296	6,930
219427_at	5,531	5,897	6,323	6,781	8,530	7,874
219428_s_at	7,339	7,413	7,554	9,061	8,400	8,854
219429_at	5,726	6,213	5,749	5,115	5,089	5,332
219430_at	5,251	5,489	5,128	6,520	6,106	6,523
219431_at	7,535	7,555	7,730	8,417	8,511	8,295
219432_at	7,718	7,904	7,935	8,547	8,645	8,678
219433_at	9,579	9,149	10,125	5,489	6,874	5,803
219434_at	6,250	6,144	5,981	6,722	6,712	6,668
219435_at	8,063	8,216	8,255	8,786	8,216	8,242
219436_s_at	5,254	5,676	5,506	6,418	6,252	6,309
219437_s_at	10,047	9,652	10,138	8,806	9,593	9,263
219438_at	8,248	7,886	8,265	6,600	6,496	6,530
219439_at	10,437	11,001	10,311	10,357	11,258	11,077
219440_at	7,295	6,877	7,173	6,126	6,642	6,033
219441_s_at	6,344	6,439	6,920	6,700	6,991	6,807

219442_at	8,289	7,998	8,256	7,711	7,716	7,834
219443_at	9,080	9,179	8,871	8,099	8,823	7,887
219444_at	8,809	8,656	8,931	9,019	9,016	9,214
219445_at	8,599	8,500	8,873	8,538	8,511	8,521
219446_at	9,414	9,264	9,046	7,980	8,349	7,888
219447_s_at	8,230	8,000	8,120	8,144	7,700	8,325
219448_at	7,961	7,997	8,234	7,181	6,904	7,417
219449_s_at	10,436	10,234	10,293	11,158	10,651	11,116
219450_at	5,059	5,626	5,581	5,286	5,243	5,555
219451_at	10,104	9,873	10,107	7,248	7,466	6,987
219452_at	6,627	6,528	6,451	6,996	6,954	7,023
219453_at	7,330	7,584	7,334	8,063	7,974	8,264
219454_at	8,485	8,755	7,900	5,641	5,351	5,577
219455_at	7,704	8,135	7,246	8,019	8,327	8,052
219456_s_at	5,894	6,183	6,044	6,822	6,494	6,853
219457_s_at	7,043	6,620	6,925	7,677	7,418	7,655
219458_s_at	8,690	8,989	8,652	8,825	8,635	8,444
219459_at	10,173	9,880	10,333	9,021	9,817	8,664
219460_s_at	9,142	8,912	9,503	9,210	9,660	9,947
219461_at	6,775	6,427	6,645	6,910	6,800	6,823
219462_at	8,786	9,060	8,870	9,094	8,369	8,951
219463_at	7,095	7,337	7,685	6,937	6,842	6,775
219464_at	11,104	11,097	11,476	8,161	7,757	8,046
219465_at	5,203	5,592	5,142	6,513	6,497	6,633
219466_s_at	5,232	5,232	5,608	6,299	6,397	6,338
219467_at	9,382	9,865	9,338	8,570	8,974	8,401
219468_s_at	6,867	6,723	6,493	7,364	7,080	7,811
219469_at	8,012	8,160	7,729	8,019	9,403	7,524
219470_x_at	9,468	8,757	8,984	6,370	6,488	6,176
219471_at	7,565	7,518	7,566	7,098	6,966	7,129
219472_at	8,915	8,392	8,836	8,942	8,757	8,847
219473_at	8,277	8,281	8,446	7,320	7,125	7,117
219474_at	10,431	10,572	10,922	9,036	8,466	9,026
219475_at	7,208	7,651	7,438	8,090	7,984	8,100
219476_at	5,385	5,451	5,398	5,929	6,060	6,157
219477_s_at	9,156	9,443	8,811	8,351	8,139	7,938
219478_at	6,460	6,643	6,666	6,811	6,689	6,924
219479_at	9,064	9,076	9,005	8,677	8,185	8,457
219480_at	7,444	7,565	7,629	7,916	8,495	8,500
219481_at	9,829	9,213	9,906	8,483	8,900	8,264
219482_at	9,269	8,979	9,435	6,969	7,158	6,819
219483_s_at	8,257	8,523	8,371	8,912	8,645	8,878
219484_at	6,986	7,406	6,490	7,940	8,479	7,831
219485_s_at	12,932	13,102	12,582	11,394	11,334	11,588
219486_at	9,169	9,294	9,453	9,185	8,872	9,219
219487_at	7,916	8,046	7,526	6,407	7,135	6,272
219488_at	7,875	7,740	7,829	8,345	8,325	8,441
219489_s_at	11,317	11,280	11,573	8,544	8,250	10,223
219490_s_at	7,882	7,762	7,818	8,514	7,773	8,252
219491_at	8,124	8,183	8,326	8,223	8,028	8,375
219492_at	7,479	7,327	7,141	8,059	7,639	8,227
219493_at	8,594	8,474	8,029	9,173	9,269	7,855
219494_at	9,250	9,555	9,239	7,135	7,296	6,350
219495_s_at	9,081	8,934	8,815	6,377	6,777	5,975
219496_at	8,031	8,778	8,173	7,529	7,375	7,718
219497_s_at	9,375	8,950	9,552	5,067	5,148	5,352

219498_s_at	9,539	8,131	9,743	7,089	6,914	7,307
219499_at	9,547	9,531	9,471	8,140	7,873	7,646
219500_at	8,162	8,042	8,107	8,813	8,560	8,973
219501_at	8,208	8,013	7,807	8,150	7,672	7,680
219502_at	8,339	8,712	7,831	7,349	7,092	6,706
219503_s_at	6,093	5,867	5,749	7,283	7,359	7,455
219504_s_at	8,258	8,189	8,019	7,074	7,224	7,068
219505_at	10,099	10,195	10,904	7,390	8,426	7,510
219506_at	8,553	8,939	8,090	8,718	10,247	10,034
219507_at	8,178	8,537	8,318	7,900	7,336	7,282
219508_at	6,436	6,440	6,469	7,728	7,405	7,103
219509_at	6,987	6,648	6,793	6,677	7,011	6,996
219510_at	10,466	10,628	10,080	8,784	9,368	8,222
219511_s_at	8,495	8,796	8,527	7,702	7,646	7,609
219512_at	8,262	8,538	7,796	7,888	7,263	7,192
219513_s_at	5,835	6,027	5,888	5,824	5,795	5,899
219514_at	6,976	6,793	7,187	7,270	7,153	7,279
219515_at	8,627	8,575	8,676	6,760	7,670	6,693
219516_at	6,785	6,995	6,770	7,733	7,887	7,979
219517_at	7,255	7,479	7,358	7,644	7,187	7,462
219518_s_at	6,957	7,857	7,644	6,723	6,552	6,851
219519_s_at	6,417	6,243	6,378	6,999	6,987	6,960
219520_s_at	9,758	9,600	9,934	7,984	8,252	8,316
219521_at	8,845	8,986	9,194	7,916	7,897	7,992
219522_at	10,335	10,049	10,689	9,841	10,071	10,153
219523_s_at	8,713	8,511	8,555	7,338	7,651	8,230
219524_s_at	5,531	5,271	5,432	6,122	6,007	5,918
219525_at	7,987	8,510	8,544	7,552	7,523	6,860
219526_at	8,943	8,749	9,385	8,690	8,166	8,493
219527_at	7,902	8,506	8,225	8,499	8,214	8,367
219528_s_at	6,095	6,247	6,077	5,410	5,135	5,411
219529_at	5,327	5,772	5,366	6,197	6,003	6,580
219530_at	11,768	11,874	11,670	9,076	8,887	8,246
219531_at	9,091	9,447	9,446	7,986	7,735	7,844
219532_at	8,054	8,484	7,904	6,999	7,165	6,530
219533_at	6,360	6,463	6,007	6,246	6,367	6,369
219534_x_at	8,240	9,196	8,156	6,565	6,069	6,722
219535_at	6,426	6,941	6,542	7,519	7,478	7,451
219536_s_at	8,239	8,144	8,111	8,053	7,584	8,607
219537_x_at	6,886	6,885	6,745	6,933	6,438	6,784
219538_at	7,448	7,190	6,997	7,641	7,046	7,247
219539_at	9,780	9,721	9,649	10,758	9,532	10,028
219540_at	9,545	9,675	9,177	8,124	8,963	6,525
219541_at	7,342	7,276	7,227	7,625	7,497	8,048
219542_at	7,270	6,957	7,291	7,703	7,953	8,013
219543_at	6,194	6,585	6,111	5,275	7,298	7,265
219544_at	9,115	9,572	9,036	7,504	7,442	7,185
219545_at	8,047	7,876	8,044	7,720	6,816	7,628
219546_at	8,676	8,815	8,756	9,354	9,619	9,025
219547_at	10,052	10,365	10,352	9,127	9,314	9,217
219548_at	8,421	8,703	8,994	8,320	8,013	8,256
219549_s_at	11,555	11,207	11,267	9,063	9,655	9,848
219550_at	8,104	8,423	8,069	7,986	8,076	8,450
219551_at	6,957	7,568	6,729	7,358	7,025	7,411
219552_at	4,553	4,594	5,138	5,886	6,618	6,061
219553_at	11,148	11,445	10,879	10,282	10,832	10,590

219554_at	6,429	6,343	6,181	7,387	7,231	7,235
219555_s_at	10,805	10,776	10,671	9,929	8,899	9,089
219556_at	9,447	9,122	9,676	8,424	8,090	8,321
219557_s_at	9,227	9,488	9,480	9,107	8,114	8,115
219558_at	8,345	8,293	7,748	8,559	8,195	8,090
219559_at	7,979	7,631	7,856	10,074	9,510	9,831
219560_at	7,305	7,098	7,203	7,934	7,515	7,997
219561_at	8,054	7,790	8,288	9,859	10,274	11,095
219562_at	7,750	7,904	7,633	7,620	7,558	7,848
219563_at	5,844	6,110	6,315	8,958	10,675	9,968
219564_at	5,071	4,880	4,857	4,908	5,001	5,008
219565_at	8,481	8,212	8,444	8,562	8,704	8,797
219566_at	7,723	7,933	7,821	8,704	8,316	8,370
219567_s_at	5,664	4,938	5,555	5,030	4,968	4,965
219568_x_at	4,566	4,790	4,461	4,388	4,341	4,307
219569_s_at	8,071	8,549	8,014	9,609	8,935	9,891
219570_at	6,843	7,458	6,988	6,462	7,881	6,658
219571_s_at	9,457	9,613	9,193	5,911	7,426	5,896
219572_at	8,766	8,709	8,549	7,938	8,518	7,581
219573_at	7,994	8,215	8,331	7,380	8,032	7,487
219574_at	5,627	6,170	5,253	4,344	4,276	4,332
219575_s_at	9,895	9,920	10,522	10,138	9,562	10,006
219576_at	7,357	7,744	7,446	7,852	7,733	7,625
219577_s_at	7,208	7,575	7,493	7,757	7,829	8,017
219578_s_at	9,120	9,883	9,579	7,096	6,745	6,300
219579_at	9,371	9,067	9,269	9,415	9,657	9,708
219580_s_at	6,955	6,393	6,924	4,119	4,181	4,104
219581_at	9,445	8,948	9,328	8,799	8,229	7,942
219582_at	8,611	8,490	8,343	10,172	9,574	10,422
219583_s_at	7,476	8,333	7,408	6,565	7,228	6,599
219584_at	5,396	5,305	5,636	5,990	6,005	6,179
219585_at	6,662	6,539	6,407	7,387	7,040	7,265
219586_at	6,171	6,912	6,034	5,662	5,636	5,649
219587_at	8,064	8,062	8,344	8,174	8,071	7,890
219588_s_at	10,572	10,591	10,123	8,424	8,961	7,762
219589_s_at	8,227	7,796	7,792	8,038	8,141	8,098
219590_x_at	10,997	11,214	10,906	11,034	10,305	10,600
219591_at	8,132	7,985	8,099	9,050	8,379	8,778
219592_at	6,093	5,985	5,971	6,285	6,452	6,588
219593_at	8,616	8,579	8,415	7,511	7,092	7,498
219594_at	6,920	6,934	7,336	7,380	7,551	7,851
219595_at	9,991	9,948	9,808	7,068	7,914	7,011
219596_at	9,259	9,419	9,399	8,346	7,768	7,793
219597_s_at	5,954	6,035	6,152	6,949	6,938	7,156
219598_s_at	10,314	10,648	10,388	11,337	11,644	11,772
219599_at	9,388	10,166	10,064	8,166	8,725	8,417
219600_s_at	9,118	9,745	9,247	10,260	10,057	10,237
219601_s_at	4,993	5,130	4,502	5,093	5,018	5,351
219602_s_at	7,865	7,554	7,649	6,567	6,067	6,235
219603_s_at	7,396	8,765	7,161	9,833	9,976	10,191
219604_s_at	6,461	6,472	6,576	6,346	6,327	6,349
219605_at	7,051	6,886	6,940	7,893	6,809	7,200
219606_at	6,068	5,848	6,367	6,066	6,598	6,241
219607_s_at	3,669	3,589	3,902	3,838	3,589	3,898
219608_s_at	6,725	6,303	6,677	6,396	6,419	6,681
219609_at	7,104	7,359	7,464	7,634	7,420	7,735

219610_at	7,515	7,281	7,183	7,888	9,035	8,095
219611_s_at	8,145	8,009	8,078	7,734	7,673	7,501
219612_s_at	5,390	5,081	5,326	5,731	5,724	5,943
219613_s_at	8,523	8,312	8,388	8,751	8,207	8,623
219614_s_at	5,072	5,015	5,014	5,297	5,072	5,108
219615_s_at	8,636	8,380	8,532	7,030	6,674	6,945
219616_at	8,077	8,839	8,043	7,803	7,744	7,318
219617_at	8,625	8,696	8,464	7,956	7,486	7,686
219618_at	6,758	6,345	6,548	7,213	7,481	7,326
219619_at	3,634	3,841	3,939	3,825	3,615	3,789
219620_x_at	6,728	7,029	7,094	7,474	7,388	7,883
219621_at	5,254	5,614	5,094	5,509	5,735	5,716
219622_at	7,969	8,427	8,443	9,290	8,639	8,934
219623_at	9,481	9,658	9,570	9,135	8,757	8,813
219624_at	7,028	7,739	7,439	6,842	6,845	6,920
219625_s_at	10,561	10,808	10,187	9,568	10,320	9,365
219626_at	8,724	8,783	8,604	8,501	9,258	8,229
219627_at	8,320	7,496	7,754	7,380	7,451	7,213
219628_at	6,991	6,831	6,506	6,641	7,696	6,913
219629_at	7,268	7,170	7,386	7,306	6,490	6,550
219630_at	6,324	6,407	5,893	6,630	6,349	6,699
219631_at	7,705	7,644	7,254	7,003	7,885	7,433
219632_s_at	6,867	6,637	6,925	6,444	6,186	6,099
219633_at	7,392	7,295	7,556	8,134	7,913	8,202
219634_at	7,551	7,609	7,570	8,406	7,529	7,798
219635_at	9,623	9,802	9,565	7,681	7,727	7,477
219636_s_at	8,790	8,656	8,786	8,624	7,604	8,079
219637_at	9,201	8,943	8,830	8,301	7,212	7,475
219638_at	6,723	7,192	6,619	7,089	6,440	6,767
219639_x_at	9,238	8,992	9,295	8,667	8,599	8,672
219640_at	8,038	7,852	8,018	7,991	7,722	8,370
219641_at	9,893	10,058	9,953	8,487	8,225	8,390
219642_s_at	5,326	5,388	5,488	5,257	5,405	5,331
219643_at	3,633	3,784	3,470	3,631	3,603	3,565
219644_at	8,962	9,028	8,522	5,795	6,656	5,778
219645_at	5,240	5,430	5,366	5,213	5,098	5,135
219646_at	9,317	9,361	9,571	10,358	9,464	10,464
219647_at	5,721	5,775	6,214	5,951	6,176	6,050
219648_at	9,831	9,683	10,224	7,282	6,570	6,860
219649_at	11,583	11,754	11,613	9,787	9,591	9,691
219650_at	9,695	9,455	9,229	8,365	7,728	7,386
219651_at	11,569	11,720	11,532	4,215	4,494	4,411
219652_s_at	7,053	6,881	6,856	7,310	7,598	7,350
219653_at	8,102	7,429	7,339	6,774	6,629	6,983
219654_at	10,448	10,803	10,779	11,616	11,136	11,711
219655_at	8,138	8,670	8,157	8,622	7,933	8,353
219656_at	7,856	7,738	7,763	8,392	8,457	8,187
219657_s_at	7,190	6,835	7,046	7,170	6,761	7,204
219658_at	8,602	8,124	7,916	6,947	7,452	7,143
219659_at	7,618	7,232	7,494	7,750	7,223	7,424
219660_s_at	6,892	6,753	6,452	6,455	6,099	6,269
219661_at	7,444	7,481	7,514	7,846	7,790	7,792
219662_at	8,539	8,512	8,709	9,402	8,288	8,852
219663_s_at	6,871	7,068	6,810	7,447	6,964	7,032
219664_s_at	9,694	10,209	10,204	9,989	9,530	10,498
219665_at	7,323	7,875	7,599	7,880	7,703	8,150

219666_at	6,579	6,313	6,359	7,092	7,080	6,737
219667_s_at	4,441	4,164	4,016	4,921	5,022	5,206
219668_at	8,104	8,445	7,677	7,162	6,944	7,306
219669_at	6,947	6,760	7,052	7,241	7,175	7,417
219670_at	7,737	8,081	7,842	6,268	6,281	6,382
219671_at	5,116	5,079	5,175	5,537	5,369	5,417
219672_at	7,316	7,398	7,170	7,524	7,139	7,551
219673_at	7,680	7,347	7,723	6,142	7,015	5,746
219674_s_at	6,536	7,019	7,331	7,849	7,892	8,032
219675_s_at	9,305	9,105	8,930	9,100	8,799	9,202
219676_at	7,376	6,670	7,060	5,903	5,634	5,623
219677_at	7,229	7,558	7,083	8,482	8,293	8,278
219678_x_at	8,816	8,522	8,633	7,589	8,553	7,896
219679_s_at	8,207	8,265	7,548	6,246	6,303	6,170
219680_at	9,200	9,220	9,243	8,675	8,511	9,077
219681_s_at	9,122	9,398	9,334	6,706	6,587	7,025
219682_s_at	6,171	5,019	5,371	8,879	7,365	7,785
219683_at	9,595	9,399	9,171	5,954	5,717	5,994
219684_at	5,246	5,618	5,636	6,189	6,042	6,026
219685_at	8,473	8,643	8,148	5,644	7,314	6,604
219686_at	8,616	8,403	8,085	7,337	7,089	7,333
219687_at	6,302	6,441	6,318	5,754	5,803	6,071
219688_at	6,932	7,503	6,515	6,478	7,592	6,237
219689_at	7,489	7,362	7,427	8,147	7,481	7,460
219690_at	7,786	8,026	7,932	7,664	7,396	7,554
219691_at	5,154	5,598	5,440	6,411	7,057	6,818
219692_at	9,049	8,663	8,813	8,570	8,348	8,464
219693_at	8,049	8,103	7,725	7,620	7,101	7,372
219694_at	6,543	6,923	6,174	4,951	4,959	5,326
219695_at	6,686	6,420	6,267	6,969	6,507	6,863
219696_at	7,518	8,204	7,597	5,912	7,471	5,897
219697_at	7,138	7,141	7,389	7,480	7,130	7,405
219698_s_at	8,278	8,426	8,095	7,478	7,640	6,839
219699_at	8,533	8,516	8,426	8,050	7,962	8,020
219700_at	5,418	5,738	5,657	5,875	6,077	6,315
219701_at	3,762	3,849	3,687	4,063	4,044	4,214
219702_at	6,277	6,620	6,171	7,096	6,850	7,265
219703_at	6,814	7,277	6,240	5,857	6,834	5,229
219704_at	8,775	8,392	8,188	8,653	8,651	8,609
219705_at	6,650	6,875	7,390	6,177	6,518	6,237
219706_at	7,892	7,605	8,009	9,558	9,099	9,390
219707_at	8,461	8,397	8,343	8,932	9,265	9,509
219708_at	7,508	7,768	7,487	7,312	7,261	7,327
219709_x_at	8,276	8,181	8,448	9,141	8,670	9,007
219710_at	5,793	5,602	5,690	5,805	5,660	5,715
219711_at	9,387	9,414	9,440	7,930	7,416	7,899
219712_s_at	7,532	7,464	7,584	7,807	7,613	7,581
219713_at	6,765	7,008	6,948	7,608	7,335	7,474
219714_s_at	7,470	7,820	7,804	6,409	6,095	6,690
219715_s_at	8,406	8,130	8,312	10,170	9,137	9,454
219716_at	5,848	5,916	6,026	6,887	6,780	6,771
219717_at	8,245	7,958	8,225	6,750	6,150	6,758
219718_at	9,191	9,776	9,295	7,967	7,977	8,292
219719_at	6,870	6,912	6,803	7,077	7,288	7,033
219720_s_at	6,598	7,522	6,780	6,544	6,740	6,804
219721_at	6,410	6,216	6,363	6,630	6,559	6,899

219722_s_at	7,052	7,146	6,899	8,050	6,835	7,664
219723_x_at	7,069	7,083	7,137	7,934	7,482	7,827
219724_s_at	4,969	5,243	5,112	5,258	5,235	5,126
219725_at	7,871	7,379	7,391	7,814	7,663	7,814
219726_at	7,717	7,298	7,347	7,520	6,838	6,921
219727_at	5,581	5,618	5,417	6,157	5,804	6,144
219728_at	4,437	4,053	4,213	5,460	5,101	5,542
219729_at	8,703	8,855	8,601	8,590	8,591	8,707
219730_at	5,075	5,276	5,539	4,930	4,619	4,780
219731_at	9,227	9,050	9,001	8,561	8,854	8,613
219732_at	6,046	6,755	6,393	5,345	5,175	5,218
219733_s_at	8,360	8,365	8,331	8,553	8,667	8,915
219734_at	3,938	4,203	4,011	4,310	4,119	4,197
219735_s_at	6,665	6,950	7,415	6,465	6,737	6,351
219736_at	8,592	8,818	8,375	4,420	4,570	4,138
219737_s_at	6,111	6,311	6,136	8,772	11,525	8,833
219738_s_at	5,509	5,813	6,075	6,059	8,033	6,417
219739_at	6,131	6,175	6,349	6,997	6,917	7,001
219740_at	8,932	8,833	8,633	5,918	5,816	5,857
219741_x_at	6,948	6,768	6,684	6,999	6,527	7,022
219742_at	6,295	6,514	6,170	7,285	6,807	7,075
219743_at	5,340	5,692	6,232	4,747	4,647	4,480
219744_at	5,983	6,250	6,156	6,098	5,863	5,756
219745_at	7,832	7,112	7,843	7,627	7,298	7,500
219746_at	6,703	6,785	6,690	6,929	6,858	6,962
219747_at	7,877	8,382	7,599	7,066	6,497	7,054
219748_at	7,263	7,012	7,124	7,161	7,265	7,514
219749_at	8,164	8,043	8,144	8,730	8,618	8,694
219750_at	4,125	4,356	3,607	4,037	3,826	4,178
219751_at	10,968	10,555	10,739	8,183	7,867	8,023
219752_at	7,471	7,361	7,819	7,979	7,915	8,166
219753_at	6,915	7,102	6,782	7,517	7,231	7,263
219754_at	7,734	7,858	7,480	5,864	6,247	5,766
219755_at	7,048	6,502	6,883	7,205	7,052	7,162
219756_s_at	4,913	5,054	4,853	5,345	5,297	5,311
219757_s_at	8,436	8,709	8,158	6,217	7,503	6,906
219758_at	7,396	7,435	7,347	7,666	7,340	6,853
219759_at	4,543	4,468	4,446	6,387	5,715	6,322
219760_at	7,283	7,913	7,327	7,328	7,088	7,797
219761_at	6,138	6,226	6,338	6,792	6,322	6,146
219762_s_at	12,658	12,348	12,427	11,983	11,528	12,309
219763_at	8,668	8,933	8,965	8,544	7,766	8,147
219764_at	5,688	6,780	6,540	4,518	4,392	4,578
219765_at	8,891	8,982	9,179	7,308	7,463	7,545
219766_at	7,536	7,478	7,482	8,205	7,832	7,900
219767_s_at	9,657	10,021	9,207	7,831	8,103	7,772
219768_at	6,204	6,195	6,117	6,763	6,756	6,702
219769_at	7,702	7,381	7,478	7,214	6,963	7,198
219770_at	8,762	9,031	8,683	8,319	8,750	8,346
219771_at	6,528	7,260	5,996	7,351	8,626	7,498
219772_s_at	5,714	5,645	5,843	5,977	5,777	5,925
219773_at	7,346	7,197	6,872	5,188	5,190	5,259
219774_at	7,746	7,853	7,867	8,652	8,936	8,499
219775_s_at	6,536	6,058	6,652	7,111	7,466	7,295
219776_s_at	6,562	6,559	6,255	7,248	7,309	7,544
219777_at	3,762	3,452	3,384	4,159	3,918	4,036

219778_at	5,993	6,043	5,692	5,741	5,522	5,575
219779_at	7,374	7,021	7,079	7,066	8,238	7,881
219780_at	5,600	5,359	4,883	5,065	4,796	4,955
219781_s_at	9,018	8,711	9,115	9,075	8,489	9,001
219782_s_at	5,402	5,253	5,329	5,847	5,838	6,230
219783_at	7,468	7,535	8,044	8,807	8,230	8,657
219784_at	8,458	8,359	8,385	9,165	8,856	8,973
219785_s_at	7,767	8,544	8,428	8,780	7,941	8,458
219786_at	8,043	8,226	7,941	6,816	6,918	7,004
219787_s_at	12,055	11,899	11,327	9,813	10,792	8,748
219788_at	5,414	5,954	5,328	6,856	6,716	7,093
219789_at	4,214	5,545	4,993	8,567	10,281	10,371
219790_s_at	5,748	5,381	4,904	6,722	7,249	7,668
219791_s_at	4,836	5,826	5,638	5,849	5,716	5,514
219792_at	6,227	6,282	6,190	6,718	6,621	7,038
219793_at	5,499	6,511	5,819	6,117	7,059	6,667
219794_at	6,284	5,783	6,007	6,363	6,310	6,289
219795_at	3,693	4,008	3,658	3,651	3,648	3,732
219796_s_at	6,853	6,891	6,617	7,662	7,696	7,554
219797_at	4,775	5,376	4,881	4,226	4,101	4,077
219798_s_at	10,390	10,004	10,546	10,187	9,456	10,024
219799_s_at	7,099	6,856	7,109	10,436	11,699	11,801
219800_s_at	7,120	6,608	6,969	5,514	5,294	5,457
219801_at	6,939	7,913	7,920	6,292	6,331	6,283
219802_at	8,884	9,061	8,823	7,417	8,752	7,906
219803_at	3,789	3,900	4,376	4,179	4,336	4,029
219804_at	4,749	4,978	4,555	4,806	4,683	5,434
219805_at	7,926	7,685	8,344	7,704	6,717	7,806
219806_s_at	10,208	10,530	10,384	8,955	8,476	9,421
219807_x_at	8,435	8,801	8,278	8,892	8,710	9,346
219808_at	8,051	7,960	8,406	8,033	7,741	7,754
219809_at	8,238	8,062	8,455	9,083	8,427	8,622
219810_at	6,501	6,410	6,249	6,668	7,159	6,952
219811_at	7,919	7,991	8,274	7,912	7,804	7,672
219812_at	5,981	6,287	6,270	6,675	6,922	7,072
219813_at	5,802	6,053	6,074	5,925	5,933	5,790
219814_at	7,225	7,866	7,107	5,949	6,038	5,868
219815_at	7,858	7,856	7,776	7,946	7,711	7,975
219816_s_at	9,518	9,218	9,350	8,972	8,597	8,732
219817_at	8,139	8,606	8,147	7,119	6,559	6,917
219818_s_at	8,166	8,238	7,974	7,714	7,555	7,406
219819_s_at	8,348	8,620	8,568	10,219	9,045	9,524
219820_at	7,053	6,455	7,096	5,870	5,753	5,901
219821_s_at	8,133	7,809	8,321	8,510	8,235	8,395
219822_at	8,745	9,229	8,516	7,533	7,449	7,277
219823_at	14,086	13,866	14,127	6,644	6,278	6,219
219824_at	6,614	6,748	6,712	7,656	7,689	7,742
219825_at	6,845	8,287	6,704	6,591	6,913	6,355
219826_at	8,387	8,242	8,180	7,343	6,919	7,138
219827_at	7,301	7,358	7,287	7,909	7,996	8,158
219828_at	10,078	9,787	9,760	9,049	8,750	8,911
219829_at	7,970	8,188	7,615	6,257	6,250	6,391
219830_at	7,480	7,570	7,556	7,770	7,701	7,857
219831_at	7,467	7,782	7,431	7,156	6,770	6,507
219832_s_at	6,691	6,638	7,021	7,698	7,430	7,251
219833_s_at	8,889	9,124	8,407	8,314	8,925	7,699

219834_at	4,595	5,781	4,718	4,712	4,726	5,051
219835_at	7,271	6,720	6,833	7,379	7,049	7,394
219836_at	5,483	5,371	5,359	6,025	5,893	6,191
219837_s_at	5,564	5,163	5,558	5,826	5,823	5,910
219838_at	8,810	8,980	8,685	7,888	7,906	7,927
219839_x_at	5,899	6,300	6,434	6,765	6,491	6,870
219840_s_at	5,482	5,214	5,804	6,091	6,424	6,462
219841_at	4,521	4,461	4,467	4,452	4,427	4,511
219842_at	5,574	6,179	5,542	5,142	4,923	5,225
219843_at	8,043	8,065	7,799	7,195	7,159	7,112
219844_at	4,958	5,750	5,304	5,280	5,584	5,272
219845_at	7,209	7,421	7,453	6,639	5,879	6,150
219846_at	5,987	5,394	5,753	4,950	5,447	5,239
219847_at	8,045	7,531	7,625	8,466	8,074	8,153
219848_s_at	8,543	8,782	8,634	6,184	9,190	7,668
219849_at	8,115	7,828	8,067	7,405	7,092	7,463
219850_s_at	4,759	4,510	4,006	5,160	4,912	4,921
219851_at	6,234	6,430	6,472	5,907	5,945	5,816
219852_s_at	8,005	7,913	8,024	8,723	8,800	9,111
219853_at	5,712	5,488	5,274	5,634	5,382	5,588
219854_at	9,074	9,225	8,529	6,885	7,391	6,924
219855_at	11,244	11,246	11,124	9,169	9,716	9,225
219856_at	6,806	7,023	6,880	7,713	7,592	7,830
219857_at	4,350	4,604	4,821	4,863	5,005	4,778
219858_s_at	4,414	4,351	4,329	4,521	4,705	4,595
219859_at	5,135	5,178	5,118	4,961	4,815	4,966
219860_at	6,590	6,992	6,895	7,646	6,851	7,321
219861_at	7,852	7,624	7,745	7,810	7,376	7,888
219862_s_at	9,286	9,425	9,454	9,189	8,918	9,290
219863_at	8,850	9,506	8,676	5,653	5,122	5,536
219864_s_at	5,275	5,051	5,098	5,411	5,277	5,555
219865_at	7,718	8,116	7,806	6,848	6,665	6,973
219866_at	6,955	7,118	7,199	7,604	7,546	7,508
219867_at	7,541	7,985	7,527	6,776	5,892	5,935
219868_s_at	8,038	7,334	7,630	7,619	7,458	7,640
219869_s_at	7,856	7,416	7,655	8,038	7,152	7,386
219870_at	7,920	8,371	7,962	7,459	7,528	7,205
219871_at	7,378	6,993	6,615	6,028	6,249	6,466
219872_at	5,574	5,813	5,354	5,525	5,806	5,968
219873_at	6,516	6,278	6,500	7,508	6,812	6,800
219874_at	7,964	8,181	8,414	7,499	7,318	7,636
219875_s_at	4,929	4,991	4,819	4,690	4,367	4,809
219876_s_at	6,842	6,622	6,639	7,243	6,926	7,113
219877_at	8,390	9,005	8,278	7,735	7,647	7,761
219878_s_at	6,657	6,622	6,110	6,358	5,972	6,562
219879_s_at	8,182	7,856	8,223	8,130	8,140	8,330
219880_at	9,073	9,466	9,381	7,793	8,055	7,682
219881_s_at	6,686	6,188	6,115	5,722	5,877	5,798
219882_at	7,986	8,095	7,201	7,191	8,108	7,453
219883_at	7,910	7,935	8,067	8,313	8,280	8,029
219884_at	7,669	7,825	7,988	7,940	7,728	7,734
219885_at	5,753	6,240	5,529	7,647	8,526	7,524
219886_at	6,342	6,261	5,731	5,653	6,015	5,283
219887_at	6,418	6,314	6,453	6,623	6,305	6,469
219888_at	6,419	6,750	6,753	7,319	7,181	7,441
219889_at	7,403	7,478	7,962	6,909	6,628	6,557

219890_at	4,599	4,431	4,693	4,776	4,525	4,518
219891_at	6,508	5,722	7,035	8,122	7,718	7,956
219892_at	5,163	6,993	5,570	5,223	5,070	5,279
219893_at	6,955	6,753	7,232	6,735	6,415	6,682
219894_at	7,804	8,385	7,911	5,988	6,325	5,915
219895_at	9,350	9,576	8,651	3,731	3,603	3,769
219896_at	5,416	4,500	5,036	5,900	5,911	5,902
219897_at	7,257	7,057	7,188	7,026	6,956	6,818
219898_at	4,693	4,527	4,655	5,088	4,988	4,920
219899_x_at	7,571	7,438	7,397	7,850	7,780	8,130
219900_s_at	8,225	8,117	8,334	8,558	8,382	8,521
219901_at	7,615	8,101	7,414	5,744	5,969	5,869
219902_at	5,253	5,686	5,220	7,434	8,068	7,885
219903_s_at	6,280	6,090	6,323	6,263	6,100	6,114
219904_at	8,425	8,061	8,769	8,257	7,807	7,862
219905_at	7,453	7,278	7,343	6,898	6,882	7,025
219906_at	9,473	9,183	9,371	7,804	8,041	7,378
219907_at	7,435	7,059	7,330	7,904	7,602	7,767
219908_at	3,945	4,245	4,309	4,426	5,296	4,841
219909_at	5,401	5,737	5,326	5,848	6,151	6,079
219910_at	7,904	7,901	7,990	8,276	7,909	8,303
219911_s_at	8,730	8,905	8,662	7,168	7,457	7,112
219912_s_at	4,982	5,012	5,122	6,130	6,201	6,182
219913_s_at	9,697	9,885	9,589	7,438	8,261	6,962
219914_at	7,584	7,691	7,601	7,431	7,397	7,410
219915_s_at	4,563	4,119	4,241	4,201	4,128	4,061
219916_s_at	7,083	7,003	7,414	7,756	7,651	7,437
219917_at	7,583	7,831	7,646	6,841	6,572	6,510
219918_s_at	11,001	11,031	10,237	8,117	9,581	6,404
219919_s_at	7,870	8,125	8,176	7,727	7,892	8,071
219920_s_at	9,685	9,467	9,836	11,159	10,239	10,996
219921_s_at	6,368	6,602	6,901	7,754	7,573	7,607
219922_s_at	7,602	6,925	6,881	7,590	8,599	8,857
219923_at	8,897	9,146	8,998	8,000	8,116	8,138
219924_s_at	8,434	8,704	8,191	8,497	7,929	8,598
219925_at	7,137	7,700	7,535	7,477	7,540	7,635
219926_at	8,381	8,626	8,819	10,520	9,683	9,995
219927_at	5,568	5,003	5,035	5,368	5,066	5,404
219928_s_at	7,374	8,254	7,608	7,088	7,062	7,184
219929_s_at	6,666	7,002	6,360	7,106	6,235	7,157
219930_at	6,355	6,498	6,868	6,360	6,740	6,495
219931_s_at	8,843	9,180	8,548	7,717	7,591	7,908
219932_at	5,291	6,462	5,999	5,289	5,080	5,180
219933_at	9,577	9,563	9,566	11,831	11,240	11,709
219934_s_at	5,679	5,781	5,789	6,038	6,075	5,981
219935_at	4,227	4,619	4,880	5,457	7,495	4,532
219936_s_at	4,136	4,535	4,723	4,765	4,636	4,719
219937_at	5,362	5,889	5,458	5,950	6,688	7,775
219938_s_at	6,514	6,845	6,771	5,891	6,722	6,832
219939_s_at	12,564	12,422	12,367	10,509	11,599	10,840
219940_s_at	10,840	10,831	10,506	10,387	10,056	10,404
219941_at	4,863	4,812	4,766	5,322	4,957	5,091
219942_at	6,609	6,753	6,938	7,599	7,583	7,309
219943_s_at	7,890	7,745	7,997	8,398	8,204	8,149
219944_at	5,742	5,491	5,722	8,022	8,086	7,808
219945_at	8,833	9,649	9,047	6,724	6,405	6,701

219946_x_at	8,003	7,522	7,767	8,487	8,216	8,496
219947_at	6,050	6,051	5,944	5,500	5,875	5,606
219948_x_at	3,424	3,696	3,723	3,432	3,492	3,562
219949_at	4,603	5,497	5,055	3,807	5,602	4,361
219950_s_at	6,232	6,262	6,302	6,387	6,027	6,071
219951_s_at	8,851	8,701	8,476	7,348	7,374	7,014
219952_s_at	7,612	7,344	7,677	8,932	7,961	8,169
219953_s_at	8,210	8,147	8,442	9,235	8,361	8,977
219954_s_at	6,171	6,124	5,989	6,540	5,963	6,015
219955_at	12,414	12,590	11,847	7,024	6,963	6,900
219956_at	5,245	5,869	6,339	6,600	5,771	5,378
219957_at	6,489	6,339	5,959	5,244	6,095	4,996
219958_at	7,969	7,856	7,533	7,096	7,165	7,191
219959_at	6,434	7,024	7,387	10,185	10,010	9,372
219960_s_at	9,971	9,790	9,438	9,347	9,565	8,861
219961_s_at	9,184	9,778	8,715	7,006	7,316	6,796
219962_at	4,801	4,398	4,691	5,381	4,877	5,541
219963_at	7,770	8,242	7,740	7,766	7,405	7,630
219964_at	5,708	5,737	5,913	6,347	6,345	6,615
219965_s_at	4,807	5,028	4,962	5,148	4,787	5,301
219966_x_at	11,025	11,293	11,717	10,599	10,306	10,558
219967_at	7,880	7,954	8,033	8,566	7,960	8,139
219968_at	9,799	9,390	9,731	7,441	7,378	7,428
219969_at	7,863	7,961	7,613	7,961	7,447	7,767
219970_at	4,611	5,115	4,817	4,425	4,468	4,723
219971_at	5,793	5,827	5,496	5,545	5,074	5,465
219972_s_at	9,468	9,435	8,886	7,440	8,897	7,797
219973_at	5,494	6,407	5,636	8,512	10,004	9,949
219974_x_at	9,125	8,919	8,644	9,381	9,558	8,810
219975_x_at	4,536	4,870	4,971	7,574	10,241	9,141
219976_at	8,039	8,700	7,441	5,115	5,288	5,148
219977_at	5,751	6,530	6,226	6,340	6,333	6,299
219978_s_at	9,488	9,548	9,059	7,644	7,153	7,596
219979_s_at	9,368	9,462	9,512	9,540	8,736	9,485
219980_at	7,387	6,608	6,973	5,950	6,073	5,801
219981_x_at	9,189	9,183	9,186	8,639	9,659	8,987
219982_s_at	7,707	7,673	7,980	7,281	6,894	7,393
219983_at	10,963	11,314	11,280	5,209	5,172	5,563
219984_s_at	8,711	8,563	8,492	4,584	4,537	4,779
219985_at	8,463	8,459	8,911	10,646	9,000	9,652
219986_s_at	7,425	7,324	7,269	7,057	7,283	7,455
219987_at	6,368	6,065	6,442	6,025	6,151	5,990
219988_s_at	10,680	10,409	10,557	9,507	8,786	9,240
219989_s_at	5,645	5,485	5,749	6,048	6,166	6,022
219990_at	8,154	8,091	8,158	5,854	6,550	5,825
219991_at	7,451	7,354	7,725	7,996	7,355	7,865
219992_at	8,416	8,855	8,599	7,935	7,275	7,583
219993_at	4,836	4,469	4,614	5,913	4,813	4,942
219994_at	7,235	6,879	7,104	8,379	8,061	8,339
219995_s_at	3,103	3,315	3,448	3,689	3,473	3,567
219996_at	7,121	7,388	7,174	7,532	7,437	7,424
219997_s_at	10,039	9,766	10,034	9,395	8,959	9,353
219998_at	7,371	7,520	7,188	6,394	6,458	6,663
219999_at	7,015	7,131	7,229	7,409	7,580	7,466
220000_at	6,568	6,675	6,778	7,554	7,379	7,429
220001_at	6,119	5,943	6,151	6,582	6,085	6,272

220002_at	5,973	5,806	6,045	6,278	6,335	6,748
220003_at	8,260	7,826	8,148	9,047	8,692	9,056
220004_at	4,663	5,075	4,937	6,385	6,265	5,622
220005_at	5,235	4,836	4,953	5,423	5,597	5,485
220006_at	6,823	6,589	6,758	7,118	6,930	7,030
220007_at	8,572	8,226	8,387	7,620	7,936	7,461
220008_at	8,508	8,198	8,254	8,737	8,456	8,217
220009_at	6,920	6,891	6,457	7,015	7,147	7,115
220010_at	5,862	5,706	5,900	6,720	6,712	6,829
220011_at	9,888	10,050	10,309	7,874	7,564	7,457
220012_at	6,025	6,472	6,277	5,840	5,646	5,905
220013_at	7,890	7,525	7,431	7,192	7,073	7,242
220014_at	4,214	4,815	4,528	8,295	9,874	10,180
220015_at	7,915	7,871	7,637	8,399	8,690	8,515
220016_at	6,559	6,222	6,386	7,866	7,321	7,581
220017_x_at	7,224	7,032	6,981	7,546	7,344	7,487
220018_at	8,269	8,105	8,319	7,172	6,829	7,032
220019_s_at	7,213	7,518	5,954	5,996	6,371	5,579
220020_at	6,870	6,206	6,798	7,003	7,113	7,077
220021_at	6,641	6,491	6,809	6,079	6,290	6,240
220022_at	7,573	7,904	6,957	5,431	5,392	5,560
220023_at	6,885	6,636	6,780	7,275	7,146	7,286
220024_s_at	7,705	7,895	8,239	8,101	8,291	8,321
220025_at	5,230	4,807	4,913	5,103	4,944	5,194
220026_at	3,924	4,001	4,117	4,077	4,014	3,961
220027_s_at	7,705	7,952	7,941	7,897	7,626	8,595
220028_at	9,306	8,899	8,827	7,852	7,515	7,746
220029_at	6,324	6,041	6,478	6,864	6,693	7,073
220030_at	7,617	8,066	7,264	6,081	4,932	4,961
220031_at	5,395	5,209	5,308	5,876	5,819	5,964
220032_at	6,470	6,185	6,212	7,517	7,787	7,602
220033_at	8,339	8,442	8,560	6,812	6,840	6,930
220034_at	5,909	6,311	6,081	7,144	7,055	6,649
220035_at	7,767	6,890	6,852	6,850	6,714	6,915
220036_s_at	8,638	8,861	8,600	8,645	7,935	8,406
220037_s_at	4,012	4,317	4,476	5,179	5,271	5,487
220038_at	4,580	5,200	4,499	4,902	4,705	4,344
220039_s_at	4,501	3,957	4,373	3,952	4,013	3,973
220040_x_at	8,555	8,641	9,218	7,202	7,182	7,380
220041_at	5,976	6,870	6,549	7,147	7,423	7,580
220042_x_at	8,545	8,442	8,591	9,318	9,321	9,741
220043_s_at	6,124	5,974	5,967	6,931	6,362	6,604
220044_x_at	12,333	12,154	11,620	9,742	11,200	9,407
220045_at	7,005	6,572	6,766	6,857	7,047	7,107
220046_s_at	10,401	11,154	10,233	8,412	11,220	9,019
220047_at	6,902	7,733	7,202	6,350	6,053	6,557
220048_at	7,675	7,679	7,450	8,177	8,262	8,165
220049_s_at	4,935	4,912	4,669	5,862	5,594	5,947
220050_at	7,317	7,368	7,341	7,981	7,516	7,746
220051_at	6,473	6,264	6,304	7,031	6,938	7,076
220052_s_at	7,866	7,767	7,795	8,307	7,750	8,645
220053_at	11,998	11,213	12,127	7,408	7,003	7,296
220054_at	7,526	7,552	7,867	7,630	7,454	7,849
220055_at	5,268	4,936	4,776	4,319	4,312	4,466
220056_at	5,780	5,990	5,901	6,230	6,296	6,488
220057_at	6,391	6,045	6,316	7,263	7,270	7,358

220058_at	6,842	7,409	7,242	7,538	6,743	7,209
220059_at	4,697	4,613	4,346	4,793	4,575	4,711
220060_s_at	8,986	9,169	8,928	7,083	7,070	6,863
220061_at	6,791	7,237	6,936	6,976	6,781	6,903
220062_s_at	5,862	5,754	5,770	6,070	5,952	5,889
220063_at	7,771	7,469	7,815	6,389	6,642	6,411
220064_at	5,697	5,441	5,618	5,465	5,633	5,525
220065_at	9,240	9,575	9,399	5,210	4,869	4,926
220066_at	5,861	6,188	6,035	5,705	5,573	5,599
220067_at	6,477	6,600	6,400	7,365	7,281	7,474
220068_at	6,157	6,288	6,393	6,474	6,536	6,721
220069_at	6,756	6,962	6,901	7,479	7,551	7,602
220070_at	8,300	7,864	7,994	7,411	7,341	7,442
220071_x_at	9,656	9,393	9,415	8,691	9,690	8,888
220072_at	8,385	8,595	8,446	7,553	7,864	7,225
220073_s_at	7,374	7,224	7,259	7,202	6,876	7,199
220074_at	6,505	6,369	6,438	7,003	6,991	6,962
220075_s_at	5,281	5,281	5,462	5,996	5,743	5,951
220076_at	4,738	4,802	5,133	4,215	5,703	4,446
220077_at	7,125	6,980	6,900	7,606	7,238	7,547
220078_at	8,975	8,814	8,429	7,200	7,709	7,064
220079_s_at	10,139	10,360	9,875	8,763	10,334	8,470
220080_at	6,174	6,360	6,129	7,059	6,946	7,253
220081_x_at	10,104	9,828	9,846	9,824	9,363	9,181
220082_at	6,094	5,990	6,288	6,940	6,464	6,606
220083_x_at	8,381	8,165	7,842	7,847	7,444	7,485
220084_at	3,789	3,867	3,848	4,076	4,013	3,933
220085_at	10,911	10,821	10,904	7,859	8,793	7,284
220086_at	8,017	7,600	7,896	8,070	8,102	7,823
220087_at	6,680	6,973	6,777	5,729	5,421	5,815
220088_at	4,224	4,321	4,182	5,042	5,049	5,761
220089_at	6,319	6,758	6,150	5,544	5,661	5,161
220090_at	7,487	7,226	7,312	7,523	7,659	7,684
220091_at	7,617	7,821	8,009	8,637	8,112	8,576
220092_s_at	6,469	6,475	6,548	7,260	7,026	7,682
220093_at	7,317	7,467	7,648	7,830	7,691	8,080
220094_s_at	10,085	10,378	10,295	10,815	9,984	10,239
220095_at	7,684	7,781	6,954	5,895	6,138	5,683
220096_at	6,874	6,778	6,862	7,258	7,108	7,061
220097_s_at	8,391	7,850	8,321	8,498	8,560	8,831
220098_at	6,039	5,721	5,794	6,115	6,148	6,129
220099_s_at	10,184	10,090	9,752	8,588	9,170	8,569
220100_at	6,421	6,285	6,157	6,791	6,556	6,676
220101_x_at	6,037	6,168	6,100	6,687	6,388	6,375
220102_at	5,948	5,988	5,844	5,387	5,381	5,392
220103_s_at	4,946	4,787	4,701	4,806	4,825	4,713
220104_at	6,410	6,330	6,454	7,258	7,067	7,052
220105_at	6,371	6,211	6,327	6,455	6,091	6,281
220106_at	7,127	7,156	7,267	7,689	7,110	7,511
220107_s_at	6,665	6,746	6,515	7,367	6,987	6,972
220108_at	5,542	5,075	5,443	5,863	5,444	5,566
220109_at	3,517	3,744	3,767	3,870	3,727	4,032
220110_s_at	5,493	5,253	5,510	5,894	5,782	5,718
220111_s_at	7,413	7,229	7,333	5,797	5,820	5,947
220112_at	5,712	5,909	5,746	5,956	5,756	5,794
220113_x_at	9,023	8,664	8,934	8,429	9,208	8,414

220114_s_at	6,724	6,739	6,652	6,993	7,122	7,365
220115_s_at	7,037	7,146	7,158	5,677	5,376	5,523
220116_at	8,872	9,059	9,324	5,113	5,043	4,898
220117_at	5,409	5,259	5,170	6,252	6,403	6,167
220118_at	6,123	5,807	6,188	6,238	5,837	6,012
220119_at	6,497	6,821	6,738	6,843	6,443	6,725
220120_s_at	4,674	4,760	4,907	4,741	4,661	4,711
220121_at	6,801	6,505	6,898	6,697	6,824	6,816
220122_at	4,588	4,510	4,686	7,458	6,055	6,542
220123_at	6,157	6,627	6,487	6,247	6,480	6,382
220124_at	5,631	5,950	5,567	4,746	5,090	4,950
220125_at	5,693	5,846	6,400	6,236	6,110	6,486
220126_at	7,332	7,390	7,350	7,868	7,363	7,418
220127_s_at	9,720	9,940	10,036	8,595	8,501	8,915
220128_s_at	7,772	7,809	8,043	8,139	7,803	7,939
220129_at	4,702	4,917	4,772	5,656	5,017	5,347
220130_x_at	6,568	6,561	6,529	6,723	6,437	6,498
220131_at	6,180	6,268	6,625	7,348	7,092	7,285
220132_s_at	4,683	5,234	4,643	4,615	4,648	4,764
220133_at	6,127	5,832	5,812	6,982	6,887	6,738
220134_x_at	7,059	7,246	7,287	8,531	8,145	8,756
220135_s_at	6,399	6,456	6,243	6,411	6,488	6,432
220136_s_at	5,524	6,049	5,806	6,268	6,150	6,014
220137_at	8,044	8,462	8,891	6,881	6,369	6,593
220138_at	6,567	6,607	6,744	7,195	7,271	7,389
220139_at	7,415	7,249	7,389	7,959	7,634	7,815
220140_s_at	8,778	8,859	9,097	9,468	8,900	9,278
220141_at	4,534	5,202	5,006	5,438	5,322	5,124
220142_at	6,668	6,329	6,473	6,253	6,187	6,321
220143_x_at	10,287	9,661	10,073	8,991	8,878	8,963
220144_s_at	8,204	8,536	7,854	6,581	6,924	6,679
220145_at	6,262	6,437	6,042	6,480	6,650	5,890
220146_at	4,520	4,830	4,370	4,812	4,824	4,914
220147_s_at	13,628	13,609	13,412	8,619	8,159	8,041
220148_at	4,627	4,770	4,666	4,874	5,143	4,735
220149_at	6,262	6,031	6,104	7,324	6,614	7,313
220150_s_at	7,938	9,457	7,568	6,309	6,297	6,207
220151_at	7,342	7,154	7,160	7,183	7,074	7,235
220152_at	6,954	6,743	6,811	7,209	7,025	7,223
220153_at	7,882	7,834	8,117	8,533	7,763	8,152
220154_at	4,792	4,732	4,889	4,880	4,846	5,005
220155_s_at	9,145	9,210	9,523	9,827	9,946	9,815
220156_at	6,132	6,191	5,927	6,196	6,227	6,184
220157_x_at	9,508	9,568	9,008	7,974	8,279	7,944
220158_at	6,485	6,751	6,584	6,980	6,698	7,106
220159_at	9,317	9,222	9,020	7,449	7,283	7,032
220160_s_at	9,268	9,097	9,472	9,036	8,619	9,195
220161_s_at	10,653	10,922	10,873	7,644	8,872	8,202
220162_s_at	7,181	7,062	7,154	8,084	7,421	7,722
220163_s_at	7,481	7,707	7,535	8,309	8,346	8,427
220164_s_at	6,270	5,951	6,826	6,989	6,679	6,846
220165_at	7,545	7,618	7,661	7,905	7,497	7,601
220166_at	6,010	5,729	6,129	6,112	6,016	6,082
220167_s_at	7,807	7,957	7,822	7,854	6,984	7,536
220168_at	3,818	3,876	3,947	4,333	4,139	4,108
220169_at	5,695	5,837	5,907	6,776	6,965	6,716

220170_at	4,994	5,415	5,100	5,542	5,538	5,436
220171_x_at	8,164	8,354	7,724	7,744	7,564	7,789
220172_at	7,671	8,173	7,326	6,122	6,540	6,037
220173_at	6,209	6,579	5,998	7,500	8,195	7,538
220174_at	6,452	6,515	6,447	7,601	6,567	6,934
220175_s_at	10,620	10,660	10,131	9,014	9,591	8,696
220176_at	8,180	8,620	7,983	7,613	7,653	7,668
220177_s_at	4,319	4,387	4,444	4,830	4,627	4,698
220178_at	7,779	8,074	7,922	7,685	7,665	8,080
220179_at	7,015	6,593	7,059	8,147	7,670	8,174
220180_at	5,626	5,854	6,052	6,957	9,758	7,780
220181_x_at	7,542	7,669	7,896	8,273	8,265	8,643
220182_at	6,992	7,273	7,183	6,488	6,448	6,421
220183_s_at	8,540	9,294	8,600	7,648	7,816	8,261
220184_at	12,561	12,808	13,301	5,414	5,382	5,415
220185_at	7,460	7,695	7,561	7,541	7,056	7,467
220186_s_at	7,451	7,560	7,789	8,473	8,501	8,551
220187_at	4,803	4,999	4,880	6,384	5,985	6,510
220188_at	6,321	6,334	6,319	6,777	7,087	6,811
220189_s_at	8,559	8,606	8,558	9,134	8,751	9,205
220190_s_at	4,694	5,297	4,268	4,977	4,954	5,390
220191_at	5,284	5,157	5,529	5,467	5,469	5,357
220192_x_at	7,595	7,621	7,733	8,453	8,536	8,631
220193_at	7,232	6,670	6,934	7,490	7,076	7,233
220194_at	4,214	4,811	4,374	4,180	4,110	4,110
220195_at	8,403	8,765	8,393	7,442	7,961	7,489
220196_at	5,627	5,997	5,816	6,053	6,045	6,538
220197_at	5,620	6,015	6,327	5,613	5,378	5,340
220198_s_at	7,188	7,452	7,500	7,463	6,980	7,058
220199_s_at	10,805	10,687	10,246	9,051	9,457	8,781
220200_s_at	6,567	6,339	5,799	7,007	6,991	6,964
220201_at	9,062	9,105	9,115	9,645	10,231	10,022
220202_s_at	8,356	8,206	7,694	6,969	7,699	7,108
220203_at	8,093	8,181	8,192	8,032	8,077	8,106
220204_s_at	7,018	6,647	6,718	7,325	7,046	7,155
220205_at	7,324	8,352	6,851	4,059	4,038	4,079
220206_at	10,320	10,567	10,101	7,487	8,243	6,691
220207_at	6,436	6,431	6,544	6,722	6,197	6,305
220208_at	7,542	7,156	7,327	8,440	8,018	8,299
220209_at	7,404	7,760	7,570	8,984	8,432	8,574
220210_at	7,104	7,341	6,974	7,428	7,370	7,694
220211_at	5,639	5,788	5,480	5,840	6,001	5,996
220212_s_at	9,926	9,754	9,955	8,444	9,243	8,665
220213_at	5,999	6,271	6,074	6,536	6,056	6,205
220214_at	8,982	9,339	8,564	7,842	8,008	7,725
220215_at	8,595	8,842	8,556	7,408	7,019	6,948
220216_at	6,764	6,210	6,555	6,798	6,834	6,498
220217_x_at	7,420	7,477	7,229	7,931	7,683	7,822
220218_at	5,953	6,049	5,649	5,984	5,912	5,954
220219_s_at	6,902	6,911	7,315	8,153	8,059	8,389
220220_at	4,174	3,649	4,077	4,204	4,067	4,097
220221_at	6,440	6,262	6,950	6,363	6,787	6,624
220222_at	5,289	5,257	5,004	5,825	5,693	6,099
220223_at	7,619	7,776	7,156	6,620	6,743	6,607
220224_at	5,024	5,220	5,210	5,160	5,191	5,279
220225_at	6,345	6,322	6,570	7,299	7,534	7,055

220226_at	6,466	6,641	6,633	6,714	6,386	6,578
220227_at	7,038	7,034	7,211	6,456	6,214	6,501
220228_at	4,626	4,751	4,728	5,179	4,950	5,408
220229_s_at	4,988	5,667	5,360	5,504	5,119	5,064
220230_s_at	10,350	10,336	10,382	8,730	7,730	8,220
220231_at	11,431	10,414	10,970	6,705	6,418	6,575
220232_at	7,432	7,081	7,405	6,977	7,347	6,770
220233_at	9,569	9,544	9,424	9,176	8,790	9,265
220234_at	6,335	6,565	6,638	6,562	6,474	6,506
220235_s_at	10,092	10,158	9,156	6,830	7,164	6,634
220236_at	6,139	5,794	6,412	5,714	5,950	5,714
220237_at	6,265	6,329	6,162	5,883	6,229	6,143
220238_s_at	9,323	9,626	9,059	8,388	6,940	7,751
220239_at	11,721	12,461	11,971	11,278	10,392	10,695
220240_s_at	8,721	8,695	8,559	8,367	8,440	8,284
220241_at	6,692	7,123	6,674	6,944	6,607	6,865
220242_x_at	8,403	8,334	8,509	7,410	8,451	7,356
220243_at	8,110	7,801	7,916	6,991	7,043	7,066
220244_at	7,886	8,061	8,273	6,980	7,160	7,168
220245_at	7,023	7,128	7,109	7,629	7,784	7,837
220246_at	5,879	5,706	5,326	5,907	5,716	5,815
220247_at	3,919	4,239	4,206	3,960	3,900	3,839
220248_x_at	9,462	9,499	9,916	9,471	9,109	9,601
220249_at	6,543	6,181	6,042	7,227	6,986	6,996
220250_at	6,929	6,672	6,789	6,681	6,856	6,972
220251_at	8,212	7,956	8,180	7,036	7,313	6,949
220252_x_at	7,763	7,858	7,530	8,318	8,545	8,322
220253_s_at	6,713	6,913	6,757	7,072	7,334	7,632
220254_at	6,011	5,901	5,518	6,773	6,653	6,999
220255_at	8,285	8,774	8,864	9,063	8,428	8,702
220256_s_at	7,231	7,254	7,042	7,869	7,282	7,735
220257_x_at	6,896	6,943	6,833	7,458	7,332	7,470
220258_s_at	8,528	8,475	8,622	8,945	8,534	8,669
220259_at	6,051	6,196	5,905	5,864	5,810	5,771
220260_at	8,334	8,750	8,458	8,717	9,622	9,335
220261_s_at	10,512	10,594	10,657	8,694	7,924	8,883
220262_s_at	7,997	7,648	7,952	8,306	8,109	8,504
220263_at	6,084	6,082	6,200	6,647	6,264	6,638
220264_s_at	7,152	6,988	7,331	8,482	8,260	8,210
220265_at	6,492	6,225	6,585	7,352	6,910	6,810
220266_s_at	6,230	6,457	6,853	6,480	7,737	7,465
220267_at	5,853	6,051	5,834	6,984	7,042	7,073
220268_at	5,167	4,951	5,375	5,522	5,442	5,494
220269_at	4,107	5,320	4,008	4,265	3,986	4,170
220270_at	5,066	4,574	5,123	5,321	5,251	5,720
220271_x_at	7,673	7,511	7,737	8,219	7,839	8,124
220272_at	7,511	7,710	7,816	7,246	8,302	7,545
220273_at	6,338	6,184	6,191	7,936	7,776	7,851
220274_at	6,843	6,198	6,668	6,571	6,242	6,311
220275_at	7,329	7,280	7,083	6,139	6,141	6,296
220276_at	4,993	4,540	5,165	4,899	4,565	4,837
220277_at	8,166	8,311	7,738	7,300	7,160	7,237
220278_at	6,970	7,991	7,057	5,715	5,656	5,925
220279_at	6,973	6,824	6,948	7,257	6,977	7,223
220280_s_at	5,983	6,121	5,845	6,301	6,432	6,641
220281_at	3,987	4,397	4,483	3,745	3,577	3,616

220282_at	7,021	7,431	6,680	7,073	6,792	6,923
220283_at	7,405	7,266	7,275	6,413	6,242	6,563
220284_at	7,166	7,024	7,248	7,177	6,800	7,242
220285_at	8,289	8,020	7,864	7,319	6,859	7,185
220286_at	6,519	6,427	6,598	6,881	6,807	7,047
220287_at	7,245	7,106	7,338	6,745	7,169	7,138
220288_at	6,620	6,744	6,407	6,975	6,982	7,321
220289_s_at	7,506	8,081	7,127	6,210	6,014	6,118
220290_at	7,339	7,344	7,612	7,572	7,436	7,601
220291_at	8,995	8,841	9,083	8,033	7,795	8,032
220292_at	7,568	7,510	7,345	7,675	7,468	7,813
220293_at	4,755	4,872	4,622	4,984	4,854	5,022
220294_at	3,859	4,032	3,998	3,900	3,851	3,843
220295_x_at	7,954	8,202	7,311	6,247	6,205	5,721
220296_at	7,235	7,036	7,647	7,428	7,680	7,565
220297_at	6,713	6,735	6,667	6,730	7,205	6,516
220298_s_at	5,876	6,408	5,562	5,495	5,164	5,567
220299_at	6,584	6,595	6,653	6,290	6,066	6,021
220300_at	6,113	6,715	6,696	6,994	6,992	7,221
220301_at	3,605	3,618	3,935	3,763	4,070	3,865
220302_at	5,474	6,050	5,120	4,999	4,966	5,121
220303_at	7,117	7,225	7,098	7,921	7,697	7,941
220304_s_at	6,902	6,911	6,901	7,249	6,991	7,192
220305_at	7,413	7,288	7,788	7,488	7,809	8,070
220306_at	6,591	6,862	6,662	6,589	6,947	6,546
220307_at	6,239	6,351	6,451	7,124	6,996	7,232
220308_at	7,335	7,526	7,410	7,889	7,416	7,791
220309_at	6,494	6,555	6,623	7,143	6,840	6,945
220310_at	5,146	5,478	5,855	6,184	6,040	5,974
220311_at	8,568	8,715	8,671	8,843	7,961	8,431
220312_at	6,633	6,556	6,554	6,483	6,544	6,599
220313_at	4,601	4,485	4,508	4,682	4,661	4,733
220314_at	6,604	7,048	6,682	7,767	7,728	7,862
220315_at	6,030	6,296	5,877	5,510	5,303	5,836
220316_at	6,762	6,735	7,086	6,844	7,172	7,137
220317_at	5,364	7,181	7,527	4,354	4,250	4,178
220318_at	5,004	5,096	4,960	5,154	5,108	5,241
220319_s_at	8,894	9,169	8,848	8,272	8,016	8,407
220320_at	7,430	7,445	7,336	8,047	7,671	7,986
220321_s_at	5,062	5,157	4,987	4,141	3,986	3,896
220322_at	5,750	5,806	5,636	6,151	5,837	5,909
220323_at	8,287	8,380	8,595	8,295	8,055	8,309
220324_at	3,694	4,293	4,044	4,637	4,909	4,623
220325_at	6,353	6,372	6,404	6,399	6,491	6,621
220326_s_at	8,779	8,455	8,528	7,928	7,824	8,121
220327_at	4,841	5,067	5,005	5,759	8,234	7,053
220328_at	4,765	4,648	4,508	4,797	4,870	4,745
220329_s_at	7,845	8,018	7,477	7,935	7,861	7,509
220330_s_at	4,182	3,863	3,945	3,853	3,814	3,937
220331_at	7,042	6,772	7,542	7,909	7,872	8,158
220332_at	7,197	6,929	7,140	7,897	7,904	7,948
220333_at	6,280	6,670	6,262	8,066	6,932	6,678
220334_at	8,365	8,696	7,847	8,612	9,614	8,146
220335_x_at	9,207	9,206	9,162	7,992	8,236	8,466
220336_s_at	5,977	5,817	6,115	5,974	6,211	6,095
220337_at	7,198	7,212	7,693	8,205	8,349	8,412

220338_at	5,132	4,856	5,154	4,674	4,705	4,424
220339_s_at	9,041	8,703	8,898	9,059	8,823	8,965
220340_at	7,214	7,429	7,354	7,280	7,018	7,006
220341_s_at	9,202	8,911	8,646	8,570	8,628	8,678
220342_x_at	7,387	7,760	7,562	6,764	7,592	7,230
220343_at	6,556	6,169	6,311	6,764	6,724	6,690
220344_at	7,512	7,416	7,564	8,580	8,509	8,716
220345_at	7,297	6,824	6,302	3,808	3,733	3,767
220346_at	6,192	6,588	5,749	8,178	8,407	7,044
220347_at	6,598	6,872	6,469	7,102	7,075	6,953
220348_at	5,833	5,424	5,790	6,448	6,299	6,511
220349_s_at	7,801	7,909	8,056	7,104	7,415	7,639
220350_at	8,383	8,232	7,806	6,996	7,273	7,152
220351_at	4,189	4,326	4,860	4,685	4,608	4,756
220352_x_at	8,317	7,879	8,211	7,240	8,399	7,448
220353_at	6,774	6,514	6,459	5,908	5,672	6,118
220354_at	7,006	6,901	6,803	7,044	6,954	6,909
220355_s_at	9,125	9,086	9,203	7,478	8,188	7,306
220356_at	3,854	4,014	3,927	4,355	5,056	6,141
220357_s_at	7,522	7,249	7,154	8,082	7,823	8,136
220358_at	7,235	7,514	7,576	7,983	7,816	7,753
220359_s_at	4,463	4,707	4,639	4,896	4,659	4,830
220360_at	6,156	6,702	5,521	4,450	4,618	4,658
220361_at	4,475	4,974	4,135	4,183	4,479	4,336
220362_at	6,219	6,262	6,477	6,639	6,586	6,766
220363_s_at	7,272	7,006	7,317	7,621	7,464	7,787
220364_at	4,855	4,608	4,939	5,106	5,189	5,229
220365_at	5,809	5,541	6,000	6,485	6,386	6,576
220366_at	6,836	6,968	6,950	7,148	7,239	7,010
220367_s_at	10,757	10,575	11,072	9,096	9,228	9,243
220368_s_at	7,811	7,480	7,667	5,811	6,265	6,344
220369_at	5,826	5,837	5,750	5,752	5,715	5,703
220370_s_at	7,354	7,452	8,410	6,718	6,648	6,936
220371_s_at	8,233	8,082	8,208	8,164	8,003	8,225
220372_at	5,602	5,692	5,498	5,205	5,445	5,221
220373_at	7,336	6,981	6,852	7,195	6,827	6,920
220374_at	6,318	6,085	6,317	6,016	5,989	5,949
220375_s_at	8,386	8,461	8,471	9,023	9,103	9,373
220376_at	3,718	3,858	3,859	3,778	3,872	3,766
220377_at	7,095	7,410	7,426	7,975	7,752	8,115
220378_at	5,894	6,440	6,288	6,612	6,387	6,751
220379_at	6,698	6,765	6,523	6,996	7,049	7,137
220380_at	4,815	4,418	4,555	4,853	4,812	4,929
220381_at	6,637	6,593	6,377	6,418	6,318	6,494
220382_s_at	6,308	6,397	5,995	5,993	5,804	6,185
220383_at	6,291	5,640	6,059	6,340	6,408	6,423
220384_at	6,060	6,029	5,773	5,160	4,998	5,182
220385_at	6,722	6,484	6,454	7,402	7,388	7,376
220386_s_at	7,625	7,608	7,034	6,776	7,098	6,996
220387_s_at	7,719	8,369	8,495	6,176	6,059	6,566
220388_at	5,590	5,350	5,325	6,008	5,537	5,915
220389_at	5,442	5,546	5,767	5,695	5,563	5,635
220390_at	4,872	4,904	5,367	5,766	5,327	5,875
220391_at	7,032	6,885	7,979	4,437	3,841	4,473
220392_at	6,272	6,298	6,291	7,569	7,426	7,287
220393_at	3,777	4,031	3,725	3,715	3,692	3,672

220394_at	4,414	4,641	4,351	5,053	5,149	5,286
220395_at	8,759	8,542	8,737	9,227	9,083	9,264
220396_at	6,669	6,501	6,807	7,762	7,622	7,731
220397_at	6,975	6,754	6,666	5,947	6,102	5,847
220398_at	6,630	6,633	6,751	7,047	7,168	7,124
220399_at	5,760	6,404	5,724	6,058	5,775	6,028
220400_at	4,546	4,885	4,999	5,379	5,216	5,164
220401_at	4,100	4,023	3,860	4,007	4,126	4,263
220402_at	4,498	4,324	4,822	4,515	4,649	4,508
220403_s_at	6,821	6,661	6,803	7,014	7,118	6,853
220404_at	6,997	6,963	6,925	7,571	6,980	7,298
220405_at	3,754	3,701	3,685	4,367	4,296	4,094
220406_at	6,306	6,592	6,488	7,582	7,549	7,345
220407_s_at	6,061	5,932	6,310	6,298	6,105	6,275
220408_x_at	9,836	10,145	9,541	8,340	8,562	8,346
220409_at	6,134	5,652	5,849	4,758	4,619	4,613
220410_s_at	7,824	7,256	7,473	5,265	5,526	5,432
220411_x_at	7,908	7,983	8,104	7,538	8,458	7,772
220412_x_at	7,730	7,626	7,867	7,853	7,882	7,891
220413_at	6,562	6,191	6,688	6,969	6,907	7,162
220414_at	6,001	5,966	6,484	6,924	6,882	6,874
220415_at	5,336	4,687	5,518	5,229	5,234	5,014
220416_at	4,266	4,331	4,502	4,260	4,511	4,320
220417_s_at	9,216	9,082	9,413	9,459	8,424	9,234
220418_at	6,220	6,108	6,555	6,713	6,705	7,246
220419_s_at	10,523	10,894	10,243	8,875	10,841	8,851
220420_at	7,503	7,216	7,367	8,121	8,173	8,346
220421_at	7,500	7,317	7,666	7,928	7,575	7,558
220422_at	4,966	4,849	4,955	5,420	5,262	5,394
220423_at	7,087	6,858	6,959	7,278	7,115	7,094
220424_at	5,948	6,525	6,544	6,546	6,536	6,477
220425_x_at	6,085	6,246	5,999	6,813	6,692	6,968
220426_at	7,142	7,016	7,126	7,375	7,020	7,302
220427_at	7,492	7,324	7,444	8,012	8,019	8,140
220428_at	5,828	5,854	5,701	6,511	6,176	6,318
220429_at	5,278	5,625	5,542	5,697	5,466	5,652
220430_at	6,647	6,527	6,557	6,600	6,420	6,429
220431_at	6,039	5,814	5,948	6,573	6,190	6,481
220432_s_at	4,137	4,770	4,264	4,592	4,327	4,726
220433_at	7,003	7,071	6,753	7,410	7,632	7,356
220434_at	5,214	4,999	4,776	5,523	5,257	5,556
220435_at	5,431	5,939	5,492	5,302	5,028	5,336
220436_at	7,053	7,129	7,433	4,791	4,975	5,522
220437_at	7,483	6,840	7,318	7,793	7,718	8,181
220438_at	5,792	5,991	6,281	6,438	6,341	6,688
220439_at	6,933	6,700	6,988	7,396	7,006	7,305
220440_at	6,357	6,258	6,152	6,565	6,459	6,575
220441_at	6,131	5,825	6,088	6,863	6,853	6,907
220442_at	6,036	6,012	6,223	6,291	5,950	6,146
220443_s_at	6,885	6,937	7,102	7,577	7,775	7,821
220444_at	6,616	5,933	5,974	6,557	5,871	5,989
220445_s_at	6,955	6,752	6,781	7,213	7,278	7,408
220446_s_at	7,791	8,392	8,508	6,692	6,723	6,541
220447_at	6,486	6,046	6,198	5,458	5,126	5,444
220448_at	8,231	8,870	9,072	6,006	5,893	6,172
220449_at	6,878	6,880	6,488	7,196	7,147	7,101

220450_at	5,772	5,241	6,002	4,530	4,383	4,375
220451_s_at	7,142	7,106	7,159	8,864	8,895	8,961
220452_x_at	8,771	8,516	8,768	7,876	8,236	8,021
220453_at	7,509	7,469	7,830	7,857	7,608	7,845
220454_s_at	8,194	8,122	8,227	7,131	7,182	7,234
220455_at	7,749	7,881	7,890	8,382	8,437	8,820
220456_at	4,887	4,664	4,520	4,662	5,028	4,883
220457_at	7,352	7,263	7,176	7,802	7,692	8,111
220458_at	5,432	5,518	5,089	5,039	4,898	4,934
220459_at	7,991	7,727	7,434	4,693	4,692	4,578
220460_at	4,955	4,810	4,957	5,198	4,794	5,009
220461_at	5,759	6,009	6,055	6,203	6,130	6,095
220462_at	5,296	5,084	4,768	5,513	5,316	5,496
220463_at	6,525	6,167	6,180	6,329	6,206	6,432
220464_at	7,130	6,919	7,097	7,261	7,161	7,312
220465_at	8,521	8,365	8,450	8,866	8,894	8,799
220466_at	6,340	6,947	6,330	6,976	6,519	6,338
220467_at	6,725	6,395	6,758	6,045	6,907	5,890
220468_at	3,939	4,352	3,994	4,200	4,303	4,360
220469_at	6,880	7,089	6,771	7,416	7,498	7,320
220470_at	6,656	6,375	6,728	7,767	7,358	7,763
220471_s_at	5,354	5,602	5,355	5,715	5,678	5,730
220472_at	5,969	6,604	6,399	6,665	6,515	6,741
220473_s_at	6,993	7,363	6,404	6,396	5,820	5,920
220474_at	3,698	4,089	4,801	3,518	3,581	3,671
220475_at	6,777	6,546	6,671	7,266	7,111	7,123
220476_s_at	7,470	7,205	7,627	8,146	7,920	7,995
220477_s_at	9,714	9,418	9,198	8,812	8,386	9,321
220478_at	6,085	5,955	6,078	6,886	6,843	7,147
220479_at	6,009	5,969	6,210	6,266	6,421	6,315
220480_at	6,137	6,348	6,636	7,259	7,055	7,104
220481_at	6,281	6,161	6,335	6,762	6,550	6,629
220482_s_at	7,105	7,454	7,119	7,447	7,347	7,627
220483_s_at	6,842	7,091	6,548	7,599	7,707	7,564
220484_at	6,665	6,828	6,878	6,687	6,330	6,349
220485_s_at	6,367	6,150	6,053	7,149	6,898	7,156
220486_x_at	9,923	9,894	10,317	9,870	9,976	10,041
220487_at	6,614	6,042	6,793	5,693	5,857	6,034
220488_s_at	8,308	8,439	8,527	7,882	7,665	8,250
220489_s_at	5,874	6,124	6,085	7,206	7,207	7,351
220490_at	6,914	6,571	6,973	7,707	7,375	7,916
220491_at	6,280	6,703	6,419	8,041	8,161	8,507
220492_s_at	6,108	5,909	6,117	6,142	6,079	6,333
220493_at	6,160	6,310	6,875	5,341	5,274	5,395
220494_s_at	6,736	7,264	7,584	7,250	7,707	6,516
220495_s_at	9,053	8,957	9,019	11,017	10,634	11,171
220496_at	5,865	5,744	5,891	6,680	6,428	6,586
220497_at	4,789	4,742	4,601	4,355	4,163	4,341
220498_at	6,707	6,654	6,628	7,643	7,714	7,702
220499_at	5,801	5,767	5,741	5,849	5,724	5,897
220500_s_at	6,825	6,738	6,545	6,646	6,192	6,624
220501_at	7,329	7,232	7,350	7,476	7,039	7,233
220502_s_at	4,008	4,370	4,083	4,171	4,428	4,343
220503_at	3,439	3,363	3,436	3,457	3,447	3,457
220504_at	4,123	4,237	4,043	4,247	4,412	4,310
220505_at	6,716	6,491	6,590	7,263	7,005	7,109

220506_at	6,049	6,079	5,820	6,492	6,313	6,649
220507_s_at	6,192	6,172	6,128	7,039	6,988	7,067
220508_at	6,298	6,010	6,366	6,612	6,345	6,711
220509_at	4,697	4,760	4,987	4,743	4,672	4,539
220510_at	8,457	8,380	8,331	8,456	8,521	8,446
220511_s_at	5,024	5,237	5,094	5,762	5,880	6,235
220512_at	5,468	5,626	5,147	6,415	8,257	6,617
220513_at	5,760	5,661	5,938	6,020	5,929	6,141
220514_at	7,083	6,722	6,963	7,446	7,246	7,385
220515_at	5,931	5,565	5,953	6,170	5,824	5,916
220516_at	7,551	7,299	7,588	8,197	8,149	8,193
220517_at	5,173	5,745	5,149	5,149	5,258	5,090
220518_at	4,577	4,678	4,978	5,755	6,443	5,284
220519_s_at	7,245	7,273	7,128	7,344	7,134	7,342
220520_s_at	7,472	7,856	6,078	4,472	4,128	4,169
220521_s_at	7,792	7,779	7,542	7,410	7,329	7,142
220522_at	7,054	6,873	6,992	7,649	7,409	7,661
220523_at	4,392	4,310	4,231	4,230	4,437	4,373
220524_at	6,496	6,081	6,464	5,991	5,869	5,894
220525_s_at	9,736	9,288	9,848	10,348	9,187	10,279
220526_s_at	12,499	12,503	12,980	13,011	12,700	13,084
220527_at	5,319	5,485	5,699	5,864	5,942	6,066
220528_at	6,805	6,358	6,389	6,876	6,800	6,843
220529_at	7,615	7,528	7,355	7,353	7,349	7,217
220530_at	5,935	6,002	6,214	6,124	5,963	5,892
220531_at	6,583	6,264	6,226	6,632	6,889	6,903
220532_s_at	6,368	6,205	6,435	6,782	6,760	6,831
220533_at	7,259	6,980	7,124	6,494	6,271	6,498
220534_at	5,995	5,928	6,026	5,795	5,592	5,828
220535_at	8,082	8,160	8,265	6,527	6,429	6,775
220536_at	8,038	8,122	8,395	8,059	8,177	8,201
220537_at	4,583	4,933	4,470	4,531	4,511	4,466
220538_at	8,120	8,057	8,125	8,151	8,162	8,373
220539_at	5,919	6,131	6,069	6,235	6,417	6,336
220540_at	4,946	4,575	5,031	4,949	4,904	5,103
220541_at	6,234	5,907	6,262	6,364	6,491	6,389
220542_s_at	7,353	7,332	7,245	8,022	7,591	7,914
220543_at	6,456	6,473	6,849	7,549	7,432	7,645
220544_at	6,221	6,500	6,510	6,756	6,782	6,780
220545_s_at	5,986	6,160	6,067	6,810	6,372	7,007
220546_at	7,787	8,015	7,714	7,303	7,359	7,186
220547_s_at	10,826	11,187	10,682	9,795	10,742	9,714
220548_at	6,302	6,332	6,164	6,622	6,416	6,373
220549_at	7,976	8,643	7,517	5,556	4,848	5,264
220550_at	4,045	4,210	4,394	4,619	4,731	4,663
220551_at	4,380	4,383	4,580	4,512	4,518	4,551
220552_at	4,547	4,283	4,440	4,752	4,441	4,619
220553_s_at	9,125	8,837	7,979	6,980	7,829	6,153
220554_at	7,193	7,111	7,134	8,096	8,058	8,233
220555_s_at	7,060	7,091	7,027	7,720	7,255	7,391
220556_at	4,411	4,394	4,294	4,878	5,005	5,328
220557_s_at	5,965	6,381	6,321	7,035	6,951	7,369
220558_x_at	7,745	7,583	7,974	8,169	7,920	8,093
220559_at	5,672	5,448	6,115	5,781	5,678	6,052
220560_at	5,228	5,387	5,374	5,873	5,321	5,717
220561_at	7,346	7,058	7,222	8,251	7,973	8,152

220562_at	6,846	6,719	6,935	8,435	8,574	8,776
220563_s_at	5,984	6,384	5,871	6,365	6,263	6,616
220564_at	5,751	5,396	5,725	7,297	7,573	7,627
220565_at	6,449	6,719	6,735	7,211	6,889	7,150
220566_at	7,297	7,379	7,241	7,352	7,146	7,337
220567_at	6,635	6,488	6,390	6,360	6,579	6,595
220568_at	4,550	4,563	4,535	4,681	4,545	4,737
220569_at	6,165	6,357	6,210	6,277	6,509	6,512
220570_at	6,381	6,050	6,475	6,969	7,258	7,225
220571_at	6,889	6,602	6,765	6,801	6,745	7,056
220572_at	5,781	5,444	6,015	5,687	5,905	5,662
220573_at	7,070	6,868	7,163	7,952	7,571	8,022
220574_at	7,389	7,034	6,985	7,088	7,228	7,261
220575_at	5,491	5,320	5,469	5,338	5,495	5,324
220576_at	9,193	9,168	8,816	5,398	7,206	5,836
220577_at	5,627	5,429	5,553	5,461	5,421	5,485
220578_at	7,404	7,107	6,863	6,829	6,910	6,707
220579_at	5,840	5,871	5,966	6,227	6,150	6,560
220580_at	6,146	5,550	6,105	6,577	6,598	6,086
220581_at	6,661	6,303	6,522	7,253	7,320	7,238
220582_at	6,386	6,117	6,024	6,990	7,076	6,842
220583_at	8,358	8,209	8,338	8,300	8,330	8,276
220584_at	7,788	7,519	7,374	7,130	6,608	7,064
220585_at	6,360	5,907	5,996	6,789	6,429	6,856
220586_at	7,597	6,698	6,681	4,281	5,816	4,485
220587_s_at	9,159	9,125	9,313	8,960	8,316	8,805
220588_at	7,387	7,810	7,119	7,601	6,967	7,737
220589_s_at	8,414	7,653	8,264	8,337	8,445	8,742
220590_at	7,893	7,385	7,704	8,001	7,883	8,068
220591_s_at	6,284	7,282	5,382	5,523	4,765	4,953
220592_at	7,041	6,914	7,038	6,622	6,146	6,580
220593_s_at	6,615	6,375	6,533	7,254	7,482	7,829
220594_at	5,748	6,327	6,174	6,166	5,905	6,106
220595_at	3,975	4,240	4,125	3,735	3,738	3,790
220596_at	6,876	6,887	7,115	6,961	6,896	7,046
220597_s_at	9,405	9,384	9,366	9,792	9,165	9,693
220598_at	6,528	6,641	6,746	7,003	6,560	6,687
220599_s_at	7,254	6,891	6,851	7,791	7,661	7,987
220600_at	6,804	6,680	7,144	7,820	7,465	7,699
220601_at	6,960	6,458	7,035	7,344	7,100	7,024
220602_s_at	9,073	9,337	9,183	7,634	7,159	7,614
220603_s_at	6,457	6,793	6,683	6,004	5,933	6,260
220604_x_at	7,868	7,518	7,668	8,091	8,556	8,570
220605_s_at	8,242	8,221	8,417	9,045	8,771	9,191
220606_s_at	7,271	7,634	7,272	7,502	7,486	7,401
220607_x_at	10,107	9,336	9,979	9,697	9,302	9,678
220608_s_at	4,829	5,071	5,043	4,800	5,111	4,885
220609_at	6,508	6,599	6,148	6,102	6,245	5,823
220610_s_at	8,657	8,819	8,452	8,780	8,692	8,723
220611_at	6,820	6,599	6,516	6,497	6,563	6,491
220612_at	7,388	7,004	7,225	6,047	6,897	6,354
220613_s_at	5,346	5,651	5,017	5,698	5,411	5,473
220614_s_at	4,297	4,405	4,394	4,463	4,383	4,673
220615_s_at	8,834	9,258	8,542	8,768	8,469	7,612
220616_at	8,319	7,717	8,425	8,395	7,767	8,166
220617_s_at	10,202	10,230	10,186	7,532	8,033	7,846

220618_s_at	7,523	7,002	7,649	8,096	8,084	8,058
220619_at	5,122	4,925	5,067	4,726	4,877	4,921
220620_at	5,658	5,381	5,739	6,069	5,489	5,423
220621_at	5,817	5,939	6,025	5,830	5,775	5,906
220622_at	4,187	4,250	4,265	4,365	4,332	4,452
220623_s_at	5,128	5,579	5,158	5,782	6,234	6,171
220624_s_at	5,501	5,741	5,799	6,609	6,451	6,807
220625_s_at	5,528	5,448	5,030	6,226	6,242	6,312
220626_at	6,853	7,105	6,971	7,433	7,667	7,649
220627_at	7,290	7,181	7,239	8,084	7,715	7,784
220628_s_at	6,823	6,960	6,982	7,463	7,355	7,404
220629_at	7,613	7,416	7,557	7,862	7,817	7,799
220630_s_at	6,407	6,429	6,668	7,027	6,867	7,192
220631_at	8,288	8,683	8,435	8,255	7,973	8,059
220632_s_at	8,686	8,435	8,632	8,918	8,981	9,095
220633_s_at	9,089	8,946	9,098	9,462	9,664	9,747
220634_at	6,299	6,438	6,270	6,677	6,860	7,007
220635_at	7,779	7,577	7,722	7,968	7,856	7,886
220636_at	5,284	5,396	5,523	5,918	5,426	5,697
220637_at	8,278	8,114	8,722	6,522	6,327	6,536
220638_s_at	6,217	6,281	6,031	6,477	6,276	6,611
220639_at	5,104	5,386	5,391	5,960	6,116	6,232
220640_at	5,516	5,245	4,949	5,663	5,406	5,392
220641_at	7,921	7,782	7,905	8,147	8,236	8,266
220642_x_at	10,187	10,067	9,767	8,864	9,040	8,621
220643_s_at	10,056	9,714	9,737	9,765	9,102	9,433
220644_at	6,278	6,327	6,325	6,550	6,587	6,593
220645_at	6,641	6,535	6,559	6,991	6,826	6,828
220646_s_at	4,099	4,271	4,041	3,879	3,842	3,819
220647_s_at	9,727	9,779	9,824	10,507	10,011	10,466
220648_at	6,269	6,083	6,188	6,519	6,572	6,832
220649_at	3,988	3,883	3,829	3,984	3,769	3,938
220650_s_at	7,490	7,726	7,865	7,762	7,518	7,889
220651_s_at	10,205	9,781	9,833	8,480	8,576	7,581
220652_at	4,018	4,114	4,316	4,142	4,317	4,227
220653_at	7,175	6,769	6,987	7,441	7,277	7,325
220654_at	6,049	5,788	6,038	6,008	5,715	6,049
220655_at	5,491	5,331	5,555	5,810	5,507	5,384
220656_at	3,531	3,556	3,528	3,891	3,541	3,645
220657_at	7,032	6,841	6,391	6,260	5,739	5,975
220658_s_at	8,649	8,888	8,097	7,595	7,725	7,121
220659_s_at	7,597	7,627	7,706	7,389	7,394	7,320
220660_at	5,697	5,827	5,581	6,147	5,879	5,945
220661_s_at	8,972	8,673	8,499	8,239	8,050	8,199
220662_s_at	7,028	7,166	7,220	7,832	8,050	8,054
220663_at	5,770	5,925	5,713	6,160	5,970	5,973
220664_at	5,362	5,153	5,771	6,126	5,778	5,961
220665_at	7,481	7,631	7,684	7,443	7,630	7,679
220666_at	5,181	3,970	4,372	3,688	3,489	3,611
220667_at	4,978	4,216	4,895	5,426	5,215	5,370
220668_s_at	13,762	13,053	14,014	8,073	7,755	7,846
220669_at	5,044	5,645	4,837	6,035	5,050	5,239
220670_at	4,708	4,510	4,666	5,329	5,308	5,239
220671_at	6,412	6,486	6,510	7,733	7,356	7,593
220672_at	5,560	6,011	5,390	5,713	5,647	5,682
220673_s_at	5,753	6,062	5,279	5,766	5,817	6,247

220674_at	5,881	5,785	5,449	6,051	6,112	6,036
220675_s_at	7,611	7,777	7,716	7,665	7,369	7,694
220676_at	6,066	6,087	5,767	6,688	6,610	6,443
220677_s_at	6,449	6,091	6,400	7,295	7,697	7,497
220678_at	6,231	6,630	6,654	6,788	6,307	6,377
220679_s_at	6,698	6,390	6,459	6,596	6,314	6,515
220680_at	4,794	5,685	4,880	4,238	3,986	4,141
220681_at	7,582	7,697	7,407	8,176	8,174	8,389
220682_s_at	5,257	5,839	5,438	5,432	5,419	5,530
220683_at	7,107	7,259	7,260	8,421	8,182	8,259
220684_at	6,860	6,886	6,928	7,408	7,168	7,272
220685_at	8,364	8,414	8,417	8,034	7,615	7,753
220686_s_at	8,605	8,773	8,535	6,675	6,683	6,625
220687_at	6,044	6,026	6,651	6,271	6,060	6,153
220688_s_at	9,952	9,797	10,119	10,958	9,783	10,318
220689_at	7,776	7,511	7,574	7,627	7,475	7,502
220690_s_at	8,267	8,435	8,697	8,989	8,101	8,945
220691_at	7,329	7,399	7,359	7,709	7,804	7,838
220692_at	7,359	7,091	7,085	7,021	6,975	7,082
220693_at	5,995	5,795	5,693	5,856	5,603	5,788
220694_at	7,149	6,991	7,387	7,260	8,209	6,875
220695_at	6,207	6,315	6,320	6,513	6,678	6,672
220696_at	5,467	4,900	5,642	5,585	5,706	5,616
220697_at	3,806	3,863	3,710	3,801	3,673	3,748
220698_at	7,317	7,098	7,279	7,583	7,666	7,606
220699_s_at	4,875	4,573	4,702	5,014	5,008	4,854
220700_at	3,415	3,575	3,660	3,348	3,551	3,371
220701_at	4,811	5,116	4,894	5,208	4,992	5,133
220702_at	7,477	7,487	7,245	5,853	5,774	5,780
220703_at	6,368	7,213	6,258	5,553	5,437	5,420
220704_at	3,658	3,452	3,420	4,230	4,125	4,100
220705_s_at	6,676	6,886	7,105	7,679	7,821	7,725
220706_at	5,825	5,807	5,859	6,297	5,806	5,943
220707_s_at	7,597	7,377	7,788	7,273	7,068	7,223
220708_at	6,569	7,022	6,996	7,447	7,370	7,184
220709_at	6,820	6,916	7,100	6,997	6,879	6,891
220710_at	4,862	4,522	4,556	4,466	4,529	4,624
220711_at	6,703	6,482	6,620	6,549	6,034	6,224
220712_at	7,797	7,747	7,628	7,853	8,108	8,066
220713_at	6,863	6,695	6,902	7,370	7,473	7,351
220714_at	9,261	9,162	10,429	7,959	7,844	7,996
220715_at	6,425	6,329	6,744	6,861	6,733	6,854
220716_at	5,711	5,893	5,881	5,527	5,698	5,864
220717_at	5,432	6,049	5,402	4,544	4,724	4,816
220718_at	4,840	5,075	4,937	4,941	5,188	5,157
220719_at	6,132	6,093	6,489	6,179	6,709	6,348
220720_x_at	8,745	8,381	8,508	7,977	8,648	8,186
220721_at	7,380	8,123	7,449	6,712	7,073	6,667
220722_s_at	6,449	6,684	6,466	6,453	6,268	6,263
220723_s_at	4,876	5,514	5,458	5,734	5,048	5,265
220724_at	4,611	5,001	4,716	5,073	5,196	5,437
220725_x_at	7,869	7,934	8,236	7,720	8,089	7,970
220726_at	3,411	3,276	3,378	3,829	3,627	3,691
220727_at	7,483	7,680	7,691	8,119	8,286	8,358
220728_at	6,614	5,989	6,086	5,218	5,684	5,637
220729_at	4,429	4,556	4,573	4,377	4,421	4,463

220730_at	7,778	7,355	7,455	7,502	7,408	7,597
220731_s_at	9,114	9,022	9,224	9,663	9,701	10,085
220732_at	5,840	5,667	6,204	5,692	5,857	5,745
220733_at	6,843	6,929	6,888	7,985	7,954	8,144
220734_s_at	7,890	7,589	8,333	8,887	8,994	9,192
220735_s_at	4,063	4,508	3,936	3,817	3,851	3,939
220736_at	6,965	7,163	6,343	6,522	6,306	6,587
220737_at	5,525	5,407	5,700	6,022	5,676	5,771
220738_s_at	6,160	6,273	6,262	5,700	5,336	5,778
220739_s_at	8,049	7,953	8,408	7,727	7,454	7,773
220740_s_at	6,828	7,025	6,945	6,748	7,180	6,874
220741_s_at	10,116	10,448	10,230	10,921	10,601	10,950
220742_s_at	10,816	11,425	10,705	10,216	10,720	10,477
220743_at	8,409	8,455	8,573	8,585	8,615	8,593
220744_s_at	9,196	9,425	9,326	8,752	8,960	8,941
220745_at	6,472	6,431	6,450	6,697	6,754	6,829
220746_s_at	9,385	9,687	9,483	8,831	9,072	8,285
220747_at	6,374	6,612	6,851	7,428	7,196	7,434
220748_s_at	8,934	8,981	9,361	9,063	8,806	9,548
220749_at	6,374	6,450	6,897	6,810	6,989	7,135
220750_s_at	9,836	9,660	9,698	9,719	9,772	10,586
220751_s_at	7,044	7,859	7,440	7,451	7,226	7,569
220752_at	4,333	4,461	4,526	4,502	4,577	4,517
220753_s_at	6,631	6,573	6,354	7,995	7,563	8,025
220754_at	4,423	4,715	4,600	4,848	4,564	4,917
220755_s_at	10,881	11,144	11,078	10,869	10,626	11,261
220756_s_at	6,411	6,765	6,620	7,562	7,672	8,073
220757_s_at	7,802	7,921	7,827	8,916	8,534	9,095
220758_s_at	7,644	7,559	7,592	8,508	7,786	8,121
220759_at	3,634	4,117	3,841	4,043	3,731	4,147
220760_x_at	9,075	8,984	8,697	7,711	8,338	7,879
220761_s_at	7,706	7,918	7,371	8,137	8,989	7,843
220762_s_at	8,697	8,370	8,570	9,276	8,821	9,227
220763_at	6,629	6,516	6,319	6,814	6,771	6,590
220764_at	6,321	6,352	6,231	6,157	5,928	6,062
220765_s_at	6,879	6,675	6,981	7,737	7,651	8,065
220766_at	5,645	5,915	6,129	6,447	6,124	6,540
220767_at	4,598	4,626	4,692	5,160	4,783	5,003
220768_s_at	9,044	9,539	8,906	8,992	9,763	8,884
220769_s_at	4,867	4,842	4,645	5,079	5,160	5,004
220770_s_at	7,706	8,238	7,034	5,485	5,476	5,254
220771_at	5,060	5,151	5,103	4,075	3,837	4,151
220772_at	4,060	4,214	4,198	4,277	4,346	4,351
220773_s_at	6,864	6,758	6,924	6,715	6,628	6,661
220774_at	5,683	5,920	6,011	5,078	5,906	4,812
220775_s_at	7,749	7,743	7,061	7,986	8,250	7,492
220776_at	6,766	6,597	6,929	7,673	7,180	7,599
220777_at	6,713	6,343	6,916	5,269	5,981	5,388
220778_x_at	8,430	8,317	8,448	8,942	9,089	9,122
220779_at	7,259	7,156	7,158	7,169	7,349	7,417
220780_at	9,196	9,387	9,194	8,604	8,196	8,352
220781_at	5,055	4,703	4,601	5,599	5,236	5,281
220782_x_at	7,621	7,735	7,750	8,590	8,405	8,615
220783_at	5,301	5,240	5,072	5,452	5,345	5,345
220784_s_at	4,457	4,464	4,307	4,517	4,443	4,688
220785_at	3,952	4,004	4,244	4,437	4,317	4,477

220786_s_at	3,878	4,576	3,761	3,825	4,235	4,051
220787_at	3,922	4,175	3,905	4,157	4,075	3,890
220788_s_at	6,947	6,721	6,825	6,965	6,990	7,145
220789_s_at	10,478	10,307	10,375	9,981	8,939	9,776
220790_s_at	6,334	5,926	6,739	6,750	6,700	6,829
220791_x_at	3,988	4,023	3,845	3,703	3,938	3,909
220792_at	6,601	6,883	6,210	6,598	6,558	6,411
220793_at	4,498	4,661	4,473	5,065	4,516	4,668
220794_at	5,372	5,443	5,215	5,920	6,508	6,426
220795_s_at	6,377	6,500	6,630	6,558	6,420	6,617
220796_x_at	10,821	10,509	10,685	10,187	11,196	10,358
220797_at	8,048	7,050	7,280	6,563	6,258	6,580
220798_x_at	7,383	7,181	7,422	7,887	7,802	8,225
220799_at	3,900	3,704	4,151	3,997	3,938	3,822
220800_s_at	4,976	5,722	5,683	6,179	6,608	6,118
220801_s_at	6,059	6,103	5,742	6,607	6,455	6,365
220802_at	7,552	7,361	7,438	7,863	7,707	7,991
220803_at	4,629	4,942	5,136	5,083	5,016	5,136
220804_s_at	6,961	6,717	6,762	7,166	6,999	7,133
220805_at	7,144	7,337	7,351	7,778	7,868	8,143
220806_x_at	4,929	4,550	4,960	5,030	4,534	4,761
220807_at	7,209	7,739	7,450	6,860	6,680	7,052
220808_at	7,249	6,793	6,966	7,182	7,563	7,072
220809_at	4,638	4,439	4,823	5,128	4,897	4,991
220810_at	3,226	3,271	3,843	3,325	3,320	3,436
220811_at	6,333	6,205	6,539	6,788	6,531	6,990
220812_s_at	4,374	4,444	4,459	4,754	4,631	4,557
220813_at	7,180	6,953	7,382	7,655	7,785	7,737
220814_at	5,835	5,692	5,583	5,915	5,917	5,705
220815_at	7,439	6,987	6,826	7,172	7,135	7,222
220816_at	7,969	8,049	7,707	8,121	8,095	8,294
220817_at	5,445	5,058	5,171	5,701	6,216	5,365
220818_s_at	6,202	6,273	6,567	7,174	7,107	7,197
220819_at	7,551	7,555	7,555	8,031	7,927	8,240
220820_at	6,190	6,779	6,698	6,670	6,654	6,582
220821_at	5,159	5,242	4,949	5,375	5,352	5,732
220822_at	5,837	6,303	5,868	6,068	6,064	6,024
220823_at	7,080	6,807	7,047	7,086	7,224	7,016
220824_at	7,344	7,315	7,073	7,415	7,460	7,535
220825_s_at	8,374	7,969	8,126	8,705	7,839	8,581
220826_at	6,847	6,944	7,612	7,492	7,521	7,779
220827_at	6,460	6,278	6,465	6,127	6,193	6,186
220828_s_at	4,836	4,750	4,716	5,244	5,446	5,772
220829_s_at	5,615	5,507	5,376	5,573	5,330	5,517
220830_at	6,245	5,643	6,132	6,826	6,765	6,848
220831_at	5,367	5,575	5,316	6,036	6,221	6,554
220832_at	5,956	5,777	5,814	6,421	6,391	6,593
220833_at	5,673	5,536	5,897	6,383	6,483	6,760
220834_at	6,114	6,399	6,713	6,498	6,385	6,588
220835_s_at	4,767	4,814	4,547	5,106	5,012	5,359
220836_at	4,016	4,285	3,943	3,858	4,190	3,861
220837_at	3,717	4,003	3,476	4,050	3,972	4,139
220838_at	7,517	7,477	7,520	8,400	8,485	8,510
220839_at	6,887	6,665	6,285	6,363	6,850	6,976
220840_s_at	9,293	9,197	9,198	6,731	6,335	6,193
220841_s_at	7,211	7,640	6,883	7,111	7,312	6,812

220842_at	5,603	6,602	4,889	5,755	6,223	5,864
220843_s_at	7,041	6,867	6,467	6,639	7,188	6,960
220844_at	4,928	4,669	4,595	5,226	5,052	5,259
220845_at	5,002	4,841	4,795	5,519	5,489	5,825
220846_s_at	7,633	7,299	7,776	7,860	7,217	7,367
220847_x_at	5,851	5,747	5,923	6,155	6,449	6,458
220848_x_at	8,246	8,013	8,330	8,417	8,232	8,208
220849_at	6,740	7,194	7,039	7,558	7,544	7,598
220850_at	3,437	3,705	3,688	3,788	3,795	3,891
220851_at	4,483	4,728	4,799	4,553	4,746	4,681
220852_at	6,193	6,285	6,379	6,633	6,437	6,387
220853_at	6,141	6,080	6,407	6,660	6,715	6,832
220854_at	4,619	5,214	5,322	6,492	6,466	6,753
220855_at	4,926	4,883	4,922	4,956	5,685	5,312
220856_x_at	10,357	10,029	10,315	10,598	10,667	11,110
220857_at	5,625	5,646	5,822	6,242	6,367	6,450
220858_at	6,425	6,430	6,401	6,698	6,628	6,504
220859_at	5,566	5,783	6,121	5,737	5,687	5,518
220860_at	5,082	4,637	4,890	4,883	4,608	4,746
220861_at	6,373	5,838	6,459	6,356	6,319	6,283
220862_s_at	6,854	6,827	6,906	8,220	7,968	8,100
220863_at	6,910	6,974	7,007	7,254	7,196	7,463
220864_s_at	12,082	12,009	11,846	11,165	10,820	11,677
220865_s_at	9,820	9,634	10,184	9,324	9,031	8,997
220866_at	5,064	5,078	5,125	5,759	5,751	5,615
220867_s_at	6,315	6,104	6,369	6,986	6,358	7,049
220868_s_at	6,759	6,415	6,745	7,271	7,117	7,151
220869_at	4,877	4,672	4,997	5,328	5,584	5,367
220870_at	6,234	6,206	6,502	7,391	7,100	7,023
220871_at	5,866	6,046	5,789	6,459	6,868	7,216
220872_at	4,248	4,188	4,427	4,172	4,194	4,175
220873_at	5,796	5,667	5,944	5,865	6,014	5,866
220874_at	5,374	5,390	5,476	4,880	5,369	4,995
220875_at	6,267	6,335	6,068	7,017	6,762	6,601
220876_at	6,074	6,937	5,637	5,087	5,230	5,081
220877_at	5,811	5,868	6,039	6,043	5,974	5,883
220878_at	4,695	4,566	4,618	5,036	4,787	4,951
220879_at	7,223	6,860	7,474	7,813	7,427	7,740
220880_at	4,235	4,591	4,373	4,605	4,524	4,498
220881_at	5,529	5,347	5,412	5,984	5,575	5,865
220882_at	6,738	6,559	6,562	7,104	6,499	7,034
220883_at	4,407	4,538	4,960	4,567	4,608	4,583
220884_at	3,546	3,605	3,586	3,986	3,795	3,846
220885_s_at	7,008	6,881	6,158	6,792	6,250	6,166
220886_at	5,780	5,797	5,689	6,205	5,970	5,964
220887_at	7,803	7,320	7,638	6,616	6,913	6,807
220888_s_at	4,751	4,820	4,767	6,050	5,633	6,187
220889_s_at	4,531	5,180	4,554	4,652	4,703	4,798
220890_s_at	13,922	14,030	13,716	11,879	12,161	11,908
220891_at	4,821	4,141	4,516	4,047	4,274	4,195
220892_s_at	10,844	10,775	10,318	8,148	8,469	8,863
220893_at	6,160	6,351	6,260	7,483	7,031	6,726
220894_x_at	7,638	7,594	7,466	8,469	8,559	8,637
220895_at	5,256	5,601	5,532	5,806	5,573	5,739
220896_at	7,601	7,991	7,848	7,909	8,012	7,996
220897_at	4,891	4,405	4,363	4,456	4,449	4,478

220898_at	6,257	5,260	5,653	5,167	5,151	5,070
220899_at	4,843	4,854	4,977	4,799	4,788	4,744
220900_at	3,503	3,871	3,584	3,703	3,600	3,632
220901_at	6,028	6,068	5,966	6,595	6,498	6,171
220902_at	6,203	6,237	6,488	7,009	6,723	6,883
220903_at	5,914	5,884	6,208	6,750	6,631	6,777
220904_at	7,607	7,190	7,874	7,237	7,391	7,273
220905_at	7,729	7,596	7,595	7,529	7,960	7,762
220906_at	5,205	5,131	5,497	5,574	5,386	5,285
220907_at	5,903	5,905	6,171	6,627	6,517	6,327
220908_at	6,282	6,448	6,562	6,991	6,879	6,923
220909_at	5,998	5,656	6,278	6,562	6,880	6,923
220910_at	7,010	6,694	6,850	7,165	7,161	7,220
220911_s_at	7,954	8,078	8,072	7,475	7,422	7,726
220912_at	6,026	6,155	6,087	6,254	6,331	6,361
220913_at	5,073	5,475	4,962	5,387	5,503	5,521
220914_at	5,606	5,342	5,978	6,374	6,281	6,544
220915_s_at	5,973	5,153	5,991	6,436	5,877	5,764
220916_at	4,626	4,347	4,184	4,643	4,665	4,756
220917_s_at	8,843	9,234	8,383	6,999	8,234	7,143
220918_at	6,325	6,542	6,129	6,582	7,300	6,796
220919_s_at	4,650	4,503	4,551	4,657	4,479	4,528
220920_at	5,738	5,438	5,849	5,895	5,811	5,858
220921_at	5,405	5,294	5,564	5,975	5,803	6,002
220922_s_at	7,506	7,346	7,438	7,978	7,492	7,772
220923_s_at	7,985	7,624	7,891	7,950	7,917	7,851
220924_s_at	11,815	12,240	11,738	10,986	12,910	12,086
220925_at	10,598	10,516	10,289	8,732	9,552	9,068
220926_s_at	7,195	7,162	6,638	6,478	8,315	6,770
220927_s_at	6,900	6,972	6,851	7,091	7,331	7,225
220928_s_at	6,833	6,640	6,904	7,225	7,263	7,507
220929_at	6,659	6,560	6,734	6,327	6,318	6,284
220930_s_at	4,717	5,059	4,928	5,100	4,773	4,827
220931_at	5,138	5,799	5,244	5,553	5,734	5,722
220932_at	5,730	6,311	6,059	6,622	6,195	6,764
220933_s_at	9,503	9,850	9,189	9,226	10,621	9,012
220934_s_at	10,115	10,604	10,627	10,279	9,333	10,155
220935_s_at	9,473	9,457	9,405	10,615	9,674	10,477
220936_s_at	7,098	7,180	7,694	6,909	6,637	6,938
220937_s_at	7,427	7,382	7,657	9,171	8,440	9,157
220938_s_at	8,788	8,573	8,613	8,022	7,911	8,126
220939_s_at	10,441	10,311	10,054	8,462	10,091	8,393
220940_at	7,118	6,598	6,895	5,794	8,337	5,851
220941_s_at	7,782	8,119	7,968	7,748	7,068	7,181
220942_x_at	11,282	11,430	10,963	10,775	10,668	10,913
220943_s_at	6,771	7,584	6,570	6,561	6,452	6,367
220944_at	6,977	6,993	7,214	7,881	7,479	7,574
220945_x_at	8,906	9,328	9,070	7,968	7,940	8,285
220946_s_at	7,864	7,482	7,612	7,301	7,324	7,629
220947_s_at	8,534	7,998	8,801	9,105	8,293	8,905
220948_s_at	11,800	11,350	11,696	11,023	10,960	11,387
220949_s_at	10,341	9,961	10,547	9,664	9,106	9,604
220950_s_at	7,602	7,270	7,632	7,976	8,056	8,152
220951_s_at	6,892	6,711	6,914	7,170	6,939	6,985
220952_s_at	11,842	12,056	11,797	6,885	8,166	6,528
220953_s_at	7,660	7,776	7,900	7,708	7,419	7,448

220954_s_at	7,856	7,578	7,571	7,466	7,428	7,374
220955_x_at	6,630	6,815	6,452	6,044	6,602	6,465
220956_s_at	9,096	9,084	9,246	9,586	8,970	9,430
220957_at	4,535	4,469	4,311	4,554	4,683	4,794
220958_at	5,072	5,119	5,431	5,821	5,602	5,708
220959_s_at	6,454	6,148	6,630	7,110	7,109	7,041
220960_x_at	12,750	12,897	12,691	12,581	12,613	12,645
220961_s_at	8,458	8,449	8,856	6,257	6,136	6,251
220962_s_at	8,702	8,444	8,635	9,380	9,544	9,493
220963_s_at	6,063	6,082	6,421	7,072	6,851	6,980
220964_s_at	10,457	10,317	10,684	11,194	10,574	11,273
220965_s_at	7,068	7,235	7,252	7,837	7,752	7,737
220966_x_at	11,072	10,940	11,422	11,469	11,013	11,256
220967_s_at	8,097	7,834	7,912	7,766	7,502	7,508
220968_s_at	10,204	9,913	10,130	9,058	9,072	9,206
220969_s_at	8,235	6,909	7,497	6,077	6,362	6,032
220970_s_at	4,871	4,861	5,164	5,687	5,335	5,316
220971_at	6,727	7,151	6,865	7,185	7,211	7,269
220972_s_at	3,956	3,847	3,891	4,512	4,220	4,357
220973_s_at	7,769	7,934	7,947	8,433	8,055	8,435
220974_x_at	6,902	6,689	6,572	9,343	9,728	9,672
220975_s_at	8,015	7,670	8,079	8,889	8,641	8,709
220976_s_at	5,840	5,968	5,779	6,858	7,626	7,884
220977_x_at	7,538	7,301	7,017	6,380	6,646	6,529
220978_at	4,570	4,619	4,641	4,801	4,793	5,147
220979_s_at	8,981	9,063	8,536	4,464	4,265	4,249
220980_s_at	10,513	10,576	10,870	10,687	10,428	10,734
220981_x_at	7,279	7,188	7,438	7,800	7,415	7,695
220982_s_at	6,343	6,244	5,928	6,993	6,991	6,700
220983_s_at	5,482	4,842	5,154	5,909	5,894	5,786
220984_s_at	5,606	5,934	6,276	5,542	5,162	5,540
220985_s_at	7,557	7,629	7,305	8,304	8,173	8,781
220986_s_at	4,025	4,257	4,426	4,713	4,707	4,663
220987_s_at	9,883	10,471	10,587	8,433	7,203	7,590
220988_s_at	7,164	6,724	7,365	7,242	7,241	7,054
220989_s_at	7,796	8,217	8,240	8,826	8,831	8,788
220990_s_at	11,073	11,061	11,152	11,464	11,905	12,009
220991_s_at	4,109	4,755	4,791	4,054	3,906	3,904
220992_s_at	9,409	9,831	9,174	7,216	7,830	6,421
220993_s_at	7,229	6,600	6,793	6,415	5,600	5,515
220994_s_at	7,357	7,658	7,562	8,161	7,610	7,601
220995_at	5,124	5,105	5,113	5,514	5,490	5,497
220996_s_at	5,468	5,312	5,518	6,016	6,148	6,219
220997_s_at	4,653	6,481	5,061	4,370	4,668	4,781
220998_s_at	7,297	7,280	7,350	7,087	6,768	7,223
220999_s_at	8,080	7,492	8,045	6,382	6,576	6,366
221000_s_at	6,978	7,257	7,214	7,368	7,464	7,786
221001_at	6,838	6,554	6,840	7,056	6,550	7,008
221002_s_at	8,870	8,723	8,820	8,795	8,495	8,900
221003_s_at	7,420	7,702	7,571	7,698	7,893	7,741
221004_s_at	10,846	10,926	10,795	9,203	8,945	9,517
221005_s_at	9,024	9,172	9,225	8,992	8,739	9,029
221006_s_at	8,612	8,632	8,105	7,954	8,234	8,243
221007_s_at	9,067	9,272	8,740	9,097	8,932	8,883
221008_s_at	5,154	5,713	5,684	4,778	4,803	5,228
221009_s_at	7,106	6,997	6,883	11,078	11,245	12,059

221010_s_at	8,394	8,593	8,371	8,148	8,258	8,402
221011_s_at	10,748	10,836	10,875	7,460	7,217	7,368
221012_s_at	8,212	7,808	8,813	9,930	9,723	10,023
221013_s_at	7,336	7,421	7,422	8,250	8,036	7,967
221014_s_at	7,232	7,523	7,068	6,028	6,580	6,264
221015_s_at	5,811	5,779	5,467	6,109	6,423	6,234
221016_s_at	10,438	10,005	11,339	6,274	6,329	6,546
221017_s_at	6,406	6,097	6,281	6,589	6,609	6,721
221018_s_at	5,380	5,261	5,252	5,897	5,791	6,185
221019_s_at	6,498	6,772	6,314	5,993	8,219	7,127
221020_s_at	9,747	9,503	9,649	10,539	10,659	10,981
221021_s_at	10,503	10,433	10,649	10,185	9,838	9,818
221022_s_at	7,109	7,222	7,171	7,231	7,283	7,282
221023_s_at	6,295	6,171	6,226	6,540	6,984	6,557
221024_s_at	9,193	9,566	9,406	8,334	10,068	10,220
221025_x_at	8,755	8,908	8,773	7,901	8,684	8,076
221026_s_at	5,123	5,060	5,187	5,601	5,299	5,450
221027_s_at	8,362	8,712	8,509	9,164	8,830	9,333
221028_s_at	9,020	8,548	8,589	8,506	8,277	8,411
221029_s_at	8,302	8,335	8,659	10,916	9,889	11,667
221030_s_at	6,256	6,563	6,673	7,216	7,586	7,218
221031_s_at	6,611	7,136	6,896	6,830	7,603	7,010
221032_s_at	7,033	7,210	6,955	7,797	7,739	7,721
221033_s_at	5,010	5,521	5,451	5,757	6,033	6,026
221034_s_at	6,885	6,862	7,279	7,313	7,216	7,222
221035_s_at	6,138	6,500	6,269	6,179	5,829	6,115
221036_s_at	7,888	7,990	7,882	9,276	9,076	9,363
221037_s_at	3,541	4,148	3,806	3,576	3,497	3,570
221038_at	4,860	4,913	4,861	4,803	4,724	4,583
221039_s_at	7,434	7,205	7,333	6,768	8,608	7,535
221040_at	3,946	4,186	4,229	4,187	4,113	4,122
221041_s_at	8,002	8,332	7,967	8,924	8,784	9,406
221042_s_at	6,515	7,292	6,300	7,082	7,396	7,027
221043_at	5,534	5,648	5,665	5,576	5,669	5,389
221044_s_at	6,035	6,585	5,867	6,990	7,361	7,338
221045_s_at	8,917	9,173	9,070	6,115	7,236	6,360
221046_s_at	10,745	10,827	10,689	10,312	10,268	10,353
221047_s_at	9,213	9,358	9,328	7,140	7,379	7,277
221048_x_at	6,239	6,228	6,154	6,778	6,715	6,677
221049_s_at	8,261	8,297	7,982	8,932	8,622	8,952
221050_s_at	8,261	8,377	8,415	7,960	7,935	8,230
221051_s_at	8,325	9,072	8,476	7,801	7,548	7,989
221052_at	5,366	5,751	5,881	4,915	4,735	4,894
221053_s_at	6,199	6,678	6,138	5,306	5,145	5,173
221054_s_at	5,821	5,635	5,342	6,499	6,521	6,668
221056_x_at	7,860	7,951	8,073	8,266	8,025	8,180
221057_at	4,819	4,072	4,419	5,485	5,320	5,804
221058_s_at	7,702	7,867	7,722	9,474	8,779	9,284
221059_s_at	10,739	11,147	11,119	11,805	11,772	12,208
221060_s_at	5,882	5,450	5,828	6,114	6,481	6,511
221061_at	8,341	8,344	8,373	9,281	9,190	9,240
221062_at	5,615	5,486	5,454	6,129	5,583	5,913
221063_x_at	7,680	7,788	7,712	7,746	6,947	7,772
221064_s_at	8,484	8,691	8,602	8,047	8,044	8,241
221065_s_at	6,880	7,424	7,597	7,496	7,391	7,631
221066_at	6,179	5,891	5,996	6,333	6,225	6,350

221067_s_at	7,699	7,621	7,669	8,128	8,101	7,961
221068_at	8,321	8,146	8,320	8,885	8,817	8,936
221069_s_at	8,912	8,765	9,025	9,878	8,803	9,310
221070_s_at	5,841	5,622	5,581	6,273	6,039	6,508
221071_at	7,081	6,804	6,791	6,356	6,624	6,580
221072_at	6,815	6,832	6,496	7,473	7,091	7,617
221073_s_at	8,539	8,708	8,506	7,910	8,521	8,354
221074_at	6,991	6,818	6,937	7,590	7,602	7,662
221075_s_at	5,282	5,202	5,240	5,903	5,539	6,094
221076_at	4,078	4,425	4,008	4,528	4,331	4,505
221077_at	6,904	6,888	6,959	7,632	7,328	7,382
221078_s_at	8,170	8,616	7,947	7,737	8,001	7,583
221079_s_at	8,089	7,890	7,658	8,014	7,163	7,824
221080_s_at	6,650	6,472	6,673	6,500	6,815	6,885
221081_s_at	6,499	6,565	6,798	7,681	7,461	7,664
221082_s_at	7,104	7,150	7,366	7,805	7,414	7,879
221083_at	7,239	7,194	7,132	7,753	7,487	7,871
221084_at	5,966	5,856	6,042	6,741	6,466	6,806
221085_at	5,087	5,142	5,092	5,216	5,233	5,257
221086_s_at	5,950	4,609	5,076	4,400	4,137	4,329
221087_s_at	4,704	5,017	5,035	5,977	5,854	5,631
221088_s_at	8,065	8,182	7,707	5,086	5,191	4,986
221089_at	5,398	5,015	4,957	5,426	5,731	5,593
221090_s_at	8,660	8,356	8,504	7,463	7,615	7,797
221091_at	5,915	6,222	6,271	6,928	6,284	6,486
221092_at	3,941	4,347	4,081	3,962	3,951	4,032
221093_at	5,324	5,522	5,529	6,176	6,091	6,170
221094_s_at	9,302	9,284	9,141	8,638	8,386	8,423
221095_s_at	6,877	6,699	6,863	7,207	7,086	7,371
221096_s_at	8,766	8,892	8,962	7,803	7,101	7,681
221097_s_at	5,764	5,785	5,858	6,162	6,017	5,769
221098_x_at	5,491	5,004	5,412	5,421	4,830	5,350
221099_at	8,167	8,010	8,098	8,214	7,977	8,294
221100_at	7,299	7,174	7,371	7,850	7,658	7,881
221101_at	6,665	6,847	6,748	7,012	6,874	6,871
221102_s_at	6,528	6,513	6,763	7,077	6,681	6,642
221103_s_at	5,216	5,044	4,932	5,285	5,251	4,977
221104_s_at	4,613	5,202	4,822	3,925	3,504	3,869
221105_at	5,938	5,775	5,964	6,138	6,348	6,328
221106_at	6,160	6,300	6,205	6,573	6,377	6,886
221107_at	6,174	6,202	6,131	6,236	5,953	6,033
221108_at	8,282	8,128	8,255	8,395	8,432	8,355
221109_at	7,206	7,163	7,259	7,828	7,391	7,515
221110_x_at	7,549	7,071	6,926	7,666	7,515	7,743
221111_at	3,919	3,715	3,749	4,058	4,203	4,313
221112_at	6,639	6,769	6,560	7,115	7,176	7,232
221113_s_at	7,337	7,421	7,584	7,820	7,616	8,080
221114_at	6,883	6,594	6,803	7,531	7,172	7,396
221115_s_at	6,784	6,836	7,009	6,636	6,858	6,907
221116_at	6,978	7,255	7,266	7,609	7,528	7,659
221117_at	4,877	4,754	4,759	5,131	4,953	5,058
221118_at	5,467	5,626	5,335	6,208	6,075	6,145
221119_at	6,368	6,409	6,610	6,533	6,199	6,226
221120_at	6,685	6,389	6,425	6,369	6,299	6,115
221121_at	6,579	6,584	6,902	6,957	6,891	7,105
221122_at	6,641	6,435	7,258	7,914	8,271	7,975

221123_x_at	8,650	8,826	8,796	7,184	7,387	7,604
221124_s_at	6,419	6,582	6,791	6,946	7,026	7,212
221125_s_at	6,466	5,944	6,423	7,218	7,360	6,979
221126_at	4,060	4,068	4,112	4,236	4,172	4,244
221127_s_at	6,155	6,504	6,363	6,873	6,906	7,323
221128_at	7,024	6,798	6,656	7,487	7,468	7,425
221129_at	6,449	6,102	6,454	6,605	6,764	6,747
221130_s_at	6,002	5,976	5,770	6,408	5,972	6,006
221131_at	6,112	6,643	6,723	6,718	6,680	6,561
221132_at	5,600	5,298	5,982	6,290	6,369	6,402
221133_s_at	6,345	6,440	6,135	6,176	6,036	5,786
221134_at	7,421	6,695	6,993	7,654	7,700	7,910
221135_s_at	8,190	8,127	8,232	7,861	7,890	7,639
221136_at	7,801	7,679	7,746	7,907	7,960	7,764
221137_at	5,686	5,474	5,849	6,442	6,467	6,371
221138_s_at	6,043	6,056	6,227	6,217	6,448	6,524
221139_s_at	6,955	6,710	6,429	5,912	7,217	7,073
221140_s_at	6,392	6,403	6,149	6,667	6,443	6,620
221141_x_at	8,236	8,015	8,230	8,583	8,510	8,671
221142_s_at	8,428	8,513	8,215	7,877	7,225	7,907
221143_at	7,369	6,970	7,289	6,446	6,351	6,307
221144_at	3,714	3,848	4,051	3,855	3,812	3,922
221145_at	4,192	4,541	4,699	4,852	4,714	4,653
221146_at	4,793	5,240	5,246	5,209	5,460	5,346
221147_x_at	7,571	7,569	7,613	7,574	7,443	7,632
221148_at	6,717	6,524	6,444	7,276	7,172	7,240
221149_at	5,185	5,288	5,479	6,008	5,622	5,934
221150_at	6,890	6,702	6,752	7,591	7,467	7,509
221151_at	5,055	5,202	5,333	6,346	6,065	6,473
221152_at	4,284	4,389	4,874	4,155	4,270	4,139
221153_s_at	4,289	4,368	4,117	4,207	4,237	4,065
221154_at	5,046	4,707	5,032	5,464	5,082	5,064
221155_x_at	8,151	8,100	8,322	7,934	8,265	7,885
221156_x_at	5,428	6,065	4,984	6,448	7,844	6,278
221157_s_at	6,569	6,516	6,453	7,286	7,595	7,782
221158_at	10,956	10,797	10,611	7,758	8,388	7,686
221159_at	5,607	5,884	6,096	6,762	6,568	6,507
221160_s_at	6,481	6,380	6,343	6,559	6,509	6,462
221161_at	6,060	5,821	5,970	6,244	6,200	6,220
221162_at	8,392	8,419	8,698	8,405	7,982	8,475
221163_s_at	8,054	8,641	8,094	8,003	7,761	7,888
221164_x_at	6,813	6,638	6,878	6,845	7,259	7,175
221165_s_at	5,521	6,244	6,038	6,498	6,367	6,498
221166_at	6,482	6,427	6,460	6,662	6,512	6,458
221167_s_at	6,586	6,679	6,744	7,186	7,107	7,091
221168_at	6,336	6,342	6,944	5,900	5,448	5,746
221169_s_at	4,651	4,775	4,826	4,956	5,090	5,175
221170_at	3,924	3,681	3,968	4,113	3,894	3,836
221171_at	6,826	6,829	7,001	6,969	6,827	6,850
221172_at	6,963	7,123	7,031	7,857	7,605	7,754
221173_at	6,494	6,895	6,658	6,812	6,720	6,892
221174_at	4,013	4,323	4,194	4,505	4,405	4,338
221175_at	5,578	6,232	5,958	6,374	5,970	6,404
221176_x_at	8,436	8,314	8,793	8,134	9,047	8,583
221177_at	3,960	3,901	3,705	4,208	4,050	4,086
221178_at	4,322	4,182	4,366	4,171	4,057	4,324

221179_at	6,682	6,251	6,623	6,641	6,858	6,652
221180_at	7,434	7,740	7,862	8,031	7,424	7,785
221181_at	6,746	6,698	6,707	8,147	7,677	7,973
221182_at	3,799	3,710	3,851	4,456	4,406	4,541
221183_at	5,871	5,829	6,197	6,615	6,362	6,650
221184_at	5,529	5,592	5,621	5,395	5,629	5,701
221185_s_at	7,805	7,531	7,104	6,352	6,571	5,938
221186_at	6,722	6,023	6,191	6,342	6,384	6,138
221187_s_at	7,131	7,294	7,474	7,845	7,935	8,041
221188_s_at	7,089	6,982	7,083	7,188	6,883	6,983
221189_s_at	9,133	9,535	9,462	8,924	8,200	8,708
221190_s_at	8,208	8,428	8,570	9,154	8,468	8,616
221191_at	6,364	6,635	6,354	6,545	6,726	6,804
221192_x_at	8,491	8,775	8,628	8,304	8,731	8,366
221193_s_at	9,987	10,223	9,789	7,651	7,974	7,879
221194_s_at	6,229	6,116	6,029	6,639	6,381	6,975
221195_at	3,798	4,320	3,923	4,263	4,229	4,403
221196_x_at	8,175	7,666	7,745	7,413	7,356	6,457
221197_s_at	6,595	6,719	6,851	7,320	7,008	7,287
221198_at	6,634	6,675	7,034	8,617	8,643	9,143
221199_at	6,700	6,322	6,520	7,504	7,128	7,430
221200_at	5,363	5,029	5,244	6,240	5,828	6,399
221201_s_at	6,424	7,035	6,448	7,910	8,053	8,138
221202_at	4,777	4,901	4,693	5,515	5,188	5,098
221203_s_at	10,203	10,468	10,311	8,482	9,146	8,509
221204_s_at	6,198	6,216	6,466	6,651	6,559	6,281
221205_at	4,147	4,045	3,898	3,492	3,954	3,536
221206_at	9,460	9,279	9,609	8,176	8,466	8,375
221207_s_at	9,147	9,109	8,910	4,860	5,431	4,526
221208_s_at	9,290	8,287	9,141	7,116	7,238	7,126
221209_s_at	6,351	5,488	6,437	6,795	6,724	6,896
221210_s_at	7,560	7,842	6,780	4,653	4,578	4,602
221211_s_at	4,434	4,078	3,475	4,986	5,352	5,601
221212_x_at	4,119	4,285	4,134	4,119	4,058	4,126
221213_s_at	4,905	5,026	4,852	6,009	6,879	5,467
221214_s_at	8,628	8,327	8,376	8,475	8,240	8,679
221215_s_at	9,102	9,457	9,207	8,171	9,417	8,244
221216_s_at	8,515	8,115	8,306	8,030	8,122	8,087
221217_s_at	7,145	6,548	6,710	6,457	6,292	6,615
221218_s_at	7,517	8,040	7,236	8,477	8,250	8,310
221219_s_at	8,253	8,349	8,339	9,280	8,576	8,367
221220_s_at	7,609	7,933	7,301	6,462	6,785	6,323
221221_s_at	8,738	8,711	8,690	7,418	8,019	7,440
221222_s_at	6,889	7,079	7,387	6,205	6,078	5,975
221223_x_at	8,130	8,071	8,165	8,188	8,117	8,316
221224_s_at	7,798	7,994	7,544	8,252	8,104	8,190
221225_at	6,383	6,541	6,270	7,166	7,409	7,531
221226_s_at	6,472	6,649	6,678	6,831	6,712	6,763
221227_x_at	9,732	9,813	9,426	9,640	9,176	9,459
221228_s_at	4,576	4,431	4,394	4,935	4,754	4,595
221229_s_at	10,964	11,129	10,780	9,312	9,302	9,163
221230_s_at	11,477	11,633	11,139	8,223	10,041	7,940
221231_s_at	5,365	5,382	5,305	5,409	5,403	5,567
221232_s_at	6,984	6,793	7,071	7,472	7,309	7,478
221233_s_at	4,283	4,228	4,238	3,872	3,766	3,719
221234_s_at	7,411	7,232	6,948	5,176	4,618	4,856

221235_s_at	9,936	9,955	10,587	9,315	9,566	9,506
221236_s_at	6,495	6,694	6,133	6,096	5,888	6,348
221237_s_at	7,414	7,318	7,498	7,640	7,260	7,575
221238_at	6,010	6,292	6,363	5,632	5,849	5,408
221239_s_at	5,977	6,316	5,768	6,855	6,362	6,567
221240_s_at	7,644	7,746	8,077	7,940	8,117	7,896
221241_s_at	7,547	7,853	7,809	6,956	6,819	6,613
221242_at	7,991	7,832	8,010	8,842	8,640	8,972
221243_s_at	7,442	7,319	7,091	7,129	6,835	6,842
221244_s_at	5,184	4,953	4,964	5,287	4,914	5,302
221245_s_at	12,526	12,412	12,175	6,683	7,186	6,623
221246_x_at	6,729	6,820	6,995	8,331	8,263	8,286
221247_s_at	9,937	9,445	9,944	9,701	9,239	9,810
221248_s_at	7,948	8,184	7,638	7,104	7,107	6,749
221249_s_at	9,517	9,602	9,717	7,400	7,132	7,289
221250_s_at	8,465	8,360	8,474	8,905	8,804	8,987
221251_x_at	7,463	7,113	7,625	8,256	7,961	8,131
221252_s_at	5,827	5,749	5,943	6,099	5,810	6,099
221253_s_at	11,932	11,837	11,946	12,475	12,640	12,942
221254_s_at	5,700	5,454	5,469	5,781	5,643	5,860
221255_s_at	10,412	10,470	10,839	11,170	10,043	10,730
221256_s_at	8,063	7,997	7,959	7,357	6,738	7,146
221257_x_at	9,509	9,640	9,296	8,911	10,031	8,546
221258_s_at	8,968	9,367	8,599	6,173	7,141	5,144
221259_s_at	6,125	6,308	6,227	5,812	5,720	5,845
221260_s_at	9,393	9,154	9,362	7,243	7,874	7,761
221261_x_at	10,797	10,689	10,835	9,140	9,315	10,512
221262_s_at	5,776	5,904	5,707	6,214	6,323	6,477
221263_s_at	9,991	10,255	10,315	11,299	10,473	11,366
221264_s_at	10,289	10,072	10,026	6,567	7,903	6,767
221265_s_at	9,697	9,561	9,626	9,249	8,596	9,060
221266_s_at	5,558	5,472	5,511	6,829	6,334	7,129
221267_s_at	9,841	9,751	9,802	10,029	9,442	10,194
221268_s_at	5,748	6,105	5,605	6,341	5,766	6,372
221269_s_at	8,951	8,705	8,933	10,671	10,610	11,319
221270_s_at	9,193	8,672	9,101	8,753	7,741	8,011
221271_at	6,219	6,096	6,447	6,577	6,615	6,737
221272_s_at	7,720	7,135	8,165	6,764	6,369	7,045
221273_s_at	7,156	6,709	7,210	7,240	7,205	7,447
221274_s_at	9,637	9,801	10,322	9,678	9,446	9,862
221275_s_at	6,587	6,256	6,064	6,343	6,264	6,302
221276_s_at	8,474	8,424	8,865	7,453	8,386	9,097
221277_s_at	8,867	8,824	8,646	8,288	8,184	7,822
221278_at	5,995	5,672	5,636	6,412	5,826	6,203
221279_at	6,166	5,965	5,766	6,051	6,253	6,122
221280_s_at	6,965	6,790	6,738	7,304	6,932	7,217
221281_at	5,949	6,185	6,185	7,474	6,972	6,842
221282_x_at	7,631	7,602	7,766	7,824	7,724	7,914
221283_at	7,320	6,755	6,964	7,267	7,106	7,011
221284_s_at	6,509	6,420	6,548	7,272	6,879	7,365
221285_at	6,146	6,397	6,349	6,454	6,344	6,186
221286_s_at	7,211	7,014	7,204	7,511	7,347	7,277
221287_at	6,475	6,848	6,434	6,827	6,461	6,697
221288_at	5,948	6,038	5,648	6,103	6,496	6,292
221289_at	5,814	6,592	6,442	7,279	7,356	7,303
221290_s_at	8,692	8,807	8,830	7,927	7,350	7,718

221291_at	6,815	6,426	6,914	8,166	7,845	8,421
221292_at	7,126	6,829	7,123	7,432	7,166	7,443
221293_s_at	6,939	7,016	6,977	7,632	7,566	7,876
221294_at	7,119	6,959	7,257	7,800	7,607	7,835
221295_at	5,888	6,461	6,017	7,129	6,973	7,264
221296_at	5,670	5,918	5,898	6,326	6,090	6,186
221297_at	6,450	6,179	6,525	6,960	6,838	7,177
221298_s_at	6,808	6,954	6,960	7,900	7,806	8,193
221299_at	6,306	6,277	6,499	6,544	6,553	6,406
221300_at	6,120	6,146	6,178	7,103	6,919	7,192
221301_at	7,274	7,071	7,297	7,423	7,726	7,755
221302_at	7,577	7,564	7,646	8,098	8,110	8,075
221303_at	5,973	5,919	6,085	6,115	5,964	6,358
221304_at	5,594	5,846	5,555	5,902	5,775	6,053
221305_s_at	5,244	5,176	5,268	5,295	4,884	4,959
221306_at	8,125	7,977	8,142	8,198	8,075	8,459
221307_at	6,721	6,832	6,814	7,237	6,920	7,230
221308_at	7,332	7,376	7,365	7,317	7,274	7,337
221309_at	6,481	6,863	6,713	7,242	7,157	7,229
221310_at	5,737	5,542	5,288	6,058	6,085	6,058
221311_x_at	6,440	6,625	6,216	7,695	6,892	7,098
221312_at	5,577	5,688	5,788	6,124	5,786	5,753
221313_at	7,327	6,217	6,912	7,924	7,500	7,527
221314_at	6,605	6,271	6,365	6,758	6,590	6,889
221315_s_at	7,116	6,955	6,952	8,062	7,812	8,026
221316_at	6,550	6,088	6,088	6,630	6,205	6,624
221317_x_at	7,896	7,706	7,990	7,742	7,434	7,857
221318_at	5,984	5,899	6,246	6,516	6,132	6,296
221319_at	4,589	4,957	4,960	4,920	4,971	4,937
221320_at	7,001	7,117	7,332	8,005	7,637	7,993
221321_s_at	5,655	5,797	5,887	6,844	6,679	6,634
221322_at	4,966	4,766	5,074	5,365	5,843	5,902
221323_at	5,584	5,626	6,090	6,763	6,605	6,659
221324_at	6,892	6,611	6,784	7,173	7,327	7,358
221325_at	4,644	4,802	5,212	5,510	5,085	5,284
221326_s_at	5,531	5,152	5,082	5,075	4,509	4,816
221327_s_at	6,036	5,993	5,996	6,339	6,259	6,509
221328_at	7,317	6,986	7,408	8,324	8,023	8,099
221329_at	6,864	6,719	6,721	6,812	6,939	7,102
221330_at	4,957	4,942	5,011	5,240	5,230	5,221
221331_x_at	6,993	6,879	6,906	7,035	6,924	6,976
221332_at	5,145	4,571	5,221	5,981	5,965	5,776
221333_at	5,554	5,634	5,782	6,444	6,574	6,396
221334_s_at	7,303	7,239	7,383	7,860	7,802	7,929
221335_x_at	7,846	7,308	7,548	7,910	7,354	8,247
221336_at	6,853	6,406	6,612	6,771	6,611	6,684
221337_s_at	5,269	4,748	5,191	4,980	4,839	4,731
221338_at	5,961	5,416	5,894	6,556	6,174	6,678
221339_at	6,326	6,008	6,501	6,591	6,310	6,373
221340_at	7,099	7,163	7,039	7,313	7,368	7,332
221341_s_at	5,315	5,093	5,454	5,936	5,571	5,819
221342_at	5,864	5,313	5,774	5,780	5,791	6,079
221343_at	5,977	5,793	5,846	5,910	5,489	5,757
221344_at	6,591	6,517	6,771	6,621	6,424	6,548
221345_at	6,116	5,960	6,118	6,115	6,104	5,943
221346_at	7,201	7,216	7,067	7,299	7,073	7,407

221347_at	5,710	5,763	5,849	6,490	6,299	6,690
221348_at	5,658	5,837	5,755	5,666	5,430	5,727
221349_at	4,642	5,122	5,078	5,106	5,048	5,285
221350_at	6,580	6,516	6,704	7,795	7,320	7,929
221351_at	7,446	7,097	7,260	7,475	7,367	7,238
221352_at	5,219	5,467	5,364	5,262	5,057	5,326
221353_at	5,450	5,465	5,511	5,931	5,785	5,866
221354_s_at	5,890	5,660	5,676	6,144	6,093	6,112
221355_at	7,100	7,147	6,822	7,832	8,048	8,111
221356_x_at	7,201	7,111	7,222	7,485	7,478	7,823
221357_at	6,945	6,741	6,825	7,560	7,014	7,409
221358_at	5,087	5,075	5,103	5,516	5,354	5,682
221359_at	6,453	6,615	6,744	6,880	6,765	6,819
221360_s_at	5,866	5,592	5,696	6,662	6,587	6,874
221361_at	6,618	6,147	6,395	6,969	6,557	6,813
221362_at	6,468	6,099	6,327	6,984	6,779	6,796
221363_x_at	4,954	4,830	5,006	5,504	5,213	5,434
221364_at	5,807	5,452	5,636	5,980	5,930	6,605
221365_at	5,559	5,580	5,988	5,737	5,703	5,771
221366_at	6,766	6,696	6,952	7,639	7,542	7,792
221367_at	6,307	6,183	6,152	5,840	5,849	6,038
221368_at	6,010	5,949	6,058	6,195	6,067	6,169
221369_at	6,729	6,730	6,658	6,919	6,672	6,782
221370_at	4,160	4,333	4,195	4,397	4,478	4,535
221371_at	6,164	6,030	6,044	6,288	6,113	6,234
221372_s_at	5,874	5,980	6,136	6,024	6,223	6,173
221373_x_at	7,265	7,725	7,386	8,286	8,271	8,398
221374_at	6,015	6,085	6,365	6,163	6,189	6,277
221375_at	6,661	6,569	6,774	7,111	7,094	6,762
221376_at	7,407	7,032	7,548	8,385	8,188	8,263
221377_s_at	7,212	6,908	7,117	7,421	7,493	7,477
221378_at	6,155	6,032	6,171	6,814	6,879	6,893
221379_at	8,006	7,631	7,739	8,678	8,667	8,785
221380_at	6,337	6,303	6,431	6,956	6,965	7,165
221381_s_at	12,422	12,274	11,716	10,933	11,212	11,224
221382_at	7,319	7,287	7,450	7,564	7,286	7,379
221383_at	7,037	6,555	6,567	7,871	8,108	8,491
221384_at	7,153	7,430	6,953	7,225	7,325	7,174
221385_s_at	6,484	6,589	6,716	7,130	7,023	6,955
221386_at	4,518	4,649	4,641	5,126	5,017	5,085
221387_at	7,334	7,053	7,144	7,397	7,473	7,366
221388_at	6,052	6,241	6,006	6,253	6,225	6,235
221389_at	7,092	7,142	6,793	7,454	7,290	7,732
221390_s_at	8,054	7,788	7,909	8,225	8,195	8,170
221391_at	5,561	5,486	5,614	5,498	5,640	5,508
221392_at	5,934	5,741	5,834	5,875	6,031	5,936
221393_at	4,898	5,378	5,277	5,287	4,986	5,096
221394_at	5,210	4,672	4,969	5,248	4,984	5,133
221395_at	5,127	4,689	5,246	5,188	5,199	5,239
221396_at	5,032	4,880	5,176	5,388	5,067	5,392
221397_at	5,398	5,135	5,236	5,708	5,547	5,728
221398_at	4,371	4,722	4,295	4,334	4,373	4,365
221399_at	6,857	6,891	6,882	7,781	7,770	7,737
221400_at	6,034	5,383	5,758	6,445	6,163	6,354
221401_at	6,786	6,816	6,484	7,561	7,741	7,618
221402_at	7,178	7,244	7,395	8,236	8,318	8,480

221403_s_at	6,324	5,917	6,318	6,829	6,647	6,665
221404_at	6,636	6,621	6,623	7,466	7,434	7,441
221405_at	6,019	6,413	6,212	6,546	6,039	6,276
221406_s_at	6,819	6,930	6,327	6,380	6,351	6,589
221407_at	7,282	7,287	7,494	8,140	8,064	8,222
221408_x_at	7,411	7,513	7,429	7,561	7,539	7,506
221409_at	6,422	6,614	6,898	7,331	7,070	7,208
221410_x_at	8,602	8,204	8,120	7,734	7,483	7,753
221411_at	4,997	5,026	5,556	5,919	6,117	6,225
221412_at	6,150	6,085	6,416	6,639	6,704	6,860
221413_at	6,442	6,561	6,429	6,738	6,840	6,399
221414_s_at	5,075	5,219	5,154	5,607	5,058	5,546
221415_s_at	5,535	5,649	5,727	5,822	5,656	5,732
221416_at	5,982	6,154	6,033	7,005	7,107	6,850
221417_x_at	6,064	6,186	5,997	6,272	6,124	6,330
221418_s_at	7,879	7,551	7,717	8,027	7,993	8,168
221419_s_at	8,271	8,237	8,608	8,989	8,931	9,353
221420_at	4,143	4,397	4,131	4,188	4,128	4,406
221421_s_at	7,160	7,133	7,240	7,527	7,474	7,709
221422_s_at	6,821	6,284	6,463	6,865	6,908	6,848
221423_s_at	7,181	7,358	6,795	7,811	7,966	8,320
221424_s_at	6,716	6,963	6,685	7,522	7,899	7,863
221425_s_at	8,507	8,656	8,288	8,783	7,873	8,395
221426_s_at	6,597	7,109	7,101	7,371	7,163	7,054
221427_s_at	8,897	8,771	8,507	8,272	8,261	8,154
221428_s_at	8,430	8,209	7,670	5,441	5,978	5,626
221429_x_at	6,146	6,008	5,736	6,560	6,147	6,737
221430_s_at	7,480	7,287	6,559	6,556	6,699	6,576
221431_s_at	6,449	6,628	6,371	6,543	6,420	6,852
221432_s_at	7,597	7,547	7,553	8,296	8,191	8,308
221433_at	6,324	6,326	6,479	6,569	6,581	6,836
221434_s_at	12,635	12,717	12,636	12,325	11,907	12,225
221435_x_at	8,262	8,268	8,181	8,739	8,352	8,443
221436_s_at	9,674	9,445	9,530	9,312	8,827	8,871
221437_s_at	9,871	9,879	9,731	10,109	9,482	9,981
221438_s_at	4,135	3,838	4,135	4,303	4,166	4,454
221439_at	5,917	5,889	6,057	6,556	6,768	6,647
221440_s_at	6,349	6,258	6,016	6,484	6,722	6,778
221441_at	4,412	4,505	4,641	5,272	4,939	5,217
221442_at	7,038	6,793	6,722	7,511	7,084	7,448
221443_x_at	7,760	7,692	7,811	7,839	7,521	7,681
221444_at	4,441	5,021	4,253	4,850	5,082	4,998
221445_at	4,788	4,612	4,582	5,012	5,110	5,003
221446_at	6,500	6,500	6,327	6,421	6,227	6,237
221447_s_at	7,410	6,631	6,723	8,043	8,056	8,076
221448_s_at	6,056	5,745	6,128	6,207	5,883	5,898
221449_s_at	9,506	9,884	9,257	9,552	10,201	9,674
221450_x_at	8,465	8,438	8,803	8,736	8,657	8,834
221451_s_at	4,587	4,610	4,724	4,795	4,895	4,881
221452_s_at	11,892	12,002	11,800	12,227	11,130	12,104
221453_at	6,865	6,621	6,982	6,964	7,206	7,128
221454_at	6,972	6,690	6,814	7,580	7,270	7,478
221455_s_at	5,971	6,038	6,155	6,658	6,654	6,731
221456_at	6,163	6,193	6,459	7,051	6,981	7,151
221457_s_at	6,438	6,378	6,662	6,905	7,162	6,987
221458_at	5,660	5,809	5,837	6,172	6,477	6,359

221459_at	6,853	6,649	6,976	7,594	7,558	7,707
221460_at	6,327	6,343	6,404	6,705	6,828	6,849
221461_at	4,649	4,617	4,984	5,446	5,482	5,411
221462_x_at	6,495	6,610	6,536	7,460	7,217	6,993
221463_at	7,099	7,230	7,246	8,326	8,490	8,565
221464_at	7,188	6,992	7,183	7,316	7,116	7,172
221465_at	6,212	6,131	6,187	6,532	6,423	6,764
221466_at	6,600	6,476	6,506	6,388	6,187	6,201
221467_at	6,111	6,022	5,830	6,193	6,186	6,101
221468_at	5,453	5,295	5,639	5,918	5,633	6,019
221469_at	6,587	6,145	6,361	7,138	7,040	7,207
221470_s_at	5,869	6,118	5,457	5,844	5,767	5,859
221471_at	9,744	10,062	10,232	11,235	11,760	11,818
221472_at	10,062	10,209	9,932	10,161	10,949	10,553
221473_x_at	9,324	9,586	9,584	10,213	11,016	11,086
221474_at	10,981	11,376	11,142	12,680	12,951	13,222
221475_s_at	14,357	14,663	14,502	13,811	13,872	14,022
221476_s_at	13,909	14,068	13,828	12,297	12,791	12,914
221477_s_at	8,653	8,345	8,726	9,736	8,754	7,178
221478_at	10,751	11,192	9,978	9,454	11,839	10,644
221479_s_at	9,232	9,475	8,919	9,275	10,168	9,956
221480_at	11,641	11,461	11,856	10,268	10,297	9,795
221481_x_at	13,773	13,555	13,696	12,408	12,579	12,243
221482_s_at	11,176	11,419	11,035	10,699	10,279	10,828
221483_s_at	11,189	11,063	10,910	10,165	10,706	10,170
221484_at	10,076	9,904	10,231	9,400	8,511	9,688
221485_at	11,216	11,256	11,714	10,319	10,054	10,199
221486_at	10,486	10,776	10,840	9,213	9,335	9,483
221487_s_at	8,795	8,708	9,505	7,995	7,616	8,070
221488_s_at	11,855	11,559	12,096	12,154	12,009	12,380
221489_s_at	9,933	9,566	9,780	8,347	8,445	8,591
221490_at	9,348	9,430	9,502	8,854	8,914	9,083
221491_x_at	4,794	4,640	4,267	4,789	4,604	4,796
221492_s_at	10,795	10,937	11,111	11,086	11,052	10,993
221493_at	10,586	10,358	10,619	11,389	11,610	11,809
221494_x_at	10,323	10,574	10,725	11,094	10,523	11,189
221495_s_at	9,699	9,570	9,914	10,070	9,826	10,418
221496_s_at	8,480	8,306	8,726	7,703	7,484	8,054
221497_x_at	7,747	7,933	7,914	8,135	7,380	7,753
221498_at	9,705	9,573	9,719	8,558	8,370	7,976
221499_s_at	8,443	8,774	8,093	8,330	8,658	8,293
221500_s_at	8,900	9,285	8,792	8,987	9,661	9,030
221501_x_at	11,242	11,170	11,259	9,649	10,333	10,327
221502_at	11,593	11,500	11,345	10,222	11,507	10,490
221503_s_at	10,259	10,183	9,715	9,344	9,824	9,141
221504_s_at	10,714	11,073	10,667	10,536	10,475	10,564
221505_at	12,987	12,791	12,531	10,714	11,290	10,204
221506_s_at	10,209	9,703	10,298	9,259	9,348	9,538
221507_at	9,086	8,511	8,753	8,173	7,834	8,092
221508_at	5,719	6,115	5,538	5,921	6,365	5,997
221509_at	12,990	12,862	12,968	11,397	12,090	11,703
221510_s_at	11,474	11,174	11,117	10,715	11,526	11,485
221511_x_at	7,009	7,408	6,331	6,154	8,101	6,487
221512_at	8,924	8,334	8,977	8,856	8,347	8,990
221513_s_at	9,161	8,656	9,502	9,181	9,103	9,067
221514_at	10,213	9,707	10,603	10,588	10,467	10,372

221515_s_at	10,542	10,733	10,828	9,542	8,995	9,616
221516_s_at	10,623	10,227	10,712	10,427	10,190	10,392
221517_s_at	9,930	9,859	9,815	9,462	9,385	9,141
221518_s_at	9,195	9,196	8,663	8,406	9,826	7,901
221519_at	9,157	9,140	9,155	9,059	8,815	9,131
221520_s_at	11,731	11,770	11,861	9,825	9,655	9,636
221521_s_at	11,268	11,082	11,309	10,782	9,912	10,109
221522_at	10,029	10,449	9,956	9,653	9,937	9,873
221523_s_at	9,470	9,868	8,861	4,922	4,965	5,154
221524_s_at	10,861	11,155	10,643	8,334	7,918	8,096
221525_at	7,897	7,596	7,942	7,707	7,648	7,716
221526_x_at	9,357	9,770	9,726	8,293	8,255	8,137
221527_s_at	8,433	8,414	8,592	7,668	7,866	7,543
221528_s_at	9,415	9,598	9,475	8,898	8,672	9,321
221529_s_at	7,166	6,769	6,771	7,544	7,397	7,634
221530_s_at	7,670	8,956	7,983	8,216	7,759	7,942
221531_at	9,700	9,710	9,266	9,354	8,949	9,581
221532_s_at	11,832	12,047	11,854	11,697	11,686	11,914
221533_at	6,624	6,488	6,700	6,677	6,582	6,659
221534_at	8,765	8,958	9,171	10,101	9,505	9,932
221535_at	9,856	9,369	9,557	8,266	8,655	8,098
221536_s_at	10,475	10,474	10,590	9,285	9,815	9,197
221537_at	8,691	8,845	8,937	9,694	9,653	9,940
221538_s_at	10,462	10,407	10,781	9,600	9,857	10,631
221539_at	8,873	8,947	9,189	9,222	8,666	9,347
221540_x_at	10,641	10,762	10,512	10,741	10,814	10,739
221541_at	8,363	8,966	8,661	10,047	10,110	10,865
221542_s_at	8,356	8,373	8,436	7,204	7,418	7,170
221543_s_at	10,641	10,757	10,831	9,476	10,090	9,731
221544_s_at	8,134	7,908	8,204	8,246	8,101	8,510
221545_x_at	8,766	8,735	8,980	8,486	8,428	8,979
221546_at	6,618	6,263	6,242	7,042	6,539	6,603
221547_at	8,898	9,285	8,399	8,755	9,286	8,486
221548_s_at	10,039	9,923	10,391	10,014	9,148	9,424
221549_at	9,096	8,906	9,444	9,791	8,716	9,224
221550_at	7,982	8,121	8,773	7,901	7,084	7,833
221551_x_at	6,579	6,697	6,675	7,842	7,306	7,909
221552_at	8,446	8,517	8,197	7,870	7,877	8,048
221553_at	8,945	8,625	8,731	7,952	8,035	8,115
221554_at	9,301	9,346	9,266	9,348	9,173	9,370
221555_x_at	9,822	10,146	9,993	8,058	8,778	8,358
221556_at	10,440	10,882	10,392	7,300	8,607	7,651
221557_s_at	6,951	6,730	6,965	7,301	7,102	7,390
221558_s_at	8,356	8,075	7,978	9,007	8,386	8,105
221559_s_at	10,567	10,741	10,392	9,183	8,969	8,538
221560_at	9,219	9,291	9,510	9,324	8,799	9,240
221561_at	8,778	8,186	8,572	8,403	9,600	9,368
221562_s_at	7,855	8,249	8,103	7,255	7,176	7,458
221563_at	8,396	8,768	8,394	8,515	8,561	8,760
221564_at	9,149	9,346	8,833	9,003	9,200	9,295
221565_s_at	7,076	7,319	7,426	8,827	8,584	8,759
221566_s_at	6,903	6,994	6,783	8,091	7,665	8,281
221567_at	7,517	6,991	7,390	7,976	7,555	8,115
221568_s_at	11,193	11,234	10,886	10,674	11,412	10,749
221569_at	8,701	9,022	8,381	8,362	9,133	8,154
221570_s_at	11,720	12,229	11,537	11,351	11,124	11,501

221571_at	8,738	8,459	8,831	8,708	8,849	8,679
221572_s_at	7,906	7,727	7,702	7,097	7,114	7,283
221573_at	8,523	8,307	8,154	8,018	8,136	7,985
221575_at	9,690	9,311	9,619	7,619	7,415	7,440
221576_at	5,833	6,203	5,829	6,262	6,097	6,211
221577_x_at	6,774	6,978	6,153	8,226	7,799	8,334
221578_at	7,658	7,387	7,304	7,152	7,209	7,201
221579_s_at	9,907	9,680	9,052	9,497	9,143	9,703
221580_s_at	10,670	10,912	10,032	10,155	10,719	10,096
221581_s_at	6,154	6,433	6,670	7,323	6,938	7,027
221582_at	8,759	9,118	9,230	8,436	8,198	8,064
221583_s_at	5,245	5,766	5,338	8,075	7,966	7,801
221584_s_at	7,379	7,683	7,694	10,540	11,616	10,142
221585_at	6,597	6,805	6,941	6,633	6,672	6,735
221586_s_at	9,653	9,400	8,704	6,944	6,546	6,773
221587_s_at	8,068	8,399	8,559	9,683	9,138	9,555
221588_x_at	8,622	9,014	8,743	6,455	6,499	7,000
221589_s_at	9,558	10,198	9,652	6,951	7,310	7,779
221590_s_at	6,798	7,311	7,174	5,391	5,430	5,494
221591_s_at	10,908	10,662	10,901	8,821	8,048	8,618
221592_at	7,068	7,075	7,240	7,299	7,040	7,303
221593_s_at	9,394	9,778	9,260	10,153	9,656	10,120
221594_at	6,078	5,877	5,573	6,540	6,268	6,371
221595_at	6,553	6,953	6,980	6,316	6,380	6,317
221596_s_at	7,633	7,633	7,442	7,104	6,717	6,719
221597_s_at	9,997	9,554	10,035	10,546	9,718	10,587
221598_s_at	9,339	9,450	9,704	9,350	9,130	9,448
221599_at	8,906	9,112	8,784	9,871	9,477	10,094
221600_s_at	7,586	7,755	7,635	8,373	8,038	8,515
221601_s_at	6,424	6,208	6,058	7,289	6,374	7,003
221602_s_at	7,778	7,634	7,529	8,849	8,386	8,595
221603_at	8,046	7,915	7,950	8,359	8,299	8,204
221604_s_at	8,584	8,032	8,105	9,018	8,731	9,203
221605_s_at	11,050	11,029	10,895	7,422	7,177	7,160
221606_s_at	10,438	10,528	9,825	5,870	6,844	5,185
221607_x_at	14,377	14,378	14,481	14,112	14,411	14,496
221608_at	6,710	6,271	6,688	6,973	6,747	6,913
221609_s_at	6,987	6,770	6,735	7,263	7,225	7,249
221610_s_at	6,863	6,936	7,112	7,265	7,298	7,605
221611_s_at	6,682	6,811	6,896	8,179	8,282	8,568
221612_at	6,561	6,682	6,460	6,794	6,904	6,859
221613_s_at	12,053	12,485	11,846	10,857	11,230	10,986
221614_s_at	7,481	7,877	7,822	7,418	7,459	7,345
221615_at	5,420	5,252	4,913	5,671	5,377	5,669
221616_s_at	6,873	7,146	7,013	5,822	7,118	6,528
221617_at	6,898	7,133	7,154	6,142	6,991	6,128
221618_s_at	7,469	6,566	5,854	4,553	5,048	4,423
221619_s_at	13,091	13,243	13,156	12,987	12,847	13,110
221620_s_at	11,127	11,374	11,262	10,096	9,367	10,035
221621_at	6,013	5,995	6,434	6,215	6,058	6,454
221622_s_at	11,104	11,304	10,810	10,627	10,683	10,804
221623_at	5,757	6,111	5,918	5,410	5,461	5,409
221624_at	5,865	5,787	6,016	6,697	6,467	6,614
221625_at	5,792	4,505	5,406	4,482	4,309	4,333
221626_at	7,375	7,478	7,420	7,137	6,857	7,224
[truncated: 531,976 more chars]
